# Alveolar macrophage subtypes express cholesterol and inflammation genes in cystic fibrosis

**DOI:** 10.26508/lsa.202503482

**Published:** 2026-04-30

**Authors:** Xin Li, Fred W Kolling, Daniel Aridgides, Lorraine Gwilt, Diane Mellinger, Claudia V Jakubzick, Alix Ashare

**Affiliations:** 1 Department of Microbiology and Immunology, Dartmouth Geisel School of Medicine, Hanover, NH, USA; 2 Department of Biomedical Data Science, Dartmouth Geisel School of Medicine, Hanover, NH, USA; 3 Department of Medicine, Dartmouth Hitchcock Medical Center , Lebanon, NH, USA

## Abstract

Cystic fibrosis (CF) is a genetic disease that causes lung inflammation. Although new treatments have improved the quality of life of people with CF, inflammation remains a problem. Alveolar macrophages have been shown to be important mediators of CF lung inflammation although the interactions between macrophages and other immune cells in CF are poorly understood. To identify and compare the cellular composition between CF and healthy airspace macrophages and monocytes, healthy control and CF subjects underwent bronchoscopy and single-cell RNA sequencing was performed on the BAL fluid. Unbiased clustering identified 12 macrophage subtypes. We found up-regulation of macrophages expressing CDKN1A and LDLR, both of which are known to alter cholesterol metabolism and promote inflammation. Pathway analysis revealed important interactions between macrophages and monocytes in CF that likely contribute to overall inflammation and adaptation of recruited monocytes to the CF lung. Some of the top pathways identified were related to inflammation and lipid metabolism, which may contribute to persistent inflammation in CF. Our results may identify novel potential therapeutic targets to treat CF lung inflammation.

## Abstract (precis)

This study demonstrates up-regulation of alveolar macrophages expressing genes important for inflammation and lipid metabolism in people with cystic fibrosis, identifying potential therapeutic targets.

## Introduction

Cystic fibrosis (CF) is caused by mutations of the *CFTR* gene, which encodes an ion channel expressed on the surface of many cell types. The absence of functional CFTR in airway epithelia leads to chronic infection by *Pseudomonas aeruginosa* (*Pa*) and other bacteria and chronic inflammation (1). Lung function decline in CF can be attributed to irreversible destruction of the airway muscle and elastic tissue, with subsequent remodeling, a process called bronchiectasis. Inflammation is a critical mediator of bronchiectasis, and markers of inflammation are increased in the CF lung (2, 3). CF treatment has seen impressive progress over the last several decades with availability of highly effective CFTR modulator therapy (HEMT), improving the longevity and quality of life of people with CF (pwCF) (4, 5, 6, 7). Although HEMT offers many improvements (8, 9, 10), lung inflammation persists and lung function continues to decline albeit at a slower rate (11).

Although CF lung inflammation is predominantly neutrophilic, alveolar macrophages (AMs) are important regulators of inflammation in CF. Animal studies have shown that CF AMs have impaired bacterial killing and are hyperinflammatory (12, 13). Similarly, we have shown that human primary CF AMs (14) have an increased inflammatory response but impaired bacterial killing. AMs have traditionally been thought to be a uniform population of cells that can be activated by different disease states (15). However, recent single-cell RNA-sequencing (scRNA-seq) studies have revealed a rich diversity in AMs in bronchoalveolar lavage fluid (BALF) from healthy subjects, with multiple subpopulations that have yet to be characterized in CF and many other diseases (16, 17). scRNA-seq studies by our group and others have found multiple populations of AMs with distinct transcriptomes in health and in pwCF (16, 17, 18). AMs as a whole have remarkable plasticity. Depending on local tissue factors, cytokines, and encountered pathogens, AMs can take on different phenotypes ranging from proinflammatory to anti-inflammatory (19).

Our current study was designed to investigate differences in the immune cell populations of BALF obtained from healthy controls (HC) and pwCF with mild-to-moderate lung disease and currently taking HEMT. In this study, 12 subtypes of AMs in BALF were identified with two subtypes significantly up-regulated in pwCF. Investigating the interactions between different immune cells, we observed that AMs and monocytes exhibited the most significant interactions involving pathways important for lipid metabolism and inflammation. Analyses of AM subtype interactions revealed complex interactions that may be important in uncovering key pathways involved in persistent inflammation in pwCF on HEMT. Overall, our dataset opens many new areas for further investigation, including addressing factors that regulate the programming of AM subtypes.

## Results

### CF BAL cells up-regulate genes involved in immune responses, inflammation, and lipid homeostasis

To assess the cellular composition in the nondiseased airspace versus that with moderate CF lung disease, 7 HC subjects and 7 pwCF underwent BAL (Table 1). All pwCF had at least one copy of the F508del *CFTR* mutation and had been on HEMT consistently for at least 12 mo. Subjects were closely age-matched. The pwCF in this study were on a stable medication regimen and had no exacerbations within the past 12 mo. Five pwCF were colonized with *P. aeruginosa*, and four pwCF cycled inhaled antibiotics every other month. All pwCF in this study were on Dornase alfa treatment. Despite colonization with *P. aeruginosa*, these subjects were exacerbation-free for at least the year before sample collection and, thus, represent a clinically stable population of pwCF. BALF samples were processed as described in the online data supplement and loaded onto the 10X platform for scRNA-seq. Sequenced cells were processed, normalized, and integrated using the Seurat package.

**Table 1.** Subject Characteristics.

Characteristics	CF (n = 7)	HC (n = 7)
Sex, female % (n)	43% (3)	43% (3)
Average age, years (SD)	26 ± 5.2	25 ± 4.7
FEV1, percent predicted (SD)	74.9 ± 20	​
*Pa* colonization % (n)	71% (5)	​
HEMT	100% (7)	​
Cyclic inhaled antibiotics	57% (4)	​

Values are means ± SD; n, number of subjects; FEV1, forced expiratory volume in 1 second; Pa, *Pseudomonas aeruginosa*; CF, cystic fibrosis; HC, healthy control.

**Figure 1.**
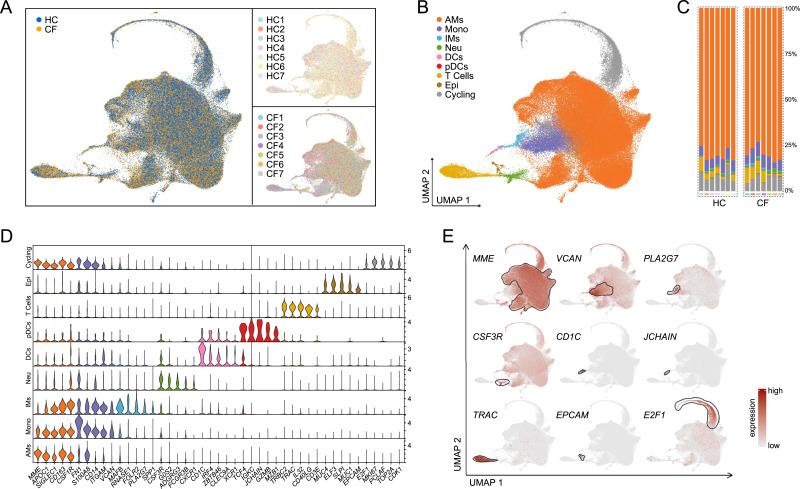
Various major cell types were retrieved in healthy (HC) and cystic fibrosis (CF) bronchoalveolar lavage (BAL) samples. **(A)** UMAP distribution showing integration of BAL cells retrieved from the 10x/Seurat pipeline from HC and CF samples, along with individual sample distribution. **(B)** UMAP demonstrating the nine major cell types: alveolar macrophages (AMs), monocytes (Mono), interstitial macrophages (IMs), neutrophils (Neu), dendritic cells (DCs), plasmacytoid DCs (pDCs), T cells (T Cells), epithelial cells (Epi), and cycling/proliferating cells (Cycling). **(C)** Percentage bar graph outlining the distribution of individual major cell types for each subject. **(D)** Violin plot showing the expression of curated marker genes in each individual major cell type. **(E)** Feature plots showing the expression of curated marker genes in each individual major cell type: MME (AMs), VCAN (Mono), PLA2G7 (IMs), CSF3R (Neu), CD1C (DCs), JCHAIN (pDCs), TRAC (T Cells), EPCAM (Epi), and E2F1 (Cycling).

Uniform Manifold Approximation and Projection of samples from 7 HC and pwCF illustrates similar cell populations across all subjects with differential expression of specific genes (Fig 1A–C). A full list of differentially expressed genes from Fig 1B is shown in Table S1. Using a curated gene list, we characterized nine major cell types (16, 17, 18, 20) (Fig 1D and E). As anticipated, the most abundant cell type is the alveolar macrophage (AM), followed by monocytes. The pwCF in this study all responded clinically to HEMT and remain on HEMT. Despite HEMT, pwCF still struggle with persistent lung inflammation (11). Although chronic infections clearly contribute to the inflammatory milieu in CF, a study in CF ferrets suggests that chronic lung inflammation develops in the absence of infection (21). However, the individual inflammatory pathways involved remain unknown.

Table S1. Full list of differentially expressed genes from Fig 1B.

**Figure 2.**
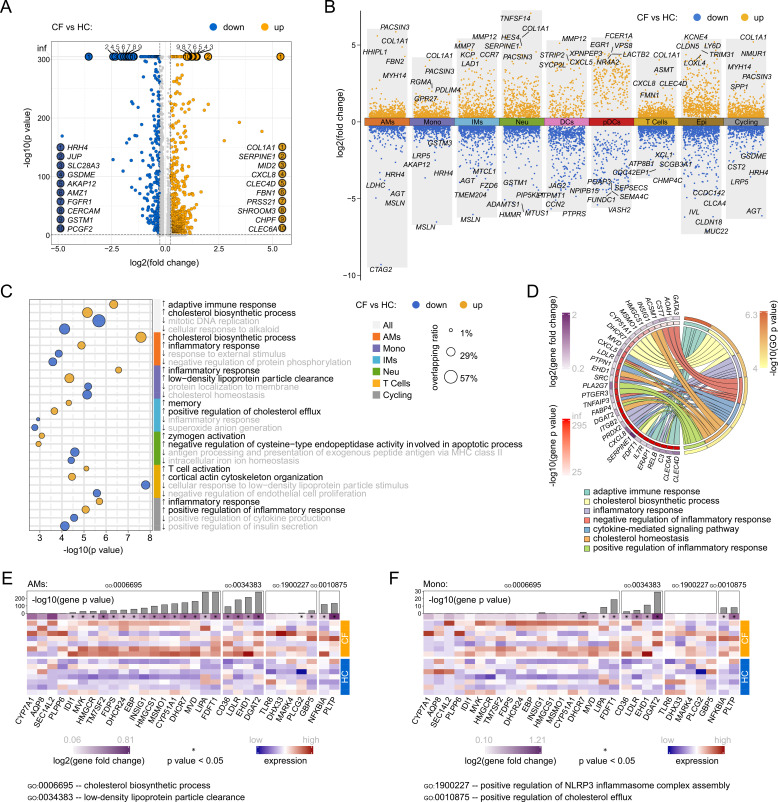
CF BAL cells up-regulate genes involved in immune response and cholesterol biosynthesis. **(A)** Volcano plot showing up-regulated and down-regulated genes when comparing all the BAL cells in CF samples versus HC samples. The top 10 genes in both directions are labeled. **(B)** sVolcano plot showing up-regulated and down-regulated genes when comparing CF samples versus HC samples in each individual major cell type. The top 5 genes in both directions in each cell type are labeled. **(C)** Dot chart showing the enriched biological process–related gene ontology (GO) in CF samples for all the BAL cells and major cell types with sufficient differentially expressed genes for GO enrichment analysis. The top 2 GO terms in both directions in all and each individual major cell type are shown. **(D)** Chord diagram representing the top 7 GO terms enriched for all the BAL cells when comparing CF samples versus HC samples. Specific terms are color-coded in the right inner bands, where chords gather. The outer bands on the right depict the −log_10_ *P*-value for the enriched GO term. The left inner bands represent the gene −log_10_ *P*-value. The outer bands on the left represent the gene log_2_ fold change. **(E, F)** Heat map shows the expression of genes in four GO terms related to cholesterol-induced inflammation across individual donors using pseudobulk data in AMs (E) and monocytes (F). The top bar displays −log_10_ *P*-value; *P*-values of zero are visualized with the second smallest value. The middle annotation shows gene log_2_ fold change between CF and HC; significant genes are marked with an asterisk. Heat map expression values are scaled by columns.

Analysis of all cells in BALF from pwCF compared with HC demonstrates up-regulation of several important inflammatory genes in pwCF, including SERPINE1, CXCL8, CLEC4D, and CLEC6A (Fig 2A). The breakdown of individual cells shows that all cell types have differential regulation of inflammatory genes and genes important for lipid metabolism and cell structure in pwCF (Fig 2B). We next performed gene ontology (GO) enrichment analysis to infer functionality based on their set of DEGs (Fig 2C and D). The data demonstrate consistent up-regulation of inflammatory pathways and lipid synthesis in AMs and monocytes from pwCF, involving biosynthesizing cholesterol and clearing low-density lipoprotein particles through critical enzyme genes like HMGCS1, MSMO1, and CYP51A1 (Fig 2D). Interestingly, both pathways lead to increased cellular cholesterol levels, which have been well studied and characterized in macrophages in atherosclerosis (22). In atherosclerosis macrophages, oxidized LDL (ox-LDL) is internalized through scavenger receptor CD36 and intracellularly converted to cholesterol crystals, which cause lysosomal destabilization and NLRP3 activation (23). NLRP3 can then lead to a series of downstream reactions including the release of IL-1β and sustained inflammation (24). Our analysis suggests a potentially similar proinflammatory pathway may be involved in CF AMs (Fig 2E and F). Differences in AM and monocyte cholesterol acquisition may indicate distinct activation status and cholesterol accessibility.

### Specific AM subtypes are increased in pwCF

**Figure 3.**
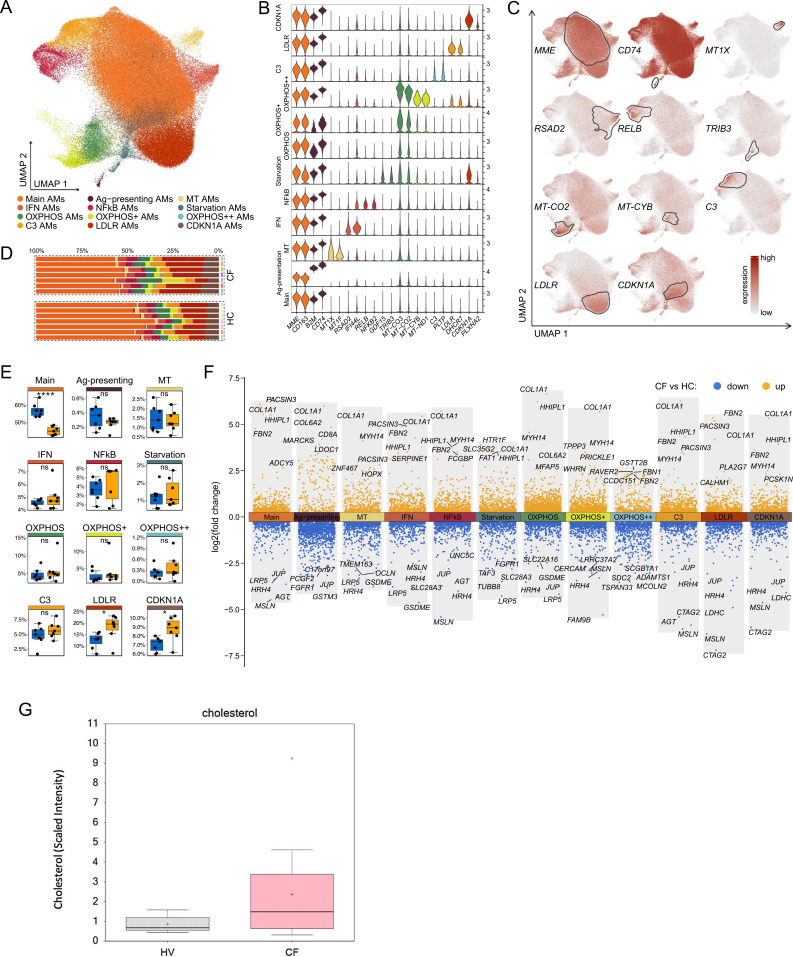
Different AM subtypes contribute to CF differently. **(A)** UMAP demonstrating the 12 AM subtypes: AM subtype with no specific markers (Main AMs), Ag-presenting AMs, metallothionein AMs (MT AMs), interferon-responding AMs (IFN AMs), AM subtype up-regulating high NFkB signaling pathway (NFkB AMs), AM subtype under nutritional starvation (Starvation AMs), AM subtype up-regulating oxidative phosphorylation (OXPHOS AMs), AM subtype with more OXPHOS activity (OXPHOS + AMs), AM subtype with the most OXPHOS activity (OXPHOS++ AMs), AM subtype highly expressing gene C3 (C3 AMs), AM subtype highly expressing the gene LDLR (LDLR AMs), and AM subtype highly expressing the gene CDKN1A (CDKN1A AMs). **(B)** Violin plot showing the expression of curated marker genes in each individual AM subtype. **(C)** Feature plots showing the expression of curated marker genes in each individual AM subtype: MME (Main AMs), CD74 (Ag-presenting AMs), MT1X (MT AMs), RSAD2 (IFN AMs), RELB (NFkB AMs), TRIB3 (Starvation AMs), MT-CO2 and MT-CYB (OXPHOS AMs, OXPHOS + AMs, and OXPHOS++ AMs), C3 (C3 AMs), LDLR (LDLR AMs), CDKN1A (CDKN1A AMs). **(D)** Percentage bar graph outlining the distribution of individual AM subtypes for each subject. **(E)** Box-and-whisker plot comparing the abundance of individual AM subtypes in CF and HC samples. Each dot represents the abundance of that AM subtype in one sample. The lower whisker represents the smallest observation greater than or equal to the lower hinge −1.5 × IQR, and the upper whisker represents the largest observation less than or equal to the upper hinge +1.5 × IQR. The middle line represents the median. The upper hinge represents the 75% quantile, and the lower hinge represents the 25% quantile. *P*-values were calculated using a two-sided *t* test. *P < 0.05; *P* < 0.01; *P < 0.001; *P* < 0.0001; ns, not significant. **(F)** jjVolcano plot showing up-regulated and down-regulated genes when comparing CF samples versus HC samples in each individual AM subtype. The top 5 genes in both directions in each AM subtype are labeled. **(G)** Untargeted metabolomics was performed on the cell-free BAL fluid from HC and pwCF. Cholesterol data are presented as scaled intensity on the y-axis, which is the value of each sample divided by the median value to normalize samples. A *t* test reveals increased cholesterol in the BAL fluid from pwCF (n = 7) compared with HC (n = 7) subjects (*P* = 0.0437).

**Figure S1.**
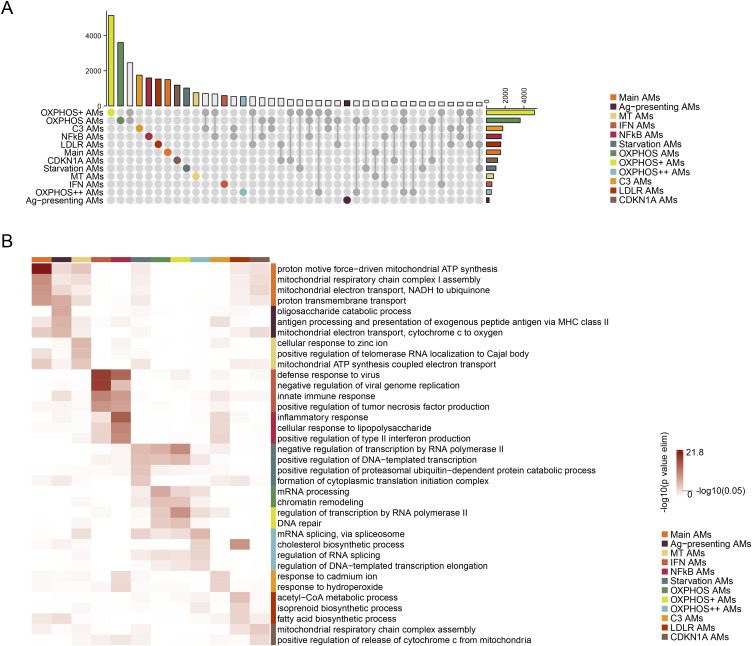
Heterogeneity of AMs in HC and CF BAL samples. **(A)** UpSet plot showing the intersections of DEGs among AM subtypes to illustrate the distinctiveness of each subset and the minimal overlap among them. The top 40 combinations with the most genes are shown. **(B)** Heat map showing the top 4 biological process–related GO terms for each individual AM subtype.

We performed unbiased clustering of the total AM population in our cohorts and discovered 12 AM subtypes with distinct transcriptional profiles and differential expression of marker genes: Main AMs express standard AM curated genes; Ag-presenting AMs express the highest levels of B2M and CD74; metallothionein AMs express metallothionein genes like MT1X; interferon-responding AMs express interferon-stimulated genes and interferon-induced genes like RSAD2; NFkB signaling AMs express genes involved in NFkB signaling pathway including RELB; nutritional starvation AMs express genes like TRIB3 with gene ontology analysis highlighting roles in mRNA processing and protein homeostasis; AMs with differing degrees of up-regulated oxidative phosphorylation (OXPHOS) activity specifically express genes like MT-CO2 and MY-CYB; C3 AMs express C3; LDLR AMs express LDLR; CDKN1A AMs express CDKN1A, a gene associated with macrophage phagocytosis and proinflammatory reprogramming (25) (Figs 3A–C and S1A and B and Table S2). We validated the heterogeneity of those 12 AM subtypes by revealing minimal overlap of their overall DEGs (Fig S1A). Interestingly, BALF from pwCF contained decreased numbers of Main AMs and increased numbers of LDLR AMs and CDKN1A AMs (Fig 3D–F and Table S3). The decrease in Main AMs likely reflects specialization of AMs into functional subtypes in the setting of lung inflammation. However, increased LDLR AMs likely reflect a proinflammatory phenotype as seen in the setting of chronic inflammation in atherosclerosis (26). Other than the previously proposed proinflammatory role of cholesterol through enhanced LDLR gene expression, LDLR AMs also up-regulate PLA2G7, a gene encoding for a protein important for lipid catabolism during inflammation and immune responses (27). Similarly, CDKN1A promotes phagocytosis (25) and has been shown to play a critical role in persistent inflammation. Interestingly, pwCF did not have significantly higher ratio of OXPHOS AMs. This is relevant because macrophages typically switch to OXPHOS as part of the process of resolving inflammation (28), and inflammation resolution is known to be impaired in pwCF (29). Given the persistent inflammatory phenotype in the CF lung, we were particularly interested in the up-regulation of lipids that may lead to increased expression of LDLR in AMs. We performed metabolomics analyses on the cell-free BAL fluid from our CF and healthy cohorts (the same 7 pwCF and healthy control subjects) and found that cholesterol levels are increased in the CF lung (Fig 3G). Although these data demonstrate elevated lipids and altered lipid metabolism in the CF lung, future studies are needed to confirm the specific lipids and lipoproteins involved and the impact of elevated lipids on CF lung inflammation.

Table S2. Full list of differentially expressed genes from Fig 3A.

Table S3. Full list of differentially expressed genes from Fig 3F.

**Figure 4.**
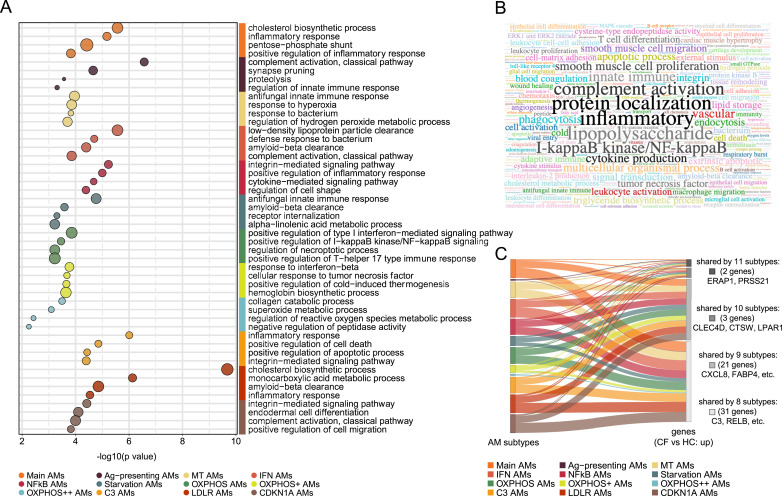
GO terms reveal enriched biological processes in CF samples for individual AM subtypes. **(A)** Dot chart showing the top 4 CF-enriched biological process–related GO terms for each individual AM subtype. **(B)** Word cloud showing the highly frequent CF-enriched biological process–related GO terms for all AM subtypes. The size of the word represents the frequency it presents in the enriched GO terms. **(C)** Sankey diagram showing the highly overlapping CF-enriched DEGs of each individual AM subtype. The left nodes represent AM subtypes, and the right nodes show the highly overlapping DEGs, including 2 genes shared by 11 subtypes, 3 genes shared by 10 subtypes, 21 genes shared by 9 subtypes, and 31 genes shared by 8 subtypes. The width of the connections indicates the quantity of shared DEGs. The colors of the left nodes and the connections represent the AM subtypes, and the colors of the right nodes represent the degree of overlapping, with darker shades indicating higher levels of overlapping.

Next, we investigated the CF-enriched GO pathways to infer the potential functional changes of each AM subtype (Fig 4A and B). The top 4 pathways found in all 12 CF AM subtypes are significantly enriched with inflammatory and lipid metabolism pathways. In addition, CF AM induced pathways related to the complement system and generation of reactive oxygen species. The latter is also corelated to other pwCF-enriched pathways, including the antifungal/bacterium response and up-regulated apoptosis (Fig 4A and B). Shared CF-specific DEGs were discovered among the subtypes (Fig 4C). Highly overlapping genes in CF AM subtypes include the proinflammatory genes CLEC4D, CXCL8, and RELB and the lipid regulating genes FABP4 and LPAR1. Together, these data suggest that AM subpopulations have overlapping functional shifts in pwCF, predominantly in the functional realms of inflammation, immune response, and lipid metabolism.

### Macrophages and monocytes contribute to the inflammatory milieu of the CF lung via cell–cell interactions

**Figure 5.**
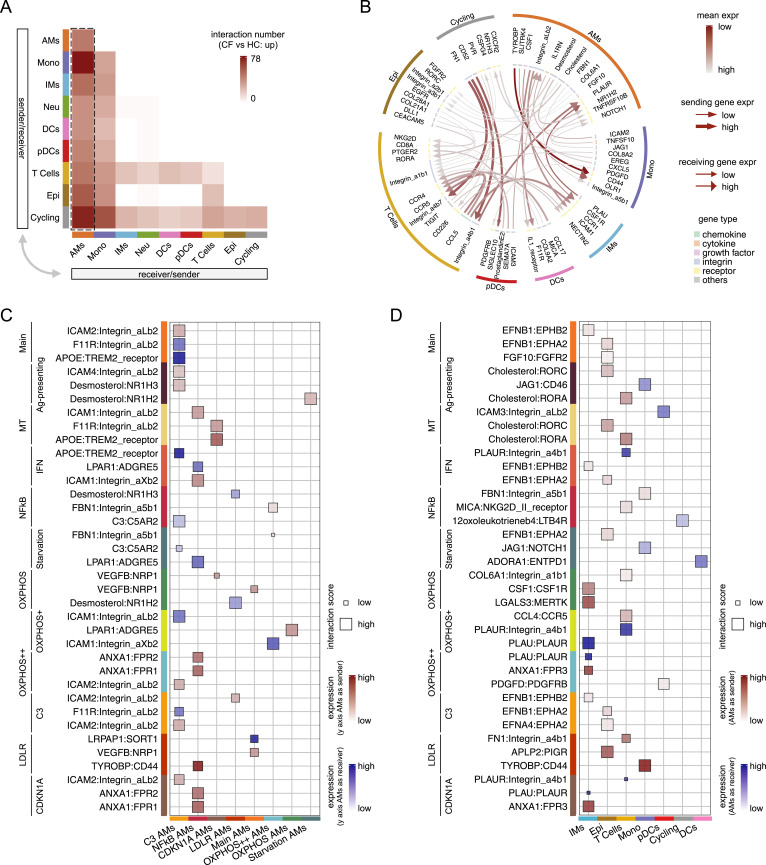
CellPhoneDB reveals enriched cell–cell interactions in CF samples. **(A)** Heat map showing the number of CF-enriched interactions across different major cell types. The dotted box highlights the interactions of AMs with different major cell types. **(B)** Circos plot showing the CF-enriched interactions with the highest interaction score (100). Each segment of the outer circle represents a distinct major cell type. Each segment of the inner circle represents different genes, color-coded for the molecule type the gene is coding, including chemokine, cytokine, growth factor, integrin, receptor, and others. Arcs connecting the inner segments indicate ligand–receptor interactions, with line thickness proportional to the expression of the sending gene (ligand) and the arrow width proportional to the expression of the receiving gene (receptor). The arcs are also color-coded to indicate the mean expression value for all the interacting partners. **(C)** Bubble plot showing the top 3 CF-enriched interactions between each individual AM subtype and other AM subtypes. The y-axis represents the interactions, and the x-axis represents the AM subtypes each individual AM subtype is interacting with, ranked by frequency, with the most interacted subtype on the left and the least interacted subtype on the right. The rectangle bubble is size-coded for the interaction score and color-coded for the mean expression value for all the interacting partners. In the case of y-axis AM subtypes being the sender, the expression is color-coded in red; in the case of y-axis AM subtypes being the receiver, the expression is color-coded in blue. **(D)** Bubble plot showing the top 3 CF-enriched interactions between each individual AM subtype and other major cell types. The y-axis represents the interactions, and the x-axis represents the major cell types each individual AM subtype is interacting with, ranked by frequency, with the most interacted cell type on the left and the least interacted cell type on the right. The rectangle bubble is size-coded for the interaction score and color-coded for the mean expression value for all the interacting partners. In the case of AM subtypes being the sender, the expression is color-coded in red; in the case of AM subtypes being the receiver, the expression is color-coded in blue.

To systematically assess the changes occurring in the samples from pwCF, we used CellPhoneDB to analyze the differential cell–cell communication patterns between pwCF and HC subjects. By comparing ligand–receptor interactions across different cell types, we identified significant interactome up-regulation in pwCF, with the most enrichment involving AMs and monocytes (Fig 5A). A more in-depth analysis showing the top 100 up-regulated interactions in CF BAL samples suggested that AMs are communicating with recruited monocytes through the TYROBP-CD44 pathway (Fig 5B). Interestingly, given the role of CD44 in regulating cell–cell and cell–matrix interaction (30), enhanced crosstalk in cells from pwCF suggests that AMs are promoting local tissue adaptation of recruited monocytes. In addition, AMs showed enhanced crosstalk with other myeloid cells, including IMs and DCs, to promote tissue retention and survival through, for example, CSF1-CSF1R, integrin–ICAM1, and integrin–F11R. Other interesting interactions were also enriched in pwCF, such as recruitment of T cells by CCL17-expressing DCs and the activation of DCs by AMs through expression of IL-1RN (Fig 5B).

Furthermore, the entire AM population exhibited high levels of enrichment in self-regulation in an autocrine manner. For example, the secretion of desmosterol-NR1H2 is a negative feedback loop to control the cholesterol overabundance (31) (Fig 5B). Given this and the fact that AM-AM interactions were significantly up-regulated (Fig 5A), we further divided AMs by subtype and studied the CF-associated crosstalk occurring across different AM subtypes (Fig 5C). Many integrin interactions were revealed between AM subtypes, along with interactions suggesting functional regulations, including desmosterol-NR1H2/NR1H3, APOE-TREM2, and C3. This suggested that CF leads to enrichments in inflammatory interactions within the AM population even in the setting of HEMT. Next, we examined the crosstalk between AM subtypes and other major cell types (Fig 5D). The results suggest, again, a cholesterol-centric interaction between AMs and other cell types (Fig 5D). Being a natural ligand for the retinoic acid receptor–related orphan receptor α (RORα), cholesterol efflux from macrophages can activate T cells for up-regulation of IL-17 and lead to Th17 cell polarization (32). Other than cholesterol, LTB4R also contributes to macrophage inflammation by stimulating chemotaxis and phagocytosis through its lipid ligand (33) (Fig 5D). Interactions regulating cell–cell adhesion and migration are also enriched in AMs in pwCF as indicated by the expression of integrin ligand genes EFNB1 and CCL4.

**Figure S2.**
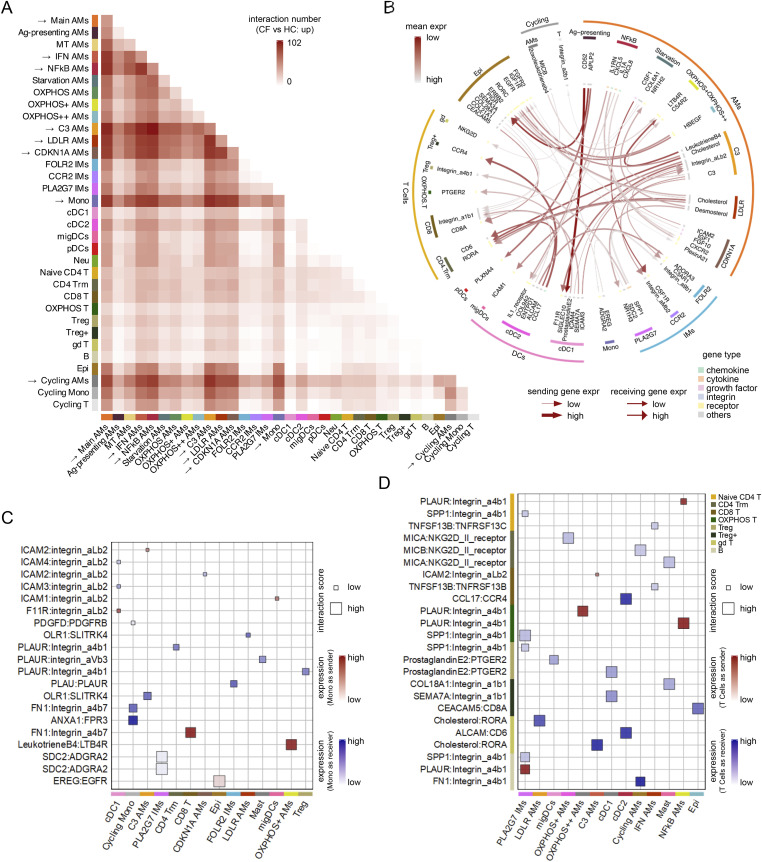
CellPhoneDB revealing the fine subtype–subtype interactions enriched in CF samples. **(A)** Heat map showing the number of CF-enriched interactions across different cell subtypes. Arrows highlight the cell subtypes with the most interactions. **(B)** Circos plot showing the CF-enriched interactions with the highest interaction score (100). Each segment of the outer circle represents a distinct cell subtype. Each segment of the inner circle represents different genes, color-coded for the molecule type the gene is coding, including chemokine, cytokine, growth factor, integrin, receptor, and others. Arcs connecting the inner segments indicate ligand–receptor interactions, with line thickness proportional to the expression of the sending gene (ligand) and the arrow width proportional to the expression of the receiving gene (receptor). The arcs are also color-coded to indicate the mean expression value for all the interacting partners. **(C)** Bubble plot showing the top 20 CF-enriched interactions between monocytes and other cell subtypes. The y-axis represents the interactions, and the x-axis represents the cell subtypes, ranked by frequency, with the most interacted subtype on the left and the least interacted subtype on the right. The rectangle bubble is size-coded for the interaction score and color-coded for the mean expression value for all the interacting partners. In the case of monocytes being the sender, the expression is color-coded in red; in the case of monocytes being the receiver, the expression is color-coded in blue. **(D)** Bubble plot showing the top 3 CF-enriched interactions between each individual lymphocyte subtype and other cell subtypes. The y-axis represents the interactions, and the x-axis represents the cell subtypes each individual lymphocyte subtype is interacting with, ranked by frequency, with the most interacted subtype on the left and the least interacted subtype on the right. The rectangle bubble is size-coded for the interaction score and color-coded for the mean expression value for all the interacting partners. In the case of y-axis lymphocyte subtypes being the sender, the expression is color-coded in red; in the case of y-axis lymphocyte subtypes being the receiver, the expression is color-coded in blue.

A more detailed assessment of cell–cell interactions was also conducted (Fig S2A), showing that the most enriched interactions occurred between Main AMs, IFN AMs, NFkB AMs, C3 AMs, LDLR AMs, CDKN1A AMs, cycling AMs, monocytes, and other cell types. With this more in-depth analysis, more interactions were revealed, including the CD52-SIGLEC10 interaction from Ag-presenting AMs to cDC1, a well-known inhibitory interaction to suppress DC inflammation through the inhibition of NFkB and induction of apoptosis (34). This also provides resolution for the interactions observed at the major cell-type level: cDC2 secretes more CCL17 for the recruitment of CCR4+ Tregs. These CF-specific inhibitory interactions suggest regulatory mechanisms to dampen overwhelming inflammation (Fig S2B). Given the repeated findings showing substantial changes in monocyte interactions in CF BALF, we plotted the top 20 up-regulated monocyte interactions, many of which involve adhesion regulations (Fig S2C). The enrichment of adhesion/chemoattraction interactions was also observed in T cells (Fig S2D). We also noted an enrichment of T-cell activation interactions, including the NKG2D II receptor and PTGER2. The latter supports Th17 cell expansion and function through induction of IL-23R expression (33, 35, 36).

**Figure 6.**
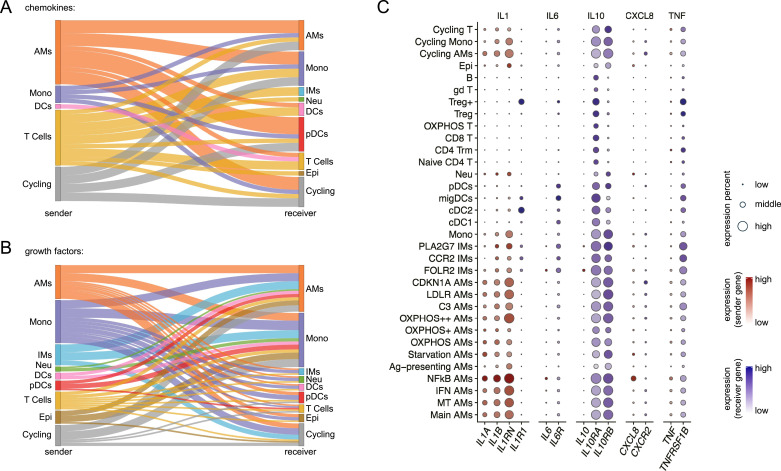
AMs and monocytes contribute to the CF microenvironment. **(A)** Sankey diagram summarizing all the CF-enriched chemotaxis interactions between major cell types. The left nodes represent chemokine-secreting major cell types, and the right nodes represent chemokine-receiving major cell types. The width of the connections indicates the quantity of CF-enriched interactions. The colors of the left nodes, right nodes, and the connections represent the major cell types. **(B)** Sankey diagram summarizing all the CF-enriched growth factor–receptor interactions between major cell types. The left nodes represent growth factor–secreting major cell types, and the right nodes represent growth factor–receiving major cell types. The width of the connections indicates the quantity of CF-enriched interactions. The colors of the left nodes, right nodes, and the connections represent the major cell types. **(C)** Dot plot showing the gene expression of CF-contributing cytokines/chemokines and their corresponding receptors in all the cell subtypes. The dot is size-coded for the percentage of cells expressing the gene and color-coded for the protein type the gene is coding for. For cytokines/chemokines (sender gene), the expression is color-coded in red; for cytokine/chemokine receptors (receiver gene), the expression is color-coded in blue.

The dysregulation of chemoattractants and growth factors exacerbates disease pathology in CF, driving both chronic inflammation and progressive lung damage (37). Thus, we analyzed the sources (senders) and recipients (receivers) of the corresponding molecules using the interaction analysis results (Fig 6A and B). AMs, monocytes, and T cells are the primary sources of chemokines in pwCF, which attract various cell types, with an enrichment of AMs, monocytes, pDCs, and T cells (Fig 6A). On the other hand, CF-specific growth factors are mainly secreted by myeloid cells, supporting predominantly myeloid cells, especially AMs and monocytes (Fig 6B). Next, we examined the best-known cytokine/receptor pairs playing a major role in CF—IL-1, IL-6, IL-10, CXCL8, and TNFα—by assessing expression levels in individual cell types to scrutinize their roles in disease development (Fig 6C). Macrophages, monocytes, and neutrophils are the major sources of IL-1, which functions on cDC2 and Tregs within the BAL. Although all have low expression, IL-6 is mostly secreted by NFkB AMs and FOLR2 IMs to act on IMs, DCs, and pDCs. Similarly, IL-10 is secreted at low levels by FOLR2 IMs. Although IL-10RA is broadly expressed across BAL cells, IL-10RB expression is limited to myeloid cells, indicating that regulation is confined to IL-10RB + myeloid cells. NFkB AMs are also the primary source of CXCL8, for which no obvious recipients were observed in the BAL, except for CDKN1A AMs and monocytes. Other than those cells, CXCL8 is also likely responsible for recruiting neutrophils into the microenvironment of the CF lung, an often underrepresentative cell type in scRNA-seq capture because of their low RNA content and high levels of RNases. Meanwhile, all cell types except epithelial cells and B cells are regulated by TNFα secreted by NFkB AMs.

## Discussion

AMs have multiple functional roles, including clearing the airspaces of inhaled particles and debris, initiating the inflammatory response to pathogens, and maintaining lipid homeostasis. Given the multiple functions of AMs, we and others have previously used single-cell sequencing to demonstrate that there are multiple functionally distinct AM and monocyte subpopulations in the lung (16, 17, 18). Here, we used scRNA-seq to define 12 subpopulations of AMs in healthy subjects and pwCF. Each subpopulation has differently regulated genes involving inflammation and tissue repair. A goal of this study was to uncover transcriptional differences in AMs isolated from pwCF on HEMT to determine whether there are specific pathways involved in the persistent inflammation in pwCF on HEMT. Interestingly, there were specific subpopulations of AMs that were increased in pwCF including AMs involved in lipid metabolism and tissue repair.

Many lines of evidence point to impaired innate immune cell function in CF. Our study suggests significant crosstalk between AMs and other immune cells suggesting that AMs, the primary innate immune cells in the lung, play a major role in orchestrating the immune response in the CF lung. The strongest potential interaction between AMs and monocytes in pwCF involves the TYROBP-CD44 pathway. CD44 has several important functions in cell–cell and cell–matrix interactions including adhesion and migration (30). This pathway contributes to monocyte recruitment and differentiation and may aid in tissue adaptation of recruited monocytes. Elevated CD44 can prevent the transition of immune cells from glycolysis to OXPHOS (38) and promotes continued inflammation. This suggests that the interaction between macrophages and monocytes may promote persistent inflammation in pwCF even in the setting of HEMT. Further studies are needed to confirm this interaction and determine the clinical and physiological consequences.

Our investigation of the interactions between AM subtypes reveals the importance of these AM subpopulations in pathways critical for both inflammation and cell homeostasis. The APOE-TREM2 pathway has been shown to increase phagocytosis and clearance of debris in monocyte-derived macrophages (39), so the interaction between these pathways in AM subtypes could impact phagocytosis and bacterial clearance and killing. Interestingly, we found an important potential interaction between OXPHOS AMs and NFkB AMs involving ANXA1-FPR2. This is an important pathway in both generation of and resolution of inflammation. FPR2 is a receptor that has been shown to respond to lipids and proteins and is immunomodulatory (40). ANXA1 encodes for the protein annexin 1, which is known to be anti-inflammatory and related to the specialized lipid mediators lipoxin A4 and resolvin D1, potent inflammation-resolving lipids that are known to be decreased in pwCF (41). The up-regulation of this pathway suggests an attempt to activate inflammation resolution pathways in the setting of chronic inflammation. This is of considerable interest as these pathways are potentially modifiable. Future studies will investigate specific pathways involved in impaired inflammation resolution in CF to uncover novel therapeutic targets.

One of the most important findings of our study is the altered expression of genes important for lipid metabolism in pwCF. Cholesterol biosynthesis and oxidized LDL are both major sources of cholesterol in cells, and our studies demonstrate elevated cholesterol in the lungs of pwCF. Prior studies have demonstrated abnormal lipid metabolism in CF (42, 43, 44). More recently, studies have found elevated serum cholesterol in pwCF treated with HEMT (45, 46), suggesting that improved weight and nutritional status may put pwCF at risk for hyperlipidemia. Interestingly, prior single-cell sequencing studies have not included pwCF who are all on HEMT and have not identified this subpopulation of AMs with up-regulation of LDLR (16, 17, 18). Thus, it is possible that this population of cells may occur more frequently in pwCF on HEMT, but further studies are needed to investigate this and to determine the impact of dietary lipids on airway lipids. In this current study, GO analysis suggests that both AMs and monocytes up-regulate pathways for acquiring cholesterol crystals, which can subsequently activate the NLRP3 inflammasome pathway. At the same time, we demonstrated that AMs, monocytes, and neutrophils are major cell types expressing IL-1B, encoding for the proinflammatory cytokine IL-1β, which needs caspase cleavage within inflammasomes for biological functions. Given our sequencing data demonstrating transcriptional differences in genes important for lipid metabolism and inflammasome-related inflammation, a potential consequence of these differences could involve differences in inflammasome activation. Besides activating inflammasome pathway, cholesterol also functions as a lipid ligand for transcription factors. T cells are significantly regulated by myeloid-derived cholesterol through receptors like RORγt and RORα, RAR-related orphan nuclear receptor (ROR) family, skewing the CD4 T cell to Th17 cells, which play a critical role in the pathogenesis of many inflammatory diseases, including CF (47, 48). Interestingly, CF patients have higher triacylglycerol levels than the general population (49), which offers the source of acetyl-CoA, an important precursor unit for the biosynthesis of cholesterol. An analogy of this would be atherosclerosis, where the imbalanced lipid metabolism leads to macrophage activation and chronic inflammation (50). Similarly, a dietary intervention is worth exploring to mitigate the inflammation in pwCF on HEMT.

In this study, we identified novel AM subtypes that are elevated in the lungs of pwCF. Importantly, our study found key pathways involved in cell–cell interactions that may be relevant to the persistent lung inflammation seen in pwCF on HEMT. We found that pwCF have increased AM subtypes that up-regulate genes important for cholesterol metabolism and inflammation. Although the individual subtypes of AMs are of interest, the interactions between AM subtypes and other AMs or other immune cells may provide critical information about pathways that could be targeted to treat persistent inflammation in pwCF. Future studies will identify the functional relevance of these pathways in primary immune cells isolated from pwCF.

## Materials and Methods

### Human subjects

Seven healthy control (HC) subjects and seven pwCF underwent research bronchoscopy as previously described (51). Briefly, after local anesthesia and intravenous conscious sedation, a flexible fiber-optic bronchoscope was inserted transorally and passed into the trachea. The bronchoscope was sequentially wedged into three tertiary bronchi, saline was instilled, and BALF was collected. This study was approved by the Institutional Review Board of Dartmouth Hitchcock Medical Center (protocol #22781). All subjects provided written informed consent, and all aspects of this study conform to the principles set out in the WMA Declaration of Helsinki and the Department of Health and Human Services Belmont Report.

### Sex as a biological variable

We enrolled both male and female subjects into our study. Our cohorts were matched by sex. Similar findings are reported for both sexes. Subgroup analyses found no sex-related differences.

### Sample preparation

Before sample processing, an aliquot of BALF was frozen for metabolomics analysis, which was performed by Metabolon (North Carolina). Subsequently, BALF from HC and pwCF was spun at 300*g* for 5 min and washed in 1X PBS before counting and viability assessment on a Cellometer K2 instrument (PerkinElmer) using AO/PI fluorescent live/dead staining. Cells were fixed according to the 10x Genomics protocol (CG000478) and stored at −80°C until further processing. The 10x Genomics Flex assay was performed according to the manufacturer’s protocol (CG000527). Briefly, cells were thawed from −80°C and counted, and up to 1 × 10^6^ cells were used as input into the hybridization reaction using Human Transcriptome Probe Kit, which contains sample-specific barcodes. In total, 20 samples were processed in two pools of 10 samples each, targeting 10,000 cells per sample. The concentration and size of sequencing libraries from the two pools were measured by Qubit (Thermo Fisher Scientific) and TapeStation (Agilent), respectively, and pooled for sequencing on a NovaSeq 6000 instrument (Illumina) to generate 25,000 reads/cell. Raw fastq files were processed using the CellRanger v7.1.0 pipeline before downstream analysis. Cells were retained if they met all of the following criteria: mitochondrial transcript percentage <5%; UMI counts (nCount_RNA) <65,000; detected genes (nFeature_RNA) <7,500.

### Data preparation

Raw sequencing reads were demultiplexed and mapped to the GRCh38 human reference genome, and gene expression matrices were generated using CellRanger v6.1 (10X Genomics). The following analyses were conducted in R 4.2 and Python 3.6. Seurat package v4.3 was used for downstream data analyses, and figures were produced using the package ggplot2. Following the standard Seurat workflow, the datasets were integrated for further analysis.

### Differentially expressed genes

DEGs were calculated with the FindAllMarkers function of Seurat in R v4.2 to study the different expression profiles in different cell types. The data matrices of the SCT assay were used, and the minimal log fold change was set to 0.25. Only genes that were detected in more than 25% of cells in either of the two populations were used to compute the DEGs with a Wilcoxon rank-sum test. Markers were identified as genes exhibiting significant up-regulation when compared to all the other subsets and having a Bonferroni-adjusted *P*-value < 0.05. The DEGs are ranked by the adjusted *P*-value to select the top DEGs for downstream analysis.

### Cell-type identification

To identify cell types, the FindClusters function with the Leiden algorithm with varying resolutions from 0.5 to 2.0 in the Seurat package was used for clustering. The FindAllMarkers function was then applied. The top DEGs of individual clusters were examined for well-studied marker genes across the literature, and the clusters were then annotated for the most likely identity until the right resolution was reached so that each cluster had a unique gene expression pattern.

### GO analysis

Gene set functional analysis was conducted with TopGO (52). Fisher’s exact test was used to determine the more represented functional categories with the DEGs in each cluster. The background genes were defined as all the detected genes in the datasets with GO analysis performed at the level of biological process. Cell-type DEGs were used as the enrichment input. The top five GO terms ranked by *P*-value (52) for every cell type were selected and compared with other cell types. Overlapping GO terms were removed from the final visualization.

### Cell–cell communication analysis

To elucidate intercellular communication within the scRNA-seq dataset, CellPhoneDB v2.0, a repository of curated ligands, receptors, and their interactions, was employed. Using the default parameters of CellPhoneDB, the statistical analysis module was conducted to detect significant ligand–receptor pairs among the annotated cell types and subtypes. The analysis involved 1,000 permutations to estimate the null distribution of average receptor expression levels, with a *P*-value threshold of 0.05 set to determine significance. Only ligand–receptor pairs expressed in more than 10% of cells in the interacting cell types were considered. To further understand the biological relevance of the identified interactions, the significant ligand–receptor pairs were categorized into functional groups based on their known biological roles, such as immune modulation, cell proliferation, and differentiation. This categorization enabled the identification of general interaction patterns and the specific biological processes potentially influenced by these interactions in both healthy and diseased states. This comprehensive cell–cell communication analysis provided crucial insights into the complex signaling networks within the CF microenvironment and highlighted potential targets for therapeutic intervention.

### Metabolomics

After spinning down cells to the resulting cell-free BAL fluid, full untargeted metabolomics was performed on the BAL fluid using Metabolon Global Discovery Platform. Samples were precipitated with methanol under vigorous shaking for 2 min followed by centrifugation. The resulting extract was dried and reconstituted in aliquots for four ultra-performance liquid chromatography methods. One aliquot was analyzed using acidic positive ion conditions optimized for more hydrophilic compounds. In this method, the extract was gradient-eluted using water and methanol containing 0.05% perfluoropentanoic acid and 0.1% formic acid (FA). Another aliquot was also analyzed using acidic positive compounds but was optimized for hydrophobic compounds. In this method, the extract was gradient-eluted using methanol, acetonitrile, water, 0.05% perfluoropentanoic acid, and 0.01% FA. Another aliquot was analyzed using basic negative ion optimized conditions. The basic extracts were eluted using methanol and water along with 6.5 mM ammonium bicarbonate at pH 8. The fourth aliquot was analyzed via negative ionization after elution using a gradient of water and acetonitrile with 10 mM ammonium formate at pH 10.8. Raw data were extracted and peak-identified using Metabolon’s Laboratory Information Management System. Metabolon maintains a library based on authenticated standards that contains the retention time/index, mass-to-charge ratio, and chromatographic data on all molecules present in the library. Biochemical identifications are based on retention index within a narrow window of the proposed identification, accurate mass match to the library within 10 ppm, and the spectral data between the experimental data and the authentic standards. Peaks were quantified using the area under the curve. Data were batch-normalized to adjust for day-to-day variation. Data are reported as scaled intensity where the value of each metabolite is normalized by dividing by the median value.

## Supplementary Material

Reviewer comments

## Data Availability

RNA-sequencing data have been and can be visualized at https://cells.ucsc.edu/?ds=bal-ams-cf. The full metabolomics data are being used as part of a separate study and will be deposited once that study is complete. In the interim, these data are available upon request by emailing Alix.Ashare@hitchcock.org.
