# Supplementary material for: Alveolar macrophage subtypes express cholesterol and inflammation genes in cystic fibrosis
Source: Life Sci Alliance. 2026 Apr 30;9(7):e202503482. doi: 10.26508/lsa.202503482 (PMC13139742; doi:10.26508/lsa.202503482)
Supplement: Supplementary file 3 [file LSA-2025-03482_TableS3.pdf]

Supplemental Data: DEGs shown in Figure 3F

	p_val	avg_log2FC	pct.1	pct.2	p_val_adj	gene
SERPINE1	0	2.28346162	0.088	0.02	0	SERPINE1
SHROOM3	0	1.33731936	0.242	0.113	0	SHROOM3
TMEM176B	0	1.1930429	0.472	0.28	0	TMEM176B
PRSS21	0	1.0938455	0.322	0.19	0	PRSS21
TMEM176A	0	0.99616962	0.295	0.165	0	TMEM176A
EIF1AY	0	0.95918969	0.309	0.166	0	EIF1AY
DDX3Y	0	0.9240723	0.386	0.21	0	DDX3Y
CTSW	0	0.75818708	0.549	0.401	0	CTSW
CLEC4E	0	0.68950049	0.536	0.414	0	CLEC4E
PRDX2	0	0.6647853	0.57	0.437	0	PRDX2
ANPEP	0	0.59585164	0.795	0.723	0	ANPEP
CMTM3	0	0.50954617	0.784	0.712	0	CMTM3
FDFT1	0	0.50049299	0.79	0.709	0	FDFT1
ERAP1	0	0.4868941	0.858	0.796	0	ERAP1
SIGLEC14	0	0.3814366	0.817	0.73	0	SIGLEC14
FABP4	0	0.26525854	0.999	0.997	0	FABP4
EVL	0	0.25406434	0.993	0.993	0	EVL
SERPING1	0	0.24042749	0.999	0.997	0	SERPING1
CST3	0	0.1960931	1	1	0	CST3
GLIPR2	1.286E-304	0.18747785	0.999	1	1.976E-300	GLIPR2
COL1A1	3.154E-285	5.54096303	0.047	0.004	4.847E-281	COL1A1
CLEC6A	9.306E-270	1.00162945	0.383	0.276	1.43E-265	CLEC6A
FBN1	6.18E-268	1.2468879	0.245	0.143	9.5E-264	FBN1
IAH1	2.141E-267	0.55652936	0.576	0.476	3.291E-263	IAH1
MS4A7	2.059E-264	0.15842406	1	1	3.165E-260	MS4A7
SECTM1	5.194E-260	0.3661195	0.819	0.74	7.984E-256	SECTM1
MMP14	2.567E-255	0.55242002	0.683	0.603	3.946E-251	MMP14
PLAAT3	8.807E-251	0.24849685	0.95	0.932	1.354E-246	PLAAT3
MID2	1.267E-242	1.56611002	0.109	0.041	1.947E-238	MID2
CLEC4D	1.047E-235	1.22161988	0.177	0.092	1.61E-231	CLEC4D
ITGB2	1.68E-231	0.18677796	0.995	0.994	2.582E-227	ITGB2
RELB	4.734E-229	1.41133559	0.176	0.094	7.277E-225	RELB
IL7R	1.672E-223	0.34570242	0.769	0.672	2.57E-219	IL7R
DGAT2	4.418E-217	0.68169226	0.357	0.25	6.791E-213	DGAT2
CD101	2.901E-207	0.45431013	0.657	0.58	4.46E-203	CD101
MGST3	1.093E-202	0.12913242	0.999	0.999	1.68E-198	MGST3
LRPAP1	3.055E-202	0.13761264	0.999	0.999	4.695E-198	LRPAP1
CD82	1.146E-201	0.39190738	0.839	0.82	1.761E-197	CD82
C1QA	6.786E-191	0.11126128	1	1	1.043E-186	C1QA
CHPF	8.773E-189	0.99038351	0.182	0.103	1.349E-184	CHPF
ALAS1	1.895E-186	0.26635289	0.936	0.926	2.913E-182	ALAS1
FAM107B	1.008E-183	0.26256437	0.899	0.879	1.549E-179	FAM107B
CD52	9.788E-181	0.14170605	1	0.999	1.505E-176	CD52
TMEM173	2.343E-180	0.19548817	0.981	0.979	3.602E-176	TMEM173
TNNT1	1.851E-177	0.91409475	0.215	0.133	2.845E-173	TNNT1
RBM3	2.288E-177	0.24370532	0.885	0.86	3.516E-173	RBM3
LMNA	3.968E-177	0.19490867	0.998	0.996	6.099E-173	LMNA
CYBA	4.132E-176	0.10903071	1	1	6.351E-172	CYBA

ANXA6	4.686E-176	1.24078415	0.1	0.043	7.202E-172	ANXA6
SNAI3	6.516E-176	0.62254567	0.373	0.279	1.002E-171	SNAI3
RAB38	3.816E-174	0.9332121	0.304	0.222	5.866E-170	RAB38
HPGD	5.988E-174	0.36946348	0.835	0.8	9.204E-170	HPGD
GNAI1	9.038E-169	1.35057616	0.119	0.059	1.389E-164	GNAI1
C3	1.718E-165	1.02109048	0.149	0.081	2.641E-161	C3
CXCL8	5.668E-163	1.5410627	0.121	0.061	8.712E-159	CXCL8
SLC7A7	6.376E-158	0.12778031	0.998	0.999	9.801E-154	SLC7A7
UBA52	2.533E-154	0.14135515	0.988	0.985	3.894E-150	UBA52
PTGER3	6.817E-151	0.65105197	0.344	0.257	1.048E-146	PTGER3
ECH1	1.755E-148	0.14481881	0.992	0.989	2.698E-144	ECH1
AMIGO2	3.316E-148	0.17901497	0.954	0.943	5.098E-144	AMIGO2
FADS1	2.204E-147	0.66616548	0.362	0.279	3.387E-143	FADS1
MYDGF	2.59E-146	0.14252198	0.988	0.986	3.982E-142	MYDGF
SNX2	8.517E-144	0.18110876	0.985	0.988	1.309E-139	SNX2
ANXA1	3.045E-143	0.13678359	0.999	0.999	4.68E-139	ANXA1
NOP53	3.182E-142	0.25198266	0.855	0.826	4.891E-138	NOP53
SQSTM1	1.483E-140	0.19059718	0.991	0.991	2.28E-136	SQSTM1
VSIG4	5.705E-140	0.15883403	0.992	0.99	8.77E-136	VSIG4
SRC	1.084E-139	0.26025656	0.857	0.842	1.667E-135	SRC
NQO2	4.716E-137	0.35262516	0.752	0.719	7.249E-133	NQO2
SQOR	1.474E-136	0.27889881	0.889	0.888	2.266E-132	SQOR
ACTA2	8.957E-135	0.87290246	0.184	0.117	1.377E-130	ACTA2
H3F3A	2.362E-133	0.12508003	0.996	0.995	3.63E-129	H3F3A
NEIL2	5.809E-133	0.35440668	0.581	0.508	8.929E-129	NEIL2
SFXN3	7.484E-132	0.26333071	0.78	0.741	1.15E-127	SFXN3
CCNG2	1.909E-131	0.42966175	0.534	0.464	2.935E-127	CCNG2
RTN4	4.707E-131	0.11851212	0.998	0.997	7.235E-127	RTN4
PGD	6.569E-131	0.12584915	0.996	0.996	1.01E-126	PGD
APOL4	8.712E-131	0.2823626	0.709	0.631	1.339E-126	APOL4
SPNS1	4.06E-130	0.23388583	0.875	0.848	6.241E-126	SPNS1
PLA2G7	5.678E-130	2.08823777	0.053	0.019	8.727E-126	PLA2G7
HEBP2	8.156E-130	0.29470627	0.724	0.679	1.254E-125	HEBP2
COL6A1	9.058E-129	1.14251011	0.128	0.072	1.392E-124	COL6A1
TIMP1	4.58E-128	0.27144437	0.842	0.816	7.039E-124	TIMP1
PACSIN3	9.027E-128	6.09124804	0.017	0	1.387E-123	PACSIN3
CD5L	1.09E-127	0.37437213	0.672	0.615	1.676E-123	CD5L
BANK1	4.63E-125	1.35687675	0.064	0.026	7.117E-121	BANK1
ZCCHC24	4.683E-125	0.4171234	0.573	0.515	7.198E-121	ZCCHC24
S100A13	6.245E-125	0.18754879	0.949	0.954	9.599E-121	S100A13
CDCA7L	9.19E-125	0.47956121	0.373	0.292	1.413E-120	CDCA7L
IQSEC3	2.871E-124	0.38181139	0.562	0.492	4.413E-120	IQSEC3
GRHL1	3.424E-124	0.8628932	0.171	0.109	5.263E-120	GRHL1
ANXA4	1.197E-123	0.19117582	0.936	0.923	1.84E-119	ANXA4
CTSH	1.655E-123	0.10726538	1	1	2.544E-119	CTSH
COL9A2	1.114E-122	0.53634171	0.335	0.257	1.712E-118	COL9A2
COL6A2	5.273E-120	2.11780945	0.048	0.016	8.105E-116	COL6A2
NFKB2	3.613E-118	0.87466826	0.218	0.154	5.554E-114	NFKB2
EEF2	4.814E-116	0.12367525	0.995	0.994	7.399E-112	EEF2
SPAG7	7.113E-116	0.24630037	0.798	0.766	1.093E-111	SPAG7
PTPN1	7.495E-115	0.28955376	0.86	0.844	1.152E-110	PTPN1
MCMBP	1.078E-114	0.21017437	0.849	0.831	1.656E-110	MCMBP

KCNE3	4.949E-113	0.25159978	0.839	0.818	7.608E-109	KCNE3
IFITM3	8.099E-113	0.34786414	0.614	0.555	1.245E-108	IFITM3
PLTP	1.072E-112	0.83502787	0.372	0.306	1.648E-108	PLTP
TWF2	1.124E-112	0.13930845	0.985	0.981	1.728E-108	TWF2
AGPAT2	1.633E-112	0.13797689	0.977	0.975	2.51E-108	AGPAT2
MARCKS	1.921E-109	1.57255127	0.079	0.039	2.953E-105	MARCKS
LPAR1	3.304E-108	0.61886346	0.292	0.224	5.079E-104	LPAR1
SPIRE2	5.419E-108	0.45914118	0.404	0.332	8.33E-104	SPIRE2
LST1	3.199E-106	0.16133041	0.949	0.942	4.918E-102	LST1
HHIPL1	8.664E-106	5.51029226	0.014	0	1.332E-101	HHIPL1
MYH14	2.94E-105	2.40946503	0.031	0.008	4.519E-101	MYH14
PLAUR	4.007E-105	0.15178596	0.998	0.997	6.159E-101	PLAUR
PKDCC	5.398E-105	0.91304189	0.17	0.112	8.298E-101	PKDCC
LMO2	6.606E-105	0.30253945	0.693	0.653	1.015E-100	LMO2
C8B	9.422E-105	0.30771372	0.763	0.743	1.448E-100	C8B
DHRS9	5.478E-104	0.76042975	0.195	0.134	8.421E-100	DHRS9
SLC12A7	6.942E-104	0.2503209	0.788	0.761	1.067E-99	SLC12A7
UCP2	1.346E-103	0.15130552	0.987	0.984	2.069E-99	UCP2
PDGFD	2.192E-103	0.49088825	0.399	0.323	3.369E-99	PDGFD
C15orf48	2.454E-102	1.15392493	0.192	0.135	3.7714E-98	C15orf48
TGFBI	4.264E-102	0.12677466	0.992	0.99	6.5545E-98	TGFBI
ZYX	4.698E-102	0.10897625	0.997	0.994	7.2212E-98	ZYX
SOD2	5.889E-102	0.35663133	0.852	0.841	9.0514E-98	SOD2
PAPSS1	8.471E-101	0.18004429	0.913	0.9	1.302E-96	PAPSS1
CXCL5	1.21E-99	0.97637567	0.332	0.266	1.8596E-95	CXCL5
CIRBP	1.6117E-97	0.15273074	0.944	0.934	2.4773E-93	CIRBP
RNASE4	8.2637E-97	0.48700069	0.328	0.259	1.2702E-92	RNASE4
CDK18	8.7357E-97	0.28700116	0.639	0.588	1.3428E-92	CDK18
DOK2	9.7751E-97	0.1110556	0.996	0.996	1.5025E-92	DOK2
LIPA	8.3783E-95	0.19911821	0.974	0.971	1.2878E-90	LIPA
COX4I1	1.8789E-93	0.15705602	0.931	0.923	2.8881E-89	COX4I1
NEK6	5.3905E-91	0.14213448	0.967	0.965	8.2857E-87	NEK6
LFNG	5.7654E-90	0.31808644	0.566	0.507	8.862E-86	LFNG
EHD1	6.1188E-90	0.42188906	0.548	0.501	9.4051E-86	EHD1
FBN2	9.739E-88	4.76356878	0.012	0.001	1.497E-83	FBN2
GYPC	1.8417E-86	0.12898928	0.972	0.969	2.8308E-82	GYPC
DHRS4	4.5537E-86	0.75083623	0.147	0.098	6.9994E-82	DHRS4
NCEH1	6.1346E-86	0.13157442	0.984	0.984	9.4295E-82	NCEH1
WDR6	6.9667E-85	0.41153224	0.366	0.304	1.0709E-80	WDR6
KBTBD11	6.6944E-84	0.70988182	0.148	0.098	1.029E-79	KBTBD11
LDLR	1.2996E-83	0.85555031	0.172	0.121	1.9976E-79	LDLR
TAPBP	5.4961E-81	0.13519127	0.99	0.99	8.4481E-77	TAPBP
DUSP27	1.2704E-80	0.56254588	0.196	0.141	1.9528E-76	DUSP27
UTY	3.0639E-80	0.46839387	0.197	0.141	4.7095E-76	UTY
EFHD2	7.7693E-80	0.11653721	0.999	0.999	1.1942E-75	EFHD2
ATP5F1D	8.9484E-80	0.21998949	0.753	0.725	1.3755E-75	ATP5F1D
NRP1	9.0167E-80	0.19489252	0.892	0.884	1.386E-75	NRP1
KDM6B	9.9343E-79	0.25361432	0.708	0.675	1.527E-74	KDM6B
CDC42EP3	1.4564E-78	0.1986659	0.867	0.855	2.2387E-74	CDC42EP3
VLDLR	9.6067E-78	1.01836324	0.069	0.036	1.4766E-73	VLDLR
TNFAIP3	1.2214E-76	0.34377119	0.561	0.517	1.8774E-72	TNFAIP3
ITGA4	5.5759E-76	0.35647788	0.517	0.471	8.5707E-72	ITGA4

TOMM7	6.8827E-76	0.12074595	0.966	0.96	1.0579E-71	TOMM7
FAS	8.0409E-76	0.53919147	0.226	0.171	1.236E-71	FAS
GPSM3	7.429E-75	0.11451912	0.979	0.977	1.1419E-70	GPSM3
MAFF	3.9902E-73	0.4566529	0.376	0.324	6.1334E-69	MAFF
LMO4	4.1283E-73	0.28010134	0.809	0.8	6.3456E-69	LMO4
SLCO3A1	6.8159E-71	0.30623193	0.562	0.52	1.0477E-66	SLCO3A1
H6PD	6.3582E-70	0.2898534	0.57	0.528	9.7731E-66	H6PD
CHST12	9.7976E-70	0.26381226	0.591	0.546	1.506E-65	CHST12
CD36	1.2728E-69	0.52467779	0.365	0.308	1.9564E-65	CD36
GPA33	4.4095E-69	0.2410328	0.517	0.456	6.7778E-65	GPA33
OPTN	4.544E-69	0.25787342	0.761	0.747	6.9846E-65	OPTN
FERMT3	1.7226E-68	0.11308732	0.98	0.978	2.6478E-64	FERMT3
RAB7B	7.9564E-68	0.25354615	0.705	0.677	1.223E-63	RAB7B
SLC25A6	8.6625E-68	0.17726727	0.82	0.798	1.3315E-63	SLC25A6
SPOCD1	1.0827E-67	0.53645943	0.398	0.348	1.6643E-63	SPOCD1
CPVL	1.6447E-67	0.12163176	0.967	0.963	2.5281E-63	CPVL
SF3B5	3.9332E-67	0.14645308	0.903	0.89	6.0458E-63	SF3B5
HLX	5.0374E-67	0.57806611	0.193	0.145	7.743E-63	HLX
SIGLEC12	6.3063E-66	0.9327677	0.194	0.151	9.6934E-62	SIGLEC12
ABHD2	4.6879E-65	0.16639058	0.914	0.906	7.2057E-61	ABHD2
TBC1D10C	5.1459E-65	0.18281262	0.83	0.814	7.9098E-61	TBC1D10C
RUNX2	5.0678E-64	0.93471828	0.072	0.041	7.7898E-60	RUNX2
MAF	9.0415E-64	0.23380796	0.712	0.682	1.3898E-59	MAF
FADS2	1.3971E-63	0.62389459	0.241	0.192	2.1475E-59	FADS2
RCAN2	6.9734E-63	1.56820878	0.032	0.013	1.0719E-58	RCAN2
ACSL1	1.2487E-62	0.30229901	0.883	0.884	1.9194E-58	ACSL1
NFKBIE	1.535E-62	0.37915195	0.462	0.42	2.3594E-58	NFKBIE
TLE5	1.9784E-62	0.1244628	0.945	0.942	3.041E-58	TLE5
SEC11A	2.6848E-62	0.1038794	0.988	0.988	4.1269E-58	SEC11A
INF2	1.1369E-61	0.12444398	0.967	0.962	1.7476E-57	INF2
BCAR1	1.2702E-61	0.21183526	0.689	0.64	1.9525E-57	BCAR1
MTPN	2.1742E-61	0.12436951	0.937	0.936	3.3419E-57	MTPN
FUOM	5.1258E-61	0.16621775	0.827	0.81	7.8788E-57	FUOM
STX11	1.8646E-60	0.12654558	0.968	0.966	2.8661E-56	STX11
SH3BP1	2.7591E-60	0.16108632	0.847	0.826	4.241E-56	SH3BP1
TRPC6	2.8291E-60	0.30759413	0.552	0.517	4.3486E-56	TRPC6
KDM5D	3.5289E-59	0.42285965	0.18	0.133	5.4243E-55	KDM5D
ST3GAL2	4.8403E-59	0.17228991	0.848	0.834	7.4401E-55	ST3GAL2
NINJ2	1.0147E-58	0.33321239	0.38	0.328	1.5597E-54	NINJ2
CEACAM4	3.9142E-58	0.62805474	0.098	0.064	6.0166E-54	CEACAM4
MT-ND6	2.1534E-57	0.6127473	0.166	0.124	3.3101E-53	MT-ND6
SSR2	6.0098E-57	0.13456179	0.879	0.868	9.2377E-53	SSR2
SYNGR1	1.0075E-56	0.64036532	0.247	0.205	1.5487E-52	SYNGR1
ATP5MC2	1.4109E-56	0.13026095	0.905	0.903	2.1687E-52	ATP5MC2
MFAP5	1.3359E-55	1.06549463	0.057	0.032	2.0533E-51	MFAP5
PLBD2	5.4608E-55	0.15797071	0.833	0.815	8.3938E-51	PLBD2
BIRC7	1.3963E-54	1.57181133	0.031	0.014	2.1462E-50	BIRC7
ADCY5	2.087E-54	2.65883183	0.012	0.002	3.2079E-50	ADCY5
FNDC3B	3.8278E-54	0.12552575	0.942	0.94	5.8837E-50	FNDC3B
CYP51A1	5.0984E-54	0.41233909	0.468	0.432	7.8367E-50	CYP51A1
EIF4E3	5.4229E-54	0.82812331	0.077	0.048	8.3356E-50	EIF4E3
CALHM2	6.5094E-54	0.24196395	0.634	0.604	1.0006E-49	CALHM2

FAM20C	2.8815E-53	0.3568732	0.339	0.289	4.4291E-49	FAM20C
GOLGA7B	3.435E-53	0.78930731	0.082	0.052	5.28E-49	GOLGA7B
PLD3	7.8075E-53	0.16781762	0.99	0.99	1.2001E-48	PLD3
SLC25A19	9.7705E-53	0.24137982	0.651	0.631	1.5018E-48	SLC25A19
GPX3	1.1677E-52	0.37351383	0.382	0.336	1.7948E-48	GPX3
TRIR	1.6605E-52	0.10732642	0.95	0.948	2.5523E-48	TRIR
DUSP6	2.4348E-52	0.42795414	0.314	0.269	3.7425E-48	DUSP6
PPDPF	4.7831E-52	0.11755468	0.929	0.909	7.3521E-48	PPDPF
STOM	6.289E-52	0.20885188	0.913	0.92	9.6669E-48	STOM
PDE6G	6.9079E-52	0.43080184	0.272	0.226	1.0618E-47	PDE6G
C18orf54	9.1209E-52	0.89568799	0.059	0.034	1.402E-47	C18orf54
TNIP1	3.1042E-51	0.27438068	0.53	0.495	4.7715E-47	TNIP1
ZFY	3.6202E-51	0.77533013	0.071	0.043	5.5647E-47	ZFY
NDUFS5	1.2951E-50	0.16841929	0.753	0.736	1.9906E-46	NDUFS5
PNPLA2	2.0896E-48	0.10698549	0.976	0.975	3.212E-44	PNPLA2
FAM162A	3.0436E-48	0.17293812	0.77	0.756	4.6784E-44	FAM162A
NEO1	3.1136E-48	0.68105826	0.094	0.064	4.7859E-44	NEO1
PCED1B	3.9786E-48	0.19985551	0.697	0.676	6.1155E-44	PCED1B
YBEY	4.3764E-48	0.5162008	0.151	0.113	6.727E-44	YBEY
PTPRE	4.3778E-48	0.36238606	0.429	0.391	6.7291E-44	PTPRE
SNHG32	1.368E-47	0.22242953	0.569	0.533	2.1027E-43	SNHG32
RAP2A	3.0301E-47	0.18532066	0.735	0.718	4.6575E-43	RAP2A
SMPD2	4.6428E-47	0.42323707	0.249	0.205	7.1365E-43	SMPD2
BCAR3	5.1505E-47	0.21716406	0.607	0.58	7.9168E-43	BCAR3
SASH1	6.1903E-47	0.28892173	0.414	0.371	9.5151E-43	SASH1
MGMT	9.1423E-47	0.20312846	0.589	0.554	1.4053E-42	MGMT
DLD	9.9144E-47	0.13069069	0.877	0.869	1.5239E-42	DLD
ITGB8	1.0453E-46	0.33172856	0.706	0.689	1.6067E-42	ITGB8
TDRD3	1.1041E-46	0.26276084	0.477	0.434	1.6971E-42	TDRD3
EGFL7	1.2606E-46	0.55158866	0.147	0.11	1.9377E-42	EGFL7
ETS2	2.0334E-46	0.1720784	0.768	0.747	3.1256E-42	ETS2
SCCPDH	3.4495E-46	0.13660802	0.921	0.92	5.3022E-42	SCCPDH
CLEC12A	3.5244E-46	0.15376474	0.705	0.675	5.4174E-42	CLEC12A
RPIA	6.7532E-46	0.22399977	0.575	0.543	1.038E-41	RPIA
GBP2	4.9118E-45	0.13731635	0.84	0.825	7.5499E-41	GBP2
AQP3	6.4681E-45	0.1544121	0.911	0.893	9.9421E-41	AQP3
CYTIP	6.957E-45	0.19598351	0.866	0.868	1.0694E-40	CYTIP
KCTD5	3.0316E-44	0.1965246	0.71	0.692	4.6598E-40	KCTD5
ARHGEF2	2.9409E-43	0.12602806	0.904	0.902	4.5205E-39	ARHGEF2
DEGS1	1.0277E-42	0.14940709	0.867	0.87	1.5797E-38	DEGS1
IL10RB	1.0334E-42	0.1301823	0.867	0.862	1.5885E-38	IL10RB
PYGL	1.5212E-42	0.13185817	0.82	0.805	2.3382E-38	PYGL
CPED1	1.9847E-42	0.14654578	0.863	0.87	3.0507E-38	CPED1
AGTRAP	2.0258E-42	0.14157881	0.822	0.814	3.1138E-38	AGTRAP
TAGLN2	2.2572E-42	0.12687712	0.909	0.897	3.4696E-38	TAGLN2
RAB34	2.4715E-42	0.17004126	0.676	0.651	3.7989E-38	RAB34
RASSF5	4.647E-42	0.10973112	0.935	0.935	7.143E-38	RASSF5
SDC4	8.8345E-42	0.42449576	0.406	0.375	1.358E-37	SDC4
KCNMA1	1.0027E-41	0.25797755	0.322	0.275	1.5413E-37	KCNMA1
NFKBIA	1.3007E-41	0.16347552	0.95	0.956	1.9993E-37	NFKBIA
DHCR7	1.5017E-41	0.53643147	0.195	0.158	2.3083E-37	DHCR7
UBXN1	1.5132E-41	0.1679085	0.751	0.739	2.3259E-37	UBXN1

ZNF215	1.6226E-41	0.86391266	0.053	0.032	2.4941E-37	ZNF215
PPP1R18	1.9376E-41	0.10964185	0.929	0.924	2.9782E-37	PPP1R18
SRI	2.6456E-41	0.1179534	0.9	0.891	4.0666E-37	SRI
KIFC3	2.8984E-41	0.42132772	0.192	0.154	4.4551E-37	KIFC3
INPP5F	5.6636E-41	0.25019139	0.483	0.447	8.7055E-37	INPP5F
CNOT1	7.7919E-41	0.34777039	0.237	0.196	1.1977E-36	CNOT1
NMB	8.6336E-41	0.38093048	0.508	0.477	1.3271E-36	NMB
TMEM179B	9.5856E-41	0.12103845	0.852	0.842	1.4734E-36	TMEM179B
MVD	1.62E-40	0.40435068	0.323	0.286	2.4901E-36	MVD
SIGIRR	2.3064E-40	0.43315702	0.187	0.15	3.5452E-36	SIGIRR
PLXNB2	2.5961E-40	0.11604486	0.936	0.936	3.9904E-36	PLXNB2
H1F0	4.2845E-40	0.29319326	0.46	0.419	6.5856E-36	H1F0
ADGRE2	5.2769E-40	0.50501879	0.174	0.138	8.1112E-36	ADGRE2
COX7C	5.7358E-40	0.11255523	0.87	0.864	8.8166E-36	COX7C
LIMA1	6.7053E-40	0.13305734	0.825	0.814	1.0307E-35	LIMA1
ACVRL1	7.6852E-40	0.1390041	0.773	0.752	1.1813E-35	ACVRL1
GBP5	8.5815E-40	0.3147078	0.402	0.365	1.3191E-35	GBP5
S100P	3.6297E-39	0.38928434	0.287	0.246	5.5792E-35	S100P
SLC9A7	4.0317E-39	0.36857399	0.249	0.21	6.1972E-35	SLC9A7
TOMM20	4.4225E-39	0.12634131	0.813	0.796	6.7978E-35	TOMM20
PRPS2	5.5281E-39	0.22576468	0.526	0.494	8.4973E-35	PRPS2
SQLE	1.2905E-38	0.75226391	0.066	0.043	1.9836E-34	SQLE
CCDC88C	1.6583E-38	0.82020581	0.051	0.031	2.5489E-34	CCDC88C
KPTN	2.8448E-38	0.31382798	0.356	0.319	4.3727E-34	KPTN
MSMO1	3.5621E-38	0.42855167	0.352	0.32	5.4753E-34	MSMO1
ZFAND3	4.0758E-38	0.11287634	0.893	0.893	6.265E-34	ZFAND3
COA6	7.1122E-38	0.18021083	0.655	0.636	1.0932E-33	COA6
SMDT1	1.7322E-37	0.14940784	0.768	0.755	2.6626E-33	SMDT1
TRAPPC6A	6.0951E-37	0.19716166	0.594	0.569	9.3688E-33	TRAPPC6A
IGBP1	7.3808E-37	0.16579096	0.71	0.695	1.1345E-32	IGBP1
EIF3L	1.657E-36	0.13181664	0.818	0.811	2.5469E-32	EIF3L
SLC25A43	3.2715E-36	0.23319199	0.484	0.452	5.0287E-32	SLC25A43
CTSL	3.3935E-36	0.14164024	0.997	0.997	5.2161E-32	CTSL
FHL3	3.8198E-36	0.36548809	0.237	0.199	5.8714E-32	FHL3
ZNF358	4.2405E-36	0.26563773	0.373	0.334	6.518E-32	ZNF358
PFKP	5.1268E-36	0.26444713	0.44	0.405	7.8805E-32	PFKP
INHBA	6.23E-36	0.10070999	0.979	0.982	9.5761E-32	INHBA
GSR	6.5211E-36	0.16124797	0.773	0.765	1.0024E-31	GSR
RBM23	6.8374E-36	0.14155414	0.777	0.761	1.051E-31	RBM23
SYF2	9.4037E-36	0.17009206	0.661	0.642	1.4454E-31	SYF2
ETHE1	1.5132E-35	0.12332696	0.876	0.873	2.326E-31	ETHE1
HCK	1.6291E-35	0.11314604	0.994	0.994	2.5041E-31	HCK
KCNA3	1.8555E-35	0.15060874	0.732	0.717	2.852E-31	KCNA3
NPRL3	2.0406E-35	0.22720508	0.477	0.445	3.1367E-31	NPRL3
USP9Y	2.133E-35	0.51984427	0.083	0.058	3.2786E-31	USP9Y
STIM1	3.8932E-35	0.10339655	0.921	0.918	5.9843E-31	STIM1
PHLDA3	4.6956E-35	0.11614183	0.894	0.895	7.2176E-31	PHLDA3
SIGLEC11	5.6309E-35	0.21258866	0.5	0.466	8.6552E-31	SIGLEC11
FLT1	5.6528E-35	0.95344212	0.054	0.034	8.6889E-31	FLT1
ENPP4	6.7296E-35	0.10680515	0.92	0.927	1.0344E-30	ENPP4
TMSB4Y	8.3581E-35	1.10718	0.027	0.013	1.2847E-30	TMSB4Y
PTX3	9.7151E-35	0.83266949	0.038	0.022	1.4933E-30	PTX3

PRMT2	9.9107E-35	0.11413535	0.844	0.833	1.5234E-30	PRMT2
MKNK1	1.3467E-34	0.11631173	0.864	0.854	2.0699E-30	MKNK1
CD33	2.1493E-34	0.18369362	0.583	0.558	3.3037E-30	CD33
PRXL2A	2.92E-34	0.35660487	0.359	0.328	4.4884E-30	PRXL2A
CSF1	3.4068E-34	0.37094367	0.538	0.513	5.2366E-30	CSF1
VPS28	4.6532E-34	0.12194164	0.793	0.785	7.1524E-30	VPS28
LACC1	5.6041E-34	0.34484735	0.288	0.254	8.6141E-30	LACC1
ATP5MC3	1.1176E-33	0.10255636	0.899	0.894	1.7179E-29	ATP5MC3
C7orf50	1.4142E-33	0.14253697	0.779	0.77	2.1738E-29	C7orf50
NBPF3	1.7995E-33	0.69311827	0.053	0.034	2.766E-29	NBPF3
G6PD	5.8732E-33	0.10003134	0.939	0.937	9.0277E-29	G6PD
UFC1	1.1747E-32	0.10006388	0.91	0.91	1.8056E-28	UFC1
CCDC124	1.4233E-32	0.13007931	0.781	0.768	2.1878E-28	CCDC124
EFNB1	1.9763E-32	0.1690454	0.619	0.594	3.0377E-28	EFNB1
G0S2	2.091E-32	0.35579635	0.131	0.101	3.2141E-28	G0S2
LMCD1	2.5591E-32	1.74389571	0.012	0.004	3.9336E-28	LMCD1
RTN1	3.639E-32	0.11421664	0.843	0.83	5.5935E-28	RTN1
HILPDA	3.9158E-32	0.34257612	0.264	0.229	6.019E-28	HILPDA
OPRK1	6.038E-32	0.51054701	0.091	0.067	9.2811E-28	OPRK1
CSTA	9.9217E-32	0.10043059	0.958	0.966	1.5251E-27	CSTA
KYNU	1.0574E-31	0.17392073	0.757	0.75	1.6253E-27	KYNU
CCL20	1.0833E-31	1.09851118	0.037	0.022	1.6651E-27	CCL20
ELMSAN1	1.8212E-31	0.13089593	0.805	0.796	2.7993E-27	ELMSAN1
HNMT	2.1498E-31	0.12386643	0.771	0.76	3.3045E-27	HNMT
SH3PXD2B	2.2893E-31	0.39076344	0.25	0.216	3.5188E-27	SH3PXD2B
NSA2	2.375E-31	0.14995371	0.651	0.63	3.6506E-27	NSA2
CR1	4.9949E-31	0.14711465	0.523	0.488	7.6777E-27	CR1
IFNGR2	5.4316E-31	0.10913468	0.943	0.949	8.3489E-27	IFNGR2
ADCY6	6.0975E-31	0.85506614	0.044	0.027	9.3725E-27	ADCY6
CKLF	1.0879E-30	0.14096498	0.8	0.795	1.6722E-26	CKLF
LETMD1	2.7262E-30	0.19488894	0.505	0.477	4.1904E-26	LETMD1
A4GALT	3.1064E-30	0.25211716	0.35	0.312	4.7748E-26	A4GALT
SLCO4C1	4.2637E-30	0.97921175	0.028	0.015	6.5538E-26	SLCO4C1
IL4I1	5.3959E-30	0.64913686	0.085	0.062	8.294E-26	IL4I1
IFITM2	6.5224E-30	0.23007666	0.418	0.387	1.0026E-25	IFITM2
OMA1	1.059E-29	0.20738474	0.398	0.363	1.6278E-25	OMA1
EGR2	1.5396E-29	0.17437357	0.577	0.546	2.3665E-25	EGR2
ATF4	1.5612E-29	0.17075551	0.617	0.597	2.3997E-25	ATF4
SLC1A5	1.7966E-29	0.14357313	0.743	0.728	2.7615E-25	SLC1A5
PHLDA1	2.2298E-29	0.42230193	0.28	0.249	3.4275E-25	PHLDA1
HDGFL3	2.5408E-29	0.40480951	0.149	0.12	3.9055E-25	HDGFL3
ID2	2.7897E-29	0.13657344	0.706	0.687	4.2881E-25	ID2
NIT1	4.7713E-29	0.14912361	0.679	0.666	7.3339E-25	NIT1
AOAH	6.3354E-29	0.20093921	0.49	0.461	9.7382E-25	AOAH
SLC2A6	2.7518E-28	0.29984631	0.325	0.293	4.2297E-24	SLC2A6
TRGJP2	3.7701E-28	0.28119194	0.295	0.261	5.7951E-24	TRGJP2
RPP25	8.3513E-28	0.29382245	0.372	0.344	1.2837E-23	RPP25
SEMA4D	9.3956E-28	0.22885051	0.437	0.409	1.4442E-23	SEMA4D
AATF	9.405E-28	0.12365985	0.765	0.755	1.4456E-23	AATF
ZC3H12C	1.2347E-27	1.51315305	0.013	0.005	1.8978E-23	ZC3H12C
CLEC11A	1.9122E-27	0.99788427	0.031	0.018	2.9392E-23	CLEC11A
PLA2R1	2.182E-27	0.66607426	0.059	0.041	3.3539E-23	PLA2R1

BAHCC1	2.4377E-27	0.57857909	0.067	0.047	3.7469E-23	BAHCC1
SLFN5	2.5287E-27	0.18888292	0.527	0.5	3.8869E-23	SLFN5
TMEM91	3.2885E-27	0.2948851	0.289	0.257	5.0547E-23	TMEM91
IL1RN	4.8533E-27	0.21688886	0.76	0.752	7.46E-23	IL1RN
TMEM273	5.24E-27	0.14608701	0.823	0.825	8.0545E-23	TMEM273
KLF2	8.7315E-27	0.15354948	0.711	0.693	1.3421E-22	KLF2
CCDC170	1.3526E-26	0.29255943	0.216	0.184	2.0791E-22	CCDC170
FBXL15	1.6582E-26	0.19779429	0.514	0.491	2.5489E-22	FBXL15
AGRP	1.7937E-26	0.16921645	0.725	0.717	2.7571E-22	AGRP
TMPRSS11E	2.382E-26	0.94807016	0.03	0.017	3.6613E-22	TMPRSS11E
NDUFA8	2.7228E-26	0.11496812	0.777	0.766	4.1853E-22	NDUFA8
CD69	3.1751E-26	0.39014019	0.433	0.413	4.8805E-22	CD69
GATA3	4.4332E-26	0.33820826	0.198	0.168	6.8143E-22	GATA3
ACSM1	5.181E-26	0.71613756	0.042	0.027	7.9637E-22	ACSM1
ACSL4	7.4334E-26	0.1153513	0.757	0.749	1.1426E-21	ACSL4
FDX1	8.5693E-26	0.19209936	0.63	0.618	1.3172E-21	FDX1
SMOX	9.2563E-26	0.92596633	0.027	0.015	1.4228E-21	SMOX
PINLYP	1.4301E-25	0.50500981	0.103	0.08	2.1982E-21	PINLYP
CNPPD1	1.5092E-25	0.11322783	0.82	0.813	2.3198E-21	CNPPD1
VNN1	2.2494E-25	0.71184601	0.037	0.023	3.4575E-21	VNN1
RBP7	2.2722E-25	0.21216612	0.378	0.346	3.4927E-21	RBP7
HOXC4	2.5046E-25	0.82006016	0.032	0.019	3.8498E-21	HOXC4
LEPROT	2.7598E-25	0.1122432	0.8	0.797	4.2421E-21	LEPROT
NFE2L1	3.0445E-25	0.10326521	0.866	0.857	4.6797E-21	NFE2L1
STAP1	3.135E-25	0.33461783	0.107	0.083	4.8188E-21	STAP1
PAX8	3.804E-25	0.33876744	0.176	0.148	5.8471E-21	PAX8
RASGEF1A	4.0422E-25	0.50373552	0.086	0.065	6.2132E-21	RASGEF1A
GDF15	4.8785E-25	1.08510851	0.03	0.017	7.4988E-21	GDF15
RAPGEF2	5.376E-25	0.16880003	0.572	0.557	8.2635E-21	RAPGEF2
TP53	8.6631E-25	0.18091502	0.504	0.48	1.3316E-20	TP53
WFS1	9.0524E-25	0.28848537	0.294	0.264	1.3914E-20	WFS1
MILR1	9.8457E-25	0.19646384	0.412	0.38	1.5134E-20	MILR1
ZNF581	1.0009E-24	0.24718347	0.375	0.347	1.5385E-20	ZNF581
P2RX1	1.1305E-24	0.20925737	0.41	0.379	1.7377E-20	P2RX1
TMEM35B	1.3087E-24	0.1072796	0.823	0.821	2.0116E-20	TMEM35B
PLEKHG6	2.013E-24	1.05246324	0.025	0.014	3.0942E-20	PLEKHG6
PNMA8A	2.4265E-24	1.20814019	0.015	0.007	3.7298E-20	PNMA8A
FAM168A	3.0566E-24	0.14615	0.588	0.569	4.6983E-20	FAM168A
KMO	3.0821E-24	0.18262542	0.638	0.623	4.7374E-20	KMO
RASSF4	3.5738E-24	0.13368646	0.656	0.639	5.4933E-20	RASSF4
CEBPA	3.6969E-24	0.19763164	0.626	0.613	5.6826E-20	CEBPA
HP	3.7556E-24	0.26406944	0.46	0.432	5.7728E-20	HP
DYSF	5.3317E-24	1.29988833	0.021	0.011	8.1954E-20	DYSF
PISD	8.4596E-24	0.18346125	0.526	0.509	1.3003E-19	PISD
SLC43A2	8.4763E-24	0.10160694	0.897	0.897	1.3029E-19	SLC43A2
PSENEN	8.5043E-24	0.13276668	0.678	0.666	1.3072E-19	PSENEN
CTNNB1	9.6221E-24	0.10029004	0.853	0.849	1.479E-19	CTNNB1
OPN3	9.8748E-24	0.12612098	0.818	0.815	1.5179E-19	OPN3
IGSF6	1.0237E-23	0.11977419	0.871	0.878	1.5735E-19	IGSF6
ASGR1	1.2296E-23	0.35665624	0.19	0.163	1.89E-19	ASGR1
MAP2K3	1.8919E-23	0.14087143	0.658	0.645	2.908E-19	MAP2K3
KCNQ1	2.1825E-23	0.11604013	0.692	0.665	3.3547E-19	KCNQ1

PRICKLE1	2.2135E-23	0.99656312	0.019	0.01	3.4023E-19	PRICKLE1
C9orf64	2.3277E-23	0.19288823	0.456	0.434	3.5779E-19	C9orf64
TMEM167B	2.3464E-23	0.1157723	0.735	0.724	3.6066E-19	TMEM167B
SEC16A	3.0542E-23	0.13040319	0.663	0.653	4.6946E-19	SEC16A
RIN2	4.1587E-23	0.13798431	0.716	0.706	6.3923E-19	RIN2
CHST7	5.0194E-23	0.44795258	0.121	0.098	7.7153E-19	CHST7
FCMR	6.7179E-23	1.34268865	0.017	0.008	1.0326E-18	FCMR
HMGCS1	7.9484E-23	0.29566223	0.366	0.342	1.2218E-18	HMGCS1
IL18	9.8121E-23	0.1405082	0.637	0.623	1.5082E-18	IL18
HPR	1.2434E-22	0.41171326	0.201	0.175	1.9112E-18	HPR
MT-CO3	1.244E-22	0.22941065	0.522	0.499	1.9122E-18	MT-CO3
RNF166	1.2589E-22	0.18585159	0.469	0.446	1.935E-18	RNF166
PDHB	1.539E-22	0.13275142	0.653	0.64	2.3655E-18	PDHB
CYC1	1.5745E-22	0.1036155	0.819	0.809	2.4201E-18	CYC1
GBP1	1.6803E-22	0.15988636	0.726	0.723	2.5828E-18	GBP1
ARL11	2.1798E-22	0.18024299	0.501	0.483	3.3506E-18	ARL11
CHST3	2.268E-22	0.50790753	0.084	0.065	3.4862E-18	CHST3
TMEM258	3.2543E-22	0.1158465	0.72	0.708	5.0022E-18	TMEM258
TSC22D1	4.2721E-22	0.37297811	0.283	0.257	6.5667E-18	TSC22D1
ENPP6	5.2075E-22	0.66560056	0.04	0.026	8.0044E-18	ENPP6
RASGRP1	5.6462E-22	1.31792775	0.013	0.006	8.6788E-18	RASGRP1
S100A16	8.5337E-22	0.40316951	0.153	0.129	1.3117E-17	S100A16
TNFAIP8L2	1.3437E-21	0.15029853	0.616	0.601	2.0653E-17	TNFAIP8L2
EFEMP2	1.4506E-21	0.89022313	0.023	0.013	2.2297E-17	EFEMP2
PGLS	1.5228E-21	0.10063771	0.83	0.831	2.3407E-17	PGLS
PRKAB1	1.7733E-21	0.18179015	0.509	0.491	2.7257E-17	PRKAB1
SLC1A3	2.027E-21	0.26202151	0.304	0.277	3.1157E-17	SLC1A3
NR4A3	2.2987E-21	0.1295971	0.465	0.535	3.5333E-17	NR4A3
TTC39C	2.4179E-21	0.20129289	0.429	0.405	3.7166E-17	TTC39C
TMEM161B	2.6193E-21	0.3148298	0.231	0.205	4.0261E-17	TMEM161B
MOGAT1	2.6285E-21	0.34957672	0.229	0.203	4.0402E-17	MOGAT1
ITGA6	2.6675E-21	0.37960332	0.141	0.117	4.1002E-17	ITGA6
IQGAP2	2.8335E-21	0.10791777	0.738	0.728	4.3554E-17	IQGAP2
FAIM	2.9173E-21	0.36727579	0.266	0.242	4.4841E-17	FAIM
HIST1H2BG	3.0366E-21	1.03106799	0.016	0.008	4.6675E-17	HIST1H2BG
OR6K3	3.1527E-21	0.47263816	0.081	0.062	4.8461E-17	OR6K3
CABLES1	5.3626E-21	0.19341204	0.421	0.396	8.2429E-17	CABLES1
CSPG4	8.1593E-21	0.25731023	0.264	0.235	1.2542E-16	CSPG4
MAFK	8.599E-21	0.19824224	0.454	0.433	1.3218E-16	MAFK
UROD	9.5167E-21	0.12100666	0.693	0.687	1.4628E-16	UROD
STK17B	1.0301E-20	0.11566633	0.826	0.829	1.5833E-16	STK17B
SDC3	1.0373E-20	0.14940455	0.423	0.39	1.5944E-16	SDC3
TNIP3	2.0831E-20	0.79976719	0.034	0.022	3.202E-16	TNIP3
NFATC2IP	2.118E-20	0.18619063	0.482	0.465	3.2556E-16	NFATC2IP
CACNA2D4	2.1551E-20	0.42649856	0.098	0.078	3.3126E-16	CACNA2D4
DLC1	2.3674E-20	0.28232777	0.297	0.272	3.639E-16	DLC1
CCDC88B	3.1321E-20	0.12261705	0.681	0.666	4.8143E-16	CCDC88B
HIP1	5.1345E-20	0.27401419	0.257	0.232	7.8923E-16	HIP1
NAMPT	8.0682E-20	0.11308621	0.859	0.862	1.2402E-15	NAMPT
PRKCZ	8.4936E-20	0.42771926	0.101	0.081	1.3055E-15	PRKCZ
CMKLR1	1.3599E-19	0.95484926	0.02	0.011	2.0903E-15	CMKLR1
USP11	1.4395E-19	0.15091635	0.556	0.54	2.2126E-15	USP11

TUSC1	1.6591E-19	0.42359659	0.098	0.079	2.5502E-15	TUSC1
TLE1	1.9223E-19	0.29909957	0.198	0.173	2.9548E-15	TLE1
ADAMTS15	1.9367E-19	0.44461147	0.119	0.098	2.977E-15	ADAMTS15
CEBPE	2.6125E-19	0.60609234	0.046	0.032	4.0157E-15	CEBPE
REPS1	3.1276E-19	0.14212807	0.607	0.594	4.8075E-15	REPS1
TEX264	3.8078E-19	0.10455439	0.777	0.776	5.8529E-15	TEX264
CBX4	3.8314E-19	0.12576038	0.68	0.67	5.8893E-15	CBX4
TGFB1	5.1363E-19	0.10157854	0.746	0.737	7.895E-15	TGFB1
CYB5A	7.0024E-19	0.11545589	0.759	0.756	1.0763E-14	CYB5A
TRIT1	7.432E-19	0.31938453	0.183	0.159	1.1424E-14	TRIT1
SH2B2	7.7971E-19	0.15555805	0.586	0.573	1.1985E-14	SH2B2
PAF1	1.0643E-18	0.11602932	0.72	0.715	1.6359E-14	PAF1
BTBD2	1.1832E-18	0.12155217	0.653	0.641	1.8188E-14	BTBD2
L3MBTL4	1.2706E-18	0.41933811	0.095	0.076	1.9531E-14	L3MBTL4
TNFAIP8	1.6956E-18	0.17273186	0.507	0.492	2.6063E-14	TNFAIP8
TNFRSF10B	2.162E-18	0.18512768	0.464	0.447	3.3232E-14	TNFRSF10B
HIST1H1D	2.255E-18	0.44801031	0.102	0.083	3.4662E-14	HIST1H1D
FN3KRP	2.6847E-18	0.16541518	0.474	0.455	4.1266E-14	FN3KRP
PRKCSH	3.2861E-18	0.1074877	0.706	0.697	5.051E-14	PRKCSH
H2AFJ	3.3032E-18	0.12348461	0.699	0.693	5.0773E-14	H2AFJ
PNPLA1	4.4403E-18	0.58619657	0.048	0.034	6.8252E-14	PNPLA1
RDH13	4.7709E-18	0.39649315	0.116	0.096	7.3334E-14	RDH13
LAMB1	5.3394E-18	0.15782123	0.297	0.267	8.2072E-14	LAMB1
CHRM3	6.3872E-18	0.28399835	0.24	0.216	9.8178E-14	CHRM3
RHOC	6.9474E-18	0.1548351	0.636	0.624	1.0679E-13	RHOC
CDC42EP2	6.9754E-18	0.52199121	0.066	0.05	1.0722E-13	CDC42EP2
PLEKHA6	8.0326E-18	0.56821757	0.056	0.042	1.2347E-13	PLEKHA6
MACC1	8.8195E-18	0.14342592	0.802	0.802	1.3556E-13	MACC1
RAB5IF	1.1539E-17	0.11957474	0.741	0.741	1.7736E-13	RAB5IF
COX20	1.1837E-17	0.24744612	0.231	0.207	1.8195E-13	COX20
ZNF217	1.4894E-17	0.10261523	0.779	0.782	2.2894E-13	ZNF217
SLC39A8	1.6776E-17	0.55284546	0.078	0.062	2.5786E-13	SLC39A8
UBTD2	1.8178E-17	0.18890375	0.465	0.449	2.7942E-13	UBTD2
ALPK3	2.5351E-17	0.22882927	0.404	0.384	3.8968E-13	ALPK3
TOP1MT	2.7115E-17	0.14754834	0.512	0.497	4.1678E-13	TOP1MT
LYST	3.1526E-17	0.13817235	0.641	0.632	4.8458E-13	LYST
PIGP	3.1828E-17	0.17274255	0.405	0.384	4.8923E-13	PIGP
SESN2	3.505E-17	0.16050173	0.666	0.663	5.3875E-13	SESN2
FCGBP	3.5609E-17	1.20076386	0.014	0.007	5.4735E-13	FCGBP
AZU1	3.7747E-17	0.4200584	0.06	0.045	5.802E-13	AZU1
FGF10	4.4174E-17	0.46866699	0.19	0.17	6.79E-13	FGF10
PTGR1	4.7312E-17	0.13988908	0.485	0.463	7.2723E-13	PTGR1
SAMM50	5.9016E-17	0.12477892	0.588	0.574	9.0714E-13	SAMM50
AEN	6.1584E-17	0.32445477	0.201	0.178	9.4661E-13	AEN
KLHL5	7.7144E-17	0.28223529	0.221	0.197	1.1858E-12	KLHL5
METRNL	8.5519E-17	0.11337233	0.893	0.902	1.3145E-12	METRNL
ATP13A4	1.2965E-16	0.93524375	0.019	0.011	1.9928E-12	ATP13A4
FHL1	2.1189E-16	0.22943458	0.265	0.242	3.257E-12	FHL1
SFXN1	2.2268E-16	0.31309439	0.133	0.113	3.4228E-12	SFXN1
RTN2	2.451E-16	0.19630255	0.364	0.343	3.7674E-12	RTN2
AOC3	2.6182E-16	0.20325136	0.279	0.253	4.0245E-12	AOC3
TSFM	3.0693E-16	0.14596082	0.499	0.483	4.7179E-12	TSFM

SLC35C2	3.2613E-16	0.12521154	0.619	0.612	5.0129E-12	SLC35C2
MFSD2A	3.265E-16	0.45748416	0.109	0.09	5.0187E-12	MFSD2A
CELA2B	3.7741E-16	0.50129766	0.217	0.199	5.8012E-12	CELA2B
UROS	4.5606E-16	0.17971328	0.403	0.384	7.0101E-12	UROS
LPIN2	5.0934E-16	0.12007919	0.593	0.579	7.8291E-12	LPIN2
TMEM159	5.1365E-16	0.184132	0.362	0.34	7.8953E-12	TMEM159
HDDC3	5.3504E-16	0.197599	0.377	0.355	8.2241E-12	HDDC3
SPTBN5	5.5118E-16	0.63506975	0.034	0.023	8.4722E-12	SPTBN5
PA2G4	6.8206E-16	0.11472941	0.639	0.632	1.0484E-11	PA2G4
TEN1	7.8398E-16	0.22301967	0.314	0.293	1.2051E-11	TEN1
PELI2	8.8411E-16	0.45793303	0.07	0.055	1.359E-11	PELI2
TRIM21	1.0392E-15	0.11541482	0.672	0.666	1.5974E-11	TRIM21
UBE2Z	1.391E-15	0.10408382	0.744	0.739	2.1381E-11	UBE2Z
ZNF90	1.4673E-15	0.56729677	0.046	0.034	2.2555E-11	ZNF90
FUBP1	1.5794E-15	0.11454858	0.631	0.617	2.4277E-11	FUBP1
LAT	1.8356E-15	0.37426344	0.144	0.124	2.8215E-11	LAT
CACNB3	1.9481E-15	0.22610908	0.47	0.458	2.9944E-11	CACNB3
IFRD1	2.1615E-15	0.13740302	0.641	0.69	3.3224E-11	IFRD1
RSPH3	2.162E-15	0.12359254	0.618	0.61	3.3232E-11	RSPH3
CXCL2	2.1998E-15	0.19282123	0.451	0.433	3.3814E-11	CXCL2
ATP5PO	2.3599E-15	0.35612001	0.152	0.132	3.6274E-11	ATP5PO
NINJ1	2.5096E-15	0.29560768	0.424	0.42	3.8575E-11	NINJ1
HINT1	2.7142E-15	0.10334995	0.722	0.72	4.172E-11	HINT1
TLE4	2.7936E-15	0.14571771	0.45	0.431	4.294E-11	TLE4
BIN2	2.8823E-15	0.10684957	0.724	0.719	4.4304E-11	BIN2
CD24	2.8887E-15	1.10089566	0.013	0.007	4.4403E-11	CD24
TRGC2	3.2551E-15	0.24712766	0.249	0.226	5.0034E-11	TRGC2
SLC66A3	3.485E-15	0.10063754	0.738	0.735	5.3568E-11	SLC66A3
IKBKE	3.4875E-15	0.2954193	0.248	0.228	5.3606E-11	IKBKE
PRKAR1B	3.7565E-15	0.53670148	0.056	0.043	5.7741E-11	PRKAR1B
FCRL6	4.4384E-15	0.50542326	0.048	0.036	6.8223E-11	FCRL6
FAM50B	4.6033E-15	0.25429655	0.229	0.208	7.0757E-11	FAM50B
SIPA1L2	4.704E-15	0.13890375	0.601	0.595	7.2305E-11	SIPA1L2
SUMO2	4.8862E-15	0.11342598	0.668	0.66	7.5106E-11	SUMO2
HOPX	5.4947E-15	1.10089022	0.01	0.005	8.4459E-11	HOPX
PCARE	6.3659E-15	0.42668285	0.109	0.091	9.7851E-11	PCARE
INSIG1	7.0123E-15	0.3133464	0.302	0.283	1.0779E-10	INSIG1
ATF5	7.3055E-15	0.24272719	0.296	0.275	1.1229E-10	ATF5
CASS4	8.1632E-15	0.65641585	0.037	0.026	1.2548E-10	CASS4
ZNF362	8.4085E-15	0.12434043	0.554	0.539	1.2925E-10	ZNF362
KRBOX1	8.437E-15	0.88853555	0.014	0.008	1.2969E-10	KRBOX1
CRACR2B	9.8281E-15	0.22226848	0.27	0.248	1.5107E-10	CRACR2B
DHCR24	1.7385E-14	0.19053416	0.69	0.694	2.6723E-10	DHCR24
MREG	1.9572E-14	0.4856157	0.094	0.078	3.0084E-10	MREG
PTER	1.9687E-14	0.20887251	0.294	0.273	3.0262E-10	PTER
RABEP1	2.073E-14	0.11695456	0.613	0.602	3.1864E-10	RABEP1
ANG	2.1719E-14	0.36082771	0.125	0.106	3.3385E-10	ANG
ELF4	2.4682E-14	0.12884797	0.597	0.587	3.7938E-10	ELF4
PIR	2.5181E-14	0.32401383	0.162	0.143	3.8706E-10	PIR
ELP5	2.97E-14	0.13672371	0.531	0.521	4.5653E-10	ELP5
HIST2H2BE	3.8756E-14	0.29614952	0.132	0.113	5.9572E-10	HIST2H2BE
PARL	4.9432E-14	0.11850134	0.598	0.59	7.5982E-10	PARL

EBP	5.8414E-14	0.16754187	0.494	0.482	8.9788E-10	EBP
GPC4	6.2938E-14	0.45871345	0.088	0.072	9.6741E-10	GPC4
NDUFA13	7.5158E-14	0.10603708	0.673	0.668	1.1553E-09	NDUFA13
RNF122	1.3053E-13	0.32222434	0.113	0.096	2.0063E-09	RNF122
SLC39A11	1.5106E-13	0.180616	0.452	0.44	2.322E-09	SLC39A11
MED30	1.5324E-13	0.16618813	0.383	0.366	2.3554E-09	MED30
ASMT	1.602E-13	1.2266249	0.012	0.006	2.4624E-09	ASMT
RGS2	1.7695E-13	0.1513642	0.48	0.539	2.7199E-09	RGS2
MAML2	1.8908E-13	0.10024519	0.63	0.617	2.9064E-09	MAML2
ECSCR	2.2545E-13	0.13021969	0.712	0.718	3.4653E-09	ECSCR
ELOVL5	2.5547E-13	0.1076865	0.61	0.601	3.9269E-09	ELOVL5
SCAMP5	2.6461E-13	0.5798837	0.048	0.037	4.0673E-09	SCAMP5
DDT	2.7981E-13	0.12217737	0.618	0.613	4.301E-09	DDT
NYNRIN	2.8683E-13	0.39814069	0.071	0.057	4.4089E-09	NYNRIN
HIST1H2BH	3.1361E-13	0.42911798	0.063	0.05	4.8205E-09	HIST1H2BH
GPAT3	3.3522E-13	0.36910539	0.085	0.07	5.1527E-09	GPAT3
ALDH1A2	3.7981E-13	0.22370963	0.238	0.216	5.8381E-09	ALDH1A2
LZTS2	4.1565E-13	0.1658557	0.432	0.417	6.389E-09	LZTS2
DUSP23	4.2601E-13	0.10347051	0.697	0.694	6.5482E-09	DUSP23
ACAT2	4.3066E-13	0.33354595	0.305	0.291	6.6196E-09	ACAT2
RAVER2	5.1794E-13	0.45821743	0.052	0.04	7.9613E-09	RAVER2
AFDN	6.0606E-13	0.11331075	0.575	0.564	9.3157E-09	AFDN
TLCD2	6.4551E-13	0.12342884	0.593	0.586	9.9221E-09	TLCD2
THYN1	7.0176E-13	0.12084818	0.55	0.54	1.0787E-08	THYN1
JMJD8	7.5142E-13	0.12092256	0.593	0.586	1.155E-08	JMJD8
DOCK5	7.8621E-13	0.22235246	0.275	0.255	1.2085E-08	DOCK5
TRIM58	9.313E-13	0.94783247	0.01	0.005	1.4315E-08	TRIM58
GTF2F1	1.0472E-12	0.11201669	0.616	0.609	1.6097E-08	GTF2F1
EREG	1.0939E-12	0.48244466	0.209	0.192	1.6814E-08	EREG
ERG28	1.9086E-12	0.24345033	0.275	0.257	2.9338E-08	ERG28
STARD4	2.0917E-12	0.23356425	0.203	0.184	3.2152E-08	STARD4
ZFYVE28	2.5528E-12	0.32992037	0.12	0.104	3.924E-08	ZFYVE28
POLR2E	2.5809E-12	0.10347683	0.742	0.744	3.9671E-08	POLR2E
BCKDHA	2.794E-12	0.12908666	0.534	0.524	4.2947E-08	BCKDHA
CSNK1E	3.2166E-12	0.12746262	0.546	0.539	4.9443E-08	CSNK1E
LIMK2	3.609E-12	0.12698164	0.691	0.69	5.5474E-08	LIMK2
P2RY12	4.5231E-12	0.37865562	0.106	0.091	6.9525E-08	P2RY12
ADAMTS6	4.7311E-12	0.55712227	0.04	0.03	7.2722E-08	ADAMTS6
RASA3	5.1804E-12	0.22808396	0.276	0.259	7.9628E-08	RASA3
TMEM43	5.4216E-12	0.1128356	0.634	0.632	8.3335E-08	TMEM43
INAFM2	5.6168E-12	0.18871777	0.362	0.345	8.6335E-08	INAFM2
CHRNB1	7.0815E-12	0.30253957	0.121	0.105	1.0885E-07	CHRNB1
XRRA1	7.2411E-12	0.22929274	0.15	0.131	1.113E-07	XRRA1
PEX6	7.2964E-12	0.17710764	0.291	0.272	1.1215E-07	PEX6
TTC39B	7.4765E-12	0.11386976	0.682	0.679	1.1492E-07	TTC39B
ACSM5	7.6947E-12	0.66955499	0.022	0.015	1.1827E-07	ACSM5
ZNF880	7.7177E-12	0.3536868	0.102	0.087	1.1863E-07	ZNF880
C5orf15	8.2113E-12	0.1281992	0.496	0.484	1.2622E-07	C5orf15
MT-ATP6	8.5153E-12	0.18079941	0.453	0.436	1.3089E-07	MT-ATP6
MTMR11	8.5797E-12	0.44242175	0.059	0.047	1.3188E-07	MTMR11
MARCKSL1	8.7964E-12	0.67467566	0.029	0.021	1.3521E-07	MARCKSL1
RTL8C	9.2587E-12	0.12205418	0.523	0.51	1.4231E-07	RTL8C

TRIB2	1.0455E-11	0.57050759	0.03	0.021	1.607E-07	TRIB2
INO80C	1.1253E-11	0.16572403	0.448	0.436	1.7297E-07	INO80C
MB21D2	1.188E-11	0.21732282	0.276	0.258	1.826E-07	MB21D2
ADRB2	1.2617E-11	0.1863601	0.459	0.45	1.9394E-07	ADRB2
CCL24	1.3967E-11	0.1811909	0.308	0.288	2.1469E-07	CCL24
IKBIP	1.4032E-11	0.24999194	0.194	0.177	2.1568E-07	IKBIP
AIFM2	1.5294E-11	0.32191927	0.136	0.12	2.3508E-07	AIFM2
EPB41L2	1.8075E-11	0.19656276	0.333	0.318	2.7783E-07	EPB41L2
NEMP2	1.8186E-11	0.40161408	0.085	0.072	2.7953E-07	NEMP2
AMOTL1	2.0135E-11	0.11838839	0.614	0.612	3.095E-07	AMOTL1
HOXA5	2.0902E-11	0.89266206	0.014	0.008	3.2128E-07	HOXA5
ADD3	2.0956E-11	0.12000957	0.489	0.477	3.2212E-07	ADD3
PLGRKT	2.1541E-11	0.15455275	0.404	0.39	3.3111E-07	PLGRKT
SNUPN	2.2586E-11	0.10581592	0.646	0.648	3.4716E-07	SNUPN
MEFV	2.3016E-11	0.15434901	0.484	0.474	3.5378E-07	MEFV
LYPD5	2.424E-11	0.23160533	0.144	0.127	3.726E-07	LYPD5
DNAJC4	2.6749E-11	0.10393131	0.624	0.618	4.1115E-07	DNAJC4
OAZ2	2.7216E-11	0.11351046	0.57	0.564	4.1834E-07	OAZ2
PHLDB3	2.7357E-11	0.7508762	0.017	0.011	4.2051E-07	PHLDB3
BCL2L11	2.8306E-11	0.22934398	0.286	0.27	4.351E-07	BCL2L11
PLAAT1	2.8331E-11	0.40718866	0.065	0.053	4.3547E-07	PLAAT1
GSTT2B	2.9218E-11	0.80311336	0.017	0.011	4.4911E-07	GSTT2B
SF3A2	3.1699E-11	0.11911707	0.583	0.581	4.8725E-07	SF3A2
ERLIN2	3.1743E-11	0.11616163	0.561	0.552	4.8793E-07	ERLIN2
IQCA1	3.4753E-11	0.70427623	0.016	0.01	5.3418E-07	IQCA1
ATP2A3	3.5614E-11	0.54123812	0.04	0.03	5.4742E-07	ATP2A3
LHPP	5.2637E-11	0.16338714	0.435	0.426	8.0909E-07	LHPP
COX5B	5.3019E-11	0.12124742	0.492	0.482	8.1495E-07	COX5B
FAXDC2	5.6932E-11	0.21861506	0.222	0.204	8.751E-07	FAXDC2
PPP3CA	6.276E-11	0.10123572	0.619	0.614	9.6469E-07	PPP3CA
TNFRSF21	7.3528E-11	0.33124007	0.26	0.245	1.1302E-06	TNFRSF21
TNNI1	7.4102E-11	0.43433738	0.056	0.045	1.139E-06	TNNI1
PNMA8B	8.4741E-11	0.58958674	0.029	0.021	1.3026E-06	PNMA8B
NT5C	8.8842E-11	0.12189924	0.519	0.511	1.3656E-06	NT5C
CYB5R2	9.7521E-11	0.27240481	0.287	0.273	1.499E-06	CYB5R2
LYRM9	1.1433E-10	0.31944159	0.104	0.09	1.7574E-06	LYRM9
BIRC3	1.2752E-10	0.36775925	0.067	0.055	1.9602E-06	BIRC3
PAQR5	1.4819E-10	0.12728433	0.69	0.698	2.2778E-06	PAQR5
PRCC	1.4879E-10	0.1034153	0.589	0.582	2.287E-06	PRCC
AAMDC	1.5414E-10	0.15483916	0.387	0.373	2.3693E-06	AAMDC
ARL4C	1.6849E-10	0.39475369	0.158	0.144	2.5899E-06	ARL4C
TP53I11	1.9063E-10	0.13541425	0.403	0.387	2.9302E-06	TP53I11
SUSD5	2.0095E-10	0.85697305	0.016	0.01	3.0888E-06	SUSD5
CSNK2B	2.1164E-10	0.10429723	0.566	0.558	3.2532E-06	CSNK2B
DNMBP	2.1507E-10	0.31840527	0.106	0.092	3.3058E-06	DNMBP
SELENOK	2.2309E-10	0.12608315	0.716	0.723	3.4291E-06	SELENOK
HSBP1L1	2.3058E-10	0.23029353	0.198	0.181	3.5443E-06	HSBP1L1
CD302	2.4965E-10	0.11563822	0.662	0.661	3.8374E-06	CD302
LONRF1	2.6425E-10	0.18433005	0.311	0.295	4.0618E-06	LONRF1
PDE4B	2.8274E-10	0.11660228	0.418	0.464	4.346E-06	PDE4B
CD7	2.9244E-10	0.50013213	0.043	0.033	4.495E-06	CD7
HIST1H2AE	3.011E-10	0.77142676	0.016	0.01	4.6282E-06	HIST1H2AE

TSLP	3.0628E-10	0.51344254	0.028	0.02	4.7078E-06	TSLP
GUCA1B	3.3816E-10	0.75733175	0.011	0.007	5.1978E-06	GUCA1B
TPGS1	3.78E-10	0.10913474	0.587	0.584	5.8103E-06	TPGS1
NFATC2	4.4854E-10	0.47309547	0.046	0.036	6.8946E-06	NFATC2
IRF3	4.5069E-10	0.10365969	0.629	0.625	6.9275E-06	IRF3
OCEL1	4.5413E-10	0.21449377	0.273	0.259	6.9805E-06	OCEL1
CCAR2	5.0539E-10	0.11288068	0.533	0.527	7.7684E-06	CCAR2
KCNK13	5.0904E-10	0.48519237	0.049	0.039	7.8244E-06	KCNK13
OVOL1	5.1362E-10	0.92328255	0.011	0.006	7.8949E-06	OVOL1
ZNF626	5.4458E-10	0.25611961	0.17	0.154	8.3707E-06	ZNF626
CD2BP2	5.6166E-10	0.10559846	0.556	0.552	8.6333E-06	CD2BP2
PHETA2	5.6786E-10	0.29286851	0.101	0.087	8.7287E-06	PHETA2
LPIN1	5.8867E-10	0.24538917	0.19	0.175	9.0484E-06	LPIN1
CDK2AP1	6.1419E-10	0.11637414	0.461	0.449	9.4408E-06	CDK2AP1
AC093323.1	6.2007E-10	0.46183975	0.046	0.036	9.531E-06	AC093323.1
SLC25A5	6.4661E-10	0.11487452	0.537	0.531	9.9391E-06	SLC25A5
DGKH	6.7291E-10	0.23372012	0.199	0.183	1.0343E-05	DGKH
MFSD4A	6.9693E-10	0.64644927	0.028	0.02	1.0712E-05	MFSD4A
THBS3	7.0754E-10	0.30885484	0.127	0.113	1.0876E-05	THBS3
CDKN1A	7.2504E-10	0.30915843	0.265	0.249	1.1145E-05	CDKN1A
MED11	8.6725E-10	0.17292888	0.32	0.305	1.333E-05	MED11
SEMA7A	8.7127E-10	0.41288993	0.037	0.028	1.3392E-05	SEMA7A
HDAC9	9.0093E-10	0.3526885	0.079	0.067	1.3848E-05	HDAC9
DRAM1	9.2894E-10	0.12978648	0.805	0.817	1.4279E-05	DRAM1
GIMAP2	9.5314E-10	0.10868084	0.556	0.553	1.4651E-05	GIMAP2
ERCC5	1.094E-09	0.11328113	0.574	0.57	1.6816E-05	ERCC5
MRFAP1L1	1.2061E-09	0.12374271	0.507	0.499	1.8539E-05	MRFAP1L1
SMYD2	1.2519E-09	0.10165274	0.562	0.555	1.9242E-05	SMYD2
PEBP4	1.4868E-09	0.3507081	0.054	0.044	2.2854E-05	PEBP4
CITED4	1.5128E-09	0.45397061	0.043	0.034	2.3254E-05	CITED4
C6orf120	1.8794E-09	0.11043396	0.595	0.591	2.8889E-05	C6orf120
TLR2	2.1037E-09	0.14824148	0.307	0.291	3.2336E-05	TLR2
KLHL22	2.1852E-09	0.2026451	0.215	0.199	3.3589E-05	KLHL22
ABTB1	2.2513E-09	0.1970243	0.239	0.223	3.4605E-05	ABTB1
POFUT1	2.2566E-09	0.12009976	0.492	0.485	3.4686E-05	POFUT1
CLDN23	2.7635E-09	0.16401892	0.398	0.388	4.2477E-05	CLDN23
VWA5A	2.7932E-09	0.12610726	0.554	0.55	4.2935E-05	VWA5A
FZD7	3.5991E-09	0.30008951	0.097	0.084	5.5321E-05	FZD7
PEX11G	3.8945E-09	0.32205117	0.075	0.063	5.9862E-05	PEX11G
BLOC1S4	4.0475E-09	0.18742709	0.273	0.258	6.2214E-05	BLOC1S4
EIF4EBP3	4.6217E-09	0.18135497	0.312	0.299	7.1041E-05	EIF4EBP3
COL14A1	4.6427E-09	0.34676108	0.033	0.025	7.1363E-05	COL14A1
MS4A6E	4.9946E-09	0.6125574	0.018	0.012	7.6772E-05	MS4A6E
MYO7B	5.4846E-09	0.22415295	0.254	0.24	8.4304E-05	MYO7B
PEX11A	5.9759E-09	0.1917384	0.27	0.255	9.1855E-05	PEX11A
PAOX	6.6597E-09	0.24738893	0.151	0.136	0.00010237	PAOX
HSD11B1	6.78E-09	0.51395584	0.033	0.025	0.00010422	HSD11B1
ZBED1	7.3942E-09	0.11059237	0.569	0.566	0.00011366	ZBED1
ZBED6CL	8.2844E-09	0.21994109	0.19	0.176	0.00012734	ZBED6CL
BORCS6	9.4069E-09	0.15224073	0.372	0.36	0.00014459	BORCS6
NOA1	1.0181E-08	0.14115018	0.409	0.399	0.00015649	NOA1
SGCG	1.2826E-08	0.65187234	0.013	0.009	0.00019715	SGCG

FDPS	1.2847E-08	0.22809349	0.215	0.201	0.00019748	FDPS
C12orf57	1.6018E-08	0.13156638	0.429	0.419	0.00024621	C12orf57
SNTA1	1.6039E-08	0.21632916	0.21	0.195	0.00024653	SNTA1
AFAP1L1	1.7952E-08	0.99080745	0.01	0.006	0.00027594	AFAP1L1
QPRT	1.8189E-08	0.17290575	0.256	0.241	0.00027958	QPRT
DELE1	1.9099E-08	0.11478861	0.472	0.464	0.00029357	DELE1
C6orf47	2.1109E-08	0.15019724	0.319	0.305	0.00032447	C6orf47
IFI27L1	2.4507E-08	0.22115511	0.193	0.179	0.0003767	IFI27L1
RALGPS2	2.9914E-08	0.37168413	0.054	0.045	0.00045981	RALGPS2
C2orf68	3.4621E-08	0.10490726	0.492	0.484	0.00053215	C2orf68
INO80B	3.5118E-08	0.10884706	0.448	0.439	0.00053981	INO80B
SGCB	3.726E-08	0.56458307	0.022	0.016	0.00057272	SGCB
CCDC9	3.9096E-08	0.15310693	0.367	0.358	0.00060094	CCDC9
NRROS	4.3535E-08	0.10932148	0.491	0.482	0.00066917	NRROS
GADD45B	4.7202E-08	0.19114402	0.162	0.148	0.00072554	GADD45B
IGF1	4.9245E-08	0.2602968	0.276	0.262	0.00075694	IGF1
SSPN	5.5803E-08	0.23604667	0.12	0.107	0.00085774	SSPN
AMIGO1	5.6047E-08	0.46689989	0.03	0.023	0.00086149	AMIGO1
LRG1	5.8185E-08	0.38532685	0.059	0.049	0.00089437	LRG1
TNFRSF1B	5.8777E-08	0.13331019	0.442	0.435	0.00090346	TNFRSF1B
DNAJB2	6.7037E-08	0.11857302	0.465	0.46	0.00103042	DNAJB2
NR1D1	6.9685E-08	0.25689921	0.12	0.107	0.00107112	NR1D1
HINT2	7.025E-08	0.10688643	0.512	0.508	0.00107981	HINT2
C7orf25	7.2846E-08	0.13181836	0.356	0.343	0.00111972	C7orf25
EFCAB2	7.7503E-08	0.34179151	0.075	0.065	0.0011913	EFCAB2
HSD17B8	8.183E-08	0.13975394	0.358	0.347	0.00125781	HSD17B8
SYNPO	8.2423E-08	0.47475134	0.088	0.077	0.00126693	SYNPO
ANKLE2	8.7473E-08	0.10792041	0.551	0.548	0.00134455	ANKLE2
TMED8	8.9446E-08	0.13247339	0.42	0.411	0.00137488	TMED8
ADORA2B	1.2878E-07	0.26752109	0.116	0.104	0.00197945	ADORA2B
CENPV	1.3003E-07	0.12718167	0.401	0.39	0.00199875	CENPV
ISG20	1.306E-07	0.53181865	0.03	0.023	0.00200745	ISG20
OTOF	1.3452E-07	1.15155918	0.019	0.014	0.00206772	OTOF
SREBF2	1.3459E-07	0.11187979	0.661	0.665	0.00206885	SREBF2
PLEKHG3	1.3726E-07	0.16259533	0.36	0.349	0.00210985	PLEKHG3
APBB2	1.3853E-07	0.22361323	0.156	0.143	0.00212938	APBB2
CEP170B	1.3909E-07	0.20692195	0.194	0.18	0.00213795	CEP170B
FAR2	1.4316E-07	0.14584991	0.722	0.736	0.00220049	FAR2
SCAND1	1.4389E-07	0.10880477	0.46	0.451	0.00221167	SCAND1
NAALADL2	1.5225E-07	0.54640699	0.016	0.011	0.00234023	NAALADL2
BCL2	1.5718E-07	0.39574158	0.056	0.047	0.00241606	BCL2
CYB5D2	1.6141E-07	0.16422681	0.301	0.29	0.00248102	CYB5D2
MPEG1	1.6335E-07	0.10674857	0.77	0.785	0.00251079	MPEG1
BTNL8	1.7181E-07	0.4600254	0.027	0.02	0.00264095	BTNL8
HELZ2	1.8059E-07	0.20308355	0.293	0.283	0.00277584	HELZ2
RAI1	1.8082E-07	0.20817311	0.2	0.187	0.00277945	RAI1
CARD11	1.8659E-07	0.46276562	0.04	0.033	0.00286807	CARD11
SMIM26	1.8719E-07	0.11175153	0.422	0.412	0.00287725	SMIM26
ENPP5	1.8722E-07	0.26408881	0.101	0.09	0.00287775	ENPP5
DSP	2.1292E-07	0.51531315	0.024	0.018	0.00327277	DSP
SNX15	2.1531E-07	0.10221555	0.491	0.485	0.0033096	SNX15
CYBRD1	2.1848E-07	0.90273315	0.01	0.006	0.00335825	CYBRD1

R3HCC1	2.2518E-07	0.16161312	0.273	0.261	0.00346129	R3HCC1
FUT4	2.3678E-07	0.14736236	0.357	0.346	0.00363957	FUT4
GABRA4	2.3897E-07	0.36737686	0.053	0.044	0.00367319	GABRA4
SUSD3	2.6631E-07	0.1799658	0.219	0.206	0.00409345	SUSD3
EXD2	3.1111E-07	0.10893173	0.512	0.509	0.00478208	EXD2
TRIP10	3.1152E-07	0.1418802	0.322	0.311	0.00478833	TRIP10
PSTPIP2	3.2651E-07	0.23042338	0.12	0.108	0.0050188	PSTPIP2
NUDT1	3.2996E-07	0.11782403	0.467	0.461	0.00507187	NUDT1
HCN2	3.3365E-07	0.55940117	0.018	0.013	0.00512856	HCN2
ECSIT	3.7757E-07	0.10723056	0.472	0.466	0.00580356	ECSIT
TTYH2	3.8356E-07	0.33543214	0.076	0.066	0.00589571	TTYH2
EXOC3L4	3.9949E-07	0.49667101	0.024	0.019	0.00614063	EXOC3L4
ZNF780A	4.3486E-07	0.16025645	0.307	0.295	0.0066843	ZNF780A
GTF2E1	4.4535E-07	0.1334105	0.405	0.398	0.00684547	GTF2E1
SPACA6	4.5639E-07	0.6312011	0.015	0.011	0.00701517	SPACA6
RAB11FIP4	4.5954E-07	0.11429568	0.525	0.525	0.00706353	RAB11FIP4
CTNNAL1	4.648E-07	0.28745751	0.045	0.037	0.00714448	CTNNAL1
TTI2	4.8758E-07	0.17829164	0.25	0.238	0.00749463	TTI2
METTL9	5.4291E-07	0.13004373	0.523	0.524	0.00834505	METTL9
BRF2	5.7751E-07	0.16087253	0.278	0.267	0.00887698	BRF2
ZCCHC3	6.2861E-07	0.12581388	0.389	0.38	0.00966241	ZCCHC3
AKAP1	6.3452E-07	0.12563777	0.43	0.424	0.00975324	AKAP1
PRMT1	6.8167E-07	0.10855815	0.485	0.485	0.01047797	PRMT1
TCF3	7.2362E-07	0.12426743	0.325	0.312	0.0111228	TCF3
CNRIP1	7.2869E-07	0.25839572	0.108	0.097	0.01120069	CNRIP1
FANCA	7.4547E-07	0.29522153	0.085	0.075	0.01145866	FANCA
TYSND1	7.5152E-07	0.23427169	0.139	0.127	0.01155154	TYSND1
ASCL2	7.7963E-07	0.3854946	0.041	0.034	0.01198377	ASCL2
SLC41A2	8.2824E-07	0.11047486	0.359	0.347	0.01273084	SLC41A2
SLAMF7	8.3511E-07	0.82022668	0.012	0.008	0.01283642	SLAMF7
TAF7	8.3555E-07	0.11320096	0.525	0.522	0.01284328	TAF7
TMEM187	8.3676E-07	0.31795617	0.085	0.075	0.01286191	TMEM187
LRRC58	8.4859E-07	0.11261712	0.418	0.408	0.01304375	LRRC58
B3GALNT1	9.898E-07	0.16429281	0.19	0.177	0.01521428	B3GALNT1
PHF1	1.0016E-06	0.10472128	0.506	0.502	0.01539606	PHF1
PAM16	1.0112E-06	0.1242624	0.453	0.45	0.01554338	PAM16
QTRT1	1.0132E-06	0.12172077	0.406	0.4	0.01557323	QTRT1
GHDC	1.0211E-06	0.1340305	0.363	0.353	0.0156957	GHDC
ARHGAP22	1.0882E-06	0.2526618	0.142	0.13	0.01672729	ARHGAP22
ZFP36L1	1.0964E-06	0.16698214	0.547	0.549	0.01685237	ZFP36L1
DVL2	1.1177E-06	0.1491672	0.314	0.304	0.01718075	DVL2
FUT10	1.1255E-06	0.24248208	0.136	0.124	0.0172997	FUT10
VWA7	1.1302E-06	0.2305832	0.126	0.115	0.01737265	VWA7
ZNF518B	1.1586E-06	0.1832905	0.199	0.186	0.01780887	ZNF518B
UCKL1	1.2571E-06	0.12524555	0.404	0.397	0.01932212	UCKL1
BBS1	1.3885E-06	0.18460247	0.211	0.199	0.02134273	BBS1
TRMT61B	1.4068E-06	0.17361949	0.202	0.19	0.02162468	TRMT61B
TOR3A	1.4469E-06	0.1073885	0.485	0.483	0.02224071	TOR3A
ST7	1.476E-06	0.10499092	0.618	0.626	0.02268717	ST7
SBK3	1.542E-06	0.68920011	0.013	0.009	0.02370232	SBK3
CCDC71L	1.5885E-06	0.15996965	0.288	0.277	0.02441693	CCDC71L
TPBGL	1.6547E-06	0.48054537	0.024	0.019	0.02543496	TPBGL

METTL21A	1.7117E-06	0.16516651	0.262	0.25	0.02631017	METTL21A
ALDOC	1.9195E-06	0.28021165	0.063	0.054	0.02950529	ALDOC
KDM5B	1.9417E-06	0.16702998	0.25	0.239	0.0298461	KDM5B
SPECC1L	2.0343E-06	0.12466744	0.379	0.371	0.03126888	SPECC1L
NUP50	2.1033E-06	0.10171125	0.457	0.453	0.03233005	NUP50
BRK1	2.1075E-06	0.1072365	0.5	0.5	0.03239446	BRK1
DAPK3	2.1172E-06	0.10272673	0.468	0.464	0.03254345	DAPK3
C17orf99	2.2002E-06	0.66332568	0.011	0.008	0.03381984	C17orf99
DPH2	2.2388E-06	0.16932462	0.304	0.295	0.03441281	DPH2
TREML4	2.2946E-06	0.52602207	0.015	0.01	0.03527096	TREML4
ACCS	2.3985E-06	0.25238971	0.128	0.117	0.03686668	ACCS
PWWP2B	2.4784E-06	0.10807901	0.439	0.434	0.03809552	PWWP2B
SLC16A7	2.5408E-06	0.75712966	0.01	0.007	0.03905427	SLC16A7
TTLL10	2.5749E-06	0.37555736	0.024	0.019	0.0395792	TTLL10
TM7SF2	2.7786E-06	0.32197129	0.063	0.054	0.0427106	TM7SF2
AREG	2.7989E-06	0.3151816	0.173	0.162	0.04302168	AREG
SAMSN1	3.104E-06	0.22352183	0.595	0.605	0.04771125	SAMSN1
C2orf92	3.2657E-06	0.43426578	0.026	0.02	0.05019759	C2orf92
C17orf49	3.2913E-06	0.1272759	0.408	0.403	0.05059023	C17orf49
ANO5	3.417E-06	0.12246138	0.307	0.296	0.05252238	ANO5
TSPAN13	3.4391E-06	0.50044418	0.043	0.036	0.05286227	TSPAN13
MELTF	4.0392E-06	0.63280777	0.012	0.008	0.0620872	MELTF
TMEM102	4.4776E-06	0.12601995	0.43	0.425	0.068825	TMEM102
NIPSNAP3B	4.5293E-06	0.28774126	0.029	0.023	0.06961927	NIPSNAP3B
ZNF512B	5.0222E-06	0.20925949	0.16	0.149	0.07719614	ZNF512B
CH25H	5.0928E-06	0.28930729	0.049	0.042	0.07828101	CH25H
MAPT	5.404E-06	0.63499506	0.014	0.01	0.0830642	MAPT
STON2	5.4042E-06	0.18327251	0.116	0.105	0.08306867	STON2
FP565260.1	5.4489E-06	0.39026376	0.032	0.026	0.08375457	FP565260.1
TST	5.6487E-06	0.15125983	0.309	0.301	0.08682642	TST
ARHGEF12	5.6569E-06	0.18536359	0.18	0.169	0.08695276	ARHGEF12
ECE1	5.9862E-06	0.2956753	0.134	0.124	0.0920144	ECE1
CXCL1	5.9882E-06	0.61217082	0.013	0.009	0.09204478	CXCL1
ATP6V0D2	6.5882E-06	0.48656352	0.1	0.091	0.10126653	ATP6V0D2
SERPINH1	6.6791E-06	0.14071193	0.303	0.294	0.1026648	SERPINH1
TIFA	6.859E-06	0.20260793	0.162	0.151	0.1054302	TIFA
DNAAF3	6.9654E-06	0.52507627	0.019	0.015	0.10706466	DNAAF3
CLCF1	7.5329E-06	0.18506866	0.187	0.176	0.11578853	CLCF1
HOXA1	7.5704E-06	0.38574227	0.04	0.033	0.11636468	HOXA1
STK19	7.6627E-06	0.18939863	0.2	0.19	0.11778314	STK19
RAB3C	9.2946E-06	0.40282565	0.017	0.013	0.14286687	RAB3C
CDNF	9.7619E-06	0.65076364	0.012	0.009	0.15004984	CDNF
GPR132	9.773E-06	0.5688551	0.032	0.026	0.15022151	GPR132
FLT3LG	9.8466E-06	0.23189694	0.078	0.069	0.15135139	FLT3LG
DCUN1D3	1.0083E-05	0.12702651	0.41	0.405	0.15498582	DCUN1D3
SIGLEC10	1.0347E-05	0.26056064	0.089	0.08	0.15904761	SIGLEC10
CERK	1.0587E-05	0.16630425	0.231	0.221	0.16273164	CERK
GLIPR1L2	1.0691E-05	0.46935748	0.024	0.019	0.1643242	GLIPR1L2
ETV5	1.1115E-05	0.51002825	0.015	0.011	0.17085428	ETV5
MUC20	1.192E-05	0.36763952	0.078	0.07	0.18322382	MUC20
MAD2L2	1.1973E-05	0.12315781	0.356	0.347	0.18403467	MAD2L2
CSKMT	1.2673E-05	0.37112513	0.03	0.024	0.19479171	CSKMT

FAM228A	1.3519E-05	0.50885408	0.017	0.013	0.20779395	FAM228A
AQP7	1.3613E-05	0.47136209	0.028	0.022	0.2092516	AQP7
PLEKHG2	1.4294E-05	0.50410853	0.023	0.018	0.2197085	PLEKHG2
CLUL1	1.5216E-05	0.35474028	0.028	0.022	0.2338883	CLUL1
PXN	1.5527E-05	0.46592183	0.02	0.015	0.23867011	PXN
ZNRD1	1.6616E-05	0.10742987	0.41	0.405	0.25540919	ZNRD1
FN3K	1.6896E-05	0.21966275	0.119	0.109	0.25970562	FN3K
HS3ST1	1.7152E-05	0.5019636	0.012	0.009	0.26364639	HS3ST1
ETV3	1.7372E-05	0.15786968	0.251	0.241	0.26702315	ETV3
NNMT	1.8805E-05	0.43379017	0.066	0.058	0.28905621	NNMT
SYP	1.9306E-05	0.22245483	0.088	0.079	0.29675758	SYP
ZKSCAN8	1.951E-05	0.1310787	0.309	0.299	0.29989507	ZKSCAN8
ALKBH6	2.0678E-05	0.12831652	0.281	0.272	0.31783429	ALKBH6
WDR31	2.1374E-05	0.44592401	0.019	0.014	0.32854524	WDR31
RAD51C	2.1414E-05	0.17935854	0.162	0.152	0.32915098	RAD51C
TTC12	2.22E-05	0.21909525	0.119	0.109	0.3412339	TTC12
MEF2C	2.2481E-05	0.28573724	0.066	0.058	0.34554982	MEF2C
PM20D1	2.2584E-05	0.40478662	0.033	0.027	0.34713844	PM20D1
MANEAL	2.3368E-05	0.22464787	0.087	0.078	0.35919115	MANEAL
EBPL	2.543E-05	0.25100889	0.09	0.082	0.3908787	EBPL
NAT9	2.5884E-05	0.13867109	0.324	0.318	0.39786239	NAT9
YDJC	2.7095E-05	0.20236155	0.139	0.129	0.41647505	YDJC
MAP1LC3C	2.7587E-05	0.28775534	0.073	0.065	0.42403344	MAP1LC3C
CATSPERG	3.0367E-05	0.23379169	0.093	0.085	0.46676436	CATSPERG
STN1	3.126E-05	0.15706793	0.237	0.228	0.48049259	STN1
OLAH	3.2285E-05	0.48097813	0.019	0.015	0.49625173	OLAH
TNFRSF10C	3.8044E-05	0.41255251	0.02	0.016	0.58477791	TNFRSF10C
GPR35	4.1369E-05	0.67187416	0.013	0.01	0.63588992	GPR35
ZNF682	4.4979E-05	0.37825098	0.033	0.027	0.69137841	ZNF682
CORO1A	4.9987E-05	0.25882924	0.187	0.178	0.76834465	CORO1A
ZNF576	5.5133E-05	0.15999981	0.217	0.207	0.84745581	ZNF576
HENMT1	5.6753E-05	0.1223363	0.359	0.353	0.87235634	HENMT1
NRARP	5.9005E-05	0.64066431	0.016	0.013	0.90697264	NRARP
EPS8	6.0701E-05	0.3117516	0.05	0.044	0.93302767	EPS8
CLIP2	6.1672E-05	0.12613471	0.372	0.368	0.94795993	CLIP2
GTF3C5	6.273E-05	0.10227651	0.411	0.407	0.96422063	GTF3C5
PIGO	6.4199E-05	0.10998773	0.396	0.391	0.98680818	PIGO
VASN	6.4968E-05	0.5484867	0.013	0.01	0.99862082	VASN
COCH	0.0002011	0.66761031	0.012	0.009	1	COCH
ELMOD1	0.007957	0.6256418	0.01	0.008	1	ELMOD1
SMKR1	0.00010763	0.6133618	0.01	0.007	1	SMKR1
TNNI3	0.00017071	0.60368252	0.01	0.007	1	TNNI3
ZNF467	0.00024218	0.55526784	0.01	0.007	1	ZNF467
AATK	7.5989E-05	0.52716413	0.017	0.013	1	AATK
CDKN1C	0.00120315	0.52438564	0.01	0.008	1	CDKN1C
SPINK6	0.00116065	0.5144513	0.01	0.008	1	SPINK6
HECW2	0.00728451	0.5123874	0.01	0.008	1	HECW2
TNFSF15	0.00368701	0.51040805	0.011	0.008	1	TNFSF15
DRC7	0.00068844	0.50573954	0.011	0.008	1	DRC7
ZNF423	0.00253213	0.49704613	0.014	0.011	1	ZNF423
CCNB3	0.0001449	0.4823152	0.016	0.012	1	CCNB3
MYO5B	0.00014034	0.47868802	0.012	0.009	1	MYO5B

PPBP	0.26455438	0.47372568	0.028	0.029	1	PPBP
EHF	0.00135012	0.47304634	0.02	0.017	1	EHF
CTTN	0.00770424	0.46154264	0.015	0.013	1	CTTN
CCL22	0.00853037	0.46124216	0.01	0.008	1	CCL22
GREB1	0.0002014	0.45479874	0.015	0.012	1	GREB1
DDX43	0.00714126	0.44817632	0.013	0.011	1	DDX43
COL26A1	0.01476102	0.44758964	0.01	0.009	1	COL26A1
F3	7.5309E-05	0.44625687	0.028	0.023	1	F3
RGL1	0.00224694	0.43906744	0.01	0.008	1	RGL1
TNFSF14	0.00232201	0.43700713	0.015	0.013	1	TNFSF14
TMEM217	0.00080025	0.4366752	0.018	0.015	1	TMEM217
BLK	0.00284662	0.43392621	0.015	0.012	1	BLK
BASP1	0.00054379	0.41590332	0.059	0.053	1	BASP1
PCBP4	0.02443762	0.41144434	0.01	0.008	1	PCBP4
RANBP3L	0.01631839	0.40883712	0.013	0.011	1	RANBP3L
CCL4	0.66497461	0.40173816	0.039	0.039	1	CCL4
MAPK8IP1	0.01627859	0.40097633	0.012	0.01	1	MAPK8IP1
NKAPL	0.00027724	0.39848018	0.019	0.015	1	NKAPL
CXCL10	0.47697518	0.39516069	0.025	0.024	1	CXCL10
CCDC122	0.0001333	0.39508737	0.018	0.014	1	CCDC122
MEIS3	0.01344532	0.39306535	0.012	0.01	1	MEIS3
TAF4B	0.00016333	0.38455875	0.028	0.024	1	TAF4B
DOCK3	0.00485201	0.38245325	0.016	0.013	1	DOCK3
LINC00672	0.03607413	0.37842866	0.011	0.01	1	LINC00672
HSH2D	0.00083083	0.37449514	0.047	0.041	1	HSH2D
ZNF415	0.00746038	0.37344233	0.013	0.011	1	ZNF415
GABBR1	0.45473966	0.37198241	0.01	0.01	1	GABBR1
DAB2IP	0.02579244	0.37055578	0.012	0.011	1	DAB2IP
CAVIN2	0.00014015	0.36962166	0.027	0.022	1	CAVIN2
PRKY	0.02613459	0.36838744	0.013	0.011	1	PRKY
DLG3	0.37225558	0.36240973	0.01	0.009	1	DLG3
TNFAIP6	0.00151488	0.3596965	0.015	0.012	1	TNFAIP6
FBXO36	0.0278191	0.35614064	0.015	0.013	1	FBXO36
ADGRL1	0.01365465	0.35097023	0.022	0.02	1	ADGRL1
PAQR7	0.01515372	0.34648217	0.012	0.01	1	PAQR7
GNA14	0.00015355	0.33945512	0.025	0.021	1	GNA14
ZSWIM4	8.1264E-05	0.33672811	0.034	0.029	1	ZSWIM4
GSG1L	0.00190554	0.33548641	0.012	0.01	1	GSG1L
CMBL	0.00145814	0.33195763	0.027	0.023	1	CMBL
PLS3	0.00627381	0.33141928	0.015	0.012	1	PLS3
FST	0.03661474	0.32969271	0.014	0.012	1	FST
RIC3	0.00245961	0.32618026	0.037	0.032	1	RIC3
GABRE	0.48325482	0.32601571	0.024	0.024	1	GABRE
TMEM158	0.00078921	0.32480937	0.127	0.12	1	TMEM158
CCDC28B	0.00639838	0.32159574	0.013	0.011	1	CCDC28B
ARHGAP23	0.00366247	0.32057685	0.033	0.029	1	ARHGAP23
TMEM265	0.00198684	0.31945075	0.022	0.019	1	TMEM265
GSDMB	0.13307504	0.31798999	0.016	0.014	1	GSDMB
GRASP	0.14242203	0.3174195	0.013	0.012	1	GRASP
EFCAB6	0.02201145	0.31681274	0.019	0.016	1	EFCAB6
ARHGEF16	0.14706363	0.31599569	0.012	0.01	1	ARHGEF16
LNP1	0.0040679	0.31556671	0.016	0.013	1	LNP1

REXO5	0.02568243	0.31386314	0.013	0.011	1	REXO5
RRAS2	0.00910707	0.31288151	0.02	0.017	1	RRAS2
RBKS	0.00020724	0.31049267	0.035	0.03	1	RBKS
MFAP3L	0.00106455	0.30868572	0.025	0.021	1	MFAP3L
PHGDH	0.15390883	0.30830768	0.039	0.037	1	PHGDH
C1orf56	0.07119785	0.30738859	0.012	0.01	1	C1orf56
ACSBG2	0.16743901	0.30687287	0.01	0.009	1	ACSBG2
ZNF594	0.10230226	0.3042377	0.012	0.011	1	ZNF594
SRCIN1	0.01209717	0.29970751	0.029	0.026	1	SRCIN1
TBX6	0.00027737	0.2996326	0.041	0.035	1	TBX6
IL27	0.00462141	0.29714694	0.037	0.033	1	IL27
INCA1	0.06128997	0.2958442	0.013	0.011	1	INCA1
TVP23C	0.05863367	0.295378	0.016	0.014	1	TVP23C
VCAN	0.0486454	0.29353877	0.215	0.214	1	VCAN
SYCP2	0.09029564	0.29086567	0.011	0.009	1	SYCP2
ETS1	0.29515112	0.29062404	0.012	0.011	1	ETS1
PRX	0.00139771	0.29048195	0.035	0.031	1	PRX
CAPN11	0.01333475	0.28967297	0.013	0.011	1	CAPN11
PLPP6	0.00015477	0.28692939	0.071	0.064	1	PLPP6
C19orf84	0.08996876	0.28377477	0.011	0.009	1	C19orf84
XKR3	0.2123736	0.28349425	0.011	0.01	1	XKR3
PRR5	0.01943631	0.28344595	0.021	0.019	1	PRR5
ZDHHC1	0.00042478	0.28295057	0.042	0.037	1	ZDHHC1
C9orf24	0.05310254	0.28259245	0.013	0.012	1	C9orf24
GAS1	0.28369586	0.28076377	0.016	0.015	1	GAS1
ULBP2	0.28399744	0.28048768	0.013	0.012	1	ULBP2
ZNF540	0.00360442	0.27785626	0.037	0.033	1	ZNF540
SPHK1	0.32561283	0.27683055	0.01	0.009	1	SPHK1
GDA	0.00010783	0.27667098	0.051	0.045	1	GDA
ADAMTS7	0.00040902	0.27297337	0.024	0.02	1	ADAMTS7
ST8SIA4	0.04047933	0.26979075	0.055	0.052	1	ST8SIA4
LRGUK	0.0289051	0.26856622	0.022	0.019	1	LRGUK
EFNA3	0.36019898	0.26808914	0.018	0.017	1	EFNA3
SMPD3	0.19305383	0.26795164	0.015	0.014	1	SMPD3
IQCC	0.0374539	0.26757776	0.028	0.025	1	IQCC
DISP2	0.00411383	0.26639427	0.034	0.03	1	DISP2
PDP2	0.00018928	0.26357485	0.073	0.066	1	PDP2
TENT5C	0.02873841	0.26325643	0.023	0.02	1	TENT5C
GCH1	0.00694985	0.26294207	0.05	0.046	1	GCH1
ATP9A	0.00070647	0.26288563	0.103	0.095	1	ATP9A
ZNF396	0.00031652	0.26217796	0.048	0.042	1	ZNF396
TMEM97	0.00023946	0.26078476	0.114	0.106	1	TMEM97
TTC30B	0.00130497	0.26013963	0.047	0.042	1	TTC30B
HIST1H1B	0.78583714	0.25994043	0.032	0.033	1	HIST1H1B
ABCB6	0.00142398	0.2596698	0.03	0.026	1	ABCB6
RNF227	0.02076038	0.25882933	0.015	0.013	1	RNF227
PGBD1	0.03686947	0.25776497	0.013	0.011	1	PGBD1
FAM78B	0.10588897	0.25666749	0.011	0.009	1	FAM78B
LRRC3	0.01076193	0.25618228	0.05	0.046	1	LRRC3
ZNF471	0.00331801	0.25556681	0.045	0.04	1	ZNF471
FAAH	0.00260604	0.25285393	0.05	0.046	1	FAAH
WDR54	0.00011789	0.25167882	0.068	0.061	1	WDR54

HAL	0.04888125	0.25167244	0.023	0.02	1	HAL
LRFN3	0.04045729	0.25059435	0.013	0.012	1	LRFN3
ARHGEF17	0.1740907	0.25044318	0.025	0.023	1	ARHGEF17
SPSB1	0.00023392	0.25023149	0.074	0.067	1	SPSB1
SCG3	0.44289501	0.24951808	0.014	0.013	1	SCG3
RAB3A	0.00092626	0.2493267	0.076	0.07	1	RAB3A
VILL	0.0001465	0.24626081	0.057	0.05	1	VILL
CCIN	0.0164885	0.24532323	0.074	0.07	1	CCIN
KIF7	0.01271173	0.2433415	0.023	0.02	1	KIF7
SCNN1A	0.01638565	0.2432832	0.03	0.027	1	SCNN1A
MYOM1	0.02808734	0.24249885	0.038	0.035	1	MYOM1
ZNF502	0.00612638	0.24179896	0.051	0.046	1	ZNF502
ZDBF2	0.00280589	0.24166858	0.037	0.033	1	ZDBF2
IRAK1BP1	0.01935314	0.24149431	0.028	0.025	1	IRAK1BP1
ICOS	0.648534	0.24120582	0.441	0.471	1	ICOS
LMNTD2	0.00050117	0.24006748	0.051	0.045	1	LMNTD2
TSGA10	0.54253732	0.23845671	0.013	0.012	1	TSGA10
PAK6	0.18388142	0.23597652	0.01	0.009	1	PAK6
LRIG1	0.00180949	0.23536933	0.043	0.038	1	LRIG1
ARNTL2	0.08923382	0.23371831	0.027	0.025	1	ARNTL2
TIGD7	0.00765077	0.23166986	0.054	0.05	1	TIGD7
PAPSS2	0.00017179	0.2308806	0.175	0.166	1	PAPSS2
NEURL1	0.02777302	0.22734458	0.028	0.025	1	NEURL1
FAM189A2	0.17586816	0.22726704	0.029	0.027	1	FAM189A2
CD163L1	0.54986279	0.22721375	0.014	0.013	1	CD163L1
VAMP1	0.05596012	0.22694116	0.015	0.014	1	VAMP1
MYOZ1	0.0355693	0.22682749	0.035	0.032	1	MYOZ1
C16orf46	0.79114498	0.22461597	0.021	0.021	1	C16orf46
YY2	0.51238259	0.22299949	0.01	0.009	1	YY2
KCTD11	0.0013364	0.22280456	0.095	0.088	1	KCTD11
SLC16A13	0.04297283	0.22216716	0.043	0.04	1	SLC16A13
ZNRF1	0.15038938	0.22158804	0.012	0.011	1	ZNRF1
CYGB	0.30169576	0.22057141	0.146	0.146	1	CYGB
LRATD2	0.05232273	0.22031694	0.036	0.033	1	LRATD2
KATNAL2	0.01711279	0.22007217	0.028	0.025	1	KATNAL2
OSGIN1	0.27155088	0.21990829	0.018	0.017	1	OSGIN1
SLAMF9	0.16442194	0.21984068	0.013	0.012	1	SLAMF9
ARRDC5	0.22446013	0.21963762	0.023	0.022	1	ARRDC5
GEMIN8	0.00032623	0.21777222	0.122	0.114	1	GEMIN8
ZDHHC23	0.10314517	0.21661613	0.059	0.056	1	ZDHHC23
WWTR1	0.00301886	0.21534146	0.126	0.12	1	WWTR1
FOSL1	0.01527552	0.21530572	0.021	0.018	1	FOSL1
PLCL1	0.0094771	0.21503105	0.057	0.052	1	PLCL1
FAM241B	0.0001354	0.21485758	0.144	0.136	1	FAM241B
ADA	0.01619599	0.21476161	0.041	0.037	1	ADA
ITPR3	0.01365325	0.21273652	0.056	0.052	1	ITPR3
ZNF214	0.17672745	0.21238046	0.011	0.01	1	ZNF214
PCDHGA12	0.07906424	0.21210782	0.039	0.036	1	PCDHGA12
AWAT2	0.00202487	0.21050121	0.071	0.066	1	AWAT2
ZNF486	0.00022558	0.20979044	0.087	0.079	1	ZNF486
CCDC191	0.07039372	0.20974361	0.047	0.045	1	CCDC191
MYO15A	0.09137737	0.20919462	0.04	0.038	1	MYO15A

PRSS33	0.50601935	0.20868118	0.017	0.016	1	PRSS33
KCNIP2	0.00475713	0.20788153	0.14	0.135	1	KCNIP2
NEK3	0.03971697	0.20778893	0.021	0.019	1	NEK3
DGKG	0.00046893	0.20698985	0.085	0.078	1	DGKG
CLBA1	0.0150115	0.20651326	0.048	0.044	1	CLBA1
DUSP5	0.04231356	0.20549541	0.061	0.058	1	DUSP5
GPR150	0.44929871	0.20501134	0.011	0.01	1	GPR150
PRKN	0.00010536	0.20456284	0.124	0.115	1	PRKN
D2HGDH	0.02721856	0.20425651	0.063	0.06	1	D2HGDH
USP2	0.05800043	0.20386066	0.044	0.041	1	USP2
MPZL3	0.02542704	0.20376017	0.062	0.058	1	MPZL3
C17orf100	0.50449983	0.2033211	0.01	0.01	1	C17orf100
NUDT13	0.02135113	0.20309662	0.057	0.053	1	NUDT13
TCF4	0.01603793	0.20280986	0.07	0.065	1	TCF4
GASK1B	0.17380075	0.20259114	0.02	0.018	1	GASK1B
TRGJP1	0.00010634	0.20225774	0.065	0.057	1	TRGJP1
MYMX	0.84594816	0.20180919	0.014	0.014	1	MYMX
PEG3	0.05469029	0.20151058	0.047	0.044	1	PEG3
C1R	0.48270776	0.20112491	0.02	0.019	1	C1R
TRGC1	0.00291526	0.19983401	0.047	0.042	1	TRGC1
CCT6B	0.04707828	0.19968006	0.045	0.042	1	CCT6B
NUDT18	8.7528E-05	0.19892446	0.116	0.107	1	NUDT18
NHSL2	0.97127051	0.19870311	0.022	0.022	1	NHSL2
ARHGEF40	0.03254716	0.19777913	0.092	0.088	1	ARHGEF40
PPFIA3	0.32086874	0.19739834	0.013	0.012	1	PPFIA3
NAT14	0.01907159	0.19739178	0.042	0.039	1	NAT14
CSRP2	0.86298333	0.19682026	0.018	0.018	1	CSRP2
UFSP1	0.15984153	0.19662893	0.015	0.014	1	UFSP1
RHEBL1	0.07352344	0.19592208	0.028	0.026	1	RHEBL1
CCL4L2	0.11427923	0.19558622	0.021	0.02	1	CCL4L2
XPNPEP3	0.00250404	0.19546745	0.104	0.098	1	XPNPEP3
MYL5	0.00042176	0.19534346	0.094	0.086	1	MYL5
CD38	0.00134277	0.19522807	0.118	0.111	1	CD38
HACL1	0.00023041	0.19465826	0.137	0.129	1	HACL1
ADAMTS13	0.31570105	0.19429769	0.013	0.012	1	ADAMTS13
HRH1	0.02564694	0.19411406	0.09	0.086	1	HRH1
PDE8B	0.02875547	0.19408562	0.046	0.043	1	PDE8B
EPDR1	0.0438955	0.19354061	0.061	0.058	1	EPDR1
TRMT61A	8.3663E-05	0.19352171	0.186	0.177	1	TRMT61A
ERO1B	0.00050678	0.19284375	0.142	0.134	1	ERO1B
PCP2	0.2721077	0.19245312	0.047	0.046	1	PCP2
ANXA2R	0.11861849	0.19199303	0.037	0.035	1	ANXA2R
ASB1	0.00037489	0.18970903	0.158	0.15	1	ASB1
SIGLEC6	0.61617522	0.18955789	0.018	0.017	1	SIGLEC6
MICU3	0.45857706	0.18933886	0.016	0.016	1	MICU3
PRKCB	0.89864605	0.18831343	0.023	0.023	1	PRKCB
FBXO44	0.00026459	0.18754048	0.153	0.145	1	FBXO44
TFB1M	8.3405E-05	0.18700932	0.18	0.171	1	TFB1M
PIWIL4	0.02651978	0.18598288	0.053	0.049	1	PIWIL4
LZTS3	0.43437041	0.1858712	0.017	0.016	1	LZTS3
APOM	0.26965711	0.18558789	0.027	0.025	1	APOM
NMNAT3	0.02239808	0.18546107	0.051	0.048	1	NMNAT3

KCNMB3	0.23445648	0.18305014	0.03	0.028	1	KCNMB3
RUNDC3A	0.05614504	0.18257181	0.022	0.02	1	RUNDC3A
ARL16	0.00684334	0.18230917	0.141	0.136	1	ARL16
SWI5	0.00013429	0.18210398	0.189	0.18	1	SWI5
VWF	0.10599802	0.18088548	0.037	0.034	1	VWF
SMARCD3	0.48973716	0.1801911	0.03	0.029	1	SMARCD3
F8A1	0.02255379	0.17910202	0.152	0.148	1	F8A1
PALLD	0.00509996	0.17819363	0.067	0.062	1	PALLD
PRELID2	0.07488309	0.17749301	0.081	0.078	1	PRELID2
MTG2	7.8034E-05	0.1770674	0.193	0.184	1	MTG2
SERPINF1	0.07226151	0.17670097	0.074	0.07	1	SERPINF1
TRIM47	0.2402592	0.17642628	0.081	0.079	1	TRIM47
NBPF1	0.0075696	0.17628551	0.083	0.078	1	NBPF1
NR4A2	0.00275047	0.17595445	0.471	0.518	1	NR4A2
SATB1	0.04409502	0.17568632	0.083	0.079	1	SATB1
THAP3	0.0009448	0.17482377	0.151	0.144	1	THAP3
FDXR	0.00179819	0.17473753	0.162	0.154	1	FDXR
HYAL3	0.15994873	0.17472433	0.015	0.014	1	HYAL3
ARHGEF19	0.35551601	0.17471367	0.025	0.024	1	ARHGEF19
SLC22A18AS	0.97425235	0.17468721	0.05	0.05	1	SLC22A18AS
C1QTNF3	0.23392705	0.17380617	0.01	0.009	1	C1QTNF3
ADGRG2	0.22817159	0.17333974	0.01	0.009	1	ADGRG2
NUDT12	0.0533458	0.1731255	0.143	0.141	1	NUDT12
TEDC1	8.0692E-05	0.17282893	0.275	0.267	1	TEDC1
TSSK6	0.49431658	0.17281332	0.014	0.014	1	TSSK6
RGL3	0.05633043	0.17275009	0.133	0.129	1	RGL3
ERRFI1	0.1330177	0.17199126	0.406	0.415	1	ERRFI1
TMEM88	0.22025494	0.17173234	0.024	0.023	1	TMEM88
TNFAIP8L1	0.15045978	0.17130619	0.063	0.061	1	TNFAIP8L1
CDPF1	0.0013349	0.17088786	0.144	0.137	1	CDPF1
MAP1LC3A	0.00128451	0.17065271	0.136	0.129	1	MAP1LC3A
PLEKHG4	0.41678519	0.17025154	0.028	0.027	1	PLEKHG4
WDR5B	0.00064546	0.16956062	0.105	0.098	1	WDR5B
ZNF681	0.07007744	0.16884755	0.026	0.024	1	ZNF681
SH3BGR	0.22608632	0.16873218	0.036	0.034	1	SH3BGR
ZNF165	0.43020059	0.16872441	0.019	0.018	1	ZNF165
GADD45A	0.00453918	0.16864514	0.186	0.18	1	GADD45A
GLTPD2	0.12050092	0.16841356	0.027	0.025	1	GLTPD2
RAC3	0.16275683	0.16837833	0.065	0.062	1	RAC3
B3GNTL1	0.01257015	0.16806956	0.11	0.105	1	B3GNTL1
FCRL1	0.10269049	0.1673393	0.182	0.18	1	FCRL1
GGACT	0.15418893	0.16713723	0.071	0.069	1	GGACT
ENGASE	0.17144646	0.16708419	0.138	0.137	1	ENGASE
EID2B	0.31825604	0.16688453	0.025	0.024	1	EID2B
METAP1D	0.23809655	0.16677592	0.027	0.026	1	METAP1D
FLNB	0.00011731	0.16653667	0.155	0.145	1	FLNB
C6orf163	0.48444532	0.16644992	0.023	0.022	1	C6orf163
ZNF48	0.32784718	0.16555916	0.038	0.036	1	ZNF48
CCDC24	0.00812159	0.165418	0.094	0.089	1	CCDC24
TCTEX1D4	0.22179526	0.16534374	0.019	0.018	1	TCTEX1D4
ZBTB46	0.13721788	0.1650877	0.034	0.032	1	ZBTB46
SNRPF	0.02961783	0.16496672	0.107	0.103	1	SNRPF

VANGL1	0.03168983	0.16406939	0.111	0.106	1	VANGL1
NEK11	0.02849392	0.16402649	0.101	0.097	1	NEK11
C7orf31	0.00041674	0.1637999	0.163	0.155	1	C7orf31
DRD5	0.09215121	0.16352401	0.023	0.021	1	DRD5
LRFN1	0.19630673	0.16349911	0.035	0.033	1	LRFN1
PBLD	0.00976355	0.16335051	0.123	0.118	1	PBLD
PRDM1	0.55991274	0.16326338	0.031	0.031	1	PRDM1
ECHDC3	0.00139654	0.16311615	0.228	0.222	1	ECHDC3
DPY19L3	0.06864315	0.16261246	0.129	0.126	1	DPY19L3
TRAF1	0.06473399	0.16221535	0.034	0.032	1	TRAF1
B3GLCT	0.00276819	0.16191783	0.173	0.166	1	B3GLCT
LRRC69	0.37947512	0.16176459	0.03	0.028	1	LRRC69
PAIP2B	0.11028825	0.16170286	0.036	0.034	1	PAIP2B
NAV1	0.00282477	0.16127757	0.18	0.174	1	NAV1
SDHAF1	0.00120437	0.16127556	0.211	0.204	1	SDHAF1
ENDOV	0.01249863	0.16084796	0.115	0.109	1	ENDOV
MTFP1	0.04972872	0.1606157	0.141	0.137	1	MTFP1
ZNF496	0.00737539	0.16052807	0.12	0.114	1	ZNF496
ZNF226	0.00237349	0.16043818	0.106	0.099	1	ZNF226
E2F5	0.98158332	0.15999902	0.016	0.016	1	E2F5
TMEM52B	0.05410897	0.15980356	0.091	0.087	1	TMEM52B
FANK1	0.43111021	0.15964193	0.011	0.01	1	FANK1
ZFP69B	0.36666982	0.15926415	0.043	0.042	1	ZFP69B
CXCR4	0.21372227	0.15922933	0.312	0.325	1	CXCR4
DUS4L	0.01778498	0.15922012	0.119	0.115	1	DUS4L
SPRY1	0.00059735	0.15907712	0.271	0.264	1	SPRY1
PIM3	0.0005212	0.15863143	0.382	0.382	1	PIM3
SPECC1	0.04263774	0.15834162	0.045	0.042	1	SPECC1
USP13	0.03088809	0.15783189	0.095	0.091	1	USP13
SPDL1	0.74633361	0.15764955	0.02	0.02	1	SPDL1
RELL2	0.042099	0.1569557	0.064	0.061	1	RELL2
SCGB1A1	0.12117191	0.15632235	0.02	0.022	1	SCGB1A1
ZNF658	0.37258828	0.15576809	0.037	0.036	1	ZNF658
CATSPER2	0.36931763	0.15556953	0.031	0.03	1	CATSPER2
C2CD2	0.15256037	0.15532586	0.123	0.121	1	C2CD2
LRTOMT	0.06883192	0.15530811	0.073	0.07	1	LRTOMT
FLVCR1	0.00118572	0.155283	0.175	0.168	1	FLVCR1
PDE4D	0.83291137	0.15515237	0.01	0.011	1	PDE4D
RIMKLB	0.00049469	0.15484408	0.212	0.205	1	RIMKLB
TBC1D25	0.04189248	0.15472933	0.083	0.079	1	TBC1D25
NOXA1	0.0175535	0.15457416	0.101	0.096	1	NOXA1
ELMO3	0.29707773	0.15446217	0.019	0.018	1	ELMO3
PTK6	0.4666073	0.15438767	0.064	0.063	1	PTK6
CDC42EP4	0.00014278	0.15431885	0.204	0.194	1	CDC42EP4
ABT1	0.08110689	0.15391775	0.115	0.112	1	ABT1
LSS	0.00010125	0.1536303	0.315	0.311	1	LSS
C19orf73	0.94123375	0.15297953	0.014	0.014	1	C19orf73
CDKN2A	0.12346601	0.1527052	0.089	0.086	1	CDKN2A
ZNF233	0.48797169	0.1526414	0.013	0.013	1	ZNF233
EPS8L1	0.04691315	0.15232236	0.131	0.128	1	EPS8L1
HIST4H4	0.95404948	0.15209076	0.028	0.028	1	HIST4H4
PARD6B	0.30143688	0.15190187	0.025	0.024	1	PARD6B

TULP3	0.08776566	0.15165389	0.092	0.089	1	TULP3
BIN3	0.01247027	0.15149005	0.144	0.139	1	BIN3
SPATA24	0.47766692	0.15117903	0.023	0.022	1	SPATA24
HOXB5	0.4179732	0.15097755	0.01	0.01	1	HOXB5
NREP	0.19490878	0.15023915	0.125	0.124	1	NREP
BORCS5	0.00076505	0.1500126	0.322	0.318	1	BORCS5
ZNF829	0.45521377	0.14955225	0.029	0.028	1	ZNF829
ARSK	0.71851945	0.14954443	0.026	0.026	1	ARSK
STAP2	0.23440571	0.14881591	0.035	0.033	1	STAP2
NCBP2AS2	0.0003621	0.14870035	0.265	0.258	1	NCBP2AS2
JAK3	0.63535015	0.1479651	0.044	0.044	1	JAK3
MVK	0.00252173	0.14721232	0.267	0.262	1	MVK
SPATA33	0.08547248	0.14714392	0.114	0.111	1	SPATA33
CIAPIN1	0.01021682	0.14691095	0.178	0.173	1	CIAPIN1
CARHSP1	0.00643268	0.14680265	0.214	0.21	1	CARHSP1
TMEM182	0.16382468	0.14645685	0.077	0.075	1	TMEM182
YEATS4	0.00011193	0.14507115	0.244	0.235	1	YEATS4
UST	0.39729448	0.14499313	0.103	0.102	1	UST
ANKZF1	0.12018198	0.14489129	0.188	0.186	1	ANKZF1
TRAF2	0.21618364	0.14470476	0.122	0.121	1	TRAF2
SOWAHD	9.4421E-05	0.14396557	0.301	0.293	1	SOWAHD
NSUN7	0.00011402	0.14390368	0.172	0.163	1	NSUN7
MPZ	0.06182281	0.14364907	0.067	0.064	1	MPZ
SMG9	0.00091983	0.14361241	0.266	0.26	1	SMG9
CYB5D1	0.57415984	0.14299473	0.056	0.056	1	CYB5D1
ZNF579	0.04867356	0.1429074	0.122	0.118	1	ZNF579
SLC29A2	0.8439203	0.14279105	0.017	0.017	1	SLC29A2
ZNF205	0.01681165	0.14246743	0.114	0.108	1	ZNF205
NPM3	0.00049664	0.14196524	0.258	0.25	1	NPM3
HMGCR	7.6378E-05	0.14162382	0.495	0.499	1	HMGCR
ZNF333	0.00042214	0.14082298	0.191	0.182	1	ZNF333
ASB6	0.02253207	0.14053366	0.214	0.211	1	ASB6
ZNF77	0.18936042	0.1404743	0.064	0.062	1	ZNF77
USP27X	0.33190311	0.14013264	0.028	0.026	1	USP27X
MORN4	0.21672547	0.13994603	0.037	0.035	1	MORN4
SLC25A12	0.00023874	0.13992585	0.303	0.298	1	SLC25A12
BTF3	0.01812248	0.13979998	0.175	0.17	1	BTF3
BCDIN3D	0.03028469	0.13965711	0.109	0.104	1	BCDIN3D
CHAF1B	0.63697954	0.13962141	0.016	0.016	1	CHAF1B
ARID5B	0.71345285	0.13942928	0.063	0.064	1	ARID5B
RBM11	0.33984687	0.13920105	0.119	0.118	1	RBM11
TPCN2	0.15009624	0.13893681	0.099	0.096	1	TPCN2
ANGPTL1	0.27414549	0.13889764	0.028	0.027	1	ANGPTL1
DDX51	0.10503948	0.13867011	0.143	0.14	1	DDX51
POU5F2	0.72556611	0.13862858	0.033	0.034	1	POU5F2
FAAP24	0.04588716	0.13853782	0.108	0.104	1	FAAP24
MAGEH1	0.05940015	0.13848452	0.118	0.114	1	MAGEH1
RALGPS1	0.34615651	0.13832701	0.089	0.088	1	RALGPS1
MAFB	0.01571918	0.13789916	0.572	0.586	1	MAFB
C6orf226	0.01094151	0.13764253	0.147	0.142	1	C6orf226
HIKESHI	0.02459059	0.13745095	0.119	0.114	1	HIKESHI
SPIN2B	0.50492501	0.13742779	0.018	0.017	1	SPIN2B

ODF3L1	0.03499338	0.13741075	0.269	0.268	1	ODF3L1
PPP1R3F	0.64063511	0.13722627	0.066	0.065	1	PPP1R3F
DNAH7	0.47859738	0.13720766	0.011	0.011	1	DNAH7
MTHFD2	0.00015272	0.13676159	0.556	0.567	1	MTHFD2
CHRNA5	0.10504028	0.13640849	0.048	0.045	1	CHRNA5
MAGEF1	0.81484521	0.13630024	0.085	0.085	1	MAGEF1
TTC30A	0.1317733	0.13627304	0.04	0.038	1	TTC30A
NAIF1	0.00961751	0.13613681	0.227	0.223	1	NAIF1
MED20	0.00220553	0.13580395	0.267	0.261	1	MED20
PIP4P2	0.15772282	0.13577922	0.053	0.05	1	PIP4P2
RPP14	0.92290711	0.1357214	0.056	0.056	1	RPP14
ARIH2OS	0.59180596	0.13543515	0.03	0.029	1	ARIH2OS
RPP25L	0.00082479	0.1354118	0.221	0.214	1	RPP25L
SUMO4	0.9415471	0.13531084	0.01	0.01	1	SUMO4
CCDC180	0.85459647	0.13529636	0.015	0.015	1	CCDC180
ZSCAN29	0.00016101	0.13454938	0.266	0.258	1	ZSCAN29
CEP70	0.43647063	0.13442919	0.059	0.058	1	CEP70
IL6	0.53939243	0.13438217	0.032	0.033	1	IL6
TTPAL	0.10413314	0.13415088	0.153	0.15	1	TTPAL
IL1B	0.00078856	0.13398008	0.515	0.546	1	IL1B
CHTF18	0.87082586	0.13391439	0.038	0.037	1	CHTF18
GNE	0.00232297	0.1338197	0.215	0.209	1	GNE
ANKRD46	0.46208959	0.13373748	0.092	0.092	1	ANKRD46
SHF	0.76817654	0.13347754	0.022	0.022	1	SHF
BCAT1	0.83665254	0.13333569	0.16	0.162	1	BCAT1
SLC25A34	0.87281411	0.13317668	0.011	0.011	1	SLC25A34
PIK3IP1	0.46882037	0.13293495	0.117	0.116	1	PIK3IP1
RABL3	0.00123454	0.13290686	0.282	0.277	1	RABL3
DDB2	0.00660647	0.13273646	0.222	0.217	1	DDB2
MIEF2	0.04214502	0.1324552	0.128	0.124	1	MIEF2
PMM1	0.00100009	0.13230436	0.231	0.224	1	PMM1
RGS18	0.0135353	0.13225732	0.118	0.113	1	RGS18
PPP1R35	8.4863E-05	0.13185663	0.332	0.326	1	PPP1R35
BCORL1	0.49540436	0.13150045	0.067	0.066	1	BCORL1
PRCD	0.5703044	0.13142879	0.02	0.019	1	PRCD
ZNF564	0.27100845	0.13131324	0.059	0.057	1	ZNF564
ZNF852	0.8034474	0.13116656	0.03	0.029	1	ZNF852
ARHGAP32	0.34973234	0.13107637	0.092	0.091	1	ARHGAP32
CCZ1	0.96388065	0.13100135	0.016	0.017	1	CCZ1
FAM47E	0.08794319	0.13066835	0.032	0.03	1	FAM47E
LCA5	0.6889666	0.12990755	0.011	0.01	1	LCA5
ZNF354C	0.21676824	0.12959554	0.12	0.119	1	ZNF354C
TBC1D1	0.08296037	0.12931593	0.05	0.047	1	TBC1D1
TRIM45	0.67573255	0.12931341	0.04	0.039	1	TRIM45
CCDC34	0.42839446	0.12923426	0.038	0.037	1	CCDC34
GSTZ1	0.01610921	0.12912147	0.217	0.213	1	GSTZ1
SPATC1	0.30713294	0.1290177	0.047	0.046	1	SPATC1
MT1F	0.12562729	0.12897969	0.098	0.095	1	MT1F
RASL11A	0.00323784	0.12896239	0.297	0.293	1	RASL11A
NEDD4L	0.89800902	0.12883111	0.038	0.039	1	NEDD4L
SMIM27	0.0105758	0.12882244	0.204	0.199	1	SMIM27
FBXO4	0.0061036	0.12878519	0.174	0.168	1	FBXO4

MYO15B	0.264455	0.12849595	0.208	0.209	1	MYO15B
FANCF	0.00213316	0.12824795	0.218	0.211	1	FANCF
TAF1B	0.00087849	0.12797134	0.235	0.228	1	TAF1B
PGGHG	0.32880716	0.12796764	0.062	0.06	1	PGGHG
ZNF554	0.06256145	0.12754388	0.088	0.085	1	ZNF554
ZNF568	0.00522286	0.12753904	0.207	0.201	1	ZNF568
ZNF440	0.26370989	0.1273445	0.078	0.076	1	ZNF440
TRIM59	0.66649404	0.12706053	0.043	0.043	1	TRIM59
ZNF544	0.00125176	0.12668852	0.277	0.271	1	ZNF544
GLIS3	0.33521809	0.12625587	0.051	0.049	1	GLIS3
FCRL2	0.26633049	0.12622786	0.102	0.106	1	FCRL2
ZNF229	0.09122122	0.1261593	0.115	0.112	1	ZNF229
CALML4	0.04166858	0.12611013	0.186	0.183	1	CALML4
THEM6	0.01645146	0.12601933	0.26	0.257	1	THEM6
TIPIN	0.0936324	0.12592846	0.055	0.052	1	TIPIN
ZNF875	0.98005202	0.12559493	0.043	0.044	1	ZNF875
HELB	0.2225624	0.12544434	0.106	0.103	1	HELB
TAS2R4	0.75783101	0.12488894	0.016	0.017	1	TAS2R4
ATP8B3	0.95332808	0.12481737	0.071	0.072	1	ATP8B3
ARHGEF5	0.04059263	0.12453795	0.27	0.269	1	ARHGEF5
HOXB7	0.37144595	0.12444683	0.165	0.165	1	HOXB7
FAHD2B	0.22791288	0.1240886	0.06	0.058	1	FAHD2B
CCDC134	0.52558226	0.12384477	0.073	0.072	1	CCDC134
PJVK	0.99049078	0.12384008	0.015	0.015	1	PJVK
ZNF688	0.07320535	0.12369673	0.142	0.139	1	ZNF688
MYL9	0.84393286	0.12364291	0.034	0.033	1	MYL9
CLCN6	0.04841133	0.12348289	0.203	0.201	1	CLCN6
RHBDD3	0.31196871	0.12341539	0.114	0.113	1	RHBDD3
GPANK1	0.00147464	0.1232571	0.307	0.302	1	GPANK1
MRTFB	0.0001247	0.12304846	0.352	0.347	1	MRTFB
FCHSD1	0.0606774	0.12284425	0.105	0.101	1	FCHSD1
ZBTB12	0.45737352	0.12274847	0.035	0.034	1	ZBTB12
NAALADL1	0.07910289	0.12266093	0.139	0.136	1	NAALADL1
HAAO	0.14770573	0.12257492	0.092	0.089	1	HAAO
DYNC2LI1	0.0008917	0.12231851	0.229	0.221	1	DYNC2LI1
PEX7	0.04240882	0.12229886	0.177	0.174	1	PEX7
DCHS1	0.88956949	0.12210723	0.01	0.01	1	DCHS1
CRTAM	0.07271648	0.12130528	0.241	0.24	1	CRTAM
VSIG10	0.10497884	0.12122344	0.088	0.085	1	VSIG10
GCSAM	0.45558055	0.12119294	0.015	0.014	1	GCSAM
CREB3L4	0.31748511	0.12110124	0.052	0.05	1	CREB3L4
TRIM35	0.0004746	0.120663	0.33	0.325	1	TRIM35
CNTROB	0.01963966	0.12053502	0.231	0.227	1	CNTROB
ST3GAL4	0.26718221	0.12048888	0.078	0.076	1	ST3GAL4
LRP2	0.70135639	0.12009968	0.011	0.011	1	LRP2
BLOC1S3	0.00532998	0.11988556	0.32	0.318	1	BLOC1S3
TMEM107	0.00208782	0.11975949	0.312	0.309	1	TMEM107
TRAF3	0.11897828	0.11974051	0.299	0.302	1	TRAF3
MBLAC2	0.97748446	0.1197401	0.065	0.065	1	MBLAC2
BRPF3	9.3376E-05	0.11964813	0.328	0.322	1	BRPF3
ERCC4	0.03923052	0.11959286	0.157	0.153	1	ERCC4
SHB	0.00013044	0.1194895	0.373	0.369	1	SHB

NIPSNAP1	0.00082133	0.11930186	0.27	0.264	1	NIPSNAP1
RBM43	0.03939987	0.11927661	0.157	0.153	1	RBM43
ZNF684	0.04865066	0.11878037	0.097	0.093	1	ZNF684
ATP5MC1	0.00010698	0.11864293	0.342	0.336	1	ATP5MC1
DNAJC30	0.13123977	0.1186047	0.136	0.134	1	DNAJC30
TOMM40L	0.15475884	0.11858117	0.207	0.207	1	TOMM40L
TCTN2	0.15259711	0.11835336	0.144	0.142	1	TCTN2
CIT	0.46632921	0.11834011	0.022	0.023	1	CIT
AL162231.1	0.334253	0.11807723	0.022	0.021	1	AL162231.1
CEBPD	0.00032023	0.11801131	0.398	0.396	1	CEBPD
FOXO1	0.00215293	0.11798368	0.35	0.348	1	FOXO1
ZNHIT2	0.03085491	0.11771104	0.209	0.206	1	ZNHIT2
ARAP2	0.00692648	0.11768504	0.218	0.213	1	ARAP2
HACD1	0.18855965	0.11752088	0.071	0.069	1	HACD1
FAM98C	0.09095867	0.11727291	0.209	0.207	1	FAM98C
FKBPL	0.91236146	0.11724842	0.102	0.102	1	FKBPL
RNF157	0.36845629	0.11717275	0.029	0.028	1	RNF157
SLC7A1	0.06499079	0.11694266	0.14	0.137	1	SLC7A1
GFER	0.00194062	0.11677898	0.271	0.266	1	GFER
TMEM18	0.00182707	0.11658793	0.303	0.299	1	TMEM18
ZNF671	0.15609513	0.11641323	0.23	0.231	1	ZNF671
TMCO6	0.41639036	0.11640831	0.084	0.082	1	TMCO6
SPATA2L	0.08053682	0.1162601	0.14	0.136	1	SPATA2L
SPICE1	0.32626675	0.11617361	0.139	0.139	1	SPICE1
RFXAP	0.25347886	0.11565963	0.088	0.086	1	RFXAP
SHMT2	0.39255939	0.11564326	0.137	0.137	1	SHMT2
GNA12	0.27272942	0.11559484	0.102	0.1	1	GNA12
C17orf64	0.93955206	0.11550712	0.029	0.029	1	C17orf64
HORMAD1	0.66055477	0.11542797	0.026	0.026	1	HORMAD1
MACROD1	0.21635638	0.11533534	0.19	0.189	1	MACROD1
AP4M1	0.30215308	0.11505967	0.155	0.154	1	AP4M1
HMGN5	0.6598627	0.11503406	0.085	0.085	1	HMGN5
USP35	0.50817825	0.11492241	0.04	0.039	1	USP35
NDRG1	0.04822513	0.11478987	0.42	0.427	1	NDRG1
BOLA1	0.48659136	0.11474783	0.08	0.079	1	BOLA1
CASTOR1	0.74596754	0.11457127	0.043	0.042	1	CASTOR1
ZNF747	0.32792102	0.1144735	0.068	0.066	1	ZNF747
TAP1	8.1576E-05	0.11426622	0.735	0.759	1	TAP1
BCL2L12	0.2249367	0.11395162	0.163	0.162	1	BCL2L12
IER2	0.00919177	0.11380594	0.69	0.7	1	IER2
TRMT10B	0.78837854	0.11377736	0.118	0.119	1	TRMT10B
SCARF1	0.21090298	0.11376369	0.102	0.1	1	SCARF1
ZNF154	0.7407119	0.11366888	0.013	0.014	1	ZNF154
PCYOX1	0.00097987	0.11330155	0.329	0.325	1	PCYOX1
IDI1	0.5462204	0.11302907	0.322	0.33	1	IDI1
UMPS	0.00326992	0.11301573	0.251	0.245	1	UMPS
B3GALT4	0.0053937	0.11300327	0.28	0.277	1	B3GALT4
PPIF	0.00402879	0.11298588	0.506	0.515	1	PPIF
FAM216A	0.7109508	0.11276556	0.037	0.036	1	FAM216A
SPEF2	0.79744858	0.11271213	0.033	0.033	1	SPEF2
ZBP1	0.53916013	0.11246478	0.167	0.167	1	ZBP1
FRMD8	0.55138928	0.11242927	0.049	0.048	1	FRMD8

BEND3	0.93211294	0.11233169	0.023	0.023	1	BEND3
H2AFY2	0.21673723	0.11229619	0.104	0.102	1	H2AFY2
DPH5	0.02589744	0.1121661	0.234	0.231	1	DPH5
MOSMO	0.23354952	0.11216266	0.153	0.151	1	MOSMO
RDX	7.9516E-05	0.11201474	0.361	0.356	1	RDX
DNAJC6	0.85453481	0.11198776	0.196	0.201	1	DNAJC6
MRM1	0.75452114	0.11180066	0.09	0.09	1	MRM1
CRYZ	0.00058354	0.11164177	0.306	0.299	1	CRYZ
TOR4A	0.00144654	0.11161614	0.33	0.326	1	TOR4A
HOMER3	0.50541928	0.11125807	0.06	0.062	1	HOMER3
MICAL3	0.14396995	0.11121591	0.089	0.086	1	MICAL3
C11orf80	0.53492829	0.11110194	0.097	0.097	1	C11orf80
CEP89	0.10374095	0.11101557	0.186	0.184	1	CEP89
PRXL2B	0.95539736	0.11092927	0.083	0.083	1	PRXL2B
SOGA3	0.98907549	0.11077653	0.039	0.039	1	SOGA3
CDKN2B	0.30126487	0.11076455	0.062	0.065	1	CDKN2B
SLC22A5	0.00026123	0.11050423	0.343	0.337	1	SLC22A5
TMEM42	0.03023097	0.11012463	0.223	0.219	1	TMEM42
DCSTAMP	0.01360433	0.10977289	0.366	0.367	1	DCSTAMP
ZFPL1	0.94682506	0.10972398	0.095	0.096	1	ZFPL1
ABRAXAS1	0.36539095	0.10962922	0.108	0.107	1	ABRAXAS1
ABHD17C	0.30771651	0.10943077	0.059	0.057	1	ABHD17C
IPP	0.8224974	0.10924175	0.092	0.092	1	IPP
LOXL3	0.01618663	0.10916443	0.224	0.22	1	LOXL3
C19orf48	0.10997403	0.1091397	0.16	0.157	1	C19orf48
DUSP2	0.11381116	0.10850532	0.055	0.052	1	DUSP2
CXorf21	0.0007894	0.10840128	0.374	0.372	1	CXorf21
HSCB	0.05933797	0.10835822	0.176	0.173	1	HSCB
ZNF675	0.41577921	0.10832424	0.11	0.109	1	ZNF675
ZNF527	0.88013513	0.1082898	0.09	0.091	1	ZNF527
MC1R	0.85414093	0.10827801	0.014	0.013	1	MC1R
ZNF697	0.54961741	0.10821066	0.076	0.075	1	ZNF697
NUDT19	0.02162724	0.10814884	0.386	0.39	1	NUDT19
SFMBT1	0.24369483	0.10799945	0.122	0.12	1	SFMBT1
MOB3C	0.08381753	0.10763012	0.323	0.325	1	MOB3C
NFIX	0.93160933	0.10761951	0.101	0.102	1	NFIX
PAN2	0.00325043	0.10757269	0.323	0.32	1	PAN2
FAM110B	0.04938815	0.10748078	0.272	0.27	1	FAM110B
ZFAND1	0.00035697	0.10714193	0.363	0.358	1	ZFAND1
CENPW	0.09302868	0.1068784	0.35	0.354	1	CENPW
ZNF836	0.70101088	0.10680365	0.094	0.095	1	ZNF836
PIM2	0.05510071	0.10661288	0.276	0.275	1	PIM2
EXOSC3	0.00615422	0.10659721	0.331	0.33	1	EXOSC3
ALDH1B1	0.21641384	0.1064649	0.162	0.16	1	ALDH1B1
INPP1	0.12169553	0.1064427	0.203	0.201	1	INPP1
KLHDC9	0.27864266	0.10638287	0.033	0.032	1	KLHDC9
PDK2	0.04843634	0.10627729	0.267	0.266	1	PDK2
CLK3	8.3297E-05	0.10627482	0.406	0.402	1	CLK3
TMEM14B	0.0142559	0.10626882	0.287	0.285	1	TMEM14B
HAUS8	0.93950642	0.10622279	0.109	0.11	1	HAUS8
SFXN5	0.08012376	0.10617145	0.191	0.188	1	SFXN5
PPP2R3B	0.06911153	0.10605602	0.189	0.187	1	PPP2R3B

ZC3H12A	0.07616541	0.1060421	0.287	0.287	1	ZC3H12A
ZNF503	0.82584896	0.10591818	0.173	0.175	1	ZNF503
ZNF277	0.00078559	0.10584843	0.361	0.358	1	ZNF277
ACTL10	0.66607339	0.10577698	0.033	0.033	1	ACTL10
ZNF835	0.16012612	0.1055737	0.085	0.082	1	ZNF835
PLGLB2	0.13040775	0.10546194	0.044	0.047	1	PLGLB2
LTO1	0.74077631	0.10538458	0.062	0.062	1	LTO1
DZANK1	0.82076152	0.10533147	0.073	0.073	1	DZANK1
ELAC1	0.0960634	0.10510495	0.185	0.183	1	ELAC1
NFKBIB	0.0303642	0.10510074	0.267	0.266	1	NFKBIB
XK	0.12577747	0.1049933	0.062	0.059	1	XK
PASK	0.99594606	0.10493502	0.06	0.061	1	PASK
ASMTL	0.11434075	0.10456226	0.14	0.137	1	ASMTL
NXF3	0.20560541	0.1044885	0.064	0.062	1	NXF3
SH3D21	0.87775979	0.10441674	0.016	0.015	1	SH3D21
LYRM1	0.0341543	0.10432285	0.339	0.34	1	LYRM1
CCDC102B	0.92416417	0.10427537	0.032	0.032	1	CCDC102B
ZFP57	0.058255	0.10427319	0.093	0.089	1	ZFP57
GGT7	0.57937908	0.10411405	0.092	0.091	1	GGT7
PXDC1	0.04461013	0.10407277	0.353	0.356	1	PXDC1
EPHA2	0.95441634	0.10399197	0.054	0.054	1	EPHA2
ZBTB22	0.00120831	0.10397841	0.35	0.346	1	ZBTB22
RAB40B	0.73054946	0.10393486	0.012	0.012	1	RAB40B
NMRK1	0.00020593	0.103762	0.388	0.384	1	NMRK1
POGLUT1	0.00070783	0.10357258	0.287	0.28	1	POGLUT1
MAN1C1	0.006043	0.10351256	0.287	0.283	1	MAN1C1
MAP3K9	0.47367419	0.10350185	0.019	0.02	1	MAP3K9
JAG1	0.9446702	0.10343825	0.231	0.236	1	JAG1
CADM4	0.69931996	0.10319079	0.013	0.012	1	CADM4
POU2F1	0.02498275	0.10315634	0.282	0.28	1	POU2F1
CPLANE2	0.79746545	0.10315058	0.046	0.046	1	CPLANE2
MAGIX	0.92349903	0.1031407	0.044	0.044	1	MAGIX
SPAG1	0.86381908	0.10293054	0.083	0.084	1	SPAG1
STARD10	0.07052492	0.10291342	0.322	0.325	1	STARD10
SIK3	0.04619904	0.10288966	0.305	0.306	1	SIK3
NR2C2AP	0.02756771	0.10284266	0.27	0.268	1	NR2C2AP
GSPT2	0.16091923	0.10273179	0.132	0.129	1	GSPT2
ELOVL7	0.4395737	0.102656	0.085	0.084	1	ELOVL7
BCOR	0.00217095	0.10263294	0.42	0.419	1	BCOR
KIAA0556	0.03682745	0.10253422	0.273	0.272	1	KIAA0556
TTC38	0.53903454	0.10248341	0.107	0.107	1	TTC38
NUDT7	0.50013922	0.10213385	0.081	0.08	1	NUDT7
CAVIN3	0.78246289	0.1020591	0.113	0.113	1	CAVIN3
PBXIP1	0.00050034	0.10205673	0.371	0.368	1	PBXIP1
PDZD7	0.13043081	0.10171001	0.077	0.074	1	PDZD7
SIPA1L1	0.08969227	0.10166395	0.141	0.138	1	SIPA1L1
GNPDA2	0.44363207	0.10162937	0.142	0.142	1	GNPDA2
TMEM201	0.64475426	0.10162539	0.121	0.121	1	TMEM201
ELL	0.21754121	0.10156994	0.291	0.295	1	ELL
WSCD2	0.19046372	0.10152548	0.009	0.011	1	WSCD2
ZNF441	0.35723158	0.10143852	0.137	0.136	1	ZNF441
ZNF595	0.42678753	0.10140467	0.175	0.175	1	ZNF595

AP5S1	0.05698089	0.10136259	0.259	0.258	1	AP5S1
GAS8	0.31266089	0.10135298	0.098	0.097	1	GAS8
NIP7	0.00011618	0.10116825	0.422	0.42	1	NIP7
CEMIP2	0.11492329	0.10112647	0.23	0.229	1	CEMIP2
IL17RC	0.00024521	0.10100137	0.417	0.416	1	IL17RC
RHPN1	0.73953274	0.10094537	0.037	0.037	1	RHPN1
DNAJC18	0.14891683	0.10090337	0.105	0.102	1	DNAJC18
MINDY4	0.67962172	0.10078784	0.022	0.022	1	MINDY4
C12orf10	0.01077098	0.10068771	0.341	0.34	1	C12orf10
PLAAT4	0.00022082	0.10066755	0.579	0.583	1	PLAAT4
MUL1	0.00056307	0.10030028	0.406	0.405	1	MUL1
RIPOR2	0.30269641	0.10027747	0.06	0.062	1	RIPOR2
TMEM222	0.28822457	0.10007506	0.169	0.168	1	TMEM222
HRH4	0	-3.6361545	0.008	0.096	0	HRH4
JUP	0	-3.5904103	0.028	0.245	0	JUP
AKAP12	0	-2.0380648	0.057	0.154	0	AKAP12
FGFR1	0	-1.9674535	0.026	0.106	0	FGFR1
PCGF2	0	-1.8051441	0.04	0.135	0	PCGF2
AMZ1	0	-1.6647259	0.079	0.24	0	AMZ1
A2M	0	-1.0930532	0.163	0.313	0	A2M
IGFBP2	0	-1.0302204	0.436	0.657	0	IGFBP2
SIGLEC5	0	-0.9590237	0.346	0.599	0	SIGLEC5
GOLM1	0	-0.8540893	0.217	0.363	0	GOLM1
C3AR1	0	-0.7571097	0.616	0.815	0	C3AR1
ITGAM	0	-0.7219992	0.518	0.695	0	ITGAM
PMP22	0	-0.6210274	0.395	0.568	0	PMP22
TGM2	0	-0.5492013	0.896	0.953	0	TGM2
FPR1	0	-0.5380511	0.787	0.885	0	FPR1
FGL2	0	-0.5286967	0.355	0.559	0	FGL2
CFD	0	-0.5087318	0.867	0.951	0	CFD
ACE	0	-0.3885645	0.85	0.912	0	ACE
NUPR1	0	-0.3291264	0.855	0.931	0	NUPR1
FLOT1	0	-0.3132114	0.89	0.939	0	FLOT1
GPNMB	0	-0.3059274	0.995	0.998	0	GPNMB
MARCO	0	-0.2683491	0.999	1	0	MARCO
TSPO	0	-0.2542289	1	1	0	TSPO
CREG1	0	-0.2022039	0.986	0.993	0	CREG1
FBP1	0	-0.2014205	0.999	1	0	FBP1
LAMP2	0	-0.1868188	0.993	0.996	0	LAMP2
FTL	0	-0.1826233	1	1	0	FTL
APOC2	2.482E-300	-0.773817	0.325	0.469	3.815E-296	APOC2
JAML	4.899E-296	-0.2245404	0.97	0.986	7.53E-292	JAML
CYP4V2	6.389E-285	-0.5450628	0.428	0.562	9.821E-281	CYP4V2
DEFB1	1.839E-268	-0.6062377	0.426	0.545	2.826E-264	DEFB1
ADCY3	9.013E-246	-0.533466	0.652	0.729	1.385E-241	ADCY3
MXRA7	1.122E-238	-0.8832947	0.149	0.256	1.724E-234	MXRA7
PCSK5	1.042E-236	-0.9635419	0.137	0.24	1.602E-232	PCSK5
GSDME	1.57E-235	-2.3274493	0.015	0.071	2.413E-231	GSDME
SLC28A3	2.035E-232	-2.8332548	0.008	0.058	3.128E-228	SLC28A3
SPTAN1	2.422E-232	-0.3089117	0.823	0.872	3.723E-228	SPTAN1
LTBR	6.203E-227	-0.286533	0.795	0.864	9.535E-223	LTBR
FPR2	7.097E-227	-0.3963056	0.613	0.719	1.091E-222	FPR2

CES1	3.763E-223	-0.2033015	0.878	0.992	5.785E-219	CES1
IL17RB	2.852E-214	-0.7470904	0.18	0.288	4.383E-210	IL17RB
CTSD	4.7E-209	-0.1326961	1	1	7.224E-205	CTSD
COMMD10	6.407E-203	-0.5046382	0.348	0.467	9.848E-199	COMMD10
ICAM4	1.026E-202	-1.1883401	0.056	0.129	1.577E-198	ICAM4
CCL18	4.044E-197	-0.4391679	0.629	0.709	6.216E-193	CCL18
CAPN3	9.627E-196	-0.7259495	0.227	0.33	1.48E-191	CAPN3
NAPRT	1.021E-194	-0.2705417	0.757	0.834	1.57E-190	NAPRT
EMILIN2	3.043E-194	-0.1719496	0.973	0.983	4.677E-190	EMILIN2
CCL23	9.22E-194	-0.5711537	0.335	0.45	1.417E-189	CCL23
COLEC12	1.145E-189	-0.2641413	0.9	0.874	1.76E-185	COLEC12
LILRB2	5.71E-189	-0.4600292	0.368	0.486	8.776E-185	LILRB2
MTUS1	7.117E-189	-1.0720106	0.065	0.138	1.094E-184	MTUS1
FCAR	4.79E-183	-0.4583371	0.151	0.253	7.363E-179	FCAR
BACE2	2.086E-176	-0.9628721	0.089	0.166	3.206E-172	BACE2
ID3	7.011E-173	-0.2990667	0.604	0.705	1.078E-168	ID3
FPR3	2.636E-169	-0.3501144	0.451	0.564	4.052E-165	FPR3
SLC46A1	3.328E-169	-0.690194	0.145	0.235	5.115E-165	SLC46A1
CERCAM	6.793E-167	-1.794146	0.019	0.065	1.044E-162	CERCAM
S100A8	6.239E-164	-0.2707459	0.866	0.909	9.59E-160	S100A8
CD55	2.054E-163	-0.199317	0.887	0.931	3.157E-159	CD55
TCN2	5.527E-162	-0.2870519	0.716	0.789	8.496E-158	TCN2
TMTC1	9.207E-161	-1.098355	0.065	0.131	1.415E-156	TMTC1
XBP1	1.044E-160	-0.2384241	0.778	0.843	1.605E-156	XBP1
LIPN	3.805E-160	-1.5818188	0.024	0.072	5.848E-156	LIPN
DUSP1	1.262E-159	-0.1875597	0.997	0.999	1.939E-155	DUSP1
SLC16A10	3.62E-158	-0.6411064	0.151	0.238	5.565E-154	SLC16A10
ARHGAP18	5.556E-155	-0.1852942	0.898	0.939	8.541E-151	ARHGAP18
GNS	4.063E-154	-0.1220124	0.995	0.997	6.246E-150	GNS
VGLL3	1.594E-153	-0.5323689	0.271	0.368	2.45E-149	VGLL3
ITGAX	4.558E-151	-0.2630434	0.801	0.855	7.006E-147	ITGAX
MTHFD1L	8.13E-150	-0.3244775	0.538	0.636	1.25E-145	MTHFD1L
GSTM1	6.21E-149	-1.6668687	0.019	0.061	9.546E-145	GSTM1
PARP4	7.678E-149	-0.1814075	0.939	0.958	1.18E-144	PARP4
MCOLN2	9.93E-147	-1.3519136	0.031	0.08	1.526E-142	MCOLN2
TTC7A	1.191E-145	-0.2038451	0.845	0.89	1.831E-141	TTC7A
RNF213	1.528E-145	-0.1905008	0.896	0.933	2.348E-141	RNF213
FOS	4.487E-145	-0.255065	0.973	0.979	6.897E-141	FOS
CXADR	6.096E-144	-1.1447354	0.042	0.096	9.37E-140	CXADR
SORT1	6.27E-143	-0.1581051	0.968	0.979	9.638E-139	SORT1
MAPRE3	1.097E-142	-0.4189208	0.387	0.485	1.686E-138	MAPRE3
SIRPB2	8.002E-142	-0.3144516	0.555	0.65	1.23E-137	SIRPB2
BTN3A2	1.547E-141	-0.3374985	0.468	0.571	2.378E-137	BTN3A2
THBS1	8.81E-141	-0.1020229	0.85	0.937	1.354E-136	THBS1
PPIC	8.167E-137	-0.2391991	0.726	0.795	1.255E-132	PPIC
MGAT4A	3.483E-134	-0.2482718	0.757	0.821	5.354E-130	MGAT4A
GAA	6.535E-133	-0.1047432	0.995	0.998	1.004E-128	GAA
HSD17B4	1.356E-132	-0.1633785	0.947	0.961	2.084E-128	HSD17B4
HIPK2	2.585E-130	-0.2062756	0.865	0.905	3.973E-126	HIPK2
SPIDR	1.392E-129	-0.3307236	0.491	0.584	2.14E-125	SPIDR
LILRA5	2.111E-128	-0.3022885	0.538	0.632	3.244E-124	LILRA5
PILRB	1.663E-127	-0.483556	0.294	0.386	2.556E-123	PILRB

EIF4G3	7.777E-127	-0.370989	0.393	0.489	1.195E-122	EIF4G3
CHDH	2.524E-126	-0.3056301	0.516	0.611	3.88E-122	CHDH
PPP1R14C	1.173E-125	-1.815503	0.015	0.051	1.804E-121	PPP1R14C
THBD	8.623E-125	-0.1975543	0.917	0.943	1.325E-120	THBD
ITGA3	3.05E-124	-0.7303079	0.122	0.192	4.688E-120	ITGA3
VEGFA	1.734E-122	-0.2483867	0.222	0.314	2.665E-118	VEGFA
MANBA	8.997E-122	-0.1892793	0.858	0.896	1.383E-117	MANBA
XAF1	1.299E-121	-0.2437029	0.634	0.721	1.997E-117	XAF1
SHMT1	2.227E-121	-0.6011835	0.154	0.23	3.423E-117	SHMT1
MAP3K6	1.291E-120	-0.8401495	0.07	0.128	1.984E-116	MAP3K6
SENP3	5.145E-118	-0.3293935	0.438	0.53	7.909E-114	SENP3
CD22	8.269E-117	-0.6751844	0.113	0.179	1.271E-112	CD22
SPARC	2.058E-116	-0.1721505	0.699	0.802	3.163E-112	SPARC
DPH3	2.385E-113	-0.2436361	0.673	0.745	3.666E-109	DPH3
FABP3	4.227E-113	-0.7738892	0.111	0.175	6.497E-109	FABP3
DDX60L	8.436E-113	-0.2984133	0.618	0.692	1.297E-108	DDX60L
TSPAN33	8.769E-113	-0.9783574	0.038	0.083	1.348E-108	TSPAN33
TSPAN15	1.562E-111	-0.2624746	0.553	0.641	2.401E-107	TSPAN15
UBE2D1	5.78E-110	-0.3025405	0.503	0.589	8.884E-106	UBE2D1
SYNC	1.056E-109	-0.3713778	0.32	0.41	1.623E-105	SYNC
SIGLEC7	2.405E-109	-0.3688684	0.328	0.417	3.697E-105	SIGLEC7
WLS	1.332E-108	-0.3459368	0.535	0.602	2.048E-104	WLS
GSN	1.866E-108	-0.1827333	0.977	0.98	2.869E-104	GSN
OSCAR	2.604E-106	-0.1463288	0.912	0.937	4.002E-102	OSCAR
FN1	3.053E-105	-0.4468298	0.822	0.833	4.693E-101	FN1
HM13	4.287E-105	-0.242421	0.669	0.735	6.589E-101	HM13
RBP4	6.665E-105	-0.1073501	0.752	0.841	1.025E-100	RBP4
SLC22A16	8.406E-105	-1.5583177	0.017	0.05	1.292E-100	SLC22A16
RND3	2.278E-103	-0.384635	0.312	0.396	3.501E-99	RND3
GNG12	1.241E-102	-0.3220747	0.404	0.492	1.907E-98	GNG12
GCHFR	1.401E-102	-0.1711746	0.953	0.97	2.1533E-98	GCHFR
MS4A14	2.116E-102	-0.3837024	0.366	0.447	3.2522E-98	MS4A14
KDM1B	3.63E-102	-0.2662768	0.578	0.656	5.5795E-98	KDM1B
CMIP	3.33E-101	-0.1625978	0.892	0.928	5.1192E-97	CMIP
VASH1	5.58E-101	-0.1834364	0.831	0.874	8.5764E-97	VASH1
LHFPL2	1.224E-99	-0.1913259	0.751	0.809	1.8818E-95	LHFPL2
PLPP1	1.299E-99	-0.3238905	0.391	0.477	1.9973E-95	PLPP1
SOCS3	1.518E-99	-0.215304	0.645	0.729	2.3333E-95	SOCS3
SPATA13	4.127E-99	-0.2623753	0.578	0.656	6.3435E-95	SPATA13
MLKL	4.72E-99	-0.2030865	0.732	0.791	7.2547E-95	MLKL
CYP3A5	5.053E-99	-1.527697	0.021	0.055	7.7665E-95	CYP3A5
CD300E	2.8763E-98	-0.7214564	0.072	0.124	4.4212E-94	CD300E
LSAMP	3.4722E-98	-0.2024448	0.746	0.811	5.3371E-94	LSAMP
NPC2	4.3596E-98	-0.1035906	0.996	0.998	6.7012E-94	NPC2
HAVCR2	1.6717E-96	-0.1731471	0.859	0.892	2.5695E-92	HAVCR2
TUBA1B	7.0806E-96	-0.1827019	0.786	0.88	1.0884E-91	TUBA1B
TMEM255A	1.1463E-94	-1.2172064	0.025	0.059	1.7619E-90	TMEM255A
TK1	1.6814E-94	-0.9843562	0.043	0.084	2.5845E-90	TK1
SERF2	2.1285E-94	-0.1502696	0.909	0.935	3.2717E-90	SERF2
ESYT1	9.0407E-94	-0.1107879	0.985	0.989	1.3896E-89	ESYT1
LAP3	3.4965E-93	-0.1326038	0.939	0.96	5.3745E-89	LAP3
PRKCE	1.9246E-92	-0.3668726	0.309	0.388	2.9583E-88	PRKCE

SRP9	3.2374E-92	-0.2828412	0.464	0.547	4.9762E-88	SRP9
SNTB1	6.6421E-91	-0.1773077	0.86	0.895	1.021E-86	SNTB1
HPGDS	2.8206E-90	-0.3068242	0.397	0.48	4.3356E-86	HPGDS
GSTM3	1.6446E-89	-1.1167683	0.025	0.058	2.5279E-85	GSTM3
CASP7	2.029E-89	-0.2205382	0.612	0.686	3.1188E-85	CASP7
HSPH1	6.8606E-88	-0.1797542	0.809	0.853	1.0545E-83	HSPH1
CDC42BPB	4.7081E-87	-0.1590502	0.87	0.903	7.2367E-83	CDC42BPB
AC067752.1	1.2647E-85	-0.5912887	0.1	0.154	1.944E-81	AC067752.1
UBASH3B	1.2675E-85	-0.169607	0.897	0.922	1.9483E-81	UBASH3B
GIMAP8	1.3936E-85	-0.4108379	0.184	0.253	2.142E-81	GIMAP8
CDH1	2.4848E-85	-1.2890256	0.033	0.068	3.8193E-81	CDH1
MLPH	3.4978E-85	-0.170076	0.891	0.92	5.3765E-81	MLPH
SIGLEC9	4.7982E-85	-0.2366216	0.552	0.628	7.3752E-81	SIGLEC9
PLXND1	1.1194E-84	-0.1365044	0.921	0.941	1.7207E-80	PLXND1
IDH2	2.2222E-84	-0.2352773	0.567	0.639	3.4158E-80	IDH2
ERAP2	2.35E-84	-0.256089	0.428	0.512	3.6122E-80	ERAP2
IFITM10	5.341E-84	-0.1719876	0.513	0.601	8.2096E-80	IFITM10
RSPO3	6.3837E-84	-0.4418915	0.261	0.331	9.8123E-80	RSPO3
VTN	1.884E-83	-0.3447018	0.333	0.41	2.8959E-79	VTN
ACAP2	6.6193E-83	-0.1938972	0.737	0.791	1.0174E-78	ACAP2
RASSF3	1.0645E-82	-0.1796035	0.786	0.835	1.6362E-78	RASSF3
SLC2A3	1.5143E-82	-0.1742237	0.708	0.769	2.3276E-78	SLC2A3
PEPD	1.5763E-81	-0.1423094	0.933	0.948	2.423E-77	PEPD
MX1	1.6091E-81	-0.1616977	0.825	0.874	2.4734E-77	MX1
NLRP2	3.2984E-81	-0.3831144	0.191	0.259	5.07E-77	NLRP2
TCEA3	3.9173E-81	-0.2284242	0.489	0.571	6.0213E-77	TCEA3
ACADSB	6.152E-81	-0.3777139	0.264	0.335	9.4562E-77	ACADSB
TNS1	1.641E-80	-0.5257774	0.14	0.198	2.5223E-76	TNS1
CAMK1D	1.8295E-80	-0.2686807	0.449	0.526	2.8122E-76	CAMK1D
ACER3	5.8706E-79	-0.1886125	0.712	0.769	9.0237E-75	ACER3
P4HA1	8.0083E-79	-0.1837733	0.75	0.806	1.231E-74	P4HA1
PRKACB	1.035E-78	-0.2266005	0.598	0.667	1.5909E-74	PRKACB
SAMD9L	1.1753E-78	-0.1364567	0.891	0.922	1.8065E-74	SAMD9L
CDC42EP1	1.6391E-78	-0.5530879	0.077	0.125	2.5194E-74	CDC42EP1
HAGHL	1.6832E-78	-0.5144692	0.132	0.188	2.5873E-74	HAGHL
CYFIP2	4.3168E-78	-0.3462787	0.276	0.349	6.6354E-74	CYFIP2
MGAT5	6.0216E-78	-0.1960953	0.713	0.77	9.2559E-74	MGAT5
CNPY3	7.9731E-78	-0.1186027	0.943	0.957	1.2256E-73	CNPY3
WARS	8.5357E-78	-0.1217246	0.939	0.952	1.312E-73	WARS
COL15A1	1.0201E-76	-1.861485	0.007	0.027	1.5679E-72	COL15A1
REEP3	1.0409E-76	-0.1459445	0.893	0.919	1.5999E-72	REEP3
PITPNA	1.2452E-76	-0.1471443	0.856	0.888	1.914E-72	PITPNA
LMBR1	4.6299E-76	-0.2586602	0.483	0.559	7.1166E-72	LMBR1
IFIT1	2.0665E-75	-0.1944877	0.586	0.662	3.1765E-71	IFIT1
IL3RA	2.3805E-75	-0.3628515	0.703	0.72	3.659E-71	IL3RA
SNX7	3.8095E-75	-0.4669824	0.123	0.178	5.8555E-71	SNX7
SLC16A6	4.0893E-75	-0.3968107	0.196	0.26	6.2856E-71	SLC16A6
AGT	4.4586E-75	-4.2763928	0	0.014	6.8533E-71	AGT
HSP90AA1	5.1707E-75	-0.1047364	0.975	0.982	7.9478E-71	HSP90AA1
HSPA1B	1.8348E-74	-0.2573331	0.68	0.741	2.8203E-70	HSPA1B
BAK1	5.8851E-74	-0.2115753	0.599	0.667	9.046E-70	BAK1
SLC35F6	6.4442E-74	-0.1415726	0.876	0.9	9.9054E-70	SLC35F6

FCHO2	2.1163E-73	-0.1969729	0.667	0.727	3.2529E-69	FCHO2
DNASE2	2.3216E-73	-0.1190847	0.971	0.976	3.5685E-69	DNASE2
USF2	3.2243E-73	-0.1249125	0.937	0.957	4.9561E-69	USF2
STAT2	5.8538E-73	-0.1552036	0.827	0.866	8.9979E-69	STAT2
PAG1	1.2842E-72	-0.1874871	0.804	0.846	1.9739E-68	PAG1
CBX6	2.4818E-72	-0.3523365	0.215	0.281	3.8147E-68	CBX6
ZMPSTE24	1.0234E-70	-0.1742372	0.734	0.786	1.5731E-66	ZMPSTE24
PTPN12	1.1262E-70	-0.1202937	0.944	0.963	1.7311E-66	PTPN12
TUBB2A	5.0779E-70	-0.2311299	0.444	0.52	7.8052E-66	TUBB2A
SELENOM	6.965E-70	-0.289194	0.365	0.437	1.0706E-65	SELENOM
GM2A	8.2482E-70	-0.1229071	0.95	0.961	1.2678E-65	GM2A
LRP5	2.8672E-69	-3.3781406	0.002	0.015	4.4072E-65	LRP5
GPBAR1	4.521E-69	-0.627455	0.071	0.112	6.9492E-65	GPBAR1
SPCS1	6.2816E-69	-0.1299081	0.879	0.909	9.6555E-65	SPCS1
ANKH	1.0458E-68	-0.5641707	0.09	0.137	1.6075E-64	ANKH
ITGB7	1.8906E-68	-0.3295873	0.295	0.361	2.906E-64	ITGB7
CLSTN1	2.7166E-68	-0.2459739	0.472	0.541	4.1757E-64	CLSTN1
SWAP70	3.0563E-68	-0.2035056	0.62	0.681	4.6978E-64	SWAP70
SLC22A18	7.4722E-68	-0.2379471	0.41	0.486	1.1485E-63	SLC22A18
CD300A	2.6012E-67	-0.22946	0.343	0.416	3.9983E-63	CD300A
SIGLEC1	5.0557E-67	-0.1226688	0.795	0.86	7.7711E-63	SIGLEC1
WSB2	8.5895E-67	-0.1490777	0.822	0.864	1.3203E-62	WSB2
HSD17B14	9.2507E-67	-0.2451818	0.511	0.577	1.4219E-62	HSD17B14
SLC4A8	9.6777E-67	-0.277308	0.34	0.41	1.4876E-62	SLC4A8
TNS3	1.7364E-66	-0.1402452	0.825	0.868	2.669E-62	TNS3
TGFBR1	2.0842E-66	-0.1958399	0.661	0.722	3.2036E-62	TGFBR1
FARP1	3.2886E-66	-0.2674147	0.318	0.389	5.055E-62	FARP1
KIDINS220	4.6123E-66	-0.2106181	0.539	0.607	7.0895E-62	KIDINS220
C1GALT1C1	5.2822E-66	-0.2206621	0.491	0.564	8.1193E-62	C1GALT1C1
CIITA	7.6841E-66	-0.146772	0.814	0.852	1.1811E-61	CIITA
DNAJA1	1.6086E-65	-0.1196698	0.922	0.947	2.4726E-61	DNAJA1
PRRT4	1.722E-65	-0.6931589	0.063	0.102	2.6469E-61	PRRT4
SEPTIN3	1.7619E-65	-0.42714	0.135	0.187	2.7082E-61	SEPTIN3
IFIT3	1.7801E-65	-0.1282243	0.906	0.933	2.7362E-61	IFIT3
GPR65	2.0273E-65	-0.2341713	0.543	0.612	3.1162E-61	GPR65
TNFSF10	2.3004E-65	-0.2349339	0.485	0.554	3.5359E-61	TNFSF10
ACOT7	2.6944E-65	-0.2430197	0.64	0.687	4.1416E-61	ACOT7
HACD4	4.4529E-65	-0.1808365	0.718	0.772	6.8445E-61	HACD4
PRR5L	5.8478E-65	-0.5915826	0.07	0.111	8.9887E-61	PRR5L
CHPT1	2.2424E-64	-0.2625426	0.449	0.516	3.4469E-60	CHPT1
WDFY4	2.4217E-64	-0.1679837	0.721	0.771	3.7224E-60	WDFY4
ADTRP	3.4774E-64	-0.1435044	0.824	0.865	5.3452E-60	ADTRP
EIF2AK2	5.0113E-64	-0.157031	0.808	0.847	7.7029E-60	EIF2AK2
EPB41L4A	1.6628E-63	-0.2434438	0.417	0.488	2.556E-59	EPB41L4A
HEG1	2.1897E-63	-0.3696228	0.188	0.246	3.3658E-59	HEG1
LIMS2	3.9261E-63	-0.4322723	0.239	0.295	6.0347E-59	LIMS2
SYTL4	4.144E-63	-0.4272441	0.12	0.169	6.3698E-59	SYTL4
FCRLB	8.8983E-63	-1.9452252	0.011	0.031	1.3678E-58	FCRLB
PHF10	1.2032E-62	-0.2380155	0.418	0.489	1.8494E-58	PHF10
HOMER2	1.3503E-62	-0.4626545	0.107	0.153	2.0756E-58	HOMER2
DDTL	2.3681E-62	-1.4241523	0.012	0.033	3.64E-58	DDTL
NLRP12	2.4943E-62	-0.4433764	0.132	0.183	3.834E-58	NLRP12

CEACAM1	3.3462E-62	-0.5734747	0.092	0.135	5.1434E-58	CEACAM1
NAGLU	6.1668E-62	-0.1837088	0.596	0.658	9.479E-58	NAGLU
GRIN2D	8.0233E-62	-0.6997993	0.05	0.084	1.2333E-57	GRIN2D
ACSS3	8.6627E-62	-1.0704706	0.023	0.048	1.3315E-57	ACSS3
SLC22A15	9.5703E-62	-0.3056644	0.276	0.341	1.4711E-57	SLC22A15
ACACB	1.1434E-61	-0.3504801	0.258	0.318	1.7575E-57	ACACB
STON1	3.6148E-61	-0.1761283	0.479	0.557	5.5562E-57	STON1
CHMP4C	4.5465E-61	-0.5628687	0.074	0.114	6.9885E-57	CHMP4C
TMEFF1	5.4663E-61	-0.2420071	0.29	0.358	8.4022E-57	TMEFF1
CFDP1	9.6008E-61	-0.1682435	0.682	0.736	1.4757E-56	CFDP1
AXL	1.3122E-60	-0.1011559	0.939	0.961	2.017E-56	AXL
ICMT	1.6617E-60	-0.1891454	0.619	0.681	2.5542E-56	ICMT
CYBB	6.519E-60	-0.1146902	0.987	0.991	1.002E-55	CYBB
C9orf72	1.4913E-59	-0.1764853	0.583	0.653	2.2922E-55	C9orf72
AACS	2.0397E-59	-0.2690235	0.292	0.358	3.1352E-55	AACS
TTL	6.2655E-59	-0.1837174	0.615	0.677	9.6307E-55	TTL
RAB11FIP5	6.3598E-59	-0.5207587	0.083	0.124	9.7757E-55	RAB11FIP5
UBXN11	7.4474E-59	-0.1395627	0.78	0.825	1.1447E-54	UBXN11
OSM	1.0611E-58	-0.2125512	0.319	0.388	1.631E-54	OSM
ATP6V1B2	1.9799E-58	-0.1023291	0.965	0.975	3.0434E-54	ATP6V1B2
KLKB1	3.0696E-58	-1.2179275	0.015	0.036	4.7183E-54	KLKB1
PTPN22	7.8399E-58	-0.1997851	0.541	0.607	1.2051E-53	PTPN22
NFIL3	8.1224E-58	-0.1089819	0.745	0.8	1.2485E-53	NFIL3
ENOSF1	1.3702E-57	-0.2233288	0.477	0.539	2.1061E-53	ENOSF1
CNNM4	9.466E-57	-0.2742055	0.259	0.321	1.455E-52	CNNM4
CTSK	1.8879E-56	-0.7672722	0.05	0.082	2.9019E-52	CTSK
PLCB2	3.2472E-56	-0.1194525	0.841	0.883	4.9913E-52	PLCB2
DNASE2B	6.227E-56	-0.3066353	0.195	0.25	9.5715E-52	DNASE2B
TMEM74B	4.5905E-55	-0.2044702	0.513	0.578	7.056E-51	TMEM74B
RGS10	1.7979E-54	-0.1958398	0.52	0.584	2.7636E-50	RGS10
TIMM10	2.7009E-54	-0.1605012	0.732	0.78	4.1516E-50	TIMM10
SLC11A2	2.8789E-54	-0.1696987	0.626	0.687	4.4252E-50	SLC11A2
DDX3X	5.7809E-54	-0.2107924	0.419	0.486	8.8858E-50	DDX3X
DCTN5	1.7863E-53	-0.3607083	0.155	0.205	2.7457E-49	DCTN5
RAB27A	1.8311E-53	-0.1989886	0.483	0.547	2.8145E-49	RAB27A
MSLN	1.9567E-53	-4.4420905	0	0.01	3.0077E-49	MSLN
SLC8A1	2.1352E-53	-0.1470321	0.875	0.902	3.282E-49	SLC8A1
TMCC3	2.9277E-53	-0.4646266	0.101	0.142	4.5001E-49	TMCC3
TRIM14	8.8369E-53	-0.1116887	0.92	0.938	1.3583E-48	TRIM14
SERPINI1	1.0095E-52	-0.5996748	0.063	0.097	1.5517E-48	SERPINI1
YES1	1.0486E-52	-0.3567918	0.168	0.219	1.6119E-48	YES1
CRIM1	1.4039E-52	-0.242025	0.358	0.42	2.158E-48	CRIM1
RDH10	1.799E-52	-0.1638311	0.622	0.68	2.7653E-48	RDH10
ZNF589	2.0702E-52	-0.1198323	0.499	0.567	3.1821E-48	ZNF589
ABHD12	5.7389E-52	-0.1379725	0.83	0.861	8.8213E-48	ABHD12
CLEC4M	6.3823E-52	-1.9335196	0.004	0.017	9.8102E-48	CLEC4M
ARHGAP26	1.0693E-51	-0.2295792	0.365	0.429	1.6436E-47	ARHGAP26
TPMT	1.9808E-51	-0.2148637	0.432	0.496	3.0446E-47	TPMT
KIF1B	5.4405E-51	-0.216166	0.408	0.473	8.3625E-47	KIF1B
C17orf97	5.72E-51	-0.6116854	0.063	0.096	8.7923E-47	C17orf97
SESN3	7.3632E-51	-0.3091441	0.232	0.288	1.1318E-46	SESN3
DHX58	1.0015E-50	-0.1807916	0.461	0.53	1.5394E-46	DHX58

SGK3	1.0148E-50	-0.1364936	0.781	0.824	1.5598E-46	SGK3
SH2D4B	1.6377E-50	-0.6189528	0.058	0.09	2.5173E-46	SH2D4B
UPP1	2.3851E-50	-0.1040566	0.911	0.935	3.6661E-46	UPP1
SLC17A9	4.8976E-50	-0.4449722	0.104	0.144	7.528E-46	SLC17A9
MARK3	5.3976E-50	-0.21793	0.448	0.511	8.2966E-46	MARK3
SEPTIN4	6.0506E-50	-0.2264687	0.413	0.475	9.3004E-46	SEPTIN4
KIAA1522	7.8758E-50	-0.2135677	0.357	0.421	1.2106E-45	KIAA1522
FGFR1OP	8.8867E-50	-0.3538507	0.165	0.214	1.366E-45	FGFR1OP
TM2D2	2.6047E-49	-0.1991066	0.449	0.513	4.0037E-45	TM2D2
SETD7	4.1203E-49	-0.1724929	0.521	0.583	6.3333E-45	SETD7
AMD1	4.4393E-49	-0.2401979	0.395	0.455	6.8236E-45	AMD1
ANXA3	6.5283E-49	-0.7281294	0.034	0.06	1.0035E-44	ANXA3
TMEM9	8.2225E-49	-0.2846266	0.255	0.31	1.2639E-44	TMEM9
LILRA6	1.0201E-48	-0.3418276	0.217	0.269	1.5679E-44	LILRA6
SUGP2	1.6285E-48	-0.2105609	0.398	0.46	2.5032E-44	SUGP2
TRIO	2.3024E-48	-0.1208155	0.804	0.845	3.539E-44	TRIO
DMAC1	2.9716E-48	-0.1509821	0.726	0.775	4.5676E-44	DMAC1
AGRN	3.5366E-48	-0.1915833	0.543	0.598	5.4361E-44	AGRN
P4HA2	3.9984E-48	-0.202385	0.38	0.443	6.1459E-44	P4HA2
KDM6A	4.326E-48	-0.2599357	0.293	0.351	6.6494E-44	KDM6A
SIDT2	5.1929E-48	-0.1388124	0.779	0.821	7.982E-44	SIDT2
PCNX2	5.7947E-48	-0.550656	0.07	0.104	8.9071E-44	PCNX2
UBL3	7.0499E-48	-0.1349722	0.803	0.841	1.0836E-43	UBL3
PROCR	7.082E-48	-0.1326619	0.461	0.531	1.0886E-43	PROCR
RFTN1	9.0979E-48	-0.2951704	0.177	0.227	1.3984E-43	RFTN1
LONRF3	1.3611E-47	-0.1490924	0.683	0.736	2.0922E-43	LONRF3
SYT11	1.3895E-47	-0.2463206	0.265	0.322	2.1358E-43	SYT11
ZBTB33	1.8969E-47	-0.1874244	0.587	0.645	2.9157E-43	ZBTB33
FOLR1	2.1034E-47	-0.2984814	0.18	0.23	3.2331E-43	FOLR1
RMDN3	2.2529E-47	-0.1055658	0.916	0.933	3.463E-43	RMDN3
HSP90B1	2.2874E-47	-0.1193442	0.845	0.873	3.516E-43	HSP90B1
STEAP3	2.7467E-47	-0.1690891	0.58	0.641	4.222E-43	STEAP3
MIF	4.297E-47	-0.1278511	0.803	0.839	6.605E-43	MIF
SLC16A1	4.4513E-47	-0.2561168	0.218	0.272	6.842E-43	SLC16A1
SHTN1	4.5605E-47	-0.1155893	0.854	0.88	7.01E-43	SHTN1
ME3	4.7795E-47	-0.2591592	0.222	0.276	7.3465E-43	ME3
ADGRA2	5.0613E-47	-0.5391375	0.087	0.123	7.7797E-43	ADGRA2
SH3BP2	5.0936E-47	-0.1547347	0.639	0.691	7.8293E-43	SH3BP2
FAM49A	5.2278E-47	-0.1991929	0.477	0.541	8.0356E-43	FAM49A
MT2A	6.3078E-47	-0.3056839	0.421	0.474	9.6957E-43	MT2A
KDM5C	7.5573E-47	-0.148797	0.622	0.68	1.1616E-42	KDM5C
RHOF	1.8886E-46	-0.4831733	0.075	0.11	2.9029E-42	RHOF
SMC1A	2.1112E-46	-0.1313861	0.788	0.83	3.2451E-42	SMC1A
MTLN	3.8052E-46	-0.1350836	0.623	0.685	5.8489E-42	MTLN
METRN	5.6429E-46	-0.3769656	0.129	0.17	8.6737E-42	METRN
FGD5	9.9029E-46	-0.1091387	0.843	0.875	1.5222E-41	FGD5
MCU	2.0153E-45	-0.2962405	0.23	0.282	3.0978E-41	MCU
CLEC1A	2.2629E-45	-0.5526976	0.06	0.091	3.4783E-41	CLEC1A
PTEN	2.9691E-45	-0.2156675	0.366	0.427	4.5638E-41	PTEN
PLXNA2	3.2278E-45	-0.3777172	0.134	0.176	4.9614E-41	PLXNA2
ICA1	3.7138E-45	-0.502358	0.07	0.102	5.7084E-41	ICA1
FGD4	3.8469E-45	-0.1568077	0.611	0.668	5.9131E-41	FGD4

PDCD1LG2	5.4078E-45	-0.1459756	0.553	0.615	8.3123E-41	PDCD1LG2
TMEM184B	9.0175E-45	-0.1076938	0.859	0.887	1.3861E-40	TMEM184B
TNFRSF10A	1.0022E-44	-0.4262082	0.098	0.135	1.5405E-40	TNFRSF10A
NUDT3	1.8648E-44	-0.1458626	0.753	0.791	2.8665E-40	NUDT3
COQ2	2.6461E-44	-0.1539636	0.605	0.663	4.0673E-40	COQ2
MGST1	3.7162E-44	-0.2568873	0.503	0.555	5.7121E-40	MGST1
LY96	4.0118E-44	-0.1344657	0.738	0.788	6.1666E-40	LY96
EPB41L1	8.0102E-44	-0.1238565	0.786	0.825	1.2312E-39	EPB41L1
ARSB	1.1433E-43	-0.1520142	0.66	0.711	1.7574E-39	ARSB
MCUB	1.2364E-43	-0.1926953	0.433	0.493	1.9005E-39	MCUB
ZFX	1.4192E-43	-0.2959429	0.184	0.232	2.1814E-39	ZFX
FRMPD1	1.507E-43	-0.4195554	0.08	0.114	2.3164E-39	FRMPD1
CD109	2.1068E-43	-0.1777259	0.47	0.531	3.2383E-39	CD109
B3GNT5	3.5188E-43	-0.1576327	0.687	0.734	5.4087E-39	B3GNT5
HOXB2	4.2087E-43	-0.2633573	0.199	0.249	6.4692E-39	HOXB2
RRAGD	5.1347E-43	-0.1345488	0.749	0.79	7.8926E-39	RRAGD
HEXIM1	5.6399E-43	-0.1459179	0.667	0.715	8.669E-39	HEXIM1
B3GALNT2	5.9453E-43	-0.2883944	0.177	0.224	9.1385E-39	B3GALNT2
TOR1B	6.7428E-43	-0.1313934	0.715	0.761	1.0364E-38	TOR1B
PHF6	1.6338E-42	-0.2218424	0.286	0.342	2.5113E-38	PHF6
PSPH	1.6564E-42	-0.324564	0.194	0.24	2.5461E-38	PSPH
CSF3R	1.9366E-42	-0.213692	0.409	0.465	2.9768E-38	CSF3R
BLOC1S2	3.0607E-42	-0.1366617	0.787	0.825	4.7046E-38	BLOC1S2
KL	3.9003E-42	-0.4056366	0.102	0.139	5.9951E-38	KL
GLUD1	4.5996E-42	-0.1405493	0.679	0.729	7.07E-38	GLUD1
ATP2B4	6.0655E-42	-0.2161465	0.247	0.301	9.3233E-38	ATP2B4
TMEM123	1.4225E-41	-0.1862079	0.603	0.654	2.1865E-37	TMEM123
TTLL5	2.3029E-41	-0.1978931	0.354	0.413	3.5399E-37	TTLL5
DIPK1A	3.1479E-41	-0.4620077	0.076	0.108	4.8386E-37	DIPK1A
TSEN2	3.9936E-41	-0.2533573	0.3	0.353	6.1386E-37	TSEN2
ZDHHC19	4.5174E-41	-0.7749126	0.028	0.049	6.9436E-37	ZDHHC19
LRRC8D	4.9189E-41	-0.1472256	0.608	0.667	7.5608E-37	LRRC8D
QSER1	6.2407E-41	-0.1596983	0.575	0.631	9.5926E-37	QSER1
ADAMTS1	6.6176E-41	-1.0918441	0.027	0.047	1.0172E-36	ADAMTS1
ZNF518A	7.239E-41	-0.1982124	0.378	0.436	1.1127E-36	ZNF518A
WIPI1	1.534E-40	-0.2232084	0.28	0.333	2.3579E-36	WIPI1
ABCA1	2.0386E-40	-0.1170452	0.352	0.411	3.1335E-36	ABCA1
GPR141	2.0696E-40	-1.4735947	0.006	0.018	3.1812E-36	GPR141
TSPAN14	2.3087E-40	-0.1066879	0.855	0.884	3.5487E-36	TSPAN14
HERC4	3.133E-40	-0.1796983	0.482	0.539	4.8157E-36	HERC4
SAMD12	3.4014E-40	-0.595657	0.039	0.064	5.2283E-36	SAMD12
VPS37C	5.5713E-40	-0.1695421	0.433	0.492	8.5637E-36	VPS37C
IL13RA1	7.3236E-40	-0.1180303	0.879	0.906	1.1257E-35	IL13RA1
DTWD1	1.2771E-39	-0.3733866	0.119	0.157	1.963E-35	DTWD1
HEATR3	1.37E-39	-0.1637063	0.514	0.572	2.1058E-35	HEATR3
LIN7A	1.4867E-39	-0.2339293	0.276	0.328	2.2852E-35	LIN7A
SNCA	1.5967E-39	-0.7326679	0.035	0.057	2.4543E-35	SNCA
HACD2	2.3778E-39	-0.2041531	0.306	0.361	3.655E-35	HACD2
CYSLTR1	3.7761E-39	-0.1805938	0.54	0.595	5.8042E-35	CYSLTR1
ZC3HAV1	6.624E-39	-0.1107948	0.671	0.724	1.0182E-34	ZC3HAV1
COPZ2	7.1636E-39	-0.5381976	0.049	0.075	1.1011E-34	COPZ2
MCM4	8.7408E-39	-0.1895398	0.363	0.421	1.3435E-34	MCM4

PNPLA4	1.1998E-38	-0.2814174	0.188	0.233	1.8442E-34	PNPLA4
CLDN7	1.4722E-38	-0.1297718	0.658	0.705	2.2629E-34	CLDN7
TJP1	1.5835E-38	-0.3304019	0.126	0.164	2.434E-34	TJP1
DTNA	1.7357E-38	-0.2092958	0.32	0.374	2.668E-34	DTNA
SLC7A6	1.8026E-38	-0.2098796	0.247	0.299	2.7708E-34	SLC7A6
TSKU	2.1756E-38	-0.3158618	0.162	0.203	3.3441E-34	TSKU
TULP4	2.2903E-38	-0.1508664	0.616	0.669	3.5204E-34	TULP4
IGFLR1	2.7739E-38	-0.1746794	0.457	0.517	4.2637E-34	IGFLR1
CALD1	3.0005E-38	-1.0363004	0.014	0.03	4.6121E-34	CALD1
GPR157	3.4169E-38	-0.2035186	0.362	0.419	5.2521E-34	GPR157
RMI2	3.7384E-38	-0.57433	0.034	0.056	5.7463E-34	RMI2
ZYG11B	4.2651E-38	-0.1116259	0.8	0.835	6.5558E-34	ZYG11B
TEP1	4.6925E-38	-0.1826755	0.354	0.411	7.2129E-34	TEP1
CD72	7.5023E-38	-0.3698	0.112	0.148	1.1532E-33	CD72
LILRB5	1.2665E-37	-1.0780685	0.015	0.03	1.9467E-33	LILRB5
HSDL2	1.4557E-37	-0.1222491	0.715	0.762	2.2376E-33	HSDL2
DENND4C	1.8206E-37	-0.1007676	0.859	0.889	2.7985E-33	DENND4C
ALDH18A1	1.9297E-37	-0.3212958	0.157	0.197	2.9662E-33	ALDH18A1
KDM2B	1.9757E-37	-0.1785084	0.367	0.425	3.0368E-33	KDM2B
TTC23	2.1574E-37	-0.2558911	0.181	0.226	3.3161E-33	TTC23
CAP1	2.2214E-37	-0.1498701	0.582	0.636	3.4145E-33	CAP1
KSR1	2.265E-37	-0.1328159	0.719	0.764	3.4815E-33	KSR1
NUP58	2.3058E-37	-0.147643	0.626	0.679	3.5442E-33	NUP58
INTS10	2.4558E-37	-0.172773	0.476	0.53	3.7749E-33	INTS10
TUBB8	2.6686E-37	-1.5419537	0.005	0.015	4.1019E-33	TUBB8
GTSF1	2.9643E-37	-0.3871658	0.11	0.145	4.5564E-33	GTSF1
CST2	3.5317E-37	-2.2459131	0.003	0.012	5.4286E-33	CST2
ANKRD52	5.3835E-37	-0.200826	0.321	0.374	8.275E-33	ANKRD52
UGDH	5.9721E-37	-0.1856469	0.413	0.468	9.1798E-33	UGDH
PLA2G6	6.5843E-37	-0.2565634	0.217	0.264	1.0121E-32	PLA2G6
MPC2	9.3925E-37	-0.1493482	0.578	0.634	1.4437E-32	MPC2
PBX3	1.0338E-36	-0.1323802	0.623	0.676	1.589E-32	PBX3
CHI3L2	1.3069E-36	-0.5198058	0.093	0.124	2.0089E-32	CHI3L2
PDE1B	1.769E-36	-0.1524634	0.718	0.757	2.7191E-32	PDE1B
SUSD1	1.8088E-36	-0.1162699	0.749	0.792	2.7804E-32	SUSD1
ENPP2	1.9749E-36	-0.8609908	0.029	0.05	3.0356E-32	ENPP2
CTNNBL1	2.1728E-36	-0.1470329	0.603	0.654	3.3398E-32	CTNNBL1
NLRP1	3.1345E-36	-0.1549556	0.629	0.674	4.8181E-32	NLRP1
CXXC5	3.2435E-36	-0.1578054	0.406	0.463	4.9856E-32	CXXC5
B4GALT6	3.6732E-36	-0.412981	0.084	0.115	5.6461E-32	B4GALT6
AP1B1	3.6841E-36	-0.1135261	0.841	0.866	5.6628E-32	AP1B1
CAMP	3.7514E-36	-0.2081716	0.384	0.439	5.7663E-32	CAMP
SMPD1	4.1475E-36	-0.1967315	0.326	0.378	6.3751E-32	SMPD1
NNT	5.5511E-36	-0.1747276	0.464	0.519	8.5325E-32	NNT
PIEZO1	6.4271E-36	-0.1808164	0.486	0.537	9.8791E-32	PIEZO1
TMEM51	7.3792E-36	-0.2237029	0.255	0.304	1.1343E-31	TMEM51
IFI44L	8.8088E-36	-0.1999823	0.408	0.46	1.354E-31	IFI44L
RAB30	1.0974E-35	-0.5977087	0.035	0.057	1.6868E-31	RAB30
CYSTM1	1.2698E-35	-0.1681306	0.482	0.534	1.9518E-31	CYSTM1
SLC17A5	3.2299E-35	-0.1420953	0.63	0.679	4.9647E-31	SLC17A5
SYNGAP1	6.1382E-35	-0.3319126	0.106	0.14	9.435E-31	SYNGAP1
HBS1L	6.451E-35	-0.1193756	0.702	0.747	9.9159E-31	HBS1L

ECHS1	8.1256E-35	-0.1009613	0.837	0.867	1.249E-30	ECHS1
CTDSP2	1.1266E-34	-0.1169657	0.764	0.807	1.7318E-30	CTDSP2
CISD1	1.1723E-34	-0.2641061	0.165	0.206	1.802E-30	CISD1
SKAP1	1.3319E-34	-0.3625162	0.084	0.115	2.0473E-30	SKAP1
MTHFD1	1.6009E-34	-0.110313	0.783	0.822	2.4608E-30	MTHFD1
ATP6V1D	1.7665E-34	-0.1275987	0.669	0.717	2.7153E-30	ATP6V1D
RNASE1	1.8202E-34	-1.3619066	0.021	0.038	2.7979E-30	RNASE1
ROBO1	2.1461E-34	-0.7081391	0.023	0.04	3.2988E-30	ROBO1
MTMR4	2.1848E-34	-0.1892741	0.361	0.414	3.3582E-30	MTMR4
HTT	3.134E-34	-0.1085356	0.743	0.782	4.8173E-30	HTT
MACF1	3.4256E-34	-0.1400317	0.559	0.614	5.2654E-30	MACF1
SLC39A10	3.6169E-34	-0.1166567	0.695	0.745	5.5596E-30	SLC39A10
APIP	3.747E-34	-0.1046992	0.756	0.804	5.7595E-30	APIP
EIF5	3.9406E-34	-0.1055545	0.81	0.841	6.0571E-30	EIF5
TLK2	4.0307E-34	-0.2052802	0.265	0.314	6.1956E-30	TLK2
SAP25	7.5271E-34	-0.4584677	0.06	0.087	1.157E-29	SAP25
FAM111A	7.8777E-34	-0.1424292	0.5	0.557	1.2109E-29	FAM111A
KCTD17	8.0021E-34	-0.1562665	0.576	0.622	1.23E-29	KCTD17
TMEM38B	8.2266E-34	-0.1569737	0.507	0.562	1.2645E-29	TMEM38B
DNAJB9	8.697E-34	-0.1534238	0.459	0.513	1.3368E-29	DNAJB9
PIK3R1	8.7721E-34	-0.148444	0.601	0.65	1.3484E-29	PIK3R1
C20orf27	1.085E-33	-0.2434903	0.22	0.265	1.6678E-29	C20orf27
OGFRL1	1.1404E-33	-0.1125239	0.716	0.763	1.7529E-29	OGFRL1
JAKMIP2	1.5599E-33	-0.110106	0.452	0.511	2.3977E-29	JAKMIP2
GFPT1	2.2056E-33	-0.1875336	0.334	0.385	3.3903E-29	GFPT1
STS	2.8061E-33	-0.2104845	0.342	0.392	4.3133E-29	STS
ERI1	3.3374E-33	-0.1421963	0.572	0.619	5.13E-29	ERI1
EXOC6	5.9382E-33	-0.1823242	0.383	0.435	9.1276E-29	EXOC6
TPD52L1	1.3469E-32	-0.3235713	0.152	0.188	2.0703E-28	TPD52L1
GPR153	1.3821E-32	-0.1822925	0.309	0.359	2.1244E-28	GPR153
PHTF2	1.788E-32	-0.1172795	0.599	0.651	2.7483E-28	PHTF2
TMEM50B	1.8714E-32	-0.1978313	0.301	0.35	2.8765E-28	TMEM50B
SLC24A4	2.0847E-32	-0.3116572	0.159	0.196	3.2045E-28	SLC24A4
GFOD1	2.3226E-32	-0.2814986	0.133	0.169	3.57E-28	GFOD1
PVALB	3.6928E-32	-0.2945089	0.129	0.164	5.6763E-28	PVALB
COL8A2	3.9729E-32	-0.1459021	0.497	0.549	6.1067E-28	COL8A2
SLC38A7	4.4624E-32	-0.1499944	0.371	0.425	6.8592E-28	SLC38A7
CKAP5	4.4736E-32	-0.1437942	0.502	0.559	6.8764E-28	CKAP5
SEC24D	4.5129E-32	-0.1688561	0.416	0.47	6.9368E-28	SEC24D
GPATCH11	4.7897E-32	-0.175685	0.376	0.426	7.3622E-28	GPATCH11
SMC6	9.05E-32	-0.1484317	0.51	0.562	1.3911E-27	SMC6
TPPP	1.089E-31	-0.1458381	0.212	0.257	1.6739E-27	TPPP
NSF	1.6514E-31	-0.1187489	0.66	0.706	2.5384E-27	NSF
PALM2-AKAP2	1.6685E-31	-0.4584774	0.066	0.092	2.5646E-27	PALM2-AKAP2
DBP	2.9008E-31	-0.2202772	0.332	0.377	4.4589E-27	DBP
ZNF106	3.1185E-31	-0.1382045	0.646	0.688	4.7934E-27	ZNF106
UBXN10	4.4786E-31	-0.2993769	0.152	0.189	6.8841E-27	UBXN10
SYCE1	4.8195E-31	-1.2955587	0.006	0.016	7.4081E-27	SYCE1
CD2	7.0692E-31	-0.4048729	0.102	0.133	1.0866E-26	CD2
FOLR2	7.1204E-31	-0.3175841	0.125	0.158	1.0945E-26	FOLR2
CCDC50	9.1899E-31	-0.1112178	0.708	0.756	1.4126E-26	CCDC50
RCN3	9.291E-31	-0.1924044	0.156	0.194	1.4281E-26	RCN3

ANGPTL6	9.425E-31	-1.1215608	0.01	0.022	1.4487E-26	ANGPTL6
RILPL1	1.0145E-30	-0.295296	0.121	0.154	1.5594E-26	RILPL1
PNMA1	1.0985E-30	-0.1332505	0.574	0.624	1.6885E-26	PNMA1
PLA1A	1.1343E-30	-0.6131081	0.034	0.053	1.7435E-26	PLA1A
ADORA1	1.2571E-30	-0.1580413	0.286	0.336	1.9323E-26	ADORA1
CCDC69	1.4175E-30	-0.1770019	0.283	0.331	2.1788E-26	CCDC69
TMEM163	1.4302E-30	-1.9460586	0.003	0.01	2.1983E-26	TMEM163
DSTN	1.4656E-30	-0.1076348	0.734	0.777	2.2527E-26	DSTN
CDR2	2.544E-30	-0.2168146	0.223	0.267	3.9104E-26	CDR2
ATP6V0A1	2.6052E-30	-0.1508444	0.52	0.57	4.0044E-26	ATP6V0A1
C2orf91	3.1792E-30	-0.575034	0.035	0.055	4.8868E-26	C2orf91
PFKFB2	4.4739E-30	-0.2022777	0.277	0.323	6.8768E-26	PFKFB2
CC2D2A	6.1335E-30	-0.1789839	0.272	0.319	9.4278E-26	CC2D2A
SPESP1	7.9152E-30	-0.4846763	0.03	0.049	1.2167E-25	SPESP1
SYTL1	8.5205E-30	-0.2384701	0.16	0.198	1.3097E-25	SYTL1
PARD3B	9.6791E-30	-0.8397636	0.014	0.028	1.4878E-25	PARD3B
DISC1	9.7107E-30	-0.227916	0.191	0.231	1.4926E-25	DISC1
SLC6A12	1.0261E-29	-0.200103	0.242	0.285	1.5772E-25	SLC6A12
CRY1	1.4271E-29	-0.2238016	0.183	0.223	2.1936E-25	CRY1
PER3	1.465E-29	-0.2240723	0.235	0.277	2.2519E-25	PER3
SULT1A1	1.6803E-29	-0.2850395	0.159	0.195	2.5828E-25	SULT1A1
NXF1	2.0585E-29	-0.1473315	0.44	0.491	3.1641E-25	NXF1
RARRES1	2.3102E-29	-0.5152284	0.039	0.059	3.5511E-25	RARRES1
TMEM255B	3.4838E-29	-0.2381973	0.152	0.188	5.3549E-25	TMEM255B
FMN1	3.6768E-29	-0.6410715	0.027	0.044	5.6517E-25	FMN1
TUT4	3.8955E-29	-0.1618581	0.411	0.46	5.9877E-25	TUT4
CENPP	4.2796E-29	-0.1833015	0.191	0.231	6.5782E-25	CENPP
TTC3	4.9173E-29	-0.1344173	0.567	0.614	7.5584E-25	TTC3
AGPAT4	5.1516E-29	-0.1496308	0.421	0.473	7.9186E-25	AGPAT4
TBC1D16	7.8705E-29	-0.1995761	0.221	0.262	1.2098E-24	TBC1D16
GALNT10	8.8009E-29	-0.1432621	0.432	0.483	1.3528E-24	GALNT10
SLC9A3R2	9.3891E-29	-0.2690407	0.108	0.14	1.4432E-24	SLC9A3R2
LDLRAD3	9.656E-29	-0.167555	0.274	0.32	1.4842E-24	LDLRAD3
IL6R	1.6865E-28	-0.2235386	0.169	0.206	2.5923E-24	IL6R
NUSAP1	1.6995E-28	-0.3052962	0.111	0.142	2.6123E-24	NUSAP1
BCR	1.8325E-28	-0.2355427	0.211	0.249	2.8167E-24	BCR
BAMBI	1.9508E-28	-0.520128	0.032	0.05	2.9986E-24	BAMBI
NPIPB15	2.0139E-28	-0.6135617	0.021	0.036	3.0956E-24	NPIPB15
DNAJB1	2.1585E-28	-0.1803521	0.54	0.588	3.3179E-24	DNAJB1
ZFYVE16	2.4969E-28	-0.1168776	0.674	0.715	3.838E-24	ZFYVE16
HIF1AN	3.1296E-28	-0.1632515	0.392	0.441	4.8105E-24	HIF1AN
ANAPC13	3.3401E-28	-0.1350905	0.473	0.526	5.134E-24	ANAPC13
NRGN	4.4792E-28	-0.229466	0.138	0.172	6.885E-24	NRGN
STMN1	5.9658E-28	-0.1291024	0.592	0.639	9.1701E-24	STMN1
EXOSC6	9.966E-28	-0.1869674	0.326	0.37	1.5319E-23	EXOSC6
AREL1	1.006E-27	-0.1969426	0.245	0.287	1.5463E-23	AREL1
ANKRD54	1.0239E-27	-0.2551043	0.148	0.182	1.5738E-23	ANKRD54
DUSP22	1.4061E-27	-0.1554243	0.472	0.522	2.1614E-23	DUSP22
CLEC18A	1.5401E-27	-1.2082617	0.005	0.014	2.3672E-23	CLEC18A
SMURF2	1.6256E-27	-0.1827878	0.31	0.356	2.4988E-23	SMURF2
DOCK1	1.6501E-27	-0.1512578	0.418	0.468	2.5363E-23	DOCK1
ICAM3	1.6814E-27	-0.2642978	0.172	0.208	2.5845E-23	ICAM3

GLMP	1.9892E-27	-0.1411252	0.449	0.497	3.0576E-23	GLMP
MAD1L1	2.1242E-27	-0.1577699	0.393	0.442	3.2651E-23	MAD1L1
CEACAM8	2.505E-27	-0.6060255	0.023	0.038	3.8504E-23	CEACAM8
PER1	2.629E-27	-0.1133247	0.574	0.621	4.041E-23	PER1
MLXIP	2.7968E-27	-0.1864007	0.268	0.312	4.299E-23	MLXIP
TUBGCP6	2.992E-27	-0.1520839	0.368	0.416	4.599E-23	TUBGCP6
KAT6B	3.4481E-27	-0.1711617	0.317	0.363	5.3001E-23	KAT6B
SYNE3	3.7358E-27	-0.2567716	0.104	0.133	5.7423E-23	SYNE3
GADD45G	4.3856E-27	-0.3292068	0.076	0.101	6.7411E-23	GADD45G
MAP3K12	5.1348E-27	-0.4192151	0.054	0.076	7.8927E-23	MAP3K12
IL2RA	5.5383E-27	-1.685999	0.005	0.013	8.513E-23	IL2RA
ACSF2	5.6574E-27	-0.1225882	0.448	0.499	8.696E-23	ACSF2
YBX3	5.7973E-27	-0.1068116	0.734	0.77	8.911E-23	YBX3
ECPAS	6.9503E-27	-0.1042861	0.716	0.755	1.0683E-22	ECPAS
SARDH	8.4954E-27	-1.5122064	0.003	0.011	1.3058E-22	SARDH
UHMK1	9.1089E-27	-0.100762	0.785	0.819	1.4001E-22	UHMK1
MAPK7	9.2786E-27	-0.1377921	0.543	0.588	1.4262E-22	MAPK7
GPR161	1.0988E-26	-0.3566536	0.08	0.105	1.689E-22	GPR161
GMPR	1.1067E-26	-0.1561554	0.289	0.333	1.7011E-22	GMPR
PCGF5	1.1355E-26	-0.1262428	0.568	0.613	1.7454E-22	PCGF5
SNX19	1.2856E-26	-0.1049821	0.635	0.682	1.9762E-22	SNX19
DHRSX	1.3603E-26	-0.127527	0.523	0.571	2.0909E-22	DHRSX
SLC25A33	1.4943E-26	-0.2209082	0.204	0.242	2.2968E-22	SLC25A33
FECH	1.5079E-26	-0.1583116	0.451	0.497	2.3177E-22	FECH
PRAG1	1.6449E-26	-0.1282163	0.529	0.572	2.5283E-22	PRAG1
P2RY11	1.8064E-26	-0.1833863	0.254	0.295	2.7766E-22	P2RY11
DDIT4L	2.1326E-26	-1.4897242	0.006	0.015	3.278E-22	DDIT4L
CCNT2	2.1725E-26	-0.1555581	0.347	0.394	3.3394E-22	CCNT2
PARP14	2.2052E-26	-0.1145633	0.712	0.745	3.3896E-22	PARP14
CAVIN4	2.2121E-26	-0.3105507	0.083	0.109	3.4002E-22	CAVIN4
GALNT6	2.2215E-26	-0.1171529	0.617	0.662	3.4147E-22	GALNT6
TTYH3	2.2957E-26	-0.1325244	0.464	0.514	3.5287E-22	TTYH3
LDLRAD4	2.6184E-26	-0.1070884	0.451	0.503	4.0248E-22	LDLRAD4
DCAF6	2.9358E-26	-0.1398933	0.454	0.502	4.5127E-22	DCAF6
TFEB	3.4033E-26	-0.2306485	0.164	0.199	5.2313E-22	TFEB
TM4SF19	3.5317E-26	-0.21695	0.077	0.102	5.4285E-22	TM4SF19
ZNF100	5.6221E-26	-0.3750267	0.067	0.091	8.6417E-22	ZNF100
TMEM60	6.4634E-26	-0.1362907	0.424	0.472	9.9349E-22	TMEM60
PIAS1	8.2982E-26	-0.1378514	0.596	0.639	1.2755E-21	PIAS1
UBA1	9.2527E-26	-0.1160741	0.641	0.682	1.4222E-21	UBA1
HTRA4	1.2753E-25	-0.2674099	0.116	0.146	1.9602E-21	HTRA4
AASDH	1.3162E-25	-0.2048943	0.205	0.243	2.0232E-21	AASDH
ZBTB8A	1.5948E-25	-0.2874163	0.101	0.129	2.4514E-21	ZBTB8A
APPL2	1.99E-25	-0.2098525	0.173	0.208	3.0589E-21	APPL2
MSH3	2.0282E-25	-0.2151704	0.252	0.293	3.1176E-21	MSH3
PTK2	2.3972E-25	-0.2393558	0.139	0.171	3.6848E-21	PTK2
LTBP4	3.2992E-25	-0.1817412	0.214	0.253	5.0712E-21	LTBP4
CPD	3.4677E-25	-0.1245293	0.553	0.6	5.3302E-21	CPD
ITGB1BP1	3.9762E-25	-0.1454788	0.434	0.483	6.1118E-21	ITGB1BP1
PLK3	4.4754E-25	-0.1581289	0.268	0.31	6.8791E-21	PLK3
ENTPD4	4.9234E-25	-0.1595571	0.325	0.37	7.5678E-21	ENTPD4
ADAMTSL2	7.3601E-25	-0.9744655	0.009	0.019	1.1313E-20	ADAMTSL2

SLC19A2	8.352E-25	-0.1781055	0.236	0.276	1.2838E-20	SLC19A2
ATP8B4	9.4508E-25	-0.7027624	0.023	0.038	1.4527E-20	ATP8B4
GZF1	1.0024E-24	-0.147302	0.275	0.318	1.5407E-20	GZF1
PHKB	1.1506E-24	-0.139306	0.465	0.511	1.7686E-20	PHKB
DNAH17	1.2662E-24	-0.7214947	0.012	0.023	1.9463E-20	DNAH17
ZHX3	1.5239E-24	-0.2818905	0.104	0.132	2.3423E-20	ZHX3
CD86	1.6317E-24	-0.1001805	0.722	0.76	2.508E-20	CD86
HERC6	1.749E-24	-0.1713279	0.254	0.295	2.6884E-20	HERC6
OCLN	1.8533E-24	-1.0283252	0.01	0.021	2.8487E-20	OCLN
TEX30	2.1351E-24	-0.232772	0.136	0.168	3.2819E-20	TEX30
VPS53	2.8061E-24	-0.1180299	0.526	0.573	4.3133E-20	VPS53
ANKRD9	2.8602E-24	-0.2929389	0.108	0.136	4.3964E-20	ANKRD9
CTSF	2.9332E-24	-0.1416866	0.326	0.371	4.5086E-20	CTSF
NFXL1	3.0845E-24	-0.2568521	0.128	0.158	4.7413E-20	NFXL1
PFKFB4	3.6215E-24	-0.2046269	0.16	0.193	5.5666E-20	PFKFB4
NOC2L	3.7048E-24	-0.1410624	0.396	0.441	5.6946E-20	NOC2L
WDFY2	4.5441E-24	-0.1326552	0.359	0.406	6.9848E-20	WDFY2
CARD16	5.3844E-24	-0.2765257	0.117	0.146	8.2764E-20	CARD16
FGFRL1	5.7723E-24	-0.2381148	0.165	0.198	8.8726E-20	FGFRL1
PAK1	6.1959E-24	-0.1259616	0.429	0.476	9.5237E-20	PAK1
ABCA7	6.2899E-24	-0.1224526	0.475	0.525	9.6681E-20	ABCA7
ATP10D	7.3065E-24	-0.1888739	0.23	0.269	1.1231E-19	ATP10D
SPINT1	9.5526E-24	-0.144088	0.47	0.513	1.4683E-19	SPINT1
ADGRD1	9.7174E-24	-0.4665723	0.041	0.059	1.4937E-19	ADGRD1
GCNT2	1.0033E-23	-0.2151282	0.151	0.183	1.5422E-19	GCNT2
BAG2	1.0728E-23	-0.2397208	0.129	0.159	1.649E-19	BAG2
MAP3K20	1.1628E-23	-0.1792943	0.206	0.244	1.7874E-19	MAP3K20
NR2F6	1.4757E-23	-0.1824133	0.213	0.25	2.2682E-19	NR2F6
SLIT3	1.6333E-23	-0.3832042	0.038	0.056	2.5105E-19	SLIT3
CDKN2C	1.9159E-23	-0.2219681	0.133	0.163	2.9449E-19	CDKN2C
SMARCC1	2.211E-23	-0.1142759	0.493	0.541	3.3986E-19	SMARCC1
TRPS1	3.525E-23	-0.1473511	0.317	0.359	5.4183E-19	TRPS1
SLC26A11	3.8113E-23	-0.1690049	0.191	0.227	5.8584E-19	SLC26A11
PPFIBP2	4.0305E-23	-0.1129238	0.443	0.49	6.1952E-19	PPFIBP2
TNK2	4.051E-23	-0.134132	0.387	0.433	6.2268E-19	TNK2
IFIT5	4.4604E-23	-0.1382176	0.525	0.569	6.8561E-19	IFIT5
AMPD2	4.8708E-23	-0.1024936	0.367	0.415	7.4868E-19	AMPD2
SH2D4A	5.1941E-23	-0.420783	0.04	0.058	7.9839E-19	SH2D4A
SLC25A13	6.2105E-23	-0.1316188	0.412	0.459	9.5461E-19	SLC25A13
KDM4B	6.8872E-23	-0.1271808	0.449	0.497	1.0586E-18	KDM4B
VPS13B	7.156E-23	-0.1168414	0.436	0.481	1.1E-18	VPS13B
DNAJA4	7.957E-23	-0.1282278	0.543	0.587	1.2231E-18	DNAJA4
NUP43	8.6471E-23	-0.1310727	0.44	0.486	1.3291E-18	NUP43
COL6A3	9.7282E-23	-0.308486	0.067	0.088	1.4953E-18	COL6A3
GIMAP7	9.9456E-23	-0.2715482	0.085	0.11	1.5287E-18	GIMAP7
STYX	1.3163E-22	-0.1265394	0.458	0.506	2.0233E-18	STYX
BDH2	1.3384E-22	-0.1524489	0.295	0.337	2.0573E-18	BDH2
PLAAT5	1.6119E-22	-0.5757673	0.023	0.037	2.4777E-18	PLAAT5
FOLR3	1.6146E-22	-0.3108362	0.382	0.42	2.4819E-18	FOLR3
SSH1	1.8932E-22	-0.1367194	0.445	0.492	2.9101E-18	SSH1
ZNF175	1.8956E-22	-0.1358697	0.286	0.327	2.9138E-18	ZNF175
EDA	1.9736E-22	-0.2637229	0.08	0.103	3.0337E-18	EDA

ARMCX3	2.233E-22	-0.15081	0.275	0.316	3.4324E-18	ARMCX3
HERC2	2.2389E-22	-0.176284	0.236	0.274	3.4414E-18	HERC2
MAL	2.8828E-22	-0.6939788	0.027	0.042	4.4312E-18	MAL
CENPM	3.1793E-22	-0.7317018	0.016	0.027	4.8869E-18	CENPM
FXYD6	3.5199E-22	-0.1352779	0.418	0.46	5.4104E-18	FXYD6
ELL2	4.294E-22	-0.1372149	0.597	0.636	6.6002E-18	ELL2
NPHP3	4.5494E-22	-0.1737828	0.164	0.197	6.9928E-18	NPHP3
SEL1L3	4.7599E-22	-0.1154669	0.602	0.646	7.3164E-18	SEL1L3
ROBO3	5.1392E-22	-0.3668008	0.064	0.085	7.8994E-18	ROBO3
GTF2A2	5.5854E-22	-0.1074781	0.643	0.679	8.5854E-18	GTF2A2
PRKX	6.225E-22	-0.3272989	0.069	0.091	9.5685E-18	PRKX
ACSM3	6.2584E-22	-0.2692524	0.093	0.118	9.6198E-18	ACSM3
CASP3	6.3041E-22	-0.1388551	0.376	0.42	9.6901E-18	CASP3
C16orf70	7.407E-22	-0.1053168	0.602	0.648	1.1385E-17	C16orf70
ENTPD6	7.5715E-22	-0.1112524	0.512	0.56	1.1638E-17	ENTPD6
RFK	8.4735E-22	-0.1528765	0.337	0.377	1.3025E-17	RFK
CCDC7	8.6413E-22	-0.2523531	0.102	0.128	1.3283E-17	CCDC7
C12orf4	9.106E-22	-0.1425176	0.329	0.372	1.3997E-17	C12orf4
AMACR	9.1707E-22	-0.1653832	0.245	0.282	1.4096E-17	AMACR
TLR3	1.1138E-21	-0.8700255	0.012	0.022	1.712E-17	TLR3
PTPDC1	1.281E-21	-0.1525924	0.246	0.284	1.9691E-17	PTPDC1
NOD1	1.3011E-21	-0.1412199	0.323	0.366	1.9999E-17	NOD1
ASNSD1	1.3057E-21	-0.1369126	0.414	0.461	2.007E-17	ASNSD1
ADCY1	1.3194E-21	-0.6733721	0.023	0.037	2.028E-17	ADCY1
CBFA2T2	1.3723E-21	-0.1539024	0.27	0.308	2.1093E-17	CBFA2T2
HUS1	1.8227E-21	-0.1153671	0.559	0.603	2.8016E-17	HUS1
SCARB1	1.8348E-21	-0.1070582	0.382	0.427	2.8203E-17	SCARB1
MICA	1.9072E-21	-0.1743584	0.167	0.2	2.9316E-17	MICA
HOMER1	2.0843E-21	-0.2264923	0.152	0.182	3.2038E-17	HOMER1
BET1	2.3169E-21	-0.1408014	0.296	0.338	3.5613E-17	BET1
COA1	2.3918E-21	-0.2155133	0.16	0.191	3.6765E-17	COA1
CLK4	2.4337E-21	-0.2072358	0.161	0.191	3.7409E-17	CLK4
NUDT2	2.8673E-21	-0.1270229	0.426	0.468	4.4073E-17	NUDT2
PI4KA	3.0979E-21	-0.1020541	0.644	0.683	4.7619E-17	PI4KA
MPHOSPH9	3.3069E-21	-0.1642874	0.222	0.257	5.083E-17	MPHOSPH9
YPEL4	3.4045E-21	-0.3316627	0.061	0.082	5.2331E-17	YPEL4
NME1	3.585E-21	-0.123929	0.392	0.437	5.5105E-17	NME1
MBOAT1	4.5255E-21	-0.1238086	0.446	0.49	6.9562E-17	MBOAT1
MAP4K1	6.0196E-21	-0.1661854	0.28	0.318	9.2528E-17	MAP4K1
CEP78	6.38E-21	-0.2045407	0.148	0.177	9.8067E-17	CEP78
SLC29A1	6.6767E-21	-0.1283119	0.645	0.676	1.0263E-16	SLC29A1
RAPH1	6.6857E-21	-0.195334	0.154	0.185	1.0277E-16	RAPH1
MEF2A	7.0899E-21	-0.1018808	0.699	0.732	1.0898E-16	MEF2A
PLAGL1	7.2196E-21	-0.1139033	0.469	0.513	1.1097E-16	PLAGL1
TMED2	7.9452E-21	-0.1122506	0.545	0.589	1.2213E-16	TMED2
TOB1	8.0116E-21	-0.1125174	0.52	0.563	1.2315E-16	TOB1
UBE2L3	8.02E-21	-0.1186586	0.515	0.559	1.2327E-16	UBE2L3
TRIB3	9.7164E-21	-0.1591437	0.099	0.124	1.4935E-16	TRIB3
CTSG	1.1916E-20	-0.4913545	0.027	0.04	1.8316E-16	CTSG
AKAP11	1.2016E-20	-0.114634	0.501	0.547	1.847E-16	AKAP11
CCR5	1.2479E-20	-0.5297892	0.025	0.039	1.9181E-16	CCR5
FAM13B	1.2668E-20	-0.1186066	0.462	0.505	1.9473E-16	FAM13B

IFFO2	1.5556E-20	-0.2116124	0.111	0.137	2.3912E-16	IFFO2
NBEAL2	1.5708E-20	-0.1164481	0.264	0.304	2.4144E-16	NBEAL2
INTS7	1.7242E-20	-0.1550696	0.272	0.31	2.6503E-16	INTS7
MEX3C	1.9357E-20	-0.262019	0.091	0.115	2.9754E-16	MEX3C
ARPP19	2.0617E-20	-0.1235055	0.504	0.546	3.169E-16	ARPP19
TIMD4	2.3476E-20	-0.5846325	0.024	0.038	3.6084E-16	TIMD4
SHLD2	2.4164E-20	-0.1027828	0.545	0.59	3.7143E-16	SHLD2
CCDC93	2.4498E-20	-0.1637362	0.198	0.232	3.7657E-16	CCDC93
RNF169	2.8596E-20	-0.1104051	0.464	0.509	4.3955E-16	RNF169
AKT3	3.0845E-20	-0.1193279	0.439	0.484	4.7412E-16	AKT3
FEM1B	3.0943E-20	-0.1070804	0.555	0.599	4.7562E-16	FEM1B
RASGRP3	3.2725E-20	-0.3942257	0.042	0.058	5.0302E-16	RASGRP3
BDP1	3.8862E-20	-0.11551	0.385	0.428	5.9735E-16	BDP1
RAB39B	4.2899E-20	-0.5905778	0.015	0.026	6.594E-16	RAB39B
MPZL2	4.3234E-20	-0.1054927	0.595	0.639	6.6455E-16	MPZL2
ECT2	4.9705E-20	-0.1908489	0.169	0.2	7.6401E-16	ECT2
MAP2K6	5.1093E-20	-0.339715	0.056	0.075	7.8535E-16	MAP2K6
CRB1	5.4234E-20	-0.7536938	0.011	0.021	8.3362E-16	CRB1
CAMKK1	6.1365E-20	-0.1509334	0.193	0.227	9.4324E-16	CAMKK1
MT-ND5	6.2158E-20	-0.2632482	0.243	0.276	9.5543E-16	MT-ND5
RUSC2	6.4554E-20	-0.1425443	0.293	0.332	9.9226E-16	RUSC2
RAD51B	6.5323E-20	-0.1210612	0.322	0.363	1.0041E-15	RAD51B
CLTC	7.1253E-20	-0.1086136	0.462	0.508	1.0952E-15	CLTC
PFKM	7.4245E-20	-0.250482	0.095	0.119	1.1412E-15	PFKM
AQR	7.542E-20	-0.1183322	0.443	0.487	1.1593E-15	AQR
LNPK	8.0702E-20	-0.1130301	0.564	0.606	1.2405E-15	LNPK
KHNYN	8.4988E-20	-0.1001503	0.58	0.621	1.3064E-15	KHNYN
ALK	8.677E-20	-1.3074303	0.004	0.011	1.3337E-15	ALK
UACA	9.1029E-20	-0.141231	0.348	0.389	1.3992E-15	UACA
VPS8	9.7324E-20	-0.1038755	0.515	0.558	1.496E-15	VPS8
MMP15	1.1596E-19	-0.2651356	0.062	0.082	1.7824E-15	MMP15
ZW10	1.2273E-19	-0.1275836	0.377	0.419	1.8865E-15	ZW10
MYO5A	1.3064E-19	-0.1127171	0.538	0.581	2.0081E-15	MYO5A
HCAR3	1.4802E-19	-0.12306	0.295	0.335	2.2752E-15	HCAR3
SHISAL2A	1.6697E-19	-0.2872813	0.063	0.083	2.5666E-15	SHISAL2A
TANGO6	1.8E-19	-0.1268325	0.305	0.345	2.7667E-15	TANGO6
FRYL	2.134E-19	-0.1295072	0.395	0.437	3.2801E-15	FRYL
GTPBP6	2.1773E-19	-0.1053311	0.548	0.588	3.3467E-15	GTPBP6
CD48	2.9794E-19	-0.3950951	0.038	0.054	4.5796E-15	CD48
TXLNG	2.9841E-19	-0.1621602	0.193	0.225	4.5869E-15	TXLNG
PLXNA3	3.3659E-19	-0.1079943	0.514	0.557	5.1736E-15	PLXNA3
PPP1R13B	3.4971E-19	-0.160973	0.16	0.19	5.3754E-15	PPP1R13B
ZNF552	3.8339E-19	-0.1582924	0.228	0.262	5.8931E-15	ZNF552
MKRN2	4.0468E-19	-0.1304896	0.298	0.337	6.2204E-15	MKRN2
HABP4	4.1636E-19	-0.1690649	0.215	0.249	6.3998E-15	HABP4
RBL1	4.1922E-19	-0.1925845	0.139	0.167	6.4438E-15	RBL1
STX2	4.8284E-19	-0.1351304	0.346	0.386	7.4217E-15	STX2
RTN4R	5.2993E-19	-0.2717117	0.097	0.12	8.1456E-15	RTN4R
RIC1	5.3044E-19	-0.1079656	0.402	0.444	8.1534E-15	RIC1
ATP5F1C	5.4117E-19	-0.1353514	0.337	0.378	8.3183E-15	ATP5F1C
PC	5.4571E-19	-0.2459699	0.094	0.117	8.3881E-15	PC
PELI1	5.6782E-19	-0.10174	0.274	0.313	8.7279E-15	PELI1

GPATCH2	5.7244E-19	-0.1373265	0.273	0.311	8.799E-15	GPATCH2
TOM1L1	6.2433E-19	-0.58144	0.022	0.034	9.5966E-15	TOM1L1
UCHL3	6.5889E-19	-0.1339782	0.341	0.38	1.0128E-14	UCHL3
LEPR	6.8226E-19	-0.2902344	0.102	0.125	1.0487E-14	LEPR
SLC25A32	7.4057E-19	-0.1370281	0.355	0.395	1.1383E-14	SLC25A32
BBS2	7.6131E-19	-0.1034643	0.49	0.534	1.1702E-14	BBS2
CDA	8.5365E-19	-0.9320694	0.005	0.012	1.3121E-14	CDA
FGFR1OP2	9.1271E-19	-0.1184859	0.478	0.521	1.4029E-14	FGFR1OP2
MT-ND4L	9.2281E-19	-0.3361347	0.257	0.285	1.4185E-14	MT-ND4L
KCNMB1	1.0216E-18	-0.3651466	0.055	0.073	1.5703E-14	KCNMB1
MBNL2	1.3788E-18	-0.130221	0.304	0.342	2.1193E-14	MBNL2
IPO5	1.4202E-18	-0.1818264	0.217	0.249	2.1831E-14	IPO5
ZFHX2	1.5304E-18	-0.3027459	0.074	0.094	2.3523E-14	ZFHX2
NUAK2	1.5852E-18	-0.2123146	0.114	0.139	2.4366E-14	NUAK2
DUSP16	1.6106E-18	-0.1819264	0.174	0.204	2.4757E-14	DUSP16
MAMLD1	1.6245E-18	-0.6760319	0.011	0.019	2.497E-14	MAMLD1
AK1	1.6567E-18	-0.1418334	0.2	0.233	2.5465E-14	AK1
RUBCNL	1.7461E-18	-0.1729662	0.223	0.255	2.6839E-14	RUBCNL
HEATR5A	1.8176E-18	-0.3028255	0.072	0.092	2.7939E-14	HEATR5A
ABCD3	1.8268E-18	-0.1285395	0.36	0.401	2.808E-14	ABCD3
DDX17	2.1148E-18	-0.1033644	0.56	0.601	3.2506E-14	DDX17
KLHDC10	2.16E-18	-0.1236161	0.375	0.416	3.3201E-14	KLHDC10
METTL4	2.3695E-18	-0.2075508	0.149	0.176	3.6421E-14	METTL4
MFGE8	2.4536E-18	-0.2184896	0.143	0.17	3.7714E-14	MFGE8
MINDY3	2.5315E-18	-0.1371303	0.345	0.385	3.8912E-14	MINDY3
AMDHD2	2.6206E-18	-0.1181703	0.312	0.351	4.0281E-14	AMDHD2
MFSD3	2.7274E-18	-0.1578316	0.161	0.19	4.1922E-14	MFSD3
TPST2	2.9874E-18	-0.1162781	0.356	0.397	4.592E-14	TPST2
TRIM6	3.058E-18	-0.2919161	0.075	0.095	4.7005E-14	TRIM6
SNAPC1	3.1972E-18	-0.1791426	0.195	0.227	4.9144E-14	SNAPC1
SNTB2	3.2535E-18	-0.1242398	0.282	0.32	5.0009E-14	SNTB2
PTPRJ	3.4761E-18	-0.1248007	0.533	0.571	5.3431E-14	PTPRJ
KANSL3	3.4901E-18	-0.1031925	0.384	0.427	5.3646E-14	KANSL3
CMTR2	4.6487E-18	-0.1002399	0.559	0.6	7.1455E-14	CMTR2
RITA1	4.6705E-18	-0.1955128	0.145	0.173	7.179E-14	RITA1
NUP210	4.866E-18	-0.1114003	0.479	0.517	7.4795E-14	NUP210
ATAD2B	5.1404E-18	-0.1477071	0.219	0.251	7.9014E-14	ATAD2B
LDHD	5.1763E-18	-0.1590851	0.259	0.294	7.9566E-14	LDHD
TPD52	5.233E-18	-0.1926482	0.163	0.192	8.0437E-14	TPD52
PKD2L1	5.379E-18	-0.1537138	0.199	0.231	8.268E-14	PKD2L1
UPF2	5.5683E-18	-0.1277213	0.383	0.421	8.5591E-14	UPF2
USP47	6.1076E-18	-0.1321922	0.335	0.374	9.388E-14	USP47
SSBP3	6.2347E-18	-0.2323867	0.109	0.132	9.5834E-14	SSBP3
DNAJC25	6.7338E-18	-0.1625559	0.22	0.252	1.035E-13	DNAJC25
ASXL2	6.8473E-18	-0.1193714	0.47	0.509	1.0525E-13	ASXL2
SNPH	7.2725E-18	-0.9096003	0.006	0.013	1.1179E-13	SNPH
DGKZ	7.4183E-18	-0.1251261	0.258	0.294	1.1403E-13	DGKZ
EFCAB13	7.4355E-18	-0.2636745	0.074	0.094	1.1429E-13	EFCAB13
FRRS1	7.448E-18	-0.1448983	0.28	0.316	1.1448E-13	FRRS1
FOXRED2	7.4506E-18	-0.2270342	0.089	0.111	1.1452E-13	FOXRED2
HSPA4	7.652E-18	-0.111302	0.469	0.509	1.1762E-13	HSPA4
SOCS1	7.8612E-18	-0.1300998	0.319	0.357	1.2083E-13	SOCS1

SMC4	8.0909E-18	-0.1685318	0.193	0.223	1.2437E-13	SMC4
CYP2S1	8.127E-18	-0.2202935	0.078	0.098	1.2492E-13	CYP2S1
PIK3C2B	8.6919E-18	-0.1779698	0.144	0.171	1.336E-13	PIK3C2B
C4orf33	8.7475E-18	-0.131428	0.252	0.287	1.3446E-13	C4orf33
ELOA	9.3883E-18	-0.1024107	0.531	0.57	1.4431E-13	ELOA
NCKIPSD	1.0291E-17	-0.1114894	0.339	0.379	1.5819E-13	NCKIPSD
XPR1	1.0602E-17	-0.1436348	0.266	0.302	1.6296E-13	XPR1
GANC	1.0968E-17	-0.1164555	0.382	0.422	1.6859E-13	GANC
RHBDF2	1.1138E-17	-0.1166341	0.343	0.382	1.7121E-13	RHBDF2
OSBPL1A	1.2105E-17	-0.1084591	0.502	0.544	1.8607E-13	OSBPL1A
GALE	1.2132E-17	-0.1040912	0.406	0.447	1.8648E-13	GALE
FILIP1L	1.2156E-17	-0.1464375	0.25	0.284	1.8685E-13	FILIP1L
MARCH9	1.2915E-17	-0.314084	0.055	0.072	1.9852E-13	MARCH9
CTR9	1.4177E-17	-0.1155042	0.594	0.634	2.1792E-13	CTR9
PABPC1L	1.4672E-17	-0.2437676	0.079	0.099	2.2553E-13	PABPC1L
ORC6	1.4898E-17	-0.38664	0.039	0.053	2.2899E-13	ORC6
RCL1	1.7548E-17	-0.1043487	0.446	0.489	2.6973E-13	RCL1
MED16	1.8939E-17	-0.1505884	0.23	0.264	2.9112E-13	MED16
SLC20A1	1.9203E-17	-0.1310744	0.385	0.424	2.9517E-13	SLC20A1
ACAD11	2.0635E-17	-0.1389011	0.149	0.177	3.1719E-13	ACAD11
ODF3B	2.0658E-17	-0.1022984	0.5	0.543	3.1753E-13	ODF3B
RBFOX2	2.1692E-17	-0.4035433	0.032	0.046	3.3343E-13	RBFOX2
ARHGEF35	2.2152E-17	-0.2915138	0.065	0.083	3.405E-13	ARHGEF35
KANSL1L	2.4558E-17	-0.1044067	0.257	0.294	3.7748E-13	KANSL1L
MAP2K4	2.464E-17	-0.1185389	0.291	0.328	3.7874E-13	MAP2K4
ZNF141	2.6813E-17	-0.22726	0.163	0.19	4.1214E-13	ZNF141
TARBP1	2.8459E-17	-0.1109265	0.332	0.371	4.3744E-13	TARBP1
HS2ST1	2.9999E-17	-0.1048804	0.441	0.481	4.6112E-13	HS2ST1
AKR1E2	3.0456E-17	-0.1858238	0.158	0.185	4.6814E-13	AKR1E2
DLG1	3.1353E-17	-0.1296278	0.335	0.373	4.8193E-13	DLG1
ZNF384	3.1561E-17	-0.1110557	0.517	0.556	4.8513E-13	ZNF384
PARP15	3.2222E-17	-0.1203118	0.26	0.295	4.9528E-13	PARP15
ITPR1	3.5129E-17	-0.1111883	0.494	0.534	5.3997E-13	ITPR1
OR52K2	3.9401E-17	-0.2753897	0.051	0.067	6.0563E-13	OR52K2
AKR7A2	4.0861E-17	-0.110668	0.449	0.491	6.2807E-13	AKR7A2
CHRNA3	4.2007E-17	-0.4237562	0.037	0.052	6.4569E-13	CHRNA3
SVBP	5.0306E-17	-0.1158993	0.465	0.505	7.7326E-13	SVBP
SELENON	5.0694E-17	-0.1221837	0.919	0.918	7.7922E-13	SELENON
PCYOX1L	5.46E-17	-0.163281	0.209	0.239	8.3926E-13	PCYOX1L
GRK5	5.5891E-17	-0.2379349	0.084	0.104	8.5911E-13	GRK5
LAMA1	5.6496E-17	-0.2803545	0.071	0.09	8.6841E-13	LAMA1
ARHGAP35	5.6842E-17	-0.1162418	0.356	0.394	8.7372E-13	ARHGAP35
PPM1H	6.2986E-17	-0.229978	0.106	0.128	9.6816E-13	PPM1H
PRR14L	6.3125E-17	-0.1006055	0.5	0.541	9.7029E-13	PRR14L
RNF111	6.3744E-17	-0.1078638	0.55	0.588	9.798E-13	RNF111
SAP30	6.4464E-17	-0.1423575	0.243	0.276	9.9087E-13	SAP30
TACC3	7.3509E-17	-0.1123323	0.331	0.369	1.1299E-12	TACC3
CCDC186	9.9596E-17	-0.164853	0.172	0.201	1.5309E-12	CCDC186
MYLIP	1.0129E-16	-0.1644363	0.142	0.168	1.557E-12	MYLIP
PLEKHA3	1.1875E-16	-0.1207392	0.42	0.459	1.8253E-12	PLEKHA3
XPOT	1.1913E-16	-0.1366229	0.267	0.302	1.8311E-12	XPOT
ADAT2	1.2405E-16	-0.2040317	0.092	0.113	1.9067E-12	ADAT2

SLC23A2	1.2788E-16	-0.1638144	0.195	0.225	1.9656E-12	SLC23A2
ATP8B1	1.323E-16	-0.378719	0.046	0.061	2.0336E-12	ATP8B1
SLC38A6	1.5737E-16	-0.1133082	0.355	0.394	2.4189E-12	SLC38A6
PRRG4	1.6209E-16	-0.2197469	0.125	0.149	2.4914E-12	PRRG4
DBF4B	1.6221E-16	-0.3021229	0.055	0.071	2.4933E-12	DBF4B
DNAJB4	1.7317E-16	-0.2057848	0.132	0.157	2.6618E-12	DNAJB4
PLAGL2	1.9647E-16	-0.1627076	0.17	0.198	3.02E-12	PLAGL2
GYS1	2.2259E-16	-0.1375243	0.255	0.288	3.4214E-12	GYS1
SLC25A30	2.2943E-16	-0.1060016	0.357	0.395	3.5266E-12	SLC25A30
ITSN1	2.3435E-16	-0.1398737	0.21	0.241	3.6022E-12	ITSN1
CYP2U1	2.4643E-16	-0.1459417	0.231	0.263	3.7879E-12	CYP2U1
TBCE.1	2.5794E-16	-0.1623214	0.159	0.186	3.9648E-12	TBCE.1
TENT4B	2.6114E-16	-0.1258652	0.311	0.348	4.0139E-12	TENT4B
MAVS	2.674E-16	-0.106998	0.38	0.419	4.1102E-12	MAVS
CCDC152	2.6964E-16	-1.1524058	0.004	0.01	4.1446E-12	CCDC152
CARD9	3.1027E-16	-0.1097678	0.356	0.394	4.7692E-12	CARD9
SNAPC3	3.2959E-16	-0.1395309	0.212	0.243	5.0661E-12	SNAPC3
LINS1	3.4088E-16	-0.1142216	0.303	0.34	5.2397E-12	LINS1
PALD1	3.6648E-16	-0.5806567	0.024	0.035	5.6331E-12	PALD1
PUS10	4.2838E-16	-0.2281716	0.096	0.117	6.5847E-12	PUS10
RCHY1	4.4623E-16	-0.1259718	0.31	0.346	6.8589E-12	RCHY1
STX3	4.5605E-16	-0.1039702	0.402	0.44	7.01E-12	STX3
RUNX3	4.8533E-16	-0.1764902	0.126	0.15	7.46E-12	RUNX3
MTF2	4.8885E-16	-0.1236333	0.314	0.351	7.5142E-12	MTF2
FAM126B	5.0309E-16	-0.141825	0.208	0.239	7.7329E-12	FAM126B
ALDH4A1	5.2184E-16	-0.2096145	0.105	0.127	8.0212E-12	ALDH4A1
NID1	5.605E-16	-0.4239759	0.025	0.036	8.6155E-12	NID1
CPNE9	5.7153E-16	-0.4441717	0.018	0.028	8.785E-12	CPNE9
IFT57	5.8066E-16	-0.1105813	0.371	0.409	8.9254E-12	IFT57
SOX13	6.3072E-16	-0.1737626	0.197	0.225	9.6948E-12	SOX13
STRBP	6.6575E-16	-0.2195665	0.113	0.136	1.0233E-11	STRBP
AC015871.1	8.0346E-16	-0.3708431	0.034	0.048	1.235E-11	AC015871.1
PLCXD1	8.6824E-16	-0.1530373	0.175	0.203	1.3346E-11	PLCXD1
MPP7	9.2219E-16	-0.247884	0.078	0.097	1.4175E-11	MPP7
MAML3	9.4139E-16	-0.1273212	0.223	0.254	1.447E-11	MAML3
TAF1C	1.0486E-15	-0.160761	0.137	0.162	1.6118E-11	TAF1C
HSD17B12	1.0923E-15	-0.111195	0.397	0.436	1.679E-11	HSD17B12
SNX16	1.1164E-15	-0.1419542	0.186	0.214	1.716E-11	SNX16
SUCO	1.1227E-15	-0.1214767	0.285	0.319	1.7257E-11	SUCO
XPO1	1.303E-15	-0.107089	0.391	0.429	2.0028E-11	XPO1
RHOBTB3	1.6122E-15	-0.4319249	0.03	0.043	2.4781E-11	RHOBTB3
SIAE	1.6487E-15	-0.1181445	0.271	0.304	2.5342E-11	SIAE
TP53I13	1.8553E-15	-0.1543688	0.167	0.194	2.8518E-11	TP53I13
MDGA1	1.8841E-15	-0.8098666	0.01	0.017	2.8961E-11	MDGA1
IL18BP	2.3869E-15	-0.1148361	0.248	0.28	3.6689E-11	IL18BP
CTDSPL	2.4156E-15	-0.1685488	0.145	0.17	3.713E-11	CTDSPL
LPGAT1	2.49E-15	-0.1075518	0.439	0.477	3.8273E-11	LPGAT1
ZFAND4	2.5499E-15	-0.2441597	0.081	0.1	3.9194E-11	ZFAND4
AKR1C2	2.5771E-15	-0.164402	0.213	0.241	3.9612E-11	AKR1C2
VPS50	2.7962E-15	-0.1124207	0.327	0.363	4.2981E-11	VPS50
PPM1K	3.3724E-15	-0.1492837	0.177	0.205	5.1837E-11	PPM1K
RELCH	3.4671E-15	-0.1032227	0.382	0.421	5.3292E-11	RELCH

HSPA14.1	3.9558E-15	-0.1336235	0.23	0.261	6.0805E-11	HSPA14.1
SERAC1	4.1062E-15	-0.1754402	0.131	0.154	6.3116E-11	SERAC1
LRP3	4.1349E-15	-0.4365297	0.032	0.044	6.3558E-11	LRP3
FTCDNL1	4.2775E-15	-0.1776366	0.186	0.214	6.5749E-11	FTCDNL1
LGR4	4.311E-15	-0.3400184	0.039	0.053	6.6264E-11	LGR4
LGALSL	4.3556E-15	-0.1199914	0.18	0.207	6.695E-11	LGALSL
CDK17	4.6233E-15	-0.1063731	0.301	0.336	7.1065E-11	CDK17
MDK	4.6348E-15	-0.1567236	0.209	0.237	7.1242E-11	MDK
DCBLD2	4.7471E-15	-0.2408753	0.071	0.089	7.2968E-11	DCBLD2
POLD1	4.7905E-15	-0.1046147	0.383	0.421	7.3635E-11	POLD1
CKS2	5.3489E-15	-0.2391768	0.082	0.1	8.2218E-11	CKS2
TBC1D14	5.5851E-15	-0.1006628	0.457	0.495	8.5848E-11	TBC1D14
EED	5.9575E-15	-0.1540108	0.189	0.217	9.1573E-11	EED
TMEM131	6.1552E-15	-0.1118198	0.456	0.493	9.4611E-11	TMEM131
HIBCH	6.3351E-15	-0.1707479	0.161	0.186	9.7377E-11	HIBCH
TET2	7.0383E-15	-0.116718	0.297	0.331	1.0819E-10	TET2
PODXL	7.0413E-15	-0.5376712	0.019	0.029	1.0823E-10	PODXL
DPY19L1	7.2579E-15	-0.1506976	0.205	0.234	1.1156E-10	DPY19L1
SCYL3	7.9723E-15	-0.1671003	0.163	0.189	1.2254E-10	SCYL3
PGM3	8.1943E-15	-0.1000344	0.405	0.443	1.2595E-10	PGM3
SLC2A13	1.0844E-14	-0.1936676	0.099	0.119	1.6669E-10	SLC2A13
PTPMT1	1.2091E-14	-0.1028464	0.339	0.375	1.8585E-10	PTPMT1
TMPPE	1.2734E-14	-0.1415193	0.186	0.213	1.9574E-10	TMPPE
THAP2	1.3674E-14	-0.1896122	0.091	0.111	2.1018E-10	THAP2
ADH5	1.4278E-14	-0.1108813	0.387	0.424	2.1946E-10	ADH5
CFH	1.6032E-14	-1.0334269	0.004	0.01	2.4643E-10	CFH
TMEM68	1.6257E-14	-0.1006933	0.445	0.484	2.4989E-10	TMEM68
FOCAD	1.7805E-14	-0.13232	0.2	0.228	2.7368E-10	FOCAD
SARM1	1.851E-14	-0.1546533	0.182	0.209	2.8451E-10	SARM1
CSE1L	1.8982E-14	-0.1397255	0.213	0.242	2.9178E-10	CSE1L
PTS	2.0361E-14	-0.1040264	0.258	0.29	3.1297E-10	PTS
HDAC4	2.2218E-14	-0.1143392	0.247	0.279	3.4151E-10	HDAC4
LURAP1	2.2919E-14	-0.2944834	0.056	0.071	3.5229E-10	LURAP1
PIK3R3	2.2981E-14	-0.3261172	0.036	0.049	3.5324E-10	PIK3R3
TXNDC16	2.7052E-14	-0.1982892	0.084	0.103	4.1582E-10	TXNDC16
ARMC2	2.7177E-14	-0.2958147	0.038	0.051	4.1774E-10	ARMC2
DIRAS3	2.847E-14	-0.8099353	0.006	0.012	4.3761E-10	DIRAS3
USP53	2.8521E-14	-0.2258201	0.081	0.099	4.3839E-10	USP53
SLC1A4	2.8919E-14	-0.1325676	0.186	0.213	4.4451E-10	SLC1A4
KCNK6	2.9162E-14	-0.1017689	0.377	0.415	4.4825E-10	KCNK6
FAM151B	3.0619E-14	-0.1360147	0.25	0.28	4.7064E-10	FAM151B
CALHM6	3.2333E-14	-0.2256567	0.113	0.134	4.9699E-10	CALHM6
ATP6V0E2	3.4831E-14	-0.1207103	0.179	0.206	5.3538E-10	ATP6V0E2
DDX55	3.641E-14	-0.1256221	0.197	0.225	5.5966E-10	DDX55
ST6GAL1	3.9995E-14	-0.3867227	0.031	0.043	6.1476E-10	ST6GAL1
BMF	4.0724E-14	-0.1072128	0.213	0.243	6.2597E-10	BMF
TMEM128	4.0895E-14	-0.1025787	0.624	0.658	6.286E-10	TMEM128
ENTPD7	4.1056E-14	-0.1644752	0.208	0.235	6.3108E-10	ENTPD7
IKZF2	4.2771E-14	-0.1086669	0.248	0.28	6.5744E-10	IKZF2
SAMD1	4.6557E-14	-0.1036911	0.363	0.399	7.1563E-10	SAMD1
MFN1	4.762E-14	-0.1407962	0.287	0.318	7.3197E-10	MFN1
ICE2	5.191E-14	-0.151488	0.175	0.201	7.979E-10	ICE2

PEAK1	5.2962E-14	-0.146537	0.203	0.231	8.1408E-10	PEAK1
DSN1	5.4682E-14	-0.1343034	0.203	0.231	8.4051E-10	DSN1
CLOCK	5.593E-14	-0.1166481	0.304	0.337	8.597E-10	CLOCK
NUDT14	5.7592E-14	-0.1015815	0.474	0.508	8.8525E-10	NUDT14
NUCB2	5.7993E-14	-0.1015528	0.394	0.432	8.914E-10	NUCB2
LARGE1	5.8644E-14	-0.5644621	0.017	0.026	9.0142E-10	LARGE1
KIAA0586	6.1609E-14	-0.1384702	0.18	0.206	9.4699E-10	KIAA0586
STBD1	7.7402E-14	-0.2102151	0.115	0.136	1.1897E-09	STBD1
BMPR1A	7.7832E-14	-0.1514031	0.161	0.186	1.1964E-09	BMPR1A
PRR15	8.1579E-14	-0.3094015	0.041	0.054	1.2539E-09	PRR15
ATG10	8.4077E-14	-0.1895072	0.117	0.138	1.2924E-09	ATG10
CECR2	8.5569E-14	-0.3789769	0.027	0.038	1.3153E-09	CECR2
MAP3K4	8.8286E-14	-0.1067639	0.362	0.397	1.357E-09	MAP3K4
GPRC5B	9.2552E-14	-0.6759357	0.011	0.018	1.4226E-09	GPRC5B
RIMKLA	9.3183E-14	-0.6903935	0.01	0.016	1.4323E-09	RIMKLA
NEXN	9.3668E-14	-0.2949775	0.054	0.069	1.4398E-09	NEXN
UHRF2	9.8926E-14	-0.1385204	0.164	0.189	1.5206E-09	UHRF2
ABCA5	1.0864E-13	-0.2562368	0.05	0.065	1.6699E-09	ABCA5
VKORC1L1	1.1161E-13	-0.1314895	0.233	0.262	1.7155E-09	VKORC1L1
PPP1R3B	1.2936E-13	-0.1346378	0.191	0.218	1.9883E-09	PPP1R3B
IL15	1.3711E-13	-0.181923	0.118	0.139	2.1075E-09	IL15
ANK2	1.4243E-13	-0.3740029	0.028	0.039	2.1893E-09	ANK2
HEXD	1.5419E-13	-0.1020873	0.244	0.274	2.3701E-09	HEXD
RAMAC	1.587E-13	-0.1531754	0.18	0.205	2.4393E-09	RAMAC
NIPA1	1.8336E-13	-0.1251911	0.199	0.226	2.8184E-09	NIPA1
RGP1	1.9684E-13	-0.1047069	0.317	0.35	3.0257E-09	RGP1
NAA16	2.1808E-13	-0.1299666	0.168	0.193	3.3521E-09	NAA16
LRR1	2.2303E-13	-0.1631545	0.177	0.201	3.4282E-09	LRR1
ZNF276	2.4394E-13	-0.1048966	0.272	0.304	3.7495E-09	ZNF276
TMEM266	2.5631E-13	-0.6087809	0.009	0.015	3.9398E-09	TMEM266
SLC37A4	2.7907E-13	-0.1028202	0.277	0.309	4.2897E-09	SLC37A4
UBTD1	2.9796E-13	-0.1089454	0.194	0.221	4.5799E-09	UBTD1
AVEN	3.014E-13	-0.157532	0.12	0.141	4.6329E-09	AVEN
CYB561D1	3.1663E-13	-0.142212	0.101	0.12	4.867E-09	CYB561D1
GMCL1	3.1885E-13	-0.1657792	0.121	0.142	4.901E-09	GMCL1
ARL3	3.2071E-13	-0.1289558	0.188	0.214	4.9297E-09	ARL3
SDC2	3.2505E-13	-0.4312377	0.043	0.055	4.9963E-09	SDC2
GKAP1	3.27E-13	-0.212782	0.084	0.101	5.0264E-09	GKAP1
PLCG1	3.667E-13	-0.1751442	0.083	0.1	5.6366E-09	PLCG1
CCNB1	3.8799E-13	-0.30698	0.054	0.068	5.9638E-09	CCNB1
GUF1	4.1161E-13	-0.1340013	0.193	0.219	6.3269E-09	GUF1
FAM104B	4.2408E-13	-0.1323598	0.213	0.24	6.5185E-09	FAM104B
KAT2B	4.3292E-13	-0.1007252	0.395	0.432	6.6545E-09	KAT2B
ZWINT	4.5233E-13	-0.1530938	0.132	0.154	6.9527E-09	ZWINT
ZNF805	4.6466E-13	-0.1315429	0.17	0.195	7.1422E-09	ZNF805
FAM3A	4.834E-13	-0.1086579	0.324	0.356	7.4304E-09	FAM3A
SIMC1	4.894E-13	-0.1573495	0.146	0.169	7.5225E-09	SIMC1
KAT14	4.9028E-13	-0.1686292	0.114	0.135	7.5361E-09	KAT14
PUS7	4.933E-13	-0.1338766	0.158	0.182	7.5825E-09	PUS7
WDR18	5.1142E-13	-0.1215968	0.258	0.289	7.8611E-09	WDR18
CADPS2	5.4478E-13	-0.1471931	0.149	0.171	8.3738E-09	CADPS2
AKTIP	5.7505E-13	-0.1171996	0.228	0.257	8.8391E-09	AKTIP

RRP8	6.2853E-13	-0.1208106	0.185	0.211	9.6612E-09	RRP8
ATAD3B	7.3211E-13	-0.2439053	0.056	0.071	1.1253E-08	ATAD3B
ENDOD1	7.3991E-13	-0.1368846	0.183	0.209	1.1373E-08	ENDOD1
SNX29	7.4004E-13	-0.1092595	0.281	0.312	1.1375E-08	SNX29
SS18L1	7.9678E-13	-0.1124788	0.182	0.208	1.2247E-08	SS18L1
LRP8	8.2252E-13	-0.227983	0.059	0.074	1.2643E-08	LRP8
SORD	8.5704E-13	-0.1302797	0.17	0.195	1.3174E-08	SORD
DDX58	8.644E-13	-0.1035129	0.453	0.488	1.3287E-08	DDX58
MDM1	9.4162E-13	-0.1273524	0.158	0.182	1.4474E-08	MDM1
PGM2L1	9.8958E-13	-0.1095025	0.168	0.193	1.5211E-08	PGM2L1
DSCC1	1.0077E-12	-0.5879127	0.013	0.021	1.5489E-08	DSCC1
PROSER3	1.0083E-12	-0.1690995	0.143	0.164	1.5499E-08	PROSER3
EXTL2	1.0809E-12	-0.1094544	0.204	0.232	1.6614E-08	EXTL2
TRIP11	1.2902E-12	-0.1179487	0.287	0.317	1.9832E-08	TRIP11
CYTH3	1.3328E-12	-0.1459554	0.14	0.162	2.0486E-08	CYTH3
MT1X	1.3583E-12	-0.5008869	0.02	0.029	2.0879E-08	MT1X
CLECL1	1.4184E-12	-0.4219718	0.027	0.037	2.1802E-08	CLECL1
TLN2	1.4377E-12	-0.3294167	0.035	0.047	2.21E-08	TLN2
ZNF398	1.4662E-12	-0.1128406	0.22	0.248	2.2536E-08	ZNF398
KBTBD8	1.5141E-12	-0.1570127	0.122	0.143	2.3273E-08	KBTBD8
TIGD2	1.6224E-12	-0.1909008	0.106	0.125	2.4938E-08	TIGD2
SSH2	1.7517E-12	-0.1058716	0.368	0.402	2.6925E-08	SSH2
PSME2	1.8279E-12	-0.1088781	0.314	0.345	2.8096E-08	PSME2
ZNF341	1.8605E-12	-0.184484	0.104	0.123	2.8598E-08	ZNF341
FNTB	1.9678E-12	-0.1335692	0.167	0.191	3.0248E-08	FNTB
SELL	2.0566E-12	-0.3867211	0.044	0.056	3.1612E-08	SELL
SLC30A6	2.0708E-12	-0.1023371	0.291	0.323	3.183E-08	SLC30A6
KLF8	2.2333E-12	-0.1163227	0.23	0.258	3.4328E-08	KLF8
SPATA5	2.4028E-12	-0.1167548	0.2	0.227	3.6933E-08	SPATA5
VNN2	2.6334E-12	-0.1421274	0.23	0.256	4.0478E-08	VNN2
CXorf40A	2.6339E-12	-0.1770485	0.199	0.224	4.0486E-08	CXorf40A
TP53INP1	2.6567E-12	-0.1395254	0.166	0.189	4.0836E-08	TP53INP1
PARP11	2.9426E-12	-0.1483936	0.142	0.164	4.5231E-08	PARP11
SLC25A16	2.9812E-12	-0.1021597	0.349	0.381	4.5824E-08	SLC25A16
ZFP92	3.2794E-12	-0.1105102	0.206	0.232	5.0408E-08	ZFP92
CASTOR2	3.8001E-12	-0.1412429	0.107	0.126	5.8411E-08	CASTOR2
KRT79	3.8135E-12	-0.1437031	0.057	0.071	5.8617E-08	KRT79
TSPAN6	3.8835E-12	-0.4923081	0.017	0.025	5.9693E-08	TSPAN6
ISL2	4.8565E-12	-0.5766114	0.01	0.017	7.4649E-08	ISL2
SCRN1	4.9036E-12	-0.1103389	0.153	0.176	7.5373E-08	SCRN1
PAQR8	5.2675E-12	-0.1746867	0.348	0.375	8.0967E-08	PAQR8
CCDC163	5.325E-12	-0.4592078	0.016	0.024	8.185E-08	CCDC163
RAB23	5.4855E-12	-0.2736628	0.051	0.064	8.4318E-08	RAB23
NT5DC3	5.6224E-12	-0.226885	0.059	0.073	8.6422E-08	NT5DC3
COMMD8	6.0313E-12	-0.1194928	0.242	0.271	9.2707E-08	COMMD8
SLC7A5	6.0844E-12	-0.2213035	0.028	0.038	9.3523E-08	SLC7A5
TBC1D30	7.4691E-12	-0.1887635	0.093	0.11	1.1481E-07	TBC1D30
TRIM24	7.9485E-12	-0.1094086	0.224	0.251	1.2218E-07	TRIM24
MCM2	8.1037E-12	-0.1739618	0.159	0.181	1.2456E-07	MCM2
SOCS7	8.5136E-12	-0.1144499	0.139	0.161	1.3086E-07	SOCS7
GRB10	8.5437E-12	-0.3007445	0.042	0.054	1.3133E-07	GRB10
CEP295	8.7952E-12	-0.1610127	0.1	0.118	1.3519E-07	CEP295

TRIM23	9.2009E-12	-0.1364776	0.157	0.179	1.4143E-07	TRIM23
AVIL	1.0763E-11	-0.5952573	0.009	0.015	1.6544E-07	AVIL
TRIM36	1.1055E-11	-0.1116057	0.215	0.241	1.6993E-07	TRIM36
STRADA	1.1237E-11	-0.1101651	0.27	0.299	1.7273E-07	STRADA
ZDHHC14	1.1401E-11	-0.2333928	0.056	0.07	1.7524E-07	ZDHHC14
SLC6A16	1.1562E-11	-0.6933614	0.009	0.016	1.7771E-07	SLC6A16
TAS2R14	1.5708E-11	-0.1784115	0.088	0.105	2.4144E-07	TAS2R14
DTWD2	1.6379E-11	-0.1353524	0.162	0.185	2.5176E-07	DTWD2
ENTPD5	1.7272E-11	-0.1107374	0.253	0.28	2.6549E-07	ENTPD5
PIP5KL1	1.75E-11	-0.352014	0.019	0.028	2.6899E-07	PIP5KL1
PHETA1	1.7787E-11	-0.1200925	0.124	0.144	2.7341E-07	PHETA1
CHI3L1	1.9616E-11	-1.2079906	0.007	0.012	3.0152E-07	CHI3L1
ZNF692	2.0933E-11	-0.1864386	0.064	0.079	3.2176E-07	ZNF692
USP28	2.1915E-11	-0.1290002	0.179	0.202	3.3686E-07	USP28
PLEKHA8	2.3234E-11	-0.1556014	0.121	0.14	3.5714E-07	PLEKHA8
AGO3	2.482E-11	-0.1022881	0.252	0.28	3.8151E-07	AGO3
ABHD18	2.7202E-11	-0.1089133	0.218	0.244	4.1813E-07	ABHD18
LY6G5B	2.8069E-11	-0.1691094	0.07	0.085	4.3144E-07	LY6G5B
KLF12	2.8333E-11	-0.2541509	0.039	0.05	4.3551E-07	KLF12
UBE2T	3.1307E-11	-0.1747478	0.077	0.092	4.8122E-07	UBE2T
ZBTB49	3.1911E-11	-0.1439033	0.094	0.111	4.9051E-07	ZBTB49
ZRSR2	3.3516E-11	-0.1241517	0.161	0.183	5.1517E-07	ZRSR2
RAD51AP1	3.381E-11	-0.2067622	0.066	0.08	5.197E-07	RAD51AP1
IER5L	3.385E-11	-0.116213	0.176	0.199	5.203E-07	IER5L
ZNF181	3.4102E-11	-0.2464656	0.057	0.07	5.2418E-07	ZNF181
RGS14	3.9804E-11	-0.1198725	0.164	0.187	6.1182E-07	RGS14
TMEM260	4.102E-11	-0.1138047	0.167	0.19	6.3051E-07	TMEM260
ZNF334	4.1138E-11	-0.3449831	0.031	0.041	6.3233E-07	ZNF334
CEP250	4.1175E-11	-0.1250617	0.153	0.175	6.329E-07	CEP250
FKBP9	4.3421E-11	-0.1144581	0.245	0.272	6.6742E-07	FKBP9
KLHL42	4.4168E-11	-0.1167633	0.175	0.198	6.7891E-07	KLHL42
UGGT2	4.4638E-11	-0.1017547	0.244	0.271	6.8612E-07	UGGT2
PTPN3	4.7411E-11	-0.3702252	0.029	0.039	7.2876E-07	PTPN3
MUSTN1	4.896E-11	-0.1795868	0.062	0.076	7.5257E-07	MUSTN1
PDXP	5.1146E-11	-0.1234056	0.166	0.188	7.8617E-07	PDXP
C19orf47	6.1085E-11	-0.1230601	0.194	0.218	9.3894E-07	C19orf47
RPF2	6.3728E-11	-0.1038682	0.271	0.3	9.7957E-07	RPF2
TPX2	6.503E-11	-0.3205499	0.021	0.029	9.9958E-07	TPX2
ELOVL3	6.7141E-11	-0.3055426	0.039	0.05	1.032E-06	ELOVL3
ZNF613	7.0304E-11	-0.1688075	0.092	0.109	1.0806E-06	ZNF613
MYO1A	7.4211E-11	-0.1671881	0.104	0.121	1.1407E-06	MYO1A
DONSON	7.9289E-11	-0.1574081	0.08	0.095	1.2188E-06	DONSON
PRSS36	8.008E-11	-0.154349	0.149	0.169	1.2309E-06	PRSS36
WFIKKN1	8.3553E-11	-0.3725719	0.023	0.031	1.2843E-06	WFIKKN1
ZWILCH	8.957E-11	-0.1487948	0.126	0.145	1.3768E-06	ZWILCH
MLF1	8.983E-11	-0.1203673	0.231	0.256	1.3808E-06	MLF1
MAP2K5	9.1199E-11	-0.1119378	0.216	0.24	1.4018E-06	MAP2K5
TBCE	1.0067E-10	-0.1332686	0.119	0.138	1.5474E-06	TBCE
ZNF669	1.0764E-10	-0.2606694	0.04	0.051	1.6545E-06	ZNF669
STARD9	1.0966E-10	-0.4255843	0.018	0.026	1.6856E-06	STARD9
CD300C	1.1282E-10	-0.1096318	0.227	0.252	1.7341E-06	CD300C
S1PR2	1.1828E-10	-0.1900676	0.089	0.105	1.8181E-06	S1PR2

KATNBL1	1.4802E-10	-0.1278934	0.104	0.122	2.2752E-06	KATNBL1
DOP1B	1.5647E-10	-0.1002959	0.22	0.245	2.4051E-06	DOP1B
C21orf91	1.6338E-10	-0.117518	0.183	0.206	2.5114E-06	C21orf91
COG3	1.6541E-10	-0.101274	0.273	0.3	2.5426E-06	COG3
NBDY	1.6881E-10	-0.161634	0.083	0.099	2.5948E-06	NBDY
MLLT3	1.7209E-10	-0.1677675	0.079	0.094	2.6452E-06	MLLT3
CCDC51	1.8213E-10	-0.1461569	0.099	0.116	2.7996E-06	CCDC51
TMC5	1.8421E-10	-0.6775106	0.005	0.01	2.8315E-06	TMC5
CCDC14	1.9144E-10	-0.1008341	0.203	0.227	2.9427E-06	CCDC14
LILRB4	2.1388E-10	-0.1934228	0.062	0.075	3.2875E-06	LILRB4
RAD9B	2.1449E-10	-0.3158479	0.025	0.034	3.2969E-06	RAD9B
HES4	2.2608E-10	-0.4849848	0.008	0.014	3.4751E-06	HES4
FGD6	2.4012E-10	-0.1887946	0.075	0.09	3.6909E-06	FGD6
TCF7L1	2.7696E-10	-0.4788959	0.019	0.026	4.2572E-06	TCF7L1
SEC16B	3.0014E-10	-0.1157063	0.085	0.101	4.6135E-06	SEC16B
FKRP	3.46E-10	-0.1346531	0.094	0.11	5.3184E-06	FKRP
TRMT12	3.6005E-10	-0.1511714	0.089	0.105	5.5344E-06	TRMT12
CYSLTR2	3.6121E-10	-0.1283308	0.125	0.143	5.5522E-06	CYSLTR2
PACRGL	4.0435E-10	-0.1019298	0.17	0.192	6.2153E-06	PACRGL
C12orf49	4.0752E-10	-0.1343771	0.131	0.15	6.2639E-06	C12orf49
DMD	4.6397E-10	-0.2081219	0.068	0.082	7.1316E-06	DMD
TWF1	4.7562E-10	-0.1024122	0.203	0.227	7.3108E-06	TWF1
CARMIL1	4.8301E-10	-0.134007	0.122	0.14	7.4243E-06	CARMIL1
CENPE	4.9465E-10	-0.1773878	0.078	0.093	7.6032E-06	CENPE
ATP5MGL	5.2225E-10	-0.4935143	0.011	0.017	8.0276E-06	ATP5MGL
TRMT13	5.3062E-10	-0.1062119	0.146	0.166	8.1561E-06	TRMT13
MAD2L1	5.5916E-10	-0.2871832	0.033	0.042	8.5949E-06	MAD2L1
ACOT11	5.5928E-10	-0.2629657	0.037	0.047	8.5966E-06	ACOT11
CEP126	5.9857E-10	-0.1821995	0.038	0.049	9.2007E-06	CEP126
VARS2	6.1666E-10	-0.1171323	0.146	0.166	9.4787E-06	VARS2
SCLY	6.4443E-10	-0.1057519	0.144	0.164	9.9055E-06	SCLY
DAAM1	6.4838E-10	-0.1131342	0.147	0.167	9.9662E-06	DAAM1
NAF1	7.0599E-10	-0.1564778	0.107	0.124	1.0852E-05	NAF1
PHLDB1	7.0942E-10	-0.307242	0.025	0.034	1.0904E-05	PHLDB1
P2RY13	7.7404E-10	-0.1034771	0.288	0.314	1.1898E-05	P2RY13
KIAA0895L	7.9902E-10	-0.1751256	0.068	0.082	1.2282E-05	KIAA0895L
MEGF6	8.1246E-10	-0.3479653	0.027	0.036	1.2488E-05	MEGF6
ZGLP1	8.1344E-10	-0.1784141	0.058	0.071	1.2503E-05	ZGLP1
VEZT	8.303E-10	-0.1124795	0.171	0.192	1.2763E-05	VEZT
GLCCI1	8.6588E-10	-0.2111569	0.053	0.065	1.3309E-05	GLCCI1
SLC48A1	8.8454E-10	-0.1104564	0.176	0.197	1.3596E-05	SLC48A1
WDR19	9.0041E-10	-0.1332714	0.08	0.095	1.384E-05	WDR19
BCL11A	9.1909E-10	-0.2473251	0.044	0.054	1.4127E-05	BCL11A
ZSCAN25	9.3772E-10	-0.1271165	0.129	0.147	1.4414E-05	ZSCAN25
CBX8	9.4131E-10	-0.1078516	0.156	0.177	1.4469E-05	CBX8
FBXL18	9.6908E-10	-0.1237741	0.101	0.118	1.4896E-05	FBXL18
MSANTD2	9.8062E-10	-0.1385809	0.137	0.156	1.5073E-05	MSANTD2
TSPYL2	1.0118E-09	-0.1343513	0.12	0.137	1.5552E-05	TSPYL2
SEMA4F	1.0275E-09	-0.1392613	0.088	0.103	1.5793E-05	SEMA4F
PLPPR2	1.0523E-09	-0.1552075	0.055	0.067	1.6174E-05	PLPPR2
ITGA1	1.0724E-09	-0.3700368	0.039	0.049	1.6484E-05	ITGA1
DCAF4	1.0761E-09	-0.1253837	0.128	0.147	1.6541E-05	DCAF4

PEAK3	1.1565E-09	-0.3381887	0.029	0.038	1.7777E-05	PEAK3
TECPR2	1.2066E-09	-0.1049177	0.196	0.218	1.8546E-05	TECPR2
KLF14	1.3109E-09	-0.6725966	0.008	0.014	2.015E-05	KLF14
TPRG1	1.313E-09	-0.1134141	0.133	0.152	2.0183E-05	TPRG1
S100Z	1.3365E-09	-0.4665458	0.012	0.018	2.0543E-05	S100Z
CAMK1	1.3778E-09	-0.1633121	0.118	0.135	2.1179E-05	CAMK1
ST6GALNAC2	1.3915E-09	-0.2394408	0.044	0.055	2.1389E-05	ST6GALNAC2
CCDC153	1.42E-09	-0.202466	0.067	0.08	2.1827E-05	CCDC153
ATR	1.4857E-09	-0.1046868	0.24	0.264	2.2837E-05	ATR
ZFYVE9	1.5143E-09	-0.1871557	0.067	0.08	2.3276E-05	ZFYVE9
TNFAIP1	1.5464E-09	-0.1148641	0.117	0.134	2.377E-05	TNFAIP1
ALG6	1.5466E-09	-0.1107	0.146	0.166	2.3772E-05	ALG6
ZNF844	1.7294E-09	-0.172102	0.07	0.084	2.6582E-05	ZNF844
USP37	1.7364E-09	-0.1179302	0.153	0.173	2.669E-05	USP37
SPAG5	1.8045E-09	-0.2059002	0.037	0.046	2.7737E-05	SPAG5
ZNF254	1.9537E-09	-0.1104736	0.154	0.174	3.003E-05	ZNF254
DNAJC27	1.971E-09	-0.2430373	0.029	0.038	3.0297E-05	DNAJC27
SMPDL3A	2.0104E-09	-0.218277	0.033	0.042	3.0902E-05	SMPDL3A
NDUFAF6	2.023E-09	-0.1071763	0.131	0.149	3.1095E-05	NDUFAF6
SEPHS1	2.0419E-09	-0.1113569	0.205	0.227	3.1385E-05	SEPHS1
CENPQ	2.1906E-09	-0.1511996	0.094	0.109	3.3672E-05	CENPQ
CCDC15	2.3029E-09	-0.2102707	0.054	0.066	3.5398E-05	CCDC15
ZNF770	2.3533E-09	-0.1044313	0.157	0.177	3.6172E-05	ZNF770
WDR91	2.5554E-09	-0.1033517	0.154	0.174	3.9279E-05	WDR91
E2F1	2.5794E-09	-0.2215261	0.051	0.062	3.9648E-05	E2F1
PKP2	2.8046E-09	-0.1020278	0.127	0.145	4.311E-05	PKP2
RBBP8	2.9725E-09	-0.1022339	0.159	0.179	4.569E-05	RBBP8
BFSP1	3.0635E-09	-0.6135885	0.007	0.011	4.709E-05	BFSP1
ITGB3BP	3.0792E-09	-0.1389953	0.124	0.141	4.733E-05	ITGB3BP
DTNB	3.1193E-09	-0.1113809	0.166	0.187	4.7947E-05	DTNB
MPP3	3.3326E-09	-0.3884376	0.015	0.022	5.1225E-05	MPP3
ABHD12B	3.37E-09	-0.6493584	0.006	0.01	5.18E-05	ABHD12B
CTC1	3.4272E-09	-0.117946	0.152	0.171	5.268E-05	CTC1
PROX1	3.4553E-09	-0.4148372	0.015	0.021	5.3111E-05	PROX1
UBXN2A	3.6309E-09	-0.1043132	0.169	0.19	5.5811E-05	UBXN2A
VAV3	4.0564E-09	-0.1100943	0.188	0.209	6.2351E-05	VAV3
FRS2	4.4691E-09	-0.1000442	0.228	0.251	6.8694E-05	FRS2
KLK1	4.5404E-09	-0.1964769	0.046	0.057	6.9791E-05	KLK1
CCDC149	5.6412E-09	-0.1346491	0.119	0.136	8.671E-05	CCDC149
ZNF460	6.3704E-09	-0.171776	0.072	0.086	9.7919E-05	ZNF460
OTUD3	6.4313E-09	-0.1118035	0.07	0.083	9.8856E-05	OTUD3
CRH	6.5157E-09	-0.7566916	0.01	0.016	0.00010015	CRH
CDC42BPA	6.7706E-09	-0.1119664	0.118	0.135	0.00010407	CDC42BPA
ZNF577	6.9266E-09	-0.1810524	0.058	0.069	0.00010647	ZNF577
KRT10	7.2477E-09	-0.1066434	0.146	0.165	0.0001114	KRT10
ZNF888	7.8984E-09	-0.1947026	0.065	0.078	0.00012141	ZNF888
ZNF439	8.0122E-09	-0.1210936	0.111	0.127	0.00012316	ZNF439
GBP7	8.6805E-09	-0.3140572	0.028	0.036	0.00013343	GBP7
STRADB	8.7323E-09	-0.1161447	0.134	0.152	0.00013422	STRADB
POGLUT2	8.981E-09	-0.2336587	0.038	0.047	0.00013805	POGLUT2
RARG	9.0181E-09	-0.2135159	0.036	0.045	0.00013862	RARG
CCDC112	9.0625E-09	-0.1083943	0.189	0.209	0.0001393	CCDC112

AASS	9.3308E-09	-0.3779678	0.016	0.022	0.00014342	AASS
ARMC9	1.0708E-08	-0.1899086	0.037	0.047	0.0001646	ARMC9
CENPJ	1.1015E-08	-0.1150803	0.105	0.121	0.00016931	CENPJ
DYRK3	1.231E-08	-0.2759068	0.03	0.039	0.00018922	DYRK3
TBKBP1	1.2315E-08	-0.270335	0.028	0.036	0.00018929	TBKBP1
IPCEF1	1.2505E-08	-0.1215049	0.153	0.172	0.00019221	IPCEF1
PPP1R14B	1.3118E-08	-0.1063641	0.172	0.191	0.00020164	PPP1R14B
RTEL1	1.3327E-08	-0.1061665	0.161	0.18	0.00020485	RTEL1
AP1S3	1.4689E-08	-0.2220227	0.043	0.053	0.00022578	AP1S3
KIAA0825	1.5009E-08	-0.1564541	0.072	0.085	0.00023071	KIAA0825
WDR76	1.6376E-08	-0.206383	0.048	0.058	0.00025172	WDR76
PTBP2	1.6506E-08	-0.1550572	0.06	0.071	0.00025371	PTBP2
POLR3B	1.7402E-08	-0.1232365	0.139	0.156	0.00026749	POLR3B
KIF21B	1.7955E-08	-0.1824737	0.071	0.084	0.00027598	KIF21B
CHEK1	1.8392E-08	-0.2193618	0.039	0.049	0.0002827	CHEK1
SETD9	1.9916E-08	-0.123976	0.156	0.175	0.00030613	SETD9
DLX4	2.0276E-08	-0.1415184	0.063	0.075	0.00031167	DLX4
USP46	2.0969E-08	-0.12728	0.095	0.109	0.00032231	USP46
IGIP	2.1171E-08	-0.1082959	0.119	0.135	0.00032541	IGIP
ZNF567	2.1441E-08	-0.1223173	0.129	0.146	0.00032956	ZNF567
PPFIBP1	2.161E-08	-0.1117445	0.148	0.166	0.00033217	PPFIBP1
CA11	2.2879E-08	-0.2572242	0.028	0.036	0.00035167	CA11
SMPDL3B	2.2907E-08	-0.6340772	0.011	0.017	0.0003521	SMPDL3B
TGDS	2.4243E-08	-0.1000317	0.155	0.173	0.00037263	TGDS
SETD4	2.454E-08	-0.1003058	0.189	0.21	0.00037721	SETD4
CLSTN3	2.5344E-08	-0.2568561	0.027	0.035	0.00038956	CLSTN3
DHDH	2.6449E-08	-0.1391806	0.107	0.122	0.00040655	DHDH
ZNF302	2.8381E-08	-0.1933301	0.054	0.065	0.00043625	ZNF302
SAMD13	3.1088E-08	-0.173882	0.1	0.114	0.00047785	SAMD13
M1AP	3.1155E-08	-0.1215309	0.137	0.154	0.00047888	M1AP
EBLN2	3.1624E-08	-0.1173044	0.067	0.079	0.0004861	EBLN2
KIF3C	3.4905E-08	-0.2053015	0.062	0.073	0.00053653	KIF3C
RBM44	3.5532E-08	-0.4846793	0.009	0.014	0.00054617	RBM44
PRR22	3.6362E-08	-0.3974443	0.014	0.019	0.00055892	PRR22
NAA25	3.6866E-08	-0.1244504	0.127	0.144	0.00056666	NAA25
TRAF5	3.8039E-08	-0.158072	0.059	0.071	0.0005847	TRAF5
SVIP	3.8369E-08	-0.4102364	0.015	0.021	0.00058978	SVIP
CCDC146	3.8738E-08	-0.308662	0.03	0.038	0.00059544	CCDC146
HOXB4	3.8996E-08	-0.1227161	0.087	0.1	0.0005994	HOXB4
NEDD4	4.1936E-08	-0.1853332	0.043	0.053	0.0006446	NEDD4
TYMS	4.2365E-08	-0.5217543	0.011	0.016	0.0006512	TYMS
FAIM2	4.2482E-08	-0.2765144	0.032	0.041	0.00065299	FAIM2
MYEF2	4.6729E-08	-0.5359963	0.008	0.013	0.00071827	MYEF2
CES3	4.6788E-08	-0.5136519	0.011	0.016	0.00071917	CES3
RIPK4	4.9124E-08	-0.3203028	0.012	0.018	0.00075509	RIPK4
OR52K1	5.0368E-08	-0.2835552	0.018	0.024	0.00077421	OR52K1
ZNF484	5.1806E-08	-0.116349	0.089	0.103	0.00079631	ZNF484
SPATS2	5.3221E-08	-0.2117182	0.049	0.059	0.00081806	SPATS2
RAD18	5.3831E-08	-0.1140385	0.092	0.106	0.00082743	RAD18
YOD1	5.6692E-08	-0.1616921	0.087	0.1	0.00087141	YOD1
ZNF354B	5.721E-08	-0.1278221	0.102	0.117	0.00087937	ZNF354B
OR6N2	5.73E-08	-0.1394414	0.035	0.043	0.00088075	OR6N2

CSNK2A1	5.8947E-08	-0.1443555	0.074	0.086	0.00090607	CSNK2A1
PPM1L	6.5763E-08	-0.1084514	0.09	0.104	0.00101084	PPM1L
NAPEPLD	6.806E-08	-0.2097437	0.058	0.068	0.00104615	NAPEPLD
EZH2	6.9273E-08	-0.1389742	0.083	0.096	0.00106479	EZH2
ZSCAN12	7.1282E-08	-0.332837	0.025	0.032	0.00109568	ZSCAN12
DYNC2H1	7.218E-08	-0.121126	0.077	0.089	0.00110949	DYNC2H1
QRICH2	7.7622E-08	-0.4487519	0.009	0.014	0.00119313	QRICH2
SSBP2	8.262E-08	-0.2802422	0.03	0.037	0.00126995	SSBP2
SIRPD	8.6074E-08	-0.1002178	0.211	0.231	0.00132305	SIRPD
C14orf28	8.9852E-08	-0.1447972	0.08	0.092	0.00138111	C14orf28
SNX25	9.0977E-08	-0.2007693	0.036	0.045	0.0013984	SNX25
C1orf54	9.1631E-08	-0.1118561	0.092	0.105	0.00140846	C1orf54
ANKRD16	9.8663E-08	-0.1666067	0.047	0.057	0.00151655	ANKRD16
SULT1C2	9.872E-08	-0.4873351	0.008	0.013	0.00151743	SULT1C2
ZNF596	9.8838E-08	-0.3182874	0.02	0.027	0.00151924	ZNF596
RINT1	1.0809E-07	-0.109024	0.193	0.212	0.0016614	RINT1
MLH3	1.1145E-07	-0.1081598	0.118	0.133	0.00171314	MLH3
EEPD1	1.2826E-07	-0.1801824	0.061	0.072	0.00197153	EEPD1
USP31	1.29E-07	-0.1011072	0.102	0.116	0.00198294	USP31
XKR6	1.4198E-07	-0.1580017	0.061	0.072	0.00218236	XKR6
BBS9	1.4992E-07	-0.1024565	0.152	0.17	0.00230434	BBS9
SPRY2	1.5069E-07	-0.4817162	0.01	0.014	0.00231622	SPRY2
CD320	1.6429E-07	-0.1226834	0.064	0.075	0.00252533	CD320
NLRP3	1.6856E-07	-0.2344248	0.037	0.045	0.00259088	NLRP3
SHLD3	1.7511E-07	-0.1380571	0.054	0.064	0.00269167	SHLD3
CENPK	1.8279E-07	-0.4119391	0.012	0.017	0.00280963	CENPK
FAM43A	1.8613E-07	-0.2045415	0.022	0.028	0.00286095	FAM43A
FBXL2	2.2126E-07	-0.1518515	0.046	0.055	0.00340094	FBXL2
TEF	2.6182E-07	-0.1048013	0.157	0.174	0.00402439	TEF
S100A3	2.6727E-07	-0.4964588	0.009	0.013	0.0041082	S100A3
ADHFE1	2.7343E-07	-0.438631	0.01	0.015	0.00420293	ADHFE1
STXBP4	2.9011E-07	-0.1231044	0.099	0.113	0.00445927	STXBP4
AC091057.6	2.992E-07	-0.2525598	0.036	0.044	0.00459902	AC091057.6
ZNF699	3.1157E-07	-0.1367156	0.085	0.098	0.00478908	ZNF699
NDST2	3.2431E-07	-0.1023797	0.102	0.116	0.00498493	NDST2
DUSP8	3.3247E-07	-0.1306473	0.069	0.081	0.0051104	DUSP8
LARP1B	3.4738E-07	-0.1059501	0.08	0.092	0.00533956	LARP1B
SLC27A5	3.5473E-07	-0.1460448	0.091	0.103	0.00545259	SLC27A5
HEXIM2	3.6256E-07	-0.140685	0.074	0.086	0.00557293	HEXIM2
ZNF670	3.6987E-07	-0.2091521	0.05	0.059	0.00568521	ZNF670
ZNF619	3.7174E-07	-0.1125158	0.115	0.13	0.00571396	ZNF619
SUV39H2	3.7966E-07	-0.2232386	0.048	0.057	0.00583583	SUV39H2
SEC31B	3.8258E-07	-0.1277803	0.046	0.055	0.0058806	SEC31B
HSF2	3.9569E-07	-0.1319403	0.098	0.112	0.00608221	HSF2
GPX7	4.1899E-07	-0.1086863	0.138	0.153	0.00644025	GPX7
RNF125	4.4034E-07	-0.1174542	0.135	0.15	0.0067685	RNF125
CNOT7	4.6276E-07	-0.1314328	0.077	0.088	0.00711307	CNOT7
ORC1	4.6731E-07	-0.2538484	0.023	0.029	0.00718296	ORC1
POLE2	5.0413E-07	-0.5638129	0.008	0.012	0.00774906	POLE2
DNAL1	5.1187E-07	-0.1103127	0.111	0.125	0.0078679	DNAL1
RFX2	5.2621E-07	-0.105485	0.152	0.169	0.00808831	RFX2
CEP97	5.5128E-07	-0.1400428	0.073	0.084	0.0084738	CEP97

KDM7A	5.7651E-07	-0.106175	0.097	0.11	0.00886153	KDM7A
TSPOAP1	5.7915E-07	-0.329937	0.018	0.024	0.0089021	TSPOAP1
SENP8	5.9591E-07	-0.1905019	0.042	0.051	0.00915967	SENP8
SDCBP2	6.2947E-07	-0.3783605	0.012	0.017	0.00967566	SDCBP2
FZD8	7.0083E-07	-0.1920933	0.036	0.044	0.01077244	FZD8
CEP72	7.0175E-07	-0.1009052	0.055	0.065	0.01078654	CEP72
ERCC8	7.3535E-07	-0.1028309	0.175	0.193	0.01130304	ERCC8
CMTM2	7.3614E-07	-0.1428486	0.047	0.057	0.01131516	CMTM2
GOLGA8B	7.4021E-07	-0.1724663	0.068	0.078	0.01137779	GOLGA8B
INPP5A	7.4366E-07	-0.2053491	0.049	0.058	0.01143079	INPP5A
ACOT1	7.6337E-07	-0.405517	0.017	0.023	0.01173373	ACOT1
GPAM	8.0132E-07	-0.1601478	0.052	0.061	0.01231713	GPAM
PALB2	8.2061E-07	-0.104621	0.156	0.172	0.01261355	PALB2
KIAA1257	8.2439E-07	-0.5251369	0.009	0.013	0.01267166	KIAA1257
TIMP4	8.3308E-07	-0.4091394	0.016	0.022	0.01280525	TIMP4
ITK	8.3658E-07	-0.4452975	0.012	0.017	0.01285906	ITK
PARS2	8.457E-07	-0.1401207	0.058	0.068	0.01299928	PARS2
C15orf62	8.5819E-07	-0.2467686	0.026	0.033	0.01319116	C15orf62
TCEANC	1.0117E-06	-0.1288468	0.063	0.074	0.0155513	TCEANC
LGALS2	1.1885E-06	-0.4260162	0.007	0.011	0.01826848	LGALS2
CDR2L	1.2179E-06	-0.1275161	0.063	0.073	0.01872041	CDR2L
RNF34	1.3266E-06	-0.1536205	0.078	0.089	0.02039191	RNF34
CCAR1	1.3272E-06	-0.1370544	0.082	0.093	0.02040112	CCAR1
COG6	1.3672E-06	-0.1217813	0.071	0.082	0.02101476	COG6
PCDHGC3	1.4432E-06	-0.4028901	0.014	0.018	0.02218392	PCDHGC3
CDC14B	1.4691E-06	-0.1428945	0.063	0.073	0.02258095	CDC14B
MAP9	1.6679E-06	-0.3254973	0.018	0.024	0.02563793	MAP9
KNL1	1.7634E-06	-0.4481632	0.01	0.014	0.02710475	KNL1
FAM135A	1.8527E-06	-0.1634704	0.059	0.069	0.02847796	FAM135A
STK36	1.9796E-06	-0.1092399	0.081	0.093	0.03042793	STK36
ST6GALNAC3	2.0593E-06	-0.2917334	0.01	0.014	0.0316533	ST6GALNAC3
SLC4A11	2.0616E-06	-0.1151	0.059	0.069	0.031689	SLC4A11
KBTBD6	2.1036E-06	-0.1416091	0.064	0.074	0.03233413	KBTBD6
LMO7	2.3297E-06	-0.4401611	0.008	0.011	0.03580923	LMO7
BARD1	2.4811E-06	-0.1444245	0.059	0.069	0.03813711	BARD1
AGAP1	2.5437E-06	-0.2147565	0.035	0.042	0.03909881	AGAP1
GLMN	2.7194E-06	-0.1220835	0.092	0.104	0.04179935	GLMN
CDK11A	2.7424E-06	-0.1361159	0.06	0.07	0.04215374	CDK11A
IL15RA	2.7483E-06	-0.1250008	0.094	0.105	0.04224471	IL15RA
ZNF391	2.8004E-06	-0.3102961	0.021	0.026	0.04304498	ZNF391
BOK	2.804E-06	-0.1461686	0.048	0.057	0.04310013	BOK
ZC3H12D	2.9273E-06	-0.1491275	0.037	0.044	0.04499502	ZC3H12D
PSTK	2.984E-06	-0.1398207	0.071	0.081	0.04586773	PSTK
ARHGAP33	3.0972E-06	-0.371552	0.01	0.014	0.04760693	ARHGAP33
ZNF605	3.1056E-06	-0.1105115	0.046	0.054	0.04773552	ZNF605
ZNF765	3.1183E-06	-0.1135862	0.087	0.098	0.04793095	ZNF765
ZFP37	3.1526E-06	-0.2978761	0.014	0.019	0.04845796	ZFP37
TMCO2	3.1946E-06	-0.2600584	0.024	0.031	0.0491047	TMCO2
BEX3	3.1978E-06	-0.2242838	0.066	0.076	0.04915395	BEX3
DNAH1	3.2035E-06	-0.1241581	0.07	0.08	0.04924101	DNAH1
CDS1	3.5794E-06	-0.1427629	0.06	0.07	0.05501956	CDS1
INPP5J	3.7057E-06	-0.1751134	0.046	0.054	0.05695958	INPP5J

ZNF714	3.8876E-06	-0.1705603	0.04	0.048	0.05975577	ZNF714
PANK1	4.0473E-06	-0.4292054	0.007	0.01	0.06221085	PANK1
HIST1H4J	4.0556E-06	-0.3704047	0.015	0.019	0.062339	HIST1H4J
TMC4	4.3007E-06	-0.3209923	0.015	0.02	0.06610654	TMC4
GLI4	4.366E-06	-0.1010828	0.1	0.112	0.06711021	GLI4
ELK3	4.4651E-06	-0.1025502	0.062	0.071	0.06863364	ELK3
C5	4.5139E-06	-0.1566975	0.055	0.064	0.06938306	C5
DHRS13	4.8857E-06	-0.1184832	0.062	0.072	0.0750984	DHRS13
HSF4	5.355E-06	-0.3831012	0.008	0.012	0.08231135	HSF4
WEE1	5.6437E-06	-0.3146867	0.011	0.015	0.08675005	WEE1
TAS2R30	5.903E-06	-0.1423897	0.037	0.045	0.09073453	TAS2R30
ZNF713	6.655E-06	-0.1199888	0.04	0.048	0.10229424	ZNF713
KLF5	7.2194E-06	-0.1457492	0.061	0.07	0.11096989	KLF5
LAMB2	7.4592E-06	-0.1119756	0.183	0.198	0.11465461	LAMB2
TCTEX1D2	7.5272E-06	-0.1948417	0.032	0.039	0.11570136	TCTEX1D2
CLHC1	7.7072E-06	-0.1631516	0.043	0.051	0.11846725	CLHC1
BTBD19	8.0866E-06	-0.1593808	0.047	0.055	0.1242999	BTBD19
EGR1	8.3772E-06	-0.1506274	0.082	0.093	0.12876632	EGR1
FRMD3	8.7989E-06	-0.2347059	0.021	0.026	0.13524779	FRMD3
ARHGAP19	8.9107E-06	-0.130094	0.073	0.083	0.13696646	ARHGAP19
ZNF790	9.0961E-06	-0.1092384	0.053	0.062	0.13981569	ZNF790
CBR3	9.1966E-06	-0.2478816	0.014	0.018	0.14136152	CBR3
ZBTB43	9.2672E-06	-0.1170073	0.061	0.07	0.14244564	ZBTB43
C1orf53	9.3323E-06	-0.1969589	0.037	0.044	0.14344705	C1orf53
ATP23	9.6572E-06	-0.1161168	0.143	0.156	0.14844105	ATP23
DDR1	1.0072E-05	-0.3347411	0.014	0.019	0.15481904	DDR1
MAP3K14	1.175E-05	-0.1590312	0.03	0.037	0.18061684	MAP3K14
TBC1D8B	1.2584E-05	-0.1218576	0.054	0.063	0.19342394	TBC1D8B
B3GNT9	1.2995E-05	-0.3465362	0.01	0.014	0.19974446	B3GNT9
IRF6	1.3281E-05	-0.1011048	0.052	0.061	0.20414503	IRF6
CDT1	1.3552E-05	-0.2215885	0.017	0.022	0.20830061	CDT1
CA5B	1.4129E-05	-0.1098355	0.075	0.085	0.21717338	CA5B
EIF1AX	1.564E-05	-0.1154821	0.087	0.097	0.24040604	EIF1AX
P2RY14	1.5662E-05	-0.1590809	0.057	0.066	0.24073717	P2RY14
OTUB2	1.5812E-05	-0.1100832	0.067	0.077	0.24304177	OTUB2
GINS1	1.6791E-05	-0.1885645	0.021	0.027	0.25809595	GINS1
PLEKHA4	1.787E-05	-0.1462163	0.065	0.074	0.27468397	PLEKHA4
AURKC	1.8272E-05	-0.21876	0.026	0.032	0.2808589	AURKC
CYB5RL	1.8681E-05	-0.1222629	0.08	0.09	0.28715069	CYB5RL
AMT	1.8729E-05	-0.1033363	0.08	0.09	0.28787795	AMT
FGF13	1.9524E-05	-0.5137069	0.007	0.01	0.30010406	FGF13
ANKEF1	2.0019E-05	-0.1636967	0.045	0.053	0.30770773	ANKEF1
ZNF280B	2.0196E-05	-0.1080868	0.058	0.067	0.31043027	ZNF280B
KAZALD1	2.0279E-05	-0.2799688	0.019	0.024	0.31171614	KAZALD1
CLEC2D	2.0665E-05	-0.2674089	0.015	0.02	0.31764359	CLEC2D
CENPO	2.2342E-05	-0.143814	0.043	0.05	0.34341758	CENPO
TAF5	2.2769E-05	-0.1064178	0.1	0.111	0.34997819	TAF5
TPBG	2.297E-05	-0.3331647	0.014	0.018	0.35307875	TPBG
NPHP1	2.5313E-05	-0.1002481	0.059	0.068	0.38908978	NPHP1
FKBP1B	2.7551E-05	-0.1382648	0.041	0.048	0.42348797	FKBP1B
CCNF	2.771E-05	-0.2083188	0.024	0.029	0.42592529	CCNF
PFDN4	2.7896E-05	-0.1125812	0.052	0.06	0.42878695	PFDN4

GPSM2	2.8047E-05	-0.1683497	0.041	0.048	0.43111651	GPSM2
ZNF519	2.8774E-05	-0.1817749	0.039	0.046	0.44228262	ZNF519
GPR155	3.0634E-05	-0.1559445	0.045	0.052	0.47087659	GPR155
RPGRIP1L	3.2565E-05	-0.1396381	0.051	0.059	0.50055387	RPGRIP1L
JAG2	3.5042E-05	-0.2185517	0.009	0.013	0.53862594	JAG2
METTL18	3.7678E-05	-0.1064766	0.082	0.092	0.57914949	METTL18
NECTIN3	3.9371E-05	-0.4370701	0.007	0.01	0.60516762	NECTIN3
POM121C	3.9495E-05	-0.153872	0.05	0.057	0.60707753	POM121C
GINS2	4.1058E-05	-0.2264312	0.02	0.024	0.63109791	GINS2
PRDM5	4.1996E-05	-0.3263327	0.01	0.014	0.64552061	PRDM5
FJX1	4.4592E-05	-0.4660744	0.007	0.01	0.68542817	FJX1
SORBS1	4.4646E-05	-0.3922782	0.009	0.013	0.6862507	SORBS1
PLK1	4.53E-05	-0.3567533	0.011	0.015	0.69630886	PLK1
DEPDC1	4.5796E-05	-0.2275614	0.018	0.023	0.70393375	DEPDC1
ZNF720	4.9736E-05	-0.2147894	0.015	0.019	0.76448785	ZNF720
CLSPN	5.1804E-05	-0.4536469	0.009	0.013	0.79628215	CLSPN
MARS2	5.2459E-05	-0.183746	0.031	0.037	0.80635347	MARS2
FSTL3	5.3291E-05	-0.105518	0.039	0.046	0.8191328	FSTL3
XYLB	5.5825E-05	-0.1529461	0.035	0.041	0.85808991	XYLB
MARCH3	5.8356E-05	-0.166352	0.024	0.029	0.89698282	MARCH3
LRRC34	5.9325E-05	-0.1088811	0.051	0.059	0.91188199	LRRC34
RPA4	5.9618E-05	-0.1691708	0.022	0.027	0.91639234	RPA4
LBH	6.1091E-05	-0.1470601	0.02	0.024	0.93903416	LBH
RAB3D	6.11E-05	-0.4097437	0.007	0.01	0.93917183	RAB3D
DPF3	6.34E-05	-0.2453147	0.015	0.02	0.97452795	DPF3
LGALS12	6.3492E-05	-0.1574678	0.034	0.04	0.97594318	LGALS12
CHIT1	0.82102128	-0.7851498	0.012	0.012	1	CHIT1
CAPS2	0.00215602	-0.4301072	0.008	0.011	1	CAPS2
FOXM1	0.00026892	-0.4195075	0.008	0.011	1	FOXM1
ANKRD35	8.8247E-05	-0.3980494	0.01	0.013	1	ANKRD35
MAGEE1	0.00093209	-0.355753	0.01	0.013	1	MAGEE1
RAB17	0.00218377	-0.3521636	0.007	0.01	1	RAB17
TTK	0.00014081	-0.3410494	0.012	0.015	1	TTK
MYLK3	0.00078544	-0.3349434	0.011	0.014	1	MYLK3
KCTD1	0.00010388	-0.3345919	0.012	0.015	1	KCTD1
NPAS2	0.0011774	-0.3175544	0.011	0.014	1	NPAS2
CHN1	7.3536E-05	-0.3167184	0.008	0.011	1	CHN1
TMED6	0.00211932	-0.3155873	0.008	0.01	1	TMED6
RADX	0.0013791	-0.299495	0.009	0.011	1	RADX
LRRC37A3	0.01399021	-0.29632	0.008	0.01	1	LRRC37A3
USP51	0.00728542	-0.2859133	0.009	0.011	1	USP51
C12orf60	0.00660855	-0.2858648	0.016	0.019	1	C12orf60
SHROOM1	0.00023276	-0.2853786	0.013	0.016	1	SHROOM1
FITM1	0.00479197	-0.2848432	0.008	0.01	1	FITM1
PTGES3	0.00182769	-0.2744932	0.013	0.015	1	PTGES3
UCN	0.00231528	-0.2716014	0.014	0.017	1	UCN
TMEM37	8.6713E-05	-0.2705713	0.007	0.01	1	TMEM37
LAPTM4B	0.00051661	-0.2668574	0.01	0.012	1	LAPTM4B
PKN3	0.00018883	-0.2625073	0.008	0.011	1	PKN3
PCDHGB7	0.00444699	-0.2562573	0.009	0.011	1	PCDHGB7
EHD3	0.00109069	-0.2557596	0.01	0.013	1	EHD3
FZD4	0.00023536	-0.2546859	0.019	0.023	1	FZD4

ASAP3	0.00010916	-0.2511403	0.013	0.017	1	ASAP3
CACNB1	7.6738E-05	-0.248861	0.022	0.027	1	CACNB1
PPP1R36	0.00468022	-0.2476203	0.012	0.015	1	PPP1R36
CCDC151	0.0026679	-0.2453059	0.015	0.019	1	CCDC151
GLB1L2	0.00093988	-0.2435667	0.009	0.012	1	GLB1L2
CHRFAM7A	0.00548491	-0.2428932	0.008	0.01	1	CHRFAM7A
OR5B21	0.00019381	-0.2377167	0.009	0.012	1	OR5B21
GK3P	0.00313202	-0.2347801	0.012	0.015	1	GK3P
CDK6	0.07309316	-0.2334548	0.01	0.011	1	CDK6
HMMR	0.00716057	-0.2329245	0.011	0.013	1	HMMR
MINAR1	0.00031086	-0.2295132	0.016	0.02	1	MINAR1
SCN9A	0.0012085	-0.2285968	0.013	0.016	1	SCN9A
SLC12A8	0.00205475	-0.2285329	0.014	0.018	1	SLC12A8
HYKK	0.04153181	-0.2263985	0.009	0.011	1	HYKK
ARMH1	0.00475857	-0.2250529	0.01	0.013	1	ARMH1
CCDC62	0.00818737	-0.218777	0.01	0.012	1	CCDC62
SNED1	8.9247E-05	-0.2163646	0.02	0.025	1	SNED1
ZP3	0.00754172	-0.212476	0.009	0.011	1	ZP3
CCDC30	0.00015631	-0.2119641	0.025	0.03	1	CCDC30
KIFC1	0.00010163	-0.2115925	0.023	0.028	1	KIFC1
OR1B1	0.02392554	-0.2111186	0.009	0.011	1	OR1B1
KCNJ5	0.00044447	-0.2100811	0.024	0.028	1	KCNJ5
SYT4	0.00684375	-0.2097842	0.009	0.011	1	SYT4
CELSR1	0.0108588	-0.2097355	0.013	0.015	1	CELSR1
C5orf30	0.00091862	-0.2097281	0.023	0.027	1	C5orf30
MYH3	0.00464716	-0.2046731	0.009	0.012	1	MYH3
FLACC1	0.01655031	-0.20079	0.012	0.014	1	FLACC1
CCNE1	0.00016227	-0.2002553	0.03	0.035	1	CCNE1
MYLK4	0.0118399	-0.2001903	0.011	0.013	1	MYLK4
ANK1	0.00045707	-0.1985697	0.018	0.022	1	ANK1
ZNF367	0.00167028	-0.1978604	0.011	0.013	1	ZNF367
GPT	0.0004896	-0.1976762	0.013	0.016	1	GPT
DMC1	0.01245344	-0.1969874	0.015	0.017	1	DMC1
RNASE2	0.19622342	-0.1968241	0.014	0.015	1	RNASE2
MOCS1	0.0028159	-0.1965758	0.015	0.018	1	MOCS1
DEPDC1B	0.00926108	-0.1964742	0.008	0.01	1	DEPDC1B
PTH2R	0.00158247	-0.1956286	0.022	0.026	1	PTH2R
FAM222A	0.02657544	-0.1932448	0.01	0.012	1	FAM222A
TRIP13	0.00343834	-0.1922361	0.019	0.023	1	TRIP13
SYT17	0.30884729	-0.1917238	0.014	0.015	1	SYT17
FKBP7	0.00257087	-0.1903509	0.016	0.019	1	FKBP7
FOXC1	0.0045282	-0.1895656	0.01	0.013	1	FOXC1
KIF17	0.00048919	-0.1893716	0.012	0.015	1	KIF17
OTUD7A	0.00909654	-0.1887913	0.008	0.01	1	OTUD7A
ZNRF3	0.0002742	-0.1883887	0.024	0.028	1	ZNRF3
PSAT1	0.00334402	-0.18799	0.013	0.016	1	PSAT1
LANCL3	0.01100775	-0.1872925	0.011	0.014	1	LANCL3
NR6A1	0.00059602	-0.1867006	0.014	0.017	1	NR6A1
TRAF3IP3	0.00025152	-0.1856983	0.017	0.021	1	TRAF3IP3
ADSSL1	0.04392356	-0.1851548	0.019	0.021	1	ADSSL1
MYRF	0.02588224	-0.1838271	0.009	0.01	1	MYRF
ZC2HC1C	0.00027951	-0.1825234	0.024	0.029	1	ZC2HC1C

C11orf95	0.01521858	-0.1820521	0.015	0.018	1	C11orf95
ATXN7L2	0.04935855	-0.1816407	0.011	0.012	1	ATXN7L2
MTCP1	0.01099323	-0.1815728	0.017	0.02	1	MTCP1
C15orf65	0.00034658	-0.1757137	0.021	0.025	1	C15orf65
TEDC2	0.04332152	-0.1745858	0.011	0.012	1	TEDC2
STIL	0.00039621	-0.1720804	0.022	0.027	1	STIL
NBPF20	0.00013741	-0.1703733	0.026	0.031	1	NBPF20
TREML2	0.00391645	-0.1703377	0.018	0.021	1	TREML2
ULBP3	0.0426658	-0.1703068	0.014	0.016	1	ULBP3
SCUBE2	0.08821411	-0.1697809	0.009	0.011	1	SCUBE2
SCN11A	0.00269582	-0.1682628	0.018	0.022	1	SCN11A
ERVK3-1	0.00201332	-0.1680393	0.036	0.041	1	ERVK3-1
CEP55	0.08196732	-0.1659872	0.013	0.015	1	CEP55
TRPV1	0.00040082	-0.1642247	0.03	0.036	1	TRPV1
PTCH1	0.03579039	-0.1632862	0.011	0.013	1	PTCH1
HESX1	0.00253084	-0.1628611	0.015	0.018	1	HESX1
ANKAR	0.01224376	-0.1625848	0.021	0.024	1	ANKAR
BMP8B	0.00287743	-0.1623347	0.013	0.016	1	BMP8B
SCN8A	0.00068397	-0.1620456	0.026	0.03	1	SCN8A
TGM1	0.00178431	-0.1619244	0.017	0.02	1	TGM1
UBE3D	0.01198006	-0.1618803	0.011	0.013	1	UBE3D
DNM1	0.00056917	-0.1614008	0.018	0.022	1	DNM1
PKP4	0.00060181	-0.1596424	0.019	0.023	1	PKP4
ACTRT3	0.00246892	-0.1580301	0.013	0.016	1	ACTRT3
HS3ST3B1	0.00011824	-0.1580217	0.012	0.016	1	HS3ST3B1
A4GNT	0.00018875	-0.1579729	0.038	0.044	1	A4GNT
ABCB9	0.00017577	-0.157782	0.021	0.026	1	ABCB9
STAG3	0.00011494	-0.1577128	0.055	0.063	1	STAG3
ARHGAP39	0.00082627	-0.1567944	0.017	0.021	1	ARHGAP39
JADE3	0.00050824	-0.1561743	0.027	0.032	1	JADE3
MSS51	0.00243975	-0.1554484	0.025	0.029	1	MSS51
ZCWPW2	0.00025317	-0.155182	0.038	0.044	1	ZCWPW2
CAPN5	8.1175E-05	-0.1549769	0.035	0.041	1	CAPN5
TNFSF9	0.0084884	-0.1513468	0.018	0.021	1	TNFSF9
TNKS1BP1	0.00060398	-0.151091	0.018	0.021	1	TNKS1BP1
PSRC1	0.00253363	-0.1507609	0.021	0.024	1	PSRC1
PLK4	0.01612938	-0.1502341	0.021	0.024	1	PLK4
PLEKHA5	0.00130389	-0.1499235	0.024	0.029	1	PLEKHA5
KCNJ14	0.07728733	-0.149914	0.011	0.013	1	KCNJ14
HIST3H2A	0.00102464	-0.1498174	0.018	0.021	1	HIST3H2A
HECTD2	0.00035098	-0.1495385	0.051	0.058	1	HECTD2
SSMEM1	0.00388619	-0.1488194	0.017	0.02	1	SSMEM1
BEX5	0.01816585	-0.1485673	0.03	0.034	1	BEX5
TPM2	0.00071106	-0.1484672	0.022	0.026	1	TPM2
LPIN3	0.0003322	-0.1474934	0.031	0.036	1	LPIN3
CDKL3	0.00165029	-0.1472373	0.042	0.047	1	CDKL3
HIST1H2AC	0.00633253	-0.1451736	0.01	0.012	1	HIST1H2AC
DND1	0.03716794	-0.1443775	0.011	0.013	1	DND1
PAICS	7.0521E-05	-0.1442786	0.061	0.07	1	PAICS
PIK3R6	0.000137	-0.1440078	0.021	0.026	1	PIK3R6
SKA3	0.01966183	-0.1433244	0.009	0.011	1	SKA3
SLC15A2	0.0002649	-0.1430248	0.031	0.036	1	SLC15A2

PLB1	0.00026184	-0.1426568	0.08	0.088	1	PLB1
ZNF772	0.00023706	-0.1419937	0.039	0.046	1	ZNF772
ZIK1	0.00029542	-0.1407592	0.029	0.034	1	ZIK1
YPEL1	0.0131085	-0.1404634	0.01	0.012	1	YPEL1
EQTN	0.00826865	-0.1388571	0.015	0.018	1	EQTN
LIME1	0.0109317	-0.1386837	0.022	0.025	1	LIME1
POPDC2	7.9935E-05	-0.1386067	0.042	0.049	1	POPDC2
RAD54B	0.01385625	-0.1382091	0.021	0.024	1	RAD54B
SEPTIN1	0.07220637	-0.1381712	0.013	0.015	1	SEPTIN1
ZNF804A	0.00036124	-0.13735	0.06	0.068	1	ZNF804A
ADORA3	0.07112184	-0.1363501	0.015	0.016	1	ADORA3
PLXNC1	0.00023714	-0.1348486	0.024	0.029	1	PLXNC1
ZNF43	0.00065423	-0.1348203	0.041	0.046	1	ZNF43
EXOG	0.00185277	-0.1347704	0.033	0.037	1	EXOG
BEGAIN	0.02362328	-0.1346085	0.014	0.016	1	BEGAIN
LYNX1	0.00213877	-0.134234	0.018	0.022	1	LYNX1
REPS2	0.00102635	-0.1338245	0.026	0.031	1	REPS2
SPRY3	0.00069664	-0.1329344	0.03	0.035	1	SPRY3
BUB1	0.00012563	-0.1328712	0.035	0.041	1	BUB1
ZNF483	0.00029916	-0.132727	0.047	0.054	1	ZNF483
UBFD1	0.00015963	-0.1319633	0.048	0.055	1	UBFD1
WFIKKN2	0.10500119	-0.1311618	0.013	0.015	1	WFIKKN2
SYTL2	0.00162329	-0.1302883	0.011	0.014	1	SYTL2
NMT2	0.00162657	-0.1276149	0.048	0.053	1	NMT2
WDR62	0.00323119	-0.1261131	0.017	0.02	1	WDR62
CCDC189	0.03095099	-0.1256852	0.018	0.02	1	CCDC189
CDC42BPG	0.01898135	-0.124733	0.025	0.028	1	CDC42BPG
SIGLEC15	0.02775673	-0.1246604	0.021	0.024	1	SIGLEC15
C8orf58	0.00041968	-0.1234471	0.052	0.059	1	C8orf58
TBC1D32	0.00306423	-0.1230541	0.028	0.033	1	TBC1D32
NBPF11	0.00093681	-0.1222548	0.038	0.043	1	NBPF11
SLC30A4	0.0002327	-0.1222518	0.025	0.03	1	SLC30A4
PYHIN1	0.0101566	-0.1212138	0.018	0.021	1	PYHIN1
GAN	0.0004952	-0.121117	0.034	0.039	1	GAN
IGFBP7	0.01119645	-0.1209605	0.019	0.022	1	IGFBP7
HOXA4	0.09663434	-0.1206466	0.01	0.012	1	HOXA4
PDE7B	0.00449078	-0.1205194	0.012	0.015	1	PDE7B
ZNF674	0.00092214	-0.1203569	0.038	0.044	1	ZNF674
RAD51	0.00687257	-0.1202474	0.017	0.02	1	RAD51
IGF2BP3	0.000694	-0.1201305	0.043	0.049	1	IGF2BP3
HMGB3	0.00476463	-0.1191541	0.027	0.031	1	HMGB3
SGO2	6.614E-05	-0.1187352	0.057	0.065	1	SGO2
STXBP1	0.00012196	-0.1178293	0.04	0.047	1	STXBP1
LY6G5C	0.010077	-0.116809	0.013	0.015	1	LY6G5C
PRSS53	0.0202851	-0.1167592	0.019	0.022	1	PRSS53
HELLS	0.00048704	-0.11663	0.068	0.075	1	HELLS
DIP2C	0.00352614	-0.1164613	0.036	0.041	1	DIP2C
ZKSCAN3	0.00093329	-0.1153587	0.054	0.06	1	ZKSCAN3
NRN1L	0.0178291	-0.1148235	0.012	0.014	1	NRN1L
ANXA9	0.21238777	-0.114448	0.015	0.016	1	ANXA9
CELSR3	0.00427449	-0.1131865	0.018	0.021	1	CELSR3
CENPF	6.5429E-05	-0.1131454	0.044	0.051	1	CENPF

SLC25A4	0.07764056	-0.1128128	0.011	0.013	1	SLC25A4
ARVCF	0.00041629	-0.1127635	0.031	0.036	1	ARVCF
PAQR6	0.0437294	-0.1124424	0.018	0.021	1	PAQR6
BGLAP	0.00672054	-0.1112219	0.036	0.04	1	BGLAP
ZNF280C	0.00362626	-0.1111837	0.041	0.046	1	ZNF280C
F5	0.14628747	-0.1111139	0.033	0.035	1	F5
OIP5	0.00043929	-0.1104893	0.025	0.03	1	OIP5
ZNF287	0.00133041	-0.1102108	0.057	0.064	1	ZNF287
CLTCL1	0.17957493	-0.1101314	0.012	0.013	1	CLTCL1
TTC25	0.03341039	-0.1101168	0.01	0.012	1	TTC25
PTPN20	0.09134333	-0.1099387	0.009	0.01	1	PTPN20
AK4	0.01364389	-0.1098814	0.022	0.025	1	AK4
PUDP	0.0035536	-0.1091711	0.031	0.036	1	PUDP
L3MBTL1	0.00307171	-0.1091618	0.035	0.04	1	L3MBTL1
LINC02693	0.00247293	-0.1088122	0.043	0.048	1	LINC02693
TTC21A	0.00050891	-0.1085038	0.046	0.052	1	TTC21A
C5orf34	0.24223318	-0.1084675	0.009	0.01	1	C5orf34
TREML1	0.00391182	-0.108257	0.02	0.024	1	TREML1
ASB9	0.01247534	-0.1080206	0.04	0.044	1	ASB9
EFCAB7	0.00333485	-0.1075844	0.037	0.042	1	EFCAB7
IL37	0.38336198	-0.1074889	0.012	0.013	1	IL37
PFKFB1	0.32313745	-0.1065241	0.01	0.011	1	PFKFB1
USP54	0.00048936	-0.1049145	0.053	0.06	1	USP54
MYZAP	0.00501359	-0.1048527	0.028	0.032	1	MYZAP
KIF11	0.02868822	-0.1047083	0.018	0.021	1	KIF11
RAPSN	0.00256147	-0.1045811	0.015	0.019	1	RAPSN
CD209	0.00085937	-0.1042713	0.07	0.077	1	CD209
CEP112	0.03404654	-0.1039875	0.015	0.017	1	CEP112
ANKRD55	0.10743551	-0.1038346	0.017	0.019	1	ANKRD55
KIF23	0.0002455	-0.1038336	0.026	0.031	1	KIF23
KLHDC7B	0.00228212	-0.103771	0.066	0.072	1	KLHDC7B
SPTY2D1OS	0.07299315	-0.1035829	0.009	0.01	1	SPTY2D1OS
CIP2A	0.03913057	-0.1029256	0.025	0.027	1	CIP2A
MCM8	0.00012532	-0.1026021	0.104	0.114	1	MCM8
SLC22A1	0.03030599	-0.1015954	0.013	0.015	1	SLC22A1
HDX	7.9175E-05	-0.1013589	0.04	0.047	1	HDX

cluster
Main AMs
Main AMs
Main AMs
Main AMs
Main AMs
Main AMs
Main AMs
Main AMs
Main AMs
Main AMs
Main AMs
Main AMs
Main AMs
Main AMs
Main AMs
Main AMs
Main AMs
Main AMs
Main AMs
Main AMs
Main AMs
Main AMs
Main AMs
Main AMs
Main AMs
Main AMs
Main AMs
Main AMs
Main AMs
Main AMs
Main AMs
Main AMs
Main AMs
Main AMs
Main AMs
Main AMs
Main AMs
Main AMs
Main AMs
Main AMs
Main AMs
Main AMs
Main AMs
Main AMs
Main AMs
Main AMs
Main AMs
Main AMs

Main AMs
Main AMs
Main AMs
Main AMs
Main AMs
Main AMs
Main AMs
Main AMs
Main AMs
Main AMs
Main AMs
Main AMs
Main AMs
Main AMs
Main AMs
Main AMs
Main AMs
Main AMs
Main AMs
Main AMs
Main AMs
Main AMs
Main AMs
Main AMs
Main AMs
Main AMs
Main AMs
Main AMs
Main AMs
Main AMs
Main AMs
Main AMs
Main AMs
Main AMs
Main AMs
Main AMs
Main AMs
Main AMs
Main AMs
Main AMs
Main AMs
Main AMs
Main AMs
Main AMs
Main AMs
Main AMs
Main AMs
Main AMs
Main AMs
Main AMs
Main AMs
Main AMs

Main AMs
Main AMs
Main AMs
Main AMs
Main AMs
Main AMs
Main AMs
Main AMs
Main AMs
Main AMs
Main AMs
Main AMs
Main AMs
Main AMs
Main AMs
Main AMs
Main AMs
Main AMs
Main AMs
Main AMs
Main AMs
Main AMs
Main AMs
Main AMs
Main AMs
Main AMs
Main AMs
Main AMs
Main AMs
Main AMs
Main AMs
Main AMs
Main AMs
Main AMs
Main AMs
Main AMs
Main AMs
Main AMs
Main AMs
Main AMs
Main AMs
Main AMs
Main AMs
Main AMs
Main AMs
Main AMs
Main AMs
Main AMs
Main AMs
Main AMs
Main AMs
Main AMs

Main AMs
Main AMs
Main AMs
Main AMs
Main AMs
Main AMs
Main AMs
Main AMs
Main AMs
Main AMs
Main AMs
Main AMs
Main AMs
Main AMs
Main AMs
Main AMs
Main AMs
Main AMs
Main AMs
Main AMs
Main AMs
Main AMs
Main AMs
Main AMs
Main AMs
Main AMs
Main AMs
Main AMs
Main AMs
Main AMs
Main AMs
Main AMs
Main AMs
Main AMs
Main AMs
Main AMs
Main AMs
Main AMs
Main AMs
Main AMs
Main AMs
Main AMs
Main AMs
Main AMs
Main AMs
Main AMs
Main AMs
Main AMs
Main AMs
Main AMs
Main AMs
Main AMs

Main AMs
Main AMs
Main AMs
Main AMs
Main AMs
Main AMs
Main AMs
Main AMs
Main AMs
Main AMs
Main AMs
Main AMs
Main AMs
Main AMs
Main AMs
Main AMs
Main AMs
Main AMs
Main AMs
Main AMs
Main AMs
Main AMs
Main AMs
Main AMs
Main AMs
Main AMs
Main AMs
Main AMs
Main AMs
Main AMs
Main AMs
Main AMs
Main AMs
Main AMs
Main AMs
Main AMs
Main AMs
Main AMs
Main AMs
Main AMs
Main AMs
Main AMs
Main AMs
Main AMs
Main AMs
Main AMs
Main AMs
Main AMs
Main AMs
Main AMs
Main AMs
Main AMs

Main AMs
Main AMs
Main AMs
Main AMs
Main AMs
Main AMs
Main AMs
Main AMs
Main AMs
Main AMs
Main AMs
Main AMs
Main AMs
Main AMs
Main AMs
Main AMs
Main AMs
Main AMs
Main AMs
Main AMs
Main AMs
Main AMs
Main AMs
Main AMs
Main AMs
Main AMs
Main AMs
Main AMs
Main AMs
Main AMs
Main AMs
Main AMs
Main AMs
Main AMs
Main AMs
Main AMs
Main AMs
Main AMs
Main AMs
Main AMs
Main AMs
Main AMs
Main AMs
Main AMs
Main AMs
Main AMs
Main AMs
Main AMs
Main AMs
Main AMs
Main AMs
Main AMs

Main AMs
Main AMs
Main AMs
Main AMs
Main AMs
Main AMs
Main AMs
Main AMs
Main AMs
Main AMs
Main AMs
Main AMs
Main AMs
Main AMs
Main AMs
Main AMs
Main AMs
Main AMs
Main AMs
Main AMs
Main AMs
Main AMs
Main AMs
Main AMs
Main AMs
Main AMs
Main AMs
Main AMs
Main AMs
Main AMs
Main AMs
Main AMs
Main AMs
Main AMs
Main AMs
Main AMs
Main AMs
Main AMs
Main AMs
Main AMs
Main AMs
Main AMs
Main AMs
Main AMs
Main AMs
Main AMs
Main AMs
Main AMs
Main AMs
Main AMs
Main AMs
Main AMs

Main AMs
Main AMs
Main AMs
Main AMs
Main AMs
Main AMs
Main AMs
Main AMs
Main AMs
Main AMs
Main AMs
Main AMs
Main AMs
Main AMs
Main AMs
Main AMs
Main AMs
Main AMs
Main AMs
Main AMs
Main AMs
Main AMs
Main AMs
Main AMs
Main AMs
Main AMs
Main AMs
Main AMs
Main AMs
Main AMs
Main AMs
Main AMs
Main AMs
Main AMs
Main AMs
Main AMs
Main AMs
Main AMs
Main AMs
Main AMs
Main AMs
Main AMs
Main AMs
Main AMs
Main AMs
Main AMs
Main AMs
Main AMs
Main AMs
Main AMs
Main AMs
Main AMs

Main AMs
Main AMs
Main AMs
Main AMs
Main AMs
Main AMs
Main AMs
Main AMs
Main AMs
Main AMs
Main AMs
Main AMs
Main AMs
Main AMs
Main AMs
Main AMs
Main AMs
Main AMs
Main AMs
Main AMs
Main AMs
Main AMs
Main AMs
Main AMs
Main AMs
Main AMs
Main AMs
Main AMs
Main AMs
Main AMs
Main AMs
Main AMs
Main AMs
Main AMs
Main AMs
Main AMs
Main AMs
Main AMs
Main AMs
Main AMs
Main AMs
Main AMs
Main AMs
Main AMs
Main AMs
Main AMs
Main AMs
Main AMs
Main AMs
Main AMs
Main AMs
Main AMs

Main AMs
Main AMs
Main AMs
Main AMs
Main AMs
Main AMs
Main AMs
Main AMs
Main AMs
Main AMs
Main AMs
Main AMs
Main AMs
Main AMs
Main AMs
Main AMs
Main AMs
Main AMs
Main AMs
Main AMs
Main AMs
Main AMs
Main AMs
Main AMs
Main AMs
Main AMs
Main AMs
Main AMs
Main AMs
Main AMs
Main AMs
Main AMs
Main AMs
Main AMs
Main AMs
Main AMs
Main AMs
Main AMs
Main AMs
Main AMs
Main AMs
Main AMs
Main AMs
Main AMs
Main AMs
Main AMs
Main AMs
Main AMs
Main AMs
Main AMs
Main AMs
Main AMs

Main AMs
Main AMs
Main AMs
Main AMs
Main AMs
Main AMs
Main AMs
Main AMs
Main AMs
Main AMs
Main AMs
Main AMs
Main AMs
Main AMs
Main AMs
Main AMs
Main AMs
Main AMs
Main AMs
Main AMs
Main AMs
Main AMs
Main AMs
Main AMs
Main AMs
Main AMs
Main AMs
Main AMs
Main AMs
Main AMs
Main AMs
Main AMs
Main AMs
Main AMs
Main AMs
Main AMs
Main AMs
Main AMs
Main AMs
Main AMs
Main AMs
Main AMs
Main AMs
Main AMs
Main AMs
Main AMs
Main AMs
Main AMs
Main AMs
Main AMs
Main AMs
Main AMs

Main AMs
Main AMs
Main AMs
Main AMs
Main AMs
Main AMs
Main AMs
Main AMs
Main AMs
Main AMs
Main AMs
Main AMs
Main AMs
Main AMs
Main AMs
Main AMs
Main AMs
Main AMs
Main AMs
Main AMs
Main AMs
Main AMs
Main AMs
Main AMs
Main AMs
Main AMs
Main AMs
Main AMs
Main AMs
Main AMs
Main AMs
Main AMs
Main AMs
Main AMs
Main AMs
Main AMs
Main AMs
Main AMs
Main AMs
Main AMs
Main AMs
Main AMs
Main AMs
Main AMs
Main AMs
Main AMs
Main AMs
Main AMs
Main AMs
Main AMs
Main AMs
Main AMs

Main AMs
Main AMs
Main AMs
Main AMs
Main AMs
Main AMs
Main AMs
Main AMs
Main AMs
Main AMs
Main AMs
Main AMs
Main AMs
Main AMs
Main AMs
Main AMs
Main AMs
Main AMs
Main AMs
Main AMs
Main AMs
Main AMs
Main AMs
Main AMs
Main AMs
Main AMs
Main AMs
Main AMs
Main AMs
Main AMs
Main AMs
Main AMs
Main AMs
Main AMs
Main AMs
Main AMs
Main AMs
Main AMs
Main AMs
Main AMs
Main AMs
Main AMs
Main AMs
Main AMs
Main AMs
Main AMs
Main AMs
Main AMs
Main AMs
Main AMs
Main AMs
Main AMs

Main AMs
Main AMs
Main AMs
Main AMs
Main AMs
Main AMs
Main AMs
Main AMs
Main AMs
Main AMs
Main AMs
Main AMs
Main AMs
Main AMs
Main AMs
Main AMs
Main AMs
Main AMs
Main AMs
Main AMs
Main AMs
Main AMs
Main AMs
Main AMs
Main AMs
Main AMs
Main AMs
Main AMs
Main AMs
Main AMs
Main AMs
Main AMs
Main AMs
Main AMs
Main AMs
Main AMs
Main AMs
Main AMs
Main AMs
Main AMs
Main AMs
Main AMs
Main AMs
Main AMs
Main AMs
Main AMs
Main AMs
Main AMs
Main AMs
Main AMs
Main AMs
Main AMs

Main AMs
Main AMs
Main AMs
Main AMs
Main AMs
Main AMs
Main AMs
Main AMs
Main AMs
Main AMs
Main AMs
Main AMs
Main AMs
Main AMs
Main AMs
Main AMs
Main AMs
Main AMs
Main AMs
Main AMs
Main AMs
Main AMs
Main AMs
Main AMs
Main AMs
Main AMs
Main AMs
Main AMs
Main AMs
Main AMs
Main AMs
Main AMs
Main AMs
Main AMs
Main AMs
Main AMs
Main AMs
Main AMs
Main AMs
Main AMs
Main AMs
Main AMs
Main AMs
Main AMs
Main AMs
Main AMs
Main AMs
Main AMs
Main AMs
Main AMs
Main AMs
Main AMs

Main AMs
Main AMs
Main AMs
Main AMs
Main AMs
Main AMs
Main AMs
Main AMs
Main AMs
Main AMs
Main AMs
Main AMs
Main AMs
Main AMs
Main AMs
Main AMs
Main AMs
Main AMs
Main AMs
Main AMs
Main AMs
Main AMs
Main AMs
Main AMs
Main AMs
Main AMs
Main AMs
Main AMs
Main AMs
Main AMs
Main AMs
Main AMs
Main AMs
Main AMs
Main AMs
Main AMs
Main AMs
Main AMs
Main AMs
Main AMs
Main AMs
Main AMs
Main AMs
Main AMs
Main AMs
Main AMs
Main AMs
Main AMs
Main AMs
Main AMs
Main AMs
Main AMs

Main AMs
Main AMs
Main AMs
Main AMs
Main AMs
Main AMs
Main AMs
Main AMs
Main AMs
Main AMs
Main AMs
Main AMs
Main AMs
Main AMs
Main AMs
Main AMs
Main AMs
Main AMs
Main AMs
Main AMs
Main AMs
Main AMs
Main AMs
Main AMs
Main AMs
Main AMs
Main AMs
Main AMs
Main AMs
Main AMs
Main AMs
Main AMs
Main AMs
Main AMs
Main AMs
Main AMs
Main AMs
Main AMs
Main AMs
Main AMs
Main AMs
Main AMs
Main AMs
Main AMs
Main AMs
Main AMs
Main AMs
Main AMs
Main AMs
Main AMs
Main AMs
Main AMs

Main AMs
Main AMs
Main AMs
Main AMs
Main AMs
Main AMs
Main AMs
Main AMs
Main AMs
Main AMs
Main AMs
Main AMs
Main AMs
Main AMs
Main AMs
Main AMs
Main AMs
Main AMs
Main AMs
Main AMs
Main AMs
Main AMs
Main AMs
Main AMs
Main AMs
Main AMs
Main AMs
Main AMs
Main AMs
Main AMs
Main AMs
Main AMs
Main AMs
Main AMs
Main AMs
Main AMs
Main AMs
Main AMs
Main AMs
Main AMs
Main AMs
Main AMs
Main AMs
Main AMs
Main AMs
Main AMs
Main AMs
Main AMs
Main AMs
Main AMs
Main AMs
Main AMs

Main AMs
Main AMs
Main AMs
Main AMs
Main AMs
Main AMs
Main AMs
Main AMs
Main AMs
Main AMs
Main AMs
Main AMs
Main AMs
Main AMs
Main AMs
Main AMs
Main AMs
Main AMs
Main AMs
Main AMs
Main AMs
Main AMs
Main AMs
Main AMs
Main AMs
Main AMs
Main AMs
Main AMs
Main AMs
Main AMs
Main AMs
Main AMs
Main AMs
Main AMs
Main AMs
Main AMs
Main AMs
Main AMs
Main AMs
Main AMs
Main AMs
Main AMs
Main AMs
Main AMs
Main AMs
Main AMs
Main AMs
Main AMs
Main AMs
Main AMs
Main AMs
Main AMs

Main AMs
Main AMs
Main AMs
Main AMs
Main AMs
Main AMs
Main AMs
Main AMs
Main AMs
Main AMs
Main AMs
Main AMs
Main AMs
Main AMs
Main AMs
Main AMs
Main AMs
Main AMs
Main AMs
Main AMs
Main AMs
Main AMs
Main AMs
Main AMs
Main AMs
Main AMs
Main AMs
Main AMs
Main AMs
Main AMs
Main AMs
Main AMs
Main AMs
Main AMs
Main AMs
Main AMs
Main AMs
Main AMs
Main AMs
Main AMs
Main AMs
Main AMs
Main AMs
Main AMs
Main AMs
Main AMs
Main AMs
Main AMs
Main AMs
Main AMs
Main AMs
Main AMs

Main AMs
Main AMs
Main AMs
Main AMs
Main AMs
Main AMs
Main AMs
Main AMs
Main AMs
Main AMs
Main AMs
Main AMs
Main AMs
Main AMs
Main AMs
Main AMs
Main AMs
Main AMs
Main AMs
Main AMs
Main AMs
Main AMs
Main AMs
Main AMs
Main AMs
Main AMs
Main AMs
Main AMs
Main AMs
Main AMs
Main AMs
Main AMs
Main AMs
Main AMs
Main AMs
Main AMs
Main AMs
Main AMs
Main AMs
Main AMs
Main AMs
Main AMs
Main AMs
Main AMs
Main AMs
Main AMs
Main AMs
Main AMs
Main AMs
Main AMs
Main AMs
Main AMs

Main AMs
Main AMs
Main AMs
Main AMs
Main AMs
Main AMs
Main AMs
Main AMs
Main AMs
Main AMs
Main AMs
Main AMs
Main AMs
Main AMs
Main AMs
Main AMs
Main AMs
Main AMs
Main AMs
Main AMs
Main AMs
Main AMs
Main AMs
Main AMs
Main AMs
Main AMs
Main AMs
Main AMs
Main AMs
Main AMs
Main AMs
Main AMs
Main AMs
Main AMs
Main AMs
Main AMs
Main AMs
Main AMs
Main AMs
Main AMs
Main AMs
Main AMs
Main AMs
Main AMs
Main AMs
Main AMs
Main AMs
Main AMs
Main AMs
Main AMs
Main AMs
Main AMs

Main AMs
Main AMs
Main AMs
Main AMs
Main AMs
Main AMs
Main AMs
Main AMs
Main AMs
Main AMs
Main AMs
Main AMs
Main AMs
Main AMs
Main AMs
Main AMs
Main AMs
Main AMs
Main AMs
Main AMs
Main AMs
Main AMs
Main AMs
Main AMs
Main AMs
Main AMs
Main AMs
Main AMs
Main AMs
Main AMs
Main AMs
Main AMs
Main AMs
Main AMs
Main AMs
Main AMs
Main AMs
Main AMs
Main AMs
Main AMs
Main AMs
Main AMs
Main AMs
Main AMs
Main AMs
Main AMs
Main AMs
Main AMs
Main AMs
Main AMs
Main AMs
Main AMs

Main AMs
Main AMs
Main AMs
Main AMs
Main AMs
Main AMs
Main AMs
Main AMs
Main AMs
Main AMs
Main AMs
Main AMs
Main AMs
Main AMs
Main AMs
Main AMs
Main AMs
Main AMs
Main AMs
Main AMs
Main AMs
Main AMs
Main AMs
Main AMs
Main AMs
Main AMs
Main AMs
Main AMs
Main AMs
Main AMs
Main AMs
Main AMs
Main AMs
Main AMs
Main AMs
Main AMs
Main AMs
Main AMs
Main AMs
Main AMs
Main AMs
Main AMs
Main AMs
Main AMs
Main AMs
Main AMs
Main AMs
Main AMs
Main AMs
Main AMs
Main AMs
Main AMs

Main AMs
Main AMs
Main AMs
Main AMs
Main AMs
Main AMs
Main AMs
Main AMs
Main AMs
Main AMs
Main AMs
Main AMs
Main AMs
Main AMs
Main AMs
Main AMs
Main AMs
Main AMs
Main AMs
Main AMs
Main AMs
Main AMs
Main AMs
Main AMs
Main AMs
Main AMs
Main AMs
Main AMs
Main AMs
Main AMs
Main AMs
Main AMs
Main AMs
Main AMs
Main AMs
Main AMs
Main AMs
Main AMs
Main AMs
Main AMs
Main AMs
Main AMs
Main AMs
Main AMs
Main AMs
Main AMs
Main AMs
Main AMs
Main AMs
Main AMs
Main AMs
Main AMs

Main AMs
Main AMs
Main AMs
Main AMs
Main AMs
Main AMs
Main AMs
Main AMs
Main AMs
Main AMs
Main AMs
Main AMs
Main AMs
Main AMs
Main AMs
Main AMs
Main AMs
Main AMs
Main AMs
Main AMs
Main AMs
Main AMs
Main AMs
Main AMs
Main AMs
Main AMs
Main AMs
Main AMs
Main AMs
Main AMs
Main AMs
Main AMs
Main AMs
Main AMs
Main AMs
Main AMs
Main AMs
Main AMs
Main AMs
Main AMs
Main AMs
Main AMs
Main AMs
Main AMs
Main AMs
Main AMs
Main AMs
Main AMs
Main AMs
Main AMs
Main AMs
Main AMs

Main AMs
Main AMs
Main AMs
Main AMs
Main AMs
Main AMs
Main AMs
Main AMs
Main AMs
Main AMs
Main AMs
Main AMs
Main AMs
Main AMs
Main AMs
Main AMs
Main AMs
Main AMs
Main AMs
Main AMs
Main AMs
Main AMs
Main AMs
Main AMs
Main AMs
Main AMs
Main AMs
Main AMs
Main AMs
Main AMs
Main AMs
Main AMs
Main AMs
Main AMs
Main AMs
Main AMs
Main AMs
Main AMs
Main AMs
Main AMs
Main AMs
Main AMs
Main AMs
Main AMs
Main AMs
Main AMs
Main AMs
Main AMs
Main AMs
Main AMs
Main AMs
Main AMs

Main AMs
Main AMs
Main AMs
Main AMs
Main AMs
Main AMs
Main AMs
Main AMs
Main AMs
Main AMs
Main AMs
Main AMs
Main AMs
Main AMs
Main AMs
Main AMs
Main AMs
Main AMs
Main AMs
Main AMs
Main AMs
Main AMs
Main AMs
Main AMs
Main AMs
Main AMs
Main AMs
Main AMs
Main AMs
Main AMs
Main AMs
Main AMs
Main AMs
Main AMs
Main AMs
Main AMs
Main AMs
Main AMs
Main AMs
Main AMs
Main AMs
Main AMs
Main AMs
Main AMs
Main AMs
Main AMs
Main AMs
Main AMs
Main AMs
Main AMs
Main AMs
Main AMs

Main AMs
Main AMs
Main AMs
Main AMs
Main AMs
Main AMs
Main AMs
Main AMs
Main AMs
Main AMs
Main AMs
Main AMs
Main AMs
Main AMs
Main AMs
Main AMs
Main AMs
Main AMs
Main AMs
Main AMs
Main AMs
Main AMs
Main AMs
Main AMs
Main AMs
Main AMs
Main AMs
Main AMs
Main AMs
Main AMs
Main AMs
Main AMs
Main AMs
Main AMs
Main AMs
Main AMs
Main AMs
Main AMs
Main AMs
Main AMs
Main AMs
Main AMs
Main AMs
Main AMs
Main AMs
Main AMs
Main AMs
Main AMs
Main AMs
Main AMs
Main AMs
Main AMs

Main AMs
Main AMs
Main AMs
Main AMs
Main AMs
Main AMs
Main AMs
Main AMs
Main AMs
Main AMs
Main AMs
Main AMs
Main AMs
Main AMs
Main AMs
Main AMs
Main AMs
Main AMs
Main AMs
Main AMs
Main AMs
Main AMs
Main AMs
Main AMs
Main AMs
Main AMs
Main AMs
Main AMs
Main AMs
Main AMs
Main AMs
Main AMs
Main AMs
Main AMs
Main AMs
Main AMs
Main AMs
Main AMs
Main AMs
Main AMs
Main AMs
Main AMs
Main AMs
Main AMs
Main AMs
Main AMs
Main AMs
Main AMs
Main AMs
Main AMs
Main AMs
Main AMs

Main AMs
Main AMs
Main AMs
Main AMs
Main AMs
Main AMs
Main AMs
Main AMs
Main AMs
Main AMs
Main AMs
Main AMs
Main AMs
Main AMs
Main AMs
Main AMs
Main AMs
Main AMs
Main AMs
Main AMs
Main AMs
Main AMs
Main AMs
Main AMs
Main AMs
Main AMs
Main AMs
Main AMs
Main AMs
Main AMs
Main AMs
Main AMs
Main AMs
Main AMs
Main AMs
Main AMs
Main AMs
Main AMs
Main AMs
Main AMs
Main AMs
Main AMs
Main AMs
Main AMs
Main AMs
Main AMs
Main AMs
Main AMs
Main AMs
Main AMs
Main AMs
Main AMs

Main AMs
Main AMs
Main AMs
Main AMs
Main AMs
Main AMs
Main AMs
Main AMs
Main AMs
Main AMs
Main AMs
Main AMs
Main AMs
Main AMs
Main AMs
Main AMs
Main AMs
Main AMs
Main AMs
Main AMs
Main AMs
Main AMs
Main AMs
Main AMs
Main AMs
Main AMs
Main AMs
Main AMs
Main AMs
Main AMs
Main AMs
Main AMs
Main AMs
Main AMs
Main AMs
Main AMs
Main AMs
Main AMs
Main AMs
Main AMs
Main AMs
Main AMs
Main AMs
Main AMs
Main AMs
Main AMs
Main AMs
Main AMs
Main AMs
Main AMs
Main AMs
Main AMs

Main AMs
Main AMs
Main AMs
Main AMs
Main AMs
Main AMs
Main AMs
Main AMs
Main AMs
Main AMs
Main AMs
Main AMs
Main AMs
Main AMs
Main AMs
Main AMs
Main AMs
Main AMs
Main AMs
Main AMs
Main AMs
Main AMs
Main AMs
Main AMs
Main AMs
Main AMs
Main AMs
Main AMs
Main AMs
Main AMs
Main AMs
Main AMs
Main AMs
Main AMs
Main AMs
Main AMs
Main AMs
Main AMs
Main AMs
Main AMs
Main AMs
Main AMs
Main AMs
Main AMs
Main AMs
Main AMs
Main AMs
Main AMs
Main AMs
Main AMs
Main AMs
Main AMs

Main AMs
Main AMs
Main AMs
Main AMs
Main AMs
Main AMs
Main AMs
Main AMs
Main AMs
Main AMs
Main AMs
Main AMs
Main AMs
Main AMs
Main AMs
Main AMs
Main AMs
Main AMs
Main AMs
Main AMs
Main AMs
Main AMs
Main AMs
Main AMs
Main AMs
Main AMs
Main AMs
Main AMs
Main AMs
Main AMs
Main AMs
Main AMs
Main AMs
Main AMs
Main AMs
Main AMs
Main AMs
Main AMs
Main AMs
Main AMs
Main AMs
Main AMs
Main AMs
Main AMs
Main AMs
Main AMs
Main AMs
Main AMs
Main AMs
Main AMs
Main AMs
Main AMs

Main AMs
Main AMs
Main AMs
Main AMs
Main AMs
Main AMs
Main AMs
Main AMs
Main AMs
Main AMs
Main AMs
Main AMs
Main AMs
Main AMs
Main AMs
Main AMs
Main AMs
Main AMs
Main AMs
Main AMs
Main AMs
Main AMs
Main AMs
Main AMs
Main AMs
Main AMs
Main AMs
Main AMs
Main AMs
Main AMs
Main AMs
Main AMs
Main AMs
Main AMs
Main AMs
Main AMs
Main AMs
Main AMs
Main AMs
Main AMs
Main AMs
Main AMs
Main AMs
Main AMs
Main AMs
Main AMs
Main AMs
Main AMs
Main AMs
Main AMs
Main AMs
Main AMs

Main AMs
Main AMs
Main AMs
Main AMs
Main AMs
Main AMs
Main AMs
Main AMs
Main AMs
Main AMs
Main AMs
Main AMs
Main AMs
Main AMs
Main AMs
Main AMs
Main AMs
Main AMs
Main AMs
Main AMs
Main AMs
Main AMs
Main AMs
Main AMs
Main AMs
Main AMs
Main AMs
Main AMs
Main AMs
Main AMs
Main AMs
Main AMs
Main AMs
Main AMs
Main AMs
Main AMs
Main AMs
Main AMs
Main AMs
Main AMs
Main AMs
Main AMs
Main AMs
Main AMs
Main AMs
Main AMs
Main AMs
Main AMs
Main AMs
Main AMs
Main AMs
Main AMs

Main AMs
Main AMs
Main AMs
Main AMs
Main AMs
Main AMs
Main AMs
Main AMs
Main AMs
Main AMs
Main AMs
Main AMs
Main AMs
Main AMs
Main AMs
Main AMs
Main AMs
Main AMs
Main AMs
Main AMs
Main AMs
Main AMs
Main AMs
Main AMs
Main AMs
Main AMs
Main AMs
Main AMs
Main AMs
Main AMs
Main AMs
Main AMs
Main AMs
Main AMs
Main AMs
Main AMs
Main AMs
Main AMs
Main AMs
Main AMs
Main AMs
Main AMs
Main AMs
Main AMs
Main AMs
Main AMs
Main AMs
Main AMs
Main AMs
Main AMs
Main AMs
Main AMs

Main AMs
Main AMs
Main AMs
Main AMs
Main AMs
Main AMs
Main AMs
Main AMs
Main AMs
Main AMs
Main AMs
Main AMs
Main AMs
Main AMs
Main AMs
Main AMs
Main AMs
Main AMs
Main AMs
Main AMs
Main AMs
Main AMs
Main AMs
Main AMs
Main AMs
Main AMs
Main AMs
Main AMs
Main AMs
Main AMs
Main AMs
Main AMs
Main AMs
Main AMs
Main AMs
Main AMs
Main AMs
Main AMs
Main AMs
Main AMs
Main AMs
Main AMs
Main AMs
Main AMs
Main AMs
Main AMs
Main AMs
Main AMs
Main AMs
Main AMs
Main AMs
Main AMs

Main AMs
Main AMs
Main AMs
Main AMs
Main AMs
Main AMs
Main AMs
Main AMs
Main AMs
Main AMs
Main AMs
Main AMs
Main AMs
Main AMs
Main AMs
Main AMs
Main AMs
Main AMs
Main AMs
Main AMs
Main AMs
Main AMs
Main AMs
Main AMs
Main AMs
Main AMs
Main AMs
Main AMs
Main AMs
Main AMs
Main AMs
Main AMs
Main AMs
Main AMs
Main AMs
Main AMs
Main AMs
Main AMs
Main AMs
Main AMs
Main AMs
Main AMs
Main AMs
Main AMs
Main AMs
Main AMs
Main AMs
Main AMs
Main AMs
Main AMs
Main AMs
Main AMs

Main AMs
Main AMs
Main AMs
Main AMs
Main AMs
Main AMs
Main AMs
Main AMs
Main AMs
Main AMs
Main AMs
Main AMs
Main AMs
Main AMs
Main AMs
Main AMs
Main AMs
Main AMs
Main AMs
Main AMs
Main AMs
Main AMs
Main AMs
Main AMs
Main AMs
Main AMs
Main AMs
Main AMs
Main AMs
Main AMs
Main AMs
Main AMs
Main AMs
Main AMs
Main AMs
Main AMs
Main AMs
Main AMs
Main AMs
Main AMs
Main AMs
Main AMs
Main AMs
Main AMs
Main AMs
Main AMs
Main AMs
Main AMs
Main AMs
Main AMs
Main AMs
Main AMs

Main AMs
Main AMs
Main AMs
Main AMs
Main AMs
Main AMs
Main AMs
Main AMs
Main AMs
Main AMs
Main AMs
Main AMs
Main AMs
Main AMs
Main AMs
Main AMs
Main AMs
Main AMs
Main AMs
Main AMs
Main AMs
Main AMs
Main AMs
Main AMs
Main AMs
Main AMs
Main AMs
Main AMs
Main AMs
Main AMs
Main AMs
Main AMs
Main AMs
Main AMs
Main AMs
Main AMs
Main AMs
Main AMs
Main AMs
Main AMs
Main AMs
Main AMs
Main AMs
Main AMs
Main AMs
Main AMs
Main AMs
Main AMs
Main AMs
Main AMs
Main AMs
Main AMs

Main AMs
Main AMs
Main AMs
Main AMs
Main AMs
Main AMs
Main AMs
Main AMs
Main AMs
Main AMs
Main AMs
Main AMs
Main AMs
Main AMs
Main AMs
Main AMs
Main AMs
Main AMs
Main AMs
Main AMs
Main AMs
Main AMs
Main AMs
Main AMs
Main AMs
Main AMs
Main AMs
Main AMs
Main AMs
Main AMs
Main AMs
Main AMs
Main AMs
Main AMs
Main AMs
Main AMs
Main AMs
Main AMs
Main AMs
Main AMs
Main AMs
Main AMs
Main AMs
Main AMs
Main AMs
Main AMs
Main AMs
Main AMs
Main AMs
Main AMs
Main AMs
Main AMs

Main AMs
Main AMs
Main AMs
Main AMs
Main AMs
Main AMs
Main AMs
Main AMs
Main AMs
Main AMs
Main AMs
Main AMs
Main AMs
Main AMs
Main AMs
Main AMs
Main AMs
Main AMs
Main AMs
Main AMs
Main AMs
Main AMs
Main AMs
Main AMs
Main AMs
Main AMs
Main AMs
Main AMs
Main AMs
Main AMs
Main AMs
Main AMs
Main AMs
Main AMs
Main AMs
Main AMs
Main AMs
Main AMs
Main AMs
Main AMs
Main AMs
Main AMs
Main AMs
Main AMs
Main AMs
Main AMs
Main AMs
Main AMs
Main AMs
Main AMs
Main AMs
Main AMs

Main AMs
Main AMs
Main AMs
Main AMs
Main AMs
Main AMs
Main AMs
Main AMs
Main AMs
Main AMs
Main AMs
Main AMs
Main AMs
Main AMs
Main AMs
Main AMs
Main AMs
Main AMs
Main AMs
Main AMs
Main AMs
Main AMs
Main AMs
Main AMs
Main AMs
Main AMs
Main AMs
Main AMs
Main AMs
Main AMs
Main AMs
Main AMs
Main AMs
Main AMs
Main AMs
Main AMs
Main AMs
Main AMs
Main AMs
Main AMs
Main AMs
Main AMs
Main AMs
Main AMs
Main AMs
Main AMs
Main AMs
Main AMs
Main AMs
Main AMs
Main AMs
Main AMs

Main AMs
Main AMs
Main AMs
Main AMs
Main AMs
Main AMs
Main AMs
Main AMs
Main AMs
Main AMs
Main AMs
Main AMs
Main AMs
Main AMs
Main AMs
Main AMs
Main AMs
Main AMs
Main AMs
Main AMs
Main AMs
Main AMs
Main AMs
Main AMs
Main AMs
Main AMs
Main AMs
Main AMs
Main AMs
Main AMs
Main AMs
Main AMs
Main AMs
Main AMs
Main AMs
Main AMs
Main AMs
Main AMs
Main AMs
Main AMs
Main AMs
Main AMs
Main AMs
Main AMs
Main AMs
Main AMs
Main AMs
Main AMs
Main AMs
Main AMs
Main AMs
Main AMs

Main AMs
Main AMs
Main AMs
Main AMs
Main AMs
Main AMs
Main AMs
Main AMs
Main AMs
Main AMs
Main AMs
Main AMs
Main AMs
Main AMs
Main AMs
Main AMs
Main AMs
Main AMs
Main AMs
Main AMs
Main AMs
Main AMs
Main AMs
Main AMs
Main AMs
Main AMs
Main AMs
Main AMs
Main AMs
Main AMs
Main AMs
Main AMs
Main AMs
Main AMs
Main AMs
Main AMs
Main AMs
Main AMs
Main AMs
Main AMs
Main AMs
Main AMs
Main AMs
Main AMs
Main AMs
Main AMs
Main AMs
Main AMs
Main AMs
Main AMs
Main AMs
Main AMs

Main AMs
Main AMs
Main AMs
Main AMs
Main AMs
Main AMs
Main AMs
Main AMs
Main AMs
Main AMs
Main AMs
Main AMs
Main AMs
Main AMs
Main AMs
Main AMs
Main AMs
Main AMs
Main AMs
Main AMs
Main AMs
Main AMs
Main AMs
Main AMs
Main AMs
Main AMs
Main AMs
Main AMs
Main AMs
Main AMs
Main AMs
Main AMs
Main AMs
Main AMs
Main AMs
Main AMs
Main AMs
Main AMs
Main AMs
Main AMs
Main AMs
Main AMs
Main AMs
Main AMs
Main AMs
Main AMs
Main AMs
Main AMs
Main AMs
Main AMs
Main AMs
Main AMs

Main AMs
Main AMs
Main AMs
Main AMs
Main AMs
Main AMs
Main AMs
Main AMs
Main AMs
Main AMs
Main AMs
Main AMs
Main AMs
Main AMs
Main AMs
Main AMs
Main AMs
Main AMs
Main AMs
Main AMs
Main AMs
Main AMs
Main AMs
Main AMs
Main AMs
Main AMs
Main AMs
Main AMs
Main AMs
Main AMs
Main AMs
Main AMs
Main AMs
Main AMs
Main AMs
Main AMs
Main AMs
Main AMs
Main AMs
Main AMs
Main AMs
Main AMs
Main AMs
Main AMs
Main AMs
Main AMs
Main AMs
Main AMs
Main AMs
Main AMs
Main AMs
Main AMs

Main AMs
Main AMs
Main AMs
Main AMs
Main AMs
Main AMs
Main AMs
Main AMs
Main AMs
Main AMs
Main AMs
Main AMs
Main AMs
Main AMs
Main AMs
Main AMs
Main AMs
Main AMs
Main AMs
Main AMs
Main AMs
Main AMs
Main AMs
Main AMs
Main AMs
Main AMs
Main AMs
Main AMs
Main AMs
Main AMs
Main AMs
Main AMs
Main AMs
Main AMs
Main AMs
Main AMs
Main AMs
Main AMs
Main AMs
Main AMs
Main AMs
Main AMs
Main AMs
Main AMs
Main AMs
Main AMs
Main AMs
Main AMs
Main AMs
Main AMs
Main AMs
Main AMs

Main AMs
Main AMs
Main AMs
Main AMs
Main AMs
Main AMs
Main AMs
Main AMs
Main AMs
Main AMs
Main AMs
Main AMs
Main AMs
Main AMs
Main AMs
Main AMs
Main AMs
Main AMs
Main AMs
Main AMs
Main AMs
Main AMs
Main AMs
Main AMs
Main AMs
Main AMs
Main AMs
Main AMs
Main AMs
Main AMs
Main AMs
Main AMs
Main AMs
Main AMs
Main AMs
Main AMs
Main AMs
Main AMs
Main AMs
Main AMs
Main AMs
Main AMs
Main AMs
Main AMs
Main AMs
Main AMs
Main AMs
Main AMs
Main AMs
Main AMs
Main AMs
Main AMs

Main AMs
Main AMs
Main AMs
Main AMs
Main AMs
Main AMs
Main AMs
Main AMs
Main AMs
Main AMs
Main AMs
Main AMs
Main AMs
Main AMs
Main AMs
Main AMs
Main AMs
Main AMs
Main AMs
Main AMs
Main AMs
Main AMs
Main AMs
Main AMs
Main AMs
Main AMs
Main AMs
Main AMs
Main AMs
Main AMs
Main AMs
Main AMs
Main AMs
Main AMs
Main AMs
Main AMs
Main AMs
Main AMs
Main AMs
Main AMs
Main AMs
Main AMs
Main AMs
Main AMs
Main AMs
Main AMs
Main AMs
Main AMs
Main AMs
Main AMs
Main AMs
Main AMs

Main AMs
Main AMs
Main AMs
Main AMs
Main AMs
Main AMs
Main AMs
Main AMs
Main AMs
Main AMs
Main AMs
Main AMs
Main AMs
Main AMs
Main AMs
Main AMs
Main AMs
Main AMs
Main AMs
Main AMs
Main AMs
Main AMs
Main AMs
Main AMs
Main AMs
Main AMs
Main AMs
Main AMs
Main AMs
Main AMs
Main AMs
Main AMs
Main AMs
Main AMs
Main AMs
Main AMs
Main AMs
Main AMs
Main AMs
Main AMs
Main AMs
Main AMs
Main AMs
Main AMs
Main AMs
Main AMs
Main AMs
Main AMs
Main AMs
Main AMs
Main AMs
Main AMs

Main AMs
Main AMs
Main AMs
Main AMs
Main AMs
Main AMs
Main AMs
Main AMs
Main AMs
Main AMs
Main AMs
Main AMs
Main AMs
Main AMs
Main AMs
Main AMs
Main AMs
Main AMs
Main AMs
Main AMs
Main AMs
Main AMs
Main AMs
Main AMs
Main AMs
Main AMs
Main AMs
Main AMs
Main AMs
Main AMs
Main AMs
Main AMs
Main AMs
Main AMs
Main AMs
Main AMs
Main AMs
Main AMs
Main AMs
Main AMs
Main AMs
Main AMs
Main AMs
Main AMs
Main AMs
Main AMs
Main AMs
Main AMs
Main AMs
Main AMs
Main AMs
Main AMs

Main AMs
Main AMs
Main AMs
Main AMs
Main AMs
Main AMs
Main AMs
Main AMs
Main AMs
Main AMs
Main AMs
Main AMs
Main AMs
Main AMs
Main AMs
Main AMs
Main AMs
Main AMs
Main AMs
Main AMs
Main AMs
Main AMs
Main AMs
Main AMs
Main AMs
Main AMs
Main AMs
Main AMs
Main AMs
Main AMs
Main AMs
Main AMs
Main AMs
Main AMs
Main AMs
Main AMs
Main AMs
Main AMs
Main AMs
Main AMs
Main AMs
Main AMs
Main AMs
Main AMs
Main AMs
Main AMs
Main AMs
Main AMs
Main AMs
Main AMs
Main AMs
Main AMs

Main AMs
Main AMs
Main AMs
Main AMs
Main AMs
Main AMs
Main AMs
Main AMs
Main AMs
Main AMs
Main AMs
Main AMs
Main AMs
Main AMs
Main AMs
Main AMs
Main AMs
Main AMs
Main AMs
Main AMs
Main AMs
Main AMs
Main AMs
Main AMs
Main AMs
Main AMs
Main AMs
Main AMs
Main AMs
Main AMs
Main AMs
Main AMs
Main AMs
Main AMs
Main AMs
Main AMs
Main AMs
Main AMs
Main AMs
Main AMs
Main AMs
Main AMs
Main AMs
Main AMs
Main AMs
Main AMs
Main AMs
Main AMs
Main AMs
Main AMs
Main AMs
Main AMs

Main AMs
Main AMs
Main AMs
Main AMs
Main AMs
Main AMs
Main AMs
Main AMs
Main AMs
Main AMs
Main AMs
Main AMs
Main AMs
Main AMs
Main AMs
Main AMs
Main AMs
Main AMs
Main AMs
Main AMs
Main AMs
Main AMs
Main AMs
Main AMs
Main AMs
Main AMs
Main AMs
Main AMs
Main AMs
Main AMs
Main AMs
Main AMs
Main AMs
Main AMs
Main AMs
Main AMs
Main AMs
Main AMs
Main AMs
Main AMs
Main AMs
Main AMs
Main AMs
Main AMs
Main AMs
Main AMs
Main AMs
Main AMs
Main AMs
Main AMs
Main AMs
Main AMs

Main AMs
Main AMs
Main AMs
Main AMs
Main AMs
Main AMs
Main AMs
Main AMs
Main AMs
Main AMs
Main AMs
Main AMs
Main AMs
Main AMs
Main AMs
Main AMs
Main AMs
Main AMs
Main AMs
Main AMs
Main AMs
Main AMs
Main AMs
Main AMs
Main AMs
Main AMs
Main AMs
Main AMs
Main AMs
Main AMs
Main AMs
Main AMs
Main AMs
Main AMs
Main AMs
Main AMs
Main AMs
Main AMs
Main AMs
Main AMs
Main AMs
Main AMs
Main AMs
Main AMs
Main AMs
Main AMs
Main AMs
Main AMs
Main AMs
Main AMs
Main AMs
Main AMs

Main AMs
Main AMs
Main AMs
Main AMs
Main AMs
Main AMs
Main AMs
Main AMs
Main AMs
Main AMs
Main AMs
Main AMs
Main AMs
Main AMs
Main AMs
Main AMs
Main AMs
Main AMs
Main AMs
Main AMs
Main AMs
Main AMs
Main AMs
Main AMs
Main AMs
Main AMs
Main AMs
Main AMs
Main AMs
Main AMs
Main AMs
Main AMs
Main AMs
Main AMs
Main AMs
Main AMs
Main AMs
Main AMs
Main AMs
Main AMs
Main AMs
Main AMs
Main AMs
Main AMs
Main AMs
Main AMs
Main AMs
Main AMs
Main AMs
Main AMs
Main AMs
Main AMs

Main AMs
Main AMs
Main AMs
Main AMs
Main AMs
Main AMs
Main AMs
Main AMs
Main AMs
Main AMs
Main AMs
Main AMs
Main AMs
Main AMs
Main AMs
Main AMs
Main AMs
Main AMs
Main AMs
Main AMs
Main AMs
Main AMs
Main AMs
Main AMs
Main AMs
Main AMs
Main AMs
Main AMs
Main AMs
Main AMs
Main AMs
Main AMs
Main AMs
Main AMs
Main AMs
Main AMs
Main AMs
Main AMs
Main AMs
Main AMs
Main AMs
Main AMs
Main AMs
Main AMs
Main AMs
Main AMs
Main AMs
Main AMs
Main AMs
Main AMs
Main AMs
Main AMs

Main AMs
Main AMs
Main AMs
Main AMs
Main AMs
Main AMs
Main AMs
Main AMs
Main AMs
Main AMs
Main AMs
Main AMs
Main AMs
Main AMs
Main AMs
Main AMs
Main AMs
Main AMs
Main AMs
Main AMs
Main AMs
Main AMs
Main AMs
Main AMs
Main AMs
Main AMs
Main AMs
Main AMs
Main AMs
Main AMs
Main AMs
Main AMs
Main AMs
Main AMs
Main AMs
Main AMs
Main AMs
Main AMs
Main AMs
Main AMs
Main AMs
Main AMs
Main AMs
Main AMs
Main AMs
Main AMs
Main AMs
Main AMs
Main AMs
Main AMs
Main AMs
Main AMs

Main AMs
Main AMs
Main AMs
Main AMs
Main AMs
Main AMs
Main AMs
Main AMs
Main AMs
Main AMs
Main AMs
Main AMs
Main AMs
Main AMs
Main AMs
Main AMs
Main AMs
Main AMs
Main AMs
Main AMs
Main AMs
Main AMs
Main AMs
Main AMs
Main AMs
Main AMs
Main AMs
Main AMs
Main AMs
Main AMs
Main AMs
Main AMs
Main AMs
Main AMs
Main AMs
Main AMs
Main AMs
Main AMs
Main AMs
Main AMs
Main AMs
Main AMs
Main AMs
Main AMs
Main AMs
Main AMs
Main AMs
Main AMs
Main AMs
Main AMs
Main AMs
Main AMs

Main AMs
Main AMs
Main AMs
Main AMs
Main AMs
Main AMs
Main AMs
Main AMs
Main AMs
Main AMs
Main AMs
Main AMs
Main AMs
Main AMs
Main AMs
Main AMs
Main AMs
Main AMs
Main AMs
Main AMs
Main AMs
Main AMs
Main AMs
Main AMs
Main AMs
Main AMs
Main AMs
Main AMs
Main AMs
Main AMs
Main AMs
Main AMs
Main AMs
Main AMs
Main AMs
Main AMs
Main AMs
Main AMs
Main AMs
Main AMs
Main AMs
Main AMs
Main AMs
Main AMs
Main AMs
Main AMs
Main AMs
Main AMs
Main AMs
Main AMs
Main AMs
Main AMs

Main AMs
Main AMs
Main AMs
Main AMs
Main AMs
Main AMs
Main AMs
Main AMs
Main AMs
Main AMs
Main AMs
Main AMs
Main AMs
Main AMs
Main AMs
Main AMs
Main AMs
Main AMs
Main AMs
Main AMs
Main AMs
Main AMs
Main AMs
Main AMs
Main AMs
Main AMs
Main AMs
Main AMs
Main AMs
Main AMs
Main AMs
Main AMs
Main AMs
Main AMs
Main AMs
Main AMs
