# Supplementary material for: Alveolar macrophage subtypes express cholesterol and inflammation genes in cystic fibrosis
Source: Life Sci Alliance. 2026 Apr 30;9(7):e202503482. doi: 10.26508/lsa.202503482 (PMC13139742; doi:10.26508/lsa.202503482)
Supplement: Supplementary file 2 [file LSA-2025-03482_TableS2.pdf]

Supplemental Data: DEGs shown in Figure 3A

	p_val	avg_log2FC	pct.1	pct.2	p_val_adj	cluster
ITIH5	0	1.25944105	0.757	0.379	0	AMs
GPD1	0	1.03692139	0.925	0.565	0	AMs
SLC19A3	0	1.11812462	0.768	0.411	0	AMs
MLPH	0	0.86641921	0.881	0.548	0	AMs
CPE	0	0.87337917	0.838	0.507	0	AMs
C8B	0	0.92824774	0.706	0.388	0	AMs
PCOLCE2	0	0.90141747	0.932	0.623	0	AMs
SLC47A1	0	0.7268709	0.932	0.629	0	AMs
ITGB5	0	0.70746303	0.84	0.542	0	AMs
MGAT3	0	0.77036762	0.791	0.502	0	AMs
PPIC	0	0.78250318	0.717	0.429	0	AMs
LSAMP	0	0.77409741	0.755	0.47	0	AMs
TRHDE	0	1.23595534	0.521	0.239	0	AMs
MYO6	0	0.74831048	0.753	0.474	0	AMs
ACO1	0	0.5629956	0.914	0.64	0	AMs
MME	0	0.64759167	0.966	0.697	0	AMs
ADTRP	0	0.72817199	0.816	0.547	0	AMs
FGD5	0	0.64711094	0.83	0.562	0	AMs
RBP4	0	0.96153263	0.784	0.52	0	AMs
GLDN	0	0.6755441	0.816	0.553	0	AMs
ADGRE1	0	0.8346381	0.649	0.387	0	AMs
FAM89A	0	0.57968615	0.928	0.682	0	AMs
PHLDA3	0	0.60863829	0.874	0.629	0	AMs
MGLL	0	0.56028125	0.817	0.573	0	AMs
KCNAB1	0	0.94263169	0.582	0.338	0	AMs
CD83	0	0.59676242	0.92	0.679	0	AMs
KMO	0	0.86848235	0.636	0.396	0	AMs
MACC1	0	0.63452031	0.775	0.536	0	AMs
ITGB8	0	0.77698453	0.667	0.43	0	AMs
ATP10A	0	0.54789415	0.736	0.499	0	AMs
TRPC6	0	0.84199356	0.523	0.289	0	AMs
TGM2	0	0.65332475	0.91	0.676	0	AMs
ANOS1	0	0.48342242	0.787	0.553	0	AMs
SGPP1	0	0.49967181	0.875	0.643	0	AMs
C2	0	0.54705138	0.806	0.576	0	AMs
EPB41L1	0	0.53360874	0.756	0.527	0	AMs
CPED1	0	0.61075623	0.865	0.639	0	AMs
UBASH3B	0	0.43226605	0.885	0.66	0	AMs
SCIMP	0	0.47621481	0.867	0.642	0	AMs
SLC44A2	0	0.32640631	0.807	0.583	0	AMs
OVCH1	0	0.8900384	0.564	0.34	0	AMs
HPGD	0	0.43602061	0.759	0.536	0	AMs
ANGPT4	0	0.8586323	0.534	0.312	0	AMs
SLC9A1	0	0.50140315	0.843	0.621	0	AMs
EPHX1	0	0.43757675	0.842	0.62	0	AMs
LIMA1	0	0.50626911	0.781	0.559	0	AMs
IRS2	0	0.5786884	0.949	0.727	0	AMs
AKR1B1	0	0.63185454	0.912	0.691	0	AMs

FAM3B	0	1.0530148	0.449	0.228	0	AMs
BHLHE41	0	0.58654935	0.915	0.696	0	AMs
TANC2	0	0.43706619	0.884	0.665	0	AMs
PAQR5	0	0.5295797	0.649	0.432	0	AMs
DTX4	0	0.66559308	0.609	0.392	0	AMs
NUPR1	0	0.75124102	0.881	0.664	0	AMs
PILRA	0	0.47371259	0.914	0.698	0	AMs
G6PD	0	0.4619462	0.91	0.694	0	AMs
FMNL2	0	0.463194	0.911	0.696	0	AMs
ALDH1A1	0	0.50467716	0.949	0.734	0	AMs
PLAAT3	0	0.45625287	0.897	0.682	0	AMs
PDLIM1	0	0.39394308	0.905	0.692	0	AMs
HVCN1	0	0.49633675	0.842	0.629	0	AMs
WARS	0	0.56798871	0.931	0.719	0	AMs
APOL1	0	0.4531141	0.784	0.572	0	AMs
CD101	0	0.66063351	0.604	0.392	0	AMs
GALNT12	0	0.52503722	0.58	0.368	0	AMs
CFD	0	0.57893812	0.89	0.678	0	AMs
SIRPB1	0	0.43953284	0.87	0.658	0	AMs
ACKR3	0	0.40804856	0.897	0.686	0	AMs
ENPP4	0	0.46427338	0.887	0.676	0	AMs
S100A13	0	0.44501207	0.93	0.72	0	AMs
HK2	0	0.45699552	0.888	0.678	0	AMs
SVIL	0	0.41788526	0.951	0.742	0	AMs
ATP1B1	0	0.43255239	0.937	0.729	0	AMs
ST6GALNAC6	0	0.28944085	0.81	0.602	0	AMs
BCL2A1	0	0.44317425	0.904	0.696	0	AMs
HOXB6	0	1.15033511	0.414	0.206	0	AMs
SLC7A8	0	0.70501859	0.552	0.345	0	AMs
ALDH3A2	0	0.35403355	0.919	0.712	0	AMs
BCAR1	0	0.60200973	0.624	0.417	0	AMs
RASAL2	0	0.50910554	0.756	0.549	0	AMs
KIF16B	0	0.45837487	0.727	0.52	0	AMs
PI4K2A	0	0.46554303	0.811	0.605	0	AMs
CDC42BPB	0	0.38085638	0.862	0.656	0	AMs
OPN3	0	0.48184021	0.777	0.572	0	AMs
PDLIM2	0	0.26730569	0.803	0.598	0	AMs
CORO2A	0	0.38733132	0.82	0.615	0	AMs
FUCA1	0	0.39408565	0.905	0.701	0	AMs
CYB5R1	0	0.40661745	0.793	0.589	0	AMs
FRMD4A	0	0.34858672	0.917	0.713	0	AMs
TCF7L2	0	0.41934604	0.948	0.744	0	AMs
ST3GAL2	0	0.39090939	0.837	0.633	0	AMs
AXL	0	0.35078803	0.932	0.729	0	AMs
TTC7B	0	0.48441417	0.725	0.522	0	AMs
ALAS1	0	0.50577864	0.915	0.713	0	AMs
EPAS1	0	0.37874885	0.949	0.747	0	AMs
DPEP2	0	0.42484812	0.83	0.628	0	AMs
HBEGF	0	0.50500611	0.934	0.733	0	AMs
ABHD5	0	0.37472682	0.882	0.681	0	AMs
MITF	0	0.43296078	0.823	0.622	0	AMs
HK3	0	0.48118345	0.951	0.75	0	AMs

TSPAN3	0	0.57809987	0.97	0.769	0	AMs
KCNE3	0	0.44192047	0.805	0.605	0	AMs
AMIGO2	0	0.34420339	0.925	0.725	0	AMs
DDAH2	0	0.40720618	0.886	0.686	0	AMs
LIMK1	0	0.3893073	0.89	0.691	0	AMs
RMDN3	0	0.33744377	0.902	0.703	0	AMs
FIG4	0	0.39119401	0.87	0.671	0	AMs
INHBA	0	0.6331304	0.964	0.765	0	AMs
CD163	0	0.38333672	0.955	0.756	0	AMs
LILRA5	0	0.64326095	0.599	0.4	0	AMs
SRC	0	0.39177411	0.836	0.637	0	AMs
DST	0	0.38789595	0.762	0.564	0	AMs
SLC43A2	0	0.31668905	0.887	0.689	0	AMs
MSRA	0	0.34452401	0.879	0.681	0	AMs
IFIT2	0	0.48465475	0.824	0.626	0	AMs
EDEM2	0	0.37244098	0.941	0.743	0	AMs
PYGL	0	0.43861831	0.778	0.581	0	AMs
STON1	0	0.80735301	0.497	0.3	0	AMs
ACSS2	0	0.52700285	0.662	0.465	0	AMs
SIK2	0	0.37873498	0.747	0.55	0	AMs
PLA2G15	0	0.37496383	0.863	0.666	0	AMs
ECSCR	0	0.5125661	0.668	0.472	0	AMs
TNFAIP2	0	0.43438423	0.885	0.689	0	AMs
NAMPT	0	0.26309081	0.857	0.661	0	AMs
CD300LF	0	0.38641201	0.938	0.742	0	AMs
TMEM184B	0	0.3006316	0.835	0.639	0	AMs
BTK	0	0.39492715	0.869	0.673	0	AMs
STX12	0	0.36849888	0.91	0.715	0	AMs
KLHL6	0	0.36569789	0.907	0.712	0	AMs
LY86	0	0.4883367	0.93	0.735	0	AMs
RRBP1	0	0.41720064	0.79	0.595	0	AMs
GNG12	0	0.89742765	0.414	0.219	0	AMs
DHCR24	0	0.52262881	0.705	0.51	0	AMs
MMP24OS	0	0.33202885	0.857	0.662	0	AMs
GM2A	0	0.31616933	0.931	0.737	0	AMs
AVPI1	0	0.34947371	0.936	0.742	0	AMs
TXNRD2	0	0.3505878	0.804	0.61	0	AMs
RND3	0	1.27592065	0.344	0.15	0	AMs
CCDC115	0	0.33716559	0.728	0.534	0	AMs
MYOF	0	0.37030837	0.868	0.674	0	AMs
ARRB1	0	0.34800286	0.902	0.709	0	AMs
RTN1	0	0.39392054	0.811	0.618	0	AMs
HSD3B7	0	0.39465943	0.784	0.591	0	AMs
C7orf50	0	0.35270075	0.743	0.55	0	AMs
SCPEP1	0	0.43343325	0.947	0.754	0	AMs
DENND5A	0	0.34089049	0.923	0.731	0	AMs
MS4A4A	0	0.23167826	0.934	0.742	0	AMs
IQSEC3	0	0.72598957	0.501	0.309	0	AMs
NFU1	0	0.32821263	0.711	0.519	0	AMs
PDCD1LG2	0	0.61167072	0.568	0.376	0	AMs
SLCO2B1	0	0.40908362	0.988	0.796	0	AMs
ACOX1	0	0.29364875	0.87	0.678	0	AMs

PKN2	0	0.35827589	0.764	0.573	0	AMs
MMD	0	0.21464892	0.791	0.6	0	AMs
IQSEC2	0	0.38222187	0.778	0.587	0	AMs
RSPO3	0	1.55027188	0.316	0.125	0	AMs
IL1RN	0	0.51599053	0.736	0.545	0	AMs
CYP27A1	0	0.53286529	0.991	0.8	0	AMs
SMIM14	0	0.30987263	0.816	0.625	0	AMs
ANKS1A	0	0.38055553	0.727	0.536	0	AMs
ITGAX	0	0.273103	0.838	0.647	0	AMs
ADAM17	0	0.29435971	0.918	0.728	0	AMs
CD40	0	0.4617098	0.676	0.486	0	AMs
CLIP4	0	0.27668384	0.859	0.669	0	AMs
AZI2	0	0.38275426	0.744	0.554	0	AMs
PHACTR1	0	0.31577578	0.865	0.675	0	AMs
OASL	0	0.35585054	0.827	0.637	0	AMs
TMEM150B	0	0.39805511	0.749	0.559	0	AMs
STK40	0	0.26501552	0.877	0.688	0	AMs
SLC31A1	0	0.3050997	0.906	0.717	0	AMs
PEPD	0	0.32223375	0.907	0.718	0	AMs
APOC1	0	0.40231451	0.907	0.718	0	AMs
TBC1D4	0	0.37611439	0.604	0.415	0	AMs
FAM162A	0	0.31773084	0.72	0.531	0	AMs
SERPINB6	0	0.38156725	0.944	0.755	0	AMs
BEX4	0	0.41442206	0.743	0.554	0	AMs
GSAP	0	0.37603639	0.783	0.595	0	AMs
IFIT3	0	0.39355302	0.89	0.702	0	AMs
CD276	0	0.3911084	0.775	0.587	0	AMs
SPINT2	0	0.32843838	0.902	0.714	0	AMs
AKAP5	0	0.47369166	0.617	0.429	0	AMs
PEX19	0	0.30687781	0.845	0.657	0	AMs
TMEM63A	0	0.25055619	0.873	0.685	0	AMs
GPR137B	0	0.2959607	0.82	0.632	0	AMs
UBE2E2	0	0.4094529	0.737	0.549	0	AMs
SEMA3C	0	0.45676826	0.699	0.511	0	AMs
SERPING1	0	0.88936058	0.986	0.798	0	AMs
CALCOCO1	0	0.30460285	0.705	0.517	0	AMs
TRIQK	0	0.48545589	0.674	0.487	0	AMs
CHP1	0	0.32951026	0.878	0.691	0	AMs
CHDH	0	0.57457327	0.551	0.365	0	AMs
PDCL	0	0.30532803	0.792	0.606	0	AMs
CD5L	0	0.66901889	0.608	0.422	0	AMs
RDH10	0	0.4586599	0.62	0.434	0	AMs
ASAH1	0	0.36257582	0.962	0.776	0	AMs
CD151	0	0.30863142	0.954	0.768	0	AMs
BSG	0	0.55534806	0.948	0.762	0	AMs
MCOLN1	0	0.28881327	0.954	0.768	0	AMs
SLC27A3	0	0.26546854	0.894	0.709	0	AMs
HSD17B4	0	0.2667634	0.929	0.744	0	AMs
CES1	0	0.60961395	0.931	0.746	0	AMs
NCEH1	0	0.24823086	0.966	0.781	0	AMs
DLD	0	0.28583437	0.845	0.66	0	AMs
FDFT1	0	0.33161611	0.739	0.554	0	AMs

C9orf16	0	0.27509333	0.843	0.658	0	AMs
CORO1B	0	0.27348292	0.94	0.755	0	AMs
ACVRL1	0	0.42046263	0.718	0.533	0	AMs
CSF2RB	0	0.27478397	0.831	0.646	0	AMs
CSTA	0	0.30974283	0.914	0.73	0	AMs
RNASET2	0	0.25078857	0.787	0.603	0	AMs
BLVRA	0	0.24511603	0.903	0.719	0	AMs
HIPK2	0	0.25272093	0.874	0.69	0	AMs
TALDO1	0	0.27618791	0.895	0.711	0	AMs
OSCAR	0	0.28567705	0.906	0.722	0	AMs
IL1B	0	0.52676291	0.508	0.324	0	AMs
CDC42EP3	0	0.16334205	0.845	0.661	0	AMs
DOK3	0	0.40487859	0.714	0.53	0	AMs
PEBP1	0	0.24228127	0.868	0.684	0	AMs
ARHGEF10L	0	0.30772806	0.9	0.717	0	AMs
PRDX1	0	0.32932691	0.922	0.739	0	AMs
SPIRE1	0	0.41888986	0.667	0.484	0	AMs
DAB2	0	0.31206835	0.969	0.786	0	AMs
ACE	0	0.26381147	0.862	0.679	0	AMs
LGALS3BP	0	0.48327891	0.989	0.806	0	AMs
LST1	0	0.32101466	0.922	0.74	0	AMs
FGD2	0	0.28558236	0.89	0.708	0	AMs
TMEM273	0	0.42618379	0.8	0.618	0	AMs
DRAM1	0	0.36878312	0.787	0.605	0	AMs
ADGRE3	0	0.95634971	0.386	0.204	0	AMs
ARHGAP24	0	0.79561197	0.454	0.272	0	AMs
CTSO	0	0.41888525	0.629	0.447	0	AMs
DAPK1	0	0.32206631	0.859	0.677	0	AMs
RAB20	0	0.42598908	0.721	0.539	0	AMs
INF2	0	0.20068739	0.95	0.768	0	AMs
SECTM1	0	0.45302255	0.738	0.556	0	AMs
ETHE1	0	0.25590674	0.827	0.645	0	AMs
MTPN	0	0.26443811	0.897	0.716	0	AMs
TMEM74B	0	0.69141878	0.545	0.364	0	AMs
SORT1	0	0.33964387	0.944	0.763	0	AMs
CPEB4	0	0.23797377	0.845	0.664	0	AMs
PDE1B	0	0.39856315	0.73	0.549	0	AMs
HMOX1	0	0.41234257	0.969	0.788	0	AMs
FGR	0	0.3412772	0.935	0.755	0	AMs
PLEK	0	0.30614584	0.936	0.756	0	AMs
SIDT2	0	0.330245	0.784	0.604	0	AMs
FCGR1A	0	0.46457265	0.644	0.464	0	AMs
QSOX1	0	0.34131464	0.969	0.789	0	AMs
RHBDD2	0	0.3570785	0.947	0.767	0	AMs
PIK3AP1	0	0.3458551	0.947	0.767	0	AMs
ZBTB4	0	0.14899111	0.82	0.64	0	AMs
SIRPA	0	0.32983093	0.971	0.791	0	AMs
PCBD1	0	0.28024763	0.807	0.628	0	AMs
OAS1	0	0.3084931	0.927	0.748	0	AMs
PYGB	0	0.25818619	0.91	0.731	0	AMs
GRK3	0	0.25678131	0.9	0.721	0	AMs
ZCCHC24	0	0.54602132	0.522	0.343	0	AMs

LMBRD1	0	0.23349736	0.866	0.687	0	AMs
ECHDC1	0	0.24583427	0.835	0.656	0	AMs
POR	0	0.33703875	0.977	0.798	0	AMs
DENND4C	0	0.25606126	0.855	0.676	0	AMs
PGM1	0	0.22942201	0.807	0.629	0	AMs
MAN2B2	0	0.19188443	0.901	0.723	0	AMs
NDST1	0	0.35915556	0.752	0.574	0	AMs
NFIL3	0	0.36278135	0.761	0.583	0	AMs
FFAR4	0	0.34541098	0.757	0.579	0	AMs
BLOC1S2	0	0.2941473	0.753	0.575	0	AMs
VEGFB	0	0.20462372	0.927	0.749	0	AMs
CSF1	0	0.68797946	0.518	0.34	0	AMs
NEIL2	0	0.57613576	0.488	0.31	0	AMs
CA2	0	0.95444042	0.512	0.334	0	AMs
CDCP1	0	0.3016166	0.827	0.649	0	AMs
ARAP1	0	0.31873493	0.968	0.79	0	AMs
PTMS	0	0.21033145	0.965	0.787	0	AMs
P2RX4	0	0.25213756	0.85	0.672	0	AMs
LGALS9	0	0.32151528	0.961	0.783	0	AMs
ABCG1	0	0.46930659	0.99	0.812	0	AMs
HADHB	0	0.26432932	0.884	0.707	0	AMs
NRP2	0	0.46216161	0.78	0.603	0	AMs
SLC11A1	0	0.42832016	0.993	0.816	0	AMs
SLC6A6	0	0.22642793	0.906	0.729	0	AMs
STAC	0	0.36581211	0.749	0.572	0	AMs
OSBPL11	0	0.20855549	0.923	0.746	0	AMs
PRAG1	0	0.52026255	0.543	0.366	0	AMs
FABP5	0	0.23206201	0.876	0.699	0	AMs
PYCARD	0	0.31515302	0.919	0.742	0	AMs
AKR1A1	0	0.21186065	0.946	0.769	0	AMs
SPATS2L	0	0.22186144	0.862	0.685	0	AMs
ADPRH	0	0.37418198	0.689	0.512	0	AMs
BACE1	0	0.31793203	0.738	0.561	0	AMs
NR1H2	0	0.20079044	0.864	0.687	0	AMs
PLXND1	0	0.18650299	0.91	0.734	0	AMs
ACSL1	0	0.35562519	0.868	0.692	0	AMs
ALDH3B1	0	0.28313983	0.771	0.595	0	AMs
DDHD1	0	0.23354509	0.793	0.617	0	AMs
CCPG1	0	0.22906541	0.876	0.7	0	AMs
TNNI2	0	0.64525683	0.504	0.328	0	AMs
SEPTIN4	0	0.87353667	0.422	0.246	0	AMs
CCDC50	0	0.28603829	0.704	0.528	0	AMs
CITED2	0	0.28039504	0.941	0.765	0	AMs
DECR1	0	0.28380003	0.956	0.78	0	AMs
ZFYVE21	0	0.27464019	0.707	0.531	0	AMs
WBP2	0	0.25939593	0.735	0.559	0	AMs
PLEKHO2	0	0.2197271	0.9	0.725	0	AMs
AMFR	0	0.22367259	0.895	0.72	0	AMs
CACNB3	0	0.79191648	0.429	0.254	0	AMs
ZDHHC8	0	0.28137745	0.627	0.452	0	AMs
HDDC2	0	0.17977572	0.862	0.687	0	AMs
STX11	0	0.2874455	0.945	0.77	0	AMs

AGPAT2	0	0.23699534	0.951	0.776	0	AMs
SH3BP5	0	0.16325533	0.919	0.745	0	AMs
RBM47	0	0.23371777	0.901	0.727	0	AMs
JAKMIP2	0	0.62953874	0.457	0.283	0	AMs
PARP4	0	0.24231805	0.927	0.753	0	AMs
ABHD2	0	0.3326951	0.892	0.718	0	AMs
TK2	0	0.25082106	0.754	0.58	0	AMs
NCF4	0	0.20086569	0.93	0.756	0	AMs
PLPP1	0	0.50913968	0.417	0.243	0	AMs
ACAA1	0	0.21225709	0.82	0.646	0	AMs
ATP2C1	0	0.3059084	0.952	0.778	0	AMs
OSTM1	0	0.23086577	0.845	0.671	0	AMs
MPC1	0	0.20014468	0.814	0.64	0	AMs
DCAF5	0	0.17149244	0.736	0.562	0	AMs
ANGEL1	0	0.45826508	0.564	0.39	0	AMs
TLCD2	0	0.55046833	0.564	0.39	0	AMs
BCL2L13	0	0.1972446	0.834	0.66	0	AMs
FCGR2A	0	0.1983923	0.907	0.734	0	AMs
AGPS	0	0.18072159	0.923	0.75	0	AMs
LAT2	0	0.28767852	0.894	0.721	0	AMs
PELI3	0	0.36745731	0.672	0.499	0	AMs
PTPRO	0	0.29208573	0.793	0.62	0	AMs
STAT6	0	0.18628888	0.933	0.76	0	AMs
C5AR1	0	0.29925661	0.987	0.814	0	AMs
NLRC4	0	0.26706795	0.832	0.659	0	AMs
CHMP3	0	0.20472736	0.838	0.665	0	AMs
SKAP2	0	0.23849474	0.844	0.671	0	AMs
CAT	0	0.37559225	0.975	0.802	0	AMs
IGSF6	0	0.32115534	0.838	0.665	0	AMs
VAT1	0	0.35189717	0.977	0.804	0	AMs
ATP6AP2	0	0.2276081	0.859	0.686	0	AMs
GYPC	0	0.23799245	0.934	0.762	0	AMs
NEU1	0	0.21232851	0.764	0.592	0	AMs
TTC39B	0	0.31645507	0.664	0.492	0	AMs
REEP3	0	0.20459265	0.86	0.688	0	AMs
GCHFR	0	0.32119072	0.935	0.763	0	AMs
GNPTG	0	0.17691836	0.911	0.739	0	AMs
TWSG1	0	0.34392693	0.638	0.466	0	AMs
MKNK1	0	0.17030157	0.84	0.668	0	AMs
SLC31A2	0	0.21669846	0.938	0.766	0	AMs
ZNF385A	0	0.20873496	0.857	0.685	0	AMs
IRF8	0	0.13911943	0.979	0.807	0	AMs
PTPN1	0	0.17913337	0.832	0.66	0	AMs
BCAP31	0	0.39300032	0.957	0.785	0	AMs
CLIC4	0	0.2374013	0.933	0.762	0	AMs
RHOQ	0	0.22876445	0.896	0.725	0	AMs
B3GNT7	0	0.36157627	0.674	0.503	0	AMs
DNAJA1	0	0.23937177	0.904	0.733	0	AMs
P4HA1	0	0.24699157	0.76	0.589	0	AMs
FUOM	0	0.24626048	0.791	0.62	0	AMs
POLD4	0	0.21151078	0.915	0.744	0	AMs
MMP19	0	0.39427112	0.676	0.505	0	AMs

LRP1	0	0.3141221	0.996	0.825	0	AMs
JPT1	0	0.2409695	0.667	0.496	0	AMs
TSPAN31	0	0.32437085	0.589	0.418	0	AMs
SFT2D1	0	0.22286603	0.843	0.672	0	AMs
NEK6	0	0.19416186	0.952	0.781	0	AMs
EGR2	0	0.56097996	0.566	0.395	0	AMs
HPS5	0	0.20417623	0.828	0.657	0	AMs
COMMD9	0	0.23340239	0.819	0.648	0	AMs
VASH1	0	0.18127493	0.856	0.685	0	AMs
DDX24	0	0.10017863	0.837	0.666	0	AMs
MTMR10	0	0.24103605	0.726	0.555	0	AMs
TRPV2	0	0.35270579	0.975	0.804	0	AMs
APOE	0	0.23780103	0.974	0.803	0	AMs
RAB7B	0	0.41920675	0.662	0.492	0	AMs
RILPL2	0	0.16751226	0.809	0.639	0	AMs
TM6SF1	0	0.24207588	0.759	0.589	0	AMs
ME1	0	0.63358978	0.488	0.318	0	AMs
SLC35F6	0	0.197232	0.848	0.678	0	AMs
RXRA	0	0.16402432	0.94	0.77	0	AMs
SHTN1	0	0.23211521	0.843	0.673	0	AMs
GPI	0	0.26514016	0.707	0.537	0	AMs
BLCAP	0	0.19812147	0.717	0.547	0	AMs
YPEL5	0	0.12930121	0.885	0.716	0	AMs
GCA	0	0.28602976	0.93	0.761	0	AMs
RNF13	0	0.25621677	0.92	0.751	0	AMs
NUS1	0	0.14891398	0.871	0.702	0	AMs
FABP4	0	0.55916095	0.989	0.82	0	AMs
HK1	0	0.16771869	0.886	0.717	0	AMs
CD82	0	0.18697187	0.803	0.634	0	AMs
MLF2	0	0.21628353	0.909	0.74	0	AMs
THBS1	0	0.33150122	0.885	0.716	0	AMs
TNFSF12	0	0.19112153	0.882	0.713	0	AMs
THBD	0	0.34239453	0.889	0.72	0	AMs
BCL2L1	0	0.10844107	0.798	0.629	0	AMs
ATP6V1E1	0	0.24908984	0.927	0.758	0	AMs
PDGFC	0	0.31357157	0.694	0.525	0	AMs
SNX2	0	0.30977399	0.963	0.794	0	AMs
DMAC1	0	0.24661206	0.705	0.536	0	AMs
NRBF2	0	0.2155184	0.724	0.555	0	AMs
APOBR	0	0.28065243	0.951	0.782	0	AMs
RIPOR1	0	0.21028131	0.837	0.668	0	AMs
APMAP	0	0.14527434	0.832	0.663	0	AMs
PRKCD	0	0.18872303	0.932	0.764	0	AMs
PKD2	0	0.27036304	0.662	0.494	0	AMs
AFDN	0	0.12463009	0.559	0.391	0	AMs
MSR1	0	0.49155372	0.991	0.823	0	AMs
LYN	0	0.21330785	0.86	0.692	0	AMs
SNTB1	0	0.15934044	0.858	0.69	0	AMs
CTNND1	0	0.1413121	0.923	0.755	0	AMs
TAGLN	0	0.39213903	0.801	0.633	0	AMs
LDHB	0	0.26837345	0.901	0.733	0	AMs
PARVG	0	0.2197863	0.905	0.737	0	AMs

SH2B2	0	0.44812672	0.579	0.411	0	AMs
SAMD4A	0	0.75803056	0.424	0.256	0	AMs
LSM6	0	0.19136589	0.837	0.669	0	AMs
FCHO2	0	0.38723129	0.692	0.524	0	AMs
GNG10	0	0.25567214	0.954	0.786	0	AMs
MSRB2	0	0.33724315	0.69	0.522	0	AMs
SEL1L	0	0.20638958	0.942	0.774	0	AMs
TXNDC11	0	0.2539908	0.944	0.776	0	AMs
CLDN7	0	0.37176668	0.688	0.52	0	AMs
TGIF1	0	0.18507957	0.825	0.657	0	AMs
BID	0	0.25023605	0.703	0.535	0	AMs
LEPROT	0	0.25160836	0.759	0.592	0	AMs
ADAMTSL4	0	0.36999181	0.996	0.829	0	AMs
SOAT1	0	0.19690491	0.888	0.721	0	AMs
HNMT	0	0.30019276	0.74	0.573	0	AMs
STIMATE	0	0.20712389	0.738	0.571	0	AMs
ATP6V1A	0	0.1640186	0.876	0.709	0	AMs
WDFY4	0	0.18224059	0.747	0.58	0	AMs
GAA	0	0.35681307	0.991	0.824	0	AMs
BCAR3	0	0.42616413	0.572	0.405	0	AMs
HECTD3	0	0.1986387	0.786	0.62	0	AMs
F11R	0	0.1708752	0.873	0.707	0	AMs
RAB1A	0	0.15964005	0.808	0.642	0	AMs
TMEM127	0	0.20365066	0.935	0.769	0	AMs
RAB7A	0	0.168915	0.9	0.734	0	AMs
MANBA	0	0.18717049	0.86	0.694	0	AMs
TREM1	0	0.41301252	0.992	0.826	0	AMs
BNIP3L	0	0.20351084	0.887	0.721	0	AMs
ERP44	0	0.18657546	0.897	0.731	0	AMs
SH3PXD2A	0	0.47886072	0.462	0.296	0	AMs
APIP	0	0.24784506	0.746	0.58	0	AMs
RGS19	0	0.16942553	0.901	0.735	0	AMs
SOD1	0	0.11032117	0.677	0.511	0	AMs
UNC5B	0	0.407791	0.625	0.459	0	AMs
CLCN4	0	0.57942838	0.503	0.337	0	AMs
CTBS	0	0.18952151	0.817	0.651	0	AMs
ATP6V1B2	0	0.24329459	0.957	0.791	0	AMs
PRAM1	0	0.21866567	0.817	0.651	0	AMs
PEX14	0	0.26691854	0.733	0.568	0	AMs
PPARG	0	0.3529709	0.995	0.83	0	AMs
ARHGAP31	0	0.23070694	0.765	0.6	0	AMs
GPNMB	0	0.27682939	0.988	0.823	0	AMs
TAX1BP1	0	0.13961868	0.886	0.721	0	AMs
YWHAG	0	0.17525957	0.86	0.695	0	AMs
PTPN12	0	0.1587326	0.937	0.772	0	AMs
TRIM14	0	0.13570413	0.911	0.746	0	AMs
TSPAN15	0	0.37154183	0.533	0.368	0	AMs
SLC15A3	0	0.31728256	0.989	0.824	0	AMs
CARS2	0	0.17487061	0.757	0.592	0	AMs
EHD4	0	0.182914	0.915	0.75	0	AMs
MLX	0	0.18570436	0.872	0.707	0	AMs
RAB31	0	0.22473718	0.92	0.755	0	AMs

PIP5K1C	0	0.23626733	0.742	0.577	0	AMs
OGFR	0	0.18495777	0.931	0.766	0	AMs
NDUFA1	0	0.15908155	0.807	0.642	0	AMs
DIXDC1	0	0.65133999	0.423	0.258	0	AMs
NCKAP5L	0	0.32162503	0.596	0.431	0	AMs
NECAP2	0	0.22828534	0.949	0.784	0	AMs
C1orf162	0	0.27368273	0.962	0.797	0	AMs
MARCH1	0	0.21762848	0.827	0.662	0	AMs
MAN1A1	0	0.21430082	0.942	0.777	0	AMs
GSN	0	0.3784824	0.968	0.803	0	AMs
DNAJB12	0	0.16445198	0.822	0.657	0	AMs
VTI1B	0	0.19208607	0.822	0.657	0	AMs
ITPK1	0	0.24475737	0.966	0.801	0	AMs
COMT	0	0.2572875	0.975	0.81	0	AMs
TMEM35B	0	0.12597201	0.784	0.62	0	AMs
ZYG11B	0	0.19803809	0.793	0.629	0	AMs
RASSF5	0	0.12791276	0.91	0.746	0	AMs
PECR	0	0.37739143	0.55	0.386	0	AMs
UPP1	0	0.18817252	0.914	0.75	0	AMs
STOM	0	0.18577499	0.892	0.728	0	AMs
COX14	0	0.12993201	0.769	0.605	0	AMs
ISCU	0	0.15821684	0.858	0.694	0	AMs
SPG21	0	0.16903562	0.903	0.739	0	AMs
SELENOW	0	0.10931579	0.897	0.733	0	AMs
NAA20	0	0.16991434	0.89	0.726	0	AMs
RENBP	0	0.23745705	0.802	0.638	0	AMs
SELENOM	0	0.4939449	0.381	0.217	0	AMs
ANXA7	0	0.13630173	0.838	0.674	0	AMs
ATP6V0C	0	0.23943509	0.947	0.783	0	AMs
CSTB	0	0.31776026	0.953	0.789	0	AMs
ATP6V0B	0	0.21046857	0.935	0.772	0	AMs
TBC1D9	0	0.29790855	0.74	0.577	0	AMs
SPARC	0	0.31165805	0.742	0.579	0	AMs
TMEM14C	0	0.16386948	0.781	0.618	0	AMs
SRI	0	0.10661809	0.852	0.689	0	AMs
CTSL	0	0.25225185	0.987	0.824	0	AMs
TMEM219	0	0.18985907	0.93	0.767	0	AMs
UBXN6	0	0.14251056	0.805	0.642	0	AMs
HCK	0	0.27918379	0.984	0.821	0	AMs
PPDPF	0	0.15812501	0.872	0.709	0	AMs
MOSPD2	0	0.19936548	0.76	0.597	0	AMs
PLPP3	0	0.4792094	0.504	0.341	0	AMs
GALNT6	0	0.33138841	0.605	0.442	0	AMs
LRRC41	0	0.16160817	0.719	0.556	0	AMs
NCSTN	0	0.19787876	0.967	0.804	0	AMs
CD46	0	0.23573755	0.946	0.783	0	AMs
SUMF2	0	0.15847954	0.943	0.78	0	AMs
CD81	0	0.36950241	0.971	0.808	0	AMs
GDE1	0	0.16963732	0.837	0.674	0	AMs
LIMK2	0	0.21629109	0.695	0.532	0	AMs
AHCYL1	0	0.17095567	0.787	0.625	0	AMs
NPL	0	0.32411228	0.646	0.484	0	AMs

RAB4A	0	0.14630292	0.74	0.578	0	AMs
NDUFB5	0	0.16771156	0.892	0.73	0	AMs
IFNGR1	0	0.14800162	0.932	0.77	0	AMs
NANS	0	0.21461087	0.808	0.646	0	AMs
RSU1	0	0.13913351	0.885	0.723	0	AMs
PLBD1	0	0.41519127	0.976	0.814	0	AMs
SLC7A7	0	0.24476855	0.986	0.824	0	AMs
LPCAT2	0	0.23878157	0.783	0.621	0	AMs
ZDHHC3	0	0.14915471	0.843	0.681	0	AMs
ZNF331	0	0.40257603	0.69	0.528	0	AMs
AGFG1	0	0.15942334	0.914	0.753	0	AMs
GTPBP2	0	0.17165502	0.765	0.604	0	AMs
STX7	0	0.15659607	0.871	0.71	0	AMs
MOB3B	0	0.23274683	0.849	0.688	0	AMs
AQP3	0	0.2194322	0.859	0.698	0	AMs
MAP3K11	0	0.18926889	0.772	0.611	0	AMs
GGA2	0	0.3262916	0.991	0.83	0	AMs
SIGLEC14	0	0.24774129	0.746	0.585	0	AMs
UQCR10	0	0.14136126	0.851	0.69	0	AMs
ZMYND15	0	0.39230802	0.599	0.438	0	AMs
RAB11FIP4	0	0.18306015	0.495	0.334	0	AMs
TMED5	0	0.26184045	0.948	0.787	0	AMs
RRAGD	0	0.24709877	0.722	0.561	0	AMs
PNPLA2	0	0.23380369	0.966	0.805	0	AMs
NOP10	0	0.18507273	0.824	0.663	0	AMs
PKM	0	0.19405597	0.958	0.797	0	AMs
LPAR3	0	0.63722601	0.4	0.24	0	AMs
FCGR3A	0	0.25439499	0.981	0.821	0	AMs
ANXA4	0	0.15286339	0.893	0.733	0	AMs
IDH1	0	0.15774886	0.861	0.701	0	AMs
LAMTOR3	0	0.15861298	0.762	0.602	0	AMs
TMEM140	0	0.16402308	0.672	0.512	0	AMs
TBC1D2	0	0.20947316	0.791	0.631	0	AMs
STX4	0	0.13623982	0.873	0.713	0	AMs
RAB4B	0	0.13099903	0.761	0.601	0	AMs
PLAUR	0	0.32641945	0.992	0.832	0	AMs
PSMA7	0	0.13622433	0.895	0.735	0	AMs
COX15	0	0.2980709	0.627	0.467	0	AMs
ATOX1	0	0.24202316	0.701	0.541	0	AMs
HTATIP2	0	0.14346152	0.836	0.676	0	AMs
VAMP3	0	0.15674614	0.92	0.761	0	AMs
CERT1	0	0.14486895	0.867	0.708	0	AMs
EPB41L4A	0	0.57752775	0.442	0.283	0	AMs
VPS41	0	0.16095664	0.785	0.626	0	AMs
LAMB1	0	1.32739807	0.274	0.115	0	AMs
PAFAH1B2	0	0.14196829	0.893	0.734	0	AMs
FAR2	0	0.27711922	0.687	0.528	0	AMs
FLVCR2	0	0.20727621	0.798	0.639	0	AMs
MINK1	0	0.38769607	0.99	0.831	0	AMs
DUSP3	0	0.19821889	0.79	0.631	0	AMs
PNPO	0	0.28152999	0.67	0.511	0	AMs
BACH1	0	0.16734581	0.912	0.753	0	AMs

DESI1	0	0.18029997	0.722	0.563	0	AMs
ZFYVE26	0	0.32897866	0.612	0.453	0	AMs
PNPLA3	0	1.25808205	0.299	0.14	0	AMs
TMEM59	0	0.12844443	0.831	0.672	0	AMs
GLRX	0	0.29297438	0.964	0.805	0	AMs
FKBP15	0	0.15348446	0.841	0.682	0	AMs
BST2	0	0.225382	0.944	0.785	0	AMs
ACOT7	0	0.20083117	0.629	0.471	0	AMs
SESN2	0	0.31579947	0.667	0.509	0	AMs
RALB	0	0.15918171	0.772	0.614	0	AMs
CRTAP	0	0.24444692	0.911	0.753	0	AMs
IMPAD1	0	0.12514303	0.895	0.737	0	AMs
TRIM44	0	0.14562515	0.775	0.617	0	AMs
NUMB	0	0.12206836	0.81	0.652	0	AMs
ACOT4	0	0.46444548	0.513	0.355	0	AMs
ARRDC4	0	0.31395991	0.767	0.609	0	AMs
SIN3B	0	0.17437149	0.642	0.484	0	AMs
LILRB1	0	0.29224974	0.674	0.516	0	AMs
AMOTL1	0	0.32996228	0.587	0.429	0	AMs
CSRP1	0	0.18706341	0.957	0.799	0	AMs
ACER3	0	0.24120904	0.713	0.555	0	AMs
EMC10	0	0.19888436	0.965	0.807	0	AMs
NCF2	0	0.28539211	0.995	0.838	0	AMs
TMEM33	0	0.13401791	0.918	0.761	0	AMs
CAPZA2	0	0.12347013	0.877	0.72	0	AMs
GSTO1	0	0.22028712	0.973	0.816	0	AMs
IVD	0	0.32506887	0.547	0.39	0	AMs
VAV1	0	0.12982028	0.729	0.572	0	AMs
MCEMP1	0	0.37961476	0.986	0.829	0	AMs
RNF114	0	0.12757478	0.737	0.58	0	AMs
CRIM1	0	0.40305682	0.407	0.25	0	AMs
VRK2	0	0.28981772	0.62	0.463	0	AMs
IL1A	0	0.93929519	0.348	0.191	0	AMs
ABHD14B	0	0.22152742	0.579	0.422	0	AMs
KCNA3	0	0.11076358	0.693	0.536	0	AMs
RNF130	0	0.18327406	0.964	0.807	0	AMs
DNAJC3	0	0.14582253	0.816	0.659	0	AMs
RIN3	0	0.14800981	0.96	0.803	0	AMs
PGD	0	0.33893797	0.984	0.828	0	AMs
GBP3	0	0.22111118	0.626	0.47	0	AMs
DPH3	0	0.20018496	0.663	0.507	0	AMs
PPM1M	0	0.12571403	0.732	0.576	0	AMs
PARP12	0	0.10485495	0.86	0.704	0	AMs
GNS	0	0.27118645	0.977	0.821	0	AMs
UBL7	0	0.13782084	0.728	0.572	0	AMs
SIGLEC11	0	0.5840369	0.482	0.326	0	AMs
VARS	0	0.14247006	0.713	0.557	0	AMs
CLEC12A	0	0.26107166	0.693	0.537	0	AMs
ARPC3	0	0.12085222	0.842	0.686	0	AMs
KYNU	0	0.26163261	0.764	0.609	0	AMs
IQSEC1	0	0.24293804	0.979	0.824	0	AMs
SSR3	0	0.13712186	0.801	0.646	0	AMs

ATP6V0E1	0	0.15352337	0.911	0.756	0	AMs
SDCBP	0	0.37596072	0.978	0.823	0	AMs
SLC29A3	0	0.33430916	0.636	0.481	0	AMs
RTN3	0	0.19539473	0.97	0.815	0	AMs
NCKAP1L	0	0.20405471	0.971	0.816	0	AMs
KCTD10	0	0.19784697	0.665	0.51	0	AMs
MLEC	0	0.12223816	0.747	0.592	0	AMs
SQOR	0	0.16255515	0.861	0.706	0	AMs
KAT8	0	0.1463374	0.752	0.597	0	AMs
STAT5A	0	0.12829022	0.749	0.594	0	AMs
UQCR11	0	0.10062385	0.774	0.619	0	AMs
TRAPPC6A	0	0.13001328	0.557	0.402	0	AMs
SUMF1	0	0.32403004	0.599	0.444	0	AMs
TMEM14A	0	0.18758849	0.579	0.424	0	AMs
KIAA0513	0	0.24154928	0.617	0.462	0	AMs
PDCD6IP	0	0.17651899	0.962	0.807	0	AMs
FBXL5	0	0.12569747	0.94	0.785	0	AMs
NCOA4	0	0.20476471	0.948	0.793	0	AMs
DSTN	0	0.10432491	0.693	0.538	0	AMs
NRIP1	0	0.24377874	0.709	0.554	0	AMs
LAPTM4A	0	0.15879066	0.764	0.61	0	AMs
ARHGEF28	0	0.26441178	0.676	0.522	0	AMs
TMEM30A	0	0.2518877	0.678	0.524	0	AMs
DCTN6	0	0.17168917	0.717	0.563	0	AMs
GPR107	0	0.13198149	0.755	0.601	0	AMs
VAPA	0	0.11277619	0.848	0.694	0	AMs
ACOT9	0	0.14374836	0.754	0.6	0	AMs
IGF1	0	1.64009656	0.246	0.092	0	AMs
VGLL3	0	1.25506411	0.294	0.14	0	AMs
RSPH3	0	0.32901263	0.59	0.436	0	AMs
P2RX1	0	0.64659166	0.431	0.277	0	AMs
NDUFS5	0	0.14688301	0.696	0.542	0	AMs
EGLN1	0	0.10681001	0.696	0.542	0	AMs
FEZ2	0	0.20787378	0.696	0.542	0	AMs
G6PC3	0	0.24145365	0.697	0.543	0	AMs
APOL2	0	0.11035219	0.696	0.542	0	AMs
LY96	0	0.20261784	0.722	0.569	0	AMs
MID1IP1	0	0.15024415	0.899	0.746	0	AMs
C1RL	0	0.31120078	0.584	0.431	0	AMs
SNX17	0	0.14948058	0.951	0.798	0	AMs
SYS1	0	0.17018564	0.697	0.544	0	AMs
PAFAH2	0	0.31962384	0.541	0.389	0	AMs
POMGNT1	0	0.116615	0.681	0.529	0	AMs
RHOB	0	0.33426911	0.984	0.832	0	AMs
RTN4	0	0.2315687	0.987	0.835	0	AMs
DDX60L	0	0.26151446	0.674	0.522	0	AMs
RNF146	0	0.23239585	0.654	0.502	0	AMs
DBNL	0	0.18076599	0.975	0.823	0	AMs
DCSTAMP	0	0.73599842	0.405	0.253	0	AMs
UGCG	0	0.24573476	0.548	0.396	0	AMs
CACUL1	0	0.14413931	0.77	0.618	0	AMs
LARP4	0	0.11613558	0.751	0.599	0	AMs

PNPLA6	0	0.3280285	0.994	0.842	0	AMs
PLD3	0	0.48905804	0.979	0.827	0	AMs
RUNX1	0	0.23794895	0.967	0.815	0	AMs
BPNT1	0	0.27596061	0.602	0.45	0	AMs
RGS12	0	0.43451149	0.575	0.423	0	AMs
UBXN2B	0	0.10954082	0.824	0.672	0	AMs
BMPR2	0	0.14596031	0.68	0.529	0	AMs
SLC12A7	0	0.18979053	0.756	0.605	0	AMs
SNX10	0	0.24792229	0.968	0.817	0	AMs
HIBADH	0	0.37520605	0.54	0.389	0	AMs
IFIT1	0	0.36789997	0.622	0.471	0	AMs
SCD	0	0.80878125	0.988	0.837	0	AMs
CD9	0	0.57534329	0.981	0.83	0	AMs
ADA2	0	0.28543097	0.972	0.821	0	AMs
GCNT1	0	0.26149293	0.608	0.457	0	AMs
MPND	0	0.33882875	0.579	0.428	0	AMs
LRRC25	0	0.25390738	0.689	0.538	0	AMs
THEMIS2	0	0.27717982	0.996	0.846	0	AMs
UNC50	0	0.12654111	0.716	0.566	0	AMs
CD63	0	0.31180909	0.985	0.835	0	AMs
SAT2	0	0.18224528	0.651	0.501	0	AMs
NME2	0	0.13118423	0.902	0.752	0	AMs
TIMP2	0	0.27607836	0.991	0.841	0	AMs
TCEAL4	0	0.28911625	0.648	0.498	0	AMs
ALPK3	0	0.86233118	0.383	0.233	0	AMs
CDK18	0	0.38018054	0.587	0.437	0	AMs
SLC25A25	0	0.26852107	0.565	0.415	0	AMs
NFE2L2	0	0.15670261	0.946	0.796	0	AMs
MGST2	0	0.23782428	0.701	0.551	0	AMs
CCND3	0	0.16882337	0.948	0.798	0	AMs
SLA	0	0.22459939	0.948	0.798	0	AMs
TCN2	0	0.27540399	0.69	0.54	0	AMs
LYPLAL1	0	0.24315636	0.634	0.485	0	AMs
RETSAT	0	0.23622276	0.619	0.47	0	AMs
CNBP	0	0.11016824	0.916	0.767	0	AMs
FCGRT	0	0.28578786	0.998	0.849	0	AMs
PTGER3	0	0.98655987	0.294	0.145	0	AMs
DRAM2	0	0.12137409	0.764	0.616	0	AMs
ERGIC1	0	0.25613646	0.538	0.39	0	AMs
AIF1	0	0.22398037	0.981	0.833	0	AMs
CLIC1	0	0.10464907	0.883	0.735	0	AMs
SELENOH	0	0.10032521	0.928	0.78	0	AMs
TMED3	0	0.19749306	0.655	0.507	0	AMs
STAT3	0	0.12981716	0.936	0.788	0	AMs
ATP6AP1	0	0.26318791	0.984	0.836	0	AMs
MAPRE3	0	0.51830243	0.419	0.271	0	AMs
ST7	0	0.23158885	0.6	0.452	0	AMs
MPZL2	0	0.32595426	0.592	0.444	0	AMs
ZFYVE16	0	0.21587293	0.698	0.55	0	AMs
ATP6V1H	0	0.16908752	0.703	0.555	0	AMs
UBE2L6	0	0.162315	0.942	0.794	0	AMs
SCP2	0	0.30543818	0.982	0.835	0	AMs

LRPAP1	0	0.27279752	0.991	0.844	0	AMs
NDUFS4	0	0.11269697	0.719	0.572	0	AMs
CHD7	0	0.28600368	0.396	0.249	0	AMs
JAML	0	0.19379701	0.971	0.824	0	AMs
SEPHS2	0	0.12738675	0.713	0.566	0	AMs
TNFSF13	0	0.35953309	0.996	0.849	0	AMs
PECAM1	0	0.25874058	0.992	0.845	0	AMs
PSMD10	0	0.13632114	0.679	0.532	0	AMs
RIPK2	0	0.27130573	0.615	0.468	0	AMs
PCCA	0	0.17250421	0.583	0.436	0	AMs
CHCHD10	0	0.24384669	0.567	0.42	0	AMs
NIT1	0	0.18849462	0.649	0.503	0	AMs
MYD88	0	0.27087992	0.986	0.84	0	AMs
DOK2	0	0.32028805	0.988	0.842	0	AMs
DNAJC5B	0	0.95137205	0.329	0.183	0	AMs
PARP10	0	0.15108473	0.644	0.498	0	AMs
VWA5A	0	0.3287695	0.512	0.366	0	AMs
APLP2	0	0.22532488	0.99	0.844	0	AMs
RABGGTA	0	0.20773747	0.667	0.521	0	AMs
TYROBP	0	0.50004249	0.988	0.842	0	AMs
LAMP2	0	0.21117719	0.972	0.826	0	AMs
HMGCL	0	0.19751295	0.601	0.455	0	AMs
HPGDS	0	0.52571521	0.408	0.262	0	AMs
MCFD2	0	0.12989278	0.69	0.544	0	AMs
PINK1	0	0.2222798	0.562	0.417	0	AMs
MGST3	0	0.32221702	0.986	0.841	0	AMs
CHMP2B	0	0.13602837	0.657	0.512	0	AMs
PPARD	0	0.27516922	0.683	0.538	0	AMs
NDUFB1	0	0.16814583	0.661	0.516	0	AMs
APH1B	0	0.32264524	0.512	0.367	0	AMs
MAN2B1	0	0.31754443	0.986	0.841	0	AMs
PPP2R3A	0	0.73843958	0.353	0.208	0	AMs
TCEA3	0	0.36958101	0.484	0.339	0	AMs
LSM10	0	0.13135103	0.611	0.466	0	AMs
MSMO1	0	0.63755411	0.408	0.263	0	AMs
RNASEK	0	0.17729237	0.949	0.804	0	AMs
SLC2A9	0	0.28161073	0.659	0.515	0	AMs
ARPC1B	0	0.17229872	0.973	0.829	0	AMs
RHOBTB2	0	0.36407238	0.539	0.395	0	AMs
RASSF4	0	0.24059336	0.642	0.498	0	AMs
IL18	0	0.30486227	0.623	0.479	0	AMs
CEBPB	0	0.24337115	0.995	0.851	0	AMs
TLR7	0	0.39232243	0.5	0.356	0	AMs
EFNB1	0	0.34268078	0.58	0.436	0	AMs
TRIB1	0	0.10757696	0.555	0.412	0	AMs
SEPTIN11	0	0.22941267	0.983	0.84	0	AMs
RETREG1	0	0.46426062	0.403	0.26	0	AMs
TRIM21	0	0.15677858	0.645	0.502	0	AMs
PTPN6	0	0.30637107	0.987	0.844	0	AMs
LONRF3	0	0.22483052	0.684	0.541	0	AMs
SASH3	0	0.14440326	0.959	0.816	0	AMs
TNFAIP8L2	0	0.26980986	0.582	0.439	0	AMs

NDUFA4	0	0.15570633	0.943	0.8	0	AMs
LACTB	0	0.18388748	0.698	0.555	0	AMs
CAPNS1	0	0.18723725	0.948	0.805	0	AMs
ACYP2	0	0.12920573	0.562	0.42	0	AMs
MSRB1	0	0.30280301	0.554	0.412	0	AMs
IFI6	0	0.25030545	0.979	0.837	0	AMs
LPXN	0	0.18603417	0.973	0.831	0	AMs
BSCL2	0	0.22487746	0.653	0.511	0	AMs
DNAJC4	0	0.1025039	0.613	0.471	0	AMs
C1QC	0	0.22602274	0.994	0.853	0	AMs
PLEKHB2	0	0.16634057	0.976	0.835	0	AMs
CD302	0	0.27909274	0.62	0.479	0	AMs
TFRC	0	0.46504921	0.991	0.85	0	AMs
SQSTM1	0	0.29019679	0.982	0.841	0	AMs
PRXL2A	0	0.71647043	0.376	0.235	0	AMs
SLC22A18	0	0.42120134	0.441	0.3	0	AMs
TIGAR	0	0.19801613	0.619	0.478	0	AMs
SEC11A	0	0.16836071	0.961	0.82	0	AMs
VMO1	0	0.42704304	0.528	0.387	0	AMs
NAGLU	0	0.29108682	0.591	0.45	0	AMs
CASP9	0	0.30613598	0.546	0.406	0	AMs
EVI5	0	0.26435177	0.634	0.494	0	AMs
KLHL29	0	0.63521816	0.377	0.237	0	AMs
ABHD16A	0	0.20915754	0.637	0.497	0	AMs
CLN5	0	0.31161055	0.518	0.378	0	AMs
ACP5	0	0.36653872	0.996	0.856	0	AMs
ALKBH7	0	0.10769205	0.577	0.437	0	AMs
CYSLTR1	0	0.26293889	0.55	0.411	0	AMs
TAF10	0	0.12774369	0.953	0.814	0	AMs
ITM2B	0	0.36934908	0.978	0.839	0	AMs
SH3BGRL	0	0.32530528	0.975	0.836	0	AMs
TMEFF1	0	0.93240064	0.297	0.158	0	AMs
FDX1	0	0.31787769	0.567	0.428	0	AMs
RILP	0	0.29770251	0.574	0.435	0	AMs
LRRC8D	0	0.11445664	0.607	0.469	0	AMs
PROS1	0	0.4981452	0.426	0.288	0	AMs
PFDN1	0	0.18992099	0.64	0.502	0	AMs
MRC1	0	0.31732704	0.996	0.858	0	AMs
ALOX5	0	0.34150026	0.999	0.861	0	AMs
SAT1	0	0.23398065	0.989	0.851	0	AMs
TOMM6	0	0.15611335	0.578	0.44	0	AMs
SC5D	0	0.21474269	0.501	0.364	0	AMs
ARF3	0	0.1749532	0.606	0.469	0	AMs
BBS2	0	0.26122425	0.497	0.36	0	AMs
HSD17B14	0	0.43320319	0.526	0.389	0	AMs
SLC50A1	0	0.14392687	0.576	0.439	0	AMs
GGA1	0	0.22373165	0.551	0.415	0	AMs
CAMSAP2	0	0.3600574	0.487	0.351	0	AMs
RAB10	0	0.17836031	0.973	0.837	0	AMs
DDO	0	0.5579886	0.422	0.286	0	AMs
TOP1MT	0	0.27427487	0.501	0.365	0	AMs
AKR1C3	0	0.44823018	0.52	0.384	0	AMs

TBXAS1	0	0.24150495	0.572	0.436	0	AMs
CETN2	0	0.19116838	0.581	0.445	0	AMs
TPRG1L	0	0.10011594	0.548	0.413	0	AMs
KAZN	0	0.63010044	0.343	0.208	0	AMs
FBP1	0	0.30330743	0.995	0.86	0	AMs
ST5	0	0.54595314	0.403	0.268	0	AMs
CD4	0	0.25259138	0.974	0.839	0	AMs
CLEC4A	0	0.24859806	0.621	0.486	0	AMs
OLR1	0	0.39556275	0.999	0.864	0	AMs
FAM222B	0	0.22996311	0.459	0.324	0	AMs
STXBP2	0	0.23436687	0.992	0.857	0	AMs
CST3	0	0.28434252	0.991	0.856	0	AMs
SGSM3	0	0.15679728	0.577	0.442	0	AMs
RAPGEF2	0	0.14314583	0.556	0.422	0	AMs
CD274	0	0.55467166	0.402	0.268	0	AMs
CD37	0	0.2919219	0.974	0.84	0	AMs
MAFF	0	0.34480676	0.357	0.223	0	AMs
C6orf120	0	0.13899673	0.563	0.429	0	AMs
TMBIM1	0	0.31680639	0.984	0.851	0	AMs
MS4A7	0	0.34363989	0.999	0.866	0	AMs
LTA4H	0	0.28093099	0.998	0.865	0	AMs
ATL1	0	0.44514426	0.463	0.33	0	AMs
MARCH2	0	0.19100548	0.572	0.439	0	AMs
CYP51A1	0	0.43969397	0.506	0.374	0	AMs
CLEC4E	0	0.44305576	0.453	0.321	0	AMs
ATP6V0A1	0	0.26639714	0.529	0.397	0	AMs
SRXN1	0	0.25263775	0.611	0.479	0	AMs
USP11	0	0.18690262	0.526	0.394	0	AMs
DSC2	0	0.26047461	0.571	0.439	0	AMs
RNF144B	0	0.39752652	0.524	0.393	0	AMs
LAMTOR1	0	0.29736036	0.981	0.85	0	AMs
CXCL16	0	0.39362492	0.995	0.864	0	AMs
CYB5R3	0	0.23342196	0.988	0.857	0	AMs
SLC4A8	0	0.50136046	0.357	0.226	0	AMs
ARHGAP6	0	0.60816005	0.365	0.234	0	AMs
RNASEL	0	0.27787442	0.49	0.36	0	AMs
TRAF3IP2	0	0.33911143	0.425	0.295	0	AMs
PLIN2	0	0.4873334	0.423	0.293	0	AMs
FBXO10	0	0.56102538	0.367	0.237	0	AMs
SYNGR2	0	0.2241809	0.992	0.862	0	AMs
A4GALT	0	0.70678134	0.32	0.19	0	AMs
FCGR1B	0	0.33713055	0.526	0.397	0	AMs
PDE8A	0	0.31489446	0.474	0.345	0	AMs
PTPRM	0	0.42559963	0.41	0.281	0	AMs
C1QB	0	0.41633464	0.995	0.867	0	AMs
DTNA	0	0.64731733	0.332	0.205	0	AMs
TMEM150A	0	0.31936786	0.372	0.245	0	AMs
REEP5	0	0.18458984	0.967	0.84	0	AMs
ANXA11	0	0.18654606	0.977	0.85	0	AMs
NR4A3	0	0.3251915	0.505	0.379	0	AMs
ARL11	0	0.38519089	0.472	0.346	0	AMs
IGFBP2	0	0.43003389	0.511	0.387	0	AMs

CRELD1	0	0.27100736	0.447	0.323	0	AMs
GRINA	0	0.33023141	0.997	0.873	0	AMs
NMB	0	0.48759106	0.452	0.328	0	AMs
CENPV	0	0.50080198	0.372	0.248	0	AMs
VASP	0	0.26671346	0.991	0.867	0	AMs
NUCB1	0	0.2414449	0.994	0.87	0	AMs
PROCR	0	0.40501369	0.45	0.326	0	AMs
ST13	0	0.21851198	0.433	0.309	0	AMs
FCER1G	0	0.30268408	0.994	0.871	0	AMs
EXD2	0	0.32400316	0.486	0.363	0	AMs
HP	0	0.60333477	0.426	0.303	0	AMs
TSPO	0	0.23912884	0.992	0.869	0	AMs
SESN1	0	0.15651839	0.43	0.308	0	AMs
TDRD3	0	0.3098046	0.434	0.312	0	AMs
TTC8	0	0.4643865	0.386	0.264	0	AMs
ANO5	0	0.77045965	0.288	0.166	0	AMs
NECAB3	0	0.44808325	0.326	0.205	0	AMs
ALDH1A2	0	1.23347126	0.23	0.109	0	AMs
ALDH2	0	0.43865024	0.998	0.877	0	AMs
RBP7	0	0.57588592	0.348	0.227	0	AMs
CAPG	0	0.33804352	0.994	0.874	0	AMs
PSD3	0	0.55912146	0.368	0.248	0	AMs
IFI27	0	0.95193184	0.388	0.268	0	AMs
ERRFI1	0	0.43114144	0.397	0.278	0	AMs
SPI1	0	0.29408703	0.999	0.88	0	AMs
AOC3	0	1.00266991	0.236	0.117	0	AMs
FBN1	0	1.54637575	0.192	0.074	0	AMs
GPR153	0	0.59596444	0.338	0.221	0	AMs
GLIPR2	0	0.20646647	0.993	0.876	0	AMs
FAXDC2	0	0.96258222	0.233	0.118	0	AMs
TMEM104	0	0.39922931	0.396	0.281	0	AMs
PARP3	0	0.55305168	0.322	0.208	0	AMs
RNF149	0	0.20593379	0.996	0.882	0	AMs
FGF10	0	1.5935869	0.175	0.061	0	AMs
BRI3	0	0.19940833	0.994	0.88	0	AMs
HSD17B8	0	0.27692003	0.351	0.238	0	AMs
CTSD	0	0.30185936	0.994	0.881	0	AMs
MOSPD1	0	0.6298456	0.277	0.165	0	AMs
PSAP	0	0.32386847	0.999	0.888	0	AMs
STARD10	0	0.19772335	0.311	0.2	0	AMs
WFS1	0	0.72976728	0.262	0.152	0	AMs
C1QA	0	0.37943199	0.997	0.887	0	AMs
EFHD2	0	0.19587821	0.993	0.885	0	AMs
COL9A2	0	0.53449002	0.306	0.198	0	AMs
SERPINA1	0	0.37651055	0.997	0.889	0	AMs
LIMS2	0	0.87665614	0.25	0.143	0	AMs
TMEM91	0	0.59409078	0.271	0.164	0	AMs
CTSS	0	0.32145123	0.994	0.887	0	AMs
GPR85	0	0.64894153	0.284	0.177	0	AMs
TGOLN2	0	0.24391445	0.993	0.887	0	AMs
ANXA5	0	0.25565131	0.995	0.889	0	AMs
LAMP1	0	0.24279402	0.992	0.886	0	AMs

RASL11A	0	0.6383471	0.276	0.171	0	AMs
CD68	0	0.31664512	0.997	0.892	0	AMs
OPLAH	0	0.69961223	0.262	0.158	0	AMs
DHCR7	0	0.64812108	0.27	0.166	0	AMs
CHRM3	0	1.05685744	0.205	0.101	0	AMs
ACACB	0	0.50286409	0.287	0.183	0	AMs
LITAF	0	0.30109499	0.995	0.891	0	AMs
CADPS2	0	1.38232299	0.162	0.06	0	AMs
ALOX5AP	0	0.2372186	0.991	0.889	0	AMs
SHROOM3	0	1.11951388	0.192	0.092	0	AMs
RAPGEF3	0	0.79641095	0.228	0.13	0	AMs
LY6E	0	0.30952445	0.991	0.893	0	AMs
ITSN1	0	0.82043296	0.222	0.126	0	AMs
CCDC85C	0	0.88163732	0.179	0.084	0	AMs
GLUL	0	0.26193293	0.999	0.904	0	AMs
CYBA	0	0.22074546	0.996	0.907	0	AMs
CYGB	0	1.47050362	0.145	0.059	0	AMs
PCSK9	0	1.44451773	0.141	0.058	0	AMs
TJP1	0	1.12019593	0.135	0.053	0	AMs
PKDCC	0	1.4620754	0.132	0.052	0	AMs
S100A11	0	0.20972718	0.993	0.915	0	AMs
CTSC	0	0.28537631	0.998	0.922	0	AMs
LGALS3	0	0.35048894	0.998	0.923	0	AMs
ENPP3	0	1.20164056	0.11	0.04	0	AMs
CD52	0	0.36262083	0.99	0.934	0	AMs
FTL	0	0.45380464	0.999	0.963	0	AMs
LYZ	0	0.3199021	1	0.965	0	AMs
OAZ1	0	0.17918541	0.998	0.971	0	AMs
FTH1	0	0.40093978	1	0.975	0	AMs
CD74	0	0.31573639	1	0.978	0	AMs
TXNIP	0	0.13999135	0.999	0.983	0	AMs
B2M	0	0.13719564	0.999	0.996	0	AMs
TMSB4X	0	0.14412239	1	0.997	0	AMs
SLC38A10	5.536E-308	0.10368262	0.863	0.699	8.509E-304	AMs
ADIPOR2	1.404E-307	0.10581725	0.665	0.525	2.158E-303	AMs
EIF6	3.311E-307	0.11401219	0.858	0.703	5.089E-303	AMs
ZNF185	3.362E-307	0.29315058	0.547	0.418	5.168E-303	AMs
SORBS3	1.061E-306	0.21530876	0.639	0.498	1.631E-302	AMs
CDK14	1.499E-306	0.27331591	0.558	0.425	2.304E-302	AMs
IPO8	2.985E-306	0.12067964	0.779	0.63	4.588E-302	AMs
KCTD5	5.64E-306	0.16480419	0.671	0.533	8.669E-302	AMs
HIGD1A	1.434E-305	0.17033779	0.54	0.41	2.205E-301	AMs
PGLS	3.17E-305	0.1066528	0.793	0.647	4.873E-301	AMs
SMCO4	3.537E-305	0.22412895	0.684	0.541	5.437E-301	AMs
CTNNA1	1.282E-304	0.18133685	0.943	0.778	1.971E-300	AMs
RNFT1	1.74E-304	0.16006916	0.633	0.497	2.674E-300	AMs
ADCY3	3.435E-304	0.21263485	0.667	0.534	5.279E-300	AMs
SLC46A3	1.116E-303	0.27390763	0.402	0.286	1.716E-299	AMs
TXNRD1	1.234E-303	0.1429061	0.898	0.74	1.897E-299	AMs
TAX1BP3	1.943E-303	0.15677083	0.819	0.664	2.987E-299	AMs
TOLLIP	9.959E-303	0.20691082	0.483	0.358	1.531E-298	AMs
ELL2	2.43E-302	0.21338366	0.626	0.493	3.735E-298	AMs

UBE2W	2.521E-302	0.12794756	0.604	0.471	3.874E-298	AMs
LGALSL	2.711E-302	0.849866	0.179	0.094	4.168E-298	AMs
VPS8	7.628E-302	0.12133143	0.538	0.407	1.172E-297	AMs
SLC16A3	1.542E-301	0.17580023	0.974	0.819	2.37E-297	AMs
CAMSAP3	5.596E-301	1.12265424	0.13	0.056	8.601E-297	AMs
PGPEP1	2.655E-300	0.13507263	0.521	0.393	4.08E-296	AMs
NR1H3	3.014E-300	0.31585872	0.644	0.509	4.633E-296	AMs
GLRX2	1.006E-299	0.22037418	0.585	0.452	1.547E-295	AMs
ACP2	1.013E-299	0.16709012	0.683	0.532	1.558E-295	AMs
FOLR1	5.569E-299	0.82556902	0.203	0.113	8.56E-295	AMs
PTGER4	1.51E-298	0.13064603	0.919	0.765	2.321E-294	AMs
PGM5	2.43E-298	0.39974358	0.398	0.281	3.735E-294	AMs
TCEAL8	2.514E-298	0.23150233	0.539	0.41	3.864E-294	AMs
ZNF589	3.843E-298	0.32267809	0.513	0.389	5.907E-294	AMs
CYB5A	6.126E-298	0.18289576	0.717	0.564	9.416E-294	AMs
RAB1B	1.543E-297	0.10063743	0.903	0.744	2.372E-293	AMs
CORO1C	2.518E-297	0.20052078	0.985	0.824	3.871E-293	AMs
ACSF2	2.988E-297	0.32755062	0.454	0.333	4.593E-293	AMs
SMIM30	1.171E-296	0.17076509	0.676	0.533	1.799E-292	AMs
P2RX7	2.268E-295	0.23479557	0.562	0.43	3.486E-291	AMs
RIPK3	2.61E-295	0.14762464	0.537	0.408	4.011E-291	AMs
PPT1	3.266E-294	0.1943484	0.976	0.837	5.02E-290	AMs
TMBIM6	5.213E-294	0.1387174	0.848	0.69	8.013E-290	AMs
IGSF8	8.037E-293	0.16955976	0.452	0.331	1.235E-288	AMs
IFI35	1.238E-292	0.14960644	0.761	0.613	1.902E-288	AMs
SUSD1	1.986E-292	0.21850616	0.727	0.585	3.052E-288	AMs
TSC22D1	2.201E-292	0.45438011	0.277	0.177	3.383E-288	AMs
ATN1	4.223E-292	0.11213285	0.752	0.593	6.492E-288	AMs
TMEM220	1.65E-291	0.33408136	0.464	0.344	2.536E-287	AMs
PLEKHM2	1.292E-290	0.12290339	0.807	0.656	1.986E-286	AMs
CASP7	1.99E-290	0.14883929	0.621	0.487	3.059E-286	AMs
PLXNB2	7.599E-290	0.14984815	0.932	0.761	1.168E-285	AMs
PLCG2	9.809E-290	0.11878883	0.711	0.569	1.508E-285	AMs
RARA	1.712E-289	0.16385281	0.974	0.822	2.631E-285	AMs
EXOSC4	2.526E-289	0.23537973	0.492	0.369	3.882E-285	AMs
KSR1	5.369E-289	0.17883513	0.741	0.597	8.252E-285	AMs
FAM110B	1.019E-287	0.52340438	0.275	0.175	1.566E-283	AMs
SPN	3.789E-287	0.17494019	0.989	0.879	5.824E-283	AMs
TENM4	3.328E-286	1.0250094	0.145	0.068	5.115E-282	AMs
VTN	4.309E-286	0.50409572	0.358	0.25	6.623E-282	AMs
DNAJB2	8.158E-286	0.12157853	0.443	0.325	1.254E-281	AMs
GLB1	1.434E-285	0.19228676	0.953	0.789	2.205E-281	AMs
TEX2	8.088E-285	0.14503354	0.815	0.656	1.243E-280	AMs
HSDL2	1.665E-284	0.13125408	0.71	0.561	2.559E-280	AMs
SNAPIN	2.303E-284	0.1375686	0.706	0.562	3.539E-280	AMs
NAT9	2.431E-284	0.3203013	0.33	0.223	3.736E-280	AMs
FERMT3	2.69E-284	0.14303121	0.96	0.813	4.135E-280	AMs
RAB12	5.301E-284	0.10434707	0.671	0.534	8.149E-280	AMs
SPIRE2	1.36E-283	0.4672888	0.367	0.257	2.09E-279	AMs
DGKQ	1.483E-283	0.29662548	0.399	0.285	2.28E-279	AMs
DOCK1	3.242E-283	0.37196196	0.434	0.319	4.984E-279	AMs
GLCE	3.063E-282	0.28407068	0.465	0.348	4.709E-278	AMs

TNNT1	2.397E-281	1.13482459	0.157	0.079	3.685E-277	AMs
TOR1B	6.185E-281	0.14909437	0.709	0.569	9.507E-277	AMs
ECHDC2	5.913E-280	0.18208437	0.443	0.326	9.088E-276	AMs
WDR13	4.179E-279	0.12842919	0.642	0.508	6.424E-275	AMs
COPS7A	1.269E-278	0.12942132	0.802	0.647	1.95E-274	AMs
UBE2D4	1.299E-278	0.28542369	0.375	0.264	1.996E-274	AMs
SLC25A1	1.764E-278	0.10870307	0.723	0.571	2.711E-274	AMs
OPRK1	2.768E-278	1.73570211	0.082	0.024	4.255E-274	AMs
CR1	3.656E-278	0.26979719	0.506	0.385	5.619E-274	AMs
ABR	7.994E-278	0.1279766	0.92	0.759	1.229E-273	AMs
PPP2R5B	8.389E-278	0.23927214	0.381	0.27	1.29E-273	AMs
TMEM128	2.681E-277	0.11159539	0.603	0.473	4.121E-273	AMs
NUDT16	3.138E-277	0.14832206	0.561	0.432	4.824E-273	AMs
MOSPD3	3.337E-277	0.13077187	0.549	0.423	5.129E-273	AMs
GPX4	7.324E-276	0.16854344	0.983	0.866	1.126E-271	AMs
TOM1	1.084E-275	0.17073252	0.538	0.413	1.666E-271	AMs
TULP4	1.085E-275	0.11718782	0.621	0.489	1.668E-271	AMs
ICAM2	8.816E-275	0.10176706	0.641	0.506	1.355E-270	AMs
ANAPC15	1.388E-274	0.12434936	0.759	0.609	2.134E-270	AMs
SLC49A4	1.522E-274	0.16478119	0.601	0.473	2.339E-270	AMs
GOLPH3L	1.798E-273	0.17808513	0.452	0.335	2.764E-269	AMs
DENND11	2.434E-273	0.12579397	0.754	0.618	3.742E-269	AMs
B3GNT5	4.251E-273	0.21595477	0.684	0.553	6.534E-269	AMs
SPART	5.405E-273	0.21067719	0.543	0.419	8.309E-269	AMs
LIPA	1.635E-272	0.21785845	0.949	0.803	2.513E-268	AMs
CD33	3.743E-272	0.26856936	0.542	0.418	5.754E-268	AMs
PTCRA	4.181E-272	0.56874194	0.307	0.207	6.426E-268	AMs
GALC	4.823E-272	0.13000753	0.721	0.577	7.414E-268	AMs
PISD	1.599E-271	0.17350979	0.515	0.394	2.458E-267	AMs
NDUFA2	2.04E-271	0.10261445	0.886	0.73	3.136E-267	AMs
GPD2	2.922E-271	0.14314978	0.764	0.614	4.492E-267	AMs
STAT2	3.45E-271	0.13748283	0.841	0.69	5.302E-267	AMs
SIL1	1.087E-270	0.17225998	0.61	0.48	1.671E-266	AMs
GIPC1	1.191E-270	0.11801871	0.617	0.484	1.831E-266	AMs
GABARAP	1.286E-270	0.10603063	0.923	0.78	1.977E-266	AMs
PDXK	3.609E-270	0.14873211	0.956	0.793	5.547E-266	AMs
TRIP6	4.235E-270	0.20699871	0.686	0.548	6.51E-266	AMs
DNASE2B	5.324E-270	0.89435565	0.211	0.125	8.184E-266	AMs
ERLIN2	9.001E-270	0.2419234	0.526	0.404	1.383E-265	AMs
ATP6V0D1	3.057E-269	0.18174249	0.965	0.802	4.699E-265	AMs
CCRL2	3.645E-269	0.33809224	0.395	0.286	5.603E-265	AMs
TDRD7	1.271E-268	0.11995088	0.61	0.481	1.953E-264	AMs
COPZ1	2.986E-266	0.1709551	0.548	0.424	4.59E-262	AMs
NDUFA8	7.889E-266	0.10438974	0.721	0.577	1.213E-261	AMs
ALDH5A1	9.532E-266	0.47260348	0.213	0.126	1.465E-261	AMs
ZBTB7C	4.949E-265	0.6747689	0.259	0.166	7.608E-261	AMs
PCK2	1.049E-264	0.15358417	0.77	0.613	1.612E-260	AMs
LAMTOR2	1.246E-264	0.1288062	0.86	0.705	1.916E-260	AMs
SPINT1	1.289E-264	0.29166523	0.465	0.348	1.981E-260	AMs
SNX3	1.311E-264	0.13023935	0.927	0.779	2.014E-260	AMs
TMEM230	2.521E-264	0.10462816	0.597	0.469	3.876E-260	AMs
MLF1	2.975E-264	0.52679813	0.22	0.132	4.573E-260	AMs

RTL8C	3.55E-264	0.16332695	0.486	0.369	5.457E-260	AMs
RTCB	6.598E-264	0.10666375	0.791	0.645	1.014E-259	AMs
KLHDC8B	1.143E-263	0.51810323	0.343	0.243	1.757E-259	AMs
PLAU	2.783E-263	0.11754025	0.762	0.623	4.277E-259	AMs
MYDGF	9.276E-263	0.16522067	0.962	0.809	1.426E-258	AMs
FNDC3B	1.332E-262	0.12129478	0.932	0.768	2.048E-258	AMs
SYNC	1.571E-262	0.51365051	0.336	0.235	2.415E-258	AMs
NT5C3B	3.585E-262	0.1650241	0.584	0.456	5.51E-258	AMs
ATP6V1D	4.996E-262	0.11868624	0.64	0.509	7.68E-258	AMs
SDCCAG8	7.174E-262	0.14842596	0.697	0.561	1.103E-257	AMs
STARD7	1.364E-261	0.13686378	0.547	0.422	2.096E-257	AMs
ZMAT3	2.204E-261	0.13646315	0.418	0.309	3.388E-257	AMs
SOD2	2.617E-261	0.15934581	0.836	0.696	4.022E-257	AMs
TOMM40	4.254E-261	0.27882304	0.413	0.304	6.54E-257	AMs
SLC12A9	1.293E-260	0.10206036	0.815	0.662	1.987E-256	AMs
GMPR2	1.821E-260	0.11051959	0.534	0.412	2.799E-256	AMs
MX1	2.518E-260	0.12068829	0.844	0.7	3.871E-256	AMs
GTF2H5	4.413E-260	0.16748869	0.506	0.387	6.783E-256	AMs
FOLR3	6.851E-260	0.46300139	0.392	0.287	1.053E-255	AMs
WDR81	1.702E-258	0.11576616	0.66	0.522	2.616E-254	AMs
FBXO6	8.405E-258	0.17639174	0.566	0.443	1.292E-253	AMs
NLK	1.352E-257	0.11678324	0.657	0.527	2.078E-253	AMs
HAVCR2	1.866E-257	0.10184371	0.857	0.704	2.869E-253	AMs
IMPACT	3.748E-257	0.15580494	0.588	0.463	5.762E-253	AMs
GNB2	4.86E-257	0.14066207	0.965	0.822	7.47E-253	AMs
SASH1	6.953E-257	0.41176335	0.375	0.271	1.069E-252	AMs
CAMSAP1	1.28E-256	0.1388989	0.61	0.481	1.967E-252	AMs
CPVL	1.332E-256	0.12514909	0.935	0.77	2.048E-252	AMs
SLC17A5	2.031E-256	0.11005005	0.622	0.494	3.123E-252	AMs
VWA8	8.416E-256	0.11133646	0.4	0.291	1.294E-251	AMs
CTSF	1.619E-255	0.41729926	0.326	0.228	2.489E-251	AMs
ZNF330	5.981E-255	0.14722787	0.558	0.437	9.194E-251	AMs
C5AR2	7.259E-255	0.32116393	0.464	0.353	1.116E-250	AMs
DUSP1	1.322E-254	0.15512697	0.996	0.883	2.032E-250	AMs
CAMKK2	4.233E-254	0.10864082	0.659	0.525	6.506E-250	AMs
DNAAF4	6.63E-254	0.37752536	0.368	0.263	1.019E-249	AMs
CAMP	1.129E-253	0.68728997	0.355	0.254	1.735E-249	AMs
APOL4	3.76E-253	0.22567834	0.621	0.496	5.78E-249	AMs
SPATA20	3.89E-253	0.19812878	0.574	0.449	5.98E-249	AMs
IFNLR1	7.338E-253	0.59114424	0.23	0.144	1.128E-248	AMs
SLC25A20	1.382E-252	0.10442027	0.61	0.484	2.124E-248	AMs
SERINC2	2.017E-252	0.20412085	0.628	0.496	3.1E-248	AMs
SIGLEC7	2.07E-252	0.42254059	0.371	0.268	3.182E-248	AMs
LCP1	5.638E-252	0.11958565	0.995	0.953	8.665E-248	AMs
FGD4	1.046E-251	0.2087281	0.655	0.524	1.608E-247	AMs
B4GALT7	1.854E-251	0.1898565	0.494	0.379	2.85E-247	AMs
IDUA	2.306E-251	0.13992092	0.565	0.442	3.544E-247	AMs
ALG2	6.181E-251	0.11344373	0.569	0.444	9.501E-247	AMs
PEX11A	1.354E-250	0.5401007	0.25	0.16	2.081E-246	AMs
ACOT13	8.574E-250	0.18014305	0.543	0.423	1.318E-245	AMs
IFRD1	2.746E-249	0.27386414	0.678	0.556	4.221E-245	AMs
SLC25A43	7.123E-249	0.15850198	0.454	0.341	1.095E-244	AMs

RASSF8	2.016E-248	0.33157864	0.35	0.248	3.098E-244	AMs
RREB1	2.14E-247	0.10077289	0.719	0.579	3.289E-243	AMs
HES2	2.264E-247	1.58639694	0.092	0.035	3.479E-243	AMs
MIF4GD	6.101E-247	0.1167445	0.693	0.56	9.378E-243	AMs
TTC28	7.515E-247	0.6656148	0.205	0.123	1.155E-242	AMs
CYFIP1	3.591E-246	0.13158763	0.855	0.697	5.52E-242	AMs
BTN2A2	1.702E-245	0.26993936	0.44	0.331	2.616E-241	AMs
CD99L2	2.488E-245	0.19016034	0.547	0.428	3.824E-241	AMs
RGCC	2.141E-244	0.1507687	0.964	0.836	3.291E-240	AMs
SNAP23	2.447E-244	0.10363168	0.394	0.285	3.761E-240	AMs
NR4A1	3.442E-244	0.12363647	0.484	0.367	5.291E-240	AMs
DMXL2	9.204E-244	0.15556179	0.829	0.679	1.415E-239	AMs
KLF8	1.617E-243	0.43254401	0.243	0.155	2.485E-239	AMs
PLD2	2.29E-243	0.18265889	0.511	0.393	3.52E-239	AMs
CPT2	4.557E-243	0.18208716	0.574	0.453	7.005E-239	AMs
PAPSS1	4.809E-243	0.12117609	0.87	0.716	7.393E-239	AMs
FAM8A1	5.052E-243	0.16272009	0.335	0.234	7.766E-239	AMs
EML4	1.112E-242	0.10389547	0.973	0.833	1.709E-238	AMs
RYR1	3.379E-242	0.36017425	0.334	0.236	5.194E-238	AMs
CCL24	8.551E-242	0.91031702	0.311	0.217	1.314E-237	AMs
LAIR1	9.032E-242	0.17470961	0.974	0.799	1.388E-237	AMs
RCOR1	3.117E-241	0.11348052	0.654	0.522	4.791E-237	AMs
DNASE1L1	3.19E-241	0.11282358	0.714	0.579	4.903E-237	AMs
PGK1	4.513E-241	0.14420812	0.638	0.509	6.937E-237	AMs
COL6A1	9.427E-241	1.30353325	0.1	0.04	1.449E-236	AMs
C2orf74	9.718E-241	0.15503522	0.424	0.317	1.494E-236	AMs
SIGLEC1	1.777E-240	0.19991427	0.83	0.682	2.732E-236	AMs
C16orf70	4.336E-240	0.12891941	0.603	0.481	6.665E-236	AMs
DHRS1	5.623E-240	0.20258896	0.333	0.234	8.643E-236	AMs
MIIP	6.096E-240	0.10269733	0.719	0.579	9.371E-236	AMs
DHX58	8.46E-240	0.16594229	0.506	0.391	1.3E-235	AMs
AWAT2	2.044E-238	1.74059967	0.081	0.028	3.142E-234	AMs
ECH1	3.083E-238	0.15348922	0.974	0.828	4.739E-234	AMs
ANXA2	4.362E-238	0.15024598	0.995	0.921	6.705E-234	AMs
CTNNBL1	5.77E-238	0.10443319	0.598	0.472	8.87E-234	AMs
FARP1	2.846E-237	0.21931861	0.338	0.231	4.375E-233	AMs
TMEM94	6.369E-237	0.17293661	0.526	0.411	9.79E-233	AMs
DOK1	2.094E-236	0.19420126	0.522	0.407	3.218E-232	AMs
NDUFA7	4.446E-236	0.10778354	0.585	0.463	6.834E-232	AMs
SELENON	2.268E-235	0.12868603	0.884	0.717	3.486E-231	AMs
LMO2	7.683E-235	0.14084494	0.648	0.512	1.181E-230	AMs
SLC6A12	1.972E-234	0.50210851	0.262	0.172	3.031E-230	AMs
CCDC32	2.76E-234	0.11798568	0.416	0.309	4.242E-230	AMs
RASGEF1B	6.081E-234	0.16077249	0.675	0.546	9.347E-230	AMs
IRF5	6.492E-234	0.22415438	0.508	0.393	9.979E-230	AMs
DUSP27	7.074E-234	0.91086142	0.158	0.086	1.087E-229	AMs
NDUFAF1	1.617E-233	0.22543467	0.43	0.323	2.486E-229	AMs
ALAD	1.645E-233	0.14961891	0.644	0.515	2.528E-229	AMs
TSEN2	3.175E-233	0.37133648	0.343	0.246	4.881E-229	AMs
GRHL1	3.549E-233	0.90906496	0.134	0.067	5.456E-229	AMs
CEACAM21	7.814E-233	0.31660202	0.385	0.284	1.201E-228	AMs
ARHGEF5	1.265E-232	0.49008181	0.256	0.17	1.945E-228	AMs

CLIP2	3.646E-232	0.27997102	0.364	0.262	5.604E-228	AMs
GLTP	4.363E-232	0.10871855	0.528	0.412	6.707E-228	AMs
MECR	6.079E-232	0.31919057	0.357	0.259	9.343E-228	AMs
MDP1	3.657E-231	0.23876291	0.439	0.333	5.621E-227	AMs
OSGIN2	8.262E-231	0.16620296	0.433	0.327	1.27E-226	AMs
ANKRD42	1.02E-230	0.30310189	0.269	0.178	1.568E-226	AMs
EIF2B3	1.378E-230	0.13892154	0.553	0.435	2.119E-226	AMs
HEBP1	6.031E-230	0.15064694	0.625	0.497	9.27E-226	AMs
CAPN1	7.612E-230	0.1004604	0.878	0.721	1.17E-225	AMs
CYP2U1	1.2E-229	0.41930453	0.24	0.154	1.845E-225	AMs
RIOX2	1.418E-229	0.10149307	0.447	0.336	2.18E-225	AMs
MNDA	2.331E-229	0.15448885	0.914	0.766	3.583E-225	AMs
SAMM50	7.622E-229	0.17411818	0.537	0.422	1.172E-224	AMs
PPA2	1.392E-228	0.12174902	0.57	0.452	2.139E-224	AMs
CPPED1	1.784E-228	0.10161493	0.768	0.626	2.742E-224	AMs
ADAP2	4.295E-228	0.12910356	0.593	0.464	6.602E-224	AMs
KPTN	4.494E-228	0.43036573	0.331	0.238	6.907E-224	AMs
SLC29A1	1.927E-227	0.15252499	0.623	0.486	2.962E-223	AMs
SHB	5.453E-227	0.41762474	0.351	0.254	8.382E-223	AMs
TWF2	6.582E-227	0.11810404	0.961	0.82	1.012E-222	AMs
STAC3	2.207E-226	0.2052819	0.627	0.502	3.392E-222	AMs
LHPP	1.687E-225	0.18209824	0.417	0.314	2.593E-221	AMs
BCKDK	3.11E-225	0.11132344	0.743	0.601	4.78E-221	AMs
PTPMT1	4.212E-225	0.35879524	0.343	0.248	6.475E-221	AMs
ATP6V1F	6.527E-225	0.12647907	0.907	0.754	1.003E-220	AMs
CABLES1	7.846E-225	0.36073526	0.388	0.289	1.206E-220	AMs
PCTP	1.54E-224	0.23563559	0.416	0.313	2.368E-220	AMs
RMND1	2.928E-224	0.1441385	0.45	0.344	4.5E-220	AMs
ASB13	9.41E-224	0.44022578	0.309	0.217	1.446E-219	AMs
NPTN	2.049E-223	0.13684952	0.966	0.81	3.15E-219	AMs
DGAT2	4.111E-223	0.43122233	0.283	0.194	6.319E-219	AMs
BTD	1.231E-222	0.17476263	0.467	0.36	1.892E-218	AMs
FUCA2	2.199E-222	0.11765105	0.818	0.665	3.38E-218	AMs
APOC2	9.235E-222	0.66323465	0.403	0.312	1.42E-217	AMs
EPN2	1.601E-221	0.29538519	0.242	0.157	2.461E-217	AMs
OSBPL1A	1.66E-221	0.25379857	0.497	0.387	2.552E-217	AMs
WDR61	2.348E-221	0.14495591	0.499	0.387	3.61E-217	AMs
TSKU	3.72E-221	0.81715853	0.168	0.096	5.718E-217	AMs
HOXB7	1.024E-220	0.78943589	0.153	0.084	1.575E-216	AMs
CEBPA	1.639E-220	0.19858797	0.618	0.496	2.519E-216	AMs
PIGP	1.864E-220	0.18811297	0.367	0.268	2.865E-216	AMs
TNFRSF10B	2.943E-220	0.18019212	0.483	0.376	4.523E-216	AMs
SERPINB1	3.289E-220	0.11387558	0.97	0.84	5.056E-216	AMs
KLF4	5.518E-220	0.14953742	0.89	0.732	8.482E-216	AMs
UNC119	6.596E-220	0.17798417	0.388	0.287	1.014E-215	AMs
BPHL	7.143E-220	0.37938764	0.306	0.216	1.098E-215	AMs
HFE	1.095E-219	0.44560276	0.299	0.209	1.683E-215	AMs
ABHD4	2.569E-219	0.3897758	0.249	0.166	3.949E-215	AMs
DBNDD2	3.257E-219	0.13133949	0.809	0.663	5.007E-215	AMs
SOWAHC	3.315E-219	0.28839379	0.442	0.338	5.095E-215	AMs
HOMEZ	8.061E-219	0.229722	0.349	0.253	1.239E-214	AMs
CREG1	3.268E-218	0.15997378	0.958	0.797	5.024E-214	AMs

ACADSB	3.541E-218	0.18632251	0.283	0.195	5.443E-214	AMs
MOB3C	3.577E-218	0.19783576	0.324	0.229	5.499E-214	AMs
KCNC3	5.019E-218	0.28025336	0.342	0.245	7.715E-214	AMs
MGAT1	6.375E-218	0.14354564	0.979	0.832	9.799E-214	AMs
SPOCD1	8.042E-217	0.52109358	0.339	0.246	1.236E-212	AMs
UBB	1.085E-216	0.13220845	0.959	0.822	1.668E-212	AMs
SERTAD3	1.678E-216	0.10911593	0.514	0.399	2.579E-212	AMs
CTSB	1.742E-216	0.13763781	0.999	0.932	2.677E-212	AMs
PDE9A	3.233E-216	0.8873701	0.103	0.046	4.969E-212	AMs
ESYT1	3.653E-216	0.11005659	0.97	0.839	5.615E-212	AMs
GPR157	2.119E-215	0.16604456	0.364	0.268	3.258E-211	AMs
SCARB2	2.275E-215	0.14860041	0.948	0.784	3.497E-211	AMs
ATP1B3	7.822E-214	0.14969164	0.779	0.635	1.202E-209	AMs
PWWP2B	1.279E-213	0.15025034	0.43	0.326	1.966E-209	AMs
CNDP2	3.695E-213	0.14359978	0.972	0.82	5.68E-209	AMs
PHYH	1.06E-212	0.14704422	0.575	0.457	1.63E-208	AMs
OAT	7.833E-212	0.25901884	0.295	0.207	1.204E-207	AMs
CTSH	5.584E-211	0.15569428	0.992	0.871	8.583E-207	AMs
UNC93B1	1.329E-210	0.14716325	0.95	0.787	2.042E-206	AMs
SLC45A4	2.01E-210	0.28646677	0.325	0.234	3.089E-206	AMs
UAP1	3.775E-210	0.15679338	0.495	0.389	5.803E-206	AMs
GSTP1	5.215E-210	0.1083781	0.944	0.795	8.015E-206	AMs
ROGDI	2.471E-209	0.13142487	0.698	0.566	3.798E-205	AMs
FARP2	3.087E-209	0.16585462	0.395	0.297	4.746E-205	AMs
COLGALT1	5.498E-209	0.1807979	0.989	0.825	8.451E-205	AMs
HOMER1	3.505E-208	0.50073098	0.162	0.092	5.387E-204	AMs
ZNF358	1.016E-207	0.28024714	0.343	0.251	1.561E-203	AMs
RRM2B	2.032E-207	0.32144404	0.334	0.243	3.124E-203	AMs
FDX2	7.287E-207	0.25911325	0.35	0.256	1.12E-202	AMs
C2CD2L	2.321E-205	0.11969166	0.521	0.412	3.568E-201	AMs
H6PD	2.592E-205	0.12766137	0.546	0.433	3.984E-201	AMs
GBGT1	4.131E-205	0.17837649	0.438	0.331	6.349E-201	AMs
GBP1	6.629E-205	0.192702	0.705	0.58	1.019E-200	AMs
CLUH	9.058E-205	0.1358515	0.521	0.41	1.392E-200	AMs
CCDC9B	1.094E-204	0.58543087	0.188	0.115	1.682E-200	AMs
FCRL1	1.296E-204	0.74903606	0.193	0.12	1.992E-200	AMs
TDRD9	6.746E-204	0.95323152	0.112	0.054	1.037E-199	AMs
ALPK1	1.159E-203	0.29284478	0.385	0.288	1.781E-199	AMs
RNH1	1.472E-203	0.17183876	0.99	0.849	2.262E-199	AMs
NKIRAS1	1.981E-203	0.2545883	0.351	0.259	3.046E-199	AMs
LAMTOR5	3.879E-203	0.13193868	0.464	0.359	5.963E-199	AMs
DEFB1	4.278E-203	0.48273412	0.437	0.346	6.575E-199	AMs
IFT43	1.834E-202	0.26824331	0.306	0.218	2.819E-198	AMs
TMEM53	2.803E-202	0.29078179	0.396	0.3	4.309E-198	AMs
KDM1B	3.288E-202	0.10265993	0.592	0.474	5.054E-198	AMs
AIG1	7.993E-202	0.27540823	0.373	0.279	1.229E-197	AMs
ARMCX2	9.871E-202	0.56865836	0.209	0.133	1.517E-197	AMs
PPP1R13B	1.609E-201	0.31564357	0.173	0.104	2.473E-197	AMs
PCCB	9.444E-201	0.16585886	0.454	0.351	1.452E-196	AMs
SIRPD	1.111E-200	0.62046942	0.206	0.132	1.707E-196	AMs
CIPC	1.463E-200	0.26743024	0.295	0.206	2.248E-196	AMs
PRKN	2.012E-200	0.87836802	0.124	0.064	3.092E-196	AMs

KIF13A	3.436E-200	0.14322698	0.613	0.497	5.281E-196	AMs
MICOS13	4.045E-200	0.11198458	0.453	0.35	6.218E-196	AMs
ABAT	4.242E-200	0.18733184	0.396	0.296	6.521E-196	AMs
NQO1	7.821E-200	0.46180384	0.215	0.139	1.202E-195	AMs
TRAPPC5	1.248E-199	0.13981547	0.899	0.747	1.919E-195	AMs
RBM11	2.817E-199	0.98208896	0.112	0.055	4.33E-195	AMs
SOCS6	1.627E-198	0.12486802	0.683	0.557	2.501E-194	AMs
ANO10	2.637E-198	0.15832542	0.453	0.352	4.054E-194	AMs
ABCG2	8.579E-198	0.20909564	0.49	0.38	1.319E-193	AMs
ATP6V0D2	9.658E-198	1.29688418	0.09	0.039	1.484E-193	AMs
FAM131A	3.109E-197	0.27639197	0.296	0.209	4.779E-193	AMs
SLC35E3	4.633E-197	0.12186237	0.448	0.346	7.121E-193	AMs
FAM53C	5.658E-197	0.10622659	0.489	0.385	8.697E-193	AMs
RNPEP	7.628E-197	0.12482956	0.923	0.757	1.172E-192	AMs
TFEC	4.624E-196	0.12657945	0.82	0.675	7.108E-192	AMs
CYB5D2	9.358E-196	0.33591225	0.28	0.197	1.438E-191	AMs
SNAPC2	2.132E-195	0.14715002	0.405	0.309	3.277E-191	AMs
SHISA4	2.383E-195	0.60480084	0.192	0.121	3.663E-191	AMs
VAMP8	4.128E-195	0.15666287	0.985	0.866	6.345E-191	AMs
CCDC106	5.73E-195	0.47578743	0.277	0.196	8.807E-191	AMs
GORASP1	2.582E-194	0.15340795	0.52	0.414	3.969E-190	AMs
TMEM254	1.652E-193	0.26486766	0.331	0.242	2.539E-189	AMs
SPIDR	1.972E-193	0.12199138	0.532	0.425	3.032E-189	AMs
CHST14	3.026E-193	0.13783875	0.57	0.459	4.651E-189	AMs
SYK	3.143E-193	0.13294241	0.96	0.792	4.831E-189	AMs
CCDC88A	1.552E-192	0.10787637	0.935	0.767	2.385E-188	AMs
TST	2.394E-192	0.44793707	0.28	0.2	3.68E-188	AMs
GNA11	5.003E-192	0.25601124	0.405	0.308	7.69E-188	AMs
SLITRK4	9.88E-192	0.75849867	0.153	0.088	1.519E-187	AMs
ISG15	4.013E-191	0.11182803	0.839	0.703	6.169E-187	AMs
CXCL3	1.388E-190	0.12994896	0.703	0.572	2.133E-186	AMs
NFKBIE	3.253E-190	0.13164966	0.452	0.353	5.001E-186	AMs
CLEC6A	8.321E-190	0.43272053	0.318	0.233	1.279E-185	AMs
USP30	1.387E-189	0.22496987	0.35	0.26	2.133E-185	AMs
TKFC	5.127E-189	0.17749489	0.35	0.259	7.88E-185	AMs
SPR	1.031E-187	0.18340582	0.577	0.464	1.585E-183	AMs
COX5B	1.362E-187	0.14087089	0.452	0.355	2.093E-183	AMs
SNX21	1.485E-187	0.53610386	0.195	0.124	2.283E-183	AMs
LDHD	1.977E-187	0.46812732	0.275	0.194	3.039E-183	AMs
LTBR	5.332E-187	0.10832285	0.798	0.653	8.196E-183	AMs
RASGRP4	7.289E-187	0.18720144	0.459	0.357	1.12E-182	AMs
SDC3	1.99E-186	0.2675804	0.38	0.286	3.059E-182	AMs
PTPN18	2.006E-186	0.14594125	0.591	0.475	3.084E-182	AMs
AGRN	4E-186	0.17864865	0.586	0.474	6.148E-182	AMs
PLXDC2	8.625E-186	0.13661867	0.987	0.821	1.326E-181	AMs
WDR1	2.537E-185	0.1419867	0.484	0.384	3.899E-181	AMs
DLX3	2.697E-185	1.59670713	0.06	0.019	4.145E-181	AMs
UBXN11	3.568E-185	0.10697996	0.816	0.674	5.485E-181	AMs
FAH	3.863E-185	0.18963971	0.397	0.303	5.938E-181	AMs
TP53INP2	5.306E-185	0.23818066	0.284	0.201	8.156E-181	AMs
SCCPDH	1.915E-184	0.11886862	0.882	0.718	2.944E-180	AMs
LAPTM5	1.983E-184	0.10497092	0.998	0.978	3.048E-180	AMs

CSPG4	2.287E-184	0.53130258	0.212	0.138	3.516E-180	AMs
FER	3.224E-184	0.11945171	0.467	0.366	4.955E-180	AMs
FLNA	3.553E-184	0.12960733	0.988	0.883	5.462E-180	AMs
ACADS	9.436E-184	0.1453671	0.512	0.406	1.45E-179	AMs
NSDHL	1.916E-182	0.13686127	0.41	0.317	2.945E-178	AMs
WWTR1	3.379E-182	0.74291835	0.11	0.056	5.194E-178	AMs
SBF2	5.337E-182	0.20919571	0.408	0.315	8.203E-178	AMs
SAV1	5.8E-182	0.13533716	0.516	0.411	8.915E-178	AMs
CUEDC1	9.073E-182	0.26182457	0.428	0.333	1.395E-177	AMs
ST3GAL6	9.965E-182	0.16015492	0.559	0.452	1.532E-177	AMs
OCRL	1.51E-181	0.20786938	0.485	0.384	2.321E-177	AMs
PLEKHB1	2.377E-180	0.34611136	0.209	0.138	3.654E-176	AMs
DAGLA	6.003E-180	0.30107484	0.37	0.281	9.227E-176	AMs
TRIO	8.812E-180	0.11346809	0.812	0.671	1.355E-175	AMs
MEFV	1.062E-179	0.1636764	0.481	0.381	1.632E-175	AMs
HILPDA	1.274E-179	0.41767752	0.241	0.167	1.958E-175	AMs
TMEM38B	1.313E-178	0.1180999	0.5	0.395	2.018E-174	AMs
SLC49A3	2.24E-178	0.18810349	0.57	0.46	3.443E-174	AMs
WLS	3.796E-178	0.16241029	0.554	0.44	5.834E-174	AMs
DNASE2	3.913E-178	0.13200109	0.946	0.777	6.015E-174	AMs
ATG4A	1.1E-177	0.19529616	0.347	0.261	1.691E-173	AMs
SH3BP4	1.499E-177	0.15214326	0.578	0.466	2.304E-173	AMs
PCYOX1	4.098E-177	0.16821134	0.315	0.232	6.299E-173	AMs
MAP7D1	8.858E-177	0.10222339	0.97	0.828	1.362E-172	AMs
WDFY3	1.082E-176	0.20692771	0.424	0.329	1.663E-172	AMs
SENP3	1.839E-176	0.20339416	0.511	0.412	2.826E-172	AMs
PRSS21	4.29E-176	0.72526069	0.247	0.175	6.594E-172	AMs
ACP6	7.157E-176	0.24667906	0.244	0.168	1.1E-171	AMs
APEX2	1.273E-175	0.11917187	0.56	0.452	1.957E-171	AMs
NINJ2	1.866E-175	0.20104002	0.329	0.245	2.868E-171	AMs
EMILIN2	1.878E-175	0.15772564	0.962	0.797	2.887E-171	AMs
BST1	1.208E-174	0.10485957	0.795	0.651	1.857E-170	AMs
JUNB	1.242E-174	0.14369331	0.981	0.897	1.91E-170	AMs
PVALB	1.563E-174	0.80148229	0.147	0.087	2.402E-170	AMs
SLC2A6	3.625E-174	0.39949954	0.305	0.224	5.572E-170	AMs
GRN	3.168E-173	0.18114107	0.999	0.908	4.869E-169	AMs
TP53I3	1.132E-172	0.24524103	0.484	0.387	1.74E-168	AMs
DOCK7	2.345E-172	0.11385749	0.503	0.402	3.605E-168	AMs
ZSCAN18	5.451E-172	0.21222682	0.218	0.146	8.378E-168	AMs
CDKL5	6.69E-172	0.35974202	0.246	0.171	1.028E-167	AMs
SH3TC1	1.455E-171	0.13219138	0.523	0.417	2.237E-167	AMs
GPA33	4.804E-171	0.27230746	0.426	0.335	7.384E-167	AMs
MT2A	4.918E-171	0.28731049	0.461	0.361	7.56E-167	AMs
UROD	5.126E-171	0.10121501	0.651	0.531	7.879E-167	AMs
PLAGL1	9.11E-171	0.14752801	0.507	0.405	1.4E-166	AMs
RAB34	1.888E-170	0.11899726	0.63	0.511	2.902E-166	AMs
ACTN1	2.123E-170	0.16485773	0.98	0.812	3.263E-166	AMs
C11orf1	2.201E-170	0.18608802	0.278	0.2	3.383E-166	AMs
NRP1	3.115E-170	0.1201602	0.855	0.7	4.788E-166	AMs
PRKAR2B	7.151E-170	0.23639064	0.395	0.306	1.099E-165	AMs
PSEN2	8.139E-170	0.23038705	0.355	0.266	1.251E-165	AMs
ZNF316	8.52E-170	0.11551146	0.364	0.277	1.31E-165	AMs

ENPP5	2.165E-169	0.9545592	0.083	0.037	3.327E-165	AMs
SLC66A2	1.361E-168	0.10790597	0.512	0.411	2.091E-164	AMs
FRRS1	6.832E-168	0.3792399	0.285	0.207	1.05E-163	AMs
KLHL18	8.361E-167	0.12251716	0.424	0.331	1.285E-162	AMs
S100A16	1.356E-166	0.4738826	0.129	0.073	2.084E-162	AMs
RGL3	2.511E-166	0.7808058	0.134	0.077	3.859E-162	AMs
UACA	4.176E-166	0.32122136	0.361	0.276	6.418E-162	AMs
TMEM86A	1.372E-165	0.24201862	0.448	0.357	2.109E-161	AMs
COTL1	1.39E-165	0.10187864	0.966	0.839	2.136E-161	AMs
MDH1	2.963E-165	0.12837468	0.579	0.468	4.555E-161	AMs
SPRY1	3.946E-165	0.18448379	0.246	0.172	6.065E-161	AMs
GRB2	4.896E-165	0.13999041	0.988	0.848	7.525E-161	AMs
BEST1	3.228E-164	0.23284272	0.351	0.264	4.961E-160	AMs
GDPGP1	6.353E-164	0.37781134	0.222	0.152	9.765E-160	AMs
FAM118B	1.265E-163	0.10839593	0.474	0.379	1.945E-159	AMs
P4HA2	1.962E-163	0.2948133	0.374	0.289	3.015E-159	AMs
PLK2	3.273E-163	0.56054935	0.191	0.125	5.031E-159	AMs
CLN8	8.455E-163	0.1076423	0.516	0.416	1.3E-158	AMs
VNN2	8.539E-163	0.29258662	0.248	0.176	1.313E-158	AMs
FAM214B	1.929E-162	0.20946287	0.362	0.276	2.964E-158	AMs
CENPBD1	4.317E-162	0.25333853	0.302	0.223	6.636E-158	AMs
AC067752.1	8.614E-162	0.78932171	0.136	0.08	1.324E-157	AMs
ACTA2	9.467E-162	0.20624637	0.182	0.116	1.455E-157	AMs
GAS2L3	4.291E-161	0.21786577	0.43	0.338	6.596E-157	AMs
NEO1	8.38E-161	0.55691508	0.073	0.031	1.288E-156	AMs
BMPR1A	1.278E-160	0.18581249	0.165	0.104	1.965E-156	AMs
TRIM34	2.225E-160	0.11237233	0.426	0.334	3.421E-156	AMs
MMAB	2.695E-160	0.12090265	0.334	0.254	4.143E-156	AMs
HYAL2	1.366E-159	0.10820033	0.589	0.479	2.099E-155	AMs
ABCD1	7.524E-159	0.18166317	0.505	0.41	1.157E-154	AMs
SSH3	8.833E-159	0.23631506	0.289	0.212	1.358E-154	AMs
CCDC125	2.663E-158	0.14008222	0.415	0.326	4.093E-154	AMs
DLC1	4.095E-158	0.37202975	0.279	0.204	6.294E-154	AMs
HEXA	7.656E-158	0.11079657	0.964	0.806	1.177E-153	AMs
MINPP1	9.632E-157	0.17158149	0.361	0.277	1.481E-152	AMs
FHL1	1.838E-156	0.4315493	0.229	0.16	2.826E-152	AMs
RAB24	2.265E-156	0.15151784	0.274	0.198	3.482E-152	AMs
ST6GALNAC4	5.729E-156	0.19047969	0.316	0.237	8.806E-152	AMs
SCRN2	1.778E-155	0.13081557	0.258	0.184	2.733E-151	AMs
KLHL8	5.146E-155	0.11481575	0.398	0.312	7.91E-151	AMs
ODF3L1	5.25E-155	0.43918056	0.235	0.166	8.069E-151	AMs
LZTS2	6.904E-155	0.12791419	0.41	0.323	1.061E-150	AMs
OSBPL10	3.101E-154	0.38432376	0.275	0.202	4.767E-150	AMs
HSD17B12	6.069E-154	0.13957517	0.4	0.312	9.329E-150	AMs
FECH	1.768E-153	0.10874764	0.451	0.361	2.718E-149	AMs
DHX32	9.21E-153	0.20108938	0.406	0.321	1.416E-148	AMs
MOGAT1	2.427E-152	0.50462648	0.234	0.165	3.731E-148	AMs
TPD52L1	3.748E-152	0.62118899	0.158	0.1	5.762E-148	AMs
VWA7	4.683E-152	0.75224547	0.113	0.063	7.199E-148	AMs
ABHD6	1.508E-150	0.14860296	0.312	0.234	2.318E-146	AMs
BACE2	7.203E-150	0.68918422	0.128	0.075	1.107E-145	AMs
DZIP1L	1.551E-149	0.25586926	0.461	0.372	2.384E-145	AMs

CAVIN4	3.074E-149	0.96695311	0.093	0.048	4.724E-145	AMs
PPFIBP2	2.063E-148	0.15487543	0.472	0.379	3.172E-144	AMs
FAM3A	2.173E-147	0.15122439	0.328	0.25	3.341E-143	AMs
NSUN7	5.791E-147	0.27361245	0.179	0.117	8.901E-143	AMs
AKAP12	7.308E-147	1.03793919	0.088	0.044	1.123E-142	AMs
AAMDC	1.657E-146	0.14780687	0.356	0.273	2.547E-142	AMs
SLC16A5	1.683E-146	0.25956739	0.31	0.234	2.587E-142	AMs
TNS3	8.125E-146	0.10451023	0.825	0.679	1.249E-141	AMs
RWDD2A	5.081E-145	0.48139969	0.17	0.112	7.81E-141	AMs
TMEM63B	6.404E-145	0.10780801	0.344	0.265	9.844E-141	AMs
ELOVL7	7.342E-145	0.90009655	0.081	0.039	1.129E-140	AMs
TLR8	2.765E-144	0.14517712	0.434	0.343	4.25E-140	AMs
QPRT	1.597E-143	0.35822325	0.242	0.174	2.454E-139	AMs
GBE1	3.152E-143	0.10458739	0.48	0.389	4.844E-139	AMs
ATP9A	5.924E-143	0.78173408	0.092	0.048	9.106E-139	AMs
GHDC	2.306E-142	0.11298632	0.347	0.267	3.545E-138	AMs
IL17RC	2.391E-142	0.20800361	0.401	0.317	3.675E-138	AMs
HCCS	2.95E-142	0.10882276	0.444	0.356	4.535E-138	AMs
CCDC170	6.774E-142	0.25061941	0.2	0.135	1.041E-137	AMs
ZDHHC7	6.975E-142	0.14441681	0.395	0.312	1.072E-137	AMs
SYTL4	2.162E-141	0.64376213	0.14	0.086	3.323E-137	AMs
DISP1	9.537E-141	0.41491332	0.199	0.137	1.466E-136	AMs
PPARGC1B	1.112E-140	0.27928682	0.27	0.198	1.71E-136	AMs
HIC2	2.554E-140	0.14502809	0.285	0.213	3.925E-136	AMs
VPS37C	3.519E-140	0.17068276	0.459	0.371	5.408E-136	AMs
GPCPD1	3.929E-140	0.11368426	0.981	0.834	6.039E-136	AMs
GNPDA1	6.568E-140	0.108994	0.43	0.345	1.01E-135	AMs
RGMB	8.254E-140	0.36116896	0.218	0.155	1.269E-135	AMs
FRMPD1	3.6E-139	0.99884583	0.09	0.046	5.533E-135	AMs
HPR	1.048E-138	0.60889619	0.175	0.117	1.611E-134	AMs
ABHD8	1.495E-138	0.14174425	0.504	0.411	2.298E-134	AMs
MUL1	3.042E-137	0.12294898	0.385	0.304	4.675E-133	AMs
WDR49	8.331E-137	0.23607991	0.286	0.214	1.281E-132	AMs
EIF4EBP3	1.067E-136	0.18514895	0.295	0.222	1.641E-132	AMs
HSPB1	1.483E-136	0.12073792	0.979	0.832	2.279E-132	AMs
THNSL2	3.245E-136	0.51603312	0.119	0.069	4.988E-132	AMs
SOWAHD	3.215E-135	0.30338719	0.296	0.225	4.941E-131	AMs
TLCD4	3.257E-135	0.69563034	0.128	0.078	5.006E-131	AMs
MID2	6.425E-135	0.49088224	0.069	0.031	9.877E-131	AMs
FAM241B	7.881E-135	0.47337041	0.127	0.078	1.211E-130	AMs
ENTPD5	1.247E-134	0.18570359	0.265	0.197	1.917E-130	AMs
STAP1	1.381E-134	0.46787756	0.101	0.055	2.122E-130	AMs
CC2D2A	2.015E-134	0.22538928	0.272	0.201	3.098E-130	AMs
TRPV4	2.67E-134	0.26794134	0.349	0.273	4.103E-130	AMs
FCGR3B	1.232E-133	0.18140004	0.347	0.27	1.893E-129	AMs
PHB	2.572E-133	0.13722395	0.366	0.288	3.953E-129	AMs
LAMA1	3.863E-133	1.10595993	0.081	0.041	5.938E-129	AMs
PKD2L1	7.627E-133	0.56475977	0.184	0.126	1.172E-128	AMs
DYNC2LI1	7.96E-133	0.30196372	0.214	0.151	1.223E-128	AMs
KCNMA1	1.004E-132	0.26657129	0.289	0.215	1.544E-128	AMs
PFKFB2	4.471E-132	0.26685087	0.295	0.224	6.873E-128	AMs
S100A4	4.911E-132	0.12062485	0.994	0.939	7.549E-128	AMs

FAT3	3.548E-131	1.1274205	0.065	0.029	5.454E-127	AMs
GDA	3.689E-131	1.23472717	0.049	0.017	5.67E-127	AMs
TUBA1A	4.43E-131	0.13952386	0.314	0.241	6.809E-127	AMs
NFIA	4.588E-131	0.25907442	0.288	0.22	7.052E-127	AMs
TMCC1	8.042E-131	0.15002895	0.264	0.196	1.236E-126	AMs
IPO13	8.113E-131	0.10610218	0.401	0.32	1.247E-126	AMs
ZNF438	9.988E-131	0.12977098	0.445	0.362	1.535E-126	AMs
USP40	8E-130	0.11830439	0.354	0.277	1.23E-125	AMs
TENT5A	2.507E-129	0.13905899	0.436	0.352	3.854E-125	AMs
SCARB1	3.561E-129	0.18886348	0.4	0.322	5.474E-125	AMs
TFCP2L1	4.57E-129	0.4246486	0.176	0.12	7.024E-125	AMs
ALG14	1.133E-128	0.21790759	0.306	0.234	1.742E-124	AMs
RILPL1	1.977E-128	0.305146	0.145	0.093	3.038E-124	AMs
PEX10	6.689E-128	0.16605888	0.349	0.273	1.028E-123	AMs
FXN	3.092E-127	0.1339583	0.266	0.198	4.753E-123	AMs
TATDN3	3.66E-127	0.13779747	0.289	0.219	5.626E-123	AMs
FAM151B	8.744E-127	0.34135217	0.266	0.201	1.344E-122	AMs
PLAAT1	5.258E-126	1.21035997	0.053	0.021	8.081E-122	AMs
CFB	1.159E-125	0.42244419	0.195	0.136	1.781E-121	AMs
EPB41L5	1.289E-125	0.26955881	0.132	0.083	1.982E-121	AMs
ADORA1	7.144E-125	0.29520005	0.297	0.229	1.098E-120	AMs
SPRYD4	9.311E-125	0.13878489	0.246	0.181	1.431E-120	AMs
WWC2	2.029E-124	0.32842008	0.236	0.173	3.119E-120	AMs
PDGFD	4.41E-124	0.2604547	0.333	0.258	6.778E-120	AMs
SLC30A1	4.626E-124	0.13638192	0.225	0.161	7.111E-120	AMs
NMNAT1	6.732E-124	0.17850716	0.293	0.223	1.035E-119	AMs
OR6K3	1.082E-123	1.33483875	0.057	0.025	1.663E-119	AMs
RTN2	1.79E-123	0.17928533	0.339	0.264	2.751E-119	AMs
ARHGAP10	2.631E-123	0.1187194	0.481	0.396	4.045E-119	AMs
FTCDNL1	7.857E-123	0.39368707	0.205	0.145	1.208E-118	AMs
MYL6	2.037E-122	0.10077441	0.988	0.877	3.131E-118	AMs
CMTM2	2.959E-122	0.76870462	0.058	0.025	4.548E-118	AMs
RBM24	5.826E-122	1.6279575	0.039	0.012	8.955E-118	AMs
HTRA4	7.999E-121	0.49543512	0.147	0.096	1.23E-116	AMs
SYNPO	4.051E-120	1.20660282	0.063	0.029	6.227E-116	AMs
ADCY9	9.635E-120	0.13856122	0.273	0.208	1.481E-115	AMs
ZNF552	2.565E-119	0.11164121	0.243	0.18	3.942E-115	AMs
NLRX1	9.593E-119	0.17068511	0.281	0.216	1.475E-114	AMs
TSPAN32	1.009E-118	0.13648488	0.172	0.118	1.551E-114	AMs
MARCO	1.127E-118	0.1532274	0.997	0.858	1.733E-114	AMs
TPP1	1.669E-118	0.12062942	0.991	0.858	2.565E-114	AMs
QPCTL	6.534E-118	0.13298336	0.255	0.192	1.004E-113	AMs
USP6NL	7.669E-118	0.19642633	0.316	0.246	1.179E-113	AMs
YPEL4	4.845E-117	0.86721915	0.079	0.042	7.447E-113	AMs
JPH4	1.613E-116	0.27112942	0.337	0.268	2.479E-112	AMs
CYB5R2	4.701E-116	0.31243625	0.268	0.205	7.226E-112	AMs
PVR	1.377E-115	0.13370079	0.548	0.456	2.116E-111	AMs
TMED1	5.209E-115	0.10076432	0.404	0.327	8.007E-111	AMs
RMDN2	5.748E-115	0.26101881	0.185	0.129	8.835E-111	AMs
SATB2	9.896E-115	0.51730407	0.151	0.101	1.521E-110	AMs
LOXL3	1.135E-114	0.38190139	0.232	0.171	1.745E-110	AMs
AKR1E2	2.913E-114	0.40681653	0.182	0.129	4.478E-110	AMs

ZDHHC9	4.156E-114	0.28304899	0.208	0.151	6.388E-110	AMs
FAIM	1.918E-113	0.129862	0.227	0.168	2.948E-109	AMs
FCRL2	2.229E-113	0.57409145	0.11	0.067	3.427E-109	AMs
RUBCNL	5.345E-113	0.20884911	0.238	0.178	8.216E-109	AMs
APBB2	9.698E-113	0.54709732	0.14	0.092	1.491E-108	AMs
INKA2	1.15E-112	0.15423153	0.299	0.233	1.767E-108	AMs
ANKRD6	5.964E-112	0.39641076	0.149	0.1	9.168E-108	AMs
PICK1	6.704E-112	0.14127591	0.399	0.325	1.03E-107	AMs
TEC	8.798E-112	0.22872801	0.296	0.231	1.352E-107	AMs
RHOD	1.164E-111	0.4716522	0.117	0.073	1.789E-107	AMs
CDC14B	1.491E-111	0.40390427	0.065	0.032	2.292E-107	AMs
TUBG2	9.695E-111	0.30831805	0.154	0.104	1.49E-106	AMs
SOX13	9.719E-111	0.37640972	0.193	0.139	1.494E-106	AMs
PLEKHA4	1.472E-110	0.82858208	0.084	0.047	2.262E-106	AMs
LYRM9	1.958E-110	0.26380237	0.094	0.054	3.01E-106	AMs
FBXL2	2.071E-110	1.16212985	0.046	0.018	3.184E-106	AMs
AMZ1	1.337E-109	0.55176551	0.154	0.106	2.056E-105	AMs
TCTN1	4.054E-108	0.17069698	0.276	0.213	6.231E-104	AMs
NLN	5.775E-108	0.1492807	0.316	0.247	8.877E-104	AMs
MRAS	6.312E-108	0.16580821	0.37	0.298	9.702E-104	AMs
SEPTIN10	2.536E-107	0.13794813	0.434	0.356	3.898E-103	AMs
SRGN	6.866E-107	0.10477352	0.991	0.93	1.055E-102	AMs
ZNF229	3.63E-106	0.62736176	0.112	0.07	5.58E-102	AMs
COL6A3	5.983E-106	0.61841471	0.077	0.042	9.196E-102	AMs
PKIG	1.51E-105	0.25254636	0.178	0.127	2.321E-101	AMs
SSPN	5.687E-105	0.64035784	0.111	0.069	8.742E-101	AMs
CLMN	7.398E-105	0.23137208	0.248	0.188	1.137E-100	AMs
DLX4	5.027E-104	0.7683929	0.071	0.037	7.727E-100	AMs
RYK	6.915E-104	0.10272769	0.188	0.135	1.063E-99	AMs
PDE6G	2.809E-103	0.3249496	0.221	0.165	4.317E-99	AMs
FN3K	3.01E-103	0.51873244	0.108	0.067	4.627E-99	AMs
DUSP8	3.836E-103	0.56234344	0.072	0.038	5.897E-99	AMs
DMD	2.845E-102	0.57882268	0.071	0.038	4.3736E-98	AMs
ARHGEF11	3.256E-102	0.10041318	0.485	0.404	5.0054E-98	AMs
FCSK	3.345E-102	0.22269931	0.185	0.132	5.1414E-98	AMs
SLC27A1	8.363E-102	0.19539899	0.271	0.211	1.2854E-97	AMs
C3	9.755E-102	0.25997601	0.138	0.093	1.4995E-97	AMs
CTSZ	1.096E-101	0.10753742	0.997	0.882	1.6846E-97	AMs
TMEM39B	1.89E-101	0.12029168	0.261	0.201	2.9056E-97	AMs
C20orf27	2.683E-101	0.28280426	0.223	0.168	4.1244E-97	AMs
MYO7B	2.778E-101	0.30555158	0.268	0.209	4.2703E-97	AMs
DDIAS	3.193E-101	0.18400965	0.376	0.309	4.9076E-97	AMs
TBC1D30	4.645E-101	0.56767265	0.106	0.066	7.1395E-97	AMs
MYO1A	9.578E-101	0.68299534	0.105	0.065	1.4722E-96	AMs
HS1BP3	9.686E-101	0.19945315	0.269	0.209	1.4888E-96	AMs
PEX5	2.79E-100	0.11322817	0.195	0.142	4.2878E-96	AMs
RNF217	4E-100	0.15864286	0.338	0.27	6.1481E-96	AMs
DTWD2	4.633E-100	0.26417473	0.17	0.12	7.1213E-96	AMs
CBR1	1.027E-99	0.109175	0.414	0.341	1.5789E-95	AMs
SLC9A3R2	3.837E-99	0.19860909	0.117	0.075	5.8986E-95	AMs
TRPM4	1.1185E-98	0.27354748	0.253	0.195	1.7193E-94	AMs
CAPN3	1.3716E-98	0.2363199	0.294	0.232	2.1083E-94	AMs

GLIS3	5.4261E-98	0.68026892	0.054	0.026	8.3404E-94	AMs
ENG	1.4956E-97	0.10069899	0.977	0.815	2.2989E-93	AMs
TCEAL1	3.9508E-97	0.13482623	0.372	0.303	6.0727E-93	AMs
ARSD	7.1743E-97	0.22119719	0.239	0.182	1.1028E-92	AMs
MFSD2A	1.2769E-96	0.59429544	0.107	0.067	1.9626E-92	AMs
SH3PXD2B	2.9064E-96	0.31360765	0.218	0.165	4.4675E-92	AMs
IFI30	7.8279E-96	0.13018123	0.999	0.912	1.2032E-91	AMs
SULT1A1	1.1208E-95	0.33213933	0.17	0.122	1.7227E-91	AMs
DYRK4	6.909E-95	0.14247569	0.267	0.209	1.062E-90	AMs
TPRG1	9.8265E-95	0.3359106	0.165	0.118	1.5104E-90	AMs
P2RY1	9.9804E-95	0.20475107	0.289	0.23	1.5341E-90	AMs
SLC1A3	4.593E-94	0.29886744	0.297	0.239	7.0598E-90	AMs
STS	2.8333E-93	0.10620066	0.336	0.271	4.355E-89	AMs
SH2D4B	6.0048E-93	0.80142797	0.077	0.044	9.23E-89	AMs
FAM189A2	1.694E-92	1.40004358	0.032	0.01	2.6039E-88	AMs
UNC13B	6.3595E-92	0.25825259	0.237	0.183	9.7752E-88	AMs
MAP3K7CL	7.1988E-92	0.28237686	0.213	0.161	1.1065E-87	AMs
VPS18	1.0459E-91	0.18068675	0.248	0.193	1.6077E-87	AMs
ZNF117	4.3486E-91	0.13122623	0.161	0.114	6.6843E-87	AMs
PADI2	2.2479E-90	0.28958346	0.19	0.141	3.4552E-86	AMs
ZNF717	2.6759E-89	0.25548875	0.151	0.106	4.1131E-85	AMs
SLC16A10	3.8982E-89	0.36202162	0.195	0.146	5.992E-85	AMs
CRACR2B	4.1925E-89	0.15653829	0.273	0.218	6.4442E-85	AMs
AR	7.1174E-89	0.53793842	0.116	0.077	1.094E-84	AMs
BBS5	9.8924E-89	0.40493301	0.118	0.078	1.5206E-84	AMs
CHML	1.3667E-88	0.24577471	0.285	0.227	2.1007E-84	AMs
CXADR	2.4855E-88	0.75084549	0.068	0.038	3.8205E-84	AMs
YES1	4.1036E-88	0.11356302	0.206	0.155	6.3076E-84	AMs
PNPLA7	5.4939E-87	0.11693287	0.157	0.112	8.4446E-83	AMs
ECHDC3	1.3874E-86	0.23328068	0.22	0.168	2.1326E-82	AMs
RPH3A	2.1E-86	1.28352889	0.035	0.013	3.228E-82	AMs
IFT46	5.946E-86	0.11169784	0.197	0.148	9.1396E-82	AMs
CEP170B	7.9559E-86	0.2193566	0.177	0.132	1.2229E-81	AMs
S100A1	8.6001E-86	1.03615935	0.112	0.074	1.3219E-81	AMs
SYNJ2BP	1.9285E-85	0.10648932	0.274	0.217	2.9643E-81	AMs
SUOX	6.5667E-85	0.38447983	0.161	0.117	1.0094E-80	AMs
CAB39L	2.8563E-84	0.14068758	0.107	0.07	4.3904E-80	AMs
CTIF	6.2686E-84	0.15516325	0.228	0.175	9.6355E-80	AMs
NXF3	7.1982E-84	0.81983617	0.063	0.035	1.1064E-79	AMs
PTK2	1.106E-83	0.27006432	0.148	0.105	1.7E-79	AMs
MPZL1	1.474E-83	0.1019767	0.349	0.286	2.2657E-79	AMs
CAMKK1	2.2281E-83	0.13258972	0.214	0.163	3.4247E-79	AMs
ORAI3	1.4669E-82	0.14253715	0.238	0.185	2.2547E-78	AMs
ZNF223	2.1907E-82	0.19922239	0.181	0.135	3.3673E-78	AMs
OR6N1	4.4917E-82	1.11880332	0.045	0.021	6.9041E-78	AMs
INPP5J	5.4352E-82	1.02125793	0.051	0.026	8.3545E-78	AMs
MFSD13A	1.3683E-81	0.10708087	0.422	0.353	2.1033E-77	AMs
FAIM2	2.0165E-81	1.06166902	0.041	0.018	3.0996E-77	AMs
CARD9	3.4818E-81	0.13048524	0.374	0.31	5.3519E-77	AMs
REEP6	4.9636E-81	0.28392541	0.112	0.075	7.6295E-77	AMs
CARD6	4.986E-81	0.14926343	0.272	0.217	7.664E-77	AMs
CASP5	6.7121E-81	0.28346736	0.225	0.176	1.0317E-76	AMs

CBX8	1.4246E-80	0.29489532	0.165	0.121	2.1897E-76	AMs
PEX7	4.8773E-80	0.22697794	0.168	0.124	7.4969E-76	AMs
ZFP92	6.3523E-80	0.25632396	0.217	0.169	9.7642E-76	AMs
ACAT2	6.7919E-80	0.11755338	0.371	0.317	1.044E-75	AMs
HLX	1.3885E-79	0.19520026	0.169	0.125	2.1342E-75	AMs
ZNF483	1.4589E-79	0.24839486	0.05	0.025	2.2425E-75	AMs
PEBP4	1.5856E-79	1.04495518	0.048	0.023	2.4372E-75	AMs
ESR1	1.7119E-78	0.44502857	0.097	0.063	2.6314E-74	AMs
LILRA6	2.0663E-78	0.17181319	0.241	0.188	3.1761E-74	AMs
GDF15	4.6112E-78	0.76061082	0.043	0.02	7.0879E-74	AMs
TBC1D16	9.2084E-78	0.16406393	0.233	0.183	1.4154E-73	AMs
TBC1D12	1.8372E-77	0.25868351	0.255	0.204	2.8239E-73	AMs
SNX7	2.9377E-77	0.36828192	0.142	0.103	4.5156E-73	AMs
PTGR2	3.5049E-77	0.25317167	0.163	0.12	5.3873E-73	AMs
SLC19A2	3.652E-77	0.11090274	0.255	0.204	5.6134E-73	AMs
FAHD1	9.1082E-77	0.10629776	0.241	0.19	1.4E-72	AMs
OR6N2	9.746E-77	1.2841406	0.038	0.017	1.4981E-72	AMs
OCEL1	1.4999E-76	0.1089244	0.255	0.204	2.3054E-72	AMs
EEF1AKMT3	4.7068E-76	0.22443511	0.137	0.098	7.2349E-72	AMs
STPG1	5.0197E-76	0.12299641	0.289	0.235	7.7158E-72	AMs
SERPINI1	8.1621E-76	0.49528645	0.078	0.048	1.2546E-71	AMs
ZNF571	3.8338E-75	0.23405073	0.1	0.066	5.8929E-71	AMs
FKBP9	5.5143E-75	0.25619958	0.242	0.194	8.476E-71	AMs
COL6A2	1.1408E-74	0.7538518	0.031	0.012	1.7536E-70	AMs
CHMP4C	5.2271E-74	0.54868499	0.087	0.056	8.0345E-70	AMs
PCARE	7.4406E-74	0.60325541	0.095	0.062	1.1437E-69	AMs
CACFD1	8.5227E-74	0.23253421	0.1	0.067	1.31E-69	AMs
CLDN23	9.5351E-74	0.10605363	0.381	0.318	1.4656E-69	AMs
C1orf198	4.693E-73	0.13836229	0.196	0.151	7.2136E-69	AMs
PPP2CB	5.0751E-73	0.17083423	0.186	0.142	7.8009E-69	AMs
BOK	6.7569E-73	0.86575226	0.052	0.028	1.0386E-68	AMs
ZFYVE9	1.487E-72	0.148664	0.071	0.043	2.2857E-68	AMs
GNG11	4.7077E-72	0.70068619	0.079	0.05	7.2362E-68	AMs
ZNF610	4.9873E-72	0.61028798	0.062	0.036	7.6659E-68	AMs
NEK8	1.0829E-71	0.24876732	0.168	0.127	1.6645E-67	AMs
TSPYL5	1.0905E-71	0.24602869	0.133	0.095	1.6762E-67	AMs
IQCK	1.208E-71	0.21557741	0.117	0.081	1.8569E-67	AMs
GABRE	3.4685E-71	1.28866315	0.026	0.009	5.3314E-67	AMs
EGFL7	8.8423E-71	0.51935228	0.114	0.08	1.3591E-66	AMs
EDA	1.9886E-70	0.31329271	0.092	0.061	3.0567E-66	AMs
PLXNA2	4.1034E-70	0.33941798	0.19	0.148	6.3074E-66	AMs
TACR2	7.9873E-70	0.36468968	0.089	0.058	1.2277E-65	AMs
AMACR	1.1999E-69	0.12708488	0.249	0.201	1.8444E-65	AMs
FAM200A	2.4549E-69	0.17264384	0.146	0.108	3.7734E-65	AMs
PNPLA4	4.0318E-69	0.1356887	0.196	0.152	6.1973E-65	AMs
PAX8	8.5931E-69	0.30329435	0.145	0.107	1.3208E-64	AMs
CALD1	9.6053E-69	1.52717917	0.022	0.007	1.4764E-64	AMs
FXYD6	1.0024E-68	0.11310138	0.407	0.346	1.5407E-64	AMs
HHAT	1.6249E-67	0.18810074	0.133	0.096	2.4976E-63	AMs
TMEM144	1.8603E-67	0.15378447	0.319	0.265	2.8595E-63	AMs
LEPR	1.2475E-66	0.15741287	0.11	0.076	1.9175E-62	AMs
FAM83H	2.8287E-66	0.15583203	0.174	0.133	4.3479E-62	AMs

RAB38	4.5646E-66	0.15035999	0.238	0.192	7.0162E-62	AMs
NLRP2	1.0899E-65	0.10062685	0.231	0.184	1.6752E-61	AMs
PLA2G4A	5.834E-65	0.12240332	0.346	0.292	8.9675E-61	AMs
KRT79	2.4824E-64	0.87615625	0.058	0.034	3.8157E-60	AMs
ICAM4	3.2401E-64	0.24819241	0.101	0.069	4.9803E-60	AMs
GABRA4	1.1465E-63	0.95382558	0.043	0.023	1.7623E-59	AMs
CRPPA	1.1512E-63	0.47666409	0.06	0.035	1.7696E-59	AMs
PRRG1	2.8109E-63	0.42045744	0.116	0.083	4.3207E-59	AMs
XK	3.0872E-63	0.68527725	0.057	0.033	4.7454E-59	AMs
CYP3A5	7.0365E-63	0.97953526	0.041	0.021	1.0816E-58	AMs
LENG9	8.179E-63	0.11297244	0.136	0.1	1.2572E-58	AMs
CD300C	4.2529E-62	0.20788911	0.223	0.179	6.5371E-58	AMs
ANKMY2	4.8368E-62	0.11235281	0.183	0.143	7.4346E-58	AMs
RPH3AL	1.5982E-61	0.28755598	0.116	0.083	2.4566E-57	AMs
PLA2R1	1.8404E-61	0.83590074	0.042	0.022	2.8289E-57	AMs
TMEM158	1.262E-60	0.42616372	0.111	0.08	1.9398E-56	AMs
NUDT18	2.5265E-60	0.31548566	0.109	0.078	3.8834E-56	AMs
FZD7	2.6593E-60	0.37895359	0.091	0.061	4.0876E-56	AMs
CD72	3.7242E-60	0.30018286	0.139	0.104	5.7244E-56	AMs
SYNGR1	4.2113E-60	0.20022813	0.22	0.178	6.4731E-56	AMs
TCF7L1	9.7009E-60	1.15711933	0.025	0.01	1.4911E-55	AMs
IL7	1.2659E-59	0.13834085	0.16	0.122	1.9458E-55	AMs
FABP3	3.1202E-59	0.9379508	0.129	0.096	4.796E-55	AMs
C11orf74	1.3732E-58	0.20701168	0.187	0.148	2.1107E-54	AMs
ADAMTS15	1.5338E-58	0.55573875	0.096	0.067	2.3576E-54	AMs
FAM149B1	1.5589E-57	0.20271174	0.104	0.073	2.3961E-53	AMs
SLC22A15	1.5795E-57	0.12077899	0.333	0.28	2.4279E-53	AMs
ENC1	1.6994E-57	0.1172641	0.223	0.18	2.6121E-53	AMs
VLDLR	4.1686E-57	0.10443939	0.046	0.026	6.4075E-53	AMs
BATF2	8.5934E-57	0.35523876	0.067	0.043	1.3209E-52	AMs
TCHH	2.4836E-56	0.66530489	0.049	0.028	3.8175E-52	AMs
CHTF8	3.9926E-56	0.10713306	0.162	0.126	6.1371E-52	AMs
C2orf91	8.3895E-56	0.80746559	0.05	0.029	1.2895E-51	AMs
TMEM236	1.4E-54	0.13537813	0.201	0.162	2.1519E-50	AMs
CABP4	2.4116E-54	0.18124438	0.111	0.081	3.7069E-50	AMs
SAMD13	4.0997E-54	0.46593614	0.106	0.076	6.3016E-50	AMs
SEC16B	4.3166E-54	0.43250771	0.102	0.074	6.635E-50	AMs
NBPF1	7.3413E-54	0.41570689	0.08	0.055	1.1284E-49	AMs
CASTOR2	1.1973E-53	0.15463307	0.112	0.082	1.8403E-49	AMs
ANK2	2.2021E-53	0.94316932	0.032	0.016	3.3848E-49	AMs
ADAMTS1	5.4209E-53	0.99863766	0.034	0.017	8.3325E-49	AMs
STON2	6.0167E-53	0.34774126	0.121	0.09	9.2482E-49	AMs
MAP4K3	1.075E-52	0.1250066	0.171	0.134	1.6524E-48	AMs
PTPDC1	1.5436E-52	0.15138672	0.262	0.22	2.3727E-48	AMs
PTPN3	2.8732E-52	0.42303978	0.031	0.015	4.4163E-48	AMs
KCNIP2	6.1695E-52	0.18390194	0.139	0.107	9.4832E-48	AMs
SLC24A4	6.2168E-52	0.17429628	0.182	0.144	9.5558E-48	AMs
PLA1A	6.4807E-52	0.70738753	0.044	0.025	9.9614E-48	AMs
UBTD1	1.7276E-51	0.13579239	0.202	0.163	2.6554E-47	AMs
FHAD1	2.0465E-51	0.22716517	0.166	0.13	3.1457E-47	AMs
C3orf33	7.7065E-51	0.13951734	0.07	0.046	1.1846E-46	AMs
GPC4	9.8788E-51	0.301046	0.077	0.053	1.5185E-46	AMs

DEF8	1.1177E-50	0.25454864	0.071	0.047	1.718E-46	AMs
MOCOS	1.197E-50	0.24187376	0.116	0.086	1.8399E-46	AMs
NHLRC1	1.8732E-50	0.16406589	0.177	0.141	2.8792E-46	AMs
SDSL	3.4442E-50	0.12038635	0.257	0.215	5.2941E-46	AMs
NBEA	5.2044E-50	0.26477055	0.044	0.025	7.9996E-46	AMs
TNFAIP6	6.6019E-50	1.1778443	0.035	0.019	1.0148E-45	AMs
CARMIL1	1.1817E-49	0.13592343	0.126	0.095	1.8164E-45	AMs
HOMER2	2.0448E-49	0.1032889	0.127	0.095	3.1431E-45	AMs
HRH1	2.7741E-49	0.43463785	0.084	0.058	4.2641E-45	AMs
TNNI1	6.1106E-49	0.57577436	0.047	0.028	9.3926E-45	AMs
RTL8B	8.1807E-49	0.4073777	0.078	0.054	1.2574E-44	AMs
RAB3A	2.0064E-48	0.29931703	0.074	0.05	3.084E-44	AMs
SMAD1	4.2932E-48	0.14305159	0.228	0.189	6.599E-44	AMs
GPRC5B	7.8144E-48	0.98845624	0.023	0.01	1.2012E-43	AMs
PIR	9.9317E-48	0.29540095	0.144	0.113	1.5266E-43	AMs
PCDH18	1.1531E-47	2.21886122	0.013	0.004	1.7724E-43	AMs
MYO15A	1.2187E-47	0.64558389	0.042	0.024	1.8732E-43	AMs
NOCT	1.6411E-47	0.20097851	0.174	0.14	2.5225E-43	AMs
AKR1C2	3.6103E-47	0.16812467	0.179	0.144	5.5494E-43	AMs
ZNF835	5.3437E-47	0.37475994	0.082	0.058	8.2138E-43	AMs
ZNF226	5.5446E-47	0.2136345	0.101	0.074	8.5227E-43	AMs
SLC22A18AS	9.3844E-47	0.51281327	0.045	0.027	1.4425E-42	AMs
ARHGEF35	9.7373E-47	0.50473578	0.07	0.048	1.4967E-42	AMs
PLEKHA6	1.69E-46	0.35996011	0.055	0.035	2.5977E-42	AMs
PRCD	2.8622E-46	1.29265503	0.02	0.008	4.3995E-42	AMs
ZNF597	3.7252E-46	0.10003465	0.079	0.055	5.726E-42	AMs
GLTPD2	1.1724E-45	0.91714259	0.029	0.014	1.8021E-41	AMs
NNMT	1.7657E-45	0.60377765	0.064	0.042	2.7141E-41	AMs
TM4SF19	4.8883E-45	0.65438373	0.069	0.048	7.5139E-41	AMs
M1AP	8.0152E-45	0.22138546	0.153	0.122	1.232E-40	AMs
EXTL2	1.0011E-44	0.17976629	0.205	0.169	1.5388E-40	AMs
CELA2B	1.0959E-44	0.23976844	0.183	0.149	1.6845E-40	AMs
ZFP57	1.158E-44	0.1658033	0.094	0.068	1.78E-40	AMs
FMO4	1.8394E-44	0.2626138	0.1	0.074	2.8273E-40	AMs
SNAI2	3.2549E-44	0.3511832	0.083	0.06	5.0031E-40	AMs
COL14A1	3.2661E-44	1.10700978	0.024	0.011	5.0203E-40	AMs
ZNF667	4.4068E-44	0.42881145	0.022	0.01	6.7737E-40	AMs
SPATC1	8.0311E-44	0.47729723	0.058	0.038	1.2345E-39	AMs
C17orf97	8.1517E-44	0.13194348	0.08	0.056	1.253E-39	AMs
ZNF844	1.0542E-43	0.17109282	0.079	0.056	1.6205E-39	AMs
WFIKKN2	1.2644E-43	1.68033711	0.016	0.006	1.9436E-39	AMs
ADAMTS7	3.6118E-43	0.9463549	0.021	0.009	5.5517E-39	AMs
NUDT12	3.6826E-43	0.25021371	0.134	0.105	5.6606E-39	AMs
C20orf202	5.3537E-43	0.52803869	0.056	0.037	8.2292E-39	AMs
AICDA	5.9096E-43	0.54412712	0.051	0.032	9.0837E-39	AMs
C16orf86	1.0216E-42	0.21864404	0.129	0.1	1.5703E-38	AMs
SIGLEC12	1.425E-42	0.30324187	0.169	0.138	2.1904E-38	AMs
HACD1	1.4837E-41	0.49920121	0.067	0.046	2.2806E-37	AMs
SETD9	2.0822E-41	0.11422638	0.159	0.128	3.2006E-37	AMs
ADGRD1	4.0513E-41	0.45551568	0.055	0.036	6.2272E-37	AMs
SOBP	5.085E-41	0.75197256	0.028	0.015	7.8161E-37	AMs
OSCP1	5.7086E-41	0.1592877	0.079	0.057	8.7746E-37	AMs

HOXA1	7.911E-41	0.35094363	0.034	0.02	1.216E-36	AMs
MCRIP2	1.075E-40	0.12159798	0.121	0.093	1.6523E-36	AMs
RARRES1	1.2171E-40	0.36667384	0.057	0.038	1.8708E-36	AMs
ATP8B1	1.6562E-40	0.39975978	0.047	0.03	2.5457E-36	AMs
FDXACB1	2.182E-40	0.14429177	0.109	0.083	3.3539E-36	AMs
USP2	2.5173E-40	0.44773679	0.041	0.025	3.8693E-36	AMs
PIPOX	2.7999E-40	0.18380321	0.163	0.132	4.3037E-36	AMs
ARMCX1	3.7322E-40	0.12169363	0.195	0.162	5.7368E-36	AMs
PDZD7	3.866E-40	0.44381848	0.074	0.052	5.9424E-36	AMs
TMTC1	5.7968E-40	0.29362649	0.094	0.069	8.9102E-36	AMs
PPP1R26	1.4522E-39	0.12824061	0.19	0.158	2.2321E-35	AMs
KREMEN1	4.9817E-39	0.22271893	0.069	0.048	7.6573E-35	AMs
TUSC1	9.2737E-39	0.33303223	0.087	0.065	1.4255E-34	AMs
MFAP5	2.3638E-38	0.60470111	0.043	0.027	3.6334E-34	AMs
TDO2	5.0267E-38	0.7571386	0.024	0.012	7.7265E-34	AMs
DRD5	6.1907E-38	1.14045276	0.022	0.01	9.5157E-34	AMs
ACSM3	7.5673E-38	0.23472727	0.111	0.086	1.1632E-33	AMs
SLC45A1	9.0854E-38	0.89250088	0.028	0.015	1.3965E-33	AMs
TSPAN9	1.17E-37	0.77386378	0.026	0.014	1.7983E-33	AMs
PFKFB4	1.8483E-37	0.11540816	0.19	0.158	2.8411E-33	AMs
LRRC75A	7.5771E-37	0.55078745	0.058	0.04	1.1647E-32	AMs
TMEM169	1.3205E-36	0.36761021	0.036	0.022	2.0297E-32	AMs
ELF5	1.7662E-36	0.88726264	0.024	0.012	2.7148E-32	AMs
TMEM255B	2.6953E-36	0.14641965	0.167	0.138	4.143E-32	AMs
TMCC3	3.1513E-36	0.11425078	0.124	0.098	4.8438E-32	AMs
DNAH14	4.0901E-36	0.40206373	0.045	0.029	6.2868E-32	AMs
LMLN	5.578E-36	0.1365224	0.082	0.061	8.574E-32	AMs
PEX11G	6.4963E-36	0.29651026	0.069	0.05	9.9854E-32	AMs
SUSD5	7.457E-36	1.56684441	0.013	0.005	1.1462E-31	AMs
FGGY	9.5673E-36	0.18794635	0.07	0.05	1.4706E-31	AMs
ARHGAP32	1.9589E-35	0.21888546	0.089	0.067	3.011E-31	AMs
GLB1L	3.0373E-35	0.21961646	0.126	0.1	4.6687E-31	AMs
LRRC27	3.5516E-35	0.10646782	0.096	0.073	5.4591E-31	AMs
MTUS1	3.6494E-35	0.20161624	0.1	0.077	5.6094E-31	AMs
BTNL8	4.322E-35	0.5324338	0.022	0.011	6.6434E-31	AMs
PCDHGA12	4.5373E-35	0.62581257	0.034	0.02	6.9743E-31	AMs
TLE6	5.2368E-35	0.60411727	0.034	0.02	8.0495E-31	AMs
S100A2	6.3441E-35	0.35861616	0.038	0.023	9.7516E-31	AMs
HSD11B1	8.0435E-35	0.82416671	0.032	0.019	1.2364E-30	AMs
BAMBI	8.7638E-35	0.65575404	0.04	0.025	1.3471E-30	AMs
PEG3	1.8834E-34	0.44002301	0.044	0.028	2.895E-30	AMs
BIRC7	8.4564E-34	1.03266656	0.021	0.01	1.2998E-29	AMs
ZNF391	1.1213E-33	0.50802372	0.024	0.012	1.7236E-29	AMs
OPRL1	1.2495E-33	0.24460887	0.078	0.058	1.9206E-29	AMs
PROX1	6.7227E-33	1.07110358	0.017	0.008	1.0334E-28	AMs
PC	9.4663E-33	0.24057241	0.111	0.088	1.4551E-28	AMs
KLHDC9	1.5189E-32	0.37787904	0.032	0.019	2.3347E-28	AMs
PTX3	3.7731E-32	0.78327245	0.036	0.023	5.7997E-28	AMs
ETFBKMT	4.0994E-32	0.22417597	0.075	0.056	6.3011E-28	AMs
C11orf45	4.1663E-32	0.12490058	0.183	0.154	6.4039E-28	AMs
ARHGAP22	4.4261E-32	0.12429487	0.136	0.11	6.8033E-28	AMs
CD22	4.9683E-32	0.10500009	0.135	0.11	7.6367E-28	AMs

IRF6	1.8028E-31	0.12856785	0.052	0.036	2.7711E-27	AMs
CYSLTR2	2.3036E-31	0.12500452	0.131	0.106	3.5408E-27	AMs
EXOC3L4	2.3426E-31	0.76891443	0.023	0.013	3.6009E-27	AMs
KL	4.9082E-31	0.13841783	0.12	0.096	7.5445E-27	AMs
NKX3-1	6.7134E-31	0.38522652	0.065	0.048	1.0319E-26	AMs
ZNF285	7.0487E-31	0.34741755	0.05	0.035	1.0835E-26	AMs
TMEM255A	7.5144E-31	0.53975653	0.041	0.027	1.155E-26	AMs
ST6GALNAC2	8.8563E-31	0.42011958	0.049	0.034	1.3613E-26	AMs
LIPC	2.0402E-30	0.34394021	0.037	0.024	3.136E-26	AMs
LRP2	5.744E-30	1.3091585	0.014	0.006	8.8291E-26	AMs
CXCL11	6.7787E-30	1.54575492	0.018	0.009	1.042E-25	AMs
B3GALNT1	8.9711E-30	0.14672751	0.176	0.149	1.3789E-25	AMs
ZNF629	9.4738E-30	0.22163142	0.073	0.055	1.4562E-25	AMs
CLUL1	1.1192E-29	0.7038245	0.023	0.013	1.7204E-25	AMs
CLBA1	1.2536E-29	0.21680797	0.045	0.031	1.9269E-25	AMs
ZSCAN31	1.4902E-29	0.15561092	0.086	0.067	2.2906E-25	AMs
STARD5	1.5748E-29	0.13484902	0.106	0.085	2.4206E-25	AMs
DNAJC6	1.6909E-29	0.15681911	0.18	0.153	2.599E-25	AMs
ZNF883	2.2369E-29	0.4764061	0.03	0.019	3.4384E-25	AMs
EHHADH	5.5284E-29	0.10359011	0.114	0.092	8.4977E-25	AMs
ME3	7.0743E-29	0.15789401	0.237	0.207	1.0874E-24	AMs
MOCS1	7.2505E-29	1.02211552	0.016	0.008	1.1145E-24	AMs
ACSM1	8.2855E-29	0.51218453	0.033	0.021	1.2736E-24	AMs
ZNF471	8.8504E-29	0.4291871	0.042	0.029	1.3604E-24	AMs
OTOF	9.0851E-29	0.68505949	0.017	0.009	1.3965E-24	AMs
AQP7	2.3194E-28	0.67406343	0.022	0.012	3.5652E-24	AMs
TSKS	3.7183E-28	0.45726848	0.041	0.028	5.7154E-24	AMs
FLT1	7.162E-28	0.38701038	0.051	0.036	1.1009E-23	AMs
SYT4	7.483E-28	1.3104821	0.01	0.004	1.1502E-23	AMs
PCDHGA10	7.8044E-28	0.80035668	0.02	0.011	1.1996E-23	AMs
MYOZ1	8.1503E-28	0.51710826	0.037	0.024	1.2528E-23	AMs
PPT2	8.1694E-28	0.11379109	0.074	0.056	1.2557E-23	AMs
DHDH	1.5274E-27	0.23077195	0.113	0.092	2.3478E-23	AMs
ZNF205	2.2018E-27	0.22746662	0.107	0.086	3.3844E-23	AMs
GPRASP2	3.1806E-27	0.3390716	0.025	0.014	4.8889E-23	AMs
CRB1	3.2611E-27	1.00324648	0.016	0.008	5.0126E-23	AMs
MFAP3L	5.2109E-27	0.53836878	0.02	0.011	8.0096E-23	AMs
AC233723.1	5.6083E-27	0.41490692	0.032	0.02	8.6206E-23	AMs
ZNF20	1.2862E-26	0.18540991	0.046	0.032	1.977E-22	AMs
PDE8B	1.9536E-26	0.19890529	0.043	0.029	3.0029E-22	AMs
ANGPTL1	6.9769E-26	0.77583099	0.025	0.015	1.0724E-21	AMs
CXCL9	7.0449E-26	1.10998542	0.031	0.02	1.0829E-21	AMs
ZFHX2	7.4364E-26	0.21210456	0.087	0.068	1.143E-21	AMs
IL27	9.5736E-26	0.40006535	0.045	0.031	1.4716E-21	AMs
SERPINA9	1.7335E-25	0.59733326	0.03	0.019	2.6645E-21	AMs
GTF2IRD1	1.8809E-25	0.1251141	0.101	0.082	2.8911E-21	AMs
PAPLN	1.1794E-24	0.1920527	0.063	0.048	1.8128E-20	AMs
SLC46A2	1.4114E-24	0.5002457	0.052	0.038	2.1695E-20	AMs
NDN	1.4757E-24	0.41645126	0.031	0.02	2.2683E-20	AMs
VWF	1.5559E-24	0.43793276	0.038	0.026	2.3915E-20	AMs
ADCY1	1.9566E-24	0.82164768	0.023	0.014	3.0075E-20	AMs
FAM78B	2.3804E-24	1.04767715	0.01	0.004	3.6589E-20	AMs

TLE2	2.8876E-24	0.40448758	0.027	0.017	4.4386E-20	AMs
F12	3.5912E-24	0.49128107	0.022	0.013	5.52E-20	AMs
ZNF697	7.5049E-24	0.19968886	0.074	0.058	1.1536E-19	AMs
CRH	1.2427E-23	1.04719097	0.014	0.007	1.9101E-19	AMs
WSCD2	2.0937E-23	0.880081	0.011	0.005	3.2182E-19	AMs
ARHGEF17	2.7123E-23	0.32987602	0.028	0.018	4.1691E-19	AMs
ZCWPW2	8.8105E-23	0.33875585	0.044	0.031	1.3543E-18	AMs
CCDC153	9.8734E-23	0.14154104	0.076	0.06	1.5176E-18	AMs
BEX3	1.16E-22	0.18041747	0.061	0.046	1.783E-18	AMs
PM20D1	1.4086E-22	0.50035018	0.026	0.016	2.1652E-18	AMs
MFSD4A	1.7322E-22	0.48374096	0.021	0.012	2.6625E-18	AMs
PLPP7	3.5602E-22	0.44456198	0.025	0.016	5.4724E-18	AMs
CAPN11	3.7985E-22	1.08371428	0.012	0.006	5.8387E-18	AMs
ENPP6	4.5375E-22	0.5704229	0.03	0.02	6.9746E-18	AMs
ZNF423	6.7787E-22	1.02299788	0.014	0.007	1.042E-17	AMs
TXNRD3	8.1704E-22	0.21962014	0.059	0.045	1.2559E-17	AMs
ACOT1	1.3103E-21	0.63391881	0.022	0.013	2.014E-17	AMs
FRK	1.6405E-21	0.20208433	0.019	0.011	2.5217E-17	AMs
KCNJ5	2.5605E-21	0.44409223	0.026	0.017	3.9357E-17	AMs
SLC4A11	5.0007E-21	0.18079718	0.066	0.051	7.6865E-17	AMs
KLF14	8.2251E-21	0.97871241	0.012	0.006	1.2643E-16	AMs
PNMA8B	1.1486E-20	0.72188435	0.023	0.014	1.7655E-16	AMs
RIPK4	1.742E-20	0.42535356	0.016	0.009	2.6777E-16	AMs
OR52K2	1.7923E-20	0.25337745	0.079	0.063	2.7549E-16	AMs
SH3BGR	2.3758E-20	0.44380124	0.034	0.024	3.6518E-16	AMs
SLIT3	6.2004E-20	0.6667501	0.039	0.028	9.5306E-16	AMs
TMEM217	1.1149E-19	0.43612504	0.02	0.012	1.7138E-15	AMs
SCN8A	1.235E-19	0.31046857	0.029	0.02	1.8983E-15	AMs
HOXB5	1.3284E-19	1.08306907	0.01	0.005	2.0418E-15	AMs
ROBO1	1.7163E-19	0.11867357	0.032	0.022	2.6381E-15	AMs
HRH4	2.0883E-19	0.1854155	0.056	0.043	3.2099E-15	AMs
IQCA1	3.9387E-19	0.36279283	0.014	0.007	6.0542E-15	AMs
SYP	7.1253E-19	0.13742603	0.077	0.062	1.0952E-14	AMs
PRSS33	1.5933E-18	0.67454693	0.014	0.008	2.4491E-14	AMs
IFNA1	1.7489E-18	0.98993609	0.017	0.01	2.6883E-14	AMs
DENND2C	2.2986E-18	0.10606914	0.025	0.017	3.5332E-14	AMs
PIGZ	2.9673E-18	0.14510505	0.082	0.067	4.5611E-14	AMs
LGR4	3.5985E-18	0.20256983	0.045	0.034	5.5312E-14	AMs
DOCK6	4.2369E-18	0.35656255	0.024	0.016	6.5126E-14	AMs
BEGAIN	7.9254E-18	0.30144011	0.016	0.01	1.2182E-13	AMs
OVOL1	1.0399E-17	0.79829731	0.01	0.005	1.5984E-13	AMs
TUBB8	1.0522E-17	0.6152343	0.011	0.006	1.6174E-13	AMs
AKR1C8P	1.1266E-17	0.12313085	0.024	0.016	1.7317E-13	AMs
CEACAM8	3.1291E-17	0.46502154	0.033	0.023	4.8097E-13	AMs
A4GNT	3.7553E-17	0.48192142	0.044	0.033	5.7722E-13	AMs
VNN1	4.4691E-17	0.14159413	0.034	0.025	6.8695E-13	AMs
TIGD6	4.6466E-17	0.18174989	0.062	0.05	7.1422E-13	AMs
ZNF396	4.8411E-17	0.14622874	0.044	0.033	7.4412E-13	AMs
ENPP7	4.876E-17	0.86777238	0.015	0.009	7.4949E-13	AMs
ZNF404	5.1068E-17	0.22492677	0.032	0.023	7.8497E-13	AMs
CCL20	7.9033E-17	0.28867191	0.044	0.033	1.2148E-12	AMs
TBC1D8B	8.904E-17	0.25258874	0.055	0.043	1.3686E-12	AMs

STAG3	1.4363E-16	0.1313267	0.07	0.057	2.2077E-12	AMs
L1TD1	1.992E-16	0.14568017	0.105	0.089	3.0619E-12	AMs
PCDHGB7	2.2293E-16	0.57464891	0.01	0.005	3.4266E-12	AMs
ZNF442	2.3921E-16	0.28368108	0.031	0.022	3.6769E-12	AMs
HEYL	2.8488E-16	0.77605773	0.016	0.01	4.3788E-12	AMs
PTK6	2.8849E-16	0.13965216	0.066	0.053	4.4344E-12	AMs
RAB3C	2.9325E-16	0.44167306	0.015	0.009	4.5075E-12	AMs
ACSS3	3.4565E-16	0.39551667	0.036	0.027	5.3131E-12	AMs
GRIN2D	3.9598E-16	0.19491487	0.07	0.057	6.0867E-12	AMs
LURAP1	4.6734E-16	0.26155608	0.062	0.05	7.1835E-12	AMs
ASAP3	5.4776E-16	0.23611544	0.014	0.008	8.4196E-12	AMs
ZNF860	1.2567E-15	0.28374972	0.039	0.029	1.9317E-11	AMs
PIP5KL1	1.4172E-15	0.20703309	0.024	0.016	2.1784E-11	AMs
OR13A1	1.4176E-15	0.63319734	0.021	0.014	2.179E-11	AMs
LRRC29	3.7177E-15	0.10597038	0.068	0.055	5.7145E-11	AMs
CHST3	6.0375E-15	0.23072042	0.068	0.056	9.2802E-11	AMs
TREML4	6.3864E-15	0.38332318	0.012	0.007	9.8165E-11	AMs
PNMA8A	7.844E-15	1.00196309	0.01	0.006	1.2057E-10	AMs
GREB1	8.3016E-15	0.54993244	0.012	0.007	1.276E-10	AMs
ZNF665	1.1317E-14	0.11015917	0.025	0.018	1.7395E-10	AMs
KLHL35	1.2895E-14	0.15222224	0.033	0.024	1.9821E-10	AMs
UBD	1.5008E-14	0.67830193	0.011	0.006	2.3068E-10	AMs
RANBP3L	2.2451E-14	0.6557324	0.01	0.006	3.4509E-10	AMs
CCT6B	2.7427E-14	0.14378892	0.041	0.031	4.2157E-10	AMs
GSTA4	3.698E-14	0.28925568	0.026	0.018	5.6842E-10	AMs
EDA2R	3.7724E-14	0.65423882	0.01	0.005	5.7986E-10	AMs
SSMEM1	3.8158E-14	0.66908387	0.017	0.011	5.8652E-10	AMs
CCDC142	5.5613E-14	0.15649886	0.02	0.013	8.5482E-10	AMs
SULT1C2	9.8684E-14	0.88230782	0.011	0.006	1.5169E-09	AMs
CMBL	1.305E-13	0.18801592	0.028	0.021	2.0059E-09	AMs
ZMYND12	1.8201E-13	0.14058877	0.025	0.018	2.7977E-09	AMs
PNPLA1	2.5983E-13	0.27254738	0.042	0.033	3.9938E-09	AMs
PLEKHG6	5.0561E-13	0.28012317	0.018	0.012	7.7717E-09	AMs
ZNF334	6.5796E-13	0.21225859	0.038	0.029	1.0114E-08	AMs
ACSBG2	7.8271E-13	0.81471429	0.01	0.006	1.2031E-08	AMs
SENP8	1.1008E-12	0.10143382	0.044	0.035	1.6921E-08	AMs
CXCL10	1.1278E-12	1.23657885	0.05	0.041	1.7335E-08	AMs
CCDC102B	1.3041E-12	0.11614056	0.033	0.025	2.0045E-08	AMs
OR1B1	1.398E-12	0.44788401	0.012	0.007	2.1489E-08	AMs
CDH1	2.3491E-12	0.36740408	0.038	0.03	3.6108E-08	AMs
ZBTB12	5.3114E-12	0.2736537	0.032	0.025	8.1641E-08	AMs
SSC4D	7.5024E-12	0.20831594	0.063	0.052	1.1532E-07	AMs
GSG1L	1.2421E-11	0.9151606	0.01	0.006	1.9092E-07	AMs
CTH	1.6577E-11	0.23886686	0.025	0.018	2.548E-07	AMs
CEACAM19	2.3141E-11	0.18954851	0.047	0.038	3.5569E-07	AMs
ZNF221	2.4783E-11	0.12132384	0.05	0.041	3.8095E-07	AMs
MT1F	3.5705E-11	0.13104722	0.104	0.091	5.4882E-07	AMs
EQTN	3.7625E-11	0.38879052	0.018	0.012	5.7834E-07	AMs
ZNF658	4.7677E-11	0.10530519	0.036	0.028	7.3285E-07	AMs
TSGA10IP	7.8373E-11	0.44184309	0.016	0.011	1.2047E-06	AMs
IL37	9.0799E-11	0.44303957	0.016	0.011	1.3957E-06	AMs
LNP1	1.0719E-10	0.28663106	0.015	0.01	1.6477E-06	AMs

COPZ2	1.2484E-10	0.15026887	0.058	0.049	1.919E-06	AMs
HDX	1.3989E-10	0.11652944	0.046	0.038	2.1502E-06	AMs
NAALADL2	1.8443E-10	0.2314052	0.014	0.01	2.8348E-06	AMs
MMP15	2.322E-10	0.17155165	0.068	0.058	3.5691E-06	AMs
STX1B	2.3451E-10	0.22936391	0.021	0.016	3.6047E-06	AMs
ELOVL3	2.8342E-10	0.27006616	0.046	0.038	4.3565E-06	AMs
PPM1N	6.7511E-10	0.16496259	0.026	0.02	1.0377E-05	AMs
CECR2	7.8502E-10	0.25590616	0.033	0.026	1.2067E-05	AMs
KIF7	8.8068E-10	0.37620117	0.02	0.014	1.3537E-05	AMs
PARD3B	9.3271E-10	0.27306047	0.022	0.016	1.4337E-05	AMs
CTF1	1.0908E-09	0.18971659	0.036	0.029	1.6767E-05	AMs
KLHL10	1.3222E-09	0.16210178	0.023	0.017	2.0324E-05	AMs
FZD4	3.2551E-09	0.36338117	0.021	0.016	5.0034E-05	AMs
CS	4.2605E-09	0.1126083	0.014	0.01	6.5488E-05	AMs
APOC4	5.1541E-09	0.36918103	0.029	0.023	7.9224E-05	AMs
SMIM10	9.5457E-09	0.1314358	0.039	0.032	0.00014673	AMs
SPATA12	1.1574E-08	0.25713155	0.055	0.047	0.0001779	AMs
CDR2L	1.1744E-08	0.14240654	0.062	0.054	0.00018052	AMs
ADAMTSL2	1.4683E-08	0.31395402	0.015	0.011	0.0002257	AMs
LMNTD2	1.4965E-08	0.13251733	0.048	0.04	0.00023002	AMs
HOXA5	1.6925E-08	0.3484131	0.01	0.007	0.00026016	AMs
TSLP	1.8221E-08	0.18200708	0.024	0.019	0.00028007	AMs
ZDHHC19	2.3908E-08	0.15612727	0.033	0.027	0.00036749	AMs
COL1A1	2.8841E-08	0.5644057	0.027	0.021	0.00044332	AMs
TCP10L	3.409E-08	0.10509798	0.016	0.011	0.000524	AMs
VASN	4.3865E-08	0.22033791	0.012	0.008	0.00067424	AMs
SCN11A	4.857E-08	0.37622026	0.018	0.014	0.00074657	AMs
ALOX15B	5.3502E-08	0.28872867	0.01	0.007	0.00082238	AMs
FZD8	5.6154E-08	0.28891401	0.039	0.032	0.00086315	AMs
CLDN11	5.9732E-08	0.36831334	0.01	0.007	0.00091813	AMs
IL6	9.1096E-08	0.25090519	0.04	0.034	0.00140024	AMs
CDNF	1.0552E-07	0.22843649	0.01	0.007	0.00162198	AMs
PTH2R	1.2166E-07	0.31406149	0.024	0.019	0.00186998	AMs
PFKFB1	2.153E-07	0.16102474	0.01	0.007	0.00330938	AMs
TMCO2	2.4676E-07	0.1132921	0.024	0.019	0.00379297	AMs
C15orf65	2.5721E-07	0.15778788	0.021	0.016	0.00395364	AMs
CLEC4M	4.8907E-07	0.49961392	0.01	0.007	0.00751755	AMs
XKR3	7.3125E-07	0.5648337	0.01	0.007	0.01124	AMs
MYMX	7.9426E-07	0.31117131	0.013	0.01	0.01220856	AMs
RUNDC3A	8.3384E-07	0.27453446	0.019	0.015	0.01281692	AMs
GSTT2B	1.1429E-06	0.29838891	0.015	0.011	0.01756711	AMs
ZNF215	1.5036E-06	0.17939432	0.04	0.035	0.0231121	AMs
CCNB3	1.5193E-06	0.23724603	0.014	0.011	0.02335304	AMs
COL9A3	1.8479E-06	0.30867786	0.019	0.015	0.02840392	AMs
ZG16B	2.7982E-06	0.10011405	0.015	0.012	0.04301121	AMs
C17orf64	4.3598E-06	0.23342039	0.026	0.021	0.06701416	AMs
ZNF311	7.7943E-06	0.26862928	0.013	0.01	0.11980653	AMs
CHRNA3	7.8897E-06	0.23095699	0.035	0.03	0.12127253	AMs
RBFOX2	1.003E-05	0.11195805	0.037	0.032	0.15417075	AMs
CCDC62	4.7186E-05	0.1080097	0.012	0.009	0.72529999	AMs
OR52K1	6.3672E-05	0.18017574	0.029	0.025	0.9787063	AMs
ARL14EPL	9.0823E-05	0.2529817	0.021	0.018	1	AMs

INCA1	0.00010375	0.24203352	0.012	0.009	1	AMs
ULBP2	0.00024716	0.20924133	0.01	0.008	1	AMs
PCDHGC3	0.00035212	0.18154385	0.014	0.011	1	AMs
MT1G	0.00138554	0.37954998	0.012	0.01	1	AMs
PLAAT5	0.00241841	0.14839071	0.029	0.026	1	AMs
CAPNS2	0.00306787	0.44082374	0.01	0.008	1	AMs
FCGR2B	0	5.64968073	0.635	0.023	0	IMs
CLEC10A	0	6.76665679	0.615	0.008	0	IMs
GPR183	0	4.84763427	0.62	0.016	0	IMs
EMP1	0	4.38836706	0.723	0.128	0	IMs
RASSF2	0	4.36201682	0.619	0.024	0	IMs
MARCKS	0	4.00960088	0.656	0.082	0	IMs
PLXNC1	0	4.80555592	0.591	0.033	0	IMs
GPR132	0	3.4782762	0.573	0.057	0	IMs
CCR5	0	2.62101617	0.569	0.064	0	IMs
SERPINB9	0	3.81128975	0.563	0.07	0	IMs
SNCA	0	3.92379105	0.56	0.071	0	IMs
C15orf48	0	3.51920811	0.671	0.183	0	IMs
CORO1A	0	2.20231453	0.691	0.206	0	IMs
PRKCB	0	3.50608155	0.537	0.053	0	IMs
FMN1	0	4.68390415	0.521	0.043	0	IMs
LIMD2	0	3.16196041	0.49	0.025	0	IMs
FPR3	0	3.31438379	0.955	0.491	0	IMs
PLEKHO1	0	2.61168883	0.879	0.419	0	IMs
TMEM176B	0	2.81492819	0.837	0.379	0	IMs
PLA2G7	0	5.3998561	0.495	0.044	0	IMs
ST8SIA4	0	2.77820264	0.515	0.08	0	IMs
A2M	0	3.56035959	0.659	0.228	0	IMs
CMKLR1	0	5.30197925	0.445	0.025	0	IMs
ARL4C	0	2.61616051	0.574	0.155	0	IMs
ANTXR2	0	3.5207599	0.441	0.024	0	IMs
ADAM8	0	4.09053531	0.422	0.017	0	IMs
RNASE1	0	5.22278095	0.448	0.045	0	IMs
CYP2S1	0	3.27775194	0.497	0.101	0	IMs
ARHGEF40	0	3.2552474	0.478	0.086	0	IMs
P2RY6	0	5.30548483	0.403	0.015	0	IMs
FGL2	0	2.62773405	0.869	0.484	0	IMs
IL4I1	0	4.09296149	0.472	0.092	0	IMs
TRPM2	0	4.41572304	0.413	0.034	0	IMs
F13A1	0	8.94583952	0.379	0.002	0	IMs
CYBRD1	0	4.91446282	0.394	0.022	0	IMs
ST6GAL1	0	3.02560244	0.425	0.053	0	IMs
PALD1	0	4.45957278	0.404	0.039	0	IMs
STAB1	0	8.24633399	0.367	0.002	0	IMs
GAS6	0	5.38731778	0.36	0.013	0	IMs
GASK1B	0	4.81293194	0.372	0.027	0	IMs
IL21R	0	4.23132364	0.36	0.017	0	IMs
KCNN4	0	3.54787765	0.404	0.063	0	IMs
CD209	0	3.83467487	0.415	0.077	0	IMs
MEF2C	0	3.65392868	0.404	0.067	0	IMs
ETV5	0	5.48041173	0.342	0.016	0	IMs
AMPD3	0	4.44520634	0.34	0.015	0	IMs

GRASP	0	4.69528675	0.346	0.021	0	IMs
NLRP3	0	3.6062233	0.372	0.055	0	IMs
SLC7A5	0	3.44839684	0.368	0.052	0	IMs
RGL1	0	5.30713911	0.324	0.015	0	IMs
EMB	0	2.29108067	0.322	0.022	0	IMs
MERTK	0	7.76005165	0.297	0.002	0	IMs
CCL2	0	7.40488527	0.291	0.004	0	IMs
FAM20A	0	5.07784219	0.299	0.013	0	IMs
LILRB5	0	5.4150923	0.311	0.027	0	IMs
CCR2	0	3.7610415	0.29	0.014	0	IMs
ENPP2	0	3.91246502	0.318	0.046	0	IMs
SGK1	0	2.60489925	0.919	0.656	0	IMs
ATP8B4	0	3.60971893	0.302	0.039	0	IMs
SLAMF7	0	3.96716883	0.29	0.027	0	IMs
CCND2	0	2.35529431	0.282	0.021	0	IMs
SLC40A1	0	6.85812742	0.254	0.008	0	IMs
NDRG2	0	4.62036316	0.256	0.01	0	IMs
CCR6	0	4.00991783	0.255	0.01	0	IMs
DYSF	0	4.22238748	0.268	0.025	0	IMs
GAL3ST4	0	6.42823809	0.248	0.007	0	IMs
SDS	0	7.74124234	0.246	0.005	0	IMs
SULF2	0	5.36465335	0.24	0.004	0	IMs
ADAM28	0	5.10583541	0.24	0.008	0	IMs
OLFML2B	0	8.32507822	0.232	0.001	0	IMs
MMP2	0	7.34894815	0.228	0.003	0	IMs
GABBR1	0	3.81575346	0.242	0.017	0	IMs
CCL13	0	9.05262639	0.222	0.002	0	IMs
GPR84	0	4.51857846	0.237	0.02	0	IMs
MS4A4E	0	3.75206906	0.245	0.03	0	IMs
GPR141	0	4.07784648	0.231	0.022	0	IMs
MATK	0	2.86256294	0.221	0.013	0	IMs
SPHK1	0	5.05161281	0.221	0.014	0	IMs
SELENOP	0	8.54478216	0.212	0.007	0	IMs
MAMDC2	0	7.12048982	0.211	0.007	0	IMs
HS3ST1	0	5.5454616	0.212	0.012	0	IMs
RAB3IL1	0	6.63513093	0.204	0.004	0	IMs
LGALS2	0	4.79608344	0.213	0.013	0	IMs
TPCN1	0	4.44659653	0.206	0.008	0	IMs
EPHB2	0	5.55023423	0.201	0.007	0	IMs
VENTX	0	5.00074952	0.191	0.008	0	IMs
OAF	0	5.04270181	0.188	0.009	0	IMs
PLD4	0	5.84047312	0.181	0.002	0	IMs
MTSS1	0	4.09662069	0.181	0.005	0	IMs
ECM1	0	3.25669364	0.187	0.013	0	IMs
TMEM37	0	4.81957072	0.186	0.012	0	IMs
IRF4	0	3.23468647	0.179	0.011	0	IMs
PKIB	0	5.03420128	0.173	0.005	0	IMs
ANKRD22	0	4.69786299	0.162	0.01	0	IMs
CBFA2T3	0	3.9388489	0.166	0.014	0	IMs
S100B	0	4.44487808	0.155	0.004	0	IMs
ACPP	0	5.31033693	0.151	0.004	0	IMs
MYO1D	0	2.74679711	0.156	0.011	0	IMs

TMEM163	0	4.93579091	0.152	0.008	0	IMs
CD1C	0	5.12320369	0.145	0.002	0	IMs
CLDN1	0	5.90704168	0.14	0.003	0	IMs
IGFBP4	0	5.538292	0.135	0.002	0	IMs
HRH2	0	5.1103177	0.135	0.003	0	IMs
IGSF21	0	8.87092626	0.13	0	0	IMs
SLC9A9	0	3.77380476	0.137	0.008	0	IMs
DEPTOR	0	4.63034865	0.136	0.007	0	IMs
P3H2	0	6.31533545	0.129	0.001	0	IMs
MCOLN3	0	4.94385643	0.13	0.006	0	IMs
FCER1A	0	4.70751165	0.123	0.002	0	IMs
KCTD15	0	3.91944618	0.126	0.008	0	IMs
MRC2	0	5.13120073	0.122	0.005	0	IMs
CX3CR1	0	6.33826897	0.114	0.001	0	IMs
OLFML3	0	8.36684701	0.112	0.001	0	IMs
IL10	0	4.67111071	0.118	0.007	0	IMs
NT5DC2	0	5.02686401	0.109	0.004	0	IMs
CD200R1	0	2.42105777	0.106	0.006	0	IMs
PID1	0	6.6962669	0.1	0.001	0	IMs
SRGAP1	0	5.19235654	0.103	0.004	0	IMs
MMP9	0	6.32171248	0.098	0.001	0	IMs
CD1E	0	5.18340595	0.097	0.001	0	IMs
CHST2	0	4.74539291	0.099	0.003	0	IMs
CCL17	0	5.06946986	0.094	0.002	0	IMs
EMP2	0	5.02627011	0.092	0.003	0	IMs
ASGR2	0	5.75076242	0.088	0.002	0	IMs
CHST15	0	4.42433391	0.086	0.002	0	IMs
HGF	0	5.74522277	0.083	0.002	0	IMs
EPHB3	0	5.29631589	0.081	0.002	0	IMs
CCL8	0	6.40037714	0.073	0.001	0	IMs
ITGA9	0	6.5973002	0.071	0	0	IMs
PARM1	0	4.26920062	0.073	0.002	0	IMs
ITGB3	0	6.51255856	0.07	0.001	0	IMs
ADGRG6	0	5.57664968	0.069	0.002	0	IMs
TNFRSF11A	0	4.82626599	0.068	0.001	0	IMs
TMEM121B	0	5.59784879	0.069	0.002	0	IMs
MYCL	0	4.99221004	0.067	0.002	0	IMs
ADAMDEC1	0	6.42366826	0.065	0.001	0	IMs
NCS1	0	4.23361246	0.066	0.002	0	IMs
SLC35F2	0	3.02468104	0.066	0.002	0	IMs
CYP1B1	0	5.9213693	0.064	0.001	0	IMs
LTBP2	0	5.77524788	0.064	0.001	0	IMs
CHST13	0	5.21749532	0.06	0.002	0	IMs
HS3ST2	0	7.19817685	0.055	0.001	0	IMs
CD1A	0	4.22020516	0.053	0.001	0	IMs
SLC7A11	0	5.0964119	0.053	0.001	0	IMs
CCL7	0	7.46549694	0.052	0.001	0	IMs
HTRA1	0	4.80701711	0.049	0.001	0	IMs
PTGIR	0	5.52638626	0.049	0.001	0	IMs
TNFAIP8L3	0	5.80316098	0.048	0.001	0	IMs
KCP	0	6.53812861	0.046	0.001	0	IMs
CLEC4F	0	4.33291943	0.045	0.001	0	IMs

NCKAP5	0	5.29679692	0.045	0.001	0	IMs
CD1B	0	6.11632381	0.039	0	0	IMs
ADRB3	0	5.30202474	0.04	0.001	0	IMs
PLA2G2D	0	7.41484286	0.038	0.001	0	IMs
HTR7	0	6.17650902	0.037	0	0	IMs
GGT5	0	6.85569378	0.036	0	0	IMs
COL23A1	0	5.85075832	0.035	0	0	IMs
MMP7	0	10.3336625	0.016	0	0	IMs
PXN	2.661E-302	2.76411369	0.211	0.024	4.09E-298	IMs
SH3RF3	9.886E-298	5.30464675	0.032	0.001	1.52E-293	IMs
HSPA6	1.448E-294	3.19236235	0.667	0.251	2.225E-290	IMs
FZD2	4.349E-293	4.29721608	0.145	0.012	6.685E-289	IMs
FLT3	4.711E-290	4.23495606	0.048	0.001	7.242E-286	IMs
HAMP	1.378E-287	6.27887928	0.091	0.005	2.118E-283	IMs
CSF1R	5.23E-286	1.89162696	0.951	0.861	8.039E-282	IMs
S1PR2	9.764E-283	3.15581043	0.431	0.101	1.501E-278	IMs
TMEM176A	1.69E-281	2.59215608	0.651	0.232	2.597E-277	IMs
PRDM1	8.873E-281	1.97263789	0.309	0.052	1.364E-276	IMs
TYMP	1.525E-280	1.25911728	0.997	0.959	2.344E-276	IMs
LAMP3	1.259E-279	4.05428208	0.099	0.006	1.934E-275	IMs
TGFB2	1.043E-278	5.25748785	0.063	0.002	1.603E-274	IMs
PDPN	2.053E-278	7.95800483	0.014	0	3.156E-274	IMs
ZNF703	1.318E-277	4.53174679	0.082	0.004	2.026E-273	IMs
TLR2	5.601E-274	2.68377926	0.721	0.323	8.609E-270	IMs
CTTNBP2	5.008E-273	5.49577276	0.024	0	7.698E-269	IMs
NPDC1	1.185E-272	2.24075963	0.09	0.005	1.821E-268	IMs
PEA15	1.252E-272	1.91650193	0.933	0.751	1.925E-268	IMs
TSPAN33	2.18E-270	3.50829298	0.334	0.064	3.351E-266	IMs
PTPN7	5.646E-269	1.70209284	0.187	0.021	8.678E-265	IMs
CRIP2	5.603E-267	3.42415469	0.134	0.011	8.613E-263	IMs
TCF4	1.015E-266	3.20176638	0.363	0.075	1.561E-262	IMs
CLEC4G	1.7E-262	6.228877	0.033	0.001	2.613E-258	IMs
STK32B	2.147E-258	5.53090305	0.046	0.001	3.3E-254	IMs
HAPLN3	1.776E-256	3.24009057	0.196	0.024	2.73E-252	IMs
MMP14	3.166E-256	2.17258162	0.863	0.603	4.866E-252	IMs
CD93	1.146E-255	3.87038327	0.381	0.087	1.762E-251	IMs
ABCA9	5.518E-255	6.98118336	0.022	0	8.482E-251	IMs
CAVIN1	2.346E-254	5.18713123	0.031	0.001	3.606E-250	IMs
SLC39A8	4.39E-252	2.88160763	0.432	0.107	6.748E-248	IMs
CCSER1	2.686E-251	3.71790992	0.059	0.002	4.128E-247	IMs
PDGFB	3.233E-251	3.33495627	0.377	0.084	4.969E-247	IMs
SERPINF1	1.582E-250	3.16446955	0.363	0.079	2.432E-246	IMs
BASP1	9.422E-250	2.81188367	0.354	0.074	1.448E-245	IMs
DOCK4	2.096E-248	2.35366412	0.718	0.333	3.221E-244	IMs
CYTL1	4.121E-247	6.03346243	0.041	0.001	6.334E-243	IMs
CXCR4	1.515E-246	1.97216536	0.762	0.355	2.329E-242	IMs
ABCA6	1.721E-246	5.39852374	0.082	0.005	2.645E-242	IMs
ZFP36L1	1.126E-245	2.18878323	0.85	0.555	1.73E-241	IMs
RNF150	2.159E-244	5.20036465	0.039	0.001	3.318E-240	IMs
ELAVL4	6.818E-242	5.6267018	0.02	0	1.048E-237	IMs
IL31RA	2.212E-239	5.91032827	0.021	0	3.4E-235	IMs
GSDMA	1.475E-234	4.9118264	0.078	0.004	2.267E-230	IMs

DCHS1	1.36E-231	3.94858119	0.135	0.013	2.09E-227	IMs
PTGS1	4.741E-231	2.71231397	0.555	0.197	7.288E-227	IMs
KDM7A	7.14E-229	2.61436085	0.408	0.105	1.098E-224	IMs
MS4A6A	5.048E-228	2.19044422	0.915	0.821	7.759E-224	IMs
IDO1	3.495E-226	4.64510974	0.079	0.005	5.373E-222	IMs
ENHO	1.213E-223	5.00919472	0.054	0.002	1.864E-219	IMs
CHST11	2.102E-223	1.86416428	0.824	0.516	3.231E-219	IMs
TLR3	1.626E-221	3.8616564	0.164	0.02	2.499E-217	IMs
BCAT1	2.234E-221	2.63060116	0.498	0.158	3.433E-217	IMs
QPCT	2.331E-220	2.71217905	0.507	0.163	3.583E-216	IMs
ADORA3	5.915E-219	4.56897999	0.145	0.016	9.093E-215	IMs
ASAP2	4.531E-216	4.20198342	0.055	0.002	6.965E-212	IMs
LILRB2	6.985E-215	2.21304348	0.762	0.432	1.074E-210	IMs
SHE	1.677E-213	4.96779526	0.044	0.001	2.577E-209	IMs
LHFPL2	3.407E-213	1.83762636	0.869	0.722	5.236E-209	IMs
SLC8A1	1.183E-212	1.40469433	0.958	0.855	1.819E-208	IMs
DPYSL3	4.03E-212	4.91068076	0.077	0.005	6.194E-208	IMs
SEMA4A	7.619E-212	2.81994717	0.54	0.196	1.171E-207	IMs
ICAM1	3.85E-211	2.3531611	0.683	0.325	5.918E-207	IMs
TNS1	7.094E-210	2.62359733	0.467	0.144	1.09E-205	IMs
METRNL	9.944E-210	1.46577476	0.954	0.855	1.529E-205	IMs
ITGAM	2.507E-208	1.6340717	0.883	0.598	3.853E-204	IMs
SATB1	1.985E-207	2.4609987	0.37	0.093	3.051E-203	IMs
ANXA6	2.675E-207	1.47638194	0.404	0.103	4.112E-203	IMs
CD48	1.271E-200	1.4717099	0.34	0.079	1.954E-196	IMs
IL1R1	3.767E-200	3.2129718	0.207	0.033	5.79E-196	IMs
HOMER3	2.785E-196	3.0518682	0.29	0.062	4.281E-192	IMs
DNM1	4.394E-196	3.85723052	0.178	0.025	6.754E-192	IMs
MARCH9	6.975E-195	2.35832726	0.303	0.067	1.072E-190	IMs
KCNMB1	9.256E-195	2.98000812	0.311	0.071	1.423E-190	IMs
MBOAT2	1.589E-192	3.20649816	0.056	0.003	2.442E-188	IMs
ST18	1.663E-189	5.86807639	0.018	0	2.556E-185	IMs
IL1RL2	4.575E-189	5.15021858	0.033	0.001	7.032E-185	IMs
MAFB	4.796E-188	2.43600348	0.797	0.551	7.371E-184	IMs
NIBAN2	2.197E-187	1.93425258	0.766	0.436	3.377E-183	IMs
ZEB2	3.146E-186	1.16054234	0.961	0.92	4.836E-182	IMs
PIK3R6	3.201E-185	3.19508755	0.199	0.032	4.92E-181	IMs
RAB3D	1.651E-181	3.78395389	0.124	0.014	2.537E-177	IMs
ACAP1	4.719E-181	0.53765647	0.179	0.026	7.254E-177	IMs
SPP1	5.317E-181	7.72227122	0.085	0.007	8.172E-177	IMs
LILRB4	8.409E-181	3.20181385	0.291	0.067	1.293E-176	IMs
CEBPD	1.042E-180	2.43073639	0.712	0.406	1.601E-176	IMs
VEGFA	5.271E-180	2.91107178	0.588	0.261	8.101E-176	IMs
PMP22	6.485E-180	2.12608539	0.762	0.473	9.968E-176	IMs
ENTPD1	7.184E-177	2.15015377	0.655	0.338	1.104E-172	IMs
SLAMF8	1.952E-176	1.5976749	0.84	0.628	3.001E-172	IMs
ARHGAP45	2.195E-176	1.76764304	0.461	0.152	3.374E-172	IMs
SDC2	8.054E-176	3.47552614	0.215	0.039	1.238E-171	IMs
S1PR3	5.246E-174	5.26863873	0.024	0.001	8.064E-170	IMs
CDK6	6.764E-173	2.07436066	0.134	0.016	1.04E-168	IMs
XYLT1	1.024E-172	2.68982863	0.119	0.013	1.574E-168	IMs
TRIM47	2.312E-172	2.80087505	0.318	0.081	3.553E-168	IMs

CIITA	1.064E-170	1.39290529	0.909	0.812	1.635E-166	IMs
CST6	1.116E-170	4.92687437	0.064	0.004	1.715E-166	IMs
GPR68	4.181E-170	1.73496393	0.069	0.005	6.427E-166	IMs
NFKB2	7.24E-170	2.16287894	0.526	0.208	1.113E-165	IMs
PSTPIP1	8.488E-170	1.74419979	0.402	0.121	1.305E-165	IMs
ZBTB46	9.026E-169	3.28350068	0.199	0.035	1.387E-164	IMs
CLEC5A	4.904E-168	3.28503138	0.492	0.191	7.538E-164	IMs
SCO2	3.599E-167	1.83811126	0.808	0.57	5.531E-163	IMs
GNAO1	1.044E-166	2.82086134	0.07	0.005	1.605E-162	IMs
KCTD12	1.908E-166	0.91190609	0.97	0.938	2.933E-162	IMs
ABCB4	1.338E-162	5.63263746	0.02	0	2.057E-158	IMs
UCK2	1.406E-162	3.09388154	0.176	0.029	2.16E-158	IMs
SH3RF1	4.142E-162	3.85600149	0.078	0.006	6.366E-158	IMs
CD300LB	8.331E-161	4.03839474	0.136	0.018	1.281E-156	IMs
CLEC2D	2.193E-160	0.53525504	0.241	0.049	3.371E-156	IMs
LGMN	9.462E-160	3.4598093	0.726	0.512	1.454E-155	IMs
TACSTD2	2.233E-159	2.29594519	0.058	0.004	3.432E-155	IMs
GEM	2.561E-159	6.09859494	0.024	0.001	3.937E-155	IMs
ANKH	4.834E-159	2.62081949	0.376	0.116	7.43E-155	IMs
GFRA2	1.634E-158	4.15524314	0.153	0.023	2.511E-154	IMs
PALM2-AKAP2	1.709E-157	2.79732515	0.295	0.075	2.627E-153	IMs
SOCS3	4.549E-156	1.44208824	0.894	0.698	6.992E-152	IMs
VCAN	1.734E-155	2.20596824	0.572	0.254	2.665E-151	IMs
APOBEC3A	2.126E-154	4.39655766	0.13	0.017	3.268E-150	IMs
TRERF1	2.759E-154	1.59707795	0.115	0.014	4.241E-150	IMs
ELK3	1.314E-153	2.4348094	0.275	0.067	2.019E-149	IMs
CD300A	1.916E-153	1.94338832	0.659	0.359	2.945E-149	IMs
NPC2	2.111E-153	0.84723096	0.981	0.946	3.245E-149	IMs
KIFC3	1.034E-151	2.29084819	0.475	0.185	1.59E-147	IMs
EMP3	1.347E-151	1.16391739	0.978	0.958	2.07E-147	IMs
CALHM6	1.876E-151	2.90836096	0.393	0.132	2.884E-147	IMs
JUP	1.202E-150	2.18042383	0.465	0.17	1.848E-146	IMs
HM13	3.605E-150	1.56642426	0.827	0.66	5.541E-146	IMs
HS3ST3B1	3.917E-150	2.58912358	0.164	0.027	6.02E-146	IMs
AHR	4.511E-150	1.42718843	0.883	0.794	6.934E-146	IMs
UTF1	8.822E-150	6.1523144	0.013	0	1.356E-145	IMs
DOCK5	2.349E-149	1.86861094	0.6	0.291	3.611E-145	IMs
PDGFA	2.891E-149	4.10469694	0.028	0.001	4.443E-145	IMs
POU2F2	2.926E-148	2.91342869	0.161	0.026	4.497E-144	IMs
FYB1	2.349E-146	1.33920317	0.766	0.524	3.611E-142	IMs
CCND1	3.954E-146	4.10604884	0.063	0.005	6.077E-142	IMs
RNASE6	5.367E-146	2.04255585	0.612	0.306	8.25E-142	IMs
IER5L	5.702E-146	2.29407364	0.492	0.201	8.765E-142	IMs
GNG2	2.984E-145	0.39082031	0.134	0.018	4.587E-141	IMs
BNC2	7.048E-145	4.14882216	0.067	0.005	1.083E-140	IMs
FLRT2	1.297E-143	5.05170795	0.037	0.002	1.994E-139	IMs
MMP25	3.694E-143	2.82699018	0.033	0.001	5.678E-139	IMs
KLF6	1.452E-142	1.33288683	0.929	0.82	2.232E-138	IMs
TTYH3	2.229E-142	2.0401151	0.694	0.461	3.426E-138	IMs
BCL2	5.129E-142	1.17863263	0.273	0.068	7.884E-138	IMs
CHRNA6	7.062E-141	5.64725947	0.014	0	1.086E-136	IMs
KCNK13	1.277E-140	3.19602746	0.21	0.044	1.963E-136	IMs

PRCP	2.455E-140	1.64285824	0.704	0.447	3.773E-136	IMs
ZFP36	1.308E-139	1.38247861	0.918	0.87	2.01E-135	IMs
TRPS1	4.192E-139	1.89014995	0.636	0.348	6.444E-135	IMs
ADGRE2	2.223E-138	2.38116336	0.447	0.173	3.417E-134	IMs
TNFSF8	3.244E-138	2.47201275	0.083	0.008	4.986E-134	IMs
RASL10A	9.271E-138	4.47702181	0.048	0.003	1.425E-133	IMs
NFATC2	4.543E-135	1.39243995	0.225	0.05	6.982E-131	IMs
MROH7	9.138E-135	4.11142945	0.071	0.006	1.405E-130	IMs
CD84	1.259E-134	1.97386555	0.641	0.389	1.936E-130	IMs
RTL5	3.799E-134	2.56679785	0.233	0.054	5.839E-130	IMs
PLEK2	7.893E-134	3.54047522	0.143	0.023	1.213E-129	IMs
SCIN	8.238E-134	4.93460983	0.044	0.002	1.266E-129	IMs
EYA2	1.214E-133	4.53027224	0.063	0.005	1.866E-129	IMs
EML1	1.316E-133	5.0402852	0.022	0.001	2.023E-129	IMs
GPR35	2.479E-133	2.90408822	0.133	0.02	3.81E-129	IMs
RASGRP3	2.877E-133	2.58919408	0.226	0.052	4.423E-129	IMs
ADAMTS10	3.192E-132	3.56572252	0.021	0.001	4.907E-128	IMs
STARD13	7.537E-132	3.31436199	0.161	0.029	1.159E-127	IMs
IER3	9.072E-132	2.64659721	0.593	0.323	1.394E-127	IMs
PPP1R14A	1.436E-131	3.97537957	0.024	0.001	2.207E-127	IMs
RGS1	2.81E-131	2.25473908	0.72	0.487	4.319E-127	IMs
SLC37A2	5.915E-131	1.70072012	0.702	0.459	9.092E-127	IMs
CRISPLD2	2.607E-130	4.6539797	0.028	0.001	4.007E-126	IMs
RASAL1	4.701E-129	4.45582533	0.017	0	7.227E-125	IMs
NHSL2	1.234E-128	2.28623764	0.173	0.033	1.897E-124	IMs
MMP12	2.575E-128	6.6895273	0.021	0.001	3.958E-124	IMs
FXYD2	3.85E-128	3.68134853	0.051	0.003	5.918E-124	IMs
CAMK1	1.003E-127	2.80335323	0.347	0.117	1.541E-123	IMs
TPPP3	1.219E-127	2.46170779	0.071	0.007	1.874E-123	IMs
LPAR5	1.556E-127	2.38549443	0.292	0.085	2.392E-123	IMs
SLC36A1	2.256E-126	2.40497597	0.347	0.119	3.467E-122	IMs
RELB	1.423E-125	1.94999294	0.409	0.152	2.187E-121	IMs
FCER2	3.341E-125	4.86746436	0.023	0.001	5.136E-121	IMs
KLHL5	3.84E-125	1.99860221	0.491	0.22	5.903E-121	IMs
NECTIN2	4.543E-125	1.69897517	0.682	0.442	6.983E-121	IMs
ALCAM	2.637E-124	1.62824497	0.806	0.681	4.054E-120	IMs
EIF4E3	1.52E-123	0.66776934	0.305	0.089	2.336E-119	IMs
CHI3L1	2.325E-121	5.32469481	0.107	0.015	3.574E-117	IMs
P2RY14	4.022E-121	2.9081721	0.235	0.06	6.183E-117	IMs
PLK3	3.008E-119	1.72596473	0.581	0.305	4.624E-115	IMs
ST14	3.367E-119	1.3671731	0.797	0.658	5.175E-115	IMs
PLBD2	1.162E-118	1.13358276	0.845	0.76	1.786E-114	IMs
ALDH1L2	2.786E-117	3.82609968	0.098	0.013	4.283E-113	IMs
TDRD10	6.272E-117	5.328251	0.016	0	9.64E-113	IMs
AP1S2	2.044E-116	1.76857258	0.583	0.316	3.141E-112	IMs
SEMA4C	4.511E-116	2.45436677	0.04	0.002	6.934E-112	IMs
GASK1A	7.053E-116	4.86787784	0.03	0.001	1.084E-111	IMs
IFITM3	1.53E-115	1.71066266	0.736	0.557	2.352E-111	IMs
DPYD	2.153E-115	1.11270336	0.879	0.763	3.31E-111	IMs
SLC2A1	5.828E-115	2.00184834	0.114	0.017	8.958E-111	IMs
ADAMTS2	1.528E-114	6.76522263	0.01	0	2.349E-110	IMs
MTHFD2	1.675E-114	1.53600273	0.741	0.549	2.574E-110	IMs

CD226	2.669E-114	1.34830285	0.1	0.013	4.102E-110	IMs
CD14	6.735E-114	1.45625641	0.871	0.814	1.035E-109	IMs
CLEC7A	1.11E-113	0.91846547	0.929	0.908	1.706E-109	IMs
ZNF467	4.343E-113	3.49767873	0.09	0.011	6.675E-109	IMs
NEDD4L	5.235E-112	2.41357996	0.198	0.046	8.047E-108	IMs
NINJ1	8.719E-112	1.91157206	0.642	0.41	1.34E-107	IMs
TGFBR1	9.487E-112	1.43852785	0.801	0.65	1.458E-107	IMs
IGFLR1	3.751E-110	1.48308417	0.706	0.485	5.766E-106	IMs
CLEC11A	4.038E-109	2.60130548	0.185	0.042	6.207E-105	IMs
ODF3B	3.567E-108	1.54814845	0.736	0.525	5.483E-104	IMs
ADAMTS14	6.105E-108	4.74849979	0.02	0.001	9.385E-104	IMs
CD99	1.598E-107	0.95685141	0.861	0.744	2.457E-103	IMs
LPAR6	1.995E-107	2.47410303	0.49	0.246	3.067E-103	IMs
TFEB	2.171E-107	2.06838607	0.41	0.173	3.336E-103	IMs
SORL1	3.42E-107	1.61058827	0.655	0.418	5.256E-103	IMs
FOS	6.458E-107	1.09533056	0.965	0.95	9.926E-103	IMs
CSF3R	1.718E-106	1.51347619	0.674	0.447	2.641E-102	IMs
SOD21	2.747E-106	1.71090678	0.87	0.808	4.223E-102	IMs
ARNTL2	4.361E-106	2.80487478	0.168	0.036	6.704E-102	IMs
TVP23A	1.795E-105	3.84672826	0.076	0.009	2.759E-101	IMs
RAP2B	2.764E-105	1.28919208	0.777	0.641	4.248E-101	IMs
TNFRSF1B	3.545E-105	1.61581588	0.671	0.439	5.449E-101	IMs
NEURL3	3.8E-105	6.00484864	0.016	0	5.841E-101	IMs
FUT7	4.819E-105	2.80134474	0.122	0.021	7.408E-101	IMs
HAVCR21	1.02E-104	0.97220562	0.89	0.826	1.568E-100	IMs
ACOT11	4.365E-104	2.772349	0.195	0.048	6.709E-100	IMs
CTSZ1	3.6E-102	0.68552444	0.996	0.974	5.5332E-98	IMs
GNB4	7.884E-102	1.0180188	0.842	0.767	1.2119E-97	IMs
CXCL91	9.343E-102	3.37948233	0.142	0.028	1.4361E-97	IMs
CHD3	1.404E-101	1.24131314	0.314	0.106	2.1577E-97	IMs
SIGLEC10	7.155E-101	2.72529874	0.272	0.087	1.0999E-96	IMs
CCL3	2.263E-100	2.63872355	0.154	0.032	3.4785E-96	IMs
SYNE3	7.171E-100	1.48222296	0.353	0.134	1.1023E-95	IMs
SCN1B	9.219E-100	3.64455655	0.039	0.003	1.417E-95	IMs
FOLR2	1.101E-99	4.18553332	0.338	0.135	1.6921E-95	IMs
NRIP3	1.833E-99	3.62916511	0.09	0.013	2.8182E-95	IMs
TMEM51	3.799E-99	1.88191074	0.516	0.276	5.84E-95	IMs
CD207	1.6784E-98	3.79356537	0.019	0.001	2.5799E-94	IMs
DENND3	7.5819E-98	1.61460149	0.499	0.252	1.1654E-93	IMs
CXCL101	8.0277E-98	2.65357416	0.19	0.047	1.2339E-93	IMs
AHNAK	1.7256E-97	0.62380663	0.982	0.979	2.6524E-93	IMs
MREG	2.5502E-97	2.54047054	0.281	0.094	3.92E-93	IMs
UBD1	2.7546E-97	3.21827876	0.075	0.009	4.2341E-93	IMs
ADAM19	7.0447E-97	1.0528253	0.11	0.018	1.0828E-92	IMs
JAK3	8.6863E-97	1.57572498	0.228	0.064	1.3352E-92	IMs
CMTM3	2.4492E-96	1.02704339	0.85	0.712	3.7647E-92	IMs
IDO2	4.8834E-96	4.3064655	0.018	0.001	7.5062E-92	IMs
CTSB1	1.6186E-95	0.76909889	1	0.985	2.4879E-91	IMs
CDH23	2.8769E-95	4.36352747	0.074	0.009	4.422E-91	IMs
LTC4S	6.533E-95	3.3708949	0.04	0.003	1.0042E-90	IMs
TAP1	9.3311E-95	1.23951079	0.826	0.73	1.4343E-90	IMs
PLCH1	6.4892E-94	3.7307982	0.019	0.001	9.9745E-90	IMs

TLR6	2.3032E-93	2.29153802	0.289	0.101	3.5403E-89	IMs
CDKN1A	2.0286E-92	1.33843513	0.614	0.336	3.1181E-88	IMs
PLEKHA7	5.4937E-92	2.46479875	0.314	0.118	8.4443E-88	IMs
PFKP	1.1422E-91	1.68148435	0.612	0.398	1.7557E-87	IMs
CREB5	5.3564E-91	2.21062292	0.366	0.156	8.2333E-87	IMs
SCN9A	3.8263E-90	3.27723684	0.096	0.015	5.8813E-86	IMs
FUT1	6.2491E-90	4.97496978	0.01	0	9.6055E-86	IMs
TREM2	6.6193E-90	1.37434578	0.723	0.559	1.0175E-85	IMs
HIC1	9.412E-90	3.8159549	0.026	0.001	1.4467E-85	IMs
BMP2	1.1558E-89	4.42886063	0.023	0.001	1.7765E-85	IMs
DNMT1	2.1462E-89	1.40528951	0.712	0.533	3.2989E-85	IMs
PAK1	2.3048E-89	1.52205759	0.634	0.427	3.5428E-85	IMs
MFSD1	9.2844E-89	0.60592055	0.957	0.943	1.4271E-84	IMs
KCNJ51	1.598E-88	3.02490954	0.122	0.024	2.4562E-84	IMs
NME8	1.614E-88	4.47568279	0.029	0.002	2.4808E-84	IMs
PAPSS2	2.0748E-88	2.07000247	0.376	0.163	3.1892E-84	IMs
MCOLN2	4.4012E-88	2.02352713	0.225	0.067	6.7651E-84	IMs
LSR	9.6412E-88	1.82169809	0.055	0.006	1.4819E-83	IMs
PTGER2	1.065E-87	1.56183935	0.268	0.09	1.637E-83	IMs
PLA2G4C	2.3586E-87	2.27631919	0.27	0.093	3.6255E-83	IMs
OTULINL	5.4946E-87	1.24092975	0.733	0.583	8.4457E-83	IMs
TGFBI	5.8308E-87	1.06802909	0.96	0.946	8.9625E-83	IMs
CSF2RA	7.9282E-87	1.02580366	0.906	0.875	1.2186E-82	IMs
IL10RA	9.0076E-87	0.81448729	0.879	0.817	1.3846E-82	IMs
KLF2	9.4464E-87	1.61814758	0.782	0.665	1.452E-82	IMs
GNA13	3.2533E-86	0.9311089	0.897	0.838	5.0006E-82	IMs
GSDME	5.5197E-86	2.88502405	0.178	0.046	8.4844E-82	IMs
CPM	1.2125E-85	1.49555019	0.795	0.739	1.8637E-81	IMs
RALGDS	1.3521E-85	1.27626286	0.698	0.533	2.0783E-81	IMs
RCSD1	1.4456E-85	1.42528836	0.508	0.273	2.222E-81	IMs
TRAF3IP3	3.0995E-85	0.55153495	0.146	0.032	4.7642E-81	IMs
GAS7	4.3537E-85	1.39074069	0.642	0.439	6.692E-81	IMs
IGFBP7	2.6237E-84	2.46328016	0.154	0.036	4.0329E-80	IMs
CXCL111	2.8517E-84	2.43078637	0.094	0.015	4.3834E-80	IMs
EPSTI1	8.7352E-84	1.03268411	0.79	0.674	1.3427E-79	IMs
FOSB	1.5497E-83	2.06062693	0.399	0.182	2.3821E-79	IMs
MAT2A	2.5463E-83	1.01027927	0.886	0.838	3.9139E-79	IMs
TMEM71	3.6992E-83	1.49714029	0.12	0.024	5.686E-79	IMs
SLC4A7	7.6514E-83	1.6695418	0.378	0.168	1.1761E-78	IMs
CST7	1.3485E-82	0.87330884	0.089	0.014	2.0727E-78	IMs
MAP3K3	2.4218E-82	1.44969011	0.513	0.284	3.7225E-78	IMs
TAGAP	3.8431E-82	1.10349934	0.744	0.572	5.9073E-78	IMs
SH3KBP1	1.0953E-81	1.33496544	0.612	0.404	1.6836E-77	IMs
TIFAB	2.4201E-81	3.66491041	0.079	0.012	3.72E-77	IMs
KCNJ15	6.9775E-81	3.00789914	0.045	0.004	1.0725E-76	IMs
PALLD	7.121E-81	2.4411082	0.22	0.069	1.0946E-76	IMs
EBI3	1.6311E-80	4.58122533	0.025	0.001	2.5071E-76	IMs
SYNJ2	2.1728E-80	2.46377632	0.066	0.008	3.3399E-76	IMs
ZC3H12C	6.7481E-80	3.26850917	0.081	0.012	1.0373E-75	IMs
RBPJ	7.1327E-80	1.11070651	0.802	0.718	1.0964E-75	IMs
CTSK	1.0994E-79	3.43679297	0.21	0.066	1.6899E-75	IMs
EGR1	2.8143E-79	2.10279574	0.266	0.093	4.3259E-75	IMs

SPRY2	5.5748E-79	2.82612528	0.087	0.014	8.569E-75	IMs
ATF3	3.3396E-78	2.30706773	0.385	0.181	5.1332E-74	IMs
NECTIN4	6.5882E-78	3.11544642	0.045	0.004	1.0127E-73	IMs
SYT17	9.9932E-78	2.98573447	0.111	0.022	1.5361E-73	IMs
FAM149A	3.8237E-77	4.48408811	0.022	0.001	5.8774E-73	IMs
PROC	7.6217E-77	4.98371585	0.015	0	1.1715E-72	IMs
IER2	7.6653E-77	1.32230556	0.786	0.68	1.1782E-72	IMs
STK38L	9.3344E-77	1.59653544	0.568	0.362	1.4348E-72	IMs
CCR1	1.9845E-76	1.25844554	0.676	0.507	3.0503E-72	IMs
GATM	7.9024E-76	2.61675707	0.188	0.056	1.2147E-71	IMs
IL1R2	9.1044E-76	2.63409591	0.032	0.002	1.3994E-71	IMs
PTPRF	5.4598E-75	3.49332357	0.025	0.001	8.3923E-71	IMs
PLEKHG2	9.5504E-75	2.70315265	0.115	0.024	1.468E-70	IMs
VMP1	9.553E-75	0.92295286	0.827	0.741	1.4684E-70	IMs
PTAFR	1.6648E-74	0.91982425	0.854	0.802	2.5589E-70	IMs
PRLR	6.8211E-74	5.83489778	0.01	0	1.0485E-69	IMs
RNF19B	9.2434E-74	1.10134703	0.809	0.716	1.4208E-69	IMs
TMEM106A	1.1775E-73	1.37639694	0.614	0.429	1.8099E-69	IMs
HIF1A	1.5776E-73	1.83966686	0.414	0.213	2.425E-69	IMs
ITGAX1	9.5105E-73	0.98808264	0.891	0.799	1.4619E-68	IMs
MUC1	1.8358E-72	1.25871682	0.06	0.008	2.8219E-68	IMs
PDE4D	6.711E-72	0.54590175	0.12	0.026	1.0315E-67	IMs
C1S	1.0596E-71	2.94811353	0.111	0.023	1.6288E-67	IMs
CBX6	1.9205E-71	1.56167316	0.462	0.255	2.952E-67	IMs
SLC37A3	4.2516E-71	1.54689357	0.048	0.005	6.5351E-67	IMs
ATF5	1.0434E-70	2.08124048	0.472	0.278	1.6038E-66	IMs
CD163L1	1.3174E-70	3.29508997	0.084	0.014	2.025E-66	IMs
VAMP5	1.3777E-70	1.80652179	0.445	0.243	2.1177E-66	IMs
MEGF6	1.3869E-70	2.07829223	0.153	0.04	2.1318E-66	IMs
SLC43A3	5.1118E-70	1.01852542	0.779	0.677	7.8574E-66	IMs
BCO2	5.8199E-70	3.19870923	0.045	0.005	8.9458E-66	IMs
ADAP21	1.293E-69	1.3050829	0.689	0.566	1.9875E-65	IMs
RSRP1	5.5086E-69	0.64588742	0.869	0.71	8.4672E-65	IMs
GIMAP7	1.0227E-68	0.96710719	0.281	0.113	1.572E-64	IMs
ENPP1	1.1729E-68	2.9085167	0.018	0.001	1.8029E-64	IMs
WIPF1	2.218E-68	0.47568107	0.948	0.945	3.4093E-64	IMs
FSCN1	7.1871E-68	3.06775497	0.052	0.006	1.1047E-63	IMs
RAPH1	7.363E-68	2.06693188	0.349	0.168	1.1318E-63	IMs
STARD8	9.1799E-68	1.64892036	0.457	0.261	1.411E-63	IMs
MICAL2	1.8346E-67	1.83425112	0.386	0.196	2.82E-63	IMs
SWAP70	5.4211E-67	1.03387297	0.718	0.603	8.3328E-63	IMs
ARID5B	8.7465E-67	1.38301178	0.244	0.089	1.3444E-62	IMs
RGS10	2.9646E-66	1.27837711	0.65	0.512	4.5568E-62	IMs
SLC18B1	7.3795E-66	2.14956082	0.151	0.041	1.1343E-61	IMs
SMPDL3A	8.2587E-66	2.66709562	0.139	0.036	1.2694E-61	IMs
GALM	1.7132E-65	1.93532382	0.309	0.137	2.6334E-61	IMs
ABCA1	3.6164E-65	1.84934686	0.518	0.332	5.5587E-61	IMs
CADM1	3.886E-65	3.19610676	0.06	0.008	5.9732E-61	IMs
CFLAR	3.9504E-65	0.68303147	0.915	0.876	6.0722E-61	IMs
PLXNA1	4.3343E-65	1.96108469	0.345	0.165	6.6623E-61	IMs
HAAO	5.1601E-65	2.11526978	0.243	0.091	7.9316E-61	IMs
IL6R	1.2038E-64	1.75201429	0.364	0.178	1.8504E-60	IMs

ATP2B4	1.6324E-64	1.18791912	0.462	0.265	2.5092E-60	IMs
LENG8	1.6412E-64	0.61621903	0.798	0.65	2.5228E-60	IMs
BCL11A	5.6547E-64	2.16430054	0.184	0.058	8.6918E-60	IMs
KLF10	9.437E-64	1.26032854	0.647	0.486	1.4506E-59	IMs
CERS4	2.094E-63	1.8470687	0.04	0.004	3.2187E-59	IMs
C3AR1	4.247E-63	1.06656198	0.77	0.68	6.5281E-59	IMs
SLC1A4	4.9187E-63	1.90543966	0.392	0.21	7.5606E-59	IMs
METTL7A	7.2209E-63	1.69509372	0.445	0.252	1.1099E-58	IMs
CAMK1D	8.2502E-63	1.2771917	0.628	0.461	1.2681E-58	IMs
BMP2K	8.5579E-63	1.31386204	0.622	0.491	1.3154E-58	IMs
LGALS1	4.7911E-62	0.55401075	0.981	0.966	7.3644E-58	IMs
FAM20C	5.9625E-62	1.85675359	0.497	0.319	9.165E-58	IMs
RASSF41	1.3784E-61	1.17223117	0.709	0.613	2.1187E-57	IMs
ITPRIPL2	7.5186E-61	1.07559445	0.703	0.6	1.1557E-56	IMs
SLC26A11	1.315E-60	1.78771971	0.386	0.205	2.0213E-56	IMs
TRAF5	2.3011E-60	1.50726553	0.205	0.072	3.537E-56	IMs
MAP3K14	3.2621E-60	2.06538046	0.143	0.04	5.0142E-56	IMs
LRRK2	5.2854E-60	1.73184223	0.407	0.222	8.1241E-56	IMs
SCRN1	5.681E-60	1.81700628	0.336	0.16	8.7322E-56	IMs
PRSS36	7.5219E-60	1.86037363	0.346	0.171	1.1562E-55	IMs
C6orf223	1.246E-59	5.16444748	0.012	0	1.9152E-55	IMs
NRROS	1.3641E-59	1.23401981	0.616	0.463	2.0967E-55	IMs
ANKRD44	1.3661E-59	1.02786273	0.623	0.458	2.0999E-55	IMs
CLCN7	1.465E-59	0.6876856	0.864	0.821	2.2519E-55	IMs
HIVEP3	1.5299E-59	1.70340786	0.393	0.213	2.3516E-55	IMs
MRO	1.6425E-59	3.46870095	0.033	0.003	2.5247E-55	IMs
ZNF618	1.745E-59	2.58991966	0.14	0.039	2.6823E-55	IMs
AFF3	2.6538E-59	3.47109546	0.027	0.002	4.0792E-55	IMs
CRYBB1	2.7042E-59	3.92962046	0.038	0.004	4.1566E-55	IMs
BCL3	4.4831E-59	1.33941851	0.599	0.437	6.8909E-55	IMs
PCNX2	5.8095E-59	1.34849449	0.254	0.103	8.9298E-55	IMs
CTTNBP2NL	5.8356E-59	1.22645474	0.603	0.444	8.9699E-55	IMs
IL18BP	5.9356E-59	1.75901092	0.436	0.252	9.1236E-55	IMs
MEF2A	6.8239E-59	0.95734421	0.775	0.677	1.0489E-54	IMs
SNX29	1.1458E-58	1.48354562	0.472	0.287	1.7611E-54	IMs
EGR3	1.2255E-58	2.73550004	0.041	0.005	1.8837E-54	IMs
RFTN1	2.4974E-58	1.50858477	0.374	0.194	3.8388E-54	IMs
CASS4	2.6561E-58	2.21287103	0.126	0.033	4.0826E-54	IMs
ZNF385A1	5.8824E-58	0.82343349	0.864	0.822	9.0418E-54	IMs
NUP62	1.881E-57	1.03123557	0.685	0.571	2.8913E-53	IMs
PELI2	3.3437E-57	2.12772519	0.2	0.071	5.1396E-53	IMs
FAM49B	4.0555E-57	0.68207407	0.858	0.827	6.2337E-53	IMs
DSE	5.0713E-57	0.80472515	0.883	0.859	7.7951E-53	IMs
HDAC9	1.9439E-56	2.22689391	0.209	0.078	2.9879E-52	IMs
NEB	2.2005E-56	4.35266409	0.023	0.002	3.3824E-52	IMs
KIF1B	2.7727E-56	1.20761024	0.581	0.435	4.2618E-52	IMs
TBC1D14	7.0601E-56	1.26641825	0.594	0.45	1.0852E-51	IMs
SLC43A1	1.0346E-55	1.67457855	0.028	0.002	1.5903E-51	IMs
SNX8	1.3639E-55	1.01403845	0.698	0.578	2.0964E-51	IMs
SCARF1	2.8407E-55	1.93525224	0.279	0.123	4.3665E-51	IMs
NEURL2	2.844E-55	3.32262963	0.056	0.009	4.3715E-51	IMs
AKAP6	4.4683E-55	3.05729447	0.02	0.001	6.8682E-51	IMs

DNPH1	8.0584E-55	1.69962022	0.278	0.124	1.2387E-50	IMs
SBNO2	9.275E-55	1.00277455	0.732	0.634	1.4257E-50	IMs
KHDRBS2	2.6808E-54	4.35121504	0.026	0.002	4.1207E-50	IMs
MXD1	3.0509E-54	1.29609366	0.644	0.495	4.6895E-50	IMs
RUFY4	4.6027E-54	3.77629602	0.051	0.007	7.0748E-50	IMs
CHIT1	5.2771E-54	5.43348628	0.067	0.012	8.1114E-50	IMs
SLC17A9	1.3685E-53	1.78621552	0.292	0.136	2.1035E-49	IMs
SLC38A7	1.8872E-53	1.28778916	0.541	0.38	2.9008E-49	IMs
FNIP2	2.6289E-53	1.9443074	0.391	0.225	4.041E-49	IMs
METTL27	4.2544E-53	3.67870716	0.017	0.001	6.5394E-49	IMs
IL17RA	5.3975E-53	0.80209473	0.814	0.76	8.2964E-49	IMs
PTPRE	7.1845E-53	1.32853301	0.575	0.414	1.1043E-48	IMs
KANK1	9.5816E-53	3.38578516	0.03	0.003	1.4728E-48	IMs
STAMBPL1	1.0378E-52	1.77318213	0.07	0.013	1.5951E-48	IMs
TREML1	2.1837E-52	2.51676609	0.119	0.032	3.3566E-48	IMs
OXER1	2.482E-52	3.20753009	0.06	0.01	3.8151E-48	IMs
PIK3R5	4.2751E-52	0.72914605	0.823	0.774	6.5712E-48	IMs
RHOC	4.9074E-52	1.04451381	0.672	0.542	7.5431E-48	IMs
ENG1	6.7627E-52	0.45181741	0.959	0.944	1.0395E-47	IMs
CD86	7.4906E-52	0.90693878	0.756	0.685	1.1514E-47	IMs
RCBTB2	8.7842E-52	1.47403276	0.522	0.361	1.3502E-47	IMs
LILRA1	1.0353E-51	3.50504569	0.062	0.011	1.5913E-47	IMs
PLXNB21	1.2279E-51	0.60791963	0.905	0.898	1.8874E-47	IMs
SLC2A5	1.2304E-51	3.60692235	0.038	0.004	1.8913E-47	IMs
SLC46A1	1.2994E-51	1.99535227	0.336	0.177	1.9973E-47	IMs
GRAMD4	1.3927E-51	1.36272369	0.51	0.351	2.1407E-47	IMs
S1PR1	2.1991E-51	0.48971688	0.042	0.005	3.3802E-47	IMs
BTBD11	2.4612E-51	2.46629569	0.018	0.001	3.7832E-47	IMs
IFI16	2.7488E-51	0.7034957	0.854	0.828	4.2252E-47	IMs
STAT1	5.6749E-51	0.95126213	0.84	0.842	8.7228E-47	IMs
AHNAK2	7.6346E-51	3.54289647	0.037	0.004	1.1735E-46	IMs
ABHD12	7.7287E-51	0.78794743	0.815	0.779	1.188E-46	IMs
MDM4	9.2841E-51	0.81634425	0.674	0.537	1.4271E-46	IMs
ITGA4	1.3544E-50	0.81215628	0.642	0.491	2.0818E-46	IMs
NAGA	1.7489E-50	0.63262907	0.876	0.841	2.6883E-46	IMs
TPD52L2	2.2699E-50	0.85792102	0.715	0.637	3.489E-46	IMs
ADAM15	3.5141E-50	1.04086914	0.661	0.549	5.4016E-46	IMs
AMDHD1	6.2909E-50	3.80394982	0.021	0.002	9.6698E-46	IMs
RGS2	6.2972E-50	1.41413581	0.639	0.491	9.6794E-46	IMs
RALA	6.5663E-50	1.60740439	0.507	0.356	1.0093E-45	IMs
CA11	7.3696E-50	2.56691849	0.119	0.034	1.1328E-45	IMs
SLC23A2	8.5401E-50	1.55101608	0.372	0.209	1.3127E-45	IMs
PSTPIP2	1.0352E-49	1.83775947	0.293	0.141	1.5911E-45	IMs
LAP3	2.111E-49	0.63966353	0.908	0.901	3.2448E-45	IMs
CD300E	4.0365E-49	2.5253453	0.25	0.111	6.2046E-45	IMs
CYFIP11	4.0729E-49	0.61958891	0.839	0.823	6.2605E-45	IMs
NOL3	4.9199E-49	2.76487263	0.05	0.008	7.5624E-45	IMs
APP	5.4315E-49	0.67229301	0.713	0.525	8.3488E-45	IMs
ZNF469	7.6864E-49	2.97397718	0.054	0.009	1.1815E-44	IMs
LHFPL6	1.4287E-48	4.69518498	0.016	0.001	2.196E-44	IMs
EEPD1	1.4403E-48	2.2325975	0.177	0.065	2.2139E-44	IMs
MGAT11	1.9194E-48	0.49670012	0.968	0.95	2.9503E-44	IMs

ITGAV	2.9634E-48	1.08998983	0.65	0.54	4.555E-44	IMs
REPS2	6.5861E-48	2.34865207	0.105	0.028	1.0124E-43	IMs
B3GNT8	8.5804E-48	3.14393558	0.038	0.005	1.3189E-43	IMs
SLC16A31	1.0268E-47	0.47307674	0.97	0.943	1.5782E-43	IMs
LY9	1.1549E-47	1.46452533	0.046	0.007	1.7752E-43	IMs
PLXDC21	1.581E-47	0.46713025	0.982	0.954	2.4302E-43	IMs
TMIGD3	2.7988E-47	3.52524852	0.043	0.006	4.302E-43	IMs
RNF213	4.6515E-47	0.5477299	0.913	0.899	7.1498E-43	IMs
MALT1	9.8094E-47	1.39605516	0.455	0.297	1.5078E-42	IMs
CD44	1.007E-46	0.51497227	0.991	0.988	1.5478E-42	IMs
RARG	1.0904E-46	2.25481837	0.128	0.039	1.676E-42	IMs
TLR5	2.0004E-46	1.88981652	0.269	0.128	3.0749E-42	IMs
GPR27	6.873E-46	3.03979461	0.025	0.002	1.0564E-41	IMs
C1orf54	1.0393E-45	1.98094906	0.218	0.093	1.5974E-41	IMs
CSGALNACT2	1.3426E-45	0.9333672	0.624	0.495	2.0637E-41	IMs
ZMIZ1	1.3763E-45	0.71281921	0.863	0.83	2.1156E-41	IMs
MGAT4A	1.5796E-45	0.76491684	0.775	0.754	2.428E-41	IMs
GPR155	2.1687E-45	1.85982949	0.158	0.056	3.3336E-41	IMs
HIST1H2BG	2.9598E-45	2.41850369	0.079	0.018	4.5496E-41	IMs
PLTP	4.3234E-45	3.04875248	0.437	0.303	6.6455E-41	IMs
PTGES	5.0296E-45	3.84908007	0.01	0	7.7309E-41	IMs
ABCC5	5.0408E-45	1.56072402	0.369	0.214	7.7482E-41	IMs
P2RY12	5.493E-45	2.1195681	0.214	0.091	8.4432E-41	IMs
CLEC17A	6.6733E-45	3.82269143	0.012	0.001	1.0258E-40	IMs
RUNX2	7.838E-45	1.27515448	0.177	0.067	1.2048E-40	IMs
CLIC2	1.5721E-44	1.37601606	0.446	0.289	2.4165E-40	IMs
ITGB2	1.7434E-44	0.4188241	0.971	0.961	2.6797E-40	IMs
SIPA1L1	2.0124E-44	1.73736336	0.281	0.141	3.0933E-40	IMs
TIMP3	2.1364E-44	6.92577518	0.01	0	3.2838E-40	IMs
CH25H	2.1861E-44	3.23510497	0.138	0.046	3.3602E-40	IMs
P2RY11	2.2572E-44	1.39095771	0.433	0.275	3.4696E-40	IMs
SUSD6	3.0803E-44	0.69836709	0.795	0.746	4.7347E-40	IMs
RAMP1	3.3099E-44	3.34397806	0.019	0.001	5.0876E-40	IMs
HERPUD1	3.5745E-44	0.79814475	0.783	0.739	5.4944E-40	IMs
MYZAP	6.2249E-44	2.61458127	0.096	0.026	9.5683E-40	IMs
IGSF61	6.7025E-44	0.72435091	0.848	0.803	1.0302E-39	IMs
PREX1	1.0476E-43	0.8023435	0.642	0.531	1.6102E-39	IMs
EPB41L3	1.6285E-43	0.71685619	0.792	0.759	2.5032E-39	IMs
MARCKSL1	1.8219E-43	3.04727802	0.103	0.029	2.8005E-39	IMs
TPP11	2.1972E-43	0.32925804	0.988	0.964	3.3773E-39	IMs
CTNND11	2.3505E-43	0.58874518	0.908	0.889	3.613E-39	IMs
CDA	2.838E-43	2.88062914	0.068	0.014	4.3623E-39	IMs
BANK1	3.243E-43	2.36005727	0.139	0.047	4.9848E-39	IMs
LCP2	6.638E-43	0.66264444	0.857	0.84	1.0203E-38	IMs
JUNB1	9.3984E-43	0.53306288	0.985	0.964	1.4446E-38	IMs
BLNK	1.194E-42	1.64491506	0.312	0.167	1.8352E-38	IMs
WHRN	1.2196E-42	1.68003025	0.04	0.006	1.8746E-38	IMs
CTSH1	1.5321E-42	0.41197275	0.996	0.967	2.3549E-38	IMs
GADD45B	1.6871E-42	1.88280162	0.301	0.159	2.5933E-38	IMs
CCL22	2E-42	3.26718028	0.062	0.012	3.0742E-38	IMs
ASTL	2.2326E-42	2.61543165	0.03	0.004	3.4317E-38	IMs
IFNGR2	2.2976E-42	0.61865193	0.9	0.889	3.5317E-38	IMs

L1CAM	2.2986E-42	2.15974864	0.022	0.002	3.5332E-38	IMs
HSP90AB1	2.5903E-42	0.48968053	0.913	0.896	3.9816E-38	IMs
KIAA0040	2.5999E-42	1.26455646	0.328	0.18	3.9963E-38	IMs
LMNB1	2.7147E-42	1.31525128	0.406	0.248	4.1727E-38	IMs
ASCL2	2.9545E-42	2.36389723	0.126	0.04	4.5413E-38	IMs
AVIL	3.0476E-42	2.52956419	0.068	0.015	4.6845E-38	IMs
TNFRSF8	3.4001E-42	3.67588402	0.021	0.002	5.2263E-38	IMs
MAX	7.6687E-42	0.61958479	0.794	0.77	1.1788E-37	IMs
MYO1E	9.4368E-42	1.09023103	0.611	0.506	1.4505E-37	IMs
CD180	1.4023E-41	2.05930317	0.201	0.086	2.1554E-37	IMs
QKI	1.4947E-41	0.4097718	0.949	0.941	2.2974E-37	IMs
GPSM1	1.9894E-41	2.26868611	0.028	0.003	3.0579E-37	IMs
ABI3	3.7373E-41	1.38527024	0.384	0.232	5.7446E-37	IMs
ABLIM3	4.3577E-41	3.7552533	0.018	0.001	6.6982E-37	IMs
DGKA	4.591E-41	0.95414799	0.34	0.19	7.0569E-37	IMs
BTBD19	5.1811E-41	1.97046124	0.159	0.06	7.9638E-37	IMs
PICALM	9.0269E-41	0.48371678	0.962	0.956	1.3875E-36	IMs
RNF125	2.1589E-40	1.13753009	0.28	0.143	3.3185E-36	IMs
RIN2	2.4507E-40	0.83972999	0.719	0.684	3.767E-36	IMs
ADAM9	3.3317E-40	0.97821671	0.629	0.551	5.1211E-36	IMs
PDXK1	3.5143E-40	0.4754409	0.923	0.923	5.4018E-36	IMs
AP1B1	3.9602E-40	0.80189531	0.8	0.797	6.0873E-36	IMs
DMXL21	5.7312E-40	0.71911069	0.809	0.798	8.8095E-36	IMs
FRMD4B	6.7822E-40	1.21250032	0.413	0.27	1.0425E-35	IMs
DIPK2A	8.7067E-40	1.15449617	0.546	0.422	1.3383E-35	IMs
KYNU1	1.7095E-39	0.85380571	0.781	0.732	2.6276E-35	IMs
IRAK1	1.732E-39	0.7651397	0.774	0.72	2.6622E-35	IMs
FHL3	1.8887E-39	1.45641823	0.378	0.229	2.9031E-35	IMs
CIART	1.9186E-39	3.86604142	0.017	0.001	2.949E-35	IMs
LPGAT1	3.0337E-39	0.92291886	0.558	0.426	4.6631E-35	IMs
CELF2	3.4535E-39	0.4586013	0.872	0.861	5.3084E-35	IMs
TNFRSF9	5.3634E-39	2.62538881	0.025	0.003	8.2441E-35	IMs
IL1RN1	5.7814E-39	0.1968193	0.436	0.699	8.8867E-35	IMs
LIMD1	5.8328E-39	1.04461563	0.57	0.448	8.9656E-35	IMs
N4BP1	8.0774E-39	0.77793831	0.772	0.713	1.2416E-34	IMs
SMURF2	8.0856E-39	1.09989877	0.474	0.33	1.2428E-34	IMs
GNAQ	8.8627E-39	0.59774118	0.893	0.876	1.3623E-34	IMs
DOCK10	1.08E-38	0.7225121	0.75	0.706	1.66E-34	IMs
NEK3	1.5936E-38	2.37382218	0.083	0.022	2.4495E-34	IMs
AZIN2	1.6773E-38	2.4076178	0.044	0.007	2.5782E-34	IMs
PLEC	1.8828E-38	0.54722142	0.878	0.876	2.894E-34	IMs
NAGK	1.9875E-38	0.53526737	0.893	0.893	3.0551E-34	IMs
CHKA	2.05E-38	1.87949619	0.201	0.088	3.1511E-34	IMs
IDH2	2.2158E-38	0.75392879	0.661	0.58	3.4059E-34	IMs
ISG20	2.4736E-38	0.82204525	0.137	0.048	3.8021E-34	IMs
SPATS2	3.4318E-38	1.77214401	0.161	0.062	5.2751E-34	IMs
C9orf72	4.5744E-38	0.71952132	0.702	0.617	7.0313E-34	IMs
CLK1	5.2773E-38	0.75400463	0.614	0.489	8.1118E-34	IMs
BMF	1.0202E-37	1.56499652	0.359	0.217	1.5681E-33	IMs
FAM118A	2.1511E-37	1.50507628	0.078	0.02	3.3065E-33	IMs
ABCC4	2.1766E-37	1.57319755	0.247	0.122	3.3456E-33	IMs
DUSP2	2.541E-37	2.11382105	0.166	0.067	3.9057E-33	IMs

INPP5D	2.9296E-37	1.01309687	0.524	0.39	4.5031E-33	IMs
TFPI	3.3747E-37	2.9454601	0.035	0.005	5.1873E-33	IMs
PARP14	3.4189E-37	0.76934036	0.771	0.718	5.2553E-33	IMs
TBKBP1	8.1384E-37	1.71319007	0.113	0.037	1.251E-32	IMs
RELL1	9.2885E-37	1.20229898	0.458	0.324	1.4277E-32	IMs
SPRED1	1.0478E-36	1.18214594	0.594	0.512	1.6105E-32	IMs
SERPINE1	1.4729E-36	2.62997304	0.136	0.049	2.264E-32	IMs
STEAP4	1.5862E-36	2.2169825	0.024	0.003	2.4382E-32	IMs
GIMAP5	2.0028E-36	1.20884469	0.244	0.121	3.0786E-32	IMs
PACSIN2	2.5457E-36	0.82055543	0.724	0.672	3.913E-32	IMs
PPP2R2B	3.2555E-36	1.62911904	0.03	0.004	5.004E-32	IMs
ADM2	5.0354E-36	2.99395005	0.019	0.002	7.7399E-32	IMs
PLXND11	5.226E-36	0.52743775	0.87	0.874	8.0329E-32	IMs
MYO18A	6.242E-36	1.25525427	0.415	0.28	9.5946E-32	IMs
UAP1L1	6.6509E-36	1.26778799	0.428	0.291	1.0223E-31	IMs
MCL1	7.299E-36	0.35134202	0.998	0.987	1.1219E-31	IMs
PXDC1	7.6705E-36	1.40621981	0.448	0.323	1.179E-31	IMs
ZFP36L2	8.0462E-36	0.4955279	0.923	0.945	1.2368E-31	IMs
CARD19	1.4734E-35	1.29429731	0.361	0.223	2.2648E-31	IMs
CYTH4	1.6973E-35	0.86999348	0.642	0.56	2.6089E-31	IMs
MMP17	1.9044E-35	2.78581179	0.051	0.01	2.9273E-31	IMs
PRELID3A	2.102E-35	2.59795083	0.077	0.021	3.2309E-31	IMs
RBP1	2.9704E-35	2.77260128	0.089	0.026	4.5658E-31	IMs
DUSP4	3.2482E-35	1.14834752	0.051	0.01	4.9928E-31	IMs
FXYD5	3.4297E-35	0.37114474	0.971	0.954	5.2718E-31	IMs
ZNRF1	4.926E-35	1.35199512	0.075	0.02	7.5717E-31	IMs
ARHGAP4	4.9379E-35	0.66837404	0.705	0.643	7.5901E-31	IMs
NRARP	5.9292E-35	3.3796778	0.065	0.015	9.1138E-31	IMs
GPAT3	6.8633E-35	2.46787398	0.173	0.073	1.055E-30	IMs
PIM3	9.6994E-35	1.35747946	0.497	0.378	1.4909E-30	IMs
TRIM58	1.6593E-34	3.37704208	0.041	0.007	2.5505E-30	IMs
RAB32	1.8361E-34	0.8872034	0.608	0.515	2.8223E-30	IMs
KCNMA11	2.0018E-34	1.44708474	0.405	0.273	3.077E-30	IMs
ETV6	2.2133E-34	0.75524887	0.751	0.706	3.402E-30	IMs
TTYH2	3.1938E-34	2.23664189	0.162	0.068	4.9092E-30	IMs
SYT11	3.5985E-34	1.37041794	0.393	0.263	5.5313E-30	IMs
OLFM2	3.9006E-34	2.72542691	0.01	0.001	5.9955E-30	IMs
PCSK6	6.8481E-34	3.58704968	0.02	0.002	1.0526E-29	IMs
IFNAR2	7.2249E-34	0.86969595	0.592	0.493	1.1105E-29	IMs
VCAM1	7.3393E-34	3.19215829	0.021	0.002	1.1281E-29	IMs
MAF	2.1666E-33	1.45643179	0.654	0.66	3.3303E-29	IMs
SMAP2	3.9569E-33	0.65439002	0.772	0.76	6.0821E-29	IMs
SLC20A1	5.0583E-33	1.03971404	0.544	0.433	7.7751E-29	IMs
TDRD1	7.4433E-33	4.57748434	0.016	0.001	1.1441E-28	IMs
TACC1	8.5865E-33	0.50813906	0.828	0.834	1.3198E-28	IMs
RHBDF2	8.9795E-33	1.28325896	0.467	0.345	1.3802E-28	IMs
FERMT2	1.6834E-32	3.69309629	0.017	0.001	2.5876E-28	IMs
TBC1D1	1.7778E-32	1.52303258	0.153	0.063	2.7326E-28	IMs
ATP2A3	2.0961E-32	0.62971245	0.142	0.055	3.222E-28	IMs
TGIF2	3.0656E-32	1.63349157	0.173	0.075	4.7121E-28	IMs
MAP3K6	4.2367E-32	1.66006785	0.212	0.103	6.5122E-28	IMs
MYO15B	4.5463E-32	1.14026011	0.368	0.233	6.988E-28	IMs

NDUFA4L2	8.7133E-32	3.21370731	0.027	0.004	1.3393E-27	IMs
SLC11A2	1.7105E-31	0.71570265	0.676	0.612	2.6293E-27	IMs
ARF6	1.8861E-31	0.4809453	0.883	0.889	2.8991E-27	IMs
IQCD	2.102E-31	2.96004198	0.017	0.002	3.231E-27	IMs
FAM71A	2.3253E-31	4.67033652	0.01	0.001	3.5742E-27	IMs
TAP2	3.2061E-31	0.7139287	0.734	0.721	4.928E-27	IMs
RFLNB	4.5466E-31	0.38857851	0.063	0.016	6.9886E-27	IMs
PLCL1	5.9575E-31	1.83187781	0.135	0.053	9.1572E-27	IMs
DEPDC7	1.0117E-30	2.97866962	0.044	0.009	1.5551E-26	IMs
DAAM1	1.0524E-30	1.75489228	0.262	0.148	1.6176E-26	IMs
ENO2	1.0799E-30	0.18928965	0.044	0.009	1.66E-26	IMs
TNIP1	1.0864E-30	1.02956434	0.574	0.491	1.6699E-26	IMs
ARAP3	1.1718E-30	2.83110351	0.039	0.007	1.8011E-26	IMs
BATF	1.2099E-30	1.29351321	0.12	0.044	1.8597E-26	IMs
MPO	1.2302E-30	3.55547631	0.021	0.002	1.8909E-26	IMs
PHC2	1.3962E-30	0.74099648	0.681	0.608	2.1461E-26	IMs
TIAF1	1.6618E-30	2.29596401	0.055	0.013	2.5543E-26	IMs
ATP13A3	1.815E-30	1.21247716	0.492	0.391	2.7898E-26	IMs
YPEL3	2.1086E-30	1.01542401	0.361	0.229	3.2412E-26	IMs
FZD1	2.1446E-30	2.25660019	0.146	0.061	3.2964E-26	IMs
NFKB1	2.6893E-30	0.99318416	0.631	0.568	4.1337E-26	IMs
SGTB	4.4613E-30	1.35803307	0.328	0.206	6.8575E-26	IMs
THBS3	4.6358E-30	1.42270284	0.232	0.12	7.1257E-26	IMs
SLC44A1	5.2452E-30	1.28538305	0.402	0.28	8.0624E-26	IMs
ARHGAP26	5.5274E-30	0.98074995	0.516	0.407	8.4961E-26	IMs
CNIH3	5.6553E-30	3.33984206	0.022	0.003	8.6927E-26	IMs
ANPEP	5.7024E-30	0.69615068	0.771	0.696	8.7651E-26	IMs
MAP3K2	5.7944E-30	0.77191043	0.663	0.602	8.9065E-26	IMs
PDLIM4	6.4872E-30	2.80603454	0.036	0.006	9.9715E-26	IMs
RNASE2	7.7591E-30	2.24000628	0.081	0.024	1.1927E-25	IMs
ENO1	9.6073E-30	0.43855878	0.892	0.887	1.4767E-25	IMs
SCARB11	1.0873E-29	1.10686539	0.492	0.384	1.6713E-25	IMs
MX2	1.321E-29	1.19465945	0.568	0.48	2.0305E-25	IMs
TNFSF13B	1.377E-29	0.97862431	0.567	0.479	2.1166E-25	IMs
NCOA3	1.415E-29	0.88906121	0.515	0.404	2.1751E-25	IMs
SLC43A21	1.4414E-29	0.55194436	0.85	0.847	2.2156E-25	IMs
NABP1	1.5195E-29	0.67588091	0.703	0.649	2.3356E-25	IMs
SMOX	1.7766E-29	2.66416138	0.068	0.019	2.7308E-25	IMs
CMTM4	2.012E-29	2.49863325	0.024	0.003	3.0926E-25	IMs
SAT11	2.4277E-29	0.57096817	0.972	0.961	3.7316E-25	IMs
HECTD2	2.4437E-29	1.69123328	0.14	0.058	3.7562E-25	IMs
CHD2	2.6346E-29	0.62930518	0.646	0.555	4.0497E-25	IMs
RELT	2.8154E-29	0.74037078	0.708	0.676	4.3276E-25	IMs
GCH1	3.6882E-29	1.86591475	0.149	0.063	5.6692E-25	IMs
TRIM7	4.3936E-29	3.06419793	0.026	0.004	6.7534E-25	IMs
FILIP1L	5.493E-29	1.46959613	0.365	0.244	8.4432E-25	IMs
TRAF4	5.5088E-29	1.55452131	0.079	0.024	8.4675E-25	IMs
GUCY1A1	6.2313E-29	3.37070855	0.01	0.001	9.5781E-25	IMs
PLSCR1	6.4845E-29	0.56134059	0.885	0.87	9.9673E-25	IMs
C31	8.2194E-29	1.67337315	0.242	0.128	1.2634E-24	IMs
SLC8B1	8.2824E-29	1.09975949	0.425	0.306	1.2731E-24	IMs
NFE2L1	8.3817E-29	0.58849694	0.797	0.804	1.2883E-24	IMs

EZR	9.4197E-29	0.67924413	0.832	0.83	1.4479E-24	IMs
NAAA	1.2318E-28	0.84394744	0.631	0.574	1.8934E-24	IMs
TES	1.2604E-28	1.00370908	0.503	0.39	1.9373E-24	IMs
PPP1R9B	1.5585E-28	0.39816962	0.908	0.892	2.3956E-24	IMs
SIPA1	1.9132E-28	0.56158744	0.737	0.699	2.9408E-24	IMs
UBC	2.0275E-28	0.59348295	0.754	0.722	3.1165E-24	IMs
PAQR7	2.8513E-28	2.57485268	0.05	0.012	4.3827E-24	IMs
DISC1	3.0073E-28	1.18249432	0.357	0.234	4.6226E-24	IMs
CXorf21	3.0101E-28	1.20396337	0.474	0.366	4.6268E-24	IMs
CYBB	3.5916E-28	0.3869032	0.979	0.959	5.5207E-24	IMs
LRRK1	3.9311E-28	1.0415332	0.499	0.393	6.0425E-24	IMs
SLC7A1	4.0645E-28	1.381906	0.259	0.148	6.2475E-24	IMs
MEIKIN	4.1472E-28	3.43501338	0.021	0.003	6.3746E-24	IMs
TMEM43	4.6244E-28	0.72204753	0.65	0.588	7.1081E-24	IMs
RNF1301	4.8677E-28	0.36700779	0.94	0.932	7.4822E-24	IMs
TLR1	5.3973E-28	1.04212356	0.507	0.412	8.2961E-24	IMs
NFKBID	6.7849E-28	1.32364736	0.368	0.248	1.0429E-23	IMs
AP2A2	7.1974E-28	1.12277947	0.611	0.581	1.1063E-23	IMs
OSBPL7	1.3399E-27	1.43728747	0.03	0.005	2.0595E-23	IMs
NLRC5	1.357E-27	0.83012211	0.387	0.267	2.0858E-23	IMs
CDC42EP1	1.6017E-27	1.58347127	0.199	0.1	2.462E-23	IMs
RASA3	1.6505E-27	1.11592788	0.371	0.254	2.537E-23	IMs
LRMP	1.916E-27	1.42773991	0.235	0.128	2.9451E-23	IMs
P2RY13	2.2038E-27	1.23505624	0.415	0.294	3.3874E-23	IMs
JAK2	2.2361E-27	0.88230705	0.667	0.629	3.4371E-23	IMs
RAB35	2.2979E-27	0.65070399	0.661	0.612	3.5321E-23	IMs
TRAF1	2.4746E-27	1.42457701	0.116	0.045	3.8037E-23	IMs
SPTLC3	2.6726E-27	2.71895061	0.037	0.007	4.1081E-23	IMs
TSC22D3	3.7384E-27	0.74127964	0.8	0.805	5.7464E-23	IMs
FOXN3	4.1976E-27	0.89004151	0.545	0.453	6.4521E-23	IMs
RHOBTB3	4.6562E-27	1.95115413	0.107	0.04	7.1571E-23	IMs
EMILIN21	5.5538E-27	0.36231652	0.959	0.929	8.5367E-23	IMs
HSH2D	6.3346E-27	1.78786774	0.142	0.061	9.7369E-23	IMs
TMEM119	7.1042E-27	2.98518249	0.029	0.005	1.092E-22	IMs
GNAI3	7.1736E-27	0.52648199	0.776	0.761	1.1026E-22	IMs
DAGLB	7.2333E-27	1.03654521	0.492	0.384	1.1118E-22	IMs
EMILIN1	8.3475E-27	2.59727145	0.043	0.009	1.2831E-22	IMs
ADCY7	8.5006E-27	0.68259717	0.71	0.685	1.3066E-22	IMs
BCL6	1.0216E-26	1.04699901	0.53	0.448	1.5703E-22	IMs
COL24A1	1.0386E-26	3.72669029	0.013	0.001	1.5964E-22	IMs
CAPN2	1.1268E-26	0.54915541	0.864	0.848	1.732E-22	IMs
MTHFR	1.1333E-26	0.92925092	0.448	0.332	1.742E-22	IMs
IFITM2	1.1404E-26	1.06835557	0.464	0.362	1.753E-22	IMs
TRIM2	1.1695E-26	2.88028577	0.021	0.003	1.7976E-22	IMs
TNFRSF4	1.2636E-26	1.87484961	0.021	0.003	1.9423E-22	IMs
BTG1	1.4967E-26	0.52371681	0.908	0.894	2.3006E-22	IMs
TSHZ3	1.6025E-26	2.12236314	0.107	0.04	2.4632E-22	IMs
PEAK3	1.8763E-26	2.02931684	0.097	0.035	2.884E-22	IMs
CREM	2.0861E-26	0.92004202	0.6	0.524	3.2066E-22	IMs
SSBP2	2.3925E-26	1.68108637	0.103	0.038	3.6774E-22	IMs
ANXA41	2.646E-26	0.5240987	0.862	0.861	4.0671E-22	IMs
BAIAP2	2.8902E-26	1.44033478	0.296	0.182	4.4425E-22	IMs

CD36	3.1752E-26	1.50428907	0.402	0.284	4.8806E-22	IMs
RUNX3	3.6927E-26	1.11206799	0.249	0.141	5.6761E-22	IMs
OSBPL3	3.9138E-26	1.1846502	0.331	0.215	6.0159E-22	IMs
MEF2D	4.5891E-26	1.00374695	0.487	0.39	7.0538E-22	IMs
PLPPR2	4.6064E-26	1.61140288	0.15	0.067	7.0804E-22	IMs
HIST2H2BE	4.7705E-26	1.17202151	0.248	0.138	7.3327E-22	IMs
ATP6V0A11	6.9235E-26	0.82714759	0.584	0.502	1.0642E-21	IMs
MT-CO3	7.523E-26	0.84184852	0.626	0.523	1.1564E-21	IMs
PHF19	7.997E-26	0.54494801	0.275	0.157	1.2292E-21	IMs
JAK1	8.195E-26	0.44503736	0.816	0.815	1.2597E-21	IMs
SLC35E4	9.3409E-26	2.13762919	0.094	0.034	1.4358E-21	IMs
ZNF532	9.3463E-26	1.56760903	0.223	0.122	1.4366E-21	IMs
EDEM1	1.2861E-25	0.61093492	0.821	0.81	1.9769E-21	IMs
LAMB3	1.2889E-25	2.68753277	0.028	0.005	1.9811E-21	IMs
SMIM29	1.5287E-25	1.22079837	0.335	0.222	2.3497E-21	IMs
NCR3LG1	1.9562E-25	1.85562066	0.155	0.072	3.0069E-21	IMs
IGF2BP2	2.5674E-25	2.01395999	0.081	0.027	3.9464E-21	IMs
CLEC9A	2.734E-25	1.96962676	0.017	0.002	4.2025E-21	IMs
NOTCH2	3.542E-25	0.8288114	0.553	0.475	5.4444E-21	IMs
YWHAH	3.7538E-25	0.66691714	0.844	0.841	5.7699E-21	IMs
DIRAS1	4.6814E-25	4.24561267	0.013	0.001	7.1958E-21	IMs
PFKFB3	4.9696E-25	1.27722	0.513	0.429	7.6388E-21	IMs
ZSWIM4	6.7494E-25	2.04617976	0.094	0.034	1.0374E-20	IMs
SERINC5	7.8572E-25	0.87341434	0.614	0.548	1.2077E-20	IMs
DPYSL2	9.2621E-25	0.36219325	0.967	0.954	1.4237E-20	IMs
IKBKB	9.8109E-25	0.52970664	0.731	0.682	1.508E-20	IMs
VASH11	1.0127E-24	0.83724812	0.801	0.821	1.5567E-20	IMs
PHF20L1	1.0184E-24	0.66302891	0.56	0.47	1.5653E-20	IMs
LIMCH1	1.06E-24	2.61307991	0.013	0.001	1.6293E-20	IMs
MILR1	1.2175E-24	0.97711009	0.486	0.385	1.8715E-20	IMs
ITGA5	1.2591E-24	0.5091615	0.885	0.897	1.9353E-20	IMs
P2RY2	1.2662E-24	3.013256	0.036	0.007	1.9463E-20	IMs
CLN81	1.3415E-24	0.79418578	0.575	0.495	2.0619E-20	IMs
SEMA6B	1.5445E-24	1.81261442	0.302	0.196	2.374E-20	IMs
SLC39A10	1.5782E-24	0.6846012	0.687	0.656	2.4259E-20	IMs
IL27RA	1.7769E-24	0.95603085	0.567	0.492	2.7313E-20	IMs
KIAA0930	1.9488E-24	0.64280287	0.759	0.754	2.9955E-20	IMs
NFKBIE1	1.9701E-24	1.02430696	0.524	0.432	3.0282E-20	IMs
CNRIP1	2.2663E-24	1.62711837	0.192	0.1	3.4835E-20	IMs
PARVB	2.4968E-24	0.79272558	0.624	0.589	3.8379E-20	IMs
PDP1	2.5011E-24	1.19277301	0.338	0.228	3.8444E-20	IMs
MOB1B	2.5151E-24	1.06026125	0.439	0.339	3.866E-20	IMs
STK26	3.1628E-24	1.44827979	0.106	0.041	4.8615E-20	IMs
KEL	3.3179E-24	3.71920049	0.019	0.002	5.1E-20	IMs
PLAU1	3.8932E-24	1.0010861	0.733	0.734	5.9842E-20	IMs
XAF1	4.716E-24	0.67641563	0.707	0.679	7.249E-20	IMs
NFKBIZ	5.6048E-24	0.78467003	0.61	0.538	8.6151E-20	IMs
RIPOR2	5.8141E-24	1.02497428	0.158	0.075	8.9369E-20	IMs
TAB2	6.1342E-24	0.47952885	0.646	0.606	9.4289E-20	IMs
NFE2L3	6.3998E-24	1.4680956	0.212	0.116	9.8371E-20	IMs
RAB39A	6.5327E-24	1.19786978	0.334	0.224	1.0041E-19	IMs
TPM4	7.7042E-24	0.4299912	0.831	0.823	1.1842E-19	IMs

TOX	8.0574E-24	0.16333084	0.023	0.003	1.2385E-19	IMs
DDR1	8.1417E-24	1.91746039	0.065	0.02	1.2515E-19	IMs
TRPV6	8.4663E-24	4.16919031	0.01	0.001	1.3014E-19	IMs
CTDSP2	1.0296E-23	0.53116734	0.764	0.747	1.5826E-19	IMs
IL13RA1	1.067E-23	0.56628782	0.814	0.828	1.64E-19	IMs
MT-ND6	1.1734E-23	1.18773623	0.256	0.152	1.8037E-19	IMs
EPS8	1.2772E-23	1.83423137	0.118	0.049	1.9631E-19	IMs
MYC	1.3273E-23	1.19067131	0.24	0.138	2.0401E-19	IMs
PON2	1.4697E-23	1.31107839	0.368	0.265	2.259E-19	IMs
LAMB2	1.8709E-23	1.38569585	0.291	0.186	2.8758E-19	IMs
ACP21	2.0462E-23	0.86295042	0.651	0.652	3.1452E-19	IMs
SH3PXD2B1	2.2057E-23	1.21971709	0.316	0.207	3.3904E-19	IMs
FHOD1	2.3053E-23	0.98782139	0.416	0.313	3.5435E-19	IMs
WDFY2	2.3587E-23	0.79765872	0.499	0.393	3.6255E-19	IMs
BCL2L11	2.8367E-23	1.21332709	0.387	0.286	4.3603E-19	IMs
STARD3	2.9199E-23	0.62580938	0.622	0.56	4.4882E-19	IMs
VPS13C	3.3511E-23	0.46960912	0.787	0.778	5.151E-19	IMs
FCHSD2	3.4678E-23	1.04321318	0.49	0.409	5.3303E-19	IMs
CXCL1	3.8894E-23	2.44713242	0.058	0.017	5.9784E-19	IMs
SLC30A4	3.8951E-23	1.96664439	0.079	0.027	5.9871E-19	IMs
MT-ATP6	3.9514E-23	0.82841975	0.556	0.459	6.0737E-19	IMs
MAPK11	4.5437E-23	1.30902444	0.028	0.005	6.9841E-19	IMs
SYCP3	4.6059E-23	2.57479531	0.025	0.004	7.0797E-19	IMs
LMNA	5.0569E-23	0.72786487	0.944	0.965	7.7729E-19	IMs
TRIM72	5.237E-23	4.33835622	0.013	0.001	8.0499E-19	IMs
RCN3	5.2629E-23	1.09822058	0.305	0.197	8.0897E-19	IMs
RSAD2	6.3231E-23	1.75560176	0.313	0.205	9.7193E-19	IMs
UBR4	6.8721E-23	0.49975797	0.744	0.716	1.0563E-18	IMs
ATP6V1B21	8.0268E-23	0.37492277	0.938	0.924	1.2338E-18	IMs
GLMP	8.3202E-23	0.99057492	0.496	0.424	1.2789E-18	IMs
ANKRD10	8.6063E-23	0.63235289	0.717	0.682	1.3229E-18	IMs
FNBP1L	8.6836E-23	2.44755462	0.032	0.007	1.3348E-18	IMs
PPM1F	1.0067E-22	1.10043114	0.446	0.358	1.5474E-18	IMs
IRF7	1.0587E-22	0.75911288	0.725	0.701	1.6274E-18	IMs
IFI44L	1.1359E-22	1.00132021	0.541	0.461	1.746E-18	IMs
RASGEF1B1	1.3193E-22	0.7515113	0.681	0.648	2.0279E-18	IMs
NDP	1.7942E-22	3.87562437	0.013	0.001	2.7579E-18	IMs
CCDC154	2.0721E-22	2.30109867	0.055	0.016	3.185E-18	IMs
NPL1	2.1933E-22	0.91057747	0.61	0.613	3.3714E-18	IMs
POU6F1	2.9997E-22	1.36192704	0.019	0.003	4.6109E-18	IMs
MLLT6	3.0039E-22	0.51342316	0.539	0.442	4.6173E-18	IMs
NR4A2	3.069E-22	0.97594334	0.571	0.487	4.7174E-18	IMs
EVI2A	3.0816E-22	0.62722145	0.687	0.652	4.7367E-18	IMs
CD244	3.2174E-22	1.68849966	0.041	0.01	4.9454E-18	IMs
TNKS1BP1	3.4239E-22	2.22243744	0.065	0.02	5.2629E-18	IMs
LRP3	3.7482E-22	1.7314249	0.118	0.051	5.7613E-18	IMs
MEX3C	3.9181E-22	1.12714138	0.195	0.105	6.0225E-18	IMs
ITGA7	4.3295E-22	3.52099898	0.02	0.003	6.6549E-18	IMs
NDFIP1	4.7199E-22	0.40540161	0.818	0.844	7.255E-18	IMs
KLHDC7B	5.4744E-22	1.50110785	0.153	0.074	8.4147E-18	IMs
OSBPL6	5.6283E-22	3.06219771	0.016	0.002	8.6513E-18	IMs
PNKD	8.0196E-22	1.29381625	0.405	0.317	1.2327E-17	IMs

TNFSF14	9.6567E-22	1.24755448	0.062	0.019	1.4843E-17	IMs
ST3GAL4	1.1032E-21	1.52033353	0.159	0.08	1.6958E-17	IMs
PSAT1	1.5762E-21	2.38683016	0.062	0.019	2.4228E-17	IMs
SGPP2	1.7254E-21	2.0024455	0.015	0.002	2.6521E-17	IMs
ZMIZ2	2.225E-21	0.73698123	0.621	0.581	3.42E-17	IMs
RAI14	2.3794E-21	3.76406683	0.015	0.002	3.6573E-17	IMs
ESYT2	2.4497E-21	0.44733111	0.727	0.716	3.7655E-17	IMs
NFAM1	2.5406E-21	0.86481299	0.486	0.389	3.9051E-17	IMs
ABL2	2.9406E-21	1.29462181	0.39	0.297	4.52E-17	IMs
ELOVL1	3.2281E-21	0.43122803	0.833	0.836	4.9619E-17	IMs
MPEG1	3.3357E-21	0.65870219	0.751	0.726	5.1273E-17	IMs
IL15RA	3.8879E-21	1.30172321	0.192	0.105	5.976E-17	IMs
KCNK6	4.5839E-21	1.01828841	0.46	0.372	7.0459E-17	IMs
TMEM154	4.817E-21	1.27408129	0.09	0.035	7.4043E-17	IMs
HAGHL	4.9515E-21	1.22867561	0.258	0.16	7.611E-17	IMs
KDM2B	5.0978E-21	0.9221712	0.484	0.4	7.8358E-17	IMs
PIK3CD	5.9125E-21	0.44309005	0.567	0.492	9.088E-17	IMs
MAGEF1	7.8618E-21	1.48475885	0.161	0.082	1.2084E-16	IMs
TMEM109	8.4139E-21	0.55483002	0.759	0.755	1.2933E-16	IMs
CSGALNACT1	8.5132E-21	1.16461241	0.023	0.004	1.3086E-16	IMs
S100A10	8.9071E-21	0.37570565	0.994	0.982	1.3691E-16	IMs
PDE4B	9.1425E-21	0.73070355	0.548	0.458	1.4053E-16	IMs
DUSP11	9.7463E-21	0.39669411	0.983	0.973	1.4981E-16	IMs
GPATCH2L	1.0315E-20	0.56001522	0.68	0.633	1.5855E-16	IMs
ZNF6971	1.1436E-20	1.71694505	0.144	0.07	1.7578E-16	IMs
VCL	1.376E-20	1.2095074	0.266	0.168	2.115E-16	IMs
WDR811	1.6025E-20	0.60990602	0.665	0.632	2.4632E-16	IMs
ARL4D	1.863E-20	2.46027425	0.037	0.009	2.8636E-16	IMs
OXCT1	1.876E-20	1.36766407	0.054	0.016	2.8835E-16	IMs
OAZ2	1.8869E-20	0.74833998	0.576	0.532	2.9003E-16	IMs
UTRN	1.9572E-20	0.66853462	0.482	0.392	3.0084E-16	IMs
USP32	2.8733E-20	0.6052673	0.675	0.65	4.4165E-16	IMs
IFIH1	3.4945E-20	0.69506317	0.674	0.66	5.3714E-16	IMs
NAB2	3.5756E-20	0.96691367	0.468	0.379	5.4961E-16	IMs
GBA	3.6436E-20	0.51882799	0.734	0.749	5.6005E-16	IMs
GIMAP6	3.7208E-20	1.06662757	0.294	0.196	5.7193E-16	IMs
DUSP6	6.1418E-20	1.15103871	0.379	0.283	9.4406E-16	IMs
S100A12	6.2231E-20	4.31620821	0.012	0.001	9.5655E-16	IMs
LUC7L3	7.8291E-20	0.48748578	0.576	0.504	1.2034E-15	IMs
TET3	8.2602E-20	0.97277794	0.405	0.311	1.2697E-15	IMs
ETS2	8.4624E-20	0.85230838	0.7	0.709	1.3008E-15	IMs
SLC1A31	9.0065E-20	1.25062987	0.378	0.285	1.3844E-15	IMs
PTTG1IP	9.0666E-20	0.31409107	0.917	0.924	1.3936E-15	IMs
CACNA2D4	9.3066E-20	1.61677711	0.16	0.084	1.4305E-15	IMs
ZNF710	9.948E-20	1.31813025	0.251	0.158	1.5291E-15	IMs
AKNA	1.0314E-19	0.49304057	0.601	0.552	1.5854E-15	IMs
GRB10	1.1598E-19	1.79602334	0.109	0.048	1.7828E-15	IMs
SH3BP2	1.3645E-19	0.52114498	0.688	0.649	2.0974E-15	IMs
SLC41A2	1.3743E-19	1.1722553	0.408	0.322	2.1125E-15	IMs
ARHGAP12	1.4086E-19	1.1780322	0.146	0.073	2.1651E-15	IMs
PLEKHA5	1.443E-19	1.31834715	0.081	0.03	2.2181E-15	IMs
TUBGCP2	1.5664E-19	0.65094107	0.636	0.599	2.4078E-15	IMs

TLR10	1.6197E-19	2.39191809	0.023	0.004	2.4897E-15	IMs
EIF4A1	1.7802E-19	0.30363793	0.929	0.93	2.7363E-15	IMs
CHST10	1.8364E-19	3.27151217	0.017	0.002	2.8228E-15	IMs
PCDH12	1.8394E-19	3.70301498	0.015	0.002	2.8273E-15	IMs
PITPNA	1.9378E-19	0.41768138	0.81	0.81	2.9786E-15	IMs
SLC2A13	2.1565E-19	1.39080666	0.196	0.111	3.3147E-15	IMs
ARRDC5	2.198E-19	1.76504707	0.073	0.027	3.3785E-15	IMs
APBB1IP	2.9387E-19	0.42696879	0.781	0.772	4.5171E-15	IMs
GIMAP8	2.9939E-19	1.20967674	0.314	0.217	4.6019E-15	IMs
TWF21	3.1119E-19	0.32007397	0.936	0.933	4.7833E-15	IMs
SDC31	3.2206E-19	1.41782427	0.435	0.36	4.9504E-15	IMs
PLP2	4.7399E-19	0.63281732	0.619	0.57	7.2856E-15	IMs
RHOG	4.8021E-19	0.31212407	0.911	0.912	7.3813E-15	IMs
NDRG1	5.4319E-19	0.82133641	0.464	0.375	8.3494E-15	IMs
LRCH4	6.1027E-19	0.48652156	0.686	0.648	9.3804E-15	IMs
NCOR2	6.879E-19	0.38095613	0.904	0.903	1.0574E-14	IMs
CLECL1	8.8036E-19	2.00200407	0.088	0.036	1.3532E-14	IMs
CCN3	1.0272E-18	3.20296962	0.017	0.002	1.5789E-14	IMs
AGAP3	1.4185E-18	0.75747266	0.558	0.499	2.1804E-14	IMs
PIWIL4	1.4539E-18	1.75183101	0.117	0.054	2.2348E-14	IMs
IGF1R	1.5105E-18	1.36811014	0.16	0.085	2.3218E-14	IMs
PLEKHO21	1.5791E-18	0.4208829	0.838	0.864	2.4273E-14	IMs
PPP1R18	1.5903E-18	0.35359236	0.86	0.883	2.4445E-14	IMs
ARHGAP18	1.6052E-18	0.54975598	0.836	0.874	2.4673E-14	IMs
KCNJ2	1.874E-18	2.25159981	0.052	0.016	2.8806E-14	IMs
SCAMP2	1.8884E-18	0.28468654	0.913	0.919	2.9027E-14	IMs
KMT2E	2.0221E-18	0.46918835	0.678	0.653	3.1082E-14	IMs
B4GALT1	2.2008E-18	0.35854307	0.829	0.848	3.3829E-14	IMs
MACF1	2.8472E-18	0.5140838	0.604	0.566	4.3764E-14	IMs
ATAD2B	2.9175E-18	1.03492544	0.346	0.253	4.4845E-14	IMs
NUDT3	3.1952E-18	0.4899357	0.738	0.727	4.9114E-14	IMs
DUSP7	3.4765E-18	1.01910739	0.465	0.396	5.3437E-14	IMs
PTPRJ	3.5518E-18	0.71883863	0.571	0.524	5.4595E-14	IMs
GNG7	3.6065E-18	2.86700159	0.022	0.004	5.5436E-14	IMs
CIAO2A	3.7091E-18	0.5735808	0.68	0.678	5.7013E-14	IMs
AFF1	5.2597E-18	0.53546669	0.7	0.695	8.0848E-14	IMs
ANTXR1	5.6623E-18	3.43342429	0.013	0.001	8.7036E-14	IMs
OTUD1	6.2831E-18	0.96673043	0.642	0.608	9.6578E-14	IMs
SERPINB8	6.7371E-18	0.76324346	0.543	0.497	1.0356E-13	IMs
WNT2B	7.0515E-18	2.21534475	0.043	0.012	1.0839E-13	IMs
PHLDB1	7.0986E-18	1.9325174	0.079	0.031	1.0911E-13	IMs
CHIC1	7.6185E-18	1.19527969	0.04	0.011	1.171E-13	IMs
MYO1F	7.7423E-18	0.32592507	0.872	0.898	1.1901E-13	IMs
PPP1R15B	8.4029E-18	0.45761003	0.809	0.825	1.2916E-13	IMs
SFXN3	8.418E-18	0.4871776	0.706	0.697	1.2939E-13	IMs
TNS31	8.9768E-18	0.58928182	0.755	0.796	1.3798E-13	IMs
ITPR2	8.9873E-18	0.65195139	0.684	0.672	1.3814E-13	IMs
TIMD4	9.2613E-18	1.91722356	0.073	0.028	1.4236E-13	IMs
NFIC	9.3011E-18	0.51316349	0.658	0.636	1.4297E-13	IMs
SGSH	1.012E-17	0.85573929	0.359	0.264	1.5555E-13	IMs
CELSR1	1.0293E-17	1.56541665	0.053	0.017	1.5821E-13	IMs
ASPH	1.0919E-17	0.79848868	0.53	0.478	1.6783E-13	IMs

GPR162	1.3098E-17	1.61304223	0.094	0.04	2.0133E-13	IMs
P2RX71	1.3406E-17	0.95624023	0.581	0.535	2.0606E-13	IMs
TFEC1	1.5353E-17	0.44284439	0.789	0.79	2.3599E-13	IMs
RAP2A	1.5471E-17	0.66383813	0.667	0.66	2.3781E-13	IMs
EHBP1L1	1.5871E-17	0.31773551	0.958	0.95	2.4396E-13	IMs
CYB5R4	1.6502E-17	0.4744119	0.721	0.721	2.5365E-13	IMs
ZNF33A	1.8426E-17	0.93640311	0.365	0.275	2.8322E-13	IMs
DNASE1L11	1.9536E-17	0.49431796	0.706	0.686	3.0028E-13	IMs
FMNL1	2.057E-17	0.33342201	0.939	0.945	3.1619E-13	IMs
ACSM5	2.2067E-17	2.48113563	0.052	0.017	3.3919E-13	IMs
IL181	2.255E-17	0.71907334	0.616	0.594	3.4662E-13	IMs
ADA	2.5564E-17	1.13193213	0.1	0.045	3.9295E-13	IMs
LRRC75B	2.5684E-17	2.2222364	0.02	0.003	3.9479E-13	IMs
CDC42SE1	2.6001E-17	0.36583427	0.878	0.88	3.9966E-13	IMs
SGK3	2.6027E-17	0.41566073	0.768	0.759	4.0006E-13	IMs
MYL9	2.6057E-17	1.90431067	0.082	0.033	4.0053E-13	IMs
ARID3A	2.659E-17	1.23177766	0.283	0.198	4.0871E-13	IMs
HNRNPF	2.7517E-17	0.30730417	0.863	0.864	4.2296E-13	IMs
ZNF641	2.9427E-17	0.85219636	0.415	0.332	4.5232E-13	IMs
DNAJB5	3.0691E-17	2.28646528	0.096	0.043	4.7176E-13	IMs
MED13L	3.2393E-17	0.87179371	0.469	0.396	4.9791E-13	IMs
TESC	3.6619E-17	0.94201264	0.02	0.003	5.6287E-13	IMs
EPCAM	3.7203E-17	1.47446396	0.014	0.002	5.7184E-13	IMs
GALNT3	3.8958E-17	1.8482329	0.036	0.009	5.9883E-13	IMs
PDLIM7	3.9236E-17	0.70436829	0.637	0.595	6.0309E-13	IMs
SETDB2	4.4155E-17	0.81895902	0.496	0.422	6.787E-13	IMs
LPCAT21	4.861E-17	0.53681662	0.754	0.75	7.4719E-13	IMs
LUC7L	5.1804E-17	0.68800252	0.473	0.394	7.9628E-13	IMs
MPP1	5.6874E-17	0.44106706	0.795	0.843	8.7421E-13	IMs
DICER1	6.4355E-17	0.5478955	0.636	0.608	9.892E-13	IMs
RELCH	6.693E-17	0.59582249	0.492	0.413	1.0288E-12	IMs
IFI44	7.0745E-17	0.60917723	0.688	0.669	1.0874E-12	IMs
FGR1	8.0437E-17	0.54505443	0.869	0.899	1.2364E-12	IMs
C1orf21	8.8371E-17	1.91819491	0.04	0.011	1.3584E-12	IMs
MBOAT7	8.9364E-17	0.81134486	0.562	0.518	1.3736E-12	IMs
SLC25A37	9.866E-17	1.18964771	0.266	0.179	1.5165E-12	IMs
MFSD12	1.1479E-16	1.00853339	0.413	0.339	1.7645E-12	IMs
NETO2	1.1713E-16	1.32224101	0.173	0.098	1.8004E-12	IMs
MCTP1	1.3062E-16	0.75786591	0.547	0.485	2.0078E-12	IMs
TPP2	1.3868E-16	0.66358154	0.515	0.45	2.1317E-12	IMs
CHAC1	1.3926E-16	2.25698818	0.016	0.002	2.1405E-12	IMs
KDM2A	1.4412E-16	0.33314489	0.817	0.82	2.2152E-12	IMs
RNF145	1.5848E-16	0.90694885	0.397	0.316	2.436E-12	IMs
PXK	1.617E-16	0.91767167	0.476	0.406	2.4855E-12	IMs
CCNT2	1.6222E-16	0.6843089	0.455	0.38	2.4935E-12	IMs
ICA1	1.8829E-16	1.4428276	0.155	0.084	2.8942E-12	IMs
SNRNP200	1.907E-16	0.39527847	0.755	0.759	2.9312E-12	IMs
TNFRSF1A	2.056E-16	0.32982787	0.842	0.871	3.1602E-12	IMs
NBL1	2.1775E-16	0.12001623	0.024	0.005	3.347E-12	IMs
KAT6A	2.1784E-16	0.60264547	0.518	0.46	3.3485E-12	IMs
KCNJ10	2.2353E-16	3.12089107	0.016	0.002	3.4359E-12	IMs
PLEKHM21	2.2375E-16	0.44506084	0.758	0.776	3.4393E-12	IMs

ME2	2.271E-16	0.71196337	0.607	0.589	3.4907E-12	IMs
SPPL2A	2.3244E-16	0.37499756	0.815	0.829	3.5729E-12	IMs
SLC3A2	2.3739E-16	0.33759076	0.926	0.924	3.6489E-12	IMs
RTKN	2.4242E-16	2.65803654	0.023	0.005	3.7262E-12	IMs
GHRL	2.7898E-16	3.03557576	0.016	0.002	4.2881E-12	IMs
PMFBP1	2.8063E-16	1.85782612	0.063	0.023	4.3136E-12	IMs
SAMSN1	3.4488E-16	0.54163561	0.637	0.596	5.3012E-12	IMs
SLC30A11	3.4744E-16	1.05537157	0.301	0.211	5.3405E-12	IMs
NOD2	3.5868E-16	0.95215224	0.458	0.392	5.5133E-12	IMs
CYC1	3.6167E-16	0.45481988	0.755	0.763	5.5593E-12	IMs
ADM	3.8247E-16	2.64278214	0.029	0.007	5.8789E-12	IMs
CAPZB	3.8661E-16	0.28871836	0.865	0.891	5.9426E-12	IMs
CARM1	4.1108E-16	0.72290212	0.523	0.466	6.3188E-12	IMs
RAPGEF1	4.2423E-16	0.4175515	0.77	0.78	6.5208E-12	IMs
NPC1	4.5489E-16	0.6820695	0.669	0.688	6.9921E-12	IMs
STX3	5.1333E-16	0.73284408	0.471	0.395	7.8904E-12	IMs
SQOR1	6.4335E-16	0.34714868	0.819	0.829	9.8889E-12	IMs
SELL	6.7435E-16	0.98617559	0.111	0.054	1.0365E-11	IMs
AK4	7.5425E-16	2.18969288	0.069	0.027	1.1594E-11	IMs
DGKD	8.3427E-16	1.08063979	0.185	0.11	1.2824E-11	IMs
NCKAP5L1	8.5002E-16	0.68020804	0.59	0.562	1.3066E-11	IMs
CD1D	8.6614E-16	1.4033075	0.201	0.123	1.3313E-11	IMs
IRF2BP2	8.9324E-16	0.54003183	0.7	0.698	1.373E-11	IMs
VSIR	8.952E-16	1.06464895	0.401	0.324	1.376E-11	IMs
SIGLEC9	1.0247E-15	0.64216893	0.591	0.562	1.575E-11	IMs
NLN1	1.2022E-15	0.99436843	0.376	0.302	1.8479E-11	IMs
ABCB5	1.2349E-15	3.95806781	0.023	0.005	1.8981E-11	IMs
TEP1	1.2508E-15	0.77244156	0.45	0.379	1.9225E-11	IMs
SAP30	1.3506E-15	0.92228501	0.348	0.262	2.076E-11	IMs
KLF9	1.5264E-15	0.8600638	0.397	0.32	2.3462E-11	IMs
STX41	1.538E-15	0.43168225	0.832	0.841	2.364E-11	IMs
GBGT11	1.5443E-15	0.84331414	0.479	0.416	2.3737E-11	IMs
KRAS	1.6834E-15	0.76141693	0.47	0.403	2.5875E-11	IMs
SMIM3	1.7966E-15	1.71806666	0.058	0.021	2.7615E-11	IMs
CLSTN1	1.8288E-15	0.57250569	0.528	0.469	2.8111E-11	IMs
PDE7A	1.9653E-15	0.55207504	0.338	0.249	3.0208E-11	IMs
CFH	1.9805E-15	2.3936232	0.032	0.009	3.0442E-11	IMs
DNAJC7	2.1927E-15	0.44160679	0.725	0.726	3.3704E-11	IMs
NR4A11	3.102E-15	1.50860337	0.499	0.46	4.7681E-11	IMs
RNH11	3.1401E-15	0.22326011	0.973	0.961	4.8267E-11	IMs
OLFML2A	3.5841E-15	2.48683321	0.058	0.021	5.5091E-11	IMs
IL1B1	3.6022E-15	0.73033583	0.295	0.472	5.537E-11	IMs
NSMF	3.7861E-15	1.08243627	0.245	0.166	5.8196E-11	IMs
CHRFAM7A	3.9319E-15	2.56984329	0.035	0.01	6.0437E-11	IMs
PLCB1	4.0601E-15	1.15552895	0.015	0.002	6.2407E-11	IMs
PARVA	4.1536E-15	1.94620843	0.023	0.005	6.3844E-11	IMs
TNFRSF14	4.1758E-15	0.69432509	0.566	0.534	6.4186E-11	IMs
SLC9A7	4.1771E-15	1.06234961	0.3	0.218	6.4205E-11	IMs
NAV1	4.1847E-15	1.39440622	0.231	0.153	6.4324E-11	IMs
MORC3	4.5932E-15	0.38508896	0.723	0.721	7.0602E-11	IMs
DBI	4.7069E-15	0.49964894	0.704	0.706	7.235E-11	IMs
DNAJC10	5.0439E-15	0.68846803	0.515	0.455	7.753E-11	IMs

SEMA4B	5.0897E-15	1.35743132	0.177	0.106	7.8235E-11	IMs
ARGLU1	5.6604E-15	0.36107667	0.624	0.589	8.7007E-11	IMs
CYTH1	5.9942E-15	0.35989102	0.815	0.832	9.2137E-11	IMs
TMEM45B	6.8114E-15	2.72117256	0.014	0.002	1.047E-10	IMs
SIGIRR	8.0982E-15	0.6373439	0.259	0.176	1.2448E-10	IMs
ACSL4	1.0175E-14	0.5028463	0.714	0.717	1.564E-10	IMs
ZFC3H1	1.1145E-14	0.47071155	0.519	0.446	1.7132E-10	IMs
NKIRAS2	1.1279E-14	0.73646319	0.495	0.436	1.7337E-10	IMs
SLC38A5	1.2047E-14	0.89156352	0.015	0.002	1.8518E-10	IMs
FABP31	1.2059E-14	0.28861363	0.203	0.121	1.8536E-10	IMs
TCIRG1	1.2146E-14	0.3430312	0.876	0.89	1.867E-10	IMs
HIPK3	1.3051E-14	0.32641076	0.837	0.854	2.0061E-10	IMs
FNBP4	1.3297E-14	0.58316507	0.436	0.356	2.0439E-10	IMs
WASHC4	1.4705E-14	0.3761931	0.754	0.76	2.2603E-10	IMs
TLCD3A	1.4882E-14	2.4557675	0.02	0.004	2.2876E-10	IMs
DOT1L	1.538E-14	0.92770173	0.416	0.344	2.3641E-10	IMs
GRAMD1A	1.5538E-14	0.77088273	0.476	0.419	2.3884E-10	IMs
ID3	1.5669E-14	0.74647797	0.658	0.632	2.4085E-10	IMs
OLFM1	1.6006E-14	3.12822749	0.018	0.003	2.4602E-10	IMs
MTDH	1.7471E-14	0.33712607	0.829	0.865	2.6854E-10	IMs
PPP4C	1.7684E-14	0.31768425	0.798	0.82	2.7182E-10	IMs
RIC1	1.8211E-14	0.64986419	0.481	0.416	2.7992E-10	IMs
SH3TC11	2.0372E-14	0.66060067	0.545	0.501	3.1314E-10	IMs
PHYKPL	2.0718E-14	0.7016827	0.456	0.395	3.1845E-10	IMs
FCHO21	2.1176E-14	0.55771135	0.663	0.658	3.255E-10	IMs
PEAK1	2.1232E-14	0.9819419	0.297	0.216	3.2636E-10	IMs
SPINK1	2.3096E-14	3.5982254	0.014	0.002	3.5501E-10	IMs
TMEM65	2.3847E-14	0.96185385	0.22	0.143	3.6656E-10	IMs
USP51	2.5374E-14	2.30260266	0.038	0.011	3.9002E-10	IMs
VPS13B	2.6237E-14	0.51405066	0.539	0.478	4.033E-10	IMs
ALOX15B1	2.7773E-14	1.96346342	0.032	0.009	4.2691E-10	IMs
SMAD3	2.8961E-14	0.38405333	0.031	0.008	4.4516E-10	IMs
STEAP3	3.2123E-14	0.64433284	0.582	0.564	4.9376E-10	IMs
AOAH	3.46E-14	0.89218403	0.498	0.449	5.3183E-10	IMs
MT-ND1	3.5059E-14	0.71594603	0.297	0.211	5.3889E-10	IMs
WDR6	3.5133E-14	0.67970152	0.404	0.329	5.4003E-10	IMs
NYNRIN	3.6858E-14	1.62779958	0.115	0.06	5.6654E-10	IMs
PIP4P2	3.8027E-14	1.59019051	0.103	0.051	5.8452E-10	IMs
NDE1	4.3929E-14	0.6540592	0.45	0.379	6.7524E-10	IMs
ARHGEF1	4.4597E-14	0.31185588	0.715	0.718	6.855E-10	IMs
EEF2K	4.535E-14	0.91074253	0.397	0.332	6.9708E-10	IMs
FCRLB	4.789E-14	2.16842141	0.058	0.022	7.3612E-10	IMs
CD38	5.0839E-14	1.42715419	0.199	0.127	7.8145E-10	IMs
FBN2	5.1162E-14	2.20627966	0.027	0.007	7.8641E-10	IMs
IL2RA	5.346E-14	0.97175604	0.038	0.012	8.2173E-10	IMs
ANO6	5.6007E-14	0.80978342	0.441	0.37	8.6088E-10	IMs
RCVRN	6.045E-14	3.447839	0.013	0.002	9.2918E-10	IMs
GPAT4	6.3657E-14	0.706774	0.518	0.484	9.7846E-10	IMs
HDAC7	6.435E-14	0.76719278	0.305	0.226	9.8913E-10	IMs
SLC23A3	6.4691E-14	2.07626062	0.014	0.002	9.9437E-10	IMs
TET2	6.8139E-14	0.93674327	0.374	0.303	1.0474E-09	IMs
FAM13A	7.7352E-14	1.24232495	0.202	0.129	1.189E-09	IMs

CPT1A	7.7518E-14	0.69089252	0.468	0.406	1.1915E-09	IMs
GNPTAB	9.4501E-14	0.49059326	0.627	0.618	1.4526E-09	IMs
CEP170	1.0123E-13	0.5086484	0.704	0.711	1.556E-09	IMs
NREP	1.0291E-13	1.19572582	0.198	0.126	1.5819E-09	IMs
CCR7	1.1532E-13	1.96434315	0.016	0.003	1.7726E-09	IMs
INTS6L	1.2464E-13	1.12384819	0.113	0.059	1.9159E-09	IMs
PMAIP1	1.2862E-13	1.59106523	0.088	0.041	1.977E-09	IMs
PCBP1	1.2941E-13	0.4411343	0.743	0.756	1.9892E-09	IMs
CLASRP	1.3339E-13	0.74310875	0.451	0.384	2.0504E-09	IMs
SUGP2	1.4204E-13	0.4778658	0.515	0.449	2.1833E-09	IMs
MT-CO2	1.4785E-13	0.64274939	0.509	0.451	2.2726E-09	IMs
OSM	1.5418E-13	1.15615852	0.472	0.397	2.3699E-09	IMs
DHX15	1.5852E-13	0.33559216	0.73	0.732	2.4366E-09	IMs
PHLDA2	1.5929E-13	1.4255429	0.181	0.112	2.4484E-09	IMs
SKIL	1.615E-13	0.35195787	0.848	0.871	2.4825E-09	IMs
SLC39A13	1.7118E-13	1.00354984	0.276	0.201	2.6312E-09	IMs
FUS	1.7399E-13	0.55484926	0.59	0.559	2.6744E-09	IMs
KIAA1614	1.7475E-13	2.84975019	0.018	0.003	2.6861E-09	IMs
GPR34	1.7726E-13	1.4836224	0.37	0.315	2.7246E-09	IMs
REL	1.8021E-13	0.63511857	0.712	0.708	2.7701E-09	IMs
DNM3	1.9455E-13	1.94261247	0.027	0.007	2.9904E-09	IMs
HELB	2.1467E-13	0.59185514	0.187	0.117	3.2997E-09	IMs
GLIPR1	2.1601E-13	0.40444964	0.776	0.787	3.3203E-09	IMs
FERMT31	2.2049E-13	0.23291201	0.927	0.931	3.3891E-09	IMs
PRKAG2	2.8316E-13	0.99675217	0.312	0.234	4.3525E-09	IMs
SAMHD1	3.1415E-13	0.17205432	0.992	0.982	4.8288E-09	IMs
UCP2	3.2536E-13	0.29334241	0.931	0.939	5.001E-09	IMs
SEC14L1	3.2707E-13	0.50756466	0.651	0.64	5.0274E-09	IMs
TNFRSF18	3.9448E-13	0.36104562	0.021	0.005	6.0635E-09	IMs
TTLL3	4.0038E-13	0.65366607	0.397	0.325	6.1543E-09	IMs
CDKN1C	4.1185E-13	2.32357281	0.031	0.009	6.3305E-09	IMs
BIRC3	4.35E-13	0.75760133	0.122	0.066	6.6864E-09	IMs
CHSY1	4.7693E-13	0.78343027	0.425	0.364	7.3309E-09	IMs
SPECC1	5.0242E-13	1.81259662	0.094	0.046	7.7227E-09	IMs
FAR1	5.0944E-13	0.4637954	0.69	0.691	7.8306E-09	IMs
RBM33	5.1915E-13	0.29773048	0.733	0.72	7.9798E-09	IMs
TM6SF11	5.2611E-13	0.49365172	0.702	0.725	8.0868E-09	IMs
OGT	5.3047E-13	0.34179121	0.587	0.548	8.1539E-09	IMs
DYRK2	5.5035E-13	0.62551709	0.399	0.327	8.4595E-09	IMs
PILRB	5.8287E-13	0.45574778	0.465	0.385	8.9593E-09	IMs
NRGN	6.1454E-13	1.16953071	0.25	0.175	9.4461E-09	IMs
TYK2	6.3702E-13	0.56821104	0.622	0.601	9.7916E-09	IMs
UCHL1	6.8926E-13	3.03909672	0.014	0.002	1.0595E-08	IMs
PTAR1	7.6479E-13	0.66969151	0.38	0.308	1.1756E-08	IMs
SDSL1	7.9011E-13	1.14723413	0.319	0.248	1.2145E-08	IMs
ITPRIPL1	9.1029E-13	0.35152069	0.019	0.004	1.3992E-08	IMs
GADD45G	9.1365E-13	1.10130814	0.16	0.095	1.4044E-08	IMs
MELTF	9.5982E-13	1.9883451	0.036	0.011	1.4753E-08	IMs
C1R	9.9263E-13	1.55385521	0.056	0.022	1.5258E-08	IMs
IL61	1.0967E-12	1.83364269	0.083	0.039	1.6858E-08	IMs
CORO7	1.2326E-12	0.39142989	0.72	0.738	1.8946E-08	IMs
TNFSF15	1.2709E-12	2.90294299	0.029	0.008	1.9535E-08	IMs

ADARB1	1.3029E-12	0.37056893	0.035	0.011	2.0027E-08	IMs
NRM	1.3038E-12	1.25021989	0.151	0.089	2.004E-08	IMs
SNX9	1.3724E-12	0.65259243	0.589	0.568	2.1095E-08	IMs
ARID5A	1.4193E-12	0.65897932	0.576	0.558	2.1816E-08	IMs
DMTF1	1.4263E-12	0.64147886	0.422	0.35	2.1924E-08	IMs
BRD2	1.4942E-12	0.3029414	0.815	0.833	2.2968E-08	IMs
NIBAN1	1.6341E-12	0.47077007	0.699	0.704	2.5118E-08	IMs
TP53	1.6638E-12	0.63792318	0.517	0.468	2.5574E-08	IMs
MS4A14	1.799E-12	0.58741884	0.495	0.437	2.7652E-08	IMs
C6orf62	1.8911E-12	0.43240362	0.653	0.659	2.9069E-08	IMs
MARVELD1	1.9356E-12	0.6036513	0.549	0.515	2.9752E-08	IMs
LIPN	2.0398E-12	1.82205712	0.104	0.054	3.1354E-08	IMs
HORMAD1	2.0558E-12	1.47411264	0.069	0.03	3.16E-08	IMs
EPB41L2	2.0851E-12	0.99806479	0.373	0.307	3.2049E-08	IMs
SLC29A31	2.1428E-12	0.67926386	0.603	0.605	3.2937E-08	IMs
NRN1	2.1512E-12	2.61178209	0.019	0.004	3.3066E-08	IMs
RYBP	2.2213E-12	0.76038547	0.422	0.364	3.4143E-08	IMs
ZC3H12D	2.2703E-12	0.85155044	0.093	0.046	3.4897E-08	IMs
PLB1	2.2782E-12	1.38040542	0.14	0.082	3.5018E-08	IMs
PHGDH	2.284E-12	1.56993847	0.101	0.052	3.5108E-08	IMs
MT-ND4	2.3321E-12	0.70633699	0.48	0.419	3.5846E-08	IMs
RBM25	2.4718E-12	0.34469536	0.69	0.686	3.7994E-08	IMs
STAG1	2.5532E-12	0.50657022	0.527	0.482	3.9246E-08	IMs
KCTD7	2.6948E-12	0.78489331	0.174	0.108	4.1422E-08	IMs
FKBP5	2.7889E-12	0.55369066	0.658	0.66	4.2869E-08	IMs
CREBL2	3.0969E-12	0.46628913	0.695	0.707	4.7602E-08	IMs
HSF4	3.1137E-12	1.41696559	0.039	0.013	4.7861E-08	IMs
RARRES11	3.1398E-12	1.71604684	0.103	0.053	4.8262E-08	IMs
JARID2	3.1532E-12	0.82346602	0.386	0.314	4.8467E-08	IMs
ITSN2	3.1805E-12	0.44153643	0.639	0.64	4.8887E-08	IMs
HPS3	3.4277E-12	0.70035607	0.464	0.409	5.2687E-08	IMs
SLC5A3	3.4684E-12	0.96217506	0.248	0.174	5.3313E-08	IMs
ARRDC2	3.5695E-12	0.55002516	0.586	0.564	5.4867E-08	IMs
USP4	3.8207E-12	0.56185483	0.552	0.514	5.8728E-08	IMs
HNRNPD	3.8348E-12	0.36893575	0.715	0.719	5.8945E-08	IMs
CD741	3.9464E-12	0.13765773	1	0.995	6.0661E-08	IMs
RGL2	3.9673E-12	0.79277892	0.404	0.345	6.0981E-08	IMs
RBM5	4.1478E-12	0.62098655	0.445	0.394	6.3756E-08	IMs
MAP1S	4.1784E-12	0.45426975	0.676	0.676	6.4227E-08	IMs
NEK61	4.4721E-12	0.30426044	0.911	0.918	6.8741E-08	IMs
OAS3	5.1798E-12	0.45242517	0.82	0.832	7.9618E-08	IMs
SDC4	5.2174E-12	0.85514727	0.435	0.388	8.0196E-08	IMs
ZFHX3	5.4511E-12	0.67659902	0.558	0.532	8.3789E-08	IMs
RFX5	6.3363E-12	0.53455079	0.646	0.649	9.7395E-08	IMs
PRMT2	7.0906E-12	0.34645468	0.763	0.789	1.0899E-07	IMs
RGS18	7.3092E-12	1.35416035	0.182	0.118	1.1235E-07	IMs
HSPA8	8.0194E-12	0.27765386	0.939	0.95	1.2327E-07	IMs
ORAI1	8.5146E-12	0.48779992	0.638	0.641	1.3088E-07	IMs
DLGAP3	8.6898E-12	2.73139247	0.012	0.002	1.3357E-07	IMs
IFNAR1	8.7679E-12	0.42058819	0.645	0.639	1.3477E-07	IMs
LBR	9.1282E-12	0.60620984	0.416	0.35	1.4031E-07	IMs
HIST3H2BB	9.6814E-12	1.88035209	0.018	0.004	1.4881E-07	IMs

DNAJC5	9.726E-12	0.36246891	0.717	0.731	1.495E-07	IMs
MAP2K2	9.914E-12	0.31791124	0.735	0.746	1.5239E-07	IMs
TRAF3	9.9925E-12	0.83470143	0.342	0.277	1.536E-07	IMs
DNAJA4	9.9957E-12	0.19410633	0.372	0.521	1.5364E-07	IMs
CD58	1.0488E-11	0.45207159	0.66	0.656	1.6121E-07	IMs
SLFN5	1.0681E-11	0.3567009	0.541	0.496	1.6417E-07	IMs
GSN1	1.0696E-11	0.25852954	0.964	0.934	1.6441E-07	IMs
MARCH3	1.0747E-11	1.65189056	0.071	0.032	1.6519E-07	IMs
ZNF219	1.0862E-11	1.14467949	0.228	0.16	1.6695E-07	IMs
TAF4B	1.1462E-11	1.71959649	0.059	0.025	1.7618E-07	IMs
AMDHD2	1.1755E-11	0.74863607	0.387	0.317	1.8068E-07	IMs
RERE	1.1986E-11	0.54834128	0.536	0.501	1.8423E-07	IMs
LMO7	1.2027E-11	1.15435181	0.042	0.015	1.8487E-07	IMs
SLC16A101	1.2755E-11	1.30060217	0.253	0.184	1.9605E-07	IMs
DAB21	1.3515E-11	0.57372725	0.894	0.933	2.0774E-07	IMs
AKAP17A	1.362E-11	0.42255739	0.53	0.485	2.0935E-07	IMs
L3MBTL4	1.3822E-11	1.23558179	0.14	0.083	2.1246E-07	IMs
PHYHD1	1.4722E-11	2.21406353	0.015	0.003	2.2629E-07	IMs
BICD1	1.5764E-11	0.95612895	0.084	0.041	2.4231E-07	IMs
CRK	1.7875E-11	0.43854052	0.656	0.645	2.7476E-07	IMs
PSMB9	1.8302E-11	0.43774433	0.77	0.814	2.8133E-07	IMs
FURIN	1.9159E-11	0.45029521	0.643	0.658	2.9449E-07	IMs
STRADA	1.9479E-11	0.81787748	0.343	0.276	2.9942E-07	IMs
DLGAP4	2.0632E-11	0.62575141	0.532	0.503	3.1714E-07	IMs
LIPA1	2.0868E-11	0.50456903	0.886	0.92	3.2076E-07	IMs
NUB1	2.1026E-11	0.58470439	0.7	0.736	3.2319E-07	IMs
ADCK2	2.6107E-11	0.56761342	0.568	0.551	4.0129E-07	IMs
TBC1D2B	2.7302E-11	0.38846371	0.631	0.609	4.1966E-07	IMs
DNAJB11	2.9175E-11	0.50209483	0.663	0.671	4.4844E-07	IMs
SLC6A8	3.1114E-11	2.67920373	0.023	0.006	4.7825E-07	IMs
RBM471	3.1204E-11	0.37611142	0.843	0.866	4.7963E-07	IMs
SNX6	3.6804E-11	0.39059765	0.728	0.753	5.6572E-07	IMs
DIP2A	3.7289E-11	0.6666905	0.319	0.25	5.7317E-07	IMs
PLEKHF2	3.9647E-11	0.66716467	0.504	0.47	6.0941E-07	IMs
DENND1A	3.9647E-11	0.64402172	0.54	0.513	6.0941E-07	IMs
ZDHHC17	3.9773E-11	0.80369825	0.343	0.273	6.1135E-07	IMs
ABI1	3.9781E-11	0.32416081	0.749	0.764	6.1147E-07	IMs
HMGA1	4.1341E-11	1.90120599	0.055	0.023	6.3545E-07	IMs
AFDN1	4.262E-11	0.85240772	0.526	0.525	6.5511E-07	IMs
IL18R1	4.3978E-11	0.35222451	0.022	0.006	6.7598E-07	IMs
ANKHD1	4.4915E-11	0.29817596	0.712	0.711	6.904E-07	IMs
TRAM1	4.8189E-11	0.23941298	0.855	0.891	7.4072E-07	IMs
MEP1A	4.9461E-11	2.75142975	0.015	0.003	7.6027E-07	IMs
CD401	5.4541E-11	0.64635079	0.638	0.638	8.3835E-07	IMs
ICAM5	5.6796E-11	2.45211482	0.023	0.006	8.7301E-07	IMs
USP9X	5.7155E-11	0.34660677	0.759	0.783	8.7853E-07	IMs
SNX30	6.3128E-11	0.54684033	0.549	0.515	9.7034E-07	IMs
TMEM86A1	6.4394E-11	0.67409838	0.477	0.429	9.8981E-07	IMs
FUBP1	6.6319E-11	0.44321466	0.613	0.609	1.0194E-06	IMs
EVC	6.7344E-11	1.29693123	0.016	0.003	1.0351E-06	IMs
KIDINS220	6.7812E-11	0.51830452	0.561	0.537	1.0423E-06	IMs
CARMIL3	7.0798E-11	2.07556021	0.015	0.003	1.0882E-06	IMs

TNFRSF10A	7.1061E-11	0.93127703	0.188	0.126	1.0923E-06	IMs
HSD11B11	7.1566E-11	1.84968356	0.064	0.029	1.1E-06	IMs
CPQ	7.1937E-11	0.52176524	0.594	0.583	1.1057E-06	IMs
YBX3	7.4123E-11	0.44286284	0.694	0.705	1.1393E-06	IMs
HTRA41	7.7522E-11	1.57486165	0.199	0.137	1.1916E-06	IMs
GAB2	7.813E-11	0.63387213	0.509	0.478	1.2009E-06	IMs
FCGR2A1	7.8628E-11	0.44008948	0.844	0.873	1.2086E-06	IMs
BTG2	8.0802E-11	0.5907208	0.731	0.754	1.242E-06	IMs
DNM1L	8.2805E-11	0.50536527	0.598	0.595	1.2728E-06	IMs
DUSP5	8.3086E-11	1.02261585	0.117	0.067	1.2771E-06	IMs
RB1	8.3189E-11	0.71330009	0.473	0.433	1.2787E-06	IMs
PAQR6	8.3461E-11	1.68481234	0.053	0.022	1.2829E-06	IMs
TTL	9.0163E-11	0.53551316	0.618	0.607	1.3859E-06	IMs
PPFIBP1	9.3823E-11	1.13540809	0.213	0.149	1.4422E-06	IMs
GNA15	9.4949E-11	0.4460805	0.677	0.679	1.4595E-06	IMs
ADA21	9.6745E-11	0.27068816	0.925	0.942	1.4871E-06	IMs
DAZAP2	9.7883E-11	0.29811893	0.808	0.842	1.5046E-06	IMs
FCGBP	9.8826E-11	1.92071909	0.035	0.012	1.519E-06	IMs
SH3D21	1.0133E-10	1.85850013	0.041	0.015	1.5575E-06	IMs
ARL8A	1.0407E-10	0.2660753	0.819	0.849	1.5997E-06	IMs
ARFGAP3	1.044E-10	0.59364223	0.613	0.615	1.6047E-06	IMs
LY961	1.0935E-10	0.47979402	0.665	0.691	1.6809E-06	IMs
PNISR	1.1095E-10	0.42621959	0.531	0.492	1.7055E-06	IMs
ELOVL5	1.2028E-10	0.43686298	0.606	0.59	1.8488E-06	IMs
ACSL3	1.2182E-10	0.54332527	0.589	0.579	1.8725E-06	IMs
ARL8B	1.2597E-10	0.36972586	0.77	0.784	1.9363E-06	IMs
APAF1	1.2609E-10	0.86440544	0.35	0.291	1.9382E-06	IMs
SPAG9	1.3034E-10	0.54346941	0.618	0.624	2.0035E-06	IMs
TRMT1	1.3303E-10	0.65654971	0.429	0.377	2.0447E-06	IMs
MYLK	1.3581E-10	1.97232779	0.012	0.002	2.0875E-06	IMs
CLK4	1.4572E-10	0.76361311	0.264	0.197	2.2399E-06	IMs
ZNF503	1.5058E-10	0.98405349	0.242	0.175	2.3146E-06	IMs
EPS8L2	1.5347E-10	0.41611681	0.018	0.004	2.3589E-06	IMs
LACC1	1.535E-10	0.93931446	0.313	0.248	2.3595E-06	IMs
ABCC2	1.6871E-10	2.77586554	0.013	0.002	2.5933E-06	IMs
CALU	1.7064E-10	0.40760762	0.708	0.735	2.6229E-06	IMs
FBXW5	1.754E-10	0.36145024	0.685	0.701	2.6961E-06	IMs
ABR1	1.7633E-10	0.25103215	0.864	0.887	2.7104E-06	IMs
PGBD5	1.8493E-10	2.90334767	0.013	0.002	2.8426E-06	IMs
AHDC1	1.9174E-10	1.21815095	0.103	0.057	2.9473E-06	IMs
SERPINH1	2.0376E-10	0.91784042	0.341	0.28	3.132E-06	IMs
H1F0	2.0848E-10	0.83702923	0.464	0.415	3.2045E-06	IMs
TSPAN4	2.1905E-10	0.71033692	0.507	0.49	3.367E-06	IMs
DHRS9	2.299E-10	1.24997499	0.207	0.146	3.5337E-06	IMs
DIP2B	2.3525E-10	0.65900713	0.474	0.435	3.6161E-06	IMs
VDR	2.494E-10	0.74147137	0.452	0.403	3.8335E-06	IMs
UBE2Q2	2.5799E-10	0.56543846	0.555	0.525	3.9656E-06	IMs
SENP7	2.6034E-10	0.82540313	0.172	0.112	4.0017E-06	IMs
CDK5RAP3	2.7311E-10	0.30426025	0.71	0.717	4.198E-06	IMs
SUCNR1	2.7983E-10	0.90016672	0.492	0.467	4.3013E-06	IMs
RRAD	2.8654E-10	2.58712977	0.013	0.002	4.4043E-06	IMs
FLT11	2.9042E-10	1.50073004	0.09	0.048	4.464E-06	IMs

CCNL2	2.9359E-10	0.37730688	0.536	0.495	4.5128E-06	IMs
DCLK2	2.9406E-10	2.33429094	0.052	0.022	4.52E-06	IMs
ENTR1	2.9597E-10	0.71466899	0.306	0.24	4.5494E-06	IMs
DEPDC5	3.0342E-10	0.63161438	0.412	0.357	4.6639E-06	IMs
R3HDM4	3.1109E-10	0.47496374	0.621	0.618	4.7818E-06	IMs
TCP11L1	3.2312E-10	0.76976465	0.326	0.266	4.9666E-06	IMs
FBXO41	3.4009E-10	1.07686739	0.179	0.119	5.2275E-06	IMs
PACC1	3.4413E-10	0.62906692	0.47	0.431	5.2897E-06	IMs
PI4KA	3.5123E-10	0.42761588	0.633	0.632	5.3987E-06	IMs
LAMP5	3.916E-10	2.96676356	0.014	0.003	6.0193E-06	IMs
RIPK21	4.0993E-10	0.65340634	0.599	0.585	6.3011E-06	IMs
SRGAP3	4.502E-10	0.99323828	0.024	0.007	6.92E-06	IMs
MYSM1	4.5813E-10	0.54966032	0.382	0.325	7.0419E-06	IMs
ZFYVE161	4.6418E-10	0.5171208	0.671	0.668	7.1349E-06	IMs
ZBTB18	4.7249E-10	0.92204225	0.358	0.3	7.2626E-06	IMs
NSD1	4.765E-10	0.4576094	0.59	0.582	7.3243E-06	IMs
RHOF	5.0645E-10	0.71821302	0.155	0.098	7.7847E-06	IMs
DOP1B	5.1317E-10	0.89193561	0.287	0.224	7.8879E-06	IMs
NRAS	5.2546E-10	0.62898957	0.491	0.464	8.0769E-06	IMs
FMNL3	5.4103E-10	0.50737109	0.651	0.665	8.3161E-06	IMs
AGPAT3	5.7782E-10	0.29364473	0.763	0.791	8.8816E-06	IMs
ZNF33B	5.7878E-10	1.15108197	0.079	0.041	8.8964E-06	IMs
LPP	5.9127E-10	0.32981179	0.674	0.684	9.0884E-06	IMs
PBRM1	6.0337E-10	0.51982175	0.536	0.533	9.2744E-06	IMs
CEACAM4	6.0889E-10	1.29748862	0.134	0.082	9.3593E-06	IMs
ARNT	6.2567E-10	0.67392417	0.462	0.429	9.6171E-06	IMs
COL8A2	6.5036E-10	0.63921529	0.539	0.504	9.9966E-06	IMs
ZBED1	7.0343E-10	0.51959642	0.552	0.536	1.0812E-05	IMs
TMCC2	7.0349E-10	2.21533347	0.018	0.004	1.0813E-05	IMs
OGA	7.4506E-10	0.15665824	0.762	0.753	1.1452E-05	IMs
TCERG1	7.4548E-10	0.51109698	0.538	0.517	1.1459E-05	IMs
CCL3L1	7.763E-10	2.01349515	0.013	0.002	1.1932E-05	IMs
ABCB6	7.9245E-10	1.1474148	0.062	0.029	1.2181E-05	IMs
SAP25	7.9309E-10	0.69279188	0.156	0.099	1.2191E-05	IMs
PKD1L3	8.2807E-10	2.33502615	0.021	0.006	1.2728E-05	IMs
WSB2	8.2937E-10	0.32635551	0.759	0.776	1.2748E-05	IMs
MAP3K8	8.6125E-10	0.42052341	0.777	0.778	1.3238E-05	IMs
TTC7A	9.1353E-10	0.36577581	0.785	0.815	1.4042E-05	IMs
TNK2	9.4675E-10	0.57728572	0.469	0.422	1.4553E-05	IMs
EHD3	9.667E-10	2.02794401	0.032	0.011	1.4859E-05	IMs
KMT2C	9.9377E-10	0.26282398	0.745	0.766	1.5275E-05	IMs
CEP68	1.0467E-09	0.9785155	0.186	0.128	1.6089E-05	IMs
UBAP2L	1.0572E-09	0.29321552	0.74	0.763	1.6251E-05	IMs
TMOD2	1.1283E-09	1.07464753	0.259	0.201	1.7343E-05	IMs
SPTBN1	1.1376E-09	0.88168588	0.051	0.022	1.7485E-05	IMs
AUP1	1.1517E-09	0.35078959	0.694	0.705	1.7703E-05	IMs
CYTH2	1.2715E-09	0.4632611	0.569	0.546	1.9545E-05	IMs
NRSN2	1.3086E-09	2.14871211	0.024	0.007	2.0115E-05	IMs
PRKX	1.3828E-09	0.89456149	0.137	0.085	2.1255E-05	IMs
AGER	1.4263E-09	0.8203892	0.108	0.062	2.1924E-05	IMs
HGSNAT	1.4324E-09	0.59875942	0.503	0.48	2.2018E-05	IMs
NHSL1	1.5165E-09	1.17272105	0.157	0.103	2.331E-05	IMs

OSGIN1	1.577E-09	2.20706306	0.041	0.016	2.4241E-05	IMs
UTS2	1.7237E-09	2.24712559	0.014	0.003	2.6495E-05	IMs
CERS2	1.819E-09	0.26946598	0.777	0.815	2.796E-05	IMs
OGDH	1.863E-09	0.30189877	0.796	0.811	2.8636E-05	IMs
EIF4A3	1.8835E-09	0.30311712	0.741	0.768	2.8952E-05	IMs
INSIG1	1.9418E-09	0.98375273	0.409	0.361	2.9848E-05	IMs
LYG1	1.9436E-09	1.76745657	0.028	0.009	2.9874E-05	IMs
PCF11	2.0335E-09	0.47888477	0.605	0.59	3.1257E-05	IMs
CC2D2B	2.0491E-09	2.1523113	0.027	0.009	3.1497E-05	IMs
RAB311	2.1518E-09	0.30508876	0.868	0.887	3.3075E-05	IMs
WDR82	2.1756E-09	0.34805416	0.647	0.66	3.3442E-05	IMs
IQGAP2	2.2342E-09	0.40293425	0.661	0.681	3.4342E-05	IMs
BACH11	2.2857E-09	0.33473396	0.865	0.88	3.5134E-05	IMs
STK4	2.3264E-09	0.17888098	0.808	0.837	3.5758E-05	IMs
MEX3B	2.3695E-09	1.37161641	0.09	0.049	3.6421E-05	IMs
LRRC37B	2.4182E-09	1.03988229	0.123	0.075	3.7171E-05	IMs
FAM50A	2.602E-09	0.28129903	0.768	0.805	3.9995E-05	IMs
CLK2	2.7147E-09	1.09241755	0.152	0.099	4.1728E-05	IMs
MT-ND3	2.7177E-09	0.5628057	0.492	0.44	4.1774E-05	IMs
DCK	2.8178E-09	0.44712032	0.541	0.527	4.3312E-05	IMs
CD721	2.9279E-09	1.07951171	0.188	0.131	4.5005E-05	IMs
BBC3	2.9704E-09	0.88050088	0.279	0.219	4.5658E-05	IMs
NMI	3.2917E-09	0.45178902	0.629	0.643	5.0597E-05	IMs
MFSD6L	3.311E-09	1.91897781	0.012	0.002	5.0894E-05	IMs
DDX3X	3.3737E-09	0.52530406	0.491	0.454	5.1857E-05	IMs
BCL9L	3.6357E-09	0.51110997	0.266	0.204	5.5885E-05	IMs
RGS14	3.7486E-09	1.12220065	0.223	0.168	5.7619E-05	IMs
C17orf49	3.7751E-09	0.72650767	0.424	0.379	5.8026E-05	IMs
BLVRB	4.2108E-09	0.52758146	0.562	0.547	6.4724E-05	IMs
GPX3	4.4624E-09	0.74579287	0.412	0.368	6.8591E-05	IMs
MRAS1	4.499E-09	0.87500682	0.402	0.355	6.9154E-05	IMs
VSTM1	4.6951E-09	2.66161249	0.024	0.008	7.2168E-05	IMs
SIGLEC13	4.7902E-09	0.36493437	0.76	0.8	7.363E-05	IMs
LRP8	4.8109E-09	1.187467	0.127	0.079	7.3949E-05	IMs
APBB3	5.0131E-09	0.70029779	0.244	0.18	7.7057E-05	IMs
FRMD3	5.0619E-09	1.65403601	0.056	0.026	7.7806E-05	IMs
TNFAIP61	5.1095E-09	0.89299409	0.065	0.032	7.8539E-05	IMs
DENND6B	5.2733E-09	1.44328898	0.076	0.04	8.1057E-05	IMs
USP53	5.4073E-09	1.32316759	0.134	0.085	8.3116E-05	IMs
ILF3	5.6904E-09	0.30000127	0.743	0.76	8.7467E-05	IMs
DRAM21	5.7141E-09	0.34612906	0.711	0.734	8.7831E-05	IMs
ASH1L	5.7255E-09	0.30703428	0.608	0.602	8.8007E-05	IMs
GEMIN7	5.9257E-09	0.88097159	0.303	0.246	9.1084E-05	IMs
LPAR1	6.8047E-09	0.78831576	0.305	0.242	0.00010459	IMs
EIF3J	6.8535E-09	0.42110474	0.596	0.589	0.00010535	IMs
PELI1	7E-09	0.85014834	0.338	0.283	0.0001076	IMs
USF2	7.0373E-09	0.26817634	0.87	0.894	0.00010817	IMs
AKT1S1	7.0695E-09	0.96022405	0.367	0.321	0.00010866	IMs
UBE3A	7.4502E-09	0.35333225	0.638	0.647	0.00011452	IMs
CCDC186	7.4724E-09	0.93192783	0.242	0.185	0.00011486	IMs
SFT2D2	8.0789E-09	0.33679078	0.699	0.716	0.00012418	IMs
PLEKHN1	8.3792E-09	1.38254693	0.029	0.01	0.0001288	IMs

TP53BP2	8.6491E-09	0.70599052	0.36	0.31	0.00013295	IMs
CTNS	8.6825E-09	0.88410626	0.337	0.286	0.00013346	IMs
MLXIP	8.8876E-09	0.70063843	0.337	0.282	0.00013661	IMs
FCHO1	8.9161E-09	0.8822112	0.183	0.129	0.00013705	IMs
MICAL1	9.0786E-09	0.299115	0.821	0.847	0.00013955	IMs
CHMP1B	9.2086E-09	0.48848503	0.746	0.793	0.00014155	IMs
UBAC1	9.5141E-09	0.55166712	0.547	0.54	0.00014624	IMs
CHD1	9.7175E-09	0.54948536	0.448	0.405	0.00014937	IMs
KIF13B	9.7298E-09	0.54196294	0.451	0.41	0.00014956	IMs
FAM193B	9.9795E-09	0.52687917	0.377	0.325	0.00015339	IMs
FNIP1	1.0257E-08	0.64493799	0.458	0.423	0.00015767	IMs
TLR4	1.1884E-08	0.38687125	0.695	0.719	0.00018266	IMs
TSPOAP1	1.2312E-08	0.93027737	0.06	0.029	0.00018925	IMs
ZC3H8	1.3005E-08	0.87262009	0.113	0.068	0.0001999	IMs
CARD11	1.3515E-08	0.2606232	0.071	0.037	0.00020774	IMs
TENT2	1.3548E-08	0.46199889	0.61	0.62	0.00020825	IMs
EYA3	1.3568E-08	0.75179464	0.363	0.312	0.00020855	IMs
ENGASE	1.3637E-08	0.80569549	0.207	0.151	0.00020961	IMs
STRIP2	1.3703E-08	2.22130927	0.021	0.006	0.00021063	IMs
ZDHHC14	1.3736E-08	1.10841016	0.114	0.07	0.00021113	IMs
PPIF	1.5233E-08	0.99365734	0.495	0.478	0.00023414	IMs
STAT21	1.5376E-08	0.33281608	0.779	0.81	0.00023635	IMs
APOL3	1.5803E-08	1.1458504	0.301	0.25	0.00024291	IMs
CALHM2	1.5827E-08	0.43162106	0.605	0.582	0.00024327	IMs
EVA1B	1.6102E-08	1.00989478	0.247	0.193	0.0002475	IMs
APH1A	1.7025E-08	0.27491667	0.722	0.758	0.00026169	IMs
EXT1	1.7517E-08	1.16276795	0.183	0.132	0.00026925	IMs
ANKDD1A	1.7636E-08	1.47863452	0.056	0.027	0.00027109	IMs
PHF21A	1.7642E-08	0.63828763	0.339	0.283	0.00027117	IMs
ARHGAP221	1.8387E-08	1.08958734	0.184	0.13	0.00028262	IMs
TUT7	1.8912E-08	0.21189203	0.874	0.88	0.0002907	IMs
TNFSF9	1.902E-08	1.66136103	0.053	0.025	0.00029235	IMs
OR52B2	2.0449E-08	2.28146551	0.01	0.002	0.00031432	IMs
PDIA6	2.0528E-08	0.49662778	0.586	0.6	0.00031554	IMs
YIPF4	2.1197E-08	0.37673181	0.629	0.637	0.00032582	IMs
SLC39A11	2.2556E-08	0.68488618	0.445	0.417	0.00034671	IMs
LILRA4	2.3047E-08	2.75945891	0.012	0.002	0.00035425	IMs
ZNF516	2.3278E-08	0.77328368	0.332	0.277	0.0003578	IMs
FCMR	2.4188E-08	0.58510453	0.036	0.014	0.0003718	IMs
PIM1	2.4253E-08	0.51009453	0.581	0.564	0.0003728	IMs
TMEM185B	2.5135E-08	0.545954	0.507	0.481	0.00038635	IMs
N4BP2	2.5409E-08	0.61728103	0.262	0.206	0.00039057	IMs
RFX3	2.9618E-08	0.94873169	0.191	0.138	0.00045525	IMs
FAM135A	2.9661E-08	1.22249369	0.108	0.065	0.00045592	IMs
UBAC2	2.9679E-08	0.66405146	0.404	0.362	0.00045619	IMs
PLPP5	3.0446E-08	0.57589055	0.439	0.404	0.00046799	IMs
ARHGAP9	3.1013E-08	0.51897089	0.52	0.506	0.0004767	IMs
IPO9	3.2076E-08	0.54964807	0.459	0.423	0.00049303	IMs
CASC3	3.5831E-08	0.34823885	0.68	0.687	0.00055075	IMs
TNFAIP8	3.5949E-08	0.60214259	0.479	0.466	0.00055257	IMs
ZNF267	3.6069E-08	0.72757248	0.291	0.235	0.00055441	IMs
RENBP1	3.708E-08	0.36518801	0.745	0.769	0.00056996	IMs

HNRNPLL	3.7608E-08	0.48379603	0.491	0.474	0.00057807	IMs
DUSP14	3.7966E-08	1.24244489	0.032	0.013	0.00058357	IMs
MAPK8IP3	3.8083E-08	0.91865054	0.167	0.117	0.00058537	IMs
ADAP1	3.92E-08	0.73893691	0.374	0.329	0.00060254	IMs
SND1	3.9923E-08	0.39952569	0.634	0.653	0.00061366	IMs
NPEPPS	4.0515E-08	0.37402814	0.636	0.649	0.00062275	IMs
PTPA	4.0716E-08	0.34250153	0.667	0.695	0.00062584	IMs
TXLNB	4.2624E-08	1.54987576	0.049	0.023	0.00065517	IMs
UTP6	4.2889E-08	0.56179376	0.468	0.446	0.00065925	IMs
GRPR	4.5347E-08	3.05603798	0.012	0.003	0.00069703	IMs
NEK10	4.5616E-08	0.91660249	0.012	0.003	0.00070116	IMs
RBM6	4.6762E-08	0.35622112	0.54	0.51	0.00071877	IMs
MARCH11	4.9114E-08	0.33962419	0.753	0.794	0.00075493	IMs
IRAK4	5.0682E-08	0.49229042	0.408	0.366	0.00077903	IMs
ST6GALNAC3	5.1505E-08	1.97108431	0.032	0.013	0.00079168	IMs
DLG4	5.1722E-08	0.81415076	0.345	0.3	0.00079501	IMs
PRG2	5.2363E-08	2.39929826	0.015	0.004	0.00080487	IMs
KBTBD2	5.3076E-08	0.47738971	0.524	0.509	0.00081583	IMs
HNRNPA3	5.3472E-08	0.41130999	0.65	0.663	0.00082193	IMs
TEDC1	5.3654E-08	0.8579147	0.334	0.286	0.00082472	IMs
LY6G5B	5.3665E-08	0.74725283	0.149	0.099	0.00082488	IMs
SPACA6	5.5516E-08	1.55821621	0.036	0.015	0.00085333	IMs
WNK1	5.8189E-08	0.22716083	0.792	0.834	0.00089442	IMs
SEMA7A	6.1199E-08	1.70181155	0.062	0.032	0.00094069	IMs
WSB1	6.1613E-08	0.18335622	0.803	0.812	0.00094705	IMs
ZNF296	6.1676E-08	1.61579826	0.076	0.042	0.00094802	IMs
CD2741	6.1769E-08	0.11520378	0.266	0.376	0.00094945	IMs
BMERB1	6.2575E-08	2.84198632	0.01	0.002	0.00096184	IMs
TIAL1	6.3445E-08	0.33462299	0.63	0.647	0.00097522	IMs
INAFM2	6.4145E-08	0.64880872	0.387	0.34	0.00098597	IMs
SRPK2	6.4948E-08	0.5643144	0.438	0.405	0.00099831	IMs
PIKFYVE	6.572E-08	0.34895504	0.579	0.561	0.00101018	IMs
MAML1	6.8343E-08	0.38908104	0.551	0.543	0.0010505	IMs
THRB	6.9095E-08	2.08768655	0.012	0.003	0.00106206	IMs
PBX2	7.0756E-08	0.54042402	0.446	0.408	0.0010876	IMs
CPVL1	7.4518E-08	0.29242295	0.855	0.902	0.00114541	IMs
CPT1B	7.6713E-08	0.84943168	0.118	0.075	0.00117915	IMs
EDC4	7.8716E-08	0.53279401	0.471	0.443	0.00120994	IMs
MYOF1	7.9573E-08	0.38634086	0.785	0.829	0.00122312	IMs
PDPR	7.9986E-08	0.60114667	0.401	0.362	0.00122946	IMs
H1FX	8.0364E-08	0.40757469	0.635	0.624	0.00123528	IMs
PLOD3	8.0817E-08	0.28422491	0.779	0.811	0.00124223	IMs
SULT1C21	8.2429E-08	1.63040985	0.027	0.01	0.00126702	IMs
ANKLE2	8.3179E-08	0.4400048	0.544	0.528	0.00127854	IMs
NUFIP2	8.4387E-08	0.2645766	0.736	0.773	0.00129711	IMs
RTN4RL2	8.7747E-08	2.65235677	0.01	0.002	0.00134876	IMs
PLCG21	8.8763E-08	0.46118803	0.668	0.683	0.00136438	IMs
ITPRID2	9.4037E-08	0.40880124	0.624	0.634	0.00144544	IMs
HSPA1B	9.4583E-08	0.66843545	0.497	0.653	0.00145383	IMs
LRRC37A3	9.8025E-08	1.64881104	0.027	0.01	0.00150674	IMs
ZYX	9.9771E-08	0.1731609	0.969	0.962	0.00153358	IMs
HMBOX1	1.019E-07	0.62697383	0.331	0.281	0.00156624	IMs

GON4L	1.0226E-07	0.46060132	0.464	0.436	0.0015719	IMs
IFFO2	1.0879E-07	0.69636997	0.174	0.125	0.00167214	IMs
MYO10	1.1156E-07	1.961639	0.027	0.01	0.00171471	IMs
TAF7	1.1344E-07	0.41663326	0.513	0.489	0.0017437	IMs
MTURN	1.1394E-07	1.24884001	0.106	0.066	0.00175134	IMs
CLEC2B	1.1479E-07	0.37147087	0.627	0.654	0.00176443	IMs
RBM38	1.2258E-07	0.53538781	0.16	0.111	0.00188418	IMs
ARHGAP15	1.2278E-07	0.27230774	0.516	0.495	0.00188722	IMs
SGPL1	1.2998E-07	0.76361301	0.431	0.407	0.00199793	IMs
CYTH3	1.3017E-07	1.06336504	0.194	0.143	0.00200077	IMs
LDB1	1.3258E-07	0.32833448	0.59	0.581	0.00203786	IMs
IMPDH2	1.3707E-07	0.75673216	0.347	0.303	0.00210683	IMs
ACTG1	1.3988E-07	0.14342191	0.982	0.97	0.00215004	IMs
TOB1	1.4451E-07	0.5569555	0.535	0.531	0.00222132	IMs
ERN1	1.5155E-07	0.36217348	0.217	0.163	0.00232943	IMs
LMCD1	1.5279E-07	1.83251971	0.025	0.009	0.00234849	IMs
YIF1B	1.5637E-07	0.545126	0.525	0.52	0.00240352	IMs
GBP5	1.577E-07	1.11130557	0.405	0.379	0.00242402	IMs
EXOC3	1.7788E-07	0.21481827	0.731	0.777	0.00273412	IMs
VPS35L	1.8181E-07	0.38493245	0.635	0.652	0.00279457	IMs
FOXP1	1.8741E-07	0.39608671	0.634	0.652	0.00288073	IMs
PRKAR1A	1.9264E-07	0.15796043	0.934	0.942	0.00296104	IMs
FHAD11	1.9403E-07	0.88354371	0.208	0.158	0.00298236	IMs
CLCN6	1.9808E-07	0.73854265	0.27	0.216	0.00304476	IMs
CUX1	2.0741E-07	0.50976044	0.496	0.485	0.00318811	IMs
ARHGEF10L1	2.1408E-07	0.28435029	0.849	0.863	0.00329061	IMs
SIAH1	2.194E-07	0.53104455	0.498	0.48	0.00337237	IMs
ACTR3	2.3849E-07	0.29326512	0.708	0.749	0.0036658	IMs
SLC16A6	2.3977E-07	0.79837021	0.266	0.214	0.00368555	IMs
CLU	2.4009E-07	0.19191552	0.021	0.007	0.00369037	IMs
CENPT	2.4416E-07	0.68681433	0.289	0.237	0.00375303	IMs
GPX8	2.5639E-07	2.46195236	0.014	0.004	0.00394101	IMs
SREBF1	2.586E-07	0.45066596	0.577	0.579	0.00397496	IMs
YARS	2.6553E-07	0.67318501	0.408	0.378	0.00408143	IMs
KHDRBS3	2.6903E-07	2.95917154	0.01	0.002	0.0041353	IMs
PHACTR4	2.6991E-07	0.48732476	0.48	0.458	0.00414872	IMs
RASAL3	2.7197E-07	0.21645126	0.271	0.217	0.00418053	IMs
BRWD3	2.8441E-07	0.6300466	0.292	0.241	0.00437163	IMs
FAM174B	2.8618E-07	1.24170314	0.019	0.006	0.00439889	IMs
MT-ND4L	2.9307E-07	0.67979894	0.34	0.289	0.00450471	IMs
PPP1R3G	2.9902E-07	2.35401403	0.015	0.004	0.00459622	IMs
GIGYF1	3.0785E-07	0.51901305	0.455	0.435	0.0047319	IMs
GABRR2	3.2744E-07	2.28665646	0.017	0.005	0.00503308	IMs
NRDC	3.296E-07	0.24444796	0.738	0.771	0.00506635	IMs
ARRDC3	3.3229E-07	0.75316879	0.422	0.388	0.00510767	IMs
RASGRF1	3.3697E-07	2.92643485	0.016	0.005	0.00517957	IMs
SLC15A2	3.4206E-07	1.31077568	0.077	0.044	0.00525783	IMs
MAPK1	3.5643E-07	0.19859467	0.874	0.907	0.00547861	IMs
CEP95	3.5928E-07	0.64236213	0.227	0.174	0.00552253	IMs
RUFY3	3.6566E-07	0.53872713	0.444	0.413	0.00562051	IMs
ZDHHC20	3.6921E-07	0.40936785	0.609	0.639	0.0056752	IMs
GIMAP4	3.7366E-07	0.54842443	0.627	0.676	0.0057435	IMs

PPM1L	3.7841E-07	1.17893968	0.138	0.094	0.00581651	IMs
NADK	4.0395E-07	0.41622031	0.651	0.681	0.00620915	IMs
PAPLN1	4.0965E-07	1.8372481	0.096	0.06	0.00629678	IMs
CEBPG	4.5406E-07	0.48699764	0.583	0.593	0.00697934	IMs
INSIG2	4.5762E-07	0.82561337	0.304	0.262	0.00703407	IMs
GLCCI1	4.5964E-07	0.68568576	0.1	0.063	0.00706518	IMs
LSS	4.6811E-07	0.62684157	0.361	0.319	0.00719527	IMs
H2AFY	4.7525E-07	0.27838551	0.796	0.839	0.00730507	IMs
CCDC112	4.7811E-07	0.90220534	0.23	0.181	0.00734899	IMs
LOXL31	5.356E-07	0.90310853	0.268	0.219	0.00823275	IMs
HPS1	5.5045E-07	0.33212276	0.672	0.704	0.0084609	IMs
TPRA1	5.635E-07	0.69289277	0.434	0.407	0.00866151	IMs
PDIA3	5.6578E-07	0.30917364	0.712	0.761	0.00869661	IMs
EP300	5.6958E-07	0.33274448	0.726	0.761	0.00875509	IMs
IL3RA	5.938E-07	0.53035698	0.672	0.661	0.00912729	IMs
FAM49A	6.0489E-07	0.59503803	0.496	0.476	0.00929775	IMs
MARCH8	6.0911E-07	0.81325965	0.323	0.28	0.00936255	IMs
OSBPL8	6.6267E-07	0.4800422	0.652	0.678	0.01018583	IMs
MGAT4B	6.7067E-07	0.31462043	0.653	0.673	0.01030893	IMs
DNAJC12	6.9908E-07	2.36406184	0.012	0.003	0.01074556	IMs
ACOT91	7.234E-07	0.33930332	0.699	0.723	0.01111939	IMs
RIMS3	7.5295E-07	1.00078705	0.02	0.007	0.01157356	IMs
CADM4	7.7692E-07	1.76194103	0.031	0.013	0.01194202	IMs
STX2	7.8608E-07	0.58887877	0.385	0.347	0.01208277	IMs
MYO5A	7.8619E-07	0.53287891	0.517	0.52	0.01208459	IMs
C1orf127	7.9318E-07	2.32692817	0.016	0.005	0.01219198	IMs
SLC39A12	8.3652E-07	2.11157355	0.025	0.01	0.01285809	IMs
ATG7	8.4115E-07	0.42761648	0.541	0.538	0.0129293	IMs
DYNC1LI2	8.4306E-07	0.42136825	0.576	0.59	0.0129587	IMs
KLHL24	8.6758E-07	0.55095722	0.434	0.408	0.01333562	IMs
CALCR	8.803E-07	2.00714131	0.02	0.007	0.01353109	IMs
SHMT2	8.8525E-07	1.03956848	0.176	0.13	0.01360721	IMs
THRAP3	8.9179E-07	0.2751345	0.7	0.728	0.01370778	IMs
TNFRSF21	9.1241E-07	1.00147659	0.259	0.211	0.01402463	IMs
SLC16A7	9.3206E-07	0.24838355	0.037	0.016	0.01432673	IMs
TMEM268	9.7179E-07	0.882211	0.202	0.155	0.01493746	IMs
C16orf72	1.0127E-06	0.36028778	0.606	0.625	0.01556564	IMs
GARS	1.0237E-06	0.76818905	0.291	0.247	0.01573524	IMs
SEMA4D	1.0445E-06	0.59123982	0.441	0.417	0.01605459	IMs
AGPAT5	1.0477E-06	0.81689716	0.243	0.196	0.01610345	IMs
FST	1.0478E-06	1.91744006	0.032	0.014	0.01610502	IMs
IAH1	1.0744E-06	0.39860136	0.496	0.476	0.01651517	IMs
C4BPB	1.1131E-06	2.13263287	0.014	0.004	0.01711015	IMs
EPC1	1.1256E-06	0.45988076	0.417	0.383	0.01730143	IMs
NF1	1.1304E-06	0.43809369	0.472	0.449	0.01737582	IMs
EIF1	1.1391E-06	0.17515027	0.932	0.94	0.01750896	IMs
TNFAIP3	1.1571E-06	0.66370725	0.53	0.531	0.01778643	IMs
NDST2	1.1649E-06	0.9449892	0.153	0.109	0.01790641	IMs
ABCD11	1.1796E-06	0.60910041	0.494	0.486	0.01813126	IMs
FOSL2	1.2402E-06	0.46308546	0.753	0.778	0.01906327	IMs
USP15	1.2659E-06	0.28394718	0.667	0.689	0.01945843	IMs
ROBO3	1.2688E-06	1.26840982	0.113	0.075	0.01950343	IMs

PRKY	1.2876E-06	1.21726308	0.03	0.013	0.01979132	IMs
FAM78A	1.2952E-06	0.48193304	0.46	0.431	0.01990776	IMs
DELE1	1.328E-06	0.52869859	0.464	0.447	0.02041198	IMs
TRAF2	1.3285E-06	0.96310883	0.173	0.127	0.02041967	IMs
ATP6V1F1	1.3413E-06	0.2527184	0.85	0.876	0.02061732	IMs
RMI2	1.3811E-06	1.15330771	0.105	0.068	0.02122927	IMs
DYM	1.4005E-06	0.35120631	0.586	0.593	0.02152637	IMs
NBEAL2	1.4034E-06	0.48991404	0.338	0.298	0.02157216	IMs
TLN1	1.4196E-06	0.1605191	0.899	0.918	0.02182003	IMs
TNRC18	1.4502E-06	0.49881317	0.478	0.462	0.02229039	IMs
TCF12	1.4897E-06	0.55215075	0.494	0.493	0.02289866	IMs
DEAF1	1.5946E-06	0.74125445	0.27	0.224	0.02451122	IMs
ZC3HAV1	1.6185E-06	0.34343296	0.65	0.677	0.02487815	IMs
EIF2AK4	1.6193E-06	0.57306287	0.481	0.481	0.02488967	IMs
TNFRSF12A	1.6832E-06	1.27827585	0.122	0.083	0.0258717	IMs
MAP7	1.6846E-06	1.13023624	0.103	0.066	0.02589474	IMs
CEP83	1.7045E-06	0.86719824	0.221	0.175	0.02619987	IMs
MAP3K12	1.7204E-06	1.01463603	0.1	0.064	0.02644438	IMs
SRRM2	1.7334E-06	0.35539651	0.579	0.573	0.02664415	IMs
AGO2	1.7693E-06	0.41712887	0.437	0.408	0.02719592	IMs
SNAP231	1.806E-06	0.68442398	0.395	0.372	0.02776078	IMs
PER3	1.8259E-06	0.70580666	0.301	0.258	0.02806546	IMs
FOSL1	1.854E-06	1.45671153	0.044	0.022	0.02849789	IMs
PRR5L	1.8939E-06	1.05926239	0.121	0.082	0.02911064	IMs
STK38	1.9189E-06	0.38258529	0.53	0.526	0.029496	IMs
NFKBIA	1.9514E-06	0.62783777	0.855	0.912	0.0299957	IMs
NFX1	1.9617E-06	0.41959899	0.5	0.488	0.03015266	IMs
DDX39A	1.9637E-06	0.52225132	0.464	0.441	0.03018397	IMs
SNN	1.9867E-06	1.00161214	0.16	0.116	0.03053779	IMs
GCNA	2.0824E-06	1.28153406	0.035	0.016	0.0320081	IMs
ZBTB10	2.1182E-06	0.79591067	0.312	0.269	0.0325586	IMs
GARNL3	2.1202E-06	3.09872718	0.013	0.003	0.03258998	IMs
LRRC8C	2.1766E-06	0.54308232	0.556	0.568	0.03345669	IMs
CACNB1	2.2575E-06	0.97460991	0.061	0.034	0.03469932	IMs
DYNC1LI1	2.285E-06	0.41095108	0.56	0.567	0.03512215	IMs
TTC14	2.3253E-06	0.30450538	0.41	0.364	0.03574196	IMs
RNF135	2.3782E-06	0.55256465	0.45	0.43	0.03655584	IMs
RNF115	2.3819E-06	0.4453165	0.472	0.456	0.03661153	IMs
FGD6	2.4274E-06	1.02611249	0.121	0.082	0.03731203	IMs
NKTR	2.4385E-06	0.34366297	0.408	0.368	0.03748266	IMs
QARS	2.5004E-06	0.2264959	0.757	0.81	0.03843395	IMs
STX16	2.5079E-06	0.28505514	0.574	0.566	0.038549	IMs
APOBEC3G	2.7289E-06	0.25700805	0.306	0.262	0.0419455	IMs
DENND4A	2.7368E-06	0.36210638	0.591	0.608	0.04206741	IMs
TNFSF4	2.7371E-06	0.54343781	0.018	0.006	0.04207265	IMs
TMCC31	2.7566E-06	1.05926637	0.16	0.118	0.0423712	IMs
ATP10D	2.7593E-06	0.68558985	0.28	0.237	0.04241248	IMs
DLL1	2.7756E-06	2.50401931	0.023	0.009	0.0426642	IMs
TP53INP1	2.8444E-06	0.71856626	0.226	0.179	0.04372084	IMs
RCN1	2.8847E-06	0.54164812	0.475	0.464	0.0443408	IMs
PDE2A	2.9074E-06	2.73184581	0.012	0.003	0.04468954	IMs
HEATR3	2.9647E-06	0.46919038	0.521	0.518	0.04557097	IMs

USP34	2.9708E-06	0.28024219	0.584	0.591	0.04566357	IMs
UBR2	3.0306E-06	0.23699247	0.685	0.717	0.046584	IMs
GABARAP1	3.1601E-06	0.19837146	0.887	0.894	0.04857458	IMs
BLOC1S6	3.1609E-06	0.65597884	0.415	0.395	0.0485869	IMs
GLUD1	3.1808E-06	0.34291931	0.621	0.647	0.04889275	IMs
SIAH2	3.2234E-06	0.61973954	0.367	0.336	0.04954675	IMs
SLC25A39	3.2595E-06	0.28111418	0.752	0.794	0.05010161	IMs
MAP4K4	3.3352E-06	0.65887811	0.447	0.431	0.05126545	IMs
CCDC93	3.3455E-06	0.78216901	0.255	0.208	0.05142358	IMs
TRAK1	3.3954E-06	0.41042248	0.603	0.609	0.05219091	IMs
SP3	3.4448E-06	0.24271755	0.713	0.751	0.05294963	IMs
IQGAP1	3.4926E-06	0.22790572	0.802	0.845	0.0536851	IMs
RBM14	3.5558E-06	0.29702759	0.537	0.529	0.05465603	IMs
PRDM8	3.6465E-06	0.46634808	0.012	0.003	0.05605092	IMs
WDR11	3.9232E-06	0.53339261	0.453	0.443	0.06030295	IMs
TMEM150A1	3.9559E-06	0.64623226	0.38	0.346	0.06080561	IMs
ERC1	3.9732E-06	0.61724556	0.354	0.322	0.06107277	IMs
SETD1B	4.0342E-06	0.2976523	0.593	0.608	0.06201024	IMs
HIST1H1E	4.1344E-06	0.55996365	0.331	0.29	0.06354911	IMs
TRPV1	4.3261E-06	1.13132133	0.067	0.039	0.06649651	IMs
DDX17	4.4705E-06	0.28818459	0.569	0.579	0.06871563	IMs
MAFG	4.5715E-06	0.69286811	0.347	0.31	0.07026802	IMs
ZNF672	4.5747E-06	0.70229992	0.356	0.328	0.07031828	IMs
INTS4	4.6888E-06	0.31778404	0.507	0.505	0.07207213	IMs
SCLT1	4.8475E-06	0.77250311	0.236	0.19	0.07451103	IMs
TRIP10	5.003E-06	0.82034296	0.327	0.292	0.07690099	IMs
PHKB	5.0313E-06	0.47896006	0.473	0.459	0.07733582	IMs
ARMCX11	5.1843E-06	0.84162985	0.232	0.188	0.07968742	IMs
ADGRE5	5.2627E-06	0.31775971	0.956	0.977	0.08089361	IMs
GDPD1	5.2678E-06	1.34061793	0.037	0.017	0.08097109	IMs
SLC25A34	5.3573E-06	1.53811498	0.026	0.011	0.08234778	IMs
SH2B3	5.6225E-06	0.28885214	0.767	0.816	0.08642421	IMs
CHMP4A	5.9293E-06	0.34743726	0.606	0.629	0.09113971	IMs
NIPSNAP3B	5.9509E-06	1.42508686	0.052	0.028	0.09147117	IMs
KIF17	5.9766E-06	1.35889175	0.03	0.013	0.09186658	IMs
SIK3	6.0151E-06	0.55582544	0.342	0.305	0.09245744	IMs
EIF4E2	6.034E-06	0.27623032	0.747	0.796	0.09274869	IMs
ZSWIM8	6.1783E-06	0.46280056	0.509	0.513	0.09496688	IMs
RNF44	6.256E-06	0.34809394	0.595	0.615	0.09616143	IMs
KMT2B	6.3044E-06	0.32186766	0.487	0.471	0.09690554	IMs
AGTPBP1	6.3653E-06	0.38710437	0.541	0.534	0.09784042	IMs
MYADM	6.369E-06	0.51243817	0.864	0.875	0.09789822	IMs
ANKRD13B	6.3856E-06	2.01394413	0.02	0.007	0.0981526	IMs
CD1631	6.4063E-06	0.10761505	0.793	0.916	0.09847108	IMs
PLD6	6.7018E-06	0.99664167	0.038	0.018	0.10301294	IMs
CRACR2A	6.7357E-06	0.2838235	0.112	0.075	0.10353462	IMs
GLA	6.8571E-06	0.48328883	0.607	0.63	0.10540119	IMs
MIDN	6.8644E-06	0.35140683	0.726	0.759	0.10551323	IMs
LYN1	6.9431E-06	0.28925877	0.776	0.826	0.10672205	IMs
RBM19	6.9483E-06	0.47990346	0.428	0.402	0.10680207	IMs
FBXL8	6.9634E-06	0.65550902	0.086	0.054	0.10703516	IMs
RNPC3	7.1087E-06	0.79108989	0.204	0.162	0.10926731	IMs

OVGP1	7.3362E-06	1.25653465	0.01	0.003	0.11276528	IMs
TCEA2	7.501E-06	1.02969569	0.124	0.088	0.11529715	IMs
CNDP21	7.7118E-06	0.16482647	0.941	0.941	0.11853869	IMs
INTS11	8.0092E-06	0.33616452	0.57	0.574	0.12310963	IMs
SLC25A4	8.2332E-06	0.75287901	0.026	0.011	0.12655241	IMs
SERTAD2	8.3912E-06	0.51504796	0.599	0.628	0.12898185	IMs
STX6	8.5628E-06	0.27373079	0.797	0.848	0.13161936	IMs
ATF6	8.9375E-06	0.37622845	0.605	0.644	0.13737782	IMs
NT5DC3	9.081E-06	1.06101977	0.098	0.065	0.13958426	IMs
PGGHG	9.4438E-06	0.4293106	0.099	0.065	0.14516048	IMs
EFCAB13	9.5403E-06	0.97634757	0.123	0.086	0.14664389	IMs
GDPD3	9.5664E-06	1.13239446	0.022	0.009	0.14704533	IMs
EPRS	9.6569E-06	0.3310257	0.658	0.687	0.14843662	IMs
VSIG10L	9.7174E-06	1.27526147	0.053	0.03	0.14936565	IMs
SLK	1.0015E-05	0.39256311	0.548	0.559	0.15394407	IMs
MT-ND5	1.0031E-05	0.47036018	0.323	0.279	0.15418805	IMs
UNC93B11	1.0057E-05	0.20180543	0.879	0.918	0.15458156	IMs
LILRA61	1.0135E-05	0.79441344	0.271	0.23	0.15578594	IMs
SMIM5	1.0273E-05	1.34072515	0.025	0.01	0.15790873	IMs
PRR7	1.029E-05	1.37764209	0.017	0.006	0.15816832	IMs
C8orf37	1.0486E-05	1.4799823	0.048	0.026	0.16117836	IMs
METTL1	1.0507E-05	1.00718283	0.221	0.18	0.16150788	IMs
TMEM255B1	1.0514E-05	1.0849909	0.203	0.161	0.16161732	IMs
MYBL1	1.0522E-05	0.57485834	0.039	0.019	0.16172995	IMs
SOCS1	1.0537E-05	0.81374648	0.374	0.345	0.16197049	IMs
UBE2D1	1.0736E-05	0.50962142	0.512	0.509	0.16502329	IMs
CCDC69	1.0939E-05	0.46608625	0.337	0.299	0.16814404	IMs
TPBGL	1.0987E-05	1.53843459	0.044	0.023	0.1688776	IMs
SLC7A6	1.1007E-05	0.56084891	0.313	0.272	0.16918114	IMs
GNPDA11	1.103E-05	0.62570118	0.419	0.413	0.16954611	IMs
USP48	1.118E-05	0.44204131	0.451	0.431	0.17184966	IMs
EBLN2	1.1324E-05	0.83490531	0.123	0.087	0.17406347	IMs
KDM5C	1.15E-05	0.33439706	0.605	0.621	0.17676525	IMs
CBR3	1.2092E-05	1.31834332	0.05	0.027	0.18587287	IMs
CCSER2	1.2183E-05	0.23903167	0.485	0.478	0.18725983	IMs
GSE1	1.2442E-05	0.44698618	0.451	0.435	0.19124904	IMs
AKAP13	1.319E-05	0.18553927	0.855	0.892	0.20274816	IMs
DNLZ	1.3276E-05	1.32029283	0.02	0.008	0.20406114	IMs
PSMD13	1.368E-05	0.27023502	0.669	0.695	0.21027728	IMs
CNEP1R1	1.3893E-05	0.46874215	0.507	0.503	0.21355307	IMs
PIP4P1	1.4288E-05	0.33134703	0.609	0.627	0.21961402	IMs
LRRC251	1.5103E-05	0.41976809	0.622	0.659	0.23214752	IMs
CDS1	1.5257E-05	1.27217197	0.09	0.059	0.23451361	IMs
SLC35D2	1.592E-05	0.53381894	0.392	0.368	0.24470669	IMs
SMPD1	1.6065E-05	0.5810265	0.345	0.315	0.24693543	IMs
ANKRD17	1.6593E-05	0.31225239	0.62	0.645	0.25505465	IMs
INPP5A	1.6626E-05	0.99580278	0.084	0.054	0.25555148	IMs
FOXN2	1.6934E-05	0.49187685	0.515	0.512	0.2602971	IMs
CHTOP	1.7141E-05	0.28839726	0.615	0.636	0.26346724	IMs
SPTY2D1OS	1.7308E-05	1.52042833	0.026	0.011	0.26604882	IMs
TRABD2A	1.7812E-05	0.25207534	0.012	0.003	0.27378966	IMs
SLC66A3	1.82E-05	0.34759917	0.634	0.66	0.27975024	IMs

VTI1A	1.8207E-05	0.46378211	0.38	0.35	0.27985726	IMs
ITGA10	1.8261E-05	1.80824577	0.012	0.003	0.2806869	IMs
GALNT1	1.877E-05	0.35080617	0.588	0.607	0.28851032	IMs
HNRNPA2B1	1.9201E-05	0.16321002	0.905	0.92	0.29514534	IMs
PRKCA	1.9473E-05	1.0645194	0.128	0.092	0.29932329	IMs
CSRP2	1.9922E-05	1.73909963	0.042	0.022	0.3062208	IMs
ATP6V1C1	2.0827E-05	0.43752238	0.594	0.617	0.32013007	IMs
SLAMF9	2.0873E-05	1.73049102	0.025	0.011	0.32084171	IMs
UBXN7	2.1475E-05	0.53024206	0.324	0.285	0.33009075	IMs
PARP11	2.1985E-05	0.76647532	0.191	0.151	0.33793696	IMs
MRTFA	2.2415E-05	0.32137184	0.688	0.733	0.34453387	IMs
STARD9	2.243E-05	0.72330902	0.047	0.026	0.34477673	IMs
KRTCAP3	2.2502E-05	1.08254037	0.038	0.019	0.34587993	IMs
ARL5B	2.2641E-05	1.07697467	0.158	0.121	0.34801688	IMs
PPP1R12A	2.2836E-05	0.2724947	0.623	0.643	0.35101213	IMs
SLC4A5	2.3431E-05	1.34859655	0.016	0.006	0.36015603	IMs
CELF6	2.3588E-05	0.83558431	0.083	0.053	0.36257566	IMs
WIPI1	2.3659E-05	0.69813495	0.319	0.284	0.3636678	IMs
SRCAP	2.4349E-05	0.31191443	0.616	0.645	0.37426142	IMs
BHLHE40	2.5469E-05	0.29443052	0.903	0.926	0.39148756	IMs
DOC2A	2.5783E-05	1.3824175	0.023	0.01	0.3963053	IMs
MOV10	2.6115E-05	0.36595127	0.578	0.585	0.40140654	IMs
ADCY4	2.6537E-05	1.19710852	0.046	0.025	0.40789362	IMs
HNRNPU	2.6829E-05	0.19519516	0.76	0.802	0.41239123	IMs
LACTB1	2.6896E-05	0.38289829	0.639	0.67	0.41341851	IMs
CLEC1A	2.8596E-05	0.98322451	0.115	0.081	0.43955016	IMs
SLC38A9	2.9274E-05	0.72089614	0.268	0.232	0.44997359	IMs
MGAT2	2.9364E-05	0.28707505	0.64	0.676	0.4513577	IMs
CCL28	2.9416E-05	1.15500788	0.022	0.009	0.45215289	IMs
CDC42EP2	2.9556E-05	1.25187348	0.088	0.058	0.45430509	IMs
HIST3H2A	2.9608E-05	1.13947751	0.04	0.021	0.45510139	IMs
ASB9	3.0841E-05	1.27947252	0.068	0.042	0.47405425	IMs
ELL	3.1471E-05	0.64513589	0.315	0.283	0.48374568	IMs
HCLS1	3.1593E-05	0.12502802	0.954	0.955	0.48562203	IMs
MMS19	3.3328E-05	0.49663732	0.367	0.339	0.51228774	IMs
SPEN	3.3628E-05	0.35447291	0.558	0.561	0.51690165	IMs
MTFP1	3.3819E-05	0.82201492	0.177	0.137	0.51983621	IMs
GRK6	3.4391E-05	0.4423456	0.537	0.544	0.52862556	IMs
LFNG	3.4587E-05	0.52466773	0.502	0.501	0.53164341	IMs
XPC	3.504E-05	0.5432987	0.425	0.412	0.538594	IMs
RRAGC	3.613E-05	0.41267016	0.521	0.528	0.55536133	IMs
PWWP3A	3.6198E-05	0.55220137	0.335	0.303	0.5564043	IMs
TBC1D9B	3.6287E-05	0.2197354	0.859	0.886	0.55776003	IMs
CKB	3.6328E-05	1.36856165	0.023	0.01	0.55840094	IMs
MB21D2	3.6347E-05	0.82143031	0.275	0.236	0.55869055	IMs
PHETA1	3.6579E-05	1.07684729	0.164	0.128	0.56225932	IMs
RSRC2	3.6918E-05	0.25760955	0.677	0.714	0.56746387	IMs
PLPP31	3.6989E-05	0.76914721	0.35	0.471	0.56856094	IMs
CPSF7	3.7919E-05	0.32695774	0.584	0.608	0.58285053	IMs
SMG1	3.7979E-05	0.63656776	0.185	0.145	0.58376812	IMs
TRIB11	3.8046E-05	0.68888831	0.519	0.526	0.58480265	IMs
MGAM	3.812E-05	1.69976664	0.019	0.007	0.58594529	IMs

TAS2R4	3.814E-05	1.0589473	0.041	0.022	0.58625214	IMs
HHIPL1	3.8832E-05	2.36567996	0.015	0.005	0.59688436	IMs
DACT1	3.8856E-05	2.71300909	0.01	0.003	0.59726083	IMs
ZNRF2	3.891E-05	0.32217154	0.647	0.677	0.59808382	IMs
IFI301	3.949E-05	0.20082414	1	0.982	0.60699691	IMs
SAFB	3.963E-05	0.2645109	0.643	0.677	0.6091548	IMs
CNPPD1	4.0196E-05	0.26920027	0.708	0.75	0.61784825	IMs
SET	4.0565E-05	0.39214786	0.549	0.565	0.62352365	IMs
CDV3	4.2112E-05	0.26800292	0.632	0.654	0.64730678	IMs
XYLB	4.3176E-05	1.30108353	0.063	0.038	0.66366506	IMs
HELZ2	4.3385E-05	0.74556822	0.328	0.3	0.66687119	IMs
HDAC3	4.3496E-05	0.59266956	0.384	0.36	0.66858358	IMs
GALK1	4.4471E-05	0.64852967	0.372	0.348	0.68356105	IMs
NPLOC4	4.484E-05	0.23159488	0.757	0.79	0.68922841	IMs
ZNF407	4.4997E-05	0.50951561	0.306	0.27	0.69165296	IMs
TNFRSF6B	4.5089E-05	1.84239801	0.039	0.02	0.6930616	IMs
AC091057.6	4.5758E-05	0.69752945	0.086	0.056	0.70334108	IMs
ABCD4	4.6065E-05	0.64472078	0.299	0.263	0.70806601	IMs
TFCP2	4.6445E-05	0.38940596	0.547	0.56	0.71389872	IMs
SLC12A8	4.6491E-05	1.49132199	0.04	0.021	0.71460996	IMs
LAPTM4B	4.6638E-05	1.40070307	0.027	0.013	0.71687088	IMs
NR2C2	4.6765E-05	0.44889637	0.38	0.351	0.71883145	IMs
ZNF644	4.7082E-05	0.26000872	0.582	0.6	0.7236917	IMs
C4orf47	4.7085E-05	1.01575275	0.01	0.003	0.7237423	IMs
SLC31A21	4.8123E-05	0.20072948	0.864	0.904	0.73970442	IMs
SYK1	4.843E-05	0.14645224	0.927	0.926	0.74441522	IMs
TEPSIN	4.9075E-05	0.72231314	0.175	0.136	0.75433424	IMs
CFP	4.9313E-05	0.22981078	0.2	0.275	0.75799723	IMs
MTA1	4.9314E-05	0.42893552	0.415	0.39	0.75800208	IMs
MADD	5.0545E-05	0.62047398	0.438	0.441	0.77692639	IMs
CCNYL1	5.0995E-05	0.44111282	0.53	0.541	0.78384353	IMs
ASMTL	5.1512E-05	0.90309261	0.171	0.135	0.79178771	IMs
PABPC4	5.1552E-05	0.46742942	0.55	0.57	0.79241217	IMs
PYROXD2	5.2435E-05	1.09437479	0.016	0.006	0.80597262	IMs
CBLN3	5.3669E-05	1.00271703	0.094	0.064	0.82493879	IMs
PPP1CB	5.4075E-05	0.2176727	0.783	0.823	0.831189	IMs
NUAK2	5.443E-05	1.07458797	0.162	0.126	0.83663711	IMs
WIPI2	5.4453E-05	0.3443876	0.579	0.607	0.83699874	IMs
MTG1	5.4501E-05	0.47834932	0.328	0.298	0.83772952	IMs
HELZ	5.5262E-05	0.33660845	0.414	0.394	0.84943512	IMs
FBXW2	5.5645E-05	0.33464652	0.608	0.626	0.85532059	IMs
PSEN1	5.5915E-05	0.22174161	0.763	0.807	0.85947556	IMs
PLCB2	5.6349E-05	0.25748169	0.797	0.839	0.86613553	IMs
XPOT	5.6925E-05	0.73094522	0.294	0.263	0.87499725	IMs
SSBP3	6.0442E-05	0.80150695	0.153	0.116	0.92905824	IMs
DENND6A	6.1717E-05	0.39308558	0.433	0.414	0.94865656	IMs
CLDN231	6.3164E-05	0.10701942	0.283	0.369	0.97088954	IMs
ST3GAL5	6.3403E-05	0.46676981	0.504	0.514	0.97456589	IMs
DDX5	6.4819E-05	0.10680783	0.929	0.941	0.99632927	IMs
SP100	6.6739E-05	0.19622804	0.76	0.809	1	IMs
CYSLTR21	6.6902E-05	0.99210814	0.161	0.125	1	IMs
KIAA0100	6.8519E-05	0.1990398	0.866	0.903	1	IMs

LARS	6.8615E-05	0.32463392	0.652	0.708	1	IMs
STXBP1	6.8953E-05	1.20043473	0.068	0.043	1	IMs
PNRC1	6.9069E-05	0.21955072	0.904	0.931	1	IMs
LYL1	6.9228E-05	0.87061283	0.274	0.24	1	IMs
ADORA2A	6.9805E-05	0.28040414	0.017	0.006	1	IMs
H3F3B	6.9882E-05	0.81014113	0.301	0.268	1	IMs
HIST1H2AE	7.0493E-05	1.26903838	0.037	0.019	1	IMs
PGM2	7.0732E-05	0.51162975	0.457	0.464	1	IMs
ZBTB7B	7.1169E-05	0.28938397	0.633	0.661	1	IMs
BEST11	7.1462E-05	0.48509373	0.368	0.333	1	IMs
CD80	7.151E-05	1.14796954	0.368	0.357	1	IMs
S100A8	7.1878E-05	0.32461725	0.66	0.845	1	IMs
PUM2	7.2884E-05	0.23903248	0.654	0.69	1	IMs
CCDC9	7.4299E-05	0.52858236	0.385	0.362	1	IMs
FASTKD1	7.6901E-05	0.52546794	0.262	0.221	1	IMs
MLLT10	7.6902E-05	0.43739269	0.362	0.335	1	IMs
SLC27A11	7.7132E-05	0.7439476	0.292	0.259	1	IMs
KLF13	7.8036E-05	0.39799217	0.476	0.477	1	IMs
MAOA	7.8501E-05	2.15340305	0.01	0.003	1	IMs
ZDHHC16	7.8691E-05	0.62170549	0.333	0.299	1	IMs
LOXL2	7.9761E-05	1.56297267	0.014	0.005	1	IMs
IARS	8.1115E-05	0.87389917	0.224	0.191	1	IMs
KLHL22	8.117E-05	0.61400943	0.251	0.215	1	IMs
FUT4	8.1421E-05	0.63659784	0.364	0.346	1	IMs
AHRR	8.2745E-05	2.65247011	0.015	0.005	1	IMs
SPTLC2	8.3525E-05	0.43361387	0.543	0.565	1	IMs
ARMH3	8.4405E-05	0.49348528	0.408	0.387	1	IMs
KLHL30	8.4826E-05	2.0440541	0.015	0.005	1	IMs
TM9SF3	8.6796E-05	0.16952902	0.804	0.842	1	IMs
IRF1	8.7849E-05	0.62252098	0.581	0.621	1	IMs
LCOR	8.9011E-05	0.33250276	0.546	0.547	1	IMs
PADI21	9.0712E-05	1.0722923	0.217	0.18	1	IMs
FJX1	9.1516E-05	1.87283453	0.02	0.008	1	IMs
ELF1	9.1807E-05	0.19034954	0.797	0.843	1	IMs
NXF1	9.2429E-05	0.42572428	0.479	0.477	1	IMs
PPP1R12B	9.254E-05	0.56764814	0.181	0.143	1	IMs
PUS1	9.297E-05	0.45269232	0.453	0.447	1	IMs
GOLT1B	9.3648E-05	0.32412185	0.587	0.614	1	IMs
TIA1	9.3758E-05	0.34527987	0.558	0.566	1	IMs
AVL9	9.4343E-05	0.37509086	0.456	0.439	1	IMs
TPM1	9.7135E-05	0.85590098	0.282	0.253	1	IMs
HNRNPUL1	9.7182E-05	0.18469238	0.782	0.834	1	IMs
MAP3K20	9.7357E-05	0.76349191	0.26	0.227	1	IMs
CAPG1	9.7846E-05	0.12031939	0.988	0.97	1	IMs
ASAP1	9.7974E-05	0.30551154	0.677	0.725	1	IMs
H2AFY2	9.9097E-05	0.93113422	0.128	0.095	1	IMs
AIMP2	9.9385E-05	0.48670105	0.433	0.42	1	IMs
AGPAT4	0.00010165	0.38004173	0.456	0.44	1	IMs
ADHFE1	0.00010241	0.9408051	0.032	0.016	1	IMs
CPOX	0.0001029	0.72601086	0.262	0.229	1	IMs
OXA1L	0.00010519	0.23920446	0.731	0.78	1	IMs
VAV3	0.00010766	0.70675004	0.23	0.192	1	IMs

DYNC1I2	0.00010855	0.19354373	0.781	0.832	1	IMs
PPAN	0.00010949	0.54559088	0.391	0.368	1	IMs
AEBP2	0.00010992	0.26333325	0.473	0.465	1	IMs
OTULIN	0.00011012	0.43655984	0.431	0.415	1	IMs
HNRNPH3	0.00011054	0.22890984	0.737	0.773	1	IMs
UBE4B	0.00011343	0.38081997	0.46	0.451	1	IMs
ANKAR	0.00011345	1.37683182	0.046	0.026	1	IMs
OGFRL1	0.00011436	0.50744322	0.617	0.669	1	IMs
NFATC1	0.00011682	0.59400145	0.346	0.321	1	IMs
SLC39A14	0.00011702	1.25058597	0.036	0.019	1	IMs
GLRX3	0.00011796	0.28132795	0.593	0.611	1	IMs
FAM43A	0.00011939	0.62327072	0.046	0.026	1	IMs
KMT5B	0.00012455	0.34807949	0.535	0.54	1	IMs
ERMN	0.00012573	1.02429219	0.025	0.012	1	IMs
DMXL1	0.00012671	0.44692415	0.453	0.447	1	IMs
GSDMB	0.00012806	0.36246672	0.039	0.021	1	IMs
SLC15A4	0.0001302	0.36261632	0.629	0.644	1	IMs
FAM91A1	0.00013069	0.35914122	0.553	0.575	1	IMs
SHROOM1	0.00013097	1.78225271	0.032	0.017	1	IMs
ZNF121	0.00013404	0.53165377	0.357	0.325	1	IMs
PRNP	0.00013471	0.23522491	0.78	0.843	1	IMs
ARPIN	0.00013557	0.38622915	0.61	0.627	1	IMs
CCT6A	0.00013582	0.29048873	0.611	0.646	1	IMs
KLF16	0.00013599	0.48266533	0.43	0.415	1	IMs
AGO4	0.0001368	0.44173896	0.46	0.455	1	IMs
VNN11	0.00014051	1.05743781	0.053	0.032	1	IMs
YTHDC2	0.00014089	0.46713174	0.35	0.324	1	IMs
AP3B1	0.0001435	0.31975013	0.626	0.67	1	IMs
DNHD1	0.00014521	0.64644729	0.113	0.082	1	IMs
ITLN1	0.00014776	1.03117101	0.031	0.016	1	IMs
PDK4	0.00015115	0.58073556	0.662	0.723	1	IMs
IL17C	0.0001529	1.97882618	0.01	0.003	1	IMs
SIRT1	0.00015746	0.57781133	0.258	0.222	1	IMs
NAA15	0.00015749	0.37449199	0.499	0.513	1	IMs
TRIP4	0.00016124	0.38978012	0.423	0.412	1	IMs
ETV7	0.00016299	1.61841616	0.023	0.011	1	IMs
RFX2	0.00016352	0.90749672	0.195	0.16	1	IMs
UBE2Z	0.00016686	0.29118605	0.641	0.687	1	IMs
IDH11	0.00017069	0.33631241	0.745	0.829	1	IMs
SYMPK	0.00017458	0.33457996	0.607	0.634	1	IMs
CCDC1701	0.00017714	0.88686002	0.219	0.187	1	IMs
SLC22A18AS1	0.00017764	1.16332549	0.065	0.041	1	IMs
MOB3A	0.00017851	0.17059248	0.801	0.844	1	IMs
JPT2	0.00017886	0.49452943	0.354	0.331	1	IMs
ZSWIM6	0.00018079	0.47611755	0.49	0.498	1	IMs
EXOG	0.00018338	0.89102057	0.06	0.037	1	IMs
IFNGR11	0.00018344	0.25927852	0.863	0.9	1	IMs
RCC2	0.00018375	0.3676825	0.644	0.669	1	IMs
RNMT	0.00018578	0.47919578	0.363	0.339	1	IMs
AHSA1	0.00018854	0.23733742	0.726	0.773	1	IMs
ZNF791	0.00019056	0.85757582	0.205	0.172	1	IMs
VAC14	0.00019112	0.38054232	0.569	0.598	1	IMs

SLC25A19	0.00019261	0.46635515	0.571	0.608	1	IMs
OGG1	0.00019664	0.5753103	0.243	0.208	1	IMs
BX255925.3	0.00020378	0.77811335	0.105	0.074	1	IMs
NDUFA10	0.00020497	0.19834717	0.715	0.758	1	IMs
ATF4	0.00020586	0.5233918	0.53	0.572	1	IMs
SSH1	0.000207	0.57597746	0.436	0.443	1	IMs
MT-ND2	0.00020946	0.72237389	0.249	0.214	1	IMs
HDHD5	0.00021507	0.52106397	0.471	0.472	1	IMs
FAM133B	0.00021771	0.73789651	0.195	0.161	1	IMs
DPP9	0.00021901	0.25812654	0.671	0.71	1	IMs
ZNF154	0.00022213	1.09452771	0.03	0.016	1	IMs
EIF3A	0.00022245	0.18771318	0.839	0.874	1	IMs
DENND5B	0.00022284	1.65461038	0.051	0.031	1	IMs
PARP121	0.00022365	0.1713464	0.794	0.829	1	IMs
PANK3	0.00022443	0.52811337	0.44	0.444	1	IMs
ASS1	0.00022583	0.60271844	0.01	0.003	1	IMs
ZNF580	0.00022729	0.43705172	0.326	0.295	1	IMs
AIFM2	0.00022766	0.88522992	0.147	0.115	1	IMs
ZC3H12A	0.00022781	0.61735877	0.32	0.292	1	IMs
SECISBP2	0.00023066	0.45656233	0.389	0.373	1	IMs
MED14	0.00024269	0.41873191	0.455	0.445	1	IMs
RGS13	0.00024665	1.39525693	0.019	0.008	1	IMs
LILRB11	0.00024784	0.52007768	0.596	0.642	1	IMs
MITD1	0.00024884	0.57203076	0.264	0.232	1	IMs
ZWILCH	0.00025106	0.6642531	0.181	0.145	1	IMs
PIEZO1	0.00025365	0.46170051	0.488	0.497	1	IMs
CBFB	0.00025443	0.18373128	0.748	0.8	1	IMs
LRRC40	0.00025485	0.55667255	0.333	0.306	1	IMs
GYS1	0.00025615	0.71212618	0.293	0.263	1	IMs
CFB1	0.00025647	0.78612892	0.218	0.183	1	IMs
CMTM1	0.00025878	1.43968396	0.026	0.013	1	IMs
IGF2R	0.00026038	0.18654383	0.799	0.856	1	IMs
MAD1L1	0.00026463	0.5459429	0.404	0.392	1	IMs
ACIN1	0.00026572	0.42173438	0.439	0.436	1	IMs
RBM12B	0.00026745	0.52206215	0.25	0.214	1	IMs
UBE2E1	0.00026933	0.58798837	0.377	0.363	1	IMs
TUBA1C	0.00027083	0.4048577	0.531	0.559	1	IMs
CMTM6	0.00027435	0.18360804	0.853	0.879	1	IMs
CDC25B	0.00027874	0.44807784	0.311	0.28	1	IMs
POU5F2	0.00028277	0.6299638	0.069	0.045	1	IMs
NLGN2	0.00028949	0.93394556	0.014	0.005	1	IMs
TBL1XR1	0.00028966	0.30132109	0.624	0.67	1	IMs
PPM1D	0.00029708	0.79863202	0.226	0.194	1	IMs
CIC	0.00029822	0.37464312	0.546	0.556	1	IMs
LMF2	0.00030786	0.39606737	0.479	0.487	1	IMs
ISY1	0.00030792	0.32230761	0.568	0.598	1	IMs
MPP6	0.00030985	0.31058351	0.015	0.006	1	IMs
VCPKMT	0.0003147	0.564431	0.161	0.127	1	IMs
MTMR14	0.00032427	0.20481	0.746	0.805	1	IMs
MARCH7	0.00032972	0.14962771	0.768	0.816	1	IMs
CPNE2	0.00032993	0.57355402	0.322	0.297	1	IMs
CATSPER2	0.00033331	1.15123958	0.054	0.034	1	IMs

SHLD2	0.00033612	0.35499657	0.518	0.528	1	IMs
SPG7	0.00033969	0.42315689	0.473	0.48	1	IMs
TRPM41	0.00034015	0.6434885	0.274	0.241	1	IMs
CYB561A3	0.00034051	0.52875705	0.457	0.463	1	IMs
SEPTIN7	0.00034302	0.35011451	0.454	0.456	1	IMs
HYOU1	0.00034347	0.4016415	0.621	0.691	1	IMs
SYNGAP1	0.00034363	0.47689786	0.162	0.128	1	IMs
ERF	0.00034665	0.55289294	0.351	0.332	1	IMs
PRKD3	0.00035115	0.4934996	0.369	0.353	1	IMs
FBRSL1	0.00036171	0.51288767	0.349	0.33	1	IMs
FRMD8	0.00036476	0.80440378	0.075	0.051	1	IMs
RCOR3	0.00036846	0.46716633	0.299	0.269	1	IMs
S100Z	0.0003791	1.49851693	0.033	0.018	1	IMs
JMJD1C	0.00038807	0.20859797	0.723	0.761	1	IMs
DEPP1	0.00039361	2.11109221	0.013	0.005	1	IMs
CTDSP1	0.00039552	0.11245546	0.73	0.771	1	IMs
SLC38A2	0.00039756	0.48052252	0.622	0.665	1	IMs
HOXB3	0.00040252	0.53071337	0.043	0.025	1	IMs
CLCN5	0.00040295	0.56328449	0.315	0.288	1	IMs
PAFAH1B1	0.00040525	0.18022571	0.793	0.833	1	IMs
ATL2	0.0004154	0.69024153	0.252	0.22	1	IMs
GDPD5	0.00042554	0.91759387	0.048	0.029	1	IMs
KPNA4	0.00043741	0.33958181	0.576	0.594	1	IMs
CTBP2	0.00044308	0.24861217	0.73	0.77	1	IMs
ZNF562	0.00045148	0.48641633	0.346	0.321	1	IMs
KDM5D	0.00045241	0.3306633	0.194	0.16	1	IMs
THADA	0.00045582	0.47533004	0.327	0.305	1	IMs
SMCHD1	0.00045609	0.20935757	0.49	0.49	1	IMs
SLC38A6	0.00045756	0.57179381	0.368	0.351	1	IMs
CEMIP2	0.0004829	0.63202228	0.251	0.222	1	IMs
CHKB	0.00048996	0.481927	0.324	0.298	1	IMs
PPP1R32	0.00049027	1.08893822	0.016	0.007	1	IMs
PTPN2	0.00049546	0.53050381	0.397	0.387	1	IMs
ZNF785	0.00050139	0.82590191	0.105	0.077	1	IMs
GDAP1	0.00050753	0.71703715	0.154	0.121	1	IMs
JAZF1	0.00052439	0.47392394	0.431	0.433	1	IMs
AS3MT	0.00053004	1.35966576	0.013	0.005	1	IMs
COG3	0.00053221	0.4781953	0.319	0.291	1	IMs
MAPK3	0.00053223	0.47149071	0.405	0.392	1	IMs
CD2AP	0.00054033	0.49324331	0.334	0.309	1	IMs
HDX1	0.000542	0.98700682	0.067	0.045	1	IMs
DUSP10	0.00054274	0.51060986	0.556	0.583	1	IMs
ITGB1BP2	0.00054688	1.33741229	0.013	0.005	1	IMs
SLC16A1	0.00055704	0.67388218	0.262	0.231	1	IMs
ELMO3	0.00055835	1.11788094	0.038	0.022	1	IMs
CASTOR1	0.00056635	0.91146399	0.067	0.045	1	IMs
FLACC1	0.00058863	1.25964079	0.027	0.014	1	IMs
GLS	0.00059045	0.47194502	0.379	0.366	1	IMs
MBNL1	0.00061705	0.18147547	0.862	0.905	1	IMs
TCP11L2	0.00061705	0.68670437	0.228	0.199	1	IMs
PUS7	0.00062196	0.69858066	0.198	0.167	1	IMs
KDM4B	0.0006223	0.46757087	0.448	0.454	1	IMs

CNN2	0.00062431	0.21153587	0.77	0.803	1	IMs
SMO	0.0006328	2.39702338	0.013	0.005	1	IMs
GANC	0.00064174	0.38500797	0.409	0.397	1	IMs
ITPRIP	0.00064841	0.53733918	0.441	0.448	1	IMs
CDKAL1	0.00065063	0.44650079	0.358	0.342	1	IMs
CIR1	0.000658	0.21188771	0.278	0.359	1	IMs
NR2C2AP	0.00066357	0.1036902	0.2	0.262	1	IMs
RUFY1	0.00066945	0.2201792	0.731	0.781	1	IMs
ZBED5	0.00067659	0.60133705	0.232	0.203	1	IMs
AFTPH	0.00068106	0.36900245	0.597	0.634	1	IMs
PMM2	0.00068563	0.44449805	0.377	0.36	1	IMs
BANP	0.00069546	1.25972	0.064	0.043	1	IMs
HACD2	0.00070331	0.5840016	0.332	0.311	1	IMs
SURF4	0.00071132	0.30225984	0.582	0.615	1	IMs
CLIP1	0.00071334	0.23037196	0.622	0.651	1	IMs
SRD5A1	0.00071613	0.82610852	0.191	0.163	1	IMs
NLRP12	0.00071664	1.03692985	0.175	0.146	1	IMs
FKBP151	0.00072381	0.19674788	0.754	0.809	1	IMs
MON2	0.00073283	0.41641749	0.393	0.377	1	IMs
FCAR	0.00073343	0.11240009	0.156	0.205	1	IMs
FAM120A	0.00073797	0.1268464	0.781	0.825	1	IMs
NCBP1	0.00074341	0.44373119	0.36	0.338	1	IMs
TIMP1	0.00074961	1.09109203	0.715	0.768	1	IMs
FGF11	0.00074966	1.61924053	0.018	0.008	1	IMs
NUDT161	0.00074991	0.44171605	0.507	0.535	1	IMs
KDM3B	0.00075056	0.29006418	0.575	0.604	1	IMs
AMH	0.00075458	2.11956793	0.01	0.004	1	IMs
TBC1D121	0.00075822	0.73172188	0.272	0.245	1	IMs
MORC2	0.00075948	0.52640841	0.286	0.256	1	IMs
WNT5A	0.00076526	2.18657344	0.018	0.008	1	IMs
ATXN2L	0.0007698	0.30172153	0.508	0.526	1	IMs
CSF2RB1	0.00078279	0.30960079	0.757	0.794	1	IMs
LTBR1	0.00079449	0.22126317	0.721	0.769	1	IMs
LCAT	0.00079667	0.98879439	0.05	0.032	1	IMs
SLC25A32	0.00081703	0.47608781	0.368	0.354	1	IMs
XPO1	0.00084386	0.41836213	0.395	0.389	1	IMs
IL20RB	0.00084889	1.03475928	0.037	0.021	1	IMs
ATXN7	0.00085507	0.34296627	0.392	0.38	1	IMs
M6PR	0.0008601	0.12145282	0.933	0.947	1	IMs
FKBP11	0.00086551	0.43192474	0.143	0.113	1	IMs
PCNX4	0.00090921	0.58330471	0.175	0.145	1	IMs
PLAUR1	0.00091037	0.31931208	0.958	0.96	1	IMs
SLC22A4	0.00091395	0.96076902	0.132	0.104	1	IMs
CAMTA2	0.00091846	0.42535284	0.394	0.38	1	IMs
PML	0.00091926	0.33620008	0.658	0.71	1	IMs
LAMC1	0.0009276	0.81980904	0.079	0.056	1	IMs
CUBN	0.0009383	1.06239504	0.017	0.007	1	IMs
ATM	0.00095574	0.23526705	0.603	0.632	1	IMs
DNAH1	0.00097938	0.6431507	0.113	0.086	1	IMs
BAZ2B	0.0009832	0.38679817	0.531	0.551	1	IMs
CCDC149	0.00099276	0.88486851	0.158	0.128	1	IMs
HSPH1	0.00099709	0.22170231	0.645	0.765	1	IMs

MT-CYB	0.00100273	0.30628101	0.264	0.232	1	IMs
CISD2	0.00101596	0.37670692	0.523	0.542	1	IMs
MFSD4B	0.00102285	0.72022857	0.179	0.149	1	IMs
TAF1D	0.0010232	0.40019197	0.487	0.489	1	IMs
EAF2	0.00103514	0.77030936	0.21	0.182	1	IMs
NR4A31	0.00106892	0.96176563	0.483	0.48	1	IMs
PPIB	0.00114076	0.31766676	0.631	0.679	1	IMs
PHF11	0.00120773	0.28277831	0.549	0.565	1	IMs
MYLIP	0.0012471	0.89380433	0.161	0.131	1	IMs
GPC2	0.00125633	1.80670496	0.01	0.004	1	IMs
GNRH1	0.00126773	0.93061739	0.022	0.011	1	IMs
AMOT	0.00127613	1.73901656	0.014	0.006	1	IMs
TTC31	0.00127752	0.54996251	0.302	0.28	1	IMs
CCDC7	0.0012785	0.54906056	0.154	0.125	1	IMs
GUCA1B	0.00128014	1.33425978	0.021	0.01	1	IMs
CCDC97	0.00128029	0.38723681	0.471	0.477	1	IMs
ARHGEF10	0.00128186	1.48089736	0.024	0.012	1	IMs
BIRC6	0.0012973	0.21157024	0.582	0.6	1	IMs
DHDDS	0.0013144	0.45411581	0.43	0.431	1	IMs
SESN11	0.00132929	0.19596096	0.322	0.406	1	IMs
CDC16	0.00133523	0.2963264	0.549	0.574	1	IMs
RABEP1	0.00133674	0.30862722	0.55	0.571	1	IMs
TUBA1A1	0.00137458	0.65945342	0.316	0.299	1	IMs
RNF24	0.00139847	1.01541853	0.112	0.086	1	IMs
MCM3AP	0.00142032	0.42797335	0.425	0.421	1	IMs
ZCCHC14	0.00143398	0.64632122	0.267	0.241	1	IMs
SLC22A151	0.00147182	0.5743941	0.343	0.322	1	IMs
SAMD1	0.00153418	0.49762789	0.393	0.376	1	IMs
CLDND1	0.00154332	0.33964176	0.486	0.502	1	IMs
TMEM130	0.0015465	2.02409014	0.01	0.004	1	IMs
ADM5	0.00155733	0.98923318	0.015	0.006	1	IMs
PNP	0.00156063	0.1185255	0.624	0.746	1	IMs
DND1	0.00157857	1.05023817	0.026	0.014	1	IMs
LRRC69	0.00158158	0.82786774	0.05	0.032	1	IMs
PPP1R3B	0.00158562	0.82297434	0.218	0.191	1	IMs
SMIM4	0.0015943	1.04509125	0.073	0.052	1	IMs
SIKE1	0.00159666	0.41611281	0.366	0.352	1	IMs
PRR14	0.00162322	0.36513282	0.427	0.424	1	IMs
MIOS	0.00164507	0.4518839	0.354	0.337	1	IMs
LXN	0.00165902	0.80193536	0.19	0.161	1	IMs
CCL15	0.00166005	1.4135024	0.015	0.006	1	IMs
UQCRC1	0.00166359	0.15057355	0.804	0.849	1	IMs
SLC35B2	0.00167677	0.59429138	0.386	0.378	1	IMs
INPP5B	0.00168261	0.4388606	0.453	0.456	1	IMs
GOLIM4	0.00169752	0.23977395	0.748	0.794	1	IMs
CLEC4D	0.00172713	0.87239951	0.166	0.138	1	IMs
GTF2F2	0.00173248	0.4875238	0.331	0.315	1	IMs
PPP3CA	0.0017544	0.3247099	0.543	0.569	1	IMs
FNBP1	0.00175474	0.10185925	0.695	0.739	1	IMs
CYREN	0.00177749	0.50184328	0.39	0.385	1	IMs
PACS1	0.00182678	0.2946213	0.567	0.592	1	IMs
ZNF593	0.0018289	0.14817662	0.215	0.28	1	IMs

BTAF1	0.0018486	0.24555098	0.418	0.413	1	IMs
PGS1	0.00186034	0.41592111	0.366	0.352	1	IMs
TRIM26	0.00187511	0.30740547	0.582	0.617	1	IMs
MARK3	0.00187527	0.25489383	0.464	0.465	1	IMs
PSMB1	0.00188345	0.14363374	0.85	0.886	1	IMs
ARL15	0.00189339	0.57259004	0.331	0.315	1	IMs
RHEBL1	0.00193294	1.27189963	0.046	0.029	1	IMs
AP2S1	0.00198619	0.1502862	0.753	0.817	1	IMs
HMGN5	0.00199587	0.10510199	0.051	0.081	1	IMs
ZNF577	0.00202218	0.88086181	0.092	0.069	1	IMs
SERPINF2	0.00206721	1.12618344	0.107	0.083	1	IMs
SUMO4	0.00210041	0.81942064	0.024	0.013	1	IMs
ZNF793	0.00210381	1.32957665	0.01	0.004	1	IMs
KANSL1	0.00215224	0.1632139	0.606	0.638	1	IMs
ATXN1	0.00215697	0.2467072	0.583	0.619	1	IMs
INPP1	0.00216048	0.61827115	0.224	0.198	1	IMs
ZNF236	0.00221185	0.50669888	0.237	0.212	1	IMs
FOXRED2	0.00222342	0.74961199	0.127	0.1	1	IMs
BCL10	0.00226414	0.28385595	0.597	0.626	1	IMs
CNOT8	0.00227789	0.29037305	0.611	0.653	1	IMs
CBFA2T2	0.00228992	0.4224601	0.31	0.291	1	IMs
UBE2D3	0.00230148	0.15711162	0.756	0.811	1	IMs
HSPD1	0.00230333	0.15772249	0.481	0.593	1	IMs
MIA	0.00230638	1.15293298	0.013	0.005	1	IMs
BZW2	0.0023341	0.56284571	0.412	0.414	1	IMs
NEMP1	0.00236683	0.56426592	0.265	0.238	1	IMs
CPPED11	0.00241144	0.27382664	0.694	0.739	1	IMs
FSTL3	0.00241287	0.90489814	0.061	0.042	1	IMs
PRKCE	0.0024464	0.10366827	0.282	0.35	1	IMs
CBLB	0.00245455	0.31663915	0.282	0.259	1	IMs
ASGR1	0.00247858	0.75884179	0.191	0.166	1	IMs
UBALD2	0.00248137	0.41691024	0.318	0.301	1	IMs
KCNH3	0.00251751	1.07222421	0.018	0.009	1	IMs
IQCN	0.00253562	0.70548625	0.041	0.026	1	IMs
NDEL1	0.00255198	0.32798677	0.538	0.561	1	IMs
SIL11	0.00259245	0.31073086	0.553	0.584	1	IMs
TTLL4	0.00261099	0.57164499	0.227	0.199	1	IMs
WDFY41	0.00262261	0.23527898	0.671	0.714	1	IMs
INO80D	0.0026277	0.44251531	0.295	0.271	1	IMs
U2AF1L4	0.00263146	0.5090301	0.283	0.26	1	IMs
ZNF26	0.00264508	0.51445282	0.203	0.175	1	IMs
MFAP51	0.00265608	0.78652921	0.059	0.04	1	IMs
S100PBP	0.00266084	0.31163223	0.365	0.348	1	IMs
PLCD1	0.0026617	0.10482178	0.173	0.224	1	IMs
FAM160A2	0.00268621	0.46072412	0.374	0.366	1	IMs
AIMP1	0.00271991	0.20436517	0.618	0.647	1	IMs
ZP3	0.00272266	1.32066997	0.021	0.011	1	IMs
KCND1	0.00272317	1.31250573	0.014	0.006	1	IMs
RNF131	0.00272562	0.18798047	0.842	0.886	1	IMs
BEND3	0.00273922	1.14865552	0.037	0.022	1	IMs
DDX39B	0.00276434	0.50327981	0.372	0.363	1	IMs
MBNL2	0.00277759	0.5107547	0.317	0.298	1	IMs

ATG9A	0.0027977	0.56371363	0.254	0.233	1	IMs
NAPA	0.00281798	0.17030691	0.851	0.906	1	IMs
CEP135	0.00282954	0.61675701	0.21	0.185	1	IMs
TAPBP	0.00286485	0.20661377	0.93	0.961	1	IMs
CLASP2	0.0028888	0.40644796	0.337	0.323	1	IMs
MAP10	0.00290126	0.87742248	0.056	0.039	1	IMs
MAP3K21	0.00290791	0.93666354	0.077	0.057	1	IMs
AL160269.1	0.00303103	1.54913035	0.013	0.005	1	IMs
WASF1	0.00303476	1.8297757	0.013	0.005	1	IMs
KIFC2	0.0030436	0.61996792	0.136	0.11	1	IMs
DYNC1H1	0.00305994	0.43354931	0.388	0.379	1	IMs
EDA1	0.003068	0.11563313	0.058	0.086	1	IMs
CCL4	0.00310401	0.36205873	0.078	0.057	1	IMs
FADS2	0.00313907	0.33116734	0.334	0.298	1	IMs
TRRAP	0.00314021	0.26254721	0.495	0.505	1	IMs
MFSD5	0.00317019	0.41370647	0.432	0.44	1	IMs
LSP1	0.00318125	0.16596629	0.926	0.945	1	IMs
RELL2	0.00318274	1.00584334	0.081	0.06	1	IMs
SFXN1	0.0032067	0.2297161	0.159	0.131	1	IMs
BPTF	0.00321922	0.21208276	0.546	0.566	1	IMs
ARHGEF2	0.00322149	0.15237626	0.802	0.857	1	IMs
ACAP3	0.00322248	0.55226719	0.304	0.284	1	IMs
UVRAG	0.00322958	0.53891077	0.388	0.398	1	IMs
C6orf163	0.00326771	0.84373934	0.041	0.026	1	IMs
EXOC4	0.00329412	0.36816771	0.437	0.444	1	IMs
ATRN	0.00330581	0.4421193	0.444	0.447	1	IMs
YKT6	0.00330588	0.19124405	0.643	0.693	1	IMs
MYCBP2	0.0033584	0.40452642	0.365	0.357	1	IMs
RRP1	0.00336845	0.52570848	0.387	0.38	1	IMs
LGALS8	0.00337673	0.21287343	0.651	0.706	1	IMs
TCTEX1D2	0.00341915	0.87797167	0.053	0.036	1	IMs
SLC12A4	0.00344808	0.547208	0.303	0.285	1	IMs
METTL21A	0.00349372	0.66318459	0.266	0.244	1	IMs
C5AR21	0.0034964	0.21615486	0.347	0.442	1	IMs
TRIM66	0.00350599	0.71908128	0.075	0.055	1	IMs
NR1H31	0.0035096	0.66802505	0.46	0.618	1	IMs
TCF25	0.00352527	0.13483767	0.791	0.843	1	IMs
GPC41	0.00354458	1.57188506	0.094	0.072	1	IMs
NAT14	0.00354819	0.9774702	0.06	0.041	1	IMs
FKBP1B	0.0035495	1.16569421	0.056	0.039	1	IMs
KCTD9	0.0035923	0.46868752	0.302	0.282	1	IMs
ANKRD12	0.00363169	0.31701617	0.394	0.388	1	IMs
POC5	0.00367709	0.14125913	0.09	0.126	1	IMs
ADK	0.00377433	0.52754354	0.339	0.329	1	IMs
SCML1	0.0037976	0.62304546	0.217	0.192	1	IMs
PHLPP1	0.00380133	0.97167483	0.082	0.061	1	IMs
IRAK2	0.00380553	1.69410085	0.067	0.048	1	IMs
XXYLT1	0.00383061	0.77136693	0.153	0.127	1	IMs
ARMT1	0.00387979	0.70015432	0.289	0.279	1	IMs
SNRPD3	0.00388362	1.32728045	0.014	0.006	1	IMs
KIF5B	0.00389735	0.13354085	0.85	0.881	1	IMs
IFITM10	0.00413457	0.17859003	0.418	0.52	1	IMs

WDFY31	0.00413627	0.47855413	0.408	0.405	1	IMs
PHKG1	0.00419273	1.68651166	0.014	0.006	1	IMs
ACTRT3	0.00419811	1.51323555	0.024	0.013	1	IMs
PHF23	0.00423696	0.40154315	0.402	0.398	1	IMs
PCK21	0.00423832	0.2363418	0.692	0.738	1	IMs
VIM	0.0042431	0.12740408	0.999	0.998	1	IMs
MAP2K6	0.00425	0.72333943	0.092	0.07	1	IMs
CALCRL	0.00429295	1.12707051	0.039	0.024	1	IMs
METTL9	0.00430027	0.36406023	0.471	0.483	1	IMs
MARS	0.0043469	0.69731767	0.186	0.162	1	IMs
UBE2B	0.0043885	0.21800414	0.742	0.796	1	IMs
GZF1	0.00439001	0.67645209	0.297	0.285	1	IMs
C12orf76	0.00440048	1.48223938	0.025	0.014	1	IMs
TNFRSF10D	0.00446117	1.07404656	0.077	0.057	1	IMs
CHRNA10	0.00447878	2.31428415	0.013	0.006	1	IMs
POPDC2	0.00451409	0.97589757	0.067	0.048	1	IMs
TAF11	0.00457383	0.44831853	0.35	0.336	1	IMs
HDAC4	0.00460394	0.51482775	0.278	0.261	1	IMs
ENTPD4	0.00467418	0.40247196	0.35	0.337	1	IMs
MYL5	0.00467803	0.85671753	0.107	0.084	1	IMs
ETV3	0.00478791	0.61070095	0.252	0.233	1	IMs
NCK1	0.00478893	0.26323517	0.676	0.746	1	IMs
ZNF669	0.00479108	0.83352925	0.063	0.045	1	IMs
DUSP16	0.00480807	0.52009549	0.196	0.17	1	IMs
DVL3	0.00481513	0.27848762	0.549	0.578	1	IMs
UPF3A	0.00482616	0.45872207	0.222	0.196	1	IMs
IRF51	0.00483679	0.39291253	0.472	0.485	1	IMs
BTBD9	0.00490208	0.29313665	0.317	0.3	1	IMs
DNM2	0.00492106	0.15074265	0.901	0.929	1	IMs
BLK	0.00493715	0.63070364	0.002	0.012	1	IMs
PRPF3	0.00494879	0.34713573	0.46	0.471	1	IMs
PLK21	0.00495238	0.13062467	0.138	0.178	1	IMs
NUTF2	0.00497771	0.43492212	0.49	0.512	1	IMs
NR3C1	0.00499938	0.18358425	0.758	0.82	1	IMs
FAM229A	0.0050403	1.16840902	0.015	0.007	1	IMs
TRAPPC6B	0.00504031	0.45130064	0.332	0.315	1	IMs
TLN2	0.00505144	1.26835876	0.054	0.038	1	IMs
FKRP	0.00506634	0.72044331	0.129	0.104	1	IMs
DHRSX	0.00506709	0.29783607	0.497	0.508	1	IMs
ZSCAN9	0.00513224	0.74913778	0.144	0.12	1	IMs
GXYLT1	0.00516776	0.64781143	0.184	0.16	1	IMs
ATAD3B	0.00517155	0.62127628	0.092	0.071	1	IMs
GRIN3A	0.00525344	1.73282046	0.018	0.009	1	IMs
EID3	0.00525697	0.71293711	0.079	0.059	1	IMs
TMEM70	0.00527489	0.65503668	0.367	0.369	1	IMs
DNAJB6	0.00534491	0.51934706	0.32	0.31	1	IMs
DHX34	0.00535922	0.50282348	0.246	0.223	1	IMs
USP36	0.00537792	0.31659275	0.38	0.364	1	IMs
PCGF3	0.00546288	0.44472239	0.328	0.312	1	IMs
NAA25	0.0055043	0.50756081	0.165	0.141	1	IMs
WWC21	0.00552831	0.13895048	0.176	0.223	1	IMs
PTGES2	0.00553944	0.4115256	0.455	0.462	1	IMs

PRXL2B	0.00554895	1.06100363	0.1	0.079	1	IMs
C9orf64	0.00557096	0.121199	0.337	0.411	1	IMs
PRRC2C	0.00557421	0.20686961	0.619	0.664	1	IMs
WBP4	0.00557609	0.11861988	0.249	0.315	1	IMs
ZMYM2	0.00561836	0.25770959	0.441	0.445	1	IMs
TRIM13	0.00564106	0.6597185	0.233	0.214	1	IMs
MYBPC3	0.00568024	0.95993119	0.038	0.024	1	IMs
ZZEF1	0.00570264	0.23909825	0.536	0.559	1	IMs
FAS	0.00572231	0.32935623	0.232	0.205	1	IMs
TMEM8A	0.00572317	0.32211019	0.59	0.646	1	IMs
GPR137B1	0.00576289	0.25167851	0.713	0.782	1	IMs
TMEM88	0.00588988	1.37675269	0.041	0.027	1	IMs
DCBLD1	0.00590552	0.75219292	0.112	0.089	1	IMs
NT5C2	0.0059094	0.20360842	0.675	0.722	1	IMs
GPBP1	0.0059493	0.26993429	0.525	0.55	1	IMs
MRM2	0.00601716	0.10261352	0.319	0.393	1	IMs
KPNA1	0.00608247	0.28240505	0.53	0.569	1	IMs
PPRC1	0.0061151	0.56077141	0.34	0.327	1	IMs
SLC6A121	0.00611691	0.19181208	0.194	0.244	1	IMs
SLC1A5	0.00614266	0.47405852	0.627	0.685	1	IMs
HOOK3	0.00614485	0.27031567	0.605	0.657	1	IMs
NUP43	0.00615579	0.32528043	0.446	0.447	1	IMs
SEC24D	0.00616929	0.45196758	0.399	0.401	1	IMs
ARFIP2	0.00618827	0.1096632	0.234	0.291	1	IMs
DDX21	0.00621732	0.2624645	0.679	0.724	1	IMs
FBXL3	0.00622691	0.20164237	0.672	0.73	1	IMs
RCBTB1	0.00623171	0.48309694	0.326	0.316	1	IMs
MARCH6	0.00623574	0.21691629	0.504	0.521	1	IMs
UBXN61	0.00623632	0.19028027	0.707	0.772	1	IMs
NUP98	0.00627232	0.30051547	0.547	0.586	1	IMs
ARF5	0.00630805	0.50591714	0.359	0.356	1	IMs
SLC25A15	0.00633791	0.76037136	0.053	0.037	1	IMs
CXCL2	0.00636414	0.39032856	0.356	0.437	1	IMs
TNFAIP1	0.00638737	0.75144303	0.143	0.12	1	IMs
PLSCR3	0.00646209	0.53376036	0.257	0.236	1	IMs
KLF41	0.00648817	0.29266901	0.787	0.858	1	IMs
SERTAD1	0.006568	0.62743133	0.26	0.246	1	IMs
SLC37A4	0.00661413	0.48993861	0.304	0.282	1	IMs
BYSL	0.00662547	0.11382748	0.209	0.263	1	IMs
USP35	0.00664309	0.50864915	0.059	0.041	1	IMs
CLN3	0.0066654	0.31861876	0.486	0.508	1	IMs
AKR1A11	0.00667848	0.1252256	0.861	0.911	1	IMs
YIPF3	0.00681731	0.17103335	0.721	0.776	1	IMs
ASCC3	0.00690701	0.47463936	0.361	0.35	1	IMs
SRSF7	0.00692592	0.39560154	0.43	0.44	1	IMs
ATP6V0A2	0.00700797	0.34830602	0.31	0.291	1	IMs
SHISAL2A	0.00703902	0.17110997	0.048	0.072	1	IMs
TIAM1	0.00704032	0.34389296	0.543	0.575	1	IMs
YWHAZ	0.00705321	0.12414813	0.889	0.92	1	IMs
PRRG2	0.0070701	1.28041547	0.015	0.007	1	IMs
RHOQ1	0.00714463	0.24432702	0.818	0.862	1	IMs
DPAGT1	0.00721908	0.49785848	0.303	0.289	1	IMs

CTTN	0.00722572	0.99086331	0.024	0.014	1	IMs
TRIB3	0.00728994	1.2279882	0.134	0.112	1	IMs
C1QTNF6	0.00733426	0.70689846	0.019	0.01	1	IMs
SBNO1	0.00742272	0.44109582	0.367	0.362	1	IMs
ASPSCR1	0.00744407	0.11433506	0.305	0.378	1	IMs
SDF2L1	0.00749699	0.10675796	0.435	0.529	1	IMs
TPCN2	0.00758645	0.75265856	0.111	0.089	1	IMs
HGS	0.00762675	0.18127944	0.649	0.699	1	IMs
DNAJC13	0.00776856	0.23940141	0.631	0.681	1	IMs
DEXI	0.007838	0.7904634	0.124	0.103	1	IMs
PRPF39	0.00790167	0.48533947	0.268	0.25	1	IMs
COMTD1	0.00793169	0.53385729	0.2	0.177	1	IMs
ISYNA1	0.00794489	0.4992492	0.012	0.005	1	IMs
ABRAXAS1	0.00795331	0.68077648	0.126	0.104	1	IMs
KBTBD8	0.00796837	0.91307872	0.138	0.116	1	IMs
GBP4	0.0079919	0.48542682	0.415	0.526	1	IMs
CCL4L2	0.0080116	1.2434344	0.043	0.029	1	IMs
RREB11	0.00808194	0.22588076	0.642	0.691	1	IMs
TM4SF191	0.00815912	1.62161999	0.084	0.065	1	IMs
ARHGAP21	0.00829136	0.62046061	0.226	0.205	1	IMs
TSPAN151	0.00829897	0.22444412	0.397	0.5	1	IMs
LRIG2	0.00830804	0.45959448	0.263	0.244	1	IMs
TUBB6	0.00834772	0.36554273	0.509	0.53	1	IMs
BDP1	0.00849874	0.35147278	0.395	0.399	1	IMs
TNFSF10	0.00865899	0.44575503	0.5	0.528	1	IMs
SHKBP1	0.00884883	0.17094171	0.766	0.821	1	IMs
RRNAD1	0.00886543	0.22969609	0.153	0.196	1	IMs
PARP9	0.00888251	0.19934363	0.713	0.772	1	IMs
BOD1L1	0.00889459	0.10159286	0.262	0.32	1	IMs
TMED6	0.0089588	1.05627568	0.02	0.011	1	IMs
TBL2	0.00896068	0.54448017	0.392	0.396	1	IMs
HECTD1	0.00896729	0.30944542	0.487	0.508	1	IMs
IRF3	0.00898668	0.22355713	0.588	0.619	1	IMs
CFL2	0.00898915	0.54480885	0.086	0.066	1	IMs
WDR17	0.00899415	1.15123014	0.043	0.029	1	IMs
MIB1	0.00901348	0.39844888	0.264	0.243	1	IMs
ABCA5	0.00909235	0.34119317	0.082	0.063	1	IMs
EPM2AIP1	0.00909803	0.61784621	0.221	0.201	1	IMs
POLR2H	0.00912875	0.3252155	0.485	0.509	1	IMs
HEXD	0.00920511	0.12833141	0.206	0.259	1	IMs
KATNBL1	0.00929205	0.51190814	0.135	0.112	1	IMs
NOC4L	0.00933502	0.10706562	0.249	0.307	1	IMs
TOR2A	0.00936953	0.22621468	0.232	0.288	1	IMs
NSD3	0.00938802	0.28339614	0.347	0.333	1	IMs
OAZ3	0.00946595	1.09822415	0.054	0.039	1	IMs
PRSS53	0.00949384	0.92377345	0.038	0.025	1	IMs
STAG2	0.00950809	0.27721276	0.571	0.61	1	IMs
SCAMP3	0.00951011	0.26648547	0.593	0.644	1	IMs
TAS2R14	0.00967506	0.55918489	0.131	0.108	1	IMs
GOT1	0.00971123	0.13468631	0.159	0.201	1	IMs
ECE1	0.00976629	1.02542237	0.138	0.117	1	IMs
TRAM2	0.00976747	0.50590731	0.165	0.141	1	IMs

STX1A	0.00980787	0.88929051	0.016	0.008	1	IMs
LATS1	0.00982797	0.23995658	0.471	0.487	1	IMs
CERS5	0.00992096	0.50159126	0.349	0.347	1	IMs
SENP6	0.00996373	0.17158853	0.608	0.648	1	IMs
VCAN1	0	3.48219706	0.756	0.223	0	Mono
TMEM176B1	0	1.30270683	0.663	0.364	0	Mono
ANXA61	0	1.38646545	0.377	0.087	0	Mono
CLEC5A1	0	2.02650522	0.455	0.176	0	Mono
EMP11	0	2.68314091	0.39	0.114	0	Mono
FPR31	0	1.52731961	0.752	0.477	0	Mono
RNASE61	0	1.54090987	0.558	0.292	0	Mono
EREG	0	1.87299998	0.462	0.207	0	Mono
PMP221	0	1.25554475	0.7	0.46	0	Mono
ITGAM1	0	1.32142978	0.824	0.585	0	Mono
NIBAN21	0	1.1282573	0.637	0.425	0	Mono
RTL51	0	2.85448968	0.252	0.042	0	Mono
PRKCB1	0	1.57689006	0.252	0.043	0	Mono
RNASE11	0	3.90715185	0.237	0.035	0	Mono
TMEM176A1	0	1.33715341	0.424	0.223	0	Mono
AQP9	0	0.96948942	0.772	0.578	0	Mono
CCR51	0	0.76651671	0.244	0.056	0	Mono
QPCT1	0	1.86694773	0.339	0.153	0	Mono
TLR21	0	1.13020954	0.499	0.314	0	Mono
CORO1A1	0	0.37488885	0.381	0.197	0	Mono
ICAM11	0	0.86329876	0.498	0.316	0	Mono
CYP2S11	0	1.8964612	0.265	0.093	0	Mono
SNCA1	0	2.03243641	0.234	0.064	0	Mono
PSTPIP11	0	0.93003863	0.282	0.112	0	Mono
SOCS31	0	0.98841979	0.857	0.689	0	Mono
ANPEP1	0	0.94490578	0.853	0.686	0	Mono
GPR1321	0	1.51331957	0.216	0.05	0	Mono
CLEC11A1	0	2.93803584	0.194	0.033	0	Mono
EIF4E31	0	0.11551959	0.24	0.08	0	Mono
PRSS361	0	1.4198129	0.317	0.162	0	Mono
MPEG11	0	0.9272223	0.864	0.717	0	Mono
IGFBP71	0	2.76776644	0.175	0.028	0	Mono
IFITM31	0	0.8266765	0.695	0.549	0	Mono
BCAT11	0	1.26782316	0.296	0.151	0	Mono
CD141	0	1.04473226	0.95	0.805	0	Mono
CD931	0	1.82130663	0.219	0.08	0	Mono
IL7R	0	0.75291431	0.785	0.647	0	Mono
CD300E1	0	1.92552896	0.239	0.104	0	Mono
RETN	0	1.03776137	0.612	0.478	0	Mono
BASP11	0	1.70223066	0.2	0.067	0	Mono
ZFP36L11	0	0.6728643	0.682	0.549	0	Mono
RASSF21	0	1.90682148	0.15	0.02	0	Mono
CCR11	0	0.818928	0.628	0.5	0	Mono
FCGR2B1	0	2.9746063	0.143	0.02	0	Mono
MS4A6A1	0	0.86637481	0.934	0.814	0	Mono
PTGER21	0	0.8253766	0.204	0.084	0	Mono
CD2091	0	1.97160198	0.187	0.072	0	Mono
NHSL21	0	2.14329728	0.139	0.027	0	Mono

S100A81	0	1.06792299	0.947	0.836	0	Mono
RIPOR21	0	0.85309961	0.172	0.069	0	Mono
PLA2G71	0	1.77935437	0.134	0.041	0	Mono
LRP31	0	1.97161196	0.133	0.046	0	Mono
ANTXR21	0	0.8007852	0.107	0.021	0	Mono
CSF1R1	0	0.74097612	0.942	0.856	0	Mono
PTAFR1	0	0.66010239	0.882	0.797	0	Mono
ALCAM1	0	0.66248355	0.762	0.677	0	Mono
CD991	0	0.44780045	0.822	0.74	0	Mono
RAB13	0	0.5559946	0.85	0.771	0	Mono
CYBRD11	0	2.09096039	0.092	0.02	0	Mono
IGF2BP21	0	2.33015114	0.094	0.023	0	Mono
RNASE21	0	2.50172406	0.09	0.02	0	Mono
SLC8A11	0	0.50075166	0.919	0.852	0	Mono
DYSF1	0	2.07709681	0.088	0.022	0	Mono
FAM20A1	0	2.71525039	0.075	0.011	0	Mono
ITGA51	0	0.45145125	0.952	0.893	0	Mono
RAB3D1	0	2.58828459	0.064	0.011	0	Mono
CLEC10A1	0	1.50001342	0.058	0.009	0	Mono
ENG2	0	0.39500748	0.988	0.941	0	Mono
CRIP21	0	1.82160658	0.056	0.009	0	Mono
CLEC7A1	0	0.37140373	0.952	0.905	0	Mono
S100A9	0	0.44250055	0.992	0.947	0	Mono
VSIG4	0	0.57927809	0.99	0.945	0	Mono
KCTD151	0	2.47968766	0.05	0.006	0	Mono
FOS1	0	0.55246439	0.99	0.947	0	Mono
CYBB1	0	0.79969224	0.999	0.956	0	Mono
PLXDC22	0	0.31113433	0.99	0.951	0	Mono
GPCPD11	0	0.35394334	0.986	0.95	0	Mono
TPP12	0	0.28894375	0.996	0.962	0	Mono
KCNJ151	0	3.20221556	0.035	0.002	0	Mono
ZYX1	0	0.31109717	0.992	0.96	0	Mono
MRC11	0	0.3349417	0.997	0.966	0	Mono
LTA4H1	0	0.27424026	0.998	0.969	0	Mono
ACTG11	0	0.2745019	0.995	0.969	0	Mono
IFI302	0	0.32869524	1	0.981	0	Mono
SAMHD11	0	0.25849388	1	0.981	0	Mono
S100A101	0	0.35186076	0.999	0.981	0	Mono
PFN1	0	0.25683605	0.999	0.983	0	Mono
CD441	0	0.55334797	1	0.987	0	Mono
ACTB	0	0.32725592	1	0.993	0	Mono
ID31	7.102E-307	0.71838281	0.743	0.625	1.092E-302	Mono
FN1	2.809E-304	0.96821949	0.85	0.798	4.318E-300	Mono
MARVELD11	1.002E-303	0.72098629	0.633	0.508	1.54E-299	Mono
RIN21	1.627E-303	0.59239702	0.77	0.679	2.5E-299	Mono
TYMP1	3.948E-302	0.37012665	0.991	0.957	6.068E-298	Mono
OAF1	4.134E-297	3.10567257	0.046	0.008	6.354E-293	Mono
LAPTM51	1.42E-292	0.16185349	1	0.994	2.183E-288	Mono
ETS21	1.857E-291	0.60056415	0.793	0.704	2.855E-287	Mono
RGCC1	3.49E-281	0.32586424	0.979	0.935	5.365E-277	Mono
GLIPR11	3.531E-274	0.45086244	0.859	0.783	5.427E-270	Mono
ST8SIA41	1.502E-270	0.28093939	0.178	0.077	2.308E-266	Mono

VENTX1	1.032E-269	2.56473096	0.042	0.007	1.587E-265	Mono
DSE1	1.751E-269	0.40843508	0.914	0.855	2.691E-265	Mono
P2RY111	2.291E-269	0.97615189	0.404	0.268	3.522E-265	Mono
NPC21	2.851E-269	0.29373242	0.988	0.944	4.382E-265	Mono
NLRP31	4.024E-268	1.56206716	0.136	0.052	6.185E-264	Mono
DOCK51	6.956E-267	0.79864368	0.423	0.285	1.069E-262	Mono
IL3RA1	4.565E-264	0.79679747	0.752	0.655	7.016E-260	Mono
MYC1	9.531E-262	1.13582311	0.248	0.131	1.465E-257	Mono
TREM21	1.449E-261	0.69086784	0.667	0.553	2.228E-257	Mono
FGL21	1.977E-261	0.6941842	0.618	0.478	3.039E-257	Mono
EMP31	1.089E-259	0.38527533	0.989	0.957	1.673E-255	Mono
GLIPR21	6.433E-257	0.2398511	0.996	0.968	9.888E-253	Mono
GABBR11	1.571E-256	1.71926858	0.065	0.016	2.414E-252	Mono
NRGN1	2.378E-255	1.22578671	0.292	0.168	3.656E-251	Mono
PLAC8	3.161E-252	0.80409898	0.646	0.517	4.859E-248	Mono
P2RY61	2.684E-249	1.78691891	0.06	0.014	4.126E-245	Mono
SLC43A31	3.282E-248	0.55306624	0.759	0.672	5.045E-244	Mono
NRROS1	1.094E-247	0.74376201	0.571	0.456	1.681E-243	Mono
LIMD21	2.347E-247	0.15380925	0.083	0.024	3.608E-243	Mono
APP1	5.172E-245	0.34636953	0.691	0.516	7.951E-241	Mono
MMP171	3.661E-241	2.72546269	0.044	0.008	5.628E-237	Mono
PLEC1	8.008E-240	0.31055993	0.919	0.873	1.231E-235	Mono
SORL11	2.801E-238	0.72872138	0.542	0.411	4.306E-234	Mono
PLEKHA71	2.992E-236	1.31025468	0.219	0.113	4.598E-232	Mono
FMN11	3.288E-236	1.32812736	0.114	0.042	5.054E-232	Mono
CMKLR11	7.561E-234	1.84764274	0.081	0.024	1.162E-229	Mono
C3AR11	1.199E-233	0.53968329	0.762	0.676	1.843E-229	Mono
PLEKHO11	2.902E-232	0.65805479	0.543	0.414	4.461E-228	Mono
NCF21	6.033E-232	0.21850813	0.998	0.961	9.273E-228	Mono
FCGR2A2	2.083E-231	0.41272933	0.92	0.869	3.201E-227	Mono
PALD11	2.597E-230	1.51603742	0.104	0.037	3.992E-226	Mono
MATK1	1.973E-229	0.3583951	0.053	0.012	3.033E-225	Mono
CBFA2T31	5.674E-227	1.7129014	0.054	0.012	8.722E-223	Mono
ABCC3	3.797E-224	0.50594161	0.693	0.6	5.837E-220	Mono
JUP1	1.629E-222	1.13331024	0.284	0.165	2.504E-218	Mono
TGFBI1	6.764E-218	0.32258841	0.981	0.944	1.04E-213	Mono
C15orf481	1.467E-217	0.91831243	0.301	0.179	2.255E-213	Mono
SLAMF81	5.552E-217	0.52146364	0.718	0.623	8.534E-213	Mono
HEXB	3.89E-216	0.29010535	0.97	0.93	5.979E-212	Mono
SLC40A11	1.849E-215	1.7114198	0.04	0.008	2.842E-211	Mono
SERPINB91	4.97E-215	0.2726389	0.152	0.067	7.639E-211	Mono
LILRB51	1.298E-214	1.80250038	0.081	0.026	1.995E-210	Mono
GPR271	2.155E-213	2.35637515	0.018	0.001	3.312E-209	Mono
RBPJ1	1.799E-211	0.44223392	0.774	0.715	2.765E-207	Mono
CXCL12	2.772E-211	2.1060588	0.056	0.014	4.26E-207	Mono
CTSA	5.658E-211	0.29087163	0.984	0.942	8.697E-207	Mono
ZEB21	2.847E-208	0.31559524	0.951	0.918	4.375E-204	Mono
OTULINL1	8.5E-208	0.54616945	0.671	0.578	1.307E-203	Mono
MPO1	3.848E-207	3.75610567	0.018	0.001	5.915E-203	Mono
ARPC5	2.129E-205	0.37308257	0.845	0.8	3.272E-201	Mono
ARHGAP181	4.107E-203	0.32439379	0.912	0.872	6.313E-199	Mono
BTG11	1.335E-202	0.28554356	0.927	0.892	2.051E-198	Mono

MEF2C1	5.765E-202	1.38291972	0.143	0.064	8.861E-198	Mono
DUSP61	1.316E-201	0.936231	0.394	0.276	2.023E-197	Mono
GASK1B1	2.488E-201	1.80075376	0.08	0.026	3.824E-197	Mono
MARCO1	3.475E-199	0.21956967	0.995	0.967	5.341E-195	Mono
RASGRP2	4.58E-199	0.39860347	0.763	0.681	7.04E-195	Mono
ITGB21	6.638E-199	0.26869433	0.992	0.959	1.02E-194	Mono
METRNL1	3.835E-197	0.35517131	0.9	0.853	5.895E-193	Mono
HRH21	3.996E-197	2.5080949	0.021	0.002	6.143E-193	Mono
FCGRT1	7.188E-197	0.18941251	0.998	0.966	1.105E-192	Mono
CCL21	7.712E-196	2.94551305	0.027	0.004	1.185E-191	Mono
ZFP361	8.163E-196	0.34068003	0.905	0.868	1.255E-191	Mono
ITGA41	9.098E-193	0.17379951	0.59	0.485	1.398E-188	Mono
CHD31	3.256E-191	0.13188439	0.196	0.102	5.005E-187	Mono
AP1S21	9.7E-191	0.79528324	0.42	0.311	1.491E-186	Mono
KIFC31	1.237E-190	1.05274498	0.288	0.18	1.901E-186	Mono
TNFSF101	7.6E-188	0.51990818	0.62	0.522	1.168E-183	Mono
TPM41	8.694E-188	0.34157823	0.869	0.82	1.336E-183	Mono
NAGA1	1.013E-187	0.3363186	0.886	0.839	1.558E-183	Mono
PIK3R51	3.397E-187	0.36662663	0.818	0.771	5.222E-183	Mono
CCR21	1.974E-186	0.83850072	0.052	0.013	3.034E-182	Mono
S100P	2.464E-186	1.16267791	0.335	0.227	3.788E-182	Mono
MYADM1	7.826E-186	0.41230881	0.921	0.871	1.203E-181	Mono
FHL31	1.151E-185	0.97776808	0.332	0.223	1.769E-181	Mono
IER5L1	3.324E-185	1.0429197	0.304	0.196	5.109E-181	Mono
LMNA1	5.183E-184	0.35511562	0.986	0.963	7.966E-180	Mono
SPRED11	8.16E-184	0.67630712	0.594	0.507	1.254E-179	Mono
SLC7A51	4.627E-183	0.66587338	0.118	0.05	7.112E-179	Mono
PIK3CD1	4.845E-183	0.24326598	0.59	0.486	7.448E-179	Mono
MFSD11	9.228E-178	0.23280353	0.976	0.94	1.418E-173	Mono
NEDD4L1	2.2E-177	1.3034716	0.106	0.043	3.382E-173	Mono
HSPB11	3.902E-176	0.25919116	0.984	0.947	5.998E-172	Mono
ELK31	7.29E-176	0.99357396	0.136	0.063	1.121E-171	Mono
COL8A21	1.574E-173	0.57642839	0.591	0.498	2.419E-169	Mono
HSPA61	4.905E-173	0.99557368	0.352	0.247	7.539E-169	Mono
CCL131	1.243E-172	1.39813495	0.021	0.003	1.91E-168	Mono
SH3BGRL3	3.354E-172	0.19431876	0.996	0.969	5.155E-168	Mono
TAGAP1	7.077E-172	0.36273689	0.657	0.568	1.088E-167	Mono
PLPPR21	1.587E-171	1.3410115	0.134	0.063	2.439E-167	Mono
KCTD121	3.798E-171	0.27314251	0.978	0.936	5.837E-167	Mono
TNS32	1.889E-170	0.36349786	0.837	0.793	2.904E-166	Mono
S100A6	6.159E-170	0.16631001	1	0.987	9.467E-166	Mono
TRPS11	1.522E-169	0.60469355	0.449	0.343	2.339E-165	Mono
PPP1R15B1	4.479E-169	0.32065118	0.864	0.823	6.885E-165	Mono
PELI21	9.096E-169	1.45020877	0.141	0.068	1.398E-164	Mono
LHFPL21	2.952E-167	0.49888306	0.769	0.72	4.537E-163	Mono
RAB3IL11	5.904E-167	2.38455911	0.025	0.004	9.074E-163	Mono
PRAM11	1.522E-166	0.3542017	0.835	0.78	2.34E-162	Mono
AHNAK1	6.126E-166	0.21381814	0.994	0.978	9.417E-162	Mono
SLC31A22	2.404E-165	0.28722942	0.944	0.901	3.695E-161	Mono
PAPSS21	1.044E-164	1.06037145	0.255	0.158	1.605E-160	Mono
IL17RA1	2.544E-164	0.36854923	0.808	0.758	3.91E-160	Mono
ARAP31	5.808E-164	2.29449741	0.03	0.006	8.928E-160	Mono

ARL4C1	8.651E-163	0.34230003	0.249	0.151	1.33E-158	Mono
CD55	3.301E-162	0.31545391	0.903	0.862	5.073E-158	Mono
MAFB1	8.829E-161	0.76044688	0.623	0.548	1.357E-156	Mono
TCIRG11	1.092E-160	0.25442376	0.919	0.888	1.678E-156	Mono
LILRB21	4.396E-160	0.74289306	0.517	0.428	6.758E-156	Mono
DPYD1	6.168E-160	0.35859196	0.812	0.761	9.481E-156	Mono
ADAM81	5.438E-159	0.16187595	0.056	0.017	8.359E-155	Mono
GAS61	1.915E-158	1.65213347	0.047	0.013	2.944E-154	Mono
AREG	2.048E-158	1.15652727	0.264	0.167	3.149E-154	Mono
QKI1	1.04E-157	0.22024926	0.967	0.939	1.598E-153	Mono
HM131	1.357E-157	0.45828179	0.71	0.658	2.085E-153	Mono
AP2S11	2.098E-157	0.3166308	0.857	0.814	3.224E-153	Mono
BCL11A1	7.777E-157	1.25409054	0.12	0.055	1.195E-152	Mono
ADAM281	4.479E-156	1.23649455	0.035	0.008	6.884E-152	Mono
EMILIN22	1.157E-155	0.23510861	0.97	0.926	1.779E-151	Mono
CTSB2	4.97E-155	0.19251757	1	0.984	7.639E-151	Mono
IL13RA11	5.355E-154	0.33614814	0.869	0.826	8.232E-150	Mono
ADAM10	3.822E-153	0.36067164	0.828	0.795	5.874E-149	Mono
SYNE31	8.654E-153	0.38296275	0.219	0.129	1.33E-148	Mono
FCGR3A1	1.804E-150	0.225057	0.986	0.946	2.773E-146	Mono
SYNGR21	4.098E-148	0.17316404	0.994	0.964	6.299E-144	Mono
MAMDC21	1.004E-147	1.64748905	0.032	0.007	1.543E-143	Mono
NREP1	5.986E-147	1.12860548	0.204	0.121	9.201E-143	Mono
GRN1	2.035E-146	0.14879708	1	0.98	3.129E-142	Mono
CD164	2.807E-146	0.24404338	0.943	0.904	4.315E-142	Mono
ST141	4.094E-144	0.46150745	0.71	0.655	6.293E-140	Mono
TIMP11	5.226E-144	0.46660658	0.814	0.764	8.033E-140	Mono
TNFRSF1A1	3.402E-142	0.26636629	0.904	0.869	5.23E-138	Mono
FXYD21	5.738E-141	2.30788313	0.019	0.003	8.82E-137	Mono
CHST131	2.038E-140	3.18758741	0.014	0.001	3.133E-136	Mono
CALHM21	2.243E-139	0.4494032	0.648	0.577	3.448E-135	Mono
OS9	3.32E-139	0.17244276	0.991	0.962	5.103E-135	Mono
SLCO4C1	1.128E-138	1.67715717	0.08	0.032	1.734E-134	Mono
KCNMB11	1.138E-138	1.3020408	0.134	0.068	1.749E-134	Mono
CDA1	8.163E-138	2.28700639	0.044	0.013	1.255E-133	Mono
GPX31	8.807E-138	0.61610265	0.458	0.362	1.354E-133	Mono
TLR41	4.122E-137	0.37914226	0.766	0.716	6.337E-133	Mono
STK261	1.09E-136	0.62160602	0.09	0.038	1.675E-132	Mono
VDR1	8.452E-136	0.63188132	0.487	0.398	1.299E-131	Mono
TMEM1631	1.129E-135	2.35745088	0.031	0.007	1.735E-131	Mono
PDLIM71	1.381E-135	0.47599909	0.659	0.591	2.122E-131	Mono
MARCH91	1.89E-135	0.8278825	0.128	0.064	2.905E-131	Mono
JUNB2	1.606E-133	0.27885173	0.991	0.962	2.468E-129	Mono
RGL11	2.845E-133	1.62491858	0.048	0.015	4.373E-129	Mono
SLC16A71	5.748E-133	0.42622569	0.048	0.014	8.835E-129	Mono
SATB11	1.607E-132	0.29822897	0.164	0.09	2.47E-128	Mono
GRASP1	3.156E-132	1.13906184	0.059	0.02	4.851E-128	Mono
TTC7A1	5.551E-132	0.30357802	0.851	0.812	8.533E-128	Mono
IFNGR21	1.574E-131	0.26338093	0.922	0.887	2.419E-127	Mono
MCL11	9.372E-131	0.16097121	0.998	0.986	1.441E-126	Mono
CD1D1	2.498E-128	1.13281001	0.196	0.119	3.84E-124	Mono
NEK62	2.828E-128	0.24647725	0.949	0.915	4.347E-124	Mono

CNN21	9.422E-128	0.27140066	0.843	0.8	1.448E-123	Mono
MGAT12	3.414E-127	0.198793	0.981	0.948	5.248E-123	Mono
AIF11	5.372E-127	0.18956859	0.991	0.948	8.258E-123	Mono
PLBD21	7.171E-127	0.33861247	0.795	0.758	1.102E-122	Mono
ORAI11	9.861E-127	0.35949915	0.687	0.638	1.516E-122	Mono
APBB1IP1	2.128E-126	0.24850524	0.805	0.77	3.271E-122	Mono
SLC38A71	1.241E-125	0.6091244	0.46	0.375	1.908E-121	Mono
PIK3R61	2.226E-125	1.43595109	0.075	0.031	3.421E-121	Mono
FPR2	2.91E-125	0.51761978	0.695	0.635	4.474E-121	Mono
EIF4A11	5.815E-125	0.20877632	0.962	0.928	8.939E-121	Mono
CALM1	6.413E-125	0.31221558	0.789	0.744	9.858E-121	Mono
ARRB2	6.423E-125	0.18201744	0.983	0.954	9.873E-121	Mono
DIPK2A1	1.107E-123	0.57956777	0.496	0.418	1.702E-119	Mono
TNS11	3.48E-123	1.04322264	0.221	0.141	5.349E-119	Mono
SPNS1	4.232E-123	0.27732645	0.851	0.813	6.504E-119	Mono
FPR1	1.487E-122	0.34860536	0.823	0.782	2.285E-118	Mono
TCF41	2.382E-120	1.02633703	0.137	0.073	3.662E-116	Mono
SULF21	3.205E-120	1.77734643	0.022	0.004	4.927E-116	Mono
ADAM151	3.693E-120	0.50684613	0.605	0.547	5.677E-116	Mono
CXCL21	8.491E-119	0.6971932	0.508	0.431	1.305E-114	Mono
TRIM71	1.096E-118	2.71812039	0.018	0.003	1.684E-114	Mono
HOMER31	3.744E-117	1.3059792	0.118	0.06	5.755E-113	Mono
PLOD31	1.598E-116	0.28686868	0.841	0.808	2.457E-112	Mono
CFP1	1.612E-116	0.85958655	0.355	0.269	2.477E-112	Mono
LAP31	1.866E-116	0.22310888	0.932	0.899	2.869E-112	Mono
YBX31	3.744E-114	0.34777978	0.746	0.702	5.755E-110	Mono
HS3ST3B11	3.936E-114	0.71695652	0.064	0.025	6.05E-110	Mono
CSF2RA1	9.464E-114	0.24056084	0.907	0.873	1.455E-109	Mono
GAL3ST41	1.127E-113	1.85842719	0.028	0.007	1.733E-109	Mono
HSH2D1	1.967E-113	1.04961558	0.114	0.058	3.023E-109	Mono
CSF3R1	1.8E-112	0.47111326	0.519	0.443	2.766E-108	Mono
IL10RA1	2.584E-112	0.20336818	0.854	0.814	3.972E-108	Mono
N4BP11	3.807E-112	0.33578449	0.751	0.71	5.852E-108	Mono
EPHB21	4.86E-112	2.23263231	0.028	0.007	7.471E-108	Mono
ATP6V0D11	6.85E-112	0.19490149	0.969	0.93	1.053E-107	Mono
CD861	2.917E-111	0.34884924	0.732	0.682	4.483E-107	Mono
CIITA1	5.553E-111	0.24175572	0.845	0.81	8.536E-107	Mono
BLVRB1	1.081E-110	0.51835754	0.603	0.543	1.662E-106	Mono
KDM2B1	1.673E-110	0.43409507	0.473	0.396	2.572E-106	Mono
RGS21	4.382E-110	0.55674192	0.571	0.486	6.736E-106	Mono
AHR1	8.99E-110	0.2791358	0.828	0.792	1.382E-105	Mono
GNAQ1	1.015E-109	0.23079769	0.903	0.875	1.561E-105	Mono
SBNO21	1.301E-109	0.3301006	0.682	0.632	1.999E-105	Mono
KBTBD11	2.098E-109	1.02829119	0.186	0.116	3.225E-105	Mono
GFRA21	2.608E-109	2.12361421	0.057	0.021	4.009E-105	Mono
UCK21	2.757E-109	1.31626934	0.066	0.027	4.238E-105	Mono
MXD11	4.661E-109	0.54814511	0.558	0.492	7.165E-105	Mono
DAZAP21	9.327E-109	0.23063253	0.877	0.84	1.434E-104	Mono
ACSL41	1.692E-108	0.3059	0.756	0.714	2.601E-104	Mono
MAP3K81	2.416E-108	0.29335914	0.813	0.775	3.714E-104	Mono
BRI31	6.076E-108	0.14466575	0.998	0.97	9.34E-104	Mono
FXYD51	2.051E-107	0.14594533	0.982	0.952	3.153E-103	Mono

VMP11	1.648E-106	0.27337992	0.772	0.74	2.534E-102	Mono
SLC17A91	7.757E-106	0.88256072	0.205	0.133	1.192E-101	Mono
ENO11	2.672E-105	0.21845014	0.915	0.885	4.108E-101	Mono
ZNF4671	2.891E-105	2.07236441	0.035	0.01	4.443E-101	Mono
CKAP4	5.075E-105	0.65343985	0.372	0.29	7.801E-101	Mono
PNP1	1.296E-104	0.30242085	0.784	0.743	1.993E-100	Mono
HGF1	1.517E-104	2.81931907	0.014	0.002	2.332E-100	Mono
AGTPBP11	1.9E-104	0.3765628	0.591	0.531	2.92E-100	Mono
SEMA6B1	1.936E-104	0.89564059	0.269	0.192	2.975E-100	Mono
PPBP	2.838E-104	3.43449258	0.064	0.026	4.362E-100	Mono
ARHGDIB	1.98E-103	0.13408152	0.994	0.972	3.043E-99	Mono
CEACAM41	2.834E-102	1.22724337	0.137	0.078	4.3558E-98	Mono
MERTK1	4.034E-102	1.58051331	0.017	0.003	6.2008E-98	Mono
SLFN11	5.429E-102	0.19764983	0.943	0.91	8.3442E-98	Mono
DDX211	7.629E-102	0.31172156	0.757	0.721	1.1727E-97	Mono
METTL91	9.091E-102	0.47894996	0.544	0.479	1.3974E-97	Mono
TMEM170B	6.621E-101	0.45159783	0.555	0.495	1.0178E-96	Mono
CORO1C1	9.579E-101	0.16517506	0.984	0.95	1.4723E-96	Mono
ID2	9.637E-101	0.31006214	0.7	0.652	1.4813E-96	Mono
STK32B1	2.495E-100	3.15092905	0.01	0.001	3.8352E-96	Mono
COMT1	2.705E-100	0.1862915	0.974	0.939	4.1574E-96	Mono
HEXA1	6.262E-100	0.20356225	0.96	0.93	9.6253E-96	Mono
ACTN11	1.963E-99	0.17399862	0.985	0.944	3.0177E-95	Mono
CRIP1	3.987E-99	0.19808773	0.997	0.988	6.1279E-95	Mono
NDRG21	7.268E-99	0.51884487	0.034	0.01	1.1172E-94	Mono
ITGAV1	7.839E-99	0.44652915	0.589	0.538	1.205E-94	Mono
ADGRE21	8.9E-99	0.82463024	0.245	0.17	1.368E-94	Mono
NCR3LG11	6.495E-98	1.14907113	0.124	0.069	9.9834E-94	Mono
CFL1	1.2894E-97	0.14956696	0.993	0.962	1.982E-93	Mono
CTNNB1	1.7943E-96	0.25511436	0.826	0.8	2.758E-92	Mono
SIGLEC15	2.195E-96	0.24527553	0.837	0.797	3.374E-92	Mono
MEGF9	4.1227E-96	0.28227754	0.865	0.829	6.337E-92	Mono
PPP4C1	5.0048E-96	0.24117146	0.851	0.818	7.6929E-92	Mono
IDH12	1.6952E-95	0.26231206	0.86	0.827	2.6058E-91	Mono
TLR81	3.4776E-95	0.54974556	0.478	0.412	5.3455E-91	Mono
IL27RA1	4.3925E-95	0.4357979	0.55	0.488	6.7517E-91	Mono
GALNT31	1.4241E-94	1.23190043	0.029	0.008	2.1889E-90	Mono
IFITM21	5.6627E-94	0.34745706	0.429	0.358	8.7042E-90	Mono
EIF4EBP1	2.244E-93	0.34778382	0.712	0.671	3.4492E-89	Mono
RARA1	5.3087E-93	0.1845818	0.973	0.941	8.1599E-89	Mono
CHST111	1.5515E-92	0.35434018	0.569	0.515	2.3849E-88	Mono
SHKBP11	1.6519E-92	0.24189248	0.852	0.819	2.5391E-88	Mono
SKI	3.2771E-91	0.18459685	0.956	0.927	5.0372E-87	Mono
TMEM511	3.7497E-91	0.68853549	0.347	0.273	5.7637E-87	Mono
S100A111	8.6241E-91	0.12255097	1	0.976	1.3256E-86	Mono
DCUN1D3	2.7959E-90	0.60626542	0.45	0.383	4.2975E-86	Mono
FZD21	3.9233E-89	1.82923147	0.035	0.011	6.0305E-85	Mono
KCNQ1	5.7153E-89	0.35032737	0.684	0.638	8.7851E-85	Mono
TGFBR11	8.7536E-89	0.3280966	0.687	0.649	1.3455E-84	Mono
ACE1	3.152E-88	0.28421891	0.845	0.824	4.8449E-84	Mono
FURIN1	6.0357E-88	0.29389078	0.686	0.656	9.2774E-84	Mono
SIGLEC91	1.37E-87	0.408625	0.607	0.559	2.1058E-83	Mono

ADGRA2	3.0256E-87	1.02705759	0.161	0.101	4.6506E-83	Mono
CXCL31	3.5016E-87	0.44856614	0.706	0.675	5.3823E-83	Mono
GRB101	1.1246E-86	1.33609302	0.089	0.046	1.7286E-82	Mono
SGMS2	1.1409E-86	0.29319424	0.865	0.836	1.7537E-82	Mono
SCO21	6.0505E-86	0.39741111	0.615	0.569	9.3002E-82	Mono
NCEH11	1.4362E-85	0.18627731	0.956	0.926	2.2076E-81	Mono
LAMB21	1.6812E-85	0.80316213	0.252	0.182	2.5842E-81	Mono
SMIM15	2.1419E-85	0.24275989	0.844	0.818	3.2924E-81	Mono
CEBPB1	3.9347E-84	0.16893512	0.997	0.964	6.0481E-80	Mono
TES1	7.2533E-84	0.35683672	0.451	0.386	1.1149E-79	Mono
GBA1	1.0795E-83	0.28429121	0.775	0.747	1.6593E-79	Mono
PICALM1	2.3296E-83	0.14910517	0.977	0.955	3.5809E-79	Mono
CAPN21	4.121E-83	0.19966825	0.879	0.847	6.3345E-79	Mono
PRCP1	5.3754E-83	0.51243542	0.505	0.444	8.2625E-79	Mono
CDC37	2.1843E-82	0.17079703	0.961	0.939	3.3575E-78	Mono
RAP2B1	1.6906E-81	0.32374867	0.675	0.64	2.5986E-77	Mono
DNAJC101	2.2794E-81	0.43762886	0.505	0.452	3.5037E-77	Mono
SIGLEC141	5.5626E-81	0.33952335	0.746	0.711	8.5503E-77	Mono
PCSK5	1.5576E-80	0.74841436	0.256	0.188	2.3942E-76	Mono
SEMA4A1	1.6832E-80	0.73427903	0.261	0.194	2.5872E-76	Mono
GNB41	1.5904E-79	0.25397673	0.801	0.766	2.4446E-75	Mono
LRRC252	1.8984E-79	0.33236781	0.69	0.656	2.918E-75	Mono
RAB321	3.8113E-79	0.41253349	0.559	0.512	5.8583E-75	Mono
WSB21	4.3397E-79	0.23024616	0.805	0.774	6.6705E-75	Mono
GRB21	1.2073E-78	0.13787375	0.988	0.958	1.8557E-74	Mono
PLEKHG21	2.4095E-78	1.1117128	0.053	0.023	3.7036E-74	Mono
DUSP71	1.3157E-77	0.56188875	0.448	0.393	2.0223E-73	Mono
YWHAH1	3.9734E-77	0.2379471	0.868	0.84	6.1075E-73	Mono
PAK11	4.5531E-77	0.4653248	0.479	0.425	6.9985E-73	Mono
IRAK3	7.0925E-77	0.26470906	0.7	0.662	1.0902E-72	Mono
ZFP36L21	1.2042E-76	0.12169059	0.963	0.944	1.851E-72	Mono
LRP11	1.733E-76	0.12679407	0.996	0.959	2.6638E-72	Mono
HCK1	1.8193E-76	0.15587666	0.987	0.949	2.7964E-72	Mono
SLC25A371	3.2E-76	0.69824525	0.24	0.176	4.9187E-72	Mono
CTSS1	4.4397E-76	0.12311336	0.999	0.971	6.8243E-72	Mono
VIM1	6.4396E-76	0.10535832	1	0.998	9.8984E-72	Mono
ENHO1	8.1508E-76	2.68447399	0.012	0.002	1.2529E-71	Mono
CD1801	8.657E-76	1.21240481	0.134	0.083	1.3307E-71	Mono
MYL61	9.8632E-76	0.13484994	0.988	0.964	1.5161E-71	Mono
SLC7A71	1.3267E-75	0.1516037	0.992	0.951	2.0392E-71	Mono
DNM11	1.4939E-75	1.32810162	0.055	0.024	2.2963E-71	Mono
MOB3A1	1.9799E-75	0.18278463	0.864	0.843	3.0433E-71	Mono
PCNX21	2.628E-75	0.17808841	0.157	0.101	4.0395E-71	Mono
ADAM91	2.6701E-75	0.37830486	0.587	0.549	4.1042E-71	Mono
SKIL1	2.7242E-75	0.1785399	0.892	0.869	4.1874E-71	Mono
SIGLEC101	3.9421E-75	1.17455357	0.136	0.085	6.0595E-71	Mono
TRPM21	4.9446E-75	1.14170064	0.069	0.034	7.6003E-71	Mono
AGRP	7.8562E-75	0.474375	0.685	0.65	1.2076E-70	Mono
MARC1	9.949E-75	1.84804054	0.043	0.017	1.5293E-70	Mono
KCNK131	1.6339E-74	1.30845102	0.082	0.043	2.5115E-70	Mono
ITPRIP1	1.9357E-74	0.45517576	0.496	0.445	2.9754E-70	Mono
TFEC2	3.1798E-74	0.21928073	0.815	0.789	4.8876E-70	Mono

SERPINB81	3.5123E-74	0.42513145	0.539	0.494	5.3987E-70	Mono
MMP141	7.0433E-74	0.46155124	0.633	0.603	1.0826E-69	Mono
TPPP31	2.1813E-73	0.66925882	0.022	0.006	3.3529E-69	Mono
PLCB21	2.7939E-73	0.17454107	0.859	0.838	4.2945E-69	Mono
ZMIZ11	4.2426E-73	0.21887188	0.853	0.828	6.5213E-69	Mono
DOCK101	4.9365E-73	0.17311172	0.728	0.705	7.5879E-69	Mono
KCNN41	7.7512E-73	0.86951229	0.108	0.062	1.1914E-68	Mono
KLF101	8.8944E-73	0.39587071	0.537	0.483	1.3672E-68	Mono
SLC3A21	1.4375E-72	0.1679703	0.953	0.922	2.2095E-68	Mono
PLCB3	1.7183E-72	0.29903263	0.691	0.668	2.6412E-68	Mono
CMTM31	4.4345E-72	0.19601768	0.758	0.71	6.8163E-68	Mono
STT3B	5.4363E-72	0.18209258	0.855	0.832	8.3561E-68	Mono
ARHGEF10L2	6.4967E-72	0.15934989	0.89	0.861	9.986E-68	Mono
ANXA21	9.0473E-72	0.11342461	0.998	0.979	1.3907E-67	Mono
PTTG1IP1	1.1161E-71	0.16412182	0.953	0.922	1.7156E-67	Mono
PTGS11	1.5057E-71	0.75314286	0.258	0.195	2.3145E-67	Mono
ARNTL21	1.6807E-71	1.09563465	0.07	0.035	2.5835E-67	Mono
TRAPPC51	1.9264E-71	0.16719355	0.889	0.867	2.961E-67	Mono
ACPP1	2.3401E-71	1.77605275	0.017	0.004	3.597E-67	Mono
MAP2K61	2.8057E-71	0.93485913	0.114	0.068	4.3127E-67	Mono
DEGS1	3.3433E-71	0.20990073	0.835	0.806	5.139E-67	Mono
GNA151	3.6774E-71	0.2935206	0.704	0.677	5.6526E-67	Mono
F5	4.0015E-71	1.26035812	0.068	0.034	6.1507E-67	Mono
C5AR11	4.4357E-71	0.18231493	0.986	0.95	6.8181E-67	Mono
SMAD7	1.3184E-70	0.23311194	0.725	0.696	2.0265E-66	Mono
SAP301	2.3519E-70	0.51021505	0.323	0.259	3.615E-66	Mono
MICAL21	8.5854E-70	0.57508653	0.254	0.193	1.3197E-65	Mono
CAPZB1	1.5915E-69	0.15125148	0.913	0.889	2.4462E-65	Mono
EIF4E21	2.0624E-69	0.21201969	0.817	0.794	3.1701E-65	Mono
PEAK31	2.0802E-69	1.33922881	0.067	0.033	3.1975E-65	Mono
TREML11	2.8135E-69	1.07030217	0.064	0.031	4.3246E-65	Mono
RAB312	3.1664E-69	0.19427575	0.919	0.885	4.8671E-65	Mono
ACTR31	3.9121E-69	0.21760927	0.77	0.748	6.0133E-65	Mono
MOB3B1	4.9647E-69	0.20497301	0.856	0.814	7.6313E-65	Mono
NCSTN1	1.0519E-68	0.1543857	0.965	0.932	1.6169E-64	Mono
FRAT2	3.8781E-68	0.35724129	0.587	0.553	5.9611E-64	Mono
IRF71	1.1854E-67	0.26228859	0.729	0.7	1.8221E-63	Mono
UBXN111	2.0705E-67	0.16270335	0.807	0.786	3.1826E-63	Mono
NPTN1	4.2247E-67	0.1599299	0.962	0.932	6.4937E-63	Mono
MS4A141	1.1873E-66	0.36684289	0.49	0.434	1.8251E-62	Mono
MAF1	1.2522E-66	0.17026024	0.675	0.659	1.9247E-62	Mono
MPV17	1.738E-66	0.17356944	0.904	0.885	2.6715E-62	Mono
TYK21	2.7081E-66	0.24304712	0.634	0.599	4.1626E-62	Mono
FOSB1	2.8907E-66	0.49862375	0.243	0.18	4.4432E-62	Mono
SQOR2	6.4476E-66	0.21786622	0.852	0.828	9.9107E-62	Mono
CYB5R41	1.1177E-65	0.23251446	0.743	0.72	1.718E-61	Mono
LTBR2	1.4173E-65	0.24269405	0.786	0.767	2.1786E-61	Mono
NCLN	1.678E-65	0.23112631	0.815	0.799	2.5793E-61	Mono
MMP21	1.9229E-65	1.57362488	0.016	0.004	2.9557E-61	Mono
IL21R1	2.8146E-65	0.24740137	0.042	0.017	4.3264E-61	Mono
ELANE	7.2467E-65	1.98954243	0.019	0.005	1.1139E-60	Mono
AQP31	1.0355E-64	0.22290403	0.861	0.824	1.5917E-60	Mono

CORO1B1	1.5029E-64	0.16626342	0.925	0.901	2.3101E-60	Mono
RALGDS1	3.1099E-64	0.27241896	0.568	0.532	4.7802E-60	Mono
TMEM121B1	5.5613E-64	2.66236795	0.01	0.002	8.5482E-60	Mono
CYFIP12	7.2527E-64	0.21440576	0.842	0.821	1.1148E-59	Mono
ZNF7031	2.0871E-63	1.81968123	0.016	0.004	3.208E-59	Mono
GPR1411	4.0012E-63	1.27306333	0.048	0.022	6.1503E-59	Mono
TET31	4.228E-63	0.48144143	0.366	0.308	6.4988E-59	Mono
SIPA11	1.2119E-62	0.21143484	0.724	0.698	1.8628E-58	Mono
SCARF11	1.4565E-62	0.49872095	0.176	0.121	2.2388E-58	Mono
LIPA2	3.4383E-62	0.19350016	0.95	0.917	5.285E-58	Mono
GALNT11	3.9247E-62	0.29553694	0.631	0.606	6.0327E-58	Mono
IFI161	4.615E-62	0.13884735	0.846	0.827	7.0937E-58	Mono
PPIB1	5.6284E-62	0.24842797	0.7	0.678	8.6515E-58	Mono
DNAJB111	7.1636E-62	0.27506463	0.69	0.669	1.1011E-57	Mono
PIK3R1	7.8448E-62	0.22913853	0.615	0.584	1.2058E-57	Mono
COLEC12	1.5731E-61	0.21560182	0.858	0.829	2.4181E-57	Mono
XBP1	5.5996E-61	0.2260758	0.783	0.759	8.6071E-57	Mono
CNRIP11	6.6278E-61	0.87663982	0.146	0.098	1.0188E-56	Mono
ASGR11	7.9175E-61	0.83943386	0.217	0.162	1.217E-56	Mono
MAT2A1	1.5914E-60	0.21266719	0.848	0.838	2.4461E-56	Mono
KLF21	1.8262E-60	0.30930332	0.696	0.663	2.807E-56	Mono
FLVCR21	2.7526E-60	0.24059579	0.786	0.765	4.2311E-56	Mono
SIGLEC5	7.1541E-60	0.42952524	0.503	0.454	1.0997E-55	Mono
APOE1	1.0879E-59	0.24180979	0.948	0.939	1.6722E-55	Mono
EHD41	1.152E-59	0.16990252	0.903	0.88	1.7708E-55	Mono
GPBAR1	1.2745E-59	0.92408744	0.132	0.086	1.9591E-55	Mono
STEAP31	2.1634E-59	0.33882219	0.598	0.562	3.3254E-55	Mono
TAP11	2.3771E-59	0.13619281	0.756	0.729	3.6539E-55	Mono
NAPRT	7.5262E-59	0.22738054	0.76	0.746	1.1569E-54	Mono
DICER11	1.1312E-58	0.25626583	0.637	0.607	1.7388E-54	Mono
LGALS81	1.2118E-58	0.20158968	0.725	0.705	1.8626E-54	Mono
GALNT2	3.3321E-58	0.28409902	0.681	0.658	5.1218E-54	Mono
UAP1L11	3.3491E-58	0.51021354	0.342	0.289	5.1479E-54	Mono
SH3RF11	4.3215E-58	1.67513455	0.02	0.006	6.6426E-54	Mono
PDGFB1	5.1887E-58	0.95993608	0.128	0.083	7.9756E-54	Mono
CTSH2	6.0847E-58	0.14070473	0.998	0.965	9.3529E-54	Mono
RGS181	9.2085E-58	0.82649656	0.166	0.115	1.4154E-53	Mono
CCL18	1.1698E-57	0.57445418	0.663	0.628	1.798E-53	Mono
C9orf721	1.7533E-57	0.23656912	0.65	0.615	2.6951E-53	Mono
GALC1	6.4813E-57	0.28656359	0.704	0.691	9.9625E-53	Mono
HACD4	9.6688E-57	0.23370249	0.704	0.676	1.4862E-52	Mono
ATP6V1F2	2.1834E-56	0.18097025	0.904	0.874	3.3561E-52	Mono
H3F3A	6.1281E-56	0.12419608	0.987	0.954	9.4195E-52	Mono
ATP6V1A1	6.9478E-56	0.18218499	0.865	0.84	1.0679E-51	Mono
STARD131	8.3413E-56	1.30111774	0.056	0.028	1.2821E-51	Mono
TTYH31	8.793E-56	0.39975812	0.498	0.46	1.3516E-51	Mono
SLC16A32	1.1075E-55	0.13504366	0.975	0.941	1.7023E-51	Mono
NRAS1	1.2888E-55	0.36924288	0.5	0.462	1.981E-51	Mono
P2RY21	1.4003E-55	1.98725454	0.021	0.007	2.1524E-51	Mono
MAP3K1	1.4727E-55	0.2975084	0.598	0.57	2.2637E-51	Mono
PHC21	2.6666E-55	0.29735705	0.635	0.607	4.0988E-51	Mono
CD581	5.2919E-55	0.2046064	0.68	0.654	8.1342E-51	Mono

OTUD11	7.858E-55	0.31340066	0.633	0.607	1.2079E-50	Mono
KLF42	1.1988E-54	0.17522219	0.877	0.857	1.8427E-50	Mono
COLGALT11	1.2398E-54	0.11258167	0.983	0.954	1.9057E-50	Mono
LAMP11	1.7666E-54	0.10854057	0.998	0.969	2.7154E-50	Mono
MAPK12	1.9889E-54	0.12722493	0.925	0.905	3.0571E-50	Mono
PDXK2	6.0034E-54	0.15067238	0.945	0.921	9.2279E-50	Mono
STMP1	7.6756E-54	0.26377756	0.664	0.644	1.1798E-49	Mono
SEMA4B1	2.8978E-53	0.77716555	0.149	0.103	4.4542E-49	Mono
RCN31	3.2441E-53	0.4352138	0.25	0.194	4.9865E-49	Mono
SLC37A21	2.4378E-52	0.44119756	0.497	0.458	3.7471E-48	Mono
GBGT12	4.2283E-52	0.42702111	0.455	0.414	6.4993E-48	Mono
IRF2BP21	1.1059E-51	0.2035036	0.715	0.697	1.6999E-47	Mono
USP321	1.1455E-51	0.24145986	0.667	0.649	1.7608E-47	Mono
SNAI3	1.9615E-51	0.47712541	0.359	0.307	3.0151E-47	Mono
CTDNEP1	2.0075E-51	0.20052864	0.764	0.751	3.0858E-47	Mono
GLMP1	3.2874E-51	0.42400013	0.458	0.422	5.053E-47	Mono
VASP1	3.3473E-51	0.11313883	0.997	0.964	5.1452E-47	Mono
TRAM21	5.8354E-51	0.66069092	0.187	0.139	8.9695E-47	Mono
TMEM8A1	1.4631E-50	0.2360692	0.666	0.644	2.2489E-46	Mono
MCOLN11	1.5919E-50	0.13861625	0.939	0.915	2.4468E-46	Mono
CHST21	1.7834E-50	1.05227524	0.013	0.003	2.7413E-46	Mono
SLC36A11	2.6455E-50	0.67893447	0.163	0.117	4.0664E-46	Mono
H2AFY1	3.3993E-50	0.17234162	0.852	0.838	5.2251E-46	Mono
ORMDL2	5.1699E-50	0.20507179	0.752	0.738	7.9467E-46	Mono
CBX61	6.0659E-50	0.48180559	0.304	0.253	9.3239E-46	Mono
TTL1	6.2482E-50	0.26359153	0.63	0.606	9.6041E-46	Mono
DNAJC71	8.0878E-50	0.19873297	0.743	0.725	1.2432E-45	Mono
TNFSF81	1.0494E-49	0.29613976	0.022	0.008	1.6131E-45	Mono
CHST101	1.3246E-49	1.72391143	0.01	0.002	2.036E-45	Mono
SLC12A91	1.43E-49	0.17845551	0.8	0.783	2.1981E-45	Mono
C19orf38	1.6053E-49	0.78862343	0.206	0.158	2.4675E-45	Mono
PDIA61	1.7505E-49	0.26536192	0.618	0.599	2.6907E-45	Mono
GADD45B1	2.581E-49	0.63477778	0.207	0.157	3.9673E-45	Mono
SYNJ21	2.9967E-49	0.76112105	0.022	0.008	4.6062E-45	Mono
KDM7A1	8.7609E-49	0.46610951	0.148	0.104	1.3466E-44	Mono
SLC39A81	1.1865E-48	0.30949907	0.153	0.106	1.8237E-44	Mono
TMEM431	1.3112E-48	0.25442766	0.61	0.587	2.0155E-44	Mono
DAB22	1.9551E-48	0.1418246	0.96	0.931	3.0051E-44	Mono
IFNAR21	2.0134E-48	0.27868541	0.524	0.492	3.0948E-44	Mono
DDOST	2.6038E-48	0.2362016	0.667	0.65	4.0023E-44	Mono
RBM472	3.9051E-48	0.19637228	0.884	0.865	6.0026E-44	Mono
STARD81	6.4915E-48	0.52652371	0.309	0.259	9.9781E-44	Mono
TMEM165	8.2117E-48	0.19501368	0.731	0.717	1.2622E-43	Mono
APEX1	8.7741E-48	0.24288095	0.696	0.681	1.3487E-43	Mono
RENBP2	1.1135E-47	0.19540709	0.79	0.767	1.7115E-43	Mono
FAM102B	1.3367E-47	0.22161772	0.708	0.692	2.0547E-43	Mono
PARVB1	2.0709E-47	0.28587521	0.607	0.588	3.1832E-43	Mono
STX42	2.0976E-47	0.12769072	0.857	0.84	3.2242E-43	Mono
DCAF12	2.1916E-47	0.24258426	0.653	0.633	3.3687E-43	Mono
IGSF62	2.5998E-47	0.174792	0.842	0.801	3.9962E-43	Mono
GNAI31	2.8763E-47	0.155727	0.769	0.761	4.4211E-43	Mono
SIGIRR1	3.4775E-47	0.14383888	0.223	0.174	5.3452E-43	Mono

FAM49B1	3.8854E-47	0.17244283	0.839	0.826	5.9722E-43	Mono
MS4A4E1	5.3821E-47	0.67146134	0.055	0.029	8.2728E-43	Mono
CEP1701	5.7064E-47	0.23750281	0.727	0.71	8.7713E-43	Mono
GLRX1	7.4536E-47	0.12318954	0.964	0.93	1.1457E-42	Mono
MTMR141	9.989E-47	0.17762901	0.817	0.803	1.5354E-42	Mono
MTDH1	1.3147E-46	0.15028342	0.885	0.864	2.0208E-42	Mono
NKIRAS21	1.4968E-46	0.38912997	0.466	0.434	2.3007E-42	Mono
ODF3B1	2.0437E-46	0.26528975	0.555	0.524	3.1413E-42	Mono
COPE	2.2142E-46	0.19041618	0.73	0.719	3.4034E-42	Mono
ADAP11	2.3849E-46	0.45198183	0.372	0.326	3.6658E-42	Mono
KIAA09301	3.1422E-46	0.21811929	0.766	0.753	4.83E-42	Mono
PIM11	3.7544E-46	0.19492732	0.599	0.562	5.771E-42	Mono
PLOD1	4.3946E-46	0.19926577	0.766	0.754	6.7549E-42	Mono
CPM1	5.3941E-46	0.26593329	0.749	0.738	8.2913E-42	Mono
VAMP51	8.0813E-46	0.45069359	0.293	0.241	1.2422E-41	Mono
OLR11	9.0937E-46	0.10937035	0.998	0.97	1.3978E-41	Mono
TNFRSF1B1	1.0072E-45	0.24641239	0.473	0.438	1.5481E-41	Mono
SLC49A31	1.0264E-45	0.27127873	0.572	0.546	1.5776E-41	Mono
ARPC2	1.1129E-45	0.11833889	0.917	0.9	1.7107E-41	Mono
WDFY21	1.1211E-45	0.27496789	0.433	0.391	1.7233E-41	Mono
MGAM1	1.137E-45	1.63269627	0.02	0.007	1.7477E-41	Mono
MT-ND4L1	1.296E-45	0.19645734	0.342	0.286	1.9921E-41	Mono
TIMM8B	3.4534E-45	0.23405735	0.66	0.641	5.3083E-41	Mono
NFE2L31	7.7036E-45	0.7381871	0.157	0.114	1.1841E-40	Mono
NFAM11	9.4063E-45	0.41447059	0.43	0.387	1.4458E-40	Mono
DBR1	1.0004E-44	0.22789299	0.703	0.685	1.5377E-40	Mono
NUFIP21	1.5633E-44	0.13717022	0.783	0.772	2.403E-40	Mono
ARHGAP41	2.547E-44	0.12711338	0.66	0.642	3.915E-40	Mono
YIF1B1	2.6398E-44	0.31224372	0.54	0.518	4.0577E-40	Mono
KCNK61	2.9001E-44	0.3667749	0.41	0.37	4.4577E-40	Mono
PPP1CA	4.0035E-44	0.12763218	0.885	0.869	6.1538E-40	Mono
SLC7A12	4.6863E-44	0.49935391	0.191	0.146	7.2033E-40	Mono
UQCRC11	9.2287E-44	0.15748336	0.866	0.848	1.4185E-39	Mono
MLKL	9.4651E-44	0.15201884	0.746	0.73	1.4549E-39	Mono
FOSL21	1.1854E-43	0.16518386	0.795	0.777	1.822E-39	Mono
RNASE4	1.3032E-43	0.62495972	0.317	0.268	2.0031E-39	Mono
SLC2A3	2.2395E-43	0.20235131	0.752	0.722	3.4424E-39	Mono
ADCY71	2.5946E-43	0.16109931	0.698	0.684	3.9881E-39	Mono
IL10RB	2.6241E-43	0.16699938	0.821	0.808	4.0335E-39	Mono
AP3D1	3.9902E-43	0.17239518	0.764	0.755	6.1334E-39	Mono
PREX11	5.4284E-43	0.18942195	0.552	0.53	8.3439E-39	Mono
PPAN1	6.1737E-43	0.38073852	0.404	0.366	9.4896E-39	Mono
ME21	7.0243E-43	0.26453004	0.6	0.589	1.0797E-38	Mono
STK38L1	7.1029E-43	0.35139458	0.4	0.361	1.0918E-38	Mono
MGAT21	8.1883E-43	0.17942622	0.688	0.675	1.2586E-38	Mono
LRRFIP1	1.2085E-42	0.12688625	0.943	0.918	1.8576E-38	Mono
NAGPA	1.4271E-42	0.25977501	0.633	0.621	2.1936E-38	Mono
SH3BP51	2.0986E-42	0.1417375	0.892	0.883	3.2258E-38	Mono
TMEM371	2.3775E-42	1.40568071	0.028	0.012	3.6545E-38	Mono
CALR	2.3888E-42	0.17456193	0.862	0.847	3.6718E-38	Mono
DOCK41	2.6743E-42	0.3694733	0.379	0.333	4.1107E-38	Mono
ASAP21	3.2016E-42	1.77601886	0.01	0.002	4.9212E-38	Mono

USP3	3.3905E-42	0.20458991	0.646	0.633	5.2115E-38	Mono
CREB51	4.1627E-42	0.66824932	0.198	0.155	6.3984E-38	Mono
NFE2	4.3709E-42	0.57572302	0.318	0.273	6.7185E-38	Mono
CCL23	4.4151E-42	0.59928817	0.429	0.392	6.7864E-38	Mono
HAVCR22	5.2003E-42	0.14261715	0.838	0.825	7.9934E-38	Mono
SIRPB2	7.7237E-42	0.2465526	0.616	0.596	1.1872E-37	Mono
ELOVL11	1.0599E-41	0.1473281	0.852	0.835	1.6292E-37	Mono
BST11	1.2074E-41	0.21372843	0.782	0.765	1.8559E-37	Mono
KANK11	1.225E-41	1.27301683	0.01	0.003	1.8829E-37	Mono
ITPRIPL21	1.2617E-41	0.26905741	0.617	0.6	1.9393E-37	Mono
ERGIC3	1.363E-41	0.13120141	0.904	0.885	2.0951E-37	Mono
PSMB8	1.603E-41	0.1302042	0.922	0.906	2.4639E-37	Mono
ZBTB7B1	2.4732E-41	0.21543864	0.677	0.66	3.8016E-37	Mono
GMFB	2.5001E-41	0.28951462	0.539	0.519	3.8429E-37	Mono
TMEM106A1	2.5809E-41	0.30536649	0.461	0.428	3.967E-37	Mono
TNFSF13B1	3.3777E-41	0.3470003	0.506	0.477	5.1918E-37	Mono
IER31	3.5357E-41	0.48794882	0.365	0.322	5.4348E-37	Mono
STX61	5.4577E-41	0.14469224	0.864	0.847	8.3891E-37	Mono
ROGDI1	9.1968E-41	0.23127812	0.685	0.671	1.4136E-36	Mono
CYC11	9.3827E-41	0.18048085	0.775	0.762	1.4422E-36	Mono
IGF1R1	1.1103E-40	0.78575605	0.12	0.083	1.7066E-36	Mono
R3HDM41	1.4505E-40	0.15587036	0.633	0.617	2.2296E-36	Mono
B3GNT81	1.521E-40	1.35621552	0.014	0.004	2.3379E-36	Mono
POU2F21	3.0819E-40	0.65955534	0.048	0.026	4.7372E-36	Mono
HPCAL1	3.3867E-40	0.17701222	0.777	0.762	5.2057E-36	Mono
C1QC1	3.7891E-40	0.10339596	0.972	0.965	5.8242E-36	Mono
YPEL31	3.8093E-40	0.19414417	0.276	0.227	5.8553E-36	Mono
SIPA1L2	5.9852E-40	0.28543048	0.576	0.555	9.1999E-36	Mono
SLC9A91	7.9472E-40	0.75696553	0.021	0.008	1.2216E-35	Mono
FUT71	8.2846E-40	0.62837436	0.04	0.02	1.2734E-35	Mono
MT-ATP61	8.33E-40	0.11697839	0.501	0.457	1.2804E-35	Mono
AIMP21	9.9038E-40	0.38305764	0.448	0.419	1.5223E-35	Mono
GCA1	9.948E-40	0.13690808	0.923	0.894	1.5291E-35	Mono
PFKP1	1.0136E-39	0.25246875	0.438	0.397	1.5581E-35	Mono
PPP3CA1	1.6678E-39	0.23730773	0.585	0.568	2.5635E-35	Mono
SCN9A1	4.1643E-39	0.97574889	0.032	0.015	6.401E-35	Mono
MAN1A11	4.9838E-39	0.12998025	0.934	0.907	7.6606E-35	Mono
HIST1H2BG1	5.2757E-39	1.00257102	0.036	0.017	8.1092E-35	Mono
SPCS3	5.7571E-39	0.18068855	0.692	0.684	8.8492E-35	Mono
MT-ND51	7.216E-39	0.1572337	0.327	0.276	1.1092E-34	Mono
FES	1.0084E-38	0.3004251	0.55	0.527	1.55E-34	Mono
CEBPD1	1.009E-38	0.34131847	0.442	0.406	1.5509E-34	Mono
BCL61	1.683E-38	0.25628641	0.481	0.447	2.587E-34	Mono
GATM1	1.7963E-38	0.88154751	0.085	0.055	2.7611E-34	Mono
RAB8A	5.4712E-38	0.12425228	0.931	0.901	8.4098E-34	Mono
HHEX	5.7205E-38	0.35692206	0.458	0.426	8.7929E-34	Mono
ABRACL	6.2E-38	0.13626334	0.814	0.806	9.5301E-34	Mono
HDHD51	6.5727E-38	0.27315383	0.499	0.47	1.0103E-33	Mono
MT-ND11	6.6107E-38	0.20176561	0.257	0.209	1.0161E-33	Mono
PELI11	1.1101E-37	0.44451961	0.324	0.281	1.7064E-33	Mono
RABAC1	1.9227E-37	0.1532649	0.718	0.71	2.9553E-33	Mono
DPYSL31	2.113E-37	1.72914954	0.014	0.005	3.248E-33	Mono

TXNDC17	2.2915E-37	0.23027208	0.657	0.651	3.5223E-33	Mono
DENND31	2.4339E-37	0.29377682	0.296	0.251	3.7411E-33	Mono
TMEM167A	2.6892E-37	0.19701114	0.703	0.694	4.1336E-33	Mono
EIF4A31	3.1254E-37	0.1526758	0.774	0.767	4.8041E-33	Mono
ABCA11	3.2934E-37	0.38409589	0.378	0.33	5.0622E-33	Mono
SLC38A21	6.5458E-37	0.16679043	0.68	0.664	1.0062E-32	Mono
LYST	6.9653E-37	0.18505975	0.639	0.625	1.0706E-32	Mono
FBN21	8.8339E-37	1.81339314	0.017	0.006	1.3579E-32	Mono
TMEM147	1.1267E-36	0.17325665	0.758	0.747	1.7318E-32	Mono
PGM21	1.1942E-36	0.29973745	0.487	0.462	1.8356E-32	Mono
LIPN1	1.5454E-36	0.97097102	0.081	0.052	2.3754E-32	Mono
LFNG1	2.0799E-36	0.21415566	0.532	0.499	3.197E-32	Mono
MILR11	2.2803E-36	0.41759156	0.416	0.384	3.5051E-32	Mono
OSM1	3.7783E-36	0.50673556	0.442	0.394	5.8077E-32	Mono
TLR61	3.9915E-36	0.75478906	0.136	0.1	6.1353E-32	Mono
UBE2J1	4.7601E-36	0.18332162	0.718	0.711	7.3167E-32	Mono
RAB39A1	4.9559E-36	0.58039942	0.263	0.222	7.6178E-32	Mono
ALG3	6.1458E-36	0.35407491	0.453	0.43	9.4467E-32	Mono
SPPL2A1	6.3735E-36	0.12774574	0.843	0.828	9.7967E-32	Mono
RFX21	2.0876E-35	0.62109858	0.197	0.158	3.2088E-31	Mono
AGTRAP	2.9617E-35	0.17499499	0.782	0.768	4.5524E-31	Mono
SCAMP21	3.4472E-35	0.11780151	0.947	0.917	5.2986E-31	Mono
TMX4	3.4896E-35	0.14656124	0.704	0.694	5.3638E-31	Mono
CLSTN11	5.3475E-35	0.24291223	0.491	0.468	8.2197E-31	Mono
STK381	5.4844E-35	0.20194118	0.541	0.525	8.4301E-31	Mono
DMXL22	5.8636E-35	0.13407659	0.81	0.798	9.0129E-31	Mono
MCOLN31	5.9014E-35	1.33435219	0.016	0.006	9.071E-31	Mono
CPNE21	8.2804E-35	0.48459463	0.333	0.295	1.2728E-30	Mono
CTSG	8.454E-35	1.22350112	0.057	0.033	1.2995E-30	Mono
KIF1B1	1.0619E-34	0.29939108	0.462	0.434	1.6323E-30	Mono
MAP1S1	1.1302E-34	0.22818899	0.682	0.675	1.7372E-30	Mono
RHOT1	1.3192E-34	0.16613415	0.733	0.72	2.0277E-30	Mono
TPD52L21	1.8321E-34	0.20937385	0.647	0.637	2.816E-30	Mono
PEA151	2.9396E-34	0.21658243	0.761	0.752	4.5184E-30	Mono
VEGFA1	3.0352E-34	0.48668498	0.303	0.26	4.6653E-30	Mono
UFC1	3.5504E-34	0.1325731	0.85	0.836	5.4574E-30	Mono
JAG1	3.9247E-34	0.58993485	0.251	0.21	6.0327E-30	Mono
CLN31	4.1503E-34	0.24913305	0.528	0.506	6.3794E-30	Mono
S1PR21	4.3004E-34	0.64082575	0.136	0.101	6.6101E-30	Mono
S100Z1	4.3311E-34	1.33374379	0.034	0.017	6.6574E-30	Mono
PRKACA	6.0372E-34	0.11537472	0.947	0.923	9.2797E-30	Mono
TMEM179B	6.3694E-34	0.16017523	0.789	0.778	9.7904E-30	Mono
TP531	7.9654E-34	0.26863145	0.491	0.466	1.2244E-29	Mono
RNF7	8.4051E-34	0.12654324	0.827	0.814	1.2919E-29	Mono
SPRY21	1.0027E-33	0.90670782	0.029	0.014	1.5412E-29	Mono
TMCO1	1.1215E-33	0.13671025	0.777	0.769	1.7238E-29	Mono
NRP11	1.181E-33	0.16746656	0.837	0.823	1.8153E-29	Mono
APRT	1.3468E-33	0.17757126	0.686	0.681	2.0702E-29	Mono
PCBP11	1.5503E-33	0.14783933	0.763	0.755	2.3829E-29	Mono
TLR51	1.6619E-33	0.76171983	0.164	0.127	2.5545E-29	Mono
HDAC91	1.9666E-33	0.61967017	0.109	0.077	3.0228E-29	Mono
MAP3K31	2.0659E-33	0.34351899	0.32	0.283	3.1755E-29	Mono

ITGB1	2.0934E-33	0.10916642	0.693	0.685	3.2178E-29	Mono
IQGAP21	3.837E-33	0.15536113	0.69	0.681	5.8978E-29	Mono
DNPH11	4.674E-33	0.51908654	0.16	0.123	7.1843E-29	Mono
SMC1A	5.9721E-33	0.10552654	0.775	0.762	9.1798E-29	Mono
SYK2	6.0586E-33	0.11750285	0.955	0.924	9.3127E-29	Mono
NCOA31	6.2184E-33	0.20272631	0.432	0.403	9.5583E-29	Mono
M6PR1	1.0648E-32	0.10292949	0.97	0.945	1.6366E-28	Mono
CLCN71	1.2119E-32	0.14746467	0.83	0.82	1.8628E-28	Mono
TM9SF31	1.3689E-32	0.11478348	0.856	0.841	2.1041E-28	Mono
SELENOP1	2.5446E-32	0.25627491	0.019	0.008	3.9113E-28	Mono
ATP8B41	2.5743E-32	0.40822591	0.063	0.039	3.957E-28	Mono
UBE2D11	2.671E-32	0.30164394	0.524	0.508	4.1056E-28	Mono
CD300A1	2.9135E-32	0.31901835	0.394	0.359	4.4783E-28	Mono
NDUFS3	3.4352E-32	0.15341259	0.773	0.767	5.2802E-28	Mono
ISOC2	3.8416E-32	0.28197572	0.589	0.582	5.9049E-28	Mono
NOP16	4.9591E-32	0.28682325	0.502	0.483	7.6226E-28	Mono
GRK61	5.0548E-32	0.23051015	0.561	0.543	7.7697E-28	Mono
GLT1D1	7.2694E-32	0.56257613	0.259	0.221	1.1174E-27	Mono
PLXNC11	8.4429E-32	0.32134579	0.058	0.035	1.2978E-27	Mono
SLC44A11	1.0753E-31	0.45319547	0.313	0.279	1.6529E-27	Mono
ADAP22	1.9819E-31	0.26221314	0.577	0.566	3.0463E-27	Mono
DGKD1	2.7339E-31	0.42308211	0.143	0.108	4.2023E-27	Mono
CCDC691	3.0941E-31	0.20679031	0.335	0.297	4.7559E-27	Mono
FHOD11	4.3134E-31	0.33461649	0.345	0.311	6.6301E-27	Mono
MROH71	4.6748E-31	1.33886877	0.016	0.006	7.1856E-27	Mono
DHX151	6.2904E-31	0.10210807	0.745	0.731	9.6689E-27	Mono
CAPZA21	6.5887E-31	0.14114282	0.857	0.845	1.0128E-26	Mono
KIDINS2201	1.0637E-30	0.22332945	0.549	0.537	1.635E-26	Mono
LPCAT22	1.4502E-30	0.17133667	0.759	0.75	2.2291E-26	Mono
CNDP22	1.5037E-30	0.10107434	0.96	0.94	2.3113E-26	Mono
MTHFD1L	1.5162E-30	0.20351834	0.589	0.575	2.3306E-26	Mono
MGAT4A1	1.5993E-30	0.11571226	0.758	0.754	2.4583E-26	Mono
KLF3	1.7371E-30	0.21717556	0.639	0.63	2.6701E-26	Mono
EMILIN11	2.2714E-30	1.36195333	0.02	0.009	3.4914E-26	Mono
QARS1	2.6857E-30	0.11818069	0.819	0.81	4.1282E-26	Mono
KRAS1	2.7385E-30	0.2468104	0.427	0.402	4.2094E-26	Mono
SPG211	2.9729E-30	0.1216423	0.884	0.869	4.5696E-26	Mono
ZDHHC201	3.9076E-30	0.18510055	0.643	0.638	6.0064E-26	Mono
ZNF4691	4.334E-30	1.11722833	0.02	0.009	6.6619E-26	Mono
MGST1	4.6567E-30	0.10294518	0.521	0.469	7.1579E-26	Mono
MPP11	5.1422E-30	0.14061309	0.857	0.841	7.904E-26	Mono
IQGAP11	5.2176E-30	0.11622072	0.861	0.844	8.02E-26	Mono
SLC25A191	8.0541E-30	0.25740979	0.613	0.607	1.238E-25	Mono
PAK2	8.3992E-30	0.12888682	0.812	0.807	1.291E-25	Mono
SLC43A22	9.385E-30	0.14004253	0.859	0.846	1.4426E-25	Mono
DYRK21	9.8382E-30	0.15398606	0.359	0.326	1.5122E-25	Mono
CAP1	1.0274E-29	0.22987322	0.565	0.549	1.5792E-25	Mono
TMOD3	1.1727E-29	0.12582727	0.792	0.788	1.8026E-25	Mono
MYDGF1	2.0951E-29	0.10905237	0.957	0.929	3.2203E-25	Mono
YIPF31	2.4654E-29	0.1312433	0.784	0.775	3.7896E-25	Mono
KLHDC7B1	3.6245E-29	0.60875548	0.102	0.073	5.5712E-25	Mono
VCL1	3.7663E-29	0.65579744	0.202	0.167	5.7891E-25	Mono

DUSP23	4.9889E-29	0.21468023	0.633	0.63	7.6684E-25	Mono
PSMB91	6.2427E-29	0.12991776	0.819	0.813	9.5956E-25	Mono
UNC93B12	6.4277E-29	0.11247253	0.944	0.916	9.8799E-25	Mono
DUSP12	9.0021E-29	0.13346648	0.994	0.971	1.3837E-24	Mono
RRP12	9.1701E-29	0.2642387	0.48	0.459	1.4095E-24	Mono
NUS11	1.0816E-28	0.13081943	0.841	0.837	1.6625E-24	Mono
ZDHHC31	1.1193E-28	0.14326187	0.821	0.809	1.7204E-24	Mono
PAPSS11	1.2164E-28	0.13976904	0.85	0.838	1.8697E-24	Mono
ICA11	1.2847E-28	0.61982488	0.114	0.083	1.9747E-24	Mono
ACOT111	1.3271E-28	0.89145523	0.071	0.047	2.0399E-24	Mono
SURF41	1.4518E-28	0.16986723	0.62	0.615	2.2316E-24	Mono
SMPDL3A1	1.4606E-28	0.92540888	0.057	0.036	2.2451E-24	Mono
SLC22A41	1.7361E-28	0.58792763	0.135	0.102	2.6686E-24	Mono
DENND111	1.7944E-28	0.10380543	0.747	0.725	2.7581E-24	Mono
SLC39A101	1.9994E-28	0.13137051	0.663	0.656	3.0733E-24	Mono
LMO21	2.0741E-28	0.20951572	0.634	0.62	3.1881E-24	Mono
ADRM1	2.1157E-28	0.10482273	0.88	0.869	3.2521E-24	Mono
CRK1	2.1159E-28	0.15605938	0.649	0.645	3.2524E-24	Mono
SLC25A13	2.4589E-28	0.25940291	0.446	0.421	3.7796E-24	Mono
SOCS61	3.2864E-28	0.21012824	0.66	0.657	5.0516E-24	Mono
FCRLB1	3.3408E-28	1.07196808	0.038	0.021	5.1351E-24	Mono
CELSR11	3.5856E-28	0.6506371	0.032	0.016	5.5115E-24	Mono
IER5	7.6732E-28	0.10960396	0.894	0.879	1.1795E-23	Mono
CTNND12	8.2889E-28	0.11339621	0.904	0.888	1.2741E-23	Mono
S1PR4	8.4707E-28	0.17622701	0.634	0.624	1.302E-23	Mono
EFTUD2	8.6042E-28	0.12731033	0.785	0.78	1.3226E-23	Mono
DPP7	8.8765E-28	0.10619284	0.902	0.891	1.3644E-23	Mono
ZSWIM61	9.0139E-28	0.2851471	0.509	0.498	1.3855E-23	Mono
GALNT7	1.0533E-27	0.40373396	0.277	0.243	1.619E-23	Mono
SMARCA4	1.1655E-27	0.15374031	0.647	0.643	1.7915E-23	Mono
GPR65	1.576E-27	0.22975329	0.563	0.55	2.4224E-23	Mono
TWF22	1.5996E-27	0.10174697	0.956	0.931	2.4587E-23	Mono
PSEN11	1.6794E-27	0.13195472	0.812	0.806	2.5814E-23	Mono
PLK31	1.8029E-27	0.27288003	0.338	0.304	2.7713E-23	Mono
THBS11	2.3583E-27	0.22964498	0.856	0.851	3.6249E-23	Mono
LSP11	2.7304E-27	0.10869477	0.962	0.944	4.1968E-23	Mono
MNDA1	2.78E-27	0.12111572	0.923	0.881	4.2732E-23	Mono
FUOM1	3.5606E-27	0.15310757	0.761	0.756	5.473E-23	Mono
SLC26A111	4.0983E-27	0.29709021	0.24	0.204	6.2995E-23	Mono
SLC27A31	4.4966E-27	0.11694014	0.868	0.856	6.9117E-23	Mono
CHKA1	5.2178E-27	0.58964825	0.117	0.087	8.0203E-23	Mono
SDC21	5.4735E-27	0.8438575	0.061	0.039	8.4134E-23	Mono
SLC31A11	5.8996E-27	0.12247568	0.871	0.868	9.0683E-23	Mono
MEGF61	8.5215E-27	0.11019862	0.062	0.04	1.3098E-22	Mono
VSTM11	8.7671E-27	1.7982512	0.017	0.007	1.3476E-22	Mono
IFNGR12	8.7827E-27	0.11645031	0.918	0.899	1.35E-22	Mono
FAM32A	9.5196E-27	0.1334042	0.757	0.751	1.4633E-22	Mono
FBXW51	1.2405E-26	0.13924011	0.702	0.701	1.9068E-22	Mono
ANKRD50	1.6321E-26	0.52730815	0.233	0.199	2.5087E-22	Mono
ADSL	1.6727E-26	0.14009338	0.685	0.681	2.5712E-22	Mono
SLC4A71	1.6864E-26	0.11520007	0.202	0.167	2.5922E-22	Mono
SERTAD21	1.9415E-26	0.19725094	0.632	0.628	2.9843E-22	Mono

SH3BP21	2.1914E-26	0.12777789	0.654	0.649	3.3684E-22	Mono
NAB21	2.3957E-26	0.32515511	0.407	0.377	3.6825E-22	Mono
DCHS11	2.5222E-26	1.21112103	0.026	0.013	3.8769E-22	Mono
ANKRD221	3.6887E-26	1.03286518	0.022	0.01	5.6698E-22	Mono
SMDT1	3.8049E-26	0.13111417	0.693	0.689	5.8485E-22	Mono
SAMD11	3.8281E-26	0.25206764	0.403	0.374	5.8842E-22	Mono
CIAO2A1	4.1595E-26	0.16998267	0.679	0.678	6.3936E-22	Mono
CASP4	4.2305E-26	0.13459309	0.739	0.74	6.5027E-22	Mono
FAAH	4.4559E-26	0.93397427	0.07	0.047	6.8491E-22	Mono
PPP1R9B1	5.107E-26	0.10066032	0.901	0.891	7.85E-22	Mono
RAC1	5.3589E-26	0.12580379	0.847	0.834	8.2372E-22	Mono
CST61	5.6671E-26	1.90598663	0.011	0.004	8.7109E-22	Mono
ANG	6.2875E-26	0.71606603	0.144	0.113	9.6646E-22	Mono
MYL91	7.6272E-26	1.01129521	0.052	0.032	1.1724E-21	Mono
TBC1D141	7.6514E-26	0.25041961	0.466	0.45	1.1761E-21	Mono
KSR11	8.7128E-26	0.12084361	0.724	0.711	1.3392E-21	Mono
EPB41L31	8.7455E-26	0.16059472	0.759	0.76	1.3443E-21	Mono
MYZAP1	9.0273E-26	0.95699731	0.042	0.025	1.3876E-21	Mono
NRN11	9.8516E-26	1.78399084	0.011	0.004	1.5143E-21	Mono
GSK3A	1.225E-25	0.13573525	0.722	0.717	1.883E-21	Mono
STX10	1.2456E-25	0.1731733	0.664	0.665	1.9146E-21	Mono
CARD191	1.7322E-25	0.30913353	0.255	0.222	2.6625E-21	Mono
EEF1AKMT4	1.9215E-25	0.64274392	0.152	0.121	2.9535E-21	Mono
NR4A21	2.1282E-25	0.20582223	0.519	0.486	3.2712E-21	Mono
IRAK11	2.3261E-25	0.13924093	0.722	0.72	3.5754E-21	Mono
TMEM1541	3.0936E-25	0.2905768	0.054	0.034	4.7552E-21	Mono
MED25	4.0537E-25	0.1236487	0.758	0.759	6.231E-21	Mono
BAZ2B1	4.165E-25	0.16092611	0.562	0.55	6.402E-21	Mono
NFKBID1	5.7374E-25	0.2653425	0.281	0.247	8.819E-21	Mono
RHOU	5.8653E-25	0.2359321	0.567	0.562	9.0155E-21	Mono
NQO2	7.1746E-25	0.1983725	0.689	0.678	1.1028E-20	Mono
SPR1	8.4302E-25	0.24441152	0.564	0.554	1.2958E-20	Mono
P4HB	8.5021E-25	0.17173934	0.624	0.619	1.3069E-20	Mono
SGMS1	8.7916E-25	0.14657292	0.639	0.633	1.3514E-20	Mono
TAGLN2	8.9092E-25	0.11753531	0.851	0.834	1.3694E-20	Mono
MYO1E1	9.1796E-25	0.26226923	0.518	0.506	1.411E-20	Mono
ADGRE51	9.6088E-25	0.11460659	0.991	0.975	1.477E-20	Mono
DEDD	9.9074E-25	0.15385407	0.677	0.678	1.5229E-20	Mono
INTS41	1.0923E-24	0.13370091	0.516	0.504	1.679E-20	Mono
OTUB1	1.2276E-24	0.12008471	0.789	0.787	1.8869E-20	Mono
MAP3K22	1.3758E-24	0.1846987	0.606	0.603	2.1148E-20	Mono
PIP4P21	1.5787E-24	0.76496285	0.072	0.049	2.4266E-20	Mono
SGK11	1.9206E-24	0.29432401	0.656	0.657	2.9521E-20	Mono
GPR108	2.4975E-24	0.15275077	0.735	0.731	3.8388E-20	Mono
GYS11	3.453E-24	0.32348658	0.292	0.262	5.3076E-20	Mono
NSD11	4.5587E-24	0.10107057	0.588	0.582	7.0072E-20	Mono
GALK11	5.4865E-24	0.38344483	0.37	0.347	8.4334E-20	Mono
MEF2A1	5.5607E-24	0.169215	0.676	0.678	8.5473E-20	Mono
MT-ND21	7.3241E-24	0.23974304	0.248	0.212	1.1258E-19	Mono
BCL101	8.3211E-24	0.17189963	0.63	0.626	1.279E-19	Mono
SVBP	8.5623E-24	0.27160174	0.457	0.441	1.3161E-19	Mono
APEH	8.6012E-24	0.13419213	0.696	0.693	1.3221E-19	Mono

IL1R11	9.0266E-24	0.36277884	0.052	0.033	1.3875E-19	Mono
DOT1L1	1.0503E-23	0.26185802	0.368	0.343	1.6144E-19	Mono
NEU11	1.5354E-23	0.16301079	0.727	0.729	2.3601E-19	Mono
ANO61	1.8327E-23	0.34536959	0.388	0.37	2.8171E-19	Mono
RAPH11	1.8435E-23	0.6131127	0.198	0.167	2.8336E-19	Mono
CRLS1	2.0938E-23	0.20598583	0.58	0.579	3.2184E-19	Mono
SPHK11	2.1715E-23	1.08899643	0.028	0.015	3.3379E-19	Mono
AMPD31	2.2215E-23	0.11822987	0.029	0.016	3.4146E-19	Mono
RASA31	2.673E-23	0.17119927	0.284	0.252	4.1087E-19	Mono
SERTAD11	2.9194E-23	0.42207438	0.274	0.244	4.4873E-19	Mono
KCTD20	3.4511E-23	0.16532403	0.599	0.593	5.3048E-19	Mono
RNF168	4.1681E-23	0.10227893	0.743	0.741	6.4067E-19	Mono
RECQL	4.3192E-23	0.12911176	0.699	0.699	6.6391E-19	Mono
TIMD41	5.3033E-23	1.05120744	0.044	0.027	8.1516E-19	Mono
MAML2	6.771E-23	0.20103344	0.581	0.576	1.0408E-18	Mono
SFXN31	8.6019E-23	0.13249818	0.696	0.697	1.3222E-18	Mono
RUFY11	9.2118E-23	0.11281648	0.781	0.78	1.4159E-18	Mono
ATP1B31	1.0544E-22	0.1353455	0.755	0.749	1.6207E-18	Mono
MIS18BP1	1.0761E-22	0.1185091	0.684	0.676	1.654E-18	Mono
FILIP1L1	1.5881E-22	0.45534556	0.273	0.243	2.4411E-18	Mono
IDH3B	2.0328E-22	0.16063158	0.669	0.672	3.1246E-18	Mono
TBKBP11	2.5543E-22	0.39434728	0.055	0.036	3.9261E-18	Mono
LATS2	2.5684E-22	0.22869036	0.528	0.518	3.9478E-18	Mono
GAS71	2.5714E-22	0.311035	0.458	0.439	3.9526E-18	Mono
NDUFS7	2.5959E-22	0.131547	0.759	0.761	3.9901E-18	Mono
SETDB21	2.7896E-22	0.20869607	0.441	0.421	4.2879E-18	Mono
MRC21	2.9218E-22	1.17668009	0.013	0.005	4.4911E-18	Mono
STAB11	3.2535E-22	0.33267065	0.01	0.003	5.001E-18	Mono
ABAT1	3.9399E-22	0.31502697	0.397	0.374	6.0561E-18	Mono
INAFM21	4.0203E-22	0.34407713	0.362	0.339	6.1796E-18	Mono
YARS1	4.059E-22	0.25280164	0.395	0.377	6.2391E-18	Mono
RAP2C	4.3167E-22	0.10546446	0.729	0.729	6.6351E-18	Mono
BCKDK1	4.414E-22	0.16482396	0.712	0.714	6.7848E-18	Mono
GPR351	4.9362E-22	0.39551406	0.034	0.02	7.5875E-18	Mono
ATXN11	5.339E-22	0.12722234	0.619	0.619	8.2065E-18	Mono
PSMD7	6.8539E-22	0.14999675	0.681	0.683	1.0535E-17	Mono
SP1	6.8893E-22	0.10976308	0.718	0.721	1.059E-17	Mono
C14orf119	7.1563E-22	0.12569473	0.757	0.755	1.1E-17	Mono
PSMC5	7.872E-22	0.12471921	0.729	0.732	1.21E-17	Mono
CHRFAM7A1	8.8376E-22	1.37625472	0.019	0.009	1.3584E-17	Mono
RRAS	1.3545E-21	0.32685885	0.393	0.373	2.082E-17	Mono
SDHB	1.4349E-21	0.11520317	0.835	0.825	2.2056E-17	Mono
PHYKPL1	1.4435E-21	0.19578286	0.414	0.395	2.2188E-17	Mono
FAM91A11	1.6349E-21	0.15649296	0.575	0.575	2.5131E-17	Mono
ZCCHC2	1.8067E-21	0.1377937	0.636	0.634	2.7771E-17	Mono
NOTCH21	2.0898E-21	0.22939756	0.484	0.474	3.2122E-17	Mono
LILRB41	2.2605E-21	0.77513635	0.091	0.067	3.4746E-17	Mono
ETV61	2.3702E-21	0.11671397	0.707	0.707	3.6433E-17	Mono
CTSK1	2.446E-21	0.62077433	0.088	0.065	3.7598E-17	Mono
TIMM13	2.5723E-21	0.18637623	0.632	0.633	3.9539E-17	Mono
CA111	2.6441E-21	0.88958864	0.051	0.033	4.0642E-17	Mono
CAMK1D1	3.0301E-21	0.25110557	0.476	0.462	4.6575E-17	Mono

SCAMP31	3.1445E-21	0.14950293	0.644	0.644	4.8333E-17	Mono
FAM20C1	3.2909E-21	0.41853451	0.344	0.319	5.0585E-17	Mono
RNF41	3.3697E-21	0.16237154	0.671	0.67	5.1795E-17	Mono
PLSCR11	3.43E-21	0.12529629	0.885	0.869	5.2723E-17	Mono
CPPED12	5.6702E-21	0.15847649	0.736	0.739	8.7156E-17	Mono
RBP11	6.0272E-21	0.95159155	0.041	0.025	9.2645E-17	Mono
TRMT6	6.1027E-21	0.30861224	0.396	0.38	9.3804E-17	Mono
PSPH	7.9976E-21	0.48399735	0.232	0.203	1.2293E-16	Mono
ETV51	8.6215E-21	0.63341397	0.031	0.017	1.3252E-16	Mono
SEH1L	9.4306E-21	0.22306877	0.512	0.506	1.4496E-16	Mono
SPTSSA	9.939E-21	0.20325062	0.595	0.596	1.5277E-16	Mono
CXCL5	1.07E-20	1.48799245	0.308	0.286	1.6447E-16	Mono
APH1A1	1.1044E-20	0.11103286	0.759	0.758	1.6976E-16	Mono
E2F4	1.2592E-20	0.12469925	0.719	0.722	1.9355E-16	Mono
NLRP121	1.3128E-20	0.58152322	0.173	0.145	2.018E-16	Mono
LYN2	1.4183E-20	0.10152909	0.829	0.826	2.1801E-16	Mono
DDX49	1.4276E-20	0.26458964	0.449	0.436	2.1944E-16	Mono
COPG1	1.4401E-20	0.10372371	0.863	0.854	2.2136E-16	Mono
NMI1	1.4613E-20	0.1592762	0.646	0.643	2.2461E-16	Mono
F13A11	1.4978E-20	0.26271752	0.01	0.004	2.3022E-16	Mono
LBR1	1.775E-20	0.10574553	0.374	0.349	2.7283E-16	Mono
PCGF2	2.3554E-20	0.56527913	0.119	0.092	3.6205E-16	Mono
CLTB	2.3637E-20	0.18880083	0.563	0.564	3.6332E-16	Mono
FLI1	2.3836E-20	0.10033873	0.731	0.728	3.6638E-16	Mono
NECTIN21	2.5056E-20	0.27170639	0.457	0.442	3.8513E-16	Mono
CPT1A1	2.6385E-20	0.26362072	0.42	0.405	4.0557E-16	Mono
DVL31	2.908E-20	0.15300118	0.579	0.577	4.4699E-16	Mono
GOLGA7	3.4463E-20	0.11329573	0.689	0.689	5.2973E-16	Mono
MAP3K5	4.3833E-20	0.16049492	0.603	0.604	6.7376E-16	Mono
SH3KBP11	4.7189E-20	0.14222229	0.421	0.404	7.2534E-16	Mono
PPIA	4.7678E-20	0.14780995	0.611	0.605	7.3285E-16	Mono
PI4KA1	4.9259E-20	0.12389093	0.63	0.632	7.5716E-16	Mono
AP2M1	5.0288E-20	0.12349967	0.753	0.758	7.7298E-16	Mono
TFEB1	5.0317E-20	0.46862068	0.201	0.173	7.7343E-16	Mono
RIPOR11	5.4632E-20	0.11398453	0.805	0.803	8.3975E-16	Mono
SLC30A7	5.5512E-20	0.181676	0.529	0.526	8.5328E-16	Mono
FAM241A	6.3826E-20	0.29626078	0.298	0.272	9.8108E-16	Mono
CHIC2	6.7525E-20	0.16292164	0.45	0.436	1.0379E-15	Mono
CCDC971	7.132E-20	0.19000933	0.485	0.477	1.0963E-15	Mono
MFSD4B1	7.7507E-20	0.33464509	0.176	0.147	1.1914E-15	Mono
HIST2H2BE1	8.2005E-20	0.38095248	0.167	0.137	1.2605E-15	Mono
IER21	8.3616E-20	0.14212819	0.677	0.681	1.2853E-15	Mono
CAMK11	8.4034E-20	0.65280706	0.144	0.117	1.2917E-15	Mono
PLIN3	9.5372E-20	0.11550214	0.787	0.784	1.466E-15	Mono
SWAP701	1.0225E-19	0.1755177	0.604	0.603	1.5717E-15	Mono
CTSZ2	1.0421E-19	0.10117316	0.999	0.972	1.6019E-15	Mono
BANK11	1.3603E-19	0.7432451	0.066	0.046	2.0908E-15	Mono
CHD11	1.5159E-19	0.18500225	0.421	0.404	2.3301E-15	Mono
PACC11	1.5611E-19	0.25895027	0.444	0.43	2.3996E-15	Mono
FAM49A1	1.6116E-19	0.23442642	0.484	0.476	2.4772E-15	Mono
IL101	1.7311E-19	0.79410186	0.016	0.007	2.6609E-15	Mono
GSDME1	1.9668E-19	0.58747164	0.066	0.046	3.0232E-15	Mono

ARHGAP261	2.2454E-19	0.2005518	0.422	0.406	3.4514E-15	Mono
BCL2L111	2.3443E-19	0.17678703	0.309	0.285	3.6035E-15	Mono
PITPNA1	2.4857E-19	0.10148045	0.81	0.81	3.8208E-15	Mono
ARRDC41	2.9623E-19	0.10012998	0.753	0.734	4.5533E-15	Mono
ZC3H12C1	3.2859E-19	1.04095067	0.023	0.012	5.0507E-15	Mono
DNM1L1	3.5963E-19	0.14889734	0.595	0.595	5.5279E-15	Mono
ITFG1	3.6196E-19	0.1155024	0.758	0.763	5.5637E-15	Mono
SERBP1	4.6035E-19	0.10628132	0.717	0.722	7.0761E-15	Mono
CLPTM1	5.4339E-19	0.10042414	0.799	0.798	8.3525E-15	Mono
SESN3	5.9507E-19	0.28188619	0.272	0.245	9.1469E-15	Mono
PSMD131	6.4895E-19	0.10815347	0.691	0.695	9.975E-15	Mono
LARS1	7.3434E-19	0.11486919	0.697	0.708	1.1288E-14	Mono
PXK1	7.6886E-19	0.2982997	0.417	0.406	1.1818E-14	Mono
PTPRJ1	8.0017E-19	0.15501507	0.527	0.524	1.2299E-14	Mono
LRFN4	8.0506E-19	0.20024432	0.495	0.488	1.2375E-14	Mono
FARSA	8.6675E-19	0.13779095	0.668	0.676	1.3323E-14	Mono
SLC24A41	1.0017E-18	0.59047069	0.2	0.172	1.5397E-14	Mono
SLC46A11	1.0164E-18	0.46413204	0.205	0.176	1.5624E-14	Mono
RUFY41	1.5632E-18	1.03194061	0.015	0.007	2.4029E-14	Mono
ASPH1	1.5917E-18	0.23142388	0.485	0.478	2.4466E-14	Mono
TMEM183A	1.8981E-18	0.12491092	0.616	0.618	2.9175E-14	Mono
SLC22A152	1.9659E-18	0.28545657	0.343	0.321	3.0217E-14	Mono
MYO15B1	2.0809E-18	0.19297864	0.261	0.232	3.1985E-14	Mono
GNL3	2.2263E-18	0.15750727	0.543	0.543	3.4221E-14	Mono
LACTB2	2.6515E-18	0.15499432	0.666	0.67	4.0756E-14	Mono
TMX1	2.824E-18	0.18260281	0.594	0.597	4.3408E-14	Mono
CLIC21	3.0662E-18	0.3612896	0.314	0.289	4.713E-14	Mono
RFXANK	3.425E-18	0.19464178	0.542	0.541	5.2646E-14	Mono
CAB39	3.7671E-18	0.11536455	0.7	0.705	5.7905E-14	Mono
ZFHX31	4.0766E-18	0.23380348	0.532	0.533	6.2662E-14	Mono
CCT6A1	5.1317E-18	0.14101035	0.644	0.646	7.8879E-14	Mono
ANXA42	6.3051E-18	0.10548768	0.873	0.86	9.6915E-14	Mono
MED13L1	6.6313E-18	0.1990774	0.41	0.396	1.0193E-13	Mono
PLEK21	8.6324E-18	0.78381646	0.037	0.023	1.3269E-13	Mono
TET21	9.0356E-18	0.30462716	0.322	0.302	1.3889E-13	Mono
BRWD31	9.1364E-18	0.23468778	0.266	0.24	1.4044E-13	Mono
FAM43A1	1.5627E-17	0.5518268	0.04	0.025	2.402E-13	Mono
P2RY131	1.7554E-17	0.30003827	0.318	0.293	2.6982E-13	Mono
PIGS	2.0738E-17	0.13892631	0.674	0.682	3.1877E-13	Mono
FKBP152	2.1286E-17	0.10660219	0.807	0.809	3.2718E-13	Mono
GRAMD41	2.2691E-17	0.3370047	0.366	0.351	3.4878E-13	Mono
SCRN11	2.3213E-17	0.424995	0.186	0.159	3.5681E-13	Mono
ATAD2B1	2.5743E-17	0.17662242	0.278	0.252	3.9569E-13	Mono
SLC33A1	2.9159E-17	0.21579579	0.422	0.412	4.482E-13	Mono
DNAJC131	3.0098E-17	0.12798059	0.676	0.681	4.6263E-13	Mono
POLR2G	3.199E-17	0.14889344	0.602	0.607	4.9172E-13	Mono
JARID21	3.3604E-17	0.23171098	0.333	0.313	5.1652E-13	Mono
PBRM11	3.5825E-17	0.15147692	0.534	0.533	5.5067E-13	Mono
DHX8	3.5939E-17	0.14418092	0.549	0.548	5.5242E-13	Mono
MT-ND61	4.3833E-17	0.12983459	0.177	0.151	6.7375E-13	Mono
BTBD191	4.8876E-17	0.56634712	0.079	0.059	7.5128E-13	Mono
IDUA1	4.9093E-17	0.15522866	0.541	0.54	7.5461E-13	Mono

DEPTOR1	5.6882E-17	0.58486764	0.015	0.007	8.7434E-13	Mono
TRMT11	5.7937E-17	0.20125075	0.391	0.376	8.9055E-13	Mono
CR11	7.1245E-17	0.27213755	0.492	0.481	1.0951E-12	Mono
MANBA1	7.9601E-17	0.10322841	0.825	0.827	1.2236E-12	Mono
FGD41	8.3588E-17	0.12465645	0.627	0.629	1.2848E-12	Mono
PMFBP11	9.2333E-17	0.95410692	0.036	0.023	1.4192E-12	Mono
PLA2G4C1	9.4004E-17	0.49986476	0.116	0.093	1.4449E-12	Mono
P2RY121	9.463E-17	0.61732658	0.114	0.091	1.4546E-12	Mono
MAST3	1.0775E-16	0.11617817	0.671	0.674	1.6562E-12	Mono
SDS1	1.3075E-16	0.54501836	0.013	0.006	2.0097E-12	Mono
TIAM11	1.3531E-16	0.16207366	0.573	0.575	2.0799E-12	Mono
PEMT	1.4415E-16	0.33679137	0.354	0.341	2.2157E-12	Mono
EPRS1	1.6706E-16	0.1062267	0.682	0.687	2.5679E-12	Mono
SERINC51	1.7633E-16	0.16530696	0.547	0.549	2.7104E-12	Mono
ST3GAL61	1.7792E-16	0.18783815	0.543	0.537	2.7348E-12	Mono
MS4A6E	1.8988E-16	0.96339646	0.023	0.013	2.9187E-12	Mono
VPS35L1	1.9288E-16	0.14107157	0.645	0.652	2.9647E-12	Mono
PDK41	2.0669E-16	0.13311969	0.723	0.723	3.177E-12	Mono
NYNRIN1	2.0932E-16	0.70554191	0.078	0.059	3.2175E-12	Mono
PLEKHF21	2.0934E-16	0.18888032	0.478	0.47	3.2178E-12	Mono
NME1	2.3186E-16	0.33966449	0.38	0.369	3.564E-12	Mono
SNX291	2.4197E-16	0.28154239	0.307	0.287	3.7193E-12	Mono
C2orf68	2.42E-16	0.20427547	0.463	0.457	3.7198E-12	Mono
SERPINE11	2.9007E-16	0.83283383	0.067	0.049	4.4586E-12	Mono
DPP3	3.0272E-16	0.35540288	0.254	0.23	4.653E-12	Mono
JDP2	3.175E-16	0.15728501	0.624	0.625	4.8802E-12	Mono
PTEN	3.4079E-16	0.24233715	0.385	0.372	5.2382E-12	Mono
RGL21	3.7408E-16	0.18806615	0.364	0.344	5.75E-12	Mono
GNG5	3.7871E-16	0.26338078	0.408	0.394	5.8212E-12	Mono
RGS101	4.3555E-16	0.15291516	0.516	0.513	6.6948E-12	Mono
ZSWIM41	5.7328E-16	0.63863823	0.049	0.034	8.8118E-12	Mono
FAM124A	7.0129E-16	0.63040552	0.09	0.07	1.078E-11	Mono
KLF131	7.6796E-16	0.13604615	0.48	0.477	1.1804E-11	Mono
CYREN1	8.9306E-16	0.22933133	0.399	0.384	1.3727E-11	Mono
VPS26C	8.9868E-16	0.12235011	0.658	0.665	1.3814E-11	Mono
THOC6	1.1696E-15	0.21838108	0.469	0.464	1.7978E-11	Mono
RTCB1	1.219E-15	0.10809964	0.762	0.761	1.8738E-11	Mono
MAP4K41	1.4281E-15	0.16200042	0.443	0.43	2.1951E-11	Mono
EDEM3	1.4307E-15	0.12946926	0.584	0.584	2.1991E-11	Mono
MYO18A1	1.7313E-15	0.16539285	0.301	0.279	2.6611E-11	Mono
PALLD1	1.7556E-15	0.61611406	0.089	0.069	2.6985E-11	Mono
AUP11	1.8228E-15	0.10119772	0.697	0.706	2.8018E-11	Mono
LPGAT11	1.8433E-15	0.21970177	0.435	0.426	2.8333E-11	Mono
FAM135B	1.8752E-15	1.50944194	0.011	0.005	2.8824E-11	Mono
HS3ST11	1.8787E-15	0.80742093	0.022	0.013	2.8877E-11	Mono
PIWIL41	2.1586E-15	0.69085174	0.071	0.053	3.318E-11	Mono
SIK31	2.2478E-15	0.19501614	0.322	0.304	3.455E-11	Mono
F2RL1	3.3099E-15	1.28805661	0.01	0.004	5.0877E-11	Mono
NUTF21	3.4017E-15	0.20755438	0.507	0.512	5.2287E-11	Mono
CEBPA1	3.8047E-15	0.16995608	0.593	0.593	5.8482E-11	Mono
ITPRID21	4.1857E-15	0.11811181	0.631	0.634	6.4338E-11	Mono
ADRB2	4.2061E-15	0.19461883	0.459	0.45	6.4651E-11	Mono

TMTC2	4.3135E-15	0.34071086	0.283	0.263	6.6303E-11	Mono
PLPP51	6.2621E-15	0.18656431	0.415	0.403	9.6254E-11	Mono
TMEM189	6.4205E-15	0.12856742	0.63	0.64	9.869E-11	Mono
GADD45G1	7.5664E-15	0.49394634	0.117	0.094	1.163E-10	Mono
ANKH1	8.4634E-15	0.25153848	0.14	0.116	1.3009E-10	Mono
PNKD1	9.0106E-15	0.35802362	0.331	0.316	1.385E-10	Mono
BLNK1	1.049E-14	0.44869267	0.19	0.167	1.6124E-10	Mono
CTNS1	1.1325E-14	0.30749062	0.304	0.285	1.7407E-10	Mono
FAM78A1	1.4704E-14	0.14186254	0.44	0.431	2.2601E-10	Mono
RREB12	1.5477E-14	0.12161426	0.68	0.692	2.379E-10	Mono
NUP621	1.8696E-14	0.13633562	0.569	0.572	2.8737E-10	Mono
LIMD11	1.8889E-14	0.18791197	0.454	0.448	2.9035E-10	Mono
TAF13	1.9375E-14	0.13156388	0.652	0.663	2.9782E-10	Mono
DAGLB1	1.9732E-14	0.24632004	0.394	0.384	3.033E-10	Mono
GLUD11	2.0583E-14	0.11880362	0.641	0.648	3.1639E-10	Mono
IFRD11	2.3279E-14	0.15853908	0.658	0.653	3.5782E-10	Mono
SRGAP11	2.3284E-14	1.03533069	0.01	0.004	3.579E-10	Mono
TMEM2361	2.7138E-14	0.35411085	0.216	0.192	4.1713E-10	Mono
VPS25	2.7263E-14	0.25935993	0.399	0.39	4.1906E-10	Mono
SREBF11	2.9381E-14	0.17950984	0.574	0.58	4.5162E-10	Mono
IFITM101	3.3582E-14	0.17159512	0.522	0.519	5.1619E-10	Mono
CPNE8	3.5132E-14	0.36613061	0.264	0.243	5.4002E-10	Mono
UBAC11	3.7509E-14	0.16520642	0.534	0.541	5.7656E-10	Mono
DYNC1LI11	4.0664E-14	0.15851457	0.56	0.567	6.2504E-10	Mono
PPARD1	4.3145E-14	0.11076687	0.656	0.653	6.6318E-10	Mono
SLC15A41	5.6186E-14	0.12336052	0.637	0.644	8.6363E-10	Mono
LSM7	6.7639E-14	0.19020714	0.451	0.45	1.0397E-09	Mono
CCDC1861	7.5294E-14	0.24172929	0.205	0.184	1.1573E-09	Mono
ARHGAP121	8.1718E-14	0.17731922	0.091	0.072	1.2561E-09	Mono
GTF3C6	8.7026E-14	0.12861344	0.582	0.589	1.3377E-09	Mono
ETFA	9.4433E-14	0.10407708	0.721	0.729	1.4515E-09	Mono
TUBGCP21	9.7157E-14	0.12112973	0.595	0.599	1.4934E-09	Mono
SLC35B1	9.7524E-14	0.16949901	0.56	0.57	1.499E-09	Mono
SIRT2	1.079E-13	0.1495231	0.577	0.583	1.6585E-09	Mono
DEPDC51	1.1504E-13	0.17052018	0.369	0.356	1.7683E-09	Mono
ZNF6411	1.3251E-13	0.23591692	0.345	0.332	2.0368E-09	Mono
SNX301	1.4664E-13	0.14136484	0.515	0.516	2.254E-09	Mono
CPD	1.5387E-13	0.17622313	0.535	0.539	2.3651E-09	Mono
MYO1C	2.1268E-13	0.13073836	0.6	0.603	3.269E-09	Mono
ARHGAP101	2.4162E-13	0.16229176	0.469	0.463	3.7139E-09	Mono
LGMN1	2.5359E-13	0.65217509	0.492	0.514	3.8979E-09	Mono
FBXO34	2.7963E-13	0.2343241	0.37	0.358	4.2982E-09	Mono
RCC21	3.0679E-13	0.11169159	0.661	0.669	4.7157E-09	Mono
RB11	3.2344E-13	0.18226137	0.437	0.433	4.9715E-09	Mono
GOLT1B1	4.6411E-13	0.10433242	0.608	0.614	7.1339E-09	Mono
BCCIP	5.2354E-13	0.13760323	0.564	0.571	8.0473E-09	Mono
KDM1B1	5.6329E-13	0.18090159	0.564	0.568	8.6583E-09	Mono
BTF3L4	6.4682E-13	0.10460638	0.689	0.7	9.9423E-09	Mono
GDPD51	6.6448E-13	0.38187597	0.041	0.029	1.0214E-08	Mono
RPN1	6.9724E-13	0.10580181	0.684	0.69	1.0717E-08	Mono
RUSC2	7.4465E-13	0.33421089	0.308	0.295	1.1446E-08	Mono
HSPA5	7.7057E-13	0.16636069	0.599	0.615	1.1844E-08	Mono

SLC38A61	8.206E-13	0.20983369	0.362	0.35	1.2613E-08	Mono
SMARCD2	8.5103E-13	0.11596142	0.56	0.571	1.3081E-08	Mono
IL4I11	8.9318E-13	0.20637508	0.114	0.093	1.3729E-08	Mono
NOP2	9.1146E-13	0.23995819	0.37	0.36	1.401E-08	Mono
ARRDC51	1.0146E-12	0.27324841	0.038	0.026	1.5596E-08	Mono
CUL1	1.041E-12	0.10652621	0.571	0.578	1.6E-08	Mono
TMEM123	1.1388E-12	0.12749549	0.57	0.574	1.7505E-08	Mono
FUS1	1.1872E-12	0.12511908	0.557	0.559	1.8248E-08	Mono
TSHZ31	1.4781E-12	0.74398525	0.054	0.04	2.272E-08	Mono
KLF91	1.7077E-12	0.20633389	0.333	0.32	2.6249E-08	Mono
PRR14L	1.8207E-12	0.13921259	0.485	0.487	2.7986E-08	Mono
DIP2B1	1.8872E-12	0.2079335	0.439	0.435	2.9009E-08	Mono
CARM11	1.8873E-12	0.20236453	0.466	0.466	2.9009E-08	Mono
HORMAD11	2.1633E-12	0.4629069	0.042	0.03	3.3252E-08	Mono
NIPSNAP2	2.7466E-12	0.13127942	0.619	0.629	4.2219E-08	Mono
WDFY32	3.1458E-12	0.24990942	0.411	0.404	4.8354E-08	Mono
GIMAP81	3.1729E-12	0.32223714	0.236	0.216	4.877E-08	Mono
ABL21	3.2116E-12	0.27514047	0.312	0.297	4.9365E-08	Mono
CHSY11	3.3074E-12	0.17276223	0.374	0.364	5.0837E-08	Mono
NFYC	3.8391E-12	0.14672477	0.522	0.527	5.9011E-08	Mono
AK41	3.9411E-12	0.62587078	0.038	0.027	6.0579E-08	Mono
FGFR1	4.0322E-12	0.48569347	0.084	0.067	6.1978E-08	Mono
ARFGEF2	4.1467E-12	0.18639417	0.476	0.477	6.3739E-08	Mono
CCDC92	4.1543E-12	0.13187661	0.522	0.526	6.3855E-08	Mono
HGSNAT1	4.2065E-12	0.17852579	0.482	0.48	6.4659E-08	Mono
SEC24D1	4.2554E-12	0.22006439	0.406	0.401	6.541E-08	Mono
CERS51	4.9897E-12	0.22051441	0.357	0.347	7.6697E-08	Mono
MFSD51	5.103E-12	0.21351415	0.442	0.44	7.8438E-08	Mono
CSGALNACT21	5.1817E-12	0.11458642	0.495	0.496	7.9647E-08	Mono
DEPDC71	5.2288E-12	1.04610822	0.015	0.009	8.0372E-08	Mono
NOL31	5.9685E-12	0.63157503	0.014	0.008	9.1741E-08	Mono
SLC8B11	6.9745E-12	0.27929582	0.321	0.306	1.072E-07	Mono
SNF8	7.7842E-12	0.18161449	0.509	0.517	1.1965E-07	Mono
HEATR31	7.9876E-12	0.15016677	0.515	0.518	1.2278E-07	Mono
CLCN51	8.1967E-12	0.31615347	0.302	0.287	1.2599E-07	Mono
PSMB6	8.2907E-12	0.14854735	0.583	0.591	1.2744E-07	Mono
NDRG11	8.8124E-12	0.25780954	0.383	0.376	1.3545E-07	Mono
GLA1	9.5235E-12	0.13937122	0.621	0.631	1.4639E-07	Mono
DNPEP	9.527E-12	0.12103146	0.644	0.659	1.4644E-07	Mono
AVIL1	1.2425E-11	0.53248036	0.023	0.014	1.9098E-07	Mono
ARNT1	1.351E-11	0.16716633	0.432	0.429	2.0766E-07	Mono
ARL4A	1.9251E-11	0.22936264	0.468	0.47	2.959E-07	Mono
HPSE	2.3231E-11	0.37637311	0.258	0.241	3.5709E-07	Mono
FNIP11	2.3318E-11	0.14879189	0.427	0.423	3.5842E-07	Mono
RABEPK	2.4875E-11	0.30992994	0.26	0.244	3.8236E-07	Mono
APAF11	3.0358E-11	0.20028008	0.304	0.29	4.6664E-07	Mono
MARCH12	3.2017E-11	0.10080948	0.793	0.794	4.9214E-07	Mono
TEP11	4.1615E-11	0.16157373	0.387	0.379	6.3966E-07	Mono
FAM135A1	4.3511E-11	0.52600529	0.08	0.064	6.6881E-07	Mono
ZNF124	5.4193E-11	0.57137423	0.077	0.062	8.33E-07	Mono
BOP1	5.9609E-11	0.36158378	0.19	0.172	9.1625E-07	Mono
NETO21	6.0833E-11	0.38065231	0.116	0.098	9.3507E-07	Mono

SEC23A	6.3643E-11	0.16902299	0.457	0.461	9.7825E-07	Mono
BZW21	6.4744E-11	0.21145644	0.417	0.414	9.9519E-07	Mono
PES1	6.4798E-11	0.19276327	0.473	0.477	9.9601E-07	Mono
CLEC12A1	6.6461E-11	0.12455801	0.653	0.662	1.0216E-06	Mono
RASGRP41	7.3306E-11	0.2098565	0.441	0.438	1.1268E-06	Mono
N4BP2L1	7.8136E-11	0.11809989	0.488	0.486	1.201E-06	Mono
CPOX1	8.5738E-11	0.29497126	0.243	0.228	1.3179E-06	Mono
KLHL51	9.044E-11	0.18971729	0.239	0.22	1.3902E-06	Mono
SDC41	9.3962E-11	0.10804835	0.402	0.387	1.4443E-06	Mono
SERPINH11	1.0038E-10	0.35106947	0.29	0.28	1.5429E-06	Mono
PNOC	1.015E-10	1.17020405	0.014	0.008	1.5602E-06	Mono
ABCD12	1.028E-10	0.1446984	0.491	0.486	1.5802E-06	Mono
RASGRP31	1.1619E-10	0.35022094	0.067	0.052	1.7859E-06	Mono
HMGA11	1.3869E-10	0.82016928	0.033	0.023	2.1318E-06	Mono
ABCB61	1.6391E-10	0.35899795	0.039	0.028	2.5195E-06	Mono
EGR11	1.6936E-10	0.17444085	0.112	0.093	2.6032E-06	Mono
SLCO3A1	1.6939E-10	0.17628798	0.501	0.508	2.6037E-06	Mono
PHKB1	1.9266E-10	0.16007126	0.459	0.46	2.9615E-06	Mono
SNAPC1	1.9824E-10	0.30559642	0.227	0.209	3.0472E-06	Mono
HYOU11	2.2044E-10	0.10200596	0.677	0.691	3.3884E-06	Mono
ATP13A31	2.2421E-10	0.15573631	0.398	0.391	3.4464E-06	Mono
BCL31	2.3811E-10	0.11715529	0.442	0.438	3.66E-06	Mono
RASGEF1B2	2.4404E-10	0.10334347	0.637	0.649	3.7511E-06	Mono
GID8	2.4479E-10	0.10024489	0.556	0.566	3.7627E-06	Mono
RRAGC1	2.5753E-10	0.14681986	0.521	0.529	3.9585E-06	Mono
ZC3H12A1	2.8004E-10	0.20020727	0.305	0.291	4.3045E-06	Mono
TRGJP1	2.9461E-10	0.46707963	0.07	0.056	4.5285E-06	Mono
PSENEN	2.9892E-10	0.12901245	0.597	0.61	4.5947E-06	Mono
PAQR61	3.0337E-10	0.51025794	0.032	0.022	4.6631E-06	Mono
KCTD3	3.6173E-10	0.3232366	0.232	0.214	5.5602E-06	Mono
HCAR3	3.7517E-10	0.27168749	0.31	0.299	5.7668E-06	Mono
NNT	4.0173E-10	0.12277439	0.46	0.46	6.175E-06	Mono
ADAMTS6	4.2721E-10	0.51232297	0.049	0.037	6.5666E-06	Mono
CLPX	4.3862E-10	0.19968863	0.378	0.375	6.742E-06	Mono
AURKAIP1	4.9177E-10	0.13262405	0.529	0.538	7.5589E-06	Mono
ABHD18	5.1715E-10	0.17528931	0.246	0.23	7.9492E-06	Mono
FOLR21	5.7002E-10	0.54407632	0.153	0.136	8.7618E-06	Mono
SLC15A21	5.8759E-10	0.43242623	0.057	0.044	9.0319E-06	Mono
COQ2	6.4572E-10	0.10656231	0.589	0.599	9.9253E-06	Mono
TTLL12	6.4688E-10	0.26458143	0.371	0.368	9.9431E-06	Mono
FUT41	6.7153E-10	0.22916	0.354	0.345	1.0322E-05	Mono
NAA50	8.4194E-10	0.17891333	0.425	0.425	1.2941E-05	Mono
NOC2L	9.389E-10	0.18499584	0.397	0.395	1.4432E-05	Mono
UBE4B1	9.7155E-10	0.13500455	0.448	0.451	1.4934E-05	Mono
ISY11	9.8842E-10	0.11464808	0.588	0.599	1.5193E-05	Mono
PLCL11	1.0242E-09	0.48883378	0.066	0.053	1.5743E-05	Mono
NSF	1.0953E-09	0.12938331	0.613	0.626	1.6836E-05	Mono
LILRA11	1.179E-09	1.25535941	0.017	0.011	1.8122E-05	Mono
EAF21	1.2384E-09	0.32703806	0.198	0.181	1.9035E-05	Mono
DNASE1L12	1.3817E-09	0.10495547	0.672	0.687	2.1238E-05	Mono
PDCL3	1.3972E-09	0.1627179	0.414	0.414	2.1477E-05	Mono
ICAM3	1.5573E-09	0.1903288	0.22	0.204	2.3938E-05	Mono

FOXN31	1.6116E-09	0.15092249	0.447	0.454	2.4771E-05	Mono
SULT1B1	1.7804E-09	0.50513471	0.138	0.121	2.7367E-05	Mono
CSNK1A1	1.8297E-09	0.16105411	0.444	0.445	2.8125E-05	Mono
TMEM205	1.841E-09	0.17000969	0.533	0.542	2.8298E-05	Mono
SYT171	2.0004E-09	0.63507009	0.031	0.022	3.0748E-05	Mono
NAA38	2.0099E-09	0.15577848	0.47	0.475	3.0894E-05	Mono
CLN82	2.0333E-09	0.11377158	0.493	0.496	3.1253E-05	Mono
PRXL2C	2.2686E-09	0.15386284	0.49	0.499	3.4871E-05	Mono
PPIP5K2	2.3701E-09	0.11564083	0.495	0.499	3.643E-05	Mono
TGIF21	2.5849E-09	0.3249157	0.09	0.075	3.9733E-05	Mono
ABCF1	2.6104E-09	0.134068	0.505	0.511	4.0125E-05	Mono
AMPD2	2.8592E-09	0.18509983	0.384	0.379	4.3949E-05	Mono
RNPC31	3.1397E-09	0.16098008	0.179	0.161	4.826E-05	Mono
CLPB	3.5369E-09	0.1830998	0.438	0.442	5.4366E-05	Mono
RARG1	3.7506E-09	0.37725739	0.05	0.039	5.765E-05	Mono
TCP11L21	3.7609E-09	0.23616661	0.214	0.198	5.7809E-05	Mono
LAMC11	3.825E-09	0.64328719	0.068	0.055	5.8794E-05	Mono
MCM3AP1	3.8709E-09	0.10255435	0.42	0.421	5.95E-05	Mono
EPHB6	4.1489E-09	0.32947533	0.229	0.214	6.3772E-05	Mono
NR6A1	4.2256E-09	0.78936561	0.024	0.016	6.4952E-05	Mono
AGA	4.2349E-09	0.14433862	0.525	0.534	6.5095E-05	Mono
AP2A21	4.3602E-09	0.11352122	0.564	0.582	6.702E-05	Mono
NOD1	4.8529E-09	0.20243345	0.342	0.334	7.4594E-05	Mono
RILP1	5.4077E-09	0.15625276	0.537	0.547	8.3122E-05	Mono
MAP3K201	6.4522E-09	0.31435339	0.24	0.226	9.9178E-05	Mono
ABI31	7.7763E-09	0.27584361	0.247	0.232	0.00011953	Mono
ERP27	8.142E-09	0.14261465	0.037	0.027	0.00012515	Mono
SLC26A6	8.2344E-09	0.31318087	0.19	0.174	0.00012657	Mono
GCNT2	8.8043E-09	0.32112189	0.182	0.165	0.00013533	Mono
ELL21	9.2162E-09	0.10436471	0.589	0.6	0.00014166	Mono
MPHOSPH6	9.9521E-09	0.21206711	0.395	0.396	0.00015297	Mono
ESRRA	1.1084E-08	0.1124454	0.564	0.577	0.00017038	Mono
PDP11	1.1437E-08	0.26870229	0.24	0.228	0.00017579	Mono
HACD21	1.1607E-08	0.24586295	0.317	0.31	0.00017841	Mono
PVR1	1.2837E-08	0.16469137	0.522	0.53	0.00019732	Mono
CHFR	1.2923E-08	0.10747527	0.397	0.396	0.00019865	Mono
MAP101	1.3442E-08	0.56445153	0.049	0.038	0.00020662	Mono
BCKDHA	1.5009E-08	0.17149479	0.484	0.493	0.00023071	Mono
CD3021	1.5994E-08	0.14105646	0.583	0.592	0.00024585	Mono
WDR812	1.6003E-08	0.10399701	0.615	0.634	0.00024599	Mono
PRKX1	1.6044E-08	0.14460905	0.099	0.084	0.00024661	Mono
MCUB	1.6404E-08	0.16769516	0.417	0.417	0.00025215	Mono
LYG11	1.7471E-08	0.4890533	0.015	0.009	0.00026855	Mono
NUBP1	1.8434E-08	0.16280083	0.528	0.54	0.00028334	Mono
CNEP1R11	2.0214E-08	0.13448635	0.496	0.504	0.00031071	Mono
CANT1	2.0868E-08	0.1226997	0.489	0.498	0.00032076	Mono
THAP11	2.238E-08	0.14593989	0.503	0.516	0.000344	Mono
LPAR61	2.2711E-08	0.25974632	0.259	0.247	0.00034909	Mono
SLC23A21	2.2721E-08	0.25279347	0.223	0.209	0.00034924	Mono
INPP11	2.3724E-08	0.37535374	0.211	0.197	0.00036467	Mono
SLC39A121	2.6824E-08	0.80320703	0.015	0.009	0.00041231	Mono
PDSS1	2.724E-08	0.35958373	0.171	0.156	0.00041871	Mono

DOC2A1	2.7989E-08	0.55119563	0.015	0.009	0.00043022	Mono
SMIM12	2.9785E-08	0.10065563	0.587	0.603	0.00045782	Mono
VPS37C1	3.2895E-08	0.17450154	0.439	0.441	0.00050562	Mono
LDLRAD3	3.3944E-08	0.37355791	0.28	0.272	0.00052175	Mono
PPFIBP11	4.176E-08	0.34398793	0.165	0.149	0.00064189	Mono
GPR841	4.256E-08	0.61577406	0.03	0.021	0.00065419	Mono
SNX81	4.4125E-08	0.12776492	0.569	0.579	0.00067824	Mono
LRMDA	4.4451E-08	0.15684171	0.494	0.5	0.00068326	Mono
PDLIM41	5.12E-08	0.78404735	0.011	0.006	0.000787	Mono
GPR160	5.2038E-08	0.4239698	0.181	0.167	0.00079987	Mono
PHACTR41	5.2807E-08	0.11888988	0.453	0.459	0.00081169	Mono
SLC52A2	6.5087E-08	0.19294925	0.414	0.419	0.00100046	Mono
UBALD1	6.7712E-08	0.25518348	0.25	0.238	0.00104081	Mono
GSPT1	6.9622E-08	0.13487213	0.505	0.516	0.00107016	Mono
CXorf211	7.2246E-08	0.23714484	0.368	0.366	0.0011105	Mono
TPCN11	7.7602E-08	0.32752856	0.015	0.009	0.00119281	Mono
EIF2B1	8.0114E-08	0.13847632	0.5	0.514	0.00123144	Mono
ILK	9.1427E-08	0.14611714	0.501	0.516	0.00140533	Mono
DIAPH2	9.5092E-08	0.18851511	0.371	0.371	0.00146166	Mono
HCFC1R1	1.0583E-07	0.10253022	0.6	0.613	0.00162679	Mono
PATL1	1.0901E-07	0.15245643	0.522	0.538	0.00167565	Mono
MRAS2	1.0935E-07	0.30122394	0.354	0.355	0.00168076	Mono
FZD11	1.1183E-07	0.5750503	0.073	0.061	0.0017189	Mono
HDAC41	1.147E-07	0.21938489	0.269	0.261	0.0017631	Mono
SEC24A	1.2093E-07	0.14005689	0.433	0.439	0.00185884	Mono
TLE3	1.2891E-07	0.23002857	0.329	0.324	0.00198143	Mono
DLGAP41	1.3715E-07	0.1123141	0.493	0.504	0.00210812	Mono
DYNC1H11	1.3753E-07	0.17830903	0.379	0.379	0.00211392	Mono
PRKCA1	1.3806E-07	0.42001402	0.105	0.091	0.00212208	Mono
LPAR11	1.3931E-07	0.32916743	0.253	0.242	0.00214139	Mono
P3H1	1.4299E-07	0.23280724	0.296	0.289	0.00219792	Mono
TBRG4	1.6354E-07	0.13564347	0.461	0.47	0.00251378	Mono
AGRN1	1.7106E-07	0.12619481	0.556	0.564	0.00262943	Mono
TMEM208	1.8391E-07	0.16035001	0.477	0.49	0.0028269	Mono
TRIM65	1.8669E-07	0.17029341	0.398	0.399	0.00286962	Mono
FRAT1	1.9554E-07	0.43899379	0.095	0.081	0.00300561	Mono
NLN2	2.2052E-07	0.27643996	0.307	0.302	0.00338967	Mono
IL11	2.2904E-07	0.84997098	0.01	0.006	0.00352064	Mono
ALDH1L21	2.3226E-07	0.92132181	0.019	0.013	0.00357009	Mono
RTN21	2.3698E-07	0.2868869	0.327	0.323	0.00364263	Mono
TVP23A1	2.4341E-07	0.54726235	0.014	0.009	0.00374142	Mono
CTNNBIP1	2.485E-07	0.19228268	0.416	0.421	0.00381967	Mono
UQCRH	2.5094E-07	0.16885572	0.353	0.35	0.0038572	Mono
ADORA2B	2.6656E-07	0.50683217	0.11	0.097	0.00409735	Mono
AATK	3.2144E-07	0.62734211	0.021	0.015	0.00494088	Mono
LRGUK	3.7441E-07	0.35278472	0.028	0.021	0.00575512	Mono
ARF51	3.9799E-07	0.20918359	0.355	0.356	0.00611751	Mono
SETDB1	4.2132E-07	0.1497704	0.415	0.418	0.00647611	Mono
AGPAT41	4.3577E-07	0.1195711	0.437	0.44	0.00669826	Mono
CYB561D2	4.3628E-07	0.10719775	0.556	0.572	0.00670611	Mono
EDC41	4.4608E-07	0.14452305	0.435	0.444	0.00685675	Mono
PSTPIP21	4.4996E-07	0.20894775	0.156	0.141	0.00691635	Mono

COA6	5.0646E-07	0.12442265	0.564	0.581	0.00778484	Mono
ST6GALNAC31	5.7427E-07	1.05835376	0.018	0.013	0.00882707	Mono
PPP1R321	6.1389E-07	0.19759808	0.011	0.006	0.00943618	Mono
KIF13B1	6.1714E-07	0.11217745	0.404	0.41	0.00948603	Mono
CEBPE	6.2377E-07	0.72040537	0.045	0.035	0.00958803	Mono
GAPT	6.2755E-07	0.39580724	0.197	0.185	0.00964611	Mono
PLCXD1	6.4187E-07	0.2256473	0.21	0.197	0.00986619	Mono
LGALS21	7.0878E-07	0.21792108	0.02	0.014	0.01089461	Mono
MYBBP1A	7.339E-07	0.15969928	0.426	0.434	0.01128073	Mono
TCF121	7.4163E-07	0.11496273	0.483	0.494	0.01139966	Mono
KIAA0895L	7.5828E-07	0.2012663	0.093	0.08	0.01165546	Mono
SLC25A321	8.2122E-07	0.14029847	0.355	0.354	0.01262294	Mono
PRRT4	8.3287E-07	0.39798503	0.085	0.073	0.01280199	Mono
SLC16A61	8.8445E-07	0.24433688	0.226	0.213	0.01359493	Mono
FBXO61	9.01E-07	0.11709316	0.533	0.542	0.01384923	Mono
GCH11	9.2084E-07	0.19850959	0.075	0.063	0.01415417	Mono
SLC38A91	9.4182E-07	0.20400264	0.241	0.231	0.01447674	Mono
AGAP31	9.6538E-07	0.13463223	0.487	0.5	0.01483889	Mono
MYBPC31	1.0651E-06	0.47581187	0.031	0.024	0.01637116	Mono
TMEM251	1.131E-06	0.10448131	0.62	0.637	0.01738515	Mono
ATG9A1	1.159E-06	0.24012475	0.241	0.232	0.01781445	Mono
CCIN	1.1673E-06	0.47589289	0.077	0.066	0.01794234	Mono
KPNA3	1.2452E-06	0.10938658	0.509	0.523	0.01913984	Mono
AGPAT51	1.2838E-06	0.25841144	0.206	0.196	0.01973391	Mono
SYT111	1.3057E-06	0.15767295	0.272	0.263	0.02006988	Mono
TMEM141	1.3585E-06	0.15797745	0.423	0.429	0.02088148	Mono
CDC34	1.398E-06	0.1447087	0.441	0.451	0.02148913	Mono
LSMEM1	1.3985E-06	0.70174104	0.015	0.01	0.02149644	Mono
TUBB61	1.4034E-06	0.1419842	0.52	0.53	0.02157241	Mono
ARL4D1	1.4939E-06	0.74897666	0.013	0.008	0.02296292	Mono
CDK12	1.5434E-06	0.10080937	0.376	0.375	0.02372339	Mono
KCNMA12	1.59E-06	0.23557812	0.282	0.273	0.02443971	Mono
ZNF513	1.6233E-06	0.19146092	0.277	0.27	0.02495154	Mono
TMEM150A2	1.6644E-06	0.2396495	0.347	0.346	0.02558403	Mono
CALCRL1	1.7033E-06	0.69746832	0.032	0.024	0.02618131	Mono
RNF138	1.7154E-06	0.11455676	0.37	0.372	0.02636752	Mono
ZNF5161	1.8065E-06	0.2240991	0.285	0.277	0.02776783	Mono
SNX15	1.8071E-06	0.16531995	0.438	0.446	0.0277768	Mono
PTBP1	1.812E-06	0.15982957	0.398	0.404	0.02785195	Mono
FCAR1	1.958E-06	0.35562341	0.215	0.204	0.03009596	Mono
TSPAN331	2.0424E-06	0.28026597	0.076	0.065	0.03139388	Mono
MTCH2	2.1952E-06	0.23353489	0.335	0.335	0.03374258	Mono
TSPAN41	2.2057E-06	0.13073723	0.487	0.491	0.03390411	Mono
SPACA61	2.2373E-06	0.58787012	0.02	0.014	0.03438911	Mono
PRPF38A	2.5948E-06	0.12409778	0.4	0.406	0.03988539	Mono
EMC6	2.5972E-06	0.16770829	0.415	0.425	0.03992087	Mono
TIMM17B	2.6084E-06	0.23770644	0.248	0.24	0.04009348	Mono
PCGF31	3.1358E-06	0.10740229	0.317	0.311	0.0482009	Mono
PHF14	3.2049E-06	0.11641752	0.341	0.34	0.04926273	Mono
HSPA1A	3.2684E-06	0.17349221	0.514	0.529	0.05023817	Mono
TAF111	3.4757E-06	0.10989302	0.338	0.336	0.05342551	Mono
RNF220	3.6492E-06	0.11388021	0.465	0.476	0.05609237	Mono

RAB9A	3.687E-06	0.17700093	0.421	0.43	0.05667238	Mono
EEF2K1	3.7408E-06	0.19552033	0.333	0.332	0.05749907	Mono
CREB1	3.7904E-06	0.12452914	0.437	0.444	0.05826217	Mono
CEMIP21	4.0981E-06	0.16013074	0.23	0.221	0.06299242	Mono
LYL11	4.346E-06	0.32377688	0.247	0.24	0.06680163	Mono
WDR18	4.4378E-06	0.2387747	0.26	0.253	0.06821412	Mono
NOL12	4.5632E-06	0.19622	0.299	0.296	0.07014027	Mono
LRP81	4.9097E-06	0.13900235	0.09	0.079	0.07546676	Mono
ZDHHC12	4.9511E-06	0.23158075	0.295	0.292	0.07610411	Mono
SRPRB	5.6433E-06	0.11567711	0.468	0.48	0.08674255	Mono
HSPBP1	5.7663E-06	0.19215685	0.359	0.361	0.08863349	Mono
PCNX3	5.9169E-06	0.13602608	0.3	0.295	0.09094831	Mono
NID1	5.9183E-06	0.74674218	0.037	0.029	0.09097071	Mono
SLC5A6	6.0585E-06	0.19394111	0.301	0.298	0.093125	Mono
SIAH11	6.1421E-06	0.12333719	0.47	0.48	0.09441005	Mono
METTL21A1	6.1785E-06	0.29100495	0.251	0.244	0.09496915	Mono
MAP3K61	6.5289E-06	0.41357433	0.115	0.103	0.10035528	Mono
LRRK21	6.6201E-06	0.29998856	0.231	0.223	0.10175705	Mono
PDPR1	6.7972E-06	0.13078637	0.36	0.363	0.10448035	Mono
SNAP29	7.0528E-06	0.13677714	0.432	0.439	0.1084082	Mono
POMP	8.1734E-06	0.10949734	0.528	0.546	0.12563338	Mono
ZNF5621	8.3069E-06	0.20455509	0.322	0.322	0.12768583	Mono
CHI3L2	8.3601E-06	0.47859212	0.124	0.113	0.12850297	Mono
DHX38	8.535E-06	0.10917428	0.458	0.468	0.13119187	Mono
JOSD2	8.6561E-06	0.13035517	0.466	0.477	0.13305365	Mono
EMC1	8.6805E-06	0.11026174	0.575	0.597	0.13342799	Mono
GMPPA	8.8609E-06	0.18722391	0.352	0.353	0.13620037	Mono
ARID1A	9.1222E-06	0.10727331	0.443	0.454	0.14021746	Mono
ZNF5031	9.1232E-06	0.3589097	0.186	0.175	0.1402323	Mono
PPM1L1	9.6604E-06	0.40747032	0.105	0.094	0.14849063	Mono
TPMT	1.0073E-05	0.17685212	0.413	0.422	0.15482486	Mono
PLSCR31	1.0082E-05	0.14789702	0.243	0.236	0.15496284	Mono
H6PD1	1.1367E-05	0.13602554	0.51	0.524	0.17471878	Mono
GTPBP4	1.2009E-05	0.10584942	0.429	0.437	0.18458943	Mono
SLC2A131	1.2499E-05	0.30379897	0.123	0.111	0.19212185	Mono
LPL	1.3513E-05	0.22475829	0.888	0.919	0.20770884	Mono
HSPE1	1.4491E-05	0.13961094	0.486	0.504	0.22274399	Mono
CCT3	1.5282E-05	0.19932405	0.353	0.358	0.23489865	Mono
HEATR1	1.5903E-05	0.11255642	0.357	0.361	0.2444453	Mono
VSIG10L1	1.6574E-05	0.53967238	0.037	0.029	0.254753	Mono
RNF181	1.6821E-05	0.11740971	0.519	0.539	0.25855708	Mono
ZNF5321	1.8613E-05	0.30688115	0.133	0.121	0.28610016	Mono
RCBTB21	1.9513E-05	0.25232899	0.359	0.362	0.2999293	Mono
SGSH1	1.9948E-05	0.18580616	0.271	0.265	0.30662825	Mono
CDC42EP11	2.0439E-05	0.42576784	0.111	0.1	0.31416837	Mono
SUGT1	2.0548E-05	0.11380422	0.475	0.494	0.31585077	Mono
GPS1	2.1365E-05	0.10534197	0.507	0.523	0.32840167	Mono
SCFD2	2.2613E-05	0.21961178	0.187	0.177	0.34758374	Mono
ENTR11	2.4114E-05	0.17279871	0.247	0.24	0.37065925	Mono
CCDC1541	2.4904E-05	0.28492211	0.021	0.016	0.38279287	Mono
FOSL11	2.5944E-05	0.57998476	0.028	0.021	0.39877859	Mono
TMED2	2.6429E-05	0.10848639	0.494	0.511	0.4062349	Mono

ZNF518A	2.7129E-05	0.10020057	0.394	0.399	0.41700227	Mono
H3F3B1	2.7491E-05	0.20347598	0.273	0.268	0.42257098	Mono
HIF1A1	3.1355E-05	0.26885387	0.22	0.214	0.48195141	Mono
SLC22A16	3.15E-05	0.69575782	0.038	0.031	0.48419101	Mono
DLG41	3.2356E-05	0.19611524	0.304	0.3	0.49733723	Mono
TP53I11	3.2398E-05	0.18105044	0.368	0.371	0.49798351	Mono
BMP1	3.4531E-05	0.28682123	0.145	0.134	0.53077836	Mono
RBM191	3.4911E-05	0.10007192	0.397	0.402	0.53661454	Mono
UTP61	3.5282E-05	0.12517537	0.434	0.447	0.542319	Mono
DMAP1	3.784E-05	0.1374119	0.394	0.4	0.58163542	Mono
TEDC11	3.953E-05	0.16415833	0.289	0.286	0.60761273	Mono
RCN11	4.3231E-05	0.14714083	0.457	0.464	0.66450908	Mono
TRIM581	4.921E-05	1.03134731	0.011	0.007	0.75641159	Mono
ZDHHC171	5.128E-05	0.12455723	0.276	0.273	0.78822865	Mono
SBK3	5.335E-05	0.7414551	0.016	0.011	0.82003859	Mono
TP53BP21	5.3368E-05	0.15759188	0.31	0.31	0.82032237	Mono
PHF21A1	5.5055E-05	0.11512254	0.286	0.283	0.84624602	Mono
FLCN	5.858E-05	0.10017871	0.468	0.479	0.90044037	Mono
C16orf87	6.2197E-05	0.261193	0.185	0.177	0.95602609	Mono
ZC3H81	6.3342E-05	0.14996397	0.078	0.068	0.97363582	Mono
RIOK1	6.4324E-05	0.15955991	0.367	0.374	0.98871982	Mono
PPM1F1	6.5743E-05	0.20610968	0.354	0.359	1	Mono
NMD3	7.1207E-05	0.10812994	0.459	0.473	1	Mono
LILRA62	7.1751E-05	0.2844067	0.235	0.23	1	Mono
SGTB1	7.6523E-05	0.17803228	0.213	0.206	1	Mono
CALHM61	7.8272E-05	0.1117649	0.143	0.133	1	Mono
PRRC1	8.8139E-05	0.10118921	0.527	0.548	1	Mono
PAGR1	8.9486E-05	0.14720024	0.435	0.447	1	Mono
IRF52	0.00010098	0.12314893	0.471	0.485	1	Mono
COX7B	0.00010222	0.12916381	0.489	0.506	1	Mono
PUDP	0.0001079	0.4415429	0.04	0.033	1	Mono
SUCNR11	0.00010818	0.15519221	0.458	0.468	1	Mono
ATXN7L3	0.00011082	0.19490192	0.262	0.259	1	Mono
COA7	0.00012193	0.339619	0.209	0.204	1	Mono
VSIR1	0.00012276	0.22988979	0.323	0.325	1	Mono
NUDT9	0.00012572	0.12547332	0.522	0.539	1	Mono
FAM136A	0.00013478	0.13561725	0.387	0.397	1	Mono
SLC35C1	0.00014265	0.18252605	0.335	0.34	1	Mono
NDUFS6	0.0001458	0.14435545	0.424	0.438	1	Mono
SPOPL	0.00014619	0.17376288	0.4	0.41	1	Mono
RALA1	0.00015311	0.13363808	0.352	0.357	1	Mono
CRABP2	0.00015606	0.40094638	0.01	0.006	1	Mono
GARS1	0.00015815	0.17111854	0.251	0.247	1	Mono
MIOS1	0.00016003	0.14988291	0.334	0.338	1	Mono
WDR46	0.00016536	0.11923297	0.46	0.476	1	Mono
SLC16A13	0.00016799	0.38721786	0.051	0.044	1	Mono
AP1AR	0.00016964	0.18534552	0.363	0.371	1	Mono
ZNF326	0.00018085	0.13999331	0.342	0.345	1	Mono
TPK1	0.00018233	0.15989962	0.413	0.424	1	Mono
GPT	0.00019061	0.56486205	0.019	0.015	1	Mono
C11orf24	0.00020319	0.17562641	0.326	0.329	1	Mono
RRP15	0.00021027	0.16682432	0.311	0.313	1	Mono

SPTLC1	0.0002116	0.14287334	0.403	0.416	1	Mono
AGO41	0.0002163	0.11217656	0.441	0.456	1	Mono
ACVR1B	0.00021836	0.10777643	0.494	0.512	1	Mono
SERTAD31	0.00023318	0.13185044	0.476	0.492	1	Mono
OSTC	0.00023354	0.13163931	0.392	0.4	1	Mono
GDAP11	0.0002484	0.24951924	0.13	0.121	1	Mono
ANGPTL6	0.00026192	0.6200221	0.022	0.017	1	Mono
SGPL11	0.00026802	0.12328194	0.398	0.407	1	Mono
CCDC1491	0.00027829	0.37144416	0.136	0.128	1	Mono
TBCE.1	0.00030974	0.30613566	0.168	0.161	1	Mono
KHK	0.00032669	0.35023162	0.139	0.131	1	Mono
MAGEF11	0.00033065	0.37307376	0.09	0.082	1	Mono
ZBTB181	0.00033822	0.19775598	0.299	0.3	1	Mono
XXYLT11	0.00035176	0.32239917	0.135	0.126	1	Mono
ANXA3	0.00035956	0.48786176	0.062	0.054	1	Mono
SIAH21	0.00036059	0.12931027	0.332	0.337	1	Mono
CDS11	0.00037836	0.34723873	0.067	0.059	1	Mono
TCP1	0.00037881	0.11133282	0.51	0.533	1	Mono
EHD31	0.0003854	0.54611278	0.015	0.011	1	Mono
NIFK	0.00039755	0.16025565	0.319	0.322	1	Mono
MEF2B	0.00040659	0.25716964	0.054	0.046	1	Mono
GART	0.00043168	0.11025592	0.458	0.474	1	Mono
ARFGEF1	0.000444	0.10904881	0.363	0.37	1	Mono
JAZF11	0.00045947	0.15156915	0.421	0.434	1	Mono
GSE11	0.0004685	0.11960929	0.426	0.436	1	Mono
C21orf91	0.00047929	0.12294859	0.186	0.18	1	Mono
TLR11	0.00048412	0.1599861	0.403	0.414	1	Mono
LYSMD3	0.00052124	0.12685007	0.367	0.376	1	Mono
MRRF	0.0005462	0.1598941	0.308	0.309	1	Mono
S100A3	0.00054882	0.64067863	0.013	0.009	1	Mono
GAS2L1	0.00056877	0.17006837	0.448	0.462	1	Mono
PDIA5	0.00058358	0.33135974	0.155	0.147	1	Mono
SINHCAF	0.00058486	0.3869534	0.042	0.035	1	Mono
BANF1	0.00059763	0.10349225	0.533	0.555	1	Mono
CRYBG3	0.00059941	0.23710003	0.222	0.217	1	Mono
OGG11	0.0006369	0.15495988	0.212	0.208	1	Mono
SHROOM11	0.00064159	0.66493604	0.021	0.016	1	Mono
FSTL31	0.00064821	0.42243016	0.048	0.041	1	Mono
XPO11	0.00066084	0.12900576	0.38	0.39	1	Mono
DDX10	0.00068525	0.19148925	0.18	0.173	1	Mono
KDELR2	0.00071619	0.12935509	0.444	0.462	1	Mono
C1GALT1C1	0.00072368	0.11492833	0.465	0.485	1	Mono
USP10	0.00074999	0.11233205	0.497	0.52	1	Mono
IL6R1	0.00080284	0.21132006	0.185	0.179	1	Mono
SH3GL1	0.0008443	0.14895949	0.338	0.344	1	Mono
ARHGEF401	0.00085143	0.46324177	0.096	0.088	1	Mono
HAL	0.0008893	0.32770642	0.03	0.025	1	Mono
C1orf122	0.00090747	0.13773197	0.451	0.469	1	Mono
YJU2	0.0009152	0.10976167	0.416	0.428	1	Mono
CD381	0.00092756	0.23058991	0.135	0.127	1	Mono
POLG2	0.00094176	0.10842495	0.164	0.156	1	Mono
ZNF804A	0.0009627	0.39051173	0.067	0.059	1	Mono

DRG2	0.00098516	0.1707817	0.326	0.332	1	Mono
SPOCD11	0.00102619	0.2311171	0.321	0.32	1	Mono
PUS71	0.00105456	0.23508978	0.172	0.167	1	Mono
ZNF6181	0.00109151	0.3411882	0.046	0.04	1	Mono
METAP1D	0.00110104	0.62366541	0.03	0.025	1	Mono
REEP4	0.00113136	0.24087813	0.225	0.222	1	Mono
ATP5PO	0.00119858	0.26310211	0.134	0.126	1	Mono
MED24	0.00121661	0.10454488	0.413	0.425	1	Mono
DND11	0.00125594	0.34664973	0.018	0.014	1	Mono
DFFB	0.0012591	0.18776678	0.089	0.081	1	Mono
TMEM64	0.00126682	0.17550232	0.211	0.206	1	Mono
ETFDH	0.00130104	0.15458328	0.355	0.362	1	Mono
ZNF550	0.0013416	0.10251178	0.19	0.182	1	Mono
PRR22	0.00135892	0.44043367	0.024	0.019	1	Mono
ANKDD1A1	0.00141857	0.21568883	0.032	0.027	1	Mono
VAV31	0.00147954	0.21310723	0.196	0.192	1	Mono
METTL7A1	0.00149936	0.13002153	0.258	0.252	1	Mono
CNIH1	0.00153418	0.12742006	0.476	0.501	1	Mono
SMTN	0.00154853	0.37189087	0.041	0.035	1	Mono
FCGR3B1	0.00159197	0.20748703	0.326	0.332	1	Mono
SPATA5L1	0.00168895	0.22747047	0.171	0.165	1	Mono
NEURL1	0.00181597	0.48857057	0.029	0.024	1	Mono
FAM13A1	0.00197099	0.22957734	0.136	0.129	1	Mono
ZNF143	0.00198377	0.15455634	0.264	0.265	1	Mono
NOD21	0.00200678	0.20028536	0.38	0.393	1	Mono
ZNF7851	0.00205675	0.27362838	0.084	0.077	1	Mono
HSBP1	0.00209998	0.15783553	0.422	0.441	1	Mono
TLR31	0.00213455	0.42016325	0.025	0.02	1	Mono
ATRN1	0.00214287	0.13738677	0.428	0.448	1	Mono
ZNF7101	0.00217116	0.25685115	0.164	0.158	1	Mono
NOC4L1	0.00219567	0.16705454	0.303	0.307	1	Mono
TMEM164	0.00223178	0.14287615	0.429	0.447	1	Mono
SIMC1	0.00224752	0.29772076	0.157	0.151	1	Mono
LRP5	0.00230983	0.24297929	0.014	0.011	1	Mono
DOP1B1	0.00239442	0.21977322	0.226	0.224	1	Mono
QSOX2	0.00239605	0.23547181	0.129	0.123	1	Mono
COMTD11	0.00239823	0.16696739	0.182	0.177	1	Mono
GCNA1	0.00252631	0.16066181	0.019	0.015	1	Mono
OCLN	0.00260896	0.21335987	0.023	0.019	1	Mono
YIPF1	0.00269625	0.18301045	0.339	0.349	1	Mono
DPAGT11	0.00277004	0.22057684	0.286	0.289	1	Mono
TTC27	0.0028047	0.15405433	0.268	0.269	1	Mono
DGKG	0.00303177	0.15826546	0.068	0.078	1	Mono
RTEL1	0.00305254	0.12531425	0.185	0.179	1	Mono
FBRSL11	0.00307035	0.11693686	0.326	0.331	1	Mono
SHLD1	0.00312642	0.12276064	0.326	0.332	1	Mono
PAQR71	0.00318565	0.34587624	0.015	0.012	1	Mono
POLR1A	0.00333883	0.10496682	0.43	0.447	1	Mono
YJEFN3	0.00342611	0.24529787	0.016	0.013	1	Mono
MANEA	0.0034757	0.13780078	0.301	0.305	1	Mono
FXYD61	0.0035021	0.17641039	0.387	0.395	1	Mono
PEX11B	0.00414024	0.10637688	0.438	0.458	1	Mono

SLC35B4	0.00423523	0.19481177	0.289	0.292	1	Mono
MMACHC	0.00427731	0.12758554	0.078	0.088	1	Mono
RBM28	0.00430145	0.15242858	0.308	0.314	1	Mono
CES3	0.00449174	0.59334952	0.018	0.014	1	Mono
MAML3	0.00482339	0.24681116	0.234	0.233	1	Mono
TDG	0.00508686	0.21961443	0.144	0.139	1	Mono
LIG3	0.00512151	0.17566068	0.272	0.275	1	Mono
SLC39A3	0.00515031	0.1246543	0.44	0.459	1	Mono
CHCHD1	0.00517375	0.10113031	0.456	0.48	1	Mono
ISL2	0.00538852	0.68581218	0.016	0.013	1	Mono
XRCC4	0.00541538	0.23189333	0.265	0.267	1	Mono
MYLIP1	0.00546426	0.23858209	0.137	0.131	1	Mono
TPRA11	0.0055239	0.12098408	0.396	0.408	1	Mono
SLC16A11	0.00552674	0.14509195	0.232	0.231	1	Mono
TTC9C	0.0056895	0.13377768	0.343	0.352	1	Mono
DDX3Y	0.00579489	0.11989937	0.27	0.269	1	Mono
PSMB3	0.00581913	0.17348051	0.351	0.363	1	Mono
TMEM701	0.00598789	0.23182448	0.355	0.37	1	Mono
HMBS	0.00665033	0.19106597	0.282	0.285	1	Mono
MEX3B1	0.00669052	0.35568332	0.055	0.049	1	Mono
KLF161	0.00671666	0.12053621	0.403	0.416	1	Mono
CEACAM191	0.00701997	0.25954782	0.05	0.045	1	Mono
CDH231	0.00718193	0.23232972	0.012	0.009	1	Mono
C1QBP	0.00721258	0.15179754	0.336	0.347	1	Mono
ZNF2191	0.00748902	0.2598587	0.165	0.16	1	Mono
MOB1B1	0.00755791	0.13521476	0.332	0.34	1	Mono
PRUNE2	0.00758712	0.1964082	0.282	0.283	1	Mono
PIGW	0.00768263	0.27458597	0.163	0.159	1	Mono
HNRNPR	0.00794561	0.10748556	0.337	0.346	1	Mono
SLC39A131	0.00881525	0.15024392	0.205	0.201	1	Mono
PDGFD1	0.00897723	0.187741	0.32	0.318	1	Mono
PARP111	0.009091	0.14791154	0.156	0.151	1	Mono
PREP	0.00910984	0.10400065	0.402	0.417	1	Mono
PROSER1	0.009312	0.10672752	0.305	0.31	1	Mono
DDIT4	0.0093892	0.24033972	0.261	0.259	1	Mono
PLEKHG4	0.00952622	0.20844683	0.035	0.031	1	Mono
TAF4B1	0.00974299	0.21287046	0.029	0.025	1	Mono
NEU4	0.00994109	0.57790322	0.011	0.008	1	Mono
SERPINB92	0	6.29535935	0.836	0.071	0	DCs
RASSF22	0	5.44550861	0.716	0.026	0	DCs
TSPAN332	0	6.45253213	0.741	0.064	0	DCs
GPR1831	0	5.61353736	0.685	0.019	0	DCs
CD1C1	0	10.5568927	0.659	0.002	0	DCs
NDRG22	0	7.46014024	0.653	0.01	0	DCs
CCR61	0	6.90760399	0.65	0.01	0	DCs
CLEC10A2	0	7.93583405	0.644	0.011	0	DCs
LIMD22	0	4.76668373	0.653	0.026	0	DCs
ADAM82	0	5.35084571	0.64	0.018	0	DCs
PALM2-AKAP2	0	5.44195875	0.681	0.075	0	DCs
FCER1A1	0	10.7477941	0.603	0.002	0	DCs
PLXNC12	0	5.4648063	0.631	0.036	0	DCs
GNAO11	0	8.21768616	0.596	0.004	0	DCs

PKIB1	0	9.18252677	0.596	0.005	0	DCs
ADAM191	0	4.25008269	0.603	0.017	0	DCs
CST71	0	5.35281201	0.596	0.013	0	DCs
FCGR2B2	0	6.63489342	0.603	0.026	0	DCs
LGALS22	0	8.30422326	0.584	0.013	0	DCs
GRASP2	0	6.6348865	0.571	0.022	0	DCs
SULF22	0	8.05488088	0.549	0.004	0	DCs
PALD12	0	6.06155084	0.577	0.04	0	DCs
MCOLN21	0	5.23032195	0.603	0.067	0	DCs
PRKCB2	0	4.41479517	0.584	0.055	0	DCs
P2RY62	0	7.15501386	0.539	0.016	0	DCs
GPR1322	0	4.69437474	0.58	0.059	0	DCs
P2RY141	0	5.72924078	0.577	0.06	0	DCs
PARM11	0	9.96595341	0.517	0.001	0	DCs
CBFA2T32	0	7.11363045	0.502	0.014	0	DCs
CD1E1	0	10.6464903	0.467	0.001	0	DCs
CCL171	0	10.5966162	0.445	0.001	0	DCs
ZBTB461	0	5.89543447	0.476	0.035	0	DCs
ANTXR22	0	4.63381545	0.464	0.026	0	DCs
ETV52	0	6.98360351	0.448	0.017	0	DCs
ADAM282	0	7.07925705	0.429	0.009	0	DCs
SLC38A1	0	3.43439366	0.438	0.018	0	DCs
IRF41	0	6.01202247	0.429	0.012	0	DCs
CCND21	0	3.51164659	0.432	0.022	0	DCs
PLD41	0	8.05061191	0.41	0.002	0	DCs
SLC2A12	0	5.54540157	0.416	0.017	0	DCs
FLT31	0	10.2105107	0.391	0.001	0	DCs
S100B1	0	9.0776139	0.388	0.004	0	DCs
GAS62	0	6.39119149	0.388	0.014	0	DCs
IL1R12	0	5.68588239	0.404	0.033	0	DCs
P2RY10	0	4.59200412	0.379	0.008	0	DCs
MS4A4E2	0	6.02271001	0.394	0.03	0	DCs
CLEC4F1	0	11.0852584	0.363	0	0	DCs
ACAP11	0	2.86417015	0.382	0.026	0	DCs
SLC30A41	0	5.64633926	0.382	0.027	0	DCs
CD1A1	0	10.9067154	0.353	0	0	DCs
CCSER11	0	9.30552203	0.347	0.002	0	DCs
P3H21	0	9.02596282	0.344	0.001	0	DCs
CCR22	0	4.30415343	0.353	0.015	0	DCs
IL21R2	0	4.83324129	0.356	0.018	0	DCs
ACPP2	0	7.56784722	0.334	0.004	0	DCs
DUSP41	0	5.38129646	0.328	0.01	0	DCs
MYO1D1	0	5.05791748	0.325	0.011	0	DCs
SCN9A2	0	6.16551919	0.312	0.015	0	DCs
CD200R11	0	5.06684798	0.3	0.006	0	DCs
CLDN13	0	8.43175206	0.293	0.003	0	DCs
CCL221	0	9.51319419	0.293	0.012	0	DCs
LAMP31	0	8.89229444	0.265	0.006	0	DCs
PPP1R14A1	0	10.0256529	0.246	0	0	DCs
IDO11	0	7.43960142	0.243	0.005	0	DCs
MYCL1	0	7.8931851	0.23	0.002	0	DCs
IL18R11	0	4.68268747	0.23	0.005	0	DCs

ENPP11	0	9.73608513	0.221	0	0	DCs
TACSTD21	0	7.0601061	0.221	0.003	0	DCs
CD2071	0	11.5956299	0.215	0	0	DCs
CLEC9A1	0	10.8213544	0.215	0.002	0	DCs
DEPTOR2	0	6.54065578	0.215	0.007	0	DCs
F13A12	0	5.42853516	0.211	0.004	0	DCs
CX3CR11	0	8.04379862	0.205	0.001	0	DCs
EPHB31	0	7.5592092	0.202	0.002	0	DCs
TLR101	0	7.19722554	0.196	0.004	0	DCs
TOX1	0	4.65441152	0.192	0.003	0	DCs
AUTS2	0	4.48511163	0.192	0.005	0	DCs
CLNK	0	7.32283607	0.18	0.001	0	DCs
CHST22	0	6.83099456	0.18	0.003	0	DCs
XCR1	0	12.784167	0.174	0	0	DCs
IDO21	0	10.0387582	0.174	0	0	DCs
RAMP11	0	8.32206221	0.167	0.001	0	DCs
DNASE1L3	0	9.57322898	0.167	0.001	0	DCs
ENHO2	0	7.7470967	0.167	0.002	0	DCs
MTSS11	0	5.28805434	0.167	0.005	0	DCs
SNX22	0	9.17785589	0.161	0.001	0	DCs
CCND11	0	6.66258807	0.164	0.005	0	DCs
GPR681	0	4.30420887	0.164	0.005	0	DCs
CD1B1	0	9.74192992	0.158	0	0	DCs
FAM149A1	0	8.39209283	0.155	0.001	0	DCs
SLC7A111	0	7.774663	0.151	0.001	0	DCs
MBOAT21	0	5.72015088	0.145	0.003	0	DCs
NCKAP51	0	8.44587192	0.142	0.001	0	DCs
ADRB31	0	9.13837953	0.142	0.001	0	DCs
CHST151	0	6.00151119	0.139	0.002	0	DCs
MMP251	0	5.99922272	0.136	0.001	0	DCs
SLCO5A1	0	10.2545863	0.126	0	0	DCs
ADGRG61	0	7.14478328	0.126	0.002	0	DCs
TNFRSF11A1	0	7.44960034	0.123	0.001	0	DCs
AFF31	0	6.7863043	0.123	0.002	0	DCs
SLC35F21	0	4.76432083	0.123	0.002	0	DCs
TNFAIP8L31	0	8.08647931	0.12	0.001	0	DCs
ASGR21	0	6.71986933	0.12	0.002	0	DCs
DBN1	0	6.36320627	0.12	0.003	0	DCs
CCR71	0	6.69379773	0.12	0.003	0	DCs
EGLN3	0	8.54381796	0.117	0.001	0	DCs
PID11	0	7.46441117	0.114	0.001	0	DCs
RAB15	0	6.20829413	0.11	0.001	0	DCs
PTGIR1	0	8.09018806	0.11	0.001	0	DCs
CYYR1	0	9.08445038	0.107	0	0	DCs
FBXO27	0	8.61428781	0.107	0.001	0	DCs
LAD1	0	8.00333555	0.101	0.002	0	DCs
MYLK1	0	7.08680764	0.101	0.002	0	DCs
RASAL11	0	8.52153664	0.088	0	0	DCs
EBF4	0	8.40987761	0.085	0	0	DCs
SLC4A3	0	9.08566696	0.085	0	0	DCs
CLEC4C	0	8.23936823	0.085	0	0	DCs
HTR71	0	8.07791415	0.085	0.001	0	DCs

LIMCH11	0	7.51571535	0.076	0.001	0	DCs
NEGR1	0	8.42211619	0.073	0	0	DCs
ANKRD33B	0	8.36820649	0.073	0	0	DCs
ADAMTS101	0	6.44626622	0.069	0.001	0	DCs
GPER1	0	10.2634287	0.066	0	0	DCs
BTLA	0	6.56874633	0.057	0	0	DCs
CHRNA61	0	9.37270707	0.054	0	0	DCs
SPIB	0	7.25426516	0.054	0	0	DCs
ABCB41	0	8.43923384	0.047	0	0	DCs
CNR2	0	6.7694124	0.044	0	0	DCs
ELAVL41	0	8.2488232	0.044	0	0	DCs
SPNS3	0	6.84773725	0.041	0	0	DCs
TNNT2	0	8.47401928	0.041	0	0	DCs
UTF11	0	9.25773347	0.041	0	0	DCs
TMEM132E	0	14.0074431	0.035	0	0	DCs
SLC12A3	0	8.1917827	0.032	0	0	DCs
KCNN1	0	11.9686471	0.032	0	0	DCs
CLEC14A	0	11.9128262	0.032	0	0	DCs
CD200	0	8.6341944	0.028	0	0	DCs
KCND3	0	11.7122711	0.022	0	0	DCs
GPR31	0	9.14799289	0.022	0	0	DCs
CPXM1	0	9.52787448	0.022	0	0	DCs
FIGLA	0	9.53613418	0.019	0	0	DCs
NLRP4	0	12.1069616	0.016	0	0	DCs
NFATC21	2.624E-303	3.61647549	0.498	0.05	4.034E-299	DCs
IL1R21	2.801E-301	5.14881969	0.107	0.002	4.306E-297	DCs
GPR33	1.63E-298	9.17217715	0.032	0	2.506E-294	DCs
AMPD32	7.316E-294	4.78841853	0.278	0.016	1.125E-289	DCs
ZEB1	8.144E-294	3.59683109	0.158	0.005	1.252E-289	DCs
CASQ1	1.97E-291	7.93167022	0.025	0	3.028E-287	DCs
PPP1R16B	3.151E-286	3.3954674	0.189	0.008	4.843E-282	DCs
FUT72	7.796E-285	5.44627604	0.309	0.021	1.198E-280	DCs
SEMA4C1	4.985E-283	4.56668571	0.104	0.002	7.662E-279	DCs
TVP23A2	1.238E-276	6.7221983	0.199	0.009	1.904E-272	DCs
MEF2C2	8.088E-268	5.25576794	0.533	0.068	1.243E-263	DCs
NCALD	1.231E-265	4.79748815	0.095	0.002	1.893E-261	DCs
BASP12	2.364E-265	5.00758864	0.555	0.074	3.634E-261	DCs
C6orf2231	3.513E-258	7.10566791	0.041	0	5.399E-254	DCs
GNG71	3.941E-256	6.38400343	0.126	0.004	6.058E-252	DCs
KIF26B	1.72E-254	7.59399542	0.032	0	2.643E-250	DCs
PRSS3	4.758E-252	9.58710651	0.019	0	7.313E-248	DCs
ATP1A4	6.112E-249	7.7415095	0.028	0	9.394E-245	DCs
GABBR12	4.248E-247	4.91667981	0.271	0.018	6.529E-243	DCs
BCL11A2	4.457E-245	5.12151125	0.476	0.058	6.851E-241	DCs
GPR1412	1.102E-244	5.50937249	0.3	0.023	1.694E-240	DCs
CADM11	9.933E-240	7.41200109	0.18	0.008	1.527E-235	DCs
CHST1	4.462E-239	9.17296316	0.016	0	6.859E-235	DCs
ST8SIA42	7.073E-234	3.89386415	0.555	0.082	1.087E-229	DCs
SLC7A52	3.709E-233	4.34060864	0.451	0.053	5.7E-229	DCs
GNG21	5.379E-233	2.43791081	0.271	0.019	8.268E-229	DCs
PTPN71	2.569E-232	3.30406311	0.287	0.021	3.949E-228	DCs
ACHE	1.226E-226	8.81404971	0.032	0	1.885E-222	DCs

BCL7A	1.701E-223	6.22670198	0.057	0.001	2.615E-219	DCs
PAWR	1.97E-223	6.15110035	0.047	0.001	3.028E-219	DCs
CD2261	3.367E-223	3.65093185	0.224	0.014	5.176E-219	DCs
FSCN11	7.612E-223	8.75413554	0.151	0.006	1.17E-218	DCs
FBXO17	1.474E-222	7.25227174	0.028	0	2.266E-218	DCs
HIC11	1.58E-221	5.59092553	0.069	0.001	2.429E-217	DCs
ZNF4672	2.567E-221	5.67589355	0.202	0.011	3.946E-217	DCs
CDH2	3.757E-219	8.97928599	0.016	0	5.775E-215	DCs
FAM118A1	2.959E-213	4.45256372	0.265	0.02	4.548E-209	DCs
NRARP1	6.888E-213	6.10588766	0.23	0.015	1.059E-208	DCs
CYP2S12	3.606E-210	4.47815924	0.58	0.103	5.543E-206	DCs
VENTX2	2.348E-209	5.45442695	0.174	0.009	3.609E-205	DCs
AOC1	3.901E-209	8.08484508	0.025	0	5.997E-205	DCs
TRABD2A1	8.067E-208	4.83535382	0.104	0.003	1.24E-203	DCs
CYBRD12	1.497E-206	4.94118034	0.287	0.024	2.301E-202	DCs
CORO1A2	4.318E-206	3.28818678	0.789	0.208	6.636E-202	DCs
HDAC92	2.048E-205	4.7217044	0.505	0.078	3.148E-201	DCs
CRLF2	1.773E-196	7.85824959	0.028	0	2.725E-192	DCs
ZNF608	7.942E-194	6.67634723	0.038	0	1.221E-189	DCs
ROR1	4.2E-191	7.59956782	0.025	0	6.455E-187	DCs
RHEX	4.67E-191	6.45552153	0.025	0	7.178E-187	DCs
ZNF462	8.379E-191	6.19044279	0.044	0.001	1.288E-186	DCs
ATP8B42	1.108E-190	4.93736278	0.353	0.04	1.703E-186	DCs
SERPINF11	3.926E-190	5.0085418	0.495	0.08	6.035E-186	DCs
BEND5	1.646E-187	7.18193795	0.035	0	2.53E-183	DCs
UPK3A	2.008E-187	9.19796065	0.013	0	3.086E-183	DCs
ENTPD11	1.535E-185	4.26887014	0.855	0.339	2.359E-181	DCs
MRC22	7.311E-184	5.69032915	0.129	0.006	1.124E-179	DCs
XYLT11	9.503E-184	4.37104546	0.202	0.013	1.461E-179	DCs
SH3RF2	3.435E-183	7.18669159	0.066	0.001	5.28E-179	DCs
ST6GAL11	2.666E-182	3.61891981	0.41	0.055	4.098E-178	DCs
MYEF2	4.49E-182	4.77623993	0.205	0.014	6.901E-178	DCs
EBI31	1.034E-178	7.35083983	0.063	0.001	1.589E-174	DCs
PLEKHO12	1.132E-177	3.46630975	0.909	0.421	1.74E-173	DCs
DSG2	8.467E-177	7.20381684	0.035	0	1.301E-172	DCs
FZD22	2.172E-174	5.44425581	0.189	0.013	3.338E-170	DCs
FGL22	8.072E-172	4.10847186	0.909	0.486	1.241E-167	DCs
PLEKHG4B	1.102E-171	6.98338864	0.019	0	1.694E-167	DCs
ITPRIPL11	2.579E-171	3.69252294	0.104	0.004	3.964E-167	DCs
MAP3K141	1.662E-169	5.10681186	0.338	0.04	2.554E-165	DCs
MYBL11	1.275E-168	4.09692494	0.23	0.019	1.961E-164	DCs
ANKRD222	1.783E-168	5.56876899	0.17	0.011	2.741E-164	DCs
CSF2RA2	2.42E-168	2.7911815	0.978	0.875	3.72E-164	DCs
MMP121	1.037E-167	7.78334615	0.041	0.001	1.593E-163	DCs
TRERF11	8.349E-167	3.46917047	0.196	0.014	1.283E-162	DCs
TIFAB1	3.272E-166	6.12575031	0.18	0.012	5.03E-162	DCs
EMB1	3.273E-165	2.63159397	0.256	0.023	5.031E-161	DCs
ENOX1	3.243E-162	8.10561119	0.028	0	4.985E-158	DCs
AKAP61	1.888E-159	6.26078823	0.057	0.001	2.902E-155	DCs
ALDH1L22	3.034E-155	5.53354593	0.183	0.013	4.663E-151	DCs
PEA152	1.728E-153	2.9179814	0.95	0.752	2.656E-149	DCs
KLHL52	1.104E-152	3.73471898	0.7	0.221	1.697E-148	DCs

ARHGAP451	2.378E-152	3.30506914	0.615	0.153	3.655E-148	DCs
CLEC2D1	7.768E-152	2.35632971	0.366	0.049	1.194E-147	DCs
C1orf115	1.048E-151	6.7943242	0.047	0.001	1.611E-147	DCs
CMTM8	1.465E-149	4.98782629	0.057	0.001	2.251E-145	DCs
ITM2C	1.271E-148	3.00713411	0.17	0.012	1.954E-144	DCs
TMEM711	1.346E-148	3.56519264	0.246	0.024	2.069E-144	DCs
SLAMF71	8.237E-147	4.71857899	0.265	0.028	1.266E-142	DCs
IL1RL21	5.037E-146	6.37664945	0.05	0.001	7.742E-142	DCs
CIITA2	5.648E-146	2.72687055	0.95	0.812	8.682E-142	DCs
CARD10	3.196E-145	8.59876853	0.019	0	4.912E-141	DCs
SEMA7A1	1.194E-142	5.49395978	0.274	0.031	1.836E-138	DCs
NLRP32	1.804E-142	4.56718397	0.369	0.056	2.773E-138	DCs
BCO21	1.75E-140	5.50165217	0.104	0.005	2.69E-136	DCs
BCL21	7.777E-134	2.76939427	0.404	0.068	1.195E-129	DCs
LTC4S1	1.7E-133	5.67855189	0.079	0.003	2.613E-129	DCs
MPPED2	1.851E-131	8.01046427	0.016	0	2.846E-127	DCs
APCDD1	2.13E-129	6.82477317	0.028	0	3.275E-125	DCs
DRGX	3.224E-128	7.95146619	0.025	0	4.956E-124	DCs
CRIP22	4.018E-128	4.25566866	0.158	0.012	6.176E-124	DCs
SLC22A23	6.7E-127	6.0101886	0.057	0.002	1.03E-122	DCs
ASAP22	1.545E-126	5.84160238	0.073	0.003	2.375E-122	DCs
TMEM1542	4.647E-126	4.22810882	0.274	0.035	7.143E-122	DCs
CDH232	1.847E-125	5.60579049	0.139	0.009	2.838E-121	DCs
FAM20A2	2.777E-125	5.1157376	0.177	0.015	4.268E-121	DCs
C12orf75	2.395E-124	3.02033968	0.11	0.006	3.681E-120	DCs
SLC8A12	5.338E-124	2.83399743	0.915	0.856	8.206E-120	DCs
ZFP36L12	1.144E-123	2.86211829	0.88	0.556	1.758E-119	DCs
HMSD	1.35E-120	7.23487432	0.013	0	2.075E-116	DCs
KCTD152	2.715E-118	4.95548176	0.129	0.009	4.173E-114	DCs
TMEM156	2.35E-117	3.93936261	0.082	0.004	3.612E-113	DCs
CD801	2.502E-116	4.20294378	0.744	0.356	3.846E-112	DCs
CLEC17A1	4.086E-116	6.34079862	0.032	0.001	6.28E-112	DCs
HIST1H2BG2	1.045E-115	4.39721731	0.189	0.018	1.607E-111	DCs
FNBP1L1	3.517E-115	5.25558954	0.11	0.006	5.406E-111	DCs
AHR2	1.863E-114	2.66478977	0.902	0.794	2.864E-110	DCs
GPR39	2.725E-114	7.66312568	0.016	0	4.188E-110	DCs
CRIP3	1.591E-113	6.80643236	0.032	0.001	2.446E-109	DCs
PICALM2	2.213E-113	1.57778629	0.959	0.956	3.401E-109	DCs
TCF42	4.893E-113	4.48452847	0.388	0.076	7.521E-109	DCs
AMOT1	2.267E-112	6.07381565	0.101	0.006	3.484E-108	DCs
PPP2R2B1	5.176E-111	3.70939855	0.085	0.004	7.956E-107	DCs
TRAF11	1.037E-110	4.16493209	0.297	0.044	1.593E-106	DCs
ITGA42	1.978E-110	2.90701568	0.814	0.491	3.041E-106	DCs
C1orf211	1.333E-108	5.64601043	0.142	0.011	2.049E-104	DCs
SHE1	7.301E-108	6.51207386	0.054	0.002	1.122E-103	DCs
MEIKIN1	1.466E-107	6.12867483	0.066	0.003	2.253E-103	DCs
GCSAM	3.382E-107	5.55971835	0.158	0.014	5.199E-103	DCs
POU6F11	7.966E-107	3.68908237	0.066	0.003	1.224E-102	DCs
CLIC3	9.306E-107	3.97283322	0.066	0.003	1.43E-102	DCs
KLF61	1.962E-106	2.17574016	0.937	0.821	3.016E-102	DCs
RUNX21	5.749E-106	3.53112173	0.356	0.067	8.836E-102	DCs
TESPA1	8.467E-106	3.02218125	0.12	0.008	1.301E-101	DCs

NAV11	9.089E-106	4.23540268	0.524	0.153	1.397E-101	DCs
CHAC11	2.217E-105	4.87928701	0.063	0.002	3.408E-101	DCs
KDM7A2	2.457E-105	3.67732719	0.445	0.106	3.777E-101	DCs
HSPA62	8.645E-105	4.4300749	0.64	0.253	1.329E-100	DCs
GGT51	8.734E-105	5.78037673	0.028	0	1.343E-100	DCs
NME81	8.291E-104	5.9347631	0.054	0.002	1.274E-99	DCs
EDEM11	2.704E-102	2.11011657	0.905	0.81	4.1556E-98	DCs
CD300LB1	3.128E-102	5.1360655	0.18	0.019	4.8085E-98	DCs
TNFSF18	3.219E-102	8.23180965	0.022	0	4.9476E-98	DCs
CFLAR1	4.63E-102	2.01219751	0.909	0.876	7.116E-98	DCs
PLEK22	4.925E-101	5.22494834	0.202	0.023	7.571E-97	DCs
CYTIP	6.071E-101	1.72552813	0.943	0.849	9.3317E-97	DCs
BCL62	1.733E-99	3.35561668	0.763	0.448	2.6642E-95	DCs
UCK22	1.8907E-98	4.29848991	0.224	0.029	2.9062E-94	DCs
ST181	3.0192E-98	7.46460836	0.022	0	4.6408E-94	DCs
RAB33A	7.4175E-98	3.98165687	0.079	0.004	1.1401E-93	DCs
MS4A6A2	7.6405E-98	2.69084543	0.868	0.821	1.1744E-93	DCs
HAPLN31	1.9464E-97	3.95765293	0.205	0.025	2.9918E-93	DCs
ARL4C2	2.3153E-97	3.07005818	0.524	0.157	3.5589E-93	DCs
WASF3	2.7552E-96	7.4019572	0.025	0	4.235E-92	DCs
CXCR41	4.6454E-96	3.23995137	0.732	0.357	7.1404E-92	DCs
CMTM41	1.2354E-94	5.14274057	0.069	0.003	1.8989E-90	DCs
NEURL31	3.2117E-92	7.56969499	0.025	0	4.9367E-88	DCs
OSBP2	6.6218E-92	5.63186645	0.028	0.001	1.0178E-87	DCs
SGK12	1.5456E-91	3.1390829	0.83	0.657	2.3758E-87	DCs
SMAD31	1.1649E-90	2.53212607	0.114	0.008	1.7906E-86	DCs
HSH2D2	4.477E-90	3.72639733	0.319	0.061	6.8816E-86	DCs
POU2F22	3.1265E-89	4.29196418	0.205	0.027	4.8058E-85	DCs
ATP2A31	3.9888E-89	2.92156522	0.303	0.055	6.1312E-85	DCs
AHNAK3	5.8974E-89	1.34367437	0.978	0.979	9.0649E-85	DCs
ANKRD441	1.1243E-88	2.58870756	0.751	0.458	1.7282E-84	DCs
SATB12	1.1451E-88	3.32454894	0.394	0.094	1.7601E-84	DCs
NDUFA4L21	7.9361E-88	5.45660032	0.073	0.004	1.2199E-83	DCs
ESAM	1.5279E-87	5.91251067	0.05	0.002	2.3485E-83	DCs
CCL19	3.6384E-87	7.37863552	0.035	0.001	5.5926E-83	DCs
DPYSL21	6.9919E-87	1.25304052	0.962	0.954	1.0747E-82	DCs
TLR32	7.5859E-86	5.24444604	0.174	0.02	1.166E-81	DCs
TPPP32	2.0489E-85	3.59309647	0.098	0.007	3.1493E-81	DCs
AKNA1	6.7596E-85	2.2044074	0.789	0.552	1.039E-80	DCs
DAPK2	2.6824E-84	3.40113155	0.085	0.005	4.1232E-80	DCs
PAK12	3.1368E-84	3.04483984	0.713	0.428	4.8216E-80	DCs
CSGALNACT11	1.1084E-83	3.18127423	0.073	0.004	1.7037E-79	DCs
RHOF1	6.2414E-83	3.38429426	0.391	0.098	9.5937E-79	DCs
METRNL2	7.327E-83	2.1010182	0.871	0.856	1.1262E-78	DCs
CCR52	2.8846E-81	2.35352991	0.325	0.067	4.4339E-77	DCs
NCCRP1	9.2675E-81	5.6389213	0.025	0	1.4245E-76	DCs
PXN1	1.4952E-80	3.24068647	0.189	0.025	2.2983E-76	DCs
LINGO3	1.788E-80	7.9379814	0.013	0	2.7484E-76	DCs
TNFSF41	2.1292E-80	5.07913519	0.088	0.006	3.2728E-76	DCs
CLEC5A2	4.4152E-80	3.90208804	0.527	0.192	6.7867E-76	DCs
ADAM12	4.4448E-80	4.86041221	0.047	0.002	6.8321E-76	DCs
SMAGP	4.5336E-80	4.83426074	0.035	0.001	6.9685E-76	DCs

DPP4	1.0655E-79	2.14106498	0.114	0.009	1.6378E-75	DCs
CDKN1A1	5.6332E-79	2.98572878	0.694	0.337	8.6588E-75	DCs
RNF1501	1.4255E-78	6.93335517	0.038	0.001	2.1912E-74	DCs
P2RY132	1.6411E-78	3.42644693	0.621	0.294	2.5226E-74	DCs
CD932	3.9297E-78	3.82503502	0.36	0.088	6.0403E-74	DCs
ATP2B41	7.1407E-78	2.77406929	0.606	0.266	1.0976E-73	DCs
PROC1	7.3341E-78	6.00656417	0.025	0.001	1.1273E-73	DCs
CLECL11	1.2439E-77	4.43380651	0.224	0.036	1.912E-73	DCs
TRAF41	1.8643E-77	3.93172753	0.183	0.024	2.8656E-73	DCs
CD481	3.3892E-77	2.38280286	0.35	0.08	5.2095E-73	DCs
PTGS12	3.966E-77	3.57072482	0.524	0.198	6.0961E-73	DCs
MARCKSL11	7.7688E-77	5.61531393	0.199	0.029	1.1941E-72	DCs
BHLHE401	1.4214E-76	1.38378179	0.953	0.926	2.1849E-72	DCs
IGFLR11	2.3025E-75	2.51486479	0.729	0.486	3.5391E-71	DCs
LTB	2.342E-75	3.09910728	0.517	0.189	3.6E-71	DCs
LSR1	4.5716E-75	3.99532446	0.085	0.006	7.027E-71	DCs
CTLA4	2.9445E-74	2.50234438	0.076	0.005	4.526E-70	DCs
IGKC	4.2095E-74	3.29466715	0.035	0.001	6.4703E-70	DCs
KCNK17	1.7635E-73	6.45355805	0.013	0	2.7107E-69	DCs
WIPF11	2.6045E-73	1.33722218	0.912	0.945	4.0034E-69	DCs
PABPN1L	5.9717E-73	8.26587813	0.016	0	9.1792E-69	DCs
AFDN2	2.0318E-72	2.51714272	0.735	0.525	3.1231E-68	DCs
CELF21	4.8248E-72	1.48906219	0.871	0.861	7.4162E-68	DCs
IGSF3	1.1396E-71	7.06690294	0.022	0	1.7517E-67	DCs
SLC6A9	2.1348E-71	5.21747261	0.032	0.001	3.2814E-67	DCs
RIPOR22	6.4866E-71	3.09398706	0.322	0.075	9.9705E-67	DCs
LPAR51	8.672E-71	3.74781578	0.341	0.086	1.333E-66	DCs
DOCK52	1.5962E-70	2.82570734	0.606	0.293	2.4535E-66	DCs
BTG21	5.2333E-70	1.85531547	0.858	0.754	8.044E-66	DCs
EIF11	1.3198E-69	1.27687128	0.94	0.94	2.0286E-65	DCs
DOCK102	7.9291E-69	1.98547555	0.792	0.706	1.2188E-64	DCs
FPR32	8.7327E-69	3.10889281	0.71	0.493	1.3423E-64	DCs
DUSP51	8.9516E-69	4.13611079	0.297	0.067	1.3759E-64	DCs
EGR31	1.5961E-68	4.0090819	0.073	0.005	2.4533E-64	DCs
LRMP1	4.2505E-67	3.72430201	0.407	0.128	6.5334E-63	DCs
SCRN12	5.3164E-67	3.27472461	0.454	0.16	8.1718E-63	DCs
CLDN9	1.9395E-66	6.3349152	0.028	0.001	2.9811E-62	DCs
STK17B	8.1461E-66	1.67400208	0.836	0.804	1.2521E-61	DCs
STAMBPL11	1.4094E-65	3.82174601	0.123	0.013	2.1665E-61	DCs
HIST1H2AG	1.6055E-65	4.89612714	0.069	0.004	2.4678E-61	DCs
BCL2L112	1.7997E-65	3.15310984	0.584	0.286	2.7663E-61	DCs
LENG81	2E-65	1.72475273	0.814	0.65	3.0743E-61	DCs
TRIB12	2.2899E-65	2.63572858	0.719	0.525	3.5198E-61	DCs
OTOA	9.0712E-65	7.00536159	0.019	0	1.3943E-60	DCs
MARCKS1	1.7579E-64	3.78298499	0.331	0.085	2.7021E-60	DCs
SRGAP12	2.6291E-64	5.33085723	0.069	0.005	4.0413E-60	DCs
USP531	5.2341E-64	4.18933282	0.325	0.085	8.0454E-60	DCs
EHBP1L11	5.4668E-64	1.21011832	0.946	0.95	8.4031E-60	DCs
NAAA1	4.7938E-62	2.87029546	0.722	0.574	7.3686E-58	DCs
S1PR22	6.7999E-62	3.93541906	0.353	0.103	1.0452E-57	DCs
RNF19B1	1.4135E-61	1.88049901	0.789	0.717	2.1727E-57	DCs
ENPP21	1.5076E-61	4.33763381	0.237	0.047	2.3173E-57	DCs

RGS182	2.2321E-61	3.44185525	0.379	0.118	3.4309E-57	DCs
ACY3	5.007E-61	5.85861537	0.016	0	7.6962E-57	DCs
SYNJ22	6.2923E-61	3.82259857	0.095	0.009	9.6718E-57	DCs
LRRK11	9.6835E-61	2.75141309	0.637	0.393	1.4884E-56	DCs
GPR171	1.0448E-60	2.02497192	0.101	0.01	1.606E-56	DCs
PMAIP11	4.4418E-60	3.97905585	0.218	0.041	6.8275E-56	DCs
KIT	3.2116E-59	3.25108403	0.025	0.001	4.9365E-55	DCs
CD742	6.4692E-59	0.58237381	1	0.995	9.9438E-55	DCs
GSN2	8.3312E-59	1.11574408	0.968	0.935	1.2806E-54	DCs
JAK31	9.055E-59	2.83715548	0.278	0.065	1.3918E-54	DCs
SLC37A31	1.0753E-58	2.92614021	0.073	0.005	1.6529E-54	DCs
OGFRL11	1.2657E-58	2.32110865	0.751	0.668	1.9455E-54	DCs
KCNN42	2.3599E-58	3.62569361	0.274	0.065	3.6274E-54	DCs
NXPH3	2.5377E-58	7.80190025	0.013	0	3.9007E-54	DCs
HRH22	3.5279E-58	4.48420361	0.057	0.003	5.4227E-54	DCs
STK38L2	5.4665E-58	2.82234748	0.612	0.363	8.4026E-54	DCs
TRAF3IP31	1.129E-57	1.79293589	0.192	0.033	1.7354E-53	DCs
RGS102	3.7457E-57	2.69784457	0.681	0.513	5.7575E-53	DCs
FAM160A1	8.2255E-57	5.62260261	0.019	0	1.2643E-52	DCs
CEBPD2	1.4806E-56	3.14811395	0.634	0.408	2.2758E-52	DCs
RGS11	2.1816E-56	2.47415258	0.707	0.488	3.3534E-52	DCs
EMP12	4.9846E-56	2.98626185	0.394	0.131	7.6619E-52	DCs
ARHGEF402	6.3062E-56	3.57230943	0.315	0.088	9.6933E-52	DCs
ICAM51	1.9181E-55	5.61112319	0.076	0.006	2.9484E-51	DCs
FZD3	2.1247E-55	4.8739285	0.028	0.001	3.2658E-51	DCs
PIK3R62	2.4182E-55	4.16349146	0.186	0.033	3.717E-51	DCs
REL1	5.3264E-55	2.50649059	0.767	0.708	8.1872E-51	DCs
NFKB21	7.0965E-55	3.04532337	0.486	0.209	1.0908E-50	DCs
LMNB11	1.939E-54	2.88280482	0.524	0.249	2.9804E-50	DCs
PLEKHA51	5.2296E-54	3.48107276	0.177	0.03	8.0384E-50	DCs
SLC9A71	1.2262E-53	3.31241354	0.483	0.218	1.8848E-49	DCs
CNN22	2.6773E-53	1.499419	0.817	0.803	4.1153E-49	DCs
TMEM121B2	2.6913E-53	5.38874914	0.044	0.002	4.1368E-49	DCs
GNAQ2	3.9294E-53	1.38843959	0.842	0.876	6.0398E-49	DCs
CCDC102A	9.4385E-53	3.57091821	0.028	0.001	1.4508E-48	DCs
CHD32	1.1721E-52	2.6759845	0.347	0.107	1.8017E-48	DCs
ETV62	1.3159E-52	2.01645008	0.751	0.707	2.0226E-48	DCs
RELB1	1.75E-52	3.26782778	0.413	0.153	2.6899E-48	DCs
SLC9A92	2.1044E-52	4.28277378	0.088	0.009	3.2347E-48	DCs
TAP12	4.8049E-52	1.90839527	0.776	0.73	7.3856E-48	DCs
STK262	6.6049E-52	3.10861996	0.205	0.041	1.0152E-47	DCs
MAPK111	1.3709E-51	4.00109335	0.066	0.005	2.1071E-47	DCs
PTPRK	1.8342E-51	5.24771969	0.028	0.001	2.8193E-47	DCs
ZFP362	2.4028E-51	1.57006494	0.861	0.871	3.6933E-47	DCs
ZNF2961	5.4405E-51	4.29931268	0.205	0.042	8.3626E-47	DCs
RSRP11	6.4063E-51	1.3942881	0.817	0.711	9.8472E-47	DCs
P2RY8	1.6102E-50	2.79066827	0.079	0.007	2.4751E-46	DCs
SEMA4A2	1.6864E-50	3.19276245	0.454	0.198	2.5921E-46	DCs
ALCAM2	1.7644E-50	2.25343698	0.735	0.682	2.712E-46	DCs
CLIC22	4.2694E-50	3.02342924	0.536	0.29	6.5625E-46	DCs
CSRP21	6.8065E-50	4.470556	0.142	0.022	1.0462E-45	DCs
ASB2	1.534E-49	4.7465628	0.022	0.001	2.3578E-45	DCs

RFTN11	2.3924E-49	3.36290637	0.448	0.195	3.6773E-45	DCs
PANX2	4.7239E-49	5.27081446	0.022	0.001	7.2612E-45	DCs
EMP21	1.1977E-48	4.77829361	0.054	0.004	1.841E-44	DCs
TNFAIP81	1.5046E-48	2.22144152	0.644	0.465	2.3127E-44	DCs
GPAT31	1.6941E-48	3.97396817	0.271	0.074	2.604E-44	DCs
ABCA61	1.7504E-48	5.65043615	0.063	0.005	2.6906E-44	DCs
SNCA2	8.8024E-48	3.43246209	0.271	0.074	1.353E-43	DCs
SH3KBP12	1.4478E-47	2.37475395	0.606	0.405	2.2254E-43	DCs
LSP12	1.967E-47	0.9544882	0.956	0.945	3.0235E-43	DCs
KDM2A1	2.0913E-47	1.28735853	0.817	0.82	3.2145E-43	DCs
GNAZ	2.9105E-47	5.45079735	0.054	0.004	4.4738E-43	DCs
PCNX22	2.996E-47	2.90814097	0.325	0.104	4.6051E-43	DCs
ELOVL51	1.2554E-46	2.29610755	0.694	0.59	1.9297E-42	DCs
C15orf482	1.5402E-46	3.06930625	0.445	0.186	2.3674E-42	DCs
CERS41	1.5944E-46	3.17574276	0.057	0.004	2.4508E-42	DCs
SYNE32	1.7958E-46	2.77779968	0.372	0.134	2.7604E-42	DCs
TTYH21	3.3952E-46	4.07808145	0.256	0.068	5.2187E-42	DCs
TMEM121	3.7048E-46	5.00206498	0.019	0	5.6946E-42	DCs
TRAF51	7.738E-46	3.01311799	0.265	0.072	1.1894E-41	DCs
UVRAG1	9.7554E-46	2.60744423	0.59	0.398	1.4995E-41	DCs
HERPUD11	1.0968E-45	1.86883213	0.748	0.74	1.6859E-41	DCs
ZNF366	1.2029E-45	4.10013407	0.262	0.072	1.8489E-41	DCs
PON21	7.4745E-45	3.11427976	0.495	0.265	1.1489E-40	DCs
IFFO21	1.338E-44	2.71433377	0.35	0.125	2.0567E-40	DCs
STK41	1.7151E-44	1.45308207	0.798	0.837	2.6363E-40	DCs
RNASE62	1.8836E-44	2.87530183	0.536	0.308	2.8952E-40	DCs
ABCA91	2.1482E-44	6.30438449	0.016	0	3.3021E-40	DCs
ARF61	2.6735E-44	1.10391134	0.836	0.89	4.1095E-40	DCs
SYNGR22	4.5817E-44	0.87361086	0.981	0.965	7.0425E-40	DCs
ADM21	4.7168E-44	4.75639469	0.035	0.002	7.2503E-40	DCs
PPM1J	5.9176E-44	4.67142856	0.174	0.035	9.0959E-40	DCs
HIST1H1B	8.8367E-44	1.23746074	0.23	0.055	1.3583E-39	DCs
LILRA12	1.2786E-43	4.63954479	0.091	0.011	1.9654E-39	DCs
SLC39A102	1.4608E-43	1.87301078	0.716	0.656	2.2454E-39	DCs
BIK	2.1896E-43	4.66961865	0.073	0.007	3.3657E-39	DCs
MAGEF12	2.3314E-43	3.51854571	0.274	0.082	3.5836E-39	DCs
CLEC7A2	4.9744E-43	1.14751293	0.852	0.908	7.6461E-39	DCs
NCOR21	7.6471E-43	1.05913669	0.868	0.903	1.1754E-38	DCs
VASH12	8.4262E-43	1.511209	0.801	0.821	1.2952E-38	DCs
CIRBP	1.062E-42	1.02539076	0.871	0.902	1.6324E-38	DCs
MTHFD21	1.1208E-42	2.44966982	0.666	0.55	1.7228E-38	DCs
AP1S22	1.2062E-42	2.45640212	0.533	0.318	1.854E-38	DCs
BANK12	1.8846E-42	3.75313636	0.202	0.047	2.8969E-38	DCs
GAREM2	5.8878E-42	5.63687	0.022	0.001	9.0502E-38	DCs
P2RY122	7.0696E-42	3.83625653	0.287	0.092	1.0867E-37	DCs
INPP5D1	1.6331E-41	2.45613841	0.577	0.39	2.5103E-37	DCs
GALNT32	1.851E-41	4.1039598	0.082	0.009	2.8451E-37	DCs
RELT1	2.1425E-41	1.56783046	0.726	0.677	3.2933E-37	DCs
BMERB11	2.1556E-41	5.92972343	0.038	0.002	3.3134E-37	DCs
ADGRA3	2.9026E-41	6.55256516	0.022	0.001	4.4616E-37	DCs
CDK61	4.4232E-41	2.80679246	0.114	0.017	6.7988E-37	DCs
IER22	1.2162E-40	1.93651581	0.719	0.681	1.8694E-36	DCs

MEF2A2	1.9816E-40	1.77056493	0.7	0.678	3.0459E-36	DCs
RUNX31	2.4731E-40	2.9587589	0.36	0.141	3.8013E-36	DCs
ADHFE11	2.672E-40	3.63043057	0.11	0.016	4.1071E-36	DCs
CTNND2	2.6789E-40	7.78607618	0.013	0	4.1178E-36	DCs
PDZD2	2.7907E-40	5.4366216	0.013	0	4.2897E-36	DCs
SETBP1	2.8991E-40	3.65294143	0.057	0.005	4.4563E-36	DCs
TLR62	3.144E-40	3.38142275	0.3	0.101	4.8326E-36	DCs
CXCR1	3.2292E-40	3.59019752	0.022	0.001	4.9635E-36	DCs
CIART1	3.3256E-40	5.89659956	0.028	0.001	5.1117E-36	DCs
GIPR	4.0315E-40	3.89728461	0.028	0.001	6.1969E-36	DCs
JAK21	1.8263E-39	1.8165048	0.688	0.629	2.8072E-35	DCs
LMO71	2.6346E-39	3.79056056	0.104	0.015	4.0497E-35	DCs
DENND4A1	3.0841E-39	1.83385267	0.678	0.608	4.7405E-35	DCs
EEF2	3.1999E-39	0.79785677	0.934	0.962	4.9186E-35	DCs
EIF2AK41	6.2058E-39	2.31905802	0.612	0.481	9.539E-35	DCs
S1PR11	9.0978E-39	2.85092782	0.06	0.005	1.3984E-34	DCs
NR4A12	1.3483E-38	2.76022283	0.615	0.46	2.0725E-34	DCs
AFF11	1.5743E-38	1.51094981	0.713	0.695	2.4199E-34	DCs
RIMS31	1.8562E-38	4.03528404	0.066	0.007	2.8531E-34	DCs
NCOA32	1.9891E-38	2.20463854	0.574	0.404	3.0574E-34	DCs
CHST112	2.0244E-38	2.11102123	0.64	0.518	3.1117E-34	DCs
CD70	2.3578E-38	3.54195671	0.025	0.001	3.6242E-34	DCs
C1orf541	2.4293E-38	4.3703686	0.281	0.093	3.7341E-34	DCs
RACK1	3.3693E-38	1.17910614	0.811	0.852	5.179E-34	DCs
SPINT21	5.7086E-38	1.18268151	0.814	0.864	8.7747E-34	DCs
VEGFA2	6.0425E-38	3.18596304	0.479	0.262	9.2879E-34	DCs
ITPRIPL22	9.5906E-38	1.88420812	0.666	0.601	1.4742E-33	DCs
LUC7L31	1.4868E-37	1.74422634	0.637	0.504	2.2853E-33	DCs
MICAL11	2.331E-37	1.15843149	0.823	0.847	3.583E-33	DCs
CEP681	3.2462E-37	3.12358299	0.331	0.128	4.9897E-33	DCs
JUND	4.3823E-37	0.8302463	0.946	0.972	6.736E-33	DCs
CAMK1D2	4.7188E-37	2.19354475	0.596	0.462	7.2532E-33	DCs
FOXP11	5.3348E-37	1.69408343	0.688	0.652	8.2001E-33	DCs
TSC22D31	6.3228E-37	1.55720188	0.795	0.805	9.7188E-33	DCs
ISYNA11	6.8868E-37	3.92962903	0.057	0.005	1.0586E-32	DCs
MBNL11	7.5346E-37	1.07287646	0.833	0.905	1.1581E-32	DCs
PPP1R9B2	1.8444E-36	1.06412902	0.83	0.892	2.835E-32	DCs
KCNMB12	1.9355E-36	3.57813519	0.24	0.072	2.975E-32	DCs
LMCD11	2.0571E-36	4.40247164	0.076	0.009	3.162E-32	DCs
NIPSNAP3B1	7.3975E-36	3.97669768	0.142	0.028	1.1371E-31	DCs
CD862	2.626E-35	1.75603203	0.694	0.685	4.0365E-31	DCs
CALCRL2	2.9823E-35	4.42535755	0.129	0.024	4.5842E-31	DCs
RHOC1	3.6069E-35	1.78718043	0.647	0.543	5.5442E-31	DCs
ANXA62	5.2378E-35	2.39688547	0.3	0.105	8.0511E-31	DCs
MICAL22	5.9697E-35	2.93241782	0.404	0.196	9.176E-31	DCs
TREML2	6.0869E-35	4.03200737	0.117	0.02	9.3561E-31	DCs
PRCP2	1.6998E-34	2.05115499	0.587	0.448	2.6127E-30	DCs
GDI2	1.75E-34	1.38126324	0.726	0.775	2.69E-30	DCs
NIBAN11	1.7877E-34	1.52832161	0.703	0.704	2.7478E-30	DCs
SLC1A41	2.1748E-34	2.69050784	0.416	0.21	3.3429E-30	DCs
PAQR72	3.04E-34	4.32761513	0.085	0.012	4.6728E-30	DCs
PLCG22	3.2016E-34	1.71424842	0.691	0.683	4.9211E-30	DCs

LRRK22	3.9747E-34	2.98161814	0.426	0.223	6.1095E-30	DCs
PSTPIP12	4.4662E-34	2.25126605	0.319	0.122	6.865E-30	DCs
SMAP21	7.6417E-34	1.60649116	0.719	0.76	1.1746E-29	DCs
CTNND13	7.7853E-34	1.36292008	0.804	0.889	1.1967E-29	DCs
KHDRBS21	7.9992E-34	5.58896448	0.035	0.002	1.2296E-29	DCs
FMNL11	1.0161E-33	0.91451928	0.877	0.945	1.5618E-29	DCs
CBX62	1.0457E-33	2.68959966	0.454	0.256	1.6074E-29	DCs
SPRY22	1.1182E-33	3.99184163	0.095	0.014	1.7187E-29	DCs
ATF41	2.653E-33	1.91181685	0.637	0.571	4.0779E-29	DCs
MFSD6L1	3.4176E-33	4.90245334	0.035	0.002	5.2531E-29	DCs
GUCY1A11	3.4651E-33	5.670867	0.019	0.001	5.3262E-29	DCs
NFKBID2	3.7808E-33	2.78136025	0.451	0.248	5.8114E-29	DCs
SPATS21	5.1207E-33	3.32921819	0.215	0.063	7.871E-29	DCs
OXCT11	5.3668E-33	3.12058714	0.101	0.016	8.2494E-29	DCs
DDX53	7.8593E-33	0.82077887	0.89	0.941	1.2081E-28	DCs
NT5DC31	9.0143E-33	3.3839028	0.218	0.065	1.3856E-28	DCs
BIRC31	9.6417E-33	4.50352864	0.221	0.066	1.482E-28	DCs
TRIP101	1.1931E-32	2.79406237	0.479	0.292	1.8339E-28	DCs
KCNK132	1.4297E-32	3.7913046	0.177	0.045	2.1976E-28	DCs
PSAT11	1.9394E-32	3.52970259	0.11	0.019	2.9811E-28	DCs
CXCR3	2.9293E-32	1.57004828	0.069	0.008	4.5027E-28	DCs
PHGDH1	5.7174E-32	3.39323918	0.192	0.052	8.7882E-28	DCs
TRPS12	7.2949E-32	2.23270196	0.521	0.349	1.1213E-27	DCs
HAVCR23	1.5146E-31	1.20867081	0.776	0.826	2.3281E-27	DCs
HNRNPLL1	1.6589E-31	2.0976552	0.587	0.474	2.5499E-27	DCs
FCGBP1	5.5684E-31	4.60774342	0.082	0.012	8.5592E-27	DCs
NPDC11	5.9258E-31	2.28680189	0.054	0.005	9.1085E-27	DCs
ANO9	7.9617E-31	2.3685143	0.044	0.004	1.2238E-26	DCs
PLVAP	8.9553E-31	6.44990137	0.013	0	1.3765E-26	DCs
IVNS1ABP	9.6755E-31	0.98502777	0.883	0.934	1.4872E-26	DCs
ARNTL22	1.4007E-30	3.89891203	0.155	0.037	2.1531E-26	DCs
LILRA41	1.4244E-30	4.61618215	0.035	0.002	2.1895E-26	DCs
TRPM22	1.9978E-30	3.50650041	0.151	0.036	3.0708E-26	DCs
LAG3	2.0549E-30	2.42351622	0.076	0.01	3.1585E-26	DCs
PACSIN21	2.1067E-30	1.77604051	0.656	0.672	3.2382E-26	DCs
RASIP1	3.181E-30	6.12164985	0.013	0	4.8895E-26	DCs
CTTNBP2NL1	4.253E-30	2.18452452	0.555	0.445	6.5372E-26	DCs
CASS41	5.7007E-30	3.20068146	0.145	0.034	8.7625E-26	DCs
SCARF12	6.2804E-30	3.02289333	0.303	0.124	9.6536E-26	DCs
RUFY42	6.4311E-30	5.10899402	0.063	0.008	9.8853E-26	DCs
PLXNB22	6.472E-30	0.87461088	0.836	0.898	9.948E-26	DCs
HS3ST3B12	8.8787E-30	3.05072407	0.129	0.027	1.3647E-25	DCs
DGKA1	8.9714E-30	2.26307467	0.385	0.191	1.379E-25	DCs
CMTM61	9.3179E-30	1.06856213	0.804	0.879	1.4322E-25	DCs
LMBR1L	9.6369E-30	2.51895687	0.473	0.302	1.4813E-25	DCs
PIM2	1.0611E-29	2.77485472	0.448	0.269	1.631E-25	DCs
HIST1H1C	1.1277E-29	1.58432785	0.599	0.463	1.7333E-25	DCs
PDGFB2	1.1467E-29	3.33519675	0.246	0.086	1.7626E-25	DCs
INSIG11	1.3285E-29	2.14535053	0.533	0.361	2.042E-25	DCs
SCO22	1.4398E-29	1.84158582	0.647	0.571	2.213E-25	DCs
MOB1B2	2.2625E-29	2.19163203	0.495	0.339	3.4778E-25	DCs
CAV1	3.5322E-29	5.56507758	0.013	0	5.4293E-25	DCs

SLC7A13	4.2513E-29	2.73915942	0.331	0.148	6.5346E-25	DCs
MARCH92	4.8825E-29	3.00515136	0.215	0.068	7.5049E-25	DCs
DCLK21	5.9812E-29	3.91287845	0.114	0.022	9.1938E-25	DCs
PLXNA4	6.0174E-29	4.31095565	0.016	0.001	9.2494E-25	DCs
JAK11	6.2149E-29	1.02404589	0.757	0.815	9.5529E-25	DCs
SDK1	9.8003E-29	4.73624726	0.022	0.001	1.5064E-24	DCs
BRD21	1.501E-28	1.01892738	0.779	0.833	2.3073E-24	DCs
RAB11FIP1	1.651E-28	1.00972942	0.833	0.908	2.5377E-24	DCs
PHTF2	2.0945E-28	1.69804657	0.637	0.585	3.2195E-24	DCs
RGS141	3.168E-28	2.54687582	0.35	0.168	4.8695E-24	DCs
TES2	3.1723E-28	2.11519491	0.527	0.39	4.8762E-24	DCs
FNBP11	3.9287E-28	1.85170956	0.678	0.739	6.0388E-24	DCs
ASTL1	4.7387E-28	3.64433751	0.041	0.004	7.2839E-24	DCs
TM4SF5	4.9316E-28	6.19402527	0.016	0.001	7.5803E-24	DCs
GPR18	5.1772E-28	3.08174204	0.038	0.003	7.9579E-24	DCs
GNA131	5.2266E-28	1.25324656	0.763	0.838	8.0338E-24	DCs
APAF12	5.2884E-28	2.39208197	0.461	0.291	8.1288E-24	DCs
SIGLEC102	5.8606E-28	3.93102513	0.243	0.087	9.0084E-24	DCs
PRMT21	7.656E-28	1.14735862	0.719	0.789	1.1768E-23	DCs
CHD21	7.7263E-28	1.48432137	0.634	0.555	1.1876E-23	DCs
SH3RF12	9.081E-28	4.21174561	0.057	0.007	1.3958E-23	DCs
OSBPL81	1.1293E-27	1.42359095	0.669	0.678	1.7358E-23	DCs
SERTAD22	1.344E-27	1.85637894	0.64	0.628	2.0659E-23	DCs
MT-ATP62	1.3702E-27	1.99284688	0.606	0.459	2.1061E-23	DCs
ZNRF11	1.8598E-27	2.52166625	0.104	0.02	2.8587E-23	DCs
GASK1A1	2.409E-27	6.11419778	0.025	0.001	3.7028E-23	DCs
KIAA00401	2.5814E-27	2.40899861	0.36	0.18	3.9679E-23	DCs
ABCC41	3.3345E-27	3.04122999	0.29	0.123	5.1254E-23	DCs
MARCH13	3.6547E-27	1.33591474	0.719	0.794	5.6176E-23	DCs
TAP21	3.8656E-27	1.59823727	0.678	0.721	5.9417E-23	DCs
SELL1	4.1829E-27	2.84095132	0.183	0.054	6.4295E-23	DCs
PLSCR12	5.2752E-27	1.08754181	0.789	0.87	8.1086E-23	DCs
DAPP1	5.8291E-27	2.37958855	0.603	0.575	8.96E-23	DCs
TNFRSF91	5.9548E-27	4.35579746	0.035	0.003	9.1532E-23	DCs
ETS1	6.0078E-27	0.2171547	0.151	0.036	9.2345E-23	DCs
MARVELD3	7.6691E-27	4.94843504	0.016	0.001	1.1788E-22	DCs
SNX82	9.0741E-27	1.7555178	0.609	0.578	1.3948E-22	DCs
PFKFB31	1.1259E-26	2.11150264	0.549	0.429	1.7306E-22	DCs
SAP251	1.2762E-26	2.65456959	0.259	0.099	1.9617E-22	DCs
RAB322	1.8179E-26	2.30559702	0.58	0.515	2.7942E-22	DCs
TPST1	2.2443E-26	5.31247379	0.019	0.001	3.4498E-22	DCs
CCSER21	2.5334E-26	1.73758022	0.568	0.478	3.8941E-22	DCs
KLF102	3.2695E-26	1.99591175	0.574	0.486	5.0255E-22	DCs
OTULINL2	3.3143E-26	1.73820475	0.621	0.584	5.0944E-22	DCs
SOCS32	4.8403E-26	1.30746748	0.735	0.7	7.44E-22	DCs
PTPRE1	4.8534E-26	2.50259837	0.533	0.415	7.4601E-22	DCs
TDRD101	4.927E-26	5.6600032	0.013	0	7.5733E-22	DCs
KMT2E1	7.0355E-26	1.44188017	0.65	0.653	1.0814E-21	DCs
TRH	1.2064E-25	7.2796167	0.013	0	1.8543E-21	DCs
CTTNBP21	1.2453E-25	4.63529328	0.013	0	1.9141E-21	DCs
CPVL2	1.555E-25	1.85813291	0.792	0.902	2.3902E-21	DCs
ASS11	1.6584E-25	3.56735255	0.038	0.003	2.5491E-21	DCs

TGFB1I1	2.922E-25	6.68770352	0.013	0	4.4914E-21	DCs
CARMIL2	2.9907E-25	2.49926658	0.079	0.013	4.597E-21	DCs
QPCT2	3.8835E-25	2.92521171	0.334	0.165	5.9693E-21	DCs
HOXA9	3.9439E-25	4.87379877	0.032	0.002	6.0622E-21	DCs
PPP1R181	4.4158E-25	0.8586834	0.798	0.883	6.7875E-21	DCs
DNMT11	4.4236E-25	1.90054547	0.59	0.534	6.7995E-21	DCs
SLC1A51	4.5095E-25	1.67472958	0.65	0.684	6.9315E-21	DCs
PPP1R3G1	4.9137E-25	4.70665611	0.041	0.004	7.5529E-21	DCs
CDC42SE11	4.9549E-25	1.02962648	0.792	0.88	7.6162E-21	DCs
PIK3CG	6.2807E-25	1.77371651	0.609	0.579	9.654E-21	DCs
EVI2A1	9.3312E-25	1.60466019	0.644	0.653	1.4343E-20	DCs
PHF191	1.2332E-24	1.89014032	0.334	0.157	1.8955E-20	DCs
CKB1	1.3745E-24	3.80490099	0.066	0.01	2.1127E-20	DCs
SPECC11	1.5363E-24	3.70675263	0.161	0.047	2.3615E-20	DCs
ATAD2B2	1.5558E-24	2.3349379	0.416	0.254	2.3913E-20	DCs
PNRC11	2.3744E-24	1.16596055	0.861	0.931	3.6497E-20	DCs
PILRB1	2.3982E-24	1.86002612	0.521	0.386	3.6863E-20	DCs
ADCY72	2.5528E-24	1.51118513	0.653	0.685	3.924E-20	DCs
KCNK62	2.5635E-24	2.26639225	0.498	0.373	3.9404E-20	DCs
DEF6	2.6215E-24	1.21635228	0.7	0.752	4.0294E-20	DCs
PXYLP1	3.2299E-24	3.98442568	0.019	0.001	4.9647E-20	DCs
PLP21	3.42E-24	1.52842432	0.615	0.57	5.2569E-20	DCs
ZBTB182	3.8779E-24	2.379901	0.445	0.3	5.9607E-20	DCs
THRB1	5.1981E-24	4.81984093	0.032	0.003	7.9901E-20	DCs
PLEKHG22	5.3753E-24	3.92556432	0.11	0.025	8.2623E-20	DCs
KDM2B2	6.0098E-24	2.70321336	0.511	0.401	9.2376E-20	DCs
NUDT31	7.9894E-24	1.31585455	0.675	0.728	1.228E-19	DCs
R3HDM42	8.8737E-24	1.62932593	0.621	0.618	1.364E-19	DCs
EPS8L21	1.0457E-23	3.23843226	0.041	0.004	1.6073E-19	DCs
CISH	1.048E-23	1.37961321	0.7	0.758	1.6108E-19	DCs
LRCH41	1.225E-23	1.48817271	0.644	0.649	1.883E-19	DCs
N4BP21	1.3196E-23	2.62004186	0.369	0.206	2.0283E-19	DCs
BCL32	1.4892E-23	2.29874674	0.536	0.438	2.289E-19	DCs
RAB7B1	1.9643E-23	0.70292978	0.211	0.629	3.0194E-19	DCs
H1FX1	1.9897E-23	1.45702703	0.656	0.624	3.0584E-19	DCs
EZR1	2.0947E-23	1.29370699	0.76	0.831	3.2198E-19	DCs
ROBO11	2.8934E-23	3.94021213	0.123	0.03	4.4474E-19	DCs
FXYD52	4.0661E-23	0.70260804	0.88	0.954	6.25E-19	DCs
ATP6V0A21	4.2379E-23	2.61253497	0.435	0.291	6.5141E-19	DCs
PLCB22	5.8959E-23	1.08666039	0.754	0.839	9.0626E-19	DCs
PDGFA1	8.109E-23	3.88890291	0.019	0.001	1.2464E-18	DCs
FCHSD21	1.1483E-22	2.06289204	0.511	0.41	1.7651E-18	DCs
GPR352	1.1585E-22	2.95511993	0.098	0.021	1.7807E-18	DCs
MEX3C1	1.166E-22	2.53765955	0.252	0.106	1.7923E-18	DCs
ARGLU11	1.1846E-22	1.68654924	0.609	0.589	1.8208E-18	DCs
INPP5F	1.2925E-22	1.96277651	0.536	0.445	1.9867E-18	DCs
CLK11	1.4264E-22	1.57433129	0.565	0.489	2.1925E-18	DCs
ADGB	1.4656E-22	4.28270556	0.016	0.001	2.2528E-18	DCs
GPSM11	1.5655E-22	3.35592043	0.035	0.003	2.4063E-18	DCs
NABP11	2.2654E-22	1.82861225	0.644	0.649	3.4821E-18	DCs
SLC41A21	2.2878E-22	2.78297898	0.451	0.323	3.5166E-18	DCs
SNX20	5.3744E-22	1.36963682	0.659	0.74	8.2609E-18	DCs

UBA52	5.5039E-22	0.71162741	0.877	0.933	8.4601E-18	DCs
KIAA01001	8.195E-22	0.9056377	0.804	0.903	1.2597E-17	DCs
SAMHD12	8.2918E-22	0.44563631	0.946	0.982	1.2745E-17	DCs
BACH2	8.4561E-22	2.70459718	0.019	0.001	1.2998E-17	DCs
BLOC1S61	8.5881E-22	2.14827039	0.492	0.395	1.3201E-17	DCs
MYO18A2	9.0812E-22	2.36114944	0.423	0.28	1.3959E-17	DCs
ICAM31	9.4616E-22	2.1957299	0.363	0.204	1.4543E-17	DCs
MAP4K1	1.1126E-21	2.13801177	0.429	0.286	1.7102E-17	DCs
SEC14L11	1.1452E-21	1.41453082	0.618	0.641	1.7603E-17	DCs
ETS22	1.1701E-21	1.86909886	0.65	0.709	1.7986E-17	DCs
RIN31	1.3381E-21	0.72050267	0.826	0.928	2.0569E-17	DCs
TSPAN2	1.6943E-21	2.27926746	0.038	0.004	2.6044E-17	DCs
EDIL3	2.0395E-21	6.82037161	0.013	0	3.135E-17	DCs
IFI162	2.4367E-21	1.10242155	0.722	0.828	3.7455E-17	DCs
FCHO11	2.5358E-21	2.65954728	0.278	0.129	3.8978E-17	DCs
EP3001	2.5508E-21	1.12226622	0.688	0.761	3.9208E-17	DCs
PDE4B1	2.8659E-21	1.62485292	0.555	0.458	4.4052E-17	DCs
OGT1	2.9124E-21	1.5107744	0.584	0.548	4.4767E-17	DCs
METTL7A2	3.0358E-21	2.42727026	0.401	0.253	4.6663E-17	DCs
TMEM8B	3.763E-21	3.68005943	0.06	0.009	5.7841E-17	DCs
ICAM12	3.7635E-21	2.20041698	0.461	0.327	5.7849E-17	DCs
ISG201	4.2568E-21	2.06461771	0.158	0.048	6.5432E-17	DCs
CNPPD11	4.5731E-21	1.26843585	0.672	0.75	7.0293E-17	DCs
IL10RA2	5.0283E-21	1.10930246	0.729	0.817	7.7291E-17	DCs
MAP3K13	5.0562E-21	2.58179549	0.467	0.355	7.7719E-17	DCs
ZNF711	6.153E-21	4.53625756	0.05	0.007	9.4577E-17	DCs
PTBP3	6.8704E-21	0.90917472	0.779	0.881	1.0561E-16	DCs
TGIF22	7.0009E-21	2.97140889	0.202	0.076	1.0761E-16	DCs
RFX31	9.2532E-21	2.48006175	0.287	0.138	1.4223E-16	DCs
MRTFA1	9.8451E-21	1.27177764	0.662	0.733	1.5133E-16	DCs
MAP3K62	1.0658E-20	3.04338012	0.243	0.104	1.6383E-16	DCs
EEF1G	1.1189E-20	0.8468761	0.814	0.911	1.7198E-16	DCs
SLC7A61	1.1476E-20	2.19742604	0.413	0.272	1.764E-16	DCs
ZMIZ21	1.1535E-20	1.78239698	0.587	0.581	1.773E-16	DCs
MDM41	1.3391E-20	1.62947066	0.58	0.538	2.0584E-16	DCs
ZNF5162	1.3923E-20	2.5032616	0.413	0.278	2.1401E-16	DCs
NEDD4L2	1.4469E-20	3.12182855	0.151	0.047	2.2241E-16	DCs
LPAR62	1.7568E-20	3.07444459	0.388	0.247	2.7003E-16	DCs
FYN	1.7584E-20	2.09351565	0.246	0.105	2.7029E-16	DCs
FILIP1L2	2.4514E-20	2.96537531	0.385	0.244	3.768E-16	DCs
PIM31	3.3856E-20	2.32853289	0.483	0.378	5.204E-16	DCs
MPP3	3.4552E-20	3.84458447	0.085	0.018	5.311E-16	DCs
MCL12	4.02E-20	0.43291086	0.978	0.987	6.1791E-16	DCs
CHRFAM7A2	4.2169E-20	4.37046699	0.06	0.01	6.4817E-16	DCs
DDX171	4.6508E-20	1.48376431	0.587	0.579	7.1488E-16	DCs
IRF72	5.0472E-20	1.54680566	0.656	0.701	7.7581E-16	DCs
CREB52	5.0517E-20	2.65321632	0.303	0.157	7.765E-16	DCs
OSBPL31	6.7556E-20	2.33044511	0.36	0.215	1.0384E-15	DCs
ELK32	8.0688E-20	2.83591586	0.186	0.068	1.2403E-15	DCs
FAM49B2	8.2883E-20	1.01273931	0.713	0.827	1.274E-15	DCs
ADORA2A1	8.3526E-20	2.9872013	0.047	0.006	1.2839E-15	DCs
RASL10A1	1.0367E-19	4.69345951	0.032	0.003	1.5936E-15	DCs

CCN2	1.3495E-19	4.10605937	0.016	0.001	2.0744E-15	DCs
CCNI	1.4818E-19	0.63686307	0.902	0.945	2.2777E-15	DCs
SKIL2	1.6342E-19	0.95365023	0.76	0.871	2.5119E-15	DCs
GRK31	1.9663E-19	0.96310526	0.754	0.864	3.0225E-15	DCs
BMF1	2.6424E-19	2.43489231	0.36	0.218	4.0616E-15	DCs
OGA1	2.6856E-19	1.02327209	0.697	0.753	4.128E-15	DCs
MALT11	2.7722E-19	2.42687225	0.423	0.298	4.2612E-15	DCs
SLC4A72	4.3183E-19	2.48625224	0.312	0.169	6.6377E-15	DCs
MAT2A2	4.4004E-19	0.98983616	0.757	0.839	6.7639E-15	DCs
PELI22	5.6949E-19	3.09059793	0.189	0.072	8.7536E-15	DCs
HSF41	5.7255E-19	3.53173103	0.069	0.013	8.8007E-15	DCs
RASGRP32	6.9486E-19	3.13401169	0.158	0.053	1.0681E-14	DCs
HIST1H1D	7.6358E-19	1.86682826	0.265	0.122	1.1737E-14	DCs
IL13RA12	9.2947E-19	1.25147344	0.691	0.829	1.4287E-14	DCs
REPS21	9.5095E-19	3.61114516	0.107	0.028	1.4617E-14	DCs
NFKB11	9.9189E-19	2.28377237	0.562	0.569	1.5246E-14	DCs
LPGAT12	1.3433E-18	1.90946384	0.505	0.427	2.0648E-14	DCs
RCSD11	1.8768E-18	1.9021435	0.41	0.274	2.8848E-14	DCs
ELOVL12	2.0676E-18	0.90341152	0.732	0.836	3.1781E-14	DCs
SMOC1	2.44E-18	6.37058474	0.016	0.001	3.7505E-14	DCs
EPOP	2.5673E-18	5.30711755	0.019	0.001	3.9463E-14	DCs
HIST2H2BE2	2.6955E-18	2.29627724	0.281	0.139	4.1432E-14	DCs
PREX12	3.4172E-18	1.56732529	0.552	0.532	5.2525E-14	DCs
CYLD	4.0911E-18	1.36390907	0.59	0.596	6.2885E-14	DCs
TNFRSF41	4.41E-18	2.22044624	0.028	0.003	6.7787E-14	DCs
MAP6	4.7329E-18	3.85560547	0.016	0.001	7.2749E-14	DCs
NSMF1	5.1011E-18	2.74706253	0.303	0.166	7.8409E-14	DCs
RBM331	5.3016E-18	1.09669967	0.659	0.72	8.149E-14	DCs
CKLF	5.5417E-18	1.35648255	0.65	0.751	8.5181E-14	DCs
SUCNR12	7.4364E-18	2.66899402	0.511	0.467	1.143E-13	DCs
DLGAP42	7.6023E-18	1.99848075	0.533	0.503	1.1686E-13	DCs
MT-ND31	1.0785E-17	1.6565712	0.543	0.44	1.6578E-13	DCs
DYSF2	1.1089E-17	3.27525417	0.101	0.026	1.7044E-13	DCs
TNFRSF181	1.2022E-17	1.8936871	0.038	0.005	1.8479E-13	DCs
OLFM21	1.3759E-17	3.51502949	0.013	0.001	2.1148E-13	DCs
ESYT21	1.4738E-17	1.22765766	0.644	0.717	2.2654E-13	DCs
CCNL21	1.9959E-17	1.57914614	0.543	0.495	3.0679E-13	DCs
BATF3	2.0021E-17	2.63798404	0.36	0.231	3.0774E-13	DCs
LHFPL61	2.5063E-17	5.71030276	0.016	0.001	3.8524E-13	DCs
PYCR1	2.5592E-17	5.1292732	0.019	0.001	3.9337E-13	DCs
TBC1D11	2.6485E-17	2.48202657	0.17	0.063	4.071E-13	DCs
AKAP131	3.674E-17	0.69981232	0.776	0.892	5.6473E-13	DCs
HAVCR1	4.3174E-17	6.05511896	0.016	0.001	6.6363E-13	DCs
SYCP2L	5.0095E-17	5.27517929	0.025	0.002	7.7E-13	DCs
LUC7L1	5.089E-17	1.73495024	0.483	0.395	7.8224E-13	DCs
TRA2A	5.9549E-17	1.13356494	0.659	0.752	9.1533E-13	DCs
SIPA1L11	6.5867E-17	2.63599864	0.271	0.141	1.0124E-12	DCs
MARCH81	6.5986E-17	2.24764667	0.394	0.28	1.0143E-12	DCs
ITGA2B	6.7691E-17	4.74639779	0.013	0.001	1.0405E-12	DCs
TIAF11	7.4109E-17	3.60550936	0.066	0.013	1.1391E-12	DCs
SIPA12	8.4048E-17	1.25037978	0.625	0.7	1.2919E-12	DCs
MGAM2	8.5282E-17	4.24894444	0.047	0.007	1.3109E-12	DCs

RFX51	9.4176E-17	1.37506529	0.603	0.649	1.4476E-12	DCs
IFITM1	1.042E-16	2.06660154	0.032	0.004	1.6016E-12	DCs
RAP2A1	1.112E-16	1.41384756	0.609	0.66	1.7093E-12	DCs
RALA2	1.1546E-16	2.20081005	0.445	0.357	1.7747E-12	DCs
ST3GAL41	1.2242E-16	2.91250793	0.192	0.08	1.8818E-12	DCs
JAML1	1.288E-16	1.06150722	0.798	0.941	1.9797E-12	DCs
IFITM22	1.4731E-16	2.08903416	0.451	0.363	2.2642E-12	DCs
TAB21	1.6379E-16	1.43284403	0.577	0.606	2.5176E-12	DCs
NOP53	1.757E-16	0.94869151	0.681	0.788	2.7007E-12	DCs
AXL1	1.8487E-16	0.78392392	0.811	0.891	2.8416E-12	DCs
KMT2C1	1.9648E-16	0.98463556	0.672	0.766	3.0201E-12	DCs
CYP2E1	1.9939E-16	4.25354206	0.041	0.006	3.0648E-12	DCs
TRIM36	2.0885E-16	2.47208046	0.338	0.212	3.2102E-12	DCs
ARHGAP42	2.1021E-16	5.63132874	0.013	0.001	3.2311E-12	DCs
LAMA5	2.1338E-16	4.09933249	0.019	0.001	3.2799E-12	DCs
MT-CO31	2.2481E-16	1.83402126	0.587	0.523	3.4555E-12	DCs
PBX21	2.2831E-16	1.93437285	0.476	0.408	3.5094E-12	DCs
SERPINF21	2.5329E-16	2.88839131	0.196	0.083	3.8934E-12	DCs
TSPOAP11	2.8303E-16	2.73323405	0.104	0.029	4.3505E-12	DCs
FAM71A1	3.032E-16	5.37735093	0.013	0.001	4.6605E-12	DCs
ACVR1C	3.0727E-16	5.51504693	0.013	0.001	4.723E-12	DCs
RAB313	3.3434E-16	0.74319755	0.801	0.887	5.1391E-12	DCs
TBL1XR11	3.6052E-16	1.18240414	0.612	0.669	5.5415E-12	DCs
ATXN71	3.6251E-16	1.75248297	0.467	0.38	5.5722E-12	DCs
CSF1R2	4.1391E-16	1.58180718	0.675	0.862	6.3622E-12	DCs
PADI22	4.4386E-16	2.43473008	0.315	0.18	6.8225E-12	DCs
AP1S3	5.5524E-16	3.45465241	0.145	0.051	8.5347E-12	DCs
SEPTIN6	5.6103E-16	1.74333618	0.511	0.485	8.6235E-12	DCs
RUFY31	9.4781E-16	2.02874677	0.479	0.413	1.4569E-11	DCs
INPP5B1	1.1031E-15	1.70919187	0.505	0.456	1.6956E-11	DCs
PALLD2	1.124E-15	3.22096342	0.174	0.07	1.7277E-11	DCs
SLC40A12	1.3745E-15	3.18587239	0.054	0.01	2.1127E-11	DCs
MT-ND62	1.4256E-15	2.52000382	0.281	0.152	2.1913E-11	DCs
CDK2AP1	1.7672E-15	2.05588395	0.464	0.401	2.7163E-11	DCs
TMEM1091	1.8242E-15	1.24621373	0.644	0.755	2.804E-11	DCs
MOCOS1	1.8819E-15	3.07479487	0.227	0.11	2.8926E-11	DCs
TRIM8	1.8854E-15	0.88566549	0.688	0.811	2.8981E-11	DCs
ZC3H12D1	2.0232E-15	2.90761457	0.136	0.046	3.1099E-11	DCs
CALHM62	2.7275E-15	2.59166774	0.256	0.133	4.1925E-11	DCs
WDFY42	2.9517E-15	3.1575225	0.615	0.714	4.5371E-11	DCs
NEK31	2.9678E-15	3.43537361	0.085	0.022	4.5619E-11	DCs
GEM1	3.3344E-15	3.77219951	0.013	0.001	5.1254E-11	DCs
RNF1251	3.5222E-15	2.00170911	0.268	0.144	5.414E-11	DCs
YPEL51	4.8384E-15	1.32451069	0.716	0.851	7.4372E-11	DCs
ITSN21	5.1088E-15	1.42990729	0.584	0.641	7.8527E-11	DCs
GNB42	5.4007E-15	1.14087235	0.64	0.768	8.3014E-11	DCs
JARID22	5.6271E-15	2.04156547	0.41	0.314	8.6494E-11	DCs
SUGP21	6.2768E-15	1.44884526	0.502	0.449	9.648E-11	DCs
KIFC21	6.9221E-15	2.5565225	0.227	0.11	1.064E-10	DCs
RGS16	7.0042E-15	2.46025277	0.035	0.005	1.0766E-10	DCs
PXDC11	7.4124E-15	2.37145196	0.41	0.324	1.1394E-10	DCs
PTPRF1	7.8605E-15	3.9775187	0.019	0.002	1.2082E-10	DCs

ATP2B1	8.6008E-15	1.34289323	0.612	0.659	1.322E-10	DCs
ZNF4692	8.9277E-15	3.78714334	0.05	0.009	1.3723E-10	DCs
JADE3	1.0268E-14	3.60040021	0.098	0.028	1.5783E-10	DCs
IKBKB1	1.2966E-14	1.13891853	0.618	0.682	1.9929E-10	DCs
IRAK21	1.3049E-14	3.44583624	0.136	0.048	2.0058E-10	DCs
SLC38A22	1.3185E-14	1.31458597	0.599	0.665	2.0266E-10	DCs
PRKAR1A1	1.3714E-14	0.61723599	0.839	0.942	2.108E-10	DCs
TGFB21	1.3952E-14	4.60457097	0.025	0.003	2.1445E-10	DCs
SMO1	1.9811E-14	4.5353465	0.035	0.005	3.0452E-10	DCs
TRIO1	2.0087E-14	1.01089195	0.688	0.784	3.0876E-10	DCs
KIAA1549	2.034E-14	4.13030799	0.013	0.001	3.1264E-10	DCs
PACS11	2.1054E-14	1.55325535	0.549	0.592	3.2363E-10	DCs
AGER1	2.2283E-14	2.7464122	0.158	0.062	3.425E-10	DCs
RAB9A1	2.6426E-14	0.24420094	0.151	0.43	4.062E-10	DCs
SUZ12	2.8443E-14	1.85440284	0.467	0.409	4.372E-10	DCs
CCDC88B	2.8709E-14	1.27974107	0.599	0.653	4.4128E-10	DCs
SBNO22	2.8806E-14	1.28953181	0.587	0.635	4.4278E-10	DCs
MT-CO21	3.993E-14	1.86268482	0.521	0.451	6.1376E-10	DCs
FHL32	4.6063E-14	2.4184959	0.341	0.229	7.0804E-10	DCs
IER32	4.7281E-14	2.24831879	0.416	0.325	7.2676E-10	DCs
ZNF827	4.7524E-14	2.14081494	0.019	0.002	7.305E-10	DCs
STK17A	5.1938E-14	1.86377574	0.344	0.229	7.9834E-10	DCs
ARHGAP5	6.3513E-14	2.27318524	0.331	0.22	9.7626E-10	DCs
CYTH11	6.6951E-14	1.02743371	0.697	0.833	1.0291E-09	DCs
CTDSP21	7.1147E-14	1.05994705	0.644	0.747	1.0936E-09	DCs
IL18RAP	7.3335E-14	1.52136787	0.035	0.005	1.1272E-09	DCs
MCC	8.3737E-14	3.66777187	0.022	0.002	1.2871E-09	DCs
CCDC88A1	9.059E-14	0.69374401	0.789	0.901	1.3925E-09	DCs
DUSP21	9.2237E-14	2.65838559	0.164	0.067	1.4178E-09	DCs
ADAP23	1.0848E-13	1.5444539	0.543	0.567	1.6674E-09	DCs
PPP1CB1	1.1857E-13	0.82029203	0.694	0.823	1.8226E-09	DCs
KLF12	1.2108E-13	2.01213284	0.132	0.048	1.8611E-09	DCs
VCAM11	1.2654E-13	3.71300455	0.022	0.002	1.9451E-09	DCs
ARL6IP5	1.3841E-13	0.81833815	0.726	0.877	2.1276E-09	DCs
CSK	1.4344E-13	0.72592008	0.751	0.883	2.2048E-09	DCs
NR4A32	1.4593E-13	2.80790943	0.517	0.479	2.2432E-09	DCs
ST8SIA1	1.5056E-13	2.58231371	0.025	0.003	2.3142E-09	DCs
DENND1B	1.507E-13	2.02476685	0.42	0.345	2.3164E-09	DCs
VPS13C1	1.5768E-13	0.85598404	0.659	0.779	2.4237E-09	DCs
MAX1	1.6199E-13	1.04375508	0.634	0.77	2.49E-09	DCs
MED13L2	1.7563E-13	1.90064429	0.451	0.397	2.6996E-09	DCs
CAPN22	2.3608E-13	0.7935164	0.741	0.849	3.6288E-09	DCs
ZFP36L22	2.519E-13	0.94003643	0.776	0.945	3.872E-09	DCs
CDC42SE2	2.5605E-13	1.46458092	0.539	0.568	3.9358E-09	DCs
P2RX72	2.6577E-13	0.46977157	0.218	0.536	4.0851E-09	DCs
IGLON5	2.8075E-13	5.13025934	0.013	0.001	4.3154E-09	DCs
YWHAH2	3.1853E-13	1.14672096	0.675	0.842	4.896E-09	DCs
CC2D2B1	3.2509E-13	3.70987789	0.047	0.009	4.9969E-09	DCs
PLEKHG1	3.3111E-13	3.80305231	0.019	0.002	5.0895E-09	DCs
GNPTAB1	3.5567E-13	1.35834175	0.558	0.618	5.467E-09	DCs
LIMD12	4.1562E-13	1.70986133	0.479	0.448	6.3885E-09	DCs
CEP128	4.6766E-13	2.12379642	0.3	0.188	7.1885E-09	DCs

MTCL1	5.6904E-13	5.48186785	0.013	0.001	8.7467E-09	DCs
DISC11	6.1019E-13	2.10801168	0.341	0.234	9.3792E-09	DCs
MARCH71	6.6341E-13	0.78508247	0.688	0.816	1.0197E-08	DCs
TGFBR12	6.8196E-13	1.43081628	0.58	0.651	1.0482E-08	DCs
EMP32	6.919E-13	0.64045568	0.915	0.959	1.0635E-08	DCs
CD2742	6.9257E-13	0.77283884	0.132	0.375	1.0645E-08	DCs
NDE11	6.9595E-13	1.98535514	0.442	0.38	1.0697E-08	DCs
MPEG12	7.2839E-13	0.84579266	0.344	0.727	1.1196E-08	DCs
TBC1D41	7.3817E-13	0.29555546	0.24	0.566	1.1346E-08	DCs
TRPV11	7.633E-13	3.29253582	0.114	0.039	1.1733E-08	DCs
TYK22	8.1206E-13	1.25524942	0.558	0.602	1.2482E-08	DCs
TTLL121	8.9878E-13	0.13814714	0.129	0.369	1.3815E-08	DCs
DCK1	1.0935E-12	1.49631955	0.527	0.527	1.6808E-08	DCs
MORC31	1.1049E-12	0.97365833	0.621	0.721	1.6984E-08	DCs
C16orf721	1.272E-12	1.12598496	0.574	0.625	1.9552E-08	DCs
MAMDC22	1.3983E-12	2.89329117	0.044	0.008	2.1493E-08	DCs
SAMSN11	1.4026E-12	0.42127594	0.268	0.597	2.1559E-08	DCs
TIAL11	1.4348E-12	1.21376868	0.58	0.647	2.2054E-08	DCs
DGKD2	1.4591E-12	2.50530235	0.215	0.11	2.2428E-08	DCs
AQP1	1.6629E-12	6.2349635	0.032	0.005	2.5561E-08	DCs
ZNF5322	1.9662E-12	2.58554733	0.227	0.122	3.0223E-08	DCs
PLK32	2.0642E-12	1.67557645	0.397	0.306	3.1729E-08	DCs
GAS2L11	2.1472E-12	0.17184994	0.186	0.462	3.3005E-08	DCs
MAP3K23	2.2308E-12	1.31188138	0.552	0.603	3.429E-08	DCs
CERK	2.2463E-12	2.11185001	0.325	0.223	3.4528E-08	DCs
EEF1B2	2.6748E-12	1.23294025	0.565	0.625	4.1115E-08	DCs
TTC22	2.9609E-12	2.59962346	0.025	0.003	4.5511E-08	DCs
SESN31	3.0493E-12	2.19731675	0.344	0.246	4.687E-08	DCs
KMT5B1	3.6284E-12	1.39908839	0.524	0.54	5.5773E-08	DCs
ANKRD101	3.7783E-12	1.15381687	0.609	0.683	5.8077E-08	DCs
PPIL1	3.8737E-12	0.1234224	0.101	0.316	5.9542E-08	DCs
TPCN12	3.9648E-12	3.03991119	0.047	0.01	6.0943E-08	DCs
ROBO31	3.9772E-12	2.8907478	0.167	0.075	6.1134E-08	DCs
UBD2	3.9825E-12	3.418044	0.047	0.01	6.1215E-08	DCs
SRSF12	4.0686E-12	5.08928661	0.013	0.001	6.2539E-08	DCs
TYMP2	4.2163E-12	0.58054148	0.94	0.96	6.4809E-08	DCs
TNK21	4.5644E-12	1.6003177	0.467	0.423	7.0159E-08	DCs
TCEA31	5.6096E-12	0.45765504	0.186	0.455	8.6225E-08	DCs
PCP4L1	5.8323E-12	4.43077614	0.035	0.006	8.9649E-08	DCs
RUNX11	5.841E-12	0.69942521	0.823	0.937	8.9782E-08	DCs
CA112	5.9488E-12	3.25927744	0.101	0.034	9.1439E-08	DCs
CEP350	6.3473E-12	1.08867328	0.618	0.709	9.7565E-08	DCs
TIGD3	6.5915E-12	4.87070232	0.019	0.002	1.0132E-07	DCs
TMEM1632	6.6288E-12	3.65577393	0.044	0.009	1.0189E-07	DCs
SMARCE1	7.2882E-12	0.12377824	0.208	0.493	1.1203E-07	DCs
MTDH2	7.7361E-12	0.72631277	0.716	0.866	1.1891E-07	DCs
ST142	8.8217E-12	1.21096348	0.596	0.659	1.356E-07	DCs
ADGRG5	9.2697E-12	3.14393542	0.05	0.011	1.4248E-07	DCs
COX17	9.585E-12	0.12615631	0.246	0.568	1.4733E-07	DCs
ALOX15	1.0366E-11	2.37062081	0.016	0.001	1.5933E-07	DCs
DBNL1	1.1011E-11	0.49704316	0.88	0.945	1.6925E-07	DCs
FRY	1.3317E-11	0.11176328	0.148	0.384	2.047E-07	DCs

IRF2BP22	1.4454E-11	1.16144328	0.596	0.699	2.2218E-07	DCs
MMP91	1.4694E-11	2.84314767	0.019	0.002	2.2587E-07	DCs
PAN3	1.4869E-11	1.58750997	0.464	0.437	2.2856E-07	DCs
RHOG1	1.5371E-11	0.56485438	0.77	0.913	2.3626E-07	DCs
PDCD1	1.5407E-11	1.51278126	0.022	0.003	2.3682E-07	DCs
RFLNB1	1.5658E-11	1.23821112	0.063	0.016	2.4068E-07	DCs
RNF1451	1.5989E-11	2.06407219	0.388	0.316	2.4577E-07	DCs
MYADM2	1.7088E-11	0.85047478	0.808	0.875	2.6266E-07	DCs
ADAT1	1.7276E-11	0.14350355	0.192	0.471	2.6555E-07	DCs
LST11	1.8024E-11	0.71841739	0.76	0.886	2.7705E-07	DCs
TOGARAM2	1.8288E-11	3.432847	0.041	0.008	2.811E-07	DCs
DNAJA3	1.9178E-11	0.15679155	0.192	0.467	2.9479E-07	DCs
SMPDL3B	2.0497E-11	3.80460575	0.057	0.014	3.1506E-07	DCs
DCHS12	2.1698E-11	3.49825138	0.057	0.014	3.3352E-07	DCs
TFEB2	2.261E-11	2.40417328	0.274	0.174	3.4754E-07	DCs
ACTG12	2.3049E-11	0.48325158	0.959	0.97	3.5428E-07	DCs
ZBTB101	2.3231E-11	2.63301027	0.353	0.269	3.5709E-07	DCs
TMEM97	2.3958E-11	2.78379387	0.243	0.142	3.6826E-07	DCs
MYH15	2.503E-11	4.66131528	0.025	0.003	3.8474E-07	DCs
GABRR21	2.5325E-11	4.15115269	0.032	0.005	3.8927E-07	DCs
NET1	2.6091E-11	2.37962474	0.391	0.321	4.0105E-07	DCs
POLR2C	2.7967E-11	0.10056934	0.161	0.407	4.2989E-07	DCs
H1F01	3.0729E-11	2.02202435	0.457	0.416	4.7234E-07	DCs
C17orf491	3.1789E-11	1.82158906	0.426	0.379	4.8863E-07	DCs
ATRN2	3.1968E-11	0.13780434	0.183	0.447	4.9138E-07	DCs
KLHL7	3.1989E-11	0.10189731	0.082	0.271	4.917E-07	DCs
C19orf33	3.3563E-11	3.76797705	0.038	0.007	5.1589E-07	DCs
MREG1	3.3586E-11	2.96113155	0.189	0.095	5.1625E-07	DCs
SETD1B1	3.3913E-11	1.18107519	0.552	0.608	5.2128E-07	DCs
DYNC1LI12	3.5863E-11	1.40564434	0.517	0.567	5.5125E-07	DCs
C5orf22	3.6159E-11	0.11942968	0.136	0.364	5.558E-07	DCs
NECTIN22	3.6232E-11	1.92183576	0.464	0.443	5.5693E-07	DCs
ADORA31	3.8179E-11	3.92072142	0.063	0.017	5.8685E-07	DCs
ZMIZ12	3.8675E-11	0.85309368	0.675	0.83	5.9448E-07	DCs
PLCH2	3.9621E-11	3.68889968	0.013	0.001	6.0901E-07	DCs
SMARCA2	4.0344E-11	0.85836682	0.637	0.777	6.2012E-07	DCs
EXOSC7	4.1297E-11	0.13188459	0.161	0.403	6.3478E-07	DCs
PRDM11	4.1921E-11	1.42623969	0.132	0.053	6.4437E-07	DCs
NLRC51	4.2154E-11	2.0205681	0.356	0.267	6.4796E-07	DCs
EIF4E	4.3817E-11	0.12253048	0.218	0.5	6.7351E-07	DCs
COG7	4.5056E-11	0.15991338	0.158	0.394	6.9256E-07	DCs
GOLGA8R	4.578E-11	4.52398165	0.019	0.002	7.0368E-07	DCs
TAGAP2	5.8832E-11	1.38383408	0.546	0.574	9.043E-07	DCs
MAP2K62	7.1229E-11	2.89262318	0.155	0.07	1.0949E-06	DCs
ERCC6	7.1879E-11	2.16259611	0.29	0.194	1.1048E-06	DCs
CCNG1	7.6073E-11	0.11098189	0.136	0.359	1.1693E-06	DCs
OVGP11	7.8016E-11	3.34217958	0.022	0.003	1.1992E-06	DCs
NLRP1	7.9793E-11	1.22755515	0.584	0.647	1.2265E-06	DCs
HNRNPA0	8.2416E-11	0.48789185	0.811	0.914	1.2668E-06	DCs
TM6SF12	8.4895E-11	1.21891708	0.603	0.725	1.3049E-06	DCs
IL4I12	9.7575E-11	4.09220261	0.186	0.095	1.4998E-06	DCs
MTA11	9.9213E-11	1.61123869	0.432	0.39	1.525E-06	DCs

VSIR2	1.0798E-10	1.74541022	0.394	0.325	1.6598E-06	DCs
NFAT5	1.0921E-10	1.87106801	0.426	0.386	1.6786E-06	DCs
EPS15L1	1.1367E-10	0.18329466	0.151	0.382	1.7472E-06	DCs
KIAA1324L	1.26E-10	2.875717	0.032	0.005	1.9368E-06	DCs
VAMP1	1.4011E-10	2.59676933	0.073	0.021	2.1536E-06	DCs
SRFBP1	1.4222E-10	0.19575854	0.205	0.471	2.1861E-06	DCs
ANKH2	1.4233E-10	2.8596924	0.211	0.117	2.1877E-06	DCs
FADS21	1.6469E-10	1.21663303	0.404	0.298	2.5314E-06	DCs
TAF6	1.6789E-10	0.15280962	0.211	0.483	2.5807E-06	DCs
NUDCD2	1.7134E-10	0.13091055	0.224	0.514	2.6336E-06	DCs
CPNE1	1.8661E-10	1.00078956	0.599	0.713	2.8685E-06	DCs
MT-ND41	1.8769E-10	1.56399924	0.476	0.419	2.8851E-06	DCs
TBC1D142	1.9246E-10	1.57166923	0.464	0.451	2.9584E-06	DCs
GTF2A1	2.0538E-10	0.10771246	0.218	0.497	3.157E-06	DCs
PCGF1	2.2496E-10	0.1430007	0.155	0.381	3.4578E-06	DCs
GPR1621	2.2615E-10	2.99659886	0.107	0.041	3.4762E-06	DCs
VPS53	2.4775E-10	0.17869948	0.237	0.521	3.8082E-06	DCs
IGFBP72	2.518E-10	2.5595436	0.101	0.037	3.8705E-06	DCs
UBC1	2.5627E-10	0.93861583	0.612	0.722	3.9392E-06	DCs
KCNC31	2.5876E-10	1.89832133	0.385	0.322	3.9773E-06	DCs
PHETA11	2.5943E-10	2.69619441	0.221	0.128	3.9877E-06	DCs
UROS	2.7206E-10	0.11898101	0.136	0.349	4.1819E-06	DCs
RPIA	2.7687E-10	0.11741677	0.224	0.506	4.2558E-06	DCs
GRAMD42	2.8044E-10	1.9227401	0.401	0.351	4.3106E-06	DCs
GLIPR12	2.8222E-10	0.99495257	0.675	0.787	4.338E-06	DCs
SNAPC21	2.9027E-10	0.18221504	0.155	0.386	4.4618E-06	DCs
SREBF2	2.9053E-10	0.86849215	0.625	0.676	4.4657E-06	DCs
TET22	3.0146E-10	1.9684017	0.369	0.303	4.6338E-06	DCs
THAP4	3.0177E-10	0.12115436	0.183	0.436	4.6385E-06	DCs
CCDC1251	3.0226E-10	0.20496468	0.164	0.397	4.646E-06	DCs
GRIN3B	3.0601E-10	4.00020057	0.013	0.001	4.7037E-06	DCs
ZNF33B1	3.2665E-10	2.54107071	0.107	0.041	5.0209E-06	DCs
PRR5L1	3.3974E-10	2.69547697	0.167	0.082	5.2222E-06	DCs
TAF71	3.441E-10	1.28293884	0.492	0.489	5.2891E-06	DCs
ARRDC52	3.4428E-10	2.73562334	0.082	0.027	5.2919E-06	DCs
MVB12A	3.4955E-10	0.17285457	0.155	0.379	5.3729E-06	DCs
WDR45	3.9577E-10	0.10996361	0.132	0.342	6.0833E-06	DCs
MANBAL	4.0734E-10	0.27202936	0.18	0.424	6.2612E-06	DCs
WNT2B1	4.1973E-10	3.26693044	0.05	0.012	6.4517E-06	DCs
CASC31	4.2351E-10	1.0415181	0.587	0.687	6.5097E-06	DCs
CCDC321	4.2416E-10	0.11681378	0.161	0.395	6.5198E-06	DCs
FAR11	4.3272E-10	0.95549771	0.596	0.692	6.6514E-06	DCs
EI24	4.3826E-10	0.12232756	0.192	0.45	6.7365E-06	DCs
PNPLA8	4.4641E-10	0.15520958	0.145	0.367	6.8618E-06	DCs
CYTH21	4.6263E-10	1.30714112	0.514	0.546	7.1112E-06	DCs
BMP6	4.6696E-10	3.65806038	0.013	0.001	7.1776E-06	DCs
H6PD2	4.7481E-10	0.1373559	0.24	0.524	7.2982E-06	DCs
SLC12A92	4.7846E-10	0.94103701	0.65	0.784	7.3544E-06	DCs
HIST3H2A1	5.0102E-10	2.93627099	0.069	0.021	7.7012E-06	DCs
PRDX6	5.0594E-10	0.134189	0.29	0.622	7.7768E-06	DCs
LRRC32	5.4041E-10	4.66661079	0.013	0.001	8.3067E-06	DCs
ATF31	5.5404E-10	2.36348995	0.278	0.182	8.5162E-06	DCs

MYO5C	5.7655E-10	3.46902081	0.019	0.002	8.8622E-06	DCs
TMEM702	6.0213E-10	0.10681175	0.148	0.369	9.2554E-06	DCs
MTMR4	6.2776E-10	0.50642321	0.145	0.363	9.6494E-06	DCs
ASH1L1	6.3225E-10	1.08664408	0.549	0.602	9.7183E-06	DCs
ZNF5931	6.3236E-10	0.14647429	0.098	0.28	9.7199E-06	DCs
DAPK3	6.9046E-10	0.1023551	0.186	0.433	1.0613E-05	DCs
SOX7	7.4508E-10	4.23913103	0.019	0.002	1.1453E-05	DCs
UTP62	7.4955E-10	1.6642944	0.454	0.446	1.1521E-05	DCs
NAGS	7.8989E-10	2.97892849	0.12	0.05	1.2141E-05	DCs
NUP214	7.9801E-10	0.18719467	0.186	0.429	1.2266E-05	DCs
TMEM102	8.0867E-10	0.22313973	0.161	0.385	1.243E-05	DCs
SLC18B11	8.1203E-10	2.87463718	0.107	0.042	1.2482E-05	DCs
FNIP21	8.407E-10	2.4734954	0.309	0.226	1.2922E-05	DCs
LRRC8C1	8.9891E-10	1.45388974	0.521	0.568	1.3817E-05	DCs
CDC40	9.2203E-10	0.22609836	0.174	0.413	1.4173E-05	DCs
TTLL9	9.3284E-10	3.03895673	0.038	0.008	1.4339E-05	DCs
PATJ	9.4296E-10	0.92520845	0.041	0.009	1.4494E-05	DCs
UTP3	1.0138E-09	0.10269443	0.227	0.506	1.5584E-05	DCs
RIC8A	1.0329E-09	0.25898067	0.208	0.474	1.5877E-05	DCs
RRS1	1.0482E-09	0.19234376	0.158	0.382	1.6111E-05	DCs
NDUFB11	1.0636E-09	0.10143895	0.287	0.598	1.6349E-05	DCs
TIMM50	1.0696E-09	0.24251754	0.243	0.531	1.644E-05	DCs
TXNDC9	1.1215E-09	0.12733624	0.11	0.297	1.7238E-05	DCs
AGO21	1.1673E-09	1.58011096	0.435	0.408	1.7943E-05	DCs
UBL3	1.1956E-09	1.02172951	0.621	0.755	1.8378E-05	DCs
GBGT13	1.2028E-09	0.1477213	0.183	0.417	1.8489E-05	DCs
NUB11	1.2301E-09	1.54363726	0.593	0.736	1.8908E-05	DCs
LARS2	1.2596E-09	0.15024563	0.079	0.244	1.9361E-05	DCs
CIB2	1.2678E-09	4.4975566	0.019	0.002	1.9488E-05	DCs
TNFAIP8L21	1.3138E-09	0.142581	0.259	0.554	2.0195E-05	DCs
HIST1H2BB	1.33E-09	2.38687613	0.06	0.017	2.0444E-05	DCs
FTSJ3	1.3486E-09	0.20195957	0.174	0.405	2.0729E-05	DCs
TNFRSF25	1.3998E-09	0.83322277	0.038	0.008	2.1516E-05	DCs
DDR11	1.4753E-09	3.09866753	0.066	0.02	2.2677E-05	DCs
PNISR1	1.4862E-09	1.32238535	0.486	0.492	2.2845E-05	DCs
SOCS2	1.5049E-09	0.57396957	0.12	0.309	2.3132E-05	DCs
OXER11	1.5301E-09	3.91473796	0.044	0.01	2.352E-05	DCs
CXXC1	1.5353E-09	0.25934481	0.227	0.498	2.3599E-05	DCs
PSMG1	1.5644E-09	0.22326733	0.12	0.316	2.4046E-05	DCs
CMPK2	1.5654E-09	0.12233812	0.271	0.552	2.4062E-05	DCs
TFG	1.5946E-09	0.29017447	0.215	0.481	2.451E-05	DCs
TRABD	1.6266E-09	1.02185096	0.603	0.728	2.5003E-05	DCs
PRDM81	1.635E-09	1.85301177	0.022	0.003	2.5131E-05	DCs
FZD12	1.6356E-09	2.94392134	0.136	0.061	2.514E-05	DCs
SPTSSA1	1.6408E-09	0.12557234	0.281	0.596	2.5221E-05	DCs
CFAP73	1.644E-09	1.89482307	0.013	0.001	2.5271E-05	DCs
WARS1	1.6549E-09	0.84622421	0.77	0.889	2.5438E-05	DCs
EMC8	1.6796E-09	0.22237837	0.224	0.498	2.5818E-05	DCs
SUSD61	1.7379E-09	0.98790676	0.615	0.747	2.6713E-05	DCs
MED241	1.7988E-09	0.12089704	0.186	0.425	2.7649E-05	DCs
ETV31	1.8208E-09	3.07160801	0.309	0.232	2.7988E-05	DCs
SLC35A5	1.8597E-09	0.1667338	0.164	0.386	2.8585E-05	DCs

MPZL11	1.8624E-09	0.15324826	0.136	0.336	2.8627E-05	DCs
FOXN21	1.8695E-09	1.52272899	0.483	0.512	2.8737E-05	DCs
DSE2	1.9186E-09	0.69102745	0.71	0.859	2.949E-05	DCs
SWAP702	1.9305E-09	1.4508642	0.53	0.604	2.9673E-05	DCs
ATP6V0A12	2.0368E-09	0.26549437	0.227	0.503	3.1308E-05	DCs
C5orf15	2.0806E-09	0.35297685	0.199	0.448	3.1981E-05	DCs
TMEM11	2.1054E-09	0.14770201	0.202	0.459	3.2362E-05	DCs
SEC61B	2.211E-09	0.18240079	0.205	0.459	3.3985E-05	DCs
APTX	2.2156E-09	0.19010597	0.126	0.323	3.4056E-05	DCs
RRP1B	2.2678E-09	0.22432182	0.183	0.427	3.4859E-05	DCs
VPS52	2.2881E-09	0.19291741	0.199	0.452	3.517E-05	DCs
YPEL32	2.3028E-09	1.89149824	0.312	0.23	3.5396E-05	DCs
AP1AR1	2.3102E-09	0.13762247	0.155	0.371	3.551E-05	DCs
SINHCAF1	2.3141E-09	3.18570074	0.095	0.036	3.557E-05	DCs
GFPT1	2.3478E-09	0.25159267	0.126	0.323	3.6089E-05	DCs
SAP130	2.3497E-09	0.10296413	0.161	0.38	3.6117E-05	DCs
GOLGA3	2.4804E-09	0.15472139	0.215	0.472	3.8126E-05	DCs
ITFG2	2.5085E-09	0.22950504	0.196	0.441	3.8558E-05	DCs
HNRNPD1	2.6142E-09	0.94444887	0.587	0.719	4.0183E-05	DCs
ZNRD2	2.761E-09	0.1164814	0.117	0.305	4.2439E-05	DCs
TAS2R41	2.7882E-09	2.61298688	0.069	0.022	4.2857E-05	DCs
REPIN1	2.8511E-09	2.06964945	0.397	0.357	4.3825E-05	DCs
SLC25A5	2.8918E-09	0.14723485	0.215	0.476	4.445E-05	DCs
EFNB2	2.9078E-09	4.00910359	0.013	0.001	4.4695E-05	DCs
KAT6A1	2.912E-09	1.47616367	0.457	0.461	4.476E-05	DCs
RNF111	2.9239E-09	0.23061564	0.252	0.536	4.4944E-05	DCs
IGSF211	2.976E-09	3.60017496	0.013	0.001	4.5744E-05	DCs
RYBP1	3.0393E-09	1.8191756	0.401	0.364	4.6718E-05	DCs
PSMD9	3.0464E-09	0.1218519	0.29	0.607	4.6827E-05	DCs
FOSB2	3.1062E-09	2.14546572	0.274	0.183	4.7745E-05	DCs
ITPRID22	3.187E-09	1.23330118	0.546	0.634	4.8987E-05	DCs
DMWD	3.1932E-09	1.52978756	0.035	0.007	4.9082E-05	DCs
RRAD1	3.194E-09	3.84006133	0.019	0.002	4.9094E-05	DCs
WDFY33	3.2543E-09	0.14996865	0.177	0.405	5.0021E-05	DCs
RGS22	3.2564E-09	1.31920174	0.517	0.492	5.0055E-05	DCs
ASAP11	3.3809E-09	1.40474625	0.58	0.725	5.1968E-05	DCs
ORAI12	3.3912E-09	1.35225566	0.546	0.641	5.2126E-05	DCs
SIN3A	3.4048E-09	1.27071858	0.489	0.508	5.2335E-05	DCs
HSPA4	3.4297E-09	0.25219223	0.199	0.442	5.2719E-05	DCs
PPA21	3.4485E-09	0.23800445	0.252	0.547	5.3006E-05	DCs
PLGRKT	3.4637E-09	0.13298273	0.148	0.36	5.324E-05	DCs
HSPBP11	3.4882E-09	0.12844813	0.151	0.361	5.3617E-05	DCs
ZNF33A1	3.5978E-09	1.95120673	0.347	0.275	5.5302E-05	DCs
FOXRED1	3.6034E-09	0.17266237	0.091	0.261	5.5387E-05	DCs
CCT31	3.6176E-09	0.15530261	0.148	0.359	5.5606E-05	DCs
CYTH41	3.626E-09	1.38809763	0.517	0.561	5.5735E-05	DCs
DDX19A	3.7145E-09	0.13966081	0.177	0.41	5.7095E-05	DCs
DHX40	3.7303E-09	0.17353423	0.17	0.397	5.7339E-05	DCs
ABHD11	3.7927E-09	0.24003005	0.11	0.288	5.8298E-05	DCs
PSME2	3.8909E-09	0.26217357	0.12	0.309	5.9807E-05	DCs
NREP2	4.0129E-09	2.52083453	0.211	0.126	6.1683E-05	DCs
ZNF175	4.0892E-09	0.30328013	0.114	0.297	6.2855E-05	DCs

ZNF6182	4.1989E-09	3.23228106	0.101	0.04	6.4541E-05	DCs
TRIM4	4.2048E-09	0.12340373	0.233	0.504	6.4632E-05	DCs
AIFM1	4.2388E-09	0.14648953	0.17	0.395	6.5155E-05	DCs
ITPKB	4.328E-09	0.225682	0.237	0.505	6.6526E-05	DCs
YIPF2	4.3652E-09	0.14913929	0.139	0.343	6.7097E-05	DCs
TLR22	4.3797E-09	1.89991287	0.379	0.325	6.7321E-05	DCs
IL6R2	4.4012E-09	2.23650715	0.265	0.179	6.7651E-05	DCs
GHRL1	4.4634E-09	4.32042364	0.019	0.002	6.8606E-05	DCs
COX18	4.4762E-09	0.32712088	0.189	0.429	6.8803E-05	DCs
ADPRHL2	4.5042E-09	0.12015499	0.268	0.557	6.9234E-05	DCs
SIRT6	4.5215E-09	0.15832241	0.114	0.298	6.9499E-05	DCs
ALDH16A1	4.6834E-09	0.1301947	0.237	0.511	7.1988E-05	DCs
TAF5L	4.7032E-09	0.18360623	0.167	0.39	7.2293E-05	DCs
FAM20B	4.7077E-09	0.13657918	0.167	0.385	7.2362E-05	DCs
CD300A2	4.7939E-09	1.89115006	0.401	0.361	7.3688E-05	DCs
CCDC90B	4.9871E-09	0.32064352	0.18	0.412	7.6657E-05	DCs
GFM2	5.0289E-09	0.13790952	0.114	0.296	7.7299E-05	DCs
CSGALNACT22	5.1272E-09	1.3721543	0.479	0.496	7.881E-05	DCs
CAMKK21	5.2477E-09	0.2045612	0.309	0.633	8.0662E-05	DCs
ZNF646	5.2917E-09	0.26228093	0.139	0.338	8.1339E-05	DCs
SHFL	5.2951E-09	0.166573	0.221	0.469	8.139E-05	DCs
BAP1	5.3181E-09	0.13852016	0.161	0.373	8.1745E-05	DCs
TMPO	5.3356E-09	0.11087964	0.199	0.435	8.2014E-05	DCs
N4BP3	5.4287E-09	2.26008226	0.013	0.001	8.3445E-05	DCs
TRIM22	5.5433E-09	0.51117893	0.798	0.921	8.5206E-05	DCs
INTS6L1	5.6029E-09	2.48796043	0.129	0.059	8.6123E-05	DCs
CNOT6	5.6427E-09	0.10524222	0.18	0.408	8.6733E-05	DCs
MTOR	5.7176E-09	0.17584209	0.202	0.448	8.7885E-05	DCs
PPIE	5.8726E-09	0.12847938	0.199	0.442	9.0268E-05	DCs
INPP5K	5.9187E-09	0.25938204	0.256	0.544	9.0976E-05	DCs
ST3GAL51	6.0399E-09	2.01215314	0.483	0.514	9.284E-05	DCs
MAP3K211	6.1439E-09	2.82173043	0.126	0.057	9.4437E-05	DCs
HIVEP2	6.2449E-09	2.45609391	0.18	0.098	9.599E-05	DCs
RAB28	6.4651E-09	0.13943489	0.11	0.287	9.9375E-05	DCs
SLMAP	6.4944E-09	0.19903276	0.161	0.376	9.9825E-05	DCs
SOX5	6.5244E-09	5.4429806	0.013	0.001	0.00010029	DCs
PIGV	6.5293E-09	0.14272028	0.117	0.299	0.00010036	DCs
ELP5	6.5517E-09	0.27617649	0.221	0.486	0.00010071	DCs
STRAP	6.5872E-09	0.12010149	0.338	0.693	0.00010125	DCs
KLHDC10	6.589E-09	0.12949301	0.155	0.363	0.00010128	DCs
TMEM106A2	6.5931E-09	1.42872049	0.445	0.43	0.00010134	DCs
XAB2	6.709E-09	0.12419962	0.303	0.623	0.00010312	DCs
SIAH12	7.1697E-09	1.47290607	0.467	0.48	0.00011021	DCs
HOOK31	7.1725E-09	1.19675734	0.552	0.657	0.00011025	DCs
PRORP	7.2108E-09	0.14603999	0.11	0.287	0.00011084	DCs
SEH1L1	7.3133E-09	0.17240847	0.243	0.507	0.00011241	DCs
CARD192	7.3461E-09	1.88389127	0.303	0.224	0.00011292	DCs
SMIM7	7.3971E-09	0.12207024	0.246	0.524	0.0001137	DCs
LMO22	7.6207E-09	1.2766486	0.546	0.621	0.00011714	DCs
MRM21	7.6348E-09	0.19782928	0.17	0.393	0.00011735	DCs
PELP1	7.8518E-09	0.21358024	0.186	0.412	0.00012069	DCs
ARMT11	8.0149E-09	0.12332682	0.104	0.28	0.0001232	DCs

NUP37	8.0373E-09	0.20177401	0.114	0.291	0.00012354	DCs
SNRK	8.3131E-09	0.10847881	0.246	0.522	0.00012778	DCs
GPKOW	8.409E-09	0.26802502	0.142	0.344	0.00012925	DCs
NDC1	8.474E-09	0.22598752	0.136	0.329	0.00013025	DCs
ATF61	8.491E-09	0.1249405	0.319	0.645	0.00013051	DCs
UBN1	8.592E-09	0.10827675	0.183	0.412	0.00013207	DCs
STRBP	8.6231E-09	2.14409486	0.205	0.121	0.00013255	DCs
INVS	8.7843E-09	0.19297728	0.12	0.302	0.00013502	DCs
VAPB	9.0224E-09	0.12273783	0.136	0.327	0.00013868	DCs
EPCAM1	9.2183E-09	3.32950541	0.016	0.002	0.00014169	DCs
PRKCE1	9.2487E-09	0.33710793	0.148	0.35	0.00014216	DCs
BLOC1S5	9.3312E-09	0.12406809	0.095	0.259	0.00014343	DCs
COQ6	9.3315E-09	0.11428023	0.101	0.27	0.00014343	DCs
KAT5	9.4926E-09	0.12934651	0.199	0.438	0.00014591	DCs
HSPD11	9.5681E-09	0.11895776	0.284	0.593	0.00014707	DCs
ITPA	9.5825E-09	0.24904666	0.303	0.626	0.00014729	DCs
FAM214B1	9.754E-09	0.17308778	0.145	0.345	0.00014993	DCs
GASK1B2	1.0043E-08	2.79052211	0.082	0.029	0.00015437	DCs
ZDHHC172	1.0113E-08	1.84657397	0.338	0.273	0.00015545	DCs
POLD2	1.0237E-08	0.17656321	0.117	0.296	0.00015735	DCs
RPF2	1.0322E-08	0.1746829	0.095	0.259	0.00015865	DCs
VAMP7	1.0368E-08	0.11078489	0.23	0.491	0.00015936	DCs
TRIM721	1.1123E-08	5.23468054	0.013	0.001	0.00017098	DCs
IER51	1.174E-08	0.75341266	0.738	0.881	0.00018046	DCs
NFE2L32	1.2239E-08	2.54464404	0.199	0.117	0.00018812	DCs
VRK3	1.2566E-08	0.10145333	0.145	0.342	0.00019315	DCs
PDE7A1	1.3247E-08	1.63543727	0.325	0.249	0.00020362	DCs
GATB	1.3527E-08	0.11921979	0.123	0.303	0.00020793	DCs
NIP7	1.3591E-08	0.34360261	0.164	0.375	0.00020891	DCs
CNOT81	1.3882E-08	1.1547229	0.543	0.653	0.00021338	DCs
MAP3K82	1.417E-08	0.84383684	0.688	0.778	0.00021781	DCs
TMEM218	1.4179E-08	0.25043591	0.151	0.356	0.00021794	DCs
WRAP73	1.4205E-08	0.13546331	0.132	0.322	0.00021834	DCs
YIF1A	1.5359E-08	0.10016679	0.265	0.546	0.00023608	DCs
CLPP	1.6486E-08	0.18800137	0.262	0.544	0.00025341	DCs
DCTPP1	1.6635E-08	0.22823309	0.237	0.499	0.0002557	DCs
SCAMP4	1.6744E-08	0.12416857	0.293	0.598	0.00025737	DCs
QKI2	1.6818E-08	0.46072724	0.836	0.941	0.00025851	DCs
RARS	1.7274E-08	0.19841544	0.252	0.521	0.00026552	DCs
MT-ND4L2	1.7532E-08	1.71760478	0.363	0.29	0.00026949	DCs
GADD45B2	1.7571E-08	2.28498886	0.243	0.159	0.00027008	DCs
FARSB	1.7608E-08	0.26234126	0.208	0.452	0.00027066	DCs
SH2B31	1.8023E-08	0.94982345	0.637	0.816	0.00027703	DCs
NUDT5	1.8566E-08	0.22198654	0.243	0.506	0.00028537	DCs
UCHL3	1.8594E-08	0.24401076	0.129	0.317	0.0002858	DCs
FOS2	1.8613E-08	0.91326675	0.839	0.95	0.0002861	DCs
MAGOH	1.8653E-08	0.22528281	0.183	0.405	0.00028671	DCs
DVL2	1.899E-08	0.24490809	0.123	0.305	0.0002919	DCs
KIAA1143	1.9044E-08	0.22920688	0.151	0.35	0.00029273	DCs
TSPYL1	1.9843E-08	0.14599309	0.24	0.509	0.00030501	DCs
WNK11	2.0049E-08	0.64813577	0.672	0.834	0.00030818	DCs
ADM51	2.0099E-08	3.6848743	0.032	0.006	0.00030894	DCs

USB1	2.0167E-08	0.2274074	0.262	0.55	0.00030999	DCs
FAM53C1	2.0233E-08	0.24421845	0.218	0.469	0.000311	DCs
OGFOD1	2.0243E-08	0.30300422	0.23	0.496	0.00031116	DCs
ANKRD40	2.0479E-08	0.11523164	0.278	0.571	0.00031478	DCs
GARNL31	2.0585E-08	4.19727734	0.022	0.003	0.00031642	DCs
NOC2L1	2.0591E-08	0.11925268	0.177	0.395	0.0003165	DCs
PIK3C3	2.0785E-08	0.27231918	0.167	0.383	0.00031948	DCs
PSMG3	2.1105E-08	0.20512057	0.208	0.442	0.00032441	DCs
RNASEH1	2.1264E-08	0.24164209	0.196	0.431	0.00032685	DCs
RBM51	2.1413E-08	1.50465564	0.416	0.394	0.00032914	DCs
BET1	2.1492E-08	0.10325661	0.114	0.287	0.00033035	DCs
COX11	2.2181E-08	0.38558618	0.123	0.305	0.00034094	DCs
PCMTD1	2.2509E-08	0.82518522	0.612	0.762	0.00034599	DCs
PHC22	2.2978E-08	1.23208501	0.53	0.609	0.00035319	DCs
BRK1	2.3191E-08	0.26212832	0.202	0.438	0.00035647	DCs
RFXAP	2.3307E-08	2.55958682	0.161	0.086	0.00035825	DCs
NUFIP22	2.3572E-08	0.87346494	0.621	0.773	0.00036233	DCs
RAB5B	2.4797E-08	0.22005058	0.356	0.707	0.00038115	DCs
VN1R1	2.5097E-08	3.47905843	0.041	0.01	0.00038577	DCs
NIPAL3	2.5712E-08	0.25861953	0.117	0.291	0.00039521	DCs
TMEM86A2	2.5728E-08	0.16337648	0.202	0.43	0.00039547	DCs
C1orf174	2.6117E-08	0.13006023	0.284	0.585	0.00040144	DCs
MED141	2.7076E-08	1.71887384	0.438	0.445	0.00041618	DCs
NUDT2	2.715E-08	0.25234784	0.196	0.424	0.00041733	DCs
MPC2	2.7426E-08	0.15663226	0.271	0.551	0.00042157	DCs
INIP	2.8188E-08	0.2159952	0.218	0.462	0.00043327	DCs
SNX91	2.8206E-08	0.15996385	0.278	0.569	0.00043356	DCs
SIGIRR2	2.8265E-08	1.60093885	0.259	0.177	0.00043446	DCs
SRA1	2.9141E-08	0.15291585	0.281	0.566	0.00044793	DCs
AKAP10	2.9584E-08	0.2038342	0.208	0.444	0.00045474	DCs
GALNT4	2.9843E-08	0.29647102	0.076	0.221	0.00045872	DCs
TNFSF102	2.9958E-08	0.10200447	0.265	0.529	0.00046049	DCs
PAGR11	3.0033E-08	0.16177921	0.208	0.447	0.00046163	DCs
PLEKHA72	3.0179E-08	2.73781208	0.199	0.119	0.00046388	DCs
UBA5	3.0611E-08	0.25007213	0.132	0.316	0.00047052	DCs
RWDD1	3.1025E-08	0.15873304	0.227	0.482	0.00047688	DCs
LRRC75B1	3.1317E-08	3.74406015	0.022	0.004	0.00048138	DCs
COG2	3.1522E-08	0.25101043	0.129	0.31	0.00048453	DCs
RPF1	3.1549E-08	0.11380416	0.151	0.345	0.00048494	DCs
RASSF42	3.1982E-08	1.6190292	0.539	0.613	0.0004916	DCs
UNC5CL	3.2443E-08	3.36082161	0.028	0.005	0.00049868	DCs
NOP9	3.2529E-08	0.25970636	0.192	0.419	0.0005	DCs
RAD50	3.3142E-08	0.16060707	0.227	0.475	0.00050942	DCs
FBXO28	3.3537E-08	0.18632861	0.199	0.426	0.0005155	DCs
NELFCD	3.4301E-08	0.11901158	0.227	0.478	0.00052725	DCs
ZBTB22	3.4442E-08	0.21596974	0.136	0.32	0.00052941	DCs
THOP1	3.4612E-08	0.1007252	0.142	0.331	0.00053202	DCs
SENP5	3.4701E-08	0.16202947	0.183	0.403	0.00053339	DCs
PTRH2	3.608E-08	0.13433958	0.095	0.251	0.00055459	DCs
SLC30A5	3.6283E-08	0.1421263	0.142	0.329	0.00055771	DCs
SNW1	3.6318E-08	0.21893752	0.155	0.357	0.00055825	DCs
SIPA1L3	3.6522E-08	1.99449267	0.338	0.282	0.00056138	DCs

STRIP1	3.7803E-08	0.20305587	0.202	0.438	0.00058107	DCs
HTT	3.9389E-08	0.17574043	0.369	0.716	0.00060544	DCs
ZBTB17	4.0992E-08	0.11362854	0.11	0.274	0.00063009	DCs
RBPJ2	4.226E-08	1.13223461	0.593	0.719	0.00064958	DCs
SLC9A8	4.23E-08	0.14677377	0.12	0.289	0.0006502	DCs
SLC25A44	4.2797E-08	0.19474347	0.18	0.398	0.00065783	DCs
CXCR2	4.294E-08	0.12889802	0.038	0.155	0.00066004	DCs
ENO21	4.2952E-08	0.58397901	0.038	0.009	0.00066022	DCs
CDC27	4.3031E-08	0.23421582	0.095	0.251	0.00066142	DCs
APC	4.3129E-08	0.1721655	0.177	0.39	0.00066294	DCs
RAVER2	4.3299E-08	2.82891395	0.101	0.042	0.00066555	DCs
HGH1	4.3521E-08	0.27063381	0.126	0.302	0.00066897	DCs
LANCL2	4.401E-08	0.16536864	0.104	0.263	0.00067648	DCs
TRIM651	4.4111E-08	0.23820293	0.183	0.399	0.00067802	DCs
FBXO38	4.4501E-08	0.27824786	0.205	0.439	0.00068403	DCs
SBF21	4.511E-08	0.23964822	0.18	0.39	0.00069338	DCs
LSG1	4.5542E-08	0.33689649	0.208	0.444	0.00070003	DCs
LRRC8A	4.6699E-08	0.13886852	0.129	0.309	0.00071782	DCs
JAG11	4.7181E-08	0.23563378	0.073	0.213	0.00072522	DCs
AACS	4.836E-08	0.16488046	0.139	0.324	0.00074334	DCs
SLC35B41	4.9175E-08	0.11524123	0.123	0.293	0.00075587	DCs
UMAD1	4.9278E-08	0.24818313	0.101	0.258	0.00075745	DCs
LCOR1	4.9357E-08	1.37956565	0.498	0.547	0.00075867	DCs
TIMM17A	5.0904E-08	0.40609077	0.265	0.552	0.00078244	DCs
NOL11	5.1314E-08	0.16780031	0.114	0.281	0.00078875	DCs
GTF2H3	5.2324E-08	0.10274484	0.136	0.318	0.00080426	DCs
DDI2	5.2432E-08	0.2381998	0.151	0.344	0.00080594	DCs
DNAJC11	5.2823E-08	0.12215613	0.23	0.477	0.00081194	DCs
NLRP21	5.3493E-08	0.15070175	0.079	0.222	0.00082224	DCs
RNF6	5.3516E-08	0.2717077	0.252	0.527	0.0008226	DCs
AGGF1	5.382E-08	0.18732216	0.281	0.576	0.00082727	DCs
GCC2	5.4294E-08	0.33006747	0.221	0.465	0.00083456	DCs
BTAF11	5.4553E-08	1.50746361	0.426	0.413	0.00083854	DCs
NEDD1	5.7794E-08	0.19650514	0.085	0.233	0.00088835	DCs
ZNF385A2	5.7877E-08	0.75432362	0.672	0.822	0.00088963	DCs
OXA1L1	5.8215E-08	0.84486247	0.625	0.781	0.00089482	DCs
GTF2B	5.8445E-08	0.18001365	0.3	0.607	0.00089836	DCs
OSBPL2	5.879E-08	0.26938158	0.246	0.513	0.00090366	DCs
RAVER1	6.0175E-08	0.15730074	0.256	0.526	0.00092495	DCs
CINP	6.0382E-08	0.15038411	0.117	0.286	0.00092813	DCs
FAM160B2	6.2809E-08	0.19147985	0.132	0.31	0.00096543	DCs
HDAC71	6.7646E-08	1.86534131	0.297	0.226	0.00103979	DCs
AARS2	6.7676E-08	0.17337051	0.123	0.294	0.00104026	DCs
ZC3H3	6.8307E-08	0.24074464	0.145	0.331	0.00104995	DCs
CAMSAP21	6.8365E-08	0.20320294	0.221	0.46	0.00105084	DCs
FBXO46	6.9107E-08	0.22035631	0.218	0.459	0.00106224	DCs
ITGB22	7.062E-08	0.33885719	0.909	0.961	0.0010855	DCs
GOSR1	7.0843E-08	0.22519522	0.237	0.487	0.00108894	DCs
MARCH5	7.1787E-08	0.25514212	0.221	0.462	0.00110343	DCs
ISG20L2	7.1793E-08	0.2561364	0.17	0.377	0.00110353	DCs
NUP133	7.181E-08	0.18933279	0.218	0.454	0.0011038	DCs
TENT4B	7.258E-08	0.17611834	0.132	0.311	0.00111562	DCs

VPS33A	7.3101E-08	0.10161275	0.104	0.261	0.00112363	DCs
PCBP12	7.3387E-08	0.87419981	0.612	0.756	0.00112803	DCs
EDARADD	7.3501E-08	1.95209078	0.016	0.002	0.00112978	DCs
ARHGEF12	7.5481E-08	0.89145595	0.596	0.718	0.00116022	DCs
NFATC3	7.595E-08	0.17558607	0.246	0.511	0.00116743	DCs
MEN1	7.6031E-08	0.1660551	0.11	0.275	0.00116868	DCs
CHUK	7.7092E-08	0.23694626	0.202	0.427	0.00118498	DCs
CUL4A	7.8752E-08	0.15278897	0.249	0.515	0.0012105	DCs
GAMT	7.8829E-08	3.48736871	0.022	0.004	0.00121168	DCs
TADA2B	7.9305E-08	0.17363545	0.218	0.461	0.001219	DCs
WIPF2	8.0663E-08	0.10162963	0.297	0.599	0.00123986	DCs
RAB2B	8.2946E-08	0.28825674	0.202	0.427	0.00127496	DCs
VPS29	8.4044E-08	0.42580484	0.136	0.316	0.00129184	DCs
ZNF622	8.6558E-08	0.12309632	0.24	0.498	0.00133049	DCs
USP322	8.6692E-08	1.15996597	0.539	0.651	0.00133255	DCs
HIST1H4B	8.8696E-08	3.76725174	0.016	0.002	0.00136335	DCs
SP31	8.9288E-08	0.94393323	0.603	0.751	0.00137244	DCs
C17orf75	8.985E-08	0.21809023	0.073	0.208	0.00138108	DCs
TESK1	9.0147E-08	0.2812394	0.23	0.481	0.00138565	DCs
PSEN21	9.1822E-08	0.21396026	0.148	0.337	0.00141139	DCs
CHST132	9.2414E-08	4.48843237	0.016	0.002	0.00142049	DCs
KANSL1L	9.3442E-08	0.21033815	0.11	0.271	0.0014363	DCs
ADPRH1	9.3691E-08	0.14999625	0.338	0.654	0.00144013	DCs
STK11	9.4261E-08	0.18965906	0.303	0.608	0.00144889	DCs
AC021097.2	9.4968E-08	3.17881982	0.022	0.004	0.00145976	DCs
GNL2	9.7225E-08	0.16812717	0.284	0.571	0.00149445	DCs
WDR821	9.8522E-08	1.06819619	0.543	0.66	0.00151438	DCs
UTP23	9.9512E-08	0.14114716	0.199	0.421	0.0015296	DCs
CARNMT1	1.0065E-07	0.11602997	0.142	0.322	0.0015471	DCs
SNX13	1.0104E-07	0.20280432	0.259	0.532	0.00155302	DCs
BYSL1	1.021E-07	0.24833801	0.104	0.263	0.0015693	DCs
SCFD1	1.0307E-07	0.25777581	0.3	0.609	0.00158434	DCs
STK16	1.0468E-07	0.13493985	0.123	0.293	0.00160906	DCs
TCTN3	1.0683E-07	0.68676737	0.211	0.449	0.00164213	DCs
RCN12	1.0692E-07	0.21295937	0.224	0.464	0.00164346	DCs
AREL1	1.0733E-07	0.11168408	0.095	0.245	0.00164982	DCs
SS18L2	1.0754E-07	0.22117783	0.139	0.318	0.00165305	DCs
ODR4	1.0785E-07	0.31717374	0.174	0.375	0.00165777	DCs
NEK9	1.0797E-07	0.21376993	0.265	0.537	0.00165963	DCs
NUP85	1.0805E-07	0.30014085	0.23	0.477	0.00166088	DCs
RTKN1	1.1067E-07	4.08179033	0.025	0.005	0.0017011	DCs
NUP50	1.1179E-07	0.29177031	0.205	0.431	0.00171834	DCs
ZBTB45	1.1189E-07	0.10841228	0.148	0.329	0.00171983	DCs
UBAP2	1.1212E-07	0.30622797	0.11	0.27	0.00172346	DCs
LGALS11	1.133E-07	0.39681276	0.94	0.966	0.00174148	DCs
USP8	1.1424E-07	0.46616862	0.205	0.433	0.00175596	DCs
FMC1	1.1521E-07	0.32833069	0.142	0.324	0.00177096	DCs
EXOC1	1.1528E-07	0.11957727	0.29	0.577	0.00177202	DCs
BID1	1.1643E-07	0.15145435	0.341	0.67	0.00178959	DCs
PRPF31	1.2E-07	0.13122805	0.265	0.539	0.00184456	DCs
TMEM69	1.201E-07	0.20046441	0.129	0.305	0.001846	DCs
RABGEF1	1.2036E-07	0.39837416	0.183	0.396	0.00185003	DCs

TTC271	1.219E-07	0.11861287	0.11	0.269	0.00187376	DCs
PSPH1	1.2555E-07	0.21281817	0.073	0.205	0.00192985	DCs
CHM	1.2569E-07	0.14891629	0.114	0.276	0.00193204	DCs
XPO7	1.2675E-07	0.23395848	0.196	0.412	0.00194821	DCs
WBP11	1.3011E-07	0.13097507	0.132	0.308	0.00199995	DCs
EXOSC3	1.3021E-07	0.1546747	0.136	0.312	0.00200149	DCs
ZNF6412	1.3171E-07	1.7814252	0.369	0.333	0.00202456	DCs
CHERP	1.322E-07	0.23605206	0.218	0.454	0.00203204	DCs
SPPL2B	1.3238E-07	0.16419528	0.164	0.361	0.00203485	DCs
OPA1	1.3316E-07	0.14266725	0.29	0.58	0.00204683	DCs
AHDC11	1.3323E-07	2.43920119	0.12	0.057	0.00204786	DCs
SEC22A	1.3486E-07	0.13310767	0.104	0.258	0.00207299	DCs
RDX	1.3506E-07	0.32455885	0.148	0.332	0.00207607	DCs
MSANTD3	1.3526E-07	0.18503245	0.126	0.294	0.00207901	DCs
CCT2	1.3563E-07	0.37406324	0.148	0.33	0.00208473	DCs
LEMD2	1.3568E-07	0.251922	0.211	0.446	0.00208552	DCs
RFK	1.3647E-07	0.19722983	0.136	0.311	0.00209764	DCs
PAPOLA	1.3656E-07	0.1393534	0.29	0.59	0.00209911	DCs
STK25	1.3743E-07	0.14085426	0.268	0.537	0.0021124	DCs
ARSD1	1.3747E-07	0.27906241	0.085	0.228	0.00211309	DCs
TRNAU1AP	1.3936E-07	0.30844628	0.211	0.438	0.00214208	DCs
SMIM20	1.4005E-07	0.35049145	0.095	0.245	0.00215268	DCs
MBD2	1.4133E-07	0.44389962	0.871	0.955	0.00217236	DCs
MORF4L2	1.4611E-07	0.16456962	0.123	0.29	0.00224591	DCs
SETDB11	1.4648E-07	0.34189402	0.199	0.418	0.00225147	DCs
PM20D2	1.4695E-07	2.4630441	0.136	0.069	0.00225881	DCs
CFAP20	1.4768E-07	0.1408315	0.107	0.265	0.00226998	DCs
RNF214	1.4846E-07	0.31393531	0.126	0.297	0.00228201	DCs
BBX	1.5218E-07	0.35188042	0.174	0.376	0.00233923	DCs
FAM110A	1.5496E-07	0.38210143	0.161	0.356	0.00238193	DCs
BCKDHA1	1.5528E-07	0.19438297	0.24	0.493	0.00238684	DCs
WBP41	1.5606E-07	0.26834823	0.139	0.315	0.00239873	DCs
LDLRAD4	1.5607E-07	1.64430252	0.432	0.438	0.00239895	DCs
PET100	1.5734E-07	0.20209733	0.265	0.542	0.00241848	DCs
SND11	1.5778E-07	0.10632948	0.331	0.654	0.00242527	DCs
DOK11	1.6561E-07	0.15642478	0.246	0.499	0.00254563	DCs
OSBP	1.6708E-07	0.15397192	0.297	0.591	0.00256826	DCs
TYW3	1.6717E-07	0.28691292	0.151	0.337	0.00256954	DCs
TKFC1	1.6813E-07	0.11285539	0.151	0.332	0.00258435	DCs
MRS2	1.6945E-07	0.19895238	0.221	0.454	0.00260456	DCs
HABP4	1.6975E-07	0.2484817	0.079	0.216	0.00260917	DCs
DHRS13	1.7046E-07	2.43937783	0.136	0.069	0.00262014	DCs
FAM192A	1.7391E-07	0.15799528	0.107	0.264	0.00267312	DCs
ATMIN	1.7412E-07	0.14925977	0.164	0.359	0.00267647	DCs
TCP11	1.7549E-07	0.15711619	0.262	0.532	0.0026975	DCs
CCT4	1.7601E-07	0.20930144	0.215	0.447	0.00270542	DCs
MAP4K5	1.7641E-07	0.21245919	0.227	0.465	0.00271163	DCs
STAMBP	1.7997E-07	0.16301994	0.167	0.36	0.00276627	DCs
GOLGA1	1.8073E-07	0.29890641	0.174	0.371	0.00277802	DCs
HDGFL2	1.8078E-07	0.15188841	0.268	0.538	0.00277878	DCs
ELP1	1.833E-07	0.25029117	0.132	0.303	0.00281751	DCs
LPCAT4	1.8476E-07	0.22231263	0.167	0.357	0.00283993	DCs

TOB11	1.8638E-07	1.33279528	0.492	0.531	0.00286483	DCs
PLAC8L1	1.8664E-07	3.66947917	0.016	0.002	0.00286883	DCs
PGAM5	1.9304E-07	0.20632709	0.161	0.35	0.0029672	DCs
ITPRIP2	1.9324E-07	0.21754699	0.218	0.448	0.00297022	DCs
GOLGA2	1.9649E-07	0.27886867	0.145	0.325	0.00302017	DCs
DHX30	1.9756E-07	0.22176733	0.24	0.494	0.00303674	DCs
PIGN	1.9868E-07	0.22663264	0.17	0.369	0.00305392	DCs
KLHDC2	1.993E-07	0.16616662	0.199	0.415	0.00306336	DCs
PLCXD11	2.0046E-07	0.3304713	0.069	0.198	0.00308131	DCs
ADRB21	2.0315E-07	0.25703203	0.224	0.451	0.00312266	DCs
STARD71	2.0608E-07	0.33701726	0.252	0.522	0.00316766	DCs
SEL1L3	2.0913E-07	1.31973724	0.517	0.588	0.00321452	DCs
ZNF821	2.0998E-07	0.2690574	0.057	0.177	0.00322767	DCs
CAAP1	2.1034E-07	0.28517459	0.101	0.25	0.0032331	DCs
DOCK71	2.1664E-07	0.21779094	0.237	0.483	0.00333004	DCs
GPR842	2.1729E-07	3.25394488	0.063	0.022	0.00333994	DCs
UBE2G2	2.1857E-07	0.11135526	0.334	0.657	0.00335968	DCs
NSL1	2.1955E-07	0.25457891	0.243	0.497	0.00337463	DCs
RIOX1	2.2127E-07	0.2000198	0.17	0.365	0.00340113	DCs
EXOC41	2.2447E-07	0.16241322	0.218	0.445	0.00345035	DCs
GTF3C1	2.2493E-07	0.21299527	0.186	0.398	0.00345742	DCs
XPA	2.2532E-07	0.41761686	0.151	0.338	0.00346346	DCs
NFKBIL1	2.2659E-07	0.39772382	0.12	0.281	0.00348284	DCs
IMPA1	2.2752E-07	0.35797067	0.148	0.33	0.00349726	DCs
TBL21	2.2788E-07	0.2864048	0.189	0.396	0.00350281	DCs
SP140	2.2889E-07	0.19979339	0.224	0.451	0.0035183	DCs
SLC15A22	2.3064E-07	2.68250605	0.101	0.045	0.00354524	DCs
PHAX	2.322E-07	0.18567278	0.205	0.424	0.00356918	DCs
UCKL1	2.3258E-07	0.21490822	0.177	0.375	0.00357495	DCs
DDX39A1	2.3892E-07	0.25418513	0.215	0.441	0.00367247	DCs
TPGS1	2.4181E-07	0.23597382	0.268	0.542	0.00371689	DCs
BTBD192	2.4234E-07	2.38847423	0.123	0.06	0.00372503	DCs
ZER1	2.4267E-07	0.31317791	0.249	0.499	0.00373003	DCs
NKTR1	2.4726E-07	1.46680473	0.397	0.368	0.00380057	DCs
GRAMD1B	2.4764E-07	0.54866216	0.167	0.361	0.00380644	DCs
NMNAT11	2.4838E-07	0.13636862	0.12	0.279	0.00381779	DCs
ACP61	2.4856E-07	0.16315915	0.088	0.229	0.00382066	DCs
ELOF1	2.488E-07	0.22107011	0.268	0.542	0.00382429	DCs
GPR55	2.5144E-07	4.20568199	0.019	0.003	0.00386483	DCs
KIF1BP	2.5277E-07	0.12731139	0.11	0.265	0.00388528	DCs
NCAPD3	2.5348E-07	0.15820889	0.139	0.31	0.00389618	DCs
PGAP2	2.536E-07	0.2020345	0.129	0.296	0.00389809	DCs
PPP1R8	2.6196E-07	0.36762229	0.17	0.37	0.00402657	DCs
RSBN1	2.6692E-07	0.33119178	0.158	0.346	0.00410283	DCs
CCNQ	2.6727E-07	0.17195198	0.129	0.296	0.00410826	DCs
AAAS	2.6767E-07	0.15499877	0.221	0.452	0.00411441	DCs
C1QBP1	2.7032E-07	0.29667474	0.158	0.346	0.00415505	DCs
TMEM651	2.7229E-07	2.03234657	0.218	0.143	0.00418532	DCs
OPA3	2.7365E-07	0.21987765	0.079	0.212	0.00420627	DCs
PANK4	2.751E-07	0.24407303	0.167	0.359	0.00422852	DCs
WWP2	2.7841E-07	0.22433702	0.297	0.585	0.00427951	DCs
CDC23	2.7881E-07	0.26312379	0.155	0.339	0.00428554	DCs

GPR341	2.7978E-07	0.38724326	0.142	0.316	0.00430052	DCs
MBD1	2.8355E-07	0.14369812	0.11	0.264	0.00435843	DCs
THUMPD3	2.8503E-07	0.20099037	0.192	0.401	0.00438121	DCs
ATP7A	2.8805E-07	0.26519281	0.114	0.27	0.00442764	DCs
FSD1L	2.8845E-07	0.23660941	0.117	0.275	0.00443384	DCs
ZNF639	2.9282E-07	0.25996145	0.104	0.255	0.004501	DCs
DENND32	2.9478E-07	2.09452511	0.312	0.254	0.00453104	DCs
TIPARP	2.9496E-07	0.22039739	0.356	0.681	0.00453386	DCs
ORC4	2.9718E-07	0.17913657	0.132	0.301	0.00456791	DCs
NUP93	2.9738E-07	0.25607976	0.306	0.598	0.00457104	DCs
ZSCAN32	2.9741E-07	0.26689438	0.117	0.274	0.00457143	DCs
BRIX1	2.9874E-07	0.13880932	0.151	0.327	0.00459193	DCs
FBXO25	3.0009E-07	0.34115735	0.18	0.382	0.0046127	DCs
FBXW8	3.0031E-07	0.2070509	0.079	0.213	0.00461604	DCs
MED30	3.03E-07	0.26092757	0.151	0.337	0.0046574	DCs
RNASET21	3.0379E-07	0.14386991	0.397	0.751	0.00466956	DCs
WBP2NL	3.0604E-07	3.72645383	0.016	0.002	0.00470414	DCs
SSBP1	3.069E-07	0.24628749	0.132	0.301	0.00471736	DCs
STK11IP	3.0816E-07	0.27100103	0.189	0.399	0.00473671	DCs
RGL22	3.0857E-07	1.66345879	0.379	0.345	0.00474297	DCs
MYO1F1	3.1085E-07	0.54230301	0.707	0.899	0.00477806	DCs
XPO5	3.168E-07	0.16924806	0.215	0.442	0.00486956	DCs
ARRDC31	3.1802E-07	0.20299617	0.189	0.389	0.00488829	DCs
MLLT1	3.2203E-07	0.17723477	0.278	0.55	0.00494996	DCs
TIGAR1	3.2243E-07	0.1450021	0.3	0.591	0.00495604	DCs
ZSWIM7	3.2323E-07	0.16835216	0.256	0.508	0.00496838	DCs
USP341	3.2434E-07	1.0948471	0.514	0.591	0.00498548	DCs
ARHGAP17	3.2668E-07	0.1442201	0.334	0.646	0.0050214	DCs
DMTF11	3.2734E-07	1.44623132	0.385	0.35	0.0050315	DCs
ARFIP21	3.2961E-07	0.21700077	0.126	0.291	0.00506641	DCs
CIR11	3.3E-07	0.26424715	0.167	0.358	0.00507243	DCs
TATDN31	3.3043E-07	0.13567814	0.117	0.275	0.00507906	DCs
XYLT2	3.3099E-07	0.23896489	0.082	0.216	0.00508769	DCs
TOMM70	3.359E-07	0.29186021	0.268	0.536	0.00516309	DCs
PTRHD1	3.3683E-07	0.33546888	0.186	0.396	0.00517745	DCs
ABHD61	3.3782E-07	0.21308045	0.129	0.297	0.0051927	DCs
SC5D1	3.3843E-07	0.22249042	0.237	0.474	0.00520206	DCs
PNRC2	3.4003E-07	0.21200448	0.227	0.466	0.00522663	DCs
WDR24	3.4257E-07	0.24932879	0.101	0.248	0.00526564	DCs
GAS72	3.4363E-07	1.55642467	0.432	0.44	0.00528192	DCs
PCID2	3.4451E-07	0.33974057	0.177	0.377	0.00529543	DCs
SLC38A72	3.4989E-07	0.25283577	0.183	0.381	0.00537816	DCs
KIF1B2	3.5047E-07	0.31314335	0.215	0.436	0.00538707	DCs
NAE1	3.5323E-07	0.20294496	0.145	0.319	0.00542956	DCs
INTS3	3.543E-07	0.24113953	0.221	0.449	0.00544597	DCs
SUPT16H	3.5518E-07	0.25561134	0.271	0.545	0.00545948	DCs
MED20	3.5872E-07	0.23017539	0.101	0.244	0.00551391	DCs
RAD23B	3.5903E-07	0.14062187	0.303	0.601	0.00551861	DCs
PSMA1	3.6115E-07	0.1074085	0.202	0.415	0.00555117	DCs
RNASE41	3.6943E-07	0.37170781	0.117	0.271	0.00567849	DCs
EXPH5	3.6973E-07	3.57756089	0.019	0.003	0.00568315	DCs
NAA30	3.7213E-07	0.28842843	0.145	0.321	0.00571998	DCs

RUVBL2	3.759E-07	0.30981456	0.142	0.316	0.00577803	DCs
ZMYND151	3.8441E-07	0.36549933	0.287	0.567	0.00590881	DCs
NRAS2	3.8685E-07	0.34951386	0.227	0.465	0.00594635	DCs
COPS6	3.872E-07	0.20664298	0.306	0.595	0.00595171	DCs
ZNF135	3.8791E-07	4.55036894	0.016	0.002	0.0059625	DCs
LDLRAD31	3.9001E-07	0.60784203	0.117	0.273	0.00599478	DCs
PIP4K2B	3.9565E-07	0.14868843	0.3	0.587	0.00608155	DCs
FNIP12	4.0312E-07	0.22420688	0.208	0.424	0.00619635	DCs
NGRN	4.0329E-07	0.24689025	0.211	0.43	0.00619891	DCs
AP5Z1	4.0457E-07	0.22766588	0.161	0.345	0.00621859	DCs
ZMYND8	4.0527E-07	0.26372399	0.265	0.529	0.00622937	DCs
RBM251	4.1105E-07	0.90107972	0.568	0.686	0.00631828	DCs
RNF14	4.1307E-07	0.2067295	0.274	0.548	0.00634935	DCs
DDX23	4.2735E-07	0.13323963	0.322	0.623	0.00656885	DCs
NDUFAF8	4.2868E-07	0.11304752	0.243	0.479	0.00658918	DCs
EIF2D	4.3103E-07	0.30634521	0.196	0.403	0.00662539	DCs
RNF1151	4.3217E-07	1.31061076	0.438	0.456	0.00664288	DCs
TOP1MT1	4.3448E-07	0.35152775	0.233	0.474	0.00667837	DCs
ASB8	4.3731E-07	0.33043157	0.151	0.33	0.00672186	DCs
MAK16	4.3822E-07	0.37542497	0.158	0.339	0.0067359	DCs
ERICH1	4.4024E-07	1.28995328	0.533	0.628	0.00676688	DCs
ZNF592	4.4298E-07	0.15920209	0.363	0.702	0.00680907	DCs
SFXN5	4.4387E-07	0.1584176	0.063	0.184	0.0068227	DCs
GOLGA5	4.4398E-07	0.12398461	0.281	0.553	0.00682445	DCs
PTOV1	4.4448E-07	0.20719038	0.221	0.456	0.00683211	DCs
ING3	4.5019E-07	0.17843766	0.098	0.241	0.00691985	DCs
NUBP11	4.5331E-07	0.19479055	0.271	0.54	0.00696778	DCs
SLC25A6	4.5425E-07	0.86348258	0.603	0.753	0.00698222	DCs
RBM41	4.5442E-07	0.19262886	0.101	0.246	0.00698491	DCs
TRAFD1	4.5828E-07	0.60549643	0.271	0.553	0.00704426	DCs
ESF1	4.6309E-07	0.28528731	0.174	0.365	0.00711819	DCs
ACAD8	4.6621E-07	0.14795017	0.104	0.252	0.00716615	DCs
TBCK	4.6864E-07	0.31037977	0.145	0.314	0.00720352	DCs
KDELR1	4.6996E-07	0.23111722	0.284	0.569	0.00722381	DCs
KLHL12	4.7002E-07	0.24644075	0.246	0.497	0.0072247	DCs
ELL1	4.7712E-07	0.37928097	0.123	0.284	0.00733382	DCs
UFSP2	4.7885E-07	0.15192448	0.136	0.303	0.00736038	DCs
LTV1	4.7886E-07	0.33927296	0.186	0.389	0.00736052	DCs
MMADHC	4.7935E-07	0.2289208	0.243	0.485	0.00736812	DCs
DCAF1	4.8029E-07	0.40479422	0.161	0.347	0.00738252	DCs
LRFN1	4.8303E-07	2.63398963	0.085	0.035	0.00742472	DCs
SDS2	4.8498E-07	2.51349866	0.028	0.006	0.00745459	DCs
SMPD4	4.9366E-07	0.27164622	0.186	0.392	0.00758805	DCs
MLH1	4.9562E-07	0.18610951	0.243	0.491	0.00761816	DCs
ZNF106	4.9914E-07	0.21142374	0.312	0.626	0.00767235	DCs
ZMYM4	4.9991E-07	0.38229143	0.211	0.432	0.00768409	DCs
HSPG2	5.021E-07	4.10839935	0.016	0.002	0.00771783	DCs
TMEM87B	5.0434E-07	0.32942682	0.161	0.346	0.00775222	DCs
HEATR32	5.0632E-07	0.36777677	0.259	0.518	0.00778262	DCs
CUTC	5.0918E-07	0.20174841	0.155	0.337	0.00782668	DCs
PWP1	5.0991E-07	0.26107162	0.281	0.563	0.0078379	DCs
HLTF	5.1182E-07	0.32967577	0.129	0.293	0.00786717	DCs

SLC25A16	5.1477E-07	0.11879085	0.167	0.354	0.00791253	DCs
ACP1	5.1853E-07	0.24666396	0.167	0.356	0.00797033	DCs
GPD1L	5.2461E-07	0.23226951	0.132	0.298	0.00806382	DCs
ZNF217	5.2981E-07	0.86303648	0.59	0.731	0.00814378	DCs
CHCHD3	5.3496E-07	0.25017115	0.164	0.347	0.0082228	DCs
HDAC2	5.4339E-07	0.28084024	0.174	0.37	0.00835241	DCs
PAFAH1B11	5.4826E-07	0.54154299	0.662	0.833	0.00842731	DCs
COP1	5.514E-07	0.12926847	0.278	0.549	0.00847554	DCs
OAZ21	5.6106E-07	0.28695961	0.271	0.533	0.00862405	DCs
PPP4R1	5.7343E-07	0.31387839	0.218	0.444	0.0088142	DCs
PICK11	5.7501E-07	0.40843414	0.183	0.384	0.00883848	DCs
B3GNT51	5.7675E-07	0.11635638	0.35	0.658	0.00886525	DCs
ARMC1	5.7699E-07	0.30252572	0.114	0.266	0.00886896	DCs
VKORC1L1	5.7956E-07	0.15355672	0.091	0.228	0.00890848	DCs
THOC61	5.8206E-07	0.24365043	0.227	0.465	0.00894681	DCs
BTG12	5.8318E-07	0.95474013	0.722	0.895	0.00896412	DCs
NAA251	5.8661E-07	2.70667219	0.211	0.141	0.00901683	DCs
MTREX	5.9247E-07	0.33459406	0.174	0.369	0.00910692	DCs
NOP56	5.9837E-07	0.2232017	0.268	0.533	0.00919754	DCs
SLC33A11	6.0654E-07	0.18652455	0.199	0.413	0.00932318	DCs
SMCR8	6.0841E-07	0.24374065	0.274	0.54	0.00935184	DCs
SHLD21	6.1189E-07	0.18514028	0.268	0.528	0.00940531	DCs
ANAPC16	6.1484E-07	0.1488057	0.366	0.693	0.00945072	DCs
C11orf68	6.1804E-07	0.25210922	0.293	0.581	0.00949982	DCs
TTLL31	6.2229E-07	1.58697493	0.363	0.326	0.00956515	DCs
ENTPD7	6.2591E-07	0.38336841	0.082	0.211	0.00962082	DCs
MFSD14A	6.2837E-07	0.25194887	0.221	0.452	0.00965872	DCs
TMEM159	6.2867E-07	0.30986076	0.145	0.312	0.0096633	DCs
THAP7	6.3067E-07	0.11708445	0.091	0.229	0.00969403	DCs
SLC44A12	6.3427E-07	2.00435708	0.328	0.281	0.0097493	DCs
DOCK42	6.4323E-07	1.93308861	0.366	0.336	0.00988702	DCs
LRRFIP2	6.4458E-07	0.35080036	0.227	0.46	0.00990786	DCs
LRBA	6.5264E-07	1.64748414	0.312	0.253	0.01003173	DCs
NAGA2	6.5478E-07	0.70052475	0.669	0.842	0.01006457	DCs
SUGT11	6.59E-07	0.26756449	0.246	0.494	0.01012942	DCs
KLHL21	6.6588E-07	0.2673752	0.192	0.396	0.01023521	DCs
SCAF1	6.7578E-07	0.16514101	0.281	0.548	0.01038738	DCs
STAM2	6.7774E-07	0.26194597	0.268	0.523	0.01041754	DCs
DPYSL32	6.8026E-07	4.06728204	0.025	0.005	0.01045627	DCs
TBRG41	6.8471E-07	0.30038369	0.233	0.47	0.01052463	DCs
SCYL1	6.8849E-07	0.17400656	0.338	0.639	0.01058282	DCs
NT5DC21	6.9151E-07	3.21069039	0.022	0.004	0.01062916	DCs
DOK4	6.9387E-07	3.43806646	0.019	0.003	0.01066551	DCs
CCDC136	6.9421E-07	1.40561029	0.013	0.002	0.01067065	DCs
MYO15B2	6.9591E-07	1.72870177	0.297	0.234	0.01069691	DCs
STRN3	6.9726E-07	0.34812009	0.183	0.38	0.01071764	DCs
TOR2A1	7.0416E-07	0.41917109	0.126	0.288	0.01082363	DCs
TIMM8A	7.0616E-07	0.1035126	0.047	0.155	0.01085439	DCs
RBBP7	7.1151E-07	0.46716346	0.148	0.326	0.0109366	DCs
ZRANB1	7.1158E-07	0.42412847	0.205	0.422	0.01093772	DCs
DESI2	7.1703E-07	0.30955892	0.126	0.282	0.01102151	DCs
SLBP	7.214E-07	0.1294033	0.331	0.634	0.01108868	DCs

PTPRC	7.218E-07	0.61445419	0.675	0.851	0.01109473	DCs
STRIP21	7.2321E-07	3.84989835	0.028	0.006	0.01111646	DCs
CLCF1	7.2899E-07	0.111404	0.06	0.175	0.01120534	DCs
METTL2A	7.3126E-07	0.33969483	0.098	0.237	0.01124017	DCs
FTO	7.3793E-07	0.29005391	0.155	0.333	0.01134269	DCs
ANKRD28	7.4604E-07	0.39801562	0.183	0.38	0.01146738	DCs
KIF16B1	7.5112E-07	0.30781618	0.36	0.686	0.01154549	DCs
YRDC	7.5391E-07	0.17159729	0.101	0.243	0.01158837	DCs
DCAF11	7.552E-07	0.13676361	0.315	0.614	0.01160824	DCs
CGRRF1	7.6662E-07	0.16413456	0.069	0.19	0.01178378	DCs
TLR9	7.6986E-07	3.32848487	0.019	0.003	0.01183356	DCs
FMN12	7.7946E-07	2.353372	0.101	0.046	0.01198105	DCs
CYP2U11	7.809E-07	0.33194472	0.088	0.223	0.01200315	DCs
ATP5MPL	7.8517E-07	0.17940365	0.091	0.228	0.01206883	DCs
CIAO2B	7.8775E-07	0.15648402	0.256	0.5	0.01210845	DCs
YAE1	7.8878E-07	0.16357349	0.057	0.169	0.0121244	DCs
PHF5A	7.8963E-07	0.18582675	0.249	0.496	0.01213737	DCs
TAOK2	7.8984E-07	0.281147	0.233	0.469	0.01214068	DCs
AP1G1	8.107E-07	0.18698908	0.265	0.521	0.01246127	DCs
SLC25A26	8.1687E-07	0.23579853	0.158	0.339	0.01255617	DCs
AGO1	8.2127E-07	0.2958252	0.243	0.488	0.01262369	DCs
GTF3C2	8.2266E-07	0.27339619	0.233	0.461	0.01264505	DCs
ADGRL1	8.2777E-07	3.22785235	0.057	0.019	0.01272366	DCs
TWISTNB	8.598E-07	0.40561789	0.098	0.236	0.01321601	DCs
DDX18	8.644E-07	0.17727031	0.287	0.564	0.01328674	DCs
UMPS	8.7169E-07	0.43163473	0.095	0.231	0.01339879	DCs
BRD8	8.7866E-07	0.2574276	0.11	0.258	0.01350589	DCs
BZW1	8.8441E-07	0.28837968	0.211	0.432	0.01359431	DCs
GSDMB1	8.8665E-07	2.14929567	0.06	0.021	0.01362872	DCs
GOLGB1	8.8716E-07	0.31853394	0.265	0.532	0.01363647	DCs
OSER1	8.9309E-07	0.30441201	0.24	0.479	0.01372764	DCs
MAPK13	9.0481E-07	0.59503448	0.744	0.907	0.0139078	DCs
MXD12	9.1278E-07	0.30383793	0.262	0.496	0.01403038	DCs
SLC50A11	9.2354E-07	0.19968267	0.284	0.549	0.01419566	DCs
AFG3L2	9.2788E-07	0.24228818	0.246	0.49	0.01426237	DCs
DEPDC72	9.4854E-07	3.26378557	0.035	0.009	0.01457995	DCs
CCSAP	9.5027E-07	0.34881439	0.215	0.431	0.01460663	DCs
KLF7	9.5456E-07	0.47474649	0.224	0.453	0.01467258	DCs
CD582	9.5938E-07	1.16175464	0.539	0.656	0.01474668	DCs
RRP36	9.6169E-07	0.22743486	0.309	0.605	0.01478216	DCs
WDR43	9.7928E-07	0.23174791	0.155	0.329	0.01505247	DCs
PUM21	9.8432E-07	0.91393966	0.571	0.69	0.01513001	DCs
UBLCP1	9.9197E-07	0.15771978	0.35	0.666	0.01524752	DCs
MATK2	9.9831E-07	0.93099873	0.047	0.014	0.01534502	DCs
NMD31	1.003E-06	0.3271703	0.237	0.473	0.01541741	DCs
FAM199X	1.0212E-06	0.38699347	0.233	0.468	0.01569622	DCs
TBK1	1.0433E-06	0.26482701	0.233	0.467	0.01603679	DCs
TMEM185A	1.0525E-06	0.37908683	0.227	0.459	0.01617801	DCs
LRRC42	1.0617E-06	0.35834367	0.098	0.236	0.01631929	DCs
MAP2K3	1.0618E-06	0.25243751	0.315	0.612	0.0163215	DCs
AMD1	1.0731E-06	0.38785216	0.186	0.378	0.01649393	DCs
MAST2	1.0845E-06	0.45906238	0.174	0.364	0.01666998	DCs

PHKA2	1.0861E-06	0.15909903	0.129	0.284	0.0166937	DCs
SRRT	1.1091E-06	0.34372713	0.202	0.409	0.01704795	DCs
CXorf212	1.1212E-06	1.83990485	0.382	0.366	0.01723396	DCs
ACTR32	1.123E-06	0.76494097	0.596	0.749	0.01726185	DCs
NIBAN22	1.1287E-06	1.33923253	0.438	0.438	0.01734854	DCs
RFNG	1.1654E-06	0.33673959	0.189	0.385	0.01791412	DCs
CSNK1G1	1.1673E-06	0.31051617	0.158	0.334	0.01794221	DCs
SMYD5	1.1674E-06	0.37740405	0.098	0.235	0.01794478	DCs
PLEC2	1.1789E-06	0.4957669	0.744	0.877	0.01812135	DCs
TMEM135	1.1838E-06	0.20969697	0.073	0.194	0.01819582	DCs
AXIN1	1.2202E-06	0.278313	0.155	0.324	0.0187554	DCs
ANKRD421	1.2298E-06	0.3965193	0.107	0.251	0.01890293	DCs
ARMCX21	1.2485E-06	0.1192896	0.073	0.194	0.01919016	DCs
TSR1	1.2519E-06	0.62064373	0.161	0.343	0.01924236	DCs
GATM2	1.2632E-06	2.66126841	0.114	0.056	0.0194174	DCs
NUP88	1.2663E-06	0.18232111	0.136	0.297	0.01946357	DCs
GTPBP6	1.267E-06	0.26259479	0.268	0.531	0.01947519	DCs
C12orf43	1.2671E-06	0.28347796	0.12	0.269	0.01947718	DCs
RANGRF	1.2782E-06	0.25850478	0.186	0.379	0.01964792	DCs
CAD	1.295E-06	0.41397463	0.12	0.271	0.0199059	DCs
TBCEL	1.2966E-06	0.13898508	0.088	0.217	0.01992929	DCs
ZNF1211	1.2973E-06	1.57221251	0.356	0.325	0.01994057	DCs
SLC49A41	1.3022E-06	0.20159933	0.3	0.576	0.02001683	DCs
FAM177A1	1.3026E-06	0.16083821	0.167	0.346	0.0200227	DCs
MARCH31	1.3128E-06	2.96641607	0.079	0.033	0.02017978	DCs
PHACTR2	1.3245E-06	0.30730578	0.246	0.485	0.0203587	DCs
SNIP1	1.3275E-06	0.31717554	0.139	0.301	0.02040525	DCs
MTFR1	1.3327E-06	0.37261679	0.117	0.265	0.02048489	DCs
RUNDC1	1.3409E-06	0.23566202	0.132	0.294	0.02061172	DCs
RBBP9	1.3412E-06	0.23786696	0.129	0.285	0.02061485	DCs
TIAM12	1.3419E-06	0.32540351	0.297	0.575	0.02062708	DCs
MTM1	1.3427E-06	0.25750608	0.174	0.359	0.02063823	DCs
MTMR12	1.3639E-06	0.27786082	0.246	0.483	0.02096522	DCs
PEX11B1	1.3666E-06	0.4005326	0.227	0.457	0.02100645	DCs
SUN1	1.3683E-06	0.46897096	0.142	0.308	0.02103251	DCs
FBXL81	1.3925E-06	2.36426761	0.11	0.054	0.02140485	DCs
HOMEZ1	1.416E-06	0.35879327	0.155	0.33	0.02176498	DCs
FOCAD	1.4161E-06	0.10607971	0.076	0.196	0.0217673	DCs
UBQLN1	1.4297E-06	0.28588293	0.246	0.486	0.02197547	DCs
IGBP1	1.44E-06	0.10028426	0.344	0.648	0.02213405	DCs
FKBP1B1	1.4479E-06	2.88176114	0.088	0.039	0.02225585	DCs
IKBKG	1.4531E-06	0.10720848	0.36	0.674	0.02233565	DCs
NOL8	1.4549E-06	0.29953904	0.164	0.343	0.02236286	DCs
FBXO21	1.4605E-06	0.24885537	0.117	0.264	0.02244869	DCs
SCRIB	1.4729E-06	0.37824476	0.23	0.458	0.02264021	DCs
CCNG2	1.4901E-06	1.90362361	0.432	0.448	0.02290464	DCs
PATL11	1.4999E-06	0.21788891	0.274	0.538	0.02305509	DCs
ACIN11	1.5151E-06	0.16977848	0.221	0.436	0.02328842	DCs
RS1	1.5191E-06	5.41510653	0.013	0.002	0.02334934	DCs
IQCE	1.5196E-06	0.56655526	0.174	0.36	0.02335799	DCs
STK3	1.529E-06	0.33756319	0.17	0.348	0.02350223	DCs
IL182	1.5339E-06	1.33964659	0.505	0.594	0.02357779	DCs

MROH72	1.5342E-06	3.83059521	0.028	0.007	0.02358256	DCs
ACBD6	1.56E-06	0.24396294	0.186	0.382	0.02397938	DCs
CMTM32	1.5791E-06	0.92243883	0.603	0.713	0.02427211	DCs
HNRNPUL11	1.5802E-06	0.65146284	0.653	0.834	0.024289	DCs
GGA3	1.6076E-06	0.21415005	0.268	0.522	0.02471095	DCs
CHAMP1	1.6182E-06	0.37903123	0.148	0.315	0.02487306	DCs
PHLDB3	1.6239E-06	2.77496257	0.047	0.015	0.02496044	DCs
C2orf681	1.6252E-06	0.24842484	0.23	0.458	0.02498062	DCs
ELMSAN1	1.63E-06	0.83397554	0.603	0.756	0.0250543	DCs
UPF1	1.6376E-06	0.22468078	0.281	0.544	0.02517146	DCs
GRWD1	1.6814E-06	0.31136483	0.17	0.355	0.02584475	DCs
MED17	1.6984E-06	0.30626571	0.208	0.416	0.02610667	DCs
CTSH3	1.7247E-06	0.34025803	0.931	0.967	0.0265097	DCs
FRA10AC1	1.7589E-06	0.24893159	0.129	0.285	0.02703578	DCs
SREBF12	1.7596E-06	0.31253477	0.3	0.58	0.02704733	DCs
ZNF664	1.7683E-06	0.47657256	0.177	0.367	0.02718088	DCs
GTF3A	1.7723E-06	0.1923824	0.246	0.484	0.02724268	DCs
EHF	1.7778E-06	3.17890789	0.054	0.018	0.02732583	DCs
PIGU	1.7837E-06	0.35110013	0.088	0.216	0.02741755	DCs
IFNGR22	1.7971E-06	0.47297767	0.732	0.889	0.02762314	DCs
MRTO4	1.8027E-06	0.42139077	0.11	0.252	0.02770904	DCs
E2F6	1.8115E-06	0.25801162	0.088	0.216	0.02784445	DCs
SBDS	1.8213E-06	0.12266413	0.088	0.215	0.02799592	DCs
IFI44L1	1.8229E-06	0.30787887	0.246	0.462	0.02801957	DCs
CYHR1	1.8303E-06	0.30872447	0.136	0.293	0.02813395	DCs
AC091057.61	1.8327E-06	2.39858101	0.114	0.056	0.02817016	DCs
PDHB	1.8434E-06	0.15104021	0.309	0.596	0.02833555	DCs
ARHGAP151	1.8509E-06	1.00026927	0.467	0.496	0.02845081	DCs
CNFN	1.8641E-06	3.08785522	0.022	0.004	0.02865238	DCs
BUB3	1.8739E-06	0.28861057	0.196	0.397	0.02880353	DCs
AGAP32	1.8851E-06	0.36709128	0.249	0.5	0.02897573	DCs
SMAP1	1.886E-06	0.3293639	0.249	0.488	0.02898964	DCs
PLPP11	1.899E-06	0.42025417	0.186	0.382	0.02918933	DCs
ACSS1	1.9022E-06	0.19242113	0.129	0.282	0.0292391	DCs
LACTB3	1.9034E-06	0.11095053	0.356	0.67	0.02925714	DCs
UGGT2	1.9076E-06	0.17666187	0.114	0.256	0.02932154	DCs
TMED21	1.921E-06	0.40822803	0.262	0.511	0.02952701	DCs
RNF2171	1.9409E-06	0.49758076	0.155	0.325	0.02983378	DCs
ZNF281	1.9507E-06	0.22731028	0.123	0.27	0.02998406	DCs
RGL12	1.9615E-06	2.69926437	0.05	0.017	0.03015021	DCs
TRIM69	1.9641E-06	4.0884434	0.013	0.002	0.03019038	DCs
GNPAT	1.9646E-06	0.29308322	0.23	0.457	0.0301982	DCs
C11orf241	1.9805E-06	0.51843201	0.155	0.329	0.0304424	DCs
USP24	1.981E-06	0.28544268	0.249	0.486	0.03045071	DCs
ZFYVE27	1.9919E-06	0.36373826	0.161	0.337	0.03061766	DCs
DHX581	2.0175E-06	0.20815598	0.252	0.483	0.03101095	DCs
MUC11	2.0289E-06	1.92491861	0.032	0.008	0.03118581	DCs
RXYLT1	2.0531E-06	0.43469073	0.139	0.301	0.03155757	DCs
PPP1R15B2	2.0967E-06	0.96442762	0.644	0.826	0.03222777	DCs
ANAPC7	2.1189E-06	0.25635902	0.252	0.497	0.03257005	DCs
TGFB1	2.1669E-06	0.14472029	0.363	0.689	0.03330701	DCs
YIPF11	2.1746E-06	0.50847003	0.167	0.348	0.03342511	DCs

SERPINH12	2.1831E-06	0.14639685	0.129	0.281	0.03355699	DCs
ATG12	2.1892E-06	0.48089607	0.151	0.316	0.03364982	DCs
SDE2	2.2021E-06	0.30177597	0.196	0.396	0.03384813	DCs
BRCC3	2.2208E-06	0.17292504	0.088	0.217	0.03413529	DCs
ORC3	2.2233E-06	0.11329019	0.085	0.208	0.03417415	DCs
CCDC501	2.2422E-06	0.23758173	0.363	0.669	0.03446495	DCs
DYNLL1	2.2429E-06	0.34338323	0.091	0.222	0.03447626	DCs
VSIG2	2.2545E-06	3.60707247	0.016	0.002	0.0346541	DCs
ABCE1	2.2743E-06	0.4264287	0.183	0.373	0.03495815	DCs
RPAP1	2.2744E-06	0.36036355	0.11	0.249	0.03496034	DCs
IL16	2.2787E-06	1.29875721	0.508	0.606	0.03502585	DCs
BCL7C	2.2874E-06	0.2677146	0.259	0.506	0.03516039	DCs
RNF4	2.2987E-06	0.86755121	0.555	0.691	0.03533323	DCs
ZNF654	2.3111E-06	0.28486476	0.142	0.304	0.03552356	DCs
ERGIC11	2.3142E-06	0.38266537	0.259	0.508	0.03557172	DCs
MLLT61	2.3912E-06	1.15389169	0.435	0.443	0.03675473	DCs
ST3GAL62	2.3981E-06	0.48726831	0.274	0.538	0.03686111	DCs
R3HCC1	2.3998E-06	0.12561605	0.104	0.242	0.03688734	DCs
ZSWIM62	2.4049E-06	0.32122408	0.256	0.499	0.03696544	DCs
TRIM56	2.4316E-06	0.17111295	0.136	0.29	0.03737671	DCs
CARD111	2.4502E-06	1.43818304	0.085	0.037	0.03766246	DCs
GBF1	2.4584E-06	0.36099676	0.167	0.343	0.03778745	DCs
GPN2	2.4633E-06	0.33183834	0.12	0.265	0.03786389	DCs
EIF2B5	2.4939E-06	0.57967138	0.192	0.388	0.0383337	DCs
KIFAP3	2.499E-06	0.35966807	0.091	0.22	0.03841243	DCs
IQCG	2.5066E-06	0.39320798	0.114	0.256	0.03852883	DCs
GPATCH3	2.556E-06	0.19105986	0.126	0.274	0.03928826	DCs
HELZ21	2.5581E-06	0.21924038	0.142	0.3	0.03932057	DCs
ZNF12	2.5902E-06	0.24280375	0.101	0.234	0.03981465	DCs
SLC35B3	2.6065E-06	0.41567127	0.12	0.265	0.0400646	DCs
IRF53	2.6074E-06	0.32760429	0.243	0.485	0.04007798	DCs
TMCC21	2.6174E-06	4.08157099	0.022	0.004	0.04023267	DCs
RHNO1	2.6236E-06	0.46757107	0.151	0.321	0.04032698	DCs
EIF2S1	2.633E-06	0.153828	0.356	0.676	0.04047163	DCs
ZNF322	2.6443E-06	0.37891766	0.095	0.225	0.04064543	DCs
TRMO	2.6579E-06	0.30397671	0.252	0.492	0.04085444	DCs
MBD4	2.6626E-06	0.30933971	0.208	0.417	0.04092645	DCs
SDF2	2.7032E-06	0.34697129	0.174	0.357	0.04155052	DCs
COPS5	2.7436E-06	0.25520483	0.334	0.641	0.04217133	DCs
LYN3	2.7671E-06	0.68522916	0.64	0.826	0.04253265	DCs
ELF4	2.7705E-06	0.1455021	0.287	0.548	0.04258466	DCs
NDRG12	2.7935E-06	1.47807111	0.391	0.376	0.0429388	DCs
CRTC3	2.7978E-06	0.43621507	0.186	0.374	0.04300531	DCs
ALG5	2.8311E-06	0.24017017	0.199	0.395	0.04351661	DCs
SF3A1	2.8707E-06	0.17193084	0.293	0.573	0.04412604	DCs
HAUS6	2.922E-06	0.13742132	0.085	0.207	0.04491343	DCs
USP38	2.9341E-06	0.49012927	0.183	0.375	0.04509958	DCs
BCAT2	2.951E-06	0.48766931	0.151	0.316	0.04536025	DCs
FAM126A	2.956E-06	0.15844873	0.268	0.512	0.04543602	DCs
LARS3	2.9686E-06	1.04464069	0.562	0.708	0.04563038	DCs
IKZF1	2.9717E-06	0.47594124	0.735	0.901	0.0456778	DCs
SAMD4B	2.9727E-06	0.36156275	0.24	0.469	0.04569301	DCs

REXO4	3.0234E-06	0.475049	0.167	0.343	0.04647295	DCs
CCDC1121	3.0536E-06	1.96416519	0.243	0.181	0.04693684	DCs
FASTKD5	3.0932E-06	0.29720835	0.123	0.271	0.04754578	DCs
MEF2D1	3.096E-06	1.63561374	0.394	0.39	0.04758856	DCs
BOD1	3.1216E-06	0.39299748	0.123	0.272	0.04798145	DCs
SFT2D21	3.1362E-06	0.8939805	0.571	0.716	0.04820644	DCs
XKR8	3.1424E-06	0.34388515	0.069	0.184	0.04830121	DCs
PIK3CA	3.1863E-06	0.37809239	0.189	0.377	0.04897705	DCs
TNFRSF141	3.2174E-06	0.49594304	0.274	0.535	0.04945468	DCs
GLE1	3.2323E-06	0.29250526	0.243	0.475	0.04968398	DCs
NFIC1	3.2361E-06	0.20036663	0.338	0.637	0.04974146	DCs
RNF25	3.2388E-06	0.2636185	0.129	0.281	0.04978349	DCs
ZNF490	3.2415E-06	2.23172634	0.281	0.226	0.0498257	DCs
PRRC2C1	3.2885E-06	0.88848698	0.539	0.664	0.05054826	DCs
FBXW21	3.3074E-06	0.22450637	0.328	0.627	0.05083869	DCs
DDX19B	3.3373E-06	0.45353208	0.145	0.306	0.05129803	DCs
MT-ND12	3.3582E-06	1.70120977	0.278	0.212	0.05161852	DCs
SRPK21	3.4106E-06	0.31327494	0.202	0.406	0.05242412	DCs
PEX16	3.4145E-06	0.3197904	0.136	0.289	0.05248378	DCs
TTC4	3.4526E-06	0.12394494	0.073	0.187	0.05306929	DCs
ERICH5	3.4652E-06	3.27160709	0.022	0.005	0.05326298	DCs
NUDT16L1	3.4688E-06	0.20842617	0.088	0.21	0.05331911	DCs
JMJD6	3.4851E-06	0.19037529	0.268	0.514	0.05356893	DCs
YTHDF1	3.4952E-06	0.18280707	0.076	0.191	0.05372408	DCs
TNKS1BP11	3.5471E-06	3.11150243	0.057	0.021	0.05452255	DCs
ATXN2L1	3.5634E-06	1.25708449	0.47	0.526	0.05477231	DCs
A2M1	3.5931E-06	2.87045217	0.284	0.231	0.05522951	DCs
GTF2E2	3.6279E-06	0.37010042	0.224	0.442	0.0557645	DCs
CYTH31	3.6431E-06	2.14194794	0.208	0.144	0.0559983	DCs
MAEA	3.6709E-06	0.32203851	0.303	0.586	0.05642611	DCs
RASGRF11	3.6944E-06	3.69397585	0.022	0.005	0.05678641	DCs
ZNF268	3.7165E-06	0.28753127	0.158	0.326	0.05712571	DCs
RABIF	3.7628E-06	0.4259657	0.158	0.325	0.05783852	DCs
CEPT1	3.7818E-06	0.17110546	0.353	0.661	0.05813037	DCs
PHF2	3.7955E-06	0.45239399	0.24	0.467	0.05833991	DCs
INTS1	3.8797E-06	0.26395648	0.274	0.533	0.05963538	DCs
SNRNP2001	3.891E-06	0.77949719	0.59	0.759	0.05980834	DCs
KIF5B1	3.9469E-06	0.46226162	0.722	0.881	0.06066768	DCs
KDM1B2	3.948E-06	0.39154216	0.3	0.569	0.06068443	DCs
PRPF4	4.0122E-06	0.21745154	0.218	0.427	0.06167206	DCs
UBR3	4.0202E-06	0.32282502	0.221	0.431	0.06179498	DCs
MAD1L11	4.0286E-06	0.16284784	0.199	0.393	0.06192328	DCs
RWDD4	4.0335E-06	0.19696784	0.107	0.241	0.06199928	DCs
FBXW11	4.0576E-06	0.19129627	0.155	0.316	0.06236947	DCs
IDH3A	4.0955E-06	0.67366576	0.199	0.398	0.0629526	DCs
ZNF25	4.1241E-06	0.45325889	0.123	0.269	0.06339212	DCs
METTL92	4.1304E-06	1.236361	0.451	0.483	0.06348807	DCs
TTLL1	4.1385E-06	0.10818676	0.06	0.165	0.06361303	DCs
KYAT3	4.1679E-06	0.13738692	0.098	0.225	0.06406493	DCs
SMG5	4.1708E-06	0.16400527	0.322	0.596	0.06410862	DCs
RNF139	4.1828E-06	0.33911778	0.199	0.392	0.06429447	DCs
ALYREF	4.2135E-06	0.43632187	0.215	0.424	0.0647655	DCs

FOXO4	4.2239E-06	0.12986538	0.11	0.244	0.06492502	DCs
MOV101	4.24E-06	0.33488898	0.312	0.585	0.06517275	DCs
TMEM242	4.2703E-06	0.44727361	0.107	0.242	0.06563824	DCs
RIF1	4.2782E-06	0.21944313	0.12	0.26	0.06575965	DCs
UBE2G1	4.2813E-06	0.3178789	0.297	0.562	0.06580822	DCs
RBM7	4.286E-06	0.51563387	0.202	0.399	0.06588068	DCs
KAT2B	4.2972E-06	1.38799742	0.404	0.404	0.06605301	DCs
REEP41	4.3184E-06	0.2721297	0.095	0.223	0.06637874	DCs
AFTPH1	4.3436E-06	0.96723683	0.521	0.634	0.06676485	DCs
ANKRD54	4.3582E-06	0.15417531	0.054	0.155	0.06698973	DCs
ARFGEF11	4.3722E-06	0.31989642	0.186	0.37	0.06720487	DCs
MED1	4.4054E-06	0.34204433	0.237	0.458	0.06771485	DCs
HPS51	4.4533E-06	1.03895785	0.609	0.793	0.0684524	DCs
POLR1B	4.4579E-06	0.19890236	0.091	0.216	0.06852241	DCs
UCHL5	4.4586E-06	0.23891584	0.139	0.29	0.06853287	DCs
COG5	4.4629E-06	0.37911065	0.24	0.472	0.06859919	DCs
KIAA1522	4.4861E-06	0.22003145	0.189	0.369	0.06895558	DCs
SRPK1	4.5094E-06	0.23713839	0.192	0.383	0.06931328	DCs
CEP63	4.5112E-06	0.43501156	0.227	0.438	0.06934148	DCs
DIS3	4.5213E-06	0.2882418	0.221	0.431	0.06949699	DCs
RIMKLB	4.5272E-06	0.19571199	0.082	0.2	0.06958715	DCs
FLACC11	4.5917E-06	3.24798874	0.044	0.014	0.07057925	DCs
CDK121	4.6584E-06	0.31502894	0.189	0.375	0.07160404	DCs
TPRA12	4.7053E-06	0.32972432	0.208	0.408	0.07232493	DCs
NSUN3	4.7118E-06	0.59872637	0.151	0.311	0.07242566	DCs
FOXJ3	4.7296E-06	0.33239782	0.17	0.347	0.07269826	DCs
GOPC	4.7391E-06	0.33084028	0.243	0.471	0.07284418	DCs
HNRNPH31	4.7659E-06	0.77624077	0.606	0.773	0.07325722	DCs
RABL3	4.7921E-06	0.42432556	0.114	0.251	0.07365937	DCs
GCFC2	4.8695E-06	0.28598531	0.073	0.183	0.07484951	DCs
FNTB	4.8864E-06	0.30149139	0.06	0.165	0.07510869	DCs
AHI1	4.8997E-06	0.23879671	0.095	0.22	0.07531336	DCs
IFT52	5.0133E-06	0.19671043	0.085	0.206	0.07705949	DCs
GNG51	5.014E-06	0.40714102	0.199	0.395	0.07707032	DCs
CDK7	5.0317E-06	0.13385266	0.047	0.144	0.07734216	DCs
CLASRP1	5.0574E-06	1.49325551	0.391	0.385	0.07773685	DCs
ZNF777	5.0697E-06	0.10328197	0.076	0.186	0.07792571	DCs
SLC20A11	5.1265E-06	1.59113174	0.423	0.434	0.0787997	DCs
METTL6	5.1758E-06	0.26779621	0.073	0.183	0.07955732	DCs
TAF8	5.2162E-06	0.21655654	0.224	0.427	0.08017817	DCs
SETD4	5.2541E-06	0.31194337	0.079	0.194	0.08076033	DCs
EXOSC5	5.2798E-06	0.21957344	0.085	0.203	0.08115574	DCs
TTC21B	5.2856E-06	0.14868141	0.079	0.193	0.08124421	DCs
FCSK1	5.2998E-06	0.35329275	0.066	0.174	0.08146383	DCs
BUD13	5.3304E-06	0.53646303	0.161	0.334	0.08193348	DCs
E2F5	5.3621E-06	3.16723324	0.044	0.014	0.08242061	DCs
BBC31	5.3654E-06	1.8810026	0.274	0.219	0.08247139	DCs
NUDT19	5.3704E-06	0.42424808	0.177	0.354	0.08254793	DCs
KLHL15	5.4707E-06	0.49845166	0.114	0.25	0.08409084	DCs
NAT10	5.5392E-06	0.34581166	0.155	0.32	0.08514373	DCs
LY6G5B1	5.5562E-06	1.92714236	0.164	0.1	0.08540464	DCs
EVA1B1	5.5793E-06	0.41621111	0.079	0.194	0.08575992	DCs

TNPO3	5.6054E-06	0.32591162	0.249	0.481	0.08616107	DCs
TNFSF151	5.626E-06	3.86231783	0.032	0.008	0.08647792	DCs
ATP8A1	5.7248E-06	2.05241985	0.129	0.071	0.0879955	DCs
SYNGR3	5.7634E-06	2.34981329	0.013	0.002	0.08858983	DCs
CWC22	5.7863E-06	0.42521648	0.126	0.273	0.08894192	DCs
DENND10	5.7963E-06	0.47604159	0.18	0.361	0.08909449	DCs
STRN4	5.8685E-06	0.17859242	0.293	0.555	0.09020493	DCs
PARP15	5.9408E-06	0.27165735	0.129	0.27	0.09131624	DCs
ZBTB33	5.9471E-06	0.28596072	0.293	0.563	0.09141275	DCs
DCAF6	5.9694E-06	0.79269427	0.221	0.435	0.091755	DCs
ZBTB21	5.9838E-06	0.38486686	0.148	0.305	0.09197775	DCs
ZNF317	5.9839E-06	0.48722185	0.11	0.242	0.09197856	DCs
B4GALT3	5.994E-06	0.31960947	0.136	0.286	0.09213338	DCs
FGD42	6.0329E-06	0.30713169	0.334	0.629	0.09273238	DCs
MAP3K32	6.1389E-06	1.64112199	0.325	0.285	0.09436176	DCs
ATG9A2	6.2127E-06	0.26375877	0.104	0.233	0.09549609	DCs
LANCL1	6.2219E-06	0.27463324	0.211	0.411	0.09563642	DCs
NIPA2	6.2309E-06	0.21754796	0.3	0.56	0.09577571	DCs
FAM76A	6.2408E-06	0.29998986	0.145	0.301	0.09592704	DCs
ING4	6.3428E-06	0.43707577	0.129	0.274	0.09749572	DCs
SRBD1	6.433E-06	0.22842645	0.079	0.193	0.09888168	DCs
EIF4EBP31	6.5127E-06	0.2922243	0.132	0.281	0.10010726	DCs
FAM120B	6.5267E-06	0.42664726	0.18	0.359	0.10032197	DCs
CCNC	6.56E-06	0.33917771	0.205	0.403	0.10083399	DCs
MARF1	6.6253E-06	0.51388084	0.174	0.347	0.10183796	DCs
ZNF7911	6.658E-06	2.06052859	0.233	0.172	0.10233994	DCs
EXOSC6	6.7139E-06	0.386331	0.167	0.336	0.10319929	DCs
MAP3K15	6.7291E-06	1.29725097	0.492	0.572	0.10343363	DCs
TUT4	6.7362E-06	0.25934453	0.221	0.428	0.10354159	DCs
CHCHD7	6.737E-06	0.25543759	0.256	0.485	0.10355407	DCs
BICD11	6.7551E-06	2.45723102	0.088	0.041	0.10383312	DCs
WDR45B	6.7725E-06	0.25833456	0.246	0.47	0.10409951	DCs
TOB2	6.8506E-06	0.38218952	0.155	0.315	0.10530069	DCs
SUMO41	6.8854E-06	2.64075174	0.041	0.013	0.10583513	DCs
SLC2A31	6.903E-06	0.16360876	0.442	0.725	0.10610525	DCs
PTTG2	6.9166E-06	3.16309812	0.013	0.002	0.10631512	DCs
SYMPK1	6.9457E-06	1.00673182	0.527	0.634	0.10676281	DCs
COMMD5	6.9712E-06	0.1969269	0.35	0.661	0.10715454	DCs
SDHA	6.9798E-06	0.26209732	0.192	0.38	0.10728677	DCs
FAM162A1	6.991E-06	0.10875621	0.372	0.683	0.10745796	DCs
KIF9	7.0212E-06	0.12509256	0.032	0.116	0.10792231	DCs
NUDT14	7.1973E-06	0.39505595	0.237	0.451	0.11062902	DCs
MTAP	7.1998E-06	0.41008607	0.104	0.233	0.11066819	DCs
PDCD5	7.2332E-06	0.45302102	0.161	0.327	0.11118178	DCs
RBMX	7.2384E-06	0.45035316	0.199	0.394	0.11126157	DCs
TRIOBP	7.2457E-06	0.12299286	0.375	0.686	0.1113742	DCs
CHRAC1	7.2749E-06	0.4196983	0.233	0.453	0.1118232	DCs
MEGF62	7.3095E-06	2.31030793	0.088	0.041	0.11235444	DCs
TMX11	7.3316E-06	0.24600004	0.312	0.598	0.11269342	DCs
MED9	7.3345E-06	0.18646686	0.069	0.178	0.11273902	DCs
METTL11	7.3978E-06	0.36063185	0.073	0.18	0.11371092	DCs
RNF113A	7.4271E-06	0.4306302	0.177	0.351	0.1141622	DCs

STX71	7.4479E-06	0.6387173	0.656	0.838	0.1144822	DCs
GPR137	7.452E-06	0.36265217	0.117	0.253	0.11454403	DCs
ILF2	7.6176E-06	0.35633326	0.249	0.483	0.11708986	DCs
SH2D3C	7.6392E-06	0.26275553	0.218	0.418	0.11742239	DCs
GALNT13	7.7631E-06	0.28573104	0.322	0.608	0.11932686	DCs
IL7R1	7.8006E-06	1.03085166	0.366	0.656	0.11990244	DCs
ZNF2231	7.83E-06	0.42656538	0.066	0.172	0.12035533	DCs
NOP161	7.8426E-06	0.33974652	0.256	0.484	0.12054818	DCs
KDSR	7.8525E-06	0.11004291	0.353	0.658	0.12070078	DCs
THAP1	7.9537E-06	0.2928391	0.098	0.222	0.12225567	DCs
WWP1	8.0339E-06	0.44002111	0.123	0.262	0.12348855	DCs
RPE	8.1376E-06	0.52695128	0.145	0.302	0.12508325	DCs
ARMCX3	8.3045E-06	0.56177872	0.126	0.271	0.12764827	DCs
GLRX31	8.3174E-06	0.17082619	0.325	0.612	0.1278473	DCs
EXOSC8	8.3611E-06	0.27679729	0.158	0.316	0.12851908	DCs
PRMT7	8.3659E-06	0.15694723	0.098	0.222	0.12859278	DCs
TOMM40L	8.3678E-06	0.15707559	0.082	0.196	0.12862145	DCs
RCCD1	8.3996E-06	0.13925871	0.076	0.187	0.12911088	DCs
ADK1	8.4075E-06	0.49353513	0.161	0.329	0.1292315	DCs
STK19	8.46E-06	0.26470159	0.073	0.181	0.13003863	DCs
LONP2	8.4862E-06	0.26118377	0.312	0.59	0.13044148	DCs
ASF1A	8.509E-06	0.36476169	0.123	0.264	0.13079182	DCs
C8orf76	8.5328E-06	0.27625899	0.158	0.32	0.13115822	DCs
BCL2L2	8.5492E-06	0.50349064	0.12	0.256	0.13140929	DCs
BMP2K1	8.5742E-06	1.37650589	0.442	0.491	0.13179424	DCs
SMPD3	8.6027E-06	3.57707679	0.041	0.013	0.1322324	DCs
XRRA1	8.6103E-06	0.30419701	0.044	0.135	0.13234958	DCs
SELENOI	8.6735E-06	0.34330538	0.098	0.222	0.13332016	DCs
ASB6	8.7458E-06	0.12588005	0.085	0.2	0.1344311	DCs
CRYZ	8.7802E-06	0.27276622	0.123	0.262	0.13496016	DCs
NEDD9	8.8174E-06	0.25255755	0.098	0.219	0.13553218	DCs
PLEKHF22	8.8362E-06	1.43303396	0.432	0.471	0.13582175	DCs
C1orf1981	8.8638E-06	0.42943789	0.076	0.187	0.13624498	DCs
PDPK1	8.9095E-06	0.30008884	0.211	0.412	0.13694859	DCs
HGSNAT2	8.9999E-06	1.4156624	0.442	0.481	0.13833716	DCs
STEAP41	9.012E-06	2.40222389	0.016	0.003	0.13852337	DCs
BCL7B	9.0474E-06	0.14234962	0.363	0.67	0.13906826	DCs
TMEM87A	9.0817E-06	0.28681539	0.287	0.54	0.1395944	DCs
PI4KA2	9.1271E-06	0.20251923	0.338	0.633	0.1402922	DCs
AGPAT52	9.16E-06	0.39500589	0.082	0.196	0.1407976	DCs
CEP831	9.2071E-06	2.08619885	0.237	0.175	0.14152254	DCs
TAF9	9.3272E-06	0.30078223	0.177	0.346	0.14336834	DCs
SYTL1	9.5415E-06	1.60191152	0.259	0.195	0.14666228	DCs
BRD3	9.547E-06	0.36984686	0.278	0.533	0.14674628	DCs
ETAA1	9.5947E-06	0.43421894	0.085	0.2	0.14747995	DCs
TLE1	9.6753E-06	0.12227253	0.069	0.173	0.14871931	DCs
TRMT61	9.7101E-06	0.42298104	0.192	0.381	0.14925367	DCs
HDAC10	9.7286E-06	0.31440761	0.23	0.439	0.14953773	DCs
PRCC	9.7619E-06	0.10731041	0.287	0.537	0.15005049	DCs
ANO8	9.788E-06	0.40258221	0.117	0.251	0.150451	DCs
SCUBE2	9.798E-06	3.90575384	0.035	0.01	0.15060557	DCs
PPP1R3B1	9.836E-06	0.3190355	0.079	0.191	0.15118973	DCs

MAP1S2	9.8432E-06	0.17524066	0.36	0.676	0.15129915	DCs
PGGT1B	9.8761E-06	0.33526212	0.278	0.533	0.15180492	DCs
GPT1	9.8788E-06	3.24978899	0.044	0.015	0.15184679	DCs
ALDH1B1	9.9158E-06	0.32648335	0.05	0.145	0.15241542	DCs
SNX12	9.9439E-06	0.20671742	0.312	0.59	0.1528483	DCs
PHYKPL2	9.9668E-06	1.70628429	0.388	0.396	0.15319954	DCs
MTFR1L	9.9747E-06	0.26475031	0.211	0.409	0.15332073	DCs
HPS4	9.9838E-06	0.44785515	0.199	0.389	0.15346174	DCs
C1orf35	1.0097E-05	0.49457279	0.114	0.247	0.15520275	DCs
ATXN1L	1.0139E-05	0.51375377	0.174	0.346	0.15584645	DCs
TRMT1L	1.0156E-05	0.27419124	0.262	0.495	0.15611031	DCs
NRSN21	1.0158E-05	3.38691594	0.028	0.007	0.15614398	DCs
GPHN	1.0299E-05	0.18952266	0.063	0.163	0.15830257	DCs
PPP2R3B	1.0371E-05	0.22038569	0.079	0.189	0.15941563	DCs
INTS12	1.0397E-05	0.3504069	0.117	0.249	0.15981716	DCs
SLC26A61	1.0471E-05	0.10680846	0.069	0.175	0.16094895	DCs
CDC14A	1.0486E-05	1.32121911	0.142	0.083	0.16117778	DCs
WHAMM	1.0525E-05	0.45685349	0.107	0.236	0.16178262	DCs
TRIM341	1.0558E-05	0.51872329	0.211	0.408	0.16227938	DCs
NOM1	1.0626E-05	0.2993443	0.189	0.37	0.16333019	DCs
PLEKHJ1	1.0641E-05	0.50918399	0.155	0.313	0.16355858	DCs
SMARCAL1	1.0678E-05	0.246834	0.117	0.248	0.16412499	DCs
ABCA51	1.0695E-05	2.08062442	0.117	0.063	0.16438568	DCs
DUS3L	1.07E-05	0.27570956	0.161	0.323	0.1644698	DCs
BRPF1	1.0725E-05	0.34139198	0.151	0.309	0.16485364	DCs
PDE8B1	1.0809E-05	3.09800843	0.085	0.04	0.16614372	DCs
DMXL11	1.0854E-05	0.39791263	0.233	0.447	0.16683927	DCs
FTSJ1	1.0937E-05	0.26776354	0.11	0.241	0.16811123	DCs
PPARGC1B1	1.0939E-05	0.59154522	0.12	0.256	0.16814288	DCs
ZNF585A	1.1068E-05	0.22472848	0.069	0.173	0.17012758	DCs
DDX3Y1	1.1079E-05	0.2323791	0.129	0.269	0.1702967	DCs
TACC11	1.118E-05	0.63670479	0.65	0.834	0.17184178	DCs
CLN32	1.1189E-05	0.36173026	0.271	0.508	0.17199325	DCs
SDHAF3	1.1364E-05	0.17341809	0.063	0.164	0.17467091	DCs
ZBED11	1.1439E-05	0.29397485	0.287	0.537	0.17583653	DCs
SNRPB	1.146E-05	0.10590414	0.319	0.599	0.17614794	DCs
SVIP	1.1462E-05	2.54752726	0.054	0.02	0.1761824	DCs
EBAG9	1.1511E-05	0.38341222	0.202	0.393	0.17692862	DCs
NOLC1	1.1536E-05	0.58043714	0.104	0.231	0.17731419	DCs
GEMIN71	1.1659E-05	1.86888258	0.29	0.246	0.17920959	DCs
SSRP1	1.173E-05	0.3039313	0.287	0.546	0.18030008	DCs
ANKRD13C	1.1747E-05	0.29776289	0.073	0.178	0.18057044	DCs
VIRMA	1.1827E-05	0.50651771	0.155	0.311	0.18179334	DCs
RAD51C	1.184E-05	0.28910554	0.054	0.149	0.18198682	DCs
BLNK2	1.1942E-05	0.3034978	0.066	0.168	0.18356277	DCs
MAP4K42	1.1996E-05	1.47181047	0.413	0.431	0.18438427	DCs
FDPS	1.2042E-05	0.18871283	0.11	0.238	0.18509826	DCs
DIPK2A2	1.2105E-05	1.50127661	0.407	0.423	0.18606397	DCs
BCCIP1	1.2171E-05	0.21518273	0.303	0.571	0.18708491	DCs
VMP12	1.2184E-05	0.77457565	0.603	0.742	0.18728109	DCs
PIK3CD2	1.2192E-05	1.2571721	0.451	0.493	0.18740906	DCs
NDUFA6	1.2269E-05	0.38884412	0.211	0.408	0.18859173	DCs

BORCS8	1.2324E-05	0.39462504	0.142	0.291	0.18943329	DCs
CEBPZ	1.2412E-05	0.43118993	0.265	0.504	0.19077789	DCs
C22orf39	1.2428E-05	0.25225636	0.091	0.209	0.19102881	DCs
MRRF1	1.2535E-05	0.47235159	0.155	0.309	0.19266893	DCs
STYX	1.2673E-05	0.39556999	0.23	0.442	0.19480432	DCs
AVIL2	1.2688E-05	2.78267501	0.044	0.015	0.19502824	DCs
RDH16	1.2767E-05	3.44687404	0.016	0.003	0.19623394	DCs
METAP2	1.2993E-05	0.29734178	0.303	0.578	0.19971987	DCs
ABL22	1.3065E-05	1.75615926	0.328	0.297	0.20081898	DCs
CDIP1	1.3172E-05	0.3340773	0.136	0.28	0.20246446	DCs
DCAF17	1.3259E-05	0.26702398	0.091	0.206	0.20380857	DCs
SPAST	1.332E-05	0.30772319	0.142	0.288	0.20474526	DCs
OSTC1	1.3385E-05	0.51890438	0.205	0.4	0.20574483	DCs
MUS81	1.3398E-05	0.35437386	0.252	0.475	0.20594557	DCs
FASTKD2	1.3485E-05	0.44144089	0.211	0.413	0.20727794	DCs
THOC7	1.3575E-05	0.36804391	0.309	0.585	0.2086587	DCs
WIZ	1.3595E-05	0.51562146	0.215	0.416	0.20896579	DCs
TWF23	1.3616E-05	0.38595371	0.83	0.933	0.20929509	DCs
TTC9C1	1.3772E-05	0.44183292	0.18	0.352	0.21169498	DCs
THAP12	1.3923E-05	0.46985963	0.167	0.334	0.21401245	DCs
RCVRN1	1.3985E-05	4.18029887	0.013	0.002	0.21496942	DCs
KIF2A	1.3988E-05	2.39759375	0.338	0.318	0.21501374	DCs
ADD3	1.4009E-05	0.37113641	0.24	0.458	0.21533639	DCs
BUD31	1.4023E-05	0.33361288	0.271	0.514	0.21555502	DCs
HMGXB4	1.4077E-05	0.33226885	0.142	0.291	0.21637214	DCs
AKAP1	1.4092E-05	0.48966123	0.208	0.404	0.2166065	DCs
TMEM184C	1.4231E-05	0.2280963	0.136	0.28	0.21873867	DCs
LCAT1	1.4252E-05	2.46491015	0.073	0.032	0.21907297	DCs
SUFU	1.4276E-05	0.19122824	0.06	0.157	0.21943222	DCs
RTCA	1.4324E-05	0.42966482	0.233	0.444	0.22017554	DCs
NSFL1C	1.4445E-05	0.15476754	0.382	0.696	0.22204063	DCs
CHEK2	1.4541E-05	0.3173445	0.088	0.202	0.22350658	DCs
MAFK	1.462E-05	0.32433428	0.221	0.419	0.22472314	DCs
NOC4L2	1.4723E-05	0.51142718	0.151	0.307	0.22630608	DCs
AKR1E21	1.4781E-05	0.16214444	0.069	0.171	0.22719646	DCs
PGAP1	1.4934E-05	2.50277574	0.041	0.013	0.2295458	DCs
NAA60	1.4937E-05	0.12849747	0.334	0.611	0.22959519	DCs
NMRK1	1.4945E-05	0.61939217	0.183	0.359	0.22972151	DCs
GIGYF2	1.5044E-05	0.4462362	0.164	0.323	0.23124873	DCs
TENT21	1.5066E-05	0.99664614	0.511	0.62	0.23158564	DCs
ZNF708	1.5105E-05	0.31759128	0.088	0.203	0.23218245	DCs
GOLT1B2	1.5139E-05	0.21881589	0.328	0.614	0.23270315	DCs
NUDT22	1.5206E-05	0.18485233	0.322	0.593	0.23373675	DCs
NRROS2	1.5295E-05	1.3756864	0.429	0.464	0.23510632	DCs
MDM1	1.5304E-05	0.18691295	0.069	0.172	0.23523099	DCs
CSNK1E	1.5348E-05	0.42549918	0.259	0.495	0.23591292	DCs
POLR3A	1.5386E-05	0.31591485	0.12	0.252	0.23649332	DCs
C19orf47	1.5505E-05	0.44352459	0.085	0.197	0.23833121	DCs
CISD21	1.557E-05	0.15515513	0.29	0.543	0.23933385	DCs
SIRT11	1.5655E-05	1.90375778	0.271	0.222	0.24062747	DCs
SH3YL1	1.5682E-05	0.18856696	0.088	0.202	0.24104534	DCs
ABHD17B	1.5684E-05	0.27960854	0.101	0.223	0.24107167	DCs

RNF126	1.5765E-05	0.38247915	0.227	0.433	0.24232648	DCs
PAN2	1.5783E-05	0.29188261	0.164	0.322	0.2426053	DCs
DENND1A1	1.606E-05	0.34323169	0.271	0.514	0.24686255	DCs
ALG11	1.6062E-05	0.18395673	0.05	0.141	0.24688806	DCs
ARL6IP6	1.6066E-05	0.33962453	0.151	0.301	0.24695535	DCs
MCPH1	1.6129E-05	0.26588188	0.161	0.319	0.24792313	DCs
PXMP4	1.6266E-05	0.10295515	0.076	0.182	0.25002533	DCs
SDAD1	1.6504E-05	0.38746354	0.132	0.273	0.25368005	DCs
RXRB	1.6519E-05	0.40408106	0.271	0.512	0.25391319	DCs
ODF3B2	1.6556E-05	1.22572594	0.47	0.526	0.25448609	DCs
PCSK7	1.6557E-05	0.25354983	0.303	0.564	0.25450475	DCs
DNAJC24	1.6683E-05	0.11808678	0.079	0.186	0.25643346	DCs
COX10	1.6784E-05	0.25072017	0.098	0.217	0.25799317	DCs
SF3B3	1.6792E-05	0.27452229	0.287	0.54	0.25810399	DCs
DNAJB14	1.6907E-05	0.27567578	0.211	0.407	0.2598748	DCs
WDR181	1.6967E-05	0.57386653	0.12	0.254	0.26079805	DCs
TTC19	1.7172E-05	0.40059876	0.18	0.351	0.26394741	DCs
E2F41	1.7233E-05	0.10733792	0.394	0.722	0.26488546	DCs
WDR62	1.731E-05	1.57781227	0.35	0.33	0.26607431	DCs
PPP4R2	1.7496E-05	0.4856476	0.164	0.322	0.26892965	DCs
DUSP231	1.7542E-05	0.23138335	0.344	0.63	0.26963789	DCs
RNF2201	1.7619E-05	0.41784069	0.252	0.476	0.27082236	DCs
SERINC52	1.7746E-05	0.53917051	0.29	0.549	0.27277833	DCs
SLC7A6OS	1.7767E-05	0.61647549	0.104	0.225	0.2730914	DCs
TXNL1	1.7884E-05	0.12351348	0.35	0.654	0.27488976	DCs
MCM9	1.8068E-05	0.36245805	0.11	0.233	0.27773015	DCs
SETD7	1.8199E-05	0.33686165	0.262	0.498	0.27973036	DCs
MFN1	1.823E-05	0.33928196	0.142	0.283	0.28021548	DCs
RFC1	1.8372E-05	0.48078184	0.126	0.261	0.28239796	DCs
SSH2	1.8448E-05	0.34847967	0.202	0.388	0.28356592	DCs
STX21	1.86E-05	0.20290058	0.18	0.347	0.28590638	DCs
GORASP11	1.8668E-05	0.28834439	0.262	0.499	0.28694784	DCs
ZBTB34	1.8711E-05	0.38055059	0.101	0.222	0.28760366	DCs
PLEKHN11	1.882E-05	2.54826521	0.035	0.01	0.28927881	DCs
SSH11	1.8835E-05	0.42043074	0.233	0.443	0.28950564	DCs
TNIP11	1.899E-05	1.16913166	0.445	0.492	0.29190034	DCs
MCTP11	1.9088E-05	0.55161195	0.256	0.486	0.29340151	DCs
PSMC6	1.9171E-05	0.45132371	0.211	0.405	0.29467147	DCs
TBC1D13	1.9334E-05	0.57545379	0.155	0.305	0.29717671	DCs
LETM1	1.9467E-05	0.13965074	0.063	0.161	0.29922176	DCs
TLR71	1.9583E-05	0.60053731	0.249	0.471	0.30101369	DCs
MGAT4A2	1.9604E-05	0.21257058	0.426	0.755	0.30132585	DCs
CRY2	1.9757E-05	0.2888551	0.205	0.394	0.30368789	DCs
USF3	1.9817E-05	0.2702489	0.091	0.205	0.30460053	DCs
MOB2	2.0308E-05	0.34419356	0.215	0.408	0.31215883	DCs
PER31	2.0319E-05	0.60780601	0.126	0.258	0.31232735	DCs
DMAC2L	2.0373E-05	0.61488769	0.161	0.319	0.31315805	DCs
ITGB7	2.0541E-05	0.28906839	0.148	0.291	0.31572876	DCs
LONRF1	2.0582E-05	1.9010762	0.319	0.287	0.31636737	DCs
N4BP2L2	2.0613E-05	0.50731104	0.647	0.809	0.31683762	DCs
EXOSC10	2.0855E-05	0.35237809	0.278	0.528	0.32056553	DCs
MOB3A2	2.0965E-05	0.68735876	0.656	0.844	0.32225923	DCs

TBC1D91	2.1072E-05	0.94017602	0.552	0.708	0.32390327	DCs
ARSG	2.1145E-05	0.26449327	0.057	0.15	0.32501341	DCs
PIGC	2.1432E-05	0.38317509	0.129	0.265	0.32943267	DCs
TMEM256	2.1541E-05	0.68726242	0.139	0.285	0.33109988	DCs
DOP1A	2.1565E-05	0.43645918	0.117	0.246	0.33147044	DCs
QTRT2	2.2087E-05	0.51097676	0.196	0.373	0.33950002	DCs
DHX381	2.234E-05	0.42055069	0.249	0.468	0.3433815	DCs
UGCG1	2.242E-05	1.30771125	0.479	0.517	0.34462471	DCs
SGTA	2.2802E-05	0.41276742	0.274	0.513	0.35049656	DCs
SERTAD12	2.2813E-05	0.67288231	0.117	0.246	0.35065285	DCs
ORAI2	2.2825E-05	0.33259969	0.338	0.624	0.35085056	DCs
FEM1B	2.3197E-05	0.36422618	0.281	0.528	0.3565536	DCs
HHIPL11	2.3286E-05	3.54598741	0.022	0.005	0.35792697	DCs
COQ8B	2.3404E-05	0.43379734	0.139	0.281	0.35975027	DCs
RNF144A	2.3452E-05	2.73789462	0.022	0.005	0.36048364	DCs
FMNL31	2.3497E-05	1.05040517	0.527	0.665	0.36117629	DCs
TGS1	2.3543E-05	0.42183965	0.196	0.374	0.36188332	DCs
ZNF668	2.3598E-05	0.7153337	0.101	0.222	0.3627245	DCs
TBPL1	2.3611E-05	0.25209546	0.091	0.204	0.36292617	DCs
HCFC2	2.3648E-05	0.44660027	0.142	0.285	0.36350011	DCs
NUBP2	2.3655E-05	0.63653661	0.158	0.314	0.36360541	DCs
FAM118B1	2.3662E-05	0.22392313	0.246	0.455	0.36370145	DCs
ULK2	2.3862E-05	0.36191257	0.088	0.2	0.36678415	DCs
KYNU2	2.4069E-05	0.91872265	0.584	0.733	0.36997227	DCs
ZNF691	2.4111E-05	0.27855335	0.066	0.165	0.37061286	DCs
UBAC21	2.4127E-05	0.5070449	0.186	0.363	0.37086214	DCs
GNA152	2.4203E-05	0.9662897	0.539	0.679	0.37202826	DCs
GRPEL1	2.4549E-05	0.48667424	0.259	0.493	0.37734991	DCs
EMC61	2.4747E-05	0.54684917	0.221	0.424	0.38038519	DCs
SPRYD41	2.478E-05	0.30352707	0.11	0.233	0.38089739	DCs
ZC3HC1	2.4833E-05	0.31078508	0.066	0.165	0.38171296	DCs
TAOK3	2.4995E-05	0.76312752	0.571	0.742	0.38420575	DCs
KANSL2	2.5065E-05	0.34624354	0.177	0.336	0.38526814	DCs
MCRS1	2.5236E-05	0.11378784	0.423	0.747	0.38789607	DCs
SLC17A92	2.5248E-05	2.18550846	0.196	0.137	0.38809164	DCs
ECE11	2.528E-05	2.30913502	0.177	0.117	0.38857515	DCs
KPNB1	2.5351E-05	0.18334612	0.293	0.534	0.38967059	DCs
CD551	2.5408E-05	0.49549646	0.71	0.864	0.390546	DCs
VPS13B1	2.5573E-05	1.05640647	0.442	0.479	0.39308719	DCs
ECI1	2.5595E-05	0.42701325	0.293	0.549	0.39341581	DCs
SEPHS1	2.5604E-05	0.42004171	0.091	0.204	0.39356008	DCs
NECAP1	2.5648E-05	0.54661561	0.177	0.345	0.39424051	DCs
FKBP3	2.5728E-05	0.61480643	0.114	0.241	0.39545937	DCs
YME1L1	2.573E-05	0.33530838	0.274	0.513	0.39549789	DCs
CDC341	2.5758E-05	0.26305021	0.24	0.451	0.39592671	DCs
PIAS4	2.5766E-05	0.46988614	0.183	0.354	0.39604605	DCs
ISY12	2.5791E-05	0.15982354	0.328	0.599	0.39643265	DCs
CANT11	2.5861E-05	0.3175842	0.268	0.498	0.39751194	DCs
TFE3	2.6065E-05	0.26157786	0.312	0.578	0.40063787	DCs
TIMM21	2.6244E-05	0.29234167	0.082	0.188	0.40339242	DCs
CNOT4	2.6474E-05	0.39468493	0.148	0.297	0.40693292	DCs
GSK3B	2.6582E-05	0.31463292	0.341	0.627	0.40859435	DCs

DIMT1	2.6649E-05	0.36143892	0.233	0.443	0.40961581	DCs
SLC66A21	2.6865E-05	0.26812101	0.265	0.492	0.41294707	DCs
KLHL36	2.6999E-05	0.36723761	0.246	0.465	0.41500794	DCs
MKRN2	2.7143E-05	0.30431051	0.148	0.296	0.41721956	DCs
NPLOC41	2.7233E-05	0.69047793	0.621	0.79	0.41860498	DCs
QRICH2	2.7365E-05	2.78950904	0.041	0.014	0.42063376	DCs
TTC33	2.7428E-05	0.33915838	0.114	0.234	0.42159722	DCs
RAB3GAP2	2.746E-05	0.40510728	0.221	0.415	0.42208991	DCs
SNRPN	2.7544E-05	0.41310659	0.237	0.443	0.4233796	DCs
ZNF544	2.7638E-05	0.39945765	0.129	0.261	0.42482177	DCs
FKBP111	2.769E-05	1.65571205	0.174	0.113	0.42562834	DCs
ZNF559	2.7788E-05	0.24807769	0.117	0.242	0.42712909	DCs
HMGCS1	2.7946E-05	1.37264584	0.397	0.392	0.42955224	DCs
WSB22	2.86E-05	0.75740888	0.606	0.776	0.43960763	DCs
CHCHD4	2.8667E-05	0.33504861	0.088	0.198	0.44064294	DCs
CNIH11	2.8991E-05	0.24158137	0.268	0.5	0.44562231	DCs
TENT5C	2.9351E-05	1.01825095	0.063	0.026	0.45114668	DCs
FAM20C2	2.9472E-05	0.48401753	0.164	0.321	0.45300832	DCs
EIPR1	3.0041E-05	0.47621475	0.189	0.363	0.46175816	DCs
LSM3	3.0143E-05	0.11613709	0.047	0.132	0.4633225	DCs
GOT11	3.0161E-05	0.34675646	0.091	0.201	0.46361026	DCs
MAP2K7	3.0378E-05	0.31606076	0.23	0.432	0.46693379	DCs
ATF7IP	3.0684E-05	1.23791996	0.426	0.462	0.47164774	DCs
SAE1	3.0699E-05	0.37245271	0.218	0.409	0.47187175	DCs
OXSM	3.071E-05	0.18963316	0.05	0.137	0.47203964	DCs
SLC25A51	3.1034E-05	0.39057979	0.066	0.163	0.47701738	DCs
SETD91	3.1343E-05	0.36601515	0.06	0.153	0.48177541	DCs
RPUSD2	3.1411E-05	0.15665305	0.038	0.117	0.4828118	DCs
TLCD3A1	3.1494E-05	3.72811528	0.019	0.004	0.48409053	DCs
P2RY15	3.1802E-05	0.40271125	0.139	0.277	0.48882695	DCs
SETD1A	3.1856E-05	0.4086517	0.066	0.162	0.48966184	DCs
SEC24A1	3.1966E-05	0.42300349	0.233	0.439	0.49135133	DCs
FBXO22	3.2033E-05	0.40273209	0.192	0.363	0.49238199	DCs
WDR491	3.2339E-05	0.50037252	0.136	0.272	0.49708236	DCs
SLC52A21	3.2389E-05	0.48272228	0.221	0.419	0.49785404	DCs
GRB102	3.2429E-05	2.66079216	0.095	0.048	0.49846703	DCs
FAM120AOS	3.2475E-05	0.54326925	0.233	0.433	0.49917897	DCs
FANCF	3.2726E-05	0.35829588	0.088	0.196	0.50303599	DCs
INSR	3.2825E-05	0.3565524	0.145	0.287	0.50454822	DCs
GMPS	3.3215E-05	0.51283346	0.132	0.269	0.51054444	DCs
MCCC1	3.3521E-05	0.43792309	0.224	0.419	0.5152492	DCs
LRRC28	3.3543E-05	0.40840521	0.139	0.278	0.51558691	DCs
RPAIN	3.3714E-05	0.35114823	0.256	0.473	0.51821455	DCs
SLC5A31	3.3907E-05	2.02495892	0.23	0.175	0.52119097	DCs
SPTLC11	3.3958E-05	0.39206942	0.221	0.415	0.52197215	DCs
BTG3	3.4011E-05	0.37011913	0.132	0.265	0.52278911	DCs
ZNF512	3.412E-05	0.27403585	0.126	0.256	0.52445444	DCs
PPIL3	3.426E-05	0.40292645	0.233	0.435	0.52660436	DCs
VPS39	3.4287E-05	0.29119427	0.35	0.642	0.52702305	DCs
CNTROB	3.4416E-05	0.45393855	0.11	0.229	0.52900098	DCs
ZNF511	3.4533E-05	0.46400775	0.145	0.29	0.53079928	DCs
CNTRL	3.4635E-05	0.81983904	0.584	0.727	0.53237623	DCs

FAS1	3.4696E-05	0.27043281	0.095	0.206	0.53331918	DCs
RETREG2	3.4738E-05	0.13442791	0.341	0.625	0.53395517	DCs
RARG2	3.4883E-05	2.30834863	0.082	0.039	0.53618715	DCs
TFB1M	3.5219E-05	0.20916279	0.069	0.167	0.54135214	DCs
SURF2	3.5641E-05	0.18603933	0.085	0.19	0.54784517	DCs
GPATCH2	3.5828E-05	0.41437551	0.145	0.287	0.55070903	DCs
RAB3IP	3.6081E-05	1.77453493	0.126	0.073	0.55460585	DCs
RABGAP1	3.6445E-05	0.48759251	0.249	0.463	0.56018866	DCs
WDR37	3.6629E-05	0.33204141	0.233	0.434	0.56302529	DCs
MXI1	3.6785E-05	0.49800996	0.088	0.196	0.56541888	DCs
ALS2	3.7021E-05	0.17694367	0.06	0.151	0.56905357	DCs
COA8	3.7197E-05	0.41899357	0.174	0.337	0.57175965	DCs
P3H11	3.7435E-05	0.62106585	0.145	0.29	0.57541288	DCs
MED4	3.7441E-05	0.2963641	0.315	0.59	0.57550426	DCs
FAM228B	3.7587E-05	0.63435437	0.107	0.227	0.57774635	DCs
SLC39A9	3.76E-05	0.36695964	0.281	0.524	0.57795318	DCs
VPS81	3.7642E-05	0.38075323	0.278	0.512	0.57860005	DCs
RNF1811	3.7662E-05	0.44443503	0.284	0.539	0.57890696	DCs
CSF2RB2	3.7709E-05	1.08569414	0.599	0.794	0.5796196	DCs
DHX341	3.7864E-05	0.10283657	0.107	0.223	0.58200078	DCs
TMEM175	3.8462E-05	0.47136579	0.271	0.499	0.59120398	DCs
SNRNP40	3.875E-05	0.15716534	0.347	0.642	0.59563376	DCs
ZC3H7B	3.8941E-05	0.54548668	0.29	0.541	0.59855699	DCs
ZFP1	3.9364E-05	0.22913515	0.054	0.14	0.60505972	DCs
DPH2	3.9461E-05	0.61810067	0.142	0.28	0.60655613	DCs
NME6	3.9719E-05	0.56695673	0.123	0.252	0.61052644	DCs
CPQ1	4.0279E-05	0.20519378	0.322	0.584	0.61912458	DCs
RPTOR	4.0295E-05	0.53309961	0.199	0.378	0.61937266	DCs
TINF2	4.046E-05	0.46525144	0.293	0.549	0.62190565	DCs
KCTD31	4.0578E-05	0.23228924	0.101	0.215	0.62372498	DCs
SMARCD1	4.0649E-05	0.39195394	0.17	0.326	0.62481198	DCs
C1GALT1	4.0694E-05	0.60536106	0.104	0.221	0.62551217	DCs
CEP76	4.0799E-05	0.45632037	0.06	0.15	0.627121	DCs
ZNF598	4.104E-05	0.2598807	0.095	0.204	0.63082569	DCs
VPS72	4.121E-05	0.3509901	0.23	0.431	0.63344613	DCs
STARD82	4.1225E-05	1.9809276	0.297	0.262	0.63367646	DCs
ENDOV	4.1333E-05	2.20794521	0.167	0.111	0.63533622	DCs
HSPA14.1	4.227E-05	0.50967459	0.11	0.231	0.64973097	DCs
RPGR	4.2284E-05	0.41534553	0.098	0.211	0.64994698	DCs
GRK2	4.2401E-05	0.36812703	0.801	0.941	0.6517395	DCs
GPR1571	4.2518E-05	3.21789146	0.347	0.345	0.65354692	DCs
NUP155	4.281E-05	0.35767021	0.12	0.245	0.65803919	DCs
HOMER21	4.3091E-05	2.50654391	0.177	0.12	0.66234987	DCs
SNHG32	4.3775E-05	1.29543888	0.448	0.512	0.67287174	DCs
ZFAND2B	4.3789E-05	0.36556739	0.259	0.471	0.67308338	DCs
JADE2	4.3946E-05	0.56722996	0.145	0.289	0.67549137	DCs
EIF3L	4.4044E-05	0.81830428	0.58	0.751	0.67699266	DCs
DDX52	4.4045E-05	0.66805067	0.158	0.305	0.67701426	DCs
SIRT3	4.4162E-05	0.18823495	0.06	0.151	0.67881325	DCs
SLC25A29	4.4215E-05	2.33431922	0.161	0.106	0.67963163	DCs
ZNF548	4.4399E-05	0.38973463	0.095	0.204	0.68245947	DCs
FOPNL	4.4419E-05	0.37715267	0.211	0.403	0.68277176	DCs

MTRR	4.5069E-05	0.39129233	0.161	0.311	0.69275044	DCs
NHLRC2	4.5157E-05	1.71698663	0.347	0.336	0.69411454	DCs
GMDS	4.5286E-05	0.32492492	0.126	0.253	0.6960975	DCs
METTL16	4.5429E-05	0.44717628	0.101	0.214	0.69828325	DCs
ZSCAN21	4.579E-05	0.10154287	0.066	0.159	0.70383688	DCs
LRP82	4.637E-05	2.16897437	0.132	0.079	0.71275179	DCs
UBE2Q21	4.6501E-05	1.30429259	0.454	0.525	0.71476968	DCs
CDPF1	4.6626E-05	0.15212205	0.047	0.13	0.71669152	DCs
INTS10	4.6808E-05	0.38306516	0.252	0.465	0.71948993	DCs
IRF2	4.6813E-05	0.25544426	0.382	0.692	0.71955975	DCs
SLC8B12	4.6841E-05	0.52828508	0.158	0.307	0.71999885	DCs
ORMDL3	4.6944E-05	0.62001055	0.136	0.271	0.72157861	DCs
SCO1	4.7048E-05	0.42755644	0.107	0.225	0.72317147	DCs
PHACTR42	4.8912E-05	0.30626751	0.249	0.459	0.75183024	DCs
ZBTB40	4.903E-05	0.36085648	0.114	0.233	0.75363332	DCs
POLRMT	4.9094E-05	0.4242508	0.098	0.209	0.75462895	DCs
RBM281	4.9567E-05	0.44474785	0.161	0.314	0.76190101	DCs
AMZ2	4.9724E-05	0.52861521	0.155	0.303	0.76430415	DCs
LRRCC1	4.9847E-05	0.31393286	0.063	0.154	0.76620348	DCs
SCML11	5.0212E-05	0.22515597	0.088	0.192	0.77181003	DCs
TPK11	5.0542E-05	0.55976519	0.227	0.424	0.7768838	DCs
ZSCAN29	5.072E-05	0.32450413	0.123	0.245	0.77961472	DCs
VSIG10	5.0722E-05	2.29488384	0.148	0.093	0.77965168	DCs
SMAD5	5.1186E-05	0.46107062	0.192	0.362	0.78677771	DCs
GTF2F1	5.1667E-05	0.2506135	0.309	0.571	0.79417958	DCs
ARHGEF7	5.1943E-05	0.329356	0.246	0.452	0.79841636	DCs
KTI12	5.2297E-05	0.12352742	0.047	0.13	0.80385121	DCs
KCNQ11	5.2437E-05	0.17116958	0.363	0.642	0.80600169	DCs
DPY19L4	5.2523E-05	0.5300091	0.066	0.159	0.80733867	DCs
TIMM10B	5.2535E-05	0.35458216	0.233	0.43	0.80750854	DCs
CHD8	5.2628E-05	0.34845796	0.278	0.516	0.80894813	DCs
ARFGAP1	5.268E-05	0.15831923	0.129	0.256	0.80974003	DCs
CCNT21	5.285E-05	1.39726164	0.379	0.38	0.81235459	DCs
KCTD71	5.3126E-05	2.06058275	0.164	0.109	0.8165979	DCs
RCHY1	5.3313E-05	0.34192236	0.158	0.302	0.81947404	DCs
PLCD3	5.3766E-05	3.63495278	0.025	0.007	0.82643188	DCs
PPP2CB1	5.3891E-05	0.39552479	0.079	0.177	0.82835622	DCs
MECP2	5.39E-05	0.31331112	0.284	0.516	0.82849046	DCs
USP47	5.4049E-05	0.47602794	0.174	0.33	0.83078736	DCs
RRP151	5.4127E-05	0.45797492	0.164	0.313	0.83198802	DCs
RANGAP1	5.4215E-05	0.45808843	0.205	0.385	0.83333478	DCs
ELP4	5.4371E-05	0.66261106	0.104	0.219	0.83573273	DCs
LRRC57	5.4524E-05	0.49717278	0.227	0.424	0.83809329	DCs
DCXR	5.4629E-05	0.39012921	0.224	0.417	0.83969995	DCs
CARD16	5.4773E-05	0.30660077	0.044	0.124	0.84191177	DCs
DMD1	5.4971E-05	2.82928073	0.114	0.064	0.84495937	DCs
PMPCB	5.5097E-05	0.94187717	0.517	0.638	0.84688904	DCs
WDR3	5.552E-05	0.45784813	0.132	0.262	0.85340005	DCs
METTL26	5.5697E-05	0.3189666	0.319	0.582	0.85611382	DCs
SP140L	5.5714E-05	0.36680902	0.132	0.263	0.85638649	DCs
FTCDNL11	5.6003E-05	0.34570983	0.088	0.193	0.86082741	DCs
BTBD10	5.6112E-05	0.54558359	0.12	0.244	0.86250357	DCs

INAFM22	5.6151E-05	0.51383004	0.18	0.341	0.86309275	DCs
PRELID3B	5.6157E-05	0.45776408	0.183	0.348	0.86318638	DCs
EIF3I	5.6434E-05	0.3999723	0.164	0.315	0.86744303	DCs
CAMK2D	5.65E-05	0.59401997	0.139	0.273	0.86845655	DCs
CXorf38	5.6902E-05	0.27944379	0.353	0.635	0.87463798	DCs
OBI1	5.6931E-05	0.5633831	0.107	0.225	0.87509362	DCs
PARP1	5.7117E-05	0.34677674	0.136	0.269	0.87793777	DCs
UBE2K	5.7297E-05	0.36021257	0.17	0.328	0.88071591	DCs
SMCHD11	5.7305E-05	1.19550907	0.438	0.49	0.88083655	DCs
NOP14	5.7316E-05	0.32635737	0.262	0.474	0.88100712	DCs
TSHZ1	5.7537E-05	0.43861533	0.164	0.315	0.88440756	DCs
PDRG1	5.7674E-05	0.3320761	0.076	0.173	0.88650955	DCs
GPC42	5.8019E-05	2.46183218	0.123	0.072	0.89180598	DCs
FAM98B	5.8128E-05	0.41713693	0.259	0.482	0.89348321	DCs
FAM219A	5.8364E-05	0.34573415	0.12	0.244	0.89710678	DCs
ELMO1	5.8463E-05	0.68831889	0.618	0.807	0.89862783	DCs
FRAT21	5.9573E-05	0.29392573	0.306	0.555	0.91569155	DCs
STAT5B	5.9628E-05	0.35190738	0.249	0.46	0.91654026	DCs
RCAN1	5.9938E-05	0.53499109	0.117	0.238	0.9213002	DCs
TOE1	5.995E-05	0.38872682	0.085	0.189	0.9214914	DCs
FOXN32	6.0054E-05	1.36985415	0.413	0.454	0.9230966	DCs
DCP1A	6.0166E-05	0.50092416	0.199	0.371	0.92481628	DCs
DCP2	6.0308E-05	1.08222585	0.457	0.525	0.92698836	DCs
ZBTB3	6.0497E-05	0.52890478	0.088	0.194	0.92990039	DCs
CUEDC2	6.051E-05	0.36892998	0.265	0.485	0.93010127	DCs
UBE2F	6.0644E-05	0.64285064	0.23	0.427	0.9321634	DCs
PDCD10	6.0644E-05	0.10670068	0.379	0.691	0.93216499	DCs
PNPLA41	6.1563E-05	0.4136554	0.085	0.188	0.94628208	DCs
PTPN21	6.1732E-05	1.62944848	0.379	0.387	0.94887642	DCs
TRIM33	6.1916E-05	0.41863083	0.271	0.492	0.95170785	DCs
DHX16	6.2076E-05	0.388098	0.287	0.534	0.95417138	DCs
PPP2R3C	6.2253E-05	0.42326199	0.271	0.498	0.95689822	DCs
FXR2	6.2818E-05	0.39872274	0.208	0.388	0.96557355	DCs
SCNM1	6.283E-05	1.26275313	0.435	0.5	0.96575386	DCs
KLF92	6.3429E-05	1.64417133	0.338	0.321	0.97496839	DCs
SIK32	6.3514E-05	0.34487559	0.164	0.305	0.9762729	DCs
CCDC183	6.3728E-05	3.35622849	0.022	0.006	0.97956476	DCs
ITPKC	6.4253E-05	0.46161966	0.158	0.301	0.98763176	DCs
NLRP122	6.4479E-05	0.15887897	0.06	0.147	0.99110367	DCs
APPBP2	6.4659E-05	0.51230254	0.158	0.303	0.99387177	DCs
STX31	6.4748E-05	1.67857056	0.382	0.396	0.9952455	DCs
FBXO3	6.4791E-05	0.30490729	0.151	0.291	0.99590965	DCs
RNLS	6.4796E-05	0.55909518	0.069	0.163	0.99598309	DCs
CSTF2T	6.4846E-05	0.37159267	0.246	0.454	0.99675382	DCs
TP53BP22	6.5764E-05	1.68364641	0.331	0.31	1	DCs
THOC5	6.5802E-05	0.63277485	0.148	0.291	1	DCs
MELTF1	6.5905E-05	3.50038415	0.035	0.011	1	DCs
TMEM62	6.6401E-05	0.40540667	0.123	0.249	1	DCs
DKC1	6.6725E-05	0.50487942	0.249	0.458	1	DCs
WDR25	6.6747E-05	0.14983766	0.05	0.132	1	DCs
RNF215	6.7482E-05	0.13554144	0.032	0.103	1	DCs
SF1	6.7485E-05	0.47557885	0.221	0.41	1	DCs

TBC1D10A	6.755E-05	0.60900788	0.259	0.479	1	DCs
DIS3L	6.7625E-05	0.58612238	0.202	0.382	1	DCs
CSNK2B	6.79E-05	0.25495742	0.274	0.506	1	DCs
WBP1	6.8275E-05	0.53943906	0.24	0.448	1	DCs
L3HYPDH	6.843E-05	0.68764835	0.107	0.221	1	DCs
RBFA	6.8856E-05	0.39514235	0.148	0.288	1	DCs
CTU2	6.9493E-05	0.50024153	0.107	0.223	1	DCs
NUTF22	6.9621E-05	0.49584074	0.278	0.512	1	DCs
ALKBH4	7.1626E-05	0.14036798	0.063	0.152	1	DCs
UBE2Z1	7.1796E-05	0.2093898	0.382	0.687	1	DCs
PNMA1	7.1894E-05	1.75111999	0.47	0.555	1	DCs
DSTYK	7.2219E-05	0.34426383	0.098	0.207	1	DCs
VAV32	7.269E-05	2.37649617	0.24	0.192	1	DCs
ATP13A1	7.2714E-05	0.3434834	0.338	0.609	1	DCs
CD69	7.274E-05	1.10890571	0.426	0.422	1	DCs
ALKBH3	7.2962E-05	0.44167561	0.114	0.231	1	DCs
ACOT8	7.3266E-05	0.56893434	0.104	0.218	1	DCs
EIF2AK3	7.3438E-05	0.43070764	0.098	0.206	1	DCs
ZPR1	7.3805E-05	0.6194311	0.211	0.399	1	DCs
PACRGL	7.3918E-05	0.49682267	0.079	0.177	1	DCs
TFAM	7.4117E-05	0.50586564	0.177	0.335	1	DCs
USP21	7.5631E-05	0.41109762	0.098	0.207	1	DCs
MZT1	7.5654E-05	0.3342956	0.256	0.468	1	DCs
DIS3L2	7.5845E-05	0.41625337	0.145	0.281	1	DCs
EXOSC1	7.6476E-05	0.54060433	0.151	0.291	1	DCs
CISD1	7.6653E-05	0.33060619	0.069	0.162	1	DCs
S100Z2	7.6685E-05	2.86491725	0.047	0.018	1	DCs
BIN1	7.6897E-05	0.36171348	0.3	0.534	1	DCs
TBC1D23	7.6904E-05	0.60648736	0.164	0.315	1	DCs
FAM136A1	7.7335E-05	0.32559989	0.215	0.396	1	DCs
BET1L	7.765E-05	0.3168042	0.315	0.569	1	DCs
SPATA5	7.8859E-05	0.26202368	0.107	0.22	1	DCs
GEMIN8	7.9144E-05	0.14702528	0.035	0.106	1	DCs
IL2RG	7.9415E-05	0.44512234	0.757	0.892	1	DCs
LPCAT23	7.9565E-05	0.7659754	0.584	0.75	1	DCs
ITGAX2	7.9708E-05	0.82819497	0.675	0.799	1	DCs
UNK	8.1757E-05	0.64444764	0.148	0.286	1	DCs
PHF111	8.202E-05	0.29479976	0.309	0.566	1	DCs
ERCC2	8.2666E-05	0.42775711	0.12	0.242	1	DCs
DNAJB1	8.3071E-05	0.40714717	0.29	0.531	1	DCs
ATF7	8.3108E-05	0.39070409	0.148	0.287	1	DCs
SIRT5	8.3207E-05	0.15403205	0.057	0.141	1	DCs
DPF2	8.3652E-05	0.44926221	0.233	0.432	1	DCs
CIAO3	8.5062E-05	0.56851064	0.142	0.274	1	DCs
NUP54	8.5461E-05	0.29164378	0.208	0.377	1	DCs
DENR	8.5808E-05	0.56460142	0.259	0.479	1	DCs
LRRC8D1	8.6152E-05	0.37709034	0.312	0.579	1	DCs
ACD	8.6696E-05	0.42558254	0.151	0.29	1	DCs
ZNF526	8.6882E-05	0.47100178	0.091	0.194	1	DCs
SIMC11	8.6955E-05	0.29653485	0.063	0.152	1	DCs
FAM135A2	8.725E-05	2.37438282	0.114	0.065	1	DCs
NOL10	8.7481E-05	0.27172901	0.073	0.165	1	DCs

ZNF318	8.7667E-05	0.52842067	0.142	0.275	1	DCs
EFL1	8.7873E-05	0.44896189	0.151	0.29	1	DCs
AQR	8.8378E-05	0.39066743	0.24	0.439	1	DCs
SLF2	8.8575E-05	0.30964069	0.233	0.421	1	DCs
INTS5	8.8799E-05	0.13481622	0.06	0.145	1	DCs
CST62	8.9167E-05	3.4761949	0.019	0.004	1	DCs
BICDL1	8.9955E-05	2.12166056	0.05	0.02	1	DCs
NPEPPS1	9.0218E-05	0.98076125	0.517	0.649	1	DCs
PPCDC	9.1367E-05	0.50416616	0.123	0.245	1	DCs
CHORDC1	9.1516E-05	0.36027462	0.132	0.26	1	DCs
DGCR2	9.1615E-05	0.41196879	0.183	0.342	1	DCs
PHF13	9.199E-05	0.12082886	0.091	0.193	1	DCs
MSTO1	9.2004E-05	0.42801632	0.098	0.205	1	DCs
FYB11	9.3358E-05	0.23764453	0.293	0.526	1	DCs
TEX10	9.4523E-05	0.45325056	0.104	0.214	1	DCs
ATE1	9.4992E-05	0.28607803	0.088	0.188	1	DCs
RTRAF	9.5479E-05	0.46745482	0.237	0.44	1	DCs
NSUN5	9.5753E-05	0.32393994	0.079	0.174	1	DCs
TAF1D1	9.636E-05	1.14802263	0.435	0.489	1	DCs
PINX1	9.6902E-05	0.28496509	0.085	0.184	1	DCs
CEBPG1	9.696E-05	1.20503254	0.483	0.593	1	DCs
ABRAXAS2	9.7219E-05	0.59989405	0.208	0.388	1	DCs
PER1	9.7374E-05	1.13019591	0.492	0.584	1	DCs
EEF1AKMT31	9.7504E-05	0.16820993	0.05	0.129	1	DCs
STK35	9.7676E-05	0.46405805	0.132	0.258	1	DCs
LDAH	9.8352E-05	0.59388469	0.155	0.297	1	DCs
SAP30L	9.8656E-05	0.2002587	0.328	0.586	1	DCs
ITGB3BP	9.9039E-05	0.19077176	0.054	0.135	1	DCs
BAZ2A	9.9065E-05	0.35673742	0.246	0.449	1	DCs
MXD4	9.9355E-05	0.34783856	0.315	0.569	1	DCs
C2orf42	9.9658E-05	0.20132664	0.044	0.119	1	DCs
NARS2	9.9698E-05	0.31161561	0.114	0.227	1	DCs
TBC1D22B	0.00010095	0.31292378	0.132	0.256	1	DCs
AIFM21	0.00010121	0.3134763	0.041	0.115	1	DCs
CSTF1	0.00010131	0.46791704	0.145	0.28	1	DCs
ERCC4	0.0001015	0.26293601	0.063	0.15	1	DCs
EGLN11	0.00010222	0.28471259	0.369	0.665	1	DCs
RALY	0.00010281	0.16988938	0.325	0.58	1	DCs
RWDD3	0.00010305	0.29337396	0.12	0.234	1	DCs
FEM1C	0.00010335	0.41992307	0.227	0.419	1	DCs
DIP2B2	0.00010371	0.47257258	0.237	0.436	1	DCs
TNFSF82	0.00010472	1.97479679	0.028	0.008	1	DCs
SERPINB82	0.00010532	0.49135959	0.271	0.497	1	DCs
ERMARD	0.00010611	0.44746426	0.132	0.255	1	DCs
ATAD1	0.00010671	0.45296117	0.249	0.455	1	DCs
RFX1	0.00010714	0.50179729	0.17	0.318	1	DCs
ILF31	0.0001074	0.10022404	0.429	0.761	1	DCs
NHP2	0.00010787	0.24753855	0.394	0.708	1	DCs
PARP6	0.00010812	0.43501864	0.132	0.256	1	DCs
ARFGEF21	0.00010829	0.40727146	0.259	0.477	1	DCs
DMAP11	0.00010837	0.50515167	0.218	0.4	1	DCs
IZUMO4	0.00010851	2.90552082	0.028	0.009	1	DCs

BCAT12	0.00010866	1.91279356	0.211	0.16	1	DCs
PIAS1	0.00010885	0.27720909	0.325	0.584	1	DCs
CBX4	0.00010902	0.41480729	0.353	0.628	1	DCs
ZFP3	0.00010939	0.1905875	0.076	0.168	1	DCs
RELCH1	0.00010954	1.37023124	0.391	0.414	1	DCs
ZNF445	0.00010965	0.1767923	0.088	0.187	1	DCs
JUNB3	0.00010987	0.22415945	0.962	0.964	1	DCs
SWI5	0.00011018	0.68328638	0.079	0.175	1	DCs
ROCK2	0.00011026	0.5749948	0.246	0.454	1	DCs
SLC35C11	0.00011049	0.57326374	0.18	0.34	1	DCs
RFT1	0.00011125	0.43412596	0.155	0.296	1	DCs
DICER12	0.00011259	0.45380139	0.328	0.609	1	DCs
ZNF16	0.00011296	0.32900502	0.041	0.114	1	DCs
SENP61	0.00011356	0.91703112	0.521	0.648	1	DCs
ZKSCAN1	0.00011442	0.53067305	0.221	0.405	1	DCs
CHMP1B1	0.00011474	0.72445092	0.618	0.793	1	DCs
PTP4A3	0.00011486	3.1873083	0.025	0.007	1	DCs
COA1	0.00011668	0.46549757	0.076	0.169	1	DCs
MTHFSD	0.00011681	0.34000076	0.082	0.177	1	DCs
TAF4	0.00011686	0.48311777	0.215	0.395	1	DCs
CASP3	0.00011687	0.58400768	0.202	0.375	1	DCs
PRPF18	0.00011734	0.41267501	0.148	0.28	1	DCs
DDX51	0.00011921	0.40306307	0.054	0.134	1	DCs
EIF2S2	0.00011931	0.50500078	0.054	0.135	1	DCs
TMEM52B	0.00011934	0.1389692	0.025	0.089	1	DCs
DDX56	0.00011937	0.55119912	0.205	0.378	1	DCs
ADGRE22	0.00011953	1.88595082	0.224	0.175	1	DCs
KIAA1191	0.00011956	0.39436242	0.233	0.424	1	DCs
GALM1	0.00012034	1.89704784	0.189	0.138	1	DCs
RBM192	0.00012176	0.4088549	0.221	0.402	1	DCs
DPP8	0.00012189	0.39955264	0.265	0.487	1	DCs
RPUSD4	0.00012238	0.40107838	0.192	0.353	1	DCs
PYROXD21	0.00012265	2.53667165	0.022	0.006	1	DCs
NBEAL21	0.00012318	1.91305378	0.319	0.299	1	DCs
SHQ1	0.00012368	0.49019143	0.063	0.148	1	DCs
CMTM7	0.00012457	0.46378491	0.312	0.564	1	DCs
ZNF574	0.00012461	0.44979492	0.104	0.212	1	DCs
TDP1	0.00012466	0.41112177	0.161	0.302	1	DCs
FAHD2A	0.00012467	0.3949756	0.095	0.196	1	DCs
TAZ	0.00012482	0.52405747	0.205	0.376	1	DCs
LSM71	0.00012577	0.4761261	0.246	0.45	1	DCs
DYRK1A	0.00012589	0.39967802	0.265	0.48	1	DCs
KIAA0232	0.00012723	0.4218365	0.208	0.377	1	DCs
IRGQ	0.00012723	0.37628766	0.104	0.209	1	DCs
HAUS1	0.00012746	0.36701175	0.091	0.192	1	DCs
TUT1	0.00012748	0.3899958	0.107	0.216	1	DCs
ERMN1	0.00012781	2.45670511	0.035	0.012	1	DCs
UBTF	0.00012812	0.39438768	0.233	0.422	1	DCs
DNTTIP2	0.00012833	0.2036161	0.341	0.618	1	DCs
ZNF628	0.00012891	0.2175491	0.05	0.129	1	DCs
SMURF1	0.00012926	0.65101227	0.151	0.288	1	DCs
ZDHHC6	0.00012929	0.40818369	0.271	0.497	1	DCs

TIGD1	0.00012971	3.04380927	0.028	0.009	1	DCs
HNRNPM	0.00013065	0.22496373	0.3	0.541	1	DCs
PTDSS2	0.0001309	0.15061553	0.063	0.147	1	DCs
HS6ST1	0.00013112	0.52223645	0.069	0.156	1	DCs
TTC5	0.00013143	0.37306752	0.101	0.206	1	DCs
PGM2L1	0.00013211	0.28264032	0.073	0.163	1	DCs
EDC3	0.0001327	0.43719777	0.155	0.293	1	DCs
RCN2	0.00013305	0.27109374	0.082	0.178	1	DCs
ERCC5	0.00013308	0.38512494	0.303	0.539	1	DCs
LENG1	0.00013367	0.33969317	0.155	0.291	1	DCs
PHF6	0.00013424	0.4803272	0.158	0.297	1	DCs
RNF144B1	0.00013427	1.31535843	0.442	0.497	1	DCs
SLC24A42	0.00013451	0.26171029	0.082	0.174	1	DCs
NR1D2	0.00013507	0.29672934	0.322	0.571	1	DCs
CDC42EP12	0.00013582	2.30049117	0.151	0.1	1	DCs
ERCC6L2	0.0001362	0.60283352	0.196	0.359	1	DCs
PPARA	0.00013634	0.27252701	0.057	0.138	1	DCs
TRIM23	0.00013636	0.49368332	0.073	0.163	1	DCs
JHY	0.00013826	0.62424107	0.189	0.355	1	DCs
PRPF38A1	0.00013947	0.51427357	0.224	0.406	1	DCs
STAG21	0.00013964	1.11720025	0.495	0.61	1	DCs
PTTG1IP2	0.00013973	0.41611716	0.785	0.925	1	DCs
GALK12	0.00014105	0.64212812	0.189	0.349	1	DCs
DENND5B1	0.00014116	2.98434619	0.066	0.031	1	DCs
NT5DC1	0.00014126	0.67914115	0.18	0.336	1	DCs
CTCF	0.00014131	0.38705335	0.281	0.507	1	DCs
TMEM138	0.00014173	0.52596749	0.186	0.34	1	DCs
CCHCR1	0.00014201	0.25152791	0.05	0.128	1	DCs
SENP2	0.00014248	0.42427904	0.233	0.423	1	DCs
EVI5L	0.00014254	0.52249931	0.117	0.232	1	DCs
SLC10A7	0.00014254	0.62078337	0.114	0.228	1	DCs
ABTB1	0.00014337	0.49465871	0.107	0.217	1	DCs
SMIM13	0.00014401	0.13371302	0.047	0.123	1	DCs
SYNCRIP	0.0001442	0.40349113	0.054	0.133	1	DCs
DNAJB61	0.00014422	0.67460309	0.164	0.31	1	DCs
MAP4	0.0001449	0.38866001	0.744	0.905	1	DCs
ARAP32	0.00014555	2.76030623	0.025	0.007	1	DCs
ZNF786	0.00014674	0.27351985	0.082	0.175	1	DCs
ABCF2	0.00014709	0.49835268	0.129	0.25	1	DCs
NFKBIB	0.00014805	0.62946246	0.129	0.253	1	DCs
PLEKHM1	0.0001482	0.15756209	0.066	0.152	1	DCs
MED10	0.00015013	0.32782964	0.278	0.507	1	DCs
C14orf93	0.00015053	0.29563434	0.054	0.133	1	DCs
DNAJC2	0.00015072	0.60051521	0.145	0.278	1	DCs
PDP12	0.0001508	1.81337681	0.265	0.228	1	DCs
RPUSD1	0.00015141	0.73295266	0.101	0.205	1	DCs
ZNF337	0.00015163	0.56078881	0.142	0.269	1	DCs
ANKRD501	0.0001517	0.50540894	0.098	0.201	1	DCs
GMEB1	0.00015404	0.54793325	0.12	0.235	1	DCs
SRSF10	0.00015466	0.55492119	0.069	0.158	1	DCs
GTF2H1	0.00015497	0.29982625	0.287	0.513	1	DCs
SEPSECS	0.00015531	0.38219819	0.11	0.219	1	DCs

C5orf51	0.00015598	0.62898075	0.158	0.298	1	DCs
DAP3	0.00015634	0.30256617	0.347	0.623	1	DCs
SYPL1	0.00015668	0.53349094	0.183	0.343	1	DCs
FBH1	0.00015801	0.61196081	0.224	0.409	1	DCs
CDYL2	0.00015812	0.61276939	0.095	0.195	1	DCs
GFER	0.00015907	0.36283985	0.132	0.253	1	DCs
NDUFS1	0.00015912	0.11648705	0.407	0.722	1	DCs
DSN1	0.00016097	0.28189291	0.114	0.222	1	DCs
FNBP41	0.00016133	1.45243256	0.356	0.356	1	DCs
SFXN4	0.00016142	0.1587697	0.06	0.141	1	DCs
RELA	0.00016153	0.48926409	0.256	0.463	1	DCs
POLR1E	0.00016197	0.54496039	0.129	0.251	1	DCs
TNF	0.0001625	0.210445	0.069	0.156	1	DCs
ZNF2192	0.00016412	0.43602972	0.073	0.161	1	DCs
WHRN1	0.00016459	1.54484002	0.022	0.006	1	DCs
HCLS11	0.00016576	0.32784688	0.845	0.955	1	DCs
IDI1	0.00016593	0.30976411	0.215	0.38	1	DCs
SLC30A6	0.000166	0.5185539	0.151	0.288	1	DCs
RAD17	0.0001673	0.56833351	0.101	0.205	1	DCs
ANAPC4	0.00016824	0.50949376	0.243	0.439	1	DCs
TSSC4	0.00016844	0.59482237	0.167	0.312	1	DCs
ZNF354A	0.0001686	0.25949217	0.095	0.193	1	DCs
TRAM22	0.00017205	0.33744089	0.06	0.142	1	DCs
TFDP1	0.00017213	0.49986085	0.205	0.37	1	DCs
LY75	0.00017321	1.90565081	0.498	0.627	1	DCs
ELK4	0.00017416	0.91780547	0.473	0.578	1	DCs
NIFK1	0.00017569	0.53342543	0.174	0.322	1	DCs
DPM3	0.00017587	0.62880158	0.076	0.167	1	DCs
TMEM185B1	0.00017591	0.52626889	0.265	0.482	1	DCs
RBM8A	0.0001767	0.17215003	0.054	0.13	1	DCs
BIVM	0.00017687	0.21154782	0.047	0.122	1	DCs
FRS2	0.0001769	0.48012495	0.117	0.228	1	DCs
SSU72	0.00017691	0.34252279	0.328	0.597	1	DCs
ARHGAP43	0.00017695	0.97861196	0.521	0.644	1	DCs
RAB22A	0.00017734	0.28423524	0.35	0.636	1	DCs
TIMM44	0.00017777	0.73153761	0.218	0.393	1	DCs
TSC1	0.00017861	0.43738242	0.101	0.205	1	DCs
GATC	0.00017885	0.58944684	0.167	0.311	1	DCs
SRR	0.00017911	0.56284959	0.05	0.126	1	DCs
RAP2C1	0.00018009	0.11281177	0.413	0.73	1	DCs
MAFG1	0.00018124	0.49348327	0.167	0.31	1	DCs
MAGED2	0.00018167	0.32893937	0.363	0.645	1	DCs
SLC11A21	0.00018172	0.15529624	0.356	0.613	1	DCs
FAM122B	0.00018402	0.43366475	0.199	0.365	1	DCs
EPS81	0.00018427	2.44671759	0.091	0.05	1	DCs
PUM3	0.00018486	0.34468638	0.091	0.19	1	DCs
AEBP21	0.00018546	1.25622014	0.413	0.465	1	DCs
RHBDD1	0.00018658	0.60277672	0.117	0.23	1	DCs
HIST1H1E1	0.00018674	1.49089273	0.315	0.29	1	DCs
SLC25A22	0.00018745	0.33180692	0.063	0.145	1	DCs
TOR1AIP2	0.00018771	0.54560785	0.278	0.504	1	DCs
RTL10	0.00018905	0.27043503	0.101	0.203	1	DCs

PYGM	0.00018979	2.83806546	0.013	0.002	1	DCs
IDH21	0.00019037	0.88356934	0.495	0.581	1	DCs
TTC141	0.00019044	1.23666933	0.366	0.365	1	DCs
MTERF3	0.00019152	0.33835013	0.123	0.237	1	DCs
KRT10	0.00019253	0.10186588	0.063	0.145	1	DCs
VPS36	0.00019271	0.41958908	0.306	0.555	1	DCs
DGAT1	0.00019282	0.30143069	0.274	0.5	1	DCs
ARF31	0.00019388	0.35330464	0.325	0.579	1	DCs
ZNF189	0.00019449	0.4209989	0.129	0.246	1	DCs
PGM22	0.00019549	0.60155619	0.256	0.464	1	DCs
LIAS	0.00019576	0.4580535	0.101	0.204	1	DCs
NPHP4	0.00019582	0.3137124	0.069	0.154	1	DCs
PKNOX1	0.00019611	0.55653264	0.12	0.234	1	DCs
THAP3	0.00019664	0.22004704	0.06	0.14	1	DCs
INO80	0.00019686	0.51621407	0.208	0.379	1	DCs
POLR3E	0.00019784	0.30667978	0.145	0.268	1	DCs
SLC30A12	0.00019891	0.46338769	0.107	0.212	1	DCs
SOS1	0.00019912	0.51568385	0.227	0.407	1	DCs
KRAS2	0.00019924	1.37469363	0.382	0.404	1	DCs
RYK1	0.00019952	0.45910714	0.085	0.178	1	DCs
VPS33B	0.00019966	0.83721176	0.076	0.165	1	DCs
PDK1	0.00020086	0.52400257	0.114	0.222	1	DCs
EIF4G2	0.00020091	0.35395247	0.379	0.681	1	DCs
GMCL1	0.00020307	0.26586439	0.05	0.126	1	DCs
ANAPC5	0.00020334	0.23612366	0.35	0.625	1	DCs
MT-CYB1	0.00020423	1.44295869	0.278	0.232	1	DCs
MIF4GD1	0.00020438	0.23423983	0.379	0.667	1	DCs
CAMKMT	0.00020482	0.28224217	0.085	0.178	1	DCs
NSUN2	0.00020617	0.30940438	0.322	0.577	1	DCs
PRKAB1	0.00020652	0.42688357	0.262	0.468	1	DCs
CIC1	0.00020691	1.15103214	0.461	0.556	1	DCs
TRAPPC8	0.0002071	0.486003	0.243	0.442	1	DCs
ODF2	0.00020795	0.72702559	0.091	0.189	1	DCs
CCNB1IP1	0.00020857	2.07137726	0.224	0.179	1	DCs
COMMD8	0.00020905	0.50044561	0.117	0.228	1	DCs
HIPK31	0.00020974	0.53163424	0.672	0.854	1	DCs
ZNF1541	0.00021104	2.71056155	0.041	0.016	1	DCs
HDAC6	0.00021163	0.46212104	0.274	0.492	1	DCs
CBLL1	0.00021185	0.38288446	0.136	0.257	1	DCs
TWF1	0.00021203	0.55831729	0.098	0.197	1	DCs
CLN83	0.00021425	0.35468847	0.281	0.496	1	DCs
DOP1B2	0.00021428	0.66820451	0.114	0.225	1	DCs
MINDY1	0.00021534	2.53361191	0.139	0.091	1	DCs
TMEM432	0.00021576	0.95012845	0.489	0.589	1	DCs
CASP8AP2	0.00021591	0.5172476	0.12	0.232	1	DCs
TEP12	0.00021602	1.40605318	0.369	0.379	1	DCs
CHST102	0.00021772	3.27161655	0.013	0.002	1	DCs
ANKRD27	0.00021832	0.55572582	0.199	0.363	1	DCs
INPP12	0.0002186	0.36845209	0.098	0.198	1	DCs
ZNF304	0.00021974	0.1590044	0.069	0.154	1	DCs
MOB4	0.00022015	0.44787246	0.233	0.421	1	DCs
TOP3A	0.00022092	0.55188397	0.12	0.234	1	DCs

PPP2R5E	0.00022168	0.26664464	0.3	0.538	1	DCs
ATG4B	0.00022171	0.38743961	0.227	0.406	1	DCs
TLE4	0.00022235	0.49800161	0.227	0.413	1	DCs
RTN4IP1	0.0002228	0.31043921	0.035	0.101	1	DCs
MAML31	0.0002277	1.93393648	0.265	0.233	1	DCs
MXRA7	0.00022794	0.27606882	0.095	0.192	1	DCs
THAP5	0.00022807	0.44492273	0.069	0.155	1	DCs
USP39	0.00023062	0.45501309	0.177	0.326	1	DCs
FAM221A	0.00023062	0.28642436	0.06	0.138	1	DCs
FURIN2	0.00023116	0.28113341	0.366	0.658	1	DCs
MED7	0.00023233	0.55446034	0.177	0.322	1	DCs
SAP30BP	0.00023436	0.61435671	0.237	0.424	1	DCs
THUMPD1	0.00023454	0.34275824	0.303	0.536	1	DCs
RASAL31	0.00023469	1.36728744	0.259	0.218	1	DCs
C9orf722	0.0002352	0.99096432	0.514	0.617	1	DCs
MITD11	0.00023699	0.38680361	0.12	0.232	1	DCs
DCPS	0.00023717	0.71236153	0.132	0.255	1	DCs
AP2A1	0.00023791	0.13873754	0.438	0.758	1	DCs
AICDA1	0.00023905	1.0327091	0.003	0.047	1	DCs
CTIF1	0.0002426	0.59626274	0.11	0.217	1	DCs
SAMD9	0.00024333	0.43941298	0.221	0.398	1	DCs
NTHL1	0.0002435	0.45857004	0.079	0.167	1	DCs
C1orf109	0.00024424	0.53495641	0.05	0.125	1	DCs
ARFRP1	0.00024695	0.46014302	0.199	0.363	1	DCs
RPH3AL1	0.00024853	0.17464675	0.041	0.109	1	DCs
MYBBP1A1	0.00024927	0.47085371	0.243	0.434	1	DCs
GTPBP3	0.00024962	0.26117833	0.079	0.166	1	DCs
SLFN12L	0.00024968	0.56100919	0.038	0.014	1	DCs
IPMK	0.00024982	0.50954573	0.101	0.201	1	DCs
MAP3K4	0.00025142	0.56005246	0.205	0.368	1	DCs
DUSP72	0.00025233	1.75509069	0.372	0.397	1	DCs
ZNF675	0.0002537	0.4971659	0.038	0.104	1	DCs
AIFM3	0.00025531	0.40037082	0.069	0.153	1	DCs
TRAF31	0.00025604	0.67361948	0.148	0.277	1	DCs
BCAS3	0.00025609	0.39902144	0.095	0.191	1	DCs
TMEM234	0.00025642	0.51121397	0.082	0.172	1	DCs
PUSL1	0.00025656	0.46512044	0.076	0.164	1	DCs
ZC3H12A2	0.00025695	0.36431563	0.161	0.292	1	DCs
NSMCE3	0.00025788	0.36998847	0.297	0.526	1	DCs
GABPB1	0.00025793	0.51573635	0.129	0.244	1	DCs
ZNF830	0.0002586	0.63262102	0.148	0.276	1	DCs
HDAC31	0.00025918	0.5670551	0.199	0.361	1	DCs
SPIN4	0.00025936	0.49658242	0.066	0.148	1	DCs
UTP25	0.00025998	0.82020716	0.129	0.247	1	DCs
EPG5	0.00026191	0.51684623	0.155	0.289	1	DCs
CEP295	0.00026222	0.2979936	0.044	0.114	1	DCs
CACFD11	0.00026254	0.19716831	0.032	0.094	1	DCs
CCDC61	0.00026645	0.57688626	0.082	0.172	1	DCs
AAGAB	0.0002672	0.45035425	0.256	0.457	1	DCs
LENG91	0.00026799	0.5128687	0.054	0.129	1	DCs
GSTCD	0.00026937	0.26985218	0.079	0.167	1	DCs
ATAT1	0.00026968	0.16987685	0.038	0.104	1	DCs

COMMD7	0.00026978	0.36708855	0.252	0.447	1	DCs
RBCK1	0.00026987	0.63404135	0.189	0.347	1	DCs
ZNF579	0.00027641	0.33258892	0.044	0.113	1	DCs
CDKN1C1	0.00027665	3.13005851	0.028	0.009	1	DCs
CASP8	0.00027677	0.20728212	0.391	0.686	1	DCs
MYO19	0.00028074	0.45883283	0.129	0.243	1	DCs
TUBGCP5	0.00028624	0.30791136	0.082	0.171	1	DCs
IQCN1	0.00028632	1.8315155	0.057	0.026	1	DCs
STARD91	0.00028845	2.50323714	0.057	0.026	1	DCs
EXO5	0.0002894	0.94453701	0.11	0.217	1	DCs
MARVELD12	0.00028987	0.32604538	0.297	0.516	1	DCs
APRT1	0.00028995	0.13409251	0.388	0.682	1	DCs
NPRL2	0.00029063	0.57960533	0.136	0.256	1	DCs
TRAK11	0.00029199	1.16460312	0.489	0.61	1	DCs
CHD12	0.00029614	1.28455268	0.382	0.405	1	DCs
AARSD1	0.0002966	0.54392697	0.098	0.195	1	DCs
RAF1	0.00029661	0.345099	0.309	0.558	1	DCs
MAPK8IP31	0.00029757	1.76028002	0.167	0.117	1	DCs
TBX19	0.00029866	2.21770568	0.079	0.041	1	DCs
HAUS8	0.0002987	0.19375549	0.044	0.113	1	DCs
ZNF420	0.00029975	0.45134439	0.025	0.083	1	DCs
SS18	0.00030025	0.5465865	0.265	0.477	1	DCs
SH3BP41	0.00030063	1.13445343	0.47	0.555	1	DCs
AIG11	0.00030192	0.65583049	0.196	0.354	1	DCs
ZNF2291	0.00030271	0.35944142	0.038	0.103	1	DCs
POLB	0.00030376	0.67647133	0.139	0.261	1	DCs
NUCKS1	0.00030525	0.16817914	0.385	0.683	1	DCs
MFSD121	0.00030694	0.7088963	0.186	0.34	1	DCs
TMEM161B	0.00030855	0.40896474	0.107	0.209	1	DCs
MED12	0.00030914	0.33128988	0.303	0.545	1	DCs
SMARCAD1	0.00031038	0.58641941	0.174	0.32	1	DCs
USPL1	0.0003107	0.3768134	0.174	0.314	1	DCs
SLC37A22	0.00031252	0.66033534	0.259	0.461	1	DCs
SCYL3	0.00031285	0.55377799	0.082	0.17	1	DCs
ZSCAN2	0.0003139	0.33214078	0.06	0.137	1	DCs
BAG2	0.00031457	0.39458834	0.06	0.137	1	DCs
YEATS4	0.00031529	0.44179739	0.117	0.225	1	DCs
NCOA7	0.00031654	0.58380761	0.221	0.397	1	DCs
EXOC8	0.00031787	0.46534013	0.196	0.354	1	DCs
CEP104	0.00031882	0.55892808	0.192	0.346	1	DCs
ZNF805	0.0003204	0.56588208	0.082	0.171	1	DCs
FBXO4	0.00032222	0.75236156	0.079	0.167	1	DCs
ZNF7171	0.00032269	0.55271168	0.063	0.142	1	DCs
ZC3H10	0.00032524	0.5594161	0.155	0.283	1	DCs
SH3BP22	0.00032692	0.20385056	0.375	0.65	1	DCs
STOML1	0.00032891	0.14243158	0.019	0.073	1	DCs
ZNF436	0.00033049	0.3563847	0.069	0.151	1	DCs
LTBP3	0.00033351	0.4705248	0.211	0.377	1	DCs
TMEM86B	0.00033356	2.01865962	0.12	0.074	1	DCs
TRAF7	0.00033676	0.30837701	0.322	0.579	1	DCs
RBL1	0.00033827	0.17673105	0.082	0.169	1	DCs
GGCT	0.00033933	0.69620813	0.161	0.295	1	DCs

HPS11	0.00034096	0.15973831	0.407	0.704	1	DCs
HIST1H3H	0.00034174	3.24046415	0.019	0.005	1	DCs
ACAD11	0.00034226	1.8160078	0.218	0.173	1	DCs
TMEM39B1	0.00034283	0.7562278	0.132	0.249	1	DCs
TNPO2	0.00034314	0.39215654	0.161	0.29	1	DCs
NAA381	0.00034474	0.44990781	0.268	0.475	1	DCs
SERGEF	0.00034607	0.60892631	0.129	0.246	1	DCs
CCDC82	0.00034664	0.37419022	0.246	0.436	1	DCs
EID2	0.00034808	0.4011792	0.091	0.185	1	DCs
C3orf38	0.0003482	0.54514361	0.246	0.44	1	DCs
SSNA1	0.00034959	0.4477767	0.265	0.462	1	DCs
USP14	0.00035029	0.26429447	0.36	0.641	1	DCs
APOO	0.00035186	0.7176985	0.082	0.171	1	DCs
NBAS	0.00035486	0.51745904	0.208	0.37	1	DCs
OFD1	0.00035496	1.94972242	0.205	0.16	1	DCs
TCF3	0.00035929	0.59103796	0.167	0.305	1	DCs
ZNF200	0.00035959	0.49535767	0.095	0.19	1	DCs
ZNF212	0.00035986	0.36751895	0.057	0.131	1	DCs
SLC5A61	0.00035995	0.60860014	0.164	0.299	1	DCs
MEX3D	0.00036096	0.4897843	0.069	0.15	1	DCs
SDHAF1	0.00036241	0.63414888	0.098	0.196	1	DCs
IK	0.00036459	0.1487161	0.41	0.713	1	DCs
KLHL28	0.00036566	0.64004277	0.132	0.246	1	DCs
RELL11	0.0003658	0.53335653	0.18	0.325	1	DCs
CLEC2B1	0.00036592	0.1851934	0.372	0.654	1	DCs
PIK3CB	0.00036749	0.44601588	0.35	0.628	1	DCs
MARC2	0.00036835	3.51717693	0.016	0.004	1	DCs
IL4R	0.00036897	0.6436526	0.098	0.195	1	DCs
ELMOD2	0.00036904	0.50452738	0.183	0.327	1	DCs
CBX3	0.00037009	0.33495629	0.309	0.551	1	DCs
PARL	0.00037051	0.44377385	0.303	0.536	1	DCs
INO80E	0.00037106	0.37120822	0.306	0.548	1	DCs
ZCCHC9	0.00037275	0.45405052	0.17	0.311	1	DCs
DNM12	0.00037378	2.75207724	0.057	0.026	1	DCs
ATP5PO1	0.00037393	0.28009722	0.054	0.127	1	DCs
KPNA31	0.00037571	0.55882513	0.29	0.523	1	DCs
TMEM80	0.00037582	0.55184478	0.091	0.184	1	DCs
TAF15	0.0003762	0.54477508	0.215	0.385	1	DCs
PARPBP	0.00037677	0.3290888	0.016	0.067	1	DCs
IRF11	0.00037686	0.62188382	0.36	0.621	1	DCs
GAB21	0.00037708	0.41903387	0.268	0.479	1	DCs
ZBTB6	0.00037847	0.76252824	0.104	0.205	1	DCs
APP2	0.00037848	0.52739977	0.517	0.527	1	DCs
GINM1	0.00037881	0.43471536	0.347	0.628	1	DCs
ABHD15	0.00038128	0.68049599	0.218	0.388	1	DCs
HMGN4	0.00038234	0.82575131	0.546	0.717	1	DCs
VOPP1	0.00038443	1.2023181	0.495	0.631	1	DCs
KATNA1	0.00038652	0.64321231	0.139	0.26	1	DCs
WDR7	0.00038698	0.52853778	0.12	0.226	1	DCs
TMEM131	0.00038848	1.43446786	0.404	0.454	1	DCs
PRR12	0.00039077	0.41741423	0.243	0.428	1	DCs
MTF2	0.00039093	0.4056153	0.183	0.327	1	DCs

MSL1	0.00039145	0.46040903	0.338	0.612	1	DCs
PIAS2	0.00039221	0.69793743	0.24	0.43	1	DCs
EGR12	0.00039315	1.69015164	0.145	0.094	1	DCs
LACC11	0.0003933	0.80047567	0.132	0.249	1	DCs
NINJ11	0.00039342	0.98415578	0.237	0.412	1	DCs
TRMT2A	0.00039517	0.59391717	0.211	0.374	1	DCs
EIF5B	0.00039609	0.25924406	0.325	0.58	1	DCs
METTL22	0.00039931	0.45111103	0.114	0.217	1	DCs
MSANTD4	0.00039958	0.61759565	0.18	0.328	1	DCs
LIN7B	0.00040077	0.43403143	0.076	0.159	1	DCs
MIER3	0.0004011	0.72517654	0.123	0.234	1	DCs
CDKN2AIPNL	0.00040186	0.17822571	0.05	0.12	1	DCs
PLAGL11	0.00040194	0.57140304	0.278	0.487	1	DCs
BRD4	0.00040303	0.7622669	0.552	0.735	1	DCs
EFNA4	0.00040329	0.15978834	0.022	0.077	1	DCs
DDIT41	0.00040599	0.2125939	0.148	0.26	1	DCs
VCL2	0.00040722	0.41523565	0.082	0.169	1	DCs
ITPR1	0.00040729	0.51406446	0.284	0.502	1	DCs
PURA	0.00040832	0.51261956	0.274	0.492	1	DCs
PARN	0.00040923	0.43421149	0.218	0.387	1	DCs
TMEM241	0.00041141	0.39505198	0.054	0.125	1	DCs
ZNF595	0.00042035	0.45062667	0.088	0.178	1	DCs
IFT88	0.000422	0.27644622	0.079	0.162	1	DCs
ZNF449	0.00042355	0.43385831	0.06	0.135	1	DCs
RBM22	0.000425	0.45985061	0.268	0.486	1	DCs
RILPL11	0.0004263	0.44084954	0.06	0.134	1	DCs
DNAJC15	0.0004286	0.38140471	0.338	0.61	1	DCs
ANKIB1	0.00042959	0.62399768	0.12	0.228	1	DCs
PITHD1	0.00043172	0.33445677	0.303	0.539	1	DCs
FBF1	0.00043884	0.29696684	0.06	0.134	1	DCs
TDRD71	0.00044123	0.39893937	0.331	0.584	1	DCs
NEK11	0.00044466	0.15577651	0.032	0.091	1	DCs
ZNF197	0.00044791	0.43452802	0.095	0.189	1	DCs
RGP1	0.00045013	0.43609048	0.17	0.309	1	DCs
CRYZL1	0.0004504	0.32781125	0.073	0.153	1	DCs
ESR11	0.00045047	0.57062735	0.032	0.09	1	DCs
TTI1	0.00045215	0.48297133	0.104	0.202	1	DCs
TRIM5	0.00045453	0.54930989	0.249	0.44	1	DCs
GPRIN3	0.00046271	0.50630157	0.694	0.879	1	DCs
AGA1	0.00046568	0.41149191	0.3	0.534	1	DCs
SIKE11	0.00046714	0.54032962	0.199	0.353	1	DCs
TREML12	0.00046718	3.27587088	0.066	0.033	1	DCs
ZFAND2A	0.00046919	0.50333104	0.142	0.26	1	DCs
ANKRD171	0.00047613	0.3388568	0.36	0.646	1	DCs
CLOCK	0.00047939	0.66005627	0.164	0.297	1	DCs
CEP131	0.00048118	2.41998323	0.164	0.118	1	DCs
EIF2B4	0.00048724	0.62599708	0.18	0.327	1	DCs
NTMT1	0.0004885	0.52199506	0.215	0.383	1	DCs
COMMD3	0.00048906	0.54278813	0.218	0.387	1	DCs
POLR3C	0.00048994	0.42295805	0.249	0.437	1	DCs
ZNF543	0.00049401	0.3340549	0.063	0.138	1	DCs
USP12	0.00049578	1.64994571	0.375	0.408	1	DCs

RWDD2B	0.00049639	0.37563037	0.054	0.124	1	DCs
LCORL	0.00049647	0.48076318	0.177	0.314	1	DCs
PHF141	0.00050202	0.7039258	0.192	0.34	1	DCs
UTP11	0.00051009	0.59650304	0.12	0.225	1	DCs
TSPAN321	0.00051208	0.25451481	0.079	0.161	1	DCs
WRAP53	0.00051684	0.68888987	0.088	0.178	1	DCs
CD1D2	0.00051708	1.90542949	0.17	0.124	1	DCs
UVSSA	0.00051752	0.50977102	0.114	0.215	1	DCs
TMEM150A3	0.00051832	0.5565012	0.196	0.346	1	DCs
CMTM11	0.00052029	2.54912621	0.035	0.013	1	DCs
WASF2	0.0005207	0.14576294	0.448	0.76	1	DCs
ZNF35	0.00052143	0.6535245	0.041	0.105	1	DCs
ZNF282	0.00052233	0.78967185	0.151	0.278	1	DCs
LETMD1	0.00052747	0.45265103	0.268	0.473	1	DCs
FMR1	0.00052783	0.46966999	0.265	0.469	1	DCs
NOL4L	0.00052916	0.72021898	0.23	0.413	1	DCs
OXSR1	0.00053602	0.43187147	0.328	0.592	1	DCs
TMEM203	0.00053729	0.61592401	0.145	0.266	1	DCs
RAE1	0.00053768	0.53654107	0.148	0.273	1	DCs
KIF3A	0.00053965	0.30835547	0.06	0.134	1	DCs
NOD22	0.00053968	1.4387599	0.369	0.392	1	DCs
MFSD8	0.00054121	0.52168171	0.104	0.201	1	DCs
FAM160B1	0.00054224	0.17908809	0.057	0.129	1	DCs
ATP13A32	0.00054318	0.53949898	0.224	0.392	1	DCs
ALG6	0.00054366	0.38580228	0.073	0.152	1	DCs
KANSL11	0.00054461	0.83181645	0.508	0.638	1	DCs
TMEM131L	0.00054758	0.45171571	0.372	0.664	1	DCs
AP4M1	0.00055096	0.255244	0.069	0.147	1	DCs
DPAGT12	0.00055154	0.45708678	0.161	0.289	1	DCs
IL11RA	0.00055591	0.37449028	0.11	0.208	1	DCs
PPIP5K21	0.00055596	0.46524598	0.284	0.499	1	DCs
DDB2	0.00055648	0.41277322	0.142	0.258	1	DCs
SNRPD1	0.00055665	0.55468177	0.069	0.148	1	DCs
ATP11C	0.00055757	0.73744089	0.085	0.171	1	DCs
CCDC14	0.00055759	0.19951136	0.129	0.235	1	DCs
URB2	0.00056068	0.3225074	0.063	0.137	1	DCs
CSNK1G3	0.00056079	0.5843074	0.091	0.182	1	DCs
USP28	0.00056106	0.50298682	0.095	0.185	1	DCs
PYGO2	0.00056317	0.43712856	0.233	0.415	1	DCs
SKIV2L	0.00056337	0.20147412	0.041	0.104	1	DCs
ZNF1431	0.00056353	0.61298315	0.145	0.265	1	DCs
HYI	0.0005673	0.57145772	0.091	0.181	1	DCs
TNRC6A	0.00057129	0.56246271	0.233	0.408	1	DCs
TRIM46	0.00057165	2.94554898	0.016	0.004	1	DCs
HELQ	0.00057247	0.60307272	0.123	0.229	1	DCs
SLC46A31	0.00057247	0.83680226	0.211	0.379	1	DCs
YTHDF3	0.00057669	0.28891233	0.394	0.688	1	DCs
ZNF1171	0.00057795	0.42110943	0.073	0.152	1	DCs
E2F3	0.0005926	0.64785245	0.183	0.329	1	DCs
ASPSCR11	0.00059285	0.46824947	0.215	0.378	1	DCs
OSBPL71	0.00059648	1.91006213	0.019	0.005	1	DCs
CTDP1	0.00060265	0.40128306	0.293	0.519	1	DCs

F11R1	0.000606	0.5981428	0.64	0.84	1	DCs
RAB30	0.00060928	2.82219628	0.076	0.04	1	DCs
ACSL42	0.00060951	0.74066165	0.555	0.717	1	DCs
SMU1	0.00061286	0.59530445	0.104	0.2	1	DCs
DHX81	0.00061416	0.37818317	0.312	0.549	1	DCs
PDZD3	0.00061466	3.82954878	0.013	0.003	1	DCs
RBM61	0.00061529	1.07883046	0.438	0.51	1	DCs
CLCC1	0.00061692	0.64001372	0.221	0.39	1	DCs
TAF9B	0.0006212	0.18524251	0.016	0.064	1	DCs
DCAF8	0.0006352	0.20862316	0.397	0.688	1	DCs
COTL11	0.00063668	0.35346624	0.823	0.94	1	DCs
BBS9	0.00063859	0.34798293	0.079	0.159	1	DCs
ARHGAP20	0.00064276	4.13443427	0.013	0.003	1	DCs
ZNF687	0.00064815	0.64597602	0.202	0.356	1	DCs
AKT1S11	0.00064881	1.8037283	0.183	0.321	1	DCs
RAD1	0.00064971	0.62782285	0.136	0.247	1	DCs
VEZF1	0.00064982	0.534103	0.262	0.456	1	DCs
UBE4B2	0.00065022	0.53161222	0.259	0.451	1	DCs
RIOX21	0.00065393	0.59174734	0.243	0.425	1	DCs
ZNF350	0.00065396	0.46520156	0.098	0.188	1	DCs
PIGM	0.00065559	0.46295357	0.085	0.171	1	DCs
MRFAP1	0.00065823	0.1163649	0.044	0.108	1	DCs
IGF2BP22	0.00065873	2.44805147	0.057	0.027	1	DCs
ZFAT	0.00065966	0.11151433	0.035	0.094	1	DCs
USP101	0.00066241	0.58218563	0.293	0.519	1	DCs
SCIN1	0.00066441	2.96595286	0.013	0.003	1	DCs
MTG2	0.00066452	0.37080269	0.088	0.175	1	DCs
SUPT4H1	0.00066491	0.26991511	0.382	0.68	1	DCs
TRMT44	0.00066624	0.41091795	0.069	0.144	1	DCs
PPP1R261	0.00066713	0.66359716	0.095	0.184	1	DCs
SCAMP1	0.00066911	0.72698699	0.139	0.256	1	DCs
AUH	0.00067494	0.85808009	0.091	0.178	1	DCs
C6orf621	0.00067734	0.85445961	0.511	0.659	1	DCs
SAT21	0.00067853	0.31048626	0.35	0.621	1	DCs
TBCCD1	0.00068327	0.5904405	0.12	0.225	1	DCs
DOCK2	0.00068476	0.40412123	0.751	0.911	1	DCs
C12orf65	0.00068695	0.4574875	0.057	0.126	1	DCs
TRAPPC11	0.00068717	0.38744226	0.278	0.481	1	DCs
ZNF274	0.00069061	0.54225206	0.145	0.264	1	DCs
JAZF12	0.00069261	1.54177601	0.388	0.434	1	DCs
CFAP97	0.00069529	0.63111057	0.151	0.275	1	DCs
COQ4	0.00069576	0.57526032	0.233	0.412	1	DCs
SARNP	0.00069984	0.65311709	0.189	0.341	1	DCs
ZNF180	0.00070226	0.53480469	0.038	0.098	1	DCs
C1orf52	0.00070763	0.50168555	0.208	0.359	1	DCs
CPSF4	0.00071325	0.55925052	0.11	0.208	1	DCs
SFSWAP	0.00071496	0.54680161	0.18	0.32	1	DCs
UBXN4	0.00071519	0.13018841	0.479	0.815	1	DCs
BCDIN3D	0.00071832	0.28670547	0.038	0.097	1	DCs
ZNF7	0.0007244	0.56246258	0.104	0.199	1	DCs
SMYD4	0.00072493	0.34181179	0.104	0.196	1	DCs
GFOD2	0.00072535	0.6330794	0.091	0.18	1	DCs

SRCAP1	0.0007261	0.7942285	0.508	0.645	1	DCs
NBEAL1	0.00072867	0.34508758	0.063	0.136	1	DCs
ABCD41	0.00072905	0.77352007	0.145	0.264	1	DCs
NR2C2AP1	0.000734	0.59472987	0.145	0.262	1	DCs
SMIM121	0.00073506	0.30030015	0.344	0.603	1	DCs
AVL91	0.00073946	0.42765099	0.249	0.44	1	DCs
SPG71	0.00074306	0.40713377	0.278	0.481	1	DCs
DDIT3	0.0007459	0.51780007	0.199	0.349	1	DCs
RASGRP42	0.00075204	0.64453071	0.246	0.439	1	DCs
AGAP2	0.00075573	0.4065423	0.151	0.271	1	DCs
GPC21	0.00075648	3.47807415	0.016	0.004	1	DCs
USF1	0.00076035	0.26920995	0.394	0.69	1	DCs
RBM10	0.00076056	0.57761533	0.249	0.436	1	DCs
PPA1	0.00076289	1.82488685	0.309	0.307	1	DCs
SMIM38	0.00076381	3.20713981	0.013	0.003	1	DCs
BORCS6	0.00076393	0.51589099	0.199	0.348	1	DCs
CLEC4A1	0.00076478	1.15893442	0.489	0.594	1	DCs
ASB3	0.00076813	0.48028102	0.145	0.261	1	DCs
ICK	0.00077255	0.3060394	0.079	0.159	1	DCs
KDM3A	0.00078334	0.49409135	0.221	0.388	1	DCs
MBNL21	0.00078429	0.7099925	0.167	0.299	1	DCs
KLC4	0.00078962	0.49513712	0.066	0.141	1	DCs
ARFGAP2	0.00079016	0.62882177	0.249	0.441	1	DCs
REXO1	0.00079323	0.70453462	0.114	0.211	1	DCs
TAF1C	0.0007966	0.38886685	0.076	0.154	1	DCs
MTRF1L	0.00079684	0.39391684	0.054	0.121	1	DCs
TOR1AIP1	0.00080174	0.16609273	0.435	0.749	1	DCs
ARMCX5	0.00080855	0.50831221	0.079	0.159	1	DCs
TRIB2	0.00081623	0.89562638	0.063	0.031	1	DCs
SAT12	0.00081725	0.65074486	0.883	0.961	1	DCs
RERE1	0.00081757	1.18249713	0.429	0.501	1	DCs
DROSHA	0.00082094	0.5454688	0.202	0.352	1	DCs
ID21	0.00082198	1.66426776	0.517	0.655	1	DCs
SH3TC12	0.00082423	1.18937172	0.432	0.502	1	DCs
ESCO1	0.00082462	0.60950703	0.145	0.263	1	DCs
GSE12	0.00082762	1.34883994	0.391	0.435	1	DCs
CCS	0.00082979	0.72639135	0.126	0.232	1	DCs
ZNF444	0.00084165	0.47987485	0.101	0.191	1	DCs
ESS2	0.0008432	0.54217904	0.11	0.207	1	DCs
PTAFR2	0.00084421	0.19321599	0.473	0.803	1	DCs
CLK41	0.00084433	1.65075109	0.233	0.197	1	DCs
TRIM24	0.00084722	3.15527902	0.013	0.003	1	DCs
GADD45G2	0.00085119	0.27506941	0.038	0.096	1	DCs
CDKN1B	0.00085288	0.62176305	0.606	0.807	1	DCs
SLFN51	0.00086177	0.14123668	0.297	0.497	1	DCs
DNAJB9	0.00087337	0.61223118	0.252	0.446	1	DCs
MGAT4B1	0.00087711	0.17368189	0.388	0.673	1	DCs
ZSCAN91	0.00087968	1.94804956	0.164	0.12	1	DCs
RNMT1	0.00088098	0.39914393	0.192	0.339	1	DCs
NDUFAF5	0.00088178	0.43694396	0.066	0.138	1	DCs
CHAF1A	0.00088394	0.44842659	0.073	0.149	1	DCs
FAM98C	0.00088508	0.53244675	0.104	0.196	1	DCs

TUBG21	0.00088526	0.94914446	0.069	0.144	1	DCs
FITM2	0.00088883	0.30585323	0.05	0.116	1	DCs
ATP5F1D	0.00088992	0.23800552	0.388	0.684	1	DCs
SPATA6	0.00089011	0.67491353	0.11	0.206	1	DCs
MIA1	0.00089021	2.83494692	0.019	0.005	1	DCs
WDR111	0.00089275	0.43497198	0.256	0.443	1	DCs
PTAR11	0.00089309	1.36444908	0.315	0.309	1	DCs
RALGAPA2	0.00089562	0.44363187	0.123	0.224	1	DCs
CEP164	0.00089586	0.82870019	0.164	0.295	1	DCs
CHD9	0.00089702	0.35201461	0.331	0.578	1	DCs
HIKESHI	0.00090078	0.4924388	0.041	0.101	1	DCs
NAIF1	0.00090857	0.45211688	0.114	0.209	1	DCs
DSC21	0.00091383	0.62414507	0.309	0.545	1	DCs
LTBP4	0.00091469	0.32582201	0.126	0.228	1	DCs
FKBP51	0.0009207	0.29326817	0.385	0.66	1	DCs
POP1	0.00092371	0.26905632	0.032	0.086	1	DCs
UBA3	0.00092568	0.3553095	0.347	0.618	1	DCs
TNFRSF10B1	0.00093071	0.56098998	0.271	0.462	1	DCs
XPR1	0.00093432	0.66394915	0.148	0.268	1	DCs
KLRF1	0.00094338	2.69677436	0.013	0.003	1	DCs
MFSD111	0.00094583	0.52482176	0.224	0.39	1	DCs
ZNF787	0.00095644	0.53220037	0.047	0.111	1	DCs
GPATCH1	0.00095845	0.4258292	0.05	0.115	1	DCs
NKRF	0.000971	0.72405143	0.107	0.203	1	DCs
UHMK1	0.0009777	0.21595999	0.426	0.738	1	DCs
RNF2	0.00097821	0.52564146	0.164	0.287	1	DCs
TTLL41	0.00097869	0.5928162	0.107	0.2	1	DCs
TXNDC15	0.00098153	0.28291271	0.366	0.64	1	DCs
TMEM168	0.00098508	0.5290754	0.189	0.332	1	DCs
GAS8	0.00098532	0.42059401	0.035	0.091	1	DCs
ZNF677	0.00098985	0.68873508	0.054	0.12	1	DCs
PLA2G4A1	0.000991	0.65346152	0.192	0.335	1	DCs
CNDP23	0.00099385	0.31615632	0.874	0.941	1	DCs
ZNF567	0.00099543	0.14908368	0.063	0.133	1	DCs
GPALPP1	0.00099778	0.63131555	0.155	0.273	1	DCs
RNGTT	0.00099973	0.48149652	0.243	0.423	1	DCs
MARCH61	0.00100023	1.10675747	0.435	0.521	1	DCs
CCDC692	0.00100791	1.28110221	0.309	0.299	1	DCs
DEF81	0.00101171	0.11195615	0.019	0.066	1	DCs
FAM114A1	0.0010129	0.32930806	0.063	0.133	1	DCs
SOS2	0.00101479	0.30997791	0.36	0.626	1	DCs
CDK11B	0.00101686	0.63934631	0.107	0.2	1	DCs
EIF5A2	0.00101758	0.33344758	0.038	0.096	1	DCs
SCMH1	0.0010272	0.49869375	0.095	0.179	1	DCs
SLC66A1	0.001028	0.63885271	0.085	0.167	1	DCs
SLC35A2	0.00102931	0.67636602	0.044	0.105	1	DCs
EHHADH1	0.00103404	0.73684627	0.047	0.11	1	DCs
MPPE1	0.00103473	0.7206963	0.237	0.411	1	DCs
RAB40C	0.00103794	0.66592808	0.044	0.105	1	DCs
LIG31	0.00103876	0.69705419	0.155	0.275	1	DCs
PLAGL2	0.00103909	0.5568057	0.098	0.186	1	DCs
MAPK8	0.00103975	0.77141349	0.17	0.302	1	DCs

PDCD6	0.00103984	0.12918096	0.442	0.749	1	DCs
C10orf143	0.00104014	0.46640632	0.035	0.09	1	DCs
PTBP11	0.00104051	0.66812738	0.23	0.404	1	DCs
BCL9L1	0.00104571	1.71060102	0.24	0.204	1	DCs
ARHGAP35	0.00104622	0.4244502	0.205	0.358	1	DCs
NDOR1	0.00104787	0.73841703	0.069	0.143	1	DCs
MED28	0.0010502	0.81844148	0.514	0.663	1	DCs
RFXANK1	0.00105495	0.36913125	0.309	0.541	1	DCs
TOGARAM1	0.00106547	0.75535586	0.11	0.205	1	DCs
TSPYL2	0.0010665	0.25395854	0.063	0.133	1	DCs
ZBTB41	0.00107132	0.62567835	0.155	0.275	1	DCs
PACC12	0.00107382	0.57237672	0.249	0.431	1	DCs
RNF2131	0.00107754	0.12455483	0.587	0.9	1	DCs
MGME1	0.0010879	0.72229579	0.208	0.361	1	DCs
STARD4	0.00109267	1.63678844	0.268	0.241	1	DCs
ELF11	0.00109398	0.41851492	0.659	0.843	1	DCs
CBX7	0.00109949	0.40167606	0.088	0.17	1	DCs
LBR2	0.00110107	1.39649851	0.341	0.35	1	DCs
GNL3L	0.0011032	0.4067344	0.325	0.566	1	DCs
MIGA1	0.00110323	0.7947771	0.129	0.235	1	DCs
PABPC1L	0.00110855	1.84740434	0.155	0.111	1	DCs
SLC41A3	0.00111194	0.63178042	0.145	0.26	1	DCs
ZNF446	0.00111515	0.23198138	0.047	0.109	1	DCs
PDE6D	0.00111659	0.65296321	0.224	0.392	1	DCs
ZNF224	0.00111678	0.60023302	0.142	0.253	1	DCs
CDR2	0.00112111	0.6364995	0.129	0.233	1	DCs
NEU3	0.00112348	0.71677299	0.098	0.184	1	DCs
ZNF7931	0.00112735	3.379659	0.016	0.004	1	DCs
ASXL2	0.00113538	0.40353305	0.271	0.469	1	DCs
PHF8	0.00113696	0.61192827	0.196	0.339	1	DCs
WDR60	0.00113935	0.52466679	0.101	0.191	1	DCs
BAK1	0.00113964	0.2908272	0.344	0.599	1	DCs
ZNF329	0.00114038	0.71022179	0.091	0.174	1	DCs
TBKBP12	0.00114238	2.1843084	0.069	0.037	1	DCs
FZD71	0.00114842	0.20326804	0.032	0.085	1	DCs
ANKRD37	0.00115755	0.61890931	0.05	0.114	1	DCs
FAM160A21	0.00116225	1.56697593	0.35	0.366	1	DCs
GATAD1	0.00116407	0.50562896	0.281	0.49	1	DCs
PAQR4	0.00116628	0.29940922	0.035	0.089	1	DCs
KPNA11	0.00116775	0.42207091	0.328	0.57	1	DCs
HDAC42	0.00116977	0.63059343	0.148	0.261	1	DCs
PNN	0.00117059	0.68793065	0.215	0.372	1	DCs
CYFIP2	0.00117258	0.29136723	0.177	0.304	1	DCs
TET32	0.00117503	0.78785077	0.18	0.312	1	DCs
ZKSCAN5	0.00117573	0.76649848	0.073	0.147	1	DCs
MIA2	0.00118148	0.31723504	0.328	0.573	1	DCs
SECISBP21	0.0011969	0.68205647	0.218	0.374	1	DCs
RMI21	0.00120073	2.85256485	0.107	0.068	1	DCs
CLDN12	0.00120711	0.75960966	0.066	0.136	1	DCs
ZNF227	0.00120992	0.66397738	0.057	0.122	1	DCs
RNF167	0.00121351	0.36680223	0.341	0.599	1	DCs
RGCC2	0.00121992	0.11202699	0.694	0.939	1	DCs

OSBPL9	0.00122246	0.55084429	0.293	0.512	1	DCs
RB12	0.00123046	1.32827038	0.385	0.433	1	DCs
BRD1	0.00123128	0.83088	0.483	0.607	1	DCs
ABL1	0.00123254	0.54070622	0.202	0.351	1	DCs
AMMECR1L	0.0012346	0.64527098	0.114	0.208	1	DCs
CDKL51	0.00124216	0.68616808	0.129	0.231	1	DCs
BCLAF3	0.00125184	0.49016861	0.145	0.254	1	DCs
CCDC972	0.00125212	0.4295707	0.278	0.478	1	DCs
KDM4C	0.00125431	0.95440015	0.479	0.596	1	DCs
MED26	0.00125908	0.79549665	0.107	0.2	1	DCs
CD5	0.00126035	1.51561825	0.098	0.059	1	DCs
ZFC3H11	0.00126434	1.05878531	0.404	0.446	1	DCs
WDR47	0.00126654	0.63314864	0.132	0.239	1	DCs
FBXL20	0.0012692	0.53278068	0.145	0.256	1	DCs
SUDS3	0.0012708	0.62404533	0.151	0.266	1	DCs
TIGD2	0.00127245	0.37391705	0.05	0.112	1	DCs
ZNF398	0.00127754	0.54609297	0.123	0.223	1	DCs
CCDC71L	0.00128119	0.80365993	0.148	0.264	1	DCs
SFI1	0.00128409	0.45355611	0.117	0.214	1	DCs
UBA2	0.00128858	0.4197359	0.278	0.476	1	DCs
HACD22	0.00128863	1.65939205	0.312	0.311	1	DCs
KYAT1	0.00129097	0.289156	0.025	0.075	1	DCs
RAB5A	0.00129472	0.69193193	0.533	0.688	1	DCs
VBP1	0.00130152	0.5697493	0.249	0.432	1	DCs
AGK	0.00130602	0.64321717	0.107	0.197	1	DCs
DYM1	0.00131047	1.0109402	0.476	0.593	1	DCs
HPGDS1	0.00131312	0.73311229	0.221	0.379	1	DCs
ORC5	0.0013197	0.3352774	0.066	0.136	1	DCs
NOL9	0.00132187	0.59768638	0.17	0.297	1	DCs
ARHGAP91	0.00133544	1.23658351	0.429	0.506	1	DCs
MED22	0.00134039	0.50786763	0.164	0.288	1	DCs
PCF111	0.0013432	1.00391583	0.473	0.59	1	DCs
MIOS2	0.00135449	0.59979595	0.199	0.338	1	DCs
ZFP69	0.00135794	0.33646168	0.038	0.094	1	DCs
GNPDA2	0.00136062	0.38355281	0.063	0.132	1	DCs
RSAD21	0.00136177	0.50569094	0.114	0.206	1	DCs
HNRNPK	0.00136247	0.12647755	0.416	0.718	1	DCs
ATPAF2	0.00136815	0.51925231	0.038	0.094	1	DCs
UTP15	0.00139092	0.60969106	0.05	0.112	1	DCs
HPRT1	0.0013913	0.61989917	0.227	0.39	1	DCs
NFXL1	0.00141004	0.50113547	0.069	0.141	1	DCs
MMGT1	0.00141166	0.41319137	0.306	0.53	1	DCs
DARS	0.00143249	0.32443526	0.353	0.622	1	DCs
UBAC12	0.00144115	0.53651866	0.309	0.541	1	DCs
GDPD31	0.00145354	1.96125859	0.025	0.009	1	DCs
FLAD1	0.00145696	0.71359373	0.221	0.377	1	DCs
EWSR1	0.00146178	0.33715845	0.347	0.6	1	DCs
BRMS1L	0.0014724	0.52914547	0.028	0.079	1	DCs
ZSCAN22	0.00148853	0.40397151	0.025	0.074	1	DCs
NCAPD2	0.00149381	0.46975359	0.189	0.322	1	DCs
SPSB2	0.00149468	0.66005043	0.139	0.245	1	DCs
C12orf45	0.00149606	1.78448148	0.183	0.142	1	DCs

TERF1	0.00149748	0.58985206	0.129	0.233	1	DCs
STARD51	0.00149918	0.41558352	0.044	0.102	1	DCs
SYTL3	0.00150017	0.52553422	0.088	0.167	1	DCs
KCNE5	0.00150695	3.68529204	0.016	0.004	1	DCs
TIMM29	0.00150825	0.40387767	0.079	0.154	1	DCs
POC51	0.00151597	0.49671512	0.06	0.125	1	DCs
SEC61A2	0.00151966	2.48884669	0.047	0.022	1	DCs
WIPI21	0.00153103	0.35046782	0.353	0.608	1	DCs
ZNF441	0.00153704	0.27872259	0.063	0.13	1	DCs
FAAP100	0.00154338	0.66205313	0.088	0.169	1	DCs
NSA2	0.00154489	0.36731105	0.338	0.581	1	DCs
RAP2B2	0.00154836	1.2538397	0.492	0.642	1	DCs
MMS22L	0.00155311	0.61048136	0.079	0.155	1	DCs
PIK3C2A	0.00155818	0.79644538	0.123	0.221	1	DCs
RNF121	0.00155873	0.499077	0.104	0.192	1	DCs
ZMAT31	0.00156703	0.62118173	0.233	0.397	1	DCs
FASTKD11	0.00156861	0.74123171	0.123	0.221	1	DCs
NCBP2AS2	0.00157006	0.60766254	0.136	0.241	1	DCs
HERC3	0.00158649	0.83397501	0.521	0.676	1	DCs
NDUFS41	0.00159646	0.21956287	0.397	0.69	1	DCs
ZFP64	0.00159695	0.60007119	0.091	0.173	1	DCs
DNPH12	0.00159722	0.4033941	0.06	0.125	1	DCs
ANKRD55	0.00159937	3.46974367	0.038	0.016	1	DCs
URB1	0.00160483	0.76094529	0.095	0.177	1	DCs
HACL1	0.00160728	0.57195635	0.06	0.126	1	DCs
RNF40	0.00161064	0.22024486	0.404	0.701	1	DCs
POMT2	0.00161214	0.42372423	0.028	0.078	1	DCs
BMI1	0.00163003	1.28605771	0.338	0.348	1	DCs
PTPN22	0.00164173	0.55396947	0.315	0.551	1	DCs
LMBRD11	0.00164662	0.13682851	0.502	0.83	1	DCs
KLHL20	0.00165514	0.730751	0.155	0.268	1	DCs
ANKRD26	0.00166976	0.27423317	0.038	0.092	1	DCs
TMEM68	0.00167103	0.68132865	0.249	0.432	1	DCs
CCDC146	0.00167127	2.02428376	0.069	0.038	1	DCs
SYNJ1	0.00168089	0.58896279	0.129	0.229	1	DCs
ZNF160	0.00168228	0.51158241	0.123	0.218	1	DCs
CNST	0.00169715	0.55438646	0.252	0.431	1	DCs
NIPBL	0.00170091	0.79895015	0.508	0.661	1	DCs
CEP951	0.00170422	1.65290408	0.211	0.175	1	DCs
NFRKB	0.00171384	0.68672189	0.145	0.256	1	DCs
MON1A	0.00171453	0.59345506	0.073	0.144	1	DCs
C7orf31	0.00171667	0.55597247	0.073	0.144	1	DCs
ACSL5	0.00171735	0.40440388	0.344	0.595	1	DCs
MTR	0.00171914	0.44757827	0.287	0.492	1	DCs
LAMB22	0.00172538	0.56707181	0.101	0.187	1	DCs
PAK21	0.00174541	0.45210523	0.621	0.807	1	DCs
NUP205	0.00174547	0.47323036	0.265	0.449	1	DCs
PEX13	0.00174547	0.48595628	0.303	0.524	1	DCs
NPAT	0.00175785	0.57535692	0.11	0.2	1	DCs
ATPSCKMT	0.00177655	0.51340551	0.054	0.116	1	DCs
NUDT21	0.00178154	0.17416656	0.41	0.698	1	DCs
MTERF1	0.00178705	0.54098624	0.066	0.134	1	DCs

DRAM22	0.00178812	0.30882407	0.432	0.734	1	DCs
HDAC8	0.00179839	0.83017934	0.186	0.322	1	DCs
ARMC6	0.00180119	0.67616959	0.186	0.323	1	DCs
SLC45A3	0.00183305	0.2996159	0.032	0.082	1	DCs
KCNIP21	0.00183479	0.46919103	0.066	0.133	1	DCs
PPM1A	0.00183524	0.36099898	0.331	0.574	1	DCs
SMARCD21	0.00183715	1.12620736	0.454	0.57	1	DCs
PDS5B	0.00183906	0.59171329	0.151	0.263	1	DCs
SERAC1	0.00185111	0.5133596	0.066	0.134	1	DCs
CASP2	0.00185802	0.80338277	0.161	0.279	1	DCs
MSH6	0.00186395	0.60765955	0.158	0.274	1	DCs
RBFOX21	0.00187026	0.12859548	0.003	0.036	1	DCs
LEAP2	0.00187304	3.09688923	0.016	0.004	1	DCs
MTRES1	0.00187465	0.80712396	0.079	0.152	1	DCs
FANCL	0.00187528	0.69027633	0.085	0.161	1	DCs
DCLRE1A	0.00189035	0.24045753	0.016	0.057	1	DCs
CSRNP1	0.00189438	1.04421749	0.473	0.581	1	DCs
LINS1	0.00190312	0.62997148	0.183	0.313	1	DCs
BAG1	0.00190385	0.25562484	0.391	0.672	1	DCs
MARVELD2	0.00191987	3.36247171	0.016	0.004	1	DCs
MINDY2	0.00192198	0.66454184	0.139	0.243	1	DCs
MCM5	0.00192613	0.74886912	0.524	0.647	1	DCs
NPR2	0.00193037	3.01665116	0.016	0.004	1	DCs
VPS13A	0.00193038	0.21281578	0.063	0.127	1	DCs
GNPNAT1	0.00193164	0.3232565	0.069	0.137	1	DCs
SAP302	0.00193327	0.69876934	0.151	0.263	1	DCs
RCAN3	0.00193807	0.22843017	0.208	0.349	1	DCs
PIP4P22	0.00194666	2.30223703	0.085	0.051	1	DCs
ZNF816	0.00195299	0.10581133	0.035	0.086	1	DCs
PLEKHA3	0.00195359	0.47285243	0.24	0.408	1	DCs
CDK8	0.00195496	0.68096823	0.136	0.237	1	DCs
CARMIL31	0.00195529	3.0329545	0.013	0.003	1	DCs
ADSL1	0.00195944	0.20320606	0.401	0.682	1	DCs
ACTR1A	0.00196515	0.19231397	0.445	0.762	1	DCs
COPS2	0.00196529	0.41600337	0.312	0.54	1	DCs
ANKRD52	0.00196929	0.64488616	0.192	0.331	1	DCs
SLC66A31	0.00197495	0.28225525	0.385	0.661	1	DCs
TBC1D15	0.00197624	0.55081577	0.385	0.681	1	DCs
HNRNPA31	0.00198416	0.2458342	0.385	0.663	1	DCs
KLF43	0.00198633	0.7318899	0.653	0.858	1	DCs
UTP20	0.00199418	0.69225832	0.082	0.157	1	DCs
ZNF529	0.00200488	0.46930097	0.063	0.129	1	DCs
DDX55	0.0020119	0.65711425	0.117	0.21	1	DCs
FADS1	0.00203005	0.84958404	0.397	0.393	1	DCs
MORF4L1	0.00203922	0.48084112	0.227	0.386	1	DCs
FAM217B	0.00204747	0.60471687	0.11	0.2	1	DCs
BARD1	0.00204819	0.51288459	0.032	0.081	1	DCs
KANK12	0.00205927	2.9984863	0.013	0.003	1	DCs
RABGAP1L	0.00206998	0.87782208	0.517	0.677	1	DCs
STYXL1	0.00207061	0.70440174	0.18	0.309	1	DCs
CLDND11	0.00207095	1.11269066	0.423	0.502	1	DCs
EP400	0.00207583	0.4666127	0.066	0.132	1	DCs

FN3K1	0.00209461	0.23174088	0.044	0.1	1	DCs
ZDHHC2	0.00210652	0.35222427	0.382	0.647	1	DCs
DUS4L	0.00212244	0.27407424	0.05	0.109	1	DCs
VRK1	0.00212661	0.65186557	0.148	0.258	1	DCs
CLCN52	0.00213951	1.6471935	0.293	0.288	1	DCs
NANP	0.00214065	0.64884796	0.05	0.109	1	DCs
FAM91A12	0.00214782	0.38968292	0.334	0.575	1	DCs
CD831	0.0021518	1.40166906	0.675	0.872	1	DCs
POLR1D	0.00215631	0.93756441	0.521	0.7	1	DCs
TMX3	0.00215878	0.53759777	0.256	0.432	1	DCs
MYNN	0.00216073	0.69508072	0.158	0.274	1	DCs
CDAN1	0.00217912	0.1775742	0.038	0.09	1	DCs
TOX4	0.00217913	0.29259394	0.322	0.555	1	DCs
CABLES2	0.00218687	0.63610028	0.038	0.091	1	DCs
LPP1	0.00220432	0.85974157	0.514	0.684	1	DCs
BMS1	0.00221429	0.41207819	0.259	0.443	1	DCs
MYPOP	0.00222231	0.33606509	0.054	0.113	1	DCs
RNF216	0.00222674	0.44410419	0.088	0.165	1	DCs
PRR71	0.0022393	3.20309063	0.019	0.006	1	DCs
ELAC1	0.00224915	0.79951006	0.091	0.17	1	DCs
USP31	0.00225302	0.50023968	0.05	0.109	1	DCs
WDYHV1	0.00225754	0.58865678	0.073	0.141	1	DCs
TNFRSF10A1	0.00226881	0.4510384	0.063	0.127	1	DCs
MED23	0.00229328	0.52781426	0.205	0.346	1	DCs
APPL2	0.00230191	0.67993957	0.101	0.184	1	DCs
CAMK12	0.00230731	2.0689818	0.158	0.119	1	DCs
CNNM4	0.00230735	0.57349092	0.158	0.271	1	DCs
ACBD4	0.00231107	0.61198834	0.047	0.104	1	DCs
PDPR2	0.00232275	0.69258262	0.215	0.363	1	DCs
SLC35E41	0.00232529	2.70530354	0.063	0.034	1	DCs
CEACAM42	0.00233178	2.5309005	0.12	0.082	1	DCs
C20orf194	0.00235369	0.72790509	0.196	0.329	1	DCs
CHST7	0.00235457	0.82034144	0.047	0.103	1	DCs
SUCLA2	0.00236331	0.4675456	0.035	0.085	1	DCs
RINT1	0.00237919	0.584186	0.107	0.193	1	DCs
NEK7	0.0023911	0.24989525	0.391	0.684	1	DCs
SNX5	0.00239495	0.50468135	0.319	0.546	1	DCs
PPP1R13L	0.00240127	0.57619533	0.047	0.104	1	DCs
CFP2	0.00240169	1.74419652	0.287	0.274	1	DCs
GORAB	0.0024075	0.37660601	0.091	0.169	1	DCs
GGT7	0.00241541	0.23296425	0.038	0.09	1	DCs
FAM120A1	0.0024203	0.50181275	0.621	0.825	1	DCs
DCAF10	0.00242306	0.64625782	0.221	0.38	1	DCs
CUL7	0.00242632	0.40065723	0.06	0.122	1	DCs
C15orf41	0.00244328	0.2506361	0.028	0.075	1	DCs
SLX4	0.00245575	0.36853399	0.073	0.141	1	DCs
ELK1	0.00245757	0.78516847	0.095	0.175	1	DCs
PRMT5	0.00245963	0.26755919	0.041	0.094	1	DCs
RIPK1	0.00247035	0.39534247	0.328	0.567	1	DCs
ZFR	0.00248214	0.72641948	0.533	0.704	1	DCs
MTA3	0.00248771	0.55381699	0.079	0.15	1	DCs
TOP3B	0.0025093	0.62898445	0.11	0.197	1	DCs

NR4A22	0.00251058	1.30057287	0.435	0.488	1	DCs
TRAPPC12	0.00251176	0.29273	0.401	0.686	1	DCs
ATG14	0.00251518	0.62935344	0.151	0.259	1	DCs
RTL8A	0.00252495	0.58205084	0.028	0.075	1	DCs
SNRPF	0.00253818	0.20455613	0.041	0.094	1	DCs
ZNF600	0.00254111	0.30035837	0.047	0.103	1	DCs
WDR53	0.00254571	0.41519977	0.057	0.118	1	DCs
ANAPC10	0.00255425	0.45290557	0.022	0.065	1	DCs
HERC2	0.00255794	0.57020032	0.148	0.253	1	DCs
ZNF397	0.00256683	0.78869188	0.158	0.271	1	DCs
TMUB1	0.00258597	0.52542489	0.265	0.45	1	DCs
ECD	0.00258956	0.69975824	0.202	0.345	1	DCs
IRF31	0.00260249	1.1554169	0.483	0.619	1	DCs
OR2AE1	0.00260532	3.16988747	0.016	0.004	1	DCs
FRG1	0.00261668	0.74486363	0.17	0.293	1	DCs
MT-ND22	0.00262687	1.52430954	0.246	0.215	1	DCs
ASCC31	0.00262973	0.49850957	0.211	0.35	1	DCs
CDK2AP2	0.00263959	0.68680765	0.123	0.218	1	DCs
R3HDM1	0.00264249	0.70920404	0.192	0.327	1	DCs
MCM8	0.00264766	0.38314151	0.063	0.126	1	DCs
ZDHHC141	0.00264973	0.11701091	0.025	0.07	1	DCs
TRMT61A	0.00265522	0.69324102	0.091	0.169	1	DCs
GRK5	0.00265813	0.19961214	0.038	0.089	1	DCs
RALGPS1	0.0026582	0.40681854	0.041	0.093	1	DCs
MAP3K51	0.00266883	0.487045	0.353	0.605	1	DCs
IDH3G	0.00267756	0.20894857	0.445	0.754	1	DCs
KCTD13	0.00270188	0.75983	0.221	0.372	1	DCs
ZNF70	0.00270371	0.37862104	0.035	0.084	1	DCs
BAZ1A	0.00271119	0.46736316	0.309	0.526	1	DCs
ULK1	0.00271453	0.76814044	0.17	0.293	1	DCs
TTC17	0.00272104	0.66387252	0.265	0.453	1	DCs
OPRL11	0.00272549	0.17685233	0.028	0.074	1	DCs
OTULIN1	0.00272612	1.20842908	0.375	0.415	1	DCs
FRS3	0.00272732	0.30926406	0.041	0.093	1	DCs
DGKZ	0.0027309	0.63507673	0.158	0.267	1	DCs
EIF1AD	0.0027332	0.59297583	0.233	0.394	1	DCs
PLEK1	0.00273672	0.52462846	0.738	0.9	1	DCs
HSPBAP1	0.00275137	0.78307533	0.151	0.262	1	DCs
NSUN6	0.00275883	0.31254228	0.079	0.148	1	DCs
PPP4R3A	0.00276682	1.09030424	0.445	0.543	1	DCs
HNRNPA2B11	0.00277634	0.2847608	0.738	0.92	1	DCs
TRAM11	0.00278379	0.44324071	0.691	0.891	1	DCs
ZBTB11	0.00279102	0.48456742	0.218	0.358	1	DCs
FARS2	0.00279809	0.81229089	0.063	0.127	1	DCs
SPINT11	0.00280398	0.57189242	0.262	0.442	1	DCs
KIAA0355	0.00282326	0.65364684	0.142	0.246	1	DCs
FER1	0.00282434	0.40409431	0.268	0.447	1	DCs
TCFL5	0.00282442	0.97616398	0.038	0.089	1	DCs
MYO5A1	0.00282722	0.47024193	0.3	0.521	1	DCs
IP6K2	0.00282741	0.63242884	0.192	0.328	1	DCs
DTD1	0.00283554	0.69116798	0.215	0.365	1	DCs
PRKAR2A	0.00283803	0.8534361	0.256	0.436	1	DCs

AZIN21	0.00284555	2.18872636	0.022	0.008	1	DCs
HPF1	0.00286182	0.4410611	0.035	0.084	1	DCs
GNAI32	0.00286393	0.244628	0.445	0.762	1	DCs
B3GNT10	0.00286452	0.48231111	0.028	0.074	1	DCs
ZNF688	0.00287762	0.83484217	0.066	0.131	1	DCs
TBCE	0.00291229	0.40732126	0.06	0.12	1	DCs
NOC3L	0.00291742	0.87021979	0.136	0.237	1	DCs
SPATA2L	0.00292	0.40553636	0.066	0.13	1	DCs
ZNF24	0.00292354	0.45176753	0.319	0.544	1	DCs
SMOX1	0.00292673	2.88075284	0.041	0.019	1	DCs
ZNF426	0.00294206	0.6058652	0.139	0.238	1	DCs
ARL16	0.0029709	0.46497403	0.069	0.134	1	DCs
EIF3A1	0.00297489	0.41640373	0.675	0.875	1	DCs
MFSD6	0.00298117	0.48602936	0.356	0.618	1	DCs
ABCB7	0.0029859	0.53794628	0.252	0.423	1	DCs
ELF2	0.002998	0.62766345	0.246	0.422	1	DCs
FAF2	0.00300065	0.61244784	0.249	0.425	1	DCs
TRAPPC6B1	0.00301517	0.60944514	0.186	0.315	1	DCs
SRF	0.00302122	0.45539195	0.297	0.506	1	DCs
FUBP11	0.00305266	0.95111577	0.479	0.609	1	DCs
KIZ	0.0030538	0.78308738	0.085	0.158	1	DCs
PUS11	0.00309195	0.60622884	0.268	0.448	1	DCs
TENM41	0.00310491	0.53626131	0.066	0.129	1	DCs
ZNF566	0.00310848	0.60442402	0.076	0.144	1	DCs
PRMT6	0.00311834	0.64260446	0.079	0.149	1	DCs
MYC2	0.00313707	0.15020766	0.073	0.138	1	DCs
MSL2	0.00315657	0.83153162	0.502	0.646	1	DCs
ZNF496	0.00315901	0.74845147	0.06	0.12	1	DCs
DAP	0.0031669	0.37984093	0.369	0.641	1	DCs
SYAP1	0.00319849	0.13448266	0.479	0.786	1	DCs
PABPC41	0.00320333	0.99337003	0.457	0.57	1	DCs
SEMA4B2	0.00321755	0.31772377	0.05	0.106	1	DCs
PMM21	0.00322404	0.60034666	0.215	0.36	1	DCs
HES6	0.00323047	0.14352622	0.025	0.068	1	DCs
DNAJC9	0.00324181	0.27344888	0.047	0.101	1	DCs
ZNF283	0.00329662	0.70935745	0.091	0.167	1	DCs
FLOT1	0.00330847	0.40695377	0.688	0.857	1	DCs
ABTB2	0.00330916	3.84656706	0.025	0.009	1	DCs
RMND5A	0.00331194	0.27766534	0.379	0.638	1	DCs
ZDHHC161	0.00331281	0.59500232	0.177	0.3	1	DCs
DPH5	0.00331845	0.87245696	0.123	0.215	1	DCs
TOR3A	0.00333457	0.69686934	0.262	0.445	1	DCs
SEC31B	0.00333527	1.71008659	0.095	0.06	1	DCs
HGS1	0.00334831	0.28319225	0.41	0.699	1	DCs
CAMTA1	0.00336194	0.67585683	0.284	0.491	1	DCs
ATXN2	0.00337265	0.58388775	0.24	0.4	1	DCs
LARP4B	0.0033827	0.4012866	0.334	0.574	1	DCs
FBRSL12	0.00338553	1.61000315	0.319	0.33	1	DCs
TMLHE	0.00338687	0.70505122	0.221	0.375	1	DCs
MAFF1	0.00340575	0.96099916	0.199	0.33	1	DCs
ASMTL1	0.00340639	1.98956112	0.17	0.135	1	DCs
NIPAL2	0.00344613	2.05984989	0.208	0.18	1	DCs

F2RL11	0.00345954	3.40385012	0.016	0.005	1	DCs
TOP2B	0.00347899	0.61898486	0.189	0.318	1	DCs
BCL2L12	0.00348067	0.75289254	0.091	0.168	1	DCs
TBL1X	0.00348157	0.59252035	0.183	0.307	1	DCs
SETD6	0.0034858	0.56368285	0.085	0.156	1	DCs
MLH3	0.00348918	0.50516738	0.063	0.124	1	DCs
RAB11FIP3	0.00350258	0.59407981	0.076	0.142	1	DCs
PIM12	0.00350753	1.20125733	0.483	0.564	1	DCs
AKAP17A1	0.00351316	1.08276858	0.416	0.486	1	DCs
C2CD2	0.00352167	0.59485792	0.06	0.119	1	DCs
KDM4A	0.00352647	0.58616761	0.24	0.403	1	DCs
QRSL1	0.00352731	0.8386314	0.063	0.125	1	DCs
JRKL	0.00354847	0.61417884	0.076	0.142	1	DCs
BBIP1	0.00355055	0.56437283	0.066	0.129	1	DCs
SHPRH	0.00356025	0.43560748	0.145	0.244	1	DCs
ZFP30	0.00358882	1.00098742	0.114	0.198	1	DCs
FGD3	0.00359086	0.46581979	0.284	0.478	1	DCs
GRAMD1A1	0.00359141	1.43683291	0.372	0.419	1	DCs
POLH	0.00359206	0.50459016	0.095	0.17	1	DCs
ELOVL71	0.00359635	0.2291294	0.028	0.073	1	DCs
FAM169A	0.00361474	2.6500457	0.016	0.005	1	DCs
ZNF546	0.00361646	0.46214064	0.035	0.082	1	DCs
ELP2	0.00365369	1.14223399	0.407	0.489	1	DCs
RASSF7	0.00366192	0.53305945	0.123	0.213	1	DCs
PPP2R1A	0.00366201	0.48129295	0.05	0.106	1	DCs
C11orf21	0.00367829	0.56766608	0.035	0.082	1	DCs
DNAJC1	0.00370402	0.58018079	0.224	0.37	1	DCs
PKP2	0.003707	0.79034027	0.066	0.128	1	DCs
TGDS	0.00374436	0.92500956	0.082	0.153	1	DCs
PRR141	0.0037457	1.331132	0.372	0.424	1	DCs
DPH7	0.00374758	0.7746549	0.129	0.224	1	DCs
EXOSC2	0.00375029	0.68804124	0.123	0.212	1	DCs
MTX3	0.00376305	0.45137209	0.104	0.184	1	DCs
GTPBP41	0.00376732	0.69128704	0.259	0.437	1	DCs
EEF1AKMT41	0.00377926	0.37110903	0.063	0.123	1	DCs
MAP2K5	0.00378667	0.61331969	0.126	0.216	1	DCs
PRRC2B	0.00381416	0.50597696	0.265	0.446	1	DCs
CPPED13	0.00381962	0.21183478	0.442	0.74	1	DCs
CIZ1	0.00383231	0.68844705	0.158	0.269	1	DCs
RRAGC2	0.00383972	0.55297355	0.312	0.529	1	DCs
PACS2	0.00384233	0.55219846	0.297	0.508	1	DCs
PEAK32	0.00384609	2.47030111	0.063	0.035	1	DCs
TRPM42	0.0038479	0.9144537	0.142	0.242	1	DCs
GLI4	0.00385879	0.49884942	0.054	0.11	1	DCs
PPP1R3F	0.00386441	0.43383103	0.022	0.063	1	DCs
ARID1B	0.00387574	0.51258199	0.202	0.335	1	DCs
DEPP11	0.00387602	4.01557392	0.016	0.005	1	DCs
IBA57	0.00387866	0.26006072	0.041	0.091	1	DCs
GTF2I	0.00388297	0.56475674	0.256	0.429	1	DCs
ZGPAT	0.00389961	0.31365432	0.353	0.601	1	DCs
TFCP21	0.00390495	0.99317197	0.451	0.56	1	DCs
WDR83	0.00393176	0.32873118	0.054	0.109	1	DCs

SRP72	0.0039408	0.39750362	0.372	0.641	1	DCs
SMPDL3A2	0.00395227	0.25858047	0.006	0.037	1	DCs
ZNF14	0.00396262	0.64953889	0.063	0.123	1	DCs
WDFY22	0.00397645	1.28880451	0.363	0.394	1	DCs
TYW1	0.00402204	0.65733717	0.035	0.081	1	DCs
FRMD4B1	0.00402421	1.59031158	0.278	0.271	1	DCs
GPATCH8	0.00403153	0.71103122	0.237	0.399	1	DCs
POLG	0.00404187	0.84706729	0.189	0.317	1	DCs
FOXRED21	0.00404726	0.5229841	0.047	0.1	1	DCs
TXNL4B	0.00405152	0.76399984	0.079	0.145	1	DCs
PDS5A	0.00406132	0.79032166	0.199	0.335	1	DCs
PRMT3	0.00408488	0.58997178	0.101	0.179	1	DCs
TMEM39A	0.00408691	0.88773916	0.164	0.277	1	DCs
CCDC96	0.00413525	0.30139948	0.013	0.047	1	DCs
NSD12	0.00414843	0.8862477	0.461	0.582	1	DCs
FBXL17	0.00417422	0.43355291	0.334	0.565	1	DCs
ST3GAL3	0.00419246	0.80935817	0.095	0.17	1	DCs
CCDC117	0.00419963	0.75238541	0.167	0.282	1	DCs
RAB39A2	0.00420859	0.74173468	0.132	0.225	1	DCs
TBXAS11	0.00422001	0.51184483	0.322	0.545	1	DCs
PDLIM72	0.0042401	1.16998835	0.476	0.595	1	DCs
MEGF8	0.00426466	1.01799746	0.063	0.123	1	DCs
SELENOO	0.00426486	0.47252455	0.211	0.355	1	DCs
LRCH1	0.00427579	0.7803373	0.192	0.325	1	DCs
ZFP82	0.00428635	0.61482274	0.06	0.117	1	DCs
MED13	0.00430412	0.59960125	0.281	0.473	1	DCs
MSLN	0.00431046	3.41879734	0.019	0.006	1	DCs
RNF166	0.00433868	0.68778764	0.262	0.44	1	DCs
POLR3D	0.00434036	0.57916282	0.12	0.207	1	DCs
PRIM2	0.00438674	0.57642324	0.082	0.151	1	DCs
ZNF148	0.00438988	0.40873713	0.322	0.549	1	DCs
FZD5	0.00439222	0.71282399	0.057	0.113	1	DCs
ZNF92	0.00440283	0.78330806	0.114	0.197	1	DCs
RUFY2	0.00443311	0.69290062	0.155	0.259	1	DCs
UBL71	0.00443628	0.27530911	0.413	0.697	1	DCs
PPAN2	0.00446127	0.69353105	0.221	0.369	1	DCs
RFX22	0.00448105	1.0701511	0.088	0.16	1	DCs
FANCM	0.00448521	0.11588927	0.022	0.062	1	DCs
SNX292	0.00448616	0.78711203	0.174	0.288	1	DCs
TOMM34	0.00449309	1.99565589	0.221	0.196	1	DCs
GOLIM41	0.00449366	0.20114336	0.486	0.794	1	DCs
PAXBP1	0.00449446	0.6400155	0.167	0.28	1	DCs
TRAPPC9	0.00450021	0.57301945	0.262	0.438	1	DCs
CLNS1A	0.00450265	1.22287871	0.385	0.448	1	DCs
MAD2L2	0.00452776	0.78634612	0.199	0.33	1	DCs
LRP10	0.0045342	0.20067085	0.454	0.769	1	DCs
DAZAP1	0.00453982	0.72845695	0.259	0.435	1	DCs
METTL3	0.00454608	0.55376653	0.259	0.429	1	DCs
NUP210	0.00457638	0.54420367	0.293	0.491	1	DCs
SCAF4	0.00461218	0.61657482	0.274	0.465	1	DCs
AIM2	0.00461339	2.19752397	0.104	0.069	1	DCs
AS3MT1	0.00463572	3.40000988	0.016	0.005	1	DCs

C17orf58	0.00464038	0.40721481	0.044	0.094	1	DCs
TASP1	0.00465663	0.53221002	0.044	0.095	1	DCs
BCOR	0.00466318	1.29965846	0.363	0.405	1	DCs
COX20	0.004665	0.61542026	0.114	0.195	1	DCs
RNF122	0.0046755	0.31969463	0.044	0.094	1	DCs
TXNDC2	0.00467966	4.11747994	0.013	0.003	1	DCs
MYO9A	0.00468538	1.58304787	0.293	0.294	1	DCs
ITGB1BP21	0.0046939	2.76870227	0.016	0.005	1	DCs
SCAMP5	0.00470532	0.9303008	0.006	0.036	1	DCs
ZNF836	0.0047064	0.28696987	0.041	0.09	1	DCs
NUDT17	0.00471594	0.25241812	0.016	0.052	1	DCs
IMMP2L	0.0047202	0.74782341	0.142	0.236	1	DCs
CLPX1	0.00472596	0.74142837	0.224	0.375	1	DCs
XXYLT12	0.00474375	0.51926216	0.066	0.127	1	DCs
SUPV3L1	0.00475352	0.83612637	0.126	0.216	1	DCs
PML1	0.00475691	0.37484826	0.426	0.71	1	DCs
C12orf66	0.00479514	0.48126044	0.076	0.139	1	DCs
RSPRY1	0.00482597	0.68784707	0.205	0.343	1	DCs
CXorf40A	0.00483041	0.51258417	0.123	0.209	1	DCs
MVB12B	0.00484433	0.58976504	0.271	0.446	1	DCs
EZH2	0.00485848	1.58816955	0.151	0.114	1	DCs
ATM1	0.00486763	0.7821513	0.495	0.632	1	DCs
UPF3B	0.00487166	0.49931323	0.076	0.139	1	DCs
STXBP5	0.00487316	0.87386945	0.189	0.319	1	DCs
CEP97	0.00487929	0.12485486	0.035	0.08	1	DCs
HLCS	0.00490469	0.85198397	0.11	0.193	1	DCs
TRMT12	0.00497161	0.43061101	0.041	0.089	1	DCs
PRELID2	0.00497727	0.25857725	0.035	0.08	1	DCs
ANP32E	0.00500028	0.50401969	0.151	0.251	1	DCs
GID4	0.00501703	0.97652779	0.126	0.216	1	DCs
RAPH12	0.00503125	2.03332907	0.199	0.169	1	DCs
RECK	0.00504068	0.53919668	0.032	0.075	1	DCs
ARHGEF3	0.00505428	0.44624954	0.322	0.546	1	DCs
ZNF582	0.00508609	0.28597458	0.028	0.07	1	DCs
IBTK	0.00508809	0.55156533	0.341	0.583	1	DCs
FES1	0.00510702	0.50156808	0.319	0.529	1	DCs
POLR3F	0.00514917	0.87645815	0.107	0.187	1	DCs
MYL51	0.00523694	0.49028283	0.038	0.085	1	DCs
CREBZF	0.00526768	1.28247093	0.338	0.354	1	DCs
N6AMT1	0.00527788	0.51118904	0.041	0.089	1	DCs
TULP3	0.00532194	0.48728498	0.038	0.085	1	DCs
PLEKHM3	0.00532332	2.34368749	0.24	0.222	1	DCs
TRIP41	0.00532412	0.61284312	0.249	0.413	1	DCs
IAH11	0.00534292	1.06014782	0.41	0.476	1	DCs
UQCRHL	0.00534782	3.19642656	0.013	0.003	1	DCs
LRIG1	0.00535212	1.94378723	0.069	0.041	1	DCs
DYNLT1	0.00535842	0.19494173	0.407	0.692	1	DCs
GEMIN2	0.00536823	0.49617051	0.022	0.061	1	DCs
SPATA1	0.00539267	3.07607727	0.019	0.006	1	DCs
FIGNL1	0.00540849	0.39806428	0.041	0.088	1	DCs
HEXD1	0.00542957	0.47249668	0.158	0.259	1	DCs
GTF2IRD11	0.00543787	0.53479149	0.047	0.098	1	DCs

ALDH8A1	0.0054591	2.31715921	0.016	0.005	1	DCs
AASDH	0.00546517	0.75753999	0.129	0.217	1	DCs
ARHGEF21	0.00549083	0.5574425	0.65	0.857	1	DCs
ACYP1	0.00551714	0.39359921	0.06	0.115	1	DCs
PPP6R3	0.00554254	0.55322656	0.309	0.52	1	DCs
PCIF1	0.00556265	0.56549154	0.262	0.439	1	DCs
IL18BP1	0.00557222	0.61606117	0.155	0.253	1	DCs
EPB41	0.00557481	1.38001441	0.278	0.271	1	DCs
SLC12A2	0.00557487	0.69832787	0.085	0.153	1	DCs
NKAPD1	0.00561899	0.54234802	0.205	0.335	1	DCs
XAF11	0.005622	0.99960749	0.511	0.68	1	DCs
CALHM22	0.00564027	0.29336109	0.36	0.582	1	DCs
ZNF18	0.00564127	0.5836366	0.085	0.152	1	DCs
ZNF5711	0.00564937	0.38310072	0.044	0.093	1	DCs
GPATCH2L1	0.00566943	0.73357891	0.495	0.634	1	DCs
FOXK1	0.00567123	0.56407354	0.243	0.399	1	DCs
SIVA1	0.00568023	0.31547545	0.369	0.623	1	DCs
UBE2O	0.00568678	0.67522422	0.189	0.312	1	DCs
GDAP2	0.00570772	0.59266242	0.325	0.541	1	DCs
GZF11	0.00574717	1.0234371	0.17	0.285	1	DCs
CACUL11	0.0057486	0.2930129	0.442	0.74	1	DCs
ZSCAN26	0.00577111	0.77021248	0.101	0.177	1	DCs
ANKHD11	0.00579048	0.67535178	0.524	0.711	1	DCs
RALGAPA1	0.00579737	0.50997885	0.082	0.148	1	DCs
BUD23	0.00579894	0.54178583	0.322	0.546	1	DCs
TMOD21	0.00580159	0.90700578	0.117	0.201	1	DCs
RGS131	0.00581889	2.74593373	0.022	0.008	1	DCs
SSPN1	0.00582284	1.08705729	0.05	0.103	1	DCs
BMP11	0.00583441	0.67051526	0.073	0.135	1	DCs
TUBB62	0.00584021	0.50912274	0.319	0.53	1	DCs
NUP622	0.00584402	1.22086478	0.445	0.572	1	DCs
BCR	0.00587897	0.60452909	0.129	0.218	1	DCs
RFC5	0.00588917	0.35490253	0.032	0.074	1	DCs
ZNF671	0.00589925	0.83156236	0.129	0.218	1	DCs
RBM12	0.00590954	0.59497434	0.167	0.278	1	DCs
BORA	0.00592209	0.61206011	0.063	0.121	1	DCs
TESK2	0.00593473	0.58199784	0.06	0.115	1	DCs
INPP5E	0.00593893	0.59768353	0.12	0.204	1	DCs
GFOD1	0.00594144	0.77353774	0.085	0.153	1	DCs
CBL	0.00600001	0.91640132	0.511	0.68	1	DCs
SLFN12	0.00600154	0.71035941	0.069	0.13	1	DCs
ARMCX6	0.0060558	0.35904692	0.047	0.096	1	DCs
ZNF473	0.00608952	0.2775214	0.032	0.074	1	DCs
MX21	0.00611643	1.44329634	0.41	0.481	1	DCs
ROCK1	0.00611804	0.56802831	0.584	0.785	1	DCs
PRXL2C1	0.00613261	0.58117224	0.293	0.499	1	DCs
CLTB1	0.00613819	0.58263655	0.331	0.564	1	DCs
RAPGEF11	0.00616313	0.71165204	0.571	0.78	1	DCs
TERF2IP	0.00617578	0.27260552	0.41	0.683	1	DCs
MIER1	0.00618351	0.38310466	0.363	0.616	1	DCs
CTBP21	0.00619868	0.71473715	0.562	0.771	1	DCs
DTWD1	0.00622553	0.99091683	0.073	0.134	1	DCs

C15orf61	0.00622805	0.63949795	0.079	0.144	1	DCs
NSMCE4A	0.00626097	0.68172577	0.224	0.371	1	DCs
RAC3	0.00626172	0.71344042	0.032	0.074	1	DCs
CDC5L	0.00626281	0.52514478	0.319	0.54	1	DCs
HPS31	0.00626857	0.65453812	0.249	0.41	1	DCs
PPP1R322	0.0062929	2.29550973	0.019	0.007	1	DCs
PHF12	0.00634033	1.20307258	0.382	0.439	1	DCs
TRIM28	0.00636318	0.62117213	0.183	0.3	1	DCs
SUPT6H	0.00637925	0.23605113	0.401	0.679	1	DCs
DDX31	0.00641441	0.6107415	0.123	0.206	1	DCs
NOA1	0.00641985	0.80665215	0.221	0.37	1	DCs
AP4B1	0.00645303	0.74998118	0.129	0.218	1	DCs
TRNT1	0.00646286	0.5383395	0.161	0.264	1	DCs
AKAP9	0.00647007	0.35428259	0.382	0.647	1	DCs
XPNPEP3	0.00652773	0.61087796	0.047	0.096	1	DCs
PEAK11	0.00655619	0.79218293	0.129	0.216	1	DCs
NAA10	0.00661532	0.41762101	0.303	0.502	1	DCs
SLC25A322	0.00663914	0.7640683	0.215	0.355	1	DCs
PIWIL42	0.00670748	2.16448408	0.085	0.055	1	DCs
DXO	0.00672248	0.32399389	0.044	0.092	1	DCs
LRRC271	0.00674935	0.36786929	0.044	0.092	1	DCs
IGF2BP3	0.00679429	0.11808817	0.013	0.045	1	DCs
UBE3A1	0.0067948	0.84917218	0.492	0.647	1	DCs
N4BP12	0.00681921	0.83095627	0.539	0.713	1	DCs
DDHD2	0.00684806	0.74524381	0.155	0.253	1	DCs
CREB3L4	0.00686331	0.17958955	0.019	0.054	1	DCs
CHURC1	0.00686965	0.11185855	0.489	0.79	1	DCs
CCDC18	0.00687283	0.75675858	0.091	0.16	1	DCs
FHDC1	0.00687872	3.5889017	0.013	0.004	1	DCs
EPDR1	0.00688037	0.62985673	0.019	0.054	1	DCs
TAF2	0.0068832	0.66972702	0.177	0.292	1	DCs
NFATC2IP	0.00690851	0.47025367	0.287	0.463	1	DCs
KLHL2	0.00691111	0.39092231	0.372	0.64	1	DCs
EPB41L21	0.00691379	1.4988012	0.3	0.308	1	DCs
MPZ	0.00692138	0.3846142	0.028	0.068	1	DCs
CEP192	0.00693383	0.54356683	0.186	0.3	1	DCs
PALB2	0.00693714	0.78692695	0.088	0.155	1	DCs
IFT20	0.00696424	1.17470899	0.404	0.486	1	DCs
OBSCN	0.00697435	0.85605144	0.041	0.02	1	DCs
PCGF6	0.0069993	0.73766291	0.041	0.087	1	DCs
SCAF8	0.0070004	0.53713726	0.303	0.516	1	DCs
ATF7IP2	0.00701872	0.81478538	0.06	0.114	1	DCs
CRLF3	0.00703209	1.18494055	0.375	0.429	1	DCs
CCNH	0.00705693	0.72384857	0.237	0.388	1	DCs
PGS11	0.00708398	0.53104535	0.218	0.352	1	DCs
HEMK1	0.00712229	0.57604313	0.136	0.225	1	DCs
TMEM176B2	0.00721739	1.49856292	0.366	0.382	1	DCs
MEX3B2	0.00724504	2.25196277	0.079	0.05	1	DCs
MEST	0.00724587	3.14440825	0.016	0.005	1	DCs
ZBTB25	0.00725798	0.44471755	0.227	0.367	1	DCs
TTF2	0.00726269	0.771819	0.177	0.29	1	DCs
SF3B1	0.00727902	0.67938832	0.262	0.434	1	DCs

SLC25A251	0.00728462	0.49167535	0.325	0.535	1	DCs
ZNF343	0.00731423	0.23008868	0.041	0.087	1	DCs
APBB31	0.00731766	0.57637337	0.107	0.181	1	DCs
OR52N4	0.00731774	2.82697309	0.013	0.004	1	DCs
THOC2	0.00734175	0.72046229	0.167	0.274	1	DCs
WDR70	0.00738372	0.72534391	0.161	0.264	1	DCs
TIMM17B1	0.00740284	0.57149887	0.145	0.24	1	DCs
ZNF181	0.00741835	0.24586275	0.022	0.059	1	DCs
OAZ31	0.00742574	0.14412386	0.009	0.039	1	DCs
BRF1	0.00742654	0.665446	0.148	0.244	1	DCs
ERCC1	0.00746447	0.61235612	0.315	0.528	1	DCs
HMGN1	0.00747962	0.70737881	0.53	0.702	1	DCs
HERC4	0.00752243	0.79213155	0.429	0.516	1	DCs
TMEM41A	0.00755328	0.91546283	0.079	0.141	1	DCs
PAAF1	0.00758826	0.82158846	0.107	0.184	1	DCs
DDX27	0.00759286	0.86427668	0.47	0.606	1	DCs
XIAP	0.00759735	0.62177903	0.237	0.389	1	DCs
METTL25	0.00760452	0.64973943	0.104	0.178	1	DCs
OSGEP	0.00761518	0.8582899	0.196	0.323	1	DCs
IPO91	0.00762048	0.68754249	0.259	0.424	1	DCs
TPM42	0.00763989	0.16155913	0.539	0.824	1	DCs
FCRL6	0.00770293	2.12137573	0.069	0.042	1	DCs
TIFA	0.00771075	0.77992767	0.091	0.16	1	DCs
ZNF84	0.00772173	0.62285522	0.091	0.16	1	DCs
PRPF4B	0.00772671	0.55086882	0.3	0.499	1	DCs
ZNF136	0.00775852	0.61148921	0.091	0.158	1	DCs
COMMD2	0.00778335	0.72134587	0.265	0.441	1	DCs
CHSY12	0.00783664	0.72620614	0.224	0.365	1	DCs
UBE3C	0.00784273	0.40868268	0.356	0.598	1	DCs
HIPK1	0.00787222	0.70641602	0.517	0.694	1	DCs
MPHOSPH10	0.00789125	0.68453849	0.192	0.317	1	DCs
PBX3	0.00796056	0.49911225	0.363	0.606	1	DCs
NUMB1	0.00796223	0.22967541	0.479	0.779	1	DCs
TMEM182	0.00799321	0.45843054	0.028	0.068	1	DCs
TRIP11	0.00799903	0.77532832	0.174	0.284	1	DCs
URI1	0.00804425	0.3600967	0.363	0.604	1	DCs
PTGER31	0.00804736	1.43736757	0.278	0.264	1	DCs
ZBTB2	0.00804903	0.6084381	0.155	0.254	1	DCs
MT-ND52	0.00805155	1.09153291	0.29	0.279	1	DCs
ANK3	0.00811624	1.62809224	0.035	0.016	1	DCs
SLC2A11	0.00812316	0.39001475	0.035	0.077	1	DCs
MRGBP	0.00812644	0.70109207	0.142	0.234	1	DCs
KLHL26	0.00813488	0.58389842	0.044	0.091	1	DCs
GCC1	0.00813514	0.83402604	0.174	0.284	1	DCs
PPIP5K1	0.00813903	0.67299507	0.066	0.122	1	DCs
SRSF4	0.00818902	0.67966129	0.549	0.744	1	DCs
C10orf88	0.00819864	0.5644402	0.05	0.1	1	DCs
ZNF140	0.00820241	0.69125321	0.073	0.131	1	DCs
DNASE1L13	0.00823618	0.3724548	0.404	0.687	1	DCs
LRRC47	0.00824913	0.45504578	0.35	0.588	1	DCs
PCNX31	0.00825315	0.82463942	0.18	0.296	1	DCs
SMG9	0.00825331	0.96071089	0.151	0.249	1	DCs

RPA2	0.00825822	0.73768865	0.192	0.317	1	DCs
NLGN21	0.00827599	2.66937069	0.016	0.005	1	DCs
ESRRA1	0.0082912	0.5540196	0.341	0.577	1	DCs
CDC42BPA	0.00829584	0.89936574	0.063	0.118	1	DCs
CD402	0.00830402	0.80462607	0.379	0.639	1	DCs
RRNAD11	0.00831014	0.69961402	0.117	0.196	1	DCs
UFL1	0.00831027	0.63834355	0.24	0.39	1	DCs
LFNG2	0.00833066	1.02774354	0.42	0.501	1	DCs
LSM14A	0.00833443	0.71792711	0.233	0.386	1	DCs
ADCY41	0.00834242	2.39385187	0.047	0.025	1	DCs
ZC3H18	0.00834349	0.60175152	0.315	0.535	1	DCs
PPIH	0.00834637	0.69390332	0.091	0.159	1	DCs
HELB1	0.00838917	1.25740878	0.151	0.117	1	DCs
EEF1AKMT1	0.0083935	0.51268123	0.019	0.053	1	DCs
CYB5RL	0.00840403	0.37998509	0.041	0.086	1	DCs
TOPORS	0.00840976	0.93283195	0.136	0.223	1	DCs
ZNF132	0.00841654	0.57826797	0.022	0.058	1	DCs
TBCB	0.00845781	0.55883394	0.587	0.787	1	DCs
TNFRSF1B2	0.00849144	1.35708778	0.379	0.441	1	DCs
DAZAP22	0.00849628	0.44676986	0.615	0.842	1	DCs
INTU	0.00850207	0.165543	0.025	0.062	1	DCs
ALKBH8	0.00858273	0.51673064	0.047	0.094	1	DCs
NDST21	0.00858927	1.64495446	0.142	0.109	1	DCs
HCN3	0.00860137	2.43020168	0.054	0.03	1	DCs
ZNF260	0.00860702	0.98978829	0.085	0.15	1	DCs
MTIF3	0.00863775	0.42881996	0.369	0.61	1	DCs
TLK2	0.00864952	0.61462808	0.174	0.28	1	DCs
FAM13B	0.00868852	0.52522173	0.278	0.455	1	DCs
LRR1	0.00869906	0.69402504	0.107	0.181	1	DCs
PSMD5	0.00870213	0.31973995	0.022	0.057	1	DCs
CEP78	0.00870913	0.69656848	0.098	0.166	1	DCs
PROSER3	0.00876034	0.52457114	0.091	0.157	1	DCs
ANKDD1B	0.00877367	3.34034548	0.013	0.004	1	DCs
ZBTB37	0.00878405	0.6724658	0.142	0.233	1	DCs
MTA2	0.00879613	0.43008136	0.388	0.644	1	DCs
MAPKBP1	0.00882293	0.78549122	0.123	0.205	1	DCs
MAP3K111	0.00885028	0.2556002	0.448	0.74	1	DCs
LRRC691	0.00888059	2.48924918	0.057	0.032	1	DCs
MTBP	0.00889625	0.75522476	0.013	0.043	1	DCs
ABCA7	0.00890336	0.50296102	0.297	0.489	1	DCs
WDR20	0.00892768	0.74174134	0.177	0.29	1	DCs
C14orf28	0.00893302	0.55764798	0.038	0.08	1	DCs
AC073111.4	0.00898715	2.14200335	0.06	0.035	1	DCs
ZNF2051	0.00898761	0.56990108	0.054	0.103	1	DCs
CRTC1	0.0089973	0.61238766	0.041	0.085	1	DCs
MPP5	0.00900787	0.78425565	0.117	0.195	1	DCs
WDPCP	0.00904014	0.56338144	0.057	0.107	1	DCs
ABR2	0.00904047	0.35914652	0.691	0.888	1	DCs
SPAG16	0.00911324	0.61835556	0.041	0.085	1	DCs
PKN1	0.00912171	0.20244768	0.464	0.762	1	DCs
FXR1	0.00912546	0.62680653	0.208	0.34	1	DCs
RIPK22	0.00914555	0.62105833	0.347	0.586	1	DCs

LPAR12	0.00920117	0.97475446	0.148	0.242	1	DCs
LRRC14	0.00921311	0.95586233	0.161	0.264	1	DCs
GSTM1	0.00921883	0.34784933	0.009	0.038	1	DCs
KCNJ14	0.00924048	2.36483031	0.028	0.012	1	DCs
GPS2	0.00924091	0.39251096	0.36	0.614	1	DCs
SENP71	0.00925138	1.68838831	0.145	0.113	1	DCs
UBTD2	0.00927943	0.83341142	0.265	0.433	1	DCs
FAM53B	0.00931291	0.68057961	0.262	0.426	1	DCs
TUBD1	0.00934138	0.82605034	0.085	0.149	1	DCs
ZNF6972	0.00935098	0.11313614	0.032	0.071	1	DCs
CEP19	0.00938847	0.52647784	0.022	0.057	1	DCs
RBM23	0.0094644	0.26329059	0.432	0.723	1	DCs
RAB3IL12	0.00951393	2.50897051	0.016	0.005	1	DCs
C15orf39	0.00960036	0.97989783	0.445	0.557	1	DCs
SGTB2	0.00961149	0.54804848	0.126	0.207	1	DCs
BOD1L11	0.00964358	0.79526449	0.196	0.32	1	DCs
TEN1	0.00966538	0.96456805	0.177	0.286	1	DCs
TOMM20	0.00972102	0.2591633	0.442	0.74	1	DCs
ALG13	0.00972571	0.91496494	0.448	0.554	1	DCs
ILRUN	0.00973673	0.20445068	0.435	0.738	1	DCs
ILK1	0.00974104	0.6199071	0.309	0.516	1	DCs
LINC02693	0.00974355	0.25420616	0.013	0.042	1	DCs
C1orf112	0.00974995	0.76023345	0.082	0.142	1	DCs
SPSB3	0.00975899	1.22387555	0.379	0.44	1	DCs
TMEM267	0.00979317	0.66945228	0.098	0.167	1	DCs
IPO5	0.00979636	0.79113099	0.139	0.227	1	DCs
ANKRA2	0.00983844	0.97417994	0.085	0.148	1	DCs
AFG1L	0.00993942	0.82277928	0.063	0.115	1	DCs
GLYCTK	0.00994573	0.62106499	0.177	0.283	1	DCs
GUF1	0.00997108	0.92642594	0.12	0.2	1	DCs
SUSD3	0.00997836	1.76601197	0.218	0.198	1	DCs
PLD42	0	10.3236687	0.757	0.003	0	pDCs
CBFA2T33	0	7.86619281	0.757	0.015	0	pDCs
IRF42	0	8.00433154	0.73	0.012	0	pDCs
IGKC1	0	8.17917433	0.541	0.001	0	pDCs
JCHAIN	0	11.7548013	0.541	0.001	0	pDCs
PTPRS	0	11.3779835	0.514	0.002	0	pDCs
SULF23	0	7.67854031	0.514	0.005	0	pDCs
C12orf751	0	6.06590015	0.514	0.006	0	pDCs
CLIC31	0	8.40512691	0.486	0.003	0	pDCs
MZB1	0	9.74794092	0.459	0	0	pDCs
GZMB	0	7.46372096	0.432	0.004	0	pDCs
FLT32	0	8.3824596	0.405	0.001	0	pDCs
AFF32	0	8.48576712	0.405	0.002	0	pDCs
CLEC4C1	0	11.3777506	0.351	0	0	pDCs
LILRA42	0	10.8188388	0.351	0.002	0	pDCs
PPP1R14A2	0	9.47776534	0.324	0.001	0	pDCs
KCNK171	0	11.3512128	0.297	0	0	pDCs
DNASE1L31	0	7.92621077	0.297	0.002	0	pDCs
PACSIN1	0	10.4912172	0.27	0	0	pDCs
PLVAP1	0	10.889815	0.27	0	0	pDCs
IGHM	0	7.38285594	0.27	0.001	0	pDCs

PROC2	0	10.4785103	0.243	0	0	pDCs
SPIB1	0	9.92198603	0.243	0	0	pDCs
RAMP12	0	8.08708493	0.243	0.001	0	pDCs
PTK7	0	8.91940206	0.216	0	0	pDCs
PLXNA41	0	8.92586919	0.216	0.001	0	pDCs
EGLN31	0	8.73499791	0.216	0.001	0	pDCs
LTK	0	7.21516363	0.216	0.001	0	pDCs
VASH2	0	13.2563458	0.189	0	0	pDCs
SLC12A31	0	11.1205384	0.162	0	0	pDCs
SCT	0	10.8803277	0.162	0	0	pDCs
SLC35F3	0	9.40656268	0.162	0.001	0	pDCs
PLEKHD1	0	10.1177638	0.135	0	0	pDCs
LONRF2	0	9.76244881	0.135	0	0	pDCs
AEBP1	0	9.5573518	0.135	0	0	pDCs
PDGFRB	0	10.7539504	0.108	0	0	pDCs
CRLF21	0	8.72758713	0.108	0	0	pDCs
LRRC26	0	10.0963209	0.108	0	0	pDCs
KRT5	0	11.5132725	0.108	0	0	pDCs
SHD	0	11.850672	0.108	0	0	pDCs
DIPK1B	0	12.3614634	0.081	0	0	pDCs
BEND6	0	12.268303	0.054	0	0	pDCs
INSYN2A	0	12.3405054	0.054	0	0	pDCs
UPK3A1	0	11.1814942	0.054	0	0	pDCs
KCNA5	0	11.5436141	0.054	0	0	pDCs
ITM2C1	2.523E-306	7.04818147	0.676	0.012	3.878E-302	pDCs
TTC39A	3.988E-286	9.94342578	0.189	0.001	6.13E-282	pDCs
WNT10A	3.022E-278	7.15770112	0.189	0.001	4.645E-274	pDCs
PHEX	7.12E-274	9.71313046	0.216	0.001	1.094E-269	pDCs
PTGDS	3.841E-272	9.96107955	0.108	0	5.903E-268	pDCs
PARM12	6.217E-265	7.76487721	0.27	0.002	9.557E-261	pDCs
GPR1832	5.021E-261	5.97197289	0.811	0.02	7.718E-257	pDCs
HMSD1	2.483E-258	9.00152363	0.054	0	3.816E-254	pDCs
MYCL2	1.269E-253	7.42821845	0.27	0.002	1.95E-249	pDCs
LAMP51	1.651E-248	9.79014585	0.297	0.003	2.538E-244	pDCs
ROR11	2.939E-232	8.58213129	0.081	0	4.518E-228	pDCs
GPR331	3.036E-232	9.09798258	0.081	0	4.667E-228	pDCs
RHEX1	3.636E-232	6.78253388	0.081	0	5.59E-228	pDCs
ADAM192	2.034E-227	5.0965027	0.73	0.018	3.127E-223	pDCs
MAP1A	8.894E-219	8.51194904	0.432	0.007	1.367E-214	pDCs
PANX21	1.334E-218	8.55692987	0.135	0.001	2.051E-214	pDCs
P2RY63	3.874E-216	7.68217157	0.676	0.017	5.955E-212	pDCs
RASAL12	2.116E-215	8.87900507	0.108	0	3.252E-211	pDCs
SLC38A11	1.747E-206	4.52063023	0.703	0.018	2.685E-202	pDCs
EPHB32	3.82E-190	8.20794964	0.243	0.002	5.872E-186	pDCs
ACY31	2.921E-189	10.9812544	0.081	0	4.49E-185	pDCs
ZNF6081	2.115E-187	7.15829907	0.108	0	3.251E-183	pDCs
CNR21	7.021E-185	8.46294819	0.081	0	1.079E-180	pDCs
PPP1R16B1	5.843E-184	5.01524561	0.432	0.008	8.982E-180	pDCs
RAPGEF4	1.243E-181	11.5634636	0.027	0	1.91E-177	pDCs
CCSER12	7.674E-180	6.6619779	0.243	0.003	1.18E-175	pDCs
CLNK1	2.2E-178	6.01491526	0.189	0.002	3.382E-174	pDCs
IGLON51	1.901E-177	9.60302895	0.135	0.001	2.922E-173	pDCs

PLEKHG4B1	7.518E-165	8.72465351	0.054	0	1.156E-160	pDCs
DERL3	2.609E-163	8.67632206	0.324	0.005	4.01E-159	pDCs
CLEC17A2	2.516E-162	8.36944983	0.108	0.001	3.867E-158	pDCs
SPNS31	1.29E-159	7.44108437	0.081	0	1.983E-155	pDCs
SPON2	1.552E-159	8.01542026	0.162	0.001	2.385E-155	pDCs
SLC7A112	1.275E-158	6.62639493	0.189	0.002	1.96E-154	pDCs
GNAO12	8.582E-157	6.94314087	0.324	0.005	1.319E-152	pDCs
SLC4A31	1.027E-153	9.74698509	0.081	0	1.578E-149	pDCs
EPHB4	1.054E-153	8.36813901	0.108	0.001	1.62E-149	pDCs
CD1C2	1.562E-153	6.034818	0.243	0.003	2.401E-149	pDCs
NR5A1	1.255E-151	9.70161001	0.027	0	1.929E-147	pDCs
CNKSR2	1.267E-151	9.36861832	0.027	0	1.947E-147	pDCs
TCL1A	1.29E-151	7.8611546	0.027	0	1.982E-147	pDCs
NIBAN3	2.269E-151	9.10798939	0.189	0.002	3.487E-147	pDCs
BCL7A1	2.919E-151	8.56217677	0.135	0.001	4.487E-147	pDCs
NEGR11	6.559E-143	7.83976548	0.081	0	1.008E-138	pDCs
S100B2	4.162E-135	9.04059241	0.297	0.005	6.398E-131	pDCs
SLC2A14	4.91E-132	5.79801039	0.541	0.017	7.547E-128	pDCs
ZC3H12B	3.485E-130	9.29686238	0.027	0	5.357E-126	pDCs
CHST23	1.377E-129	7.34041923	0.243	0.004	2.116E-125	pDCs
GAS63	1.702E-127	7.06876805	0.486	0.015	2.617E-123	pDCs
SLCO5A11	2.541E-127	6.53496363	0.081	0	3.906E-123	pDCs
ZEB11	4.115E-127	4.86745446	0.297	0.005	6.325E-123	pDCs
P2RY101	5.013E-125	4.628514	0.378	0.009	7.705E-121	pDCs
CD1A2	3.806E-123	8.34347723	0.135	0.001	5.851E-119	pDCs
RASD1	5.364E-123	9.12907869	0.189	0.002	8.244E-119	pDCs
FCRLA	3.21E-121	8.48063938	0.054	0	4.934E-117	pDCs
RASSF23	1.247E-120	5.84722197	0.649	0.028	1.917E-116	pDCs
TRAF42	5.339E-116	7.06877267	0.595	0.024	8.207E-112	pDCs
NME82	1.351E-115	8.20855504	0.162	0.002	2.076E-111	pDCs
CXCR31	5.07E-115	5.19699035	0.351	0.008	7.792E-111	pDCs
DSG21	6.032E-115	8.82439175	0.081	0	9.272E-111	pDCs
CPXM11	4.057E-114	11.399601	0.027	0	6.236E-110	pDCs
NPTX2	4.057E-114	12.12441	0.027	0	6.236E-110	pDCs
LOXL4	4.085E-114	10.3958927	0.027	0	6.279E-110	pDCs
GOT1L1	4.113E-114	10.2011708	0.027	0	6.321E-110	pDCs
NDRG4	4.14E-114	9.84426764	0.027	0	6.364E-110	pDCs
LINGO4	4.225E-114	8.26433734	0.027	0	6.495E-110	pDCs
ASIP	9.779E-114	10.7594531	0.054	0	1.503E-109	pDCs
CD1E2	4.769E-113	6.63100942	0.162	0.002	7.33E-109	pDCs
GPM6B	7.849E-109	9.01940351	0.108	0.001	1.206E-104	pDCs
PKIB2	1.478E-108	7.11079566	0.297	0.006	2.272E-104	pDCs
TNFRSF11A2	2.706E-104	7.39372792	0.135	0.001	4.16E-100	pDCs
GRASP3	7.952E-103	6.43640004	0.541	0.023	1.2224E-98	pDCs
KCNK10	1.318E-101	10.5562789	0.027	0	2.0265E-97	pDCs
P3H22	2.337E-101	6.65656165	0.162	0.002	3.5923E-97	pDCs
LIMD23	2.274E-100	4.809796	0.595	0.027	3.4949E-96	pDCs
TLCD5	1.232E-99	8.49986865	0.081	0.001	1.8938E-95	pDCs
FCER1A2	5.364E-99	6.83411712	0.189	0.003	8.2455E-95	pDCs
SEMA7A2	1.5562E-98	7.10043472	0.622	0.032	2.3921E-94	pDCs
DBN11	1.8274E-95	5.55350846	0.189	0.003	2.8089E-91	pDCs
CHST152	8.9164E-95	6.09347587	0.162	0.002	1.3705E-90	pDCs

SMAGP1	6.3076E-94	7.37129966	0.108	0.001	9.6954E-90	pDCs
CCDC102A1	1.1727E-92	6.09738802	0.108	0.001	1.8026E-88	pDCs
GPR3	1.3623E-91	9.60604852	0.027	0	2.094E-87	pDCs
MTUS2	1.3623E-91	9.14121915	0.027	0	2.094E-87	pDCs
SNX221	5.5765E-91	6.85308462	0.108	0.001	8.5716E-87	pDCs
ADGRG51	1.9821E-89	6.02973965	0.351	0.011	3.0466E-85	pDCs
TLR102	3.7235E-89	7.44691647	0.216	0.004	5.7234E-85	pDCs
PRKCB3	3.9517E-89	5.38427453	0.784	0.056	6.0741E-85	pDCs
CIB21	5.2749E-89	7.63490706	0.162	0.002	8.1081E-85	pDCs
KIF26B1	5.3505E-89	8.446461	0.054	0	8.2242E-85	pDCs
KCNK1	6.2845E-88	7.94117837	0.081	0.001	9.6599E-84	pDCs
AMN	9.0564E-88	7.32547092	0.108	0.001	1.3921E-83	pDCs
RHBDL3	6.3709E-87	9.53520248	0.054	0	9.7927E-83	pDCs
KLHL13	6.8876E-85	7.58533058	0.054	0	1.0587E-80	pDCs
IDO22	8.5572E-82	7.47268539	0.081	0.001	1.3153E-77	pDCs
HRH23	1.0629E-81	6.95352016	0.189	0.003	1.6338E-77	pDCs
PTP4A31	1.7449E-81	6.50604603	0.27	0.007	2.6821E-77	pDCs
ASB21	3.4075E-80	6.57032105	0.081	0.001	5.2376E-76	pDCs
SLC18A2	2.0365E-79	7.03062826	0.054	0	3.1303E-75	pDCs
FBXO16	1.4096E-76	9.45770541	0.027	0	2.1667E-72	pDCs
CDH21	1.4679E-76	7.95203434	0.027	0	2.2563E-72	pDCs
CYYR11	7.7156E-76	8.81992062	0.081	0.001	1.186E-71	pDCs
CD2072	4.2764E-75	7.31286654	0.081	0.001	6.5733E-71	pDCs
SMPD31	7.3502E-75	7.8417252	0.351	0.013	1.1298E-70	pDCs
FHL2	1.2733E-74	7.31394095	0.054	0	1.9572E-70	pDCs
TACSTD22	1.8128E-74	5.83652441	0.189	0.004	2.7865E-70	pDCs
GEM2	8.941E-74	7.5376253	0.081	0.001	1.3743E-69	pDCs
KIT1	1.1332E-73	4.24954237	0.081	0.001	1.7418E-69	pDCs
MSRB3	3.807E-73	7.07366416	0.054	0	5.8517E-69	pDCs
GUCY1A12	3.8832E-73	7.46167034	0.081	0.001	5.9689E-69	pDCs
LAMP32	1.4976E-71	6.8826562	0.243	0.007	2.302E-67	pDCs
KCNJ11	8.6138E-71	7.74342792	0.027	0	1.324E-66	pDCs
GNG72	4.079E-70	8.31783956	0.189	0.004	6.2698E-66	pDCs
AKAP62	5.6599E-70	6.91126146	0.108	0.001	8.6998E-66	pDCs
RIMS32	2.2709E-69	6.62568428	0.243	0.007	3.4906E-65	pDCs
CCDC1831	1.6354E-67	7.62207432	0.216	0.005	2.5138E-63	pDCs
CST72	5.8147E-66	3.79171712	0.351	0.014	8.9378E-62	pDCs
SLC16A2	7.778E-66	8.73842421	0.027	0	1.1956E-61	pDCs
BSPRY	1.0431E-65	8.24883147	0.162	0.003	1.6034E-61	pDCs
PCDH9	1.9126E-65	8.01526242	0.054	0	2.9399E-61	pDCs
TLR91	2.0347E-65	7.2741016	0.162	0.003	3.1276E-61	pDCs
HIC12	5.7686E-65	6.65478013	0.108	0.001	8.8669E-61	pDCs
MFSD6L2	2.3366E-63	8.33434931	0.135	0.002	3.5916E-59	pDCs
CCND22	2.9684E-63	4.41919457	0.432	0.023	4.5627E-59	pDCs
OTOA1	3.0693E-63	9.44375516	0.054	0	4.7179E-59	pDCs
TCF43	1.4662E-62	7.28655686	0.757	0.077	2.2536E-58	pDCs
MMP252	1.9704E-62	5.85650639	0.108	0.001	3.0287E-58	pDCs
MMP11	3.5291E-62	9.07551995	0.054	0	5.4246E-58	pDCs
ABCA92	3.6471E-62	7.71456659	0.054	0	5.606E-58	pDCs
RAB151	3.8941E-62	5.81309318	0.108	0.001	5.9856E-58	pDCs
PRSS31	1.5486E-61	8.36344479	0.027	0	2.3804E-57	pDCs
ACPP3	2.5674E-60	6.08022895	0.189	0.005	3.9464E-56	pDCs

LRRC36	3.8164E-60	7.10002994	0.054	0	5.8661E-56	pDCs
NKG7	4.4896E-60	2.98922551	0.351	0.016	6.9009E-56	pDCs
ENPP12	7.2923E-60	6.72519142	0.081	0.001	1.1209E-55	pDCs
ADAM283	8.6856E-60	5.52268504	0.27	0.01	1.3351E-55	pDCs
CCR62	7.5089E-59	4.99173422	0.297	0.012	1.1542E-54	pDCs
SETBP11	2.1005E-58	6.12994564	0.189	0.005	3.2287E-54	pDCs
ZBED2	2.9739E-58	6.44254029	0.054	0	4.5712E-54	pDCs
SLAMF72	7.6327E-58	5.01995153	0.459	0.029	1.1732E-53	pDCs
SEMA4C2	8.0283E-58	5.63870562	0.135	0.003	1.234E-53	pDCs
FAM177B	8.6376E-58	9.8576632	0.027	0	1.3277E-53	pDCs
SEMA4G	8.8444E-58	7.46161547	0.027	0	1.3595E-53	pDCs
SLC7A53	1.2156E-57	6.13703308	0.622	0.054	1.8684E-53	pDCs
PALD13	8.7256E-57	6.51009165	0.541	0.041	1.3412E-52	pDCs
ZNF8271	1.0115E-56	5.05339884	0.108	0.002	1.5548E-52	pDCs
ADRB32	1.4249E-56	6.42183858	0.081	0.001	2.1902E-52	pDCs
KLHL33	1.0741E-55	8.74481588	0.054	0	1.651E-51	pDCs
FAM160A11	1.1188E-55	8.6030356	0.054	0	1.7197E-51	pDCs
SLA2	1.2347E-55	4.4839206	0.243	0.008	1.8978E-51	pDCs
SERPINF12	1.6092E-55	6.78034115	0.73	0.081	2.4735E-51	pDCs
FZD31	3.9132E-55	7.04329033	0.081	0.001	6.015E-51	pDCs
IGHA1	7.43E-55	6.08674726	0.054	0	1.1421E-50	pDCs
MCOLN22	8.4933E-55	5.33356573	0.676	0.068	1.3055E-50	pDCs
POU2AF1	4.6013E-54	7.42691967	0.054	0	7.0727E-50	pDCs
ACAP12	3.2001E-52	3.45471378	0.432	0.027	4.9189E-48	pDCs
GALNT15	1.6027E-51	9.31352714	0.027	0	2.4635E-47	pDCs
CPLX1	1.6075E-51	8.91969163	0.027	0	2.4709E-47	pDCs
IL21R3	3.9915E-51	4.36314127	0.351	0.019	6.1353E-47	pDCs
AUTS21	4.1549E-51	4.05317332	0.189	0.005	6.3864E-47	pDCs
CCR72	6.4793E-51	3.98733271	0.135	0.003	9.9594E-47	pDCs
NDRG23	3.0812E-50	6.28930131	0.27	0.011	4.7361E-46	pDCs
IL18R12	6.5942E-49	4.23319455	0.189	0.006	1.0136E-44	pDCs
SKIDA1	6.9713E-49	9.96085856	0.027	0	1.0716E-44	pDCs
TTC24	7.0311E-49	8.14841709	0.027	0	1.0808E-44	pDCs
ADGRG62	1.1331E-48	7.32439588	0.108	0.002	1.7417E-44	pDCs
MYO1D2	2.5259E-47	4.80836179	0.27	0.012	3.8826E-43	pDCs
GRIP1	2.821E-47	8.35823714	0.054	0	4.3361E-43	pDCs
FAM118A2	6.0311E-47	5.37881726	0.351	0.02	9.2704E-43	pDCs
MBOAT22	6.8575E-47	5.70194544	0.135	0.003	1.0541E-42	pDCs
MPPED21	1.6718E-46	8.68594017	0.027	0	2.5698E-42	pDCs
TSPAN13	2.867E-46	7.68768404	0.459	0.036	4.4068E-42	pDCs
MMP23B	2.8767E-45	7.70331204	0.081	0.001	4.4218E-41	pDCs
XCR11	5.7239E-45	5.49570382	0.054	0.001	8.7982E-41	pDCs
SCN9A3	3.2025E-44	6.45998951	0.297	0.016	4.9226E-40	pDCs
BCL11A3	4.071E-44	6.61393127	0.568	0.059	6.2576E-40	pDCs
SERPINB93	4.5054E-43	5.73373358	0.622	0.072	6.9252E-39	pDCs
CRYM	6.0708E-43	7.9095388	0.081	0.001	9.3314E-39	pDCs
ADAM83	1.9451E-42	4.22501588	0.324	0.019	2.9898E-38	pDCs
EPHB1	2.1223E-42	8.40982602	0.027	0	3.2621E-38	pDCs
RUNX22	3.3305E-42	5.62237471	0.595	0.067	5.1194E-38	pDCs
CCL172	3.3828E-42	5.43800514	0.108	0.002	5.1997E-38	pDCs
MCC1	4.0495E-42	5.92029062	0.108	0.002	6.2246E-38	pDCs
NCKAP52	1.3323E-41	6.86002913	0.081	0.001	2.0479E-37	pDCs

ANTXR23	1.5783E-41	3.88074304	0.378	0.026	2.4259E-37	pDCs
MS4A1	4.4168E-41	6.81883995	0.054	0.001	6.789E-37	pDCs
GPR391	1.3238E-40	8.1834177	0.027	0	2.0349E-36	pDCs
GIPR1	1.4065E-40	6.4205027	0.081	0.001	2.1619E-36	pDCs
IDO12	1.8972E-40	5.90751305	0.162	0.005	2.9162E-36	pDCs
DMWD1	2.4104E-40	4.31123161	0.189	0.007	3.705E-36	pDCs
LY91	2.5742E-40	4.09041827	0.189	0.007	3.9569E-36	pDCs
SLC23A1	3.2213E-40	7.40580655	0.054	0.001	4.9515E-36	pDCs
LGALS23	9.2671E-40	5.46788534	0.27	0.014	1.4244E-35	pDCs
FXYD22	3.506E-39	6.70187726	0.135	0.004	5.389E-35	pDCs
SLC38A51	3.746E-39	4.82101263	0.108	0.002	5.758E-35	pDCs
CD2001	5.7653E-39	7.01981342	0.027	0	8.8618E-35	pDCs
P2RY81	1.9482E-38	3.87658761	0.189	0.007	2.9945E-34	pDCs
WASF11	2.6172E-38	7.22058135	0.162	0.005	4.0228E-34	pDCs
PARD6G	3.388E-38	6.46576285	0.081	0.001	5.2077E-34	pDCs
GHRL2	6.2702E-38	7.37871374	0.108	0.002	9.638E-34	pDCs
DPP41	9.0093E-38	3.63675772	0.216	0.01	1.3848E-33	pDCs
FAM81A	4.6225E-37	7.82459608	0.081	0.001	7.1052E-33	pDCs
PTGES3L	6.062E-37	7.18160659	0.162	0.006	9.3179E-33	pDCs
ATP2A32	7.8105E-37	4.26641036	0.514	0.055	1.2006E-32	pDCs
CARD101	4.5591E-36	7.54418107	0.027	0	7.0077E-32	pDCs
PAWR1	1.2219E-35	5.79660428	0.054	0.001	1.8781E-31	pDCs
RFLNB2	2.2809E-35	3.78831217	0.27	0.016	3.5059E-31	pDCs
BAIAP2L1	5.4207E-35	6.11714915	0.081	0.002	8.3321E-31	pDCs
ASAP23	6.5401E-35	6.57187793	0.108	0.003	1.0053E-30	pDCs
SH3GL2	8.605E-35	10.2882086	0.027	0	1.3227E-30	pDCs
SELL2	1.6859E-34	6.22874119	0.486	0.054	2.5914E-30	pDCs
SEPTIN1	2.4004E-34	3.53367195	0.351	0.027	3.6897E-30	pDCs
EPS8L22	2.8158E-34	5.70607322	0.135	0.004	4.3282E-30	pDCs
CARD112	3.4637E-34	4.38812533	0.405	0.037	5.324E-30	pDCs
CD79A	8.9833E-34	7.80133455	0.081	0.002	1.3808E-29	pDCs
KIF5A	1.4125E-33	6.27500452	0.027	0	2.1711E-29	pDCs
XYLT12	1.9339E-33	5.12744574	0.243	0.014	2.9725E-29	pDCs
CORO1A3	8.4338E-33	4.12431917	0.865	0.209	1.2964E-28	pDCs
ST6GAL12	1.1604E-32	3.90000119	0.486	0.055	1.7837E-28	pDCs
PPP1R1A	1.2168E-32	7.59088073	0.054	0.001	1.8703E-28	pDCs
STEAP1	1.7686E-32	8.58451277	0.027	0	2.7186E-28	pDCs
RAMP2	1.7719E-32	8.9221047	0.027	0	2.7237E-28	pDCs
UGT8	1.7786E-32	8.67681259	0.027	0	2.7338E-28	pDCs
TRERF12	2.5768E-32	3.91953461	0.243	0.014	3.9608E-28	pDCs
SLC22A231	4.3334E-32	6.93588644	0.081	0.002	6.6608E-28	pDCs
MYEF21	5.8028E-32	4.8887975	0.243	0.014	8.9195E-28	pDCs
FNBP1L2	1.7798E-31	6.26723859	0.162	0.007	2.7358E-27	pDCs
FSCN12	1.8031E-31	7.18706426	0.162	0.007	2.7716E-27	pDCs
GXYLT2	1.934E-31	9.01519606	0.027	0	2.9728E-27	pDCs
OR5AN1	1.9375E-31	9.48822442	0.027	0	2.9782E-27	pDCs
DACT3	1.9799E-31	8.31912131	0.027	0	3.0433E-27	pDCs
FAM171B	8.8876E-31	6.41860324	0.054	0.001	1.3661E-26	pDCs
MEF2C3	2.8479E-30	5.03551409	0.514	0.069	4.3775E-26	pDCs
AOC11	1.4891E-29	10.3701713	0.027	0	2.2889E-25	pDCs
SV2A	1.5429E-29	6.00103377	0.027	0	2.3716E-25	pDCs
DLGAP31	1.9738E-28	7.11701252	0.081	0.002	3.034E-24	pDCs

EMB2	2.4702E-28	3.22866224	0.297	0.023	3.7969E-24	pDCs
CD300LD	6.9125E-28	8.50000286	0.027	0	1.0625E-23	pDCs
CLEC9A2	9.5004E-28	6.13967441	0.081	0.002	1.4603E-23	pDCs
AZIN22	1.5754E-27	6.15861592	0.162	0.008	2.4216E-23	pDCs
PREX2	3.9855E-27	9.13902294	0.027	0	6.1261E-23	pDCs
RTP5	4.1169E-27	6.00699246	0.027	0	6.3281E-23	pDCs
P2RY142	6.2171E-27	5.50960053	0.459	0.061	9.5563E-23	pDCs
TOX2	1.9376E-26	4.3355125	0.108	0.004	2.9783E-22	pDCs
GPR1323	2.436E-26	3.77187573	0.459	0.06	3.7444E-22	pDCs
MEIS1	3.3496E-26	6.21457737	0.081	0.002	5.1487E-22	pDCs
ZNF2962	4.3671E-26	4.90452887	0.378	0.042	6.7127E-22	pDCs
BCAS4	9.165E-26	5.67434371	0.081	0.002	1.4088E-21	pDCs
CACNB4	9.4444E-26	7.22715618	0.081	0.002	1.4517E-21	pDCs
DACH1	1.022E-25	7.04398049	0.027	0	1.5709E-21	pDCs
PLXNC13	1.535E-25	4.93462014	0.351	0.037	2.3595E-21	pDCs
TSPAN333	3.3736E-25	5.70188102	0.459	0.065	5.1856E-21	pDCs
PNOC1	3.7425E-25	7.07154375	0.162	0.008	5.7526E-21	pDCs
DOK5	4.4214E-25	8.70934728	0.027	0	6.7962E-21	pDCs
FBXO171	4.4341E-25	8.70847306	0.027	0	6.8156E-21	pDCs
HIST1H2BG3	7.0395E-25	5.61664728	0.243	0.018	1.082E-20	pDCs
TESPA11	9.2594E-25	3.22228469	0.162	0.008	1.4233E-20	pDCs
PXYLP11	1.2052E-24	5.25628886	0.054	0.001	1.8526E-20	pDCs
FAM30A	1.8556E-24	6.28657606	0.027	0	2.8522E-20	pDCs
CYP46A1	6.9062E-24	8.92652718	0.027	0	1.0615E-19	pDCs
IGFALS	6.9249E-24	7.79406455	0.027	0	1.0644E-19	pDCs
RBMS3	6.9436E-24	8.19132731	0.027	0	1.0673E-19	pDCs
KHDRBS22	8.5137E-24	6.65483633	0.081	0.002	1.3086E-19	pDCs
NFATC22	8.7926E-24	3.00367604	0.405	0.051	1.3515E-19	pDCs
BLK1	1.1532E-23	5.94049266	0.189	0.012	1.7726E-19	pDCs
MARCKSL12	1.6769E-23	5.62755252	0.297	0.029	2.5775E-19	pDCs
UCK23	3.8894E-23	5.09952475	0.297	0.029	5.9785E-19	pDCs
TIFAB2	4.4082E-23	6.66161502	0.189	0.012	6.7759E-19	pDCs
PPM1J1	5.1204E-23	5.78912695	0.324	0.035	7.8705E-19	pDCs
NGFR	8.3019E-23	9.26583354	0.027	0	1.2761E-18	pDCs
MYO1B	1.2006E-22	5.30222149	0.054	0.001	1.8455E-18	pDCs
BACH21	2.306E-22	4.60882807	0.054	0.001	3.5446E-18	pDCs
GPR174	3.9337E-22	3.21518079	0.135	0.007	6.0465E-18	pDCs
EML5	5.0453E-22	5.9242391	0.054	0.001	7.7551E-18	pDCs
MS4A4E3	6.1557E-22	4.78474361	0.297	0.031	9.462E-18	pDCs
PTGIR2	1.1584E-21	6.44214398	0.054	0.001	1.7806E-17	pDCs
FUT73	1.9345E-21	5.94496877	0.243	0.021	2.9736E-17	pDCs
EPS8L3	2.3085E-21	8.04073598	0.027	0	3.5484E-17	pDCs
CARMIL21	2.4386E-21	3.75774834	0.189	0.013	3.7484E-17	pDCs
ENHO3	2.4838E-21	5.06491076	0.081	0.003	3.8178E-17	pDCs
DUSP52	2.7498E-21	4.98814292	0.432	0.067	4.2267E-17	pDCs
TTN	2.935E-21	4.98622699	0.054	0.001	4.5114E-17	pDCs
ZNF7111	3.6647E-21	6.56220161	0.135	0.007	5.633E-17	pDCs
ACHE1	6.3567E-21	7.84362754	0.027	0	9.7709E-17	pDCs
CLEC4F2	1.21E-20	6.56225959	0.054	0.001	1.8599E-16	pDCs
LTBP21	1.2271E-20	6.17550681	0.054	0.001	1.8862E-16	pDCs
FAM149A2	1.5998E-20	6.25767278	0.054	0.001	2.4591E-16	pDCs
HDAC93	1.6184E-20	4.3135721	0.459	0.079	2.4877E-16	pDCs

CPNE5	1.7338E-20	4.79004281	0.027	0	2.6651E-16	pDCs
AKNA2	2.3642E-20	2.9542556	0.946	0.553	3.6339E-16	pDCs
HS3ST3B13	2.4078E-20	5.05922184	0.27	0.028	3.7011E-16	pDCs
SOX51	4.1277E-20	6.35315737	0.054	0.001	6.3448E-16	pDCs
GPER11	4.3709E-20	5.92211331	0.027	0	6.7185E-16	pDCs
SLC30A42	4.4855E-20	5.09967152	0.27	0.028	6.8946E-16	pDCs
ETV53	6.757E-20	4.94096236	0.216	0.018	1.0386E-15	pDCs
HSH2D3	7.5088E-20	3.76272152	0.405	0.062	1.1542E-15	pDCs
SMIM51	9.3707E-20	5.71777667	0.162	0.011	1.4404E-15	pDCs
ADAT3	1.0543E-19	7.06547005	0.027	0	1.6205E-15	pDCs
DUSP42	1.2996E-19	3.62068638	0.162	0.01	1.9976E-15	pDCs
SMO2	1.7964E-19	5.85832842	0.108	0.005	2.7612E-15	pDCs
TNFSF181	2.4264E-19	7.53351591	0.027	0	3.7297E-15	pDCs
CDK5R1	3.0206E-19	5.7067831	0.135	0.008	4.643E-15	pDCs
BCO22	3.2341E-19	5.49531517	0.108	0.005	4.9711E-15	pDCs
STRBP1	3.8544E-19	4.30136267	0.541	0.121	5.9246E-15	pDCs
EPOP1	4.7491E-19	7.1747189	0.054	0.001	7.2998E-15	pDCs
EYA4	5.5218E-19	7.03904646	0.027	0	8.4876E-15	pDCs
ELAVL42	5.6163E-19	6.69982805	0.027	0	8.6328E-15	pDCs
ST8SIA43	7.9694E-19	3.46268595	0.459	0.083	1.225E-14	pDCs
ZBTB462	7.9847E-19	5.36591756	0.297	0.036	1.2273E-14	pDCs
SCAMP51	1.0488E-18	6.06299633	0.297	0.036	1.6122E-14	pDCs
COL13A1	1.2051E-18	8.79893436	0.027	0	1.8523E-14	pDCs
ENOX11	1.2139E-18	7.35536779	0.027	0	1.8659E-14	pDCs
TAS2R7	1.2177E-18	7.48326068	0.027	0	1.8717E-14	pDCs
ST182	1.2485E-18	6.61822651	0.027	0	1.919E-14	pDCs
OSBPL72	1.9545E-18	4.69515301	0.108	0.005	3.0043E-14	pDCs
BDKRB2	2.6121E-18	7.07538304	0.027	0	4.0151E-14	pDCs
RIPOR23	3.4172E-18	3.92988765	0.432	0.075	5.2525E-14	pDCs
AP1S31	3.5833E-18	4.68734888	0.351	0.051	5.5079E-14	pDCs
LTC4S2	5.8142E-18	5.29400625	0.081	0.003	8.937E-14	pDCs
ADA1	1.2937E-17	4.53037102	0.324	0.045	1.9886E-13	pDCs
ZNF4673	1.5215E-17	4.24352854	0.162	0.012	2.3387E-13	pDCs
TPM2	1.9085E-17	4.83258047	0.27	0.032	2.9335E-13	pDCs
TNFRSF13C	2.2558E-17	6.6034684	0.054	0.001	3.4674E-13	pDCs
LAMA51	3.2468E-17	6.06281298	0.054	0.001	4.9906E-13	pDCs
CMTM42	1.2739E-16	4.74515963	0.081	0.003	1.9581E-12	pDCs
OGT2	1.4594E-16	2.85264231	0.892	0.548	2.2432E-12	pDCs
ANKRD33B1	2.6724E-16	5.93489384	0.027	0	4.1077E-12	pDCs
MAP3K142	2.7547E-16	4.9347389	0.297	0.041	4.2342E-12	pDCs
RFTN12	3.2904E-16	3.95370113	0.622	0.196	5.0576E-12	pDCs
CHD33	3.4138E-16	3.39678332	0.486	0.108	5.2473E-12	pDCs
LMCD12	3.775E-16	7.04554309	0.135	0.009	5.8025E-12	pDCs
MED12L	4.7113E-16	6.28867304	0.027	0	7.2417E-12	pDCs
JAK32	5.7438E-16	3.21430276	0.378	0.065	8.8288E-12	pDCs
ARGLU12	6.2225E-16	2.3382874	0.892	0.589	9.5646E-12	pDCs
CCDC1361	8.1774E-16	3.90713863	0.054	0.002	1.2569E-11	pDCs
CADM41	1.3166E-15	5.63426005	0.162	0.013	2.0238E-11	pDCs
SLC7A62	1.348E-15	3.41325011	0.703	0.272	2.072E-11	pDCs
TNFRSF13B	1.4083E-15	6.24682819	0.027	0	2.1646E-11	pDCs
TDRD102	3.8528E-15	8.51511911	0.027	0	5.9221E-11	pDCs
APCDD11	3.8882E-15	7.03163574	0.027	0	5.9766E-11	pDCs

PLXDC1	4.2206E-15	4.83937418	0.054	0.002	6.4875E-11	pDCs
VWA2	6.3507E-15	6.62374393	0.027	0	9.7617E-11	pDCs
CLEC2D2	7.129E-15	2.03817166	0.324	0.05	1.0958E-10	pDCs
VPREB3	1.0125E-14	8.18428591	0.027	0	1.5562E-10	pDCs
BEND51	1.0231E-14	6.7929431	0.027	0	1.5727E-10	pDCs
FEZ1	1.0339E-14	5.55762745	0.027	0	1.5893E-10	pDCs
FSD1	1.0491E-14	5.63928891	0.027	0	1.6125E-10	pDCs
FBXO271	1.278E-14	5.18598896	0.054	0.002	1.9644E-10	pDCs
ATP8A11	1.3108E-14	3.88928106	0.378	0.071	2.0149E-10	pDCs
GPR1711	1.3271E-14	1.87770184	0.135	0.01	2.0398E-10	pDCs
DRD4	1.5385E-14	6.48153596	0.054	0.002	2.3648E-10	pDCs
IGHG1	1.6165E-14	2.87692049	0.027	0	2.4848E-10	pDCs
PILRB2	1.6406E-14	2.91454947	0.784	0.386	2.5218E-10	pDCs
DEUP1	1.6714E-14	5.71718593	0.027	0	2.5692E-10	pDCs
DENND5B2	1.7946E-14	4.91937655	0.243	0.031	2.7585E-10	pDCs
SATB13	2.5879E-14	3.21461458	0.432	0.095	3.9779E-10	pDCs
BTG22	2.7538E-14	2.3331559	0.919	0.754	4.2329E-10	pDCs
WDFY43	2.9036E-14	2.52213927	0.892	0.713	4.4631E-10	pDCs
SIGLEC6	2.9518E-14	5.99511882	0.162	0.015	4.5372E-10	pDCs
HAPLN32	3.1373E-14	3.96741792	0.216	0.025	4.8223E-10	pDCs
ADM22	4.5016E-14	5.59269376	0.054	0.002	6.9194E-10	pDCs
GNG22	5.336E-14	2.55122892	0.189	0.019	8.2019E-10	pDCs
ARL4C3	5.7876E-14	3.28142909	0.541	0.157	8.8961E-10	pDCs
IGSF31	6.1155E-14	6.00444084	0.027	0	9.4001E-10	pDCs
PLEKHO13	8.0296E-14	3.39397845	0.757	0.422	1.2342E-09	pDCs
HSPA12A	8.9538E-14	7.89269744	0.027	0	1.3763E-09	pDCs
BEX2	9.1329E-14	5.33942359	0.081	0.004	1.4038E-09	pDCs
OBSCN1	1.2208E-13	4.07972948	0.189	0.02	1.8765E-09	pDCs
HERPUD12	1.5022E-13	2.92880474	0.892	0.74	2.3091E-09	pDCs
RAB33A1	1.7251E-13	3.45217805	0.081	0.004	2.6517E-09	pDCs
SLC2A121	1.9779E-13	7.27475872	0.027	0	3.0402E-09	pDCs
IGFBP3	2.0425E-13	5.00201293	0.027	0	3.1396E-09	pDCs
ITPRIPL12	2.2657E-13	3.64743971	0.081	0.004	3.4826E-09	pDCs
REPIN11	2.9088E-13	3.35279559	0.73	0.357	4.4711E-09	pDCs
SLC13A3	3.0033E-13	5.57743538	0.027	0	4.6164E-09	pDCs
BTLA1	3.0098E-13	4.39475627	0.027	0	4.6264E-09	pDCs
CXCR42	3.2043E-13	3.07291019	0.73	0.357	4.9253E-09	pDCs
C15orf391	3.2635E-13	3.02565337	0.811	0.556	5.0164E-09	pDCs
PIM21	3.5381E-13	3.52909336	0.649	0.269	5.4384E-09	pDCs
SLC25A41	4.1478E-13	4.15281739	0.135	0.011	6.3756E-09	pDCs
EBF41	4.347E-13	4.96186923	0.027	0	6.6818E-09	pDCs
TRAF3IP32	5.0084E-13	2.77213102	0.243	0.033	7.6985E-09	pDCs
LUC7L32	5.0372E-13	2.46937527	0.811	0.504	7.7428E-09	pDCs
POU2F23	5.3358E-13	3.93587632	0.216	0.027	8.2017E-09	pDCs
ST3GAL42	5.6799E-13	4.39569756	0.378	0.08	8.7306E-09	pDCs
NRARP2	5.9092E-13	5.302973	0.162	0.016	9.083E-09	pDCs
LTB1	6.9777E-13	3.80229766	0.568	0.19	1.0725E-08	pDCs
CERS42	8.6625E-13	3.67729028	0.081	0.004	1.3315E-08	pDCs
DEPTOR3	9.7168E-13	5.80830606	0.108	0.007	1.4936E-08	pDCs
RABGAP1L1	1.0848E-12	2.55974304	0.865	0.676	1.6674E-08	pDCs
OLFML1	1.1958E-12	8.05351947	0.027	0.001	1.838E-08	pDCs
RUFY43	2.046E-12	6.14888801	0.108	0.008	3.1448E-08	pDCs

TMEM1211	2.3521E-12	5.71029734	0.027	0.001	3.6153E-08	pDCs
PTPN72	2.8566E-12	2.66225244	0.189	0.022	4.3909E-08	pDCs
NCCRP11	3.2542E-12	3.63435602	0.027	0.001	5.0021E-08	pDCs
TOGARAM21	4.4683E-12	5.20825807	0.108	0.008	6.8683E-08	pDCs
RASGRF12	4.9134E-12	5.56116606	0.081	0.005	7.5523E-08	pDCs
CLEC10A3	5.5981E-12	2.55877137	0.135	0.012	8.6048E-08	pDCs
WDR64	5.861E-12	8.33331118	0.027	0.001	9.0089E-08	pDCs
CDH11	5.9913E-12	3.82022148	0.243	0.036	9.2092E-08	pDCs
OR52B21	6.6799E-12	5.70805848	0.054	0.002	1.0268E-07	pDCs
RHOF2	6.8245E-12	3.03424935	0.405	0.099	1.049E-07	pDCs
LENG82	8.5771E-12	2.03314897	0.838	0.651	1.3184E-07	pDCs
BASP13	9.1847E-12	3.52984788	0.351	0.075	1.4118E-07	pDCs
MDM42	1.0912E-11	2.6786775	0.811	0.538	1.6773E-07	pDCs
SLC20A12	1.1697E-11	3.97655341	0.73	0.434	1.7979E-07	pDCs
CNKSR1	1.2087E-11	5.23043048	0.054	0.002	1.8578E-07	pDCs
IRF73	1.4689E-11	3.82300903	0.838	0.701	2.2579E-07	pDCs
CYFIP21	1.5491E-11	3.2968297	0.649	0.304	2.3811E-07	pDCs
CNGA1	1.8388E-11	7.83385073	0.027	0.001	2.8264E-07	pDCs
SH3D19	1.8615E-11	6.25478586	0.027	0.001	2.8613E-07	pDCs
NCALD1	2.6104E-11	4.75989278	0.054	0.002	4.0124E-07	pDCs
CCND12	2.6293E-11	5.48884488	0.081	0.005	4.0415E-07	pDCs
HIST1H1B1	3.5364E-11	2.87835306	0.297	0.056	5.4358E-07	pDCs
IZUMO41	3.8456E-11	4.55072897	0.108	0.009	5.9111E-07	pDCs
OSBP21	4.0877E-11	7.04033753	0.027	0.001	6.2832E-07	pDCs
COBLL1	4.3559E-11	5.1980655	0.27	0.049	6.6954E-07	pDCs
HIST1H1C1	4.6759E-11	2.14660515	0.784	0.463	7.1873E-07	pDCs
MYO5C1	4.7009E-11	4.81471312	0.054	0.002	7.2257E-07	pDCs
ETV63	5.2096E-11	2.04083764	0.811	0.707	8.0076E-07	pDCs
C11orf94	5.3774E-11	6.38851755	0.027	0.001	8.2656E-07	pDCs
ELOVL6	5.4891E-11	3.67428262	0.054	0.002	8.4374E-07	pDCs
CADM12	6.1173E-11	5.0871941	0.108	0.009	9.4029E-07	pDCs
SMAD32	6.2028E-11	2.87013862	0.108	0.009	9.5343E-07	pDCs
PRIMA1	6.7093E-11	7.09026625	0.027	0.001	1.0313E-06	pDCs
PLCB4	6.7484E-11	6.384574	0.027	0.001	1.0373E-06	pDCs
ASGR22	6.8711E-11	5.27339506	0.054	0.002	1.0562E-06	pDCs
ISG202	7.4333E-11	3.37770199	0.27	0.048	1.1426E-06	pDCs
SLAIN1	7.666E-11	4.10356321	0.108	0.009	1.1783E-06	pDCs
STAMBPL12	9.2359E-11	3.51571198	0.135	0.013	1.4196E-06	pDCs
ALDH1L23	9.7575E-11	4.34524476	0.135	0.013	1.4998E-06	pDCs
CBX63	1.0205E-10	3.39783393	0.595	0.256	1.5686E-06	pDCs
PXN2	1.0631E-10	2.89580343	0.189	0.025	1.6341E-06	pDCs
ARHGAP452	1.1099E-10	3.0179241	0.486	0.153	1.706E-06	pDCs
HIST1H1D1	1.381E-10	2.93409613	0.432	0.122	2.1228E-06	pDCs
OLFM22	1.4256E-10	3.82283819	0.027	0.001	2.1913E-06	pDCs
PIK3R63	1.4527E-10	4.94535238	0.216	0.033	2.2329E-06	pDCs
GNLY	1.6708E-10	1.71718384	0.081	0.005	2.5682E-06	pDCs
FAM214A	1.9603E-10	2.97283933	0.622	0.293	3.0132E-06	pDCs
ZFP363	1.9632E-10	1.69018433	0.892	0.871	3.0176E-06	pDCs
TAP13	2.1529E-10	1.93772351	0.838	0.73	3.3091E-06	pDCs
LRMP2	2.2515E-10	3.64195064	0.432	0.128	3.4608E-06	pDCs
UTS2B	2.6558E-10	9.22446215	0.027	0.001	4.0823E-06	pDCs
LRRN1	2.6689E-10	6.84281218	0.027	0.001	4.1024E-06	pDCs

UGCG2	2.9118E-10	3.77646283	0.757	0.517	4.4757E-06	pDCs
DAPK21	2.9799E-10	3.64367714	0.081	0.005	4.5804E-06	pDCs
TMEM8B1	3.1584E-10	4.68576713	0.108	0.009	4.8548E-06	pDCs
SMIM41	3.2984E-10	7.21198276	0.027	0.001	5.0699E-06	pDCs
GALNT33	4.1498E-10	4.81720256	0.108	0.009	6.3787E-06	pDCs
RNASE63	4.9199E-10	3.27868764	0.622	0.308	7.5624E-06	pDCs
SLC37A32	4.9582E-10	3.6996178	0.081	0.005	7.6212E-06	pDCs
CETP	5.0131E-10	6.4600897	0.027	0.001	7.7056E-06	pDCs
PNISR2	5.8746E-10	2.45929684	0.73	0.492	9.0299E-06	pDCs
SERPINB2	6.2604E-10	4.4566648	0.027	0.001	9.6228E-06	pDCs
NFKB22	6.7446E-10	3.17760892	0.541	0.21	1.0367E-05	pDCs
ZNF730	6.7617E-10	6.34204434	0.054	0.003	1.0393E-05	pDCs
PALM2-AKAP2	7.753E-10	3.7824626	0.324	0.077	1.1917E-05	pDCs
HTR72	9.2759E-10	5.7976806	0.027	0.001	1.4258E-05	pDCs
MT-CO32	9.3617E-10	2.56802426	0.784	0.523	1.439E-05	pDCs
CHRFAM7A3	9.4413E-10	4.77275977	0.108	0.01	1.4512E-05	pDCs
CD2AP1	1.0166E-09	3.47213291	0.622	0.31	1.5626E-05	pDCs
TRPM23	1.0239E-09	4.42680759	0.216	0.036	1.5739E-05	pDCs
MTSS12	1.4693E-09	3.95697697	0.081	0.006	2.2584E-05	pDCs
JAM2	1.5854E-09	7.07353818	0.027	0.001	2.4369E-05	pDCs
C3orf70	1.587E-09	7.01961535	0.027	0.001	2.4393E-05	pDCs
PIK3CD3	1.7317E-09	2.66851519	0.73	0.493	2.6618E-05	pDCs
MRC23	1.8244E-09	4.77950422	0.081	0.006	2.8043E-05	pDCs
SH3RF31	1.9052E-09	6.72222017	0.027	0.001	2.9285E-05	pDCs
CIITA3	1.9973E-09	2.27214535	0.838	0.813	3.0701E-05	pDCs
ZFAT1	1.998E-09	4.56403029	0.351	0.093	3.0712E-05	pDCs
FCHSD22	2.1926E-09	3.3902995	0.676	0.41	3.3702E-05	pDCs
GFI1	2.3889E-09	4.27747897	0.108	0.01	3.672E-05	pDCs
ANXA63	2.5499E-09	2.92863827	0.378	0.105	3.9194E-05	pDCs
KLF62	2.6648E-09	2.02181599	0.892	0.821	4.096E-05	pDCs
ADAMTS141	2.7849E-09	5.40338766	0.027	0.001	4.2807E-05	pDCs
RUBCN	2.8296E-09	3.41486404	0.703	0.501	4.3493E-05	pDCs
AHDC12	2.8603E-09	3.89750977	0.27	0.057	4.3966E-05	pDCs
ZBTB183	3.1073E-09	3.37477017	0.595	0.3	4.7763E-05	pDCs
TNFSF42	3.4639E-09	4.57541191	0.081	0.006	5.3244E-05	pDCs
KCNG2	3.7848E-09	6.83263299	0.054	0.003	5.8177E-05	pDCs
SPTBN11	4.5399E-09	4.12014228	0.162	0.022	6.9783E-05	pDCs
ENPP22	5.1462E-09	4.81199841	0.243	0.048	7.9102E-05	pDCs
ARMC12	5.2825E-09	6.05373959	0.027	0.001	8.1197E-05	pDCs
DCLK22	5.7176E-09	4.5747948	0.162	0.023	8.7886E-05	pDCs
CD200R12	6.1975E-09	2.43343089	0.081	0.006	9.5262E-05	pDCs
ITGA71	9.9972E-09	5.78083986	0.054	0.003	0.00015367	pDCs
LDLRAD41	1.0383E-08	3.30934151	0.676	0.438	0.0001596	pDCs
LRP51	1.1128E-08	5.26181089	0.108	0.011	0.00017105	pDCs
SDR42E1	1.1479E-08	5.57238037	0.027	0.001	0.00017644	pDCs
EEF21	1.1503E-08	1.12807581	0.892	0.962	0.00017682	pDCs
RSRP12	1.2743E-08	1.45929372	0.865	0.711	0.00019587	pDCs
CCR3	1.3171E-08	6.29026553	0.027	0.001	0.00020246	pDCs
RBPMS	1.3693E-08	4.55239107	0.027	0.001	0.00021048	pDCs
MT-ND63	1.399E-08	3.78629103	0.432	0.152	0.00021504	pDCs
PLSCR4	1.4611E-08	5.2379702	0.081	0.007	0.00022459	pDCs
ANKRD53	1.5153E-08	8.08279772	0.027	0.001	0.00023292	pDCs

BRD22	1.9526E-08	1.27772402	0.865	0.833	0.00030013	pDCs
GRAMD1C	2.0283E-08	5.89719543	0.027	0.001	0.00031177	pDCs
CD8B2	2.0948E-08	3.48410152	0.027	0.001	0.00032199	pDCs
AGER2	2.1016E-08	3.64679038	0.27	0.062	0.00032304	pDCs
C1orf212	2.1544E-08	5.10991789	0.108	0.011	0.00033115	pDCs
IRF2BP23	2.2233E-08	2.19792179	0.784	0.698	0.00034175	pDCs
DACT11	2.2599E-08	5.89397569	0.054	0.003	0.00034737	pDCs
SERTAD23	2.9648E-08	2.282347	0.73	0.628	0.00045572	pDCs
SPATS22	3.0652E-08	4.26504455	0.27	0.063	0.00047115	pDCs
PIK3CG1	3.1525E-08	2.48779522	0.703	0.579	0.00048457	pDCs
CALCRL3	3.2237E-08	4.72195025	0.162	0.024	0.00049552	pDCs
LMNB12	3.431E-08	2.74617391	0.541	0.249	0.00052738	pDCs
FMNL12	4.0733E-08	1.14462823	0.892	0.945	0.00062611	pDCs
EIF2AK42	4.5486E-08	2.48796367	0.676	0.481	0.00069916	pDCs
SYNE33	5.3202E-08	2.60012388	0.405	0.135	0.00081776	pDCs
MT-ND42	5.3976E-08	2.16485533	0.703	0.419	0.00082966	pDCs
SDK2	5.7083E-08	6.48463851	0.027	0.001	0.00087743	pDCs
RUNX32	5.8163E-08	3.61521577	0.405	0.141	0.00089403	pDCs
CIRBP1	6.3906E-08	1.12235461	0.892	0.902	0.00098229	pDCs
SLC9A72	6.5402E-08	3.6499861	0.486	0.218	0.0010053	pDCs
GPSM12	7.0871E-08	4.26287536	0.054	0.003	0.00108935	pDCs
SLC15A42	7.6658E-08	3.19453721	0.73	0.644	0.00117831	pDCs
LCN12	8.1628E-08	6.93683649	0.027	0.001	0.0012547	pDCs
MYBL2	9.5606E-08	3.45802614	0.162	0.026	0.00146955	pDCs
BIK1	9.6795E-08	5.10503131	0.081	0.007	0.00148783	pDCs
ZNF385C	1.0283E-07	7.25957617	0.027	0.001	0.00158062	pDCs
LRP83	1.0588E-07	3.3959219	0.297	0.079	0.00162752	pDCs
DDX172	1.1283E-07	2.00289045	0.73	0.579	0.00173438	pDCs
CD300LB2	1.1476E-07	5.19497044	0.135	0.019	0.00176392	pDCs
LILRB42	1.2969E-07	3.94772252	0.27	0.069	0.00199348	pDCs
GABBR13	1.3028E-07	3.56601447	0.135	0.019	0.0020026	pDCs
TRABD2A2	1.3307E-07	3.05276525	0.054	0.003	0.00204544	pDCs
DBNL2	1.3337E-07	1.01958606	0.892	0.945	0.00205004	pDCs
TMEM1543	1.63E-07	3.53585132	0.189	0.035	0.00250554	pDCs
DOCK103	1.6315E-07	1.49880418	0.757	0.706	0.00250776	pDCs
ASS12	1.6557E-07	3.75717857	0.054	0.003	0.00254503	pDCs
MT-CO22	1.724E-07	2.73739608	0.676	0.451	0.00265001	pDCs
CLDN14	1.8349E-07	5.10542654	0.054	0.003	0.00282046	pDCs
SLC6A91	1.9893E-07	6.2211273	0.027	0.001	0.00305778	pDCs
MAP61	2.0365E-07	4.39905231	0.027	0.001	0.00313031	pDCs
WNT9A	2.0458E-07	3.72158058	0.027	0.001	0.00314454	pDCs
CYB561A31	2.1507E-07	2.95188182	0.649	0.463	0.00330591	pDCs
SEPTIN61	2.3109E-07	2.65852763	0.649	0.485	0.00355216	pDCs
CD482	2.4091E-07	2.37637694	0.297	0.081	0.00370295	pDCs
NAV12	2.6288E-07	3.65807884	0.405	0.154	0.0040408	pDCs
NAT8L	3.0289E-07	6.11352156	0.081	0.008	0.00465571	pDCs
NTN1	3.3144E-07	5.63925889	0.027	0.001	0.00509457	pDCs
PLP22	3.5109E-07	2.36295704	0.703	0.57	0.00539661	pDCs
BCL22	3.713E-07	2.50023663	0.27	0.069	0.00570723	pDCs
ATP8B2	3.8717E-07	2.07484324	0.108	0.013	0.00595111	pDCs
ZNRF12	3.9114E-07	2.76823773	0.135	0.02	0.0060122	pDCs
PBX22	3.9486E-07	2.03719483	0.622	0.408	0.00606943	pDCs

SLC38A23	4.1589E-07	1.63555821	0.73	0.665	0.00639257	pDCs
ECE12	4.344E-07	3.06567303	0.351	0.117	0.00667713	pDCs
C11orf96	4.416E-07	5.81061262	0.027	0.001	0.00678781	pDCs
CYTH42	4.4665E-07	2.35965437	0.676	0.561	0.00686539	pDCs
PLCH21	4.5573E-07	3.11886768	0.027	0.001	0.00700504	pDCs
FCRL3	4.8375E-07	5.90809326	0.027	0.001	0.00743565	pDCs
LEF1	4.8774E-07	1.83911988	0.054	0.004	0.00749699	pDCs
DLG3	5.0089E-07	3.73185755	0.081	0.008	0.0076991	pDCs
SH2B32	5.1296E-07	1.21446726	0.811	0.816	0.00788467	pDCs
TRA2A1	5.1979E-07	1.28308892	0.784	0.752	0.00798976	pDCs
LUC7L2	5.8765E-07	1.57209474	0.784	0.736	0.00903279	pDCs
GJB2	6.3238E-07	6.3924626	0.027	0.001	0.00972032	pDCs
C11orf91	6.3725E-07	5.56407991	0.027	0.001	0.00979523	pDCs
SCRN13	6.6404E-07	3.52132851	0.405	0.161	0.01020699	pDCs
OFD11	6.8728E-07	3.29246224	0.405	0.16	0.01056412	pDCs
GNAZ1	6.9386E-07	4.45372321	0.054	0.004	0.01066538	pDCs
MAPKAPK2	6.9561E-07	2.49510045	0.811	0.833	0.01069229	pDCs
RGS132	7.4286E-07	6.1195386	0.081	0.008	0.01141848	pDCs
STAT4	7.5917E-07	2.26539734	0.135	0.021	0.01166914	pDCs
LGSN	8.2014E-07	6.71554212	0.027	0.001	0.01260637	pDCs
EZR2	8.3538E-07	1.63886723	0.838	0.83	0.0128406	pDCs
SHF	8.8213E-07	4.22392751	0.135	0.021	0.01355928	pDCs
RELT2	8.871E-07	2.48004791	0.73	0.677	0.01363563	pDCs
GSDMB2	8.8908E-07	4.12113954	0.135	0.021	0.01366609	pDCs
TCF31	9.456E-07	2.63091622	0.541	0.304	0.01453489	pDCs
BAHCC1	9.7783E-07	4.68337907	0.243	0.063	0.01503026	pDCs
MAMDC23	9.9023E-07	2.91051868	0.081	0.008	0.01522076	pDCs
PTPRK1	1.008E-06	4.53397431	0.027	0.001	0.01549474	pDCs
TGIF23	1.0254E-06	3.85745188	0.27	0.076	0.01576068	pDCs
MEX3C2	1.1342E-06	3.75515141	0.324	0.106	0.01743353	pDCs
SMIM6	1.1486E-06	6.64962228	0.027	0.001	0.01765508	pDCs
FCMR1	1.2543E-06	3.0650921	0.108	0.014	0.01927976	pDCs
WNT6	1.3497E-06	6.58708429	0.027	0.001	0.02074675	pDCs
CDC42SE21	1.4198E-06	1.96757041	0.676	0.568	0.02182315	pDCs
IKBKB2	1.4417E-06	1.87281832	0.757	0.682	0.02216013	pDCs
TSPAN21	1.5033E-06	3.54293112	0.054	0.004	0.023107	pDCs
MALT12	1.5357E-06	2.59802076	0.541	0.298	0.02360486	pDCs
COL26A1	1.5627E-06	6.17099061	0.081	0.009	0.02402032	pDCs
DGKA2	1.6014E-06	2.41243021	0.432	0.191	0.02461491	pDCs
ATP8B43	1.6077E-06	3.51816107	0.189	0.04	0.02471255	pDCs
FBLN7	1.626E-06	4.2351974	0.027	0.001	0.02499311	pDCs
CLSTN3	1.6526E-06	2.50773568	0.189	0.039	0.0254021	pDCs
BMP61	1.7258E-06	4.74146386	0.027	0.001	0.02652718	pDCs
YPEL52	1.7931E-06	1.21862248	0.865	0.851	0.02756227	pDCs
F13A13	1.8101E-06	3.65419574	0.054	0.004	0.02782357	pDCs
HIST1H4I	1.8915E-06	5.37185204	0.027	0.001	0.02907475	pDCs
LIME1	1.9046E-06	2.98804935	0.162	0.031	0.02927588	pDCs
COL24A11	1.9974E-06	5.96761795	0.027	0.001	0.03070269	pDCs
ZMIZ22	2.1657E-06	2.00463781	0.676	0.581	0.03328864	pDCs
ETV2	2.3041E-06	8.24228454	0.027	0.001	0.03541639	pDCs
FCHO12	2.6206E-06	3.1588114	0.351	0.129	0.04028134	pDCs
RLN1	2.7011E-06	4.9473471	0.027	0.001	0.04151876	pDCs

SDK11	2.7816E-06	3.75572361	0.027	0.001	0.042756	pDCs
DDX54	2.8595E-06	1.11213502	0.865	0.941	0.04395313	pDCs
STK263	2.8794E-06	3.11635684	0.189	0.042	0.0442589	pDCs
DEPDC73	2.9499E-06	4.97691578	0.081	0.009	0.04534353	pDCs
IGFLR12	2.9732E-06	2.72603728	0.622	0.486	0.04570082	pDCs
CC2D2B2	3.2027E-06	3.96676548	0.081	0.009	0.04922919	pDCs
SLC35G5	3.3133E-06	5.82745356	0.027	0.001	0.05092925	pDCs
PXDN	3.4196E-06	5.99453615	0.054	0.004	0.05256315	pDCs
MT-CYB2	3.7307E-06	2.18650231	0.486	0.232	0.05734497	pDCs
REL2	3.958E-06	1.96745501	0.73	0.708	0.06083821	pDCs
PLXNB23	4.0221E-06	0.9729633	0.838	0.898	0.06182402	pDCs
KAT2B1	4.0826E-06	2.07635333	0.595	0.404	0.06275408	pDCs
IL1RL22	4.0837E-06	5.60461391	0.027	0.001	0.06277119	pDCs
C12orf74	4.0862E-06	5.51558832	0.027	0.001	0.06280913	pDCs
ZNF1542	4.1559E-06	4.49740183	0.108	0.016	0.06388055	pDCs
SCUBE1	4.1647E-06	3.65705689	0.027	0.001	0.06401559	pDCs
GCNA2	4.1656E-06	4.46347187	0.108	0.016	0.06402933	pDCs
TGFBR3L	4.6535E-06	5.70773975	0.027	0.001	0.07152955	pDCs
DNAH6	4.7405E-06	4.46975486	0.027	0.001	0.07286659	pDCs
TTYH22	4.8776E-06	3.29019721	0.243	0.069	0.0749739	pDCs
DDIT42	5.6308E-06	2.34464182	0.514	0.259	0.08655075	pDCs
EMILIN12	5.6527E-06	5.09691134	0.081	0.009	0.08688703	pDCs
SLC5A32	5.7607E-06	3.59302216	0.405	0.175	0.08854701	pDCs
CELF22	5.9334E-06	1.28860939	0.838	0.861	0.09120239	pDCs
FLNB	6.0453E-06	3.67306498	0.351	0.134	0.09292286	pDCs
TMEM45A	6.2916E-06	4.81809295	0.054	0.004	0.09670874	pDCs
MARCH93	6.2977E-06	3.12267489	0.243	0.068	0.09680203	pDCs
EDEM12	6.4242E-06	1.65283787	0.73	0.81	0.09874641	pDCs
N4BP31	6.5289E-06	4.56464133	0.027	0.001	0.10035619	pDCs
UVRAG2	6.6941E-06	2.58878656	0.568	0.398	0.10289506	pDCs
ZNF7032	6.8447E-06	5.36626039	0.054	0.005	0.10521026	pDCs
CDH233	6.8448E-06	4.62700909	0.081	0.01	0.10521112	pDCs
TPCN13	7.0737E-06	4.38933688	0.081	0.01	0.10872935	pDCs
MT-ATP63	7.4181E-06	2.39990952	0.649	0.459	0.11402415	pDCs
N4BP22	7.7035E-06	2.92531581	0.432	0.206	0.11841037	pDCs
ATP1A3	8.2245E-06	5.08034672	0.027	0.001	0.12641808	pDCs
ERN11	8.3483E-06	4.61234188	0.378	0.164	0.12832229	pDCs
LUC7L4	8.5452E-06	2.54151982	0.595	0.395	0.13134873	pDCs
CASS42	8.5595E-06	3.73706854	0.162	0.034	0.13156802	pDCs
SEL1L31	8.5654E-06	3.33231015	0.649	0.588	0.1316584	pDCs
SLC3A1	8.6621E-06	6.01679701	0.027	0.001	0.13314552	pDCs
WDFY23	8.7257E-06	2.71235723	0.568	0.394	0.13412198	pDCs
SNRNP70	8.7324E-06	1.89323139	0.649	0.484	0.13422604	pDCs
AKAP17A2	8.7666E-06	2.16003238	0.622	0.485	0.13475198	pDCs
IKZF11	9.8617E-06	1.07106116	0.838	0.901	0.15158399	pDCs
FAM169A1	1.001E-05	4.38948854	0.054	0.005	0.15386229	pDCs
SLC16A72	1.0057E-05	2.97982971	0.108	0.017	0.15459137	pDCs
BLNK3	1.0384E-05	3.78146332	0.378	0.168	0.15960729	pDCs
CYBRD13	1.0661E-05	3.32810879	0.135	0.025	0.16387515	pDCs
AC073111.41	1.0942E-05	3.83720692	0.162	0.035	0.16819488	pDCs
MAP2K63	1.1029E-05	3.69258776	0.243	0.07	0.16953379	pDCs
USP532	1.1209E-05	4.09650864	0.27	0.085	0.17228751	pDCs

PAN31	1.1694E-05	2.44837164	0.595	0.437	0.17974876	pDCs
SIGLEC103	1.1894E-05	4.00243617	0.27	0.088	0.18282237	pDCs
AMPD33	1.1902E-05	3.99176839	0.108	0.017	0.18294833	pDCs
GNRH2	1.2268E-05	5.41500507	0.027	0.001	0.18857628	pDCs
RGS161	1.2518E-05	2.16621137	0.054	0.005	0.19240783	pDCs
LRRK12	1.2837E-05	2.36690901	0.568	0.394	0.19731651	pDCs
PDE11A	1.2883E-05	6.64885511	0.027	0.001	0.19802655	pDCs
NREP3	1.3638E-05	3.57675209	0.324	0.126	0.20962623	pDCs
FMN13	1.4442E-05	3.59711104	0.189	0.046	0.22199091	pDCs
EGR32	1.5031E-05	2.46071637	0.054	0.005	0.23104624	pDCs
AP1S23	1.6065E-05	2.35681289	0.514	0.318	0.24693448	pDCs
CCDC1862	1.6084E-05	2.99367298	0.405	0.185	0.24722844	pDCs
CTLA41	1.6101E-05	0.74358275	0.054	0.005	0.24748089	pDCs
FBRSL13	1.684E-05	2.75496941	0.514	0.33	0.25885193	pDCs
BANK13	1.7075E-05	4.39421835	0.189	0.047	0.26245642	pDCs
SH3KBP13	1.7721E-05	2.32022641	0.568	0.405	0.27239632	pDCs
HIST1H3H1	1.808E-05	5.0353236	0.054	0.005	0.2779131	pDCs
RACK11	1.8211E-05	1.46427993	0.73	0.852	0.27991639	pDCs
KRT36	1.8807E-05	6.01152622	0.027	0.001	0.28908628	pDCs
TMEM86B1	1.9055E-05	3.09172312	0.243	0.074	0.29288871	pDCs
PARD3	2.0399E-05	4.2364662	0.027	0.001	0.31354703	pDCs
KDM7A3	2.202E-05	3.19269035	0.297	0.107	0.33847008	pDCs
ANKRD442	2.269E-05	2.19875782	0.595	0.459	0.34877029	pDCs
SNCA3	2.2833E-05	3.80345787	0.243	0.074	0.35096091	pDCs
CLECL12	2.3113E-05	4.82670707	0.162	0.036	0.35527475	pDCs
NCOA33	2.3262E-05	2.14249147	0.568	0.404	0.35756165	pDCs
IL18RAP1	2.461E-05	2.22741544	0.054	0.005	0.3782799	pDCs
ABCA62	2.4816E-05	5.68684808	0.054	0.005	0.38144187	pDCs
KIAA01002	2.4828E-05	1.01357101	0.811	0.903	0.38163078	pDCs
ST143	2.5536E-05	1.7946712	0.676	0.659	0.39251126	pDCs
FAAH1	2.6048E-05	4.1077719	0.189	0.049	0.4003911	pDCs
HNRNPD2	2.6472E-05	1.47555237	0.73	0.719	0.40690591	pDCs
TDRD11	2.6608E-05	6.26859165	0.027	0.001	0.40899302	pDCs
CYTH22	2.6845E-05	2.16706808	0.649	0.546	0.41263277	pDCs
FUBP12	2.7245E-05	1.70557632	0.676	0.609	0.41879043	pDCs
NUGGC	2.7631E-05	3.5122245	0.027	0.001	0.42471641	pDCs
GPR682	2.9086E-05	2.4058105	0.054	0.005	0.44707977	pDCs
CEP1281	2.9099E-05	2.78109155	0.405	0.188	0.44727758	pDCs
CCDC89	2.9377E-05	5.73312923	0.027	0.001	0.45155241	pDCs
FADS22	3.0082E-05	1.65658195	0.541	0.298	0.46239114	pDCs
ANKRD223	3.1761E-05	4.17112383	0.081	0.011	0.48820572	pDCs
NLGN22	3.2777E-05	4.45836738	0.054	0.005	0.50381017	pDCs
MPEG13	3.3988E-05	3.0012127	0.676	0.726	0.52242961	pDCs
EHF1	3.4504E-05	4.26857247	0.108	0.018	0.53035765	pDCs
ALOX151	3.5105E-05	2.7653462	0.027	0.001	0.53959378	pDCs
ABCB1	3.6521E-05	3.40680405	0.027	0.001	0.56136693	pDCs
IGHG3	3.6882E-05	3.26155824	0.027	0.001	0.56691628	pDCs
MAPK112	3.7157E-05	4.40457377	0.054	0.005	0.57113845	pDCs
HNRNPA2B12	3.7599E-05	0.79331874	0.865	0.92	0.57793511	pDCs
CMTM81	3.9071E-05	5.13889255	0.027	0.001	0.60056661	pDCs
EPHA2	3.9487E-05	4.63251474	0.189	0.05	0.60695961	pDCs
ISYNA12	4.0634E-05	4.50478941	0.054	0.005	0.62458875	pDCs

LILRA13	4.1134E-05	4.82701193	0.081	0.011	0.63226323	pDCs
SLC41A22	4.154E-05	2.59055971	0.514	0.323	0.63851591	pDCs
FAM117A	4.3311E-05	3.51276729	0.189	0.05	0.66573784	pDCs
TMEM249	4.4095E-05	6.1523278	0.027	0.001	0.67778837	pDCs
FNBP12	4.422E-05	1.39348275	0.703	0.739	0.67969992	pDCs
CDKN1A2	4.4559E-05	2.89355455	0.541	0.338	0.68491511	pDCs
OCIAD2	4.5396E-05	2.85976175	0.243	0.079	0.69778301	pDCs
TNFAIP8L32	4.5683E-05	4.4584047	0.027	0.001	0.70219565	pDCs
ANKRD11	4.6533E-05	1.50494833	0.703	0.716	0.71526293	pDCs
MYOM1	4.6735E-05	3.92863097	0.162	0.039	0.71836034	pDCs
SLC22A14	4.8172E-05	5.80180243	0.027	0.001	0.7404532	pDCs
SLC39A103	4.9167E-05	1.3410303	0.676	0.656	0.7557504	pDCs
JADE31	4.9627E-05	4.10915835	0.135	0.028	0.76281325	pDCs
TFEB3	5.0055E-05	3.31019311	0.378	0.174	0.76939228	pDCs
MICAL12	5.4093E-05	1.37545291	0.757	0.847	0.83146407	pDCs
ADCY10	5.466E-05	6.05213271	0.027	0.001	0.84018517	pDCs
ATP2B42	5.6404E-05	2.3973865	0.459	0.267	0.86699144	pDCs
MED13L3	5.6694E-05	2.6469908	0.541	0.397	0.87144073	pDCs
ETS11	5.7606E-05	0.895149	0.162	0.037	0.88546314	pDCs
HIST1H2AE1	5.9171E-05	3.85875967	0.108	0.019	0.90951916	pDCs
SLC25A61	6.1347E-05	1.36071072	0.73	0.752	0.94297	pDCs
ZNRF21	6.42E-05	1.89566009	0.676	0.676	0.9868124	pDCs
MT-ND53	6.7085E-05	1.77253324	0.486	0.279	1	pDCs
PLAC81	6.8995E-05	2.10982913	0.676	0.525	1	pDCs
PPM1K	6.9358E-05	3.02988038	0.378	0.188	1	pDCs
ADGRL11	7.1758E-05	4.09389815	0.108	0.019	1	pDCs
MYO9A1	7.4091E-05	2.08409742	0.486	0.294	1	pDCs
CPNE11	7.4602E-05	1.40830921	0.703	0.712	1	pDCs
CCDC693	7.5499E-05	2.60464572	0.486	0.299	1	pDCs
RAB301	7.7545E-05	4.26362137	0.162	0.04	1	pDCs
MYBL12	7.8915E-05	1.56872395	0.108	0.019	1	pDCs
LRIT3	8.193E-05	5.34720874	0.027	0.002	1	pDCs
TNFAIP82	8.3847E-05	2.15663248	0.595	0.466	1	pDCs
TSPOAP12	8.469E-05	2.76648361	0.135	0.029	1	pDCs
C12orf451	9.197E-05	3.13419692	0.324	0.142	1	pDCs
FCGBP2	9.3279E-05	5.88315988	0.081	0.012	1	pDCs
CRIP23	9.768E-05	3.01011306	0.081	0.012	1	pDCs
UCP21	9.9352E-05	0.89273628	0.865	0.939	1	pDCs
APP3	0.00010084	2.50136676	0.622	0.527	1	pDCs
GASK1B3	0.00010117	3.69357823	0.135	0.03	1	pDCs
PYROXD22	0.00010218	4.32901865	0.054	0.006	1	pDCs
PTPRF2	0.00010246	3.47340026	0.027	0.002	1	pDCs
AFF12	0.00010319	1.37732609	0.676	0.695	1	pDCs
TBX191	0.00010696	3.5584854	0.162	0.041	1	pDCs
HOOK1	0.00010728	4.9605489	0.027	0.002	1	pDCs
PHGDH2	0.00010994	3.24204652	0.189	0.053	1	pDCs
NOP531	0.0001132	1.06010065	0.757	0.788	1	pDCs
TSEN54	0.00011389	3.05610965	0.297	0.123	1	pDCs
IQCD1	0.00012086	4.21230875	0.027	0.002	1	pDCs
TRO	0.00012257	5.83701057	0.027	0.002	1	pDCs
TRABD1	0.00012662	1.5842705	0.703	0.728	1	pDCs
PLS3	0.00012689	4.72088572	0.081	0.012	1	pDCs

MAST4	0.00012873	2.23893319	0.081	0.012	1	pDCs
FAM90A1	0.00013083	5.34317161	0.054	0.006	1	pDCs
SPRED3	0.000132	5.02955256	0.027	0.002	1	pDCs
ELK33	0.00013511	3.02949797	0.216	0.068	1	pDCs
REC8	0.00013513	3.23748154	0.27	0.104	1	pDCs
CCNL22	0.00013573	1.87022203	0.622	0.495	1	pDCs
H1FX2	0.0001378	2.08841474	0.649	0.624	1	pDCs
IRF81	0.00014339	2.86204142	0.811	0.945	1	pDCs
CX3CR12	0.00014447	3.98111482	0.027	0.002	1	pDCs
GPR353	0.00014536	3.5735647	0.108	0.021	1	pDCs
ZNF727	0.00014572	5.86382328	0.027	0.002	1	pDCs
LSR2	0.00015117	2.7568113	0.054	0.006	1	pDCs
CERK1	0.00015321	2.43281224	0.405	0.223	1	pDCs
DCK2	0.00015967	2.22816221	0.595	0.527	1	pDCs
PGBD1	0.00016177	3.70852994	0.081	0.013	1	pDCs
MS4A6A3	0.00016646	2.0202437	0.703	0.821	1	pDCs
CCL222	0.00016676	7.00289004	0.081	0.013	1	pDCs
CYP2S13	0.00016681	2.95896239	0.27	0.104	1	pDCs
PFKP2	0.00016965	1.96532712	0.541	0.399	1	pDCs
DYNC1I1	0.00017406	4.67848383	0.054	0.006	1	pDCs
ENTPD12	0.00017675	3.54270171	0.486	0.34	1	pDCs
PLEKHA52	0.0001874	2.61381269	0.135	0.031	1	pDCs
SGK13	0.00019126	0.20640259	0.243	0.657	1	pDCs
ABHD151	0.00019221	2.44228896	0.514	0.388	1	pDCs
MLC1	0.00019602	5.45374345	0.027	0.002	1	pDCs
SHTN11	0.00020829	0.45989447	0.324	0.809	1	pDCs
CCDC141	0.00023394	4.19144284	0.027	0.002	1	pDCs
ARHGAP92	0.000234	2.33552094	0.595	0.506	1	pDCs
PFKFB32	0.00023472	2.15284573	0.541	0.43	1	pDCs
ICAM52	0.00024171	4.10664624	0.054	0.006	1	pDCs
ICAM13	0.00024507	2.12381004	0.486	0.328	1	pDCs
PLEKHG11	0.00025483	3.60052717	0.027	0.002	1	pDCs
HNRNPA01	0.00025814	1.08580347	0.811	0.914	1	pDCs
TFR2	0.00026621	5.40462979	0.054	0.006	1	pDCs
CCDC114	0.00027082	3.43050097	0.027	0.002	1	pDCs
DNAJC72	0.00027233	1.2577254	0.676	0.726	1	pDCs
DEGS2	0.00028729	3.927657	0.027	0.002	1	pDCs
COTL12	0.00029993	0.24864289	0.459	0.94	1	pDCs
IFFO22	0.00030464	2.37619826	0.297	0.125	1	pDCs
CD382	0.00031877	3.66284283	0.297	0.127	1	pDCs
ARHGEF403	0.00032344	3.00755812	0.243	0.089	1	pDCs
CCDC189	0.00033043	3.62854558	0.108	0.022	1	pDCs
RAB11FIP11	0.00033282	1.13036991	0.784	0.908	1	pDCs
PPP1R323	0.00033661	4.09693567	0.054	0.007	1	pDCs
ADAM121	0.00034028	4.2685715	0.027	0.002	1	pDCs
BCL2L113	0.00034207	2.46813064	0.459	0.286	1	pDCs
ZRANB2	0.00034784	1.99853852	0.541	0.448	1	pDCs
SMPDL3B1	0.00035	5.07545832	0.081	0.014	1	pDCs
TNFRSF211	0.00035361	3.38060922	0.378	0.211	1	pDCs
FXYD1	0.00035682	4.9221968	0.027	0.002	1	pDCs
RELB2	0.00038729	2.8226225	0.324	0.153	1	pDCs
SYMPK2	0.00040179	1.5956431	0.622	0.634	1	pDCs

RORC	0.00040787	4.60096531	0.027	0.002	1	pDCs
ICAM32	0.00041644	2.49517098	0.378	0.205	1	pDCs
SH3RF13	0.00042063	4.27396397	0.054	0.007	1	pDCs
EPM2AIP11	0.00042491	2.33733663	0.378	0.201	1	pDCs
GPR1413	0.00043102	4.22036638	0.108	0.023	1	pDCs
RAPGEF12	0.00043717	0.97357407	0.703	0.78	1	pDCs
NEDD4L3	0.00044092	3.51142947	0.162	0.047	1	pDCs
ERCC11	0.00044475	1.99687714	0.595	0.528	1	pDCs
ADGRG1	0.00045044	3.29704594	0.027	0.002	1	pDCs
GCSAM1	0.00045863	3.58817922	0.081	0.014	1	pDCs
GYS12	0.00045924	2.65822469	0.432	0.264	1	pDCs
ZNF587B	0.00046247	2.86246154	0.324	0.153	1	pDCs
RGS15	0.00047026	2.17860072	0.595	0.489	1	pDCs
SLFN12L1	0.00048006	1.91392788	0.081	0.014	1	pDCs
PTPRA	0.00048528	0.23643122	0.162	0.557	1	pDCs
STK17B1	0.00048894	1.16234125	0.703	0.804	1	pDCs
CNN23	0.00049341	1.44504812	0.703	0.803	1	pDCs
NFATC4	0.00052326	5.58735385	0.027	0.002	1	pDCs
IL6R3	0.00053062	2.62409236	0.351	0.179	1	pDCs
MSTN	0.0005364	6.00637378	0.027	0.002	1	pDCs
FUT11	0.00054017	0.13842011	0.216	0.638	1	pDCs
TPPP33	0.00054635	2.23114276	0.054	0.007	1	pDCs
NAGPA1	0.00055326	0.31044076	0.243	0.622	1	pDCs
MT-ND4L3	0.00055383	2.17015053	0.459	0.29	1	pDCs
PABPC1L1	0.00055593	2.99830046	0.27	0.111	1	pDCs
MEF2B1	0.00056756	2.99016641	0.162	0.047	1	pDCs
SNX131	0.00057629	0.16723382	0.189	0.531	1	pDCs
ITGAX3	0.00058254	0.55030106	0.378	0.799	1	pDCs
DHTKD1	0.00059531	2.83379341	0.378	0.209	1	pDCs
ELOVL52	0.00059608	1.75818894	0.595	0.59	1	pDCs
SDC42	0.00061093	1.66359505	0.081	0.388	1	pDCs
MBOAT1	0.00061608	0.21023036	0.108	0.45	1	pDCs
POLDIP3	0.00062565	1.48826893	0.622	0.638	1	pDCs
DEC1	0.00062623	3.43517183	0.027	0.002	1	pDCs
KLF121	0.00064266	2.22299548	0.162	0.048	1	pDCs
SPRY23	0.00064457	4.07154364	0.081	0.015	1	pDCs
KLF132	0.00064696	1.70043863	0.568	0.477	1	pDCs
SRGAP31	0.00064808	4.24492385	0.054	0.007	1	pDCs
SPG72	0.00065143	1.81454876	0.568	0.48	1	pDCs
GDF11	0.00065256	3.93973586	0.027	0.002	1	pDCs
GEMIN72	0.0006621	2.50600463	0.405	0.246	1	pDCs
PTPRE2	0.00068031	2.23247726	0.514	0.415	1	pDCs
CCDC502	0.00068117	2.21718682	0.649	0.669	1	pDCs
TNIP12	0.00069157	1.73495959	0.568	0.492	1	pDCs
RBM62	0.00069469	1.79319602	0.568	0.51	1	pDCs
NKTR2	0.00070712	1.90768457	0.514	0.368	1	pDCs
STK38L3	0.00070964	2.56489901	0.486	0.363	1	pDCs
AOX1	0.00071243	6.18216679	0.027	0.002	1	pDCs
CD320	0.00071344	3.75560514	0.189	0.064	1	pDCs
GDI21	0.00071445	1.27197531	0.676	0.775	1	pDCs
MAPK8IP32	0.00071621	2.59038401	0.27	0.117	1	pDCs
CNPY3	0.00072364	1.06043289	0.73	0.902	1	pDCs

SIPA13	0.00076216	1.41547742	0.676	0.699	1	pDCs
TUBGCP6	0.00076953	1.91296119	0.514	0.403	1	pDCs
L1CAM1	0.00078633	3.65722171	0.027	0.002	1	pDCs
PHLDB31	0.00080484	4.49375983	0.081	0.015	1	pDCs
LAD11	0.00081996	5.09344762	0.027	0.002	1	pDCs
LIMD13	0.00082703	2.05094495	0.541	0.448	1	pDCs
PITPNM2	0.00082704	3.63775162	0.054	0.007	1	pDCs
PLAAT2	0.00083478	5.74111199	0.027	0.002	1	pDCs
MYLK2	0.00083606	5.48069454	0.027	0.002	1	pDCs
CXorf213	0.00086746	2.65137984	0.486	0.366	1	pDCs
GLT1D11	0.00087878	3.4211339	0.378	0.223	1	pDCs
DNAJC51	0.00089669	1.15210934	0.676	0.731	1	pDCs
GNB5	0.00090485	2.96185574	0.243	0.101	1	pDCs
DNAJB13	0.00091626	0.28072389	0.162	0.531	1	pDCs
KDM4C1	0.00092754	1.36609828	0.622	0.596	1	pDCs
HOXB31	0.00094438	3.59154936	0.108	0.025	1	pDCs
PDE7A2	0.00095064	2.54711851	0.405	0.25	1	pDCs
TLR63	0.00096478	3.53295238	0.243	0.102	1	pDCs
KCNC32	0.00096923	2.55033418	0.459	0.322	1	pDCs
ILDR2	0.00099491	4.87608021	0.027	0.002	1	pDCs
ZNF6721	0.00099834	2.51230934	0.459	0.328	1	pDCs
SPHK12	0.00101104	4.78726714	0.081	0.015	1	pDCs
DMPK	0.00104252	3.27100656	0.027	0.002	1	pDCs
PPP1R14B	0.00107605	3.6175479	0.324	0.173	1	pDCs
MAT2A3	0.00107672	0.82197099	0.73	0.838	1	pDCs
SH2D3C1	0.00108915	2.1512866	0.514	0.417	1	pDCs
SLIT1	0.00112078	5.90664626	0.027	0.002	1	pDCs
IQCN2	0.0011284	3.14559629	0.108	0.026	1	pDCs
SCARF2	0.00112945	4.71967651	0.054	0.008	1	pDCs
FUT8	0.0011809	2.61572096	0.297	0.145	1	pDCs
PTPN11	0.00120843	1.12018957	0.73	0.797	1	pDCs
NFX11	0.00124013	2.19907396	0.568	0.488	1	pDCs
PPP1R12B1	0.00124073	2.4421307	0.297	0.143	1	pDCs
CD300A3	0.00125733	2.2614355	0.486	0.361	1	pDCs
LTA	0.00126972	3.74140167	0.027	0.002	1	pDCs
CNTNAP1	0.00128863	5.41160605	0.054	0.008	1	pDCs
DAAM11	0.00129247	3.07538646	0.297	0.149	1	pDCs
CCDC88C	0.00129558	2.27916685	0.162	0.051	1	pDCs
SLC23A31	0.00131988	4.68585223	0.027	0.002	1	pDCs
C6orf1631	0.00132522	3.47406397	0.108	0.026	1	pDCs
SSBP21	0.00133773	3.42390316	0.135	0.038	1	pDCs
TMEM106A3	0.00134857	2.28797494	0.514	0.43	1	pDCs
VPS13C2	0.00135742	0.86720588	0.703	0.778	1	pDCs
PHF20L11	0.00136575	1.86352634	0.541	0.471	1	pDCs
PPME1	0.00138443	0.11988763	0.054	0.32	1	pDCs
BMERB12	0.00138643	4.6214938	0.027	0.002	1	pDCs
MCM51	0.00138844	1.1993089	0.649	0.647	1	pDCs
C1orf1221	0.00139852	0.12947514	0.135	0.468	1	pDCs
NOXRED1	0.00141944	5.24196609	0.027	0.002	1	pDCs
MYO15B3	0.00142345	2.82220857	0.378	0.234	1	pDCs
MAST31	0.00142351	1.29759243	0.649	0.674	1	pDCs
LACTB21	0.00142957	0.16235325	0.162	0.494	1	pDCs

TGFBR3	0.00143377	3.46204381	0.027	0.002	1	pDCs
DYSF3	0.00143811	3.74919703	0.108	0.026	1	pDCs
NADK1	0.00143934	1.5018928	0.676	0.68	1	pDCs
RBM332	0.00145854	1.4814564	0.649	0.72	1	pDCs
RND1	0.00149661	6.83405158	0.027	0.002	1	pDCs
IDH3A1	0.00150784	2.54392234	0.486	0.398	1	pDCs
TOM11	0.00152862	0.19185236	0.162	0.513	1	pDCs
PCNX23	0.00153271	2.36407294	0.243	0.104	1	pDCs
VCAM12	0.00156584	4.60047434	0.027	0.002	1	pDCs
CEP682	0.00159134	3.09089833	0.27	0.129	1	pDCs
CCNL1	0.00160198	1.44517441	0.595	0.54	1	pDCs
LSP13	0.00162978	0.69968693	0.919	0.945	1	pDCs
HSPG21	0.0016396	6.43787686	0.027	0.002	1	pDCs
PPP1R12C	0.0016569	0.61441972	0.162	0.493	1	pDCs
WNT5A1	0.00167255	4.1019372	0.054	0.008	1	pDCs
ANK31	0.00168394	2.56449909	0.081	0.016	1	pDCs
PHC1	0.00170837	2.34568156	0.378	0.223	1	pDCs
CHAF1A1	0.00171841	2.77203426	0.297	0.148	1	pDCs
NFAT51	0.00176192	2.10955779	0.486	0.386	1	pDCs
TRIM81	0.00177593	0.97163782	0.73	0.811	1	pDCs
SLC1A42	0.00178135	3.11334427	0.351	0.211	1	pDCs
NCS11	0.00179997	3.41604129	0.027	0.002	1	pDCs
STARD72	0.00180923	0.11713898	0.162	0.522	1	pDCs
GAL3ST42	0.00181093	4.31569862	0.054	0.008	1	pDCs
SEPTIN5	0.00183505	4.94602579	0.027	0.002	1	pDCs
ABHD14A	0.0018406	0.42116581	0.081	0.366	1	pDCs
TRPC1	0.00189015	6.07851203	0.027	0.002	1	pDCs
TBC1D42	0.00190797	0.29361861	0.189	0.566	1	pDCs
UBR21	0.00192319	1.28127443	0.676	0.716	1	pDCs
APAF13	0.00198326	2.31902266	0.432	0.291	1	pDCs
PREX13	0.00199235	1.71887511	0.568	0.532	1	pDCs
ZDHHC23	0.00200737	3.19307482	0.162	0.054	1	pDCs
HIST1H2BB1	0.00202897	3.18234041	0.081	0.017	1	pDCs
PRKAG1	0.00208827	0.33847777	0.216	0.598	1	pDCs
TERC	0.00209268	5.87466061	0.027	0.002	1	pDCs
THAP41	0.0021367	0.14813105	0.135	0.436	1	pDCs
MAGED1	0.00214066	2.9093672	0.378	0.241	1	pDCs
GPR1622	0.00215564	3.9436607	0.135	0.041	1	pDCs
RGS23	0.00218202	1.81553271	0.568	0.492	1	pDCs
GPRC5C	0.00219854	4.47681049	0.054	0.008	1	pDCs
DDX3X1	0.00223882	1.48244544	0.541	0.454	1	pDCs
KCNMB13	0.00226583	3.81506835	0.189	0.072	1	pDCs
KLHL53	0.00230033	2.46357126	0.378	0.222	1	pDCs
ABCC21	0.00231731	4.87475072	0.027	0.002	1	pDCs
UBE2V1	0.00231888	1.22540426	0.649	0.678	1	pDCs
CDK62	0.00235368	2.27367893	0.081	0.017	1	pDCs
SUB1	0.00237464	2.21098514	0.459	0.342	1	pDCs
HYOU12	0.00237793	1.74638555	0.649	0.69	1	pDCs
GNA153	0.00241304	1.44268716	0.622	0.679	1	pDCs
BTAF12	0.00248957	2.19682962	0.486	0.413	1	pDCs
REXO2	0.00254245	0.27512006	0.351	0.767	1	pDCs
CHAC12	0.00255221	5.11323807	0.027	0.002	1	pDCs

LSM14B	0.00258655	0.32740975	0.189	0.538	1	pDCs
NXF11	0.00260982	1.68642794	0.541	0.477	1	pDCs
MYO18A3	0.00263053	2.25129738	0.405	0.28	1	pDCs
PCCB1	0.00263102	0.348494	0.135	0.433	1	pDCs
NIPSNAP3B2	0.00266696	3.94836538	0.108	0.029	1	pDCs
C6orf622	0.00267967	0.18318782	0.297	0.659	1	pDCs
RNF141	0.00271184	0.16727054	0.162	0.497	1	pDCs
CRELD2	0.00271898	0.21135363	0.162	0.494	1	pDCs
CTPS1	0.00274179	2.71257083	0.324	0.188	1	pDCs
MTA12	0.00275766	1.78540042	0.486	0.39	1	pDCs
NFKB12	0.00278445	1.66672377	0.595	0.569	1	pDCs
KCNH31	0.00279727	4.17676409	0.054	0.009	1	pDCs
NFE2L11	0.00290251	0.11950902	0.378	0.804	1	pDCs
MSC	0.00293219	2.89277091	0.027	0.003	1	pDCs
HDGFL21	0.00294928	1.64686999	0.568	0.538	1	pDCs
CHRNA1	0.00295969	6.18958741	0.027	0.003	1	pDCs
NKD1	0.00296833	2.66380052	0.027	0.003	1	pDCs
UBE2J11	0.00300399	1.72487551	0.622	0.712	1	pDCs
KCTD153	0.00301391	3.838916	0.054	0.009	1	pDCs
SENP81	0.00306451	3.72788354	0.135	0.042	1	pDCs
PIGP1	0.00306468	0.34870385	0.081	0.347	1	pDCs
GAR1	0.00307603	0.23683279	0.135	0.443	1	pDCs
IL1R22	0.00309115	1.8021269	0.027	0.003	1	pDCs
CFLAR2	0.0031332	1.58668866	0.703	0.876	1	pDCs
POLR2K	0.00314018	0.49788363	0.243	0.599	1	pDCs
CCDC138	0.00314246	3.79127942	0.108	0.029	1	pDCs
MPP31	0.00314535	4.00018702	0.081	0.018	1	pDCs
CPS1	0.00323312	5.642745	0.027	0.003	1	pDCs
SLC43A11	0.00323667	3.63132399	0.027	0.003	1	pDCs
ATIC	0.00330135	0.24284253	0.297	0.691	1	pDCs
NEK101	0.00330289	2.82735344	0.027	0.003	1	pDCs
CPSF71	0.00335876	1.41709931	0.622	0.608	1	pDCs
DLGAP43	0.00337242	1.66564988	0.541	0.503	1	pDCs
DNMT3A	0.00339303	0.21667379	0.297	0.697	1	pDCs
TMEM531	0.00344812	0.34876893	0.108	0.377	1	pDCs
ZNF33A2	0.00345407	1.90012151	0.405	0.275	1	pDCs
ENO22	0.00345962	2.17149227	0.054	0.009	1	pDCs
LUZP1	0.00345983	0.29054399	0.108	0.39	1	pDCs
SNX16	0.00349581	3.14649447	0.324	0.189	1	pDCs
CIAO2B1	0.00351279	0.18369235	0.162	0.5	1	pDCs
FKBP112	0.00359893	2.49746347	0.243	0.113	1	pDCs
HGF2	0.00362596	5.42032048	0.027	0.003	1	pDCs
KDM5C1	0.00365255	1.29082813	0.595	0.621	1	pDCs
GRAMD43	0.00365733	2.46734576	0.459	0.351	1	pDCs
LPIN1	0.00366646	2.64461222	0.324	0.191	1	pDCs
CTNS2	0.00367258	2.9779091	0.405	0.286	1	pDCs
SETD1B2	0.0037709	1.17399775	0.595	0.608	1	pDCs
TNFAIP8L22	0.00379748	0.21635883	0.216	0.554	1	pDCs
ARHGEF51	0.00380788	0.870536	0.027	0.239	1	pDCs
MPRIP	0.00381843	1.36883036	0.595	0.608	1	pDCs
CDKN1C2	0.0038303	4.07683238	0.054	0.009	1	pDCs
ABI2	0.00383658	2.43188787	0.378	0.248	1	pDCs

PAK13	0.00390753	2.72832567	0.486	0.429	1	pDCs
SFMBT2	0.00394473	2.19399178	0.486	0.412	1	pDCs
ZNF4693	0.0040203	4.26663811	0.054	0.009	1	pDCs
MAP4K11	0.00408783	1.88792833	0.405	0.286	1	pDCs
POU6F12	0.00409294	2.83195438	0.027	0.003	1	pDCs
ATP6V0A22	0.00410554	1.78102136	0.405	0.291	1	pDCs
NDUFA13	0.00411782	0.26561408	0.243	0.604	1	pDCs
TVP23A3	0.00411843	3.91106421	0.054	0.009	1	pDCs
SNRNP2002	0.00417221	1.15641558	0.649	0.759	1	pDCs
TBL1XR12	0.00421587	1.33925512	0.622	0.669	1	pDCs
OGA2	0.00432549	1.08882669	0.649	0.753	1	pDCs
ESYT22	0.00434643	1.33328448	0.622	0.717	1	pDCs
CDK5	0.0044313	0.43340759	0.081	0.334	1	pDCs
PNRC12	0.0044583	0.95715409	0.811	0.931	1	pDCs
CSNK1D	0.00446131	0.10198281	0.324	0.744	1	pDCs
SLC35F22	0.00449748	2.99458762	0.027	0.003	1	pDCs
BCAT13	0.0046277	2.22704198	0.297	0.16	1	pDCs
SMOX2	0.00463277	3.50782286	0.081	0.019	1	pDCs
SEC31B1	0.00466176	2.99861857	0.162	0.06	1	pDCs
PIM32	0.00468118	2.07653764	0.459	0.378	1	pDCs
STEAP42	0.00469654	4.72092545	0.027	0.003	1	pDCs
SLC9A5	0.00481556	4.63665295	0.027	0.003	1	pDCs
KMT2E2	0.00486798	1.26787842	0.622	0.653	1	pDCs
LRBA1	0.00487189	1.71760081	0.378	0.253	1	pDCs
HP1BP3	0.00487607	0.39452918	0.216	0.564	1	pDCs
BMF2	0.00488358	2.83048411	0.351	0.218	1	pDCs
LRRC8D2	0.00489695	0.14133828	0.243	0.579	1	pDCs
HSDL21	0.00493354	0.29525788	0.297	0.68	1	pDCs
MT-ND13	0.00502533	2.40263577	0.351	0.212	1	pDCs
PRKAR1A2	0.00506076	0.60711187	0.892	0.942	1	pDCs
XRN1	0.00506251	1.28744114	0.595	0.585	1	pDCs
PEAK12	0.00508099	2.42444935	0.351	0.216	1	pDCs
FAM204A	0.00509867	0.13781979	0.189	0.476	1	pDCs
BCL63	0.00513645	2.74646269	0.486	0.449	1	pDCs
RRP121	0.00516084	0.18696826	0.162	0.46	1	pDCs
JOSD1	0.0052307	0.20634483	0.324	0.73	1	pDCs
OVGP12	0.00526594	4.30461162	0.027	0.003	1	pDCs
TNFRSF92	0.00531872	4.78340996	0.027	0.003	1	pDCs
DMAC2	0.00537161	0.46093964	0.108	0.375	1	pDCs
C17orf99	0.00537332	3.84846646	0.054	0.01	1	pDCs
MAP9	0.0054213	3.59099283	0.081	0.019	1	pDCs
ABCD2	0.00550523	2.65724866	0.027	0.003	1	pDCs
MLLT11	0.00552852	1.31412615	0.568	0.549	1	pDCs
IFNAR22	0.00563751	2.15235421	0.514	0.494	1	pDCs
KIAA15221	0.00572339	0.30148805	0.108	0.368	1	pDCs
PTBP31	0.00573406	1.05743862	0.73	0.88	1	pDCs
GNMT	0.00574988	3.40620246	0.081	0.02	1	pDCs
RNF212	0.0057818	4.39598715	0.027	0.003	1	pDCs
DNAJC41	0.00584525	1.54282297	0.568	0.585	1	pDCs
ARHGAP44	0.00586399	1.85271631	0.622	0.643	1	pDCs
CHST113	0.00596926	1.78334359	0.541	0.518	1	pDCs
KLHL23	0.00598713	4.72964201	0.027	0.003	1	pDCs

EGFL8	0.00602655	4.0565299	0.054	0.01	1	pDCs
RALGPS2	0.0060956	3.38767627	0.135	0.046	1	pDCs
PDLIM5	0.00613003	0.6863231	0.162	0.454	1	pDCs
EXOSC41	0.00614967	0.23019841	0.162	0.467	1	pDCs
TRAF12	0.00623619	2.34378463	0.135	0.045	1	pDCs
DNAJC121	0.00624666	5.35384405	0.027	0.003	1	pDCs
MAP3K7	0.00625091	0.32591656	0.216	0.561	1	pDCs
CD2441	0.00628643	2.55169627	0.054	0.01	1	pDCs
UBE2I	0.00635233	0.93837287	0.622	0.758	1	pDCs
PPFIBP21	0.00638787	0.27280394	0.162	0.453	1	pDCs
FOXP12	0.00646342	1.66140832	0.595	0.652	1	pDCs
ST8SIA11	0.00647438	2.91263022	0.027	0.003	1	pDCs
DDR12	0.00655733	3.25113308	0.081	0.02	1	pDCs
CSF2RB3	0.00656619	2.15628925	0.649	0.794	1	pDCs
MT-ND23	0.00658485	2.26958073	0.351	0.215	1	pDCs
MAML11	0.00664015	1.61614181	0.541	0.543	1	pDCs
GJC2	0.0067705	5.34323093	0.027	0.003	1	pDCs
MBNL12	0.00679197	0.80556029	0.784	0.905	1	pDCs
TIPARP1	0.00681356	0.17937665	0.297	0.681	1	pDCs
USP45	0.00687176	2.65614142	0.216	0.1	1	pDCs
ZNF2171	0.00688557	1.01625105	0.649	0.731	1	pDCs
KHDC4	0.00692687	2.05423445	0.351	0.229	1	pDCs
RHOC2	0.00694475	2.05294136	0.541	0.543	1	pDCs
AGTPBP12	0.00696119	0.11369245	0.216	0.534	1	pDCs
GLCCI11	0.00696327	2.80429944	0.162	0.063	1	pDCs
ATXN7L3B	0.00702827	1.51704719	0.541	0.532	1	pDCs
NSMCE1	0.00703574	0.16043155	0.297	0.667	1	pDCs
ZNF431	0.00705673	3.60738	0.108	0.033	1	pDCs
C2orf49	0.00706872	0.47372275	0.054	0.267	1	pDCs
SMYD2	0.00712143	0.28219635	0.189	0.516	1	pDCs
RABGEF11	0.00715193	0.25579225	0.135	0.396	1	pDCs
ATP5F1A	0.00722398	0.28540081	0.324	0.752	1	pDCs
VN1R11	0.00724011	4.38026884	0.054	0.01	1	pDCs
SUPT16H1	0.00725257	0.2494429	0.216	0.544	1	pDCs
TBC1D17	0.00732605	2.56496583	0.162	0.063	1	pDCs
POLE3	0.00735417	0.4914867	0.27	0.608	1	pDCs
DAPP11	0.00736055	1.68386786	0.568	0.575	1	pDCs
TRAM12	0.00738003	0.85919729	0.784	0.891	1	pDCs
GTF3C21	0.00739358	0.52950134	0.162	0.461	1	pDCs
ARHGAP262	0.00743639	0.19764405	0.135	0.407	1	pDCs
NDUFS11	0.00751662	0.45668302	0.297	0.722	1	pDCs
ZC3H12D2	0.00753141	2.57508765	0.135	0.047	1	pDCs
EHBP1L12	0.00754739	1.01945971	0.865	0.95	1	pDCs
SMARCA21	0.00760483	0.94500307	0.649	0.777	1	pDCs
PUM1	0.0076106	0.96097575	0.676	0.797	1	pDCs
TRIM331	0.00764148	0.46805648	0.189	0.492	1	pDCs
MARCH32	0.00769481	3.60352747	0.108	0.033	1	pDCs
ZDHHC71	0.00782696	0.55569138	0.135	0.378	1	pDCs
CGGBP1	0.00785973	0.85215102	0.73	0.8	1	pDCs
HSD17B81	0.00791716	0.36490583	0.108	0.328	1	pDCs
GRK21	0.00797625	0.65016651	0.865	0.941	1	pDCs
EIF12	0.00797984	0.74679256	0.838	0.94	1	pDCs

TREML21	0.0079869	3.45929479	0.081	0.021	1	pDCs
LILRB12	0.00806525	0.14266579	0.27	0.642	1	pDCs
ZDHHC173	0.00810048	2.83323885	0.378	0.273	1	pDCs
MYL92	0.00812169	4.54307714	0.108	0.033	1	pDCs
ELMSAN11	0.00814232	1.36343259	0.649	0.756	1	pDCs
DSE3	0.00820231	0.27122342	0.405	0.859	1	pDCs
KANK13	0.00832015	4.45807814	0.027	0.003	1	pDCs
CDKN2D	0.00860317	1.83949411	0.432	0.333	1	pDCs
EML2	0.00861852	0.34327594	0.189	0.506	1	pDCs
H3F3B2	0.0087033	2.35050974	0.378	0.268	1	pDCs
NCOR22	0.00870552	0.55822676	0.784	0.903	1	pDCs
HMCES	0.00877403	0.17900686	0.27	0.62	1	pDCs
YPEL33	0.00878158	2.07695356	0.351	0.23	1	pDCs
RNMT2	0.00881471	1.99164986	0.432	0.339	1	pDCs
PIEZO11	0.00884391	0.39274426	0.189	0.497	1	pDCs
STT3B1	0.00885256	0.74423816	0.73	0.833	1	pDCs
RNF1671	0.00891551	0.10622213	0.27	0.599	1	pDCs
IL2RG1	0.00891979	0.69617852	0.757	0.891	1	pDCs
SLC7A14	0.00895567	2.77145397	0.27	0.149	1	pDCs
STK17A1	0.00899024	1.92791522	0.351	0.229	1	pDCs
PGGHG1	0.0089957	2.99356261	0.162	0.065	1	pDCs
ARNT2	0.00900349	1.85312983	0.486	0.429	1	pDCs
GON4L1	0.00910018	0.64168806	0.162	0.437	1	pDCs
TBCD	0.00910556	0.22742932	0.162	0.43	1	pDCs
ITPRIP3	0.00922237	0.64081648	0.162	0.448	1	pDCs
USP401	0.00932885	0.11317289	0.108	0.338	1	pDCs
GMPPB	0.00936611	2.04663443	0.486	0.471	1	pDCs
PARN1	0.00937679	0.3436469	0.135	0.387	1	pDCs
CACNB11	0.00938712	2.66857031	0.108	0.034	1	pDCs
PRDM82	0.0093908	3.26313821	0.027	0.003	1	pDCs
ABCC42	0.00939261	2.45217197	0.243	0.123	1	pDCs
PAXX	0.00939353	1.73545741	0.432	0.329	1	pDCs
TAB1	0.00951779	0.15181544	0.108	0.353	1	pDCs
CXorf65	0.00981248	2.23960243	0.108	0.034	1	pDCs
CENPC	0.0099061	2.4448042	0.297	0.177	1	pDCs
MROH6	0.00992294	0.52285128	0.108	0.336	1	pDCs
ARFGAP31	0.00993773	1.53040862	0.568	0.615	1	pDCs
GPR181	0.00995127	4.06301804	0.027	0.003	1	pDCs
IL1R23	1.043E-285	7.37638035	0.046	0.002	1.603E-281	Neu
TAP14	4.459E-170	0.10708891	0.26	0.736	6.854E-166	Neu
ADGRG3	9.97E-152	7.43840517	0.035	0.002	1.532E-147	Neu
MAP3K112	1.496E-148	0.12873568	0.274	0.745	2.3E-144	Neu
TMEM1401	1.265E-139	0.42414705	0.222	0.645	1.945E-135	Neu
HSPA1A1	7.413E-138	0.18100213	0.156	0.533	1.14E-133	Neu
DOK31	1.313E-136	0.12320695	0.245	0.682	2.018E-132	Neu
SRSF5	1.28E-135	0.10831674	0.237	0.666	1.967E-131	Neu
YKT61	1.488E-132	0.14359932	0.254	0.698	2.287E-128	Neu
SIPA14	1.031E-131	0.2034891	0.262	0.704	1.585E-127	Neu
PACSIN22	1.474E-131	0.21336132	0.248	0.677	2.266E-127	Neu
GALNS	1.56E-131	0.18010011	0.218	0.63	2.398E-127	Neu
FURIN3	1.572E-131	0.13982869	0.234	0.663	2.417E-127	Neu
ARHGAP25	3.408E-131	0.18364896	0.202	0.607	5.238E-127	Neu

ITPA1	4.702E-131	0.10192268	0.213	0.631	7.227E-127	Neu
IFIH11	4.467E-130	0.21968031	0.241	0.665	6.866E-126	Neu
DNAJC52	9.402E-130	0.14129111	0.282	0.736	1.445E-125	Neu
SLC12A93	5.278E-129	0.13108512	0.322	0.789	8.113E-125	Neu
MGRN1	2.801E-128	0.14084148	0.245	0.672	4.306E-124	Neu
TUBB63	5.265E-128	0.13888291	0.161	0.534	8.093E-124	Neu
MED251	6.06E-128	0.13450293	0.303	0.764	9.314E-124	Neu
ADAM92	1.608E-127	0.12269442	0.173	0.556	2.472E-123	Neu
DNAJC8	5.488E-127	0.10443209	0.266	0.709	8.435E-123	Neu
PURB	7.565E-127	0.11808811	0.175	0.553	1.163E-122	Neu
SLC19A1	7.71E-127	0.13216481	0.238	0.654	1.185E-122	Neu
PSD4	8.71E-127	0.11665865	0.246	0.672	1.339E-122	Neu
PSMA2	1.706E-125	0.10950319	0.222	0.639	2.622E-121	Neu
KIAA2013	5.393E-125	0.16114328	0.269	0.712	8.29E-121	Neu
HSPA51	6.373E-125	0.11956968	0.215	0.619	9.796E-121	Neu
PHC23	7.165E-125	0.25551564	0.212	0.613	1.101E-120	Neu
TMEM259	1.188E-124	0.11424864	0.314	0.776	1.826E-120	Neu
MRTFA2	1.465E-124	0.15892108	0.286	0.738	2.252E-120	Neu
SAFB1	3.457E-124	0.1123309	0.25	0.681	5.313E-120	Neu
MIDN1	4.857E-124	0.31329057	0.307	0.764	7.466E-120	Neu
HGS2	6.769E-124	0.23733518	0.268	0.704	1.04E-119	Neu
IRAK12	9.03E-124	0.10215568	0.291	0.725	1.388E-119	Neu
CHMP1A	1.265E-123	0.17625487	0.218	0.628	1.944E-119	Neu
TXNDC171	2.842E-123	0.14639722	0.237	0.657	4.368E-119	Neu
PSMD91	3.169E-123	0.11893618	0.211	0.61	4.871E-119	Neu
GSDMD	5.048E-123	0.17100184	0.308	0.761	7.759E-119	Neu
CCR12	7.456E-123	0.13639038	0.155	0.512	1.146E-118	Neu
BCKDK2	9.285E-123	0.12759515	0.273	0.719	1.427E-118	Neu
SH2B21	1.232E-122	0.32384253	0.176	0.549	1.894E-118	Neu
ACSL51	4.107E-122	0.19540481	0.207	0.599	6.312E-118	Neu
ACSS21	4.304E-122	0.10907326	0.22	0.627	6.616E-118	Neu
NADK3	7.124E-122	0.31691492	0.263	0.685	1.095E-117	Neu
ZMPSTE24	9.341E-122	0.16705756	0.259	0.69	1.436E-117	Neu
ELMO2	9.35E-122	0.13218897	0.234	0.648	1.437E-117	Neu
SRA11	2.866E-121	0.12956488	0.189	0.57	4.405E-117	Neu
NIBAN12	4.292E-121	0.43639332	0.277	0.709	6.597E-117	Neu
SNX19	9.159E-121	0.11127744	0.221	0.626	1.408E-116	Neu
ATP2A2	1.494E-120	0.10734017	0.189	0.573	2.297E-116	Neu
STRN41	2.947E-120	0.1345143	0.183	0.559	4.529E-116	Neu
C1RL1	3.461E-120	0.14299904	0.182	0.558	5.32E-116	Neu
PLIN4	3.87E-120	8.24429151	0.017	0.001	5.949E-116	Neu
ACOT92	4.465E-120	0.18446319	0.285	0.728	6.864E-116	Neu
ATP6V1C11	4.989E-120	0.103942	0.217	0.621	7.668E-116	Neu
TRABD2	6.18E-120	0.16318321	0.29	0.733	9.5E-116	Neu
EHMT1	1.538E-119	0.11336223	0.27	0.701	2.364E-115	Neu
R3HDM43	1.572E-119	0.39710795	0.221	0.622	2.417E-115	Neu
FEZ21	2.698E-119	0.10686189	0.247	0.67	4.146E-115	Neu
RNF42	2.887E-119	0.11236967	0.263	0.696	4.437E-115	Neu
CAMKK22	3.305E-119	0.21782386	0.229	0.637	5.08E-115	Neu
CCDC921	3.526E-119	0.12479741	0.164	0.53	5.421E-115	Neu
NSMAF	4.707E-119	0.11775723	0.201	0.588	7.234E-115	Neu
SERTAD24	5.694E-119	0.10681565	0.23	0.633	8.753E-115	Neu

VAV11	1.056E-118	0.14463649	0.27	0.702	1.623E-114	Neu
SGMS11	1.258E-118	0.13118831	0.233	0.638	1.933E-114	Neu
CXXC11	5.591E-118	0.11599693	0.151	0.501	8.594E-114	Neu
MKNK2	8.077E-118	0.13917335	0.191	0.57	1.242E-113	Neu
GRIPAP1	1.094E-117	0.15852204	0.243	0.653	1.682E-113	Neu
KIAA05131	1.117E-117	0.12343561	0.207	0.59	1.718E-113	Neu
SEC22B	1.122E-117	0.12966997	0.336	0.799	1.724E-113	Neu
RBM231	1.331E-117	0.10501541	0.288	0.727	2.046E-113	Neu
GRK62	3.203E-117	0.1230972	0.179	0.548	4.924E-113	Neu
HPS12	3.355E-117	0.15295637	0.276	0.708	5.157E-113	Neu
SBNO23	3.816E-117	0.51756096	0.234	0.639	5.866E-113	Neu
POLR2E	4.22E-117	0.10934381	0.264	0.69	6.486E-113	Neu
UBE2J12	4.542E-117	0.11423625	0.281	0.717	6.981E-113	Neu
DVL32	5.737E-117	0.17669033	0.2	0.582	8.818E-113	Neu
CIAO2A2	9.893E-117	0.11586548	0.26	0.683	1.521E-112	Neu
LGALS82	1.069E-116	0.13927713	0.279	0.711	1.644E-112	Neu
MARK2	1.088E-116	0.15993013	0.224	0.626	1.672E-112	Neu
CLTA	1.158E-116	0.12447844	0.271	0.704	1.78E-112	Neu
BID2	2.363E-116	0.21161545	0.254	0.674	3.632E-112	Neu
IRF32	1.236E-115	0.11994013	0.225	0.623	1.899E-111	Neu
BAG11	1.713E-115	0.20119979	0.256	0.676	2.634E-111	Neu
EGLN2	2.88E-115	0.24740616	0.219	0.614	4.427E-111	Neu
NRBF21	3.081E-115	0.11011613	0.267	0.695	4.736E-111	Neu
NUDT221	3.197E-115	0.1129171	0.212	0.597	4.915E-111	Neu
DCAF81	4.454E-115	0.12717581	0.269	0.692	6.846E-111	Neu
ACAA11	5.308E-115	0.1426306	0.334	0.79	8.159E-111	Neu
ELOVL53	1.125E-114	0.10411267	0.211	0.594	1.729E-110	Neu
MEA1	1.971E-114	0.1265739	0.252	0.664	3.029E-110	Neu
GMIP	2.125E-114	0.11334393	0.305	0.746	3.266E-110	Neu
CDK4	2.188E-114	0.21678375	0.191	0.563	3.363E-110	Neu
SPIN1	2.496E-114	0.15002921	0.292	0.729	3.837E-110	Neu
IFI441	2.927E-114	0.11864287	0.268	0.674	4.499E-110	Neu
MED121	3.216E-114	0.23602532	0.184	0.548	4.944E-110	Neu
HSPA1B1	3.595E-114	0.21923903	0.248	0.657	5.526E-110	Neu
UBXN1	1.573E-113	0.12006031	0.285	0.713	2.418E-109	Neu
OAZ22	2.336E-113	0.22262492	0.174	0.537	3.591E-109	Neu
SMARCD22	2.52E-113	0.21275758	0.196	0.574	3.874E-109	Neu
ANKLE21	2.887E-113	0.11212462	0.172	0.533	4.438E-109	Neu
CLEC2B2	4.952E-113	0.1072419	0.248	0.658	7.612E-109	Neu
IKBKB3	5.843E-113	0.12583673	0.269	0.687	8.981E-109	Neu
IFNAR11	6.351E-113	0.15495886	0.242	0.644	9.762E-109	Neu
SP110	9.154E-113	0.10553411	0.295	0.728	1.407E-108	Neu
E2F42	1.001E-112	0.13570653	0.29	0.727	1.539E-108	Neu
MAEA1	2.534E-112	0.31604461	0.205	0.59	3.896E-108	Neu
BANF11	2.708E-112	0.13939242	0.188	0.558	4.162E-108	Neu
MTMR3	2.742E-112	0.25163313	0.258	0.679	4.215E-108	Neu
WIPF21	2.918E-112	0.21252943	0.215	0.603	4.485E-108	Neu
TPD52L22	4.377E-112	0.54605984	0.239	0.642	6.728E-108	Neu
DTX41	4.508E-112	0.12676882	0.199	0.569	6.929E-108	Neu
WAS	5.405E-112	0.18876665	0.343	0.801	8.308E-108	Neu
PPP6R2	6.644E-112	0.17324681	0.222	0.611	1.021E-107	Neu
NIT11	9.098E-112	0.16460256	0.23	0.624	1.398E-107	Neu

CSNK1E1	1.122E-111	0.12010755	0.154	0.498	1.725E-107	Neu
EIF2B31	1.28E-111	0.10823416	0.173	0.534	1.968E-107	Neu
ITGAV2	1.624E-111	0.10360504	0.184	0.545	2.496E-107	Neu
YME1L11	1.9E-111	0.14244882	0.165	0.517	2.92E-107	Neu
UBE2J2	2.228E-111	0.17203284	0.179	0.541	3.425E-107	Neu
GTPBP1	2.317E-111	0.3760897	0.177	0.536	3.562E-107	Neu
GGA11	4.932E-111	0.24973716	0.174	0.527	7.581E-107	Neu
ANKRD111	6.504E-111	0.10870253	0.288	0.721	9.997E-107	Neu
SMARCB1	8.932E-111	0.21785904	0.185	0.55	1.373E-106	Neu
ATP5MF	1.043E-110	0.11390592	0.25	0.656	1.603E-106	Neu
TXNL11	1.182E-110	0.1481927	0.248	0.658	1.817E-106	Neu
RAB323	1.281E-110	0.12355591	0.169	0.519	1.968E-106	Neu
TMEM245	1.378E-110	0.12816869	0.248	0.658	2.118E-106	Neu
SOCS62	1.46E-110	0.15358461	0.252	0.662	2.243E-106	Neu
RTF1	2.17E-110	0.11718971	0.255	0.666	3.335E-106	Neu
FRAT22	2.404E-110	0.35187904	0.193	0.559	3.695E-106	Neu
ENTPD6	2.72E-110	0.13009701	0.161	0.508	4.181E-106	Neu
SPOP	3.051E-110	0.12145389	0.176	0.532	4.69E-106	Neu
ZMAT2	3.369E-110	0.10331787	0.331	0.785	5.178E-106	Neu
ETFA1	3.915E-110	0.11756568	0.299	0.734	6.018E-106	Neu
DYNLT11	4.994E-110	0.20795041	0.276	0.697	7.676E-106	Neu
GBP2	5.172E-110	0.21690554	0.33	0.775	7.949E-106	Neu
ATP6V0A13	5.683E-110	0.21378415	0.16	0.506	8.736E-106	Neu
ADPRHL21	5.855E-110	0.18681678	0.195	0.56	8.999E-106	Neu
OPA11	8.618E-110	0.10499952	0.204	0.583	1.325E-105	Neu
RAB8B	1.098E-109	0.24292718	0.282	0.707	1.688E-105	Neu
HNRNPA32	1.281E-109	0.14745513	0.254	0.667	1.969E-105	Neu
AP3B11	1.313E-109	0.12071768	0.263	0.674	2.019E-105	Neu
DGUOK	1.352E-109	0.13594557	0.245	0.655	2.078E-105	Neu
RAB22A1	1.451E-109	0.14469858	0.239	0.64	2.231E-105	Neu
USB11	1.559E-109	0.14243315	0.188	0.553	2.396E-105	Neu
EMD	1.856E-109	0.23433233	0.218	0.601	2.853E-105	Neu
RABEP11	2.228E-109	0.13320818	0.204	0.575	3.425E-105	Neu
POLDIP31	3.326E-109	0.11528097	0.244	0.642	5.112E-105	Neu
B4GALT5	4.042E-109	0.20491712	0.227	0.617	6.213E-105	Neu
FBRS	4.291E-109	0.26644259	0.252	0.664	6.596E-105	Neu
FBXW22	7.855E-109	0.18394126	0.238	0.631	1.207E-104	Neu
ARHGAP46	1.013E-108	0.2234265	0.252	0.648	1.558E-104	Neu
IFI163	1.182E-108	0.13031945	0.377	0.833	1.816E-104	Neu
COPRS	1.36E-108	0.24205996	0.229	0.62	2.091E-104	Neu
GALNT121	1.577E-108	0.14954394	0.184	0.541	2.424E-104	Neu
TNIP13	1.649E-108	0.24609975	0.156	0.496	2.535E-104	Neu
NUDCD21	1.808E-108	0.10713648	0.169	0.517	2.778E-104	Neu
CRK2	1.9E-108	0.36723247	0.244	0.649	2.92E-104	Neu
SLC35F5	2.517E-108	0.17606873	0.276	0.696	3.868E-104	Neu
RIT1	2.604E-108	0.16677036	0.186	0.543	4.002E-104	Neu
PRKAG11	3.579E-108	0.12219475	0.216	0.603	5.501E-104	Neu
RNF1812	3.941E-108	0.11283171	0.182	0.542	6.057E-104	Neu
MAP2K31	4.224E-108	0.46931779	0.227	0.615	6.493E-104	Neu
CPSF72	4.795E-108	0.21885697	0.227	0.613	7.371E-104	Neu
CREBBP	5.326E-108	0.10279352	0.229	0.619	8.187E-104	Neu
GIT2	5.649E-108	0.12338775	0.242	0.64	8.683E-104	Neu

IER23	5.893E-108	0.80150595	0.275	0.686	9.058E-104	Neu
PTPN181	6.871E-108	0.17247559	0.204	0.572	1.056E-103	Neu
ANKHD12	1.015E-107	0.2412038	0.29	0.716	1.56E-103	Neu
CYC12	1.016E-107	0.11091083	0.323	0.768	1.562E-103	Neu
GPS11	1.215E-107	0.19213213	0.175	0.526	1.867E-103	Neu
TRAFD11	1.22E-107	0.17128118	0.192	0.557	1.876E-103	Neu
TATDN2	1.253E-107	0.20324152	0.16	0.497	1.926E-103	Neu
EMC2	1.294E-107	0.15660053	0.224	0.612	1.988E-103	Neu
OST4	1.739E-107	0.10394692	0.191	0.553	2.673E-103	Neu
TMEM1651	2.015E-107	0.11415931	0.294	0.723	3.097E-103	Neu
KDM1B3	2.078E-107	0.13684053	0.204	0.572	3.195E-103	Neu
MEF2A3	2.268E-107	0.18654079	0.266	0.682	3.486E-103	Neu
ARAF	2.383E-107	0.16540699	0.26	0.671	3.663E-103	Neu
GTF2F11	2.497E-107	0.15376272	0.207	0.575	3.838E-103	Neu
PREX14	2.501E-107	0.50491423	0.181	0.536	3.844E-103	Neu
TFE31	2.545E-107	0.33348247	0.205	0.582	3.913E-103	Neu
CSNK1G2	3.081E-107	0.1237125	0.195	0.559	4.735E-103	Neu
WDR26	3.085E-107	0.12318173	0.306	0.742	4.742E-103	Neu
PSMD12	3.184E-107	0.18161988	0.208	0.583	4.894E-103	Neu
ZSWIM81	4.682E-107	0.28450855	0.171	0.517	7.196E-103	Neu
MYO5A2	5.625E-107	0.1199314	0.176	0.524	8.647E-103	Neu
MAN2A1	5.923E-107	0.1125909	0.217	0.595	9.104E-103	Neu
TRAPPC1	6.619E-107	0.10721036	0.229	0.619	1.017E-102	Neu
TPRG1L1	6.988E-107	0.1352235	0.174	0.525	1.074E-102	Neu
CPQ2	7.572E-107	0.4371932	0.212	0.587	1.164E-102	Neu
USP19	8.888E-107	0.13661128	0.155	0.491	1.366E-102	Neu
PSMD6	1.007E-106	0.20053699	0.203	0.575	1.548E-102	Neu
IFI27L2	1.034E-106	0.15902254	0.3	0.723	1.59E-102	Neu
MED8	1.156E-106	0.1737278	0.179	0.531	1.777E-102	Neu
CDC342	1.334E-106	0.15607717	0.133	0.454	2.051E-102	Neu
SPAG91	1.923E-106	0.41043791	0.234	0.628	2.956E-102	Neu
CCT6A2	2.025E-106	0.23506414	0.25	0.65	3.113E-102	Neu
DENND4B	2.045E-106	0.21153303	0.209	0.582	3.143E-102	Neu
UQCRC2	2.073E-106	0.15710509	0.234	0.625	3.187E-102	Neu
ZFP91	2.275E-106	0.11287913	0.298	0.725	3.497E-102	Neu
CSNK1D1	2.521E-106	0.35642685	0.307	0.749	3.875E-102	Neu
SP11	2.659E-106	0.16993621	0.299	0.726	4.087E-102	Neu
CPSF2	2.688E-106	0.10517259	0.252	0.65	4.132E-102	Neu
CCNK	2.909E-106	0.24076077	0.223	0.607	4.472E-102	Neu
CKLF1	3.212E-106	0.1163038	0.32	0.755	4.937E-102	Neu
DTNBP1	3.348E-106	0.17360232	0.196	0.565	5.147E-102	Neu
PKD21	3.526E-106	0.18632875	0.241	0.633	5.42E-102	Neu
TAF131	3.766E-106	0.14931396	0.258	0.667	5.789E-102	Neu
UBAP1	3.971E-106	0.2601011	0.209	0.58	6.103E-102	Neu
NDUFA101	4.493E-106	0.1277219	0.318	0.763	6.906E-102	Neu
SAFB2	4.568E-106	0.15371067	0.253	0.655	7.021E-102	Neu
SNRNP701	4.676E-106	0.37247844	0.155	0.488	7.187E-102	Neu
EXOC11	5.757E-106	0.10251336	0.208	0.581	8.848E-102	Neu
PPM1B	6.068E-106	0.13063204	0.234	0.622	9.326E-102	Neu
DCTN4	6.199E-106	0.29149951	0.227	0.616	9.529E-102	Neu
FCHO22	7.588E-106	0.15268471	0.262	0.663	1.166E-101	Neu
CWC15	8.99E-106	0.1197093	0.263	0.677	1.382E-101	Neu

UBAC13	9.35E-106	0.14357878	0.184	0.544	1.437E-101	Neu
SHFL1	1.098E-105	0.11243305	0.146	0.473	1.688E-101	Neu
EWSR11	1.269E-105	0.2343489	0.221	0.604	1.95E-101	Neu
PISD1	1.288E-105	0.2074622	0.159	0.494	1.979E-101	Neu
GBP41	1.589E-105	0.18605427	0.181	0.53	2.443E-101	Neu
EIF4EBP11	1.646E-105	0.10802853	0.27	0.679	2.53E-101	Neu
DHRSX1	1.961E-105	0.25255135	0.168	0.512	3.015E-101	Neu
MAPK131	2.603E-105	0.11196178	0.134	0.448	4.001E-101	Neu
PET1001	3.104E-105	0.24863542	0.187	0.546	4.771E-101	Neu
ANKS1A1	3.531E-105	0.14794845	0.278	0.693	5.428E-101	Neu
DFFA	3.831E-105	0.1032076	0.177	0.527	5.889E-101	Neu
PRKAA1	4.196E-105	0.20533917	0.245	0.637	6.45E-101	Neu
ACSL11	4.504E-105	0.13621911	0.402	0.837	6.922E-101	Neu
PIK3CB1	6.771E-105	0.21687481	0.24	0.632	1.041E-100	Neu
PITPNA2	8.179E-105	0.28113099	0.364	0.815	1.257E-100	Neu
COA3	9.023E-105	0.13591643	0.18	0.533	1.387E-100	Neu
ACER31	9.312E-105	0.19284144	0.272	0.686	1.431E-100	Neu
MTIF31	9.499E-105	0.11867055	0.226	0.614	1.46E-100	Neu
ATXN2L2	1.219E-104	0.19933118	0.178	0.53	1.873E-100	Neu
AFF4	1.269E-104	0.18446253	0.256	0.665	1.951E-100	Neu
PIP4P11	1.285E-104	0.18878653	0.241	0.631	1.976E-100	Neu
ADCY73	1.759E-104	0.12830938	0.281	0.689	2.705E-100	Neu
DDT	2.277E-104	0.14257911	0.207	0.572	3.501E-100	Neu
LRMDA1	2.484E-104	0.18370792	0.165	0.504	3.819E-100	Neu
PITHD11	3.184E-104	0.10563592	0.19	0.542	4.894E-100	Neu
SIDT21	3.445E-104	0.1315909	0.317	0.752	5.296E-100	Neu
SGK14	3.501E-104	0.67781596	0.267	0.662	5.382E-100	Neu
BTBD1	3.792E-104	0.10584626	0.272	0.679	5.829E-100	Neu
GPD21	3.837E-104	0.12947742	0.309	0.738	5.897E-100	Neu
FYB12	4.41E-104	0.35662355	0.181	0.53	6.779E-100	Neu
DOK12	4.444E-104	0.21869109	0.165	0.503	6.831E-100	Neu
PRAM12	6.069E-104	0.11113433	0.347	0.789	9.329E-100	Neu
NFYC1	6.531E-104	0.15478839	0.181	0.53	1.004E-99	Neu
SIN3B1	6.733E-104	0.30701561	0.234	0.614	1.035E-99	Neu
RNF19B2	6.798E-104	0.42564956	0.301	0.722	1.045E-99	Neu
MRFAP1L1	7.168E-104	0.11019951	0.148	0.473	1.102E-99	Neu
LARP7	7.21E-104	0.10803828	0.217	0.594	1.108E-99	Neu
DNM1L2	8.07E-104	0.20370275	0.222	0.6	1.24E-99	Neu
RIOK3	9.35E-104	0.24609985	0.298	0.717	1.437E-99	Neu
FAM53C2	1.214E-103	0.28316148	0.149	0.472	1.867E-99	Neu
GOLGA51	1.418E-103	0.15632905	0.194	0.557	2.179E-99	Neu
SMDT11	1.486E-103	0.12667862	0.281	0.694	2.284E-99	Neu
SNRK1	1.756E-103	0.21442926	0.179	0.526	2.699E-99	Neu
KLF11	1.877E-103	0.10570373	0.257	0.661	2.885E-99	Neu
STK10	1.897E-103	0.15499701	0.347	0.795	2.915E-99	Neu
CRKL	1.963E-103	0.16432117	0.197	0.559	3.017E-99	Neu
KIAA0319L	1.991E-103	0.14609002	0.167	0.506	3.06E-99	Neu
PITPNB	2.12E-103	0.19551775	0.189	0.545	3.258E-99	Neu
MAPRE1	2.201E-103	0.16745726	0.244	0.642	3.383E-99	Neu
C16orf722	2.494E-103	0.17720951	0.242	0.629	3.833E-99	Neu
VHL	2.528E-103	0.3448652	0.248	0.651	3.886E-99	Neu
HSPA9	2.756E-103	0.10201745	0.141	0.465	4.236E-99	Neu

ABCC31	2.884E-103	0.17817157	0.233	0.61	4.433E-99	Neu
URI11	2.915E-103	0.15364212	0.228	0.608	4.48E-99	Neu
FPR11	3.007E-103	0.3114057	0.357	0.79	4.622E-99	Neu
CHCHD101	3.122E-103	0.27768174	0.188	0.541	4.798E-99	Neu
ADI1	3.638E-103	0.105668	0.266	0.664	5.592E-99	Neu
KDM5A	3.707E-103	0.1324177	0.19	0.541	5.699E-99	Neu
PPIF1	5.057E-103	1.31915996	0.152	0.482	7.773E-99	Neu
WRNIP1	5.363E-103	0.1429942	0.213	0.583	8.244E-99	Neu
IKBKG1	6.219E-103	0.31654558	0.272	0.678	9.559E-99	Neu
NPEPL1	6.27E-103	0.22641533	0.234	0.614	9.637E-99	Neu
RBM141	7.071E-103	0.21247495	0.187	0.533	1.0869E-98	Neu
DEDD1	7.117E-103	0.18780362	0.273	0.682	1.094E-98	Neu
TTC7B1	7.319E-103	0.14853988	0.28	0.689	1.1251E-98	Neu
METTL23	7.484E-103	0.12795954	0.209	0.576	1.1503E-98	Neu
EIF2AK2	7.664E-103	0.12286989	0.349	0.789	1.178E-98	Neu
RRAGC3	8.395E-103	0.17509299	0.183	0.532	1.2903E-98	Neu
SH3BP23	9.587E-103	0.50027251	0.256	0.654	1.4736E-98	Neu
FES2	1.07E-102	0.16681605	0.184	0.533	1.6441E-98	Neu
CHD91	1.155E-102	0.18976058	0.212	0.581	1.7759E-98	Neu
CLIP11	1.368E-102	0.12769987	0.26	0.655	2.1022E-98	Neu
NFKB13	1.387E-102	0.36525461	0.206	0.573	2.132E-98	Neu
PSMD101	1.569E-102	0.21403723	0.256	0.654	2.4121E-98	Neu
NDUFS42	1.676E-102	0.11200599	0.282	0.694	2.5764E-98	Neu
SUCLG1	2.465E-102	0.22320983	0.216	0.592	3.7889E-98	Neu
KCTD201	2.752E-102	0.17452078	0.226	0.598	4.2305E-98	Neu
ARHGAP27	3.287E-102	0.50985923	0.173	0.512	5.0529E-98	Neu
ZC3H181	3.938E-102	0.29070906	0.187	0.539	6.0529E-98	Neu
HPS52	4.332E-102	0.12237309	0.355	0.798	6.6585E-98	Neu
RMND5A1	5.361E-102	0.13589392	0.25	0.642	8.2404E-98	Neu
FAF1	5.522E-102	0.10138027	0.191	0.54	8.4875E-98	Neu
CTDSP22	5.856E-102	0.20299307	0.317	0.752	9.002E-98	Neu
ERH	6.095E-102	0.10128783	0.159	0.495	9.3681E-98	Neu
ELOF11	6.236E-102	0.19316289	0.189	0.545	9.5848E-98	Neu
SYAP11	6.78E-102	0.10754272	0.345	0.791	1.0421E-97	Neu
HSPA41	7.653E-102	0.15937882	0.132	0.445	1.1764E-97	Neu
RRP122	7.989E-102	0.37143228	0.145	0.464	1.228E-97	Neu
SEC22C	8.109E-102	0.16716451	0.235	0.622	1.2464E-97	Neu
UBE2W1	8.365E-102	0.16676761	0.212	0.581	1.2857E-97	Neu
ALDH9A1	9.299E-102	0.11378837	0.285	0.699	1.4294E-97	Neu
SNRNP2003	1.02E-101	0.17297574	0.323	0.764	1.5676E-97	Neu
STIMATE1	1.095E-101	0.11349405	0.296	0.709	1.6825E-97	Neu
DR1	1.234E-101	0.21522314	0.274	0.689	1.8964E-97	Neu
OTUD12	1.256E-101	0.2017053	0.239	0.613	1.9306E-97	Neu
ATF62	1.848E-101	0.17755905	0.255	0.649	2.8407E-97	Neu
HEATR33	2.014E-101	0.11535956	0.179	0.522	3.0964E-97	Neu
EIF3J1	2.083E-101	0.14926867	0.22	0.593	3.2016E-97	Neu
AAK1	2.204E-101	0.11039182	0.292	0.709	3.3873E-97	Neu
HNRNPC	2.263E-101	0.24309142	0.286	0.704	3.478E-97	Neu
DLGAP44	2.501E-101	0.12252567	0.168	0.507	3.8446E-97	Neu
DHX161	2.607E-101	0.18296203	0.188	0.538	4.0069E-97	Neu
RFC2	2.697E-101	0.14076775	0.162	0.496	4.1461E-97	Neu
GNL21	2.919E-101	0.10832865	0.209	0.575	4.4866E-97	Neu

MPC21	3.009E-101	0.20345532	0.196	0.554	4.6249E-97	Neu
MBP	3.292E-101	0.20732904	0.189	0.538	5.0607E-97	Neu
FOXP13	3.905E-101	0.15278226	0.258	0.656	6.0021E-97	Neu
CAMK1D3	4.208E-101	0.13496537	0.148	0.466	6.4683E-97	Neu
IP6K1	4.299E-101	0.12043706	0.194	0.549	6.6083E-97	Neu
TCF251	4.908E-101	0.10468212	0.386	0.848	7.5437E-97	Neu
DEDD2	5.193E-101	0.29364591	0.338	0.783	7.9826E-97	Neu
SSH12	5.752E-101	0.12076674	0.133	0.446	8.8408E-97	Neu
PHYH1	6.64E-101	0.11522272	0.197	0.555	1.0206E-96	Neu
NDUFA71	7.043E-101	0.14664202	0.203	0.564	1.0826E-96	Neu
TMEM41B	7.466E-101	0.16300907	0.165	0.497	1.1476E-96	Neu
TMEM243	7.73E-101	0.10836385	0.233	0.618	1.1882E-96	Neu
KHNYN	8.224E-101	0.29819096	0.213	0.576	1.2642E-96	Neu
TMEM1641	8.447E-101	0.14597527	0.137	0.45	1.2984E-96	Neu
PIK3CG2	8.845E-101	0.19125573	0.217	0.583	1.3595E-96	Neu
NEMF	1.311E-100	0.17896566	0.207	0.568	2.0159E-96	Neu
SMG51	1.385E-100	0.1808875	0.226	0.6	2.1291E-96	Neu
CHST114	1.389E-100	0.35234556	0.18	0.522	2.135E-96	Neu
COX7A2	1.484E-100	0.29821004	0.251	0.648	2.2805E-96	Neu
TAOK31	1.699E-100	0.12411234	0.319	0.747	2.6113E-96	Neu
XPO6	1.869E-100	0.37742188	0.163	0.496	2.8734E-96	Neu
GTPBP21	1.888E-100	0.1995125	0.31	0.737	2.9022E-96	Neu
MOSPD31	2.023E-100	0.13528505	0.186	0.528	3.1098E-96	Neu
IDUA2	2.152E-100	0.10694429	0.195	0.544	3.3074E-96	Neu
RNF411	2.318E-100	0.15468145	0.27	0.675	3.5631E-96	Neu
EFNB11	2.326E-100	0.19635694	0.2	0.555	3.5755E-96	Neu
ZNF5921	2.534E-100	0.10203503	0.289	0.706	3.8956E-96	Neu
BCCIP2	2.652E-100	0.13181641	0.213	0.574	4.0762E-96	Neu
SIPA1L21	3.364E-100	0.20420674	0.205	0.56	5.1713E-96	Neu
ACTR8	3.586E-100	0.14231799	0.146	0.462	5.5125E-96	Neu
TCF122	3.677E-100	0.13368155	0.167	0.497	5.6512E-96	Neu
TMEM167B	3.811E-100	0.2738661	0.268	0.67	5.8574E-96	Neu
VPS531	4.044E-100	0.21376404	0.184	0.524	6.2166E-96	Neu
GLYR1	4.095E-100	0.1529096	0.169	0.503	6.2938E-96	Neu
SRRM1	4.113E-100	0.15084742	0.28	0.691	6.3226E-96	Neu
REST	4.752E-100	0.15235269	0.253	0.645	7.3044E-96	Neu
GMPR21	4.964E-100	0.21181543	0.173	0.513	7.6298E-96	Neu
ZNF241	7.264E-100	0.12333387	0.197	0.548	1.1165E-95	Neu
YBX32	7.327E-100	0.12032957	0.297	0.709	1.1262E-95	Neu
TDRD72	7.732E-100	0.14830201	0.217	0.588	1.1885E-95	Neu
NSFL1C1	8.6E-100	0.24119686	0.288	0.7	1.3218E-95	Neu
DUSP232	9.009E-100	0.10850625	0.247	0.634	1.3848E-95	Neu
LPIN2	9.061E-100	0.25251608	0.196	0.55	1.3928E-95	Neu
MIA21	9.289E-100	0.20371116	0.211	0.577	1.4278E-95	Neu
RAB1A1	9.73E-100	0.15043843	0.337	0.78	1.4955E-95	Neu
SRP721	9.989E-100	0.1290614	0.252	0.645	1.5354E-95	Neu
ACTR10	1.237E-99	0.24804224	0.256	0.649	1.9012E-95	Neu
AFF13	1.319E-99	0.15793748	0.287	0.699	2.0279E-95	Neu
STAG22	1.435E-99	0.14598472	0.233	0.614	2.2054E-95	Neu
RNF185	1.736E-99	0.24862658	0.175	0.513	2.6687E-95	Neu
NPEPPS2	2.032E-99	0.24458418	0.259	0.653	3.1233E-95	Neu
DCAF51	2.137E-99	0.25609673	0.288	0.706	3.2844E-95	Neu

DEK	2.149E-99	0.10914493	0.181	0.521	3.3029E-95	Neu
HSDL22	2.297E-99	0.18750001	0.281	0.684	3.5304E-95	Neu
AP1G11	2.319E-99	0.22491693	0.183	0.525	3.5645E-95	Neu
SRSF3	2.362E-99	0.11020451	0.28	0.684	3.6306E-95	Neu
UBE3A2	2.528E-99	0.13835304	0.256	0.652	3.8865E-95	Neu
PLEKHO22	2.568E-99	0.10866194	0.418	0.869	3.9477E-95	Neu
FAM3C	2.712E-99	0.12577033	0.198	0.552	4.1691E-95	Neu
UBE2V11	3.391E-99	0.2554477	0.277	0.683	5.2127E-95	Neu
RALGAPB	3.507E-99	0.18314292	0.173	0.512	5.3902E-95	Neu
ECI2	3.944E-99	0.15342061	0.18	0.517	6.0628E-95	Neu
FOXN22	4.106E-99	0.15162398	0.179	0.515	6.3115E-95	Neu
MTMR6	5.058E-99	0.15133138	0.281	0.688	7.7748E-95	Neu
TRAPPC10	5.188E-99	0.10064899	0.258	0.655	7.9746E-95	Neu
VMA21	5.335E-99	0.13722901	0.219	0.589	8.1997E-95	Neu
SLU7	5.362E-99	0.16164829	0.254	0.648	8.2413E-95	Neu
UBR5	6.139E-99	0.11768078	0.216	0.582	9.4358E-95	Neu
DAP31	6.65E-99	0.2214379	0.241	0.627	1.0222E-94	Neu
ZDHHC4	8.625E-99	0.16903498	0.188	0.534	1.3257E-94	Neu
TMEM258	9.505E-99	0.11298835	0.254	0.647	1.461E-94	Neu
LACTB4	1.0855E-98	0.11841258	0.277	0.674	1.6685E-94	Neu
FEM1B1	1.0885E-98	0.12113493	0.184	0.531	1.6732E-94	Neu
TRMT1L1	1.0914E-98	0.15227691	0.168	0.498	1.6776E-94	Neu
MSRB11	1.1809E-98	0.23651078	0.187	0.529	1.8152E-94	Neu
MGMT	1.232E-98	0.18515683	0.18	0.519	1.8938E-94	Neu
ARID4B	1.4961E-98	0.22904125	0.195	0.545	2.2997E-94	Neu
TLCD21	1.5539E-98	0.10955616	0.189	0.533	2.3885E-94	Neu
RUBCN1	1.6345E-98	0.13458159	0.171	0.505	2.5124E-94	Neu
ITPRID23	1.7566E-98	0.26519447	0.247	0.638	2.7001E-94	Neu
GTF2A2	1.799E-98	0.17779257	0.226	0.602	2.7653E-94	Neu
DGAT11	1.8411E-98	0.1847277	0.173	0.504	2.83E-94	Neu
PPCS	1.8954E-98	0.18918755	0.285	0.696	2.9134E-94	Neu
SLCO3A11	2.1558E-98	0.13255062	0.176	0.511	3.3137E-94	Neu
STXBP3	2.3873E-98	0.15320401	0.281	0.69	3.6695E-94	Neu
AGAP33	2.4426E-98	0.20650893	0.171	0.503	3.7545E-94	Neu
SMIM71	2.648E-98	0.18811278	0.183	0.527	4.0703E-94	Neu
ASPH2	2.9441E-98	0.19798029	0.159	0.482	4.5254E-94	Neu
PATL12	3.1945E-98	0.31236252	0.193	0.541	4.9103E-94	Neu
TBC1D143	3.2082E-98	0.39413734	0.142	0.455	4.9313E-94	Neu
CYLD1	3.9271E-98	0.18913372	0.229	0.6	6.0364E-94	Neu
COX141	3.9934E-98	0.15733192	0.317	0.741	6.1382E-94	Neu
PPIA1	4.0704E-98	0.13348704	0.242	0.609	6.2566E-94	Neu
USO1	4.8477E-98	0.16898573	0.28	0.693	7.4514E-94	Neu
EEA1	5.6982E-98	0.2060799	0.228	0.599	8.7587E-94	Neu
HMGN3	6.1107E-98	0.17453679	0.327	0.757	9.3928E-94	Neu
LSM101	7.1148E-98	0.18494688	0.221	0.586	1.0936E-93	Neu
OSBPL91	8.0397E-98	0.12000536	0.176	0.515	1.2358E-93	Neu
RIPK11	8.4552E-98	0.22766607	0.21	0.57	1.2996E-93	Neu
MICU2	1.0314E-97	0.13343027	0.209	0.568	1.5854E-93	Neu
CLN33	1.0316E-97	0.1112195	0.177	0.512	1.5857E-93	Neu
IREB2	1.0453E-97	0.1354222	0.184	0.524	1.6067E-93	Neu
MBOAT71	1.2265E-97	1.13301275	0.18	0.522	1.8853E-93	Neu
PQBP1	1.4561E-97	0.25412055	0.183	0.522	2.2382E-93	Neu

INPP4A	1.8431E-97	0.14356934	0.25	0.632	2.833E-93	Neu
ERGIC12	1.8499E-97	0.2581339	0.177	0.512	2.8435E-93	Neu
SMARCA5	2.0971E-97	0.18067733	0.294	0.712	3.2235E-93	Neu
CD300A4	2.1939E-97	0.59171751	0.091	0.364	3.3723E-93	Neu
PLD21	2.2045E-97	0.25408202	0.165	0.491	3.3885E-93	Neu
UBE2D12	2.3729E-97	0.19655998	0.176	0.513	3.6473E-93	Neu
RHOU1	2.6626E-97	0.18387884	0.21	0.566	4.0926E-93	Neu
AGGF11	2.7208E-97	0.19747989	0.218	0.58	4.1822E-93	Neu
BTF3L41	3.0627E-97	0.10016782	0.292	0.704	4.7077E-93	Neu
ST3GAL21	3.4849E-97	0.12015709	0.361	0.801	5.3566E-93	Neu
ADAT11	3.6509E-97	0.14540967	0.157	0.474	5.6118E-93	Neu
LRCH42	3.6523E-97	0.33616948	0.263	0.653	5.614E-93	Neu
TNPO1	3.7277E-97	0.1708326	0.136	0.444	5.7298E-93	Neu
PAFAH21	3.9469E-97	0.17452535	0.18	0.514	6.0667E-93	Neu
MIF4GD2	4.6907E-97	0.14017047	0.273	0.67	7.2101E-93	Neu
PDLIM21	4.7665E-97	0.11228944	0.333	0.766	7.3266E-93	Neu
RHEB	4.8881E-97	0.25075706	0.237	0.608	7.5136E-93	Neu
ZNF1061	6.397E-97	0.20271922	0.25	0.63	9.8329E-93	Neu
NUP107	7.2769E-97	0.19366391	0.154	0.472	1.1185E-92	Neu
CCT41	7.3116E-97	0.168995	0.14	0.451	1.1239E-92	Neu
SCAP	7.4392E-97	0.18728969	0.157	0.476	1.1435E-92	Neu
TNFRSF142	7.5884E-97	0.42150387	0.193	0.538	1.1664E-92	Neu
CCDC88B1	7.6623E-97	0.4358168	0.266	0.657	1.1778E-92	Neu
COX171	7.6747E-97	0.27823147	0.212	0.571	1.1797E-92	Neu
ATP11B	8.2518E-97	0.21635097	0.266	0.658	1.2684E-92	Neu
FBXO461	1.0052E-96	0.18965483	0.151	0.462	1.545E-92	Neu
NFU11	1.016E-96	0.16703327	0.276	0.677	1.5616E-92	Neu
HERC5	1.0793E-96	0.10495667	0.176	0.503	1.659E-92	Neu
LYAR	1.0912E-96	0.12466009	0.261	0.648	1.6773E-92	Neu
ITPR21	1.1266E-96	0.26463689	0.276	0.677	1.7317E-92	Neu
DCAF15	1.1471E-96	0.10447393	0.13	0.428	1.7632E-92	Neu
HIGD1A1	1.1894E-96	0.12948437	0.179	0.517	1.8282E-92	Neu
KDM1A	1.2092E-96	0.12055589	0.259	0.649	1.8587E-92	Neu
STMP11	1.2567E-96	0.16755107	0.26	0.65	1.9317E-92	Neu
SLC25A62	1.4071E-96	0.10913759	0.326	0.757	2.1629E-92	Neu
TRIM441	1.4501E-96	0.13955971	0.318	0.748	2.2289E-92	Neu
YTHDC1	1.5445E-96	0.15021653	0.186	0.527	2.374E-92	Neu
GNAI33	1.5638E-96	0.31945777	0.331	0.766	2.4037E-92	Neu
TECPR1	1.5655E-96	0.12514128	0.14	0.446	2.4064E-92	Neu
FAR21	1.6431E-96	0.13415178	0.269	0.66	2.5255E-92	Neu
POLR2K1	1.6769E-96	0.12312325	0.229	0.603	2.5776E-92	Neu
EP3002	1.9606E-96	0.17321438	0.327	0.766	3.0136E-92	Neu
RSRC1	2.0476E-96	0.16707757	0.163	0.487	3.1473E-92	Neu
GPAT41	2.0789E-96	0.13243534	0.163	0.488	3.1955E-92	Neu
PLAU2	2.3503E-96	0.7411353	0.327	0.738	3.6127E-92	Neu
OTUD5	2.3692E-96	0.38399639	0.201	0.554	3.6417E-92	Neu
PLPP32	2.3695E-96	0.56354027	0.164	0.474	3.6421E-92	Neu
DNAJC111	2.9589E-96	0.20596711	0.159	0.481	4.5481E-92	Neu
TNKS2	2.9708E-96	0.12911445	0.203	0.553	4.5664E-92	Neu
PYM1	3.1328E-96	0.2560493	0.171	0.505	4.8154E-92	Neu
SPTY2D1	3.1825E-96	0.15850667	0.132	0.434	4.8918E-92	Neu
UCK1	3.2334E-96	0.2367364	0.14	0.448	4.97E-92	Neu

KLF31	3.3285E-96	0.19780683	0.252	0.635	5.1162E-92	Neu
WASHC3	3.3419E-96	0.15456606	0.218	0.583	5.1369E-92	Neu
TMEM38B1	3.6101E-96	0.13441793	0.16	0.483	5.5491E-92	Neu
DNAJC31	4.2805E-96	0.1038681	0.351	0.79	6.5796E-92	Neu
IFIT5	5.165E-96	0.18779958	0.173	0.505	7.9391E-92	Neu
PDCD61	6.0139E-96	0.12140631	0.322	0.753	9.2439E-92	Neu
PARL1	6.7789E-96	0.22042383	0.193	0.54	1.042E-91	Neu
GATAD11	7.1549E-96	0.15143452	0.169	0.493	1.0998E-91	Neu
TTC39B1	7.4593E-96	0.19793523	0.254	0.634	1.1466E-91	Neu
UTP63	7.5354E-96	0.12491097	0.141	0.449	1.1583E-91	Neu
POLR2G1	7.8093E-96	0.19885359	0.237	0.611	1.2004E-91	Neu
RP2	1.0119E-95	0.16633101	0.303	0.721	1.5553E-91	Neu
COX5B1	1.0271E-95	0.16971218	0.134	0.436	1.5787E-91	Neu
SMS	1.0285E-95	0.17159894	0.345	0.782	1.5809E-91	Neu
CPD1	1.1525E-95	0.52220411	0.197	0.543	1.7715E-91	Neu
IGF2R1	1.2966E-95	0.16977824	0.414	0.861	1.993E-91	Neu
PGM23	1.3044E-95	0.14032639	0.151	0.467	2.0049E-91	Neu
GMFB1	1.5235E-95	0.12093131	0.188	0.524	2.3417E-91	Neu
NUDT32	1.6952E-95	0.23824258	0.31	0.732	2.6057E-91	Neu
ALKBH71	1.7005E-95	0.1392677	0.203	0.553	2.6139E-91	Neu
RUFY12	1.8477E-95	0.11226993	0.349	0.785	2.8401E-91	Neu
FLCN1	1.9084E-95	0.5422688	0.161	0.482	2.9335E-91	Neu
ESRRA2	1.9206E-95	0.22787556	0.218	0.581	2.9521E-91	Neu
ZMYND81	2.0079E-95	0.17975716	0.191	0.533	3.0864E-91	Neu
SP32	2.1949E-95	0.19677006	0.323	0.756	3.3738E-91	Neu
RALB1	2.4937E-95	0.22832495	0.316	0.745	3.8331E-91	Neu
PRKCH	2.6506E-95	0.34363199	0.141	0.449	4.0743E-91	Neu
SPIDR1	3.0478E-95	0.13843743	0.183	0.514	4.6847E-91	Neu
ETF1	4.2382E-95	0.27725547	0.156	0.472	6.5146E-91	Neu
NFKBIE2	5.1839E-95	0.50265297	0.136	0.436	7.9681E-91	Neu
CEPT11	5.6712E-95	0.17073762	0.273	0.665	8.7171E-91	Neu
PTPRJ2	5.8616E-95	0.21863799	0.188	0.528	9.0099E-91	Neu
UQCC2	6.0517E-95	0.23916212	0.163	0.486	9.302E-91	Neu
FAM78A2	6.8783E-95	0.12165372	0.133	0.435	1.0573E-90	Neu
ZC3H15	7.1519E-95	0.19046311	0.275	0.681	1.0993E-90	Neu
MBTPS1	7.2893E-95	0.14289616	0.221	0.584	1.1204E-90	Neu
VCPIP1	7.4146E-95	0.13422158	0.203	0.552	1.1397E-90	Neu
VPS45	8.1927E-95	0.25714154	0.267	0.666	1.2593E-90	Neu
DNAJC42	9.1828E-95	0.14121288	0.227	0.589	1.4115E-90	Neu
PPIE1	9.6032E-95	0.17053671	0.14	0.445	1.4761E-90	Neu
UBN11	9.6775E-95	0.19600733	0.122	0.414	1.4875E-90	Neu
INO80C	9.6862E-95	0.12246602	0.128	0.424	1.4889E-90	Neu
UBE2E21	9.9314E-95	0.25243807	0.295	0.704	1.5266E-90	Neu
ZNF655	1.0066E-94	0.25318447	0.236	0.607	1.5472E-90	Neu
ATM2	1.0744E-94	0.11495667	0.254	0.636	1.6514E-90	Neu
IPO92	1.1502E-94	0.16234285	0.129	0.427	1.768E-90	Neu
SYNRG	1.3713E-94	0.12627553	0.243	0.614	2.1078E-90	Neu
MOB21	1.4121E-94	0.12450036	0.121	0.411	2.1706E-90	Neu
CDC161	1.4142E-94	0.12092826	0.22	0.578	2.1737E-90	Neu
NMD32	1.6324E-94	0.1763446	0.159	0.476	2.5091E-90	Neu
SHOC2	1.6772E-94	0.14791944	0.303	0.716	2.578E-90	Neu
RBM39	2.1026E-94	0.39979921	0.165	0.486	3.2318E-90	Neu

KCMF1	2.2409E-94	0.19644942	0.257	0.645	3.4445E-90	Neu
TRIM25	2.2983E-94	0.56551858	0.303	0.706	3.5327E-90	Neu
SDE21	2.7082E-94	0.10192336	0.112	0.399	4.1627E-90	Neu
NEK71	2.8987E-94	0.12871215	0.284	0.688	4.4556E-90	Neu
USP9X1	3.3768E-94	0.13693005	0.351	0.787	5.1905E-90	Neu
FAM50A1	3.7031E-94	0.19582465	0.359	0.809	5.692E-90	Neu
C18orf32	4.3667E-94	0.15279048	0.315	0.732	6.712E-90	Neu
LSM14B1	4.5308E-94	0.11611299	0.197	0.542	6.9644E-90	Neu
RNF61	4.682E-94	0.11629509	0.19	0.53	7.1968E-90	Neu
UBE2Q1	4.7937E-94	0.1765856	0.314	0.739	7.3683E-90	Neu
IRF12	4.9265E-94	1.08909342	0.252	0.625	7.5725E-90	Neu
DCUN1D1	5.8339E-94	0.16040067	0.199	0.543	8.9674E-90	Neu
AMOTL11	5.8988E-94	0.18742582	0.209	0.559	9.0671E-90	Neu
TOM12	6.1487E-94	0.58553958	0.183	0.517	9.4512E-90	Neu
CASP81	6.2385E-94	0.17795273	0.285	0.69	9.5891E-90	Neu
RASGEF1B3	7.3382E-94	0.1585459	0.272	0.653	1.128E-89	Neu
IER3IP1	7.3714E-94	0.22582759	0.18	0.512	1.1331E-89	Neu
OSER11	7.5155E-94	0.27370805	0.165	0.482	1.1552E-89	Neu
CCNY	7.5374E-94	0.14575464	0.273	0.669	1.1586E-89	Neu
RAB5A1	7.7769E-94	0.30491918	0.288	0.692	1.1954E-89	Neu
MAPK9	7.8798E-94	0.14871538	0.219	0.578	1.2112E-89	Neu
OSBPL82	8.0634E-94	0.24918199	0.285	0.683	1.2394E-89	Neu
GAB22	8.0916E-94	0.39745381	0.161	0.482	1.2438E-89	Neu
PIK3R52	8.8158E-94	0.35680918	0.346	0.779	1.3551E-89	Neu
CRTC2	8.8247E-94	0.22265442	0.161	0.481	1.3565E-89	Neu
ITPRIP4	1.0515E-93	0.62703781	0.147	0.451	1.6163E-89	Neu
DGLUCY	1.0551E-93	0.28423864	0.145	0.451	1.6218E-89	Neu
KRTCAP2	1.0786E-93	0.15998478	0.201	0.548	1.6579E-89	Neu
HTATIP21	1.1295E-93	0.10262145	0.364	0.809	1.7361E-89	Neu
GLTP1	1.1757E-93	0.14340867	0.176	0.509	1.8072E-89	Neu
GOLGA71	1.2657E-93	0.15696552	0.285	0.694	1.9455E-89	Neu
SH3BP42	1.4816E-93	0.17194282	0.213	0.559	2.2774E-89	Neu
PTPRA1	1.84E-93	0.35109821	0.207	0.561	2.8282E-89	Neu
ZCRB1	1.898E-93	0.12143945	0.243	0.611	2.9174E-89	Neu
WAPL	2.0465E-93	0.14720853	0.228	0.593	3.1456E-89	Neu
GGNBP2	2.0844E-93	0.33242937	0.242	0.617	3.2039E-89	Neu
EXOC7	2.1826E-93	0.1415589	0.311	0.731	3.3549E-89	Neu
TERF2	2.1935E-93	0.13472435	0.155	0.47	3.3716E-89	Neu
SYF2	2.2085E-93	0.12578341	0.236	0.608	3.3947E-89	Neu
NT5C21	2.3171E-93	0.43886385	0.31	0.726	3.5616E-89	Neu
ZNF609	2.4324E-93	0.16307181	0.215	0.566	3.7388E-89	Neu
CDC5L1	2.5667E-93	0.12476347	0.2	0.543	3.9453E-89	Neu
CASP91	2.6211E-93	0.17009108	0.191	0.522	4.0288E-89	Neu
ITGAM2	2.7047E-93	0.1819164	0.25	0.604	4.1574E-89	Neu
RNF144B2	2.8881E-93	0.18628317	0.178	0.501	4.4393E-89	Neu
ATG71	2.8911E-93	0.5483508	0.199	0.542	4.444E-89	Neu
TDP2	2.9734E-93	0.34016854	0.262	0.651	4.5704E-89	Neu
DCAF121	3.0163E-93	0.1081508	0.256	0.639	4.6364E-89	Neu
SP1001	3.0875E-93	0.19081992	0.363	0.814	4.7457E-89	Neu
SIRPB21	3.5127E-93	0.24987851	0.237	0.601	5.3994E-89	Neu
IRF74	3.7489E-93	0.4380433	0.302	0.706	5.7624E-89	Neu
VPS37C2	4.829E-93	0.20113035	0.144	0.445	7.4227E-89	Neu

BMPR21	4.9642E-93	0.28600465	0.262	0.654	7.6304E-89	Neu
ZCCHC241	5.5579E-93	0.30159888	0.169	0.489	8.5431E-89	Neu
DHX82	6.196E-93	0.17557662	0.207	0.552	9.5239E-89	Neu
AKNA3	6.2859E-93	0.12248622	0.211	0.557	9.6621E-89	Neu
SCAF11	6.4151E-93	0.26438618	0.323	0.754	9.8606E-89	Neu
INO80E1	6.6148E-93	0.21748451	0.205	0.552	1.0168E-88	Neu
DICER13	7.1196E-93	0.28591132	0.244	0.613	1.0944E-88	Neu
CYP4V2	7.1945E-93	0.1550014	0.156	0.463	1.1059E-88	Neu
EIF4E22	7.4045E-93	0.11331068	0.358	0.8	1.1381E-88	Neu
SOS21	7.6703E-93	0.35134825	0.252	0.63	1.179E-88	Neu
PBX31	7.696E-93	0.2173504	0.241	0.61	1.1829E-88	Neu
CTNNBIP11	7.914E-93	0.19203306	0.129	0.424	1.2165E-88	Neu
EXOC5	7.9973E-93	0.15076699	0.299	0.705	1.2293E-88	Neu
OXSR11	8.1739E-93	0.29449966	0.229	0.596	1.2564E-88	Neu
KDM4B1	8.7433E-93	0.26996232	0.151	0.457	1.3439E-88	Neu
GTPBP42	9.7198E-93	0.10069561	0.139	0.44	1.494E-88	Neu
BLOC1S21	1.036E-92	0.1218366	0.31	0.722	1.5924E-88	Neu
ARRDC21	1.061E-92	0.21850276	0.214	0.568	1.6309E-88	Neu
PPP3CB	1.1237E-92	0.13900375	0.224	0.584	1.7272E-88	Neu
TMEM1281	1.1441E-92	0.2589992	0.221	0.581	1.7586E-88	Neu
CDC123	1.1855E-92	0.1132899	0.224	0.58	1.8223E-88	Neu
ACIN12	1.2097E-92	0.18746404	0.139	0.439	1.8594E-88	Neu
NQO21	1.2733E-92	0.16147536	0.292	0.683	1.9572E-88	Neu
ADIPOR21	1.2835E-92	0.22962246	0.261	0.641	1.9729E-88	Neu
TOMM5	1.4097E-92	0.13254675	0.277	0.673	2.1669E-88	Neu
HIBADH1	1.4355E-92	0.19368846	0.18	0.513	2.2065E-88	Neu
MAPK14	1.6911E-92	0.12550256	0.337	0.763	2.5994E-88	Neu
IFI44L2	1.8384E-92	0.15745166	0.16	0.465	2.8258E-88	Neu
ETS23	1.8644E-92	0.24247012	0.313	0.714	2.8657E-88	Neu
MAP4K43	2.024E-92	0.24454223	0.136	0.435	3.1111E-88	Neu
CMAS	2.0656E-92	0.1224625	0.193	0.534	3.1751E-88	Neu
LY962	2.1545E-92	0.29204769	0.294	0.696	3.3117E-88	Neu
RNF1461	2.3823E-92	0.27800612	0.251	0.628	3.6618E-88	Neu
GALNT14	3.2039E-92	0.26101803	0.242	0.611	4.9247E-88	Neu
ACAP2	3.388E-92	0.31142691	0.301	0.717	5.2077E-88	Neu
SEC14L12	4.3933E-92	0.8218406	0.257	0.645	6.7529E-88	Neu
PCED1A	5.1143E-92	0.23950652	0.193	0.526	7.8612E-88	Neu
PPP6C	5.5423E-92	0.10222217	0.375	0.824	8.519E-88	Neu
NDEL11	6.4581E-92	0.50097899	0.213	0.564	9.9267E-88	Neu
SMIM141	7.0183E-92	0.10335561	0.347	0.783	1.0788E-87	Neu
ARHGEF18	7.1629E-92	0.15336351	0.179	0.505	1.101E-87	Neu
NUP58	7.4567E-92	0.25046153	0.245	0.618	1.1462E-87	Neu
ZNRF22	8.209E-92	0.30110427	0.282	0.681	1.2618E-87	Neu
PDPK11	8.4313E-92	0.21986898	0.125	0.415	1.296E-87	Neu
ZNF410	8.5197E-92	0.21684202	0.306	0.716	1.3096E-87	Neu
TASOR	1.257E-91	0.13895098	0.291	0.693	1.9321E-87	Neu
PLEKHF23	1.339E-91	0.1879153	0.161	0.474	2.0582E-87	Neu
CASP10	1.35E-91	0.19317106	0.158	0.47	2.075E-87	Neu
YARS2	1.3832E-91	0.12945825	0.215	0.566	2.1261E-87	Neu
MED281	1.508E-91	0.28029184	0.274	0.667	2.3179E-87	Neu
STRN	1.5774E-91	0.23184787	0.204	0.547	2.4246E-87	Neu
SNX27	1.651E-91	0.29011886	0.268	0.657	2.5377E-87	Neu

NIT2	1.7017E-91	0.13334914	0.114	0.396	2.6156E-87	Neu
RCC22	1.8625E-91	0.29254477	0.275	0.673	2.8629E-87	Neu
STAM	1.8636E-91	0.2065082	0.149	0.455	2.8646E-87	Neu
PSEN12	1.8851E-91	0.19222667	0.361	0.812	2.8976E-87	Neu
SLC35E1	1.9239E-91	0.18022209	0.151	0.459	2.9572E-87	Neu
ANKRD102	1.999E-91	0.15632082	0.294	0.687	3.0726E-87	Neu
RAF11	2.0039E-91	0.3292305	0.211	0.561	3.0802E-87	Neu
FAM120A2	2.0395E-91	0.13316107	0.376	0.829	3.1349E-87	Neu
LATS21	2.1462E-91	0.23042808	0.189	0.522	3.2989E-87	Neu
GNPAT1	2.1978E-91	0.17631642	0.151	0.46	3.3782E-87	Neu
ARHGAP102	2.3054E-91	0.13985107	0.159	0.467	3.5437E-87	Neu
PRKAR2A1	2.3078E-91	0.1101287	0.139	0.439	3.5473E-87	Neu
THYN1	2.4279E-91	0.14627679	0.172	0.495	3.732E-87	Neu
JOSD11	3.0388E-91	0.14796957	0.318	0.735	4.671E-87	Neu
SF3A3	3.1209E-91	0.11501811	0.189	0.521	4.7971E-87	Neu
ERLEC1	3.1453E-91	0.15437486	0.287	0.693	4.8347E-87	Neu
ABCA71	3.2646E-91	0.32952839	0.174	0.492	5.018E-87	Neu
NBN	3.3961E-91	0.57088788	0.23	0.591	5.2202E-87	Neu
MTHFD1L1	3.7889E-91	0.23309545	0.229	0.58	5.8239E-87	Neu
LDB11	3.9392E-91	0.33813125	0.226	0.585	6.0549E-87	Neu
ST71	3.9717E-91	0.20555859	0.221	0.574	6.1049E-87	Neu
RNF103	4.0029E-91	0.17935745	0.141	0.44	6.1528E-87	Neu
ANO101	4.0959E-91	0.36657077	0.139	0.436	6.2958E-87	Neu
ATXN3	4.43E-91	0.17091708	0.224	0.578	6.8093E-87	Neu
CYSTM1	4.7222E-91	0.61717768	0.154	0.462	7.2584E-87	Neu
KMT2B1	4.9328E-91	0.33457175	0.16	0.474	7.5822E-87	Neu
PRXL2C2	5.0377E-91	0.11162386	0.179	0.502	7.7434E-87	Neu
MAP3K16	5.1373E-91	0.22500532	0.222	0.576	7.8966E-87	Neu
CUX11	5.2796E-91	0.24911624	0.168	0.489	8.1152E-87	Neu
PARP141	6.0738E-91	0.31973708	0.318	0.723	9.336E-87	Neu
MSL11	6.6005E-91	0.32110387	0.243	0.616	1.0146E-86	Neu
DPH31	7.2794E-91	0.24995975	0.259	0.636	1.1189E-86	Neu
UBA6	7.6882E-91	0.29655398	0.234	0.602	1.1818E-86	Neu
C2orf682	7.8743E-91	0.2613961	0.153	0.461	1.2104E-86	Neu
SMAD4	8.3752E-91	0.24762513	0.2	0.538	1.2874E-86	Neu
TFCP22	8.6657E-91	0.29329861	0.216	0.564	1.332E-86	Neu
KLF103	9.2702E-91	0.24010576	0.175	0.49	1.4249E-86	Neu
PRRC2C2	9.9342E-91	0.14842275	0.278	0.668	1.527E-86	Neu
RC3H2	1.137E-90	0.20597357	0.237	0.603	1.7477E-86	Neu
TNRC6B	1.2951E-90	0.20945765	0.171	0.484	1.9907E-86	Neu
MAML21	1.3563E-90	0.20900333	0.224	0.581	2.0847E-86	Neu
STX5	1.5144E-90	0.32781327	0.238	0.604	2.3279E-86	Neu
AFDN3	1.5612E-90	0.1880662	0.195	0.529	2.3998E-86	Neu
CLPTM1L	1.571E-90	0.11938325	0.362	0.794	2.4148E-86	Neu
UBE2H	1.6198E-90	0.33327959	0.153	0.46	2.4897E-86	Neu
STK39	1.7569E-90	0.23651807	0.265	0.646	2.7005E-86	Neu
TXN2	1.8301E-90	0.12516621	0.116	0.396	2.813E-86	Neu
USP241	1.8939E-90	0.13620408	0.172	0.489	2.9111E-86	Neu
CHURC11	2.2128E-90	0.12712464	0.355	0.794	3.4013E-86	Neu
INPP5B2	2.3504E-90	0.23955382	0.153	0.459	3.6128E-86	Neu
TRIM82	2.3516E-90	0.1981461	0.374	0.816	3.6147E-86	Neu
DCTN61	2.3549E-90	0.2488813	0.291	0.691	3.6197E-86	Neu

MARCH62	2.587E-90	0.34327793	0.192	0.525	3.9765E-86	Neu
KLHL27	2.6163E-90	0.37167501	0.263	0.644	4.0215E-86	Neu
MPPE11	2.6522E-90	0.12360823	0.128	0.414	4.0768E-86	Neu
CD142	3.1822E-90	0.3358439	0.404	0.819	4.8914E-86	Neu
PHF1	3.4465E-90	0.17447087	0.178	0.495	5.2977E-86	Neu
PPID	3.4711E-90	0.12710022	0.139	0.435	5.3355E-86	Neu
KAT81	3.473E-90	0.20170668	0.314	0.725	5.3383E-86	Neu
PLAGL12	3.7821E-90	0.40085803	0.173	0.49	5.8134E-86	Neu
MACC11	4.0527E-90	0.14896705	0.335	0.731	6.2295E-86	Neu
AP3S2	4.2117E-90	0.28319191	0.183	0.507	6.4738E-86	Neu
GNPDA12	4.313E-90	0.16872389	0.129	0.416	6.6295E-86	Neu
ETV64	4.3435E-90	0.33180929	0.303	0.711	6.6763E-86	Neu
DDX212	4.4884E-90	0.1664146	0.314	0.728	6.8992E-86	Neu
RBM101	4.5529E-90	0.12438358	0.143	0.439	6.9983E-86	Neu
WBP12	4.9394E-90	0.11486244	0.148	0.451	7.5923E-86	Neu
HNRNPDL	6.1668E-90	0.14827047	0.285	0.681	9.479E-86	Neu
ITFG11	6.4605E-90	0.13696348	0.342	0.768	9.9304E-86	Neu
BBS21	7.9225E-90	0.10700702	0.164	0.473	1.2178E-85	Neu
TIPARP2	9.0999E-90	0.16918932	0.287	0.685	1.3987E-85	Neu
CCNG21	1.0383E-89	0.11197104	0.152	0.451	1.5959E-85	Neu
GAPVD1	1.1499E-89	0.24543945	0.156	0.468	1.7675E-85	Neu
PPP1R12C1	1.2174E-89	0.35776114	0.178	0.497	1.8712E-85	Neu
PHF231	1.2678E-89	0.12997246	0.12	0.401	1.9488E-85	Neu
DELE11	1.4988E-89	0.30965636	0.149	0.45	2.3038E-85	Neu
STAT5B1	1.6267E-89	0.31673985	0.157	0.463	2.5004E-85	Neu
C3orf381	1.754E-89	0.18108555	0.144	0.443	2.696E-85	Neu
PIK3CD4	1.8563E-89	0.11269103	0.181	0.496	2.8533E-85	Neu
CSNK1A11	1.9777E-89	0.18164401	0.147	0.449	3.0399E-85	Neu
RERE2	2.0352E-89	0.17009648	0.183	0.505	3.1282E-85	Neu
EEF1B21	2.1908E-89	0.15082359	0.251	0.629	3.3674E-85	Neu
PPP2R5C	2.2171E-89	0.22073784	0.252	0.627	3.4079E-85	Neu
EXOC2	2.4486E-89	0.1099224	0.197	0.533	3.7637E-85	Neu
FOXO3	2.4691E-89	0.28668538	0.183	0.507	3.7953E-85	Neu
LEMD21	2.5296E-89	0.12844011	0.148	0.449	3.8882E-85	Neu
TCAIM	2.596E-89	0.22773669	0.152	0.456	3.9903E-85	Neu
PPIL2	2.6236E-89	0.12679119	0.153	0.456	4.0327E-85	Neu
TAF81	2.7028E-89	0.23292105	0.138	0.43	4.1545E-85	Neu
MKNK11	2.7032E-89	0.23016721	0.371	0.811	4.1551E-85	Neu
EIF5	2.9189E-89	0.19491485	0.352	0.782	4.4866E-85	Neu
DYM2	3.0666E-89	0.16417133	0.238	0.597	4.7136E-85	Neu
AOAH1	3.1544E-89	0.13009635	0.153	0.452	4.8486E-85	Neu
CHCHD11	3.1796E-89	0.33756075	0.167	0.482	4.8873E-85	Neu
SLC49A42	3.2358E-89	0.24584723	0.226	0.579	4.9737E-85	Neu
HERC31	3.3894E-89	0.27417902	0.289	0.68	5.2099E-85	Neu
GID81	3.4035E-89	0.18863405	0.218	0.57	5.2315E-85	Neu
CLTC	3.7582E-89	0.16917845	0.141	0.438	5.7767E-85	Neu
CMTR2	4.0918E-89	0.23189263	0.198	0.533	6.2895E-85	Neu
TMEM170A	4.5339E-89	0.25261875	0.21	0.553	6.969E-85	Neu
SEC23A1	5.3234E-89	0.15251619	0.158	0.464	8.1826E-85	Neu
VPS391	5.7227E-89	0.17458693	0.264	0.646	8.7964E-85	Neu
CDC401	5.854E-89	0.10079892	0.128	0.416	8.9982E-85	Neu
NOTCH1	5.8741E-89	0.38063991	0.333	0.76	9.0291E-85	Neu

CSF2RB4	5.9623E-89	0.53283911	0.371	0.799	9.1647E-85	Neu
SLC4A1AP	6.3002E-89	0.37053285	0.14	0.437	9.684E-85	Neu
MBD6	6.4517E-89	0.40907496	0.288	0.68	9.917E-85	Neu
FAM222B1	6.7355E-89	0.10658701	0.141	0.435	1.0353E-84	Neu
RNF1681	7.0164E-89	0.21835356	0.323	0.746	1.0785E-84	Neu
PAIP2	7.5887E-89	0.17571556	0.159	0.468	1.1665E-84	Neu
HSBP11	7.9986E-89	0.22165455	0.144	0.443	1.2295E-84	Neu
PRPF40A	8.0143E-89	0.16134803	0.299	0.706	1.2319E-84	Neu
NIPSNAP21	8.89E-89	0.15113513	0.256	0.632	1.3665E-84	Neu
DBR11	9.9296E-89	0.14626823	0.297	0.691	1.5263E-84	Neu
FEM1A	1.0399E-88	0.18690993	0.133	0.422	1.5985E-84	Neu
PPP1R10	1.043E-88	0.1926122	0.207	0.541	1.6031E-84	Neu
NAP1L4	1.051E-88	0.1828546	0.168	0.481	1.6155E-84	Neu
TAB22	1.0768E-88	0.23879276	0.246	0.61	1.6551E-84	Neu
KPNA32	1.1326E-88	0.11168059	0.194	0.526	1.741E-84	Neu
ZBTB44	1.1562E-88	0.15080518	0.189	0.517	1.7773E-84	Neu
TCERG11	1.2262E-88	0.23706298	0.192	0.521	1.8848E-84	Neu
TRIM332	1.3383E-88	0.10456859	0.176	0.495	2.0571E-84	Neu
MAP3K71	1.3395E-88	0.18272546	0.216	0.565	2.059E-84	Neu
SLC35B21	1.3469E-88	0.13103188	0.111	0.381	2.0702E-84	Neu
UBE2B1	1.3575E-88	0.1525173	0.36	0.8	2.0866E-84	Neu
MTHFS	1.3807E-88	0.41884244	0.155	0.46	2.1222E-84	Neu
SEC61B1	1.4483E-88	0.28535342	0.155	0.462	2.2263E-84	Neu
CLDN71	1.4954E-88	0.16718424	0.279	0.658	2.2986E-84	Neu
HNRNPLL2	1.6769E-88	0.12297946	0.167	0.478	2.5775E-84	Neu
BAZ1A1	1.7615E-88	0.45918246	0.196	0.53	2.7075E-84	Neu
TNRC181	1.7945E-88	0.52366982	0.159	0.466	2.7583E-84	Neu
ARIH2	1.8467E-88	0.14288044	0.173	0.486	2.8385E-84	Neu
ARGLU13	1.9612E-88	0.18070626	0.239	0.594	3.0146E-84	Neu
ZXDC	2.3247E-88	0.2095264	0.171	0.484	3.5733E-84	Neu
GNL31	2.3462E-88	0.15115012	0.208	0.547	3.6063E-84	Neu
SNX14	2.515E-88	0.23613474	0.297	0.698	3.8658E-84	Neu
TMEM2511	2.5824E-88	0.25556173	0.265	0.64	3.9694E-84	Neu
CASC32	2.8802E-88	0.31473488	0.293	0.692	4.4271E-84	Neu
RNF1111	2.9546E-88	0.23925805	0.203	0.54	4.5415E-84	Neu
FAM120AOS1	3.0425E-88	0.13405242	0.142	0.436	4.6766E-84	Neu
PMPCB1	3.2188E-88	0.20085077	0.261	0.642	4.9476E-84	Neu
SLC43A23	3.2658E-88	0.16578005	0.418	0.852	5.0198E-84	Neu
P4HA11	4.5777E-88	0.16627338	0.316	0.73	7.0364E-84	Neu
ROCK11	5.1457E-88	0.12036972	0.352	0.789	7.9095E-84	Neu
GAS73	5.5135E-88	0.11457227	0.149	0.444	8.4748E-84	Neu
MORC32	6.1703E-88	0.19770293	0.311	0.726	9.4844E-84	Neu
SLC52A22	6.3576E-88	0.18743421	0.135	0.422	9.7722E-84	Neu
SLC35A1	6.3802E-88	0.21203327	0.176	0.494	9.807E-84	Neu
TMUB2	6.4738E-88	0.33580482	0.311	0.717	9.9509E-84	Neu
ZDHHC202	6.619E-88	0.22557383	0.264	0.643	1.0174E-83	Neu
MTMR101	7.3515E-88	0.2824492	0.295	0.696	1.13E-83	Neu
USP323	7.5649E-88	0.47957578	0.272	0.655	1.1628E-83	Neu
FBXL171	7.5831E-88	0.17645806	0.22	0.568	1.1656E-83	Neu
TAF72	7.6374E-88	0.14075197	0.18	0.492	1.1739E-83	Neu
RASA1	7.7279E-88	0.20694668	0.244	0.607	1.1879E-83	Neu
EIF2AK43	7.7599E-88	0.10986597	0.172	0.485	1.1928E-83	Neu

SMPD41	8.0261E-88	0.11009418	0.119	0.395	1.2337E-83	Neu
ZNF2172	8.2882E-88	0.18762245	0.321	0.736	1.274E-83	Neu
RILPL21	8.6341E-88	0.41157757	0.349	0.78	1.3271E-83	Neu
SMAP11	9.3061E-88	0.20897015	0.175	0.491	1.4304E-83	Neu
MBD41	9.3503E-88	0.1474017	0.135	0.42	1.4372E-83	Neu
POGZ	1.1709E-87	0.15034212	0.173	0.486	1.7998E-83	Neu
HELZ22	1.2663E-87	0.32513005	0.066	0.303	1.9464E-83	Neu
RRP1B1	1.3091E-87	0.11813847	0.139	0.429	2.0122E-83	Neu
PRPF32	1.4234E-87	0.2160617	0.167	0.475	2.1879E-83	Neu
TYK23	1.6083E-87	0.49140269	0.246	0.606	2.4722E-83	Neu
WDFY34	1.8259E-87	0.37038684	0.125	0.408	2.8067E-83	Neu
SSR1	1.9292E-87	0.13380394	0.15	0.449	2.9654E-83	Neu
ARPP19	2.0731E-87	0.21766835	0.167	0.48	3.1866E-83	Neu
TRMO1	2.0949E-87	0.13630053	0.179	0.495	3.22E-83	Neu
IL1RN2	2.1347E-87	0.73217794	0.314	0.702	3.2812E-83	Neu
MAP4K2	2.2317E-87	0.11727909	0.111	0.383	3.4304E-83	Neu
LCMT1	2.235E-87	0.13227984	0.151	0.452	3.4354E-83	Neu
GNA132	2.3835E-87	0.13141174	0.399	0.843	3.6638E-83	Neu
LYPLAL11	2.4787E-87	0.26274014	0.244	0.608	3.8099E-83	Neu
TBK11	2.4843E-87	0.30322085	0.161	0.47	3.8186E-83	Neu
COQ21	2.5005E-87	0.28766801	0.238	0.602	3.8436E-83	Neu
COG4	2.5841E-87	0.16364424	0.157	0.459	3.9719E-83	Neu
CNOT9	2.5937E-87	0.12491617	0.164	0.474	3.9868E-83	Neu
NUP501	2.7257E-87	0.11436258	0.141	0.434	4.1897E-83	Neu
AKAP51	2.7389E-87	0.15520818	0.233	0.583	4.2099E-83	Neu
POLR2H1	2.8788E-87	0.27737569	0.189	0.513	4.4251E-83	Neu
RBMS1	3.2372E-87	0.23593285	0.308	0.715	4.976E-83	Neu
TIAL12	3.4972E-87	0.20068696	0.268	0.651	5.3755E-83	Neu
MTIF2	3.5141E-87	0.19602232	0.199	0.529	5.4015E-83	Neu
WNK12	3.7525E-87	0.10383155	0.388	0.839	5.768E-83	Neu
IGBP11	3.9269E-87	0.15064925	0.271	0.652	6.0361E-83	Neu
PPIG	4.0488E-87	0.21274074	0.307	0.723	6.2234E-83	Neu
CTCF1	4.0548E-87	0.25744078	0.186	0.51	6.2326E-83	Neu
IFNAR23	4.3095E-87	0.34373779	0.18	0.497	6.6241E-83	Neu
SAMD4B1	4.3621E-87	0.36379188	0.164	0.472	6.705E-83	Neu
ANTKMT	4.5148E-87	0.12823625	0.116	0.392	6.9396E-83	Neu
STK382	4.8429E-87	0.17075994	0.2	0.529	7.4441E-83	Neu
HNRNPH32	5.6819E-87	0.31812759	0.347	0.778	8.7336E-83	Neu
TTC171	5.7186E-87	0.14783359	0.155	0.456	8.79E-83	Neu
BZW11	5.9997E-87	0.18110158	0.141	0.435	9.2221E-83	Neu
LCOR2	6.088E-87	0.2935208	0.213	0.551	9.3578E-83	Neu
DIP2B3	6.1655E-87	0.35988613	0.145	0.439	9.477E-83	Neu
CMIP	8.1152E-87	0.10524998	0.411	0.856	1.2474E-82	Neu
TENT22	8.2779E-87	0.18349073	0.255	0.624	1.2724E-82	Neu
JAZF13	8.4512E-87	0.21436603	0.144	0.437	1.299E-82	Neu
RSRC21	8.5631E-87	0.21761238	0.318	0.718	1.3162E-82	Neu
LPGAT13	9.1089E-87	0.13802012	0.141	0.43	1.4001E-82	Neu
GTF3A1	9.3645E-87	0.19503918	0.172	0.487	1.4394E-82	Neu
NTAN1	9.6568E-87	0.10338591	0.14	0.431	1.4843E-82	Neu
ARHGEF111	1.0539E-86	0.26764698	0.164	0.472	1.62E-82	Neu
PNRC21	1.0764E-86	0.13852837	0.163	0.469	1.6545E-82	Neu
GTF2I1	1.1146E-86	0.26059803	0.141	0.432	1.7133E-82	Neu

IMMT	1.2302E-86	0.1252447	0.329	0.745	1.891E-82	Neu
PPP1R15B3	1.2557E-86	0.22844216	0.388	0.83	1.9301E-82	Neu
CNOT11	1.4336E-86	0.25623973	0.144	0.437	2.2036E-82	Neu
TRIP42	1.5761E-86	0.19266199	0.132	0.415	2.4226E-82	Neu
ZFP36L13	1.6739E-86	0.55346872	0.228	0.561	2.573E-82	Neu
ECHDC21	1.7666E-86	0.17505551	0.139	0.423	2.7154E-82	Neu
SNRPB2	1.783E-86	0.21840044	0.288	0.684	2.7407E-82	Neu
SAP30BP1	1.9059E-86	0.33194564	0.138	0.427	2.9296E-82	Neu
CEBPZ1	1.9493E-86	0.1811178	0.185	0.507	2.9963E-82	Neu
ZNF4381	2.2583E-86	0.33457431	0.141	0.432	3.4712E-82	Neu
LSM8	2.4108E-86	0.2984448	0.264	0.642	3.7056E-82	Neu
KLF71	2.8855E-86	0.2766044	0.156	0.456	4.4352E-82	Neu
ABHD3	3.0496E-86	0.23430151	0.206	0.541	4.6876E-82	Neu
DAPP12	3.1084E-86	0.39829489	0.228	0.579	4.778E-82	Neu
AKIRIN1	3.1401E-86	0.25468597	0.218	0.564	4.8267E-82	Neu
PRPF4B1	3.1664E-86	0.21057401	0.182	0.503	4.867E-82	Neu
CWF19L1	3.559E-86	0.12077151	0.144	0.437	5.4705E-82	Neu
NDUFAF81	3.9413E-86	0.23685992	0.172	0.482	6.0581E-82	Neu
HECTD11	3.9606E-86	0.17149265	0.19	0.512	6.0879E-82	Neu
BROX	4.135E-86	0.28279706	0.286	0.677	6.356E-82	Neu
RMC1	4.437E-86	0.23956835	0.189	0.512	6.8201E-82	Neu
MAP3K52	4.4789E-86	0.26740578	0.249	0.608	6.8845E-82	Neu
ANAPC13	4.7052E-86	0.10115678	0.149	0.445	7.2324E-82	Neu
MFF	4.7846E-86	0.25504481	0.268	0.655	7.3544E-82	Neu
GOSR11	5.5015E-86	0.22141998	0.176	0.49	8.4564E-82	Neu
ATN11	5.6935E-86	0.297503	0.315	0.725	8.7514E-82	Neu
ATG13	5.8612E-86	0.28507624	0.159	0.462	9.0093E-82	Neu
GALNT111	6.3488E-86	0.17327631	0.186	0.503	9.7587E-82	Neu
BMP2K2	6.3954E-86	0.29556398	0.18	0.495	9.8304E-82	Neu
RSPH31	6.6255E-86	0.26877211	0.22	0.563	1.0184E-81	Neu
DYNC1LI13	6.8726E-86	0.21566783	0.224	0.571	1.0564E-81	Neu
STK11IP1	7.8049E-86	0.1841598	0.125	0.402	1.1997E-81	Neu
GCNT11	9.1739E-86	0.13144425	0.232	0.582	1.4101E-81	Neu
ACOT41	1.0637E-85	0.10813063	0.174	0.485	1.6351E-81	Neu
PICK12	1.0651E-85	0.14357081	0.117	0.387	1.6371E-81	Neu
BUB31	1.1008E-85	0.12635871	0.125	0.399	1.692E-81	Neu
CUEDC11	1.1252E-85	0.20616247	0.132	0.412	1.7295E-81	Neu
LRRC58	1.1741E-85	0.16700521	0.116	0.386	1.8046E-81	Neu
TNNI21	1.2092E-85	0.17060918	0.169	0.472	1.8586E-81	Neu
PHF21	1.2431E-85	0.1674813	0.166	0.47	1.9108E-81	Neu
DPY30	1.2804E-85	0.2565835	0.168	0.477	1.9681E-81	Neu
INTS42	1.3239E-85	0.3266288	0.188	0.508	2.035E-81	Neu
ZNF384	1.3358E-85	0.28390672	0.193	0.517	2.0532E-81	Neu
CCDC821	1.4E-85	0.16385429	0.148	0.439	2.152E-81	Neu
COQ10B	1.4346E-85	0.14948981	0.165	0.471	2.2051E-81	Neu
HDAC5	1.4841E-85	0.14781187	0.164	0.468	2.2812E-81	Neu
ARIH1	1.7466E-85	0.22659134	0.175	0.487	2.6848E-81	Neu
NUP2141	1.854E-85	0.1703135	0.143	0.432	2.8498E-81	Neu
AGO11	1.8728E-85	0.33327066	0.177	0.491	2.8787E-81	Neu
NAB1	1.8951E-85	0.19111335	0.24	0.599	2.913E-81	Neu
POLR2I	1.9687E-85	0.26749564	0.219	0.564	3.0261E-81	Neu
HAT1	2.0514E-85	0.15004107	0.221	0.566	3.1531E-81	Neu

C17orf492	2.0794E-85	0.24360879	0.113	0.382	3.1962E-81	Neu
CASC4	2.1209E-85	0.24796005	0.187	0.509	3.2601E-81	Neu
CELF1	2.2846E-85	0.22027708	0.341	0.759	3.5117E-81	Neu
SNX132	2.4162E-85	0.17627019	0.205	0.535	3.7139E-81	Neu
SMARCC1	2.431E-85	0.24535519	0.177	0.487	3.7367E-81	Neu
NUP981	2.5856E-85	0.31927584	0.235	0.59	3.9744E-81	Neu
MAP1LC3B	2.728E-85	0.15004351	0.318	0.726	4.1932E-81	Neu
PDGFC1	2.9528E-85	0.17232759	0.285	0.664	4.5387E-81	Neu
DMXL12	3.1255E-85	0.13045688	0.154	0.45	4.8042E-81	Neu
ZYG11B1	3.3873E-85	0.18660266	0.348	0.764	5.2067E-81	Neu
RNFT11	3.4699E-85	0.33112068	0.247	0.61	5.3336E-81	Neu
SLAIN2	3.7382E-85	0.16384798	0.29	0.684	5.746E-81	Neu
CMC2	3.7754E-85	0.26246036	0.248	0.614	5.8032E-81	Neu
MAML12	3.9192E-85	0.3350539	0.213	0.547	6.0242E-81	Neu
DHX36	3.9493E-85	0.18845423	0.305	0.706	6.0705E-81	Neu
TBC1D43	3.9689E-85	0.15771145	0.225	0.569	6.1006E-81	Neu
PPP1CC	4.2424E-85	0.10032286	0.177	0.491	6.521E-81	Neu
NAA501	4.7067E-85	0.24380044	0.141	0.429	7.2346E-81	Neu
PER2	4.7425E-85	0.12415181	0.203	0.526	7.2897E-81	Neu
ABL11	5.4414E-85	0.13157288	0.098	0.354	8.364E-81	Neu
DIDO1	5.7067E-85	0.11530979	0.142	0.429	8.7718E-81	Neu
ARL2BP	5.9535E-85	0.16341341	0.131	0.415	9.1511E-81	Neu
ZNF1481	6.0598E-85	0.26148526	0.214	0.553	9.3145E-81	Neu
PRR142	6.0914E-85	0.25988839	0.141	0.427	9.3632E-81	Neu
PAK14	7.0256E-85	0.36553524	0.143	0.432	1.0799E-80	Neu
RASSF1	7.1109E-85	0.14991136	0.133	0.413	1.093E-80	Neu
ZCCHC21	7.1467E-85	0.31148147	0.265	0.638	1.0985E-80	Neu
POLR2C1	7.2432E-85	0.12443554	0.13	0.41	1.1134E-80	Neu
EIF4G21	7.3847E-85	0.31844339	0.29	0.684	1.1351E-80	Neu
PWWP2B1	7.5269E-85	0.21202923	0.132	0.412	1.157E-80	Neu
ATG3	7.5817E-85	0.17209061	0.37	0.802	1.1654E-80	Neu
GOLGB11	7.7226E-85	0.31704577	0.207	0.535	1.187E-80	Neu
PANK31	8.7133E-85	0.14709545	0.152	0.448	1.3393E-80	Neu
SLC25A36	1.0055E-84	0.14507047	0.18	0.49	1.5455E-80	Neu
TM2D1	1.0776E-84	0.35361802	0.171	0.478	1.6564E-80	Neu
OTUD4	1.2351E-84	0.20315611	0.168	0.476	1.8984E-80	Neu
ABHD152	1.2356E-84	0.2802382	0.12	0.391	1.8992E-80	Neu
GLE11	1.4509E-84	0.12175902	0.171	0.478	2.2302E-80	Neu
UAP11	1.4532E-84	0.15820708	0.173	0.477	2.2338E-80	Neu
SELENOK	1.4575E-84	0.23179957	0.274	0.654	2.2404E-80	Neu
MMADHC1	1.4678E-84	0.15147542	0.176	0.488	2.2561E-80	Neu
AGTPBP13	1.4897E-84	0.39988081	0.209	0.538	2.2898E-80	Neu
MADD1	1.5507E-84	0.21741811	0.153	0.445	2.3836E-80	Neu
SRP19	1.5574E-84	0.39998031	0.173	0.485	2.3938E-80	Neu
TENT5A1	1.8209E-84	0.15014294	0.138	0.422	2.799E-80	Neu
ECPAS	1.9737E-84	0.16313637	0.302	0.701	3.0338E-80	Neu
SETDB12	2.0984E-84	0.15150932	0.137	0.421	3.2255E-80	Neu
IL10RB1	2.1074E-84	0.21460729	0.378	0.814	3.2392E-80	Neu
FBXO9	2.1375E-84	0.22995003	0.197	0.522	3.2856E-80	Neu
ATG5	2.1751E-84	0.24724882	0.173	0.481	3.3433E-80	Neu
SMC6	2.2345E-84	0.16101454	0.185	0.501	3.4347E-80	Neu
DUSP22	2.388E-84	0.30849402	0.171	0.476	3.6706E-80	Neu

MLKL1	2.6887E-84	0.31869956	0.325	0.735	4.1328E-80	Neu
JAK22	3.1268E-84	0.39489837	0.262	0.633	4.8062E-80	Neu
FNDC3A	3.2573E-84	0.27706359	0.3	0.694	5.0069E-80	Neu
PAM16	3.4999E-84	0.15267076	0.145	0.429	5.3797E-80	Neu
UBE2E3	3.8858E-84	0.25930786	0.149	0.443	5.9728E-80	Neu
USP41	4.1442E-84	0.58678365	0.195	0.518	6.37E-80	Neu
CNOT6L	4.2678E-84	0.18077455	0.184	0.498	6.5601E-80	Neu
WASHC41	4.5951E-84	0.23820748	0.339	0.765	7.0631E-80	Neu
FBXO381	5.7648E-84	0.26596035	0.148	0.442	8.8611E-80	Neu
EIF4E1	6.3203E-84	0.27840713	0.185	0.503	9.7149E-80	Neu
ZNF638	6.517E-84	0.21164461	0.218	0.554	1.0017E-79	Neu
CCRL21	6.6348E-84	0.55075198	0.111	0.376	1.0198E-79	Neu
XAF12	7.7345E-84	0.26701961	0.301	0.684	1.1889E-79	Neu
RNF31	7.7855E-84	0.34848326	0.128	0.403	1.1967E-79	Neu
MBOAT11	9.0195E-84	0.20103263	0.158	0.453	1.3864E-79	Neu
RNF19A	9.0671E-84	0.16838362	0.179	0.488	1.3937E-79	Neu
MCM3AP2	9.1978E-84	0.12544265	0.141	0.424	1.4138E-79	Neu
GALNT10	9.821E-84	0.21084882	0.135	0.417	1.5096E-79	Neu
DYRK1A1	9.8369E-84	0.17041264	0.174	0.483	1.512E-79	Neu
SUPT20H	1.0074E-83	0.30715386	0.164	0.468	1.5484E-79	Neu
SCO23	1.0106E-83	0.24222343	0.234	0.575	1.5534E-79	Neu
CCDC159	1.0673E-83	0.24439276	0.14	0.423	1.6406E-79	Neu
COG51	1.0786E-83	0.35859211	0.169	0.475	1.6579E-79	Neu
RAB3GAP21	1.245E-83	0.20650999	0.135	0.417	1.9138E-79	Neu
PDE3B	1.2582E-83	0.22177956	0.176	0.484	1.9339E-79	Neu
EHMT2	1.2621E-83	0.10083063	0.147	0.432	1.94E-79	Neu
JMJD61	1.2622E-83	0.56751782	0.195	0.517	1.9402E-79	Neu
EIF1AD1	1.2661E-83	0.16383095	0.123	0.397	1.9461E-79	Neu
PSMC1	1.2885E-83	0.23591199	0.162	0.463	1.9806E-79	Neu
TMEM1231	1.2899E-83	0.10883941	0.23	0.578	1.9827E-79	Neu
NFATC2IP1	1.3885E-83	0.13436236	0.165	0.466	2.1342E-79	Neu
HIGD2A	1.3905E-83	0.18978343	0.359	0.787	2.1374E-79	Neu
ARL111	1.4535E-83	0.25191019	0.155	0.45	2.2342E-79	Neu
SLC30A9	1.5128E-83	0.23845691	0.182	0.497	2.3254E-79	Neu
TAGAP3	1.6075E-83	0.9586003	0.235	0.577	2.4709E-79	Neu
PPA22	1.6294E-83	0.21171027	0.213	0.55	2.5045E-79	Neu
MED171	1.6738E-83	0.17575647	0.137	0.419	2.5729E-79	Neu
NCBP3	1.854E-83	0.13091527	0.129	0.403	2.8498E-79	Neu
UTP18	1.8643E-83	0.35188416	0.132	0.411	2.8656E-79	Neu
TRMT2A1	2.1046E-83	0.18433427	0.113	0.377	3.235E-79	Neu
WDR41	2.1316E-83	0.20346665	0.175	0.482	3.2764E-79	Neu
TBL3	2.1988E-83	0.21554385	0.104	0.364	3.3798E-79	Neu
SIGLEC111	2.3008E-83	0.11625417	0.159	0.454	3.5365E-79	Neu
RDH101	2.4126E-83	0.14212577	0.24	0.587	3.7083E-79	Neu
RALGDS2	2.4998E-83	0.83542893	0.208	0.538	3.8424E-79	Neu
SPSB31	2.9851E-83	0.1375169	0.155	0.443	4.5884E-79	Neu
PCCA1	3.0523E-83	0.19806254	0.221	0.557	4.6917E-79	Neu
GAS2L31	3.1382E-83	0.15055953	0.135	0.414	4.8238E-79	Neu
GIGYF11	3.1675E-83	0.19226231	0.152	0.438	4.8688E-79	Neu
IDE	3.3319E-83	0.10139374	0.116	0.383	5.1214E-79	Neu
SLK1	3.5806E-83	0.22166112	0.222	0.563	5.5037E-79	Neu
RNF1351	3.9216E-83	0.25269324	0.146	0.433	6.0279E-79	Neu

GPATCH2L2	3.9894E-83	0.39496636	0.268	0.637	6.1321E-79	Neu
ARRDC42	4.1071E-83	0.12731197	0.339	0.74	6.3131E-79	Neu
CRYBG1	4.1669E-83	0.11049007	0.378	0.813	6.4049E-79	Neu
PGM11	4.375E-83	0.14445623	0.35	0.776	6.7248E-79	Neu
TMEM167A1	4.5379E-83	0.22293642	0.302	0.699	6.9752E-79	Neu
HERPUD2	4.687E-83	0.1825089	0.332	0.747	7.2043E-79	Neu
KIF1B3	4.7479E-83	0.24583472	0.15	0.439	7.2979E-79	Neu
SPPL3	5.1322E-83	0.22626914	0.133	0.412	7.8887E-79	Neu
TMEM60	5.1599E-83	0.16080492	0.132	0.411	7.9313E-79	Neu
LILRB22	5.3564E-83	0.734225	0.149	0.437	8.2333E-79	Neu
METRNL3	5.4248E-83	0.16419692	0.42	0.861	8.3385E-79	Neu
GTF2H11	5.5848E-83	0.42415628	0.193	0.516	8.5844E-79	Neu
PELI31	5.6514E-83	0.23350444	0.274	0.642	8.6867E-79	Neu
LSM72	5.8805E-83	0.20468901	0.157	0.453	9.039E-79	Neu
NDRG13	5.9164E-83	0.12493915	0.119	0.379	9.0942E-79	Neu
ZNF5891	5.9575E-83	0.21319786	0.187	0.492	9.1572E-79	Neu
GGA31	6.2737E-83	0.35766645	0.201	0.525	9.6432E-79	Neu
PCTP1	6.311E-83	0.25990932	0.125	0.399	9.7006E-79	Neu
NOP141	7.1288E-83	0.21235131	0.171	0.477	1.0958E-78	Neu
CBL1	8.2183E-83	0.30251979	0.294	0.685	1.2632E-78	Neu
STARD73	9.0976E-83	0.25778684	0.2	0.525	1.3984E-78	Neu
EIF4G3	9.7158E-83	0.28025699	0.142	0.426	1.4934E-78	Neu
USP310	1.0436E-82	0.32994058	0.265	0.638	1.604E-78	Neu
RBM193	1.0527E-82	0.14854865	0.132	0.405	1.6182E-78	Neu
FAM126A1	1.1395E-82	0.28902105	0.196	0.515	1.7515E-78	Neu
HBP1	1.2444E-82	0.24569539	0.275	0.65	1.9127E-78	Neu
TRIM41	1.3413E-82	0.17820799	0.191	0.507	2.0617E-78	Neu
MAFK1	1.3586E-82	0.25008267	0.141	0.421	2.0884E-78	Neu
EXOC6	1.3695E-82	0.19173609	0.128	0.399	2.105E-78	Neu
CHIC21	1.4713E-82	0.13788197	0.151	0.44	2.2615E-78	Neu
DDX181	1.48E-82	0.23551642	0.224	0.567	2.2748E-78	Neu
PDE4A	1.4925E-82	0.16113819	0.315	0.716	2.2942E-78	Neu
TP53I111	1.5525E-82	0.1691157	0.111	0.374	2.3863E-78	Neu
COPS21	1.5585E-82	0.1604313	0.209	0.543	2.3956E-78	Neu
IQSEC31	1.6874E-82	0.12971243	0.172	0.465	2.5937E-78	Neu
PPP4R3A1	1.7106E-82	0.12367021	0.214	0.546	2.6294E-78	Neu
ACYP21	1.7739E-82	0.16689433	0.209	0.537	2.7266E-78	Neu
WSB23	1.8245E-82	0.23788295	0.362	0.781	2.8044E-78	Neu
ELP21	1.8665E-82	0.16287984	0.183	0.492	2.869E-78	Neu
NCOA1	2.0148E-82	0.14840225	0.227	0.57	3.097E-78	Neu
MARK31	2.1044E-82	0.17375261	0.169	0.468	3.2347E-78	Neu
ARHGAP93	2.5141E-82	0.53085363	0.194	0.51	3.8644E-78	Neu
GMPPA1	2.5318E-82	0.18005846	0.102	0.356	3.8916E-78	Neu
ITCH	2.6749E-82	0.19610325	0.337	0.753	4.1116E-78	Neu
PRPF38B	2.8143E-82	0.26355852	0.26	0.622	4.3259E-78	Neu
RELT3	2.9333E-82	0.67280218	0.294	0.681	4.5088E-78	Neu
MMGT11	3.0593E-82	0.25513165	0.204	0.534	4.7025E-78	Neu
DPP81	3.1187E-82	0.10946758	0.18	0.49	4.7937E-78	Neu
TRIM342	3.3058E-82	0.28112746	0.134	0.41	5.0814E-78	Neu
C15orf392	3.5799E-82	0.89038098	0.221	0.56	5.5027E-78	Neu
TIMM10B1	3.8349E-82	0.20785473	0.147	0.433	5.8946E-78	Neu
PKIA	3.8356E-82	0.23137058	0.139	0.419	5.8957E-78	Neu

GDAP21	3.8982E-82	0.2309941	0.21	0.545	5.9919E-78	Neu
SC5D2	4.668E-82	0.13913714	0.176	0.477	7.1752E-78	Neu
AKAP91	4.7798E-82	0.22349471	0.277	0.651	7.347E-78	Neu
CXorf381	5.0808E-82	0.3447808	0.264	0.638	7.8097E-78	Neu
TPP21	5.5541E-82	0.20314037	0.16	0.453	8.5373E-78	Neu
DDHD11	6.1341E-82	0.16999872	0.35	0.762	9.4287E-78	Neu
SMYD21	6.3827E-82	0.22996751	0.199	0.52	9.8108E-78	Neu
PPP2R5E1	6.9471E-82	0.34706613	0.209	0.541	1.0678E-77	Neu
TRAPPC111	7.1943E-82	0.19655094	0.177	0.484	1.1058E-77	Neu
NLK1	7.3192E-82	0.27906866	0.266	0.635	1.125E-77	Neu
NTPCR	7.5377E-82	0.17436419	0.11	0.369	1.1586E-77	Neu
OGFRL12	7.5713E-82	0.24012503	0.287	0.673	1.1638E-77	Neu
RABGAP11	7.6194E-82	0.19604698	0.168	0.466	1.1712E-77	Neu
NUP153	7.952E-82	0.26846818	0.182	0.494	1.2223E-77	Neu
DCUN1D31	8.1808E-82	0.17096721	0.123	0.39	1.2575E-77	Neu
GOLGA21	9.2001E-82	0.19487818	0.085	0.327	1.4142E-77	Neu
CSGALNACT23	1.0039E-81	0.20878755	0.188	0.5	1.5431E-77	Neu
TPK12	1.1036E-81	0.10493718	0.143	0.426	1.6964E-77	Neu
CSDE1	1.2113E-81	0.13670879	0.343	0.765	1.8618E-77	Neu
NXT1	1.2529E-81	0.13344823	0.142	0.424	1.9258E-77	Neu
INO80B	1.2533E-81	0.22903191	0.14	0.418	1.9264E-77	Neu
PPM1A1	1.2896E-81	0.25585753	0.231	0.577	1.9822E-77	Neu
KIAA2026	1.3091E-81	0.19426815	0.212	0.539	2.0122E-77	Neu
AVL92	1.3515E-81	0.15429131	0.154	0.443	2.0774E-77	Neu
TMX31	1.3772E-81	0.20502291	0.148	0.435	2.1169E-77	Neu
OCRL1	1.4425E-81	0.31516532	0.167	0.468	2.2172E-77	Neu
KANSL12	1.5271E-81	0.34033559	0.27	0.642	2.3474E-77	Neu
PDZD11	1.5676E-81	0.21510068	0.146	0.432	2.4095E-77	Neu
PDE12	1.6507E-81	0.15568131	0.131	0.404	2.5373E-77	Neu
SRFBP11	1.7357E-81	0.2813772	0.172	0.474	2.668E-77	Neu
PCM1	1.7382E-81	0.19657661	0.347	0.77	2.6717E-77	Neu
FAM102B1	1.8652E-81	0.22834924	0.3	0.698	2.867E-77	Neu
USP81	1.8739E-81	0.18925684	0.149	0.435	2.8804E-77	Neu
MUS811	2.0413E-81	0.23827995	0.174	0.478	3.1377E-77	Neu
CR12	2.0737E-81	0.37649067	0.18	0.485	3.1874E-77	Neu
CASP41	2.1163E-81	0.39957643	0.331	0.745	3.253E-77	Neu
UBXN112	2.1202E-81	0.17423861	0.371	0.792	3.2589E-77	Neu
USF11	2.1874E-81	0.40216544	0.298	0.694	3.3623E-77	Neu
SIGLEC51	2.2355E-81	0.23589109	0.169	0.46	3.4362E-77	Neu
CHPT1	2.6023E-81	0.18253911	0.151	0.439	4E-77	Neu
CC2D1B	2.6157E-81	0.12348655	0.122	0.387	4.0206E-77	Neu
MPHOSPH8	2.6406E-81	0.20010553	0.231	0.576	4.0589E-77	Neu
FAM91A13	2.6696E-81	0.44110936	0.23	0.579	4.1034E-77	Neu
UBR22	2.7728E-81	0.28822224	0.318	0.721	4.2621E-77	Neu
KBTBD21	2.8024E-81	0.21740723	0.196	0.513	4.3076E-77	Neu
CACUL12	2.8456E-81	0.36822682	0.33	0.744	4.374E-77	Neu
MTFR1L1	3.3734E-81	0.22108046	0.135	0.411	5.1853E-77	Neu
SPG73	3.4236E-81	0.28729799	0.179	0.484	5.2624E-77	Neu
SURF1	3.5308E-81	0.20716078	0.314	0.715	5.4272E-77	Neu
IBTK1	3.6447E-81	0.30884139	0.237	0.586	5.6023E-77	Neu
PTK2B	3.6964E-81	0.12640215	0.433	0.884	5.6817E-77	Neu
LPP2	4.2145E-81	0.15978225	0.299	0.688	6.4781E-77	Neu

R3HDM2	4.3843E-81	0.30885679	0.253	0.613	6.7391E-77	Neu
SETX	4.7526E-81	0.16640758	0.334	0.746	7.3052E-77	Neu
FXR21	4.9778E-81	0.12287417	0.124	0.391	7.6514E-77	Neu
SPTLC12	5.0528E-81	0.29913582	0.138	0.418	7.7667E-77	Neu
NFAM12	5.8788E-81	0.3524372	0.128	0.392	9.0364E-77	Neu
ZADH2	6.2129E-81	0.22611631	0.163	0.456	9.5499E-77	Neu
YTHDF31	6.7795E-81	0.3169327	0.299	0.692	1.0421E-76	Neu
CAP11	7.3695E-81	0.21358538	0.223	0.554	1.1328E-76	Neu
INPP5F1	7.762E-81	0.3855536	0.159	0.449	1.1931E-76	Neu
PPM1F2	8.3E-81	0.24476126	0.107	0.361	1.2758E-76	Neu
UFL11	1.0878E-80	0.15765821	0.125	0.393	1.672E-76	Neu
NIPSNAP3A	1.2274E-80	0.19176343	0.175	0.478	1.8866E-76	Neu
PTPN221	1.23E-80	0.18198106	0.22	0.554	1.8906E-76	Neu
RUFY32	1.2924E-80	0.25345425	0.14	0.417	1.9865E-76	Neu
MLLT62	1.3575E-80	0.29963817	0.158	0.446	2.0867E-76	Neu
EXOSC101	1.4146E-80	0.23538551	0.206	0.532	2.1745E-76	Neu
ATP6V1D1	1.4602E-80	0.22981629	0.257	0.618	2.2445E-76	Neu
CPT1A2	1.6136E-80	0.16107448	0.136	0.409	2.4803E-76	Neu
DNAJB141	1.6335E-80	0.13227523	0.136	0.409	2.5108E-76	Neu
ORMDL1	1.677E-80	0.1576675	0.239	0.585	2.5777E-76	Neu
YY1AP1	1.7354E-80	0.24091848	0.14	0.417	2.6676E-76	Neu
ARHGEF6	1.9706E-80	0.17880558	0.211	0.538	3.029E-76	Neu
TBC1D151	2.0178E-80	0.33344939	0.295	0.684	3.1016E-76	Neu
MX22	2.3161E-80	0.68924573	0.181	0.484	3.56E-76	Neu
PCGF5	2.324E-80	0.27777361	0.216	0.546	3.5721E-76	Neu
KIF13A1	2.3491E-80	0.44221902	0.244	0.594	3.6108E-76	Neu
UBA21	2.4049E-80	0.28058414	0.176	0.479	3.6966E-76	Neu
DOCK12	2.472E-80	0.24585074	0.137	0.414	3.7998E-76	Neu
AZI21	2.512E-80	0.16508571	0.317	0.71	3.8613E-76	Neu
ITSN22	2.6737E-80	0.32335974	0.274	0.645	4.1097E-76	Neu
ATP13A33	2.7129E-80	0.46542997	0.128	0.395	4.17E-76	Neu
OXR1	2.9379E-80	0.17845198	0.237	0.584	4.5158E-76	Neu
ASNSD1	3.0618E-80	0.12988327	0.129	0.398	4.7064E-76	Neu
PPP1R15A	3.0886E-80	0.8732391	0.357	0.771	4.7475E-76	Neu
TNFAIP31	3.3649E-80	0.85264542	0.21	0.535	5.1722E-76	Neu
STT3B2	3.4128E-80	0.12992877	0.397	0.838	5.2457E-76	Neu
RWDD11	5.033E-80	0.24217889	0.18	0.485	7.7362E-76	Neu
PSMG2	5.1098E-80	0.1395904	0.362	0.788	7.8543E-76	Neu
ELF12	5.143E-80	0.13472783	0.406	0.847	7.9052E-76	Neu
ASAP12	5.37E-80	0.2672637	0.327	0.729	8.2543E-76	Neu
GPR137B2	5.4178E-80	0.19645906	0.367	0.787	8.3276E-76	Neu
DGKQ1	5.6441E-80	0.18184024	0.119	0.379	8.6756E-76	Neu
RRAGD1	5.9265E-80	0.24610976	0.308	0.694	9.1097E-76	Neu
GCC21	6.1379E-80	0.24359634	0.171	0.468	9.4346E-76	Neu
ERICH11	6.1672E-80	0.33646646	0.272	0.632	9.4797E-76	Neu
ZMYND152	6.5033E-80	0.41693432	0.232	0.57	9.9962E-76	Neu
KLHL211	6.5294E-80	0.80284053	0.129	0.399	1.0036E-75	Neu
SEC24A2	7.1544E-80	0.29875317	0.155	0.442	1.0997E-75	Neu
MYO61	7.4259E-80	0.17133936	0.307	0.701	1.1414E-75	Neu
SLF21	7.4551E-80	0.12105879	0.146	0.424	1.1459E-75	Neu
SRRM21	7.6526E-80	0.36030202	0.235	0.577	1.1763E-75	Neu
ADSS	7.6794E-80	0.25593827	0.298	0.684	1.1804E-75	Neu

SLC37A1	7.6964E-80	0.22536378	0.137	0.41	1.183E-75	Neu
GPN1	8.0534E-80	0.1599678	0.094	0.339	1.2379E-75	Neu
BTBD7	8.1671E-80	0.22757796	0.177	0.478	1.2554E-75	Neu
TBRG1	8.4925E-80	0.25457667	0.233	0.574	1.3054E-75	Neu
CRLF31	8.5467E-80	0.19467966	0.149	0.432	1.3137E-75	Neu
SS181	9.0124E-80	0.27090723	0.177	0.48	1.3853E-75	Neu
TLK1	9.0578E-80	0.20492947	0.226	0.565	1.3923E-75	Neu
ZNF32	9.1691E-80	0.20668237	0.17	0.467	1.4094E-75	Neu
RTRAF1	9.1972E-80	0.21995439	0.154	0.443	1.4137E-75	Neu
KLHL181	9.2755E-80	0.19345029	0.136	0.409	1.4257E-75	Neu
PSME4	9.6613E-80	0.25850609	0.294	0.673	1.485E-75	Neu
MED142	1.0246E-79	0.30918524	0.159	0.448	1.5749E-75	Neu
EPC2	1.0703E-79	0.13451853	0.158	0.444	1.6452E-75	Neu
CNST1	1.1124E-79	0.13566623	0.151	0.433	1.7099E-75	Neu
ODF3B3	1.1269E-79	0.5012776	0.209	0.53	1.7322E-75	Neu
EHD1	1.203E-79	0.88445907	0.196	0.509	1.8492E-75	Neu
NUMB2	1.3111E-79	0.34958068	0.355	0.783	2.0152E-75	Neu
PRMT22	1.346E-79	0.18952827	0.371	0.794	2.069E-75	Neu
RBCK11	1.381E-79	0.28150677	0.101	0.349	2.1228E-75	Neu
PAG1	1.3997E-79	0.17378487	0.375	0.788	2.1515E-75	Neu
CCNC1	1.4239E-79	0.21585861	0.133	0.406	2.1887E-75	Neu
ASB81	1.4373E-79	0.11748442	0.09	0.333	2.2092E-75	Neu
FMR11	1.5338E-79	0.2709528	0.173	0.472	2.3577E-75	Neu
UFM1	1.6138E-79	0.18506227	0.363	0.785	2.4806E-75	Neu
WDR371	1.632E-79	0.40714121	0.153	0.437	2.5086E-75	Neu
RIPK23	1.6444E-79	0.3515512	0.242	0.59	2.5276E-75	Neu
RAB11FIP2	1.6696E-79	0.17591024	0.223	0.556	2.5663E-75	Neu
SLC38A24	1.6939E-79	0.2628598	0.289	0.669	2.6036E-75	Neu
CALCOCO2	1.8252E-79	0.12225836	0.38	0.809	2.8055E-75	Neu
PHF3	1.9823E-79	0.36816032	0.23	0.574	3.047E-75	Neu
AMD11	2.2799E-79	0.21714249	0.12	0.38	3.5045E-75	Neu
TPMT1	2.4844E-79	0.26868332	0.145	0.424	3.8188E-75	Neu
EDEM31	2.5138E-79	0.31409471	0.24	0.588	3.864E-75	Neu
GLRX5	2.5933E-79	0.2009873	0.116	0.374	3.9862E-75	Neu
FAM118B2	2.6511E-79	0.24725828	0.165	0.458	4.075E-75	Neu
APIP1	2.7077E-79	0.14664783	0.321	0.717	4.162E-75	Neu
KLF162	3.0331E-79	0.22397674	0.143	0.419	4.6622E-75	Neu
PCIF11	3.0587E-79	0.14867506	0.157	0.442	4.7016E-75	Neu
VPS361	3.0653E-79	0.16922547	0.225	0.559	4.7116E-75	Neu
NOTCH22	3.8973E-79	0.38203396	0.179	0.479	5.9905E-75	Neu
GPBP11	4.0771E-79	0.36653586	0.221	0.554	6.267E-75	Neu
BORCS7	4.1596E-79	0.19569838	0.109	0.365	6.3937E-75	Neu
TRAF3IP21	4.4239E-79	0.14802536	0.132	0.402	6.7999E-75	Neu
PHKB2	4.4443E-79	0.31206622	0.169	0.463	6.8314E-75	Neu
FAM49A2	4.5066E-79	0.28515757	0.18	0.48	6.9271E-75	Neu
MOB3C1	4.5263E-79	0.16960911	0.077	0.307	6.9574E-75	Neu
EIF3L1	4.5995E-79	0.19658511	0.345	0.756	7.0699E-75	Neu
FAR12	4.7759E-79	0.2986292	0.306	0.696	7.3411E-75	Neu
CYTH12	4.9065E-79	0.26017388	0.397	0.837	7.5417E-75	Neu
NUFIP23	5.0087E-79	0.19691612	0.356	0.777	7.6988E-75	Neu
LONRF31	5.148E-79	0.29728883	0.287	0.66	7.9129E-75	Neu
PAK1IP1	5.7255E-79	0.20470408	0.119	0.382	8.8007E-75	Neu

C6orf623	6.2122E-79	0.36907965	0.283	0.663	9.5487E-75	Neu
RNF8	6.4899E-79	0.16252999	0.119	0.378	9.9757E-75	Neu
AKT3	6.784E-79	0.1748071	0.147	0.428	1.0428E-74	Neu
OGT3	7.2596E-79	0.17003197	0.224	0.552	1.1159E-74	Neu
IL1RAP	7.4249E-79	0.59433205	0.247	0.599	1.1413E-74	Neu
TANK	7.6712E-79	0.64261331	0.271	0.639	1.1791E-74	Neu
GPA331	7.7913E-79	0.12367776	0.138	0.411	1.1976E-74	Neu
BBX1	8.5003E-79	0.16640251	0.119	0.379	1.3066E-74	Neu
MGME11	9.6218E-79	0.17271715	0.111	0.363	1.479E-74	Neu
C1QBP2	9.996E-79	0.13075748	0.101	0.349	1.5365E-74	Neu
XPO51	1.1136E-78	0.2902824	0.157	0.445	1.7117E-74	Neu
CSRNP11	1.1567E-78	0.87707571	0.246	0.585	1.7779E-74	Neu
ATP2B11	1.2996E-78	0.48212092	0.285	0.663	1.9976E-74	Neu
ZFYVE162	1.3033E-78	0.39262921	0.293	0.672	2.0032E-74	Neu
LILRA51	1.3523E-78	0.19653439	0.237	0.562	2.0786E-74	Neu
PDLIM51	1.4297E-78	0.26503483	0.164	0.457	2.1976E-74	Neu
DSTN1	1.5364E-78	0.27952289	0.287	0.666	2.3616E-74	Neu
OTULIN2	1.7977E-78	0.18368452	0.145	0.418	2.7633E-74	Neu
TRA2A2	1.7979E-78	0.21854943	0.346	0.756	2.7635E-74	Neu
TGIF11	1.9806E-78	0.12528666	0.382	0.796	3.0443E-74	Neu
MOB41	2.0075E-78	0.22601735	0.146	0.424	3.0857E-74	Neu
FCGR3B2	2.1147E-78	0.66978954	0.093	0.334	3.2505E-74	Neu
MKLN1	2.3401E-78	0.31838948	0.178	0.478	3.5969E-74	Neu
TIA11	2.53E-78	0.3972156	0.233	0.57	3.8888E-74	Neu
VMP13	2.7101E-78	0.34916186	0.341	0.747	4.1656E-74	Neu
GMFG	2.8388E-78	0.14378579	0.398	0.833	4.3636E-74	Neu
TMX12	2.8699E-78	0.18701659	0.25	0.601	4.4114E-74	Neu
SLC12A6	3.1463E-78	0.42974775	0.322	0.719	4.8362E-74	Neu
IQCE1	3.2848E-78	0.25586894	0.111	0.362	5.0491E-74	Neu
DGCR21	3.3003E-78	0.10623152	0.1	0.344	5.0729E-74	Neu
LPCAT1	3.3369E-78	1.24976618	0.16	0.45	5.1292E-74	Neu
HHEX1	3.6053E-78	0.12004577	0.151	0.431	5.5418E-74	Neu
CD832	4.0474E-78	0.10603077	0.47	0.876	6.2213E-74	Neu
ANKRD401	4.4149E-78	0.30908605	0.234	0.574	6.7861E-74	Neu
ARHGEF281	5.182E-78	0.28534882	0.281	0.649	7.9652E-74	Neu
AP5Z11	5.3675E-78	0.53238052	0.101	0.348	8.2505E-74	Neu
TTC3	5.4413E-78	0.2922038	0.233	0.572	8.3638E-74	Neu
ZC2HC1A	5.528E-78	0.25483141	0.146	0.423	8.4972E-74	Neu
BTN3A1	5.7404E-78	0.19523433	0.096	0.339	8.8236E-74	Neu
TRIO2	5.9346E-78	0.22784756	0.367	0.788	9.1221E-74	Neu
RAD21	6.0845E-78	0.17241636	0.132	0.401	9.3524E-74	Neu
ALPK11	6.515E-78	0.17307259	0.114	0.368	1.0014E-73	Neu
PNO1	6.5501E-78	0.30033984	0.12	0.382	1.0068E-73	Neu
NFATC31	7.1351E-78	0.10949481	0.201	0.514	1.0967E-73	Neu
SGK31	7.9196E-78	0.28596414	0.349	0.764	1.2173E-73	Neu
ZC3H7A	7.9298E-78	0.11201115	0.144	0.42	1.2189E-73	Neu
ROCK21	7.9306E-78	0.14738442	0.166	0.457	1.219E-73	Neu
MFSD112	8.0176E-78	0.24931916	0.128	0.392	1.2324E-73	Neu
PPP1R21	8.4162E-78	0.20057141	0.136	0.403	1.2937E-73	Neu
AKAP17A3	8.5083E-78	0.39359802	0.186	0.489	1.3078E-73	Neu
UBP1	1.0043E-77	0.35321071	0.27	0.637	1.5437E-73	Neu
ATXN21	1.0277E-77	0.10875962	0.135	0.403	1.5797E-73	Neu

RMND5B	1.0799E-77	0.13574432	0.102	0.349	1.6599E-73	Neu
FEM1C1	1.0861E-77	0.31996561	0.145	0.421	1.6694E-73	Neu
FBXO281	1.149E-77	0.2263173	0.151	0.428	1.7661E-73	Neu
MAN1A2	1.1607E-77	0.16108177	0.145	0.417	1.784E-73	Neu
UBE3C1	1.1862E-77	0.3145985	0.248	0.602	1.8232E-73	Neu
DARS2	1.3138E-77	0.19378486	0.094	0.335	2.0194E-73	Neu
ATP9B	1.3243E-77	0.25470317	0.313	0.694	2.0356E-73	Neu
UBE2F1	1.3877E-77	0.14321969	0.151	0.43	2.133E-73	Neu
GLDN1	1.5911E-77	0.12272996	0.369	0.768	2.4457E-73	Neu
LRSAM1	1.6039E-77	0.23034094	0.106	0.352	2.4654E-73	Neu
ZNF3161	1.6843E-77	0.47570794	0.104	0.349	2.589E-73	Neu
MIER11	2.2105E-77	0.24203894	0.261	0.62	3.3977E-73	Neu
RAB4A1	2.2246E-77	0.24377987	0.319	0.712	3.4195E-73	Neu
CREB11	2.4014E-77	0.24145462	0.161	0.447	3.6912E-73	Neu
TDRD31	2.4982E-77	0.19765849	0.144	0.412	3.8399E-73	Neu
DOCK72	2.5194E-77	0.3377849	0.185	0.486	3.8726E-73	Neu
RAP2C2	2.5958E-77	0.30902279	0.332	0.734	3.9899E-73	Neu
CERT11	2.6848E-77	0.20556016	0.41	0.84	4.1269E-73	Neu
ZDHHC18	2.8726E-77	0.81056332	0.149	0.429	4.4154E-73	Neu
TAF112	2.956E-77	0.10848489	0.099	0.339	4.5436E-73	Neu
ZMYM41	3.0159E-77	0.23383674	0.153	0.435	4.6357E-73	Neu
N4BP2L11	3.2568E-77	0.27573362	0.188	0.49	5.006E-73	Neu
SNX302	3.4085E-77	0.49632931	0.201	0.519	5.2392E-73	Neu
NLRC41	3.9263E-77	0.16951092	0.379	0.802	6.0352E-73	Neu
RBM252	4.0127E-77	0.3155798	0.305	0.69	6.1679E-73	Neu
RNF169	4.4412E-77	0.33211594	0.177	0.474	6.8266E-73	Neu
PIM33	5.035E-77	1.44078344	0.12	0.381	7.7393E-73	Neu
EVI51	5.1763E-77	0.44639634	0.255	0.61	7.9565E-73	Neu
HUS1	5.5492E-77	0.31508403	0.229	0.561	8.5298E-73	Neu
ZNF6871	5.9132E-77	0.14775418	0.11	0.359	9.0892E-73	Neu
ALG131	5.9676E-77	0.24065888	0.227	0.558	9.1728E-73	Neu
GBGT14	6.1306E-77	0.31231353	0.146	0.419	9.4233E-73	Neu
AP5M1	6.2024E-77	0.23354685	0.207	0.522	9.5337E-73	Neu
LAMTOR31	6.8225E-77	0.21383224	0.331	0.734	1.0487E-72	Neu
KRR1	7.0708E-77	0.21020226	0.142	0.414	1.0869E-72	Neu
JPT21	8.9898E-77	0.14189514	0.095	0.333	1.3818E-72	Neu
FARSB1	9.3647E-77	0.13977331	0.166	0.455	1.4394E-72	Neu
FAM177A11	9.8593E-77	0.21146045	0.104	0.348	1.5155E-72	Neu
LIMK21	1.037E-76	1.02108418	0.292	0.666	1.594E-72	Neu
CPTP	1.0509E-76	0.27025187	0.094	0.333	1.6154E-72	Neu
TAB3	1.0587E-76	0.30235565	0.149	0.425	1.6273E-72	Neu
NACC2	1.0692E-76	0.11789704	0.086	0.318	1.6435E-72	Neu
UNC1191	1.1073E-76	0.19808082	0.116	0.37	1.7021E-72	Neu
IQGAP12	1.1162E-76	0.11372477	0.411	0.85	1.7157E-72	Neu
SIAH13	1.1464E-76	0.36607838	0.184	0.483	1.7621E-72	Neu
DNMT3A1	1.1799E-76	0.29129893	0.314	0.701	1.8136E-72	Neu
UCKL11	1.2664E-76	0.163976	0.122	0.378	1.9465E-72	Neu
DDX561	1.2691E-76	0.2436423	0.122	0.38	1.9507E-72	Neu
OXLD1	1.3918E-76	0.10132654	0.114	0.364	2.1393E-72	Neu
EPS15L11	1.4336E-76	0.28540312	0.124	0.385	2.2035E-72	Neu
RSF1	1.4712E-76	0.14267558	0.094	0.332	2.2614E-72	Neu
AMPD21	1.4806E-76	0.54878994	0.124	0.382	2.2758E-72	Neu

XPO71	1.5001E-76	0.26988474	0.142	0.415	2.3058E-72	Neu
GSKIP	1.6243E-76	0.1941054	0.112	0.364	2.4967E-72	Neu
SH3TC13	1.6292E-76	0.26035239	0.197	0.505	2.5043E-72	Neu
ELL22	1.6431E-76	0.50922986	0.256	0.603	2.5257E-72	Neu
APOL41	1.6627E-76	0.15334884	0.256	0.6	2.5558E-72	Neu
PRR14L1	1.7078E-76	0.26003056	0.188	0.491	2.625E-72	Neu
CTDSP11	1.7113E-76	0.27594608	0.359	0.775	2.6304E-72	Neu
POM121	1.9487E-76	0.16104661	0.151	0.426	2.9954E-72	Neu
NAT91	2.0283E-76	0.16698106	0.082	0.311	3.1177E-72	Neu
ATP5MC2	2.0482E-76	0.12095176	0.408	0.853	3.1484E-72	Neu
RIOK11	2.3115E-76	0.19764368	0.119	0.377	3.553E-72	Neu
GRAMD1A2	2.3279E-76	0.44273969	0.149	0.422	3.5782E-72	Neu
FNTA	2.4753E-76	0.22520175	0.282	0.653	3.8047E-72	Neu
ERBIN	3.0567E-76	0.33578385	0.337	0.743	4.6985E-72	Neu
SLC25A40	3.1158E-76	0.35562356	0.191	0.497	4.7893E-72	Neu
CDK141	3.4253E-76	0.39895293	0.211	0.535	5.265E-72	Neu
PSPC1	3.7624E-76	0.36447269	0.185	0.483	5.7832E-72	Neu
GTF3C3	3.7846E-76	0.21247335	0.136	0.402	5.8172E-72	Neu
FLAD11	3.7954E-76	0.10148782	0.123	0.38	5.834E-72	Neu
OSTC2	3.9487E-76	0.22413473	0.136	0.403	6.0695E-72	Neu
ASH1L2	4.0485E-76	0.30750304	0.258	0.606	6.2229E-72	Neu
ARMH31	4.0517E-76	0.15954963	0.129	0.39	6.2278E-72	Neu
SF11	4.7816E-76	0.17234566	0.143	0.413	7.3498E-72	Neu
NDUFV2	5.1134E-76	0.13856209	0.382	0.813	7.8598E-72	Neu
TMCO4	6.0008E-76	0.16976382	0.086	0.316	9.2238E-72	Neu
SART3	6.3723E-76	0.38868526	0.185	0.487	9.7948E-72	Neu
PLEKHG3	6.648E-76	0.146812	0.106	0.346	1.0219E-71	Neu
FOPNL1	6.6764E-76	0.18753852	0.138	0.405	1.0262E-71	Neu
TLR42	7.2325E-76	0.32036588	0.327	0.724	1.1117E-71	Neu
SRPK11	7.3419E-76	0.20132091	0.127	0.385	1.1285E-71	Neu
MAPKAPK5	7.5882E-76	0.37834857	0.205	0.518	1.1664E-71	Neu
SVBP1	7.7176E-76	0.10708076	0.162	0.446	1.1863E-71	Neu
RNGTT1	8.0221E-76	0.24033988	0.151	0.426	1.2331E-71	Neu
UBQLN11	8.1552E-76	0.26913936	0.187	0.489	1.2535E-71	Neu
ABCB71	8.3461E-76	0.16536164	0.151	0.425	1.2829E-71	Neu
RMND11	8.4744E-76	0.23233145	0.153	0.432	1.3026E-71	Neu
PCMTD11	9.1537E-76	0.16669667	0.352	0.766	1.407E-71	Neu
ZBTB451	9.3813E-76	0.15011905	0.095	0.332	1.442E-71	Neu
CREM1	9.4179E-76	0.44993158	0.216	0.528	1.4476E-71	Neu
QSER1	9.6977E-76	0.33478775	0.237	0.572	1.4906E-71	Neu
GNG121	1.0147E-75	0.11508105	0.122	0.378	1.5597E-71	Neu
DHX321	1.0342E-75	0.17158949	0.13	0.392	1.5896E-71	Neu
ANAPC41	1.1418E-75	0.32001817	0.16	0.442	1.7551E-71	Neu
TPM43	1.1609E-75	0.14879856	0.403	0.828	1.7844E-71	Neu
PIK3C31	1.2763E-75	0.20126581	0.125	0.386	1.9618E-71	Neu
PLCB23	1.379E-75	0.22488028	0.413	0.844	2.1197E-71	Neu
FAM49B3	1.3911E-75	0.21663236	0.405	0.832	2.1382E-71	Neu
IL183	1.6207E-75	0.30178116	0.252	0.598	2.4912E-71	Neu
PXK2	1.8234E-75	0.21604332	0.141	0.41	2.8028E-71	Neu
TCF20	1.8399E-75	0.18424666	0.093	0.328	2.8281E-71	Neu
HP1BP31	1.8533E-75	0.28253248	0.232	0.568	2.8488E-71	Neu
HIF1AN	1.8848E-75	0.18277475	0.13	0.389	2.8972E-71	Neu

LRCH3	1.9239E-75	0.11213851	0.129	0.389	2.9573E-71	Neu
SAMSN12	2.0607E-75	0.17526069	0.264	0.6	3.1675E-71	Neu
FBRSL14	2.0783E-75	0.32992491	0.096	0.333	3.1946E-71	Neu
AMDHD21	2.2592E-75	0.37247049	0.088	0.32	3.4726E-71	Neu
TRAPPC91	2.5412E-75	0.22434106	0.161	0.441	3.9061E-71	Neu
RB13	2.6187E-75	0.30197695	0.157	0.436	4.0251E-71	Neu
REV1	2.757E-75	0.21703979	0.156	0.435	4.2378E-71	Neu
NOD23	3.2345E-75	0.46055164	0.135	0.395	4.9717E-71	Neu
CCDC1252	3.4164E-75	0.33262166	0.135	0.4	5.2514E-71	Neu
CNOT61	3.7281E-75	0.22538231	0.141	0.411	5.7305E-71	Neu
FGD31	3.8065E-75	0.43583591	0.188	0.481	5.8509E-71	Neu
GBP51	4.0154E-75	0.65783321	0.128	0.382	6.172E-71	Neu
TMEM1311	4.1785E-75	0.26985159	0.17	0.457	6.4228E-71	Neu
NOL7	4.2235E-75	0.24637904	0.122	0.376	6.4919E-71	Neu
COMMD31	4.4494E-75	0.1371864	0.129	0.39	6.8391E-71	Neu
HDAC21	4.7063E-75	0.18713152	0.119	0.372	7.2341E-71	Neu
STAC1	5.0482E-75	0.25728324	0.327	0.717	7.7596E-71	Neu
PIKFYVE1	6.2203E-75	0.31717204	0.234	0.565	9.5613E-71	Neu
C2CD5	6.573E-75	0.28398074	0.189	0.489	1.0103E-70	Neu
KDM2A2	6.632E-75	0.28653488	0.395	0.825	1.0194E-70	Neu
ZRANB21	6.9793E-75	0.10283646	0.167	0.452	1.0728E-70	Neu
ARHGAP152	7.0961E-75	0.63896513	0.197	0.499	1.0907E-70	Neu
MAP4K51	7.6784E-75	0.32150781	0.175	0.467	1.1803E-70	Neu
RBM27	7.8359E-75	0.17260315	0.132	0.394	1.2045E-70	Neu
RABGEF12	8.5103E-75	0.31880891	0.135	0.399	1.3081E-70	Neu
RAB3GAP1	8.6476E-75	0.488139	0.246	0.588	1.3292E-70	Neu
ARL4A1	8.765E-75	0.26115108	0.181	0.474	1.3473E-70	Neu
STAM21	9.2439E-75	0.29458313	0.209	0.527	1.4209E-70	Neu
SHTN12	9.4284E-75	0.18754015	0.388	0.813	1.4492E-70	Neu
PIK3R4	9.5277E-75	0.2750786	0.145	0.415	1.4645E-70	Neu
CREBRF	1.0154E-74	0.44977705	0.235	0.564	1.5608E-70	Neu
STAT61	1.02E-74	0.11129271	0.473	0.903	1.5679E-70	Neu
PDHX	1.0245E-74	0.20305177	0.149	0.421	1.5748E-70	Neu
C5AR22	1.0718E-74	0.51140799	0.165	0.444	1.6475E-70	Neu
DCP21	1.1106E-74	0.37554927	0.213	0.529	1.7071E-70	Neu
ANKRD443	1.1337E-74	0.27075696	0.176	0.462	1.7427E-70	Neu
HIPK21	1.1738E-74	0.1057848	0.417	0.841	1.8043E-70	Neu
WDR112	1.2773E-74	0.18087389	0.162	0.446	1.9633E-70	Neu
AKR7A2	1.3716E-74	0.1989482	0.152	0.428	2.1082E-70	Neu
FAM199X1	1.4732E-74	0.57720051	0.177	0.471	2.2645E-70	Neu
TNRC6A1	1.4958E-74	0.16205952	0.144	0.411	2.2992E-70	Neu
CBX31	1.5591E-74	0.29560009	0.224	0.554	2.3965E-70	Neu
UVRAG3	1.66E-74	0.1975048	0.139	0.401	2.5516E-70	Neu
COG71	1.7226E-74	0.45782861	0.134	0.397	2.6478E-70	Neu
MTMR1	2.1707E-74	0.10192267	0.099	0.335	3.3366E-70	Neu
SDHA1	2.2662E-74	0.22179351	0.125	0.383	3.4834E-70	Neu
RSL1D1	2.3114E-74	0.23450818	0.161	0.443	3.5529E-70	Neu
MCTP12	2.7478E-74	0.63598745	0.188	0.489	4.2237E-70	Neu
SAMD8	2.7563E-74	0.20136558	0.147	0.418	4.2367E-70	Neu
ATXN1L1	2.8529E-74	0.14441146	0.107	0.349	4.3851E-70	Neu
PCGF11	3.0022E-74	0.11057991	0.127	0.384	4.6146E-70	Neu
SPOPL1	3.3879E-74	0.31855166	0.144	0.412	5.2076E-70	Neu

DSE4	3.5995E-74	0.19550439	0.435	0.864	5.5328E-70	Neu
CPEB41	3.6411E-74	0.26653089	0.383	0.814	5.5967E-70	Neu
NSMCE4A1	3.7291E-74	0.20529866	0.122	0.373	5.732E-70	Neu
DDX58	3.9996E-74	0.47142791	0.173	0.46	6.1478E-70	Neu
SNAP232	4.1533E-74	0.23005347	0.122	0.375	6.384E-70	Neu
ZDHHC61	5.0509E-74	0.31428237	0.195	0.5	7.7638E-70	Neu
FAM136A2	5.1276E-74	0.17565306	0.137	0.399	7.8816E-70	Neu
TBC1D5	5.2233E-74	0.32891963	0.223	0.549	8.0288E-70	Neu
NCOR1	5.6389E-74	0.187866	0.391	0.827	8.6675E-70	Neu
SLC25A46	5.7092E-74	0.3452561	0.301	0.679	8.7757E-70	Neu
TRAPPC13	6.0634E-74	0.26412888	0.118	0.367	9.32E-70	Neu
SUMO2	6.1875E-74	0.42804312	0.265	0.621	9.5108E-70	Neu
INTS101	6.626E-74	0.23625342	0.176	0.468	1.0185E-69	Neu
NFIL31	6.7051E-74	0.13507673	0.352	0.729	1.0306E-69	Neu
INAFM23	6.8672E-74	0.29825932	0.104	0.343	1.0556E-69	Neu
ZSWIM63	7.3895E-74	0.38477488	0.198	0.502	1.1358E-69	Neu
VPS521	7.6665E-74	0.34052484	0.17	0.454	1.1784E-69	Neu
NIPBL1	7.9092E-74	0.43705529	0.29	0.665	1.2157E-69	Neu
PPP2R3C1	8.8597E-74	0.29439752	0.197	0.501	1.3618E-69	Neu
ESF11	1.0161E-73	0.20744948	0.119	0.368	1.5618E-69	Neu
MORF4L11	1.1803E-73	0.28364902	0.13	0.389	1.8142E-69	Neu
SP1401	1.2107E-73	0.39847766	0.168	0.453	1.8609E-69	Neu
LCP21	1.2351E-73	0.46125708	0.416	0.845	1.8985E-69	Neu
TTC81	1.4826E-73	0.23696999	0.116	0.364	2.279E-69	Neu
MBD3	1.5762E-73	0.24168439	0.133	0.391	2.4228E-69	Neu
USP121	1.6119E-73	0.20207938	0.144	0.411	2.4776E-69	Neu
STYX1	1.8415E-73	0.33760884	0.164	0.445	2.8306E-69	Neu
RIC11	1.8454E-73	0.12582165	0.152	0.42	2.8365E-69	Neu
ZNF6641	1.8464E-73	0.36391355	0.12	0.37	2.8381E-69	Neu
USP381	1.8796E-73	0.25675726	0.124	0.377	2.8892E-69	Neu
SPPL2B1	1.9418E-73	0.23203121	0.118	0.363	2.9848E-69	Neu
VPS37B	2.0223E-73	0.16769845	0.088	0.316	3.1084E-69	Neu
RRP7A	2.1852E-73	0.1793735	0.08	0.302	3.3589E-69	Neu
NSMCE2	2.4126E-73	0.49704087	0.171	0.456	3.7084E-69	Neu
CPSF6	2.6959E-73	0.19315134	0.128	0.382	4.1439E-69	Neu
FAM174A	2.7455E-73	0.50914545	0.092	0.323	4.2201E-69	Neu
FAM160B21	2.7944E-73	0.10811884	0.086	0.313	4.2952E-69	Neu
NOA11	2.8616E-73	0.16813357	0.122	0.372	4.3985E-69	Neu
KLHL81	2.9991E-73	0.21915295	0.128	0.384	4.6099E-69	Neu
CAMTA21	3.0506E-73	0.32002531	0.127	0.383	4.6891E-69	Neu
RCBTB11	3.2783E-73	0.20819019	0.091	0.319	5.039E-69	Neu
PSMG31	3.2922E-73	0.18462155	0.167	0.445	5.0604E-69	Neu
DHX401	3.3279E-73	0.22973408	0.137	0.4	5.1153E-69	Neu
POLK	3.3306E-73	0.37078653	0.184	0.479	5.1195E-69	Neu
UBR31	3.3699E-73	0.12531012	0.157	0.434	5.1799E-69	Neu
FAM160A22	3.3828E-73	0.11191126	0.121	0.369	5.1998E-69	Neu
SETDB22	3.5084E-73	0.34387415	0.154	0.426	5.3927E-69	Neu
XIAP1	3.801E-73	0.16570526	0.133	0.391	5.8425E-69	Neu
MAIP1	3.8269E-73	0.27995204	0.127	0.381	5.8824E-69	Neu
CCDC90B1	4.3393E-73	0.29886462	0.145	0.415	6.6699E-69	Neu
DENND4C1	4.4057E-73	0.16490477	0.4	0.824	6.772E-69	Neu
CTBS1	4.4166E-73	0.18834437	0.373	0.789	6.7887E-69	Neu

ATG4B1	4.5502E-73	0.26590539	0.145	0.409	6.9941E-69	Neu
DIXDC11	4.6013E-73	0.2972278	0.134	0.392	7.0726E-69	Neu
KANSL3	4.7316E-73	0.37910941	0.135	0.393	7.273E-69	Neu
PDE8A1	5.1741E-73	0.41819069	0.169	0.451	7.953E-69	Neu
RMDN1	5.3691E-73	0.17639976	0.173	0.46	8.2529E-69	Neu
CEP631	5.8599E-73	0.35971547	0.164	0.441	9.0073E-69	Neu
ADRB22	6.1455E-73	0.16557479	0.174	0.454	9.4463E-69	Neu
CAMSAP22	6.2282E-73	0.2357457	0.176	0.463	9.5734E-69	Neu
C16orf54	6.2361E-73	0.12921815	0.121	0.367	9.5855E-69	Neu
NXF12	6.7868E-73	0.38921941	0.189	0.48	1.0432E-68	Neu
RPUSD41	6.8012E-73	0.14708266	0.112	0.355	1.0454E-68	Neu
LBR3	6.8917E-73	0.1997306	0.111	0.353	1.0593E-68	Neu
NCBP11	7.4961E-73	0.10664922	0.104	0.341	1.1522E-68	Neu
RASGRP43	8.7407E-73	0.36533389	0.163	0.441	1.3435E-68	Neu
SLC38A62	9.0101E-73	0.30148504	0.111	0.354	1.3849E-68	Neu
MOCS2	9.0924E-73	0.25128846	0.201	0.508	1.3976E-68	Neu
SLC25A131	9.1447E-73	0.4618165	0.154	0.426	1.4056E-68	Neu
FHOD12	9.1564E-73	0.1490879	0.09	0.316	1.4074E-68	Neu
FAM204A1	9.2521E-73	0.24720527	0.183	0.479	1.4221E-68	Neu
IMPA2	9.4891E-73	0.16843405	0.085	0.308	1.4586E-68	Neu
TTL2	1.0251E-72	0.404943	0.261	0.611	1.5757E-68	Neu
MARCH72	1.081E-72	0.21528882	0.388	0.821	1.6616E-68	Neu
FAM13B1	1.1001E-72	0.48980191	0.172	0.458	1.6909E-68	Neu
MAN2A2	1.2052E-72	0.46612218	0.288	0.653	1.8524E-68	Neu
PDLIM73	1.2242E-72	0.61071536	0.26	0.599	1.8817E-68	Neu
RBM52	1.2921E-72	0.34784357	0.139	0.397	1.9861E-68	Neu
AIG12	1.3899E-72	0.14761104	0.114	0.356	2.1363E-68	Neu
RBBP5	1.438E-72	0.18255862	0.115	0.359	2.2103E-68	Neu
NKIRAS11	1.4797E-72	0.27759002	0.1	0.335	2.2744E-68	Neu
RHOT2	1.49E-72	0.27061674	0.106	0.344	2.2904E-68	Neu
NUP160	1.8631E-72	0.28628309	0.201	0.503	2.8638E-68	Neu
SMIM26	1.9384E-72	0.22041281	0.124	0.377	2.9795E-68	Neu
ARFGEF12	1.9879E-72	0.19736794	0.122	0.372	3.0556E-68	Neu
PSEN22	2.012E-72	0.1684606	0.104	0.34	3.0926E-68	Neu
NOM11	2.0827E-72	0.16642991	0.122	0.372	3.2014E-68	Neu
NDUFB4	2.5163E-72	0.27681044	0.161	0.438	3.8679E-68	Neu
SESN12	2.5292E-72	0.28522844	0.146	0.408	3.8877E-68	Neu
COX181	2.559E-72	0.33167823	0.158	0.432	3.9335E-68	Neu
EBAG91	2.6369E-72	0.23545021	0.136	0.396	4.0533E-68	Neu
RBM18	2.773E-72	0.28257964	0.12	0.371	4.2623E-68	Neu
PHF121	2.7798E-72	0.22786418	0.165	0.442	4.2729E-68	Neu
CD3022	2.8306E-72	0.20193712	0.255	0.595	4.351E-68	Neu
CEP1702	3.0502E-72	0.37229923	0.326	0.716	4.6884E-68	Neu
TNPO31	3.3348E-72	0.38666015	0.188	0.484	5.1259E-68	Neu
FAM210A	3.8352E-72	0.21285778	0.106	0.342	5.8951E-68	Neu
FBXO101	4.0219E-72	0.11640704	0.107	0.344	6.1821E-68	Neu
SENP21	4.1761E-72	0.12208606	0.155	0.426	6.4191E-68	Neu
NFATC11	4.4125E-72	0.1558601	0.095	0.324	6.7824E-68	Neu
PDPR3	4.4384E-72	0.26407252	0.12	0.365	6.8223E-68	Neu
ABAT2	4.8604E-72	0.14683892	0.129	0.378	7.4709E-68	Neu
PTPRM1	4.9269E-72	0.20195491	0.133	0.387	7.5732E-68	Neu
C5orf221	5.4192E-72	0.11484831	0.119	0.366	8.3298E-68	Neu

SLC20A13	5.5931E-72	0.59941116	0.164	0.437	8.5972E-68	Neu
VRK31	6.0741E-72	0.12242977	0.107	0.344	9.3365E-68	Neu
PIGX	6.5588E-72	0.22781831	0.124	0.375	1.0082E-67	Neu
VPS37A	7.1562E-72	0.30152134	0.135	0.393	1.1E-67	Neu
NF11	7.3558E-72	0.50533595	0.171	0.452	1.1307E-67	Neu
KDM3A1	7.5376E-72	0.21825154	0.135	0.39	1.1586E-67	Neu
PDK42	7.7281E-72	0.22658051	0.349	0.727	1.1879E-67	Neu
AGPAT42	7.7903E-72	0.34167709	0.167	0.443	1.1975E-67	Neu
PPIP5K22	7.898E-72	0.35051729	0.199	0.502	1.214E-67	Neu
FBXO11	8.5432E-72	0.36971146	0.18	0.469	1.3132E-67	Neu
FBXW7	8.9604E-72	0.46391512	0.226	0.544	1.3773E-67	Neu
MINPP11	9.4221E-72	0.10090567	0.108	0.346	1.4483E-67	Neu
STRN31	9.5055E-72	0.27958213	0.129	0.383	1.4611E-67	Neu
MINDY3	1.24E-71	0.23644122	0.112	0.353	1.906E-67	Neu
HECA	1.2457E-71	0.218115	0.407	0.844	1.9147E-67	Neu
RTN22	1.2569E-71	0.14140511	0.096	0.326	1.932E-67	Neu
ZC3H31	1.2602E-71	0.21410205	0.102	0.333	1.9371E-67	Neu
ZDHHC121	1.3138E-71	0.15886886	0.078	0.294	2.0195E-67	Neu
GPR651	1.4167E-71	0.25621605	0.235	0.555	2.1775E-67	Neu
ST131	1.6029E-71	0.20842071	0.146	0.411	2.4638E-67	Neu
B3GNT52	1.6055E-71	0.44759095	0.299	0.662	2.4679E-67	Neu
SNRNP27	1.6198E-71	0.24698877	0.102	0.336	2.4898E-67	Neu
FOXJ2	1.6683E-71	0.14065566	0.104	0.339	2.5644E-67	Neu
STX43	1.6861E-71	0.33503748	0.408	0.846	2.5917E-67	Neu
FBXL6	1.8136E-71	0.18278925	0.096	0.324	2.7877E-67	Neu
AZIN1	1.852E-71	0.34030449	0.169	0.453	2.8467E-67	Neu
IDI11	1.9321E-71	0.10759893	0.133	0.382	2.9699E-67	Neu
UBE2N	2.2217E-71	0.17849048	0.123	0.371	3.4149E-67	Neu
CD802	2.3407E-71	0.22528738	0.117	0.36	3.5979E-67	Neu
NOL81	2.3839E-71	0.26923465	0.109	0.346	3.6643E-67	Neu
PRDM2	2.4429E-71	0.4144104	0.222	0.541	3.755E-67	Neu
ANKRD281	2.5161E-71	0.34082087	0.128	0.382	3.8675E-67	Neu
SLMAP1	2.5241E-71	0.16866975	0.127	0.379	3.8797E-67	Neu
COX7A2L	2.5675E-71	0.19451924	0.382	0.802	3.9465E-67	Neu
UBE2D31	2.569E-71	0.31170639	0.391	0.816	3.9489E-67	Neu
CEBPD3	2.68E-71	0.65624313	0.149	0.411	4.1194E-67	Neu
RNF1152	3.1685E-71	0.22556685	0.176	0.459	4.8704E-67	Neu
DSC22	3.3633E-71	0.2469662	0.23	0.548	5.1697E-67	Neu
NISCH	3.7952E-71	0.27908923	0.124	0.37	5.8336E-67	Neu
TRPM7	4.0281E-71	0.2858561	0.18	0.465	6.1916E-67	Neu
BDP11	4.108E-71	0.15941242	0.143	0.402	6.3144E-67	Neu
HSPB111	4.2385E-71	0.17197495	0.127	0.377	6.515E-67	Neu
NCOA6	4.3829E-71	0.24536912	0.165	0.441	6.737E-67	Neu
SLC8B13	4.6824E-71	0.30026358	0.088	0.31	7.1973E-67	Neu
CLK3	5.8432E-71	0.49936067	0.133	0.389	8.9816E-67	Neu
TRIQK1	6.5446E-71	0.27592807	0.282	0.64	1.006E-66	Neu
USP361	6.8042E-71	0.11083594	0.122	0.367	1.0459E-66	Neu
PRPS1	7.1772E-71	0.19866967	0.129	0.38	1.1032E-66	Neu
BTAF13	7.4352E-71	0.11660519	0.152	0.416	1.1429E-66	Neu
CENPV1	7.5789E-71	0.17249331	0.112	0.35	1.165E-66	Neu
MILR12	7.8802E-71	0.17375737	0.136	0.389	1.2113E-66	Neu
DDX173	9.2992E-71	0.44522555	0.249	0.582	1.4294E-66	Neu

EIF3H	9.4706E-71	0.27594379	0.348	0.751	1.4557E-66	Neu
NUS12	9.7844E-71	0.17780607	0.415	0.842	1.504E-66	Neu
KSR12	1.0078E-70	0.33246237	0.334	0.716	1.5491E-66	Neu
UROS1	1.0303E-70	0.18014297	0.113	0.351	1.5836E-66	Neu
RPRD2	1.0778E-70	0.20570333	0.137	0.392	1.6567E-66	Neu
ST3GAL63	1.1299E-70	0.31438867	0.224	0.541	1.7368E-66	Neu
RIOK2	1.2655E-70	0.14760666	0.106	0.339	1.9452E-66	Neu
VEZF11	1.2942E-70	0.29983905	0.177	0.459	1.9893E-66	Neu
DLD1	1.3E-70	0.21367586	0.392	0.813	1.9983E-66	Neu
STX22	1.4109E-70	0.11491466	0.113	0.35	2.1687E-66	Neu
NFS1	1.4528E-70	0.1855805	0.127	0.375	2.2331E-66	Neu
TRMT14	1.488E-70	0.20543031	0.13	0.38	2.2873E-66	Neu
FBH11	1.4947E-70	0.34030423	0.147	0.412	2.2976E-66	Neu
CLIC11	1.5397E-70	0.10908501	0.439	0.858	2.3667E-66	Neu
TMEM1381	1.5581E-70	0.12374974	0.108	0.342	2.3949E-66	Neu
KDM6B	1.5884E-70	0.91414249	0.286	0.645	2.4415E-66	Neu
CABLES11	1.7875E-70	0.13198931	0.125	0.371	2.7475E-66	Neu
MAPK6	1.9613E-70	0.49037342	0.176	0.462	3.0148E-66	Neu
SDF21	2.0159E-70	0.27327782	0.118	0.359	3.0987E-66	Neu
GBA2	2.0684E-70	0.25135161	0.111	0.347	3.1793E-66	Neu
TMLHE1	2.11E-70	0.33151263	0.127	0.377	3.2433E-66	Neu
RALA3	2.1141E-70	0.24943778	0.118	0.36	3.2496E-66	Neu
PRKD31	2.2076E-70	0.19757923	0.115	0.356	3.3933E-66	Neu
ACSF3	2.2124E-70	0.42260532	0.122	0.368	3.4007E-66	Neu
SLC35A3	2.2815E-70	0.26001433	0.135	0.391	3.5068E-66	Neu
INTS6	2.7768E-70	0.25876593	0.136	0.39	4.2683E-66	Neu
FER2	2.8883E-70	0.14031108	0.171	0.449	4.4395E-66	Neu
ZNF394	2.9213E-70	0.6984298	0.133	0.387	4.4903E-66	Neu
NUP42	2.9988E-70	0.13847508	0.144	0.402	4.6094E-66	Neu
PNPLA81	3.363E-70	0.33696538	0.123	0.369	5.1693E-66	Neu
SIK33	3.3752E-70	0.26522671	0.087	0.308	5.188E-66	Neu
E2F31	3.3931E-70	0.27203997	0.1	0.331	5.2155E-66	Neu
TRIB13	3.5727E-70	0.74578086	0.22	0.529	5.4915E-66	Neu
MEFV1	3.7005E-70	0.86368466	0.18	0.464	5.688E-66	Neu
CELF23	3.7111E-70	0.10781502	0.432	0.866	5.7043E-66	Neu
KDM4C2	3.9496E-70	0.39509303	0.259	0.6	6.0709E-66	Neu
DENND101	4.9841E-70	0.21439329	0.122	0.364	7.6611E-66	Neu
COMMD6	5.1489E-70	0.20244651	0.223	0.539	7.9145E-66	Neu
CDK122	5.3103E-70	0.19180648	0.128	0.378	8.1624E-66	Neu
FBXO221	5.4263E-70	0.18891272	0.122	0.366	8.3408E-66	Neu
KMT5B2	5.9151E-70	0.37501964	0.227	0.543	9.0921E-66	Neu
BLZF1	7.3559E-70	0.25840475	0.123	0.369	1.1307E-65	Neu
SEPTIN71	9.224E-70	0.28250276	0.177	0.459	1.4178E-65	Neu
NDE12	9.682E-70	0.63370047	0.131	0.383	1.4882E-65	Neu
PRDM4	1.0381E-69	0.18284544	0.149	0.41	1.5957E-65	Neu
PIAS11	1.0715E-69	0.44406491	0.252	0.587	1.6471E-65	Neu
WWC3	1.0839E-69	0.23768985	0.076	0.287	1.666E-65	Neu
ATF42	1.1156E-69	0.2798986	0.249	0.575	1.7148E-65	Neu
PEX161	1.1183E-69	0.11276134	0.078	0.291	1.7189E-65	Neu
CHSY13	1.2231E-69	0.93805388	0.122	0.367	1.88E-65	Neu
MAT2A4	1.2441E-69	0.15639371	0.422	0.843	1.9123E-65	Neu
BCL33	1.375E-69	0.84330366	0.168	0.441	2.1136E-65	Neu

MMAB1	1.3967E-69	0.14140053	0.096	0.321	2.1468E-65	Neu
DENND1B1	1.5807E-69	0.28940349	0.112	0.348	2.4297E-65	Neu
ZNF6413	1.6361E-69	0.31519634	0.105	0.335	2.5148E-65	Neu
PARN2	1.813E-69	0.23578202	0.137	0.39	2.7868E-65	Neu
STAU2	1.8375E-69	0.30506455	0.313	0.693	2.8245E-65	Neu
TLE31	1.9293E-69	0.36857209	0.1	0.327	2.9655E-65	Neu
TMEM126B	1.9355E-69	0.30373149	0.196	0.493	2.975E-65	Neu
TUBGCP61	2.0971E-69	0.14404861	0.149	0.406	3.2235E-65	Neu
P2RY16	2.1223E-69	0.11630937	0.071	0.279	3.2621E-65	Neu
SLC30A51	2.3267E-69	0.22896604	0.102	0.332	3.5764E-65	Neu
CYCS	2.3991E-69	0.42628765	0.233	0.555	3.6876E-65	Neu
MED131	2.6992E-69	0.4146005	0.184	0.476	4.149E-65	Neu
PGAM1	2.7752E-69	0.16726872	0.089	0.309	4.2658E-65	Neu
PPIL4	2.8852E-69	0.12140573	0.094	0.317	4.4349E-65	Neu
PLA2G4A2	3.0768E-69	0.17825231	0.107	0.338	4.7294E-65	Neu
USP16	3.1182E-69	0.11624213	0.128	0.375	4.793E-65	Neu
MIEF1	3.2084E-69	0.14684646	0.083	0.3	4.9316E-65	Neu
KAT2B2	3.4332E-69	0.20304424	0.149	0.407	5.2771E-65	Neu
SFMBT21	3.4728E-69	0.21898678	0.154	0.415	5.338E-65	Neu
IFFO1	3.6871E-69	0.39619815	0.102	0.328	5.6675E-65	Neu
EIF2A	4.1654E-69	0.20329298	0.351	0.752	6.4027E-65	Neu
TUT41	4.3307E-69	0.209888	0.161	0.43	6.6567E-65	Neu
CDC42EP31	4.4293E-69	0.22287695	0.404	0.812	6.8083E-65	Neu
WAC	5.0636E-69	0.17787094	0.124	0.368	7.7833E-65	Neu
CYSLTR11	5.8716E-69	0.27820887	0.217	0.526	9.0252E-65	Neu
STK38L4	6.4791E-69	0.44176038	0.123	0.366	9.9591E-65	Neu
MSRA1	7.8608E-69	0.11510069	0.413	0.844	1.2083E-64	Neu
ST6GALNAC61	7.9786E-69	0.18004345	0.372	0.773	1.2264E-64	Neu
TBC1D22A	8.4619E-69	0.40550588	0.125	0.37	1.3007E-64	Neu
TAF1D2	8.4745E-69	0.32999602	0.199	0.492	1.3026E-64	Neu
MMP253	8.508E-69	6.47075361	0.018	0.001	1.3078E-64	Neu
ALPL	8.7887E-69	6.30409595	0.017	0.001	1.3509E-64	Neu
TMEM681	8.8024E-69	0.12268944	0.167	0.435	1.353E-64	Neu
SBNO11	9.574E-69	0.18335722	0.123	0.365	1.4716E-64	Neu
COP11	9.6588E-69	0.61724553	0.23	0.552	1.4847E-64	Neu
TMEM170B1	1.1892E-68	0.43575827	0.204	0.502	1.8278E-64	Neu
NSA21	1.2019E-68	0.38249262	0.25	0.585	1.8474E-64	Neu
MTPAP	1.2087E-68	0.32512969	0.128	0.376	1.8579E-64	Neu
CHCHD31	1.3396E-68	0.28653383	0.112	0.349	2.0591E-64	Neu
UBE2D41	1.3692E-68	0.1380357	0.118	0.356	2.1046E-64	Neu
ANKRD13D	1.4257E-68	0.52933723	0.329	0.714	2.1915E-64	Neu
CYTH43	1.4291E-68	1.10841459	0.241	0.564	2.1966E-64	Neu
PRPF38A2	1.4392E-68	0.24308504	0.149	0.408	2.2121E-64	Neu
ECHDC11	1.4898E-68	0.12227301	0.387	0.804	2.29E-64	Neu
FARP21	1.501E-68	0.25830534	0.131	0.378	2.3071E-64	Neu
GTF2E1	1.5092E-68	0.31360909	0.125	0.368	2.3199E-64	Neu
TAB11	1.5301E-68	0.13547732	0.118	0.356	2.3519E-64	Neu
HACD41	1.8162E-68	0.33380348	0.311	0.682	2.7916E-64	Neu
TUBGCP3	1.8599E-68	0.27371726	0.158	0.422	2.8588E-64	Neu
CCSER22	1.9716E-68	0.17221854	0.19	0.481	3.0306E-64	Neu
MOB3A3	2.2467E-68	0.28492893	0.416	0.849	3.4535E-64	Neu
PHF20L12	2.3776E-68	0.64588754	0.188	0.474	3.6546E-64	Neu

KANSL21	2.5606E-68	0.24950717	0.108	0.338	3.9359E-64	Neu
HRAS	2.5668E-68	0.36862157	0.098	0.321	3.9454E-64	Neu
ZNF3581	2.6473E-68	0.32692123	0.101	0.327	4.0691E-64	Neu
DEGS11	2.7925E-68	0.10661496	0.394	0.813	4.2924E-64	Neu
TPRKB	2.9439E-68	0.3114402	0.114	0.349	4.525E-64	Neu
CPEB2	3.0991E-68	0.17992714	0.104	0.332	4.7637E-64	Neu
EGR21	3.1232E-68	0.15512294	0.237	0.535	4.8007E-64	Neu
PJA2	3.9556E-68	0.10110362	0.454	0.887	6.0802E-64	Neu
NRDC1	4.6349E-68	0.38659295	0.36	0.775	7.1243E-64	Neu
PPP4R11	4.7942E-68	0.52440305	0.172	0.447	7.3692E-64	Neu
CPNE22	4.8332E-68	0.12519164	0.085	0.3	7.4291E-64	Neu
CD583	4.8802E-68	0.50565389	0.294	0.66	7.5014E-64	Neu
GOSR2	4.9951E-68	0.35654839	0.159	0.423	7.678E-64	Neu
MAP3K24	5.3476E-68	0.49682946	0.262	0.607	8.2198E-64	Neu
UBXN2B1	5.4885E-68	0.25620181	0.379	0.798	8.4364E-64	Neu
PARP8	5.6838E-68	0.20167727	0.181	0.456	8.7365E-64	Neu
MAP3K41	6.0054E-68	0.11510596	0.128	0.37	9.2309E-64	Neu
ATMIN1	6.1474E-68	0.133899	0.121	0.361	9.4491E-64	Neu
MPHOSPH101	6.8692E-68	0.15097355	0.096	0.319	1.0559E-63	Neu
FNIP13	7.8524E-68	0.6354767	0.159	0.426	1.207E-63	Neu
METTL17	7.8572E-68	0.27448477	0.145	0.397	1.2077E-63	Neu
RB1CC1	7.9909E-68	0.216406	0.161	0.429	1.2283E-63	Neu
THRA	8.2998E-68	0.16371607	0.081	0.293	1.2758E-63	Neu
STX161	8.5977E-68	0.32599461	0.248	0.57	1.3216E-63	Neu
ZNF561	9.1501E-68	0.2019233	0.111	0.342	1.4065E-63	Neu
PTEN1	1.0059E-67	0.16635119	0.13	0.376	1.5462E-63	Neu
MED13L4	1.0165E-67	0.2334689	0.145	0.4	1.5625E-63	Neu
SENP62	1.0352E-67	0.42060092	0.292	0.652	1.5912E-63	Neu
OXA1L2	1.0636E-67	0.26698423	0.379	0.785	1.6349E-63	Neu
RABGGTB	1.2388E-67	0.25043686	0.361	0.766	1.9041E-63	Neu
PLCL2	1.2888E-67	0.21666454	0.134	0.382	1.981E-63	Neu
HPGD1	1.311E-67	0.10093036	0.347	0.718	2.0151E-63	Neu
ADNP2	1.4447E-67	0.29709609	0.114	0.348	2.2207E-63	Neu
DDX3X2	1.4724E-67	0.83387355	0.179	0.457	2.2632E-63	Neu
RHOQ2	1.5033E-67	0.11331296	0.444	0.866	2.3108E-63	Neu
CHFR1	1.5067E-67	0.41838926	0.145	0.399	2.316E-63	Neu
USP402	1.5245E-67	0.23240241	0.111	0.341	2.3433E-63	Neu
PRKRA	1.5707E-67	0.12702209	0.139	0.391	2.4143E-63	Neu
LYSMD31	1.8578E-67	0.18796012	0.132	0.378	2.8556E-63	Neu
SBF22	1.9017E-67	0.26611279	0.142	0.393	2.9232E-63	Neu
CLEC12A2	1.9357E-67	0.26570285	0.306	0.666	2.9754E-63	Neu
SLC25A323	1.9518E-67	0.1262724	0.119	0.357	3.0001E-63	Neu
RBM63	1.9532E-67	0.52631987	0.213	0.513	3.0022E-63	Neu
CHD13	1.9871E-67	0.37715908	0.152	0.408	3.0543E-63	Neu
GOLGA11	2.0154E-67	0.31827638	0.129	0.373	3.0979E-63	Neu
VPS54	2.2432E-67	0.35200308	0.208	0.503	3.448E-63	Neu
RNF1391	2.2581E-67	0.1490043	0.142	0.395	3.4709E-63	Neu
USP301	2.4174E-67	0.217151	0.107	0.335	3.7158E-63	Neu
MAFF2	2.4407E-67	1.13537286	0.106	0.333	3.7515E-63	Neu
STX62	2.5173E-67	0.18222334	0.418	0.853	3.8694E-63	Neu
COPS7B	2.6066E-67	0.14203436	0.114	0.346	4.0067E-63	Neu
HMG20A	2.6094E-67	0.10913893	0.108	0.334	4.0109E-63	Neu

SETD5	2.8167E-67	0.42624497	0.225	0.536	4.3296E-63	Neu
LTN1	3.1634E-67	0.24524529	0.148	0.404	4.8625E-63	Neu
DAPK11	3.3658E-67	0.23387836	0.413	0.827	5.1736E-63	Neu
BCAS2	3.6857E-67	0.16004395	0.092	0.311	5.6653E-63	Neu
WDR451	4.0426E-67	0.23746314	0.112	0.344	6.2139E-63	Neu
PPFIBP22	4.0465E-67	0.44527472	0.179	0.456	6.2198E-63	Neu
TSHZ11	4.2585E-67	0.11040557	0.096	0.317	6.5458E-63	Neu
IMPA11	4.2602E-67	0.14044001	0.106	0.332	6.5484E-63	Neu
STK31	4.7624E-67	0.31917421	0.116	0.351	7.3202E-63	Neu
CCDC71L1	5.0491E-67	0.31087778	0.067	0.266	7.761E-63	Neu
CRCP	5.1975E-67	0.17546261	0.101	0.325	7.9891E-63	Neu
ITGB81	5.5747E-67	0.20171222	0.291	0.623	8.5688E-63	Neu
ADAP12	6.5859E-67	0.27782108	0.106	0.332	1.0123E-62	Neu
ARF52	6.7551E-67	0.20653634	0.122	0.358	1.0383E-62	Neu
STK161	7.1465E-67	0.22460532	0.083	0.295	1.0985E-62	Neu
ZNF3261	7.8714E-67	0.29824724	0.116	0.348	1.2099E-62	Neu
RYR11	8.1316E-67	0.10400238	0.097	0.317	1.2499E-62	Neu
ABCB10	8.18E-67	0.26079857	0.131	0.374	1.2574E-62	Neu
SEPTIN101	8.4607E-67	0.27483029	0.157	0.422	1.3005E-62	Neu
NAPG	9.3529E-67	0.25458101	0.119	0.354	1.4376E-62	Neu
DHRS11	9.4771E-67	0.14962939	0.096	0.316	1.4567E-62	Neu
SUZ121	9.7172E-67	0.44348408	0.152	0.412	1.4936E-62	Neu
XPO12	1.0295E-66	0.3343984	0.14	0.392	1.5825E-62	Neu
SECISBP22	1.0729E-66	0.3727222	0.133	0.376	1.6491E-62	Neu
FAM8A11	1.1126E-66	0.28865249	0.096	0.317	1.7102E-62	Neu
CACNB31	1.3103E-66	0.27965057	0.145	0.396	2.0141E-62	Neu
NBEAL22	1.3578E-66	0.56828706	0.089	0.301	2.0871E-62	Neu
NOC4L3	1.4557E-66	0.12559609	0.092	0.309	2.2376E-62	Neu
MAFG2	1.5471E-66	0.38245316	0.094	0.312	2.378E-62	Neu
PNISR3	1.6563E-66	0.31209813	0.207	0.495	2.5459E-62	Neu
ICAM14	1.7486E-66	1.13107489	0.106	0.33	2.6878E-62	Neu
ZFYVE19	1.7516E-66	0.15243281	0.077	0.282	2.6924E-62	Neu
KLHL291	1.8128E-66	0.20985795	0.118	0.352	2.7864E-62	Neu
LMBR1	1.8612E-66	0.48568464	0.192	0.481	2.8609E-62	Neu
CMC1	1.8901E-66	0.1609361	0.083	0.296	2.9052E-62	Neu
RANBP9	1.8982E-66	0.45338451	0.213	0.513	2.9177E-62	Neu
AKAP101	1.9408E-66	0.50284943	0.173	0.447	2.9832E-62	Neu
TMEM183A1	1.9847E-66	0.30787363	0.276	0.622	3.0506E-62	Neu
CEP57	2.0067E-66	0.2496074	0.12	0.354	3.0845E-62	Neu
APC1	2.1193E-66	0.29463151	0.14	0.392	3.2576E-62	Neu
C7orf25	2.2301E-66	0.14170545	0.094	0.312	3.4279E-62	Neu
GAB3	2.3281E-66	0.44207474	0.18	0.457	3.5785E-62	Neu
PAXX1	2.4077E-66	0.14036518	0.106	0.331	3.7009E-62	Neu
SLC2A32	2.4626E-66	0.20943085	0.354	0.729	3.7853E-62	Neu
CAMK2D1	2.6015E-66	0.10129903	0.073	0.275	3.9987E-62	Neu
ULK11	2.6381E-66	0.48008914	0.085	0.295	4.055E-62	Neu
PAN32	2.6452E-66	0.36994076	0.169	0.441	4.066E-62	Neu
DDX50	2.7602E-66	0.27970608	0.151	0.405	4.2427E-62	Neu
ZNF800	2.8507E-66	0.47415965	0.266	0.608	4.3818E-62	Neu
NECAB31	2.8564E-66	0.14487665	0.09	0.304	4.3906E-62	Neu
PTPRE3	3.2032E-66	0.82581803	0.16	0.418	4.9237E-62	Neu
ECD1	3.2848E-66	0.17473125	0.116	0.347	5.0491E-62	Neu

DCUN1D4	3.3578E-66	0.25018756	0.126	0.365	5.1612E-62	Neu
SS18L21	3.5818E-66	0.11891919	0.098	0.32	5.5055E-62	Neu
WBP42	3.6123E-66	0.28628217	0.097	0.317	5.5525E-62	Neu
KIAA1109	3.6822E-66	0.11507328	0.148	0.402	5.6598E-62	Neu
DDX59	4.594E-66	0.17242069	0.152	0.405	7.0615E-62	Neu
KIAA02321	4.788E-66	0.14511983	0.136	0.38	7.3596E-62	Neu
SGSH2	5.7249E-66	0.20012401	0.069	0.267	8.7997E-62	Neu
CDK5RAP31	5.9137E-66	0.41398653	0.334	0.721	9.0899E-62	Neu
DDX60L1	6.1927E-66	0.41692396	0.296	0.647	9.5189E-62	Neu
TRAPPC81	6.6875E-66	0.38245685	0.175	0.445	1.0279E-61	Neu
VAPA1	6.6985E-66	0.19087811	0.404	0.822	1.0296E-61	Neu
SLTM	7.8446E-66	0.3603451	0.316	0.683	1.2058E-61	Neu
SLC25A261	7.864E-66	0.14229204	0.113	0.341	1.2088E-61	Neu
SGF29	7.8767E-66	0.26410114	0.163	0.424	1.2107E-61	Neu
CTBP22	8.7805E-66	0.31230026	0.367	0.775	1.3496E-61	Neu
FPR21	8.9712E-66	0.37457469	0.31	0.643	1.379E-61	Neu
MGAT4A3	1.0156E-65	0.20796278	0.363	0.759	1.561E-61	Neu
TLR12	1.0516E-65	0.50600487	0.156	0.416	1.6164E-61	Neu
PHF10	1.1156E-65	0.29291382	0.176	0.443	1.7148E-61	Neu
PDE4B2	1.1363E-65	0.6189019	0.191	0.461	1.7466E-61	Neu
PCNT	1.1757E-65	0.19372593	0.135	0.377	1.8072E-61	Neu
TRMT62	1.2344E-65	0.37404148	0.137	0.384	1.8974E-61	Neu
BLOC1S3	1.3339E-65	0.36311162	0.092	0.308	2.0503E-61	Neu
CHP11	1.352E-65	0.18157401	0.42	0.845	2.0781E-61	Neu
NOD11	1.358E-65	0.38213584	0.111	0.337	2.0873E-61	Neu
RSBN1L	1.4042E-65	0.25237403	0.173	0.441	2.1584E-61	Neu
CYHR11	1.4279E-65	0.28103207	0.085	0.295	2.1948E-61	Neu
RNF11	1.51E-65	0.42382985	0.329	0.709	2.3211E-61	Neu
EPB41L4A1	1.6739E-65	0.23706773	0.156	0.412	2.573E-61	Neu
FAM122B1	1.8363E-65	0.26290173	0.129	0.367	2.8225E-61	Neu
SLC36A4	1.9574E-65	0.41700701	0.172	0.441	3.0087E-61	Neu
PTPN23	1.9831E-65	0.17293716	0.143	0.39	3.0482E-61	Neu
TNFRSF1B3	2.1E-65	0.9159524	0.173	0.444	3.2279E-61	Neu
NCOA5	2.1006E-65	0.20770361	0.098	0.315	3.2289E-61	Neu
ITGB11	2.1895E-65	0.16326613	0.32	0.689	3.3655E-61	Neu
PPP2R1B	2.2035E-65	0.47527134	0.144	0.393	3.387E-61	Neu
STYXL11	2.2854E-65	0.13689411	0.095	0.311	3.5128E-61	Neu
TBC1D231	2.6821E-65	0.25592154	0.099	0.317	4.1227E-61	Neu
AARS21	2.6867E-65	0.23783465	0.086	0.296	4.1297E-61	Neu
PHF142	2.7297E-65	0.18393813	0.114	0.343	4.1958E-61	Neu
HDDC21	2.8785E-65	0.10013615	0.418	0.832	4.4245E-61	Neu
LRRFIP21	3.1785E-65	0.62297814	0.183	0.463	4.8857E-61	Neu
ATP5F1C	3.2347E-65	0.24371869	0.097	0.314	4.9721E-61	Neu
NAT101	3.3266E-65	0.18015165	0.102	0.323	5.1133E-61	Neu
FNBP42	3.387E-65	0.10358393	0.127	0.359	5.2062E-61	Neu
HERC1	3.6142E-65	0.27417667	0.145	0.393	5.5554E-61	Neu
SCYL2	3.6225E-65	0.39677679	0.176	0.448	5.5682E-61	Neu
TCF32	3.9838E-65	0.14456397	0.092	0.307	6.1235E-61	Neu
MMUT	4.7075E-65	0.13166272	0.104	0.327	7.236E-61	Neu
WSB11	5.3649E-65	0.46236776	0.398	0.817	8.2463E-61	Neu
ACBD61	5.3801E-65	0.27138581	0.138	0.384	8.2698E-61	Neu
AK6	5.4047E-65	0.2283714	0.111	0.339	8.3076E-61	Neu

SFSWAP1	5.6762E-65	0.23576078	0.103	0.322	8.7249E-61	Neu
MAF11	5.9438E-65	0.19615897	0.431	0.856	9.1362E-61	Neu
PIK3CA1	5.9735E-65	0.2813716	0.135	0.38	9.1819E-61	Neu
PHYKPL3	6.2193E-65	0.3784417	0.148	0.399	9.5596E-61	Neu
PHIP	6.4699E-65	0.32289928	0.166	0.43	9.9449E-61	Neu
PPP4R21	6.5373E-65	0.36438879	0.104	0.325	1.0048E-60	Neu
USP342	6.8019E-65	0.48598868	0.262	0.595	1.0455E-60	Neu
RPRD1A	8.196E-65	0.51225996	0.167	0.433	1.2598E-60	Neu
KRCC1	8.2976E-65	0.39353943	0.151	0.406	1.2754E-60	Neu
WDFY24	9.7532E-65	0.23292648	0.149	0.396	1.4992E-60	Neu
NECAP11	9.8226E-65	0.40215132	0.116	0.347	1.5098E-60	Neu
ATXN72	9.9937E-65	0.27090052	0.14	0.383	1.5361E-60	Neu
ST6GALNAC41	1.0878E-64	0.15295061	0.091	0.303	1.6721E-60	Neu
PET117	1.3163E-64	0.11623296	0.091	0.302	2.0232E-60	Neu
STON11	1.4087E-64	0.11403287	0.191	0.46	2.1652E-60	Neu
SESTD1	1.4738E-64	0.30615765	0.133	0.373	2.2654E-60	Neu
ITPKC1	1.5283E-64	0.28399065	0.092	0.304	2.3492E-60	Neu
NR4A23	1.5531E-64	0.27588038	0.213	0.491	2.3873E-60	Neu
TRAF32	1.6411E-64	0.2900691	0.078	0.279	2.5225E-60	Neu
HNRNPR1	1.6802E-64	0.1309425	0.118	0.348	2.5827E-60	Neu
UBE2E11	1.9884E-64	0.3659716	0.128	0.366	3.0564E-60	Neu
C1orf521	2.1458E-64	0.24179446	0.125	0.362	3.2983E-60	Neu
RAE11	2.3117E-64	0.23022786	0.075	0.275	3.5533E-60	Neu
CEP1641	2.3353E-64	0.15076482	0.089	0.297	3.5896E-60	Neu
PARVG1	2.3357E-64	0.16263316	0.456	0.876	3.5901E-60	Neu
CD2AP2	2.3463E-64	0.15286868	0.098	0.312	3.6065E-60	Neu
LDAH1	2.3476E-64	0.13488882	0.091	0.299	3.6084E-60	Neu
MOB1B3	2.6954E-64	0.11381967	0.115	0.342	4.1431E-60	Neu
ZNF76	2.8261E-64	0.12496782	0.108	0.329	4.344E-60	Neu
ARHGEF10L3	3.1963E-64	0.22163074	0.451	0.868	4.913E-60	Neu
PPP1R35	3.3264E-64	0.43414385	0.104	0.323	5.1131E-60	Neu
MTMR2	3.3584E-64	0.10334042	0.089	0.299	5.1622E-60	Neu
P2RY133	3.4052E-64	0.29853234	0.09	0.297	5.2341E-60	Neu
SLC30A61	4.1805E-64	0.11343703	0.085	0.291	6.4259E-60	Neu
ZDHHC22	4.2543E-64	0.33283994	0.294	0.651	6.5393E-60	Neu
SLC25A12	4.4312E-64	0.1305838	0.079	0.281	6.8112E-60	Neu
UBASH3B1	4.4886E-64	0.14984117	0.445	0.844	6.8994E-60	Neu
AGO42	4.6519E-64	0.58790435	0.183	0.459	7.1504E-60	Neu
FPGT	4.9442E-64	0.44698235	0.115	0.341	7.5997E-60	Neu
YPEL2	5.3105E-64	0.38053362	0.125	0.358	8.1628E-60	Neu
NINJ12	5.3892E-64	1.05363414	0.16	0.414	8.2838E-60	Neu
CRTC31	5.463E-64	0.44019967	0.135	0.376	8.3972E-60	Neu
WDR431	5.4964E-64	0.1756257	0.109	0.331	8.4485E-60	Neu
TMCO3	5.6893E-64	0.34431985	0.127	0.363	8.7451E-60	Neu
C5orf24	6.0111E-64	0.13032214	0.112	0.337	9.2397E-60	Neu
BOD1L12	6.141E-64	0.29014811	0.104	0.322	9.4393E-60	Neu
ERC11	6.4357E-64	0.2196778	0.107	0.325	9.8923E-60	Neu
SDCCAG81	6.6246E-64	0.36456555	0.311	0.674	1.0183E-59	Neu
KLHDC21	6.9155E-64	0.1383952	0.161	0.418	1.063E-59	Neu
MIS18BP11	7.318E-64	0.35747561	0.311	0.681	1.1248E-59	Neu
GNG52	7.8008E-64	0.15474231	0.149	0.398	1.1991E-59	Neu
CYP20A1	8.0573E-64	0.19941108	0.124	0.357	1.2385E-59	Neu

SMARCAD11	8.76E-64	0.25495429	0.103	0.322	1.3465E-59	Neu
NUP881	9.0089E-64	0.1296596	0.09	0.299	1.3848E-59	Neu
GTDC1	9.4766E-64	0.49973824	0.152	0.408	1.4566E-59	Neu
RHNO11	9.6309E-64	0.15899941	0.105	0.323	1.4804E-59	Neu
ZNF518A1	1.0425E-63	0.36516145	0.151	0.402	1.6024E-59	Neu
LETMD11	1.1459E-63	0.28087296	0.195	0.476	1.7614E-59	Neu
MRTFB	1.1983E-63	0.29202362	0.107	0.326	1.8419E-59	Neu
FAM200B	1.2404E-63	0.24975301	0.151	0.4	1.9066E-59	Neu
MON21	1.3192E-63	0.28657812	0.139	0.38	2.0277E-59	Neu
C18orf25	1.3257E-63	0.19956955	0.12	0.347	2.0378E-59	Neu
ASCC1	1.379E-63	0.19029645	0.081	0.283	2.1197E-59	Neu
DHX37	1.3929E-63	0.16952758	0.093	0.303	2.1411E-59	Neu
SHLD11	1.5164E-63	0.29266921	0.11	0.334	2.3308E-59	Neu
MCPH11	1.557E-63	0.2695085	0.103	0.321	2.3932E-59	Neu
LRRC401	1.626E-63	0.12951564	0.096	0.309	2.4993E-59	Neu
ZBTB1	1.6355E-63	0.18434085	0.408	0.809	2.514E-59	Neu
CDK13	1.742E-63	0.4185538	0.204	0.496	2.6777E-59	Neu
RPAIN1	1.7861E-63	0.50495774	0.193	0.476	2.7455E-59	Neu
LATS11	1.8546E-63	0.2802548	0.203	0.49	2.8507E-59	Neu
SMURF21	1.9634E-63	0.17329648	0.112	0.333	3.0179E-59	Neu
KRAS3	1.9705E-63	0.32558754	0.155	0.407	3.0289E-59	Neu
MTF1	2.1394E-63	0.2063438	0.112	0.334	3.2885E-59	Neu
PLEKHA31	2.3057E-63	0.48468512	0.155	0.41	3.5442E-59	Neu
HDHD3	2.3098E-63	0.1689577	0.092	0.3	3.5504E-59	Neu
SLC4A81	2.3931E-63	0.28593636	0.112	0.333	3.6785E-59	Neu
SYPL11	2.4669E-63	0.32011812	0.117	0.345	3.7919E-59	Neu
AXIN11	2.4826E-63	0.16091341	0.107	0.326	3.8159E-59	Neu
DENND1A2	2.6483E-63	0.54122797	0.216	0.517	4.0707E-59	Neu
SSH31	2.7573E-63	0.18363019	0.077	0.276	4.2382E-59	Neu
VTI1A1	3.2743E-63	0.47597936	0.123	0.353	5.033E-59	Neu
KLF44	3.7185E-63	0.13389423	0.46	0.862	5.7156E-59	Neu
TACC3	3.7532E-63	0.18400103	0.131	0.363	5.769E-59	Neu
FRG11	4.3167E-63	0.12465838	0.089	0.295	6.6352E-59	Neu
DZIP1L1	4.6529E-63	0.14074818	0.184	0.446	7.152E-59	Neu
NT5C3A	5.2741E-63	0.27737557	0.095	0.307	8.1069E-59	Neu
KRIT1	5.5774E-63	0.15776262	0.127	0.36	8.573E-59	Neu
VPS13D	5.5818E-63	0.31594213	0.129	0.362	8.5797E-59	Neu
SPG11	5.7532E-63	0.39361805	0.35	0.744	8.8432E-59	Neu
PHF61	6.1885E-63	0.17908129	0.091	0.299	9.5123E-59	Neu
STK17B2	6.2534E-63	0.14166761	0.395	0.808	9.6121E-59	Neu
CCNH1	6.3541E-63	0.34243083	0.145	0.39	9.7669E-59	Neu
FGR2	6.4539E-63	0.23395132	0.518	0.903	9.9203E-59	Neu
PIGP2	6.4933E-63	0.27354505	0.12	0.35	9.9808E-59	Neu
ENTPD13	7.6539E-63	0.32357903	0.118	0.343	1.1765E-58	Neu
DDX39B1	8.3907E-63	0.31120335	0.133	0.365	1.2897E-58	Neu
TANGO6	8.628E-63	0.27759892	0.1	0.312	1.3262E-58	Neu
TYW31	8.7694E-63	0.14535585	0.116	0.339	1.3479E-58	Neu
LINS11	9.6507E-63	0.1202222	0.102	0.315	1.4834E-58	Neu
ELF21	1.0176E-62	0.25987477	0.167	0.425	1.5641E-58	Neu
SMAD2	1.0627E-62	0.32563987	0.36	0.759	1.6334E-58	Neu
MRRF2	1.0729E-62	0.30181634	0.098	0.311	1.6492E-58	Neu
RLIM	1.1452E-62	0.35455083	0.206	0.493	1.7603E-58	Neu

MTREX1	1.2231E-62	0.16553886	0.133	0.371	1.88E-58	Neu
RABEP2	1.4317E-62	0.14426734	0.071	0.264	2.2007E-58	Neu
MARF11	1.4398E-62	0.29544765	0.121	0.35	2.2131E-58	Neu
WDSUB1	1.4435E-62	0.3438009	0.117	0.341	2.2187E-58	Neu
IFT57	1.4614E-62	0.36110629	0.122	0.352	2.2464E-58	Neu
C8orf761	1.4999E-62	0.39754229	0.104	0.322	2.3054E-58	Neu
NTMT11	1.5987E-62	0.34975918	0.143	0.385	2.4574E-58	Neu
CSNK1G11	1.5993E-62	0.1759874	0.114	0.336	2.4583E-58	Neu
MEF2D2	1.6198E-62	0.56782631	0.147	0.393	2.4898E-58	Neu
FAM76A1	1.6776E-62	0.12143341	0.095	0.303	2.5787E-58	Neu
UQCRH1	1.8248E-62	0.32098575	0.124	0.352	2.8049E-58	Neu
VIRMA1	1.8557E-62	0.12262947	0.1	0.313	2.8524E-58	Neu
LIN54	1.9161E-62	0.19733672	0.101	0.315	2.9453E-58	Neu
LMBRD12	2.0033E-62	0.16991197	0.415	0.835	3.0793E-58	Neu
ARFGAP11	2.0711E-62	0.16341325	0.068	0.258	3.1836E-58	Neu
ACAP31	2.1E-62	0.15415436	0.086	0.287	3.2279E-58	Neu
SMAD51	2.1956E-62	0.29808249	0.129	0.364	3.3749E-58	Neu
TSTD2	2.2931E-62	0.1417424	0.074	0.27	3.5247E-58	Neu
RYBP2	2.3245E-62	0.68821078	0.131	0.367	3.573E-58	Neu
VPS50	2.6318E-62	0.18730529	0.109	0.328	4.0453E-58	Neu
CSTF3	2.641E-62	0.4007039	0.174	0.437	4.0595E-58	Neu
ZBTB48	2.7008E-62	0.19246892	0.086	0.289	4.1514E-58	Neu
CDK5RAP1	2.7702E-62	0.19643796	0.099	0.313	4.2581E-58	Neu
ENPP41	2.8669E-62	0.10709009	0.444	0.849	4.4067E-58	Neu
SMURF11	3.0258E-62	0.17358513	0.086	0.29	4.6509E-58	Neu
NR4A13	3.0395E-62	0.26757796	0.199	0.463	4.6719E-58	Neu
TNFRSF1A2	3.0886E-62	0.37961214	0.45	0.876	4.7475E-58	Neu
NHLRC21	3.34E-62	0.29865518	0.116	0.338	5.1339E-58	Neu
UQCRFS1	3.6058E-62	0.30478776	0.102	0.319	5.5425E-58	Neu
DHX29	3.6834E-62	0.4242273	0.177	0.445	5.6617E-58	Neu
EIF3G	4.1716E-62	0.32384764	0.123	0.35	6.4122E-58	Neu
CUL4B	4.2671E-62	0.34236398	0.16	0.414	6.5589E-58	Neu
FBXO33	4.2761E-62	0.31834503	0.093	0.302	6.5727E-58	Neu
DPM2	4.4021E-62	0.23642015	0.078	0.276	6.7664E-58	Neu
OARD1	4.5667E-62	0.19467867	0.124	0.352	7.0195E-58	Neu
RPA21	5.0845E-62	0.17045127	0.103	0.319	7.8153E-58	Neu
HTRA2	5.3934E-62	0.32406601	0.104	0.321	8.2902E-58	Neu
VWA81	5.4779E-62	0.14929134	0.141	0.381	8.42E-58	Neu
RNF1381	5.5705E-62	0.31975875	0.136	0.374	8.5625E-58	Neu
SLC22A153	5.9942E-62	0.18284791	0.109	0.325	9.2137E-58	Neu
RNMT3	6.1007E-62	0.28961893	0.118	0.341	9.3774E-58	Neu
AMFR1	6.1607E-62	0.13616717	0.453	0.865	9.4696E-58	Neu
DOT1L2	6.1719E-62	0.46444666	0.122	0.347	9.4868E-58	Neu
FARP11	6.2438E-62	0.14249424	0.106	0.319	9.5973E-58	Neu
RHOT11	6.3599E-62	0.34544908	0.339	0.726	9.7758E-58	Neu
AIDA	6.4536E-62	0.19510669	0.116	0.34	9.9198E-58	Neu
SMARCD11	6.7616E-62	0.26757381	0.11	0.329	1.0393E-57	Neu
FBXO251	7.641E-62	0.30897324	0.143	0.385	1.1745E-57	Neu
GZF12	9.9483E-62	0.26954482	0.086	0.287	1.5292E-57	Neu
FAM131A1	1.02E-61	0.50009117	0.082	0.281	1.5679E-57	Neu
ARPC1A	1.0508E-61	0.29154134	0.117	0.342	1.6152E-57	Neu
DMTF12	1.0637E-61	0.30339964	0.126	0.353	1.6349E-57	Neu

NCOA2	1.0644E-61	0.30834979	0.119	0.342	1.6361E-57	Neu
ADPGK	1.1613E-61	0.18958138	0.454	0.876	1.785E-57	Neu
SH3BP52	1.1752E-61	0.14099096	0.477	0.889	1.8064E-57	Neu
DUSP111	1.1863E-61	0.42662456	0.124	0.352	1.8234E-57	Neu
MBNL3	1.3706E-61	0.30854155	0.144	0.386	2.1067E-57	Neu
CLEC4A2	1.3866E-61	0.44659783	0.273	0.597	2.1313E-57	Neu
EPC11	1.4866E-61	0.37905839	0.145	0.386	2.2851E-57	Neu
TMEFF11	1.5935E-61	0.11379479	0.077	0.271	2.4493E-57	Neu
MSANTD31	1.6384E-61	0.15656369	0.092	0.296	2.5185E-57	Neu
TBCK1	1.6565E-61	0.3028981	0.103	0.316	2.5463E-57	Neu
RBBP71	1.7099E-61	0.19660865	0.11	0.328	2.6283E-57	Neu
PPP2R2D	1.812E-61	0.46265192	0.182	0.452	2.7852E-57	Neu
MAP3K131	1.8574E-61	0.58516341	0.125	0.358	2.855E-57	Neu
C19orf25	1.8946E-61	0.32261142	0.101	0.312	2.9121E-57	Neu
KLHL71	2.0446E-61	0.11329272	0.078	0.273	3.1428E-57	Neu
NSUN31	2.0934E-61	0.27028195	0.101	0.313	3.2177E-57	Neu
TMBIM4	2.1581E-61	0.30893105	0.357	0.745	3.3172E-57	Neu
RC3H1	2.2278E-61	0.47175288	0.158	0.408	3.4244E-57	Neu
TIMM9	2.4438E-61	0.46027649	0.129	0.362	3.7563E-57	Neu
ARFIP22	2.8464E-61	0.24036663	0.089	0.293	4.3751E-57	Neu
EIF2S3	2.8853E-61	0.44769593	0.231	0.534	4.4349E-57	Neu
NLRX11	2.9181E-61	0.13290816	0.076	0.271	4.4855E-57	Neu
LENG11	3.142E-61	0.27694718	0.089	0.293	4.8296E-57	Neu
PRKCD1	3.2249E-61	0.1675277	0.492	0.903	4.957E-57	Neu
NDC11	3.2338E-61	0.21309956	0.112	0.331	4.9707E-57	Neu
FIG41	3.6224E-61	0.20719248	0.419	0.834	5.568E-57	Neu
BTG23	3.6456E-61	0.65725272	0.38	0.758	5.6036E-57	Neu
CEP1041	3.6825E-61	0.22949858	0.122	0.348	5.6604E-57	Neu
NOL91	3.9855E-61	0.18880135	0.093	0.299	6.1261E-57	Neu
FBXL19	4.0064E-61	0.24815101	0.071	0.261	6.1582E-57	Neu
ZNF5622	4.1709E-61	0.18731975	0.108	0.324	6.411E-57	Neu
CRBN	4.2398E-61	0.19919874	0.135	0.367	6.517E-57	Neu
PIAS21	4.2531E-61	0.47632607	0.173	0.433	6.5374E-57	Neu
IL13RA13	4.3642E-61	0.16808241	0.423	0.833	6.7082E-57	Neu
USP151	4.5349E-61	0.68298772	0.322	0.693	6.9706E-57	Neu
SF3B11	4.8155E-61	0.51809258	0.175	0.437	7.4019E-57	Neu
SFPQ	5.3337E-61	0.34127641	0.112	0.332	8.1984E-57	Neu
FOSL22	5.3386E-61	0.53181241	0.394	0.782	8.2059E-57	Neu
CES2	5.971E-61	0.14889192	0.094	0.3	9.178E-57	Neu
DCUN1D5	5.9767E-61	0.40166191	0.094	0.3	9.1868E-57	Neu
ZNF277	6.1382E-61	0.12853584	0.118	0.34	9.435E-57	Neu
TTLL5	6.1451E-61	0.31630875	0.157	0.4	9.4456E-57	Neu
TBC1D131	6.1852E-61	0.12907827	0.098	0.307	9.5073E-57	Neu
N4BP13	6.1967E-61	0.51389911	0.341	0.717	9.5249E-57	Neu
ZFAND3	6.2822E-61	0.35258759	0.427	0.85	9.6564E-57	Neu
USP33	6.7458E-61	0.37701376	0.309	0.67	1.0369E-56	Neu
ARL151	7.1583E-61	0.14344561	0.106	0.317	1.1003E-56	Neu
WDR74	7.2428E-61	0.28573842	0.088	0.291	1.1133E-56	Neu
CCDC43	7.5539E-61	0.14573243	0.087	0.287	1.1611E-56	Neu
QPCTL1	7.6002E-61	0.13802033	0.062	0.245	1.1682E-56	Neu
CBX64	7.8647E-61	0.1273033	0.07	0.258	1.2089E-56	Neu
CHD71	8.4487E-61	0.42243517	0.137	0.369	1.2987E-56	Neu

FAM172A	8.863E-61	0.53015543	0.14	0.376	1.3623E-56	Neu
ARHGAP351	8.8653E-61	0.18797771	0.131	0.361	1.3627E-56	Neu
ZBTB221	8.8945E-61	0.17503943	0.107	0.322	1.3672E-56	Neu
BET11	1.1365E-60	0.10201143	0.087	0.289	1.7469E-56	Neu
AGO22	1.1534E-60	0.59671734	0.16	0.411	1.7729E-56	Neu
NR3C11	1.1603E-60	0.24671714	0.411	0.825	1.7835E-56	Neu
ABL23	1.2682E-60	0.24431997	0.095	0.3	1.9494E-56	Neu
KYNU3	1.3021E-60	0.39103108	0.358	0.737	2.0015E-56	Neu
NFYA	1.473E-60	0.26540236	0.104	0.316	2.2641E-56	Neu
ATP5PF	1.6248E-60	0.33777659	0.097	0.305	2.4975E-56	Neu
ANKRD121	1.6573E-60	0.28178746	0.149	0.391	2.5475E-56	Neu
PLSCR13	1.6933E-60	0.16427537	0.467	0.875	2.6028E-56	Neu
AP1S24	1.8809E-60	0.17366796	0.107	0.32	2.8911E-56	Neu
TRAF6	1.9078E-60	0.38821743	0.094	0.298	2.9325E-56	Neu
P2RX11	1.9493E-60	0.3224292	0.16	0.402	2.9963E-56	Neu
EDEM13	2.0574E-60	0.19671244	0.404	0.814	3.1625E-56	Neu
GPATCH11	2.0708E-60	0.12801977	0.132	0.363	3.183E-56	Neu
LMBR1L1	2.1006E-60	0.15659403	0.1	0.305	3.2288E-56	Neu
USP391	2.1121E-60	0.25295634	0.11	0.328	3.2465E-56	Neu
ARID1B1	2.1292E-60	0.24725615	0.116	0.337	3.2728E-56	Neu
GTF2F21	2.3213E-60	0.33089851	0.106	0.318	3.5681E-56	Neu
HMGXB41	2.4299E-60	0.18458728	0.091	0.293	3.735E-56	Neu
KMT2C2	2.7482E-60	0.39256	0.369	0.771	4.2242E-56	Neu
THOC1	2.9971E-60	0.18119677	0.107	0.319	4.6069E-56	Neu
RPRD1B	3.1752E-60	0.21958553	0.088	0.289	4.8806E-56	Neu
BORCS5	3.2585E-60	0.21590881	0.103	0.31	5.0087E-56	Neu
NR1D21	3.3735E-60	0.22764597	0.264	0.574	5.1854E-56	Neu
BRIX11	3.7292E-60	0.36445625	0.111	0.329	5.7321E-56	Neu
CYFIP22	3.8647E-60	0.27300107	0.1	0.306	5.9404E-56	Neu
EYA31	3.899E-60	0.68546104	0.103	0.315	5.9932E-56	Neu
UPRT	4.0342E-60	0.26941584	0.084	0.281	6.201E-56	Neu
OSGIN21	4.3538E-60	0.27637697	0.163	0.414	6.6922E-56	Neu
SUPT7L	4.6156E-60	0.23049677	0.095	0.3	7.0946E-56	Neu
JARID23	4.6615E-60	0.37962451	0.106	0.317	7.1652E-56	Neu
SLC6A61	4.7263E-60	0.30231224	0.461	0.875	7.2647E-56	Neu
IL1B2	4.9897E-60	1.4677549	0.204	0.474	7.6697E-56	Neu
UHRF1BP1L	5.0331E-60	0.43731702	0.131	0.36	7.7364E-56	Neu
CCNT22	5.1402E-60	0.31080663	0.148	0.383	7.901E-56	Neu
NFAT52	5.7294E-60	0.59275714	0.147	0.389	8.8067E-56	Neu
ZCCHC17	5.7313E-60	0.27511383	0.097	0.303	8.8096E-56	Neu
CNOT10	5.7618E-60	0.2860478	0.132	0.361	8.8565E-56	Neu
BDH2	5.8195E-60	0.15476462	0.088	0.289	8.9452E-56	Neu
C12orf4	6.2704E-60	0.46624954	0.11	0.325	9.6383E-56	Neu
NDUFB51	6.5797E-60	0.14299427	0.444	0.864	1.0114E-55	Neu
KIF3B	6.7214E-60	0.38268411	0.109	0.325	1.0331E-55	Neu
BORCS81	6.9029E-60	0.20149113	0.092	0.293	1.061E-55	Neu
DEPDC52	7.151E-60	0.31527629	0.132	0.36	1.0992E-55	Neu
NXPE3	7.7232E-60	0.34676376	0.237	0.539	1.1871E-55	Neu
MTO1	8.022E-60	0.32825167	0.132	0.359	1.2331E-55	Neu
NIFK2	8.2368E-60	0.27839915	0.108	0.324	1.2661E-55	Neu
UBE2R2	9.0882E-60	0.18756431	0.448	0.876	1.3969E-55	Neu
TENT4B1	9.0912E-60	0.26988312	0.103	0.313	1.3974E-55	Neu

NMRK11	9.6948E-60	0.32056267	0.132	0.361	1.4902E-55	Neu
OTUD6B	1.0277E-59	0.15607469	0.1	0.305	1.5797E-55	Neu
FAM192A1	1.0372E-59	0.12165429	0.075	0.266	1.5943E-55	Neu
BABAM2	1.1632E-59	0.24225903	0.109	0.323	1.788E-55	Neu
H3F3B3	1.2367E-59	0.24372632	0.079	0.271	1.9009E-55	Neu
R3HDM11	1.2636E-59	0.18641324	0.112	0.329	1.9423E-55	Neu
PTPMT11	1.3154E-59	0.33923434	0.11	0.326	2.0219E-55	Neu
ECSCR1	1.3739E-59	0.37740994	0.296	0.632	2.1118E-55	Neu
RPE1	1.4209E-59	0.48698093	0.098	0.304	2.184E-55	Neu
CIZ11	1.4903E-59	0.14583962	0.078	0.271	2.2908E-55	Neu
ADORA11	1.5298E-59	0.21589937	0.087	0.285	2.3515E-55	Neu
XPA1	1.6669E-59	0.29423766	0.119	0.34	2.5622E-55	Neu
ALDH3A21	1.8108E-59	0.12298952	0.46	0.882	2.7834E-55	Neu
TET33	1.8406E-59	0.52581193	0.105	0.314	2.8292E-55	Neu
FRYL	2.0187E-59	0.28100106	0.163	0.411	3.1029E-55	Neu
CDK17	2.1509E-59	0.14701325	0.104	0.312	3.3062E-55	Neu
HACD23	2.2934E-59	0.20281516	0.104	0.313	3.5252E-55	Neu
HK21	2.36E-59	0.13900459	0.439	0.85	3.6275E-55	Neu
STXBP51	2.3738E-59	0.35969264	0.109	0.321	3.6488E-55	Neu
R3HCC1L	2.4377E-59	0.25076788	0.099	0.305	3.747E-55	Neu
PSMB7	2.5531E-59	0.19996969	0.092	0.295	3.9244E-55	Neu
UQCC1	2.6373E-59	0.18090343	0.12	0.338	4.0538E-55	Neu
TMEM2731	2.6898E-59	0.13872654	0.388	0.768	4.1345E-55	Neu
TLK21	2.7434E-59	0.29028974	0.085	0.282	4.2169E-55	Neu
CLEC4E1	2.8033E-59	0.54168788	0.177	0.429	4.3089E-55	Neu
SIKE12	2.8377E-59	0.25015245	0.128	0.355	4.3619E-55	Neu
RXRA1	2.9667E-59	0.22608749	0.493	0.91	4.5602E-55	Neu
VSIR3	3.1029E-59	0.46293635	0.115	0.327	4.7695E-55	Neu
WIPI11	3.1144E-59	0.42019551	0.087	0.287	4.7871E-55	Neu
STAG11	3.3513E-59	0.49225812	0.207	0.486	5.1513E-55	Neu
NMNAT12	3.3877E-59	0.16878306	0.086	0.281	5.2073E-55	Neu
SELENOW1	3.7463E-59	0.10779344	0.471	0.868	5.7585E-55	Neu
PIGG	4.1299E-59	0.30297411	0.09	0.289	6.348E-55	Neu
KLHL201	4.349E-59	0.10501333	0.08	0.27	6.6848E-55	Neu
DNAAF41	4.5609E-59	0.31498336	0.125	0.349	7.0106E-55	Neu
TAGLN1	4.8905E-59	0.31452093	0.387	0.771	7.5172E-55	Neu
GLYCTK1	5.04E-59	0.23527151	0.088	0.285	7.747E-55	Neu
ING41	6.0042E-59	0.11417354	0.083	0.276	9.229E-55	Neu
DUSP62	6.5965E-59	0.34614641	0.09	0.286	1.0139E-54	Neu
CLASRP2	6.8064E-59	0.4090239	0.152	0.387	1.0462E-54	Neu
IKBKE	7.1018E-59	0.21194441	0.072	0.257	1.0916E-54	Neu
FGD43	7.2302E-59	0.56864229	0.291	0.633	1.1114E-54	Neu
RNF2141	8.0577E-59	0.12842615	0.096	0.299	1.2385E-54	Neu
DMXL23	8.8245E-59	0.26118429	0.402	0.803	1.3564E-54	Neu
NARF	8.8448E-59	0.36716768	0.134	0.359	1.3595E-54	Neu
NKAPD11	8.8935E-59	0.2996031	0.119	0.337	1.367E-54	Neu
NAE11	9.4085E-59	0.38066013	0.109	0.321	1.4462E-54	Neu
THAP121	9.7182E-59	0.27990177	0.119	0.336	1.4938E-54	Neu
GFM21	9.7638E-59	0.21723731	0.097	0.298	1.5008E-54	Neu
ARMC11	9.9335E-59	0.13205973	0.077	0.268	1.5269E-54	Neu
LTB4R	1.0731E-58	0.26861275	0.134	0.355	1.6495E-54	Neu
ATP5MC1	1.1639E-58	0.24269628	0.1	0.307	1.789E-54	Neu

KCTD2	1.2256E-58	0.12687011	0.074	0.261	1.8838E-54	Neu
TP53INP21	1.2819E-58	0.51712228	0.079	0.269	1.9704E-54	Neu
CCDC91	1.4453E-58	0.76662278	0.136	0.365	2.2215E-54	Neu
PPP2R3A1	1.4634E-58	0.26539228	0.113	0.326	2.2493E-54	Neu
UCHL51	1.4815E-58	0.10307315	0.092	0.292	2.2772E-54	Neu
FBXO30	1.5997E-58	0.21818912	0.107	0.318	2.4589E-54	Neu
SEMA4A3	1.6828E-58	0.21989516	0.039	0.2	2.5867E-54	Neu
WWOX	1.7461E-58	0.26437137	0.136	0.362	2.6839E-54	Neu
MORF4L21	1.7792E-58	0.22119414	0.092	0.292	2.7349E-54	Neu
BUD131	1.831E-58	0.22342841	0.119	0.336	2.8145E-54	Neu
BOD11	1.8334E-58	0.10785799	0.083	0.274	2.8181E-54	Neu
CDKN2D1	1.8741E-58	0.24616731	0.119	0.335	2.8807E-54	Neu
INSR1	1.9268E-58	0.1607872	0.091	0.289	2.9617E-54	Neu
REL3	2.019E-58	0.42745991	0.339	0.712	3.1034E-54	Neu
ZBTB251	2.1712E-58	0.29300633	0.14	0.37	3.3373E-54	Neu
CCNT1	2.3816E-58	0.29959663	0.123	0.341	3.6608E-54	Neu
CUL5	2.4323E-58	0.14770507	0.133	0.359	3.7387E-54	Neu
AKAP8	2.5891E-58	0.3176046	0.115	0.327	3.9797E-54	Neu
ZC3H12A3	2.591E-58	0.7575564	0.095	0.294	3.9826E-54	Neu
ARHGAP61	2.6412E-58	0.24007301	0.123	0.341	4.0598E-54	Neu
ADNP	2.8521E-58	0.1519432	0.088	0.284	4.3839E-54	Neu
VPS181	2.9581E-58	0.2312628	0.061	0.239	4.5468E-54	Neu
ADGRE31	2.9663E-58	0.3957379	0.133	0.351	4.5596E-54	Neu
MED6	3.1166E-58	0.31797975	0.125	0.347	4.7905E-54	Neu
NEPRO	3.1293E-58	0.25790699	0.092	0.29	4.8101E-54	Neu
ZNF780A	3.1538E-58	0.24866244	0.089	0.284	4.8477E-54	Neu
DTNA1	3.154E-58	0.32063013	0.106	0.309	4.8481E-54	Neu
CNOT41	3.2519E-58	0.21699084	0.097	0.299	4.9985E-54	Neu
DDX521	3.4055E-58	0.23063857	0.102	0.307	5.2346E-54	Neu
GYS13	3.431E-58	0.19921148	0.078	0.266	5.2738E-54	Neu
CEMIP22	3.5776E-58	0.15163241	0.052	0.224	5.4992E-54	Neu
ZBTB14	3.6543E-58	0.17258628	0.11	0.319	5.617E-54	Neu
ARID4A	3.7594E-58	0.28884901	0.115	0.327	5.7786E-54	Neu
ZNF3971	3.8006E-58	0.13551568	0.082	0.273	5.8419E-54	Neu
RCHY11	3.8335E-58	0.24448125	0.101	0.304	5.8925E-54	Neu
DHRS12	3.956E-58	0.12810055	0.076	0.263	6.0808E-54	Neu
DST1	4.07E-58	0.41535805	0.346	0.726	6.256E-54	Neu
TRPM43	4.376E-58	0.23072507	0.065	0.244	6.7264E-54	Neu
IFITM23	4.5218E-58	1.11802251	0.136	0.366	6.9504E-54	Neu
LUC7L5	4.6977E-58	0.43039197	0.159	0.398	7.2209E-54	Neu
CLCN53	4.8059E-58	0.22619062	0.092	0.29	7.3872E-54	Neu
MANEA1	4.9004E-58	0.39750321	0.101	0.307	7.5323E-54	Neu
MROH61	5.0695E-58	0.46834473	0.122	0.339	7.7924E-54	Neu
KLHL151	5.239E-58	0.13739703	0.069	0.251	8.0529E-54	Neu
NLRC52	5.502E-58	0.13390332	0.082	0.27	8.4572E-54	Neu
SEC24B	6.1088E-58	0.5473919	0.202	0.478	9.3899E-54	Neu
PTPN111	6.118E-58	0.43943999	0.099	0.305	9.404E-54	Neu
LYST1	6.2612E-58	0.57930326	0.293	0.63	9.6241E-54	Neu
INVS1	6.6489E-58	0.40578058	0.101	0.304	1.022E-53	Neu
FBXL12	6.6787E-58	0.35479875	0.072	0.255	1.0266E-53	Neu
VPS291	6.8627E-58	0.50213319	0.108	0.318	1.0549E-53	Neu
RASSF51	7.2414E-58	0.16778206	0.475	0.882	1.1131E-53	Neu

KCNC33	7.4637E-58	0.19727871	0.115	0.325	1.1472E-53	Neu
ZHX1	8.1454E-58	0.42364159	0.137	0.37	1.252E-53	Neu
ZNF5111	9.0786E-58	0.35243342	0.093	0.292	1.3955E-53	Neu
PHKA21	9.1581E-58	0.11468665	0.091	0.286	1.4077E-53	Neu
CWC27	9.5269E-58	0.37378303	0.129	0.354	1.4644E-53	Neu
ZDHHC24	1.0191E-57	0.22700792	0.065	0.245	1.5664E-53	Neu
SOWAHD1	1.0345E-57	0.50822772	0.088	0.284	1.5901E-53	Neu
E4F1	1.0677E-57	0.23905983	0.101	0.305	1.6411E-53	Neu
CAPZA22	1.3424E-57	0.19384155	0.432	0.85	2.0634E-53	Neu
VPS82	1.3508E-57	0.74603014	0.225	0.515	2.0763E-53	Neu
POLR3E1	1.3786E-57	0.10533626	0.081	0.27	2.1191E-53	Neu
FOXJ31	1.5502E-57	0.35035635	0.127	0.349	2.3828E-53	Neu
SNX18	1.641E-57	0.22771349	0.067	0.248	2.5223E-53	Neu
SLC16A51	1.9129E-57	0.25474385	0.099	0.297	2.9404E-53	Neu
SNTB2	2.0205E-57	0.21190644	0.096	0.296	3.1058E-53	Neu
LPAR31	2.1634E-57	0.15833023	0.142	0.37	3.3253E-53	Neu
LILRA2	2.3754E-57	0.13813873	0.155	0.39	3.6512E-53	Neu
GGCT1	2.6876E-57	0.33748007	0.097	0.297	4.1311E-53	Neu
RESF1	2.7639E-57	0.63279036	0.289	0.62	4.2485E-53	Neu
APPBP21	2.764E-57	0.30026335	0.102	0.305	4.2486E-53	Neu
CDKAL11	3.0055E-57	0.31435794	0.127	0.344	4.6198E-53	Neu
PTGFRN	3.2611E-57	0.27944839	0.064	0.241	5.0127E-53	Neu
MFN11	4.2827E-57	0.28529784	0.09	0.285	6.583E-53	Neu
PPP1R182	4.6816E-57	0.19495272	0.47	0.888	7.196E-53	Neu
STK24	4.9565E-57	0.12821284	0.476	0.897	7.6186E-53	Neu
ILKAP	4.9644E-57	0.44168782	0.11	0.319	7.6308E-53	Neu
ARID2	5.0156E-57	0.27867121	0.107	0.312	7.7095E-53	Neu
SPAST1	5.0973E-57	0.13359323	0.094	0.29	7.8351E-53	Neu
DDIT31	5.7094E-57	1.04324479	0.129	0.351	8.776E-53	Neu
SNX11	5.817E-57	0.53135729	0.075	0.26	8.9414E-53	Neu
TAOK1	6.1234E-57	0.29685523	0.414	0.831	9.4123E-53	Neu
ZNF1212	6.8107E-57	0.16754912	0.117	0.327	1.0469E-52	Neu
RNF21	6.8579E-57	0.12592254	0.092	0.289	1.0541E-52	Neu
COQ61	6.8647E-57	0.38289358	0.082	0.272	1.0552E-52	Neu
CNTRL1	7.7736E-57	0.34912649	0.353	0.731	1.1949E-52	Neu
TNPO21	7.9748E-57	0.41101352	0.094	0.292	1.2258E-52	Neu
SSH21	9.1284E-57	0.68784747	0.153	0.39	1.4031E-52	Neu
SORL12	1.0019E-56	0.70888689	0.175	0.422	1.54E-52	Neu
OMA1	1.2946E-56	0.23803729	0.136	0.36	1.9899E-52	Neu
IPO11	1.3738E-56	0.32520592	0.09	0.283	2.1117E-52	Neu
TRHDE1	1.4573E-56	0.23811043	0.204	0.467	2.24E-52	Neu
AMZ21	1.5168E-56	0.20544754	0.102	0.305	2.3314E-52	Neu
PLSCR32	1.667E-56	0.14355219	0.063	0.238	2.5623E-52	Neu
MYL12B	1.7866E-56	0.30815783	0.128	0.349	2.7461E-52	Neu
RBBP6	1.8534E-56	0.28675661	0.1	0.299	2.8489E-52	Neu
SLC19A31	1.9728E-56	0.12693678	0.36	0.7	3.0323E-52	Neu
SUGP1	2.0887E-56	0.15090084	0.064	0.241	3.2106E-52	Neu
MIOS3	2.4313E-56	0.20540173	0.124	0.34	3.7372E-52	Neu
ZNF1432	2.5848E-56	0.11786618	0.081	0.267	3.9731E-52	Neu
YAF2	2.7788E-56	0.20828369	0.097	0.294	4.2713E-52	Neu
CDKN1A3	2.8012E-56	0.26853193	0.131	0.341	4.3057E-52	Neu
UPF2	3.0168E-56	0.46738487	0.153	0.389	4.6371E-52	Neu

RGL23	3.1388E-56	0.79925721	0.129	0.348	4.8246E-52	Neu
MRTO41	3.1794E-56	0.20386713	0.072	0.253	4.8871E-52	Neu
WDR44	4.385E-56	0.18861824	0.085	0.275	6.7402E-52	Neu
FAM210B	4.6691E-56	0.42156457	0.145	0.375	7.1769E-52	Neu
RNF1452	4.9785E-56	0.36402814	0.112	0.319	7.6524E-52	Neu
DLG1	5.6902E-56	0.28247631	0.128	0.347	8.7464E-52	Neu
VPS33A1	5.7262E-56	0.25321404	0.079	0.263	8.8018E-52	Neu
CXorf214	6.3198E-56	0.44756744	0.143	0.369	9.7142E-52	Neu
KMT2E3	6.9685E-56	0.49362599	0.309	0.657	1.0711E-51	Neu
ATG121	7.1892E-56	0.34015249	0.112	0.318	1.105E-51	Neu
ZBTB211	7.301E-56	0.20253412	0.106	0.307	1.1222E-51	Neu
PTAR12	7.4085E-56	0.25107005	0.108	0.311	1.1388E-51	Neu
TARBP1	7.6263E-56	0.40550439	0.129	0.347	1.1722E-51	Neu
TEN11	8.5548E-56	0.43212968	0.093	0.288	1.315E-51	Neu
GFER1	9.2936E-56	0.157159	0.074	0.255	1.4285E-51	Neu
NR4A33	9.3637E-56	0.25970415	0.225	0.483	1.4393E-51	Neu
FUNDC1	1.0132E-55	0.10873148	0.081	0.267	1.5574E-51	Neu
KLF63	1.0394E-55	0.31595894	0.44	0.826	1.5977E-51	Neu
MBNL22	1.0539E-55	0.14141615	0.103	0.301	1.62E-51	Neu
VPS13B2	1.0816E-55	0.56998727	0.21	0.482	1.6625E-51	Neu
OPLAH1	1.0866E-55	0.27309045	0.067	0.243	1.6702E-51	Neu
GNAQ3	1.1258E-55	0.33367545	0.461	0.881	1.7305E-51	Neu
RFK1	1.1713E-55	0.39188323	0.107	0.313	1.8004E-51	Neu
BRF11	1.1719E-55	0.1686494	0.069	0.246	1.8014E-51	Neu
TMEM2561	1.2066E-55	0.12523417	0.092	0.286	1.8547E-51	Neu
TMEM1681	1.2536E-55	0.28502446	0.123	0.335	1.9269E-51	Neu
ABHD111	1.2773E-55	0.24715537	0.096	0.29	1.9634E-51	Neu
BRD3OS	1.3402E-55	0.1640726	0.08	0.266	2.0601E-51	Neu
NLRP11	1.356E-55	0.58712855	0.31	0.65	2.0843E-51	Neu
CYB5R21	1.361E-55	0.28482729	0.075	0.257	2.0919E-51	Neu
CARNMT11	1.4097E-55	0.17683772	0.116	0.324	2.1669E-51	Neu
DPYD2	1.4576E-55	0.40185477	0.378	0.769	2.2404E-51	Neu
MOSPD11	1.4643E-55	0.17194115	0.075	0.256	2.2508E-51	Neu
BBC32	1.4662E-55	0.21330409	0.054	0.221	2.2537E-51	Neu
RPF21	1.5384E-55	0.10154854	0.077	0.26	2.3646E-51	Neu
SMG8	1.5528E-55	0.26456179	0.076	0.259	2.3867E-51	Neu
RCAN11	1.6194E-55	0.24025351	0.064	0.239	2.4891E-51	Neu
SPG212	1.7636E-55	0.10339847	0.46	0.875	2.7108E-51	Neu
PAN21	1.8727E-55	0.38956382	0.115	0.324	2.8786E-51	Neu
CAAP11	1.9157E-55	0.19601764	0.072	0.252	2.9445E-51	Neu
WDR71	1.9402E-55	0.12826962	0.058	0.228	2.9823E-51	Neu
UBQLN4	1.9428E-55	0.18824812	0.083	0.269	2.9863E-51	Neu
KIF1BP1	2.4373E-55	0.32442803	0.08	0.267	3.7464E-51	Neu
ASRGL1	2.5066E-55	0.16599205	0.06	0.23	3.8529E-51	Neu
PSKH1	2.5189E-55	0.405297	0.131	0.35	3.8718E-51	Neu
JAGN1	2.5579E-55	0.1274726	0.095	0.289	3.9317E-51	Neu
SLC45A41	2.6792E-55	0.43851478	0.107	0.309	4.1182E-51	Neu
TRPT1	3.1812E-55	0.24060481	0.099	0.295	4.8898E-51	Neu
LUC7L33	3.2703E-55	0.32351859	0.228	0.508	5.0268E-51	Neu
HMBS1	3.6059E-55	0.24211548	0.094	0.287	5.5427E-51	Neu
SLC25A161	3.6245E-55	0.31552526	0.136	0.356	5.5711E-51	Neu
SLC26A112	4.0018E-55	0.20740799	0.047	0.208	6.1511E-51	Neu

TBC1D7	4.4651E-55	0.93606121	0.094	0.289	6.8632E-51	Neu
KIAA11431	4.4713E-55	0.27560536	0.133	0.352	6.8728E-51	Neu
CXCR11	4.5719E-55	8.17182388	0.011	0.001	7.0275E-51	Neu
TXNDC91	4.6313E-55	0.39368696	0.102	0.299	7.1187E-51	Neu
DPH21	5.0553E-55	0.38360693	0.091	0.282	7.7705E-51	Neu
CIPC1	5.4226E-55	0.1697441	0.09	0.279	8.335E-51	Neu
TEC1	5.6531E-55	0.30215719	0.093	0.285	8.6895E-51	Neu
FRY1	5.8394E-55	0.51200018	0.154	0.386	8.9757E-51	Neu
NFKBIZ1	5.9467E-55	0.72427926	0.25	0.542	9.1407E-51	Neu
LILRA63	6.1428E-55	0.10713529	0.062	0.232	9.4421E-51	Neu
ELL4	6.3048E-55	0.70722138	0.094	0.286	9.6911E-51	Neu
MYSM11	6.5463E-55	0.22862157	0.121	0.328	1.0062E-50	Neu
PIGC1	6.7662E-55	0.15448541	0.081	0.266	1.04E-50	Neu
TFEC3	6.8898E-55	0.33716185	0.393	0.795	1.059E-50	Neu
NXT2	7.8669E-55	0.20116886	0.102	0.299	1.2092E-50	Neu
AREL11	7.9767E-55	0.14098188	0.07	0.247	1.2261E-50	Neu
NOL121	8.3403E-55	0.17999648	0.102	0.299	1.282E-50	Neu
MED71	8.6552E-55	0.23124726	0.117	0.325	1.3304E-50	Neu
TMEM181	9.2986E-55	0.25669862	0.087	0.276	1.4293E-50	Neu
EIF4EBP32	9.943E-55	0.23223709	0.092	0.282	1.5283E-50	Neu
HERC41	1.0517E-54	0.49145323	0.233	0.519	1.6166E-50	Neu
PBX23	1.1117E-54	0.52403461	0.171	0.411	1.7087E-50	Neu
MED11	1.1394E-54	0.42838481	0.095	0.289	1.7514E-50	Neu
RELCH2	1.2537E-54	0.48652326	0.174	0.417	1.9271E-50	Neu
TMEM39B2	1.3255E-54	0.10859654	0.073	0.251	2.0375E-50	Neu
CLOCK1	1.3519E-54	0.14730074	0.102	0.299	2.078E-50	Neu
ZNF6541	1.3581E-54	0.29986507	0.106	0.306	2.0876E-50	Neu
STPG11	1.4153E-54	0.17410199	0.091	0.28	2.1755E-50	Neu
AARS	1.6834E-54	0.24826427	0.102	0.301	2.5876E-50	Neu
EGR33	1.7243E-54	5.83263683	0.03	0.005	2.6504E-50	Neu
RAB281	1.7896E-54	0.4880307	0.096	0.289	2.7507E-50	Neu
SUN11	1.8847E-54	0.14114988	0.11	0.31	2.897E-50	Neu
EXOC6B	1.8935E-54	0.28270155	0.11	0.312	2.9105E-50	Neu
ARPC31	1.9117E-54	0.31047624	0.409	0.815	2.9384E-50	Neu
MED16	2.1062E-54	0.28826704	0.069	0.243	3.2375E-50	Neu
DGCR8	2.187E-54	0.25101496	0.085	0.269	3.3616E-50	Neu
KAT6A2	2.4085E-54	0.55103018	0.201	0.464	3.7022E-50	Neu
STX72	2.4184E-54	0.31422315	0.434	0.843	3.7173E-50	Neu
MEGF91	2.4913E-54	0.21756282	0.426	0.836	3.8294E-50	Neu
FNIP22	2.5288E-54	0.27270071	0.06	0.228	3.887E-50	Neu
POLB1	2.594E-54	0.32447887	0.08	0.263	3.9872E-50	Neu
TAPT1	2.7258E-54	0.42752083	0.118	0.325	4.1899E-50	Neu
TCTN11	2.8871E-54	0.20214039	0.082	0.265	4.4377E-50	Neu
CCDC1171	3.4226E-54	0.29607429	0.093	0.284	5.2609E-50	Neu
ARMCX31	3.6386E-54	0.40416953	0.087	0.272	5.5929E-50	Neu
YPEL53	3.6513E-54	0.27292679	0.443	0.855	5.6125E-50	Neu
PLEK3	3.6522E-54	0.53602573	0.512	0.904	5.6138E-50	Neu
TOP2B1	4.0189E-54	0.16320132	0.115	0.321	6.1775E-50	Neu
BTBD101	4.3918E-54	0.2126522	0.07	0.246	6.7507E-50	Neu
CXCL32	4.5E-54	0.1413167	0.356	0.68	6.917E-50	Neu
CEP85	4.643E-54	0.18361679	0.082	0.264	7.1367E-50	Neu
CEP44	4.7186E-54	0.34938234	0.125	0.334	7.2529E-50	Neu

RSAD22	4.8133E-54	0.39894058	0.049	0.208	7.3985E-50	Neu
LIG32	5.6582E-54	0.10053944	0.089	0.277	8.6973E-50	Neu
SUGP22	5.81E-54	0.33818153	0.199	0.452	8.9306E-50	Neu
KIAA0556	6.5038E-54	0.38484671	0.078	0.258	9.997E-50	Neu
ZNF581	7.1296E-54	0.39625345	0.127	0.339	1.0959E-49	Neu
BIRC2	7.5612E-54	0.59090053	0.162	0.4	1.1622E-49	Neu
PSME3	7.6564E-54	0.10830595	0.073	0.25	1.1769E-49	Neu
PEX3	8.7853E-54	0.14650885	0.079	0.259	1.3504E-49	Neu
CLK12	9.0637E-54	0.53169473	0.219	0.493	1.3932E-49	Neu
CRYZ1	9.3216E-54	0.13673294	0.082	0.264	1.4328E-49	Neu
CCDC130	9.5763E-54	0.24836908	0.112	0.313	1.472E-49	Neu
OGFOD2	1.0402E-53	0.30362184	0.096	0.287	1.5989E-49	Neu
TPM11	1.0756E-53	0.27015241	0.077	0.255	1.6533E-49	Neu
HCFC21	1.154E-53	0.15254516	0.096	0.287	1.7739E-49	Neu
CXCL22	1.1699E-53	0.38465147	0.195	0.439	1.7983E-49	Neu
UPP11	1.2535E-53	0.17511545	0.475	0.885	1.9267E-49	Neu
STK42	1.313E-53	0.45326738	0.423	0.842	2.0183E-49	Neu
CREBZF1	1.3328E-53	0.27261363	0.141	0.357	2.0487E-49	Neu
SNRPE	1.4537E-53	0.10352565	0.088	0.276	2.2345E-49	Neu
MDM43	1.5001E-53	0.42391391	0.253	0.541	2.3058E-49	Neu
INTS13	1.5093E-53	0.28118887	0.092	0.283	2.32E-49	Neu
IFRD12	1.6726E-53	0.36473945	0.331	0.657	2.571E-49	Neu
CASP21	1.8435E-53	0.21291219	0.092	0.281	2.8337E-49	Neu
METTL93	1.8588E-53	0.48491567	0.218	0.486	2.8571E-49	Neu
ARMC10	1.8589E-53	0.52126463	0.155	0.388	2.8573E-49	Neu
CCDC59	1.9271E-53	0.37467818	0.116	0.32	2.9622E-49	Neu
UBE2M	2.0752E-53	0.22098456	0.073	0.249	3.1898E-49	Neu
MALT13	2.114E-53	0.19416712	0.106	0.301	3.2495E-49	Neu
PTRH21	2.1572E-53	0.28291317	0.076	0.253	3.3159E-49	Neu
SUCO	2.2311E-53	0.16705658	0.1	0.292	3.4294E-49	Neu
SCAMP11	2.3436E-53	0.29328361	0.078	0.257	3.6024E-49	Neu
LONRF11	2.5122E-53	0.31351785	0.097	0.289	3.8614E-49	Neu
IRAK41	2.5454E-53	0.4384817	0.147	0.369	3.9126E-49	Neu
ZNF6681	2.7235E-53	0.20421899	0.058	0.223	4.1863E-49	Neu
CGGBP11	2.9709E-53	0.32719808	0.401	0.805	4.5666E-49	Neu
MSH2	3.0824E-53	0.11346136	0.089	0.274	4.7379E-49	Neu
TTI2	3.1159E-53	0.13052523	0.06	0.226	4.7895E-49	Neu
ADAM171	3.3215E-53	0.19822327	0.464	0.884	5.1055E-49	Neu
ZMYM6	3.6975E-53	0.36152916	0.125	0.333	5.6834E-49	Neu
THOC51	4.2743E-53	0.40296315	0.1	0.293	6.57E-49	Neu
CD691	4.3439E-53	0.40875164	0.19	0.425	6.677E-49	Neu
DOCK43	4.4592E-53	1.10981004	0.127	0.338	6.8542E-49	Neu
PDK3	4.482E-53	0.34968808	0.1	0.294	6.8893E-49	Neu
RARS2	4.5919E-53	0.36966804	0.096	0.287	7.0583E-49	Neu
POP5	4.6202E-53	0.19291903	0.111	0.31	7.1018E-49	Neu
ZFC3H12	4.6283E-53	0.73443311	0.195	0.449	7.1141E-49	Neu
PPWD1	5.4088E-53	0.35990604	0.122	0.326	8.3139E-49	Neu
GANC1	5.4547E-53	0.41598508	0.165	0.4	8.3844E-49	Neu
CYB5R42	5.7843E-53	0.60096753	0.35	0.726	8.8911E-49	Neu
IER5L2	6.3868E-53	0.13080086	0.048	0.205	9.8171E-49	Neu
TMEM74B1	6.6225E-53	0.52569841	0.236	0.511	1.0179E-48	Neu
CNTROB1	6.9729E-53	0.15511908	0.063	0.231	1.0718E-48	Neu

ECHDC31	7.0761E-53	0.13296098	0.052	0.211	1.0877E-48	Neu
RASA2	7.1602E-53	0.37587136	0.132	0.345	1.1006E-48	Neu
INKA21	8.41E-53	0.62126673	0.098	0.288	1.2927E-48	Neu
SMYD51	8.8508E-53	0.10007413	0.067	0.236	1.3605E-48	Neu
RNF2132	9.1026E-53	0.25220981	0.495	0.904	1.3992E-48	Neu
DNAJC21	9.2818E-53	0.25971686	0.094	0.28	1.4267E-48	Neu
PROSER11	9.6084E-53	0.23068029	0.112	0.312	1.4769E-48	Neu
MXD13	1.0005E-52	1.41219851	0.224	0.499	1.5378E-48	Neu
IL18BP2	1.1318E-52	0.21732675	0.079	0.255	1.7397E-48	Neu
TBP	1.4199E-52	0.13646248	0.071	0.244	2.1825E-48	Neu
IFT521	1.5227E-52	0.10338658	0.05	0.207	2.3406E-48	Neu
LCORL1	1.5664E-52	0.30687124	0.115	0.316	2.4077E-48	Neu
ZNF2741	1.5734E-52	0.40122359	0.084	0.265	2.4185E-48	Neu
TRMT10C	1.6689E-52	0.10851712	0.086	0.267	2.5653E-48	Neu
ERCC21	1.7853E-52	0.16111076	0.071	0.244	2.7442E-48	Neu
PEX6	1.8044E-52	0.11592332	0.083	0.263	2.7735E-48	Neu
ATG2A	1.8061E-52	1.33663105	0.089	0.274	2.7761E-48	Neu
HDAC43	1.9556E-52	0.23464734	0.084	0.263	3.0059E-48	Neu
ANKRD422	1.9637E-52	0.19201461	0.077	0.253	3.0184E-48	Neu
IDNK	1.9998E-52	0.15040347	0.062	0.229	3.0739E-48	Neu
DCLRE1C	2.0257E-52	0.12409199	0.058	0.221	3.1137E-48	Neu
MYO9A2	2.2166E-52	0.22897083	0.104	0.296	3.4072E-48	Neu
NFKBIB1	2.3245E-52	0.42358958	0.078	0.254	3.573E-48	Neu
CENPP	2.8112E-52	0.16407064	0.065	0.231	4.3211E-48	Neu
ESCO11	2.8441E-52	0.23329008	0.084	0.265	4.3717E-48	Neu
TBC1D161	3.1858E-52	0.28980795	0.06	0.225	4.8969E-48	Neu
SOCS33	3.2155E-52	0.56845968	0.373	0.704	4.9425E-48	Neu
ARRDC32	3.7513E-52	0.63333344	0.163	0.391	5.7661E-48	Neu
CAPN15	3.8786E-52	0.14812611	0.06	0.224	5.9618E-48	Neu
ZBTB184	4.3638E-52	0.47140185	0.107	0.302	6.7075E-48	Neu
TMCC11	4.4586E-52	0.19916288	0.078	0.252	6.8533E-48	Neu
PPP1R37	4.6671E-52	0.31177836	0.066	0.233	7.1739E-48	Neu
SUDS31	4.8371E-52	0.30014865	0.086	0.268	7.4351E-48	Neu
FAM193A	4.9907E-52	0.44572154	0.135	0.346	7.6712E-48	Neu
U2AF1L41	5.0548E-52	0.3657673	0.083	0.262	7.7698E-48	Neu
DDIAS1	5.35E-52	0.3047775	0.145	0.365	8.2235E-48	Neu
THAP6	5.4625E-52	0.32877776	0.074	0.247	8.3965E-48	Neu
MTFR11	5.5214E-52	0.17713384	0.086	0.266	8.487E-48	Neu
LIG4	5.7524E-52	0.30969093	0.074	0.247	8.8421E-48	Neu
TSC22D11	5.8142E-52	0.16702963	0.082	0.259	8.937E-48	Neu
ZNF5801	6.7932E-52	0.23374518	0.106	0.298	1.0442E-47	Neu
DIP2A1	7.0051E-52	0.20173524	0.078	0.252	1.0768E-47	Neu
ENKD1	7.1199E-52	0.2168783	0.078	0.252	1.0944E-47	Neu
NCBP2AS21	7.1573E-52	0.1944279	0.072	0.242	1.1002E-47	Neu
SMIM201	8.3679E-52	0.12922455	0.074	0.247	1.2862E-47	Neu
RPAP11	8.6492E-52	0.15190276	0.078	0.251	1.3295E-47	Neu
FOCAD1	8.9405E-52	0.10962721	0.045	0.197	1.3742E-47	Neu
MSH3	9.3562E-52	0.18828773	0.079	0.255	1.4381E-47	Neu
MED201	9.7225E-52	0.11928092	0.074	0.246	1.4945E-47	Neu
PIM22	1.0218E-51	0.363979	0.091	0.271	1.5706E-47	Neu
FOLR31	1.1009E-51	0.20504717	0.153	0.373	1.6922E-47	Neu
MLLT101	1.3709E-51	0.45988662	0.131	0.337	2.1072E-47	Neu

ATXN7L31	1.4031E-51	0.26013746	0.083	0.261	2.1568E-47	Neu
ACAD81	1.4149E-51	0.3021981	0.079	0.253	2.1748E-47	Neu
DNAJA11	1.4205E-51	0.13838943	0.474	0.874	2.1835E-47	Neu
RXYLT11	1.462E-51	0.14670047	0.108	0.303	2.2472E-47	Neu
MTMR9	1.5104E-51	0.30685725	0.102	0.291	2.3216E-47	Neu
DCP1B	1.631E-51	0.28803907	0.076	0.25	2.507E-47	Neu
OVCH11	1.6477E-51	0.31165313	0.248	0.522	2.5327E-47	Neu
INTS7	1.6658E-51	0.36141021	0.097	0.284	2.5604E-47	Neu
INTS8	1.719E-51	0.3471857	0.132	0.343	2.6423E-47	Neu
ABHD13	1.7613E-51	0.2077127	0.094	0.28	2.7073E-47	Neu
TTLL32	1.8996E-51	0.4644976	0.127	0.328	2.9199E-47	Neu
LRRC421	2.1051E-51	0.20273825	0.069	0.238	3.2357E-47	Neu
FAHD11	2.1782E-51	0.17438501	0.066	0.232	3.3482E-47	Neu
FAM219A1	2.2131E-51	0.23224424	0.075	0.246	3.4017E-47	Neu
PFKFB33	2.2883E-51	1.36736735	0.189	0.432	3.5173E-47	Neu
PRORP1	2.3697E-51	0.41658325	0.1	0.289	3.6425E-47	Neu
AKIRIN2	2.4136E-51	0.15794151	0.527	0.922	3.71E-47	Neu
BICRAL	2.6128E-51	0.35640938	0.143	0.361	4.0161E-47	Neu
AKAP7	2.9084E-51	0.21268532	0.078	0.251	4.4705E-47	Neu
ZNF2811	3.2592E-51	0.16441192	0.091	0.272	5.0098E-47	Neu
ACOX3	3.8744E-51	0.17895772	0.064	0.227	5.9554E-47	Neu
VPS9D1	3.9391E-51	1.19195921	0.129	0.336	6.0547E-47	Neu
MAPK81	3.9692E-51	0.35632954	0.11	0.304	6.1011E-47	Neu
ZSCAN291	3.9927E-51	0.25130441	0.075	0.247	6.1372E-47	Neu
ZNF740	4.0218E-51	0.21119751	0.086	0.264	6.1819E-47	Neu
IFNGR13	4.1524E-51	0.10222268	0.497	0.904	6.3826E-47	Neu
PHTF1	4.3351E-51	0.44897071	0.094	0.278	6.6635E-47	Neu
RAPGEF31	4.4806E-51	0.3157685	0.053	0.21	6.8871E-47	Neu
HDAC72	4.4946E-51	0.10608547	0.064	0.228	6.9087E-47	Neu
GNE	4.7388E-51	0.15367523	0.055	0.214	7.284E-47	Neu
FRA10AC11	4.888E-51	0.2369205	0.099	0.286	7.5133E-47	Neu
ZNF1751	4.8966E-51	0.27789378	0.107	0.299	7.5266E-47	Neu
TMEM14B	4.8983E-51	0.21226853	0.082	0.258	7.5292E-47	Neu
KIAA1841	5.0406E-51	0.49163513	0.101	0.288	7.7479E-47	Neu
LRIF1	5.1473E-51	0.19110708	0.084	0.261	7.912E-47	Neu
FLOT11	5.4685E-51	0.39626532	0.465	0.861	8.4057E-47	Neu
XPOT1	5.7037E-51	0.17720597	0.086	0.265	8.7672E-47	Neu
STX32	5.9714E-51	1.14683768	0.165	0.398	9.1786E-47	Neu
NUDT16L11	6.2039E-51	0.214034	0.054	0.212	9.5361E-47	Neu
RACK12	6.2677E-51	0.15725195	0.447	0.857	9.6341E-47	Neu
SLC8A13	6.4518E-51	0.23963388	0.463	0.86	9.9171E-47	Neu
ZNF524	6.6361E-51	0.26357173	0.078	0.251	1.02E-46	Neu
HEMK11	6.656E-51	0.21780912	0.064	0.227	1.0231E-46	Neu
RNF2172	7.1244E-51	0.59223523	0.124	0.327	1.0951E-46	Neu
ACTR6	8.4118E-51	0.14834978	0.09	0.27	1.293E-46	Neu
ZSCAN321	9.4469E-51	0.17648451	0.094	0.276	1.4521E-46	Neu
ENTPD41	1.1253E-50	0.49735763	0.132	0.34	1.7297E-46	Neu
REV3L	1.1388E-50	0.11216766	0.094	0.277	1.7504E-46	Neu
ZNF5163	1.193E-50	0.62499194	0.096	0.28	1.8338E-46	Neu
CDKL52	1.2269E-50	0.12218809	0.067	0.233	1.8859E-46	Neu
ZNF680	1.2273E-50	0.33502622	0.114	0.311	1.8865E-46	Neu
GMEB11	1.5139E-50	0.2378327	0.07	0.237	2.327E-46	Neu

TATDN32	1.8678E-50	0.22660595	0.094	0.277	2.871E-46	Neu
FASTKD51	1.9154E-50	0.22538797	0.091	0.273	2.9442E-46	Neu
FAM89A1	1.9867E-50	0.1159864	0.493	0.883	3.0538E-46	Neu
TTC13	2.063E-50	0.13754956	0.095	0.278	3.171E-46	Neu
XPO4	2.2922E-50	0.29181218	0.1	0.285	3.5234E-46	Neu
MAP3K202	2.4223E-50	0.18099945	0.066	0.229	3.7233E-46	Neu
ZBTB401	2.5911E-50	0.20607561	0.07	0.235	3.9828E-46	Neu
PRKAG21	2.6212E-50	0.3749771	0.069	0.236	4.0291E-46	Neu
RABEPK1	2.7573E-50	0.23323043	0.076	0.247	4.2382E-46	Neu
HSPBAP11	2.8241E-50	0.41940424	0.087	0.263	4.3409E-46	Neu
FBXL4	2.9734E-50	0.14738147	0.07	0.234	4.5705E-46	Neu
PHF131	3.1696E-50	0.17676631	0.045	0.194	4.872E-46	Neu
ATP10D1	3.2948E-50	0.15199633	0.072	0.24	5.0645E-46	Neu
MICOS10	3.3514E-50	0.2790323	0.427	0.83	5.1515E-46	Neu
ATR	3.3636E-50	0.13559688	0.081	0.253	5.1702E-46	Neu
MTF21	3.3928E-50	0.42151453	0.126	0.329	5.215E-46	Neu
ZNF122	3.5426E-50	0.19355696	0.07	0.236	5.4454E-46	Neu
ME31	3.8841E-50	0.31069677	0.068	0.233	5.9703E-46	Neu
VIPAS39	4.3352E-50	0.24397693	0.072	0.239	6.6637E-46	Neu
UFSP21	4.3371E-50	0.3238269	0.111	0.305	6.6666E-46	Neu
XPR11	4.8446E-50	0.1610049	0.091	0.27	7.4466E-46	Neu
PCGF32	4.859E-50	0.47397412	0.118	0.314	7.4688E-46	Neu
FRRS11	5.0319E-50	0.27887274	0.092	0.271	7.7345E-46	Neu
SYNJ11	5.2221E-50	0.17704171	0.068	0.231	8.0269E-46	Neu
BAZ2B2	5.353E-50	0.66182922	0.261	0.554	8.228E-46	Neu
SLC9A81	5.7056E-50	0.74697622	0.104	0.291	8.7701E-46	Neu
RGMB1	5.798E-50	0.18979904	0.053	0.207	8.9122E-46	Neu
NIPSNAP1	6.1521E-50	0.23907326	0.079	0.251	9.4564E-46	Neu
VNN21	7.7132E-50	0.68193402	0.07	0.235	1.1856E-45	Neu
ALKBH1	8.0391E-50	0.12830738	0.047	0.197	1.2357E-45	Neu
IFNGR23	8.828E-50	0.12883625	0.496	0.894	1.357E-45	Neu
ZNF2241	9.3642E-50	0.1287067	0.083	0.255	1.4394E-45	Neu
BCL2L114	1.0552E-49	0.5077186	0.103	0.288	1.6219E-45	Neu
RPP25L	1.1597E-49	0.11528368	0.05	0.202	1.7825E-45	Neu
PGS12	1.1603E-49	1.20272557	0.141	0.354	1.7835E-45	Neu
TWISTNB1	1.1669E-49	0.11901796	0.071	0.237	1.7936E-45	Neu
STARD101	1.364E-49	0.21985848	0.104	0.291	2.0967E-45	Neu
POLL	1.5262E-49	0.20959508	0.074	0.241	2.3459E-45	Neu
TENT4A	1.5737E-49	0.22701229	0.093	0.272	2.4189E-45	Neu
CSTF11	1.5969E-49	0.39132614	0.098	0.282	2.4545E-45	Neu
KAT6B	1.6451E-49	0.4003196	0.138	0.344	2.5286E-45	Neu
PIGF	1.7574E-49	0.17117837	0.087	0.264	2.7014E-45	Neu
PDS5B1	1.981E-49	0.2137345	0.088	0.265	3.045E-45	Neu
WDR701	2.0032E-49	0.25654027	0.089	0.266	3.0791E-45	Neu
PDIK1L	2.1924E-49	0.27540741	0.074	0.242	3.3699E-45	Neu
TMEM911	2.3168E-49	0.4496956	0.08	0.251	3.5611E-45	Neu
CDC271	2.3539E-49	0.17840969	0.081	0.253	3.6181E-45	Neu
DGKZ1	2.4219E-49	0.13020036	0.092	0.269	3.7227E-45	Neu
ZBTB102	2.6848E-49	0.51833167	0.092	0.271	4.1268E-45	Neu
PAPOLG	2.7404E-49	0.1116516	0.081	0.252	4.2123E-45	Neu
ASB7	2.802E-49	0.13541347	0.066	0.227	4.307E-45	Neu
CEP1921	2.8185E-49	0.35373061	0.112	0.302	4.3324E-45	Neu

TEDC12	2.8304E-49	0.4802347	0.104	0.288	4.3506E-45	Neu
RBM333	2.8313E-49	0.53692531	0.36	0.724	4.352E-45	Neu
DDHD21	3.5007E-49	0.10948648	0.083	0.255	5.3809E-45	Neu
USPL11	3.8867E-49	0.33537731	0.12	0.316	5.9742E-45	Neu
BPGM	4.2638E-49	0.2046105	0.087	0.262	6.554E-45	Neu
ZNF276	4.6672E-49	0.52635881	0.108	0.295	7.1739E-45	Neu
RIF11	4.9765E-49	0.11277247	0.088	0.262	7.6494E-45	Neu
PMS1	5.0246E-49	0.19302312	0.102	0.284	7.7233E-45	Neu
IFNLR11	5.4811E-49	0.11788312	0.059	0.215	8.425E-45	Neu
ING2	5.8365E-49	0.23006282	0.084	0.257	8.9712E-45	Neu
SLC7A6OS1	6.1599E-49	0.1164381	0.066	0.227	9.4683E-45	Neu
GGPS1	7.3019E-49	0.2081387	0.102	0.287	1.1224E-44	Neu
FBXO32	9.4773E-49	0.49952926	0.106	0.293	1.4568E-44	Neu
PNN1	1.0218E-48	0.68751075	0.156	0.375	1.5706E-44	Neu
MTG11	1.0271E-48	0.43067047	0.112	0.3	1.5787E-44	Neu
ZNF5591	1.1301E-48	0.32461714	0.077	0.244	1.737E-44	Neu
DEAF11	1.1609E-48	0.29163359	0.067	0.226	1.7844E-44	Neu
HIPK32	1.1836E-48	0.21620883	0.447	0.858	1.8194E-44	Neu
RAD54L2	1.3366E-48	0.10129167	0.06	0.216	2.0545E-44	Neu
MICAL23	1.4663E-48	0.14514306	0.05	0.199	2.2539E-44	Neu
LIN7A	1.6388E-48	0.35789785	0.106	0.291	2.5191E-44	Neu
MARCH82	1.7189E-48	0.36970242	0.1	0.282	2.6421E-44	Neu
SLC38A92	1.8132E-48	0.20514089	0.071	0.234	2.787E-44	Neu
HIP1	1.947E-48	0.2597479	0.066	0.224	2.9927E-44	Neu
NSD31	2.1626E-48	0.27917384	0.133	0.335	3.3242E-44	Neu
PRPF391	2.3538E-48	0.20193202	0.083	0.252	3.618E-44	Neu
XRCC41	2.4225E-48	0.37838149	0.091	0.269	3.7237E-44	Neu
SENP31	2.4982E-48	0.47952967	0.231	0.494	3.84E-44	Neu
TLR23	2.5026E-48	1.53500861	0.127	0.328	3.8468E-44	Neu
ETV32	2.7583E-48	0.28167017	0.071	0.235	4.2398E-44	Neu
CDK81	2.8056E-48	0.13241211	0.074	0.238	4.3125E-44	Neu
CASP6	3.0432E-48	0.12224619	0.068	0.229	4.6777E-44	Neu
SESN32	3.5176E-48	0.3021828	0.08	0.248	5.4069E-44	Neu
CCDC931	3.7882E-48	0.28486524	0.058	0.21	5.8229E-44	Neu
C18orf21	4.0802E-48	0.25953633	0.089	0.262	6.2716E-44	Neu
FTSJ11	4.3285E-48	0.11760319	0.076	0.243	6.6533E-44	Neu
TRIM38	4.4031E-48	0.32093634	0.441	0.855	6.768E-44	Neu
STARD41	4.4605E-48	0.23401605	0.078	0.243	6.8563E-44	Neu
RSPO31	4.6281E-48	0.19947934	0.1	0.279	7.1139E-44	Neu
TSC22D32	4.8629E-48	0.56918765	0.421	0.81	7.4748E-44	Neu
DGAT21	4.969E-48	0.55911016	0.091	0.267	7.6378E-44	Neu
PTS	5.0121E-48	0.38209523	0.091	0.267	7.7041E-44	Neu
JMJD4	5.1957E-48	0.11235669	0.047	0.192	7.9864E-44	Neu
MYO7B1	5.2516E-48	0.48497194	0.087	0.258	8.0722E-44	Neu
PRMT9	5.7047E-48	0.24664729	0.088	0.26	8.7686E-44	Neu
LYN4	6.6685E-48	0.57962417	0.43	0.831	1.025E-43	Neu
ARHGAP263	7.1636E-48	1.38795386	0.177	0.41	1.1011E-43	Neu
TRUB1	7.5257E-48	0.34165208	0.096	0.275	1.1568E-43	Neu
CPOX2	7.5698E-48	0.30528281	0.07	0.231	1.1636E-43	Neu
PILRB3	7.8684E-48	0.21184008	0.171	0.388	1.2094E-43	Neu
EZH1	8.2107E-48	0.25370137	0.071	0.232	1.2621E-43	Neu
ARAP11	8.3968E-48	0.1685652	0.554	0.937	1.2907E-43	Neu

NFRKB1	9.2637E-48	0.21900061	0.087	0.257	1.4239E-43	Neu
ERMARD1	9.3202E-48	0.2328121	0.086	0.257	1.4326E-43	Neu
ZNF5441	9.8798E-48	0.24332552	0.09	0.262	1.5186E-43	Neu
JMJD1C1	1.0552E-47	0.54388751	0.384	0.765	1.622E-43	Neu
TJAP1	1.1062E-47	0.21221582	0.063	0.219	1.7003E-43	Neu
RANBP10	1.1849E-47	0.29689878	0.069	0.229	1.8214E-43	Neu
LENG83	1.2417E-47	0.38318975	0.334	0.654	1.9087E-43	Neu
PKN21	1.2545E-47	0.45825644	0.367	0.73	1.9283E-43	Neu
ATP6V0A23	1.319E-47	0.32641467	0.11	0.293	2.0274E-43	Neu
ZNF4261	1.3612E-47	0.24081393	0.075	0.24	2.0923E-43	Neu
IQCG1	1.4111E-47	0.16003052	0.087	0.257	2.1691E-43	Neu
RAD51B	1.6211E-47	0.55724276	0.144	0.352	2.4918E-43	Neu
FAM151B1	1.6352E-47	0.23315627	0.085	0.255	2.5135E-43	Neu
DPH51	1.7486E-47	0.10645902	0.062	0.217	2.6878E-43	Neu
COA5	1.8001E-47	0.43148371	0.071	0.232	2.767E-43	Neu
WDR471	1.8405E-47	0.26793562	0.076	0.241	2.829E-43	Neu
C2orf491	1.9602E-47	0.32938516	0.094	0.269	3.0131E-43	Neu
ZNF1891	2.0803E-47	0.25044406	0.082	0.248	3.1976E-43	Neu
ENTPD51	2.3187E-47	0.48199385	0.083	0.253	3.5641E-43	Neu
PPP1R16A	2.3191E-47	0.13481138	0.04	0.179	3.5647E-43	Neu
FAM193B1	2.4283E-47	0.50002941	0.131	0.328	3.7326E-43	Neu
RAD51D	2.4465E-47	0.14610462	0.053	0.201	3.7605E-43	Neu
ZNF652	2.4784E-47	0.26531254	0.108	0.292	3.8095E-43	Neu
TTC331	2.4987E-47	0.16414364	0.074	0.235	3.8407E-43	Neu
KDM6A	2.773E-47	0.64092516	0.129	0.327	4.2623E-43	Neu
ZNF3371	2.8388E-47	0.21534989	0.095	0.271	4.3635E-43	Neu
MAP2K4	2.8843E-47	0.47450721	0.103	0.283	4.4334E-43	Neu
PHF21A2	3.0749E-47	0.63528963	0.104	0.285	4.7265E-43	Neu
GRK32	3.2809E-47	0.26812226	0.458	0.868	5.043E-43	Neu
GMDS1	3.4758E-47	0.18325899	0.086	0.255	5.3426E-43	Neu
RAB11FIP12	3.5746E-47	0.12610964	0.521	0.912	5.4945E-43	Neu
RAD11	3.595E-47	0.28998561	0.082	0.249	5.5259E-43	Neu
TMTC21	3.8416E-47	0.53853797	0.092	0.266	5.9049E-43	Neu
BLNK4	4.5173E-47	0.10829551	0.035	0.17	6.9436E-43	Neu
HMBOX11	4.5489E-47	0.26547589	0.104	0.283	6.992E-43	Neu
NOC3L1	4.5621E-47	0.15303299	0.076	0.238	7.0124E-43	Neu
PARG	4.7028E-47	0.24958635	0.076	0.24	7.2287E-43	Neu
ACOT81	4.8891E-47	0.1200369	0.064	0.219	7.5151E-43	Neu
LOXL32	5.169E-47	0.11746177	0.066	0.221	7.9452E-43	Neu
METTL2A1	5.1839E-47	0.17832962	0.075	0.239	7.9681E-43	Neu
ZNF33A3	5.208E-47	0.11127212	0.101	0.277	8.0053E-43	Neu
DNAJC25	5.307E-47	0.19739704	0.064	0.22	8.1575E-43	Neu
RBAK	5.4617E-47	0.21174697	0.071	0.231	8.3951E-43	Neu
SLC9A73	5.7476E-47	0.30218617	0.065	0.22	8.8346E-43	Neu
ZFAND2A1	5.8781E-47	0.42229642	0.09	0.262	9.0353E-43	Neu
SPIRE21	6.1046E-47	0.48713411	0.143	0.347	9.3834E-43	Neu
PER32	6.1127E-47	0.10709846	0.09	0.26	9.3959E-43	Neu
AQP91	6.5444E-47	0.83189919	0.308	0.593	1.0059E-42	Neu
PRPF181	6.5533E-47	0.3249069	0.102	0.282	1.0073E-42	Neu
NDUFAF7	6.6371E-47	0.12268848	0.066	0.221	1.0202E-42	Neu
UGGT21	6.6944E-47	0.26962785	0.087	0.257	1.029E-42	Neu
FAM214A1	6.9731E-47	0.19100019	0.112	0.295	1.0718E-42	Neu

MMAA	7.8184E-47	0.16275787	0.062	0.215	1.2018E-42	Neu
ZUP1	8.1717E-47	0.31716578	0.08	0.244	1.2561E-42	Neu
SLC25A17	8.5819E-47	0.19396092	0.07	0.227	1.3191E-42	Neu
ARAP2	8.8628E-47	0.199609	0.067	0.222	1.3623E-42	Neu
RALGAPA21	8.9252E-47	0.18498851	0.069	0.226	1.3719E-42	Neu
PLEKHB11	9.5423E-47	0.2777435	0.051	0.197	1.4668E-42	Neu
MOB3B2	9.5812E-47	0.2567239	0.436	0.821	1.4727E-42	Neu
KIN	9.824E-47	0.21368398	0.071	0.23	1.51E-42	Neu
ZNF22	9.9604E-47	0.10732761	0.073	0.233	1.531E-42	Neu
CLMN1	1.04E-46	0.36619836	0.076	0.237	1.5985E-42	Neu
SMPD2	1.084E-46	0.28538695	0.063	0.217	1.6662E-42	Neu
RUBCNL1	1.0897E-46	0.80121838	0.07	0.228	1.675E-42	Neu
PKNOX11	1.1637E-46	0.18839175	0.075	0.235	1.7887E-42	Neu
MBTD1	1.2505E-46	0.525894	0.109	0.291	1.9222E-42	Neu
ENTPD71	1.2993E-46	0.23854095	0.06	0.212	1.9972E-42	Neu
NEK63	1.3888E-46	0.11537227	0.531	0.922	2.1347E-42	Neu
TRAM13	1.406E-46	0.12509004	0.487	0.895	2.1612E-42	Neu
REXO11	1.4517E-46	0.13776617	0.06	0.212	2.2314E-42	Neu
TBC1D31	1.457E-46	0.32611185	0.065	0.22	2.2395E-42	Neu
LPAR63	1.4667E-46	0.13461875	0.084	0.249	2.2545E-42	Neu
MCM91	1.612E-46	0.10776619	0.075	0.234	2.4778E-42	Neu
CFP3	1.8242E-46	0.32013022	0.102	0.276	2.804E-42	Neu
TSEN21	2.1295E-46	0.4044636	0.131	0.326	3.2732E-42	Neu
IL1A1	2.1554E-46	0.74005923	0.126	0.318	3.313E-42	Neu
ATG2B	2.1666E-46	0.16056108	0.076	0.236	3.3302E-42	Neu
LGALSL1	2.2053E-46	0.43654557	0.032	0.164	3.3898E-42	Neu
LST12	2.3186E-46	0.13197957	0.509	0.89	3.5639E-42	Neu
PARP151	2.7702E-46	0.32204588	0.098	0.271	4.258E-42	Neu
NRBP2	2.8702E-46	0.17194188	0.052	0.197	4.4118E-42	Neu
CERK2	2.892E-46	0.28732841	0.069	0.225	4.4453E-42	Neu
ENOX2	2.9642E-46	0.17418837	0.096	0.27	4.5562E-42	Neu
LYRM1	3.195E-46	0.28739207	0.122	0.312	4.9111E-42	Neu
ASB61	3.8259E-46	0.14834936	0.055	0.201	5.8808E-42	Neu
ZNF75A	3.9371E-46	0.22790155	0.074	0.231	6.0517E-42	Neu
UTP4	4.0252E-46	0.17177209	0.066	0.22	6.1871E-42	Neu
BCLAF31	4.1163E-46	0.61432579	0.088	0.256	6.3271E-42	Neu
CSE1L	4.6421E-46	0.24062022	0.066	0.22	7.1353E-42	Neu
UMPS1	5.1693E-46	0.14789322	0.074	0.233	7.9457E-42	Neu
PLA2G6	5.1953E-46	0.45246507	0.071	0.228	7.9857E-42	Neu
CHD6	5.31E-46	0.24844688	0.097	0.27	8.162E-42	Neu
ZCCHC8	5.5764E-46	0.45851517	0.096	0.269	8.5715E-42	Neu
PPP1R3B2	6.1013E-46	0.4889672	0.05	0.193	9.3783E-42	Neu
POLA2	6.3501E-46	0.33129368	0.064	0.217	9.7607E-42	Neu
ZSCAN181	6.6232E-46	0.3308369	0.058	0.205	1.018E-41	Neu
ING31	6.7207E-46	0.23766225	0.08	0.242	1.033E-41	Neu
TAF21	6.7324E-46	0.61140281	0.111	0.294	1.0348E-41	Neu
ZDHHC21	6.7385E-46	0.45499439	0.101	0.276	1.0358E-41	Neu
SP140L1	7.3109E-46	0.31621969	0.094	0.265	1.1238E-41	Neu
YEATS41	7.3895E-46	0.1436603	0.07	0.226	1.1358E-41	Neu
TIRAP	7.6054E-46	0.10185381	0.055	0.201	1.169E-41	Neu
PLK33	7.7233E-46	1.77238261	0.118	0.309	1.1871E-41	Neu
BAG4	7.7326E-46	0.57082374	0.103	0.282	1.1886E-41	Neu

ENTR12	8.0844E-46	0.1444095	0.08	0.242	1.2427E-41	Neu
SNAPC3	8.4588E-46	0.14185761	0.066	0.219	1.3002E-41	Neu
ODF3L11	9.4742E-46	0.22762004	0.068	0.223	1.4563E-41	Neu
CHD22	9.7145E-46	0.61371155	0.274	0.559	1.4932E-41	Neu
VKORC1L11	1.054E-45	0.36624887	0.072	0.23	1.6201E-41	Neu
GSAP1	1.0989E-45	0.46720956	0.378	0.749	1.6892E-41	Neu
ZFAND1	1.1119E-45	0.43190184	0.139	0.339	1.709E-41	Neu
FILIP1L3	1.1756E-45	0.21898931	0.083	0.246	1.807E-41	Neu
ZNF5121	1.2819E-45	0.3081412	0.089	0.258	1.9703E-41	Neu
TBL1X1	1.3825E-45	0.60537333	0.12	0.309	2.125E-41	Neu
CASP51	1.4428E-45	0.31757332	0.064	0.217	2.2177E-41	Neu
ATG141	1.6271E-45	0.35256308	0.092	0.261	2.501E-41	Neu
MRGBP1	1.6716E-45	0.29219678	0.076	0.236	2.5694E-41	Neu
CDC42SE12	1.7733E-45	0.33648712	0.476	0.884	2.7258E-41	Neu
TMOD22	1.8519E-45	0.29474044	0.056	0.203	2.8466E-41	Neu
DHX33	1.8557E-45	0.3771013	0.055	0.201	2.8524E-41	Neu
SLC23A22	2.2994E-45	0.15725591	0.063	0.212	3.5344E-41	Neu
LAMB11	2.454E-45	0.46016608	0.083	0.244	3.7721E-41	Neu
APAF14	2.4677E-45	0.46003676	0.113	0.293	3.7932E-41	Neu
BTBD91	2.6409E-45	0.42084107	0.118	0.302	4.0594E-41	Neu
FAM219B	2.7832E-45	0.51282369	0.151	0.358	4.2781E-41	Neu
PEX11A1	3.2103E-45	0.31868262	0.076	0.234	4.9346E-41	Neu
TNKS	3.4596E-45	0.45606593	0.099	0.273	5.3177E-41	Neu
KIFC32	3.6946E-45	0.7366385	0.048	0.188	5.679E-41	Neu
ARID3A1	3.7448E-45	0.19642016	0.056	0.2	5.7562E-41	Neu
ZNF207	3.9501E-45	0.17671058	0.094	0.263	6.0717E-41	Neu
LRBA2	3.9517E-45	0.10891686	0.089	0.255	6.0742E-41	Neu
QPRT1	4.0882E-45	0.2298023	0.073	0.23	6.284E-41	Neu
C22orf391	4.2004E-45	0.15432171	0.061	0.21	6.4565E-41	Neu
RCOR31	4.2922E-45	0.30217597	0.099	0.271	6.5975E-41	Neu
ZNF557	4.4657E-45	0.13025259	0.064	0.214	6.8642E-41	Neu
CRACR2B1	4.5479E-45	0.46164623	0.094	0.264	6.9906E-41	Neu
CEP170B1	4.7431E-45	0.13770603	0.037	0.169	7.2906E-41	Neu
FAS2	4.8006E-45	0.4569706	0.059	0.207	7.3789E-41	Neu
CSF2RA3	5.0686E-45	0.21897916	0.463	0.88	7.791E-41	Neu
SMIM10L1	6.0562E-45	0.11826522	0.065	0.216	9.309E-41	Neu
ATP5F1E	6.332E-45	0.11665344	0.501	0.897	9.733E-41	Neu
DHFR	6.7846E-45	0.56741748	0.121	0.307	1.0429E-40	Neu
POU2F1	7.1431E-45	0.5697744	0.1	0.275	1.098E-40	Neu
ZNF4441	7.2556E-45	0.26837388	0.051	0.193	1.1153E-40	Neu
GIMAP82	8.0247E-45	0.10926131	0.068	0.219	1.2335E-40	Neu
POMGNT2	1.0273E-44	0.20624792	0.044	0.181	1.5791E-40	Neu
ZNF2671	1.0394E-44	1.08860642	0.078	0.238	1.5977E-40	Neu
IMMP2L1	1.1144E-44	0.47880646	0.079	0.238	1.713E-40	Neu
CASZ1	1.24E-44	0.16068149	0.058	0.204	1.9061E-40	Neu
TBC1D8	1.365E-44	0.2118687	0.057	0.201	2.0982E-40	Neu
PAXBP11	1.3721E-44	0.3788923	0.106	0.282	2.1091E-40	Neu
MAPKBP11	1.3724E-44	0.1174647	0.06	0.206	2.1096E-40	Neu
PAQR51	1.38E-44	0.41403792	0.305	0.609	2.1212E-40	Neu
PPP1R262	1.397E-44	0.31418676	0.047	0.185	2.1473E-40	Neu
PANK2	1.4217E-44	0.20016718	0.049	0.188	2.1852E-40	Neu
ZNF4071	1.5172E-44	0.13475475	0.101	0.272	2.332E-40	Neu

TRMT2B	1.8091E-44	0.30206694	0.1	0.272	2.7808E-40	Neu
CAPN31	2.0041E-44	0.21575881	0.11	0.283	3.0805E-40	Neu
PCBP2	2.2128E-44	0.51191044	0.136	0.334	3.4014E-40	Neu
POLM	2.2607E-44	0.29157159	0.073	0.227	3.4748E-40	Neu
FBXL201	2.5495E-44	0.44063374	0.091	0.258	3.9188E-40	Neu
ZNF4901	2.7486E-44	0.43642173	0.073	0.228	4.2249E-40	Neu
SBDS1	2.8813E-44	0.14199887	0.067	0.217	4.4288E-40	Neu
CCNL23	3.0018E-44	0.65355401	0.239	0.498	4.6141E-40	Neu
CDC37L1	3.0602E-44	0.11978421	0.061	0.208	4.7039E-40	Neu
DHX35	3.3042E-44	0.14724205	0.067	0.217	5.0789E-40	Neu
RIMKLB1	3.3263E-44	0.26774742	0.058	0.201	5.1129E-40	Neu
PYROXD1	3.4193E-44	0.46491792	0.091	0.257	5.2558E-40	Neu
THADA1	3.8652E-44	0.27472896	0.123	0.307	5.9412E-40	Neu
BACH12	4.598E-44	0.36367704	0.48	0.884	7.0676E-40	Neu
MED21	5.4545E-44	0.2687354	0.083	0.244	8.384E-40	Neu
NET11	5.5458E-44	0.15018131	0.133	0.323	8.5244E-40	Neu
KCTD18	6.2988E-44	0.21743163	0.066	0.214	9.6819E-40	Neu
MS4A142	6.4781E-44	0.45477715	0.207	0.44	9.9574E-40	Neu
BEST12	6.8107E-44	1.25251913	0.14	0.336	1.0469E-39	Neu
HEXD2	6.8389E-44	0.35987971	0.095	0.261	1.0512E-39	Neu
TSC11	7.1448E-44	0.24799228	0.061	0.206	1.0982E-39	Neu
RASSF71	7.3498E-44	0.27151947	0.066	0.215	1.1297E-39	Neu
ESS21	7.4816E-44	0.30544038	0.062	0.208	1.15E-39	Neu
GLT8D1	8.783E-44	0.29998286	0.072	0.226	1.35E-39	Neu
ZFP364	8.8716E-44	0.77890048	0.481	0.875	1.3637E-39	Neu
LPAR2	8.9008E-44	0.31883316	0.078	0.233	1.3681E-39	Neu
ARMCX12	8.9107E-44	0.12586413	0.051	0.19	1.3697E-39	Neu
TFDP2	8.9526E-44	0.19728972	0.07	0.222	1.3761E-39	Neu
NKTR3	9.3296E-44	0.55237326	0.163	0.37	1.4341E-39	Neu
TMEM184C1	9.4937E-44	0.27640112	0.106	0.281	1.4593E-39	Neu
NCF41	9.7257E-44	0.23829433	0.504	0.899	1.4949E-39	Neu
ZNF83	9.9695E-44	0.3937496	0.088	0.251	1.5324E-39	Neu
CTPS11	1.0725E-43	0.16719548	0.051	0.189	1.6485E-39	Neu
ZBTB341	1.1095E-43	0.37357712	0.071	0.224	1.7054E-39	Neu
DISC12	1.2593E-43	0.40480664	0.08	0.236	1.9357E-39	Neu
FSD1L1	1.3176E-43	0.59547665	0.105	0.277	2.0253E-39	Neu
PARP41	1.3625E-43	0.15911589	0.493	0.897	2.0943E-39	Neu
UTP111	1.3848E-43	0.27937374	0.073	0.226	2.1285E-39	Neu
IKZF2	1.417E-43	0.6905828	0.093	0.259	2.1781E-39	Neu
AGO3	1.4764E-43	0.46947319	0.101	0.271	2.2694E-39	Neu
LRR11	1.8666E-43	0.13673711	0.047	0.183	2.8691E-39	Neu
PHLPP2	1.8714E-43	0.10507824	0.046	0.181	2.8765E-39	Neu
SEMA6B2	1.8882E-43	0.71892116	0.057	0.199	2.9023E-39	Neu
VEGFA3	2.0449E-43	1.36986248	0.099	0.265	3.1433E-39	Neu
ZFP301	2.5167E-43	0.14682194	0.058	0.2	3.8684E-39	Neu
UQCC3	2.591E-43	0.10605138	0.055	0.196	3.9827E-39	Neu
RAB314	2.6365E-43	0.13438651	0.495	0.891	4.0526E-39	Neu
KLHL54	2.7791E-43	0.64366515	0.072	0.223	4.2718E-39	Neu
METTL12	2.7951E-43	0.24471549	0.047	0.182	4.2964E-39	Neu
SLC10A71	2.8284E-43	0.35600207	0.075	0.229	4.3476E-39	Neu
FBXO45	3.032E-43	0.27823767	0.059	0.202	4.6604E-39	Neu
SGTB3	3.0706E-43	0.47817862	0.063	0.208	4.7198E-39	Neu

NHLRC11	3.0853E-43	0.12406411	0.041	0.171	4.7424E-39	Neu
BRWD32	3.0863E-43	0.43851591	0.084	0.243	4.744E-39	Neu
CTIF2	3.1158E-43	0.44489617	0.069	0.219	4.7893E-39	Neu
ZNF252	3.2041E-43	0.25045039	0.1	0.271	4.925E-39	Neu
CRYBG31	3.4594E-43	0.25960416	0.07	0.219	5.3175E-39	Neu
USP6NL1	3.5865E-43	0.64926393	0.122	0.304	5.5128E-39	Neu
TERF11	3.6606E-43	0.14049847	0.079	0.234	5.6268E-39	Neu
ARHGAP211	3.7579E-43	0.21385252	0.062	0.207	5.7762E-39	Neu
ZNF71	3.9326E-43	0.30974058	0.072	0.222	6.0448E-39	Neu
USP211	3.9386E-43	0.1431744	0.063	0.208	6.054E-39	Neu
MITD12	4.2276E-43	0.32141867	0.079	0.234	6.4983E-39	Neu
METTL21A2	4.2287E-43	0.21017701	0.086	0.246	6.4999E-39	Neu
FRS21	4.3942E-43	0.10855524	0.076	0.229	6.7543E-39	Neu
DYNLL11	4.4446E-43	0.44674328	0.071	0.223	6.8318E-39	Neu
DENND5A1	4.7796E-43	0.24376724	0.484	0.889	7.3468E-39	Neu
RNASEH2B	5.9449E-43	0.12582522	0.058	0.2	9.1378E-39	Neu
ALDH6A1	5.997E-43	0.26509921	0.057	0.199	9.2179E-39	Neu
SLC19A21	6.1524E-43	0.41063309	0.087	0.247	9.4568E-39	Neu
CMSS1	6.509E-43	0.28984278	0.049	0.185	1.0005E-38	Neu
PRSS362	6.7748E-43	0.35802542	0.042	0.173	1.0414E-38	Neu
SREK1IP1	6.9818E-43	0.59036463	0.097	0.265	1.0732E-38	Neu
MKKS	7.8393E-43	0.45327236	0.094	0.259	1.205E-38	Neu
BCL2A11	8.3364E-43	0.51927832	0.495	0.867	1.2814E-38	Neu
RAD171	9.0072E-43	0.1769886	0.062	0.206	1.3845E-38	Neu
ZDHHC174	9.1635E-43	0.58634839	0.104	0.275	1.4085E-38	Neu
METTL221	9.3488E-43	0.27186983	0.07	0.218	1.437E-38	Neu
USP25	9.3771E-43	0.28364692	0.461	0.866	1.4414E-38	Neu
CENPT1	9.7242E-43	0.3319366	0.083	0.239	1.4947E-38	Neu
SLC48A1	1.0672E-42	0.32575755	0.039	0.168	1.6404E-38	Neu
OGA3	1.1331E-42	0.59565293	0.392	0.757	1.7416E-38	Neu
TEX101	1.171E-42	0.21627836	0.068	0.216	1.7999E-38	Neu
CLCN61	1.2415E-42	0.28502329	0.071	0.218	1.9083E-38	Neu
IARS1	1.3147E-42	0.18245909	0.054	0.193	2.0209E-38	Neu
ALKBH31	1.3441E-42	0.54648284	0.078	0.233	2.0661E-38	Neu
NABP12	1.3546E-42	1.09996884	0.329	0.653	2.0821E-38	Neu
SNX1	1.3671E-42	0.1547212	0.523	0.923	2.1014E-38	Neu
JAG12	1.3994E-42	0.10451001	0.068	0.215	2.1511E-38	Neu
EIF2B2	1.4103E-42	0.14662339	0.055	0.193	2.1678E-38	Neu
GFOD21	1.5922E-42	0.10228243	0.047	0.181	2.4473E-38	Neu
RBM26	1.7317E-42	0.68887074	0.147	0.344	2.6618E-38	Neu
SNX33	1.8464E-42	0.44867441	0.05	0.185	2.8381E-38	Neu
DCAKD	2.0983E-42	0.44360074	0.062	0.204	3.2253E-38	Neu
FAM83H1	2.1017E-42	0.33140723	0.039	0.167	3.2305E-38	Neu
EPHB61	2.3506E-42	0.2576772	0.069	0.217	3.6131E-38	Neu
MYNN1	2.4431E-42	0.45849391	0.105	0.276	3.7554E-38	Neu
MCEE	2.5659E-42	0.20225314	0.058	0.199	3.9441E-38	Neu
DISP11	2.7284E-42	0.17279681	0.052	0.188	4.1938E-38	Neu
LEMD3	3.2983E-42	0.22914681	0.071	0.219	5.0697E-38	Neu
MTERF31	4.6629E-42	0.30480025	0.083	0.239	7.1674E-38	Neu
TOGARAM11	4.8738E-42	0.38598904	0.063	0.207	7.4915E-38	Neu
GEN1	5.1439E-42	0.29141569	0.068	0.214	7.9066E-38	Neu
MARS1	5.2221E-42	0.17380179	0.038	0.164	8.0268E-38	Neu

AHI11	5.6837E-42	0.28150709	0.074	0.221	8.7365E-38	Neu
MYO191	5.733E-42	0.22378953	0.088	0.245	8.8122E-38	Neu
NEU31	5.8434E-42	0.42131131	0.051	0.185	8.9819E-38	Neu
GTF3C4	5.8542E-42	0.20180628	0.043	0.172	8.9985E-38	Neu
LSM12	6.9321E-42	0.20755142	0.053	0.189	1.0655E-37	Neu
TET23	7.0296E-42	0.64436678	0.124	0.305	1.0805E-37	Neu
CWC25	7.2651E-42	1.03508366	0.082	0.237	1.1167E-37	Neu
GPATCH21	7.2866E-42	0.39321729	0.115	0.289	1.12E-37	Neu
WDR73	7.5384E-42	0.34701451	0.088	0.246	1.1587E-37	Neu
DUSP18	8.3634E-42	0.25915144	0.068	0.213	1.2855E-37	Neu
RRNAD12	1.0049E-41	0.4359575	0.058	0.197	1.5447E-37	Neu
IQCB1	1.022E-41	0.37419095	0.089	0.248	1.571E-37	Neu
FHL11	1.0264E-41	0.12014032	0.071	0.216	1.5777E-37	Neu
ZNF783	1.2175E-41	0.19507583	0.083	0.234	1.8715E-37	Neu
PFDN5	1.4387E-41	0.16688376	0.473	0.873	2.2115E-37	Neu
CNOT3	1.48E-41	0.31451256	0.06	0.2	2.2749E-37	Neu
TNFAIP21	1.6115E-41	0.66766485	0.465	0.85	2.477E-37	Neu
BRWD1	1.6156E-41	0.41876877	0.061	0.201	2.4833E-37	Neu
CHKB1	1.6485E-41	0.88085023	0.122	0.3	2.534E-37	Neu
SOD22	1.6638E-41	1.63605736	0.441	0.812	2.5574E-37	Neu
ANKRD13C1	1.9106E-41	0.34391253	0.048	0.18	2.9368E-37	Neu
NME21	2.3486E-41	0.11316891	0.49	0.876	3.61E-37	Neu
MPHOSPH9	2.8996E-41	0.17643411	0.094	0.254	4.4569E-37	Neu
CCDC611	2.9899E-41	0.38097749	0.044	0.173	4.5958E-37	Neu
SEPSECS1	3.0437E-41	0.20009892	0.073	0.22	4.6784E-37	Neu
CDYL21	3.0862E-41	0.13137212	0.058	0.196	4.7439E-37	Neu
TP53INP11	3.2944E-41	0.21114343	0.05	0.181	5.0638E-37	Neu
DHTKD11	3.3428E-41	0.29638046	0.067	0.211	5.1382E-37	Neu
ETAA11	4.0141E-41	0.30362932	0.062	0.202	6.17E-37	Neu
RTEL11	4.1657E-41	0.11895759	0.05	0.181	6.4031E-37	Neu
TNS12	4.3573E-41	0.1090294	0.03	0.147	6.6976E-37	Neu
TOMM40L1	4.4431E-41	0.41108103	0.059	0.197	6.8294E-37	Neu
RUFY21	4.5397E-41	0.4108669	0.098	0.261	6.978E-37	Neu
RWDD31	4.5906E-41	0.60442335	0.082	0.235	7.0563E-37	Neu
RNF132	4.7183E-41	0.13781087	0.504	0.89	7.2526E-37	Neu
NADK2	4.7945E-41	0.3361042	0.072	0.218	7.3696E-37	Neu
C1GALT11	4.8644E-41	0.10782393	0.075	0.223	7.4771E-37	Neu
EPM2AIP12	4.9272E-41	0.28271549	0.063	0.203	7.5737E-37	Neu
GUF11	5.0059E-41	0.14115278	0.062	0.202	7.6946E-37	Neu
PLEKHM31	5.035E-41	0.50951753	0.076	0.224	7.7393E-37	Neu
TTC142	5.053E-41	0.57962606	0.167	0.367	7.7669E-37	Neu
ANKRD49	5.4788E-41	0.16517533	0.054	0.189	8.4214E-37	Neu
KYAT31	5.7293E-41	0.4004298	0.077	0.227	8.8064E-37	Neu
PLA2G12A	6.5466E-41	0.44234305	0.075	0.224	1.0063E-36	Neu
SLC66A11	7.2271E-41	0.17445981	0.042	0.168	1.1109E-36	Neu
PEAK13	7.814E-41	0.10375165	0.072	0.218	1.2011E-36	Neu
ERMAP	7.8476E-41	0.5051976	0.098	0.258	1.2063E-36	Neu
ZNF5521	7.8792E-41	0.51666612	0.081	0.232	1.2111E-36	Neu
FASTKD12	7.914E-41	0.47505146	0.075	0.222	1.2165E-36	Neu
EED	8.5228E-41	0.31679819	0.064	0.205	1.31E-36	Neu
DOP1A1	9.4813E-41	0.22714245	0.09	0.247	1.4574E-36	Neu
CRTAM	9.7765E-41	0.36603639	0.075	0.222	1.5027E-36	Neu

TMEM2362	9.9206E-41	0.13021138	0.058	0.195	1.5249E-36	Neu
PUS3	9.9602E-41	0.11615269	0.059	0.196	1.531E-36	Neu
METTL161	1.1337E-40	0.3649612	0.071	0.215	1.7426E-36	Neu
HYI1	1.2507E-40	0.11026963	0.051	0.182	1.9225E-36	Neu
PLIN5	1.519E-40	6.80614923	0.013	0.001	2.3349E-36	Neu
USP281	1.5264E-40	0.15398335	0.053	0.186	2.3462E-36	Neu
DCLRE1B	1.5673E-40	0.11700278	0.054	0.187	2.409E-36	Neu
POLE	1.6567E-40	0.12905569	0.053	0.186	2.5464E-36	Neu
CCDC9B1	1.8367E-40	0.49953903	0.047	0.175	2.8231E-36	Neu
SERGEF1	1.8996E-40	0.38331056	0.091	0.248	2.9198E-36	Neu
AP4B11	1.9128E-40	0.22672366	0.074	0.22	2.9402E-36	Neu
HEATR5B	2.0036E-40	0.41408727	0.412	0.794	3.0797E-36	Neu
NRF1	2.3644E-40	0.18853409	0.051	0.182	3.6343E-36	Neu
CRAT	2.7735E-40	0.15517877	0.041	0.165	4.2631E-36	Neu
MLF11	2.9631E-40	0.32605918	0.064	0.204	4.5546E-36	Neu
ZNF6711	3.1238E-40	0.10473838	0.074	0.219	4.8016E-36	Neu
METTL61	3.2852E-40	0.22626453	0.052	0.184	5.0497E-36	Neu
RHOBTB1	3.4802E-40	0.47548901	0.056	0.19	5.3495E-36	Neu
N4BP2L21	3.5196E-40	0.47916994	0.425	0.813	5.41E-36	Neu
POLI	4.2283E-40	0.13900955	0.083	0.233	6.4993E-36	Neu
TRIM131	4.7638E-40	0.14752797	0.073	0.216	7.3224E-36	Neu
PPP2R3B1	6.1688E-40	0.16282245	0.058	0.19	9.482E-36	Neu
TRPC61	6.4692E-40	0.46481687	0.235	0.479	9.9438E-36	Neu
CCNL11	7.2831E-40	0.98513189	0.273	0.543	1.1195E-35	Neu
GLT1D12	8.0005E-40	0.94208444	0.078	0.225	1.2298E-35	Neu
MAP3K83	8.3886E-40	0.40299457	0.422	0.782	1.2894E-35	Neu
KHDC41	8.4341E-40	0.21425227	0.082	0.231	1.2964E-35	Neu
MBTPS2	8.7426E-40	0.21664699	0.05	0.178	1.3438E-35	Neu
MAP2K51	8.969E-40	0.2158345	0.074	0.218	1.3786E-35	Neu
SMG6	9.1224E-40	0.1664976	0.055	0.187	1.4022E-35	Neu
TRMU	9.5166E-40	0.25799412	0.081	0.228	1.4628E-35	Neu
KANSL1L1	1.127E-39	0.53339045	0.108	0.273	1.7323E-35	Neu
MTX31	1.1298E-39	0.16484487	0.054	0.185	1.7366E-35	Neu
RCCD11	1.1438E-39	0.34647936	0.056	0.188	1.7581E-35	Neu
FGFR1OP	1.1923E-39	0.18310725	0.056	0.188	1.8327E-35	Neu
TTLL42	1.2617E-39	0.62516451	0.063	0.201	1.9393E-35	Neu
PHACTR11	1.2617E-39	0.67064317	0.454	0.831	1.9394E-35	Neu
TIFA1	1.3232E-39	0.11617393	0.039	0.161	2.0338E-35	Neu
VAV2	1.3248E-39	0.14316891	0.06	0.197	2.0364E-35	Neu
ZNF3501	1.345E-39	0.22983537	0.056	0.189	2.0673E-35	Neu
INAFM1	1.4808E-39	0.34671228	0.062	0.197	2.2762E-35	Neu
SHPRH1	1.6029E-39	0.22554821	0.092	0.246	2.4638E-35	Neu
ZNF2361	1.6163E-39	0.27912838	0.072	0.214	2.4843E-35	Neu
SMCHD12	1.6452E-39	1.00261326	0.239	0.493	2.5289E-35	Neu
RSL24D1	1.6838E-39	0.34468652	0.096	0.254	2.5881E-35	Neu
ZNF3981	1.6898E-39	0.33898787	0.078	0.224	2.5974E-35	Neu
RFX7	1.8019E-39	0.11574906	0.062	0.197	2.7698E-35	Neu
ZNF292	1.8055E-39	0.78593498	0.073	0.216	2.7753E-35	Neu
PFKFB41	1.9069E-39	0.39079459	0.054	0.185	2.931E-35	Neu
TCP11L22	2.0315E-39	0.10823842	0.064	0.201	3.1226E-35	Neu
TMEM42	2.0721E-39	0.3346848	0.063	0.2	3.185E-35	Neu
UVSSA1	2.101E-39	0.44479538	0.075	0.217	3.2294E-35	Neu

PAPSS22	2.3703E-39	0.32112719	0.043	0.166	3.6434E-35	Neu
KLF81	2.4769E-39	0.37297469	0.08	0.227	3.8072E-35	Neu
RCE1	2.5887E-39	0.1237066	0.046	0.171	3.9792E-35	Neu
ABCC51	2.9849E-39	0.47917523	0.075	0.217	4.5881E-35	Neu
CCDC143	3.1803E-39	0.53593485	0.087	0.237	4.8885E-35	Neu
RAB241	3.3195E-39	0.81106777	0.101	0.26	5.1024E-35	Neu
TMEM216	3.355E-39	0.2340093	0.056	0.188	5.1569E-35	Neu
SPRTN	3.3618E-39	0.239374	0.057	0.189	5.1675E-35	Neu
IFI27L1	3.5151E-39	0.24748878	0.048	0.175	5.403E-35	Neu
C9orf85	3.6561E-39	0.24469272	0.071	0.212	5.6198E-35	Neu
WDR492	3.9822E-39	0.46118154	0.11	0.273	6.121E-35	Neu
EPN21	4.0537E-39	0.57035998	0.08	0.226	6.2309E-35	Neu
B4GALT4	5.4437E-39	0.14423893	0.043	0.165	8.3675E-35	Neu
DNAJC16	5.6257E-39	0.32685104	0.047	0.172	8.6473E-35	Neu
ZBTB371	5.8014E-39	0.46241821	0.085	0.234	8.9173E-35	Neu
TMA16	5.9377E-39	0.10999959	0.046	0.171	9.1268E-35	Neu
COX101	5.9858E-39	0.31891244	0.075	0.218	9.2007E-35	Neu
HELQ1	6.6082E-39	0.2219729	0.083	0.23	1.0157E-34	Neu
GCLM	6.6653E-39	0.28944198	0.056	0.187	1.0245E-34	Neu
DOCK21	6.8889E-39	0.18502713	0.508	0.915	1.0589E-34	Neu
TRIM361	7.0797E-39	0.13144004	0.072	0.214	1.0882E-34	Neu
ZSCAN261	7.87E-39	0.15328363	0.051	0.178	1.2097E-34	Neu
TBPL11	7.9708E-39	0.21459137	0.067	0.205	1.2252E-34	Neu
APOO1	8.5693E-39	0.21815271	0.047	0.172	1.3172E-34	Neu
MCAT	8.9798E-39	0.21138043	0.06	0.192	1.3803E-34	Neu
ELAC11	1.0033E-38	0.1041953	0.047	0.172	1.5422E-34	Neu
TOMM341	1.0163E-38	0.29322016	0.063	0.198	1.5621E-34	Neu
NKRF1	1.1308E-38	0.14806171	0.067	0.204	1.7382E-34	Neu
ZNF3221	1.2372E-38	0.31178439	0.081	0.227	1.9018E-34	Neu
SETD41	1.3248E-38	0.19835667	0.062	0.196	2.0364E-34	Neu
MTFR2	1.4151E-38	0.17815713	0.041	0.161	2.1751E-34	Neu
ICAM33	1.6212E-38	0.50791906	0.069	0.206	2.492E-34	Neu
RPA3	1.9399E-38	0.18169601	0.052	0.179	2.9818E-34	Neu
TP53I13	1.9464E-38	0.18408603	0.058	0.188	2.9918E-34	Neu
ATXN7L1	1.9771E-38	0.35415181	0.095	0.249	3.039E-34	Neu
IL4R1	2.079E-38	0.52488472	0.063	0.196	3.1957E-34	Neu
C15orf483	2.1304E-38	0.5802054	0.059	0.188	3.2746E-34	Neu
NFKBIA1	2.2157E-38	0.77950334	0.541	0.916	3.4057E-34	Neu
NEDD91	2.2879E-38	0.41612275	0.078	0.22	3.5168E-34	Neu
CAMKK11	2.3114E-38	0.63144163	0.068	0.205	3.5528E-34	Neu
LIAS1	2.3365E-38	0.4764033	0.068	0.205	3.5914E-34	Neu
TTC23	2.3845E-38	0.17060515	0.063	0.197	3.6652E-34	Neu
B3GALNT11	2.4141E-38	0.26459238	0.047	0.172	3.7107E-34	Neu
GTPBP10	2.4898E-38	0.60998532	0.106	0.266	3.827E-34	Neu
SNAPC11	2.6071E-38	0.69737554	0.072	0.212	4.0073E-34	Neu
ABCA2	2.6859E-38	0.31105654	0.068	0.205	4.1286E-34	Neu
USF31	2.6876E-38	0.30891282	0.069	0.206	4.131E-34	Neu
GABARAP2	2.8562E-38	0.14100093	0.514	0.899	4.3903E-34	Neu
TFEB4	3.0246E-38	0.18688332	0.05	0.176	4.6491E-34	Neu
KCNAB2	3.1567E-38	0.14843042	0.561	0.931	4.8522E-34	Neu
SPATA2	3.1574E-38	0.15248777	0.039	0.158	4.8533E-34	Neu
MXRA71	3.3103E-38	0.15588583	0.061	0.193	5.0883E-34	Neu

POFUT2	3.3966E-38	0.2921965	0.046	0.166	5.2209E-34	Neu
ZNF354A1	3.6084E-38	0.30545389	0.061	0.194	5.5465E-34	Neu
DHX57	3.7735E-38	0.28873683	0.058	0.189	5.8002E-34	Neu
PPP1R14B1	3.8348E-38	0.17352708	0.05	0.175	5.8945E-34	Neu
SOCS5	4.9896E-38	0.64119935	0.084	0.231	7.6695E-34	Neu
DTNB	5.1071E-38	0.15296945	0.054	0.182	7.8501E-34	Neu
ZMYND19	5.1892E-38	0.25596775	0.043	0.162	7.9764E-34	Neu
RNF2161	5.5987E-38	0.17247955	0.045	0.166	8.6058E-34	Neu
FAN1	5.6883E-38	0.17174816	0.051	0.177	8.7435E-34	Neu
MAP2K1	6.5515E-38	0.16301528	0.477	0.872	1.007E-33	Neu
CEP1282	6.9973E-38	0.14602126	0.059	0.189	1.0756E-33	Neu
ARNTL	8.5692E-38	0.20747218	0.046	0.168	1.3172E-33	Neu
ZNF3	8.6013E-38	0.51336933	0.056	0.185	1.3221E-33	Neu
ZNF700	9.6502E-38	0.38230772	0.088	0.234	1.4833E-33	Neu
TRIM39	9.7529E-38	0.43088348	0.07	0.207	1.4991E-33	Neu
TBCCD11	9.9902E-38	0.50729621	0.082	0.226	1.5356E-33	Neu
ATG4D	1.0844E-37	0.15386382	0.044	0.165	1.6668E-33	Neu
UBOX5	1.4816E-37	0.12812198	0.061	0.192	2.2773E-33	Neu
TTC41	1.5208E-37	0.33816968	0.059	0.188	2.3376E-33	Neu
ZNF585A1	1.5679E-37	0.22629344	0.05	0.174	2.41E-33	Neu
HILPDA1	1.6199E-37	0.6176164	0.083	0.228	2.49E-33	Neu
ZNF6391	1.6219E-37	0.41791839	0.101	0.256	2.493E-33	Neu
ZNF263	1.6446E-37	0.25982458	0.054	0.179	2.5279E-33	Neu
SCML12	1.7473E-37	0.5600397	0.062	0.193	2.6858E-33	Neu
RINT11	1.9364E-37	0.37850203	0.063	0.194	2.9764E-33	Neu
DDX311	1.9766E-37	0.30589532	0.071	0.208	3.0383E-33	Neu
FNTB1	2.0052E-37	0.1275873	0.046	0.166	3.0822E-33	Neu
PUS7L	2.011E-37	0.3461721	0.091	0.24	3.0912E-33	Neu
PPM1D1	2.1876E-37	0.48148563	0.064	0.196	3.3626E-33	Neu
AASDH1	2.2244E-37	0.49531417	0.078	0.219	3.4192E-33	Neu
ZBTB61	2.2817E-37	0.36420054	0.07	0.207	3.5072E-33	Neu
C9orf723	2.3134E-37	0.7402856	0.319	0.621	3.5559E-33	Neu
ZNF72	2.6403E-37	0.39389809	0.067	0.2	4.0585E-33	Neu
GCA2	2.709E-37	0.17293943	0.522	0.9	4.164E-33	Neu
ZBED51	2.8258E-37	0.20808253	0.07	0.204	4.3436E-33	Neu
AC118549.1	2.9286E-37	0.40876887	0.087	0.233	4.5016E-33	Neu
RWDD41	3.023E-37	0.3968265	0.092	0.243	4.6467E-33	Neu
YPEL34	3.027E-37	0.86644474	0.087	0.232	4.6528E-33	Neu
CD5L1	3.2041E-37	0.5708629	0.299	0.574	4.9251E-33	Neu
RBM12B1	3.2163E-37	0.29734617	0.076	0.216	4.9438E-33	Neu
RLF	3.2545E-37	0.32192759	0.072	0.208	5.0024E-33	Neu
COIL	3.9243E-37	0.13258464	0.061	0.191	6.0321E-33	Neu
WDR601	3.9922E-37	0.45339666	0.062	0.192	6.1364E-33	Neu
C3orf14	4.348E-37	0.14302856	0.04	0.156	6.6834E-33	Neu
CCDC1122	4.7049E-37	0.11942409	0.056	0.183	7.2319E-33	Neu
MASTL	4.9663E-37	0.47079619	0.074	0.211	7.6337E-33	Neu
PDK11	5.6399E-37	0.31948333	0.082	0.224	8.6691E-33	Neu
PIK3C2A1	6.0529E-37	0.31996264	0.081	0.223	9.3039E-33	Neu
CCDC126	6.2954E-37	0.38435186	0.07	0.204	9.6766E-33	Neu
C11orf741	6.369E-37	0.20166567	0.055	0.181	9.7899E-33	Neu
TUBGCP51	6.5717E-37	0.11479793	0.051	0.172	1.0101E-32	Neu
FTCDNL12	6.5767E-37	0.21028137	0.064	0.195	1.0109E-32	Neu

MYO1F2	6.6827E-37	0.4187707	0.5	0.903	1.0272E-32	Neu
SYTL31	7.047E-37	0.3791342	0.048	0.169	1.0832E-32	Neu
AKR1C21	7.1013E-37	0.28549	0.051	0.173	1.0915E-32	Neu
SMU11	7.3846E-37	0.38983051	0.067	0.201	1.1351E-32	Neu
TTC281	7.3863E-37	0.33491214	0.061	0.19	1.1354E-32	Neu
UBN2	7.5063E-37	0.49836543	0.099	0.251	1.1538E-32	Neu
CPED11	7.82E-37	0.18370805	0.452	0.824	1.202E-32	Neu
JMY	8.7041E-37	0.15264272	0.062	0.191	1.3379E-32	Neu
YTHDF11	9.2918E-37	0.14495862	0.062	0.193	1.4282E-32	Neu
PHC11	1.0012E-36	0.47633729	0.082	0.225	1.5389E-32	Neu
PUSL11	1.1686E-36	0.24649823	0.046	0.165	1.7963E-32	Neu
NPHP3	1.2241E-36	0.62878144	0.064	0.194	1.8815E-32	Neu
PSPH2	1.2244E-36	0.68383475	0.071	0.207	1.882E-32	Neu
POLR3F1	1.2686E-36	0.35394621	0.059	0.188	1.95E-32	Neu
DCTN5	1.3122E-36	0.12325156	0.048	0.167	2.0169E-32	Neu
RMDN21	1.3295E-36	0.31699453	0.052	0.175	2.0435E-32	Neu
TADA2A	1.3304E-36	0.33759524	0.056	0.182	2.0449E-32	Neu
CAPRIN2	1.3492E-36	0.16275926	0.086	0.229	2.0738E-32	Neu
RELB3	1.5361E-36	0.76790226	0.041	0.155	2.3611E-32	Neu
SCFD21	1.6164E-36	0.13626038	0.055	0.179	2.4846E-32	Neu
ZNF7861	1.6408E-36	0.26568932	0.053	0.176	2.5221E-32	Neu
DHX342	1.6672E-36	0.85189985	0.083	0.225	2.5627E-32	Neu
MTHFSD1	1.7012E-36	0.36907001	0.055	0.179	2.6149E-32	Neu
TOP3B1	1.7748E-36	0.12741892	0.067	0.198	2.728E-32	Neu
NAA35	1.981E-36	0.3724451	0.085	0.227	3.045E-32	Neu
PPCDC1	2.1728E-36	1.85021323	0.095	0.246	3.3399E-32	Neu
MFSD81	2.4197E-36	0.277304	0.07	0.203	3.7193E-32	Neu
SETD1A1	2.5876E-36	0.17684804	0.046	0.163	3.9774E-32	Neu
DGKH	3.1224E-36	0.39144492	0.065	0.195	4.7994E-32	Neu
ZMYM1	3.2413E-36	0.27395737	0.06	0.188	4.9823E-32	Neu
ERCC61	3.4315E-36	0.60023924	0.066	0.196	5.2746E-32	Neu
ZNF1971	3.4482E-36	0.26001808	0.062	0.19	5.3002E-32	Neu
L3MBTL3	3.4636E-36	0.1132417	0.062	0.19	5.3239E-32	Neu
PRMT31	3.4745E-36	0.45351269	0.056	0.18	5.3407E-32	Neu
COA11	3.6369E-36	0.40101347	0.05	0.17	5.5903E-32	Neu
FAM189B	3.6528E-36	0.44467641	0.051	0.172	5.6148E-32	Neu
IDS	3.7092E-36	0.20196676	0.514	0.907	5.7014E-32	Neu
TBC1D122	3.7366E-36	0.18425985	0.098	0.247	5.7435E-32	Neu
RAMAC	3.8284E-36	0.30829213	0.054	0.178	5.8847E-32	Neu
FAXDC21	3.9682E-36	0.21075962	0.076	0.211	6.0995E-32	Neu
GK5	4.2192E-36	0.33126644	0.064	0.192	6.4853E-32	Neu
ZNF841	4.487E-36	0.30302432	0.064	0.193	6.8969E-32	Neu
EIF4ENIF1	5.3471E-36	0.31391246	0.085	0.226	8.219E-32	Neu
KLHL9	5.8445E-36	0.29894345	0.072	0.206	8.9836E-32	Neu
PPP1R13B1	6.0386E-36	0.13024042	0.044	0.161	9.2819E-32	Neu
ABTB11	6.0667E-36	1.09640509	0.08	0.218	9.3252E-32	Neu
MFSD3	6.694E-36	0.20938736	0.047	0.165	1.0289E-31	Neu
CCNB1IP11	6.7191E-36	0.22940016	0.056	0.181	1.0328E-31	Neu
TIMM211	7.3244E-36	0.10029748	0.062	0.189	1.1258E-31	Neu
SQSTM11	7.5334E-36	0.22302819	0.647	0.957	1.158E-31	Neu
BCAS31	7.6127E-36	0.40762019	0.064	0.192	1.1701E-31	Neu
ASB31	8.2883E-36	0.41260176	0.107	0.262	1.274E-31	Neu

ATAD2B3	8.4957E-36	0.31245892	0.105	0.256	1.3059E-31	Neu
AGFG11	8.7307E-36	0.21808821	0.489	0.886	1.342E-31	Neu
DUSP121	8.904E-36	0.13104998	0.06	0.186	1.3686E-31	Neu
DALRD3	8.9294E-36	0.29822852	0.058	0.182	1.3725E-31	Neu
TRMT61B	8.9598E-36	0.26746876	0.063	0.191	1.3772E-31	Neu
ORC31	9.4045E-36	0.60172332	0.074	0.209	1.4456E-31	Neu
PNPLA42	9.7016E-36	0.15579672	0.062	0.189	1.4912E-31	Neu
RAD51C1	1.0501E-35	0.24172037	0.038	0.15	1.614E-31	Neu
BCL64	1.0722E-35	1.36316726	0.222	0.451	1.6481E-31	Neu
TFB1M1	1.2472E-35	0.31488752	0.05	0.168	1.917E-31	Neu
CRY1	1.2763E-35	0.68762323	0.065	0.194	1.9618E-31	Neu
DNPH13	1.496E-35	0.11876691	0.025	0.126	2.2995E-31	Neu
HLCS1	1.5186E-35	0.21561833	0.066	0.194	2.3342E-31	Neu
SMC5	1.7458E-35	0.1080452	0.053	0.173	2.6835E-31	Neu
CUL9	1.7813E-35	0.15331769	0.044	0.159	2.7381E-31	Neu
BTRC	1.9345E-35	0.3192369	0.063	0.19	2.9736E-31	Neu
ARHGAP182	2.0304E-35	0.31507543	0.486	0.879	3.121E-31	Neu
EDRF1	2.071E-35	0.33115541	0.068	0.197	3.1833E-31	Neu
MBNL13	2.1006E-35	0.20047967	0.506	0.909	3.2288E-31	Neu
HP1	2.1328E-35	0.20206283	0.21	0.403	3.2783E-31	Neu
GPR180	2.1466E-35	0.16625764	0.063	0.189	3.2995E-31	Neu
ING5	2.2036E-35	0.21543899	0.053	0.172	3.3872E-31	Neu
SULT1A11	2.2127E-35	0.4189086	0.046	0.162	3.4012E-31	Neu
ZC3HC11	2.5957E-35	0.29773035	0.048	0.166	3.9899E-31	Neu
PACRGL1	2.6209E-35	0.37885898	0.056	0.178	4.0285E-31	Neu
PAQR3	2.6942E-35	0.22316771	0.058	0.182	4.1412E-31	Neu
MAP1LC3A	2.8652E-35	0.79156761	0.029	0.133	4.4041E-31	Neu
METTL8	3.1103E-35	0.17973148	0.047	0.163	4.7809E-31	Neu
PUS72	3.2428E-35	0.3594022	0.05	0.168	4.9844E-31	Neu
SIRT31	3.2519E-35	0.10930402	0.041	0.152	4.9985E-31	Neu
LCLAT1	3.4118E-35	0.33123347	0.044	0.158	5.2443E-31	Neu
SKIL3	3.5526E-35	0.36337762	0.475	0.875	5.4607E-31	Neu
NFE21	3.596E-35	0.62159758	0.119	0.278	5.5274E-31	Neu
HCAR31	3.6916E-35	2.10097662	0.131	0.302	5.6743E-31	Neu
SH3GLB1	3.7164E-35	0.30940348	0.471	0.859	5.7124E-31	Neu
COX201	3.8566E-35	0.35955156	0.067	0.197	5.928E-31	Neu
SGSM2	4.1313E-35	0.36116259	0.061	0.185	6.3502E-31	Neu
ATP6V1B22	4.2197E-35	0.11797967	0.572	0.928	6.4861E-31	Neu
IFT881	4.4635E-35	0.30080869	0.047	0.163	6.8609E-31	Neu
TRGC2	4.4725E-35	0.24649704	0.076	0.21	6.8747E-31	Neu
PLAGL21	4.7595E-35	0.47235819	0.062	0.187	7.3158E-31	Neu
FAM228B1	4.9456E-35	0.31058683	0.088	0.229	7.6018E-31	Neu
FTH11	5.4914E-35	0.20758499	0.997	0.995	8.4409E-31	Neu
TIMM8A1	5.7317E-35	0.26048957	0.043	0.156	8.8102E-31	Neu
INTS51	5.9739E-35	0.12376259	0.037	0.146	9.1825E-31	Neu
IRAK31	6.3452E-35	0.89024714	0.346	0.668	9.7531E-31	Neu
ALKBH41	6.4497E-35	0.11815833	0.041	0.153	9.9138E-31	Neu
NTHL11	8.0742E-35	0.14915481	0.051	0.168	1.2411E-30	Neu
PALB21	8.7722E-35	0.19535527	0.043	0.157	1.3484E-30	Neu
CBR4	9.6518E-35	0.56569715	0.071	0.201	1.4836E-30	Neu
HAUS3	1.0353E-34	0.12151678	0.063	0.188	1.5914E-30	Neu
SORD	1.0377E-34	0.38660219	0.047	0.163	1.5951E-30	Neu

GXYLT11	1.1139E-34	0.1419944	0.047	0.161	1.7122E-30	Neu
CD221	1.2204E-34	0.47095445	0.029	0.131	1.8759E-30	Neu
CBX81	1.253E-34	0.13310242	0.044	0.158	1.926E-30	Neu
RBL11	1.3239E-34	0.13365843	0.052	0.17	2.035E-30	Neu
TXNDC111	1.3937E-34	0.12067578	0.533	0.914	2.1423E-30	Neu
CEP162	1.4197E-34	0.69389845	0.079	0.213	2.1822E-30	Neu
RWDD2A1	1.6262E-34	0.25559056	0.046	0.16	2.4997E-30	Neu
ANKRD541	1.7E-34	0.24535354	0.044	0.156	2.613E-30	Neu
ZNF707	1.7892E-34	0.11524599	0.038	0.145	2.7502E-30	Neu
RBBP8	1.8228E-34	0.37816873	0.047	0.162	2.8019E-30	Neu
SENP1	1.8242E-34	0.19903807	0.076	0.209	2.8039E-30	Neu
KIAA0586	1.8896E-34	0.3153531	0.066	0.192	2.9045E-30	Neu
MDN1	1.8971E-34	0.14235462	0.06	0.182	2.916E-30	Neu
FANCL1	1.9064E-34	0.21365812	0.048	0.163	2.9303E-30	Neu
E2F61	1.9412E-34	0.58325489	0.082	0.217	2.9838E-30	Neu
CROT	1.9989E-34	0.13572023	0.033	0.138	3.0725E-30	Neu
RNF170	2.092E-34	0.35472413	0.057	0.178	3.2156E-30	Neu
METTL15	2.1412E-34	0.24596586	0.054	0.173	3.2912E-30	Neu
PLD1	2.1974E-34	0.67304343	0.106	0.256	3.3776E-30	Neu
CD552	2.2122E-34	0.53642476	0.483	0.869	3.4004E-30	Neu
CAMK2G	2.2268E-34	0.26669092	0.06	0.183	3.4228E-30	Neu
ZNF808	2.3294E-34	0.31617381	0.079	0.213	3.5805E-30	Neu
SNX161	2.349E-34	0.52311767	0.066	0.191	3.6107E-30	Neu
LYPD5	2.383E-34	0.26227943	0.031	0.134	3.6629E-30	Neu
ZNF333	2.3959E-34	0.16524128	0.064	0.188	3.6827E-30	Neu
HIF1A2	2.4248E-34	1.08264272	0.08	0.216	3.7272E-30	Neu
HSBP1L1	2.4966E-34	0.21130891	0.054	0.172	3.8375E-30	Neu
MEX3D1	2.572E-34	0.27520418	0.041	0.151	3.9534E-30	Neu
NSUN71	2.7322E-34	0.4941048	0.051	0.168	4.1996E-30	Neu
VPS33B1	3.0614E-34	0.33114923	0.05	0.166	4.7057E-30	Neu
ZBP1	3.0635E-34	0.70513495	0.06	0.182	4.7089E-30	Neu
PELI12	3.3432E-34	1.11528705	0.125	0.286	5.1388E-30	Neu
ERCC41	3.4465E-34	0.10923254	0.041	0.151	5.2976E-30	Neu
CSTA1	3.6735E-34	0.13379161	0.512	0.881	5.6465E-30	Neu
TMEM2671	3.8068E-34	0.14563655	0.052	0.168	5.8514E-30	Neu
OSBPL111	4.0029E-34	0.27367374	0.503	0.892	6.1529E-30	Neu
ZNF2232	4.6728E-34	0.44542548	0.055	0.174	7.1826E-30	Neu
KLC41	4.6974E-34	0.15931327	0.036	0.142	7.2204E-30	Neu
COX19	4.7016E-34	0.38940142	0.051	0.166	7.2268E-30	Neu
BTG13	4.7479E-34	0.10413753	0.519	0.899	7.298E-30	Neu
SNRNP48	4.7807E-34	0.42180395	0.068	0.195	7.3484E-30	Neu
IL6R4	4.887E-34	0.79760291	0.059	0.18	7.5118E-30	Neu
AIFM31	5.0904E-34	0.20824913	0.044	0.154	7.8245E-30	Neu
SPATA5L11	5.2701E-34	0.36200271	0.051	0.167	8.1007E-30	Neu
SLA1	5.3218E-34	0.15693856	0.572	0.922	8.1801E-30	Neu
FAM98C1	5.6447E-34	0.69688126	0.07	0.197	8.6765E-30	Neu
SGMS21	5.8275E-34	0.21879271	0.468	0.842	8.9574E-30	Neu
TEPSIN1	5.8881E-34	0.24896804	0.033	0.137	9.0506E-30	Neu
TECPR2	7.9978E-34	1.25102698	0.067	0.193	1.2293E-29	Neu
CEP89	8.1267E-34	0.27999404	0.059	0.178	1.2491E-29	Neu
SUPT3H	8.2996E-34	0.26850963	0.058	0.178	1.2757E-29	Neu
TRIM52	8.3255E-34	0.57282274	0.087	0.225	1.2797E-29	Neu

CEP832	8.6576E-34	0.35544696	0.058	0.177	1.3308E-29	Neu
MAP4K31	1.005E-33	0.1972344	0.05	0.165	1.5447E-29	Neu
PIGW1	1.0066E-33	0.2282962	0.047	0.16	1.5473E-29	Neu
SIRT12	1.0304E-33	0.52216502	0.087	0.224	1.5839E-29	Neu
SVIL1	1.0491E-33	0.14944126	0.535	0.913	1.6125E-29	Neu
SNAPC4	1.307E-33	0.36887565	0.058	0.177	2.0089E-29	Neu
TUT71	1.3747E-33	0.38837313	0.491	0.885	2.113E-29	Neu
SURF21	1.8281E-33	0.43267612	0.067	0.191	2.81E-29	Neu
HTRA42	1.9935E-33	0.2638588	0.035	0.138	3.0643E-29	Neu
ST3GAL31	2.0599E-33	0.10738745	0.055	0.172	3.1663E-29	Neu
APPL21	2.0943E-33	0.47734021	0.064	0.185	3.2192E-29	Neu
CLIC41	2.2041E-33	0.11357458	0.522	0.903	3.388E-29	Neu
EHBP1	2.2546E-33	0.24116895	0.052	0.166	3.4656E-29	Neu
FAM126B	2.4575E-33	0.56058386	0.088	0.224	3.7775E-29	Neu
RICTOR	2.5424E-33	0.96105776	0.098	0.241	3.9078E-29	Neu
IPPK	2.79E-33	0.13999855	0.051	0.165	4.2885E-29	Neu
FAM117B	3.0657E-33	0.24957446	0.055	0.171	4.7122E-29	Neu
ECT2	3.164E-33	0.40578059	0.065	0.187	4.8634E-29	Neu
TNF1	3.2559E-33	0.14245199	0.047	0.157	5.0046E-29	Neu
USE1	3.2848E-33	0.45243289	0.04	0.146	5.0491E-29	Neu
PTGR21	3.3367E-33	0.50618072	0.045	0.155	5.1289E-29	Neu
CLK42	3.4631E-33	0.61568847	0.072	0.199	5.3231E-29	Neu
CSNK1G31	3.8055E-33	0.33768145	0.062	0.183	5.8494E-29	Neu
GADD45B3	3.9803E-33	1.09814416	0.049	0.161	6.118E-29	Neu
TRAM23	4.0967E-33	0.22820578	0.038	0.143	6.2971E-29	Neu
FMO5	4.3514E-33	0.16015628	0.06	0.178	6.6885E-29	Neu
ZNF6911	4.699E-33	0.4185745	0.052	0.166	7.2228E-29	Neu
MON1A1	4.7505E-33	0.41009161	0.039	0.145	7.3021E-29	Neu
NSMF2	4.7607E-33	0.4132887	0.053	0.168	7.3176E-29	Neu
CAMKMT1	4.9884E-33	0.19712076	0.06	0.179	7.6676E-29	Neu
SWI51	5.8375E-33	0.17199697	0.059	0.176	8.9728E-29	Neu
DDX101	6.1183E-33	0.40438295	0.058	0.175	9.4044E-29	Neu
MPP51	6.1373E-33	0.51910871	0.071	0.196	9.4336E-29	Neu
ANKRD502	6.338E-33	0.19112939	0.075	0.202	9.7421E-29	Neu
LRRFIP11	6.6127E-33	0.16185728	0.528	0.925	1.0164E-28	Neu
CRADD	6.8901E-33	0.1452978	0.056	0.172	1.0591E-28	Neu
FLVCR1	7.1433E-33	0.13314169	0.059	0.176	1.098E-28	Neu
DNAJB4	7.3294E-33	0.1493636	0.037	0.141	1.1266E-28	Neu
TFCP2L11	8.1555E-33	0.44647676	0.052	0.166	1.2536E-28	Neu
C11orf71	8.2206E-33	0.34598685	0.045	0.154	1.2636E-28	Neu
SS18L1	8.6497E-33	0.39315891	0.072	0.197	1.3295E-28	Neu
ARL5A	8.6891E-33	0.30305035	0.044	0.154	1.3356E-28	Neu
GYPC1	9.0511E-33	0.11918704	0.539	0.904	1.3912E-28	Neu
CIDEB	9.187E-33	0.19332582	0.061	0.18	1.4121E-28	Neu
MYO15B4	9.7444E-33	0.64671446	0.098	0.236	1.4978E-28	Neu
ADGRE23	1.0366E-32	1.70812872	0.059	0.176	1.5934E-28	Neu
CSF3R2	1.1106E-32	2.97858403	0.221	0.451	1.7072E-28	Neu
NME7	1.2139E-32	0.45188105	0.042	0.148	1.866E-28	Neu
CPEB3	1.4395E-32	0.40426789	0.067	0.189	2.2127E-28	Neu
TBCE.11	1.5141E-32	0.4483494	0.051	0.163	2.3273E-28	Neu
ZSCAN211	1.5296E-32	0.30962619	0.05	0.16	2.3512E-28	Neu
MYB	1.5842E-32	0.57537794	0.167	0.346	2.4351E-28	Neu

RPGR1	1.6133E-32	0.59910462	0.082	0.212	2.4798E-28	Neu
RFX23	2.282E-32	0.42124619	0.05	0.161	3.5077E-28	Neu
RBM45	2.5791E-32	0.11932139	0.046	0.155	3.9644E-28	Neu
UTP201	2.9255E-32	0.56383997	0.048	0.158	4.4968E-28	Neu
UHRF2	3.2051E-32	0.15681487	0.069	0.19	4.9265E-28	Neu
TNRC6C	3.6302E-32	0.49225469	0.079	0.207	5.58E-28	Neu
PNPLA71	3.9551E-32	0.57644883	0.043	0.149	6.0794E-28	Neu
NPAT1	4.2612E-32	0.39084105	0.076	0.201	6.5498E-28	Neu
UBXN2A	4.282E-32	0.20092731	0.056	0.171	6.5819E-28	Neu
PROK2	4.3677E-32	5.97682062	0.014	0.002	6.7136E-28	Neu
CDK71	4.5385E-32	0.17812037	0.041	0.145	6.9762E-28	Neu
MIGA2	7.1871E-32	0.32670828	0.042	0.147	1.1047E-27	Neu
DCAF16	7.7703E-32	0.41164255	0.082	0.211	1.1944E-27	Neu
OSGEPL1	7.8937E-32	0.10527415	0.038	0.141	1.2133E-27	Neu
HSPA63	8.1754E-32	0.92794278	0.11	0.255	1.2566E-27	Neu
CCDC181	8.7102E-32	0.35493336	0.051	0.162	1.3388E-27	Neu
CARMIL11	8.9555E-32	0.10517895	0.027	0.121	1.3766E-27	Neu
PADI23	9.5795E-32	0.61462828	0.064	0.182	1.4725E-27	Neu
EIF3E	9.6416E-32	0.62330377	0.14	0.305	1.482E-27	Neu
ZNF7871	9.8574E-32	0.12909813	0.021	0.112	1.5152E-27	Neu
NLRP123	9.8804E-32	0.49785833	0.043	0.148	1.5187E-27	Neu
PRKCI	1.1004E-31	0.18734252	0.052	0.163	1.6914E-27	Neu
POLR1C	1.1113E-31	0.35101477	0.043	0.148	1.7082E-27	Neu
ZNF626	1.1134E-31	0.34984275	0.046	0.152	1.7114E-27	Neu
MRI1	1.1209E-31	0.48617052	0.042	0.147	1.7229E-27	Neu
SP4	1.2258E-31	0.24353322	0.074	0.196	1.8842E-27	Neu
UPF3A1	1.4103E-31	0.27368107	0.074	0.198	2.1678E-27	Neu
TMEM255B2	1.4115E-31	0.50439806	0.052	0.162	2.1696E-27	Neu
SPRED2	1.4298E-31	0.16701286	0.051	0.162	2.1978E-27	Neu
PTK21	1.558E-31	0.16475061	0.039	0.14	2.3949E-27	Neu
WRN	1.5948E-31	0.27957315	0.048	0.156	2.4514E-27	Neu
ZNF736	1.6142E-31	0.57172156	0.091	0.225	2.4811E-27	Neu
TGDS1	1.6722E-31	0.22307307	0.047	0.154	2.5704E-27	Neu
ICE2	1.6901E-31	0.2761418	0.066	0.183	2.5978E-27	Neu
POMT1	1.7411E-31	0.24456719	0.034	0.133	2.6763E-27	Neu
ABHD51	1.8785E-31	0.46633349	0.466	0.846	2.8874E-27	Neu
NPHP41	1.9703E-31	0.35120703	0.048	0.155	3.0286E-27	Neu
RGS142	2.1217E-31	0.12911002	0.057	0.17	3.2613E-27	Neu
PHETA12	2.6091E-31	0.33460999	0.032	0.129	4.0104E-27	Neu
ZNF839	2.7668E-31	0.4313451	0.042	0.146	4.2528E-27	Neu
CEP1351	2.7672E-31	0.31977603	0.068	0.187	4.2534E-27	Neu
TRMT441	2.9142E-31	0.27107494	0.042	0.145	4.4794E-27	Neu
FBXO44	2.9145E-31	0.25970575	0.039	0.14	4.4799E-27	Neu
CKAP2	3.3227E-31	0.24162941	0.059	0.171	5.1073E-27	Neu
NFKBID3	3.6505E-31	1.52202473	0.108	0.25	5.6113E-27	Neu
DDX511	4.1739E-31	0.1598871	0.036	0.135	6.4157E-27	Neu
ALG61	4.4094E-31	0.37053034	0.047	0.154	6.7777E-27	Neu
CCDC1863	4.8455E-31	0.2336371	0.068	0.187	7.4481E-27	Neu
NFKB23	4.921E-31	1.84979576	0.083	0.211	7.5641E-27	Neu
ZNF5501	4.9525E-31	0.82746069	0.067	0.184	7.6125E-27	Neu
ZNF195	5.2409E-31	0.20937277	0.042	0.145	8.0557E-27	Neu
WDR91	5.4034E-31	0.49290289	0.054	0.164	8.3056E-27	Neu

SPIN41	5.4591E-31	0.1051299	0.045	0.149	8.3912E-27	Neu
FKTN	5.8437E-31	0.26134905	0.04	0.141	8.9824E-27	Neu
TMEM260	6.0501E-31	0.59398802	0.064	0.179	9.2997E-27	Neu
MAML32	6.1488E-31	0.45425003	0.099	0.235	9.4513E-27	Neu
TSPAN322	6.2286E-31	0.34240732	0.053	0.162	9.574E-27	Neu
NAA80	6.2631E-31	0.25037307	0.043	0.145	9.6271E-27	Neu
TAF1C1	6.3494E-31	0.14576294	0.048	0.155	9.7597E-27	Neu
ZNF568	6.4867E-31	0.60686727	0.073	0.194	9.9706E-27	Neu
LXN1	6.5197E-31	0.12158957	0.053	0.163	1.0021E-26	Neu
CRAMP1	7.1349E-31	0.56643376	0.057	0.169	1.0967E-26	Neu
ORC2	7.1438E-31	0.50964828	0.063	0.178	1.0981E-26	Neu
ZNF2831	7.3641E-31	0.33342084	0.056	0.168	1.1319E-26	Neu
ULK4	7.6446E-31	0.67345077	0.085	0.212	1.1751E-26	Neu
ARPC21	8.0078E-31	0.26333828	0.523	0.906	1.2309E-26	Neu
SLX41	8.0606E-31	0.33540298	0.041	0.142	1.239E-26	Neu
ZCCHC7	8.2239E-31	0.64545518	0.091	0.222	1.2641E-26	Neu
CREB53	8.3194E-31	1.48177678	0.05	0.158	1.2788E-26	Neu
FAM104B	9.5835E-31	0.75566703	0.078	0.202	1.4731E-26	Neu
MYLIP2	9.5935E-31	0.48809439	0.035	0.132	1.4746E-26	Neu
RBM43	9.6377E-31	0.2357658	0.046	0.151	1.4814E-26	Neu
BIN3	9.6379E-31	0.16901764	0.035	0.133	1.4814E-26	Neu
ZNF5951	9.9057E-31	0.45803591	0.063	0.179	1.5226E-26	Neu
PIBF1	1.1168E-30	0.3385613	0.059	0.172	1.7166E-26	Neu
TXNL4B1	1.1445E-30	0.35787134	0.043	0.146	1.7592E-26	Neu
SNRPD11	1.1635E-30	0.34845527	0.045	0.149	1.7884E-26	Neu
FBN11	1.1881E-30	0.18009711	0.059	0.169	1.8263E-26	Neu
NIPAL21	1.2397E-30	0.46240388	0.064	0.181	1.9055E-26	Neu
TAMM41	1.2996E-30	0.28170578	0.053	0.162	1.9977E-26	Neu
PCNX41	1.3149E-30	0.31893884	0.044	0.146	2.0211E-26	Neu
BMPR1A1	1.4959E-30	0.29438524	0.048	0.154	2.2993E-26	Neu
PARP112	1.4984E-30	0.10968758	0.047	0.152	2.3032E-26	Neu
LRRCC11	1.4992E-30	0.38021812	0.049	0.155	2.3044E-26	Neu
ERCC8	1.5296E-30	0.49214999	0.055	0.166	2.3511E-26	Neu
TMTC3	1.7933E-30	0.26710259	0.035	0.132	2.7565E-26	Neu
SCLT11	1.8024E-30	0.82137839	0.073	0.192	2.7704E-26	Neu
NAA252	1.8126E-30	0.26056612	0.041	0.142	2.7861E-26	Neu
B4GALT2	1.9148E-30	0.1472433	0.018	0.102	2.9432E-26	Neu
EAF22	1.9838E-30	0.38190141	0.067	0.184	3.0493E-26	Neu
SLC25A511	2.0897E-30	0.28251942	0.055	0.164	3.2121E-26	Neu
SOCS7	2.2633E-30	0.31915933	0.046	0.149	3.4789E-26	Neu
LIN7B1	2.3536E-30	0.32365698	0.052	0.16	3.6178E-26	Neu
IPMK1	2.4218E-30	0.52519611	0.079	0.202	3.7226E-26	Neu
UBXN10	2.6083E-30	0.12592749	0.041	0.141	4.0092E-26	Neu
TTBK2	2.6557E-30	0.13266573	0.05	0.156	4.082E-26	Neu
ERO1B	2.6686E-30	0.36242024	0.042	0.143	4.1019E-26	Neu
SEPHS11	2.8374E-30	0.6828588	0.08	0.206	4.3614E-26	Neu
NFE2L21	2.9578E-30	0.19969978	0.55	0.92	4.5464E-26	Neu
ERN12	3.2338E-30	0.7389603	0.056	0.165	4.9707E-26	Neu
SYTL11	3.2865E-30	0.28436255	0.077	0.197	5.0516E-26	Neu
ALS21	3.3131E-30	0.36639879	0.048	0.152	5.0925E-26	Neu
ARMCX51	3.3388E-30	0.34184917	0.052	0.16	5.132E-26	Neu
HCLS12	3.5487E-30	0.11202821	0.621	0.959	5.4548E-26	Neu

PSTPIP22	3.6048E-30	0.89818286	0.043	0.143	5.5409E-26	Neu
MDM11	3.644E-30	0.40686909	0.061	0.173	5.6012E-26	Neu
ZNF1361	3.8097E-30	0.25608266	0.052	0.16	5.8559E-26	Neu
SNX71	4.3787E-30	0.32191401	0.038	0.135	6.7305E-26	Neu
KLF22	4.5511E-30	1.46288221	0.371	0.669	6.9955E-26	Neu
ZNF514	4.8523E-30	0.27135911	0.051	0.156	7.4585E-26	Neu
PARP16	4.8702E-30	0.12473639	0.05	0.155	7.486E-26	Neu
APBB21	5.2951E-30	0.11153964	0.035	0.132	8.1391E-26	Neu
ZMYM5	5.8799E-30	0.32140293	0.058	0.166	9.038E-26	Neu
ATG16L2	6.9549E-30	1.03380473	0.329	0.621	1.069E-25	Neu
ZRSR2	7.0491E-30	0.29856536	0.057	0.167	1.0835E-25	Neu
FAM122A	7.6975E-30	0.32950424	0.058	0.167	1.1832E-25	Neu
CYTH32	7.7411E-30	0.18461835	0.044	0.145	1.1899E-25	Neu
MOGAT11	7.758E-30	0.37798633	0.094	0.222	1.1925E-25	Neu
TYSND1	7.7842E-30	0.38475739	0.035	0.131	1.1965E-25	Neu
ZSCAN25	7.807E-30	0.19878338	0.038	0.136	1.2E-25	Neu
PPFIBP12	7.9305E-30	0.40894548	0.048	0.151	1.219E-25	Neu
SLFN121	8.4914E-30	0.17461667	0.035	0.131	1.3052E-25	Neu
DPCD	8.5908E-30	0.52975246	0.066	0.18	1.3205E-25	Neu
ARHGAP51	8.934E-30	0.62773752	0.091	0.222	1.3732E-25	Neu
USP37	9.6239E-30	0.36622979	0.062	0.174	1.4793E-25	Neu
OSBPL5	1.0368E-29	0.12789911	0.026	0.115	1.5937E-25	Neu
ZNF738	1.0392E-29	0.41203043	0.06	0.17	1.5973E-25	Neu
MTRF1	1.099E-29	0.49583646	0.071	0.188	1.6892E-25	Neu
FAM241B1	1.1305E-29	0.13389662	0.028	0.118	1.7377E-25	Neu
IL71	1.2703E-29	0.19072851	0.05	0.154	1.9526E-25	Neu
DCAF13	1.2884E-29	0.25694509	0.056	0.164	1.9804E-25	Neu
CCDC1702	1.3711E-29	0.27883321	0.072	0.188	2.1075E-25	Neu
ACTR5	1.4196E-29	0.29911792	0.05	0.154	2.1821E-25	Neu
CEP1311	1.5029E-29	0.14753072	0.029	0.119	2.31E-25	Neu
IL15	1.5081E-29	0.44594344	0.036	0.131	2.3181E-25	Neu
RBM8A1	1.5828E-29	0.13936759	0.036	0.131	2.433E-25	Neu
THAP51	1.5996E-29	0.36540857	0.051	0.156	2.4588E-25	Neu
DTWD21	1.7057E-29	0.37565453	0.054	0.161	2.6219E-25	Neu
TMEM2341	1.7359E-29	0.4335246	0.062	0.173	2.6682E-25	Neu
TMCC32	1.7383E-29	0.1970303	0.029	0.119	2.672E-25	Neu
TRIT1	1.7532E-29	0.64289473	0.054	0.16	2.6949E-25	Neu
PLEKHA1	1.7664E-29	0.19956655	0.051	0.155	2.7152E-25	Neu
SPA17	1.8721E-29	0.39599045	0.047	0.149	2.8776E-25	Neu
ZNF842	1.9542E-29	0.18896141	0.055	0.161	3.0038E-25	Neu
CENPC1	2.0389E-29	0.22745018	0.066	0.179	3.134E-25	Neu
TAF1	2.0678E-29	0.27829888	0.068	0.182	3.1784E-25	Neu
VEZT	2.1488E-29	0.30271622	0.061	0.172	3.303E-25	Neu
ZNF44	2.2664E-29	0.27602904	0.063	0.174	3.4837E-25	Neu
CSRNP2	2.5242E-29	0.32112074	0.038	0.134	3.88E-25	Neu
ACACA	2.5486E-29	0.40884999	0.037	0.131	3.9174E-25	Neu
PTPN121	2.5683E-29	0.25927162	0.525	0.908	3.9477E-25	Neu
NAA16	2.7617E-29	0.12730909	0.074	0.19	4.245E-25	Neu
SPATA2L1	2.9345E-29	0.4162507	0.037	0.131	4.5107E-25	Neu
ARL5B1	2.9672E-29	0.59594093	0.031	0.122	4.5609E-25	Neu
TCEAL9	3.1533E-29	0.1851664	0.028	0.117	4.8469E-25	Neu
CDADC1	3.4068E-29	0.12694183	0.042	0.14	5.2366E-25	Neu

KIAA1328	3.757E-29	0.19207416	0.054	0.158	5.7749E-25	Neu
AKR1E22	4.4086E-29	0.45837715	0.063	0.172	6.7765E-25	Neu
ZNF75D	4.6799E-29	0.31784797	0.051	0.155	7.1935E-25	Neu
WDR251	4.7338E-29	0.35429503	0.038	0.132	7.2764E-25	Neu
ZNF549	5.3837E-29	0.71369622	0.068	0.181	8.2752E-25	Neu
CUL71	5.5854E-29	0.28559377	0.031	0.123	8.5853E-25	Neu
CBX71	6.5092E-29	0.39306567	0.062	0.171	1.0005E-24	Neu
ZFP28	6.9495E-29	0.12003269	0.033	0.124	1.0682E-24	Neu
UPF3B1	8.9645E-29	0.51513917	0.043	0.14	1.3779E-24	Neu
TRMT13	9.4041E-29	0.47516617	0.056	0.161	1.4455E-24	Neu
NEURL4	9.5784E-29	0.19929614	0.039	0.133	1.4723E-24	Neu
ZNF480	1.0429E-28	0.52705221	0.041	0.137	1.603E-24	Neu
IPCEF1	1.1546E-28	0.20503116	0.055	0.16	1.7747E-24	Neu
ARHGEF121	1.1914E-28	0.15482533	0.058	0.165	1.8313E-24	Neu
SIPA1L12	1.2011E-28	0.80217031	0.044	0.143	1.8463E-24	Neu
CAMSAP31	1.2202E-28	0.24907957	0.028	0.116	1.8755E-24	Neu
RYK2	1.2466E-28	0.39288472	0.067	0.179	1.9161E-24	Neu
TDG1	1.2633E-28	0.35709711	0.043	0.14	1.9418E-24	Neu
NAV13	1.3335E-28	0.23691801	0.052	0.155	2.0498E-24	Neu
KIAA1586	1.5794E-28	0.32046528	0.043	0.14	2.4277E-24	Neu
C14orf931	1.592E-28	0.15382608	0.039	0.134	2.4471E-24	Neu
TMEM1581	1.7395E-28	0.3199041	0.022	0.106	2.6738E-24	Neu
SZT2	1.7973E-28	0.36585087	0.049	0.15	2.7626E-24	Neu
TANC21	1.8661E-28	0.43350358	0.468	0.844	2.8684E-24	Neu
VAV33	2.0274E-28	0.49149329	0.076	0.194	3.1164E-24	Neu
CCDC174	2.2242E-28	0.33799676	0.052	0.155	3.4188E-24	Neu
TAF1A	2.2254E-28	0.19964749	0.034	0.125	3.4206E-24	Neu
DOCK11	2.9416E-28	0.18717834	0.537	0.926	4.5215E-24	Neu
ZNF261	3.1921E-28	0.61954011	0.067	0.176	4.9066E-24	Neu
OFD12	3.7807E-28	0.32912936	0.057	0.161	5.8113E-24	Neu
KNTC1	3.9336E-28	0.31099711	0.044	0.141	6.0463E-24	Neu
ZNF266	3.9985E-28	0.55517967	0.075	0.189	6.1461E-24	Neu
PTGS2	4.1522E-28	2.26627695	0.084	0.205	6.3823E-24	Neu
ZNF319	4.1618E-28	0.14788818	0.026	0.111	6.3972E-24	Neu
DYNLRB1	5.3132E-28	0.14952422	0.547	0.912	8.1669E-24	Neu
TEF	5.7905E-28	0.32442839	0.058	0.162	8.9006E-24	Neu
SCAPER	5.8756E-28	0.26685328	0.051	0.152	9.0314E-24	Neu
CCDC1492	6.1024E-28	0.10927375	0.037	0.129	9.3801E-24	Neu
SNRPA1	6.3832E-28	0.26936734	0.05	0.15	9.8116E-24	Neu
ANKRD61	6.782E-28	0.46044279	0.044	0.14	1.0425E-23	Neu
M1AP1	7.4717E-28	0.28096059	0.049	0.148	1.1485E-23	Neu
ZNF776	7.5359E-28	0.46747067	0.055	0.158	1.1583E-23	Neu
CEP952	7.9961E-28	0.50803243	0.067	0.176	1.2291E-23	Neu
C1orf1121	8.1814E-28	0.52239923	0.046	0.143	1.2576E-23	Neu
GTPBP31	8.33E-28	0.51609017	0.062	0.167	1.2804E-23	Neu
ASMTL2	8.6523E-28	0.23325459	0.042	0.136	1.33E-23	Neu
GAPT1	9.0731E-28	0.26394027	0.074	0.187	1.3946E-23	Neu
GNPDA21	9.3648E-28	0.25960084	0.039	0.133	1.4395E-23	Neu
DHDH1	1.042E-27	0.17767006	0.026	0.11	1.6017E-23	Neu
LYRM4	1.0708E-27	0.15305064	0.046	0.143	1.6459E-23	Neu
IQCK1	1.0762E-27	0.2885692	0.026	0.111	1.6543E-23	Neu
DAAM12	1.1204E-27	0.46246625	0.05	0.15	1.7221E-23	Neu

OSM2	1.1977E-27	0.68529602	0.221	0.399	1.841E-23	Neu
ALDH4A1	1.2321E-27	0.19817739	0.025	0.109	1.8939E-23	Neu
ENGASE1	1.237E-27	0.43985397	0.052	0.152	1.9014E-23	Neu
CXCR21	1.3728E-27	1.51204165	0.054	0.156	2.1101E-23	Neu
HOMER22	1.6179E-27	0.25362339	0.033	0.121	2.4869E-23	Neu
ZNF7081	1.6432E-27	0.74524186	0.085	0.204	2.5257E-23	Neu
CARD161	1.6544E-27	0.26414963	0.035	0.125	2.543E-23	Neu
PPARA1	1.7503E-27	0.52011102	0.044	0.139	2.6904E-23	Neu
PIGL	2.2673E-27	0.3588614	0.075	0.186	3.4851E-23	Neu
ZFP11	2.373E-27	0.3332478	0.045	0.141	3.6476E-23	Neu
HOXB71	2.4016E-27	0.32527977	0.045	0.14	3.6915E-23	Neu
TMEM143	2.4329E-27	0.33583652	0.035	0.124	3.7397E-23	Neu
LRWD1	2.6117E-27	0.82436999	0.032	0.119	4.0144E-23	Neu
USP311	2.6788E-27	0.39624177	0.026	0.11	4.1176E-23	Neu
TUBG22	2.9525E-27	0.36780255	0.048	0.145	4.5383E-23	Neu
BTN2A1	3.0157E-27	0.80282691	0.062	0.168	4.6354E-23	Neu
TTPAL	3.0737E-27	0.51399173	0.044	0.14	4.7247E-23	Neu
AMIGO21	3.1406E-27	0.2342352	0.508	0.889	4.8273E-23	Neu
SLC24A43	3.2841E-27	0.19308105	0.068	0.175	5.048E-23	Neu
GPHN1	3.3171E-27	0.49572283	0.06	0.164	5.0988E-23	Neu
NUBPL	3.4551E-27	0.33062017	0.046	0.142	5.3108E-23	Neu
NSD2	3.6122E-27	0.42124677	0.054	0.155	5.5523E-23	Neu
ZMAT1	3.6171E-27	0.65546667	0.09	0.21	5.5599E-23	Neu
QRSL11	3.6209E-27	0.22952551	0.036	0.126	5.5656E-23	Neu
NR2C1	3.8131E-27	0.66737084	0.055	0.156	5.861E-23	Neu
ZNF6881	3.8439E-27	0.14790016	0.04	0.132	5.9085E-23	Neu
SATB21	3.868E-27	0.21915589	0.046	0.142	5.9454E-23	Neu
ZNF624	3.9358E-27	0.32295223	0.049	0.146	6.0497E-23	Neu
OXSM1	4.0685E-27	0.55350019	0.044	0.138	6.2537E-23	Neu
BAG21	4.6082E-27	0.34550626	0.044	0.138	7.0833E-23	Neu
C3orf62	5.2268E-27	0.66737503	0.042	0.135	8.0341E-23	Neu
LYSMD4	5.2715E-27	0.61228581	0.055	0.156	8.1028E-23	Neu
EHHADH2	5.2966E-27	0.13483872	0.027	0.111	8.1414E-23	Neu
MTRES11	5.4462E-27	0.18642363	0.053	0.153	8.3714E-23	Neu
C2CD3	6.2677E-27	0.61477544	0.063	0.168	9.6341E-23	Neu
G2E3	6.402E-27	0.19802777	0.052	0.151	9.8405E-23	Neu
RHBDD21	6.6313E-27	0.12857168	0.563	0.915	1.0193E-22	Neu
XRRA11	7.0119E-27	0.37241481	0.043	0.135	1.0778E-22	Neu
FAM76B	7.4008E-27	0.5420316	0.073	0.184	1.1376E-22	Neu
YES11	7.6304E-27	0.55634925	0.082	0.197	1.1729E-22	Neu
PIGB	8.7331E-27	0.43029886	0.05	0.146	1.3424E-22	Neu
NREP4	9.3511E-27	0.32981823	0.038	0.127	1.4374E-22	Neu
ZNF573	1.034E-26	0.28233732	0.06	0.162	1.5893E-22	Neu
JRKL1	1.1334E-26	0.32233855	0.048	0.143	1.7421E-22	Neu
LSM31	1.31E-26	0.38901559	0.042	0.133	2.0136E-22	Neu
STEAP43	1.5116E-26	5.43541116	0.016	0.003	2.3234E-22	Neu
ZNF6771	1.5563E-26	0.27990184	0.034	0.121	2.3922E-22	Neu
ALDH7A1	1.8687E-26	0.24438406	0.025	0.105	2.8724E-22	Neu
BORA1	1.9895E-26	0.30423778	0.035	0.122	3.0581E-22	Neu
ORC51	2.0483E-26	0.4603264	0.044	0.137	3.1485E-22	Neu
ZNF5661	2.1188E-26	0.38132559	0.05	0.145	3.2568E-22	Neu
HOMER11	2.1644E-26	0.62584717	0.051	0.149	3.3269E-22	Neu

ZNF823	2.2573E-26	0.20916512	0.052	0.15	3.4698E-22	Neu
ANKMY1	2.321E-26	0.64240838	0.067	0.172	3.5676E-22	Neu
TDRD91	2.4228E-26	0.27883553	0.022	0.101	3.7241E-22	Neu
RAP1GAP2	2.4428E-26	0.76044506	0.039	0.128	3.7548E-22	Neu
TRIB31	2.448E-26	0.1154691	0.03	0.113	3.7628E-22	Neu
CARS	2.4979E-26	0.3152771	0.034	0.119	3.8396E-22	Neu
SEMA4B3	2.551E-26	0.44030461	0.026	0.107	3.9212E-22	Neu
CDKN2AIPNL1	2.7167E-26	0.30683203	0.035	0.121	4.1758E-22	Neu
SKP2	3.0993E-26	0.19873414	0.041	0.131	4.7639E-22	Neu
ZSCAN23	3.3899E-26	0.43430116	0.046	0.138	5.2106E-22	Neu
PGBD2	3.4204E-26	0.35611485	0.05	0.146	5.2575E-22	Neu
NBEAL11	3.4842E-26	0.18681905	0.044	0.136	5.3556E-22	Neu
DENND33	3.5942E-26	1.93810137	0.12	0.255	5.5246E-22	Neu
KIF3A1	3.6336E-26	0.22235342	0.043	0.135	5.5851E-22	Neu
CHST71	4.2462E-26	1.17158232	0.025	0.104	6.5269E-22	Neu
HLX1	4.4445E-26	0.5844956	0.06	0.161	6.8316E-22	Neu
AFG1L1	4.6568E-26	0.29006517	0.032	0.116	7.1579E-22	Neu
TRMT111	4.8525E-26	0.18019914	0.043	0.134	7.4588E-22	Neu
FBXO31	5.8214E-26	0.17938116	0.035	0.121	8.9481E-22	Neu
C7orf311	5.8559E-26	0.15565921	0.05	0.145	9.0011E-22	Neu
POLG21	5.9801E-26	0.28324543	0.059	0.158	9.192E-22	Neu
PI3	6.0065E-26	7.99962297	0.012	0.002	9.2325E-22	Neu
CYTIP1	6.7409E-26	0.35713523	0.489	0.853	1.0361E-21	Neu
LRRK23	8.4236E-26	1.37232017	0.101	0.225	1.2948E-21	Neu
FZD51	8.798E-26	0.38745552	0.031	0.114	1.3523E-21	Neu
USP42	9.0256E-26	0.2294893	0.034	0.119	1.3873E-21	Neu
SNN1	9.664E-26	0.95870117	0.033	0.117	1.4855E-21	Neu
SIGLEC104	9.7376E-26	0.32758444	0.016	0.089	1.4968E-21	Neu
RALGAPA11	9.8604E-26	0.30238791	0.053	0.149	1.5156E-21	Neu
IER33	9.903E-26	1.57945414	0.166	0.327	1.5222E-21	Neu
SPICE1	1.0037E-25	0.43047804	0.047	0.14	1.5428E-21	Neu
FBF11	1.0287E-25	0.34480551	0.044	0.134	1.5811E-21	Neu
FAM133B1	1.0397E-25	0.5629089	0.062	0.162	1.5981E-21	Neu
WDR531	1.042E-25	0.35735405	0.034	0.119	1.6017E-21	Neu
NDUFAF6	1.044E-25	0.532594	0.046	0.137	1.6048E-21	Neu
PPM1H	1.05E-25	0.45784436	0.029	0.111	1.614E-21	Neu
C6orf226	1.0717E-25	0.22039628	0.042	0.13	1.6472E-21	Neu
GPX7	1.092E-25	0.62458878	0.043	0.134	1.6785E-21	Neu
TRIM3	1.0988E-25	0.23005869	0.039	0.126	1.689E-21	Neu
CLYBL	1.1185E-25	0.43807588	0.047	0.139	1.7192E-21	Neu
GPR1601	1.2088E-25	0.44958273	0.066	0.169	1.8581E-21	Neu
ZNF335	1.291E-25	0.4562519	0.042	0.131	1.9844E-21	Neu
TMEM116	1.3168E-25	0.20861311	0.038	0.125	2.0241E-21	Neu
AC067752.11	1.3574E-25	0.18959251	0.039	0.126	2.0864E-21	Neu
NUAK21	1.4331E-25	0.65154236	0.04	0.127	2.2029E-21	Neu
RHOD1	1.5491E-25	0.43056411	0.028	0.109	2.3811E-21	Neu
NDST22	1.7018E-25	0.35203295	0.029	0.11	2.6158E-21	Neu
ALDH1A21	1.7549E-25	0.18804135	0.095	0.207	2.6974E-21	Neu
GDAP12	1.7941E-25	0.24764944	0.037	0.123	2.7578E-21	Neu
ABCC43	1.8405E-25	0.2167975	0.038	0.124	2.829E-21	Neu
ZSCAN30	1.8505E-25	0.19983119	0.031	0.113	2.8444E-21	Neu
AP3M2	1.9201E-25	0.42971527	0.044	0.135	2.9514E-21	Neu

ZBTB24	1.952E-25	0.22547469	0.054	0.15	3.0004E-21	Neu
SERAC11	2.0586E-25	0.56924712	0.044	0.135	3.1643E-21	Neu
SLC17A93	2.1385E-25	0.60503415	0.047	0.138	3.2871E-21	Neu
ZNF351	2.1731E-25	0.48863858	0.027	0.106	3.3402E-21	Neu
TBCE1	2.2453E-25	0.40284176	0.036	0.121	3.4513E-21	Neu
ZNF2271	2.2911E-25	0.48655334	0.037	0.123	3.5217E-21	Neu
MTUS11	2.3735E-25	0.24369207	0.021	0.097	3.6483E-21	Neu
CA13	2.78E-25	0.34644487	0.048	0.141	4.2731E-21	Neu
SETD92	2.8199E-25	0.19677126	0.056	0.153	4.3344E-21	Neu
TTLL11	2.8641E-25	0.24154008	0.023	0.099	4.4024E-21	Neu
ZHX2	2.9505E-25	0.28331419	0.05	0.142	4.5353E-21	Neu
EMSY	2.9628E-25	0.4652428	0.067	0.171	4.5542E-21	Neu
RBIS	3.1018E-25	0.31586063	0.034	0.118	4.7678E-21	Neu
STRADB	3.256E-25	0.60615886	0.042	0.13	5.0048E-21	Neu
CYP2R1	3.2705E-25	0.42870833	0.06	0.158	5.0271E-21	Neu
VPS13A1	3.6924E-25	0.26442938	0.041	0.128	5.6756E-21	Neu
RUNX12	4.2093E-25	0.17094707	0.586	0.941	6.4701E-21	Neu
ZHX3	5.0899E-25	0.49870705	0.031	0.112	7.8237E-21	Neu
FBXL18	5.1166E-25	0.13994079	0.029	0.109	7.8647E-21	Neu
TIGD21	5.3622E-25	0.25860078	0.032	0.113	8.2422E-21	Neu
TMEM223	5.4395E-25	0.31700953	0.029	0.109	8.3611E-21	Neu
NANP1	5.6378E-25	0.1502855	0.03	0.11	8.6659E-21	Neu
CLEC4D1	6.8665E-25	0.6530934	0.049	0.14	1.0555E-20	Neu
TTC38	7.4046E-25	0.11442925	0.027	0.105	1.1382E-20	Neu
ZNF2601	7.6575E-25	0.49707029	0.056	0.151	1.177E-20	Neu
MARK4	8.2541E-25	0.11992159	0.019	0.091	1.2687E-20	Neu
KIAA1958	8.4716E-25	0.20899852	0.033	0.115	1.3022E-20	Neu
SMG11	9.6862E-25	0.81952405	0.054	0.147	1.4889E-20	Neu
CCNJ	9.6941E-25	0.44398233	0.051	0.144	1.4901E-20	Neu
GTF2IRD12	1.0578E-24	0.13165762	0.023	0.098	1.626E-20	Neu
THBS31	1.1285E-24	0.440232	0.038	0.122	1.7346E-20	Neu
GORAB1	1.1658E-24	0.34004147	0.068	0.17	1.792E-20	Neu
KIAA0753	1.1991E-24	0.29340165	0.034	0.116	1.8431E-20	Neu
ZNF619	1.2017E-24	0.14826546	0.038	0.122	1.8471E-20	Neu
BBS51	1.223E-24	0.16083716	0.031	0.111	1.8799E-20	Neu
SMIM131	1.2318E-24	0.28389655	0.039	0.124	1.8934E-20	Neu
ZNF606	1.266E-24	0.51087835	0.038	0.122	1.946E-20	Neu
THNSL21	1.3658E-24	0.34972392	0.03	0.11	2.0993E-20	Neu
DPY19L3	1.458E-24	0.41176387	0.039	0.123	2.2411E-20	Neu
C2orf76	1.6232E-24	0.15854155	0.027	0.104	2.495E-20	Neu
ZNF383	1.786E-24	0.6783295	0.059	0.155	2.7453E-20	Neu
CCDC88A2	1.8154E-24	0.33617807	0.529	0.905	2.7905E-20	Neu
MFSD4B2	1.8165E-24	0.69404936	0.056	0.15	2.7921E-20	Neu
ANKRA21	2.0225E-24	0.56582104	0.055	0.149	3.1088E-20	Neu
CDC42EP13	2.0275E-24	0.11906236	0.026	0.101	3.1164E-20	Neu
KBTBD111	2.0584E-24	0.12811384	0.038	0.121	3.164E-20	Neu
RPH3AL2	2.2237E-24	0.36903717	0.031	0.11	3.4181E-20	Neu
ATP5PO2	2.2935E-24	0.31126732	0.042	0.128	3.5253E-20	Neu
MOSMO	2.2994E-24	0.40023902	0.052	0.145	3.5345E-20	Neu
CHI3L21	2.3283E-24	0.20149345	0.034	0.114	3.5788E-20	Neu
CAB39L1	2.4703E-24	0.49251524	0.025	0.101	3.7971E-20	Neu
DDX57	2.8257E-24	0.20668826	0.582	0.945	4.3433E-20	Neu

EZH21	3.0157E-24	0.35914437	0.035	0.115	4.6355E-20	Neu
CNTLN	3.0393E-24	0.8788391	0.092	0.206	4.6718E-20	Neu
RRAGB	3.4747E-24	0.66105428	0.042	0.127	5.341E-20	Neu
BBS7	3.5242E-24	0.37387576	0.042	0.127	5.4171E-20	Neu
F8	3.8558E-24	0.41338499	0.029	0.107	5.9267E-20	Neu
BCS1L	3.865E-24	0.1235463	0.03	0.107	5.941E-20	Neu
TUBD11	4.4453E-24	0.40311627	0.056	0.15	6.8329E-20	Neu
ZNF558	4.7291E-24	0.42557145	0.041	0.126	7.269E-20	Neu
AK1	5.2567E-24	0.6380805	0.114	0.234	8.08E-20	Neu
ZNF6751	5.7511E-24	0.38067571	0.029	0.105	8.8399E-20	Neu
RWDD2B1	5.8125E-24	0.10627226	0.041	0.125	8.9345E-20	Neu
POC52	6.063E-24	0.24313765	0.042	0.126	9.3194E-20	Neu
ZBTB8A	6.1518E-24	0.40844887	0.031	0.11	9.4559E-20	Neu
ZNF254	6.3728E-24	0.47481401	0.071	0.172	9.7957E-20	Neu
SMIM8	6.6783E-24	0.30606813	0.035	0.116	1.0265E-19	Neu
AHCTF1	7.2008E-24	0.43952655	0.043	0.129	1.1068E-19	Neu
PPP1R12B2	7.2043E-24	0.84973534	0.054	0.144	1.1074E-19	Neu
CHRNB1	8.9452E-24	0.18686075	0.029	0.105	1.375E-19	Neu
ZNF182	9.4843E-24	0.10806076	0.046	0.133	1.4578E-19	Neu
MSH5	9.6003E-24	0.63861697	0.049	0.137	1.4757E-19	Neu
PPP1R13L1	1.0371E-23	0.20908974	0.029	0.105	1.5941E-19	Neu
PRAF2	1.0773E-23	0.19143978	0.035	0.115	1.6558E-19	Neu
IRS21	1.0902E-23	0.21007129	0.572	0.908	1.6758E-19	Neu
WDR831	1.1058E-23	0.3088002	0.032	0.11	1.6998E-19	Neu
RNF2151	1.1991E-23	0.14214049	0.028	0.103	1.8431E-19	Neu
ZNF234	1.2261E-23	0.51307977	0.048	0.135	1.8846E-19	Neu
EHBP1L13	1.2376E-23	0.19922773	0.604	0.954	1.9023E-19	Neu
ACCS	1.6379E-23	0.35353434	0.042	0.125	2.5176E-19	Neu
FNDC3B1	1.6876E-23	0.51017323	0.535	0.903	2.594E-19	Neu
CHD34	1.7821E-23	0.13542627	0.032	0.109	2.7392E-19	Neu
EXD3	2.3563E-23	0.41137961	0.041	0.123	3.6218E-19	Neu
ARHGAP453	2.4192E-23	0.30836345	0.062	0.155	3.7186E-19	Neu
TRIM37	2.4795E-23	0.24325427	0.033	0.111	3.8112E-19	Neu
TLR64	2.5203E-23	0.73254602	0.028	0.103	3.8739E-19	Neu
APBB32	2.5245E-23	1.15579487	0.079	0.182	3.8804E-19	Neu
ANGEL2	2.5793E-23	0.51151892	0.051	0.139	3.9646E-19	Neu
KPNA5	2.9349E-23	0.31359261	0.053	0.142	4.5112E-19	Neu
ABRAXAS11	2.9962E-23	0.12539209	0.03	0.105	4.6055E-19	Neu
CSAD	3.0336E-23	0.8719726	0.061	0.155	4.6629E-19	Neu
QSOX21	3.108E-23	0.15676202	0.042	0.124	4.7773E-19	Neu
ESR12	3.1625E-23	0.10680738	0.021	0.091	4.8611E-19	Neu
PKP21	3.2604E-23	0.3305869	0.045	0.129	5.0115E-19	Neu
MRM3	3.28E-23	0.26801081	0.027	0.102	5.0417E-19	Neu
EXT11	3.6042E-23	0.55850479	0.047	0.133	5.54E-19	Neu
WDR5B	4.174E-23	0.16467698	0.026	0.099	6.4159E-19	Neu
TSNARE1	4.7861E-23	0.23714015	0.031	0.107	7.3567E-19	Neu
PARP2	5.0024E-23	0.43152871	0.03	0.106	7.6891E-19	Neu
EPS8L1	5.5916E-23	0.54442549	0.043	0.127	8.5948E-19	Neu
BBIP11	5.7404E-23	0.4125672	0.046	0.13	8.8235E-19	Neu
MRFAP11	6.4566E-23	0.35053245	0.033	0.109	9.9245E-19	Neu
NAALADL1	6.8234E-23	0.46640157	0.038	0.118	1.0488E-18	Neu
STBD1	6.918E-23	0.32255405	0.035	0.113	1.0634E-18	Neu

C20orf96	7.0784E-23	0.13810372	0.032	0.108	1.088E-18	Neu
TAF5	7.1422E-23	0.14292192	0.032	0.108	1.0978E-18	Neu
ZNF5291	7.6959E-23	0.36255527	0.046	0.129	1.1829E-18	Neu
MRNIP	8.3668E-23	0.13353385	0.062	0.153	1.2861E-18	Neu
FADS3	8.8656E-23	0.23318541	0.028	0.101	1.3627E-18	Neu
GMCL11	9.5935E-23	0.27233166	0.044	0.127	1.4746E-18	Neu
FOSB3	1.0903E-22	2.01586698	0.082	0.185	1.6759E-18	Neu
PRKN1	1.329E-22	0.26986151	0.035	0.113	2.0428E-18	Neu
ICA12	1.6371E-22	0.30314705	0.019	0.086	2.5164E-18	Neu
VGLL31	1.6499E-22	0.31156366	0.138	0.264	2.536E-18	Neu
ZNF324	1.9343E-22	0.13818988	0.025	0.096	2.9732E-18	Neu
PRIM21	2.0492E-22	0.54161469	0.06	0.152	3.1498E-18	Neu
PCGF21	2.1187E-22	0.19696598	0.025	0.095	3.2567E-18	Neu
CABP41	2.1596E-22	0.15213754	0.031	0.106	3.3195E-18	Neu
PRRG11	2.242E-22	0.11064145	0.034	0.11	3.4462E-18	Neu
DNAJC91	2.4042E-22	0.31858044	0.029	0.102	3.6956E-18	Neu
GPBAR11	2.5503E-22	0.41932628	0.022	0.09	3.9201E-18	Neu
DOLPP1	2.7638E-22	0.50484895	0.028	0.1	4.2483E-18	Neu
SLF1	2.7696E-22	0.20477519	0.045	0.127	4.2571E-18	Neu
RGS24	3.099E-22	1.06545618	0.289	0.494	4.7634E-18	Neu
LAT	3.1335E-22	0.16681588	0.067	0.159	4.8166E-18	Neu
RNPC32	3.3612E-22	0.50095227	0.069	0.163	5.1666E-18	Neu
CCDC71	3.5442E-22	0.12272031	0.044	0.126	5.4477E-18	Neu
WDPCP1	3.5602E-22	0.21644932	0.033	0.108	5.4725E-18	Neu
MT-ND32	3.565E-22	0.36515984	0.26	0.442	5.4798E-18	Neu
GPATCH12	3.6832E-22	0.13752441	0.038	0.115	5.6615E-18	Neu
MTRF1L1	3.9052E-22	0.12179167	0.042	0.122	6.0026E-18	Neu
SPATA33	3.9483E-22	0.36594682	0.041	0.12	6.069E-18	Neu
CABLES21	3.9859E-22	0.33360735	0.023	0.091	6.1268E-18	Neu
IBA571	5.0138E-22	0.33750906	0.023	0.092	7.7067E-18	Neu
DGKD3	5.7649E-22	0.38732561	0.036	0.111	8.8612E-18	Neu
CEACAM1	5.8281E-22	0.75038717	0.042	0.121	8.9583E-18	Neu
HARBI1	6.1318E-22	0.54455843	0.019	0.085	9.4252E-18	Neu
B3GNTL1	6.1335E-22	0.44404498	0.039	0.116	9.4278E-18	Neu
KCTD6	6.168E-22	0.14061774	0.027	0.098	9.4808E-18	Neu
YOD1	6.2382E-22	0.16491154	0.023	0.091	9.5887E-18	Neu
ZNF253	7.5406E-22	0.24113625	0.033	0.107	1.1591E-17	Neu
HINFP	7.5906E-22	0.41020471	0.031	0.104	1.1668E-17	Neu
SLC45A31	1.0563E-21	0.68075294	0.018	0.083	1.6237E-17	Neu
ZNF586	1.0935E-21	0.75777453	0.031	0.104	1.6808E-17	Neu
C2CD21	1.1069E-21	0.30739634	0.041	0.119	1.7014E-17	Neu
PRMT51	1.1417E-21	0.32221466	0.025	0.094	1.7549E-17	Neu
TMEM120B	1.1517E-21	0.3211392	0.03	0.101	1.7703E-17	Neu
NFIX	1.1898E-21	0.28124785	0.029	0.099	1.8289E-17	Neu
ATG10	1.2385E-21	0.56065565	0.046	0.127	1.9037E-17	Neu
EIF4EBP2	1.2828E-21	0.17863098	0.565	0.924	1.9718E-17	Neu
FGF101	1.3363E-21	0.23642714	0.064	0.153	2.0541E-17	Neu
DNAL1	1.4289E-21	0.35123822	0.034	0.108	2.1963E-17	Neu
TBC1D301	1.5342E-21	0.40241093	0.029	0.099	2.3582E-17	Neu
KIZ1	1.5946E-21	0.65017881	0.067	0.159	2.4511E-17	Neu
ECE13	1.6084E-21	1.08614481	0.041	0.118	2.4723E-17	Neu
HIKESHI1	1.6607E-21	0.25796232	0.03	0.102	2.5526E-17	Neu

TMEM52B1	1.7698E-21	0.10030308	0.023	0.09	2.7203E-17	Neu
CACFD12	1.855E-21	0.34686755	0.026	0.094	2.8513E-17	Neu
KCNC4	1.9493E-21	0.23211473	0.03	0.102	2.9963E-17	Neu
ZNF614	1.9912E-21	0.17008362	0.027	0.097	3.0607E-17	Neu
TTC12	1.9987E-21	0.60470124	0.036	0.111	3.0722E-17	Neu
MLH31	2.0328E-21	0.30999687	0.045	0.125	3.1247E-17	Neu
ATP6V0D21	2.0401E-21	0.18711189	0.017	0.08	3.1359E-17	Neu
PLA2G4C2	2.1881E-21	0.39645669	0.026	0.095	3.3633E-17	Neu
MS4A71	2.235E-21	0.26597333	0.867	0.974	3.4355E-17	Neu
FMO41	2.2446E-21	0.12040029	0.026	0.095	3.4502E-17	Neu
PRDM10	2.3011E-21	0.31866058	0.037	0.111	3.537E-17	Neu
ZNF862	2.3693E-21	0.52343328	0.031	0.103	3.6419E-17	Neu
ZNF17	2.4021E-21	0.1899764	0.029	0.099	3.6923E-17	Neu
TESK21	2.7977E-21	0.78416001	0.039	0.116	4.3004E-17	Neu
ZSWIM3	2.8258E-21	0.17800691	0.029	0.098	4.3435E-17	Neu
REEP61	3.0875E-21	0.40440454	0.033	0.106	4.7458E-17	Neu
DTX2	3.2917E-21	0.52344854	0.027	0.095	5.0596E-17	Neu
DGKG1	3.3362E-21	0.3680141	0.017	0.078	5.128E-17	Neu
CXCL8	3.3795E-21	5.50126799	0.158	0.103	5.1947E-17	Neu
CEP2951	3.504E-21	0.582341	0.039	0.114	5.3861E-17	Neu
ACVR2B	3.8646E-21	0.37528736	0.041	0.118	5.9403E-17	Neu
PPP2R1A1	3.9243E-21	0.54681351	0.034	0.106	6.0321E-17	Neu
ACSM31	4.2213E-21	0.38265213	0.034	0.107	6.4886E-17	Neu
CAST	4.2685E-21	0.19049342	0.576	0.935	6.5611E-17	Neu
ZNF5323	4.5235E-21	0.29235429	0.044	0.123	6.9531E-17	Neu
PLEKHA8	4.6599E-21	0.49627182	0.044	0.123	7.1627E-17	Neu
PUS10	4.8688E-21	0.5206489	0.034	0.106	7.4839E-17	Neu
ZNF569	4.9449E-21	0.38210952	0.025	0.091	7.6008E-17	Neu
PLB11	4.9792E-21	0.76562193	0.019	0.083	7.6536E-17	Neu
SWT1	5.038E-21	0.41037192	0.033	0.104	7.7439E-17	Neu
ADAT2	5.23E-21	0.25751879	0.034	0.106	8.0391E-17	Neu
C2orf421	6.1133E-21	0.31692861	0.043	0.12	9.3968E-17	Neu
RCN32	6.2051E-21	1.14119715	0.095	0.199	9.5379E-17	Neu
PRDM15	6.7624E-21	0.21749852	0.021	0.084	1.0395E-16	Neu
THAP2	7.0312E-21	0.3432696	0.03	0.099	1.0808E-16	Neu
ZNF225	7.1987E-21	0.18745324	0.031	0.102	1.1065E-16	Neu
KCNIP22	7.4197E-21	0.61543773	0.052	0.134	1.1405E-16	Neu
MRM1	7.9713E-21	0.10693275	0.022	0.086	1.2253E-16	Neu
ZNF275	8.0349E-21	0.27759592	0.023	0.089	1.2351E-16	Neu
ADAMTS151	8.071E-21	0.24704524	0.025	0.091	1.2406E-16	Neu
N6AMT11	8.1585E-21	0.26811948	0.024	0.09	1.254E-16	Neu
ZFAT2	9.7049E-21	0.14693397	0.027	0.094	1.4917E-16	Neu
CDAN11	1.0618E-20	0.35620585	0.025	0.09	1.6321E-16	Neu
MT-ND43	1.0999E-20	0.44652925	0.246	0.421	1.6906E-16	Neu
ZGRF1	1.2983E-20	0.14280935	0.022	0.086	1.9956E-16	Neu
RTN4IP11	1.3224E-20	0.23730922	0.031	0.101	2.0326E-16	Neu
UBE2V2	1.5194E-20	0.24312689	0.03	0.099	2.3355E-16	Neu
ZNF1801	1.5308E-20	0.37054067	0.03	0.098	2.353E-16	Neu
ZNF345	1.5946E-20	0.1278924	0.035	0.106	2.451E-16	Neu
TACR21	1.6128E-20	0.72722164	0.021	0.084	2.4791E-16	Neu
ZNF23	1.9317E-20	0.54612929	0.046	0.124	2.9692E-16	Neu
SLC22A42	2.0395E-20	0.88878632	0.034	0.105	3.135E-16	Neu

CCDC84	2.3462E-20	0.38430993	0.037	0.108	3.6063E-16	Neu
ZNF493	2.3568E-20	0.84025993	0.075	0.168	3.6226E-16	Neu
TAF101	2.5537E-20	0.17137013	0.568	0.929	3.9252E-16	Neu
ZNF141	2.5825E-20	0.36217276	0.046	0.124	3.9695E-16	Neu
CLEC1A1	2.7815E-20	0.13118532	0.02	0.082	4.2754E-16	Neu
HOMER32	2.9308E-20	0.15486583	0.01	0.064	4.505E-16	Neu
AK9	3.1026E-20	0.51903603	0.045	0.121	4.769E-16	Neu
NSUN61	3.3631E-20	0.54861984	0.063	0.149	5.1694E-16	Neu
IGIP	3.407E-20	0.39034413	0.049	0.128	5.2369E-16	Neu
DTD2	3.5891E-20	0.6472564	0.039	0.112	5.5168E-16	Neu
TRMT121	3.706E-20	0.23307878	0.025	0.09	5.6964E-16	Neu
BCDIN3D1	3.7726E-20	0.13962619	0.03	0.098	5.7988E-16	Neu
ZNF211	3.9162E-20	0.17913671	0.037	0.109	6.0196E-16	Neu
MED31	4.2568E-20	0.32257487	0.038	0.11	6.5432E-16	Neu
POC1B	4.3079E-20	0.40960859	0.034	0.104	6.6217E-16	Neu
ATPAF21	4.9441E-20	0.1577689	0.028	0.094	7.5995E-16	Neu
TARS	5.1849E-20	0.6139017	0.025	0.09	7.9698E-16	Neu
ENDOV1	5.2586E-20	0.35259231	0.039	0.112	8.083E-16	Neu
MBD5	6.1618E-20	0.34173469	0.028	0.094	9.4713E-16	Neu
TRDMT1	6.3401E-20	0.55614755	0.048	0.126	9.7454E-16	Neu
TLCD41	6.803E-20	0.29460688	0.044	0.119	1.0457E-15	Neu
SPAG1	7.6787E-20	0.26508287	0.022	0.084	1.1803E-15	Neu
DNMBP	7.7242E-20	0.37084701	0.03	0.096	1.1873E-15	Neu
PTCD2	7.7346E-20	0.27257615	0.027	0.093	1.1889E-15	Neu
LILRB43	8.0045E-20	0.28244976	0.013	0.069	1.2304E-15	Neu
MKS1	8.5728E-20	0.75543267	0.041	0.113	1.3177E-15	Neu
KCTD72	8.9036E-20	0.31496601	0.038	0.109	1.3686E-15	Neu
TMEM187	9.2797E-20	0.16264046	0.017	0.076	1.4264E-15	Neu
FAM149B11	9.4527E-20	0.24725759	0.031	0.098	1.453E-15	Neu
MTERF2	1.0232E-19	0.50929244	0.043	0.117	1.5728E-15	Neu
SENP72	1.0262E-19	0.67881924	0.041	0.114	1.5773E-15	Neu
RDH13	1.0267E-19	0.17447329	0.033	0.101	1.5781E-15	Neu
RAD18	1.031E-19	0.32130318	0.037	0.107	1.5847E-15	Neu
RGL31	1.0666E-19	0.43498158	0.047	0.123	1.6395E-15	Neu
KIAA0895L1	1.0767E-19	0.11497943	0.021	0.081	1.6551E-15	Neu
VCPKMT1	1.2989E-19	0.50381265	0.051	0.128	1.9965E-15	Neu
ZNF1172	1.4667E-19	0.88147328	0.067	0.153	2.2544E-15	Neu
TWNK	1.5547E-19	0.11416551	0.03	0.096	2.3898E-15	Neu
PRKAB2	1.8204E-19	0.24844327	0.02	0.08	2.7982E-15	Neu
MT-ATP64	1.8205E-19	0.27360982	0.281	0.461	2.7983E-15	Neu
STXBP4	1.8426E-19	0.48644672	0.035	0.104	2.8323E-15	Neu
GLI41	2.279E-19	0.17786102	0.039	0.11	3.503E-15	Neu
ZNF585B	2.4029E-19	0.57397372	0.026	0.09	3.6935E-15	Neu
WDR5	2.4652E-19	0.27849195	0.035	0.104	3.7893E-15	Neu
LMLN1	2.8207E-19	0.13609769	0.019	0.078	4.3358E-15	Neu
LRRC272	2.8713E-19	0.68000738	0.028	0.092	4.4135E-15	Neu
GRK22	2.9618E-19	0.19674285	0.577	0.945	4.5526E-15	Neu
LRP84	3.1455E-19	0.14799737	0.021	0.08	4.8349E-15	Neu
ICAM41	3.1632E-19	1.10050671	0.03	0.095	4.8622E-15	Neu
PSTPIP13	3.2049E-19	0.47089115	0.048	0.123	4.9263E-15	Neu
ZFP62	3.4693E-19	0.57471065	0.057	0.137	5.3327E-15	Neu
LRMP3	3.4702E-19	0.93751052	0.052	0.129	5.334E-15	Neu

FIGNL11	3.8943E-19	0.34509452	0.026	0.089	5.986E-15	Neu
HSD17B11	4.2622E-19	0.18071731	0.563	0.919	6.5514E-15	Neu
ZNF583	4.3961E-19	0.4321561	0.025	0.087	6.7572E-15	Neu
ST3GAL43	4.6463E-19	0.70178086	0.021	0.081	7.1418E-15	Neu
AMT	4.9666E-19	0.55028097	0.029	0.093	7.6341E-15	Neu
AIM21	5.1357E-19	0.42871606	0.015	0.07	7.894E-15	Neu
SETMAR	5.204E-19	0.28142081	0.024	0.085	7.9991E-15	Neu
CCDC40	5.5536E-19	0.33097928	0.03	0.094	8.5364E-15	Neu
SLC41A1	6.1042E-19	0.11963345	0.025	0.087	9.3828E-15	Neu
MINDY11	6.2392E-19	0.63545591	0.028	0.092	9.5903E-15	Neu
HDAC94	6.444E-19	0.17829702	0.021	0.079	9.9051E-15	Neu
REC81	6.4499E-19	0.95795156	0.037	0.105	9.9141E-15	Neu
PRR5L2	7.0332E-19	1.0127769	0.023	0.083	1.0811E-14	Neu
RBM4B	8.7785E-19	0.27912149	0.027	0.089	1.3493E-14	Neu
ZNF599	8.7858E-19	0.23713093	0.03	0.094	1.3505E-14	Neu
BBS91	8.9066E-19	0.79489177	0.074	0.16	1.369E-14	Neu
CNOT7	9.1333E-19	0.17530136	0.019	0.077	1.4039E-14	Neu
CXCR43	9.3086E-19	1.22447233	0.212	0.359	1.4308E-14	Neu
FGD61	9.8042E-19	0.28571011	0.023	0.083	1.507E-14	Neu
TAS2R141	1.0165E-18	0.23968085	0.04	0.109	1.5625E-14	Neu
ZNF250	1.0232E-18	0.11114231	0.019	0.076	1.5727E-14	Neu
FMNL13	1.0233E-18	0.28677479	0.589	0.949	1.5729E-14	Neu
HIVEP21	1.0796E-18	0.9064831	0.033	0.099	1.6594E-14	Neu
MYL52	1.1277E-18	0.20969018	0.025	0.085	1.7333E-14	Neu
ZNF484	1.131E-18	0.12600068	0.031	0.095	1.7384E-14	Neu
UTP14A	1.1576E-18	0.25497472	0.023	0.083	1.7794E-14	Neu
RNF241	1.2885E-18	0.91537947	0.026	0.087	1.9806E-14	Neu
CDKL1	1.417E-18	0.45242471	0.033	0.099	2.178E-14	Neu
ZNF6291	1.4219E-18	0.12632654	0.015	0.07	2.1856E-14	Neu
GNB1L	1.5012E-18	0.23772019	0.027	0.088	2.3076E-14	Neu
ZNF6001	1.6193E-18	0.56407128	0.037	0.104	2.489E-14	Neu
KCNMB14	1.651E-18	0.52063038	0.017	0.073	2.5377E-14	Neu
TGFBR2	1.7283E-18	0.14511284	0.58	0.937	2.6565E-14	Neu
ZBTB39	1.9278E-18	0.2681632	0.022	0.081	2.9632E-14	Neu
CEACAM43	1.9762E-18	0.41133879	0.023	0.083	3.0376E-14	Neu
ZFAND4	2.1538E-18	0.4503931	0.028	0.09	3.3105E-14	Neu
FRMPD11	2.2592E-18	0.27585328	0.023	0.082	3.4725E-14	Neu
NEMP2	2.2843E-18	0.21953918	0.03	0.092	3.5111E-14	Neu
DEF82	2.463E-18	0.11609347	0.014	0.067	3.7858E-14	Neu
H3F3A1	2.5689E-18	0.10927431	0.657	0.959	3.9487E-14	Neu
RBM111	2.638E-18	0.57278567	0.035	0.101	4.0548E-14	Neu
ZNF69	3.2145E-18	0.35892225	0.025	0.084	4.9411E-14	Neu
TLR52	3.6864E-18	0.42587045	0.054	0.13	5.6664E-14	Neu
CCDC134	3.8364E-18	0.1885216	0.019	0.076	5.897E-14	Neu
CCDC24	3.9857E-18	0.17005812	0.026	0.086	6.1265E-14	Neu
ZFP571	4.0398E-18	0.38923622	0.028	0.089	6.2095E-14	Neu
ZNF202	4.1502E-18	0.27249118	0.025	0.085	6.3792E-14	Neu
ZNF251	5.631E-18	0.20746425	0.031	0.093	8.6555E-14	Neu
ZNF500	5.6526E-18	0.80638614	0.026	0.085	8.6886E-14	Neu
RP9	6.0913E-18	0.33182327	0.026	0.085	9.3629E-14	Neu
USP533	6.2831E-18	0.11802412	0.026	0.086	9.6577E-14	Neu
TCF7L21	6.2911E-18	0.28198563	0.55	0.911	9.67E-14	Neu

GPR161	6.3705E-18	0.23545168	0.028	0.089	9.792E-14	Neu
ZNF5971	6.5539E-18	0.15153468	0.019	0.075	1.0074E-13	Neu
ZNF799	7.1135E-18	0.53480559	0.025	0.083	1.0934E-13	Neu
GRK4	7.2885E-18	0.62341008	0.035	0.1	1.1203E-13	Neu
FRAT11	7.4236E-18	0.74519471	0.025	0.083	1.1411E-13	Neu
CACNA2D41	7.6853E-18	0.37811479	0.026	0.085	1.1813E-13	Neu
WDR4	8.9668E-18	0.49673983	0.026	0.085	1.3783E-13	Neu
MT-CYB3	9.5286E-18	0.46217313	0.128	0.233	1.4646E-13	Neu
CFLAR3	1.0163E-17	0.66077214	0.516	0.88	1.5622E-13	Neu
EFNA41	1.045E-17	0.24417513	0.021	0.077	1.6062E-13	Neu
ZNF8361	1.1509E-17	0.28335909	0.03	0.091	1.7691E-13	Neu
TXNDC16	1.2089E-17	0.27614773	0.031	0.093	1.8582E-13	Neu
DZANK1	1.227E-17	0.20286321	0.017	0.071	1.886E-13	Neu
ZNF439	1.3236E-17	0.32663742	0.047	0.117	2.0344E-13	Neu
ZNF440	1.5951E-17	0.122691	0.02	0.075	2.4518E-13	Neu
CYB561D1	1.6255E-17	0.24861046	0.042	0.109	2.4985E-13	Neu
LMTK2	1.6673E-17	0.80132839	0.031	0.093	2.5628E-13	Neu
UNC119B	1.6876E-17	0.29451839	0.018	0.071	2.5939E-13	Neu
C11orf80	1.8454E-17	0.20221282	0.035	0.098	2.8365E-13	Neu
MICAL3	1.9006E-17	0.18358404	0.031	0.092	2.9214E-13	Neu
RTL8B1	1.94E-17	0.19824321	0.019	0.074	2.982E-13	Neu
SERPINF22	2.079E-17	0.38498498	0.026	0.084	3.1956E-13	Neu
C17orf80	2.2363E-17	0.16872625	0.031	0.092	3.4374E-13	Neu
EPB41L51	2.3813E-17	0.47845855	0.051	0.123	3.6603E-13	Neu
EEPD11	2.4582E-17	0.87577532	0.015	0.066	3.7785E-13	Neu
CD933	2.5793E-17	1.6287039	0.029	0.089	3.9646E-13	Neu
ZNF765	2.6689E-17	0.71369981	0.029	0.089	4.1024E-13	Neu
MAP1LC3C	2.8088E-17	0.5428336	0.015	0.066	4.3174E-13	Neu
FUT10	3.1089E-17	0.43094854	0.052	0.123	4.7787E-13	Neu
ZNF692	3.2029E-17	0.54530858	0.021	0.075	4.9232E-13	Neu
KIF3C	3.4921E-17	0.22101522	0.018	0.07	5.3677E-13	Neu
SUCLA21	3.6751E-17	0.49480925	0.027	0.086	5.6491E-13	Neu
S100A82	3.779E-17	0.25406761	0.565	0.847	5.8087E-13	Neu
KDM7A4	3.7977E-17	0.42016121	0.042	0.107	5.8374E-13	Neu
PCGF61	3.8436E-17	0.27850641	0.029	0.088	5.908E-13	Neu
USP49	4.7763E-17	0.27619005	0.026	0.083	7.3417E-13	Neu
CNNM2	5.7263E-17	0.24512404	0.03	0.09	8.8019E-13	Neu
ZGLP1	5.8359E-17	0.18075868	0.015	0.066	8.9703E-13	Neu
PABPC1L2	5.8561E-17	0.89519379	0.044	0.111	9.0015E-13	Neu
DNAJC18	6.7329E-17	0.85440116	0.035	0.098	1.0349E-12	Neu
EFCAB2	7.9321E-17	0.40567609	0.019	0.072	1.2192E-12	Neu
ZNF701	8.1211E-17	0.10561446	0.027	0.085	1.2483E-12	Neu
RTL8A1	8.9416E-17	0.64560513	0.022	0.076	1.3744E-12	Neu
ZSCAN311	8.9655E-17	0.43480427	0.026	0.083	1.3781E-12	Neu
CLK21	9.1925E-17	0.84661947	0.037	0.1	1.413E-12	Neu
LSM11	9.4914E-17	0.20000992	0.022	0.075	1.4589E-12	Neu
ST8SIA44	9.6945E-17	0.68848535	0.027	0.084	1.4901E-12	Neu
FHIT	1.0436E-16	0.55696853	0.026	0.083	1.6041E-12	Neu
FGGY1	1.0714E-16	0.50281186	0.016	0.066	1.6468E-12	Neu
PROCA1	1.0825E-16	0.43713661	0.042	0.106	1.6639E-12	Neu
ZNF4731	1.1409E-16	0.77155997	0.021	0.075	1.7537E-12	Neu
SEC16B1	1.1496E-16	0.66536211	0.036	0.097	1.7671E-12	Neu

OTUD3	1.1534E-16	0.56679865	0.028	0.085	1.7729E-12	Neu
TATDN1	1.2198E-16	0.49569468	0.05	0.12	1.875E-12	Neu
MT-ND4L4	1.2206E-16	0.40474913	0.172	0.291	1.8762E-12	Neu
C8orf82	1.2436E-16	0.1294309	0.025	0.081	1.9115E-12	Neu
MTFMT	1.2756E-16	0.11771476	0.024	0.079	1.9608E-12	Neu
ZNF678	1.2916E-16	0.37488229	0.03	0.088	1.9853E-12	Neu
MT-CO23	1.2973E-16	0.42317084	0.281	0.453	1.9941E-12	Neu
ZNF486	1.3042E-16	0.28819888	0.024	0.079	2.0047E-12	Neu
EIF1AX	1.3703E-16	0.47068553	0.03	0.089	2.1063E-12	Neu
RPP40	1.4391E-16	0.15143725	0.014	0.062	2.212E-12	Neu
QKI3	1.4554E-16	0.21708642	0.593	0.945	2.2371E-12	Neu
SGO2	1.498E-16	0.42387061	0.019	0.071	2.3026E-12	Neu
TGIF24	1.7523E-16	0.15229778	0.023	0.077	2.6935E-12	Neu
ARHGAP321	1.7926E-16	0.54700281	0.028	0.085	2.7553E-12	Neu
LY6G5B2	1.8499E-16	0.64604965	0.038	0.1	2.8434E-12	Neu
CATSPERG	1.8605E-16	0.22206314	0.033	0.091	2.8598E-12	Neu
ALKBH81	2.0247E-16	0.35666682	0.034	0.095	3.1121E-12	Neu
RALGPS11	2.2454E-16	0.17758608	0.034	0.094	3.4514E-12	Neu
G0S2	2.2936E-16	4.96713498	0.157	0.109	3.5255E-12	Neu
NBDY	2.3279E-16	0.80648306	0.027	0.084	3.5783E-12	Neu
PRRT41	2.3619E-16	0.23025025	0.022	0.074	3.6305E-12	Neu
IFT140	2.4246E-16	0.67978615	0.033	0.092	3.7268E-12	Neu
ARL6	2.4476E-16	0.15821293	0.025	0.08	3.7622E-12	Neu
ZNF575	2.5717E-16	0.50743658	0.026	0.082	3.953E-12	Neu
DOCK53	2.6231E-16	2.51745173	0.164	0.295	4.0319E-12	Neu
GCH12	2.7173E-16	0.34145023	0.015	0.064	4.1768E-12	Neu
ZNF382	2.7189E-16	0.25978961	0.023	0.077	4.1793E-12	Neu
ZNF79	2.7421E-16	0.14121608	0.019	0.07	4.2149E-12	Neu
DNAAF2	2.7567E-16	0.56619032	0.029	0.086	4.2373E-12	Neu
ATP8B3	2.9129E-16	0.13567487	0.023	0.076	4.4774E-12	Neu
HES61	3E-16	0.62776482	0.018	0.069	4.6113E-12	Neu
BRAF	3.532E-16	0.66907151	0.043	0.107	5.4291E-12	Neu
ZNF248	3.5355E-16	0.73964018	0.034	0.093	5.4343E-12	Neu
HELB2	3.7914E-16	0.63030168	0.051	0.118	5.8278E-12	Neu
PSTK	4.2528E-16	0.17053409	0.022	0.074	6.537E-12	Neu
DYNC2H1	4.4703E-16	0.27386573	0.026	0.081	6.8713E-12	Neu
PCOLCE	4.7366E-16	0.34807058	0.023	0.075	7.2806E-12	Neu
DZIP3	5.6743E-16	0.21606241	0.026	0.08	8.722E-12	Neu
C14orf281	5.7276E-16	0.36705625	0.026	0.081	8.8038E-12	Neu
VSIG101	6.057E-16	0.39378078	0.035	0.094	9.3102E-12	Neu
COG6	6.5604E-16	0.55325931	0.021	0.072	1.0084E-11	Neu
METTL18	7.1411E-16	0.21091756	0.027	0.082	1.0977E-11	Neu
MAPK8IP33	7.4681E-16	0.54622127	0.052	0.118	1.1479E-11	Neu
USP451	7.749E-16	0.68624995	0.039	0.101	1.1911E-11	Neu
RAD9A	7.8788E-16	0.93722974	0.024	0.077	1.211E-11	Neu
NEK111	8.6054E-16	0.39187146	0.034	0.092	1.3227E-11	Neu
CMTM21	8.9149E-16	0.38687983	0.009	0.051	1.3703E-11	Neu
NRDE2	9.3964E-16	0.52259375	0.038	0.099	1.4443E-11	Neu
DHRS131	9.5416E-16	0.42320373	0.02	0.07	1.4666E-11	Neu
PLEKHH3	1.0123E-15	0.48986339	0.011	0.055	1.556E-11	Neu
KIFC22	1.0185E-15	0.95058525	0.047	0.111	1.5655E-11	Neu
C11orf211	1.0492E-15	0.1149605	0.028	0.083	1.6127E-11	Neu

RNF1302	1.077E-15	0.12768552	0.612	0.936	1.6555E-11	Neu
MT-ND14	1.0919E-15	0.59490543	0.119	0.213	1.6783E-11	Neu
LRRC34	1.1332E-15	0.19648385	0.009	0.052	1.7419E-11	Neu
IGF1R2	1.1507E-15	1.11906378	0.03	0.086	1.7687E-11	Neu
ZNF100	1.1568E-15	0.53626449	0.025	0.078	1.7781E-11	Neu
KATNBL11	1.1733E-15	0.81717733	0.048	0.113	1.8035E-11	Neu
SULT1B11	1.2036E-15	0.42007453	0.055	0.123	1.8501E-11	Neu
COL6A31	1.2448E-15	0.28678726	0.021	0.071	1.9134E-11	Neu
HEATR5A	1.2615E-15	0.90133242	0.029	0.084	1.939E-11	Neu
OPRL12	1.2682E-15	0.48681904	0.023	0.075	1.9493E-11	Neu
ZNF780B	1.3231E-15	0.33157847	0.034	0.091	2.0338E-11	Neu
BX255925.31	1.3438E-15	0.41961791	0.023	0.075	2.0655E-11	Neu
S100A131	1.3555E-15	0.2819897	0.555	0.892	2.0835E-11	Neu
ZNF5821	1.4455E-15	0.19253517	0.021	0.071	2.2219E-11	Neu
CEP152	1.4715E-15	0.46462359	0.037	0.095	2.2619E-11	Neu
ZNF653	1.5424E-15	0.14790641	0.023	0.074	2.3708E-11	Neu
ARHGEF404	1.6468E-15	1.48103689	0.033	0.089	2.5314E-11	Neu
CCAR1	1.6583E-15	0.22710656	0.029	0.083	2.549E-11	Neu
PPP1R3F1	1.6653E-15	0.22837849	0.016	0.063	2.5597E-11	Neu
LYRM2	1.7898E-15	0.5958435	0.034	0.092	2.7511E-11	Neu
ZFP691	1.803E-15	0.68790951	0.036	0.094	2.7713E-11	Neu
EREG1	2.006E-15	0.49697082	0.127	0.224	3.0834E-11	Neu
ICA1L	2.0907E-15	0.30389083	0.021	0.071	3.2136E-11	Neu
TYW11	2.2598E-15	0.4792496	0.028	0.082	3.4736E-11	Neu
ZNF85	2.2657E-15	0.3108999	0.022	0.073	3.4826E-11	Neu
GPAT32	2.3508E-15	0.34509374	0.023	0.075	3.6134E-11	Neu
GRIN2D1	2.3589E-15	0.73005601	0.019	0.068	3.6258E-11	Neu
SPATS23	2.7067E-15	0.14463849	0.017	0.063	4.1604E-11	Neu
ACOT112	3.0779E-15	0.22070293	0.008	0.049	4.7311E-11	Neu
FKBP14	3.1322E-15	0.48048877	0.018	0.065	4.8144E-11	Neu
KLF5	3.6362E-15	0.24900856	0.021	0.069	5.5892E-11	Neu
TMEM231	3.8183E-15	0.94251422	0.022	0.071	5.869E-11	Neu
HMGN51	3.9357E-15	0.19981456	0.028	0.081	6.0495E-11	Neu
C17orf971	4.3765E-15	0.35423951	0.025	0.076	6.7271E-11	Neu
LNX2	4.9594E-15	0.49851486	0.038	0.097	7.6231E-11	Neu
OPRK11	5.0887E-15	0.25667329	0.022	0.071	7.8219E-11	Neu
BATF21	5.1248E-15	1.031593	0.017	0.063	7.8774E-11	Neu
C5	5.6369E-15	0.54311014	0.024	0.074	8.6644E-11	Neu
TGFA	5.7587E-15	0.13222857	0.013	0.057	8.8517E-11	Neu
SSC4D1	5.7954E-15	0.23813587	0.015	0.061	8.9081E-11	Neu
ZFP14	6.4048E-15	0.70998959	0.031	0.086	9.8449E-11	Neu
TUFT1	6.8525E-15	0.30270598	0.013	0.056	1.0533E-10	Neu
ZNF554	6.8964E-15	0.58168864	0.03	0.084	1.0601E-10	Neu
HYLS1	6.94E-15	0.10121066	0.017	0.063	1.0667E-10	Neu
KCNJ152	7.419E-15	5.76173363	0.017	0.004	1.1404E-10	Neu
PRPF40B	7.4891E-15	0.42609875	0.013	0.056	1.1512E-10	Neu
PIGZ1	8.4507E-15	0.80717751	0.027	0.08	1.299E-10	Neu
ARMC5	8.9815E-15	0.10037367	0.018	0.064	1.3805E-10	Neu
BTBD8	9.5008E-15	0.68954286	0.026	0.077	1.4604E-10	Neu
DNAH11	9.9992E-15	0.62553032	0.033	0.087	1.537E-10	Neu
EFCAB131	1.0424E-14	0.44481685	0.033	0.087	1.6023E-10	Neu
RMI22	1.0762E-14	0.68939323	0.021	0.068	1.6542E-10	Neu

SELL3	1.0903E-14	0.38220502	0.012	0.055	1.6759E-10	Neu
SLC2A111	1.1194E-14	0.47077646	0.026	0.077	1.7206E-10	Neu
CLDN15	1.1832E-14	0.63677204	0.039	0.097	1.8187E-10	Neu
INTU1	1.1858E-14	0.36326507	0.017	0.063	1.8227E-10	Neu
CHST153	1.2261E-14	5.54689604	0.011	0.002	1.8847E-10	Neu
FKBP113	1.3302E-14	0.95486046	0.051	0.114	2.0447E-10	Neu
CDCA4	1.4224E-14	0.15735706	0.014	0.058	2.1864E-10	Neu
MT-ND54	1.4292E-14	0.56245072	0.168	0.28	2.1968E-10	Neu
ZNF8441	1.4687E-14	0.62742272	0.025	0.075	2.2575E-10	Neu
CYB5D1	1.472E-14	1.35045533	0.012	0.054	2.2626E-10	Neu
DNHD11	1.5559E-14	0.37069629	0.03	0.083	2.3916E-10	Neu
ZNF7852	1.5809E-14	0.29414452	0.027	0.078	2.43E-10	Neu
BASP14	1.7438E-14	1.91468691	0.026	0.076	2.6805E-10	Neu
PM20D21	1.7455E-14	0.25989904	0.022	0.07	2.6831E-10	Neu
DUSP53	1.8256E-14	0.50137151	0.021	0.068	2.8061E-10	Neu
NBPF12	2.0092E-14	0.26821461	0.026	0.076	3.0884E-10	Neu
GINS2	2.0297E-14	0.35174125	0.008	0.047	3.1199E-10	Neu
CCDC171	2.1415E-14	0.98477474	0.031	0.084	3.2917E-10	Neu
THUMPD2	2.3949E-14	0.42545101	0.022	0.07	3.6812E-10	Neu
CBLN31	2.4877E-14	0.37815864	0.019	0.065	3.8239E-10	Neu
KAT2A	2.5525E-14	0.81718819	0.032	0.085	3.9235E-10	Neu
PRELID21	2.671E-14	0.17092374	0.029	0.08	4.1057E-10	Neu
CEP70	2.7585E-14	0.17766416	0.017	0.061	4.2401E-10	Neu
PAICS	2.7795E-14	0.21817065	0.018	0.063	4.2724E-10	Neu
UBA521	3.0814E-14	0.14631178	0.601	0.936	4.7364E-10	Neu
ATAD3B1	3.2072E-14	0.47274425	0.023	0.071	4.9299E-10	Neu
PDE9A1	3.2484E-14	0.64915132	0.037	0.092	4.9931E-10	Neu
LRTOMT	3.2485E-14	0.14327115	0.019	0.064	4.9933E-10	Neu
KBTBD6	3.4173E-14	0.26438307	0.019	0.064	5.2528E-10	Neu
GINS4	3.5767E-14	0.36220343	0.009	0.047	5.4977E-10	Neu
B4GALT6	3.6047E-14	0.34498187	0.033	0.085	5.5409E-10	Neu
MUSTN1	3.6622E-14	0.46778533	0.03	0.082	5.6292E-10	Neu
MT-ND24	3.8021E-14	0.52761961	0.124	0.216	5.8442E-10	Neu
PEX11G1	4.5351E-14	1.25962676	0.02	0.066	6.9708E-10	Neu
MAGOHB	4.6009E-14	0.27561868	0.019	0.064	7.072E-10	Neu
CEP191	4.6985E-14	0.15187927	0.015	0.058	7.2221E-10	Neu
CAVIN41	4.8157E-14	0.60537437	0.032	0.084	7.4022E-10	Neu
IFT81	4.939E-14	0.36864424	0.028	0.078	7.5918E-10	Neu
P2RY143	5.2001E-14	0.55584141	0.018	0.062	7.9931E-10	Neu
HPF11	5.7004E-14	0.46841512	0.032	0.084	8.7621E-10	Neu
KBTBD3	6.0703E-14	0.24912149	0.023	0.071	9.3307E-10	Neu
EDA2	6.2795E-14	0.38816204	0.034	0.086	9.6523E-10	Neu
TMEM99	6.4825E-14	0.32561047	0.015	0.057	9.9643E-10	Neu
ZNF850	6.6112E-14	0.61060915	0.018	0.063	1.0162E-09	Neu
TMEM1821	7.0251E-14	0.40620753	0.022	0.068	1.0798E-09	Neu
FBXL51	7.0822E-14	0.39867555	0.557	0.912	1.0886E-09	Neu
SPATA121	7.6408E-14	0.44542025	0.013	0.054	1.1745E-09	Neu
GRK51	7.8821E-14	0.41744832	0.036	0.09	1.2116E-09	Neu
MPZ1	7.9453E-14	0.8754351	0.022	0.069	1.2213E-09	Neu
KIF27	7.9708E-14	0.83120409	0.023	0.07	1.2252E-09	Neu
SCARF13	8.5041E-14	2.717815	0.059	0.125	1.3072E-09	Neu
ZBTB49	8.7045E-14	0.54333176	0.044	0.101	1.338E-09	Neu

GSDME2	9.1584E-14	0.5506255	0.009	0.047	1.4077E-09	Neu
RELL21	1.0342E-13	0.18433161	0.017	0.06	1.5896E-09	Neu
GINS1	1.2693E-13	0.15006775	0.009	0.047	1.951E-09	Neu
C20orf2021	1.2885E-13	0.31708442	0.013	0.053	1.9806E-09	Neu
OPHN1	1.3557E-13	0.52415654	0.021	0.066	2.0839E-09	Neu
MEF2C4	1.4085E-13	0.13961594	0.023	0.07	2.165E-09	Neu
FBXO5	1.581E-13	0.32551323	0.015	0.057	2.4302E-09	Neu
ZEB22	1.6688E-13	0.39880633	0.596	0.924	2.5652E-09	Neu
TNNT11	1.9662E-13	0.52743405	0.074	0.142	3.0222E-09	Neu
ZNF45	2.2097E-13	0.4188227	0.035	0.087	3.3965E-09	Neu
ANAPC101	2.2295E-13	0.18695097	0.021	0.066	3.4269E-09	Neu
HRH41	2.3587E-13	0.30918817	0.014	0.054	3.6255E-09	Neu
DCLRE1A1	2.7747E-13	0.49743451	0.016	0.058	4.265E-09	Neu
IMMP1L	2.8601E-13	0.45853839	0.022	0.066	4.3963E-09	Neu
RASGRP33	2.9127E-13	0.68361784	0.014	0.054	4.4772E-09	Neu
KLHDC1	3.055E-13	0.49086083	0.029	0.077	4.6959E-09	Neu
MYL6B	3.2172E-13	0.13347871	0.013	0.053	4.9451E-09	Neu
SNAI21	3.3784E-13	0.49866151	0.03	0.079	5.193E-09	Neu
AMN1	3.5517E-13	1.18960658	0.033	0.083	5.4594E-09	Neu
SHLD3	3.5562E-13	0.500815	0.019	0.062	5.4663E-09	Neu
PLPPR22	3.6228E-13	1.1559312	0.023	0.068	5.5685E-09	Neu
KCNN43	3.6308E-13	0.17756642	0.022	0.066	5.581E-09	Neu
ZNF724	3.9215E-13	0.47979964	0.019	0.062	6.0277E-09	Neu
THEM4	3.9637E-13	0.16378504	0.02	0.063	6.0926E-09	Neu
ZNF34	4.1023E-13	0.52722718	0.038	0.09	6.3057E-09	Neu
PPARG1	4.3856E-13	0.32434021	0.768	0.964	6.7411E-09	Neu
ZNF284	4.5744E-13	1.09014373	0.025	0.071	7.0312E-09	Neu
OTUB2	5.2043E-13	0.80855859	0.019	0.062	7.9995E-09	Neu
ETFBKMT1	5.2044E-13	0.76074315	0.026	0.072	7.9997E-09	Neu
FRMD81	5.2437E-13	0.80739755	0.013	0.051	8.06E-09	Neu
PASK	5.3997E-13	0.5864697	0.023	0.068	8.2998E-09	Neu
KLK1	5.5011E-13	0.16780465	0.017	0.057	8.4557E-09	Neu
DNA2	5.6732E-13	0.52006616	0.023	0.067	8.7203E-09	Neu
SERPINB11	5.7888E-13	0.14173453	0.631	0.947	8.898E-09	Neu
MUC20	5.9562E-13	0.32793962	0.034	0.083	9.1553E-09	Neu
NKX3-11	6.021E-13	0.32241564	0.019	0.062	9.2549E-09	Neu
RAB3A1	6.5568E-13	0.4487474	0.025	0.07	1.0078E-08	Neu
MAP3K212	6.9168E-13	0.18959151	0.017	0.057	1.0632E-08	Neu
ANXA31	7.5349E-13	0.39483223	0.015	0.055	1.1582E-08	Neu
SPIN3	7.679E-13	0.70193633	0.018	0.06	1.1803E-08	Neu
ASTE1	7.8789E-13	0.58440448	0.031	0.08	1.2111E-08	Neu
GPR1324	8.223E-13	0.32850819	0.019	0.06	1.264E-08	Neu
ADORA2A2	8.2413E-13	4.1794396	0.02	0.006	1.2668E-08	Neu
ZNF470	8.3303E-13	0.4058195	0.019	0.062	1.2804E-08	Neu
PAPLN2	8.368E-13	0.67265164	0.019	0.061	1.2862E-08	Neu
ZBTB26	8.5425E-13	0.42506743	0.018	0.06	1.3131E-08	Neu
PRKCB4	9.2217E-13	0.5730479	0.017	0.057	1.4175E-08	Neu
FANCM1	9.3198E-13	0.69492867	0.02	0.062	1.4325E-08	Neu
ZNF747	1.0105E-12	0.70270144	0.023	0.068	1.5533E-08	Neu
NPHP1	1.0565E-12	0.17386948	0.018	0.059	1.624E-08	Neu
CCDC15	1.0631E-12	0.28372213	0.024	0.068	1.6341E-08	Neu
CTNNAL1	1.1912E-12	0.70416505	0.015	0.054	1.8311E-08	Neu

EBLN21	1.2152E-12	0.92387912	0.037	0.088	1.868E-08	Neu
GPC43	1.3943E-12	0.41857055	0.027	0.073	2.1432E-08	Neu
FCRL11	1.3951E-12	0.77724166	0.102	0.179	2.1444E-08	Neu
IL271	1.4447E-12	0.45945099	0.008	0.042	2.2206E-08	Neu
SERPINB94	1.4701E-12	1.57525166	0.027	0.073	2.2596E-08	Neu
VILL	1.4834E-12	0.56073064	0.019	0.06	2.2802E-08	Neu
GPAM	1.5693E-12	0.65882283	0.014	0.052	2.4122E-08	Neu
EEF1AKMT11	1.6307E-12	0.3689573	0.015	0.054	2.5065E-08	Neu
ARHGEF9	1.6393E-12	0.66632092	0.026	0.071	2.5197E-08	Neu
WDR27	1.6868E-12	0.37802606	0.026	0.07	2.5928E-08	Neu
ZNF584	1.8883E-12	0.67726325	0.016	0.055	2.9025E-08	Neu
MED27	1.9282E-12	0.47792515	0.014	0.052	2.9638E-08	Neu
USP54	2.0031E-12	0.29823874	0.016	0.055	3.0789E-08	Neu
ZC3H82	2.0669E-12	0.32422516	0.025	0.069	3.177E-08	Neu
CSNK2A1	2.1291E-12	0.96897392	0.035	0.085	3.2727E-08	Neu
KIAA0825	2.29E-12	0.58160962	0.034	0.083	3.52E-08	Neu
CXADR1	2.3122E-12	0.44863975	0.021	0.063	3.554E-08	Neu
LGR41	2.3567E-12	0.23413446	0.009	0.043	3.6225E-08	Neu
NAMPT1	2.3778E-12	1.32555022	0.483	0.821	3.6549E-08	Neu
PIP4P23	2.3829E-12	0.79875193	0.014	0.051	3.6628E-08	Neu
INTS6L2	2.4151E-12	0.24115569	0.019	0.059	3.7122E-08	Neu
EFHC1	2.7153E-12	0.70656663	0.047	0.101	4.1737E-08	Neu
NEDD4L4	2.9384E-12	0.57618868	0.011	0.047	4.5166E-08	Neu
CPT1B1	2.9397E-12	0.48703224	0.03	0.075	4.5187E-08	Neu
MCL13	3.1731E-12	0.3201305	0.865	0.989	4.8774E-08	Neu
PELI23	3.3161E-12	0.4135335	0.028	0.073	5.0972E-08	Neu
BBOF1	3.5033E-12	0.45609808	0.013	0.05	5.3849E-08	Neu
ZNF6183	3.6008E-12	0.35875757	0.007	0.04	5.5348E-08	Neu
B9D1	3.674E-12	0.27489885	0.013	0.049	5.6473E-08	Neu
MTURN1	4.0597E-12	0.63989025	0.024	0.067	6.2402E-08	Neu
TPRN	4.0647E-12	0.52666026	0.017	0.056	6.2478E-08	Neu
AMER1	4.1644E-12	0.1034547	0.015	0.052	6.4011E-08	Neu
HOOK2	4.1709E-12	0.9788966	0.018	0.058	6.4111E-08	Neu
PTK61	4.1901E-12	0.49108291	0.022	0.064	6.4407E-08	Neu
LRP32	4.3221E-12	0.42954746	0.014	0.051	6.6435E-08	Neu
MBD21	4.4492E-12	0.20215794	0.64	0.958	6.8388E-08	Neu
TFAP4	4.5637E-12	0.3817281	0.015	0.052	7.0148E-08	Neu
NUDT8	4.5787E-12	0.31293616	0.014	0.051	7.038E-08	Neu
PMAIP12	4.7782E-12	0.62835536	0.009	0.042	7.3445E-08	Neu
IGF2BP31	4.9619E-12	0.2621337	0.01	0.045	7.6269E-08	Neu
KYAT11	5.2402E-12	0.69769142	0.03	0.075	8.0547E-08	Neu
WDR35	5.29E-12	0.55358042	0.016	0.054	8.1313E-08	Neu
PLA2G72	5.6144E-12	0.30117406	0.012	0.048	8.6299E-08	Neu
ZNF347	6.2002E-12	0.457755	0.015	0.052	9.5304E-08	Neu
EID31	6.2402E-12	0.63961375	0.02	0.06	9.5918E-08	Neu
LIPN2	6.4883E-12	1.52255409	0.017	0.055	9.9731E-08	Neu
TNFRSF10D1	6.5695E-12	0.76697682	0.019	0.058	1.0098E-07	Neu
ATP8A12	6.6658E-12	0.33362749	0.028	0.072	1.0246E-07	Neu
C3orf18	6.7169E-12	0.47301627	0.015	0.053	1.0325E-07	Neu
DUSP24	6.7631E-12	1.25440321	0.025	0.068	1.0396E-07	Neu
PRKAR1B	6.7687E-12	0.46038638	0.012	0.047	1.0404E-07	Neu
TARSL2	6.969E-12	0.43601223	0.019	0.058	1.0712E-07	Neu

MEGF63	7.5109E-12	0.49774092	0.009	0.042	1.1545E-07	Neu
ZNF1241	7.5997E-12	0.67001181	0.022	0.063	1.1681E-07	Neu
PIDD1	7.7484E-12	0.64489402	0.011	0.046	1.191E-07	Neu
LRRC291	7.8505E-12	0.83332847	0.024	0.066	1.2067E-07	Neu
HECTD21	9.0398E-12	0.38893839	0.019	0.059	1.3895E-07	Neu
KREMEN11	9.2298E-12	1.3062507	0.023	0.065	1.4187E-07	Neu
ZNF107	9.7898E-12	0.56902694	0.024	0.066	1.5048E-07	Neu
OR6N11	1.0647E-11	0.17261086	0.008	0.04	1.6366E-07	Neu
TYW5	1.0956E-11	0.32567207	0.021	0.061	1.6841E-07	Neu
CERCAM	1.1551E-11	0.10887037	0.009	0.041	1.7755E-07	Neu
PTBP2	1.1578E-11	0.87302275	0.027	0.071	1.7797E-07	Neu
MPZL3	1.1751E-11	0.33460704	0.02	0.059	1.8062E-07	Neu
KRBOX4	1.1973E-11	0.65905985	0.018	0.056	1.8403E-07	Neu
USP351	1.3027E-11	0.56199983	0.009	0.042	2.0023E-07	Neu
NEIL1	1.3284E-11	0.36033988	0.017	0.055	2.0419E-07	Neu
ZCWPW21	1.3344E-11	0.1212111	0.009	0.042	2.0511E-07	Neu
CLBA11	1.4352E-11	0.29511231	0.01	0.043	2.206E-07	Neu
ZNF302	1.4454E-11	0.39759725	0.022	0.062	2.2217E-07	Neu
AICDA2	1.5181E-11	0.6075362	0.013	0.047	2.3334E-07	Neu
ADGRD11	1.6161E-11	0.34261347	0.015	0.052	2.484E-07	Neu
DNAJB51	1.62E-11	0.47359112	0.01	0.044	2.4902E-07	Neu
SPATA7	1.7198E-11	0.68024041	0.025	0.067	2.6435E-07	Neu
HSH2D4	1.725E-11	1.48273157	0.022	0.062	2.6515E-07	Neu
CHAC2	1.7679E-11	0.16928672	0.013	0.048	2.7175E-07	Neu
ARNTL23	1.969E-11	0.50322942	0.007	0.038	3.0265E-07	Neu
NUMBL	2.1487E-11	0.68251144	0.017	0.053	3.3028E-07	Neu
CCDC961	2.2766E-11	0.43759689	0.013	0.048	3.4994E-07	Neu
DNAH141	2.2797E-11	0.14329895	0.01	0.042	3.5041E-07	Neu
CCDC13	2.3584E-11	0.19165903	0.015	0.051	3.6251E-07	Neu
ZNF790	2.463E-11	0.52163263	0.017	0.053	3.7859E-07	Neu
ZNF804A1	2.5006E-11	0.79658309	0.021	0.06	3.8437E-07	Neu
FAM229B	2.62E-11	0.22278052	0.011	0.044	4.0272E-07	Neu
AOC2	2.6601E-11	0.33769082	0.014	0.048	4.0888E-07	Neu
KRBA1	2.7374E-11	0.49432594	0.01	0.043	4.2076E-07	Neu
ZNF2851	2.8593E-11	0.39626113	0.013	0.048	4.395E-07	Neu
FOXM1	2.9389E-11	0.16277277	0.006	0.035	4.5175E-07	Neu
MEX3B3	3.0575E-11	0.41832106	0.015	0.05	4.6997E-07	Neu
ZNF2211	3.2679E-11	0.18891862	0.014	0.048	5.0231E-07	Neu
POGLUT2	3.3154E-11	0.12121521	0.009	0.04	5.0962E-07	Neu
DLG5	3.3794E-11	0.15739665	0.016	0.052	5.1944E-07	Neu
CCDC57	3.4115E-11	0.87799095	0.046	0.096	5.2438E-07	Neu
XK1	3.4579E-11	0.3095006	0.017	0.053	5.3151E-07	Neu
TXNRD31	3.7762E-11	0.8008919	0.019	0.056	5.8043E-07	Neu
ZNF90	4.3229E-11	0.66236426	0.013	0.046	6.6447E-07	Neu
ZBED8	4.3498E-11	0.68021722	0.012	0.045	6.6861E-07	Neu
ZNF630	4.3821E-11	0.58714985	0.023	0.062	6.7357E-07	Neu
DLX41	4.5102E-11	0.53323966	0.025	0.065	6.9326E-07	Neu
BATF1	4.6136E-11	1.20822947	0.012	0.045	7.0916E-07	Neu
CARF	4.6237E-11	0.40313154	0.033	0.077	7.1071E-07	Neu
LRRC6	4.7996E-11	0.80387998	0.026	0.066	7.3775E-07	Neu
RSRP13	5.4527E-11	0.765216	0.439	0.714	8.3814E-07	Neu
DFFB1	6.121E-11	0.93047434	0.037	0.082	9.4087E-07	Neu

MORN4	6.1793E-11	0.60936572	0.004	0.031	9.4982E-07	Neu
CENPI	6.2606E-11	0.19080147	0.015	0.049	9.6231E-07	Neu
PLA2R11	6.5503E-11	0.26910534	0.008	0.038	1.0068E-06	Neu
PRR3	7.9135E-11	0.5825124	0.023	0.061	1.2164E-06	Neu
ZCWPW1	8.684E-11	0.83355448	0.016	0.051	1.3348E-06	Neu
TNIP3	9.0842E-11	0.11655395	0.009	0.04	1.3963E-06	Neu
ASB91	9.2639E-11	0.38282813	0.011	0.043	1.424E-06	Neu
CHTF18	9.3784E-11	1.43987068	0.016	0.051	1.4416E-06	Neu
MID21	1.0326E-10	0.6911757	0.023	0.062	1.5871E-06	Neu
PLAUR2	1.0849E-10	0.25443416	0.773	0.962	1.6676E-06	Neu
PIK3R64	1.1071E-10	0.29313975	0.006	0.034	1.7018E-06	Neu
ZNF169	1.2013E-10	0.667467	0.014	0.047	1.8465E-06	Neu
EPS82	1.2233E-10	0.26468906	0.016	0.05	1.8803E-06	Neu
JAK33	1.2764E-10	1.60869454	0.026	0.066	1.962E-06	Neu
VWF1	1.3193E-10	0.60650927	0.007	0.036	2.0278E-06	Neu
ZNF782	1.4654E-10	0.42980782	0.023	0.06	2.2525E-06	Neu
DNAJC28	1.487E-10	0.86022377	0.017	0.052	2.2856E-06	Neu
USP9Y	1.4878E-10	0.23162188	0.03	0.071	2.2869E-06	Neu
MT-CO33	1.505E-10	0.45301942	0.35	0.525	2.3133E-06	Neu
C8orf58	1.5207E-10	0.61015322	0.019	0.055	2.3374E-06	Neu
ZSCAN20	1.5862E-10	0.67478372	0.02	0.056	2.4381E-06	Neu
PEG31	1.6112E-10	0.5954078	0.01	0.041	2.4766E-06	Neu
CPLANE2	1.6496E-10	0.4416669	0.013	0.045	2.5356E-06	Neu
PIWIL43	1.7147E-10	0.12846189	0.019	0.055	2.6357E-06	Neu
GPR1551	1.8601E-10	0.38660753	0.021	0.057	2.8591E-06	Neu
SEMA7A3	1.9106E-10	0.70564043	0.005	0.032	2.9367E-06	Neu
NUDT13	2.0396E-10	1.02737352	0.021	0.058	3.135E-06	Neu
SH2D4A	2.1222E-10	0.10629951	0.014	0.047	3.262E-06	Neu
ZNF485	2.2067E-10	0.83246663	0.02	0.056	3.3919E-06	Neu
POM121C	2.3944E-10	0.59699144	0.018	0.052	3.6804E-06	Neu
ZNF5771	2.4649E-10	0.99883216	0.029	0.069	3.7887E-06	Neu
ZNF1811	2.5592E-10	0.47923974	0.022	0.059	3.9337E-06	Neu
TIGD7	2.5911E-10	0.88286811	0.018	0.053	3.9827E-06	Neu
PSRC1	2.6497E-10	0.17340493	0.005	0.032	4.0729E-06	Neu
SERPINI2	2.6832E-10	0.99926843	0.048	0.097	4.1243E-06	Neu
CRPPA1	2.927E-10	0.88204496	0.02	0.055	4.499E-06	Neu
PEAK33	3.0339E-10	1.24631506	0.007	0.036	4.6634E-06	Neu
EGR13	3.1091E-10	0.81869383	0.048	0.095	4.779E-06	Neu
SAT13	3.3428E-10	1.22619671	0.781	0.963	5.1382E-06	Neu
LMNTD21	3.3905E-10	0.3356606	0.014	0.046	5.2116E-06	Neu
ZNF6581	3.4332E-10	0.16611431	0.007	0.034	5.2771E-06	Neu
ZNF813	3.5022E-10	0.34780253	0.015	0.047	5.3832E-06	Neu
DCUN1D2	3.5617E-10	0.96962974	0.037	0.079	5.4746E-06	Neu
CELF61	3.6193E-10	1.32113257	0.019	0.053	5.5632E-06	Neu
MEF2B2	3.9593E-10	0.75702335	0.015	0.047	6.0858E-06	Neu
BOK1	4.0668E-10	0.45091513	0.015	0.047	6.251E-06	Neu
ZNF57	4.1305E-10	0.61475894	0.018	0.052	6.349E-06	Neu
MYO15A1	4.3032E-10	0.32706831	0.01	0.039	6.6144E-06	Neu
ENO3	4.6104E-10	0.10868383	0.015	0.048	7.0867E-06	Neu
D2HGDH	4.7416E-10	0.30430583	0.03	0.07	7.2883E-06	Neu
ZNF1321	4.7535E-10	0.89298926	0.022	0.058	7.3067E-06	Neu
SEC31B2	5.1286E-10	0.28628842	0.024	0.06	7.8831E-06	Neu

HDAC11	5.2768E-10	0.26380921	0.009	0.037	8.111E-06	Neu
ZNF519	5.5304E-10	0.56749506	0.02	0.054	8.5008E-06	Neu
ZNF670	5.5806E-10	0.21895282	0.019	0.053	8.578E-06	Neu
ZNF517	5.6713E-10	0.2764834	0.021	0.056	8.7173E-06	Neu
ATP2A33	5.7005E-10	0.12798777	0.021	0.056	8.7622E-06	Neu
ARVCF	5.8281E-10	0.19377957	0.007	0.035	8.9584E-06	Neu
SAP252	6.1718E-10	1.11618473	0.052	0.1	9.4867E-06	Neu
SERPINA91	6.5144E-10	0.2539499	0.003	0.028	1.0013E-05	Neu
IL62	6.5395E-10	0.35152257	0.01	0.039	1.0052E-05	Neu
BCL9	6.798E-10	0.14163746	0.009	0.037	1.0449E-05	Neu
SPATC11	7.2639E-10	0.84300257	0.02	0.054	1.1165E-05	Neu
TRAF13	7.6599E-10	0.37386852	0.014	0.045	1.1774E-05	Neu
CDCA5	7.8647E-10	0.14957969	0.003	0.028	1.2089E-05	Neu
CCDC191	8.1192E-10	0.47080346	0.016	0.048	1.248E-05	Neu
ZNF792	8.1759E-10	0.41556457	0.015	0.046	1.2567E-05	Neu
OAZ11	8.5756E-10	0.11533586	0.952	0.993	1.3182E-05	Neu
NLRP33	8.6296E-10	0.94303417	0.022	0.057	1.3265E-05	Neu
ZNF74	8.9156E-10	0.69826687	0.019	0.053	1.3704E-05	Neu
MARCKS2	9.4794E-10	2.68401044	0.042	0.086	1.4571E-05	Neu
CROCC	9.6988E-10	0.51540105	0.032	0.071	1.4908E-05	Neu
RARA2	9.733E-10	0.18812674	0.634	0.947	1.4961E-05	Neu
RAD54B	9.8552E-10	0.24386449	0.007	0.034	1.5149E-05	Neu
XYLB1	1.0149E-09	0.16231047	0.01	0.039	1.56E-05	Neu
ELL3	1.0427E-09	0.70544908	0.013	0.042	1.6027E-05	Neu
GAN	1.0498E-09	0.52586743	0.009	0.036	1.6136E-05	Neu
RBFOX22	1.1021E-09	0.19730889	0.009	0.036	1.6941E-05	Neu
RBM48	1.1489E-09	0.38686403	0.029	0.066	1.766E-05	Neu
BTBD193	1.1633E-09	2.08999501	0.025	0.061	1.7881E-05	Neu
SCAI	1.1723E-09	0.2347653	0.012	0.041	1.802E-05	Neu
CAPN5	1.1967E-09	0.52644963	0.009	0.037	1.8394E-05	Neu
ZDHHC191	1.3298E-09	1.23099435	0.006	0.032	2.0441E-05	Neu
ZNF4831	1.3374E-09	0.30444339	0.015	0.046	2.0557E-05	Neu
RAD9B	1.3407E-09	0.35910045	0.005	0.03	2.0608E-05	Neu
AGER3	1.3464E-09	0.62309253	0.026	0.063	2.0696E-05	Neu
MT-ND64	1.3664E-09	1.17756232	0.092	0.153	2.1002E-05	Neu
DIP2C	1.3998E-09	0.44961323	0.01	0.038	2.1517E-05	Neu
ZNF419	1.4976E-09	0.49921384	0.011	0.039	2.302E-05	Neu
PFDN4	1.5023E-09	0.21040851	0.016	0.047	2.3092E-05	Neu
GPR1623	1.5492E-09	0.56789167	0.012	0.041	2.3812E-05	Neu
UBFD1	1.6174E-09	0.15850562	0.018	0.05	2.4861E-05	Neu
SCAMP52	1.6298E-09	0.17879082	0.009	0.036	2.5051E-05	Neu
LRRC1	1.8137E-09	0.52413932	0.011	0.039	2.7878E-05	Neu
TLN21	1.8213E-09	0.24745988	0.01	0.038	2.7996E-05	Neu
ZNF6691	1.9277E-09	0.3398593	0.015	0.045	2.9631E-05	Neu
CYP3A51	1.9431E-09	0.56538584	0.01	0.037	2.9867E-05	Neu
HIST2H2BE3	1.9768E-09	1.10569372	0.082	0.14	3.0385E-05	Neu
TRIM661	2.0066E-09	0.7920893	0.022	0.055	3.0843E-05	Neu
LINC026931	2.0588E-09	0.25551938	0.013	0.043	3.1646E-05	Neu
ZNF3341	2.1894E-09	0.91448259	0.009	0.036	3.3653E-05	Neu
MTBP1	2.2034E-09	0.23772989	0.014	0.043	3.3869E-05	Neu
CEACAM3	2.2502E-09	2.14201385	0.033	0.072	3.4588E-05	Neu
EFCAB7	2.2645E-09	0.35381968	0.011	0.039	3.4807E-05	Neu

AP002495.1	2.304E-09	0.34237664	0.018	0.049	3.5415E-05	Neu
COBLL11	2.3243E-09	0.67650246	0.018	0.049	3.5726E-05	Neu
MCTP2	2.3327E-09	3.16006931	0.013	0.004	3.5856E-05	Neu
BANP1	2.3525E-09	0.71330954	0.014	0.043	3.616E-05	Neu
HMGB3	2.4225E-09	0.16239864	0.009	0.036	3.7237E-05	Neu
RTTN	2.458E-09	0.22271861	0.013	0.041	3.7783E-05	Neu
FAIM21	2.4729E-09	0.53570471	0.01	0.037	3.8011E-05	Neu
ZNF8601	2.4897E-09	0.2006867	0.01	0.037	3.8269E-05	Neu
SLC9B2	2.7689E-09	0.51368534	0.02	0.053	4.2561E-05	Neu
TTC26	3.0453E-09	0.64280057	0.011	0.039	4.681E-05	Neu
ZNF19	3.154E-09	0.79364473	0.029	0.066	4.8481E-05	Neu
CASTOR11	3.2272E-09	0.29191037	0.015	0.045	4.9605E-05	Neu
THNSL1	3.2488E-09	0.13017878	0.009	0.035	4.9938E-05	Neu
ZKSCAN2	3.3595E-09	0.72637933	0.003	0.026	5.1639E-05	Neu
BGLAP	3.5837E-09	0.20937796	0.011	0.038	5.5086E-05	Neu
OR6N21	3.6081E-09	0.12859336	0.008	0.034	5.546E-05	Neu
OAZ32	3.7665E-09	0.58363409	0.011	0.039	5.7895E-05	Neu
MZF1	3.9193E-09	0.90002468	0.013	0.042	6.0243E-05	Neu
ZFYVE91	4.0604E-09	0.72939435	0.03	0.066	6.2413E-05	Neu
HSPA2	4.0659E-09	0.7365186	0.013	0.041	6.2496E-05	Neu
MYOZ11	4.1368E-09	0.33224982	0.009	0.034	6.3587E-05	Neu
TBX192	4.3383E-09	0.3061959	0.013	0.042	6.6684E-05	Neu
TMEM86B2	4.4529E-09	0.5777554	0.036	0.075	6.8446E-05	Neu
PLA2G4B	4.776E-09	0.95746538	0.033	0.071	7.3412E-05	Neu
SLC25A372	4.9729E-09	3.0178964	0.11	0.181	7.6438E-05	Neu
SLC18B12	5.0771E-09	0.50474819	0.014	0.042	7.8039E-05	Neu
ZFP69B	5.2547E-09	0.65373495	0.012	0.04	8.0771E-05	Neu
TSKS1	5.3596E-09	0.25254856	0.011	0.039	8.2382E-05	Neu
PATL2	5.57E-09	0.61109676	0.01	0.037	8.5616E-05	Neu
C17orf107	5.6138E-09	1.87496558	0.037	0.076	8.629E-05	Neu
PDE8B2	6.6595E-09	0.64500314	0.013	0.04	0.00010236	Neu
ZNF564	6.7766E-09	1.08970217	0.026	0.06	0.00010416	Neu
ANKRD16	6.9381E-09	0.86935376	0.018	0.049	0.00010665	Neu
EME2	7.0487E-09	0.51825232	0.017	0.047	0.00010835	Neu
GBP7	7.3799E-09	0.40279011	0.006	0.03	0.00011344	Neu
MTHFD2L	1.0447E-08	0.27570635	0.019	0.05	0.00016058	Neu
PCP2	1.0471E-08	0.50670168	0.022	0.054	0.00016095	Neu
CCDC102B1	1.0484E-08	0.13920778	0.007	0.032	0.00016115	Neu
AC119396.1	1.0678E-08	0.27886588	0.026	0.059	0.00016412	Neu
ZNF682	1.1583E-08	0.40391002	0.008	0.032	0.00017804	Neu
C19orf44	1.1628E-08	0.57725036	0.013	0.04	0.00017873	Neu
ZNF33B2	1.1715E-08	0.500096	0.014	0.041	0.00018008	Neu
ANAPC1	1.1951E-08	0.52759697	0.013	0.04	0.0001837	Neu
PLEKHA61	1.2107E-08	0.36616519	0.021	0.052	0.0001861	Neu
RHPN1	1.336E-08	0.35905288	0.014	0.041	0.00020536	Neu
AC015871.1	1.4423E-08	0.58750892	0.013	0.04	0.00022169	Neu
PNPLA11	1.488E-08	0.70981853	0.013	0.04	0.00022872	Neu
NAT141	1.5984E-08	0.13777856	0.014	0.042	0.00024569	Neu
ZNF772	1.6054E-08	0.63199751	0.014	0.042	0.00024677	Neu
ERVK3-1	1.6228E-08	0.15802239	0.012	0.038	0.00024945	Neu
ZNF502	1.656E-08	0.50008443	0.017	0.046	0.00025455	Neu
TTC32	1.6801E-08	0.52078972	0.015	0.043	0.00025825	Neu

PPP1R14C	1.7487E-08	0.7718021	0.005	0.027	0.0002688	Neu
CLEC12B	1.7661E-08	0.82112909	0.018	0.047	0.00027146	Neu
POPDC21	1.8051E-08	0.28523371	0.019	0.049	0.00027746	Neu
ZNF501	1.887E-08	0.52024532	0.014	0.042	0.00029005	Neu
FAM216A	2.0691E-08	0.3954761	0.011	0.036	0.00031804	Neu
MAP102	2.0868E-08	0.51978603	0.013	0.039	0.00032076	Neu
ARMC9	2.2699E-08	0.39433349	0.013	0.04	0.0003489	Neu
LETM2	2.3072E-08	0.26337103	0.006	0.029	0.00035464	Neu
HASPIN	2.4569E-08	0.17531771	0.005	0.026	0.00037765	Neu
CDKL3	2.5639E-08	0.49674487	0.014	0.041	0.00039409	Neu
PLAG1	2.7114E-08	0.49544562	0.013	0.039	0.00041677	Neu
MOK	2.8398E-08	0.10661568	0.01	0.035	0.0004365	Neu
CLEC7A3	2.9013E-08	0.57531405	0.582	0.912	0.00044596	Neu
JADE32	2.9249E-08	0.30237995	0.006	0.029	0.00044958	Neu
ENPP61	3.0783E-08	0.21768884	0.006	0.028	0.00047317	Neu
ZNF714	3.121E-08	0.96117309	0.018	0.047	0.00047974	Neu
ZNF3961	3.1357E-08	0.4552865	0.015	0.042	0.000482	Neu
AC099489.1	3.4448E-08	5.35222135	0.021	0.008	0.0005295	Neu
NBPF20	3.5022E-08	0.18352542	0.008	0.031	0.00053832	Neu
SMARCD3	3.5051E-08	0.3152392	0.007	0.029	0.00053877	Neu
ZNF461	3.5275E-08	0.2110234	0.009	0.033	0.00054221	Neu
ZNF4311	3.5488E-08	0.62078381	0.009	0.033	0.00054548	Neu
CFAP44	3.6859E-08	0.31304572	0.019	0.047	0.00056656	Neu
KLHL11	3.9956E-08	0.23975725	0.021	0.051	0.00061417	Neu
TRPM24	4.1211E-08	1.31489859	0.011	0.036	0.00063345	Neu
BBS12	4.1597E-08	0.77526622	0.015	0.042	0.00063939	Neu
PGGHG2	4.1787E-08	1.89839975	0.031	0.066	0.0006423	Neu
TRPV12	4.908E-08	0.19568338	0.014	0.04	0.00075441	Neu
LPIN3	4.9237E-08	0.5880317	0.009	0.032	0.00075682	Neu
ZNF2151	4.9873E-08	0.56576837	0.014	0.04	0.0007666	Neu
DLX31	5.4073E-08	0.40311977	0.022	0.052	0.00083115	Neu
KRT8	5.5541E-08	0.61798788	0.016	0.043	0.00085372	Neu
ANK21	5.5681E-08	0.85104487	0.007	0.029	0.00085587	Neu
SINHCAF2	5.855E-08	0.43593972	0.011	0.036	0.00089997	Neu
ZSWIM42	6.2083E-08	0.56722157	0.011	0.035	0.00095428	Neu
RAB6B	6.3651E-08	0.42823418	0.009	0.032	0.00097839	Neu
ELOVL31	6.6913E-08	0.52251999	0.018	0.045	0.00102851	Neu
SLC15A23	6.7074E-08	0.13933813	0.018	0.045	0.001031	Neu
NHSL22	7.4849E-08	0.39045489	0.01	0.034	0.00115051	Neu
USP22	7.624E-08	0.46599543	0.013	0.038	0.00117188	Neu
MARCKSL13	7.8163E-08	1.32059678	0.007	0.029	0.00120144	Neu
SCNN1A	7.9837E-08	0.20522345	0.006	0.027	0.00122718	Neu
KLHL17	8.3468E-08	0.775691	0.015	0.041	0.00128299	Neu
ZNF530	9.1068E-08	0.80895414	0.013	0.037	0.0013998	Neu
KIAA1211L	9.4154E-08	0.38660061	0.013	0.038	0.00144725	Neu
CASKIN2	9.5093E-08	0.44353087	0.009	0.032	0.00146168	Neu
CEP295NL	9.5729E-08	1.15620184	0.024	0.054	0.00147146	Neu
ZNF177	9.6854E-08	0.72693457	0.018	0.045	0.00148874	Neu
TMEM17	9.8802E-08	0.91975126	0.007	0.029	0.00151869	Neu
GLTPD21	1.0559E-07	0.56009824	0.006	0.026	0.00162305	Neu
ZNF713	1.0848E-07	0.4507344	0.018	0.045	0.00166746	Neu
TLE61	1.1282E-07	0.61318492	0.009	0.032	0.00173416	Neu

SLCO4C11	1.1558E-07	0.74013956	0.011	0.035	0.00177663	Neu
VNN12	1.1632E-07	1.10273121	0.01	0.032	0.00178799	Neu
CDC42EP21	1.1797E-07	2.96303316	0.027	0.059	0.00181332	Neu
AC233723.11	1.1996E-07	0.7434807	0.008	0.03	0.00184388	Neu
MAGIX	1.2491E-07	0.65431316	0.016	0.042	0.00191997	Neu
DENND5B3	1.2581E-07	0.48000725	0.009	0.031	0.00193383	Neu
LRFN11	1.3298E-07	1.06085769	0.012	0.036	0.00204402	Neu
KLHL101	1.41E-07	0.39387936	0.003	0.022	0.00216731	Neu
ZNF112	1.4955E-07	0.50153531	0.009	0.031	0.00229874	Neu
SLC35G1	1.5704E-07	0.38375255	0.009	0.031	0.00241385	Neu
APOBEC3A1	1.7813E-07	4.75409179	0.034	0.018	0.00273802	Neu
THAP9	1.7827E-07	0.29555993	0.018	0.044	0.00274024	Neu
KLHDC91	1.9533E-07	0.10758899	0.009	0.03	0.00300238	Neu
C1orf53	2.0689E-07	0.53903277	0.013	0.036	0.00318018	Neu
RBKS	2.1084E-07	0.70875174	0.009	0.03	0.00324083	Neu
FAM24B	2.1479E-07	0.99985402	0.019	0.045	0.00330155	Neu
SSBP22	2.1483E-07	0.69725641	0.014	0.039	0.0033022	Neu
KLHL25	2.4696E-07	0.71870915	0.006	0.026	0.00379601	Neu
CCDC120	2.724E-07	0.4290997	0.009	0.03	0.00418711	Neu
TRIM45	2.7428E-07	0.55212751	0.017	0.042	0.00421594	Neu
ZNF4711	2.8495E-07	0.82676992	0.015	0.04	0.00437997	Neu
MST1	2.8935E-07	0.47305722	0.016	0.041	0.00444768	Neu
FAM47E	3.0061E-07	0.26167378	0.007	0.028	0.00462065	Neu
PLEKHG23	3.0702E-07	0.58336036	0.006	0.025	0.00471914	Neu
GPR843	3.1577E-07	0.59134063	0.004	0.022	0.00485364	Neu
EME1	3.1692E-07	0.19905095	0.002	0.019	0.00487132	Neu
ZNF829	3.1769E-07	0.38233504	0.007	0.028	0.00488327	Neu
CLSTN31	3.2746E-07	0.18690417	0.015	0.04	0.0050334	Neu
LCAT2	3.4254E-07	0.53155384	0.01	0.032	0.00526524	Neu
C5orf30	3.4693E-07	0.36463032	0.006	0.026	0.00533267	Neu
HSF5	3.5424E-07	0.20714992	0.006	0.025	0.00544505	Neu
FAM189A21	3.5794E-07	0.12791956	0.007	0.027	0.00550183	Neu
INPP5A1	3.8005E-07	1.87268364	0.026	0.054	0.00584169	Neu
LRRC63	3.9493E-07	0.11099861	0.014	0.037	0.00607045	Neu
ZNF8831	4.0567E-07	0.34578186	0.008	0.028	0.00623549	Neu
LRRC692	4.2137E-07	0.11943369	0.011	0.033	0.00647683	Neu
HORMAD12	4.5059E-07	0.65161849	0.01	0.031	0.00692609	Neu
ADAMTS61	4.5711E-07	0.7923814	0.014	0.038	0.00702627	Neu
RBM34	4.7826E-07	0.19641888	0.006	0.025	0.00735133	Neu
CLHC1	5.1194E-07	0.62355693	0.021	0.047	0.00786895	Neu
ACSM11	5.2524E-07	0.26700152	0.01	0.031	0.00807341	Neu
FCAR2	5.2957E-07	2.1847585	0.135	0.205	0.00814005	Neu
MYOM11	5.2974E-07	0.45946989	0.015	0.039	0.00814261	Neu
GLIS31	5.721E-07	0.66423945	0.022	0.049	0.0087938	Neu
NHS	5.7699E-07	0.51773327	0.01	0.03	0.00886888	Neu
HS3ST3B14	5.8885E-07	0.34659488	0.008	0.028	0.00905118	Neu
C15orf62	5.9962E-07	0.42841912	0.008	0.028	0.00921673	Neu
TPBGL1	6.0879E-07	0.59522544	0.005	0.023	0.00935768	Neu
S100A5	6.1438E-07	0.41374754	0.008	0.028	0.00944359	Neu
MTMR8	6.8631E-07	0.76909796	0.017	0.041	0.0105492	Neu
SMOX3	7.1762E-07	0.24808484	0.003	0.019	0.01103061	Neu
PRDM83	7.2429E-07	3.84411332	0.01	0.003	0.0111331	Neu

METAP1D1	7.3297E-07	0.26149671	0.007	0.026	0.01126656	Neu
ZSCAN12	7.5037E-07	0.15180901	0.008	0.028	0.01153388	Neu
RNF208	7.6149E-07	0.4887695	0.014	0.036	0.01170481	Neu
PODXL2	7.6705E-07	0.29254676	0.006	0.025	0.01179027	Neu
GNA14	7.8737E-07	0.71287685	0.004	0.021	0.01210263	Neu
REPS22	8.5581E-07	1.34202751	0.009	0.028	0.01315466	Neu
CATSPER21	8.7042E-07	0.20456728	0.013	0.034	0.01337921	Neu
NPIPB15	9.791E-07	0.42652247	0.013	0.035	0.01504978	Neu
KANTR	1.0841E-06	0.87797594	0.014	0.037	0.01666351	Neu
ZNRF3	1.1645E-06	0.58449881	0.007	0.026	0.01789977	Neu
FCRL21	1.1791E-06	1.1336787	0.063	0.102	0.01812354	Neu
RPA4	1.1844E-06	0.7570628	0.01	0.031	0.01820605	Neu
CTRL	1.1909E-06	0.41167739	0.016	0.039	0.0183056	Neu
OR52K21	1.1984E-06	0.88820378	0.043	0.076	0.01842117	Neu
TNFAIP62	1.3022E-06	1.50331553	0.011	0.032	0.02001673	Neu
PODXL	1.4079E-06	0.11370146	0.006	0.024	0.0216403	Neu
TBC1D32	1.4213E-06	0.87923368	0.01	0.03	0.02184753	Neu
AMIGO1	1.5566E-06	0.12426726	0.008	0.027	0.02392588	Neu
SAPCD1	1.6754E-06	0.93398805	0.022	0.047	0.02575202	Neu
HIST4H4	1.7663E-06	0.69622122	0.012	0.033	0.02715008	Neu
SCX	1.8707E-06	1.00573151	0.007	0.025	0.02875521	Neu
TRAF3IP33	1.9092E-06	0.4654096	0.013	0.033	0.02934558	Neu
TREML13	1.9661E-06	0.4585655	0.013	0.033	0.03022108	Neu
NBPF11	1.9833E-06	0.73129836	0.019	0.042	0.03048465	Neu
DET1	2.1386E-06	0.26895254	0.014	0.036	0.03287295	Neu
BEND31	2.2045E-06	0.42742651	0.006	0.023	0.03388556	Neu
CCL41	2.2832E-06	1.42535771	0.03	0.057	0.03509493	Neu
CCDC1891	2.2925E-06	0.41064447	0.006	0.023	0.03523742	Neu
PHOSPHO2	2.3025E-06	0.43546297	0.012	0.032	0.03539155	Neu
EID2B	2.3402E-06	0.19081742	0.007	0.025	0.03597193	Neu
TOM1L1	2.4222E-06	0.15222401	0.008	0.026	0.03723133	Neu
SOGA3	2.4681E-06	0.24882064	0.018	0.041	0.03793675	Neu
ARRB21	2.6289E-06	0.24656659	0.672	0.959	0.04040869	Neu
KLKB1	2.7735E-06	0.5442178	0.01	0.029	0.04263135	Neu
ZNF3911	2.8315E-06	0.4436382	0.005	0.021	0.04352364	Neu
SPEF2	2.8875E-06	0.22343071	0.014	0.034	0.04438326	Neu
OSGIN11	2.965E-06	0.70994226	0.002	0.017	0.04557533	Neu
ZNF273	3.0454E-06	0.49468914	0.012	0.032	0.04681099	Neu
RIPOR24	3.2656E-06	1.42664811	0.044	0.076	0.05019599	Neu
CTU1	3.6811E-06	0.88548573	0.009	0.027	0.05658159	Neu
FAM83G	3.7173E-06	0.84951373	0.01	0.029	0.05713928	Neu
TESMIN	3.894E-06	0.26485737	0.005	0.021	0.059855	Neu
CACNB12	4.072E-06	0.63136774	0.014	0.034	0.06259014	Neu
ROM1	4.2514E-06	0.34250582	0.01	0.029	0.06534824	Neu
TXK	4.2603E-06	0.23285537	0.009	0.026	0.06548508	Neu
DCLK23	4.2744E-06	0.91626902	0.006	0.023	0.06570169	Neu
HIST3H2A2	4.3333E-06	0.56406596	0.005	0.021	0.0666073	Neu
ZNF66	4.3631E-06	0.72077881	0.015	0.036	0.0670653	Neu
LARGE1	4.4167E-06	0.44942312	0.005	0.021	0.06788977	Neu
MYORG	4.4652E-06	0.84839061	0.008	0.025	0.06863384	Neu
ARMCX4	4.5487E-06	1.15553251	0.012	0.031	0.06991868	Neu
F121	4.604E-06	0.59655705	0.005	0.02	0.07076797	Neu

SLAMF73	4.7108E-06	1.2300272	0.01	0.029	0.0724094	Neu
CXorf651	4.9103E-06	0.49053089	0.014	0.034	0.07547631	Neu
ARRDC53	4.9586E-06	0.65149565	0.009	0.027	0.07621885	Neu
RGL13	5.0866E-06	0.26631602	0.003	0.017	0.07818642	Neu
CDC25A	5.1185E-06	0.76128728	0.002	0.016	0.07867631	Neu
TPM21	5.2768E-06	0.6308524	0.013	0.032	0.08110954	Neu
GPR1414	5.678E-06	1.3796747	0.007	0.023	0.08727662	Neu
CCDC1381	5.8015E-06	0.43847729	0.011	0.029	0.08917466	Neu
GABRA41	6.1051E-06	0.19876287	0.018	0.039	0.09384217	Neu
TNNI11	6.4028E-06	0.93801535	0.021	0.043	0.0984171	Neu
ANGPTL61	6.7464E-06	1.66090871	0.003	0.018	0.10369967	Neu
CALD11	6.8586E-06	0.42856322	0.005	0.02	0.10542291	Neu
CDC7	6.9427E-06	0.31780324	0.01	0.028	0.10671642	Neu
EXOC3L41	6.9745E-06	0.36327244	0.006	0.021	0.10720429	Neu
EIF13	7.1049E-06	0.27751422	0.619	0.944	0.10920882	Neu
CDK3	7.2485E-06	0.69664682	0.018	0.04	0.11141711	Neu
TICRR	7.5322E-06	0.71641891	0.004	0.018	0.11577785	Neu
BRI32	7.7326E-06	0.1504544	0.839	0.973	0.1188575	Neu
RIBC1	8.0596E-06	0.23924333	0.003	0.016	0.12388437	Neu
PTX31	8.2565E-06	0.74863369	0.014	0.034	0.1269102	Neu
RUNDC3A1	8.5823E-06	0.16422573	0.004	0.018	0.13191871	Neu
TMCO21	8.8416E-06	1.1306605	0.007	0.023	0.13590472	Neu
ZNF491	8.8609E-06	0.35595099	0.007	0.023	0.13620058	Neu
ADGRB1	9.181E-06	0.88935731	0.006	0.022	0.14112134	Neu
NIPSNAP3B3	9.2079E-06	0.14313454	0.011	0.029	0.141534	Neu
TREM11	9.8802E-06	0.11461922	0.784	0.96	0.15186906	Neu
RAB39B	9.9286E-06	0.25009012	0.004	0.018	0.15261216	Neu
SYT172	9.9533E-06	0.39316	0.007	0.023	0.15299187	Neu
ZDBF2	1.0437E-05	0.89010206	0.013	0.031	0.16042787	Neu
ZMYND121	1.1598E-05	0.73920329	0.007	0.023	0.17827378	Neu
CEACAM192	1.1778E-05	0.96039839	0.023	0.045	0.18104039	Neu
TMEM81	1.2634E-05	0.47696987	0.01	0.027	0.19419607	Neu
EVI2B	1.2886E-05	0.26963363	0.601	0.926	0.19807495	Neu
TBKBP13	1.3919E-05	0.89408768	0.017	0.037	0.21394478	Neu
CRYGS	1.4576E-05	0.75862745	0.013	0.031	0.22405043	Neu
SFR1	1.5011E-05	0.77635049	0.011	0.029	0.23073032	Neu
KRTCAP31	1.7529E-05	0.23170633	0.005	0.019	0.26944531	Neu
PPM1N1	1.7649E-05	0.12251642	0.009	0.025	0.27128508	Neu
FOSL12	1.8264E-05	1.30318458	0.007	0.022	0.28073587	Neu
CCDC30	1.9317E-05	0.82209089	0.013	0.031	0.29691929	Neu
RIPK41	1.9389E-05	0.12813399	0.002	0.015	0.2980302	Neu
PARD3B1	2.0353E-05	0.44788706	0.006	0.021	0.31284925	Neu
ACSM51	2.1075E-05	0.31542111	0.004	0.017	0.32394883	Neu
SCN11A1	2.1587E-05	0.63286942	0.004	0.017	0.33180673	Neu
COL14A11	2.2324E-05	0.16999166	0.007	0.022	0.34314058	Neu
RIMKLA	2.3532E-05	0.26107557	0.001	0.012	0.36171438	Neu
SNED1	2.3548E-05	0.38451151	0.007	0.023	0.36194945	Neu
ASAP31	2.3877E-05	0.68286021	0.002	0.013	0.36700617	Neu
CDC42BPG	2.4293E-05	1.59302787	0.011	0.028	0.37340509	Neu
PRCD1	2.5491E-05	0.30398753	0.005	0.018	0.3918175	Neu
ARL14EPL1	2.6065E-05	0.23823296	0.006	0.021	0.40065028	Neu
TMEM265	2.6607E-05	0.51837463	0.006	0.021	0.40897107	Neu

DDTL	2.6951E-05	0.54741006	0.009	0.025	0.41426319	Neu
PKP4	3.0521E-05	0.24772664	0.009	0.024	0.46913896	Neu
RNF1491	3.0549E-05	0.29443358	0.783	0.975	0.46957015	Neu
BIRC71	3.0741E-05	0.40533562	0.005	0.019	0.47252062	Neu
STX1B1	3.2835E-05	0.3823092	0.006	0.02	0.50470704	Neu
ZNF681	3.3916E-05	1.04139676	0.009	0.025	0.52131732	Neu
CDA2	3.4927E-05	1.57617096	0.003	0.015	0.53685961	Neu
MINDY4	3.6609E-05	0.43323329	0.006	0.02	0.56272405	Neu
MAPK8IP1	3.7137E-05	0.10674491	0.002	0.013	0.57082773	Neu
LYNX1	3.7434E-05	0.40805723	0.007	0.022	0.57539215	Neu
EFEMP2	3.786E-05	0.59190522	0.005	0.018	0.58194256	Neu
OTUD7B	3.7893E-05	0.42194699	0.005	0.018	0.58245947	Neu
MS4A4E4	3.8424E-05	0.79326892	0.014	0.031	0.59060857	Neu
CEP126	3.9676E-05	0.87229638	0.021	0.041	0.60985284	Neu
PRELID3A1	4.0442E-05	0.46757705	0.007	0.021	0.62163071	Neu
MFAP3L1	4.3361E-05	1.30024988	0.005	0.018	0.66649503	Neu
TDO21	4.4621E-05	0.93228992	0.007	0.022	0.6858739	Neu
SPRY3	4.545E-05	0.66444425	0.015	0.032	0.69861504	Neu
MARC11	4.7568E-05	0.80034921	0.006	0.019	0.73116862	Neu
RAPSN	4.8215E-05	0.51683082	0.008	0.023	0.74111729	Neu
PIP5KL11	4.9462E-05	0.53329761	0.008	0.023	0.7602867	Neu
WFIKKN1	4.9846E-05	1.51002544	0.01	0.026	0.76618617	Neu
UCN	5.0399E-05	0.45067125	0.006	0.019	0.77468594	Neu
FP565260.1	5.2791E-05	0.97768052	0.011	0.027	0.81145439	Neu
GABBR14	5.3876E-05	0.23780941	0.006	0.019	0.82812661	Neu
CASS43	5.3973E-05	1.72322995	0.016	0.034	0.82962296	Neu
ZNF165	5.639E-05	0.55458482	0.005	0.018	0.86677473	Neu
TMEM117	5.8466E-05	0.80696977	0.007	0.021	0.89868631	Neu
ABCB9	5.9575E-05	0.60545923	0.009	0.023	0.91572835	Neu
AASS	6.2637E-05	1.11300101	0.006	0.02	0.96278867	Neu
SHF1	6.3844E-05	0.5120913	0.007	0.021	0.98134811	Neu
CBFA2T34	6.526E-05	0.52133633	0.003	0.015	1	Neu
TMC4	6.5599E-05	0.61383087	0.005	0.017	1	Neu
ADGRL12	6.598E-05	0.62230917	0.006	0.02	1	Neu
TSLP1	6.6363E-05	0.44746614	0.009	0.023	1	Neu
RPH3A1	6.6377E-05	0.54394265	0.014	0.03	1	Neu
CD163L11	7.1104E-05	0.22203314	0.003	0.015	1	Neu
DOCK3	7.2884E-05	0.35559553	0.003	0.015	1	Neu
RAPGEFL1	7.5703E-05	0.86986247	0.009	0.024	1	Neu
GPT2	7.6835E-05	0.20286724	0.003	0.015	1	Neu
TNFRSF10C	7.8865E-05	2.0808127	0.006	0.019	1	Neu
AKR1C8P1	8.0602E-05	0.91127774	0.009	0.023	1	Neu
PRKY1	8.1816E-05	0.28976416	0.002	0.013	1	Neu
GCNA3	8.1837E-05	0.70750495	0.004	0.016	1	Neu
MTCP1	8.2172E-05	0.85121656	0.008	0.022	1	Neu
IRAK22	8.6355E-05	3.27386083	0.027	0.049	1	Neu
OCLN1	8.7498E-05	0.55039392	0.006	0.019	1	Neu
FAM222A	9.3149E-05	0.28050479	0.003	0.015	1	Neu
C9orf116	9.6091E-05	0.22477588	0.006	0.018	1	Neu
TCEAL6	9.9128E-05	0.80119263	0.004	0.016	1	Neu
CCDC180	0.00010441	0.15626085	0.005	0.017	1	Neu
FZD41	0.00010485	0.21343831	0.007	0.02	1	Neu

SPTBN5	0.00010505	1.03451372	0.014	0.031	1	Neu
OR52K11	0.00010625	0.82698392	0.013	0.028	1	Neu
GPR135	0.00011051	0.62723625	0.013	0.029	1	Neu
ALOX51	0.00011094	0.13404196	0.871	0.972	1	Neu
PGAP11	0.00011221	0.33171433	0.003	0.014	1	Neu
ZG16B1	0.00011382	0.17738841	0.003	0.014	1	Neu
ZNF781	0.00011536	1.21532594	0.009	0.023	1	Neu
GNMT1	0.00012242	0.9574315	0.007	0.02	1	Neu
C1orf213	0.00012251	0.4282303	0.002	0.012	1	Neu
DUSP19	0.00012614	0.18415617	0.005	0.016	1	Neu
ITGAX4	0.00012665	1.336904	0.537	0.802	1	Neu
GLIPR1L2	0.00013044	0.83605835	0.007	0.02	1	Neu
CCDC1461	0.00013164	1.09256304	0.02	0.038	1	Neu
PANK1	0.00013445	0.47462545	0.002	0.011	1	Neu
DNAAF3	0.00014081	0.18602536	0.004	0.015	1	Neu
ADCY42	0.00014189	1.10846005	0.011	0.025	1	Neu
TIFAB3	0.00014227	0.76973294	0.002	0.012	1	Neu
RNF227	0.00015411	0.40859539	0.004	0.015	1	Neu
UFSP1	0.00015455	0.24048066	0.003	0.014	1	Neu
AMPD34	0.00015613	1.21314727	0.005	0.017	1	Neu
ZNF6671	0.00016162	0.42185671	0.007	0.019	1	Neu
ANKDD1A2	0.00016326	1.23668295	0.013	0.028	1	Neu
GPRASP21	0.00016405	0.93117086	0.009	0.023	1	Neu
ELMO31	0.00016605	0.57003518	0.009	0.022	1	Neu
S100Z3	0.00017304	0.36621045	0.006	0.018	1	Neu
CLUL11	0.0001795	0.88945857	0.008	0.021	1	Neu
S100A91	0.00018289	0.14913389	0.814	0.951	1	Neu
CCDC1421	0.00018415	0.89891809	0.006	0.018	1	Neu
ETV54	0.00018895	0.32736555	0.006	0.018	1	Neu
PLGLB2	0.0001927	0.95581913	0.031	0.053	1	Neu
POU2F24	0.00020248	0.21828116	0.013	0.027	1	Neu
SPHK13	0.00020771	1.8149613	0.005	0.016	1	Neu
FUT74	0.00021146	1.11470235	0.009	0.022	1	Neu
NEIL3	0.0002196	0.11235335	0.005	0.016	1	Neu
CD300LB3	0.00022339	0.96944724	0.007	0.019	1	Neu
ISG203	0.00023762	1.9180687	0.029	0.049	1	Neu
FAM118A3	0.00024006	0.82992624	0.008	0.021	1	Neu
C6orf1632	0.00026525	0.51180875	0.012	0.026	1	Neu
IL1R13	0.00026874	2.56570152	0.018	0.034	1	Neu
EFNA3	0.00027919	0.65217153	0.006	0.017	1	Neu
DRC7	0.00028402	0.37890477	0.001	0.01	1	Neu
CCNB31	0.00028931	1.24381214	0.003	0.013	1	Neu
CDC25C	0.0002894	0.89059031	0.001	0.01	1	Neu
CCDC17	0.00029525	0.20893337	0.009	0.022	1	Neu
TGM1	0.00029655	0.9316107	0.009	0.021	1	Neu
CAVIN2	0.00034349	0.70295265	0.008	0.02	1	Neu
ANTXR24	0.00034695	1.05914922	0.013	0.027	1	Neu
KCNJ21	0.0003897	1.76078219	0.006	0.017	1	Neu
C2orf92	0.00039397	0.59065425	0.013	0.027	1	Neu
MYO101	0.00040571	0.62979378	0.002	0.01	1	Neu
EFCAB6	0.0004169	0.65687901	0.006	0.017	1	Neu
LRRC37A31	0.0004217	0.33456859	0.002	0.01	1	Neu

C15orf651	0.00042253	0.44686793	0.008	0.02	1	Neu
E2F7	0.00042377	0.92570843	0.002	0.01	1	Neu
OR13A11	0.00043148	0.68081027	0.008	0.02	1	Neu
GK3P	0.00043554	0.97390891	0.004	0.014	1	Neu
HOXB32	0.00045232	0.696552	0.012	0.025	1	Neu
VNN3	0.00045307	5.40940787	0.011	0.005	1	Neu
TCTEX1D4	0.0004669	1.44110892	0.007	0.018	1	Neu
CCDC122	0.00047987	0.19957506	0.006	0.017	1	Neu
MPP32	0.00049184	1.5552076	0.007	0.018	1	Neu
KAZALD1	0.00049736	1.11505394	0.011	0.024	1	Neu
CCDC1542	0.0005085	1.00974931	0.006	0.016	1	Neu
CD2442	0.00051187	0.58250749	0.002	0.01	1	Neu
ANK1	0.00052632	0.95342657	0.007	0.018	1	Neu
DNAH17	0.00054586	2.43385755	0.007	0.019	1	Neu
LRRC49	0.00055577	0.26859167	0.004	0.014	1	Neu
LRG1	0.00055949	3.70757431	0.039	0.061	1	Neu
C12orf761	0.0005696	0.50503138	0.005	0.014	1	Neu
PAQR62	0.00057042	0.84524127	0.01	0.023	1	Neu
E2F51	0.00057491	0.73901438	0.005	0.014	1	Neu
TTLL10	0.00057592	0.42865522	0.008	0.019	1	Neu
POU5F21	0.00057934	1.41956241	0.027	0.045	1	Neu
ERP271	0.00059355	0.70484397	0.014	0.028	1	Neu
STXBP21	0.0005958	0.10224759	0.732	0.968	1	Neu
C5orf34	0.00061157	0.67658128	0.004	0.013	1	Neu
PIH1D2	0.000614	1.16083307	0.005	0.014	1	Neu
ATXN7L2	0.00063464	0.89173585	0.003	0.012	1	Neu
ADAM284	0.00064603	0.8660607	0.002	0.01	1	Neu
DND12	0.00064621	0.64598104	0.005	0.014	1	Neu
INCA11	0.00065355	0.47864104	0.003	0.012	1	Neu
MC1R	0.00068142	0.57575075	0.005	0.014	1	Neu
SLC29A2	0.00069675	1.18022477	0.01	0.022	1	Neu
PLXNC14	0.0007079	2.10684398	0.021	0.037	1	Neu
ABTB21	0.00074444	0.55994896	0.002	0.01	1	Neu
SIGLEC151	0.00075026	1.17989383	0.01	0.022	1	Neu
GUCA1B1	0.00075126	0.52304999	0.002	0.011	1	Neu
GALNT34	0.00078871	0.65829363	0.002	0.01	1	Neu
BTNL81	0.00079231	2.31630889	0.009	0.02	1	Neu
CTTN1	0.00079977	0.47455475	0.005	0.014	1	Neu
ANKS6	0.00080855	0.68876288	0.012	0.025	1	Neu
TREML41	0.00084815	1.6174556	0.003	0.011	1	Neu
LNP11	0.00087893	0.32417773	0.005	0.014	1	Neu
TCTE3	0.00095881	0.65541766	0.003	0.012	1	Neu
DCHS13	0.00096197	0.77585155	0.005	0.014	1	Neu
ST6GALNAC32	0.00096373	0.79484183	0.004	0.013	1	Neu
NR6A11	0.00098626	1.95835707	0.006	0.016	1	Neu
C19orf57	0.00098862	1.30892599	0.004	0.013	1	Neu
CLEC18A	0.00100016	0.38617283	0.003	0.012	1	Neu
ADAM20	0.00103761	0.22969714	0.003	0.012	1	Neu
CNIH2	0.00103835	1.09484495	0.003	0.012	1	Neu
TMEM266	0.00104537	1.44060366	0.003	0.011	1	Neu
LGALS24	0.00110239	0.337588	0.005	0.015	1	Neu
TAS2R42	0.00110921	0.27526543	0.01	0.022	1	Neu

ZC3H12C2	0.00113649	1.60064935	0.004	0.013	1	Neu
FAM161A	0.00113688	0.65943318	0.005	0.015	1	Neu
CAPS2	0.00115677	0.48326799	0.002	0.01	1	Neu
NPM2	0.00117671	0.54674304	0.005	0.014	1	Neu
UBE3D	0.00118806	0.13240678	0.004	0.013	1	Neu
CCDC194	0.0012225	0.8793719	0.004	0.013	1	Neu
PCBP3	0.0012386	0.91253206	0.005	0.014	1	Neu
ARHGEF101	0.00124926	0.71451408	0.004	0.013	1	Neu
MICU3	0.00128132	0.57573556	0.006	0.016	1	Neu
PFN4	0.00132667	0.90690357	0.003	0.011	1	Neu
ZFP37	0.00135593	0.82778971	0.006	0.015	1	Neu
ZNF214	0.0013734	0.79866896	0.002	0.01	1	Neu
EQTN1	0.00143851	0.53823603	0.007	0.017	1	Neu
CCL31	0.00146196	3.02414645	0.019	0.033	1	Neu
MTSS2	0.00146563	1.25656719	0.006	0.015	1	Neu
SLC22A1	0.00151587	1.51389845	0.005	0.014	1	Neu
PPP1R36	0.00154132	0.64306859	0.004	0.012	1	Neu
CR1L	0.00165192	1.15869476	0.005	0.013	1	Neu
PLEKHN12	0.00165299	0.34059263	0.003	0.011	1	Neu
TNFRSF6B1	0.00166381	0.84592741	0.01	0.021	1	Neu
MAP3K9	0.00169725	0.11257168	0.008	0.018	1	Neu
QRICH21	0.00172869	0.27155345	0.005	0.014	1	Neu
CCL4L21	0.00173682	3.53020188	0.016	0.029	1	Neu
LRRC24	0.0018391	0.92571206	0.006	0.015	1	Neu
CMTM12	0.0018433	0.96954583	0.005	0.013	1	Neu
UCP3	0.00188118	0.92090552	0.01	0.02	1	Neu
MYH14	0.0018827	0.79321286	0.007	0.016	1	Neu
MEIS3	0.00193438	1.80420386	0.003	0.01	1	Neu
TVP23C	0.0019979	0.45894029	0.006	0.015	1	Neu
GREB11	0.00209013	1.02165214	0.003	0.011	1	Neu
TMEM1544	0.00211921	2.27338349	0.021	0.035	1	Neu
TMED61	0.00213408	0.97596273	0.003	0.011	1	Neu
HSD11B2	0.00217239	0.68719932	0.003	0.01	1	Neu
GAS1	0.00225847	0.22693412	0.006	0.015	1	Neu
CLTCL1	0.00226336	0.83025741	0.005	0.013	1	Neu
VAMP11	0.00238544	0.47229682	0.011	0.022	1	Neu
ERMN2	0.00240343	0.56104297	0.004	0.012	1	Neu
ZNF4674	0.00248309	0.88408386	0.004	0.012	1	Neu
PRDM5	0.00252965	1.4071365	0.004	0.012	1	Neu
ANKRD224	0.0026011	1.25483171	0.003	0.011	1	Neu
KCTD1	0.00265622	1.01909577	0.005	0.013	1	Neu
CS1	0.00273468	0.19636904	0.005	0.013	1	Neu
RAB3C1	0.00279128	1.60391865	0.006	0.014	1	Neu
GSTT2B1	0.00281867	0.57855727	0.006	0.014	1	Neu
RBSN	0.00288517	0.55348913	0.006	0.015	1	Neu
PCSK4	0.0029189	0.52978342	0.003	0.01	1	Neu
KLF141	0.00297208	0.5795408	0.003	0.011	1	Neu
PMEL	0.00298846	1.04328792	0.003	0.01	1	Neu
POMC	0.00305727	0.72608954	0.005	0.013	1	Neu
WDR31	0.00307894	0.9612458	0.007	0.016	1	Neu
LRP21	0.00311302	0.40103683	0.005	0.012	1	Neu
MYMX1	0.00342843	1.51486369	0.005	0.012	1	Neu

C17orf991	0.00357573	0.10651512	0.003	0.01	1	Neu
NRIP31	0.00361451	2.21544574	0.005	0.013	1	Neu
C11orf65	0.00374052	0.62212707	0.004	0.011	1	Neu
ZNF763	0.00374214	1.33768614	0.005	0.013	1	Neu
BRICD5	0.00381387	0.77909186	0.004	0.011	1	Neu
PRR221	0.00382276	0.92813398	0.01	0.019	1	Neu
NRN1L	0.00384993	1.15426783	0.007	0.015	1	Neu
FFAR2	0.00394764	3.82983253	0.037	0.055	1	Neu
HOXA4	0.00400222	0.81762305	0.003	0.01	1	Neu
SNX101	0.00405604	0.237702	0.663	0.941	1	Neu
TSPAN6	0.00418759	0.78706011	0.01	0.02	1	Neu
UBAP1L	0.00420462	1.12214335	0.007	0.015	1	Neu
MID1	0.00423905	0.32856349	0.005	0.013	1	Neu
ADAMTS13	0.00443639	0.6754395	0.006	0.014	1	Neu
ATP6V1FNB	0.00453175	0.70386166	0.006	0.014	1	Neu
SDCBP2	0.00466324	1.05824997	0.007	0.015	1	Neu
TSGA10IP1	0.00473241	0.16888588	0.007	0.015	1	Neu
CCDC621	0.004918	1.07247656	0.004	0.011	1	Neu
BEGAIN1	0.00503265	0.64419514	0.007	0.015	1	Neu
SLC25A341	0.00510397	1.17494556	0.004	0.011	1	Neu
TMPRSS13	0.00519879	0.76331426	0.006	0.014	1	Neu
GPRASP1	0.00521617	0.41376914	0.007	0.015	1	Neu
ANXA9	0.0060512	1.31985295	0.006	0.014	1	Neu
GUCY1B1	0.00613815	0.99958169	0.005	0.012	1	Neu
PLS31	0.00618548	0.52302557	0.005	0.012	1	Neu
OLAH	0.00623003	0.87721297	0.006	0.014	1	Neu
DEPDC4	0.00627031	1.7301425	0.005	0.012	1	Neu
TMEM52	0.00653475	0.87238556	0.004	0.011	1	Neu
FOS3	0.0068142	0.71800606	0.799	0.952	1	Neu
CASTOR3	0.00733738	0.56445204	0.01	0.019	1	Neu
PPFIA3	0.00734906	1.23970718	0.005	0.012	1	Neu
CDH234	0.0078816	0.37257437	0.003	0.01	1	Neu
ZNF3111	0.00815786	0.54930244	0.005	0.012	1	Neu
CHI3L11	0.0082606	2.54698894	0.007	0.015	1	Neu
RANBP3L1	0.00851277	1.79629854	0.003	0.01	1	Neu
MYRF	0.00852929	1.06608678	0.003	0.01	1	Neu
ADAMTSL21	0.0089325	1.12647312	0.007	0.014	1	Neu
LILRA14	0.00927951	0.766216	0.005	0.011	1	Neu
TMEM881	0.00942139	2.41991525	0.017	0.027	1	Neu
HYKK	0.00963527	0.66934749	0.004	0.01	1	Neu
AVIL3	0.00964728	1.83881503	0.007	0.015	1	Neu
TRAC	0	10.3715871	0.739	0.005	0	T Cells
TRBC2	0	10.3693455	0.718	0.006	0	T Cells
IL32	0	8.99614459	0.721	0.015	0	T Cells
SYNE2	0	8.76860877	0.683	0.012	0	T Cells
ETS12	0	9.25910521	0.674	0.014	0	T Cells
RAB21	0	0.10705334	0.282	0.881	0	T Cells
ADRM11	0	0.11454343	0.294	0.89	0	T Cells
TRIM381	0	0.13161691	0.278	0.87	0	T Cells
MAF12	0	0.10278858	0.281	0.872	0	T Cells
NDUFV1	0	0.17412125	0.282	0.873	0	T Cells
KIF5B2	0	0.19330711	0.313	0.901	0	T Cells

ELOVL13	0	0.13275411	0.269	0.856	0	T Cells
HSF1	0	0.22003667	0.271	0.858	0	T Cells
NBR1	0	0.17072792	0.255	0.842	0	T Cells
RER1	0	0.18050611	0.319	0.905	0	T Cells
EMC4	0	0.2134349	0.278	0.864	0	T Cells
MAPKAPK3	0	0.21622813	0.312	0.897	0	T Cells
DTX3L	0	0.11221423	0.256	0.841	0	T Cells
COX4I1	0	0.13164913	0.29	0.875	0	T Cells
STAU1	0	0.2463611	0.293	0.876	0	T Cells
CLPTM1L1	0	0.12416045	0.228	0.809	0	T Cells
SNX110	0	0.10795384	0.357	0.938	0	T Cells
USF21	0	0.10154316	0.332	0.913	0	T Cells
PDHA1	0	0.24426772	0.245	0.825	0	T Cells
CRYBG11	0	0.21673489	0.249	0.828	0	T Cells
NDUFB10	0	0.20905825	0.251	0.829	0	T Cells
SAMD9L	0	0.13129946	0.313	0.89	0	T Cells
RAB18	0	0.23917204	0.242	0.819	0	T Cells
MTDH3	0	0.28399658	0.309	0.885	0	T Cells
ALKBH5	0	0.32613932	0.287	0.863	0	T Cells
LASP1	0	0.12578052	0.348	0.924	0	T Cells
POLR2A	0	0.22043525	0.32	0.895	0	T Cells
MAT2A5	0	0.13718705	0.284	0.858	0	T Cells
SEC62	0	0.23957714	0.335	0.909	0	T Cells
RUFY13	0	0.12051944	0.226	0.8	0	T Cells
UBE2A	0	0.12101217	0.228	0.802	0	T Cells
SPCS1	0	0.25917464	0.268	0.842	0	T Cells
SRI1	0	0.18357649	0.265	0.839	0	T Cells
SRPRA	0	0.29581126	0.275	0.849	0	T Cells
ATP5MG	0	0.20156973	0.272	0.845	0	T Cells
ISCU1	0	0.13253205	0.271	0.844	0	T Cells
VPS26A	0	0.11213775	0.206	0.778	0	T Cells
PLCB24	0	0.10217188	0.287	0.859	0	T Cells
TRIP12	0	0.21779825	0.343	0.914	0	T Cells
PUF60	0	0.26439095	0.277	0.848	0	T Cells
DOCK111	0	0.10528326	0.37	0.941	0	T Cells
PPP1CA1	0	0.21842045	0.319	0.889	0	T Cells
TMOD31	0	0.22563247	0.237	0.807	0	T Cells
DNAJA2	0	0.33392617	0.283	0.853	0	T Cells
C4orf3	0	0.19864356	0.263	0.833	0	T Cells
MAGT1	0	0.13644528	0.217	0.786	0	T Cells
UFC11	0	0.30453055	0.288	0.857	0	T Cells
PPP3R1	0	0.29573133	0.263	0.832	0	T Cells
DCTD	0	0.16037145	0.213	0.781	0	T Cells
STUB1	0	0.15223495	0.213	0.78	0	T Cells
AP3D11	0	0.11967318	0.208	0.775	0	T Cells
SF3B5	0	0.227319	0.27	0.837	0	T Cells
FBXO7	0	0.33277301	0.292	0.859	0	T Cells
ZMAT21	0	0.18848735	0.233	0.799	0	T Cells
PFDN51	0	0.28848387	0.322	0.888	0	T Cells
PAK22	0	0.28476362	0.26	0.826	0	T Cells
SEC31A	0	0.34289614	0.269	0.835	0	T Cells
PPP1R11	0	0.16070033	0.227	0.792	0	T Cells

CIB1	0	0.18024954	0.359	0.924	0	T Cells
ATP5MC21	0	0.30158609	0.302	0.867	0	T Cells
ZBTB7A	0	0.28754168	0.318	0.882	0	T Cells
APOL6	0	0.11635542	0.371	0.934	0	T Cells
DERL1	0	0.33867671	0.25	0.813	0	T Cells
TSPAN14	0	0.21926572	0.276	0.839	0	T Cells
C11orf58	0	0.36196024	0.278	0.841	0	T Cells
ARL6IP4	0	0.28867198	0.238	0.799	0	T Cells
NRBP1	0	0.29983826	0.255	0.816	0	T Cells
UBAP2L1	0	0.24467034	0.222	0.782	0	T Cells
MYO1F3	0	0.36546446	0.358	0.917	0	T Cells
AP1M1	0	0.2369684	0.231	0.79	0	T Cells
SKIL4	0	0.39422745	0.331	0.89	0	T Cells
GMFG1	0	0.37333713	0.288	0.847	0	T Cells
USP251	0	0.4128533	0.321	0.88	0	T Cells
PPM1G	0	0.241868	0.223	0.781	0	T Cells
PPP1CB2	0	0.40444087	0.284	0.842	0	T Cells
PTK2B1	0	0.373879	0.339	0.897	0	T Cells
HNRNPH2	0	0.14075941	0.202	0.76	0	T Cells
CYB5B	0	0.2650329	0.243	0.8	0	T Cells
USP9X2	0	0.28191079	0.244	0.801	0	T Cells
RNF10	0	0.38380328	0.276	0.833	0	T Cells
TXLNA	0	0.18958532	0.225	0.781	0	T Cells
HIGD2A1	0	0.29335237	0.245	0.801	0	T Cells
TAOK11	0	0.41374723	0.289	0.845	0	T Cells
EIF2AK21	0	0.27412468	0.248	0.803	0	T Cells
SERINC3	0	0.34223024	0.364	0.919	0	T Cells
ATP5F1E1	0	0.16624004	0.356	0.911	0	T Cells
WAS1	0	0.33815562	0.26	0.815	0	T Cells
SH3GLB11	0	0.33421973	0.319	0.873	0	T Cells
CSDE11	0	0.25173199	0.225	0.779	0	T Cells
TMEM2591	0	0.35456734	0.236	0.79	0	T Cells
IL17RA2	0	0.17544228	0.226	0.78	0	T Cells
RNF71	0	0.38671208	0.282	0.834	0	T Cells
MCRS11	0	0.2135564	0.213	0.765	0	T Cells
COX7A2L1	0	0.36009076	0.264	0.816	0	T Cells
RBM3	0	0.42432708	0.277	0.829	0	T Cells
RABGGTB1	0	0.20299508	0.229	0.78	0	T Cells
SCAF111	0	0.27417519	0.218	0.768	0	T Cells
DNAJC73	0	0.18900258	0.195	0.745	0	T Cells
GDI1	0	0.46433181	0.308	0.858	0	T Cells
CALCOCO21	0	0.4115052	0.273	0.823	0	T Cells
NDUFV21	0	0.51299009	0.277	0.827	0	T Cells
TRIM27	0	0.3705533	0.246	0.795	0	T Cells
RNF5	0	0.14020023	0.205	0.754	0	T Cells
EIF51	0	0.22358652	0.247	0.796	0	T Cells
AGPAT1	0	0.17549105	0.2	0.749	0	T Cells
ZFP365	0	0.1730306	0.34	0.889	0	T Cells
WASF21	0	0.29235999	0.23	0.778	0	T Cells
CNPPD12	0	0.239243	0.221	0.769	0	T Cells
CLINT1	0	0.11662481	0.191	0.739	0	T Cells
EIF3A2	0	0.42118554	0.345	0.893	0	T Cells

ORMDL21	0	0.22608975	0.209	0.757	0	T Cells
MVP	0	0.1013786	0.204	0.752	0	T Cells
ZBTB47	0	0.33581232	0.254	0.802	0	T Cells
PJA21	0	0.50219499	0.354	0.901	0	T Cells
DCTN2	0	0.18405173	0.211	0.758	0	T Cells
ELAVL1	0	0.33131872	0.229	0.776	0	T Cells
NUMA1	0	0.47496714	0.299	0.846	0	T Cells
TOR1AIP11	0	0.27916443	0.221	0.767	0	T Cells
ABI11	0	0.33572932	0.236	0.782	0	T Cells
CASP42	0	0.207763	0.213	0.759	0	T Cells
LRP101	0	0.29473521	0.241	0.787	0	T Cells
UBE4A	0	0.27554617	0.212	0.757	0	T Cells
CSNK1D2	0	0.19384775	0.217	0.762	0	T Cells
SMAD21	0	0.36504199	0.228	0.773	0	T Cells
NDFIP11	0	0.53796464	0.318	0.863	0	T Cells
MICAL13	0	0.26462144	0.32	0.865	0	T Cells
PPP1R7	0	0.29255427	0.23	0.774	0	T Cells
RNF1682	0	0.28614325	0.215	0.759	0	T Cells
ADD1	0	0.49003565	0.336	0.88	0	T Cells
PDCD62	0	0.37230981	0.223	0.767	0	T Cells
SNRPC	0	0.27791209	0.211	0.755	0	T Cells
PPP6C1	0	0.51621611	0.293	0.837	0	T Cells
MCMBP	0	0.42309021	0.268	0.812	0	T Cells
LIN7C	0	0.25219737	0.211	0.755	0	T Cells
MIDN2	0	0.21525007	0.233	0.777	0	T Cells
RAB11B	0	0.4351324	0.26	0.804	0	T Cells
ACTR33	0	0.29457735	0.224	0.767	0	T Cells
ETV65	0	0.11667732	0.182	0.725	0	T Cells
EFCAB14	0	0.4802636	0.294	0.836	0	T Cells
CD2	0	6.62304194	0.644	0.102	0	T Cells
SHC1	0	0.29930519	0.207	0.749	0	T Cells
TMEM1092	0	0.32714066	0.231	0.773	0	T Cells
HNRNPUL2	0	0.4334774	0.259	0.801	0	T Cells
RBM232	0	0.15040491	0.199	0.741	0	T Cells
MYL12A	0	0.31200332	0.255	0.797	0	T Cells
MRTFA3	0	0.22199629	0.21	0.752	0	T Cells
NRDC2	0	0.44742507	0.248	0.789	0	T Cells
DDX241	0	0.47502327	0.28	0.821	0	T Cells
EIF2A1	0	0.34177585	0.225	0.765	0	T Cells
SURF11	0	0.24834253	0.189	0.729	0	T Cells
ZFP911	0	0.22412888	0.199	0.739	0	T Cells
CALM11	0	0.2371465	0.225	0.765	0	T Cells
SLC25A63	0	0.31426443	0.231	0.771	0	T Cells
GMIP1	0	0.25092764	0.221	0.76	0	T Cells
CDC42EP32	0	0.34405995	0.287	0.826	0	T Cells
HIPK33	0	0.52109319	0.333	0.872	0	T Cells
HMOX2	0	0.38796996	0.251	0.789	0	T Cells
UBL31	0	0.38611724	0.236	0.773	0	T Cells
TXNL4A	0	0.40477294	0.264	0.801	0	T Cells
UBE2D32	0	0.51729716	0.292	0.829	0	T Cells
TCF252	0	0.53856146	0.324	0.861	0	T Cells
ARMC8	0	0.14800379	0.183	0.719	0	T Cells

ARCN1	0	0.47296442	0.233	0.769	0	T Cells
SRP54	0	0.22741742	0.193	0.729	0	T Cells
EXOC31	0	0.49574756	0.26	0.795	0	T Cells
SNX61	0	0.43186427	0.236	0.771	0	T Cells
JOSD12	0	0.24448393	0.213	0.748	0	T Cells
TM9SF32	0	0.57608916	0.325	0.86	0	T Cells
RNF112	0	0.18732778	0.189	0.723	0	T Cells
ENSA	0	0.32069577	0.212	0.746	0	T Cells
NHP21	0	0.27503301	0.192	0.726	0	T Cells
ELMSAN12	0	0.3973997	0.24	0.774	0	T Cells
NDUFS12	0	0.26190478	0.207	0.74	0	T Cells
SLC44A21	0	0.14538241	0.247	0.78	0	T Cells
DNAJC53	0	0.27309413	0.216	0.749	0	T Cells
YTHDF2	0	0.24679667	0.192	0.725	0	T Cells
PI4KB	0	0.15734921	0.185	0.718	0	T Cells
OGDH1	0	0.5217443	0.296	0.829	0	T Cells
PRNP1	0	0.44922846	0.328	0.861	0	T Cells
CERS21	0	0.57217056	0.3	0.832	0	T Cells
TSR3	0	0.20656906	0.185	0.717	0	T Cells
COPE1	0	0.31052907	0.206	0.738	0	T Cells
PTPN14	0	0.32623885	0.283	0.815	0	T Cells
EIF1B	0	0.46077454	0.235	0.766	0	T Cells
CCL5	0	9.64132369	0.541	0.01	0	T Cells
GSK3A1	0	0.3038251	0.205	0.736	0	T Cells
STT3B3	0	0.63910787	0.32	0.851	0	T Cells
TLNRD1	0	0.10137362	0.191	0.722	0	T Cells
RIOK31	0	0.19486702	0.2	0.731	0	T Cells
PUM11	0	0.63525847	0.285	0.815	0	T Cells
SNX141	0	0.14158554	0.181	0.711	0	T Cells
NUB12	0	0.36592322	0.224	0.754	0	T Cells
GSR	0	0.1924885	0.194	0.724	0	T Cells
DCAF52	0	0.1720072	0.189	0.719	0	T Cells
PTPN9	0	0.25802426	0.189	0.719	0	T Cells
PPP6R1	0	0.53797015	0.299	0.829	0	T Cells
IMMT1	0	0.42804343	0.23	0.759	0	T Cells
ST6GALNAC62	0	0.35355387	0.257	0.786	0	T Cells
JMJD1C2	0	0.43059998	0.25	0.779	0	T Cells
BCL2L14	0	0.38300112	0.253	0.782	0	T Cells
COX7C	0	0.53955639	0.286	0.815	0	T Cells
UBE2B2	0	0.62929138	0.284	0.813	0	T Cells
FAM102B2	0	0.13202726	0.183	0.711	0	T Cells
NUDT33	0	0.38080947	0.217	0.745	0	T Cells
CYB5R43	0	0.27678409	0.211	0.739	0	T Cells
TMEM248	0	0.59810398	0.319	0.847	0	T Cells
PSMC51	0	0.40196553	0.222	0.75	0	T Cells
RNF19B3	0	0.15135062	0.208	0.735	0	T Cells
STT3A	0	0.34989576	0.208	0.735	0	T Cells
UBE2I1	0	0.46852876	0.249	0.776	0	T Cells
CYBC1	0	0.45980783	0.247	0.774	0	T Cells
UBXN41	0	0.63882597	0.305	0.832	0	T Cells
FNDC3A1	0	0.12490755	0.181	0.708	0	T Cells
ZNF4101	0	0.24284675	0.203	0.73	0	T Cells

VPS4A	0	0.21645098	0.192	0.719	0	T Cells
APEH1	0	0.23383399	0.185	0.711	0	T Cells
CDK9	0	0.51648741	0.268	0.794	0	T Cells
COPB1	0	0.49769152	0.233	0.759	0	T Cells
SUSD62	0	0.38396254	0.239	0.765	0	T Cells
SRSF2	0	0.36098933	0.211	0.737	0	T Cells
EIF3L2	0	0.39708164	0.243	0.769	0	T Cells
CHURC12	0	0.61924843	0.282	0.807	0	T Cells
ERAP1	0	0.44876913	0.255	0.78	0	T Cells
EIF3B	0	0.44651997	0.235	0.76	0	T Cells
MAP1LC3B1	0	0.3423221	0.214	0.739	0	T Cells
DUS1L	0	0.44290903	0.226	0.751	0	T Cells
GAK	0	0.35383572	0.213	0.737	0	T Cells
SFXN32	0	0.18788758	0.191	0.715	0	T Cells
ZNF5922	0	0.21971603	0.195	0.719	0	T Cells
RNF401	0	0.32122381	0.195	0.719	0	T Cells
TMUB21	0	0.3158564	0.208	0.731	0	T Cells
SNRPB21	0	0.21539346	0.174	0.697	0	T Cells
MAPKAPK21	0	0.68772705	0.329	0.851	0	T Cells
DNMT3A2	0	0.10005623	0.192	0.714	0	T Cells
TIPARP3	0	0.13844518	0.176	0.698	0	T Cells
DHRS7	0	0.47948014	0.245	0.767	0	T Cells
TMCO11	0	0.58931788	0.266	0.787	0	T Cells
SPG111	0	0.39405233	0.236	0.757	0	T Cells
PML2	0	0.24270632	0.207	0.728	0	T Cells
GPBP1L1	0	0.73665731	0.313	0.833	0	T Cells
DEDD3	0	0.12553944	0.175	0.695	0	T Cells
DDX213	0	0.44671228	0.221	0.741	0	T Cells
ARHGAP1	0	0.2571059	0.203	0.723	0	T Cells
ZBTB13	0	0.40458294	0.302	0.822	0	T Cells
EXOC71	0	0.45418251	0.225	0.744	0	T Cells
FAM120A3	0	0.73660504	0.323	0.842	0	T Cells
FAR13	0	0.24682773	0.19	0.709	0	T Cells
TBC1D152	0	0.18593302	0.179	0.698	0	T Cells
IDH3B1	0	0.12372275	0.17	0.689	0	T Cells
TOP1	0	0.12766559	0.174	0.693	0	T Cells
TOMM201	0	0.50572006	0.239	0.757	0	T Cells
PRPF40A1	0	0.37758684	0.201	0.719	0	T Cells
STAT11	0	0.59751319	0.342	0.86	0	T Cells
NCL	0	0.52759234	0.233	0.751	0	T Cells
IRF21	0	0.28448405	0.191	0.709	0	T Cells
OXA1L3	0	0.60020042	0.28	0.798	0	T Cells
TMC6	0	0.61069528	0.317	0.835	0	T Cells
XBP11	0	0.3448575	0.26	0.778	0	T Cells
SNU13	0	0.69129175	0.322	0.84	0	T Cells
ACBD3	0	0.15346963	0.165	0.682	0	T Cells
BTBD12	0	0.1633464	0.175	0.692	0	T Cells
FAM174C	0	0.2938235	0.19	0.707	0	T Cells
ERLEC11	0	0.34126692	0.19	0.706	0	T Cells
UQCRB	0	0.47978894	0.228	0.744	0	T Cells
UBE2Z2	0	0.25818947	0.188	0.704	0	T Cells
RAP2C3	0	0.44087896	0.231	0.747	0	T Cells

UFM11	0	0.72263155	0.282	0.797	0	T Cells
EID1	0	0.59182061	0.296	0.811	0	T Cells
NPLOC42	0	0.67280144	0.293	0.808	0	T Cells
ROCK12	0	0.68815213	0.287	0.802	0	T Cells
DNAJC81	0	0.41805356	0.207	0.722	0	T Cells
UBE2Q11	0	0.51432222	0.236	0.751	0	T Cells
NDUFA102	0	0.61182583	0.26	0.775	0	T Cells
STIMATE2	0	0.18534049	0.207	0.722	0	T Cells
EIF2AK1	0	0.44689643	0.221	0.736	0	T Cells
EIF3H1	0	0.55728431	0.248	0.763	0	T Cells
HNRNPK1	0	0.45251517	0.22	0.735	0	T Cells
CD3E	0	10.1506529	0.518	0.003	0	T Cells
RAB5B1	0	0.42366343	0.208	0.723	0	T Cells
SMC1A1	0	0.5423159	0.266	0.781	0	T Cells
SERBP11	0	0.53211619	0.225	0.739	0	T Cells
TMEM167A2	0	0.33465952	0.198	0.712	0	T Cells
ILRUN1	0	0.54384206	0.241	0.755	0	T Cells
SETD3	0	0.70475402	0.277	0.791	0	T Cells
MED15	0	0.62606816	0.253	0.767	0	T Cells
SH3BP1	0	0.49071728	0.298	0.811	0	T Cells
TMEM167B1	0	0.1663643	0.17	0.683	0	T Cells
MOGS	0	0.36793794	0.204	0.717	0	T Cells
E2F43	0	0.49832191	0.226	0.739	0	T Cells
SUPT5H	0	0.61123004	0.247	0.76	0	T Cells
MIA3	0	0.39267727	0.193	0.706	0	T Cells
NUCKS11	0	0.22107348	0.188	0.7	0	T Cells
MGAT4B2	0	0.1890404	0.178	0.69	0	T Cells
CBL2	0	0.16729668	0.186	0.698	0	T Cells
MESD	0	0.14448198	0.159	0.671	0	T Cells
HERPUD13	0	0.4689359	0.245	0.757	0	T Cells
GLIPR13	0	0.45694483	0.294	0.805	0	T Cells
GNAI34	0	0.6440158	0.269	0.779	0	T Cells
VPS451	0	0.13699405	0.169	0.679	0	T Cells
FOSL23	0	0.39090594	0.285	0.795	0	T Cells
SNX201	0	0.48393325	0.247	0.757	0	T Cells
CALM3	0	0.33104395	0.213	0.723	0	T Cells
TRABD3	0	0.53042623	0.235	0.745	0	T Cells
BCL7B1	0	0.20873068	0.178	0.687	0	T Cells
NOTCH11	0	0.53177671	0.263	0.772	0	T Cells
RECQL1	0	0.44865614	0.208	0.717	0	T Cells
RASSF3	0	0.58677645	0.261	0.77	0	T Cells
STRAP1	0	0.48547194	0.2	0.709	0	T Cells
DPYD3	0	0.58098082	0.273	0.781	0	T Cells
DHX9	0	0.40522364	0.199	0.707	0	T Cells
LPP3	0	0.28281897	0.193	0.701	0	T Cells
FDFT11	0	0.11564886	0.211	0.719	0	T Cells
ITCH1	0	0.63642692	0.257	0.765	0	T Cells
UXT	0	0.69265639	0.281	0.789	0	T Cells
NSMCE11	0	0.22513532	0.177	0.684	0	T Cells
VPS26C1	0	0.2853969	0.175	0.682	0	T Cells
ATP5PB	0	0.14446106	0.17	0.677	0	T Cells
RBX1	0	0.16564043	0.167	0.674	0	T Cells

ABRACL1	0	0.67548382	0.317	0.824	0	T Cells
RDH11	0	0.29796129	0.193	0.7	0	T Cells
ATF63	0	0.12399934	0.155	0.661	0	T Cells
NDUFA5	0	0.37088833	0.188	0.694	0	T Cells
YTHDF32	0	0.42569124	0.199	0.705	0	T Cells
FUBP3	0	0.13623931	0.153	0.659	0	T Cells
PSMD132	0	0.42581066	0.206	0.712	0	T Cells
MBD61	0	0.26376613	0.187	0.693	0	T Cells
UBC2	0	0.43251642	0.233	0.739	0	T Cells
RETREG3	0	0.36373077	0.189	0.695	0	T Cells
TBCB1	0	0.68824921	0.298	0.804	0	T Cells
SLC66A32	0	0.14620442	0.172	0.677	0	T Cells
MAPK1IP1L	0	0.70896673	0.278	0.783	0	T Cells
DDB1	0	0.32741559	0.191	0.696	0	T Cells
PPIB2	0	0.31668041	0.191	0.696	0	T Cells
MTMR31	0	0.26408369	0.186	0.691	0	T Cells
SSR2	0	0.73972164	0.318	0.822	0	T Cells
ECPAS1	0	0.50079606	0.21	0.714	0	T Cells
CWC151	0	0.37959235	0.185	0.689	0	T Cells
USP324	0	0.11825438	0.164	0.668	0	T Cells
AFF41	0	0.3007208	0.174	0.677	0	T Cells
VPS4B	0	0.32044197	0.184	0.687	0	T Cells
DDX1	0	0.22161572	0.166	0.669	0	T Cells
CELF11	0	0.56621963	0.268	0.771	0	T Cells
ACTR101	0	0.13845712	0.158	0.661	0	T Cells
HNRNPU1	0	0.78763372	0.316	0.819	0	T Cells
EIF2S11	0	0.40835325	0.189	0.692	0	T Cells
IKBKG2	0	0.27416608	0.187	0.69	0	T Cells
HYOU13	0	0.36308798	0.205	0.707	0	T Cells
SECISBP2L	0	0.27365447	0.176	0.678	0	T Cells
HMGN11	0	0.40686912	0.217	0.719	0	T Cells
ACBD5	0	0.12522206	0.158	0.659	0	T Cells
IL2RB	0	10.3852865	0.504	0.003	0	T Cells
SFT2D22	0	0.47670313	0.233	0.733	0	T Cells
ABCF3	0	0.14363423	0.15	0.65	0	T Cells
REXO21	0	0.78832056	0.284	0.784	0	T Cells
HOOK32	0	0.2606392	0.174	0.673	0	T Cells
CUTA	0	0.62648766	0.238	0.737	0	T Cells
AATF	0	0.60145779	0.229	0.728	0	T Cells
SEC11C	0	0.51621455	0.226	0.725	0	T Cells
ATP9B1	0	0.3477791	0.208	0.707	0	T Cells
EIF4G22	0	0.38967019	0.198	0.697	0	T Cells
SAR1B	0	0.28081487	0.172	0.67	0	T Cells
UHMK11	0	0.68537052	0.257	0.755	0	T Cells
PPIG1	0	0.69573103	0.237	0.735	0	T Cells
PLEKHA2	0	0.67180152	0.27	0.768	0	T Cells
RABL6	0	0.26132159	0.171	0.669	0	T Cells
KLF32	0	0.1809394	0.151	0.648	0	T Cells
NDUFB8	0	0.47776945	0.203	0.7	0	T Cells
MAST32	0	0.30362453	0.194	0.691	0	T Cells
REL4	0	0.49911146	0.229	0.725	0	T Cells
TMEM2581	0	0.16176888	0.163	0.659	0	T Cells

CPSF21	0	0.27764342	0.167	0.663	0	T Cells
CORO71	0	0.6360239	0.259	0.755	0	T Cells
ADSL2	0	0.41166008	0.202	0.698	0	T Cells
RTF11	0	0.38453519	0.183	0.678	0	T Cells
UBE2G21	0	0.35609578	0.178	0.673	0	T Cells
WDFY1	0	0.16792145	0.156	0.651	0	T Cells
FUT111	0	0.21235447	0.16	0.655	0	T Cells
VMP14	0	0.51442418	0.264	0.759	0	T Cells
SLAIN21	0	0.48846643	0.201	0.696	0	T Cells
GNL1	0	0.20383098	0.161	0.655	0	T Cells
YKT62	0	0.58531821	0.215	0.709	0	T Cells
FBXW52	0	0.55394696	0.224	0.718	0	T Cells
CLEC2D3	0	7.3841658	0.527	0.033	0	T Cells
CXorf382	0	0.11904191	0.157	0.651	0	T Cells
USP331	0	0.40751521	0.189	0.682	0	T Cells
ATP1A1	0	0.47526586	0.207	0.7	0	T Cells
CRK3	0	0.27572447	0.169	0.662	0	T Cells
ATP5F1D1	0	0.44623737	0.208	0.701	0	T Cells
VOPP11	0	0.12003767	0.155	0.647	0	T Cells
ST3GAL1	0	0.69853891	0.28	0.772	0	T Cells
UBLCP11	0	0.46367753	0.191	0.682	0	T Cells
AIP	0	0.73874545	0.296	0.787	0	T Cells
PDCD101	0	0.54591711	0.216	0.707	0	T Cells
RASGRP21	0	0.35389226	0.211	0.702	0	T Cells
ABCC1	0	0.58294359	0.22	0.711	0	T Cells
KCNA31	0	0.34127485	0.188	0.678	0	T Cells
CCT6A3	0	0.27770618	0.172	0.662	0	T Cells
MGAT5	0	0.5104382	0.211	0.701	0	T Cells
PARP142	0	0.45231226	0.245	0.735	0	T Cells
MROH1	0	0.14606175	0.167	0.657	0	T Cells
CHMP1A1	0	0.10073658	0.15	0.64	0	T Cells
TMEM1891	0	0.2698355	0.166	0.656	0	T Cells
BROX1	0	0.52500695	0.199	0.689	0	T Cells
IWS1	0	0.50107507	0.201	0.691	0	T Cells
NUFIP24	0	0.92510475	0.3	0.789	0	T Cells
ZC3H151	0	0.53348244	0.204	0.693	0	T Cells
SCYL11	0	0.19752014	0.166	0.655	0	T Cells
MCRIP1	0	0.30669209	0.168	0.657	0	T Cells
ELMO21	0	0.27799576	0.171	0.66	0	T Cells
IFNAR12	0	0.29329302	0.167	0.656	0	T Cells
BSDC1	0	0.48205688	0.193	0.681	0	T Cells
BCL102	0	0.20691097	0.155	0.643	0	T Cells
EDF1	0	0.63711567	0.23	0.718	0	T Cells
VPS11	0	0.12723329	0.155	0.643	0	T Cells
UQCRC21	0	0.16414848	0.149	0.637	0	T Cells
STMP12	0	0.31948144	0.175	0.662	0	T Cells
ILF32	0	0.78080645	0.29	0.777	0	T Cells
COPS51	0	0.32420333	0.17	0.657	0	T Cells
BABAM1	0	0.10448532	0.144	0.631	0	T Cells
NMI2	0	0.27935217	0.173	0.659	0	T Cells
AP3B12	0	0.51908788	0.2	0.686	0	T Cells
KDM6B1	0	0.24206663	0.171	0.657	0	T Cells

ITPR22	0	0.3458382	0.203	0.689	0	T Cells
ZBTB7B2	0	0.38900905	0.193	0.678	0	T Cells
MFF1	0	0.46705958	0.182	0.667	0	T Cells
SOD11	0	0.30725665	0.175	0.66	0	T Cells
CPNE3	0	0.36884931	0.173	0.657	0	T Cells
VPS392	0	0.33529548	0.174	0.658	0	T Cells
UBR41	0	0.72917061	0.249	0.733	0	T Cells
SPCS31	0	0.67077512	0.217	0.701	0	T Cells
HEXIM1	0	0.22358858	0.166	0.65	0	T Cells
ZNFX1	0	0.31121965	0.172	0.655	0	T Cells
DCAF82	0	0.67097925	0.221	0.704	0	T Cells
RRAGA	0	0.11635874	0.138	0.621	0	T Cells
UBXN12	0	0.67599761	0.242	0.725	0	T Cells
NIN	0	0.62425062	0.207	0.69	0	T Cells
HGS3	0	0.64434284	0.232	0.715	0	T Cells
ASCC2	0	0.37075132	0.167	0.65	0	T Cells
GLUD12	0	0.38421063	0.182	0.664	0	T Cells
APOL21	0	0.36135898	0.199	0.681	0	T Cells
EPRS2	0	0.69555957	0.221	0.703	0	T Cells
GALNS1	0	0.15552199	0.16	0.642	0	T Cells
SMDT12	0	0.69277739	0.224	0.706	0	T Cells
YIPF5	0	0.43407175	0.182	0.663	0	T Cells
LARP1	0	0.39985293	0.176	0.657	0	T Cells
USP141	0	0.37511775	0.176	0.657	0	T Cells
PPP2R5A	0	0.73178851	0.243	0.724	0	T Cells
RBMS11	0	0.76468284	0.246	0.727	0	T Cells
UBA7	0	0.20706232	0.168	0.649	0	T Cells
SUPT6H1	0	0.62014606	0.214	0.695	0	T Cells
HNRNPL	0	0.6334722	0.221	0.702	0	T Cells
FBXO42	0	0.17160617	0.147	0.627	0	T Cells
DGUOK1	0	0.47187581	0.188	0.667	0	T Cells
GRIPAP11	0	0.39133495	0.186	0.665	0	T Cells
PHC24	0	0.12684817	0.146	0.625	0	T Cells
THRAP31	0	0.81459001	0.265	0.744	0	T Cells
RAB5A2	0	0.71588046	0.225	0.704	0	T Cells
RCC1L	0	0.18583739	0.152	0.631	0	T Cells
TRAPPC4	0	0.54527634	0.216	0.695	0	T Cells
FLI11	0	0.79812369	0.265	0.744	0	T Cells
PRPF6	0	0.74603462	0.241	0.72	0	T Cells
TMEM8A2	0	0.3516066	0.184	0.662	0	T Cells
LGALS83	0	0.72498505	0.244	0.722	0	T Cells
SCFD11	0	0.17509128	0.147	0.625	0	T Cells
SOS22	0	0.24174989	0.164	0.642	0	T Cells
CORO1A4	0	5.33798484	0.67	0.193	0	T Cells
WDR261	0	0.79982706	0.276	0.753	0	T Cells
SRSF41	0	0.87456785	0.284	0.76	0	T Cells
STXBP31	0	0.69954735	0.226	0.702	0	T Cells
HMCES1	0	0.15861784	0.16	0.636	0	T Cells
GOLGA72	0	0.76468034	0.229	0.705	0	T Cells
SETX1	0	0.85011148	0.281	0.757	0	T Cells
PHPT1	0	0.16064644	0.164	0.64	0	T Cells
PRKAG12	0	0.10453028	0.139	0.615	0	T Cells

FMNL32	0	0.46051193	0.206	0.681	0	T Cells
PRPF8	0	0.5610018	0.188	0.663	0	T Cells
DENND2D	0	0.68877042	0.245	0.72	0	T Cells
SP12	0	0.82894052	0.262	0.737	0	T Cells
SELENOK1	0	0.43526493	0.192	0.666	0	T Cells
SRP722	0	0.43149891	0.182	0.656	0	T Cells
SPRYD3	0	0.11607862	0.14	0.614	0	T Cells
HNRNPC1	0	0.73262465	0.242	0.716	0	T Cells
GALNT16	0	0.18104256	0.149	0.623	0	T Cells
TGFB11	0	0.68309035	0.23	0.704	0	T Cells
NPEPPS3	0	0.51100429	0.192	0.665	0	T Cells
CD96	0	10.2082412	0.476	0.003	0	T Cells
SEC16A	0	0.25833575	0.164	0.637	0	T Cells
TUBGCP22	0	0.1380962	0.142	0.615	0	T Cells
CCNY1	0	0.61221562	0.208	0.68	0	T Cells
GATAD2A	0	0.55839041	0.203	0.675	0	T Cells
IK1	0	0.85339973	0.256	0.728	0	T Cells
ARRDC1	0	0.1921929	0.155	0.627	0	T Cells
SHOC21	0	0.8626403	0.255	0.727	0	T Cells
SNX191	0	0.37600427	0.165	0.637	0	T Cells
POLR1D1	0	0.74503807	0.243	0.715	0	T Cells
ATP13A11	0	0.16515732	0.153	0.625	0	T Cells
TDP21	0	0.50983204	0.191	0.662	0	T Cells
SND12	0	0.68377629	0.198	0.669	0	T Cells
DR11	0	0.72073028	0.23	0.701	0	T Cells
TRIM261	0	0.3903628	0.162	0.633	0	T Cells
DYNLT12	0	0.73030606	0.237	0.708	0	T Cells
EPSTI11	0	0.40632716	0.219	0.69	0	T Cells
ADCY74	0	0.69802247	0.23	0.701	0	T Cells
MAP2K32	0	0.17858786	0.156	0.627	0	T Cells
SLC1A52	0	0.68073749	0.229	0.7	0	T Cells
HM132	0	0.47957857	0.207	0.677	0	T Cells
SNRNP401	0	0.53664818	0.187	0.657	0	T Cells
SLU71	0	0.55415975	0.19	0.66	0	T Cells
SP1101	0	0.79259565	0.269	0.739	0	T Cells
DDX46	0	0.80590488	0.248	0.718	0	T Cells
LARS4	0	0.86379617	0.253	0.723	0	T Cells
TAP22	0	0.80812883	0.267	0.737	0	T Cells
ORAI21	0	0.23793255	0.17	0.64	0	T Cells
TMEM134	0	0.14094933	0.143	0.613	0	T Cells
RAB8B1	0	0.87560608	0.248	0.718	0	T Cells
B4GALT51	0	0.27268611	0.159	0.629	0	T Cells
LRPPRC	0	0.72567514	0.2	0.669	0	T Cells
PPP4R3B	0	0.90497309	0.284	0.753	0	T Cells
MIF	0	0.86374956	0.284	0.753	0	T Cells
SRRM11	0	0.7590971	0.234	0.703	0	T Cells
ACTR1B	0	0.75573155	0.253	0.722	0	T Cells
TSN	0	0.26948409	0.151	0.62	0	T Cells
POLE31	0	0.23594778	0.155	0.623	0	T Cells
KTN1	0	0.76625734	0.234	0.702	0	T Cells
BTBD2	0	0.17998175	0.139	0.607	0	T Cells
SIN3B2	0	0.20287968	0.158	0.626	0	T Cells

FURIN4	0	0.74140099	0.207	0.674	0	T Cells
TMED4	0	0.74518161	0.224	0.691	0	T Cells
PPIA2	0	0.12679631	0.154	0.621	0	T Cells
RANBP3	0	0.43294975	0.171	0.638	0	T Cells
FKBP52	0	0.62502192	0.21	0.676	0	T Cells
VPS26B	0	0.6290763	0.194	0.66	0	T Cells
ETNK1	0	0.5876895	0.206	0.671	0	T Cells
ZDHHC25	0	0.5091907	0.197	0.662	0	T Cells
TRAPPC14	0	0.37980551	0.165	0.63	0	T Cells
RAB22A2	0	0.63015295	0.187	0.651	0	T Cells
IGBP12	0	0.60115149	0.2	0.664	0	T Cells
PLRG1	0	0.34239384	0.163	0.627	0	T Cells
TMEM2431	0	0.41611429	0.165	0.629	0	T Cells
ME22	0	0.17589096	0.141	0.605	0	T Cells
KDSR1	0	0.73751195	0.21	0.673	0	T Cells
SACM1L	0	0.92759709	0.272	0.735	0	T Cells
SMARCA51	0	0.92088187	0.26	0.723	0	T Cells
BRMS1	0	0.14135485	0.147	0.61	0	T Cells
DNM1L3	0	0.13100508	0.148	0.611	0	T Cells
CCNK1	0	0.34331361	0.156	0.619	0	T Cells
CMC21	0	0.40086625	0.163	0.626	0	T Cells
ARFGAP32	0	0.3853978	0.168	0.631	0	T Cells
LYST2	0	0.33908863	0.18	0.642	0	T Cells
UGGT1	0	0.45993733	0.174	0.636	0	T Cells
WIPI22	0	0.32862474	0.161	0.623	0	T Cells
ZNRF23	0	0.73318596	0.23	0.692	0	T Cells
TMX13	0	0.31178777	0.151	0.613	0	T Cells
LONP1	0	0.34730239	0.143	0.605	0	T Cells
PPM1B1	0	0.51096563	0.173	0.634	0	T Cells
LARP71	0	0.27052593	0.144	0.605	0	T Cells
COPS61	0	0.23011972	0.149	0.61	0	T Cells
SIPA15	0	0.77456333	0.254	0.715	0	T Cells
NAA601	0	0.38162339	0.165	0.626	0	T Cells
SUMO21	0	0.40295124	0.172	0.632	0	T Cells
OXSR12	0	0.13669936	0.148	0.608	0	T Cells
GRSF1	0	0.15653479	0.143	0.603	0	T Cells
DNAJC43	0	0.12402611	0.14	0.6	0	T Cells
RSRC22	0	0.91605198	0.27	0.73	0	T Cells
HUWE1	0	0.96600619	0.278	0.738	0	T Cells
CLIP12	0	0.64723677	0.207	0.666	0	T Cells
KPNA6	0	0.64449785	0.194	0.653	0	T Cells
GIT21	0	0.55781195	0.192	0.651	0	T Cells
MOV102	0	0.11409593	0.141	0.6	0	T Cells
IRF2BP24	0	0.86501221	0.255	0.714	0	T Cells
CAB391	0	0.89908541	0.262	0.721	0	T Cells
BAK11	0	0.31718589	0.155	0.614	0	T Cells
MEPCE	0	0.33501396	0.165	0.624	0	T Cells
RNF20	0	0.14381859	0.133	0.592	0	T Cells
MGAT22	0	0.75696775	0.232	0.691	0	T Cells
WWP21	0	0.21302758	0.141	0.6	0	T Cells
API5	0	0.53678056	0.179	0.637	0	T Cells
MTIF32	0	0.48075785	0.168	0.626	0	T Cells

APRT2	0	0.81735283	0.239	0.697	0	T Cells
NDUFB111	0	0.32807265	0.154	0.612	0	T Cells
CDV31	0	0.7112045	0.212	0.669	0	T Cells
POLR2B	0	0.84653465	0.226	0.683	0	T Cells
SRSF31	0	0.78772946	0.238	0.695	0	T Cells
PRPF19	0	0.32909871	0.148	0.605	0	T Cells
GSK3B1	0	0.56862263	0.186	0.642	0	T Cells
DCAF111	0	0.47906232	0.173	0.629	0	T Cells
NUDT222	0	0.24699504	0.152	0.608	0	T Cells
DICER14	0	0.44260891	0.168	0.624	0	T Cells
ANKRD402	0	0.13257211	0.129	0.585	0	T Cells
SAFB3	0	0.82355661	0.236	0.692	0	T Cells
NPEPL11	0	0.41073879	0.169	0.625	0	T Cells
PSME41	0	0.65380073	0.229	0.684	0	T Cells
AIMP11	0	0.78489118	0.207	0.662	0	T Cells
LZIC	0	0.68480904	0.189	0.644	0	T Cells
DHX361	0	0.90978159	0.262	0.717	0	T Cells
PSMD92	0	0.41994348	0.166	0.621	0	T Cells
GADD45GIP1	0	0.26549162	0.145	0.6	0	T Cells
CXCR44	0	4.82925778	0.796	0.342	0	T Cells
ZDHHC203	0	0.69476334	0.2	0.654	0	T Cells
PIP4P12	0	0.61874996	0.188	0.642	0	T Cells
SSU721	0	0.41912294	0.157	0.611	0	T Cells
AFF14	0	0.93900162	0.256	0.71	0	T Cells
USP152	0	0.84634948	0.25	0.704	0	T Cells
NUDT211	0	0.92459064	0.259	0.713	0	T Cells
EGLN21	0	0.46120435	0.171	0.625	0	T Cells
HNRNPA33	0	0.73276374	0.225	0.678	0	T Cells
LY751	0	0.54964269	0.189	0.642	0	T Cells
SEC22C1	0	0.51478757	0.18	0.633	0	T Cells
ZC3H4	0	0.59696537	0.189	0.642	0	T Cells
SMIM122	0	0.41881979	0.164	0.617	0	T Cells
STX51	0	0.44235971	0.162	0.615	0	T Cells
R3HDM21	0	0.42679375	0.171	0.624	0	T Cells
VHL1	0	0.74428492	0.21	0.662	0	T Cells
UBA31	0	0.59579276	0.181	0.633	0	T Cells
PIAS12	0	0.26734119	0.147	0.599	0	T Cells
ANKRD172	0	0.71372295	0.21	0.661	0	T Cells
USP312	0	0.5661568	0.198	0.649	0	T Cells
SPAG92	0	0.65101494	0.188	0.639	0	T Cells
JTB	0	0.43024316	0.16	0.611	0	T Cells
GORASP2	0	0.51820083	0.156	0.607	0	T Cells
POLR2G2	0	0.45948112	0.171	0.622	0	T Cells
ATP2A21	0	0.18427132	0.133	0.584	0	T Cells
FNTA1	0	0.76752788	0.214	0.664	0	T Cells
TENT23	0	0.66338103	0.185	0.635	0	T Cells
C19orf53	0	0.59704643	0.181	0.631	0	T Cells
GTF3C61	0	0.34294151	0.154	0.604	0	T Cells
ZNHIT1	0	0.24424305	0.145	0.595	0	T Cells
EMG1	0	0.32546352	0.145	0.595	0	T Cells
TBC1D10B	0	0.10240437	0.135	0.585	0	T Cells
MFNG	0	0.81170185	0.241	0.691	0	T Cells

CUL3	0	0.83353082	0.215	0.664	0	T Cells
C6orf624	0	0.7729752	0.225	0.674	0	T Cells
AFTPH2	0	0.74492921	0.2	0.649	0	T Cells
WASHC31	0	0.31871189	0.146	0.595	0	T Cells
SLC35C2	0	0.18171574	0.138	0.587	0	T Cells
ZBTB331	0	0.124968	0.129	0.578	0	T Cells
HBP11	0	0.6878244	0.213	0.661	0	T Cells
THOC71	0	0.32012838	0.152	0.6	0	T Cells
WAPL1	0	0.4305436	0.156	0.604	0	T Cells
DAP32	0	0.64639941	0.191	0.638	0	T Cells
PRKDC	0	0.63362279	0.199	0.646	0	T Cells
SIVA11	0	0.63404139	0.191	0.638	0	T Cells
GNL22	0	0.23385272	0.139	0.586	0	T Cells
PABPC42	0	0.19613541	0.138	0.585	0	T Cells
AGGF12	0	0.28762508	0.144	0.591	0	T Cells
CUL2	0	0.19001104	0.135	0.582	0	T Cells
IRF75	0	0.74817563	0.27	0.717	0	T Cells
SMAD71	0	0.83511136	0.266	0.713	0	T Cells
DNTTIP21	0	0.63539524	0.186	0.632	0	T Cells
ISY13	0	0.47446947	0.167	0.613	0	T Cells
ZFR1	0	1.02538471	0.273	0.719	0	T Cells
MALSU1	0	0.32503113	0.127	0.573	0	T Cells
TERF2IP1	0	0.92289997	0.252	0.698	0	T Cells
UBE2V12	0	0.90702487	0.248	0.694	0	T Cells
MED282	0	0.82864552	0.233	0.678	0	T Cells
DPM1	0	0.70272175	0.185	0.63	0	T Cells
NABP13	0	0.4848441	0.22	0.664	0	T Cells
TXNDC151	0	0.81135769	0.211	0.655	0	T Cells
CCDC85B	0	0.51122649	0.189	0.633	0	T Cells
XAB21	0	0.7221883	0.194	0.638	0	T Cells
UXS1	0	0.30157836	0.146	0.59	0	T Cells
OPA12	0	0.41622516	0.15	0.594	0	T Cells
EHMT11	0	0.95430604	0.268	0.712	0	T Cells
AP2A22	0	0.33738006	0.152	0.596	0	T Cells
CASC33	0	0.99739656	0.258	0.702	0	T Cells
CD584	0	0.7470652	0.228	0.671	0	T Cells
RMND5A2	0	0.77971339	0.209	0.652	0	T Cells
TMEM250	0	0.68869068	0.201	0.644	0	T Cells
KHDRBS1	0	0.52154487	0.168	0.611	0	T Cells
PIP4K2C	0	0.38005405	0.149	0.592	0	T Cells
ERP29	0	0.36553932	0.156	0.599	0	T Cells
ANKRD103	0	0.76541534	0.254	0.697	0	T Cells
SNRPB1	0	0.44819085	0.171	0.614	0	T Cells
RALY1	0	0.37776152	0.152	0.595	0	T Cells
MAP3K53	0	0.50484589	0.177	0.619	0	T Cells
SRM	0	0.30439699	0.146	0.588	0	T Cells
DYNC1LI14	0	0.2807106	0.14	0.582	0	T Cells
TMEM106B	0	0.47869844	0.157	0.599	0	T Cells
PAPOLA1	0	0.56820698	0.162	0.604	0	T Cells
PSMD121	0	0.42970593	0.152	0.594	0	T Cells
KCMF11	0	0.82064322	0.215	0.656	0	T Cells
HINT1	0	0.84349233	0.222	0.663	0	T Cells

KDM3B1	0	0.61613601	0.178	0.619	0 T Cells
MSL12	0	0.55677394	0.185	0.626	0 T Cells
DVL33	0	0.36555641	0.151	0.592	0 T Cells
SF3B2	0	0.49623584	0.155	0.596	0 T Cells
CDK41	0	0.1959257	0.133	0.574	0 T Cells
METAP21	0	0.4310551	0.151	0.592	0 T Cells
UBA61	0	0.53360896	0.173	0.613	0 T Cells
EDEM32	0	0.47160277	0.159	0.599	0 T Cells
HAT11	0	0.29459316	0.137	0.577	0 T Cells
ZMYND11	0	0.52452114	0.166	0.606	0 T Cells
LONP21	0	0.52491868	0.164	0.604	0 T Cells
VMA211	0	0.44764592	0.159	0.599	0 T Cells
TGFBR13	0	0.82259642	0.227	0.666	0 T Cells
MAN2A21	0	0.7669364	0.225	0.664	0 T Cells
SBNO24	0	0.6552889	0.211	0.65	0 T Cells
SYMPK3	0	0.77483852	0.21	0.649	0 T Cells
GOLT1B3	0	0.72233578	0.19	0.629	0 T Cells
FAM3C1	0	0.20165173	0.124	0.563	0 T Cells
NIPA21	0	0.2569451	0.135	0.574	0 T Cells
XAF13	0	0.78640672	0.255	0.694	0 T Cells
IFI442	0	0.74810989	0.246	0.684	0 T Cells
TMX2	0	0.6703604	0.176	0.614	0 T Cells
CMTM71	0	0.32110875	0.141	0.579	0 T Cells
ATXN31	0	0.47848158	0.151	0.589	0 T Cells
ELAC2	0	0.35396524	0.149	0.587	0 T Cells
YIPF41	0	0.92685548	0.215	0.652	0 T Cells
TMEM2451	0	0.89540597	0.231	0.668	0 T Cells
SMARCA41	0	0.8434975	0.222	0.659	0 T Cells
GLRX32	0	0.69224352	0.189	0.626	0 T Cells
DENND4B1	0	0.27049122	0.156	0.593	0 T Cells
NEK72	0	1.02376241	0.261	0.698	0 T Cells
HERC32	0	0.96372436	0.254	0.691	0 T Cells
RBM17	0	0.86479219	0.216	0.652	0 T Cells
GPS21	0	0.7401268	0.192	0.628	0 T Cells
MANF	0	0.44613151	0.16	0.596	0 T Cells
IQGAP22	0	0.98026737	0.26	0.696	0 T Cells
SEC23IP	0	0.35049364	0.137	0.573	0 T Cells
PI4KA3	0	0.76757287	0.212	0.647	0 T Cells
SMG52	0	0.53624949	0.175	0.61	0 T Cells
C15orf393	0	0.36812115	0.136	0.571	0 T Cells
ALKBH72	0	0.20635129	0.129	0.564	0 T Cells
DARS1	0	0.89027579	0.203	0.637	0 T Cells
PSMD61	0	0.38950531	0.151	0.585	0 T Cells
ZMIZ23	0	0.47107716	0.162	0.596	0 T Cells
MIEN1	0	0.35422659	0.146	0.58	0 T Cells
ABHD14B1	0	0.10677136	0.129	0.562	0 T Cells
SLBP1	0	0.83476914	0.216	0.649	0 T Cells
RNF142	0	0.13899902	0.129	0.562	0 T Cells
CBX41	0	0.76715687	0.209	0.642	0 T Cells
SAP30L1	0	0.64306977	0.167	0.6	0 T Cells
CEBPZOS	0	0.13250993	0.119	0.551	0 T Cells
TCP12	0	0.10245979	0.114	0.546	0 T Cells

IKZF3	0	10.3510995	0.435	0.003	0	T Cells
STK111	0	0.70063679	0.19	0.622	0	T Cells
TIPRL	0	0.46499581	0.147	0.579	0	T Cells
FAM168A	0	0.16464296	0.13	0.561	0	T Cells
PDHB1	0	0.6466748	0.179	0.61	0	T Cells
LRRC471	0	0.61481041	0.171	0.602	0	T Cells
IBTK2	0	0.5092873	0.166	0.597	0	T Cells
CDC1231	0	0.46590258	0.159	0.59	0	T Cells
PMPCB2	0	0.94590731	0.222	0.652	0	T Cells
ERICH12	0	0.79064049	0.213	0.643	0	T Cells
S1PR41	0	0.66037662	0.209	0.639	0	T Cells
EIF5B1	0	0.59638358	0.165	0.595	0	T Cells
FAM91A14	0	0.50526758	0.159	0.589	0	T Cells
PRKAA11	0	0.87907464	0.218	0.647	0	T Cells
AASDHPPT	0	0.31123165	0.132	0.561	0	T Cells
DDX231	0	0.80933636	0.208	0.637	0	T Cells
KPNA41	0	0.58844686	0.179	0.608	0	T Cells
PTDSS1	0	0.67522962	0.182	0.611	0	T Cells
CSNK2A2	0	0.63692007	0.176	0.605	0	T Cells
NAAA2	0	0.4476935	0.16	0.589	0	T Cells
NSMAF1	0	0.58296191	0.17	0.599	0	T Cells
UBAP11	0	0.55088554	0.161	0.59	0	T Cells
GOLGA52	0	0.38775619	0.138	0.567	0	T Cells
TMEM170A1	0	0.3170916	0.135	0.564	0	T Cells
GTF2F12	0	0.4819961	0.156	0.585	0	T Cells
AURKAIP11	0	0.12612394	0.124	0.552	0	T Cells
REST1	0	0.93592595	0.227	0.655	0	T Cells
POLDIP32	0	0.93109439	0.224	0.652	0	T Cells
MAP3K25	0	0.66920821	0.189	0.617	0	T Cells
KCTD202	0	0.69769829	0.18	0.608	0	T Cells
C11orf681	0	0.60846555	0.167	0.595	0	T Cells
MARK21	0	0.81732576	0.209	0.636	0	T Cells
RAB11A	0	0.22476591	0.129	0.556	0	T Cells
SLC39A6	0	0.27876986	0.128	0.555	0	T Cells
RFXANK2	0	0.16928895	0.128	0.555	0	T Cells
LSM14B2	0	0.22080994	0.126	0.553	0	T Cells
RBM15B	0	0.83942049	0.2	0.627	0	T Cells
ACAP13	0	8.09933675	0.439	0.012	0	T Cells
PRCC1	0	0.24119613	0.125	0.551	0	T Cells
DCUN1D11	0	0.21792167	0.128	0.554	0	T Cells
VDAC3	0	0.18379807	0.122	0.548	0	T Cells
MDM2	0	0.60282268	0.226	0.652	0	T Cells
ATP2B12	0	0.87990269	0.247	0.673	0	T Cells
SYF21	0	0.72120009	0.192	0.618	0	T Cells
GUK1	0	0.63632629	0.179	0.605	0	T Cells
EWSR12	0	0.79969378	0.188	0.614	0	T Cells
CUL11	0	0.71938267	0.167	0.593	0	T Cells
SNX51	0	0.29033002	0.135	0.56	0	T Cells
DYNC1LI21	0	0.69061024	0.179	0.604	0	T Cells
CTR9	0	0.39079819	0.146	0.571	0	T Cells
MLLT12	0	0.32675274	0.139	0.564	0	T Cells
ENOPH1	0	0.34431945	0.137	0.561	0	T Cells

PCED1B	0	0.89054856	0.23	0.654	0	T Cells
ZC3H7B1	0	0.31342738	0.131	0.555	0	T Cells
ELF41	0	0.31722922	0.138	0.562	0	T Cells
RRP361	0	0.83130679	0.195	0.619	0	T Cells
EIF3D	0	0.82675783	0.194	0.618	0	T Cells
COP12	0	0.35151709	0.139	0.563	0	T Cells
MAP3K72	0	0.43910418	0.151	0.575	0	T Cells
SMARCD23	0	0.5606298	0.16	0.584	0	T Cells
STRN42	0	0.34198405	0.145	0.569	0	T Cells
CNOT82	0	1.03544047	0.244	0.667	0	T Cells
MED122	0	0.19745319	0.136	0.559	0	T Cells
SRP68	0	0.7017464	0.176	0.599	0	T Cells
PEX2	0	0.54288855	0.155	0.578	0	T Cells
MAGED21	0	1.08911255	0.237	0.659	0	T Cells
HCFC1	0	0.29513527	0.136	0.558	0	T Cells
DDX541	0	0.36527188	0.145	0.567	0	T Cells
DDT1	0	0.6013718	0.161	0.583	0	T Cells
SF3A11	0	0.67127163	0.165	0.587	0	T Cells
GDAP22	0	0.33069807	0.134	0.555	0	T Cells
NXPE31	0	0.12942347	0.128	0.549	0	T Cells
ATP5ME	0	0.1098865	0.119	0.54	0	T Cells
ANP32B	0	0.82795443	0.205	0.626	0	T Cells
GPATCH2L3	0	0.84920478	0.227	0.648	0	T Cells
SRCAP2	0	0.98673381	0.238	0.659	0	T Cells
PPHLN1	0	0.84882117	0.198	0.619	0	T Cells
SLC38A12	0	8.9169937	0.425	0.004	0	T Cells
WIPF22	0	0.78413176	0.192	0.613	0	T Cells
MAP3K17	0	0.55460203	0.165	0.586	0	T Cells
SEC63	0	0.57952423	0.152	0.573	0	T Cells
POLR2I1	0	0.51159893	0.153	0.574	0	T Cells
ZDHHC5	0	0.23966584	0.126	0.546	0	T Cells
MTA21	0	1.00679981	0.238	0.658	0	T Cells
RAB351	0	0.8275264	0.206	0.626	0	T Cells
SMCR81	0	0.27209653	0.134	0.554	0	T Cells
SEC24C	0	0.79043383	0.192	0.612	0	T Cells
DDX271	0	0.82076277	0.2	0.62	0	T Cells
FYTTD1	0	0.49139184	0.15	0.57	0	T Cells
BCCIP3	0	0.59315394	0.164	0.584	0	T Cells
PWP11	0	0.55061987	0.157	0.577	0	T Cells
BAG5	0	0.59552792	0.154	0.574	0	T Cells
GNL3L1	0	0.61920044	0.16	0.58	0	T Cells
NEK91	0	0.25521117	0.131	0.55	0	T Cells
TPGS11	0	0.34572809	0.137	0.556	0	T Cells
CCM2	0	0.79268472	0.18	0.599	0	T Cells
NUP623	0	0.64638291	0.167	0.586	0	T Cells
RRAGC4	0	0.33302442	0.124	0.542	0	T Cells
ACYP22	0	0.29663973	0.129	0.547	0	T Cells
TOMM701	0	0.35229406	0.132	0.55	0	T Cells
LSM81	0	1.02689137	0.234	0.652	0	T Cells
MED41	0	0.80373212	0.185	0.603	0	T Cells
KPNA12	0	0.59553845	0.165	0.583	0	T Cells
TMEM183A2	0	0.97418686	0.215	0.632	0	T Cells

ATP5MD	0	0.19084606	0.117	0.534	0	T Cells
ANAPC51	0	0.96475023	0.222	0.639	0	T Cells
TRBC1	0	10.1619922	0.42	0.003	0	T Cells
SMNDC1	0	0.70221474	0.175	0.592	0	T Cells
NUDCD3	0	0.6535703	0.17	0.587	0	T Cells
BET1L1	0	0.62947742	0.166	0.583	0	T Cells
MICU21	0	0.61904335	0.162	0.579	0	T Cells
MIA22	0	0.62606635	0.17	0.587	0	T Cells
DDX42	0	0.65441512	0.168	0.585	0	T Cells
TANGO2	0	0.22043432	0.121	0.537	0	T Cells
MAN2A11	0	0.85725008	0.189	0.605	0	T Cells
TRAFD12	0	0.48591638	0.151	0.567	0	T Cells
TOX41	0	0.65950755	0.153	0.569	0	T Cells
SPTLC21	0	0.59503282	0.163	0.579	0	T Cells
TPRG1L2	0	0.22017287	0.12	0.535	0	T Cells
NFYC2	0	0.27540004	0.125	0.54	0	T Cells
POLR3GL	0	0.90959413	0.205	0.62	0	T Cells
KPNB11	0	0.28183527	0.133	0.548	0	T Cells
IER3IP11	0	0.1114747	0.107	0.522	0	T Cells
RAB3GAP11	0	0.76828355	0.183	0.598	0	T Cells
NAB11	0	0.83017316	0.194	0.609	0	T Cells
CSRNP12	0	0.45863211	0.18	0.595	0	T Cells
WRNIP11	0	0.69639647	0.178	0.593	0	T Cells
EMD1	0	0.80621857	0.196	0.611	0	T Cells
RABEP12	0	0.63891325	0.17	0.585	0	T Cells
LNPK	0	0.34578303	0.131	0.545	0	T Cells
BUD231	0	0.54460867	0.146	0.56	0	T Cells
TAPBPL	0	0.19128138	0.125	0.539	0	T Cells
PMVK	0	0.55179161	0.159	0.573	0	T Cells
RAB11FIP21	0	0.52935077	0.152	0.566	0	T Cells
PTPRA2	0	0.62521375	0.157	0.571	0	T Cells
TYK24	0	0.737681	0.203	0.616	0	T Cells
FUS2	0	0.5825182	0.16	0.573	0	T Cells
ZNF6551	0	0.86602238	0.205	0.617	0	T Cells
ZC3H13	0	0.29870164	0.124	0.536	0	T Cells
RNF1112	0	0.3823969	0.138	0.55	0	T Cells
HDHD2	0	0.14871932	0.119	0.531	0	T Cells
RNF1851	0	0.15306941	0.111	0.523	0	T Cells
BRD7	0	0.74102038	0.175	0.587	0	T Cells
C7orf26	0	0.69763879	0.171	0.583	0	T Cells
UBP11	0	1.05264639	0.235	0.646	0	T Cells
AGA2	0	0.42529664	0.136	0.547	0	T Cells
FBXO91	0	0.18037763	0.121	0.532	0	T Cells
SURF42	0	0.90733442	0.218	0.629	0	T Cells
TRA2B	0	0.78503832	0.186	0.597	0	T Cells
OSBP1	0	0.90669734	0.193	0.604	0	T Cells
FBXW23	0	1.02193407	0.23	0.64	0	T Cells
CPD2	0	0.48318671	0.143	0.553	0	T Cells
YWHAQ	0	0.8536892	0.195	0.605	0	T Cells
NSUN21	0	0.73458591	0.18	0.59	0	T Cells
NCOA11	0	0.71679754	0.17	0.58	0	T Cells
OST41	0	0.61295872	0.153	0.563	0	T Cells

FAF11	0	0.41343026	0.141	0.55	0	T Cells
HAX1	0	0.21646525	0.114	0.523	0	T Cells
STK251	0	0.43697044	0.141	0.55	0	T Cells
RARS1	0	0.34696191	0.126	0.535	0	T Cells
KPNA33	0	0.30158012	0.127	0.536	0	T Cells
PRPF311	0	0.46655093	0.143	0.552	0	T Cells
USP102	0	0.25590653	0.124	0.532	0	T Cells
HDGFL22	0	0.45433727	0.144	0.552	0	T Cells
PET1002	0	0.56370646	0.148	0.556	0	T Cells
UBE3C2	0	0.98932	0.205	0.612	0	T Cells
BAZ1A2	0	0.34401971	0.133	0.54	0	T Cells
MYO5A3	0	0.33750512	0.127	0.534	0	T Cells
RNF1672	0	0.95821833	0.206	0.613	0	T Cells
TSC22D2	0	0.65763377	0.17	0.577	0	T Cells
CBX32	0	0.65475376	0.157	0.564	0	T Cells
RHOC3	0	0.34107665	0.151	0.557	0	T Cells
GALNT112	0	0.13147497	0.107	0.513	0	T Cells
SLC39A91	0	0.39318764	0.132	0.538	0	T Cells
NUTF23	0	0.12979428	0.119	0.525	0	T Cells
NFKB14	0	0.64896928	0.176	0.582	0	T Cells
ATP5IF1	0	0.35683777	0.137	0.542	0	T Cells
DHX162	0	0.4925291	0.143	0.548	0	T Cells
FBXL172	0	0.67186236	0.173	0.578	0	T Cells
DDA1	0	0.20226018	0.116	0.52	0	T Cells
AKT2	0	0.47667627	0.133	0.537	0	T Cells
NDRG3	0	0.56951298	0.154	0.558	0	T Cells
NSA22	0	0.84796002	0.191	0.595	0	T Cells
BRD31	0	0.44967647	0.143	0.546	0	T Cells
SUPT16H2	0	0.67208092	0.155	0.558	0	T Cells
HNRNPM1	0	0.60813354	0.151	0.554	0	T Cells
PIN1	0	0.63901567	0.158	0.561	0	T Cells
ELOF12	0	0.62999525	0.152	0.555	0	T Cells
TMEM160	0	0.14106196	0.116	0.519	0	T Cells
TSTA3	0	0.71658233	0.175	0.578	0	T Cells
ELOA	0	0.33071148	0.116	0.518	0	T Cells
KLHL121	0	0.10361576	0.108	0.51	0	T Cells
GFM1	0	0.19954711	0.114	0.516	0	T Cells
ZNF6221	0	0.12421914	0.109	0.511	0	T Cells
SERINC53	0	0.67844852	0.16	0.562	0	T Cells
CSNK2B1	0	0.27696963	0.117	0.519	0	T Cells
SNX133	0	0.55446145	0.143	0.545	0	T Cells
DGAT12	0	0.13465569	0.112	0.514	0	T Cells
CD2BP2	0	0.30060015	0.123	0.525	0	T Cells
EIF4E32	0	6.28238543	0.478	0.076	0	T Cells
EIF3J2	0	0.92428448	0.2	0.602	0	T Cells
CDC5L2	0	0.67200358	0.152	0.553	0	T Cells
AP5M11	0	0.37174155	0.131	0.532	0	T Cells
GSPT11	0	0.37853984	0.128	0.529	0	T Cells
UPF11	0	0.59836704	0.156	0.557	0	T Cells
OSBPL92	0	0.38903855	0.125	0.525	0	T Cells
ENTPD61	0	0.23906306	0.118	0.518	0	T Cells
TSR2	0	0.29953362	0.126	0.526	0	T Cells

NUP982	0	0.98170081	0.2	0.6	0	T Cells
RPA1	0	0.67625719	0.171	0.571	0	T Cells
ZNF362	0	0.21519505	0.119	0.518	0	T Cells
IP6K11	0	0.6119053	0.16	0.559	0	T Cells
EIF4E4	0	0.23488346	0.114	0.513	0	T Cells
FOXP4	0	0.2371705	0.123	0.522	0	T Cells
BUD311	0	0.34757693	0.128	0.527	0	T Cells
ACSL52	0	0.89272298	0.209	0.608	0	T Cells
OSBPL21	0	0.30650733	0.127	0.526	0	T Cells
DDX182	0	0.92485914	0.178	0.577	0	T Cells
PIK3CG3	0	0.78518357	0.194	0.593	0	T Cells
UBE2G11	0	0.77129871	0.176	0.575	0	T Cells
MKNK21	0	0.6915867	0.18	0.579	0	T Cells
TOR1AIP21	0	0.22303392	0.119	0.517	0	T Cells
MOCS21	0	0.25120985	0.12	0.518	0	T Cells
CKAP5	0	0.21582449	0.115	0.513	0	T Cells
SYVN1	0	0.15305507	0.114	0.512	0	T Cells
ATG101	0	0.13634666	0.108	0.506	0	T Cells
PA2G4	0	0.94307233	0.206	0.604	0	T Cells
NONO	0	0.25749725	0.121	0.519	0	T Cells
MAPK91	0	0.96172381	0.19	0.588	0	T Cells
REPS1	0	0.87651896	0.186	0.584	0	T Cells
GID82	0	0.8793578	0.181	0.579	0	T Cells
GOLGB12	0	0.60698035	0.148	0.545	0	T Cells
EIF2B11	0	0.43544261	0.13	0.527	0	T Cells
CIAO2B2	0	0.24714111	0.116	0.513	0	T Cells
KARS	0	0.344678	0.115	0.512	0	T Cells
PQBP11	0	0.4663609	0.134	0.531	0	T Cells
PIP4K2B1	0	0.96527695	0.203	0.6	0	T Cells
ARRDC22	0	0.68906118	0.181	0.578	0	T Cells
STRN1	0	0.74927036	0.161	0.557	0	T Cells
YME1L12	0	0.37227356	0.13	0.526	0	T Cells
MMGT12	0	0.57198551	0.147	0.543	0	T Cells
LRRC8C2	0	0.82325431	0.186	0.582	0	T Cells
RIPK12	0	0.86627103	0.184	0.58	0	T Cells
KHNYN1	0	0.78642174	0.189	0.585	0	T Cells
NEMF1	0	0.75807935	0.181	0.577	0	T Cells
METTL261	0	0.96336243	0.199	0.595	0	T Cells
CDS2	0	0.83011293	0.173	0.569	0	T Cells
ARHGAP251	0	0.99625377	0.22	0.615	0	T Cells
PPIP5K23	0	0.28906969	0.117	0.512	0	T Cells
HARS	0	0.3183469	0.128	0.523	0	T Cells
LRFN41	0	0.16665915	0.107	0.502	0	T Cells
SPOP1	0	0.49916997	0.146	0.541	0	T Cells
SMIM72	0	0.63373963	0.142	0.537	0	T Cells
ZGPAT1	0	1.0301692	0.219	0.614	0	T Cells
PI4K2B	0	0.9216264	0.198	0.593	0	T Cells
TNFRSF143	0	0.44937781	0.153	0.547	0	T Cells
TEX261	0	0.1558008	0.11	0.504	0	T Cells
TFG1	0	0.10708324	0.1	0.494	0	T Cells
TMEM1751	0	0.16029268	0.118	0.512	0	T Cells
MAEA2	0	1.04129812	0.205	0.599	0	T Cells

RNF62	0	0.66578661	0.145	0.539	0	T Cells
IFT201	0	0.12183196	0.105	0.499	0	T Cells
SIRT7	0	0.56062371	0.152	0.546	0	T Cells
UBL5	0	0.23853173	0.118	0.512	0	T Cells
EIF2S31	0	0.6033074	0.15	0.544	0	T Cells
ITK	0	8.16874925	0.41	0.016	0	T Cells
VPS362	0	0.80663007	0.174	0.568	0	T Cells
MTHFD22	0	0.6713731	0.17	0.564	0	T Cells
UBE2J21	0	0.65625166	0.157	0.55	0	T Cells
SSRP11	0	0.74926268	0.166	0.559	0	T Cells
THYN11	0	0.20029497	0.112	0.505	0	T Cells
PYM11	0	0.24892285	0.122	0.515	0	T Cells
TINF21	0	0.74790758	0.169	0.562	0	T Cells
GNG23	0	8.59856589	0.399	0.006	0	T Cells
ERH1	0	0.23037443	0.112	0.505	0	T Cells
UBR51	0	0.93544532	0.198	0.591	0	T Cells
GBP42	0	0.35889254	0.147	0.539	0	T Cells
SCNM11	0	0.29840245	0.121	0.513	0	T Cells
NSL11	0	0.34681904	0.118	0.51	0	T Cells
ADAM193	0	8.15577124	0.397	0.005	0	T Cells
EIF3M	0	0.43010034	0.131	0.523	0	T Cells
DERL2	0	0.58399279	0.144	0.536	0	T Cells
DHX83	0	0.79066512	0.17	0.562	0	T Cells
TRAPPC6A1	0	0.50677103	0.147	0.539	0	T Cells
ZMYND82	0	0.52529538	0.15	0.542	0	T Cells
PEX131	0	0.6053735	0.146	0.537	0	T Cells
RSRC11	0	0.21602179	0.106	0.497	0	T Cells
TMED22	0	0.50936736	0.133	0.524	0	T Cells
IREB21	0	0.55034043	0.142	0.533	0	T Cells
AP3S21	0	0.39657968	0.126	0.517	0	T Cells
INPP5K1	0	0.73170169	0.166	0.557	0	T Cells
TTC37	0	0.94953749	0.19	0.581	0	T Cells
ABCF11	0	0.42680863	0.134	0.524	0	T Cells
RAF12	0	0.88342327	0.18	0.57	0	T Cells
CDC162	0	1.0015336	0.198	0.588	0	T Cells
USP191	0	0.24797909	0.112	0.501	0	T Cells
CCDC28A	0	0.11961934	0.102	0.491	0	T Cells
NELFB	0	0.72200753	0.161	0.55	0	T Cells
CLN34	0	0.40765672	0.132	0.521	0	T Cells
CNEP1R12	0	0.42719632	0.127	0.516	0	T Cells
GLG1	0	0.77162958	0.154	0.543	0	T Cells
MBTPS11	0	0.97154228	0.204	0.593	0	T Cells
SSR4	0	0.80410477	0.171	0.56	0	T Cells
PURB1	0	0.82489712	0.174	0.562	0	T Cells
RNPS1	0	0.8434569	0.173	0.561	0	T Cells
NFKBIZ2	0	0.37022379	0.164	0.552	0	T Cells
PRXL2C3	0	0.47188089	0.124	0.512	0	T Cells
IFIT51	0	0.39467663	0.126	0.514	0	T Cells
GGA32	0	0.53927346	0.147	0.535	0	T Cells
JMJD62	0	0.49324597	0.139	0.527	0	T Cells
PGPEP11	0	0.21984067	0.121	0.509	0	T Cells
TNIP2	0	0.89704813	0.189	0.577	0	T Cells

TFCP23	0	0.89320491	0.185	0.573	0	T Cells
CAND1	0	0.99273425	0.184	0.572	0	T Cells
METTL231	0	1.024212	0.197	0.585	0	T Cells
PBRM12	0	0.77292134	0.16	0.547	0	T Cells
GRPEL11	0	0.39795057	0.119	0.506	0	T Cells
PPP2R5E2	0	0.84338897	0.164	0.551	0	T Cells
TMEM87A1	0	0.85646503	0.166	0.553	0	T Cells
CRELD21	0	0.25091441	0.12	0.507	0	T Cells
TBRG11	0	0.88435921	0.196	0.583	0	T Cells
RPIA1	0	0.4879362	0.133	0.519	0	T Cells
GRK63	0	0.66240288	0.171	0.557	0	T Cells
RPP30	0	0.22797661	0.113	0.499	0	T Cells
ERGIC2	0	0.59285966	0.133	0.519	0	T Cells
COMMD61	0	0.78774988	0.162	0.548	0	T Cells
PPP2R3C2	0	0.51992996	0.125	0.511	0	T Cells
OSER12	0	0.1537133	0.106	0.492	0	T Cells
SPEN1	0	0.94719643	0.188	0.574	0	T Cells
HUS11	0	0.91211206	0.184	0.57	0	T Cells
TNKS21	0	0.83525163	0.177	0.562	0	T Cells
GNL32	0	0.92811381	0.171	0.556	0	T Cells
VTA1	0	0.17793586	0.106	0.491	0	T Cells
INTS15	0	0.73376163	0.16	0.545	0	T Cells
AP1G12	0	0.6801806	0.149	0.534	0	T Cells
CXXC12	0	0.32827834	0.125	0.51	0	T Cells
CLPP1	0	0.89190886	0.171	0.556	0	T Cells
HP1BP32	0	0.96390381	0.192	0.577	0	T Cells
ILF21	0	0.32063416	0.112	0.496	0	T Cells
VPS541	0	0.48224022	0.129	0.513	0	T Cells
SUCLG2	0	0.3755784	0.117	0.501	0	T Cells
NUDCD22	0	0.6566662	0.142	0.526	0	T Cells
TC2N	0	9.354611	0.387	0.003	0	T Cells
CASC41	0	0.55370689	0.134	0.518	0	T Cells
TM2D3	0	0.59043671	0.137	0.521	0	T Cells
POLR3K	0	0.31335179	0.114	0.498	0	T Cells
SF3A31	0	0.67779021	0.147	0.53	0	T Cells
RUBCN2	0	0.50324795	0.131	0.514	0	T Cells
UTP31	0	0.54746795	0.135	0.518	0	T Cells
ANAPC71	0	0.46766465	0.127	0.51	0	T Cells
FEM1B2	0	0.82052681	0.157	0.54	0	T Cells
PCED1A1	0	0.60678253	0.152	0.535	0	T Cells
NDEL12	0	0.96870931	0.19	0.573	0	T Cells
ETF11	0	0.20489656	0.1	0.482	0	T Cells
CALHM23	0	0.75227253	0.213	0.595	0	T Cells
CANT12	0	0.45213416	0.129	0.511	0	T Cells
RXRB1	0	0.60513772	0.143	0.524	0	T Cells
SMIM19	0	0.53684274	0.13	0.511	0	T Cells
HECTD12	0	0.63161373	0.14	0.521	0	T Cells
LMNB2	0	0.13419001	0.108	0.489	0	T Cells
BCKDHA2	0	0.30758035	0.125	0.506	0	T Cells
NF2	0	0.35137108	0.128	0.509	0	T Cells
GTPBP11	0	0.73171743	0.164	0.545	0	T Cells
CIC2	0	0.88720204	0.188	0.569	0	T Cells

MLH11	0	0.43069471	0.123	0.503	0	T Cells
SHLD22	0	0.8506669	0.161	0.541	0	T Cells
RIC8A1	0	0.14619363	0.106	0.486	0	T Cells
C2CD51	0	0.39873614	0.118	0.498	0	T Cells
GART1	0	0.16151894	0.106	0.486	0	T Cells
STARD31	0	0.9598664	0.194	0.574	0	T Cells
MMADHC2	0	0.35704892	0.118	0.497	0	T Cells
SIAH14	0	0.33556143	0.114	0.493	0	T Cells
DLGAP45	0	0.53812191	0.137	0.516	0	T Cells
ZNF1482	0	1.07093901	0.184	0.562	0	T Cells
PPP1R101	0	0.81728605	0.172	0.55	0	T Cells
COPS22	0	0.89823641	0.174	0.552	0	T Cells
PITPNB1	0	0.96442644	0.176	0.554	0	T Cells
SMYD22	0	0.70660516	0.151	0.529	0	T Cells
DPY301	0	0.30923283	0.108	0.486	0	T Cells
GTF2H12	0	0.65518668	0.147	0.525	0	T Cells
NUP1071	0	0.19132574	0.103	0.481	0	T Cells
N4BP2L12	0	0.23352484	0.121	0.499	0	T Cells
OGFOD11	0	0.49697148	0.13	0.508	0	T Cells
RAVER11	0	0.8010732	0.161	0.539	0	T Cells
GTPBP61	0	0.82908913	0.166	0.544	0	T Cells
VCPIP11	0	1.02552687	0.184	0.561	0	T Cells
ABHD31	0	0.91586461	0.173	0.55	0	T Cells
SLC30A71	0	0.78160145	0.162	0.539	0	T Cells
SMC61	0	0.44863493	0.133	0.51	0	T Cells
GMPPB1	0	0.19568732	0.107	0.484	0	T Cells
TRAPPC112	0	0.3470894	0.117	0.493	0	T Cells
PAIP21	0	0.20132647	0.102	0.478	0	T Cells
ECI21	0	0.64856595	0.15	0.526	0	T Cells
LSM1	0	0.6457605	0.137	0.513	0	T Cells
ZSWIM82	0	0.61833605	0.149	0.525	0	T Cells
TM2D11	0	0.4058647	0.113	0.488	0	T Cells
RDH14	0	0.15818478	0.102	0.477	0	T Cells
RAP1GDS1	0	0.62763661	0.139	0.514	0	T Cells
HSPA91	0	0.11722742	0.099	0.474	0	T Cells
UGCG3	0	0.35428301	0.155	0.53	0	T Cells
GLE12	0	0.37671197	0.112	0.487	0	T Cells
SC5D3	0	0.28278953	0.112	0.487	0	T Cells
VPS532	0	0.73814284	0.158	0.533	0	T Cells
KIDINS2202	0	0.91573828	0.176	0.55	0	T Cells
KIAA0319L1	0	0.70043939	0.141	0.515	0	T Cells
TESK11	0	0.29398087	0.119	0.493	0	T Cells
PPTC7	0	0.75201801	0.156	0.53	0	T Cells
CSNK1E2	0	0.45846817	0.133	0.507	0	T Cells
CSNK1G21	0	1.06331284	0.193	0.567	0	T Cells
THUMPD11	0	0.96444651	0.175	0.548	0	T Cells
UBQLN2	0	1.00361282	0.176	0.549	0	T Cells
TRIM42	0	0.70304581	0.143	0.516	0	T Cells
MAPKAPK51	0	0.78284701	0.154	0.527	0	T Cells
PCYT2	0	0.25012638	0.112	0.485	0	T Cells
NAA101	0	0.62294076	0.142	0.515	0	T Cells
PITHD12	0	1.06157856	0.179	0.551	0	T Cells

INO80E2	0	1.00787751	0.188	0.56	0	T Cells
RAB29	0	0.45171134	0.123	0.495	0	T Cells
ALG9	0	0.36638198	0.109	0.481	0	T Cells
GOSR12	0	0.54830681	0.127	0.499	0	T Cells
SLC10A3	0	0.58975073	0.137	0.509	0	T Cells
CYP4V21	0	0.15196681	0.102	0.473	0	T Cells
DUSP221	0	0.24437322	0.114	0.485	0	T Cells
CYB561A32	0	0.1689616	0.104	0.475	0	T Cells
CTDP11	0	0.83135697	0.16	0.531	0	T Cells
MED29	0	0.4417266	0.112	0.483	0	T Cells
ARFGEF22	0	0.39213225	0.119	0.49	0	T Cells
UBE2L3	0	0.55184063	0.124	0.495	0	T Cells
ZNF242	0	1.0455514	0.186	0.556	0	T Cells
ST3GAL52	0	0.7251	0.156	0.526	0	T Cells
WDR45B1	0	0.49270103	0.113	0.483	0	T Cells
SAMD4B2	0	0.3447266	0.111	0.481	0	T Cells
KRTCAP21	0	1.08375479	0.188	0.557	0	T Cells
TBC1D20	0	1.04733977	0.192	0.561	0	T Cells
NOP561	0	0.9707784	0.176	0.545	0	T Cells
CD483	0	6.13283606	0.437	0.068	0	T Cells
NOP162	0	0.56047138	0.127	0.496	0	T Cells
CGAS	0	0.65509796	0.15	0.519	0	T Cells
UBQLN12	0	0.54827204	0.129	0.498	0	T Cells
FAM98B1	0	0.59847062	0.125	0.494	0	T Cells
ERCC51	0	0.99614408	0.183	0.551	0	T Cells
AGO12	0	0.48277958	0.132	0.5	0	T Cells
NMD33	0	0.44875867	0.117	0.485	0	T Cells
CCAR2	0	0.63738503	0.141	0.509	0	T Cells
TMEM126B1	0	0.6805959	0.135	0.503	0	T Cells
CCDC922	0	0.80948058	0.171	0.539	0	T Cells
GTF2A11	0	0.71881826	0.141	0.509	0	T Cells
CDK16	0	0.35129992	0.112	0.48	0	T Cells
FAM199X2	0	0.36867361	0.113	0.481	0	T Cells
FOXN23	0	0.76994565	0.157	0.524	0	T Cells
XRCC5	0	0.41369322	0.105	0.472	0	T Cells
CAMK1D4	0	0.15208432	0.108	0.475	0	T Cells
ZNF321	0	0.17190375	0.109	0.476	0	T Cells
CHD81	0	0.91652359	0.161	0.528	0	T Cells
MAP4K52	0	0.39974373	0.11	0.477	0	T Cells
TCF123	0	0.77641335	0.139	0.506	0	T Cells
NT5C	0	0.32973223	0.115	0.482	0	T Cells
PGM24	0	0.3686216	0.11	0.476	0	T Cells
COPS4	0	0.27080196	0.104	0.47	0	T Cells
KLF104	0	0.57177326	0.133	0.499	0	T Cells
NUP188	0	0.77124485	0.15	0.516	0	T Cells
PTPRJ3	0	0.84688479	0.171	0.537	0	T Cells
ADAT12	0	0.39892279	0.117	0.483	0	T Cells
EXOSC102	0	0.98766199	0.175	0.54	0	T Cells
BRAT1	0	0.17926195	0.102	0.467	0	T Cells
SF3B31	0	1.07730303	0.187	0.552	0	T Cells
MTMR121	0	0.51167876	0.13	0.495	0	T Cells
HMGCR	0	0.57252485	0.166	0.531	0	T Cells

RBM221	0	0.66503506	0.133	0.498	0	T Cells
WDR461	0	0.45461297	0.122	0.487	0	T Cells
FOXO31	0	0.81052475	0.15	0.515	0	T Cells
SUGT12	0	0.76181325	0.141	0.506	0	T Cells
SMAD41	0	1.04364178	0.183	0.547	0	T Cells
PTOV11	0	0.2963767	0.104	0.468	0	T Cells
SRPRB1	0	0.65895304	0.128	0.492	0	T Cells
GZMA	0	10.5088724	0.366	0.002	0	T Cells
SSR11	0	0.29839915	0.094	0.458	0	T Cells
GPAT42	0	0.57874108	0.133	0.497	0	T Cells
CUL4A1	0	0.93423973	0.163	0.527	0	T Cells
NAA382	0	0.52149161	0.123	0.487	0	T Cells
GIT1	0	0.70320167	0.14	0.504	0	T Cells
NSMCE21	0	0.2968017	0.102	0.465	0	T Cells
TMEM185B2	0	0.64499499	0.131	0.494	0	T Cells
PELO	0	0.94209603	0.163	0.526	0	T Cells
POLK1	0	0.55782196	0.125	0.488	0	T Cells
COG52	0	0.48122827	0.121	0.484	0	T Cells
CHCHD71	0	0.6424648	0.134	0.497	0	T Cells
SEC61B2	0	0.38619253	0.107	0.47	0	T Cells
WBP1L	0	0.17905595	0.106	0.469	0	T Cells
TAF12	0	0.43914526	0.116	0.478	0	T Cells
SRP9	0	0.42618112	0.113	0.475	0	T Cells
GTF3C22	0	0.360084	0.111	0.473	0	T Cells
CCND23	0	7.69858103	0.372	0.01	0	T Cells
DENR1	0	0.56492563	0.129	0.491	0	T Cells
BCL7C1	0	0.73278367	0.156	0.518	0	T Cells
STX8	0	0.15643318	0.096	0.458	0	T Cells
UFD1	0	0.17878843	0.102	0.463	0	T Cells
NRAS3	0	0.40194949	0.116	0.477	0	T Cells
CCT42	0	0.24148614	0.098	0.459	0	T Cells
SRF1	0	0.77583986	0.157	0.518	0	T Cells
PHACTR21	0	0.72744862	0.136	0.497	0	T Cells
LMBR11	0	0.63181487	0.129	0.49	0	T Cells
TMEM111	0	0.37733977	0.11	0.471	0	T Cells
TFIP11	0	0.59612615	0.126	0.487	0	T Cells
ROCK22	0	0.33180951	0.106	0.466	0	T Cells
DNMT12	0	0.80659893	0.186	0.546	0	T Cells
AP3M1	0	0.52331798	0.119	0.479	0	T Cells
PPP1R12C2	0	0.73050857	0.145	0.505	0	T Cells
RWDD12	0	0.7727637	0.135	0.494	0	T Cells
PTGES21	0	0.4096283	0.116	0.475	0	T Cells
PITRM1	0	0.61349315	0.125	0.484	0	T Cells
KAT51	0	0.10653864	0.091	0.45	0	T Cells
NABP2	0	0.11949646	0.093	0.452	0	T Cells
SEC23A2	0	0.47656123	0.114	0.473	0	T Cells
TAOK21	0	0.58948651	0.122	0.481	0	T Cells
PHKB3	0	0.40691916	0.113	0.472	0	T Cells
RLIM1	0	0.79302071	0.143	0.502	0	T Cells
STAM1	0	0.37617799	0.105	0.463	0	T Cells
GNPAT2	0	0.43267349	0.111	0.469	0	T Cells
SLC25A361	0	0.70346009	0.141	0.499	0	T Cells

DDX60	0	0.89225518	0.155	0.513	0	T Cells
RAD501	0	0.60598731	0.129	0.487	0	T Cells
FAM126A2	0	0.91671104	0.166	0.524	0	T Cells
UCK11	0	0.23507619	0.098	0.456	0	T Cells
ZDHHC62	0	0.86582214	0.151	0.509	0	T Cells
SMARCE11	0	0.78862098	0.146	0.504	0	T Cells
NOTCH23	0	0.54278066	0.13	0.487	0	T Cells
DHX301	0	0.86844023	0.149	0.506	0	T Cells
ANKRD13A	0	0.82172481	0.152	0.509	0	T Cells
NUP851	0	0.6041439	0.131	0.488	0	T Cells
FARSB2	0	0.36804323	0.108	0.464	0	T Cells
MBOAT12	0	0.26774528	0.106	0.462	0	T Cells
MAD2L1BP	0	0.39707384	0.105	0.461	0	T Cells
CXCR6	0	10.6865073	0.358	0.002	0	T Cells
GOLGA31	0	0.60473861	0.128	0.484	0	T Cells
UTP64	0	0.30904284	0.103	0.458	0	T Cells
PEX11B2	0	0.40693281	0.114	0.469	0	T Cells
COMMD21	0	0.19108946	0.098	0.453	0	T Cells
PRKAB11	0	0.5328662	0.125	0.48	0	T Cells
MZT11	0	0.55675082	0.125	0.48	0	T Cells
DNAJA31	0	0.5951407	0.124	0.479	0	T Cells
HDAC51	0	0.51655491	0.121	0.476	0	T Cells
MTOR1	0	0.42355735	0.106	0.46	0	T Cells
NUP1331	0	0.46953652	0.111	0.465	0	T Cells
TMEM1312	0	0.4466438	0.112	0.466	0	T Cells
FADD	0	0.4617901	0.114	0.468	0	T Cells
THAP111	0	0.97301939	0.173	0.527	0	T Cells
PNRC22	0	0.5622037	0.123	0.477	0	T Cells
SVBP2	0	0.22008511	0.1	0.454	0	T Cells
NUDT51	0	0.98368311	0.164	0.518	0	T Cells
RMC11	0	0.9733373	0.167	0.521	0	T Cells
TECPR11	0	0.2067783	0.102	0.455	0	T Cells
TRIM333	0	0.87608041	0.151	0.504	0	T Cells
CEP632	0	0.16476948	0.097	0.45	0	T Cells
SCRIB1	0	0.37538742	0.116	0.469	0	T Cells
TRMO2	0	0.91618203	0.151	0.504	0	T Cells
GAPVD11	0	0.57839725	0.123	0.476	0	T Cells
NUP1601	0	0.9476992	0.159	0.512	0	T Cells
EML21	0	0.87722179	0.165	0.518	0	T Cells
POLR1A1	0	0.43572223	0.106	0.458	0	T Cells
DMXL13	0	0.3731552	0.107	0.459	0	T Cells
WDR113	0	0.27229467	0.103	0.455	0	T Cells
IRF2BPL	0	0.35752474	0.116	0.468	0	T Cells
PAGR12	0	0.29211633	0.107	0.459	0	T Cells
C2orf683	0	0.45690778	0.117	0.469	0	T Cells
ACTR81	0	0.50757961	0.118	0.47	0	T Cells
UBE2H1	0	0.55316908	0.117	0.469	0	T Cells
NELFCD1	0	0.66826187	0.137	0.489	0	T Cells
SCYL21	0	0.39009225	0.106	0.457	0	T Cells
TNPO11	0	0.3139621	0.101	0.452	0	T Cells
WDR36	0	0.1216643	0.088	0.439	0	T Cells
TBRG42	0	0.63903306	0.13	0.481	0	T Cells

RABGAP12	0	0.5824976	0.124	0.475	0	T Cells
TBK12	0	0.69872939	0.128	0.479	0	T Cells
RB14	0	0.21347329	0.094	0.445	0	T Cells
MX23	0	0.56966951	0.142	0.493	0	T Cells
CSNK1A12	0	0.35510309	0.107	0.457	0	T Cells
DHDDS1	0	0.14298321	0.093	0.443	0	T Cells
SLC4A1AP1	0	0.2442148	0.096	0.446	0	T Cells
ATG131	0	0.59030125	0.12	0.47	0	T Cells
NMRAL1	0	0.51710549	0.121	0.471	0	T Cells
RPAIN2	0	0.64006124	0.134	0.484	0	T Cells
FAM222B2	0	0.18161946	0.094	0.444	0	T Cells
PDZD111	0	0.19007843	0.091	0.441	0	T Cells
ATG51	0	0.82753465	0.141	0.49	0	T Cells
INTS102	0	0.6294542	0.128	0.477	0	T Cells
FCF1	0	0.12882558	0.087	0.436	0	T Cells
YY1	0	0.78145677	0.145	0.494	0	T Cells
NUDT141	0	0.42522393	0.115	0.463	0	T Cells
POLR2F	0	0.41385386	0.104	0.452	0	T Cells
RNF2202	0	0.73115051	0.139	0.487	0	T Cells
IGSF81	0	0.15211586	0.092	0.44	0	T Cells
PPP2R5D	0	0.69124368	0.128	0.476	0	T Cells
CHUK1	0	0.13126145	0.09	0.438	0	T Cells
EBP	0	0.77601354	0.158	0.506	0	T Cells
TCTA	0	0.40314941	0.103	0.45	0	T Cells
GAR11	0	0.51305381	0.108	0.455	0	T Cells
ISOC1	0	0.43951333	0.105	0.452	0	T Cells
LCMT11	0	0.59427081	0.114	0.461	0	T Cells
BCL23	0	6.48274258	0.404	0.057	0	T Cells
POLR3C1	0	0.34678084	0.101	0.448	0	T Cells
SH3BP5L	0	0.11456297	0.093	0.44	0	T Cells
MARCH51	0	0.67969093	0.127	0.474	0	T Cells
RASGRP1	0	8.2656063	0.361	0.014	0	T Cells
ARPP191	0	0.7738798	0.141	0.488	0	T Cells
TMEM185A1	0	0.61378601	0.123	0.47	0	T Cells
AGO43	0	0.64878715	0.121	0.467	0	T Cells
RTRAF2	0	0.44240826	0.105	0.451	0	T Cells
COG41	0	0.5602137	0.121	0.467	0	T Cells
CRTC21	0	0.80724923	0.143	0.489	0	T Cells
TAF61	0	0.89237166	0.148	0.494	0	T Cells
LRRC571	0	0.15699815	0.089	0.435	0	T Cells
KIF1B4	0	0.29827813	0.103	0.448	0	T Cells
DNAJC102	0	0.62137803	0.122	0.467	0	T Cells
BMS11	0	0.39923552	0.11	0.455	0	T Cells
MTR1	0	0.96566866	0.158	0.503	0	T Cells
BZW12	0	0.33159926	0.098	0.443	0	T Cells
TMEM1411	0	0.19755998	0.095	0.44	0	T Cells
SSNA11	0	0.60639497	0.128	0.473	0	T Cells
NTAN11	0	0.27030527	0.094	0.439	0	T Cells
TP532	0	0.75524013	0.135	0.48	0	T Cells
DAPK31	0	0.30209008	0.099	0.444	0	T Cells
PLXNA3	0	0.89309872	0.166	0.511	0	T Cells
TRAF3IP34	0	7.32357813	0.365	0.021	0	T Cells

COQ10B1	0	0.7772589	0.135	0.479	0	T Cells
FAM120AOS2	0	0.35152802	0.101	0.445	0	T Cells
RELA1	0	0.75672078	0.13	0.474	0	T Cells
NME3	0	0.89746681	0.152	0.496	0	T Cells
XPO61	0	1.01026237	0.16	0.504	0	T Cells
COPS3	0	0.76934248	0.133	0.477	0	T Cells
PIH1D1	0	0.70960031	0.137	0.481	0	T Cells
LMF21	0	0.90022094	0.154	0.498	0	T Cells
NDUFS61	0	0.30149275	0.105	0.448	0	T Cells
CHRAC11	0	0.66165975	0.121	0.464	0	T Cells
CHERP1	0	0.6537176	0.122	0.465	0	T Cells
PPIL21	0	0.54935512	0.121	0.464	0	T Cells
PREB	0	0.11211568	0.092	0.435	0	T Cells
NNT1	0	0.62572217	0.129	0.472	0	T Cells
NUDT1	0	0.28521187	0.1	0.443	0	T Cells
SIGMAR1	0	0.33009145	0.097	0.44	0	T Cells
TMX32	0	0.32413791	0.101	0.444	0	T Cells
ECSIT	0	0.21389219	0.098	0.441	0	T Cells
PHF5A1	0	1.03378503	0.164	0.507	0	T Cells
INPP5F2	0	0.44390266	0.115	0.457	0	T Cells
PLEKHF24	0	0.87314176	0.14	0.482	0	T Cells
DPP82	0	0.94945777	0.156	0.498	0	T Cells
DHX382	0	0.7810863	0.137	0.479	0	T Cells
ABCA72	0	0.86422385	0.158	0.5	0	T Cells
VBP11	0	0.49264335	0.102	0.443	0	T Cells
RAB2B1	0	0.33862074	0.097	0.438	0	T Cells
BAHD1	0	0.23047746	0.101	0.442	0	T Cells
THEMIS	0	10.5345963	0.342	0.002	0	T Cells
AGPAT43	0	0.37367495	0.112	0.452	0	T Cells
TRIM51	0	0.40435197	0.111	0.451	0	T Cells
TADA2B1	0	0.78662515	0.132	0.472	0	T Cells
NUDT23	0	0.30904724	0.095	0.435	0	T Cells
INPP5B3	0	0.53820428	0.128	0.467	0	T Cells
PPIL31	0	0.36701121	0.107	0.446	0	T Cells
CCR53	0	6.1138544	0.395	0.056	0	T Cells
VPS522	0	0.65310658	0.124	0.463	0	T Cells
NGRN1	0	0.52961073	0.102	0.441	0	T Cells
RSL1D11	0	0.58668	0.112	0.451	0	T Cells
CDYL	0	0.10121125	0.089	0.428	0	T Cells
TNRC182	0	0.7435018	0.135	0.474	0	T Cells
BZW22	0	0.10484538	0.087	0.426	0	T Cells
CYP51A11	0	0.25714397	0.152	0.491	0	T Cells
MUS812	0	0.85479918	0.147	0.486	0	T Cells
SRSF1	0	0.19509618	0.09	0.429	0	T Cells
DELE12	0	0.61363308	0.12	0.458	0	T Cells
RCL1	0	0.4828016	0.114	0.452	0	T Cells
DDX581	0	0.52789106	0.13	0.468	0	T Cells
CRNKL1	0	0.53616773	0.106	0.444	0	T Cells
SRP191	0	0.96265487	0.155	0.493	0	T Cells
TCOF1	0	0.91128901	0.151	0.489	0	T Cells
XPO52	0	0.61245137	0.116	0.453	0	T Cells
PUS12	0	0.56248336	0.122	0.459	0	T Cells

FN3KRP	0	0.54235486	0.111	0.448	0	T Cells
CSF11	0	0.14263762	0.156	0.493	0	T Cells
CHCHD5	0	0.17195953	0.091	0.428	0	T Cells
FAM204A2	0	0.95928784	0.15	0.487	0	T Cells
CNOT2	0	0.86856897	0.145	0.482	0	T Cells
EAPP	0	0.82928397	0.138	0.475	0	T Cells
FBXO462	0	0.72177077	0.133	0.47	0	T Cells
FBXO382	0	0.52344819	0.114	0.45	0	T Cells
CDC343	0	0.76995406	0.126	0.462	0	T Cells
NAA502	0	0.38012412	0.101	0.437	0	T Cells
FAF21	0	0.56672926	0.1	0.436	0	T Cells
GOPC1	0	0.90231681	0.146	0.482	0	T Cells
TNPO32	0	1.00300543	0.156	0.492	0	T Cells
MAPK61	0	0.80519699	0.134	0.47	0	T Cells
FEM1A1	0	0.36068628	0.094	0.43	0	T Cells
CCDC1591	0	0.17928928	0.096	0.432	0	T Cells
CCDC973	0	0.97155693	0.153	0.489	0	T Cells
TBC1D10A1	0	0.89190782	0.154	0.49	0	T Cells
MED132	0	0.93159381	0.149	0.484	0	T Cells
PSMG32	0	0.67226637	0.119	0.453	0	T Cells
PPIE2	0	0.58486775	0.119	0.452	0	T Cells
METTL5	0	0.53007712	0.11	0.443	0	T Cells
DIMT11	0	0.6968636	0.12	0.453	0	T Cells
SNX151	0	0.52324644	0.124	0.457	0	T Cells
SSH13	0	0.52602666	0.121	0.454	0	T Cells
BFAR	0	0.44402208	0.103	0.436	0	T Cells
OTUD41	0	0.90726156	0.151	0.484	0	T Cells
PSMA11	0	0.25696627	0.093	0.426	0	T Cells
ZFYVE1	0	0.19463934	0.09	0.423	0	T Cells
NDUFAF82	0	0.92237515	0.156	0.489	0	T Cells
TAB31	0	0.41382729	0.1	0.433	0	T Cells
DKC11	0	0.89321576	0.136	0.469	0	T Cells
EHMT21	0	0.5151461	0.108	0.44	0	T Cells
LSM73	0	0.72243921	0.129	0.461	0	T Cells
PRMT1	0	0.67888317	0.122	0.454	0	T Cells
ECHDC22	0	0.18492261	0.099	0.431	0	T Cells
RUFY33	0	0.17124451	0.093	0.425	0	T Cells
SPTLC13	0	0.29725264	0.094	0.426	0	T Cells
MFAP1	0	0.26581824	0.087	0.419	0	T Cells
VPS721	0	0.54914121	0.111	0.442	0	T Cells
C3orf382	0	0.76766504	0.12	0.451	0	T Cells
MAPK132	0	0.63616931	0.125	0.456	0	T Cells
PPP4R12	0	0.78499389	0.124	0.455	0	T Cells
CNOT91	0	0.95352759	0.151	0.482	0	T Cells
SGF291	0	0.35656494	0.101	0.432	0	T Cells
INO80C1	0	0.4412533	0.102	0.432	0	T Cells
SEPTIN12	0	7.96150892	0.346	0.016	0	T Cells
DIPK2A3	0	0.44954262	0.105	0.434	0	T Cells
MYBBP1A2	0	0.66958574	0.116	0.445	0	T Cells
ERI3	0	0.19345402	0.086	0.415	0	T Cells
FBXO111	0	1.00868797	0.147	0.476	0	T Cells
DNAJB21	0	0.35924695	0.101	0.43	0	T Cells

MPG	0	0.39001502	0.099	0.428	0	T Cells
TRIM652	0	0.12492571	0.081	0.41	0	T Cells
RNASEH11	0	0.66246167	0.114	0.442	0	T Cells
ACVR1	0	0.6316465	0.11	0.438	0	T Cells
GYG1	0	0.60958312	0.107	0.435	0	T Cells
ARFGAP21	0	0.7564361	0.124	0.452	0	T Cells
ABCB72	0	0.4780151	0.105	0.433	0	T Cells
PHACTR43	0	0.92506939	0.141	0.469	0	T Cells
C11orf54	0	0.11160822	0.084	0.412	0	T Cells
IDH3A2	0	0.12070991	0.081	0.409	0	T Cells
PRKAR2A2	0	0.74966025	0.12	0.447	0	T Cells
JAZF14	0	0.62620533	0.118	0.445	0	T Cells
TMEM682	0	0.63896408	0.116	0.443	0	T Cells
ARHGEF71	0	0.80654291	0.135	0.462	0	T Cells
GSE13	0	0.64153004	0.119	0.446	0	T Cells
ALYREF1	0	0.49815849	0.107	0.434	0	T Cells
RPP38	0	0.42869507	0.091	0.418	0	T Cells
ERCC3	0	0.70975025	0.12	0.446	0	T Cells
SEC24A3	0	0.79011276	0.124	0.45	0	T Cells
AOAH2	0	0.95641616	0.134	0.46	0	T Cells
NUP2142	0	0.70753418	0.114	0.44	0	T Cells
RPRD1A1	0	0.67430802	0.115	0.441	0	T Cells
MAP2K71	0	0.62130265	0.117	0.443	0	T Cells
MTFR1L2	0	0.22015567	0.093	0.419	0	T Cells
TMEM129	0	0.30526428	0.095	0.421	0	T Cells
XPO72	0	0.41405746	0.097	0.423	0	T Cells
PKIA1	0	0.19096369	0.101	0.427	0	T Cells
TLE41	0	0.36099265	0.097	0.423	0	T Cells
SGPL12	0	0.3414335	0.092	0.418	0	T Cells
RNF26	0	0.23217349	0.088	0.414	0	T Cells
NDUFA61	0	0.41507069	0.093	0.419	0	T Cells
WBP13	0	0.77538433	0.133	0.458	0	T Cells
RNF1031	0	0.69863811	0.123	0.448	0	T Cells
MCCC11	0	0.38069739	0.105	0.43	0	T Cells
PPID1	0	0.68887634	0.117	0.442	0	T Cells
PREP1	0	0.49520681	0.102	0.427	0	T Cells
AZIN11	0	0.90489718	0.135	0.46	0	T Cells
CSTF2T1	0	0.86501464	0.14	0.465	0	T Cells
FBXO282	0	0.6221216	0.112	0.436	0	T Cells
TMUB11	0	0.89999538	0.137	0.461	0	T Cells
NOP91	0	0.38118649	0.105	0.429	0	T Cells
NPRL3	0	0.37233946	0.102	0.426	0	T Cells
AAR2	0	0.19579368	0.082	0.406	0	T Cells
URM1	0	0.22343882	0.089	0.413	0	T Cells
TBC1D144	0	0.82378744	0.139	0.462	0	T Cells
RIC12	0	0.44767229	0.105	0.428	0	T Cells
CLNS1A1	0	0.89107797	0.136	0.459	0	T Cells
TUBGCP31	0	0.49862971	0.107	0.43	0	T Cells
CCDC322	0	0.13498487	0.083	0.406	0	T Cells
TRAPPC82	0	0.85580985	0.13	0.453	0	T Cells
UGDH	0	0.34278696	0.087	0.41	0	T Cells
OSTC3	0	0.4333209	0.088	0.411	0	T Cells

LMBRD2	0	0.52410653	0.101	0.424	0	T Cells
SPPL31	0	0.44420337	0.097	0.42	0	T Cells
DDX19A1	0	0.48128097	0.097	0.42	0	T Cells
PHF20	0	0.21276424	0.086	0.409	0	T Cells
FNIP14	0	0.76791755	0.112	0.434	0	T Cells
GTF2E21	0	0.87709622	0.131	0.453	0	T Cells
UTP231	0	0.69806912	0.11	0.432	0	T Cells
RPAP3	0	0.63772079	0.105	0.427	0	T Cells
EMC62	0	0.64164815	0.113	0.435	0	T Cells
RNF1261	0	0.72657234	0.121	0.443	0	T Cells
FCHSD23	0	0.44173886	0.099	0.421	0	T Cells
MBD42	0	0.6011828	0.107	0.428	0	T Cells
LSG11	0	0.88071487	0.134	0.455	0	T Cells
APC2	0	0.14475305	0.079	0.4	0	T Cells
BLOC1S62	0	0.27652987	0.085	0.406	0	T Cells
PDPK12	0	0.57541557	0.102	0.423	0	T Cells
AIFM11	0	0.20907049	0.084	0.405	0	T Cells
EIF2D1	0	0.35748402	0.092	0.413	0	T Cells
SESN13	0	0.39151774	0.095	0.416	0	T Cells
LCK	0	10.3454161	0.322	0.002	0	T Cells
BCL65	0	0.89348184	0.14	0.46	0	T Cells
WDR372	0	0.68011744	0.125	0.445	0	T Cells
USP82	0	0.73631122	0.123	0.443	0	T Cells
MED242	0	0.68019672	0.115	0.435	0	T Cells
DHPS	0	0.83469194	0.131	0.451	0	T Cells
CEBPD4	0	0.22820959	0.099	0.419	0	T Cells
PSMC61	0	0.54012677	0.096	0.416	0	T Cells
RUVBL1	0	0.64443444	0.111	0.43	0	T Cells
FEM1C2	0	0.68305915	0.11	0.429	0	T Cells
PHAX1	0	0.77915646	0.116	0.435	0	T Cells
CWF19L11	0	0.77615266	0.126	0.445	0	T Cells
XRCC1	0	0.55460627	0.109	0.428	0	T Cells
NGDN	0	0.59024516	0.099	0.418	0	T Cells
COG72	0	0.13153313	0.087	0.405	0	T Cells
STRIP11	0	0.83717207	0.13	0.448	0	T Cells
C6orf89	0	0.76532664	0.117	0.435	0	T Cells
CDC402	0	0.67859779	0.105	0.423	0	T Cells
POM1211	0	0.76116568	0.116	0.434	0	T Cells
PELP11	0	0.56431812	0.104	0.422	0	T Cells
FTSJ31	0	0.45894989	0.098	0.416	0	T Cells
UBR32	0	0.81028751	0.125	0.442	0	T Cells
LTV11	0	0.23759278	0.082	0.399	0	T Cells
SAMD81	0	0.54284547	0.109	0.426	0	T Cells
UBN12	0	0.51606602	0.105	0.422	0	T Cells
NXT11	0	0.77710051	0.115	0.432	0	T Cells
THUMPD31	0	0.44476701	0.094	0.411	0	T Cells
RABGEF13	0	0.38199262	0.09	0.407	0	T Cells
UVRAG4	0	0.31704477	0.092	0.409	0	T Cells
PIK3R41	0	0.64981617	0.107	0.423	0	T Cells
CCDC822	0	0.92088627	0.13	0.446	0	T Cells
FASTK	0	0.34978504	0.09	0.405	0	T Cells
ELF22	0	0.70661293	0.117	0.432	0	T Cells

SRRT1	0	0.54281517	0.105	0.42	0	T Cells
CCDC90B2	0	0.65959818	0.107	0.422	0	T Cells
IMP3	0	0.6074059	0.107	0.422	0	T Cells
CCNC2	0	0.5628968	0.099	0.414	0	T Cells
RBM71	0	0.4846115	0.095	0.41	0	T Cells
PXK3	0	0.53309433	0.103	0.417	0	T Cells
PLPP12	0	0.10236614	0.078	0.392	0	T Cells
MAFK2	0	0.6290445	0.115	0.429	0	T Cells
SAP30BP2	0	0.67877258	0.121	0.435	0	T Cells
DCXR1	0	0.63371399	0.113	0.427	0	T Cells
NUP502	0	0.90350627	0.128	0.442	0	T Cells
FAM136A3	0	0.45470663	0.093	0.407	0	T Cells
TBL22	0	0.34224765	0.093	0.406	0	T Cells
ALG51	0	0.51649657	0.092	0.405	0	T Cells
SLC25A28	0	0.79749187	0.122	0.435	0	T Cells
ARL2BP1	0	0.73438302	0.11	0.423	0	T Cells
MFSD113	0	0.18349515	0.088	0.4	0	T Cells
PAM161	0	0.792181	0.125	0.437	0	T Cells
RFNG1	0	0.19513793	0.083	0.395	0	T Cells
ANXA64	0	5.09051099	0.407	0.095	0	T Cells
NCOA71	0	0.42035857	0.095	0.407	0	T Cells
TMEM214	0	0.22101344	0.086	0.397	0	T Cells
WDR55	0	0.21081633	0.087	0.398	0	T Cells
TMEM120A	0	0.27593827	0.086	0.397	0	T Cells
PIP5K1A	0	0.74790684	0.117	0.428	0	T Cells
MPPE12	0	0.56813234	0.11	0.421	0	T Cells
EBAG92	0	0.42575021	0.093	0.403	0	T Cells
PARN3	0	0.44803764	0.088	0.398	0	T Cells
ATPAF1	0	0.79167969	0.119	0.429	0	T Cells
SH2D3C2	0	0.45429249	0.118	0.428	0	T Cells
PDE6D1	0	0.41446254	0.093	0.402	0	T Cells
RANGAP11	0	0.38697952	0.086	0.395	0	T Cells
EIF4G31	0	0.68836816	0.124	0.433	0	T Cells
FASTKD21	0	0.6728321	0.114	0.423	0	T Cells
LRRC581	0	0.28882151	0.084	0.393	0	T Cells
ABHD153	0	0.14885907	0.09	0.398	0	T Cells
PNO11	0	0.3282475	0.081	0.389	0	T Cells
EXOSC71	0	0.66052289	0.105	0.413	0	T Cells
PTPRE4	0	0.44400045	0.118	0.426	0	T Cells
ZC3H7A1	0	0.77040498	0.119	0.427	0	T Cells
WIZ1	0	0.75641069	0.118	0.426	0	T Cells
RRP11	0	0.20346886	0.082	0.39	0	T Cells
ARMC101	0	0.40990387	0.089	0.396	0	T Cells
INTS14	0	0.58315678	0.098	0.405	0	T Cells
ZAP70	0	9.91654305	0.309	0.002	0	T Cells
NUCB2	0	0.23457064	0.081	0.388	0	T Cells
TRIP43	0	0.65629308	0.116	0.423	0	T Cells
ZMAT32	0	0.45109431	0.1	0.407	0	T Cells
SF3B6	0	0.27545707	0.086	0.392	0	T Cells
COPS8	0	0.46366523	0.098	0.404	0	T Cells
ODR41	0	0.28718523	0.079	0.385	0	T Cells
DNAJB142	0	0.77679391	0.111	0.417	0	T Cells

SAE11	0	0.61418726	0.113	0.419	0	T Cells
GRAP2	0	10.1105119	0.308	0.002	0	T Cells
TRMT63	0	0.2432795	0.086	0.391	0	T Cells
SNRPG	0	0.26022338	0.081	0.386	0	T Cells
DHX402	0	0.63187624	0.102	0.407	0	T Cells
GET1	0	0.26172981	0.082	0.387	0	T Cells
PCGF12	0	0.34443945	0.087	0.391	0	T Cells
FBXO252	0	0.50069592	0.088	0.392	0	T Cells
CMTR1	0	0.67652171	0.114	0.418	0	T Cells
ZKSCAN11	0	0.68344571	0.111	0.415	0	T Cells
ZPR11	0	0.69089169	0.105	0.409	0	T Cells
POLR2C2	0	0.77753088	0.113	0.417	0	T Cells
TRMT2A2	0	0.19748539	0.08	0.384	0	T Cells
NOC2L2	0	0.62260165	0.102	0.405	0	T Cells
CRY21	0	0.60962016	0.101	0.404	0	T Cells
LLPH	0	0.49040055	0.091	0.394	0	T Cells
TIMM441	0	0.52146425	0.1	0.403	0	T Cells
DMAP12	0	0.66975256	0.107	0.41	0	T Cells
AKAP14	0	0.7018513	0.111	0.414	0	T Cells
PTBP12	0	0.62564668	0.111	0.414	0	T Cells
NFS11	0	0.14998247	0.08	0.383	0	T Cells
UFL12	0	0.57504109	0.098	0.4	0	T Cells
PDSS2	0	0.35022101	0.09	0.392	0	T Cells
MORF4L12	0	0.43877468	0.094	0.396	0	T Cells
ZNF428	0	0.4681338	0.094	0.396	0	T Cells
KRCC11	0	0.68755541	0.111	0.413	0	T Cells
SENP51	0	0.69339686	0.111	0.413	0	T Cells
SNAP233	0	0.13548675	0.08	0.382	0	T Cells
IFT27	0	0.254767	0.082	0.384	0	T Cells
LUZP11	0	0.72101297	0.099	0.4	0	T Cells
CYREN2	0	0.34872907	0.094	0.395	0	T Cells
TRAPPC131	0	0.12118021	0.074	0.375	0	T Cells
DIAPH21	0	0.14197383	0.08	0.381	0	T Cells
FAM20B1	0	0.57484481	0.095	0.395	0	T Cells
PMPCA	0	0.6011212	0.097	0.397	0	T Cells
HPS41	0	0.60099949	0.099	0.399	0	T Cells
PIGX1	0	0.13258107	0.083	0.383	0	T Cells
AP1AR2	0	0.2768981	0.081	0.381	0	T Cells
ANO62	0	0.28983085	0.081	0.381	0	T Cells
TAF41	0	0.75015918	0.106	0.405	0	T Cells
EMB3	0	7.34733714	0.312	0.013	0	T Cells
PNPLA82	0	0.21059339	0.077	0.376	0	T Cells
PIGQ	0	0.18341402	0.081	0.38	0	T Cells
NIP71	0	0.37472522	0.085	0.384	0	T Cells
SMIM261	0	0.35350397	0.085	0.384	0	T Cells
FXR22	0	0.63602425	0.1	0.398	0	T Cells
NFATC23	0	6.40297507	0.339	0.041	0	T Cells
HPRT11	0	0.65097384	0.101	0.399	0	T Cells
TGFBRAP1	0	0.2808036	0.085	0.383	0	T Cells
TUSC2	0	0.12508568	0.077	0.375	0	T Cells
ABHD14A1	0	0.27569234	0.078	0.376	0	T Cells
ADH5	0	0.18570505	0.075	0.373	0	T Cells

NOL71	0	0.41891539	0.085	0.383	0	T Cells
BORCS71	0	0.30477036	0.074	0.372	0	T Cells
XPO13	0	0.68388153	0.102	0.399	0	T Cells
TMED8	0	0.6549244	0.096	0.393	0	T Cells
FAM210B1	0	0.41477401	0.086	0.383	0	T Cells
HSPA13	0	0.69978063	0.096	0.393	0	T Cells
CD3D	0	5.3767125	0.448	0.152	0	T Cells
STAMBP1	0	0.12273308	0.073	0.369	0	T Cells
BCL11B	0	10.3950506	0.298	0.002	0	T Cells
RNF81	0	0.4615112	0.09	0.385	0	T Cells
BLZF11	0	0.36899811	0.082	0.377	0	T Cells
UBE2E12	0	0.19443456	0.078	0.373	0	T Cells
PIGN1	0	0.40536393	0.083	0.378	0	T Cells
VWA82	0	0.50708323	0.094	0.388	0	T Cells
DIS3L1	0	0.68981088	0.098	0.392	0	T Cells
ASPSCR12	0	0.45699045	0.093	0.387	0	T Cells
ALG12	0	0.60494747	0.096	0.39	0	T Cells
CCT32	0	0.22258437	0.074	0.368	0	T Cells
MPLKIP	0	0.39851501	0.085	0.379	0	T Cells
PLGRKT1	0	0.14574218	0.076	0.37	0	T Cells
CC2D1B1	0	0.66644805	0.101	0.394	0	T Cells
BAP11	0	0.37825678	0.09	0.383	0	T Cells
DYNC1H12	0	0.48002468	0.096	0.389	0	T Cells
CHMP6	0	0.37211596	0.086	0.379	0	T Cells
HDAC32	0	0.19528079	0.077	0.37	0	T Cells
JHY1	0	0.3499835	0.072	0.365	0	T Cells
SLC35B22	0	0.43150997	0.096	0.388	0	T Cells
RRS11	0	0.66388251	0.099	0.391	0	T Cells
TTLL122	0	0.33915232	0.086	0.378	0	T Cells
CD803	0	0.27898487	0.075	0.367	0	T Cells
TMCO31	0	0.23052243	0.078	0.37	0	T Cells
GSKIP1	0	0.3210514	0.079	0.371	0	T Cells
FBXO222	0	0.41607282	0.081	0.373	0	T Cells
TBL31	0	0.26916473	0.079	0.371	0	T Cells
CST73	0	7.7047528	0.296	0.004	0	T Cells
STRN32	0	0.62341803	0.099	0.39	0	T Cells
NOA12	0	0.52339622	0.089	0.379	0	T Cells
TRIM411	0	0.32709278	0.091	0.381	0	T Cells
ACTL6A	0	0.45985339	0.085	0.375	0	T Cells
ABCB101	0	0.52216171	0.092	0.381	0	T Cells
RRM1	0	0.38680041	0.096	0.385	0	T Cells
UBE2N1	0	0.57524505	0.089	0.378	0	T Cells
TFDP11	0	0.4654952	0.091	0.38	0	T Cells
SDF22	0	0.31187824	0.077	0.366	0	T Cells
CRIM11	0	0.50928699	0.097	0.385	0	T Cells
MTMR41	0	0.43539181	0.085	0.373	0	T Cells
RMND5B1	0	0.10524715	0.069	0.356	0	T Cells
ALG1	0	0.19862464	0.072	0.359	0	T Cells
TECR	0	0.20461847	0.078	0.365	0	T Cells
ESF12	0	0.63707601	0.089	0.375	0	T Cells
IFT571	0	0.18613191	0.073	0.359	0	T Cells
TMEM209	0	0.44972038	0.079	0.365	0	T Cells

CREB3	0	0.30260385	0.073	0.359	0	T Cells
MCUR1	0	0.54776411	0.088	0.373	0	T Cells
RBBP51	0	0.37155362	0.081	0.366	0	T Cells
NUDT191	0	0.26595453	0.078	0.363	0	T Cells
PEX26	0	0.39526223	0.08	0.365	0	T Cells
RBM181	0	0.54618857	0.094	0.378	0	T Cells
SPOCK2	0	10.0745666	0.286	0.002	0	T Cells
TIMM91	0	0.4831605	0.085	0.369	0	T Cells
C1QBP3	0	0.29365734	0.072	0.356	0	T Cells
SPPL2B2	0	0.44983039	0.087	0.37	0	T Cells
UROS2	0	0.16917342	0.075	0.358	0	T Cells
SNW11	0	0.4334755	0.083	0.366	0	T Cells
ABCD3	0	0.33019079	0.076	0.358	0	T Cells
SYPL12	0	0.19373251	0.07	0.352	0	T Cells
SELENOO1	0	0.24428832	0.082	0.364	0	T Cells
NDUFA12	0	0.3902049	0.075	0.356	0	T Cells
TAB12	0	0.39541795	0.082	0.363	0	T Cells
ACP11	0	0.5059906	0.085	0.365	0	T Cells
EEFSEC	0	0.21632771	0.075	0.354	0	T Cells
TTC9C2	0	0.40861765	0.082	0.361	0	T Cells
AK61	0	0.14032225	0.068	0.346	0	T Cells
RIOK21	0	0.29487842	0.068	0.346	0	T Cells
MED301	0	0.19019951	0.068	0.346	0	T Cells
RHBDF21	0	0.14573749	0.077	0.355	0	T Cells
MOB1B4	0	0.23835169	0.073	0.349	0	T Cells
TYW32	0	0.13306588	0.071	0.346	0	T Cells
TSR11	0	0.45757781	0.077	0.352	0	T Cells
GPKOW1	0	0.34454696	0.078	0.353	0	T Cells
PJA1	0	0.36009266	0.077	0.352	0	T Cells
DDI21	0	0.31830518	0.079	0.353	0	T Cells
FAM210A1	0	0.42989544	0.075	0.349	0	T Cells
C2orf69	0	0.41513464	0.075	0.348	0	T Cells
PRF1	0	10.6348385	0.274	0.002	0	T Cells
THOP11	0	0.15707396	0.068	0.34	0	T Cells
NKG71	0	9.19901221	0.279	0.007	0	T Cells
HSD17B82	0	0.17662856	0.066	0.337	0	T Cells
CD51	0	6.36009551	0.32	0.05	0	T Cells
C11orf242	0	0.21797387	0.07	0.338	0	T Cells
VPS501	0	0.14014675	0.068	0.334	0	T Cells
FAM102A	0	5.71329171	0.354	0.088	0	T Cells
LTB2	0	5.65702746	0.446	0.181	0	T Cells
GIMAP71	0	5.65076415	0.37	0.105	0	T Cells
MBIP	0	0.14303912	0.068	0.333	0	T Cells
LIMD24	0	6.22956721	0.279	0.019	0	T Cells
ATP2A34	0	5.82506777	0.306	0.047	0	T Cells
KLRB1	0	10.300328	0.26	0.002	0	T Cells
PRDM12	0	6.27675687	0.301	0.044	0	T Cells
CD6	0	5.3115492	0.395	0.138	0	T Cells
LBH	0	7.37904665	0.275	0.019	0	T Cells
CD3G	0	7.47390952	0.275	0.02	0	T Cells
KLRK1	0	10.4721428	0.255	0.002	0	T Cells
PDE4D1	0	7.28702402	0.27	0.018	0	T Cells

SKAP1	0	5.36491863	0.33	0.084	0	T Cells
TRAT1	0	10.0091932	0.244	0.002	0	T Cells
CD40LG	0	10.3565625	0.242	0.001	0	T Cells
CD247	0	10.440147	0.239	0.001	0	T Cells
ZFP36L23	0	1.79639947	0.715	0.953	0	T Cells
SAMD3	0	10.4016953	0.237	0.001	0	T Cells
PTPN73	0	7.02373329	0.245	0.014	0	T Cells
PYHIN1	0	7.36733827	0.249	0.019	0	T Cells
ATP8B21	0	8.36547232	0.232	0.005	0	T Cells
CD8A	0	10.8653955	0.228	0.001	0	T Cells
DPP42	0	9.40632164	0.229	0.002	0	T Cells
GPR1712	0	9.47287114	0.229	0.002	0	T Cells
GPR1833	0	5.71519041	0.239	0.012	0	T Cells
FLT3LG	0	5.67766494	0.301	0.075	0	T Cells
ITM2C2	0	8.00088171	0.229	0.004	0	T Cells
RFLNB3	0	7.8042164	0.227	0.008	0	T Cells
NLRC3	0	10.4233428	0.219	0.001	0	T Cells
CCDC88C1	0	5.78611084	0.261	0.044	0	T Cells
PLCG1	0	5.41087732	0.308	0.092	0	T Cells
STAT41	0	7.35929005	0.224	0.013	0	T Cells
CHD35	0	4.90801	0.312	0.101	0	T Cells
ARL4C4	0	4.4248377	0.359	0.15	0	T Cells
FYN1	0	5.15372244	0.305	0.098	0	T Cells
CXCR32	0	9.52266595	0.208	0.001	0	T Cells
ATP2B43	0	4.35089003	0.466	0.26	0	T Cells
SLA21	0	9.65046198	0.203	0.001	0	T Cells
TENT5C1	0	7.04396073	0.22	0.019	0	T Cells
HOPX	0	7.60933048	0.208	0.008	0	T Cells
WIPF12	0	1.90711184	0.751	0.951	0	T Cells
IKZF12	0	2.1096857	0.708	0.907	0	T Cells
ISG204	0	5.48119036	0.241	0.042	0	T Cells
ST8SIA45	0	5.04080836	0.273	0.076	0	T Cells
SLAMF1	0	8.38832451	0.202	0.006	0	T Cells
SH2D2A	0	9.79585888	0.196	0.001	0	T Cells
P2RY102	0	8.19664728	0.197	0.002	0	T Cells
CCR23	0	6.31359256	0.196	0.009	0	T Cells
CYTIP2	0	2.01604719	0.668	0.855	0	T Cells
ITM2A	0	9.77422513	0.187	0.001	0	T Cells
TNFRSF251	0	9.39866933	0.185	0.002	0	T Cells
ARHGAP454	0	4.21860318	0.328	0.147	0	T Cells
DUSP43	0	7.20346058	0.185	0.004	0	T Cells
TUBA4A	0	7.33336808	0.191	0.011	0	T Cells
TRAF14	0	5.62346964	0.219	0.039	0	T Cells
SLFN12L2	0	7.95837138	0.187	0.008	0	T Cells
JAK34	0	5.1691888	0.237	0.059	0	T Cells
PPP1R16B2	0	8.64350145	0.179	0.002	0	T Cells
SH2D1A	0	9.86870523	0.178	0.001	0	T Cells
TRIB21	0	6.49570908	0.2	0.025	0	T Cells
CCR4	0	10.1565993	0.174	0.001	0	T Cells
ENO23	0	8.47483412	0.174	0.003	0	T Cells
EVL	0	0.87512735	0.813	0.982	0	T Cells
TCF7	0	7.73464153	0.183	0.014	0	T Cells

CD7	0	6.03289961	0.206	0.038	0	T Cells
CD2262	0	7.4901175	0.176	0.008	0	T Cells
SLAMF6	0	5.78055196	0.219	0.052	0	T Cells
ST6GAL13	0	5.35075637	0.212	0.05	0	T Cells
LY92	0	9.09907369	0.162	0.002	0	T Cells
ADAM84	0	5.39416142	0.172	0.014	0	T Cells
PRKCQ	0	7.56687989	0.166	0.01	0	T Cells
MATK3	0	6.82597554	0.165	0.009	0	T Cells
PRKCB5	0	4.745526	0.206	0.051	0	T Cells
PSTPIP14	0	4.30382876	0.271	0.117	0	T Cells
DGKA3	0	4.40181823	0.339	0.186	0	T Cells
GPR1741	0	9.7510223	0.153	0.001	0	T Cells
CD28	0	9.22201312	0.153	0.002	0	T Cells
CAMK4	0	8.12172528	0.156	0.005	0	T Cells
CARD113	0	5.90193622	0.182	0.032	0	T Cells
KIF21B	0	5.0175381	0.228	0.079	0	T Cells
GPR1325	0	4.4721686	0.204	0.055	0	T Cells
P2RY82	0	8.58253744	0.15	0.002	0	T Cells
RUNX23	0	5.06572755	0.209	0.062	0	T Cells
AHNAK4	0	1.36462015	0.838	0.984	0	T Cells
DOCK9	0	8.22013034	0.149	0.003	0	T Cells
OBSCN2	0	6.80284369	0.16	0.015	0	T Cells
ANTXR25	0	5.58689063	0.166	0.022	0	T Cells
PATJ1	0	7.85510782	0.148	0.004	0	T Cells
CARMIL22	0	7.4333162	0.152	0.008	0	T Cells
GZMK	0	10.9788994	0.142	0.001	0	T Cells
SMAD33	0	7.22037507	0.145	0.004	0	T Cells
SYTL2	0	6.5372783	0.155	0.015	0	T Cells
SLC7A54	0	4.71323503	0.189	0.049	0	T Cells
KLF122	0	5.58737559	0.182	0.043	0	T Cells
CDC14A1	0	5.00365836	0.216	0.078	0	T Cells
KLF64	0	2.39717781	0.689	0.826	0	T Cells
NELL2	0	9.36125267	0.138	0.001	0	T Cells
TESPA12	0	8.20275181	0.14	0.004	0	T Cells
BTG14	0	2.22327202	0.767	0.899	0	T Cells
TRAF52	0	4.95189554	0.199	0.068	0	T Cells
BICDL11	0	6.52133679	0.146	0.016	0	T Cells
KLRD1	0	9.63690014	0.13	0.002	0	T Cells
C12orf752	0	7.88441244	0.128	0.002	0	T Cells
CCL42	0	3.98484726	0.178	0.053	0	T Cells
ZEB12	0	8.62588963	0.123	0.001	0	T Cells
ZNF507	0	8.21262072	0.124	0.003	0	T Cells
ZNF683	0	10.4755915	0.121	0.001	0	T Cells
GNLY1	0	10.1060371	0.12	0.001	0	T Cells
TIGIT	0	10.1689699	0.12	0.001	0	T Cells
SIT1	0	9.8757987	0.119	0.001	0	T Cells
LAG31	0	7.62949329	0.124	0.006	0	T Cells
IL18RAP2	0	8.76277986	0.118	0.001	0	T Cells
TRERF13	0	6.60037549	0.126	0.01	0	T Cells
LINC02694	0	9.99379215	0.116	0.001	0	T Cells
GZMM	0	9.42555429	0.116	0.001	0	T Cells
TMEM200A	0	10.3940872	0.115	0.001	0	T Cells

GZMH	0	11.0914495	0.113	0	0	T Cells
CTLA42	0	9.14325018	0.111	0.001	0	T Cells
TBX21	0	10.0170972	0.11	0.001	0	T Cells
CLSTN32	0	5.27406837	0.145	0.036	0	T Cells
PXN3	0	5.30987142	0.129	0.021	0	T Cells
TNFRSF182	0	9.11403663	0.108	0.001	0	T Cells
PLEKHF1	0	7.19010546	0.113	0.008	0	T Cells
TNFSF141	0	5.79297256	0.121	0.016	0	T Cells
S1PR12	0	8.83896972	0.105	0.002	0	T Cells
PBX4	0	7.42751459	0.109	0.006	0	T Cells
ZNRF13	0	5.92141472	0.118	0.016	0	T Cells
MAST41	0	6.94757741	0.11	0.009	0	T Cells
RASSF24	0	4.36109194	0.123	0.024	0	T Cells
UBASH3A	0	9.50663116	0.1	0.001	0	T Cells
DMWD2	0	7.77178259	0.103	0.004	0	T Cells
NBL11	0	8.39349203	0.1	0.002	0	T Cells
GZMB1	0	8.59221827	0.099	0.001	0	T Cells
RNF157	0	5.86601188	0.125	0.027	0	T Cells
LAX1	0	8.10800534	0.102	0.004	0	T Cells
FCMR2	0	6.44887507	0.108	0.011	0	T Cells
ABLIM1	0	8.31413831	0.097	0.001	0	T Cells
CD200R13	0	7.21498901	0.097	0.003	0	T Cells
SCML4	0	9.88237095	0.094	0	0	T Cells
CDK63	0	6.27249092	0.107	0.014	0	T Cells
FASLG	0	10.5877524	0.092	0	0	T Cells
CCR63	0	5.43997597	0.1	0.009	0	T Cells
GPR683	0	7.78543398	0.093	0.002	0	T Cells
LIME11	0	5.32883696	0.119	0.028	0	T Cells
APOBEC3D	0	7.3721506	0.096	0.006	0	T Cells
MYBL13	0	6.03645553	0.105	0.016	0	T Cells
IL12RB2	0	9.33281431	0.089	0.001	0	T Cells
Z82206.1	0	10.1000757	0.088	0	0	T Cells
SLC2A15	0	5.57519411	0.102	0.015	0	T Cells
RGS162	0	8.51533665	0.089	0.002	0	T Cells
LEF11	0	9.6631661	0.088	0.001	0	T Cells
TSPAN22	0	8.76796676	0.087	0.001	0	T Cells
PERP	0	6.24588779	0.092	0.007	0	T Cells
AUTS22	0	7.23531799	0.088	0.003	0	T Cells
CD27	0	9.81943197	0.086	0.001	0	T Cells
SLC16A73	0	6.06505309	0.097	0.014	0	T Cells
IL21R4	0	5.17699824	0.099	0.016	0	T Cells
GPR25	0	10.0258155	0.082	0	0	T Cells
ANO91	0	8.58847383	0.083	0.001	0	T Cells
KLRC4	0	10.110113	0.083	0.001	0	T Cells
TMEM712	0	4.69879831	0.103	0.022	0	T Cells
STMN3	0	9.2406528	0.081	0.001	0	T Cells
KIF5C	0	9.84226422	0.08	0.001	0	T Cells
ADARB11	0	6.66603938	0.086	0.008	0	T Cells
NPDC12	0	7.41307624	0.079	0.003	0	T Cells
KLRC1	0	10.9409659	0.076	0	0	T Cells
IL18R13	0	7.31052367	0.078	0.003	0	T Cells
CSGALNACT12	0	7.80568021	0.076	0.001	0	T Cells

ITGA43	0	3.63340176	0.564	0.489	0	T Cells
IRF43	0	5.26871293	0.083	0.01	0	T Cells
TOX3	0	8.23187828	0.074	0.001	0	T Cells
ST8SIA12	0	9.39988262	0.073	0	0	T Cells
IFNG	0	9.52298149	0.072	0	0	T Cells
MCTP21	0	7.1853871	0.074	0.002	0	T Cells
ADORA2A3	0	5.93227497	0.076	0.004	0	T Cells
CLU1	0	5.51309927	0.076	0.005	0	T Cells
IFITM11	0	8.62496676	0.072	0.001	0	T Cells
DAPK22	0	7.5054082	0.073	0.003	0	T Cells
GFI11	0	6.56529558	0.077	0.008	0	T Cells
ABCD21	0	9.36806992	0.068	0.001	0	T Cells
TMEM204	0	9.78572299	0.068	0.001	0	T Cells
HLF	0	9.57122784	0.068	0.001	0	T Cells
TRDC	0	8.40345936	0.072	0.005	0	T Cells
ITPRIPL13	0	8.05898912	0.067	0.002	0	T Cells
PDCD12	0	9.64517635	0.065	0	0	T Cells
AFAP1	0	6.8133516	0.072	0.007	0	T Cells
SIDT1	0	8.79314501	0.065	0.001	0	T Cells
KIF21A	0	6.89267444	0.066	0.002	0	T Cells
MYO1D3	0	5.64886746	0.073	0.01	0	T Cells
ZNF831	0	9.32352455	0.064	0.001	0	T Cells
SLC37A33	0	6.7350228	0.065	0.003	0	T Cells
EPHA1	0	8.19147653	0.063	0.002	0	T Cells
APOBEC3H	0	6.84786015	0.065	0.005	0	T Cells
EOMES	0	10.7355217	0.059	0	0	T Cells
KLRC3	0	10.0829659	0.059	0	0	T Cells
CCR73	0	6.42684336	0.059	0.001	0	T Cells
MSC1	0	8.98249586	0.056	0.001	0	T Cells
PPP2R2B2	0	7.62535473	0.056	0.002	0	T Cells
MCF2L2	0	6.83394569	0.058	0.005	0	T Cells
SYNE1	0	5.35005224	0.061	0.008	0	T Cells
SH2D3A	0	7.06113177	0.056	0.003	0	T Cells
NSG1	0	9.84559728	0.05	0	0	T Cells
TMEM1561	0	7.62056402	0.052	0.002	0	T Cells
F2R	0	8.25882638	0.052	0.002	0	T Cells
LSR3	0	6.11622593	0.053	0.004	0	T Cells
TGFBR31	0	9.18133398	0.048	0.001	0	T Cells
CLIC5	0	7.43942094	0.049	0.002	0	T Cells
WHRN2	0	6.10400057	0.051	0.004	0	T Cells
DEC11	0	9.08615358	0.047	0	0	T Cells
TESC1	0	7.63737757	0.049	0.002	0	T Cells
TTC9	0	6.11495532	0.05	0.003	0	T Cells
FOXP3	0	9.41067007	0.048	0.001	0	T Cells
PRDM84	0	6.48407632	0.048	0.002	0	T Cells
GDF111	0	9.69809451	0.046	0	0	T Cells
L1CAM2	0	8.74434317	0.046	0.001	0	T Cells
RESF11	0	3.57557715	0.658	0.614	0	T Cells
XCL2	0	9.90368036	0.044	0	0	T Cells
SLC38A52	0	8.16894825	0.045	0.001	0	T Cells
CCDC1411	0	9.87388039	0.043	0	0	T Cells
NCR3	0	6.9064573	0.047	0.005	0	T Cells

LTA1	0	8.47763134	0.043	0.001	0	T Cells
CLIC32	0	6.73985144	0.043	0.001	0	T Cells
DMTN	0	6.63265746	0.045	0.004	0	T Cells
RORC1	0	8.32663229	0.041	0.001	0	T Cells
PLXDC11	0	8.89229629	0.04	0	0	T Cells
FBXL16	0	7.90771828	0.041	0.002	0	T Cells
IL9R	0	9.9743939	0.039	0	0	T Cells
SETBP12	0	6.33146284	0.042	0.004	0	T Cells
PTGDR	0	10.8092689	0.038	0	0	T Cells
CERS43	0	5.88714608	0.041	0.003	0	T Cells
PLCXD2	0	6.87993407	0.04	0.003	0	T Cells
SLC35F23	0	7.0057835	0.038	0.001	0	T Cells
DMPK1	0	7.74905089	0.038	0.001	0	T Cells
NCALD2	0	7.14777258	0.037	0.001	0	T Cells
APBA2	0	9.98275499	0.035	0	0	T Cells
TOX21	0	8.87143436	0.035	0	0	T Cells
SEMA4C3	0	6.77559176	0.036	0.001	0	T Cells
TSPAN5	0	8.91516498	0.036	0.001	0	T Cells
TMPRSS3	0	7.496085	0.036	0.001	0	T Cells
CRYBG2	0	7.66096227	0.035	0.001	0	T Cells
RASGRF2	0	8.23786747	0.035	0.001	0	T Cells
SIRPG	0	10.3833733	0.034	0	0	T Cells
TNFRSF42	0	7.55731153	0.035	0.002	0	T Cells
B3GALT2	0	11.4090064	0.033	0	0	T Cells
NUGGC1	0	9.52514283	0.033	0	0	T Cells
POU6F13	0	7.21604105	0.035	0.002	0	T Cells
ZNF8272	0	8.36332949	0.033	0.001	0	T Cells
BCAS41	0	7.79062382	0.033	0.001	0	T Cells
EDARADD1	0	7.79839979	0.032	0.001	0	T Cells
DTHD1	0	5.41333732	0.032	0.001	0	T Cells
ABCB11	0	9.14360453	0.031	0	0	T Cells
COL5A1	0	9.88607539	0.031	0	0	T Cells
RAB27B	0	8.8439789	0.031	0	0	T Cells
TTN1	0	10.4380511	0.03	0	0	T Cells
APBB1	0	7.25914358	0.032	0.002	0	T Cells
CYP4F22	0	10.750542	0.03	0	0	T Cells
IL26	0	10.1911082	0.03	0	0	T Cells
IL23R	0	8.82708409	0.028	0.001	0	T Cells
CLNK2	0	6.10381421	0.028	0.001	0	T Cells
ELOVL61	0	7.62514144	0.028	0.001	0	T Cells
DTX3	0	6.61671868	0.028	0.001	0	T Cells
CPNE7	0	7.84307883	0.028	0.001	0	T Cells
EVC2	0	8.05169538	0.027	0.001	0	T Cells
CCDC1362	0	8.05518777	0.027	0.001	0	T Cells
ADGRG11	0	6.77091519	0.027	0.001	0	T Cells
EPHA4	0	9.58707028	0.025	0	0	T Cells
BACH22	0	9.28943692	0.025	0	0	T Cells
SLC27A2	0	6.43725784	0.026	0.001	0	T Cells
WNT10A1	0	8.4545037	0.024	0	0	T Cells
SYNGR31	0	7.77672026	0.025	0.001	0	T Cells
RGL4	0	7.3547524	0.024	0	0	T Cells
NCAM1	0	9.10005014	0.023	0	0	T Cells

WNT10B	0	9.4491771	0.023	0	0	T Cells
CD701	0	7.95379683	0.023	0	0	T Cells
CAMK2N1	0	7.40848664	0.021	0	0	T Cells
BTBD111	0	7.74433648	0.021	0	0	T Cells
NCR1	0	7.83175671	0.02	0	0	T Cells
TSHZ2	0	6.87920124	0.021	0.001	0	T Cells
SCUBE11	0	8.13015265	0.02	0	0	T Cells
PPIAL4G	0	8.88692769	0.019	0	0	T Cells
CD8B21	0	9.58092621	0.019	0	0	T Cells
FBLN71	0	8.2070331	0.019	0	0	T Cells
CRMP1	0	9.01482799	0.019	0	0	T Cells
N4BP32	0	7.60034793	0.02	0.001	0	T Cells
NTNG2	0	8.06266661	0.019	0	0	T Cells
PLCH22	0	7.50632769	0.018	0	0	T Cells
SH2D1B	0	8.59834682	0.017	0	0	T Cells
CCDC102A2	0	7.43783674	0.017	0	0	T Cells
ACSL6	0	10.3501576	0.016	0	0	T Cells
LZTS1	0	9.85082534	0.015	0	0	T Cells
B3GAT1	0	9.02230014	0.015	0	0	T Cells
HEATR9	0	9.63377307	0.015	0	0	T Cells
TSPAN18	0	11.1394256	0.015	0	0	T Cells
CCR8	0	9.17969115	0.013	0	0	T Cells
FSD11	0	10.3568377	0.012	0	0	T Cells
LETMD12	5.099E-308	0.91124737	0.153	0.484	7.837E-304	T Cells
PKN11	5.731E-308	0.77075946	0.3	0.778	8.808E-304	T Cells
MBNL31	9.599E-308	0.63430025	0.101	0.393	1.475E-303	T Cells
TPRKB1	1.046E-307	0.45647847	0.08	0.356	1.608E-303	T Cells
NOP142	1.322E-307	0.99491659	0.153	0.485	2.033E-303	T Cells
AAGAB1	1.457E-307	0.96151299	0.144	0.468	2.239E-303	T Cells
WDTC1	1.472E-307	1.03034637	0.178	0.53	2.263E-303	T Cells
MAGOH1	1.476E-307	0.85927132	0.113	0.415	2.268E-303	T Cells
MCUB1	1.66E-307	0.76709736	0.121	0.427	2.551E-303	T Cells
ZNF518A2	2.123E-307	0.64761301	0.111	0.409	3.263E-303	T Cells
CLPX2	2.545E-307	0.58280799	0.098	0.385	3.912E-303	T Cells
AGTPBP14	2.55E-307	0.97068513	0.187	0.547	3.92E-303	T Cells
AVL93	3.948E-307	0.85705165	0.133	0.45	6.068E-303	T Cells
EIF2B41	4.352E-307	0.25070037	0.07	0.336	6.689E-303	T Cells
FAM168B	4.883E-307	1.03314333	0.162	0.503	7.506E-303	T Cells
SELENOM1	5.922E-307	0.40186924	0.082	0.357	9.102E-303	T Cells
SLC25A30	6.995E-307	0.32108656	0.078	0.35	1.075E-302	T Cells
PHF232	7.72E-307	0.70557936	0.111	0.408	1.187E-302	T Cells
RBMX1	7.756E-307	0.69189526	0.109	0.404	1.192E-302	T Cells
RO60	9.247E-307	1.05529545	0.153	0.486	1.421E-302	T Cells
IL2RG2	9.804E-307	1.73946287	0.675	0.899	1.507E-302	T Cells
ZBTB16	1.049E-306	6.84972803	0.037	0.003	1.613E-302	T Cells
ZER11	1.484E-306	1.04861012	0.166	0.51	2.281E-302	T Cells
CHMP7	1.794E-306	1.07366679	0.168	0.513	2.758E-302	T Cells
MFSD61	3.002E-306	1.06467079	0.227	0.631	4.615E-302	T Cells
IFFO11	3.018E-306	0.10535696	0.069	0.335	4.64E-302	T Cells
SPTY2D11	3.355E-306	0.95148917	0.128	0.441	5.157E-302	T Cells
PPM1A2	4.13E-306	1.07494581	0.207	0.586	6.348E-302	T Cells
GGNBP21	5.143E-306	1.1149389	0.227	0.626	7.905E-302	T Cells

IL27RA2	5.267E-306	0.78260791	0.165	0.504	8.096E-302	T Cells
RPUSD42	5.426E-306	0.4581362	0.085	0.362	8.341E-302	T Cells
COPS9	8.994E-306	0.35709462	0.071	0.338	1.382E-301	T Cells
EIPR11	9.046E-306	0.51069238	0.091	0.372	1.39E-301	T Cells
EIF1AD2	1.291E-305	0.80876966	0.107	0.404	1.984E-301	T Cells
ABCB8	2.519E-305	0.4748945	0.092	0.376	3.872E-301	T Cells
NTMT12	4.11E-305	0.68559196	0.103	0.392	6.318E-301	T Cells
GTDC11	6.034E-305	0.76177512	0.115	0.415	9.275E-301	T Cells
MMUT1	8.547E-305	0.32720398	0.068	0.333	1.314E-300	T Cells
AMBRA1	1.074E-304	0.81577209	0.127	0.438	1.651E-300	T Cells
GRWD11	1.235E-304	0.54497694	0.086	0.365	1.899E-300	T Cells
FBRS1	1.346E-304	1.02213922	0.251	0.673	2.07E-300	T Cells
PRELID3B1	1.424E-304	0.59974425	0.082	0.357	2.189E-300	T Cells
NOL4L1	1.998E-304	0.74085175	0.119	0.423	3.071E-300	T Cells
RUVBL21	4.664E-304	0.12137578	0.063	0.324	7.17E-300	T Cells
FOXN33	6.109E-304	0.98599473	0.143	0.465	9.39E-300	T Cells
CIR12	9.084E-304	0.48973797	0.088	0.368	1.396E-299	T Cells
ARIH11	3.641E-303	0.97418791	0.161	0.495	5.597E-299	T Cells
PPP3CA2	6.514E-303	1.08938642	0.206	0.582	1.001E-298	T Cells
LSM2	6.651E-303	0.7830755	0.108	0.401	1.022E-298	T Cells
WDR452	8.915E-303	0.38755608	0.079	0.35	1.37E-298	T Cells
MYH9	1.183E-302	0.29660674	0.363	0.911	1.818E-298	T Cells
DNAJB62	1.37E-302	0.1381744	0.061	0.319	2.105E-298	T Cells
PRR14L2	1.765E-302	1.02950165	0.161	0.499	2.713E-298	T Cells
N4BP14	1.9E-302	0.85662428	0.28	0.728	2.921E-298	T Cells
NDC12	1.902E-302	0.32938339	0.072	0.337	2.923E-298	T Cells
ZNF3841	2.168E-302	1.06875641	0.176	0.526	3.333E-298	T Cells
FLAD12	2.591E-302	0.61841104	0.1	0.387	3.982E-298	T Cells
RAB3GAP22	4.56E-302	0.84086223	0.12	0.425	7.009E-298	T Cells
CNOT101	4.665E-302	0.51423214	0.089	0.368	7.171E-298	T Cells
HNRNPDL1	5.349E-302	1.01487178	0.261	0.691	8.222E-298	T Cells
NAPG1	6.757E-302	0.54816687	0.084	0.36	1.039E-297	T Cells
ARID5A1	7.996E-302	1.01160443	0.2	0.571	1.229E-297	T Cells
SDE22	8.013E-302	0.75131399	0.112	0.405	1.232E-297	T Cells
FAM8A12	9.395E-302	0.19661911	0.063	0.323	1.444E-297	T Cells
TAF91	1.59E-301	0.57470242	0.081	0.355	2.445E-297	T Cells
PGAM51	1.641E-301	0.50332197	0.085	0.359	2.522E-297	T Cells
SMAP12	1.946E-301	1.07752235	0.163	0.499	2.992E-297	T Cells
ZHX11	1.972E-301	0.68361412	0.095	0.377	3.031E-297	T Cells
GOLGA12	2.157E-301	0.62884165	0.096	0.38	3.315E-297	T Cells
PPME11	2.599E-301	0.25838541	0.067	0.329	3.996E-297	T Cells
PIK3CA2	3.511E-301	0.72716795	0.101	0.387	5.396E-297	T Cells
FAM111A	3.548E-301	0.88535698	0.197	0.559	5.454E-297	T Cells
MED143	6.315E-301	0.92293098	0.138	0.456	9.707E-297	T Cells
CEP3501	9.017E-301	0.94153036	0.278	0.724	1.386E-296	T Cells
ARID1A1	1.408E-300	0.97169551	0.143	0.464	2.165E-296	T Cells
GTPBP43	1.429E-300	1.00178444	0.133	0.447	2.197E-296	T Cells
EXOC42	1.436E-300	0.84056284	0.138	0.455	2.207E-296	T Cells
ADAM122	1.651E-300	7.22868468	0.023	0.001	2.538E-296	T Cells
PPP6R31	2.057E-300	1.1017005	0.181	0.531	3.161E-296	T Cells
DHX152	2.064E-300	0.94888861	0.288	0.748	3.172E-296	T Cells
AQR1	3.737E-300	0.97929146	0.134	0.449	5.744E-296	T Cells

U2AF2	3.936E-300	0.33621667	0.07	0.334	6.051E-296	T Cells
AMD12	6.63E-300	0.66055884	0.101	0.387	1.019E-295	T Cells
ATF2	8.653E-300	1.04828244	0.186	0.541	1.33E-295	T Cells
ZNF3262	1.328E-299	0.46124852	0.081	0.355	2.042E-295	T Cells
MMAB2	1.637E-299	0.17233762	0.067	0.327	2.516E-295	T Cells
UHRF1BP1L1	1.691E-299	0.45113818	0.09	0.367	2.599E-295	T Cells
GPR652	1.95E-299	1.00503218	0.198	0.563	2.997E-295	T Cells
C8orf762	4.367E-299	0.20673681	0.068	0.329	6.713E-295	T Cells
HIRA	4.453E-299	0.4515049	0.085	0.359	6.845E-295	T Cells
TACC12	6.642E-299	0.69869254	0.337	0.851	1.021E-294	T Cells
CCDC12	6.739E-299	0.95319136	0.146	0.47	1.036E-294	T Cells
MBP1	8.762E-299	0.97637047	0.188	0.546	1.347E-294	T Cells
SLC25A441	9.523E-299	0.80499138	0.113	0.407	1.464E-294	T Cells
E2F32	9.957E-299	0.30419433	0.073	0.338	1.531E-294	T Cells
SHLD12	1.237E-298	0.31851752	0.075	0.341	1.902E-294	T Cells
AKIRIN11	1.382E-298	1.15771654	0.202	0.573	2.125E-294	T Cells
DCAF101	1.615E-298	0.71653333	0.102	0.39	2.483E-294	T Cells
ARMC61	1.677E-298	0.22158009	0.068	0.332	2.578E-294	T Cells
GPR182	1.757E-298	7.15224621	0.031	0.002	2.7E-294	T Cells
SF3A2	1.758E-298	1.14247733	0.196	0.561	2.702E-294	T Cells
INTS9	3.128E-298	0.16305503	0.068	0.327	4.808E-294	T Cells
TELO2	4.115E-298	0.66571078	0.099	0.384	6.325E-294	T Cells
C5orf222	4.19E-298	0.62674854	0.094	0.373	6.44E-294	T Cells
SMARCC11	4.811E-298	1.06414025	0.16	0.495	7.394E-294	T Cells
ATG4B2	8.186E-298	0.77589524	0.117	0.416	1.258E-293	T Cells
EEF2K2	8.241E-298	0.2881089	0.075	0.341	1.267E-293	T Cells
SRRM22	8.782E-298	1.01780434	0.211	0.586	1.35E-293	T Cells
TPGS2	1.219E-297	0.74344697	0.114	0.408	1.873E-293	T Cells
YJU21	1.275E-297	0.91382026	0.13	0.438	1.96E-293	T Cells
ACIN13	1.63E-297	0.87141212	0.135	0.446	2.506E-293	T Cells
CEBPG2	2.252E-297	1.28764145	0.221	0.606	3.462E-293	T Cells
ARL6IP51	3.224E-297	0.38616296	0.357	0.895	4.956E-293	T Cells
NOM12	3.812E-297	0.67430766	0.097	0.379	5.859E-293	T Cells
PRRC2A	1.142E-296	1.12602039	0.196	0.561	1.756E-292	T Cells
TRIAP1	1.473E-296	0.34102279	0.062	0.317	2.264E-292	T Cells
MAP4K21	1.514E-296	0.79054378	0.103	0.389	2.328E-292	T Cells
REXO41	2.467E-296	0.51096467	0.082	0.352	3.792E-292	T Cells
ELP3	3.016E-296	0.98643096	0.123	0.425	4.635E-292	T Cells
RIPOR25	4.069E-296	4.64567661	0.196	0.071	6.254E-292	T Cells
BRD9	4.329E-296	0.87771795	0.129	0.436	6.654E-292	T Cells
ARF53	6.511E-296	0.56614963	0.089	0.365	1.001E-291	T Cells
NACC21	1.004E-295	0.19521088	0.066	0.324	1.544E-291	T Cells
CHD4	1.118E-295	1.06860754	0.184	0.538	1.718E-291	T Cells
SYNE34	1.137E-295	4.38208019	0.276	0.13	1.747E-291	T Cells
RPUSD3	1.325E-295	0.1347299	0.066	0.323	2.036E-291	T Cells
ARMH32	2.455E-295	0.69820869	0.106	0.397	3.774E-291	T Cells
TBC1D22A1	3.701E-295	0.61645654	0.096	0.377	5.688E-291	T Cells
KLHDC101	5.132E-295	0.62415076	0.094	0.372	7.888E-291	T Cells
PDE121	5.569E-295	0.90325139	0.114	0.41	8.56E-291	T Cells
UBE2Q22	5.611E-295	1.04676457	0.185	0.537	8.625E-291	T Cells
ARPC1A1	9.2E-295	0.50342617	0.081	0.349	1.414E-290	T Cells
INO801	1.064E-294	0.66435441	0.104	0.388	1.635E-290	T Cells

HPCAL4	1.127E-294	9.29862033	0.012	0	1.732E-290	T Cells
NSUN4	1.3E-294	0.5182922	0.077	0.344	1.998E-290	T Cells
CHFR2	1.71E-294	0.74486097	0.113	0.406	2.628E-290	T Cells
UQCRH2	2.718E-294	0.56026992	0.087	0.359	4.177E-290	T Cells
RBM142	2.921E-294	0.92533994	0.19	0.541	4.49E-290	T Cells
MOB42	3.226E-294	1.03399112	0.127	0.431	4.959E-290	T Cells
KIAA11911	6.777E-294	0.95052874	0.129	0.434	1.042E-289	T Cells
ECD2	1.431E-293	0.53031879	0.084	0.354	2.199E-289	T Cells
CPSF73	1.562E-293	1.12462136	0.228	0.622	2.402E-289	T Cells
CHD72	2.663E-293	0.60515549	0.096	0.376	4.093E-289	T Cells
PIK3C32	3.209E-293	0.67560273	0.106	0.392	4.933E-289	T Cells
CNOT62	3.861E-293	0.88251764	0.12	0.418	5.934E-289	T Cells
TMEM41B1	3.973E-293	1.13436958	0.168	0.505	6.107E-289	T Cells
SCAF41	4.037E-293	0.99304056	0.151	0.476	6.205E-289	T Cells
POGZ1	5.488E-293	1.00628008	0.164	0.494	8.435E-289	T Cells
ANP32A	7.486E-293	0.73151396	0.102	0.386	1.151E-288	T Cells
CHPF2	8.542E-293	0.92779437	0.149	0.472	1.313E-288	T Cells
RANGRF1	2.332E-292	0.72962795	0.105	0.388	3.584E-288	T Cells
POP51	2.477E-292	0.20644475	0.063	0.316	3.808E-288	T Cells
GPATCH4	2.568E-292	0.64353339	0.086	0.356	3.947E-288	T Cells
MAT2B	2.714E-292	0.86433158	0.31	0.792	4.172E-288	T Cells
ZCCHC171	3.084E-292	0.16132846	0.059	0.309	4.74E-288	T Cells
GCN1	4.176E-292	1.01057371	0.162	0.494	6.419E-288	T Cells
RGS103	4.462E-292	0.99073456	0.18	0.525	6.859E-288	T Cells
IFI44L3	5.673E-292	0.76118843	0.15	0.473	8.72E-288	T Cells
DLST	6.465E-292	0.63186291	0.092	0.368	9.937E-288	T Cells
APTX1	7.778E-292	0.3323497	0.072	0.331	1.196E-287	T Cells
ZBTB452	9.266E-292	0.36508588	0.076	0.338	1.424E-287	T Cells
PGRMC2	1.065E-291	1.13994051	0.194	0.552	1.636E-287	T Cells
ARNT3	1.107E-291	1.00686519	0.132	0.44	1.701E-287	T Cells
DCUN1D41	1.359E-291	0.58845769	0.095	0.371	2.089E-287	T Cells
PIEZO12	1.869E-291	0.94534104	0.171	0.508	2.873E-287	T Cells
PCYOX11	2.442E-291	0.11413728	0.057	0.306	3.753E-287	T Cells
AKAP102	3.724E-291	0.96260705	0.141	0.454	5.724E-287	T Cells
TMCO41	8.657E-291	0.16072102	0.067	0.322	1.331E-286	T Cells
PIKFYVE2	1.401E-290	1.0880066	0.206	0.574	2.154E-286	T Cells
PLEKHG31	2.033E-290	0.37356331	0.085	0.353	3.125E-286	T Cells
RSAD1	3.661E-290	0.8182262	0.114	0.407	5.628E-286	T Cells
HDDC3	3.909E-290	0.47506256	0.077	0.34	6.009E-286	T Cells
KANSL31	5.228E-290	0.81481475	0.111	0.4	8.036E-286	T Cells
YPEL21	5.876E-290	0.55158411	0.091	0.365	9.032E-286	T Cells
DIS31	7.856E-290	0.95285973	0.135	0.441	1.207E-285	T Cells
NR3C12	3.496E-289	0.78047098	0.333	0.837	5.373E-285	T Cells
ZXDC1	4.902E-289	0.93928146	0.162	0.492	7.534E-285	T Cells
DCP22	5.956E-289	0.97737197	0.186	0.537	9.155E-285	T Cells
BBX2	6.91E-289	0.78625702	0.103	0.385	1.062E-284	T Cells
MLYCD	8.818E-289	0.15023218	0.063	0.314	1.355E-284	T Cells
WRAP731	9.162E-289	0.40121014	0.072	0.33	1.408E-284	T Cells
DHFR1	9.696E-289	0.12709137	0.063	0.313	1.49E-284	T Cells
PNMA11	1.24E-288	1.07609276	0.202	0.568	1.906E-284	T Cells
ZADH21	1.242E-288	0.85381787	0.148	0.463	1.909E-284	T Cells
SLC25A45	1.293E-288	0.42043013	0.09	0.361	1.988E-284	T Cells

PRPSAP1	1.445E-288	0.70480744	0.09	0.362	2.221E-284	T Cells
CDC231	1.941E-288	0.57338563	0.081	0.348	2.983E-284	T Cells
C9orf78	2.015E-288	1.12611115	0.236	0.634	3.097E-284	T Cells
GTF3C11	2.266E-288	0.86578218	0.116	0.407	3.484E-284	T Cells
RGS3	3.047E-288	0.56761332	0.089	0.359	4.683E-284	T Cells
LEMD22	4.715E-288	0.97027812	0.142	0.456	7.248E-284	T Cells
LRSAM11	5.377E-288	0.54153536	0.088	0.359	8.265E-284	T Cells
KDM4A1	6.541E-288	0.8424551	0.118	0.412	1.005E-283	T Cells
NIPBL2	7.94E-288	1.11661668	0.255	0.675	1.22E-283	T Cells
SH3GL11	8.786E-288	0.47185101	0.086	0.352	1.351E-283	T Cells
RABAC11	1.009E-287	0.93927909	0.282	0.725	1.551E-283	T Cells
ZNF6872	1.019E-287	0.58232376	0.092	0.365	1.566E-283	T Cells
U2SURP	1.116E-287	1.15197549	0.232	0.622	1.715E-283	T Cells
BCAT21	1.302E-287	0.13563037	0.07	0.324	2.001E-283	T Cells
HIP1R	1.6E-287	6.6934502	0.028	0.002	2.459E-283	T Cells
NAA151	2.192E-287	1.17050341	0.181	0.524	3.37E-283	T Cells
USP43	2.683E-287	1.01508904	0.18	0.526	4.124E-283	T Cells
ACBD62	3.159E-287	0.82094447	0.106	0.391	4.856E-283	T Cells
CYTH44	4.002E-287	0.96395519	0.208	0.573	6.151E-283	T Cells
DDX39A2	4.147E-287	0.96154479	0.141	0.452	6.374E-283	T Cells
TPR	4.623E-287	1.1653367	0.202	0.567	7.106E-283	T Cells
KLRG1	6.375E-287	6.38344869	0.068	0.011	9.799E-283	T Cells
SON	2.058E-286	1.07968137	0.185	0.535	3.163E-282	T Cells
LPIN21	2.084E-286	1.08229612	0.198	0.558	3.204E-282	T Cells
STYXL12	2.144E-286	0.27716118	0.065	0.317	3.295E-282	T Cells
C20orf1941	2.776E-286	0.40515166	0.077	0.338	4.267E-282	T Cells
ABL12	3.016E-286	0.64874905	0.09	0.36	4.637E-282	T Cells
TMEM106C	4.503E-286	0.4900825	0.095	0.369	6.922E-282	T Cells
MTF11	4.607E-286	0.53216614	0.078	0.34	7.082E-282	T Cells
ABCE11	4.816E-286	0.80606966	0.102	0.382	7.403E-282	T Cells
CD47	7.058E-286	0.50421891	0.359	0.883	1.085E-281	T Cells
SMC2	7.596E-286	0.13735219	0.062	0.312	1.168E-281	T Cells
BTBD71	8.364E-286	1.01192052	0.161	0.486	1.286E-281	T Cells
MTSS13	8.678E-286	5.39306764	0.043	0.004	1.334E-281	T Cells
PIK3CD5	9.563E-286	3.33778813	0.532	0.491	1.47E-281	T Cells
ITPR3	9.893E-286	4.832338	0.179	0.062	1.521E-281	T Cells
CRIPT	3.013E-285	0.92895442	0.123	0.418	4.632E-281	T Cells
SMPD42	3.422E-285	0.75411872	0.114	0.402	5.259E-281	T Cells
ZC3H14	3.522E-285	0.49323348	0.078	0.337	5.414E-281	T Cells
DPF21	3.826E-285	0.98469813	0.137	0.441	5.881E-281	T Cells
CPT1A3	5.094E-285	0.88261991	0.122	0.416	7.829E-281	T Cells
TRAPPC2L	5.608E-285	0.43891558	0.073	0.33	8.62E-281	T Cells
PDE3B1	7.008E-285	1.20788322	0.162	0.492	1.077E-280	T Cells
SDR39U1	8.931E-285	0.98616542	0.136	0.441	1.373E-280	T Cells
CTCF2	1.056E-284	1.08659818	0.179	0.518	1.623E-280	T Cells
COQ41	1.156E-284	0.87956854	0.125	0.422	1.777E-280	T Cells
L3MBTL2	1.322E-284	0.70347637	0.095	0.369	2.032E-280	T Cells
DRG21	1.506E-284	0.46328839	0.079	0.34	2.315E-280	T Cells
PSMG11	1.517E-284	0.3420123	0.07	0.324	2.332E-280	T Cells
GALT	1.674E-284	0.83374662	0.106	0.39	2.574E-280	T Cells
AKNA4	2.928E-284	3.28150741	0.556	0.553	4.501E-280	T Cells
DHRS14	2.93E-284	0.16289072	0.069	0.322	4.504E-280	T Cells

EPS15L12	3.127E-284	0.83007391	0.108	0.391	4.806E-280	T Cells
CPTP1	3.549E-284	0.5083934	0.078	0.339	5.455E-280	T Cells
PHF22	4.569E-284	1.08017825	0.157	0.478	7.023E-280	T Cells
BRD41	4.943E-284	1.00029907	0.293	0.75	7.597E-280	T Cells
MAP2K21	6.205E-284	0.98792995	0.299	0.762	9.537E-280	T Cells
KDM4B2	6.808E-284	0.91901993	0.149	0.464	1.046E-279	T Cells
ZNF5811	8.255E-284	0.4957668	0.082	0.346	1.269E-279	T Cells
RXYLT12	9.469E-284	0.20159946	0.062	0.309	1.456E-279	T Cells
OSGEP1	9.769E-284	0.33511905	0.075	0.331	1.502E-279	T Cells
GABPA	1.304E-283	0.66517834	0.087	0.356	2.004E-279	T Cells
PPP3CB1	1.713E-283	1.11428293	0.218	0.592	2.633E-279	T Cells
WDR77	1.722E-283	0.15634547	0.064	0.313	2.646E-279	T Cells
UBE3B	1.897E-283	0.49378268	0.075	0.332	2.916E-279	T Cells
AACS1	2.174E-283	0.44899026	0.075	0.332	3.342E-279	T Cells
TMEM1591	2.272E-283	0.33062447	0.069	0.321	3.493E-279	T Cells
GFPT11	3.109E-283	0.48866418	0.075	0.332	4.779E-279	T Cells
CTDSP12	3.949E-283	0.7292105	0.315	0.786	6.07E-279	T Cells
SLF22	4.343E-283	0.89323885	0.131	0.431	6.676E-279	T Cells
B3GAT3	6.711E-283	0.76751962	0.103	0.381	1.032E-278	T Cells
IPO93	7.575E-283	1.00612212	0.132	0.434	1.164E-278	T Cells
HDAC1	9.151E-283	1.14678852	0.221	0.603	1.407E-278	T Cells
EPS15	2.044E-282	1.18986158	0.227	0.611	3.142E-278	T Cells
PSME21	2.291E-282	0.10964497	0.067	0.317	3.522E-278	T Cells
ZNF2681	2.795E-282	0.50471958	0.076	0.334	4.297E-278	T Cells
UCHL31	2.845E-282	0.54585054	0.07	0.325	4.373E-278	T Cells
MAK161	3.224E-282	0.63249259	0.085	0.347	4.955E-278	T Cells
VEZF12	3.243E-282	1.08694301	0.149	0.466	4.985E-278	T Cells
SS182	3.367E-282	1.09872536	0.162	0.488	5.176E-278	T Cells
STAG23	3.41E-282	1.10705982	0.234	0.623	5.241E-278	T Cells
AHCYL2	6.441E-282	0.44661853	0.079	0.339	9.901E-278	T Cells
HMGXB3	1.189E-281	0.2702177	0.063	0.311	1.828E-277	T Cells
SNRNP271	1.51E-281	0.57666668	0.082	0.342	2.321E-277	T Cells
ARL152	1.538E-281	0.36501484	0.07	0.324	2.363E-277	T Cells
FBXL61	1.64E-281	0.33929115	0.074	0.33	2.521E-277	T Cells
MSMO11	1.908E-281	0.19876512	0.109	0.388	2.933E-277	T Cells
CDK5RAP2	1.944E-281	1.03654349	0.143	0.451	2.988E-277	T Cells
ATAD11	3.808E-281	1.08062	0.151	0.465	5.853E-277	T Cells
SLC33A12	3.822E-281	0.97331694	0.127	0.423	5.875E-277	T Cells
SARNP1	3.837E-281	0.61306649	0.086	0.349	5.899E-277	T Cells
ASNSD11	4.619E-281	0.98334465	0.116	0.405	7.1E-277	T Cells
MGME12	5.233E-281	0.74677952	0.096	0.37	8.044E-277	T Cells
INO80B1	7.169E-281	0.90352704	0.129	0.425	1.102E-276	T Cells
SNHG321	8.792E-281	1.18542072	0.183	0.523	1.351E-276	T Cells
TLE5	9.124E-281	0.43124848	0.363	0.896	1.402E-276	T Cells
RAB11FIP41	1.658E-280	0.70727522	0.157	0.473	2.549E-276	T Cells
SLC2A33	2.148E-280	0.42049206	0.299	0.739	3.302E-276	T Cells
TMEM691	2.443E-280	0.32446672	0.064	0.313	3.755E-276	T Cells
NDUFAF2	2.994E-280	0.57720876	0.079	0.338	4.602E-276	T Cells
LUC7L21	3.957E-280	1.04270837	0.295	0.752	6.083E-276	T Cells
NUP421	4.423E-280	0.91423911	0.119	0.409	6.799E-276	T Cells
UBE4B3	5.519E-280	1.00525265	0.15	0.462	8.483E-276	T Cells
SLC35E11	6.014E-280	1.03821097	0.151	0.467	9.244E-276	T Cells

SERPINB95	8.63E-280	4.06099914	0.188	0.069	1.327E-275	T Cells
MXD41	9.104E-280	1.06757724	0.212	0.581	1.399E-275	T Cells
VRK32	9.779E-280	0.67221407	0.086	0.35	1.503E-275	T Cells
DMAC2L1	1.166E-279	0.46002099	0.072	0.327	1.792E-275	T Cells
RAB33A2	1.187E-279	6.76415501	0.035	0.003	1.825E-275	T Cells
IGFLR13	1.408E-279	0.90936326	0.168	0.497	2.165E-275	T Cells
DAXX	1.596E-279	1.19029656	0.23	0.616	2.453E-275	T Cells
ERF1	3.579E-279	0.43507966	0.081	0.341	5.502E-275	T Cells
SETDB23	4.101E-279	0.8069796	0.134	0.433	6.303E-275	T Cells
USP382	4.616E-279	0.77488818	0.106	0.384	7.095E-275	T Cells
MLLT63	4.898E-279	3.4862126	0.505	0.44	7.529E-275	T Cells
CUL4B1	5.235E-279	0.99972084	0.125	0.421	8.046E-275	T Cells
MRRF3	7.051E-279	0.15326743	0.069	0.317	1.084E-274	T Cells
PDCL31	1.048E-278	1.00112083	0.128	0.424	1.611E-274	T Cells
EAF1	1.136E-278	1.06914921	0.146	0.455	1.746E-274	T Cells
PMF1	1.529E-278	1.02629367	0.157	0.475	2.35E-274	T Cells
RBPJ3	2.049E-278	1.05701433	0.288	0.734	3.15E-274	T Cells
RPF11	2.081E-278	0.70265923	0.088	0.354	3.199E-274	T Cells
ELP6	3.777E-278	0.40350232	0.071	0.323	5.806E-274	T Cells
TBC1D232	4.272E-278	0.47838577	0.072	0.323	6.566E-274	T Cells
C12orf10	4.761E-278	0.11759086	0.068	0.314	7.319E-274	T Cells
SRSF51	5.157E-278	1.09140988	0.26	0.675	7.927E-274	T Cells
PRKRA1	5.992E-278	0.91930998	0.114	0.398	9.211E-274	T Cells
SNRPD2	6.044E-278	1.15253824	0.172	0.502	9.289E-274	T Cells
NOC4L4	6.795E-278	0.23173659	0.068	0.315	1.045E-273	T Cells
RIOK12	7.825E-278	0.80063669	0.106	0.383	1.203E-273	T Cells
GCC22	9.093E-278	1.07684951	0.157	0.475	1.398E-273	T Cells
NKAPD12	1.286E-277	0.5600742	0.084	0.344	1.977E-273	T Cells
QRICH1	1.984E-277	1.02143959	0.299	0.755	3.05E-273	T Cells
PTPN112	2.023E-277	0.29355395	0.064	0.311	3.11E-273	T Cells
GTF2F22	2.602E-277	0.52522704	0.071	0.324	4E-273	T Cells
WASL	3.099E-277	0.40467164	0.072	0.324	4.764E-273	T Cells
COA81	4.118E-277	0.74177604	0.084	0.346	6.33E-273	T Cells
KLHL361	4.709E-277	1.02723316	0.157	0.476	7.238E-273	T Cells
RBBP91	7.013E-277	0.10903704	0.055	0.293	1.078E-272	T Cells
NR1D22	7.152E-277	0.97487928	0.215	0.583	1.099E-272	T Cells
RNF1661	7.311E-277	0.95519094	0.144	0.45	1.124E-272	T Cells
MADD2	9.764E-277	0.96177141	0.144	0.452	1.501E-272	T Cells
ZNF761	1.645E-276	0.5001076	0.078	0.336	2.529E-272	T Cells
TASOR1	5.023E-276	1.09333242	0.274	0.703	7.72E-272	T Cells
TSC2	7.916E-276	0.63379947	0.089	0.352	1.217E-271	T Cells
PCBP13	1.01E-275	0.85015241	0.306	0.771	1.553E-271	T Cells
RNF38	1.051E-275	1.15949651	0.249	0.652	1.616E-271	T Cells
MOB3C2	1.369E-275	0.11607277	0.067	0.313	2.104E-271	T Cells
NUP371	1.665E-275	0.16784669	0.058	0.299	2.559E-271	T Cells
TERF21	2.124E-275	1.17850825	0.157	0.477	3.265E-271	T Cells
IL12RB1	4.017E-275	1.04212376	0.147	0.455	6.174E-271	T Cells
MAPK113	4.328E-275	6.29627141	0.04	0.004	6.653E-271	T Cells
DDX19B1	6.069E-275	0.30541211	0.068	0.314	9.328E-271	T Cells
FAM13B2	9.908E-275	1.05935387	0.151	0.466	1.523E-270	T Cells
CABIN1	1.261E-274	0.88295295	0.135	0.433	1.938E-270	T Cells
HEBP2	1.546E-274	1.11917579	0.237	0.625	2.376E-270	T Cells

TRIM281	2.019E-274	0.24621711	0.065	0.308	3.104E-270	T Cells
CRTC32	2.17E-274	0.89200937	0.107	0.383	3.335E-270	T Cells
ZNF6091	2.452E-274	1.18162191	0.211	0.574	3.768E-270	T Cells
XIAP2	2.94E-274	0.91398579	0.115	0.398	4.519E-270	T Cells
DBP	4.675E-274	0.56906768	0.094	0.362	7.186E-270	T Cells
TTC34	5.69E-274	1.2587874	0.214	0.581	8.746E-270	T Cells
HMGN41	6.158E-274	1.0658159	0.288	0.732	9.465E-270	T Cells
IPO111	7.554E-274	0.1149613	0.053	0.289	1.161E-269	T Cells
ORC41	7.722E-274	0.38684208	0.064	0.309	1.187E-269	T Cells
SEC61G	1.062E-273	0.487996	0.072	0.323	1.633E-269	T Cells
LYSMD32	1.069E-273	0.91323254	0.107	0.384	1.643E-269	T Cells
TFAM1	1.288E-273	0.64847673	0.085	0.343	1.979E-269	T Cells
CDC73	1.682E-273	1.19254888	0.255	0.662	2.585E-269	T Cells
RNF22	3.138E-273	0.24547115	0.058	0.295	4.824E-269	T Cells
FAM120B1	4.035E-273	0.67051535	0.099	0.368	6.203E-269	T Cells
DDX20	4.323E-273	0.46686817	0.068	0.315	6.645E-269	T Cells
UBE2K1	5.539E-273	0.55344932	0.081	0.336	8.514E-269	T Cells
LIMD14	9.339E-273	1.00236948	0.15	0.459	1.436E-268	T Cells
GALK2	1.161E-272	0.8336217	0.108	0.384	1.784E-268	T Cells
PPP1R15B4	2.101E-272	0.7407475	0.34	0.842	3.23E-268	T Cells
MTREX2	2.761E-272	0.78021576	0.105	0.378	4.244E-268	T Cells
AKAP11	3.044E-272	1.17502845	0.169	0.496	4.679E-268	T Cells
PIGV1	3.092E-272	0.15815667	0.065	0.307	4.752E-268	T Cells
SPINDOC	3.302E-272	0.28222773	0.076	0.326	5.075E-268	T Cells
PHF112	3.77E-272	1.18569985	0.213	0.577	5.794E-268	T Cells
OTUD51	3.98E-272	1.1307009	0.206	0.562	6.118E-268	T Cells
TRNAU1AP1	4.263E-272	1.08262616	0.143	0.448	6.552E-268	T Cells
HGH11	6.806E-272	0.32099731	0.065	0.31	1.046E-267	T Cells
HSPB112	7.569E-272	0.91041044	0.108	0.384	1.163E-267	T Cells
FOXK2	9.726E-272	0.91270295	0.125	0.414	1.495E-267	T Cells
CSK1	1.46E-271	0.44206192	0.365	0.901	2.244E-267	T Cells
KRR11	1.58E-271	1.04639852	0.129	0.421	2.429E-267	T Cells
IGSF9B	2.881E-271	10.0345813	0.011	0	4.428E-267	T Cells
HIF1AN1	5.257E-271	1.01977876	0.113	0.396	8.08E-267	T Cells
FOPNL2	6.227E-271	1.0292845	0.124	0.412	9.572E-267	T Cells
SF12	7.976E-271	1.00645358	0.129	0.42	1.226E-266	T Cells
ITPR11	8.829E-271	0.99078996	0.18	0.513	1.357E-266	T Cells
ANAPC2	1.298E-270	0.50130987	0.078	0.331	1.996E-266	T Cells
TAF82	1.371E-270	1.00194803	0.139	0.437	2.108E-266	T Cells
CWC271	1.564E-270	0.68664856	0.096	0.36	2.404E-266	T Cells
PPP2R2A	1.789E-270	1.01760412	0.122	0.411	2.75E-266	T Cells
ZRANB11	1.949E-270	0.92596998	0.135	0.432	2.996E-266	T Cells
ZNF5112	3.719E-270	0.15181538	0.06	0.298	5.717E-266	T Cells
KIR2DL4	5.358E-270	10.7514491	0.01	0	8.235E-266	T Cells
HTATSF1	5.794E-270	1.01945003	0.133	0.426	8.907E-266	T Cells
PAFAH1B12	7.813E-270	0.79435756	0.344	0.85	1.201E-265	T Cells
BLOC1S31	8.486E-270	0.15008037	0.069	0.314	1.304E-265	T Cells
COG21	9.546E-270	0.39854639	0.071	0.318	1.467E-265	T Cells
TMEM1382	2.874E-269	0.74091502	0.088	0.349	4.417E-265	T Cells
RALGAPB1	3.32E-269	1.1958998	0.183	0.52	5.103E-265	T Cells
UBA22	3.424E-269	1.17285076	0.166	0.487	5.263E-265	T Cells
GTF3C31	4.144E-269	0.99386837	0.124	0.409	6.369E-265	T Cells

SLC25A162	4.467E-269	0.67055117	0.098	0.362	6.867E-265	T Cells
MED101	6.033E-269	1.18619488	0.181	0.518	9.274E-265	T Cells
DNAJC211	6.62E-269	1.22214762	0.202	0.555	1.018E-264	T Cells
GTF3A2	6.989E-269	1.23152951	0.171	0.494	1.074E-264	T Cells
FBXO2	7.528E-269	7.90816072	0.017	0.001	1.157E-264	T Cells
ZNRD1	7.536E-269	0.86316798	0.106	0.379	1.158E-264	T Cells
ZC3H32	7.693E-269	0.57690331	0.083	0.339	1.183E-264	T Cells
TMEM2562	1.013E-268	0.14426184	0.057	0.292	1.556E-264	T Cells
FMC11	1.081E-268	0.49481479	0.08	0.332	1.661E-264	T Cells
LPCAT11	1.276E-268	0.96639752	0.15	0.458	1.961E-264	T Cells
SLC30A52	1.572E-268	0.67891405	0.083	0.338	2.416E-264	T Cells
KIAA18411	2.097E-268	0.17793108	0.057	0.294	3.224E-264	T Cells
CINP1	2.172E-268	0.34373326	0.057	0.294	3.339E-264	T Cells
DAZAP11	3.871E-268	1.00853663	0.143	0.445	5.95E-264	T Cells
USP392	4.687E-268	0.59608346	0.081	0.334	7.204E-264	T Cells
BIRC21	4.985E-268	0.95138791	0.122	0.407	7.662E-264	T Cells
RBFA1	5.803E-268	0.31100437	0.058	0.295	8.919E-264	T Cells
TARBP11	8.254E-268	0.59680198	0.092	0.354	1.269E-263	T Cells
UQCC11	1.089E-267	0.56803368	0.088	0.344	1.674E-263	T Cells
BUD132	1.431E-267	0.7394092	0.085	0.342	2.199E-263	T Cells
MCAM	1.531E-267	9.4728177	0.011	0	2.354E-263	T Cells
LLGL1	1.64E-267	0.74482686	0.111	0.386	2.521E-263	T Cells
RRP7A1	4.31E-267	0.22584724	0.067	0.308	6.624E-263	T Cells
TAZ1	5.003E-267	0.77281966	0.11	0.385	7.69E-263	T Cells
DHX371	7.977E-267	0.42978345	0.067	0.309	1.226E-262	T Cells
GPN11	9.857E-267	0.65547249	0.087	0.345	1.515E-262	T Cells
TAF151	1.413E-266	0.83937978	0.117	0.394	2.173E-262	T Cells
ERAP2	1.56E-266	1.03653741	0.15	0.456	2.398E-262	T Cells
MTPAP1	2.106E-266	0.85072334	0.11	0.383	3.236E-262	T Cells
IGHM1	2.238E-266	7.28995106	0.014	0	3.441E-262	T Cells
VPS13D1	2.414E-266	0.83010195	0.1	0.369	3.711E-262	T Cells
KMT5A	3.222E-266	0.94496302	0.122	0.406	4.953E-262	T Cells
SSH22	3.767E-266	0.7790187	0.117	0.397	5.79E-262	T Cells
SLC35C12	4.32E-266	0.67227232	0.09	0.348	6.64E-262	T Cells
ZBTB38	6.81E-266	1.23003217	0.152	0.461	1.047E-261	T Cells
PIAS41	1.047E-265	0.79040391	0.098	0.363	1.609E-261	T Cells
PTPN24	1.453E-265	0.86637036	0.118	0.397	2.233E-261	T Cells
TOM1L2	1.504E-265	0.10956659	0.056	0.288	2.312E-261	T Cells
NUP541	1.745E-265	0.98353571	0.11	0.386	2.683E-261	T Cells
TBC1D71	2.001E-265	0.22648943	0.06	0.295	3.076E-261	T Cells
SPG74	2.75E-265	1.06101277	0.171	0.491	4.227E-261	T Cells
AIDA1	4.718E-265	0.7845432	0.089	0.346	7.252E-261	T Cells
ZNF1752	4.767E-265	0.20980865	0.066	0.305	7.328E-261	T Cells
SAP1301	4.856E-265	0.97243261	0.114	0.389	7.465E-261	T Cells
ARFGEF13	6.263E-265	0.86304489	0.107	0.378	9.626E-261	T Cells
SNRPA	6.89E-265	1.154835	0.196	0.54	1.059E-260	T Cells
HPS32	7.452E-265	1.04697631	0.129	0.42	1.145E-260	T Cells
TTLL51	1.031E-264	0.80872379	0.125	0.407	1.585E-260	T Cells
PSKH11	1.52E-264	0.69206294	0.095	0.356	2.336E-260	T Cells
INTS111	2.885E-264	1.18972196	0.22	0.587	4.435E-260	T Cells
PURA1	3.097E-264	1.17170817	0.177	0.503	4.761E-260	T Cells
ZBTB185	4.737E-264	0.48515039	0.067	0.308	7.281E-260	T Cells

EIF4B	6.655E-264	1.01414052	0.292	0.735	1.023E-259	T Cells
BABAM21	7.362E-264	0.61314381	0.079	0.329	1.132E-259	T Cells
RNF311	8.616E-264	0.82277541	0.126	0.409	1.324E-259	T Cells
METTL14	1.1E-263	0.79264703	0.096	0.355	1.692E-259	T Cells
SHFL2	1.221E-263	1.13024187	0.164	0.48	1.876E-259	T Cells
GPATCH81	1.532E-263	1.07186926	0.124	0.408	2.355E-259	T Cells
TPP22	1.611E-263	1.13218554	0.153	0.46	2.476E-259	T Cells
MCM3	1.622E-263	0.55919991	0.098	0.36	2.493E-259	T Cells
KIT2	1.696E-263	9.09989297	0.013	0	2.608E-259	T Cells
GATC1	2.356E-263	0.60728639	0.073	0.319	3.622E-259	T Cells
ZMYM61	2.668E-263	0.65101274	0.085	0.339	4.101E-259	T Cells
MINDY31	3.883E-263	0.73133921	0.097	0.359	5.969E-259	T Cells
MAD1L12	5.351E-263	1.02379593	0.123	0.402	8.224E-259	T Cells
NAT102	8.155E-263	0.59648862	0.079	0.329	1.253E-258	T Cells
ELMOD21	8.575E-263	0.66950116	0.083	0.335	1.318E-258	T Cells
FBXW111	1.045E-262	0.71029221	0.077	0.324	1.606E-258	T Cells
MAPRE2	1.098E-262	1.23877462	0.22	0.584	1.688E-258	T Cells
APPBP22	1.26E-262	0.37308579	0.07	0.311	1.936E-258	T Cells
TET24	1.432E-262	0.37728025	0.07	0.312	2.201E-258	T Cells
PRKD32	2.226E-262	0.84475499	0.099	0.363	3.422E-258	T Cells
CCNL12	2.407E-262	0.9715806	0.205	0.551	3.699E-258	T Cells
ITFG21	3.312E-262	1.09905124	0.148	0.451	5.091E-258	T Cells
STK17B3	3.646E-262	2.4397847	0.632	0.81	5.605E-258	T Cells
SDHA2	3.67E-262	0.9223441	0.114	0.389	5.641E-258	T Cells
CAPZB2	7.574E-262	0.40742735	0.374	0.909	1.164E-257	T Cells
CCNDBP1	1.006E-261	0.98116776	0.307	0.766	1.546E-257	T Cells
RNF1392	1.018E-261	0.90228564	0.123	0.401	1.564E-257	T Cells
UAP1L12	1.107E-261	0.3710294	0.063	0.3	1.701E-257	T Cells
CYHR12	1.383E-261	0.3221841	0.065	0.301	2.126E-257	T Cells
ZNF2173	1.42E-261	0.99756143	0.3	0.746	2.183E-257	T Cells
AP5Z12	1.431E-261	0.6828066	0.094	0.354	2.2E-257	T Cells
HDAC81	2.199E-261	0.49485523	0.081	0.33	3.381E-257	T Cells
PGAP21	3.526E-261	0.29780306	0.066	0.304	5.42E-257	T Cells
BET12	3.636E-261	0.27508852	0.061	0.295	5.589E-257	T Cells
CDC26	5.1E-261	0.70383881	0.086	0.337	7.84E-257	T Cells
RPRD1B1	5.938E-261	0.49364481	0.06	0.295	9.127E-257	T Cells
ASB82	8.415E-261	0.66045416	0.086	0.339	1.294E-256	T Cells
GTF2H4	1.079E-260	0.37716545	0.062	0.298	1.659E-256	T Cells
LRRC281	1.135E-260	0.17794781	0.057	0.286	1.744E-256	T Cells
RASAL32	1.393E-260	4.16163918	0.349	0.213	2.141E-256	T Cells
SPATA13	1.575E-260	1.04160075	0.228	0.598	2.421E-256	T Cells
FBH12	2.828E-260	1.0705312	0.131	0.419	4.348E-256	T Cells
GOLPH3	3.396E-260	0.87300296	0.335	0.824	5.219E-256	T Cells
NSUN32	3.53E-260	0.60150858	0.075	0.319	5.426E-256	T Cells
C17orf493	5.787E-260	0.83257617	0.115	0.389	8.895E-256	T Cells
BORCS61	1.328E-259	0.8232592	0.097	0.357	2.041E-255	T Cells
CFAP36	1.581E-259	0.71545159	0.085	0.336	2.431E-255	T Cells
DNAJB91	1.86E-259	1.2011178	0.152	0.456	2.859E-255	T Cells
MAPK31	2.424E-259	1.00171474	0.123	0.401	3.726E-255	T Cells
TTC172	2.476E-259	1.10747682	0.157	0.463	3.806E-255	T Cells
LCORL2	2.507E-259	0.54923408	0.077	0.322	3.854E-255	T Cells
CDK5RAP11	2.682E-259	0.62315998	0.075	0.319	4.122E-255	T Cells

GNPTAB2	2.771E-259	1.30725209	0.245	0.631	4.259E-255	T Cells
MIEF11	3.09E-259	0.39512876	0.068	0.306	4.75E-255	T Cells
BTBD6	3.243E-259	1.04933262	0.132	0.423	4.985E-255	T Cells
DNAJB15	5.119E-259	1.19868409	0.2	0.542	7.869E-255	T Cells
SRRD	5.878E-259	0.25954744	0.054	0.283	9.035E-255	T Cells
JAGN11	8.796E-259	0.19085193	0.062	0.295	1.352E-254	T Cells
SFPQ1	8.818E-259	0.60338605	0.086	0.338	1.355E-254	T Cells
PPP2R2D1	8.88E-259	1.09926724	0.155	0.46	1.365E-254	T Cells
C8orf33	1.866E-258	0.38951426	0.066	0.303	2.868E-254	T Cells
PPP1R81	2.087E-258	0.92687705	0.11	0.379	3.208E-254	T Cells
LANCL11	2.606E-258	1.05435034	0.133	0.421	4.006E-254	T Cells
DTD11	2.606E-258	0.94062011	0.107	0.374	4.006E-254	T Cells
RTCA1	3.308E-258	1.16141308	0.15	0.454	5.085E-254	T Cells
DUS3L1	3.36E-258	0.60253494	0.083	0.331	5.164E-254	T Cells
ATF1	6.394E-258	0.85093086	0.088	0.342	9.828E-254	T Cells
TMEM1232	7.771E-258	1.23326978	0.222	0.586	1.194E-253	T Cells
KIAA11432	9.342E-258	0.93902243	0.098	0.359	1.436E-253	T Cells
GOLGA22	1.252E-257	0.68288579	0.085	0.333	1.924E-253	T Cells
RPTOR1	1.471E-257	0.90559283	0.114	0.386	2.261E-253	T Cells
OSBPL83	1.575E-257	1.14943303	0.277	0.692	2.421E-253	T Cells
PET1171	1.661E-257	0.55209478	0.07	0.308	2.553E-253	T Cells
BDH21	1.701E-257	0.45201163	0.062	0.294	2.615E-253	T Cells
MYADM3	1.812E-257	0.18996082	0.392	0.891	2.785E-253	T Cells
DENND102	1.826E-257	0.93313201	0.105	0.37	2.807E-253	T Cells
TBC1D132	2.022E-257	0.50119403	0.073	0.313	3.107E-253	T Cells
NCOA61	2.73E-257	1.2090372	0.148	0.448	4.196E-253	T Cells
USP122	3.175E-257	1.00601971	0.132	0.418	4.88E-253	T Cells
MTM11	3.654E-257	0.94481599	0.103	0.368	5.616E-253	T Cells
ACAT21	4.144E-257	0.38129698	0.105	0.369	6.37E-253	T Cells
STAU21	4.419E-257	1.11902066	0.28	0.703	6.793E-253	T Cells
BRI3BP	6.396E-257	0.34065706	0.067	0.303	9.831E-253	T Cells
XPA2	7.456E-257	0.76268905	0.092	0.346	1.146E-252	T Cells
DCUN1D51	9.341E-257	0.6480823	0.068	0.306	1.436E-252	T Cells
TTF21	9.869E-257	0.43773861	0.063	0.297	1.517E-252	T Cells
EIF2B51	1.193E-256	1.00003737	0.121	0.397	1.834E-252	T Cells
CEBPZ2	1.688E-256	1.26636806	0.184	0.514	2.594E-252	T Cells
CREB12	2.141E-256	1.05987753	0.153	0.454	3.291E-252	T Cells
NXF13	2.215E-256	0.97644606	0.172	0.487	3.405E-252	T Cells
HBS1L	2.976E-256	1.12527629	0.279	0.697	4.575E-252	T Cells
OLFM23	3.12E-256	8.02523163	0.012	0	4.795E-252	T Cells
PIK3R53	3.719E-256	0.81583633	0.321	0.79	5.717E-252	T Cells
WBP111	4.31E-256	0.60911757	0.075	0.316	6.625E-252	T Cells
SLC5A62	5.02E-256	0.35055846	0.069	0.306	7.716E-252	T Cells
ZNF5623	6.145E-256	0.66992782	0.082	0.33	9.445E-252	T Cells
SMURF12	6.627E-256	0.29828966	0.063	0.295	1.019E-251	T Cells
MED72	8.994E-256	0.71531291	0.084	0.331	1.382E-251	T Cells
PPP6R21	1.069E-255	1.19016552	0.241	0.619	1.643E-251	T Cells
CUTC1	1.222E-255	0.79437017	0.091	0.345	1.879E-251	T Cells
DYNLL2	1.249E-255	1.02560369	0.301	0.746	1.919E-251	T Cells
SLC35B31	1.821E-255	0.16801909	0.049	0.272	2.8E-251	T Cells
EIF4A2	2.012E-255	1.03910075	0.303	0.751	3.093E-251	T Cells
LONRF12	2.413E-255	0.30409984	0.063	0.295	3.709E-251	T Cells

TP53BP1	2.874E-255	0.60164662	0.086	0.333	4.417E-251	T Cells
NOB1	4.98E-255	0.6614778	0.079	0.325	7.655E-251	T Cells
HMG20A1	5.543E-255	0.82731366	0.09	0.34	8.521E-251	T Cells
CCDC431	5.735E-255	0.41231559	0.062	0.293	8.815E-251	T Cells
CNNM41	5.953E-255	0.12399859	0.055	0.279	9.15E-251	T Cells
ATXN10	7.293E-255	0.95610169	0.112	0.381	1.121E-250	T Cells
ZC3H182	7.608E-255	1.24247312	0.202	0.547	1.169E-250	T Cells
MFSD14A1	1.58E-254	1.20804981	0.158	0.461	2.429E-250	T Cells
NSMCE4A2	2.018E-254	0.92238496	0.112	0.379	3.102E-250	T Cells
EEF1B22	2.069E-254	1.21716962	0.249	0.638	3.18E-250	T Cells
STRADA1	2.165E-254	0.18155092	0.057	0.284	3.327E-250	T Cells
SSBP11	2.856E-254	0.5409787	0.072	0.309	4.39E-250	T Cells
PRPS11	3.204E-254	1.02459481	0.115	0.386	4.924E-250	T Cells
C5orf511	3.876E-254	0.61351112	0.069	0.306	5.957E-250	T Cells
PDK31	3.96E-254	0.47349824	0.067	0.3	6.087E-250	T Cells
FOXJ21	4.017E-254	0.8102628	0.093	0.345	6.174E-250	T Cells
URI12	4.89E-254	1.30406423	0.238	0.617	7.516E-250	T Cells
UBAC22	4.917E-254	0.98565071	0.107	0.372	7.558E-250	T Cells
STK38L5	5.522E-254	0.88268986	0.108	0.372	8.487E-250	T Cells
RBM391	5.91E-254	1.17411945	0.175	0.493	9.085E-250	T Cells
BAZ2A1	6.364E-254	1.07761686	0.156	0.459	9.782E-250	T Cells
PPP1R211	1.044E-253	1.06093383	0.128	0.41	1.604E-249	T Cells
ARFIP23	1.158E-253	0.42003243	0.066	0.299	1.781E-249	T Cells
UBA51	1.73E-253	0.6637032	0.079	0.324	2.66E-249	T Cells
PPP1R3D	2.708E-253	0.26087293	0.062	0.291	4.163E-249	T Cells
BAZ1B	2.968E-253	1.20647875	0.223	0.581	4.562E-249	T Cells
LNPEP	3.342E-253	0.95665512	0.318	0.783	5.136E-249	T Cells
PIM34	4.013E-253	0.87154845	0.115	0.388	6.168E-249	T Cells
REV11	7.801E-253	1.09380295	0.147	0.442	1.199E-248	T Cells
SUPT20H1	2.694E-252	1.17548828	0.165	0.475	4.141E-248	T Cells
C1orf522	3.962E-252	0.87754807	0.106	0.368	6.089E-248	T Cells
CTDSP23	4.258E-252	0.97719612	0.308	0.762	6.545E-248	T Cells
LEO1	4.547E-252	0.81502275	0.088	0.339	6.988E-248	T Cells
TGS11	6.025E-252	1.0163466	0.113	0.383	9.261E-248	T Cells
NOL82	6.577E-252	0.8500407	0.097	0.352	1.011E-247	T Cells
R3HCC1L1	6.939E-252	0.48834077	0.072	0.311	1.067E-247	T Cells
C6orf47	9.984E-252	0.44528829	0.067	0.299	1.535E-247	T Cells
DEK1	1.041E-251	1.18447702	0.196	0.529	1.6E-247	T Cells
VAPB1	1.09E-251	0.75974464	0.087	0.335	1.675E-247	T Cells
RBM102	1.442E-251	1.09649181	0.15	0.446	2.216E-247	T Cells
TMEM184C2	1.534E-251	0.26752169	0.06	0.287	2.358E-247	T Cells
ZNF5611	1.738E-251	0.85022279	0.095	0.348	2.672E-247	T Cells
ORMDL11	1.969E-251	1.27602481	0.229	0.594	3.027E-247	T Cells
RGL24	2.117E-251	0.77483653	0.097	0.354	3.255E-247	T Cells
LENG84	6.622E-251	2.55280417	0.59	0.653	1.018E-246	T Cells
FTO1	9.424E-251	0.82376585	0.091	0.341	1.449E-246	T Cells
TBL1X2	1.263E-250	0.5987884	0.077	0.315	1.941E-246	T Cells
FAM89B	1.439E-250	1.09940472	0.259	0.655	2.212E-246	T Cells
GPRIN31	3.403E-250	0.53074167	0.373	0.896	5.231E-246	T Cells
SH3GLB2	4.146E-250	0.98817336	0.126	0.403	6.373E-246	T Cells
USP242	5.904E-250	1.19345792	0.178	0.496	9.075E-246	T Cells
ZNRD21	7.125E-250	0.62024068	0.074	0.312	1.095E-245	T Cells

ATMIN2	1.133E-249	0.89803997	0.106	0.368	1.742E-245	T Cells
MVB12B1	1.244E-249	1.01944579	0.157	0.456	1.913E-245	T Cells
EXOSC9	1.505E-249	1.15178909	0.154	0.452	2.313E-245	T Cells
MTMR21	1.531E-249	0.57847853	0.07	0.304	2.354E-245	T Cells
TENT4B2	2.649E-249	0.69438424	0.079	0.319	4.072E-245	T Cells
GBF11	3.008E-249	0.78688001	0.099	0.352	4.623E-245	T Cells
ZCCHC91	6.399E-249	0.67026896	0.079	0.319	9.836E-245	T Cells
PPA11	7.999E-249	0.59352006	0.076	0.315	1.23E-244	T Cells
TAPT11	9.808E-249	0.70688365	0.086	0.331	1.508E-244	T Cells
CHM1	1.328E-248	0.27394519	0.058	0.283	2.041E-244	T Cells
PHKG2	1.689E-248	0.72545899	0.088	0.333	2.596E-244	T Cells
NT5C3A1	2.763E-248	0.56035673	0.075	0.312	4.248E-244	T Cells
SEC14L2	5.981E-248	7.82664073	0.013	0	9.193E-244	T Cells
ATP13A34	5.989E-248	0.98739312	0.126	0.401	9.206E-244	T Cells
CAMLG	6.208E-248	1.22837723	0.149	0.444	9.543E-244	T Cells
CLEC2B3	7.31E-248	1.27203169	0.266	0.667	1.124E-243	T Cells
BCAS21	9.82E-248	0.73331203	0.077	0.317	1.509E-243	T Cells
PELI13	1.031E-247	0.16609504	0.064	0.291	1.585E-243	T Cells
CD79B	1.058E-247	6.94397856	0.027	0.002	1.627E-243	T Cells
RBBP72	1.112E-247	0.74143779	0.087	0.334	1.709E-243	T Cells
UNC45A	1.2E-247	0.43279451	0.07	0.304	1.845E-243	T Cells
KCNK5	1.287E-247	7.57406868	0.012	0	1.978E-243	T Cells
DCK3	1.477E-247	1.17920208	0.202	0.539	2.271E-243	T Cells
SMC3	1.593E-247	1.25941557	0.204	0.545	2.448E-243	T Cells
STARD102	1.696E-247	0.41878931	0.066	0.297	2.607E-243	T Cells
TP53RK	1.85E-247	0.86060105	0.093	0.342	2.844E-243	T Cells
MAP3K10	1.993E-247	0.32937154	0.058	0.284	3.063E-243	T Cells
GBA21	2.534E-247	0.6794895	0.1	0.353	3.896E-243	T Cells
LDAH2	4.093E-247	0.51538712	0.072	0.305	6.291E-243	T Cells
CCDC711	4.351E-247	0.47652816	0.068	0.298	6.687E-243	T Cells
BORCS82	5.955E-247	0.47600382	0.068	0.299	9.153E-243	T Cells
LTK1	8.085E-247	6.93638438	0.015	0	1.243E-242	T Cells
FUNDC11	9.188E-247	0.27609721	0.053	0.272	1.412E-242	T Cells
ARMC13	9.327E-247	0.22392492	0.054	0.273	1.434E-242	T Cells
PTEN2	1.161E-246	1.0944994	0.115	0.382	1.784E-242	T Cells
GON7	1.308E-246	0.3958002	0.061	0.286	2.011E-242	T Cells
MCM3AP3	1.404E-246	1.11340196	0.142	0.43	2.158E-242	T Cells
BOD12	1.477E-246	0.33048829	0.057	0.28	2.271E-242	T Cells
MVK	1.487E-246	0.38905563	0.073	0.308	2.285E-242	T Cells
ZMYM3	3.363E-246	0.47003377	0.072	0.305	5.169E-242	T Cells
SLC4A10	4.496E-246	9.73476433	0.01	0	6.911E-242	T Cells
DRAXIN	4.589E-246	9.81004027	0.01	0	7.053E-242	T Cells
EXTL3	9.318E-246	0.10352791	0.056	0.276	1.432E-241	T Cells
PCNX1	1.071E-245	1.07382957	0.293	0.721	1.646E-241	T Cells
TXNDC92	1.112E-245	0.64130768	0.072	0.305	1.709E-241	T Cells
HDHD31	1.258E-245	0.444797	0.073	0.306	1.933E-241	T Cells
PLPP52	1.397E-245	1.0832201	0.134	0.414	2.147E-241	T Cells
COQ8B1	1.416E-245	0.33028951	0.062	0.288	2.176E-241	T Cells
UBAP21	1.618E-245	0.31324444	0.056	0.277	2.487E-241	T Cells
VAMP4	1.627E-245	0.92895691	0.105	0.362	2.501E-241	T Cells
FAM53B1	1.764E-245	1.15834819	0.146	0.436	2.711E-241	T Cells
NUBP21	2.051E-245	0.70910531	0.083	0.322	3.153E-241	T Cells

TLE32	2.475E-245	0.68690593	0.088	0.333	3.805E-241	T Cells
ZBTB222	3.054E-245	0.68434563	0.085	0.327	4.694E-241	T Cells
TRAPPC92	4.548E-245	1.08700816	0.154	0.448	6.991E-241	T Cells
ERBIN1	6.282E-245	1.10581998	0.309	0.754	9.655E-241	T Cells
LYRM11	1.284E-244	0.68312965	0.081	0.318	1.974E-240	T Cells
ELP22	2.388E-244	1.28157939	0.182	0.5	3.67E-240	T Cells
DDX562	2.565E-244	0.99786509	0.119	0.386	3.943E-240	T Cells
ARHGAP264	2.741E-244	0.93389068	0.138	0.417	4.214E-240	T Cells
UBTF1	4.189E-244	1.07693449	0.145	0.432	6.438E-240	T Cells
STYX2	4.266E-244	1.23852242	0.155	0.452	6.557E-240	T Cells
NFX12	6.038E-244	1.20340748	0.182	0.499	9.281E-240	T Cells
CAST1	7.569E-244	0.19353651	0.406	0.95	1.163E-239	T Cells
GBP21	8.794E-244	0.92731592	0.325	0.786	1.352E-239	T Cells
IMPA12	9.864E-244	0.79728651	0.092	0.338	1.516E-239	T Cells
SIPA1L31	1.028E-243	0.36850445	0.064	0.29	1.581E-239	T Cells
RRP1B2	1.313E-243	1.22728071	0.147	0.436	2.018E-239	T Cells
COMMD71	1.379E-243	1.19576007	0.159	0.457	2.12E-239	T Cells
OXLD11	1.75E-243	0.89260108	0.111	0.37	2.69E-239	T Cells
MSANTD41	1.751E-243	0.84590303	0.091	0.336	2.691E-239	T Cells
FOXK11	1.88E-243	1.02863212	0.132	0.408	2.889E-239	T Cells
PEX162	2.643E-243	0.52075699	0.068	0.296	4.063E-239	T Cells
TIMM10B2	3.049E-243	1.20661886	0.15	0.439	4.686E-239	T Cells
CCNH2	3.183E-243	1.04439618	0.125	0.396	4.893E-239	T Cells
UPRT1	6.027E-243	0.43763308	0.062	0.286	9.264E-239	T Cells
STK17A2	6.949E-243	4.18682577	0.353	0.225	1.068E-238	T Cells
UBE3A3	7.726E-243	1.23059154	0.264	0.66	1.188E-238	T Cells
HTRA21	7.767E-243	0.78165327	0.085	0.327	1.194E-238	T Cells
ANAPC42	8.495E-243	1.12332836	0.155	0.448	1.306E-238	T Cells
STK391	9.669E-243	1.14541265	0.264	0.655	1.486E-238	T Cells
MIOS4	9.889E-243	0.84295208	0.096	0.346	1.52E-238	T Cells
SLC35A31	1.241E-242	1.07005779	0.125	0.397	1.908E-238	T Cells
RERE3	1.385E-242	1.18493557	0.189	0.512	2.129E-238	T Cells
MACO1	1.477E-242	0.92161963	0.097	0.347	2.27E-238	T Cells
TRMT15	1.831E-242	0.92596633	0.121	0.386	2.814E-238	T Cells
RBM194	1.836E-242	1.14982153	0.133	0.412	2.821E-238	T Cells
VSIG1	1.856E-242	9.34581725	0.01	0	2.853E-238	T Cells
SAFB21	2.097E-242	1.17688383	0.266	0.664	3.223E-238	T Cells
EFL11	2.14E-242	0.55695111	0.069	0.298	3.289E-238	T Cells
MED18	2.562E-242	1.18320414	0.166	0.468	3.938E-238	T Cells
WWOX1	3.144E-242	0.81095088	0.109	0.368	4.833E-238	T Cells
AARS22	3.599E-242	0.51535272	0.072	0.301	5.532E-238	T Cells
DDX522	3.638E-242	0.59318487	0.077	0.313	5.592E-238	T Cells
DENND1C	3.747E-242	1.12715676	0.176	0.488	5.759E-238	T Cells
ORAI13	4.642E-242	1.23482801	0.26	0.654	7.135E-238	T Cells
FAM177A12	5.976E-242	0.91251534	0.102	0.354	9.186E-238	T Cells
DUSP73	6.051E-242	1.04214885	0.13	0.406	9.301E-238	T Cells
GRAMD44	6.33E-242	0.79964186	0.106	0.36	9.73E-238	T Cells
GIGYF21	6.575E-242	0.75063245	0.089	0.331	1.011E-237	T Cells
TMEM14B1	6.983E-242	0.21579879	0.05	0.264	1.073E-237	T Cells
TPST2	8.355E-242	0.87849982	0.097	0.348	1.284E-237	T Cells
TARBP2	1.226E-241	0.15537795	0.05	0.264	1.885E-237	T Cells
NCAPH2	1.289E-241	1.21299912	0.171	0.478	1.982E-237	T Cells

DESI21	1.422E-241	0.44402526	0.065	0.29	2.185E-237	T Cells
UPF21	2.022E-241	1.05521442	0.124	0.395	3.108E-237	T Cells
RRP152	2.111E-241	0.76733385	0.082	0.321	3.245E-237	T Cells
LTN11	3.083E-241	1.13819072	0.134	0.411	4.739E-237	T Cells
NT5DC11	3.489E-241	0.88165738	0.097	0.344	5.363E-237	T Cells
MAN1A21	4.528E-241	1.1237172	0.142	0.424	6.96E-237	T Cells
YY1AP11	5.538E-241	1.14393202	0.141	0.423	8.512E-237	T Cells
FAAP20	5.83E-241	1.0223952	0.122	0.389	8.962E-237	T Cells
CCNG11	6.122E-241	0.97409562	0.11	0.367	9.409E-237	T Cells
FBXO331	7.147E-241	0.64850983	0.075	0.308	1.099E-236	T Cells
RFT11	7.368E-241	0.53459757	0.073	0.303	1.133E-236	T Cells
SNRNP702	8.043E-241	1.24934613	0.18	0.495	1.236E-236	T Cells
EXOSC31	1.333E-240	0.82550285	0.082	0.32	2.049E-236	T Cells
C1orf216	1.478E-240	0.64134206	0.077	0.311	2.272E-236	T Cells
MAN1C1	1.53E-240	0.33515858	0.056	0.275	2.351E-236	T Cells
PHIP1	1.913E-240	1.19013412	0.148	0.437	2.94E-236	T Cells
ZBTB32	2.124E-240	8.03171057	0.01	0	3.264E-236	T Cells
GPATCH31	2.525E-240	0.33647312	0.061	0.281	3.881E-236	T Cells
MARF12	3.823E-240	0.95207831	0.102	0.356	5.877E-236	T Cells
NOP21	4.718E-240	0.99711072	0.111	0.369	7.253E-236	T Cells
LZTFL1	4.764E-240	0.25404847	0.056	0.273	7.322E-236	T Cells
CLASP21	5.675E-240	0.81959432	0.089	0.331	8.723E-236	T Cells
MOB22	6.097E-240	1.16245279	0.138	0.417	9.372E-236	T Cells
PRDM41	6.483E-240	1.10807697	0.137	0.416	9.965E-236	T Cells
CRCP1	7.84E-240	0.85572212	0.089	0.331	1.205E-235	T Cells
CAMTA22	8.408E-240	1.07539202	0.121	0.389	1.292E-235	T Cells
TACO1	9.447E-240	0.31558649	0.06	0.279	1.452E-235	T Cells
SERINC1	1.182E-239	0.17937316	0.401	0.944	1.817E-235	T Cells
GEMIN4	1.344E-239	0.31630563	0.06	0.28	2.066E-235	T Cells
NUP2051	1.512E-239	1.2473538	0.161	0.459	2.325E-235	T Cells
PHKA22	1.652E-239	0.38751698	0.067	0.291	2.539E-235	T Cells
FBRSL15	1.739E-239	0.86305254	0.093	0.339	2.672E-235	T Cells
HIC21	1.954E-239	0.26717468	0.059	0.278	3.003E-235	T Cells
SEC24B1	2.113E-239	1.29660276	0.176	0.486	3.247E-235	T Cells
WDR741	2.654E-239	0.54211544	0.069	0.297	4.079E-235	T Cells
HLTF1	3.027E-239	0.56506155	0.072	0.3	4.652E-235	T Cells
DYM3	4.647E-239	1.29247185	0.24	0.605	7.142E-235	T Cells
STX33	5.134E-239	1.26047808	0.131	0.405	7.891E-235	T Cells
FAM160A23	5.658E-239	0.96631615	0.115	0.375	8.698E-235	T Cells
PDCD11	5.952E-239	0.83902965	0.096	0.342	9.149E-235	T Cells
IMPDH21	7.428E-239	0.6986306	0.078	0.311	1.142E-234	T Cells
ITPKC2	1.002E-238	0.47975	0.078	0.309	1.54E-234	T Cells
GCLC	1.125E-238	0.96700601	0.109	0.365	1.729E-234	T Cells
ASCC11	1.551E-238	0.44483228	0.065	0.288	2.384E-234	T Cells
SMARCC2	1.614E-238	1.12065808	0.13	0.404	2.481E-234	T Cells
PITPNM1	1.902E-238	1.16347524	0.203	0.535	2.923E-234	T Cells
ZNF6642	3.506E-238	1.03288785	0.115	0.376	5.39E-234	T Cells
PCNX24	6.119E-238	4.48955203	0.222	0.1	9.406E-234	T Cells
RNF43	6.265E-238	1.16428855	0.287	0.705	9.63E-234	T Cells
ATXN12	8.223E-238	1.28213263	0.253	0.632	1.264E-233	T Cells
MED221	9.034E-238	0.58992266	0.069	0.296	1.389E-233	T Cells
CLASRP3	1.479E-237	0.95324558	0.125	0.394	2.274E-233	T Cells

ATP7A1	1.487E-237	0.27618655	0.059	0.277	2.286E-233	T Cells
ZNF780A1	3.985E-237	0.36841582	0.066	0.289	6.125E-233	T Cells
KIAA02322	4.446E-237	1.11265362	0.12	0.386	6.833E-233	T Cells
PSME31	4.623E-237	0.12727556	0.046	0.255	7.107E-233	T Cells
RABEP21	4.679E-237	0.13606524	0.054	0.269	7.192E-233	T Cells
FXR11	5.35E-237	0.9623758	0.099	0.348	8.224E-233	T Cells
UBR7	6.087E-237	0.61670037	0.077	0.308	9.356E-233	T Cells
ANKRD521	8.41E-237	0.81998507	0.095	0.339	1.293E-232	T Cells
EP3003	8.549E-237	1.07736573	0.32	0.777	1.314E-232	T Cells
NCAPD21	1.168E-236	0.52769205	0.09	0.33	1.795E-232	T Cells
KDM5C2	1.625E-236	1.23403112	0.255	0.634	2.499E-232	T Cells
COQ62	1.705E-236	0.44900027	0.059	0.277	2.62E-232	T Cells
EDC42	2.498E-236	1.17356664	0.16	0.454	3.839E-232	T Cells
VPS33A2	2.52E-236	0.24443527	0.054	0.268	3.873E-232	T Cells
CLOCK2	3.902E-236	0.6877292	0.076	0.305	5.998E-232	T Cells
PDCD51	3.944E-236	0.85130971	0.094	0.335	6.063E-232	T Cells
LFNG3	5.086E-236	1.07025771	0.192	0.512	7.818E-232	T Cells
SPAST2	5.365E-236	0.52977447	0.07	0.296	8.246E-232	T Cells
SLC25A262	6.738E-236	0.9602105	0.099	0.347	1.036E-231	T Cells
RFX11	7.536E-236	0.79122003	0.087	0.326	1.158E-231	T Cells
PPRC11	8.433E-236	0.94936768	0.093	0.335	1.296E-231	T Cells
PRPF38B1	9.464E-236	1.2558247	0.252	0.631	1.455E-231	T Cells
NFATC2IP2	1.199E-235	1.21349054	0.17	0.473	1.843E-231	T Cells
EXOC61	2.107E-235	1.20293456	0.133	0.405	3.238E-231	T Cells
RAD51B1	3.37E-235	0.82088499	0.107	0.358	5.181E-231	T Cells
EIF3G1	3.395E-235	0.98292287	0.105	0.356	5.219E-231	T Cells
HENMT1	3.867E-235	0.96182035	0.096	0.34	5.943E-231	T Cells
TSSC41	4.785E-235	0.75824054	0.084	0.32	7.355E-231	T Cells
SORL13	8.301E-235	0.91269323	0.149	0.429	1.276E-230	T Cells
NCOA51	9.13E-235	0.83414041	0.084	0.321	1.403E-230	T Cells
SLC12A41	9.981E-235	0.52882633	0.068	0.292	1.534E-230	T Cells
ARID5B1	1.075E-234	4.48393732	0.203	0.086	1.652E-230	T Cells
PREPL	1.365E-234	1.15356584	0.122	0.387	2.099E-230	T Cells
PRR121	1.631E-234	1.23097203	0.15	0.438	2.508E-230	T Cells
BRPF11	1.743E-234	0.76279922	0.082	0.316	2.679E-230	T Cells
TRABD2A3	2.036E-234	6.6641288	0.029	0.003	3.129E-230	T Cells
HACD24	2.166E-234	0.77228442	0.084	0.319	3.33E-230	T Cells
TTC191	2.366E-234	0.97101945	0.107	0.359	3.637E-230	T Cells
HMGXB42	2.389E-234	0.63198949	0.072	0.298	3.672E-230	T Cells
CHAMP11	3.225E-234	0.81153421	0.087	0.322	4.957E-230	T Cells
DCAF151	3.679E-234	1.23279604	0.149	0.434	5.655E-230	T Cells
ATXN2L3	3.814E-234	1.28142351	0.203	0.537	5.862E-230	T Cells
MEAF6	8.358E-234	1.32200342	0.221	0.567	1.285E-229	T Cells
MANEA2	1.295E-233	0.83126778	0.082	0.313	1.991E-229	T Cells
WDR432	1.663E-233	0.98050741	0.094	0.337	2.556E-229	T Cells
CPEB21	1.891E-233	0.81006641	0.095	0.337	2.907E-229	T Cells
AKAP92	2.753E-233	1.22236562	0.269	0.66	4.232E-229	T Cells
UBE2D2	3.302E-233	1.32486829	0.152	0.44	5.076E-229	T Cells
PCIF12	6.496E-233	1.22725977	0.157	0.449	9.985E-229	T Cells
RAB152	7.058E-233	6.26123907	0.019	0.001	1.085E-228	T Cells
AKT31	8.937E-233	1.28351948	0.149	0.435	1.374E-228	T Cells
SMG81	1.237E-232	0.1862274	0.053	0.264	1.902E-228	T Cells

R3HDM12	1.251E-232	0.94207056	0.094	0.334	1.923E-228	T Cells
TMEM87B1	1.445E-232	1.02830143	0.105	0.354	2.22E-228	T Cells
TMEM18	1.509E-232	0.51315997	0.068	0.289	2.319E-228	T Cells
SART31	2.52E-232	1.29643917	0.181	0.494	3.874E-228	T Cells
BOD1L13	2.637E-232	0.81059831	0.09	0.328	4.053E-228	T Cells
CERS52	2.654E-232	0.95488036	0.106	0.356	4.08E-228	T Cells
BRIX12	3.258E-232	1.05327013	0.093	0.335	5.007E-228	T Cells
CHTOP1	6.77E-232	1.20456312	0.262	0.649	1.041E-227	T Cells
PPP1R12A1	8.298E-232	1.23707989	0.267	0.656	1.275E-227	T Cells
RUNDC11	9.903E-232	0.63798245	0.075	0.301	1.522E-227	T Cells
RPF22	1.422E-231	0.30081847	0.055	0.265	2.185E-227	T Cells
MRTO42	1.452E-231	0.25757308	0.05	0.259	2.232E-227	T Cells
C5orf241	2.082E-231	1.0239776	0.099	0.343	3.2E-227	T Cells
CCNQ1	3.934E-231	0.61693608	0.077	0.303	6.048E-227	T Cells
EXOSC81	5.625E-231	0.8294597	0.089	0.324	8.646E-227	T Cells
CEP1642	6.549E-231	0.54611331	0.076	0.302	1.007E-226	T Cells
PCBP21	1.206E-230	0.95411966	0.098	0.34	1.854E-226	T Cells
FRA10AC12	1.406E-230	0.68078027	0.069	0.292	2.162E-226	T Cells
SNRNP35	2.003E-230	1.01319954	0.113	0.365	3.078E-226	T Cells
ZDHHC211	2.541E-230	0.36638023	0.064	0.281	3.906E-226	T Cells
RNF113A1	3.717E-230	1.10748311	0.109	0.359	5.713E-226	T Cells
KLHL241	3.913E-230	1.17798192	0.141	0.417	6.015E-226	T Cells
CREBBP1	4.13E-230	1.2967746	0.253	0.628	6.349E-226	T Cells
RBM282	6.841E-230	0.82747478	0.087	0.321	1.051E-225	T Cells
TMEM50A	1.334E-229	0.49468297	0.383	0.911	2.051E-225	T Cells
ZC3H101	1.816E-229	0.55848592	0.07	0.29	2.791E-225	T Cells
TEP13	2.001E-229	1.00842881	0.126	0.388	3.076E-225	T Cells
FRG12	2.216E-229	0.7914919	0.075	0.301	3.405E-225	T Cells
RPP21	2.617E-229	0.5497593	0.066	0.283	4.022E-225	T Cells
ATXN7L3B1	3.323E-229	1.28293034	0.211	0.544	5.108E-225	T Cells
CCDC1172	3.854E-229	0.52829731	0.069	0.289	5.924E-225	T Cells
ARIH21	6.685E-229	1.3046742	0.184	0.493	1.028E-224	T Cells
ATF71	7.967E-229	0.55673535	0.073	0.294	1.225E-224	T Cells
PPP4R22	9.656E-229	0.83527491	0.092	0.33	1.484E-224	T Cells
UFSP22	1.551E-228	0.86785301	0.081	0.311	2.384E-224	T Cells
FBXO301	1.71E-228	0.94948282	0.089	0.324	2.629E-224	T Cells
NDUFAF4	1.817E-228	0.49067885	0.056	0.266	2.793E-224	T Cells
TRPT11	1.936E-228	0.56832407	0.076	0.301	2.976E-224	T Cells
PSMB81	2.255E-228	0.31341297	0.395	0.925	3.467E-224	T Cells
TRUB11	3.366E-228	0.62337376	0.064	0.28	5.174E-224	T Cells
GCC11	3.429E-228	0.74660729	0.069	0.292	5.271E-224	T Cells
ZSCAN322	4.414E-228	0.47821244	0.065	0.281	6.785E-224	T Cells
KIF13B2	7.606E-228	1.24900365	0.144	0.419	1.169E-223	T Cells
SNRPE1	9.525E-228	0.62890514	0.064	0.281	1.464E-223	T Cells
TBCK2	9.87E-228	0.74862748	0.088	0.322	1.517E-223	T Cells
DVL21	1.067E-227	0.81071707	0.083	0.313	1.641E-223	T Cells
KNOP1	1.093E-227	0.48980168	0.058	0.271	1.68E-223	T Cells
POLD21	1.164E-227	0.75520302	0.077	0.303	1.789E-223	T Cells
POLR2D	1.199E-227	0.88309067	0.088	0.323	1.843E-223	T Cells
RNF251	1.436E-227	0.7246283	0.068	0.288	2.208E-223	T Cells
KIF3B1	1.776E-227	1.01753825	0.092	0.331	2.73E-223	T Cells
WDR241	1.921E-227	0.17681212	0.049	0.254	2.952E-223	T Cells

AHR3	1.987E-227	0.97044924	0.343	0.81	3.054E-223	T Cells
NBAS1	2.146E-227	1.10785404	0.121	0.378	3.299E-223	T Cells
ASS13	3.778E-227	5.73773934	0.029	0.003	5.807E-223	T Cells
HRAS1	3.899E-227	0.87282543	0.091	0.327	5.994E-223	T Cells
ZFAND2B1	5.24E-227	1.30076195	0.176	0.481	8.055E-223	T Cells
IRF2BP1	5.642E-227	0.90192827	0.092	0.327	8.672E-223	T Cells
TCHP	6.815E-227	0.80424369	0.084	0.313	1.048E-222	T Cells
DEPDC53	8.956E-227	0.98357714	0.114	0.366	1.377E-222	T Cells
RALA4	1.387E-226	1.04575966	0.113	0.366	2.133E-222	T Cells
ARL6IP61	1.811E-226	0.86663252	0.081	0.309	2.784E-222	T Cells
NLRC53	2.35E-226	3.71441199	0.379	0.264	3.612E-222	T Cells
DCP1A1	5.603E-226	1.15645901	0.12	0.379	8.612E-222	T Cells
ALG132	1.501E-225	1.24399373	0.223	0.566	2.308E-221	T Cells
DENND6A1	1.576E-225	1.2408028	0.147	0.424	2.422E-221	T Cells
DPH6	1.631E-225	0.41634375	0.059	0.27	2.506E-221	T Cells
SLC39A104	2.657E-225	1.21953808	0.276	0.67	4.084E-221	T Cells
NUP1531	2.955E-225	1.34676244	0.189	0.501	4.543E-221	T Cells
FOXJ32	3.064E-225	1.06771128	0.107	0.355	4.71E-221	T Cells
RASSF11	4.242E-225	1.21868561	0.145	0.419	6.52E-221	T Cells
TRAPPC101	4.912E-225	1.25831147	0.271	0.664	7.55E-221	T Cells
EYA32	5.503E-225	0.85697314	0.088	0.32	8.459E-221	T Cells
SERTAD13	5.535E-225	0.23208989	0.049	0.253	8.508E-221	T Cells
FAM160B22	6.157E-225	0.79033964	0.087	0.318	9.464E-221	T Cells
TCERG12	6.677E-225	1.26908328	0.203	0.528	1.026E-220	T Cells
RIC8B	8.404E-225	0.65279543	0.072	0.292	1.292E-220	T Cells
SMAP22	1.067E-224	1.03201695	0.325	0.776	1.64E-220	T Cells
PLEKHA32	1.486E-224	1.28641889	0.144	0.417	2.284E-220	T Cells
PRPF4B2	2.124E-224	1.31636681	0.193	0.51	3.265E-220	T Cells
CRKL1	3.413E-224	1.39814036	0.222	0.566	5.247E-220	T Cells
GFM22	4.269E-224	0.72183951	0.079	0.303	6.561E-220	T Cells
OTUD6B1	4.293E-224	0.78718669	0.082	0.31	6.599E-220	T Cells
PHF62	5.234E-224	0.75049833	0.079	0.304	8.045E-220	T Cells
ITSN23	5.648E-224	1.23910268	0.269	0.654	8.682E-220	T Cells
SRPK22	6.06E-224	1.23782514	0.143	0.415	9.315E-220	T Cells
METTL21A3	6.157E-224	0.15370559	0.049	0.251	9.464E-220	T Cells
PLXNA42	6.793E-224	7.07501829	0.011	0	1.044E-219	T Cells
FBXL191	7.813E-224	0.29010842	0.058	0.266	1.201E-219	T Cells
ADSS1	8.002E-224	1.19450676	0.286	0.694	1.23E-219	T Cells
C18orf211	1.344E-223	0.47191144	0.058	0.267	2.065E-219	T Cells
SLC44A13	1.603E-223	0.67625114	0.071	0.288	2.465E-219	T Cells
FMR12	2.645E-223	1.38038109	0.178	0.479	4.065E-219	T Cells
LCOR3	3.249E-223	1.35218722	0.22	0.559	4.994E-219	T Cells
FBXL31	3.404E-223	1.17370316	0.31	0.744	5.233E-219	T Cells
TLK22	3.88E-223	0.72122565	0.069	0.287	5.964E-219	T Cells
SLMAP2	3.946E-223	1.17046696	0.125	0.385	6.065E-219	T Cells
UBE2M1	5.26E-223	0.33463683	0.05	0.254	8.086E-219	T Cells
ZNF255	5.582E-223	0.60821355	0.063	0.276	8.581E-219	T Cells
AGMAT	5.78E-223	8.0776503	0.013	0	8.884E-219	T Cells
JPT22	6.483E-223	0.9942163	0.1	0.339	9.966E-219	T Cells
POLB2	8.607E-223	0.54408712	0.058	0.268	1.323E-218	T Cells
ADNP21	9.199E-223	1.09149061	0.108	0.354	1.414E-218	T Cells
WDR182	1.016E-222	0.14387656	0.055	0.26	1.562E-218	T Cells

CDK19	1.023E-222	0.22237553	0.054	0.26	1.573E-218	T Cells
ZBTB212	1.178E-222	0.88868649	0.084	0.312	1.81E-218	T Cells
HNRNPR2	1.778E-222	1.09070782	0.108	0.354	2.733E-218	T Cells
ARFRP11	1.789E-222	1.09453127	0.119	0.371	2.749E-218	T Cells
TMEM621	2.344E-222	0.28924681	0.052	0.255	3.604E-218	T Cells
DROSHA1	2.544E-222	1.15272908	0.111	0.36	3.91E-218	T Cells
LSM14A1	2.68E-222	1.18534935	0.131	0.395	4.119E-218	T Cells
NELFA	4.325E-222	1.15652721	0.133	0.396	6.648E-218	T Cells
MAP2K41	5.551E-222	0.73437129	0.07	0.289	8.533E-218	T Cells
KATNB1	7.23E-222	0.30607982	0.059	0.268	1.111E-217	T Cells
ITGA1	8.667E-222	5.20183198	0.145	0.05	1.332E-217	T Cells
MTHFR1	9.005E-222	0.95314353	0.102	0.341	1.384E-217	T Cells
C1orf351	1.09E-221	0.22686517	0.052	0.254	1.676E-217	T Cells
MECP21	1.134E-221	1.32274202	0.203	0.527	1.743E-217	T Cells
MAP4K44	1.138E-221	1.18175441	0.156	0.441	1.749E-217	T Cells
DOT1L3	2.422E-221	0.91009595	0.108	0.353	3.724E-217	T Cells
SLFN52	2.618E-221	3.30914992	0.51	0.496	4.024E-217	T Cells
KMT2D	3.9E-221	1.1705453	0.298	0.719	5.995E-217	T Cells
P2RX5	4.333E-221	5.84993977	0.045	0.006	6.661E-217	T Cells
ZNF2771	4.929E-221	1.11856821	0.104	0.346	7.576E-217	T Cells
MOCS3	5.005E-221	0.15877038	0.046	0.245	7.694E-217	T Cells
CFAP201	5.134E-221	0.60165376	0.061	0.272	7.892E-217	T Cells
ARHGEF13	5.346E-221	2.6222846	0.596	0.722	8.218E-217	T Cells
TACC31	5.384E-221	0.73426977	0.119	0.369	8.276E-217	T Cells
MCU	6.35E-221	0.16315925	0.049	0.25	9.761E-217	T Cells
TMX41	7.306E-221	1.20692191	0.297	0.709	1.123E-216	T Cells
ISG20L21	7.417E-221	1.24070807	0.127	0.385	1.14E-216	T Cells
CHD14	1.212E-220	1.11955277	0.142	0.414	1.862E-216	T Cells
NICN1	1.599E-220	0.50325178	0.065	0.278	2.457E-216	T Cells
PIP4K2A	2.104E-220	1.26706864	0.295	0.708	3.235E-216	T Cells
ARFGAP12	2.325E-220	0.37860103	0.057	0.263	3.574E-216	T Cells
ZBTB481	2.619E-220	0.58646097	0.076	0.295	4.026E-216	T Cells
MOAP1	2.994E-220	1.18318727	0.134	0.4	4.602E-216	T Cells
ATG2A1	3.892E-220	0.3752905	0.068	0.279	5.983E-216	T Cells
PRORP2	4.638E-220	0.76929776	0.075	0.294	7.128E-216	T Cells
FBXL121	4.672E-220	0.24677752	0.055	0.26	7.181E-216	T Cells
GGPS11	4.979E-220	0.75245664	0.074	0.292	7.654E-216	T Cells
UCHL52	5.453E-220	0.74759671	0.077	0.297	8.382E-216	T Cells
NUP431	6.075E-220	1.31295043	0.167	0.456	9.339E-216	T Cells
RNF2142	8.177E-220	0.89986976	0.081	0.305	1.257E-215	T Cells
DAD1	8.311E-220	0.56328369	0.068	0.282	1.278E-215	T Cells
NECAP12	8.53E-220	1.09393574	0.108	0.353	1.311E-215	T Cells
MTRR1	1.068E-219	0.9231801	0.089	0.318	1.641E-215	T Cells
PDZD8	1.131E-219	1.12054591	0.108	0.353	1.738E-215	T Cells
INTS121	1.176E-219	0.2833466	0.053	0.256	1.807E-215	T Cells
DCAF14	1.196E-219	1.11430906	0.11	0.355	1.838E-215	T Cells
TSTD21	2.298E-219	0.53007458	0.064	0.275	3.532E-215	T Cells
FAM192A2	4.684E-219	0.60100515	0.062	0.271	7.199E-215	T Cells
POU2F11	7.851E-219	0.62448924	0.067	0.28	1.207E-214	T Cells
ANKRD13D1	8.102E-219	0.9647472	0.305	0.724	1.245E-214	T Cells
UCKL12	1.044E-218	1.12713874	0.128	0.384	1.604E-214	T Cells
AP1S25	1.953E-218	0.87554465	0.095	0.326	3.001E-214	T Cells

RB1CC11	1.96E-218	1.32091649	0.156	0.435	3.012E-214	T Cells
DPH22	2.862E-218	0.59746558	0.072	0.287	4.399E-214	T Cells
THOC11	2.904E-218	0.91954542	0.094	0.325	4.464E-214	T Cells
RC3H21	3.276E-218	1.35016002	0.248	0.611	5.036E-214	T Cells
ATXN1L2	7.381E-218	0.99160778	0.11	0.354	1.134E-213	T Cells
RNMT4	7.616E-218	1.04843878	0.105	0.347	1.171E-213	T Cells
ANKLE22	8.676E-218	1.3672389	0.213	0.54	1.334E-213	T Cells
EXOC6B1	1.363E-217	0.87587632	0.089	0.318	2.096E-213	T Cells
TSHZ12	1.592E-217	0.9421198	0.093	0.323	2.447E-213	T Cells
FXYD53	1.635E-217	1.15551679	0.699	0.963	2.514E-213	T Cells
CHSY14	1.705E-217	1.09453764	0.122	0.373	2.621E-213	T Cells
RSPRY11	1.865E-217	1.1684763	0.109	0.351	2.866E-213	T Cells
CCDC71L2	2.006E-217	0.54046148	0.062	0.271	3.084E-213	T Cells
SREK1IP11	2.459E-217	0.57497812	0.062	0.27	3.78E-213	T Cells
RBMXL1	2.911E-217	0.92526507	0.089	0.318	4.475E-213	T Cells
NFATC12	4.541E-217	0.9248249	0.097	0.329	6.98E-213	T Cells
MED61	5.147E-217	1.25256647	0.109	0.353	7.912E-213	T Cells
HCST	8.503E-217	0.83853489	0.316	0.744	1.307E-212	T Cells
COPS7B1	1.151E-216	1.05201188	0.11	0.352	1.77E-212	T Cells
ZCCHC4	1.207E-216	0.73613419	0.069	0.282	1.856E-212	T Cells
ARPC5L	1.432E-216	1.36055874	0.181	0.482	2.201E-212	T Cells
NSMCE31	1.825E-216	1.40338856	0.211	0.536	2.805E-212	T Cells
PAQR8	2.945E-216	0.82460733	0.097	0.331	4.526E-212	T Cells
TMEM2031	3.723E-216	0.59922048	0.064	0.273	5.722E-212	T Cells
TNRC6A2	4.665E-216	1.33835558	0.146	0.417	7.171E-212	T Cells
LDLRAP1	5.243E-216	1.14988273	0.115	0.363	8.058E-212	T Cells
ACSF31	5.48E-216	1.13100667	0.122	0.373	8.423E-212	T Cells
DNAAF5	5.85E-216	0.66027809	0.066	0.277	8.992E-212	T Cells
IQCG2	5.969E-216	0.33460234	0.058	0.262	9.174E-212	T Cells
MSH31	6.508E-216	0.48953647	0.056	0.26	1E-211	T Cells
VIRMA2	6.667E-216	0.96952049	0.091	0.319	1.025E-211	T Cells
ACADSB1	7.831E-216	0.53983428	0.064	0.272	1.204E-211	T Cells
MSANTD32	1.196E-215	0.86877976	0.081	0.302	1.838E-211	T Cells
ZNF6381	1.226E-215	1.33888776	0.225	0.562	1.885E-211	T Cells
CLCC11	1.338E-215	1.34840027	0.135	0.399	2.056E-211	T Cells
ZBTB141	1.497E-215	0.94911822	0.094	0.324	2.301E-211	T Cells
DYRK1A2	1.631E-215	1.35083879	0.187	0.49	2.506E-211	T Cells
ZNF5522	1.949E-215	0.10850667	0.044	0.237	2.997E-211	T Cells
THRA1	1.95E-215	0.77578318	0.079	0.298	2.998E-211	T Cells
ZNF1892	2.317E-215	0.19138743	0.053	0.253	3.562E-211	T Cells
CLK31	2.494E-215	1.25892478	0.133	0.395	3.834E-211	T Cells
POT1	3.569E-215	0.49438856	0.059	0.265	5.486E-211	T Cells
TMEM161A	3.656E-215	0.18700855	0.049	0.246	5.619E-211	T Cells
SAYSD1	4.223E-215	0.74730195	0.074	0.289	6.492E-211	T Cells
KIAA1671	5.951E-215	5.78324028	0.049	0.008	9.147E-211	T Cells
EVC1	9.892E-215	6.44510534	0.028	0.003	1.521E-210	T Cells
PPIL41	1.058E-214	1.02082621	0.093	0.322	1.626E-210	T Cells
CREM2	1.187E-214	1.11536443	0.211	0.536	1.825E-210	T Cells
SLAIN11	2.698E-214	6.02274616	0.048	0.007	4.148E-210	T Cells
COG8	3.101E-214	0.79450249	0.072	0.285	4.767E-210	T Cells
ZFAND11	3.487E-214	1.07187841	0.107	0.345	5.36E-210	T Cells
SNRPN1	4.188E-214	1.32649743	0.167	0.453	6.437E-210	T Cells

ZNF2821	5.523E-214	0.63250722	0.072	0.285	8.489E-210	T Cells
BLMH	5.565E-214	0.73478238	0.072	0.287	8.554E-210	T Cells
VPS13C3	5.633E-214	1.05370041	0.334	0.794	8.659E-210	T Cells
OGFOD21	7.763E-214	0.74669632	0.076	0.292	1.193E-209	T Cells
BRPF3	8.536E-214	0.91199507	0.088	0.314	1.312E-209	T Cells
LIG33	1.205E-213	0.77889549	0.07	0.282	1.851E-209	T Cells
ZNF264	1.467E-213	1.37849123	0.164	0.447	2.256E-209	T Cells
CLSTN12	1.623E-213	1.28428245	0.182	0.479	2.495E-209	T Cells
MED111	1.715E-213	0.80468319	0.077	0.294	2.636E-209	T Cells
PAFAH1B3	2.196E-213	0.70612676	0.075	0.289	3.376E-209	T Cells
MKKS1	2.507E-213	0.50877664	0.06	0.264	3.854E-209	T Cells
RHNO12	2.703E-213	1.0016586	0.098	0.329	4.155E-209	T Cells
CMC4	4.799E-213	1.09960787	0.101	0.336	7.376E-209	T Cells
ZNF5241	5.868E-213	0.30991725	0.056	0.256	9.019E-209	T Cells
NFYA1	1.064E-212	0.99806888	0.094	0.321	1.635E-208	T Cells
PLEKHJ11	1.18E-212	0.98489411	0.092	0.32	1.815E-208	T Cells
ZNF6414	1.242E-212	0.99810373	0.105	0.341	1.909E-208	T Cells
NR2C2AP2	1.773E-212	0.53509936	0.063	0.269	2.725E-208	T Cells
GRAMD1B1	2.558E-212	1.0839125	0.12	0.369	3.932E-208	T Cells
TIMM22	8.73E-212	0.85136844	0.073	0.286	1.342E-207	T Cells
PNRC13	1.273E-211	1.34087891	0.683	0.94	1.957E-207	T Cells
SLK2	1.912E-211	1.44077704	0.232	0.57	2.94E-207	T Cells
GPN21	2.306E-211	0.64629526	0.065	0.272	3.544E-207	T Cells
SHISA5	2.718E-211	0.34478947	0.4	0.921	4.178E-207	T Cells
MBTD11	2.952E-211	0.78979102	0.079	0.296	4.538E-207	T Cells
CRTAM1	3.176E-211	0.10561937	0.04	0.227	4.881E-207	T Cells
LRCH11	3.198E-211	1.05334065	0.101	0.333	4.916E-207	T Cells
SDAD11	3.521E-211	0.85468198	0.069	0.28	5.411E-207	T Cells
AP5S1	3.826E-211	0.343751	0.054	0.252	5.881E-207	T Cells
IKZF21	4.201E-211	0.70739644	0.061	0.264	6.457E-207	T Cells
SLC30A62	4.606E-211	0.91990651	0.079	0.296	7.08E-207	T Cells
NIFK3	5.585E-211	1.11504193	0.099	0.329	8.585E-207	T Cells
TRMT10C1	5.701E-211	0.7173621	0.065	0.272	8.763E-207	T Cells
FAM122B2	7.178E-211	1.23353269	0.122	0.373	1.103E-206	T Cells
MOB3A4	7.737E-211	0.81601666	0.366	0.861	1.189E-206	T Cells
CDK5RAP32	7.855E-211	1.04619783	0.311	0.731	1.207E-206	T Cells
PHYKPL4	8.154E-211	1.18165853	0.141	0.405	1.253E-206	T Cells
ANO81	9.019E-211	0.3293692	0.058	0.258	1.386E-206	T Cells
LPGAT14	1.047E-210	1.26977446	0.16	0.436	1.61E-206	T Cells
MZB11	1.314E-210	6.3259714	0.011	0	2.02E-206	T Cells
ALKBH6	1.338E-210	0.63255041	0.067	0.274	2.057E-206	T Cells
MEN11	1.545E-210	0.66069052	0.071	0.282	2.374E-206	T Cells
SMARCAD12	1.795E-210	1.04654934	0.098	0.328	2.759E-206	T Cells
REV3L1	2.183E-210	0.66773906	0.072	0.282	3.355E-206	T Cells
DOCK8	2.56E-210	0.29484043	0.411	0.945	3.934E-206	T Cells
EPC21	3.006E-210	1.30071338	0.167	0.451	4.621E-206	T Cells
MKRN21	3.313E-210	0.94240285	0.083	0.303	5.093E-206	T Cells
KANSL22	3.821E-210	1.06744331	0.107	0.344	5.874E-206	T Cells
HIRIP3	4.609E-210	0.14396126	0.042	0.231	7.084E-206	T Cells
LARS21	4.883E-210	0.43029762	0.053	0.25	7.506E-206	T Cells
RECQL5	5.189E-210	0.32660221	0.054	0.251	7.976E-206	T Cells
VTI1A2	5.464E-210	1.07584212	0.117	0.359	8.399E-206	T Cells

RORA	7.143E-210	4.03312646	0.38	0.275	1.098E-205	T Cells
TLK11	7.476E-210	1.36688198	0.233	0.573	1.149E-205	T Cells
PPAN3	9.279E-210	1.19863629	0.125	0.377	1.426E-205	T Cells
MAD2L21	1.187E-209	1.08334565	0.103	0.337	1.825E-205	T Cells
ZNF146	1.212E-209	1.42846017	0.251	0.609	1.863E-205	T Cells
USP7	1.371E-209	1.25553113	0.125	0.373	2.108E-205	T Cells
CHMP4A1	1.389E-209	1.36087707	0.265	0.642	2.135E-205	T Cells
CLEC16A	1.635E-209	0.97713769	0.093	0.32	2.514E-205	T Cells
CAMK2D2	2.217E-209	0.79226727	0.071	0.28	3.407E-205	T Cells
BRD3OS1	3.002E-209	0.63829385	0.066	0.271	4.615E-205	T Cells
INVS2	3.12E-209	0.91640875	0.088	0.309	4.796E-205	T Cells
SCRN21	3.397E-209	0.31771343	0.054	0.25	5.221E-205	T Cells
SAMD14	4.216E-209	0.97433434	0.132	0.385	6.481E-205	T Cells
CSNK1G12	7.622E-209	1.07358629	0.106	0.341	1.172E-204	T Cells
ZSWIM1	7.974E-209	0.1212352	0.037	0.221	1.226E-204	T Cells
SAMD91	1.269E-208	1.31060253	0.144	0.407	1.951E-204	T Cells
WDR731	1.296E-208	0.46026236	0.054	0.251	1.992E-204	T Cells
AKAP71	1.71E-208	0.67269773	0.057	0.256	2.628E-204	T Cells
FAM228B2	1.735E-208	0.165515	0.044	0.233	2.667E-204	T Cells
AP1G2	1.983E-208	1.29308936	0.205	0.518	3.048E-204	T Cells
IFNAR24	2.14E-208	1.37042927	0.196	0.504	3.289E-204	T Cells
ZNF414	2.69E-208	0.79501053	0.069	0.276	4.135E-204	T Cells
YRDC1	2.866E-208	0.38353646	0.054	0.25	4.405E-204	T Cells
PANK41	4.56E-208	1.18925465	0.122	0.367	7.009E-204	T Cells
ATXN22	4.623E-208	1.28735854	0.144	0.409	7.106E-204	T Cells
AMZ22	4.901E-208	1.03955943	0.088	0.31	7.533E-204	T Cells
MS4A11	4.93E-208	7.03566252	0.011	0	7.578E-204	T Cells
PSD41	6.916E-208	1.28121831	0.283	0.68	1.063E-203	T Cells
ZNF5131	1.01E-207	0.61451917	0.07	0.277	1.552E-203	T Cells
FAM76A2	1.127E-207	0.89033463	0.089	0.308	1.732E-203	T Cells
STYK1	1.139E-207	7.05544836	0.012	0	1.751E-203	T Cells
SCAF81	1.202E-207	1.3768447	0.208	0.526	1.848E-203	T Cells
RPA22	1.701E-207	1.01423076	0.097	0.324	2.614E-203	T Cells
UNK1	1.822E-207	0.80333093	0.078	0.293	2.801E-203	T Cells
KLHL212	2.071E-207	1.22326169	0.142	0.404	3.184E-203	T Cells
CHST12	2.132E-207	1.4160574	0.216	0.539	3.277E-203	T Cells
CYTH23	2.193E-207	1.30812583	0.226	0.557	3.371E-203	T Cells
DOP1A2	2.431E-207	0.4162839	0.055	0.252	3.737E-203	T Cells
PRPF38A3	4.09E-207	1.33396977	0.148	0.415	6.287E-203	T Cells
ATG142	4.399E-207	0.5140907	0.063	0.266	6.761E-203	T Cells
SPRYD42	5.322E-207	0.19673273	0.049	0.24	8.18E-203	T Cells
TMEM39A1	5.4E-207	0.71729842	0.074	0.283	8.3E-203	T Cells
ANKRD271	8.032E-207	1.19013738	0.124	0.371	1.235E-202	T Cells
ZNF8001	8.299E-207	1.35390401	0.255	0.616	1.276E-202	T Cells
CUL51	8.65E-207	1.21485148	0.121	0.365	1.33E-202	T Cells
PTER	1.047E-206	0.73334071	0.064	0.268	1.609E-202	T Cells
CDIP11	1.457E-206	0.84849475	0.076	0.287	2.24E-202	T Cells
PHB2	1.678E-206	0.10488454	0.044	0.231	2.579E-202	T Cells
VPS37B1	1.763E-206	0.91107011	0.097	0.321	2.709E-202	T Cells
FAM114A2	1.83E-206	0.72505253	0.067	0.271	2.813E-202	T Cells
IDNK1	1.898E-206	0.17039276	0.045	0.233	2.917E-202	T Cells
PRMT91	2.148E-206	0.67984587	0.063	0.265	3.301E-202	T Cells

SENP22	2.305E-206	1.39301544	0.159	0.432	3.543E-202	T Cells
SPRY11	2.307E-206	0.33591396	0.047	0.238	3.546E-202	T Cells
NIF3L1	2.75E-206	0.41899452	0.045	0.235	4.227E-202	T Cells
CAAP12	2.856E-206	0.59180947	0.058	0.257	4.39E-202	T Cells
GEMIN5	3.873E-206	0.20016245	0.04	0.226	5.953E-202	T Cells
STK241	5.073E-206	0.5718316	0.395	0.909	7.797E-202	T Cells
PEX61	5.842E-206	0.59762473	0.065	0.268	8.98E-202	T Cells
RBM271	6.461E-206	1.32806545	0.14	0.399	9.931E-202	T Cells
SCAMP12	9.359E-206	0.49588613	0.062	0.262	1.439E-201	T Cells
PMM22	1.522E-205	1.27007488	0.123	0.368	2.339E-201	T Cells
SUPT7L1	1.671E-205	0.95465897	0.087	0.305	2.569E-201	T Cells
SIKE13	2.087E-205	1.30345705	0.118	0.361	3.207E-201	T Cells
PEX31	2.968E-205	0.6658814	0.062	0.263	4.562E-201	T Cells
ABCF21	3.012E-205	0.57263151	0.059	0.257	4.629E-201	T Cells
NFKBIB2	3.169E-205	0.50471802	0.06	0.259	4.871E-201	T Cells
EXOC81	4.426E-205	1.19805464	0.12	0.361	6.803E-201	T Cells
DSN11	5.14E-205	0.140647	0.042	0.228	7.901E-201	T Cells
ZBTB411	9.284E-205	0.87658604	0.073	0.281	1.427E-200	T Cells
SLC25A324	1.163E-204	1.24408731	0.119	0.363	1.787E-200	T Cells
BIRC32	1.199E-204	4.736218	0.162	0.063	1.844E-200	T Cells
PRRC2C3	2.169E-204	1.32448935	0.285	0.677	3.335E-200	T Cells
ATF43	2.399E-204	1.29498712	0.239	0.583	3.687E-200	T Cells
MSH61	2.424E-204	0.71948808	0.073	0.28	3.725E-200	T Cells
PAM	2.865E-204	0.922754	0.092	0.313	4.404E-200	T Cells
NELFE	3.865E-204	0.1168174	0.047	0.235	5.941E-200	T Cells
BCL34	4.187E-204	1.1771468	0.169	0.447	6.436E-200	T Cells
MTAP1	5.418E-204	0.38253316	0.049	0.239	8.328E-200	T Cells
SMARCAL11	5.605E-204	0.49412996	0.058	0.255	8.615E-200	T Cells
UTP112	1.031E-203	0.34751651	0.044	0.231	1.584E-199	T Cells
TMPO1	1.078E-203	1.18901572	0.166	0.444	1.656E-199	T Cells
ZNF3181	1.417E-203	0.89227582	0.074	0.282	2.178E-199	T Cells
FBXO341	1.482E-203	1.21293942	0.123	0.367	2.278E-199	T Cells
C16orf723	1.482E-203	1.3598386	0.267	0.637	2.279E-199	T Cells
MLXIP1	1.868E-203	0.77325444	0.078	0.289	2.871E-199	T Cells
DGCR22	2.137E-203	1.15420815	0.114	0.35	3.284E-199	T Cells
PDPR4	2.406E-203	1.14082196	0.127	0.371	3.698E-199	T Cells
SUB11	3.196E-203	1.14581559	0.114	0.35	4.913E-199	T Cells
TTI21	3.21E-203	0.15450388	0.044	0.23	4.934E-199	T Cells
PCGF33	5.44E-203	1.02897931	0.095	0.319	8.362E-199	T Cells
ZNF6542	5.548E-203	1.04047302	0.091	0.311	8.528E-199	T Cells
HIPK11	6.974E-203	1.24438305	0.301	0.707	1.072E-198	T Cells
TRIR	9.838E-203	0.49364782	0.394	0.914	1.512E-198	T Cells
FAM118A4	1.184E-202	4.86671115	0.078	0.018	1.82E-198	T Cells
LRCH43	1.185E-202	1.19485603	0.28	0.662	1.821E-198	T Cells
IL2RA1	1.522E-202	6.27417322	0.056	0.01	2.34E-198	T Cells
SENP63	1.683E-202	1.34014477	0.278	0.661	2.586E-198	T Cells
B3GALT6	1.766E-202	0.34707568	0.055	0.248	2.715E-198	T Cells
MED231	2.325E-202	1.18883711	0.116	0.354	3.573E-198	T Cells
NCBP31	2.482E-202	1.32090956	0.147	0.408	3.816E-198	T Cells
LIN541	2.533E-202	1.0954764	0.097	0.32	3.893E-198	T Cells
C19orf12	2.594E-202	1.32595214	0.15	0.412	3.988E-198	T Cells
ZSCAN292	3.056E-202	0.52389987	0.057	0.251	4.698E-198	T Cells

PIGF1	3.291E-202	0.77392115	0.067	0.269	5.058E-198	T Cells
CEP1042	4.121E-202	1.22343541	0.116	0.353	6.335E-198	T Cells
CETN3	6.931E-202	0.12189363	0.041	0.224	1.065E-197	T Cells
FHOD13	7.492E-202	0.98103462	0.097	0.321	1.152E-197	T Cells
TAF113	9.227E-202	1.146798	0.11	0.344	1.418E-197	T Cells
IP6K21	1.018E-201	1.03534325	0.106	0.336	1.565E-197	T Cells
ITGB12	1.072E-201	1.33338316	0.297	0.699	1.647E-197	T Cells
TIAM2	1.097E-201	7.23490776	0.017	0.001	1.686E-197	T Cells
TRIM11	1.334E-201	0.80462391	0.073	0.279	2.05E-197	T Cells
XPR12	2.108E-201	0.83246591	0.07	0.275	3.241E-197	T Cells
MED202	3.076E-201	0.45517561	0.058	0.251	4.728E-197	T Cells
SGSH3	3.106E-201	0.67765241	0.069	0.272	4.775E-197	T Cells
BLOC1S51	4.258E-201	0.70562376	0.066	0.266	6.545E-197	T Cells
NME61	5.648E-201	0.64751069	0.061	0.258	8.681E-197	T Cells
GFER2	6.328E-201	0.61522505	0.062	0.26	9.726E-197	T Cells
RWDD42	9.031E-201	0.61957181	0.055	0.248	1.388E-196	T Cells
IL18BP3	9.785E-201	0.33402809	0.063	0.26	1.504E-196	T Cells
ERC12	1.082E-200	1.0412395	0.104	0.33	1.663E-196	T Cells
SURF6	1.085E-200	0.4353783	0.05	0.239	1.668E-196	T Cells
MFN12	1.122E-200	0.85871785	0.08	0.29	1.724E-196	T Cells
MAPK82	1.129E-200	1.03993598	0.091	0.309	1.735E-196	T Cells
TMEM433	1.618E-200	1.30936535	0.249	0.601	2.486E-196	T Cells
NOL111	1.835E-200	1.00499394	0.079	0.287	2.821E-196	T Cells
ERMP1	1.884E-200	1.37611006	0.154	0.419	2.895E-196	T Cells
TSPYL11	1.988E-200	1.35641358	0.209	0.519	3.056E-196	T Cells
FBXW4	3.578E-200	1.45467625	0.23	0.564	5.499E-196	T Cells
ACTN4	3.885E-200	0.54749303	0.394	0.91	5.972E-196	T Cells
DUSP63	4.614E-200	0.47244242	0.081	0.291	7.092E-196	T Cells
RABL31	5.107E-200	0.63076735	0.061	0.258	7.85E-196	T Cells
FASTKD52	6.986E-200	0.82753413	0.073	0.278	1.074E-195	T Cells
EIF3E1	7.85E-200	1.03074557	0.092	0.311	1.207E-195	T Cells
MT-ATP65	1.323E-199	2.75744152	0.496	0.458	2.033E-195	T Cells
OBI11	1.385E-199	0.37813303	0.046	0.231	2.128E-195	T Cells
RABIF1	2.057E-199	1.15779655	0.105	0.332	3.161E-195	T Cells
LAT1	2.238E-199	3.82915848	0.273	0.154	3.441E-195	T Cells
KCTD22	2.589E-199	0.7474691	0.067	0.266	3.979E-195	T Cells
RC3H11	3.873E-199	1.23615669	0.152	0.414	5.953E-195	T Cells
FAM219A2	3.916E-199	0.50273545	0.058	0.251	6.019E-195	T Cells
DAZAP23	4.065E-199	0.76995282	0.37	0.859	6.248E-195	T Cells
TOP3A1	4.682E-199	0.43613787	0.051	0.24	7.196E-195	T Cells
INTS131	5.538E-199	0.91621143	0.078	0.287	8.513E-195	T Cells
UBALD11	5.667E-199	0.40483737	0.055	0.246	8.711E-195	T Cells
LIG41	1.042E-198	0.60010145	0.058	0.251	1.602E-194	T Cells
ING42	1.222E-198	0.85663652	0.076	0.281	1.878E-194	T Cells
ZNF2742	2.29E-198	0.84034025	0.07	0.27	3.519E-194	T Cells
CENPP1	2.88E-198	0.19700777	0.05	0.235	4.427E-194	T Cells
MPHOSPH81	3.386E-198	1.45140417	0.242	0.584	5.204E-194	T Cells
PHRF1	6.569E-198	1.2654116	0.126	0.37	1.01E-193	T Cells
PLA2G12A1	7.444E-198	0.29716602	0.045	0.228	1.144E-193	T Cells
CNOT42	7.824E-198	1.01554337	0.089	0.304	1.203E-193	T Cells
TCF33	7.836E-198	1.01024487	0.094	0.312	1.205E-193	T Cells
TBC1D171	1.033E-197	1.15152487	0.108	0.336	1.588E-193	T Cells

ITGB1BP1	1.326E-197	1.45015755	0.162	0.434	2.039E-193	T Cells
ZBTB342	1.532E-197	0.2037699	0.045	0.228	2.355E-193	T Cells
RHOF3	1.842E-197	4.43397435	0.204	0.095	2.831E-193	T Cells
CNTROB2	2.024E-197	0.29126187	0.049	0.236	3.111E-193	T Cells
TEDC13	2.545E-197	0.67095207	0.083	0.293	3.911E-193	T Cells
CSTF12	2.964E-197	0.92461973	0.079	0.287	4.556E-193	T Cells
SATB14	3.933E-197	4.48761372	0.199	0.091	6.046E-193	T Cells
ZFYVE271	4.717E-197	1.07429125	0.114	0.345	7.251E-193	T Cells
CWC221	6.218E-197	0.88654986	0.075	0.28	9.558E-193	T Cells
DDX501	6.553E-197	1.35292427	0.15	0.41	1.007E-192	T Cells
PCNA	8.134E-197	0.20202653	0.054	0.241	1.25E-192	T Cells
FAM219B1	8.608E-197	1.1429195	0.124	0.364	1.323E-192	T Cells
MTF22	9.378E-197	1.12375263	0.108	0.335	1.441E-192	T Cells
CELF24	1.242E-196	2.02331172	0.631	0.869	1.909E-192	T Cells
PCGF51	1.736E-196	1.4771628	0.228	0.554	2.668E-192	T Cells
PANK32	2.89E-196	1.37484122	0.176	0.454	4.443E-192	T Cells
EHD11	3.3E-196	1.07440606	0.21	0.516	5.073E-192	T Cells
ENDOG	3.727E-196	0.75482607	0.072	0.273	5.728E-192	T Cells
CASP61	4.057E-196	0.48011451	0.048	0.233	6.236E-192	T Cells
FBL	5.323E-196	1.39746627	0.16	0.426	8.181E-192	T Cells
SMPD11	6.528E-196	1.02575197	0.1	0.323	1.003E-191	T Cells
SMARCD12	7.071E-196	1.15822585	0.107	0.334	1.087E-191	T Cells
COA51	7.267E-196	0.50667337	0.05	0.236	1.117E-191	T Cells
METTL171	7.326E-196	1.27953657	0.147	0.403	1.126E-191	T Cells
TRGJP2	8.65E-196	0.61572145	0.057	0.247	1.33E-191	T Cells
PRKACB	8.803E-196	1.32055398	0.247	0.588	1.353E-191	T Cells
MBD11	9.402E-196	0.75058358	0.071	0.27	1.445E-191	T Cells
ZDHHC162	9.474E-196	0.9890331	0.092	0.307	1.456E-191	T Cells
KDM3A2	1.37E-195	1.30099018	0.143	0.396	2.106E-191	T Cells
HEATR6	1.731E-195	0.71211886	0.056	0.248	2.661E-191	T Cells
TNFSF103	1.948E-195	1.18112837	0.222	0.539	2.995E-191	T Cells
MKLN11	2.066E-195	1.44066301	0.193	0.485	3.175E-191	T Cells
STAMBPL13	2.188E-195	5.68501028	0.06	0.012	3.363E-191	T Cells
ARRDC33	3.925E-195	1.11775847	0.144	0.397	6.034E-191	T Cells
NOC3L2	6.156E-195	0.60275097	0.055	0.243	9.462E-191	T Cells
PPCDC2	6.496E-195	0.4828634	0.059	0.251	9.985E-191	T Cells
WAC1	6.556E-195	1.32622609	0.13	0.374	1.008E-190	T Cells
PROSER12	7.43E-195	1.16104032	0.098	0.317	1.142E-190	T Cells
BTBD102	9.147E-195	0.68026334	0.059	0.25	1.406E-190	T Cells
SLTM1	1.102E-194	1.26040779	0.298	0.693	1.694E-190	T Cells
PPP1R371	1.323E-194	0.33136502	0.053	0.238	2.034E-190	T Cells
LENG12	1.464E-194	0.97893612	0.087	0.298	2.251E-190	T Cells
LDLRAD42	1.56E-194	1.25682177	0.172	0.447	2.398E-190	T Cells
CNOT111	1.928E-194	1.39877713	0.169	0.443	2.964E-190	T Cells
ADK2	2.303E-194	1.17240797	0.109	0.337	3.54E-190	T Cells
INPP4A1	2.304E-194	1.33624784	0.271	0.64	3.541E-190	T Cells
WDR48	3.149E-194	0.97940355	0.089	0.3	4.84E-190	T Cells
GPATCH22	3.808E-194	0.89880074	0.084	0.294	5.853E-190	T Cells
CPNE12	3.875E-194	1.1271767	0.311	0.726	5.957E-190	T Cells
WDR32	4.283E-194	0.84701886	0.07	0.268	6.583E-190	T Cells
GSDMB3	4.88E-194	5.23132174	0.078	0.019	7.501E-190	T Cells
RTKN2	5.377E-194	7.38424014	0.026	0.002	8.266E-190	T Cells

NOL122	5.815E-194	0.99646313	0.091	0.304	8.938E-190	T Cells
NCAPD31	6.699E-194	1.064218	0.098	0.317	1.03E-189	T Cells
RAP1A	7.754E-194	0.5232499	0.396	0.909	1.192E-189	T Cells
ELP11	8.596E-194	1.084137	0.094	0.31	1.321E-189	T Cells
TTC221	2.123E-193	6.39405367	0.026	0.002	3.263E-189	T Cells
PHTF11	2.481E-193	0.84757985	0.079	0.283	3.814E-189	T Cells
CD300A5	3.808E-193	1.19636348	0.129	0.369	5.853E-189	T Cells
POLL1	5.537E-193	0.44591153	0.057	0.245	8.511E-189	T Cells
SLC9A82	5.628E-193	0.92256073	0.087	0.296	8.651E-189	T Cells
CIZ12	5.752E-193	0.81438292	0.074	0.276	8.842E-189	T Cells
ZNF3941	5.859E-193	1.26373784	0.142	0.393	9.006E-189	T Cells
NARS21	6.293E-193	0.4920932	0.05	0.233	9.673E-189	T Cells
CAD1	7.137E-193	0.82605965	0.076	0.278	1.097E-188	T Cells
TMEM1682	7.887E-193	1.0887889	0.113	0.34	1.212E-188	T Cells
HIP11	1.001E-192	0.20356736	0.048	0.229	1.539E-188	T Cells
MED13L5	1.195E-192	1.35240507	0.15	0.405	1.837E-188	T Cells
TRAK2	1.431E-192	1.26744237	0.121	0.355	2.199E-188	T Cells
NCBP12	2.678E-192	1.29823207	0.116	0.346	4.116E-188	T Cells
CCS1	2.793E-192	0.41042154	0.054	0.238	4.294E-188	T Cells
RARS21	3.173E-192	0.96666471	0.084	0.292	4.877E-188	T Cells
NHLRC22	4.007E-192	1.21021203	0.114	0.344	6.159E-188	T Cells
KCTD131	6.554E-192	1.29550208	0.135	0.38	1.007E-187	T Cells
KIN1	1.325E-191	0.3529292	0.052	0.235	2.036E-187	T Cells
MORC33	1.537E-191	1.27489697	0.319	0.735	2.363E-187	T Cells
NFKBID4	1.663E-191	0.49755097	0.063	0.255	2.556E-187	T Cells
RANBP91	1.668E-191	1.48166877	0.212	0.52	2.564E-187	T Cells
R3HCC11	1.988E-191	0.63240184	0.059	0.248	3.056E-187	T Cells
NIPSNAP11	2.112E-191	0.59456595	0.064	0.255	3.247E-187	T Cells
ZBTB441	2.736E-191	1.50119487	0.215	0.524	4.205E-187	T Cells
PAN33	2.738E-191	1.34380562	0.172	0.447	4.208E-187	T Cells
GOSR21	3.477E-191	1.44163654	0.163	0.429	5.344E-187	T Cells
TIGD5	5.845E-191	0.76206327	0.062	0.252	8.985E-187	T Cells
DHX351	7.113E-191	0.11459237	0.044	0.221	1.093E-186	T Cells
RAE12	1.165E-190	0.94475796	0.077	0.28	1.79E-186	T Cells
CRBN1	1.534E-190	1.39058445	0.132	0.373	2.358E-186	T Cells
POLR3A1	2.108E-190	0.7315398	0.066	0.259	3.24E-186	T Cells
ZNF689	2.741E-190	0.21966877	0.045	0.223	4.213E-186	T Cells
EXOSC61	2.951E-190	1.16725593	0.115	0.344	4.536E-186	T Cells
ATRX	3.927E-190	1.42222517	0.212	0.519	6.036E-186	T Cells
RPAP12	4.304E-190	0.64749028	0.065	0.256	6.616E-186	T Cells
AKAP17A4	1.123E-189	1.29918518	0.2	0.496	1.726E-185	T Cells
GIMAP2	1.202E-189	1.48929511	0.221	0.536	1.847E-185	T Cells
ERMARD2	1.606E-189	0.7496249	0.068	0.262	2.469E-185	T Cells
RAD12	1.936E-189	0.78038817	0.063	0.254	2.976E-185	T Cells
TTC272	2.336E-189	0.87827349	0.077	0.276	3.59E-185	T Cells
GCDH	2.724E-189	0.32296137	0.042	0.218	4.187E-185	T Cells
CDC272	3.231E-189	0.75390688	0.065	0.257	4.967E-185	T Cells
TMF1	3.559E-189	1.50501877	0.211	0.519	5.471E-185	T Cells
PIM13	6.611E-189	2.68158216	0.534	0.565	1.016E-184	T Cells
ZUP11	1.235E-188	0.68260823	0.06	0.248	1.899E-184	T Cells
EDC31	1.293E-188	1.04139778	0.09	0.299	1.987E-184	T Cells
OPA31	1.351E-188	0.10479215	0.043	0.218	2.076E-184	T Cells

PDS5A1	1.466E-188	1.2755517	0.115	0.343	2.254E-184	T Cells
MVD	1.518E-188	0.98523327	0.125	0.358	2.333E-184	T Cells
VKORC1L12	1.628E-188	0.55086721	0.052	0.234	2.502E-184	T Cells
TOB21	1.977E-188	1.02077586	0.104	0.322	3.038E-184	T Cells
SPSB21	2.923E-188	0.53633839	0.063	0.252	4.493E-184	T Cells
OXCT12	3.439E-188	5.44900142	0.066	0.015	5.287E-184	T Cells
TBC1D22B1	3.451E-188	0.84312523	0.069	0.262	5.305E-184	T Cells
LRRC402	3.648E-188	1.19888886	0.098	0.314	5.607E-184	T Cells
BAG3	3.893E-188	0.14205	0.043	0.217	5.985E-184	T Cells
CD841	4.867E-188	1.31165268	0.148	0.399	7.481E-184	T Cells
ARHGAP212	5.406E-188	0.12070816	0.039	0.211	8.309E-184	T Cells
TMA7	5.432E-188	1.42580676	0.182	0.461	8.35E-184	T Cells
PPP2R1B1	5.544E-188	1.35593177	0.148	0.398	8.522E-184	T Cells
KLHL72	8.565E-188	1.03939349	0.078	0.278	1.317E-183	T Cells
FBXO211	9.295E-188	0.94472589	0.073	0.27	1.429E-183	T Cells
LAS1L	9.815E-188	0.9948873	0.082	0.285	1.509E-183	T Cells
USPL12	1.026E-187	1.16014476	0.102	0.321	1.578E-183	T Cells
NSD13	1.041E-187	1.41409494	0.252	0.594	1.6E-183	T Cells
SUGP11	1.093E-187	0.66103404	0.059	0.245	1.68E-183	T Cells
WDR472	1.297E-187	0.79671196	0.059	0.245	1.994E-183	T Cells
MTMR11	1.885E-187	1.255863	0.114	0.34	2.897E-183	T Cells
IPO51	1.954E-187	0.42318833	0.052	0.233	3.004E-183	T Cells
CDK82	2.954E-187	0.69758844	0.057	0.243	4.541E-183	T Cells
FSD1L2	4.775E-187	0.82924885	0.081	0.281	7.339E-183	T Cells
SRSF8	5.383E-187	1.45521863	0.166	0.433	8.275E-183	T Cells
UMPS2	6.323E-187	0.57854969	0.055	0.237	9.72E-183	T Cells
ANKRD112	8.261E-187	1.26715181	0.315	0.73	1.27E-182	T Cells
JAG2	9.683E-187	5.36966064	0.062	0.013	1.488E-182	T Cells
UNG	1.277E-186	0.20592106	0.044	0.218	1.962E-182	T Cells
TBCEL1	1.852E-186	0.28968879	0.046	0.223	2.847E-182	T Cells
ZZEF11	2.006E-186	1.39428667	0.241	0.57	3.083E-182	T Cells
EXOSC51	2.286E-186	0.19024726	0.038	0.208	3.514E-182	T Cells
YEATS42	2.439E-186	0.40428451	0.051	0.231	3.749E-182	T Cells
TANGO61	3.275E-186	1.17792007	0.101	0.317	5.033E-182	T Cells
DNAJC251	3.49E-186	0.52267717	0.047	0.224	5.364E-182	T Cells
AARS1	3.666E-186	1.30072106	0.095	0.306	5.634E-182	T Cells
MMAA1	5.436E-186	0.22202301	0.045	0.219	8.356E-182	T Cells
CRIP24	5.676E-186	4.66865676	0.054	0.01	8.725E-182	T Cells
CDK123	8.23E-186	1.28698211	0.139	0.383	1.265E-181	T Cells
RIOX11	9.192E-186	1.49633521	0.133	0.373	1.413E-181	T Cells
ARID4A1	1.015E-185	1.21945587	0.111	0.332	1.56E-181	T Cells
MED19	1.557E-185	0.22356432	0.039	0.211	2.393E-181	T Cells
FTSJ12	1.641E-185	0.70588596	0.061	0.247	2.522E-181	T Cells
NUDT15	1.664E-185	0.48753974	0.046	0.222	2.558E-181	T Cells
METTL2A2	2.368E-185	0.7328983	0.058	0.243	3.64E-181	T Cells
PNN2	2.723E-185	1.28325111	0.138	0.38	4.186E-181	T Cells
RASSF52	3.945E-185	0.56925084	0.394	0.894	6.064E-181	T Cells
GALNT101	4.83E-185	1.35615599	0.161	0.422	7.424E-181	T Cells
HSDL1	8.118E-185	0.87793939	0.065	0.254	1.248E-180	T Cells
MAX2	9.027E-185	1.15275105	0.341	0.785	1.388E-180	T Cells
ACOT82	1.208E-184	0.38452047	0.048	0.223	1.857E-180	T Cells
UTP41	1.765E-184	0.47579652	0.048	0.225	2.713E-180	T Cells

DNAJC22	1.774E-184	0.94300544	0.083	0.284	2.727E-180	T Cells
MSH21	1.822E-184	1.05170064	0.08	0.279	2.801E-180	T Cells
RGP11	2.025E-184	1.13637316	0.102	0.316	3.113E-180	T Cells
TWISTNB2	3.282E-184	0.6174069	0.058	0.242	5.045E-180	T Cells
MTERF32	3.56E-184	0.73759107	0.059	0.243	5.472E-180	T Cells
OARD11	3.873E-184	1.33344184	0.126	0.357	5.953E-180	T Cells
TANK1	4.363E-184	1.40521836	0.279	0.648	6.706E-180	T Cells
ACOX31	7.082E-184	0.50355679	0.052	0.231	1.089E-179	T Cells
KRI1	7.524E-184	1.4861138	0.181	0.456	1.157E-179	T Cells
TOPBP1	8.104E-184	0.82878546	0.072	0.265	1.246E-179	T Cells
MMS191	9.446E-184	1.20699243	0.121	0.347	1.452E-179	T Cells
MIB2	9.631E-184	0.64355274	0.057	0.24	1.48E-179	T Cells
MED172	1.001E-183	1.48341014	0.162	0.425	1.539E-179	T Cells
SCO11	1.274E-183	0.39835273	0.053	0.231	1.958E-179	T Cells
WDR33	1.322E-183	1.47353552	0.245	0.579	2.032E-179	T Cells
ZNF3972	1.425E-183	1.00004229	0.079	0.278	2.191E-179	T Cells
RBM411	1.747E-183	0.71470237	0.065	0.252	2.685E-179	T Cells
MARK32	2.286E-183	1.44744284	0.191	0.475	3.513E-179	T Cells
ARHGEF61	2.714E-183	1.45680179	0.229	0.545	4.172E-179	T Cells
TNIP14	3.557E-183	1.3786961	0.207	0.502	5.468E-179	T Cells
GPBP12	7.405E-183	1.46539698	0.238	0.561	1.138E-178	T Cells
TFDP21	7.463E-183	0.52821316	0.049	0.226	1.147E-178	T Cells
UMAD11	8.829E-183	0.94707628	0.072	0.264	1.357E-178	T Cells
NOSIP	1.119E-182	1.27796358	0.118	0.346	1.72E-178	T Cells
NUDCD1	1.128E-182	0.71194005	0.059	0.242	1.733E-178	T Cells
KMT5B3	1.575E-182	1.46625575	0.232	0.55	2.421E-178	T Cells
BRF2	1.867E-182	0.83846444	0.067	0.255	2.87E-178	T Cells
KDM5A1	3.531E-182	1.47021096	0.231	0.548	5.427E-178	T Cells
PIGU1	3.542E-182	0.42121176	0.048	0.222	5.445E-178	T Cells
SARAF	5.543E-182	0.68708332	0.391	0.896	8.52E-178	T Cells
WDR702	6.492E-182	0.91778264	0.075	0.27	9.978E-178	T Cells
RNF1382	9.57E-182	1.35239175	0.139	0.38	1.471E-177	T Cells
POLA21	1.058E-181	0.43260431	0.047	0.221	1.626E-177	T Cells
CSE1L1	1.169E-181	0.61934954	0.049	0.224	1.797E-177	T Cells
HEXD3	1.262E-181	0.80113118	0.073	0.266	1.94E-177	T Cells
ADRB23	1.687E-181	1.31948999	0.185	0.46	2.593E-177	T Cells
SYNJ12	2.071E-181	0.5676003	0.055	0.235	3.183E-177	T Cells
TOMM342	2.412E-181	0.17841272	0.036	0.202	3.707E-177	T Cells
HEATR11	2.708E-181	1.49916653	0.133	0.368	4.162E-177	T Cells
ALDH5A11	4.995E-181	0.11304863	0.036	0.201	7.678E-177	T Cells
ZNF623	5.36E-181	1.16188156	0.098	0.309	8.24E-177	T Cells
ZBTB171	6.018E-181	0.97991296	0.083	0.281	9.25E-177	T Cells
SARS	7.535E-181	1.31719362	0.096	0.305	1.158E-176	T Cells
RNF1252	7.976E-181	4.21612955	0.252	0.141	1.226E-176	T Cells
WNT9A1	8.322E-181	6.86125111	0.013	0.001	1.279E-176	T Cells
RNGTT2	8.608E-181	1.430698	0.169	0.432	1.323E-176	T Cells
SRSF71	1.012E-180	1.44510714	0.178	0.449	1.556E-176	T Cells
XYLT13	1.625E-180	5.30984111	0.059	0.012	2.498E-176	T Cells
NAA301	1.698E-180	1.34057679	0.11	0.328	2.611E-176	T Cells
BTG31	1.926E-180	0.87458645	0.077	0.271	2.96E-176	T Cells
ZNF3171	2.185E-180	0.74827446	0.063	0.248	3.359E-176	T Cells
RBCK12	2.453E-180	1.25082185	0.124	0.354	3.771E-176	T Cells

DOLK	3.831E-180	0.32818068	0.04	0.208	5.889E-176	T Cells
BMF3	3.894E-180	0.32162334	0.05	0.224	5.985E-176	T Cells
ZNF7401	4.628E-180	0.82237745	0.076	0.268	7.113E-176	T Cells
CASP31	4.9E-180	1.38307464	0.141	0.383	7.531E-176	T Cells
VSIR4	6.777E-180	1.02787263	0.114	0.332	1.042E-175	T Cells
ZSCAN182	7.768E-180	0.13198072	0.041	0.209	1.194E-175	T Cells
SNIP11	7.864E-180	1.21716036	0.098	0.307	1.209E-175	T Cells
ATXN7L32	1.324E-179	0.90048302	0.074	0.265	2.034E-175	T Cells
AP2B1	1.56E-179	1.04447475	0.087	0.287	2.397E-175	T Cells
THAP122	2.069E-179	1.30105119	0.118	0.341	3.18E-175	T Cells
ISCA1	2.243E-179	1.01356992	0.069	0.258	3.448E-175	T Cells
CPSF61	2.272E-179	1.486906	0.144	0.388	3.492E-175	T Cells
ACAP32	2.493E-179	1.05165278	0.09	0.291	3.831E-175	T Cells
RNF1691	2.654E-179	1.54031249	0.195	0.48	4.079E-175	T Cells
MIER12	4.633E-179	1.46591143	0.272	0.628	7.122E-175	T Cells
RAB282	8.119E-179	1.19377438	0.09	0.294	1.248E-174	T Cells
TTLL33	8.947E-179	1.01413127	0.114	0.333	1.375E-174	T Cells
AREL12	1.187E-178	0.81065286	0.066	0.251	1.825E-174	T Cells
CCDC137	1.227E-178	0.4031696	0.049	0.222	1.886E-174	T Cells
LMBR1L2	1.559E-178	1.10859805	0.1	0.31	2.396E-174	T Cells
SLC26A2	1.994E-178	1.08620572	0.075	0.267	3.064E-174	T Cells
DHRS121	2.25E-178	0.9803911	0.076	0.268	3.458E-174	T Cells
ZNF4262	2.282E-178	0.76505375	0.062	0.244	3.507E-174	T Cells
PIGG1	2.355E-178	1.02207937	0.091	0.294	3.62E-174	T Cells
PLEKHM32	2.541E-178	0.46696697	0.053	0.228	3.906E-174	T Cells
KLC1	2.677E-178	1.51351984	0.244	0.575	4.115E-174	T Cells
NOL92	2.962E-178	1.27897939	0.096	0.304	4.552E-174	T Cells
P2RY112	3.944E-178	0.89952796	0.085	0.283	6.062E-174	T Cells
AASDH2	4.245E-178	0.34409603	0.05	0.223	6.525E-174	T Cells
PCSK71	4.57E-178	1.45961129	0.243	0.575	7.025E-174	T Cells
NADK21	5.209E-178	0.51192878	0.049	0.222	8.007E-174	T Cells
TSPAN17	6.151E-178	0.92548654	0.077	0.268	9.454E-174	T Cells
COG1	7.385E-178	1.32604981	0.11	0.327	1.135E-173	T Cells
THOC52	7.97E-178	1.10894258	0.094	0.297	1.225E-173	T Cells
RBM64	9.841E-178	1.33054135	0.219	0.52	1.513E-173	T Cells
TNPO22	1.008E-177	1.04445026	0.094	0.296	1.55E-173	T Cells
CEP851	1.238E-177	0.93048587	0.077	0.268	1.904E-173	T Cells
ZNF4902	1.349E-177	0.62576726	0.055	0.232	2.073E-173	T Cells
GOT12	1.43E-177	0.23135222	0.041	0.206	2.199E-173	T Cells
FZR1	1.625E-177	1.51522814	0.193	0.472	2.497E-173	T Cells
BRD81	2.155E-177	0.90374168	0.074	0.264	3.313E-173	T Cells
TUT11	2.489E-177	0.48061453	0.049	0.222	3.826E-173	T Cells
GEMIN73	5.057E-177	0.78402955	0.068	0.253	7.773E-173	T Cells
DENND1B2	6.321E-177	1.3614093	0.126	0.353	9.716E-173	T Cells
MYO15B5	8.351E-177	0.34793989	0.06	0.24	1.284E-172	T Cells
SLC25A53	1.242E-176	6.7295011	0.023	0.002	1.908E-172	T Cells
NAXD	1.432E-176	0.10290414	0.035	0.198	2.201E-172	T Cells
PCNX32	1.936E-176	1.15391842	0.097	0.303	2.976E-172	T Cells
GALNT71	2.177E-176	0.77177406	0.068	0.251	3.346E-172	T Cells
RUSC1	2.31E-176	0.66283962	0.058	0.236	3.551E-172	T Cells
FBXL202	3.656E-176	0.95158402	0.073	0.263	5.62E-172	T Cells
ABHD131	3.821E-176	1.09673544	0.087	0.285	5.874E-172	T Cells

DOP1B3	4.875E-176	0.50460127	0.055	0.23	7.494E-172	T Cells
FASTKD13	6.551E-176	0.53080044	0.053	0.227	1.007E-171	T Cells
ULK12	9.572E-176	1.05264818	0.096	0.299	1.471E-171	T Cells
SLC38A93	1.226E-175	0.72899503	0.059	0.238	1.885E-171	T Cells
SNAPC12	1.272E-175	0.2742027	0.047	0.216	1.955E-171	T Cells
MGA	1.295E-175	1.38488683	0.125	0.353	1.991E-171	T Cells
PYROXD11	1.39E-175	1.00238306	0.074	0.262	2.136E-171	T Cells
PPP5C	1.662E-175	0.73098551	0.062	0.242	2.554E-171	T Cells
ANKRD282	2.142E-175	1.4319221	0.146	0.388	3.293E-171	T Cells
MPHOSPH102	2.638E-175	1.32182287	0.109	0.324	4.055E-171	T Cells
GID41	2.725E-175	0.67059114	0.05	0.222	4.188E-171	T Cells
HCFC22	2.819E-175	1.14646998	0.091	0.292	4.333E-171	T Cells
ANAPC161	2.836E-175	1.32352875	0.31	0.706	4.359E-171	T Cells
ZNF3222	2.935E-175	0.69050858	0.055	0.231	4.512E-171	T Cells
KBTBD4	5.647E-175	0.67376765	0.059	0.237	8.68E-171	T Cells
SMYD52	5.995E-175	0.79506383	0.062	0.24	9.214E-171	T Cells
CLCN62	9.288E-175	0.33289872	0.051	0.222	1.428E-170	T Cells
TIRAP1	9.291E-175	0.181727	0.041	0.205	1.428E-170	T Cells
INSIG21	9.876E-175	1.09248129	0.077	0.269	1.518E-170	T Cells
PANX1	1.045E-174	0.62252216	0.049	0.22	1.606E-170	T Cells
GRAMD1A3	1.25E-174	1.43010105	0.169	0.428	1.922E-170	T Cells
PLEC3	1.296E-174	0.65565077	0.396	0.893	1.992E-170	T Cells
TMOD23	1.418E-174	0.31822082	0.041	0.206	2.18E-170	T Cells
ERO1A	1.577E-174	0.94965558	0.373	0.844	2.424E-170	T Cells
TRIP111	1.594E-174	1.20601125	0.09	0.29	2.45E-170	T Cells
NPM3	2.604E-174	0.87995151	0.064	0.246	4.003E-170	T Cells
LRRC141	2.68E-174	1.00955784	0.079	0.27	4.12E-170	T Cells
TRIM221	2.779E-174	0.36634086	0.424	0.938	4.272E-170	T Cells
YTHDC11	2.889E-174	1.51536088	0.226	0.533	4.441E-170	T Cells
SLC41A31	3.079E-174	0.99382121	0.077	0.266	4.732E-170	T Cells
USP362	3.802E-174	1.18974351	0.138	0.372	5.844E-170	T Cells
MAVS	3.847E-174	1.28664265	0.15	0.392	5.913E-170	T Cells
PHF31	4.165E-174	1.4495671	0.25	0.581	6.402E-170	T Cells
ABCD42	4.906E-174	1.07322688	0.079	0.27	7.541E-170	T Cells
CTU21	5.248E-174	0.57263382	0.055	0.228	8.067E-170	T Cells
CFAP410	5.601E-174	0.41588785	0.043	0.209	8.609E-170	T Cells
DCPS1	6.622E-174	0.94521375	0.073	0.261	1.018E-169	T Cells
SLC1A43	7.253E-174	0.40033339	0.048	0.216	1.115E-169	T Cells
GLOD4	1.289E-173	0.92873824	0.065	0.246	1.982E-169	T Cells
METTL31	1.29E-173	1.51402311	0.174	0.438	1.983E-169	T Cells
DOCK54	1.686E-173	1.00859528	0.097	0.3	2.592E-169	T Cells
INSL3	2.07E-173	7.58743663	0.01	0	3.182E-169	T Cells
CCP110	2.442E-173	0.59829038	0.051	0.223	3.753E-169	T Cells
RAD211	2.653E-173	1.5388584	0.157	0.407	4.078E-169	T Cells
BMI11	2.786E-173	1.4278156	0.129	0.356	4.283E-169	T Cells
PRKAG22	4.775E-173	0.84554177	0.062	0.24	7.339E-169	T Cells
CD2AP3	5.083E-173	1.31584887	0.106	0.317	7.812E-169	T Cells
NAIF11	5.319E-173	0.28645364	0.047	0.215	8.176E-169	T Cells
IFT522	5.538E-173	0.39995226	0.045	0.211	8.512E-169	T Cells
IRGQ1	5.679E-173	0.3726194	0.047	0.215	8.729E-169	T Cells
ZDHHC181	6.303E-173	1.43008383	0.173	0.435	9.688E-169	T Cells
DNAJC14	1.336E-172	1.36747164	0.143	0.377	2.054E-168	T Cells

RIMKLB2	1.4E-172	0.19645763	0.042	0.205	2.152E-168	T Cells
GNE1	2.101E-172	0.5352424	0.049	0.218	3.229E-168	T Cells
CRYZ2	2.131E-172	0.92851438	0.079	0.268	3.276E-168	T Cells
ACTR61	2.422E-172	1.25667802	0.081	0.274	3.723E-168	T Cells
ARHGAP30	3.074E-172	0.31888214	0.42	0.945	4.725E-168	T Cells
ZNF576	4.782E-172	0.28262832	0.039	0.201	7.35E-168	T Cells
SLC25A171	5.09E-172	0.70619685	0.057	0.231	7.824E-168	T Cells
MIPEP	7.302E-172	0.14321905	0.041	0.202	1.122E-167	T Cells
RANBP101	7.54E-172	0.58073505	0.059	0.233	1.159E-167	T Cells
CCDC591	8.258E-172	1.44916421	0.111	0.325	1.269E-167	T Cells
TMEM2421	8.796E-172	0.92749002	0.067	0.248	1.352E-167	T Cells
PEX51	1.028E-171	0.10970291	0.033	0.19	1.58E-167	T Cells
TIA12	1.218E-171	1.42424704	0.249	0.577	1.872E-167	T Cells
NUP1551	1.445E-171	1.01666657	0.068	0.251	2.221E-167	T Cells
KLHL152	1.56E-171	0.98785294	0.072	0.256	2.398E-167	T Cells
KDM2A3	1.899E-171	0.95097399	0.37	0.836	2.919E-167	T Cells
ELP41	2.142E-171	0.675618	0.053	0.225	3.292E-167	T Cells
EIF2B21	2.539E-171	0.28639709	0.037	0.197	3.902E-167	T Cells
TRPM71	3.388E-171	1.51532147	0.194	0.471	5.207E-167	T Cells
TBP1	5.012E-171	0.91300124	0.068	0.248	7.704E-167	T Cells
KCTD181	5.836E-171	0.58140442	0.05	0.218	8.97E-167	T Cells
GPD1L1	6.334E-171	1.27874021	0.1	0.304	9.736E-167	T Cells
KDM6A1	9.261E-171	1.2970242	0.116	0.332	1.423E-166	T Cells
ESS22	1.239E-170	0.47868229	0.046	0.212	1.904E-166	T Cells
NIPAL31	2.33E-170	1.08488964	0.098	0.298	3.582E-166	T Cells
THOC21	2.331E-170	1.13563115	0.087	0.281	3.583E-166	T Cells
MAP3K42	2.334E-170	1.46930793	0.14	0.376	3.588E-166	T Cells
SFSWAP2	2.413E-170	1.30934094	0.114	0.327	3.709E-166	T Cells
TRIM561	2.881E-170	1.11236394	0.096	0.296	4.429E-166	T Cells
USP471	3.54E-170	1.35970821	0.119	0.337	5.442E-166	T Cells
METTL222	4.111E-170	0.61753981	0.053	0.222	6.32E-166	T Cells
SLC43A12	4.707E-170	6.61605203	0.022	0.002	7.235E-166	T Cells
PROK21	5.212E-170	4.46210627	0.019	0.001	8.011E-166	T Cells
HMGCS11	5.228E-170	1.27869036	0.156	0.4	8.036E-166	T Cells
ARL14EP	8.208E-170	1.27779154	0.095	0.294	1.262E-165	T Cells
RCBTB12	1.226E-169	1.34032077	0.112	0.324	1.885E-165	T Cells
CASZ11	1.384E-169	0.33152406	0.044	0.208	2.127E-165	T Cells
MAP2K52	1.478E-169	0.61471552	0.053	0.222	2.272E-165	T Cells
ABI32	1.608E-169	0.62563523	0.063	0.239	2.472E-165	T Cells
FOCAD2	1.727E-169	0.21623118	0.041	0.201	2.654E-165	T Cells
WDFY25	1.959E-169	1.22229851	0.159	0.402	3.012E-165	T Cells
H3F3B4	2.314E-169	1.0529582	0.084	0.275	3.557E-165	T Cells
AHI12	2.444E-169	0.53316022	0.055	0.225	3.757E-165	T Cells
MAPKBP12	2.626E-169	0.43006876	0.045	0.21	4.037E-165	T Cells
SPRYD7	3.792E-169	0.39472493	0.039	0.197	5.829E-165	T Cells
RSBN1L1	4.241E-169	1.52783599	0.182	0.447	6.518E-165	T Cells
CEP1621	4.457E-169	0.42197503	0.05	0.217	6.851E-165	T Cells
SLC10A72	5.376E-169	0.62263657	0.06	0.233	8.264E-165	T Cells
NEK4	7.204E-169	0.94949617	0.061	0.236	1.107E-164	T Cells
FIP1L1	7.972E-169	1.48571372	0.145	0.384	1.225E-164	T Cells
CBR41	8.825E-169	0.32061494	0.043	0.205	1.356E-164	T Cells
GLT8D11	1.181E-168	0.86626777	0.057	0.23	1.815E-164	T Cells

RFWD3	1.318E-168	1.14186962	0.085	0.278	2.025E-164	T Cells
PRR143	1.346E-168	1.49053805	0.174	0.433	2.069E-164	T Cells
SPECC1L	1.49E-168	1.47232491	0.139	0.369	2.29E-164	T Cells
BORCS51	1.756E-168	1.31755776	0.107	0.315	2.699E-164	T Cells
KDM4C3	1.881E-168	1.37923483	0.266	0.608	2.891E-164	T Cells
POGLUT1	2.234E-168	1.01015597	0.081	0.269	3.434E-164	T Cells
HNRNPA2B13	2.348E-168	0.45289261	0.419	0.938	3.609E-164	T Cells
GMDS2	2.95E-168	0.91456249	0.075	0.259	4.535E-164	T Cells
SMIM10L11	3.075E-168	0.82975391	0.051	0.22	4.726E-164	T Cells
SAP303	3.456E-168	0.98369876	0.081	0.269	5.313E-164	T Cells
INPP13	5.467E-168	0.25845969	0.043	0.204	8.403E-164	T Cells
RSF11	5.574E-168	1.40233478	0.12	0.336	8.568E-164	T Cells
KDM2B3	8.3E-168	1.40753141	0.161	0.409	1.276E-163	T Cells
EXO51	8.511E-168	0.79760059	0.053	0.223	1.308E-163	T Cells
ZNF7361	1.034E-167	0.6744513	0.057	0.23	1.589E-163	T Cells
YES12	1.104E-167	0.14020538	0.041	0.201	1.697E-163	T Cells
DHX571	1.296E-167	0.17516402	0.036	0.193	1.993E-163	T Cells
TEFM	1.69E-167	0.67777545	0.055	0.225	2.597E-163	T Cells
ZNF2071	1.852E-167	1.0301476	0.08	0.267	2.846E-163	T Cells
TTC311	2.04E-167	1.09503867	0.091	0.286	3.136E-163	T Cells
ZNF1433	2.14E-167	1.13501884	0.082	0.271	3.29E-163	T Cells
TIMM212	2.246E-167	0.26641119	0.036	0.193	3.452E-163	T Cells
DHCR71	2.368E-167	0.29384737	0.074	0.255	3.639E-163	T Cells
RPP25L1	2.75E-167	0.4287525	0.044	0.205	4.227E-163	T Cells
FAM78A3	2.888E-167	1.53796834	0.179	0.44	4.439E-163	T Cells
RTP4	4.485E-167	0.21010561	0.039	0.196	6.894E-163	T Cells
SPOUT1	4.633E-167	1.05262522	0.081	0.27	7.121E-163	T Cells
KYAT32	6.817E-167	0.81654074	0.058	0.231	1.048E-162	T Cells
EPHB62	9.916E-167	0.76600494	0.053	0.221	1.524E-162	T Cells
CSTF31	1.123E-166	1.50636385	0.18	0.443	1.726E-162	T Cells
NAA40	1.342E-166	0.65360755	0.054	0.223	2.063E-162	T Cells
THG1L	1.436E-166	0.37366928	0.045	0.207	2.207E-162	T Cells
RCHY12	1.442E-166	1.28349313	0.105	0.309	2.217E-162	T Cells
ZNF184	1.745E-166	0.91802574	0.065	0.242	2.682E-162	T Cells
DDX3X3	1.917E-166	1.4372597	0.192	0.463	2.947E-162	T Cells
DHX331	2.508E-166	0.41379252	0.044	0.205	3.856E-162	T Cells
PIK3C2A2	2.612E-166	0.8111224	0.057	0.227	4.015E-162	T Cells
INTS71	2.788E-166	1.20062974	0.093	0.289	4.285E-162	T Cells
THAP13	2.939E-166	0.83310951	0.057	0.227	4.518E-162	T Cells
SLC38A25	3.186E-166	1.34332328	0.301	0.678	4.897E-162	T Cells
SEC22A1	3.291E-166	1.09422329	0.079	0.264	5.058E-162	T Cells
RBAK1	3.476E-166	0.77950997	0.061	0.235	5.343E-162	T Cells
FBXO451	3.855E-166	0.60567999	0.044	0.206	5.926E-162	T Cells
SS18L22	4.537E-166	1.3909057	0.114	0.325	6.974E-162	T Cells
ATG122	6.648E-166	1.41296809	0.113	0.323	1.022E-161	T Cells
PATZ1	9.614E-166	1.048287	0.086	0.277	1.478E-161	T Cells
FADS23	9.808E-166	0.22237133	0.104	0.305	1.508E-161	T Cells
HNRNPF1	1.161E-165	0.76211927	0.394	0.88	1.785E-161	T Cells
NFRKB2	1.609E-165	1.01221273	0.078	0.262	2.473E-161	T Cells
AARSD11	1.822E-165	0.41181914	0.041	0.2	2.801E-161	T Cells
PCNT1	1.838E-165	1.38200788	0.149	0.382	2.825E-161	T Cells
SUDS32	2.223E-165	1.1775683	0.083	0.272	3.416E-161	T Cells

NOLC11	2.228E-165	0.9700165	0.063	0.237	3.425E-161	T Cells
ZNF5592	2.261E-165	0.94372047	0.069	0.248	3.476E-161	T Cells
PWWP3A1	2.441E-165	1.23043633	0.106	0.31	3.751E-161	T Cells
CCDC1261	2.697E-165	0.42565828	0.047	0.208	4.145E-161	T Cells
CPOX3	2.889E-165	0.80055423	0.062	0.235	4.441E-161	T Cells
ING32	3.4E-165	0.87683237	0.069	0.246	5.226E-161	T Cells
E4F11	3.518E-165	1.25166382	0.106	0.31	5.408E-161	T Cells
YEATS2	4.037E-165	1.24527844	0.102	0.303	6.206E-161	T Cells
CGGBP12	4.47E-165	1.13610486	0.365	0.816	6.871E-161	T Cells
TWF11	5.034E-165	0.44478086	0.043	0.203	7.738E-161	T Cells
ECT21	5.071E-165	0.26861238	0.036	0.191	7.794E-161	T Cells
ODC1	6.754E-165	1.20858141	0.079	0.263	1.038E-160	T Cells
WDR12	8.84E-165	0.29539675	0.035	0.188	1.359E-160	T Cells
NEMP11	9.363E-165	0.96358754	0.067	0.244	1.439E-160	T Cells
POLG1	1.288E-164	1.31537207	0.114	0.324	1.98E-160	T Cells
EEF1AKMT2	1.498E-164	1.30799508	0.11	0.316	2.303E-160	T Cells
PHF143	1.556E-164	1.39420444	0.128	0.348	2.392E-160	T Cells
CHIC22	1.585E-164	1.55659189	0.183	0.446	2.436E-160	T Cells
FDPS1	1.607E-164	0.6530582	0.068	0.243	2.471E-160	T Cells
NGLY1	1.676E-164	1.31012496	0.112	0.32	2.576E-160	T Cells
SNAI31	1.948E-164	1.04247495	0.111	0.318	2.994E-160	T Cells
IQCB11	2.02E-164	0.98577463	0.072	0.252	3.105E-160	T Cells
PDIK1L1	2.208E-164	1.06169094	0.068	0.246	3.394E-160	T Cells
ZNF280D	2.28E-164	1.03332759	0.076	0.259	3.504E-160	T Cells
OTULIN3	2.354E-164	1.516446	0.172	0.424	3.618E-160	T Cells
TRIM35	2.487E-164	1.303538	0.106	0.309	3.823E-160	T Cells
IRF13	2.801E-164	1.21481168	0.282	0.632	4.305E-160	T Cells
UTP251	5.12E-164	1.00861547	0.073	0.253	7.869E-160	T Cells
YARS3	6.851E-164	1.4955751	0.151	0.386	1.053E-159	T Cells
ZC3H12A4	6.921E-164	1.09915665	0.1	0.299	1.064E-159	T Cells
MT-CO24	7.017E-164	2.65856578	0.476	0.45	1.079E-159	T Cells
PANK21	7.449E-164	0.26892706	0.038	0.192	1.145E-159	T Cells
POLR2H2	7.692E-164	1.45391207	0.224	0.519	1.182E-159	T Cells
AMMECR1L1	1.143E-163	0.62300245	0.05	0.213	1.758E-159	T Cells
OSBPL73	1.518E-163	5.82897999	0.032	0.004	2.334E-159	T Cells
TMEM421	1.858E-163	0.64789091	0.044	0.204	2.856E-159	T Cells
ACD1	1.893E-163	1.27854048	0.099	0.297	2.909E-159	T Cells
TSC22D4	2.735E-163	1.15691711	0.354	0.795	4.203E-159	T Cells
HARS2	2.847E-163	0.65757635	0.057	0.225	4.377E-159	T Cells
ELF13	3.076E-163	0.98060755	0.381	0.859	4.727E-159	T Cells
SEPTIN72	3.106E-163	1.53616298	0.195	0.465	4.774E-159	T Cells
ZCCHC81	4.28E-163	1.11467366	0.086	0.273	6.578E-159	T Cells
TOPORS1	4.725E-163	0.84653775	0.059	0.229	7.263E-159	T Cells
HNRNPD3	5.121E-163	1.32494552	0.328	0.733	7.871E-159	T Cells
PRMT23	6.11E-163	1.13440586	0.36	0.804	9.392E-159	T Cells
PTBP32	7.693E-163	0.71895753	0.403	0.897	1.182E-158	T Cells
ALKBH11	7.729E-163	0.43216186	0.043	0.201	1.188E-158	T Cells
KMT2B2	8.582E-163	1.53009303	0.202	0.48	1.319E-158	T Cells
RRAS2	2.526E-162	5.62082221	0.067	0.017	3.882E-158	T Cells
ABCC52	3.06E-162	0.70929896	0.055	0.221	4.704E-158	T Cells
DHX343	3.634E-162	0.63911544	0.06	0.229	5.585E-158	T Cells
POLRMT1	4.193E-162	0.60785889	0.052	0.215	6.445E-158	T Cells

CEP1922	5.436E-162	1.16499749	0.106	0.306	8.356E-158	T Cells
XYLT21	8.26E-162	0.8090652	0.055	0.221	1.27E-157	T Cells
TAF1B	1.138E-161	0.77490493	0.06	0.229	1.749E-157	T Cells
FRS22	2.213E-161	0.9291751	0.063	0.234	3.401E-157	T Cells
TRAPPC6B2	2.739E-161	1.364047	0.115	0.322	4.211E-157	T Cells
CCDC94	3.437E-161	1.36778017	0.143	0.37	5.283E-157	T Cells
MIS18A	3.516E-161	0.18386899	0.034	0.185	5.405E-157	T Cells
EXOSC11	3.757E-161	1.30949733	0.1	0.297	5.775E-157	T Cells
TTC42	3.905E-161	0.41925129	0.038	0.192	6.002E-157	T Cells
DAG1	4.222E-161	0.85075895	0.064	0.235	6.49E-157	T Cells
YTHDF12	8.508E-161	0.59038403	0.041	0.196	1.308E-156	T Cells
RALGAPA22	9.17E-161	0.83517674	0.061	0.23	1.41E-156	T Cells
ZNF2812	9.784E-161	1.28309024	0.087	0.276	1.504E-156	T Cells
BPGM1	1.015E-160	1.33293127	0.082	0.266	1.56E-156	T Cells
BTN3A3	1.043E-160	1.36287502	0.133	0.353	1.603E-156	T Cells
CDKAL12	1.254E-160	1.44093356	0.131	0.349	1.928E-156	T Cells
FAHD2A1	1.266E-160	0.49162414	0.044	0.201	1.946E-156	T Cells
TMEM1351	1.553E-160	0.50902748	0.042	0.199	2.387E-156	T Cells
HEMK12	1.647E-160	0.88988851	0.061	0.231	2.532E-156	T Cells
MTMR91	2.482E-160	1.13649792	0.1	0.295	3.815E-156	T Cells
CAPN151	2.617E-160	0.69789743	0.06	0.228	4.023E-156	T Cells
BAD	3.495E-160	0.19350837	0.037	0.189	5.372E-156	T Cells
MRFAP1L11	4.988E-160	1.58098502	0.203	0.479	7.667E-156	T Cells
LRRC45	5.695E-160	0.53413219	0.047	0.205	8.753E-156	T Cells
SF3B12	8.326E-160	1.50844275	0.184	0.442	1.28E-155	T Cells
COX111	8.88E-160	1.35517032	0.11	0.311	1.365E-155	T Cells
MIER31	1.267E-159	1.01242609	0.067	0.24	1.948E-155	T Cells
HSPA14.11	1.354E-159	0.97842397	0.064	0.236	2.081E-155	T Cells
PARP13	1.492E-159	1.10754382	0.088	0.276	2.293E-155	T Cells
MCM92	1.817E-159	1.00496103	0.066	0.238	2.793E-155	T Cells
PARP61	1.857E-159	1.205452	0.08	0.262	2.854E-155	T Cells
CCDC1301	2.058E-159	1.38060809	0.112	0.317	3.163E-155	T Cells
PSMB92	2.192E-159	0.84700048	0.373	0.829	3.369E-155	T Cells
LIAS2	2.285E-159	0.60436027	0.049	0.209	3.512E-155	T Cells
RWDD32	2.47E-159	0.84679875	0.067	0.24	3.796E-155	T Cells
IER5L3	2.698E-159	0.44628237	0.049	0.208	4.147E-155	T Cells
COMMD81	2.868E-159	0.94281475	0.064	0.234	4.408E-155	T Cells
SH2B1	5.045E-159	1.30471737	0.118	0.327	7.754E-155	T Cells
FOXO41	5.443E-159	1.06820371	0.073	0.25	8.366E-155	T Cells
TRAF61	1.204E-158	1.30165836	0.105	0.303	1.851E-154	T Cells
EPG51	1.491E-158	1.35369123	0.1	0.295	2.292E-154	T Cells
BIN2	1.661E-158	1.28987688	0.306	0.688	2.553E-154	T Cells
CCDC932	1.819E-158	0.72854991	0.052	0.214	2.797E-154	T Cells
ZNF5571	1.9E-158	0.77692563	0.055	0.218	2.92E-154	T Cells
TBCD1	2.268E-158	1.59939938	0.182	0.439	3.486E-154	T Cells
KIAA03551	2.437E-158	1.00809244	0.075	0.251	3.746E-154	T Cells
CSGALNACT24	2.609E-158	1.50534599	0.218	0.506	4.01E-154	T Cells
STK351	2.914E-158	1.14075026	0.081	0.264	4.479E-154	T Cells
GNA133	3.039E-158	0.91263233	0.386	0.854	4.671E-154	T Cells
VAMP52	3.106E-158	0.49620892	0.075	0.251	4.775E-154	T Cells
EXOC21	3.267E-158	1.59726966	0.236	0.539	5.022E-154	T Cells
ZNF712	3.751E-158	0.80493518	0.06	0.226	5.765E-154	T Cells

HERC6	3.937E-158	1.17027602	0.096	0.287	6.051E-154	T Cells
XPO41	4.706E-158	1.28223686	0.097	0.29	7.234E-154	T Cells
ADNP1	5.578E-158	1.2146563	0.096	0.288	8.574E-154	T Cells
ZNF6392	7.063E-158	1.16358482	0.08	0.261	1.086E-153	T Cells
SET1	7.105E-158	1.53739628	0.255	0.575	1.092E-153	T Cells
DHTKD12	8.044E-158	0.71076789	0.053	0.215	1.236E-153	T Cells
ZBTB111	8.575E-158	1.45541655	0.141	0.365	1.318E-153	T Cells
ASB71	1.283E-157	0.8812612	0.063	0.231	1.972E-153	T Cells
TTLL43	1.294E-157	0.57836908	0.047	0.205	1.989E-153	T Cells
ACSS11	1.583E-157	1.18272907	0.097	0.288	2.433E-153	T Cells
C19orf471	1.777E-157	0.57935607	0.045	0.202	2.731E-153	T Cells
UBE2O1	2.346E-157	1.4266692	0.115	0.319	3.607E-153	T Cells
C19orf251	2.396E-157	1.40011751	0.113	0.317	3.682E-153	T Cells
RPA31	2.509E-157	0.37818822	0.034	0.183	3.857E-153	T Cells
PCMTD12	3.01E-157	1.29790788	0.349	0.776	4.626E-153	T Cells
C2orf492	4.098E-157	1.2318115	0.088	0.273	6.299E-153	T Cells
KLHL202	4.616E-157	1.26961788	0.088	0.274	7.095E-153	T Cells
MLLT102	4.706E-157	1.32605476	0.128	0.342	7.234E-153	T Cells
NDRG14	5.308E-157	1.25192223	0.153	0.384	8.16E-153	T Cells
FAM117A1	5.322E-157	4.80146123	0.125	0.048	8.181E-153	T Cells
FAM172A1	5.431E-157	1.50448168	0.151	0.381	8.348E-153	T Cells
CEP571	6.265E-157	1.53258343	0.138	0.359	9.629E-153	T Cells
RBM253	7.097E-157	1.30743764	0.315	0.699	1.091E-152	T Cells
STK32C	1.058E-156	0.14970462	0.035	0.184	1.626E-152	T Cells
CMSS11	1.092E-156	0.39645902	0.039	0.189	1.678E-152	T Cells
MGAT4A4	1.116E-156	1.20872217	0.348	0.769	1.716E-152	T Cells
NEDD11	1.385E-156	1.06821887	0.068	0.239	2.128E-152	T Cells
ZNF354A2	1.54E-156	0.63886087	0.044	0.198	2.368E-152	T Cells
LRR12	1.979E-156	0.50142661	0.036	0.186	3.041E-152	T Cells
RAC2	2.019E-156	1.08068145	0.671	0.947	3.104E-152	T Cells
SLC7A6OS2	2.301E-156	0.92766759	0.063	0.231	3.537E-152	T Cells
NUP882	2.589E-156	1.43602521	0.106	0.303	3.979E-152	T Cells
AP4E1	2.827E-156	1.08771991	0.071	0.245	4.345E-152	T Cells
DDX591	3.133E-156	1.60012838	0.167	0.411	4.815E-152	T Cells
SLC16A62	3.263E-156	0.56339942	0.057	0.219	5.015E-152	T Cells
CTDSPL2	3.41E-156	1.24428767	0.091	0.278	5.242E-152	T Cells
CREBRF1	6.554E-156	1.55823429	0.253	0.571	1.007E-151	T Cells
HERC21	6.862E-156	1.08595181	0.08	0.259	1.055E-151	T Cells
ERN13	7.871E-156	4.27313974	0.262	0.16	1.21E-151	T Cells
UBQLN41	8.27E-156	1.21511416	0.088	0.273	1.271E-151	T Cells
MON22	8.6E-156	1.44708018	0.154	0.385	1.322E-151	T Cells
GIGYF12	8.918E-156	1.50763446	0.186	0.444	1.371E-151	T Cells
CBLL11	9.24E-156	1.25154707	0.082	0.263	1.42E-151	T Cells
GATAD2B	1.26E-155	1.60370925	0.22	0.506	1.937E-151	T Cells
PEAK14	1.435E-155	0.82619619	0.058	0.222	2.206E-151	T Cells
ZNF5802	1.471E-155	1.12116327	0.106	0.302	2.26E-151	T Cells
LIG1	2.966E-155	1.18235538	0.096	0.284	4.559E-151	T Cells
HNRNPH33	3.232E-155	1.2197582	0.354	0.788	4.967E-151	T Cells
TMEM263	3.602E-155	1.46795374	0.114	0.319	5.537E-151	T Cells
UBN21	3.755E-155	1.14809652	0.077	0.255	5.772E-151	T Cells
SKI1	4.258E-155	0.38342675	0.431	0.946	6.546E-151	T Cells
PXMP41	4.537E-155	0.49242722	0.038	0.187	6.974E-151	T Cells

PHF132	6.576E-155	0.42710333	0.045	0.198	1.011E-150	T Cells
SMU12	7.65E-155	0.82851059	0.048	0.205	1.176E-150	T Cells
VAMP12	7.815E-155	5.02460211	0.072	0.02	1.201E-150	T Cells
BCL2L121	1.097E-154	0.12372132	0.029	0.172	1.686E-150	T Cells
CCDC107	1.34E-154	1.40489114	0.119	0.324	2.06E-150	T Cells
INTS2	1.543E-154	0.8471576	0.054	0.215	2.372E-150	T Cells
AGL	1.552E-154	1.13636786	0.078	0.254	2.385E-150	T Cells
STK191	1.695E-154	0.29697027	0.038	0.186	2.606E-150	T Cells
KLHL91	1.771E-154	0.88192758	0.051	0.21	2.722E-150	T Cells
CPSF41	1.882E-154	0.69993482	0.054	0.213	2.893E-150	T Cells
RAPGEF5	2.212E-154	7.45524029	0.01	0	3.4E-150	T Cells
FAM104B1	2.612E-154	0.65296631	0.05	0.206	4.015E-150	T Cells
ZNHIT2	2.775E-154	0.44402628	0.042	0.193	4.266E-150	T Cells
SEPHS12	3.088E-154	0.86581616	0.051	0.21	4.747E-150	T Cells
ARID1B2	3.38E-154	1.43126592	0.129	0.342	5.195E-150	T Cells
TOGARAM12	4.1E-154	0.65258263	0.052	0.211	6.302E-150	T Cells
SBDS2	5.004E-154	1.04984595	0.058	0.221	7.691E-150	T Cells
RPUSD11	5.438E-154	0.64797167	0.052	0.21	8.359E-150	T Cells
TAF22	6.436E-154	1.39351176	0.103	0.299	9.893E-150	T Cells
ZCCHC3	8.593E-154	1.51426193	0.151	0.381	1.321E-149	T Cells
CDK131	9.118E-154	1.61077473	0.219	0.502	1.401E-149	T Cells
ASCC32	9.714E-154	1.49001731	0.138	0.358	1.493E-149	T Cells
PPP4R3A2	9.98E-154	1.61294484	0.245	0.553	1.534E-149	T Cells
WRAP531	1.05E-153	0.43877263	0.035	0.182	1.614E-149	T Cells
GYS14	1.245E-153	1.10745686	0.088	0.27	1.913E-149	T Cells
STIM2	1.317E-153	1.50955557	0.143	0.366	2.025E-149	T Cells
PRPF182	1.583E-153	1.29870146	0.097	0.286	2.434E-149	T Cells
CDK2AP21	2.71E-153	0.79203686	0.06	0.223	4.166E-149	T Cells
PAXBP12	2.726E-153	1.28821816	0.097	0.286	4.19E-149	T Cells
LRRC8A1	3.346E-153	1.40879588	0.115	0.315	5.143E-149	T Cells
TRAF33	3.664E-153	1.26299622	0.095	0.283	5.632E-149	T Cells
GMNN	4.774E-153	0.16322578	0.038	0.185	7.338E-149	T Cells
LSM5	1.085E-152	0.51726779	0.035	0.181	1.668E-148	T Cells
IAH12	1.637E-152	1.45727576	0.209	0.485	2.516E-148	T Cells
LRIF11	1.648E-152	1.23937739	0.084	0.265	2.533E-148	T Cells
ZNF6801	1.989E-152	1.36487659	0.115	0.315	3.057E-148	T Cells
CPEB31	3.373E-152	0.27664712	0.043	0.192	5.184E-148	T Cells
CACTIN	3.806E-152	1.03763304	0.071	0.241	5.851E-148	T Cells
ZNF5442	3.826E-152	1.19605182	0.086	0.267	5.881E-148	T Cells
MTHFSD2	5.27E-152	0.16734689	0.037	0.182	8.101E-148	T Cells
MRGBP2	5.651E-152	1.11277826	0.07	0.24	8.686E-148	T Cells
PTGER22	8.273E-152	4.71319552	0.181	0.088	1.272E-147	T Cells
ZFP302	9.202E-152	0.68068013	0.049	0.203	1.414E-147	T Cells
GPALPP11	9.218E-152	1.35028806	0.093	0.279	1.417E-147	T Cells
RYBP3	1.433E-151	1.51697059	0.147	0.372	2.202E-147	T Cells
FGFRL1	1.568E-151	0.37141608	0.035	0.18	2.411E-147	T Cells
SOCS51	1.606E-151	1.07770377	0.068	0.235	2.468E-147	T Cells
B4GALT31	1.656E-151	1.38176047	0.101	0.292	2.546E-147	T Cells
HAUS11	1.78E-151	0.56421475	0.045	0.197	2.736E-147	T Cells
USP161	2.312E-151	1.63848162	0.152	0.38	3.553E-147	T Cells
C11orf49	2.757E-151	0.65863623	0.052	0.208	4.237E-147	T Cells
DPY19L1	2.784E-151	0.68312487	0.053	0.21	4.279E-147	T Cells

PTS1	3.893E-151	1.25330783	0.089	0.271	5.984E-147	T Cells
STX17	4.132E-151	1.37100222	0.109	0.304	6.352E-147	T Cells
XPC1	4.888E-151	1.56663224	0.174	0.42	7.513E-147	T Cells
PARD6G1	5.897E-151	7.15137661	0.015	0.001	9.065E-147	T Cells
GMPS1	7.353E-151	1.27735026	0.091	0.275	1.13E-146	T Cells
RYK3	1.104E-150	0.27414827	0.038	0.182	1.697E-146	T Cells
RHBDD11	1.518E-150	1.04267647	0.068	0.235	2.333E-146	T Cells
POLA1	1.86E-150	0.71867621	0.048	0.201	2.859E-146	T Cells
IDI12	2.739E-150	1.238263	0.157	0.387	4.211E-146	T Cells
DCLRE1C1	3.388E-150	0.93999823	0.062	0.225	5.207E-146	T Cells
PPP1R3B3	3.807E-150	0.38137417	0.046	0.196	5.851E-146	T Cells
FAM193A1	4.803E-150	1.51621956	0.137	0.351	7.383E-146	T Cells
INTS43	8.876E-150	1.58422061	0.228	0.514	1.364E-145	T Cells
PHF20L13	9.362E-150	1.56090495	0.207	0.48	1.439E-145	T Cells
KRIT11	9.396E-150	1.53878477	0.144	0.365	1.444E-145	T Cells
AKAP8L	1.021E-149	1.46666954	0.138	0.353	1.569E-145	T Cells
PRPF33	1.486E-149	1.56146198	0.208	0.48	2.284E-145	T Cells
ASXL21	1.654E-149	1.62563443	0.206	0.477	2.543E-145	T Cells
TIMM17B2	1.825E-149	1.05377418	0.075	0.246	2.805E-145	T Cells
RPRD21	2.925E-149	1.63938498	0.163	0.397	4.496E-145	T Cells
KATNA11	3.98E-149	1.35129767	0.086	0.266	6.117E-145	T Cells
RBM53	4.03E-149	1.52603511	0.165	0.402	6.194E-145	T Cells
RSL24D11	4.833E-149	1.28640697	0.082	0.258	7.428E-145	T Cells
UQCC31	5.225E-149	0.748893	0.047	0.199	8.031E-145	T Cells
WHAMM1	5.577E-149	1.09883851	0.072	0.241	8.573E-145	T Cells
KBTBD22	5.879E-149	1.57453664	0.229	0.519	9.037E-145	T Cells
PPM1D2	7.046E-149	0.66250795	0.048	0.199	1.083E-144	T Cells
DPM31	7.329E-149	0.21246942	0.031	0.171	1.127E-144	T Cells
TRADD	7.561E-149	1.63620199	0.226	0.513	1.162E-144	T Cells
GAB31	7.701E-149	1.54124561	0.2	0.463	1.184E-144	T Cells
ZBTB31	9.593E-149	0.78681288	0.047	0.199	1.475E-144	T Cells
ANKHD13	1.568E-148	1.33683652	0.329	0.724	2.41E-144	T Cells
MTA13	1.692E-148	1.55559397	0.164	0.398	2.601E-144	T Cells
NARF1	1.985E-148	1.43834928	0.144	0.364	3.051E-144	T Cells
ORC32	2.311E-148	0.94258692	0.056	0.213	3.552E-144	T Cells
ZMYM21	3.467E-148	1.5869712	0.195	0.454	5.33E-144	T Cells
JARID24	3.66E-148	1.39117987	0.12	0.321	5.625E-144	T Cells
C22orf392	4.959E-148	0.87842209	0.056	0.214	7.623E-144	T Cells
DDB21	5.095E-148	1.12702794	0.086	0.264	7.832E-144	T Cells
FKBP31	6.06E-148	1.22856014	0.076	0.247	9.315E-144	T Cells
MAGED11	1.048E-147	1.19051111	0.076	0.247	1.611E-143	T Cells
CMTM82	1.078E-147	6.38115343	0.015	0.001	1.657E-143	T Cells
MEF2D3	1.262E-147	1.51133565	0.165	0.398	1.939E-143	T Cells
TENT4A1	1.817E-147	1.24727645	0.094	0.276	2.794E-143	T Cells
NKD11	1.911E-147	6.59287417	0.02	0.002	2.938E-143	T Cells
MT-CO34	1.92E-147	2.27191659	0.511	0.524	2.951E-143	T Cells
XKR81	1.954E-147	0.47101327	0.042	0.188	3.004E-143	T Cells
AKAP81	2.296E-147	1.43278518	0.127	0.331	3.53E-143	T Cells
LRCH31	2.511E-147	1.52947156	0.162	0.394	3.86E-143	T Cells
SLC25A121	2.789E-147	1.36570076	0.099	0.285	4.287E-143	T Cells
ATXN7L11	3.493E-147	0.9741528	0.081	0.253	5.37E-143	T Cells
DDIT32	4.047E-147	1.61054909	0.141	0.356	6.22E-143	T Cells

TRAM14	4.999E-147	0.61606225	0.412	0.907	7.684E-143	T Cells
FAM50B	5.423E-147	0.70432362	0.054	0.208	8.336E-143	T Cells
CYP20A11	5.559E-147	1.61887099	0.144	0.361	8.545E-143	T Cells
DCP1B1	7.568E-147	1.18349106	0.081	0.254	1.163E-142	T Cells
GUF12	9.388E-147	0.88341589	0.052	0.205	1.443E-142	T Cells
C15orf40	1.023E-146	1.14269157	0.06	0.221	1.572E-142	T Cells
ZBED4	1.078E-146	1.38553643	0.102	0.29	1.656E-142	T Cells
THAP71	1.197E-146	1.07517083	0.07	0.235	1.84E-142	T Cells
PRRC2B1	1.278E-146	1.6187572	0.196	0.455	1.964E-142	T Cells
NCBP2AS22	1.405E-146	1.1876855	0.077	0.246	2.16E-142	T Cells
PGS13	1.471E-146	1.4299606	0.143	0.359	2.261E-142	T Cells
NRBP21	1.696E-146	0.63328853	0.049	0.201	2.606E-142	T Cells
PLEKHG12	1.986E-146	6.73123823	0.016	0.001	3.053E-142	T Cells
SWI52	2.201E-146	0.38806166	0.037	0.18	3.384E-142	T Cells
CIAPIN1	2.303E-146	0.16815226	0.029	0.166	3.54E-142	T Cells
BBS4	2.707E-146	0.28098587	0.036	0.177	4.16E-142	T Cells
CCNT11	2.72E-146	1.42521726	0.135	0.345	4.181E-142	T Cells
BTRC1	3.017E-146	0.69537382	0.045	0.194	4.638E-142	T Cells
PIGC2	3.271E-146	1.30134513	0.091	0.27	5.028E-142	T Cells
TMEM2161	3.931E-146	0.60763393	0.044	0.192	6.043E-142	T Cells
DBT	4.089E-146	0.89213717	0.055	0.21	6.285E-142	T Cells
ANKIB11	5.491E-146	1.06284605	0.069	0.234	8.44E-142	T Cells
POLR3F2	7.23E-146	0.56816372	0.045	0.192	1.111E-141	T Cells
FBXW81	7.723E-146	1.01774762	0.06	0.218	1.187E-141	T Cells
SLC39A132	8.479E-146	0.78518449	0.054	0.207	1.303E-141	T Cells
MRE11	1.108E-145	0.99910399	0.067	0.23	1.703E-141	T Cells
SELENOI1	1.232E-145	1.10602592	0.065	0.227	1.894E-141	T Cells
ZNF6712	1.694E-145	1.0473927	0.063	0.223	2.604E-141	T Cells
EIF4EBP21	1.873E-145	0.36218529	0.433	0.937	2.879E-141	T Cells
C9orf851	1.874E-145	0.98645585	0.059	0.215	2.881E-141	T Cells
HELZ23	2.256E-145	1.22713553	0.113	0.307	3.468E-141	T Cells
CARNMT12	2.339E-145	1.58171753	0.127	0.329	3.595E-141	T Cells
KMT2C3	3.17E-145	1.20515424	0.356	0.781	4.873E-141	T Cells
TTC332	5.795E-145	1.14007248	0.073	0.239	8.908E-141	T Cells
DSTYK1	5.911E-145	0.8287775	0.057	0.212	9.086E-141	T Cells
RAD51D1	6.76E-145	0.7843189	0.052	0.204	1.039E-140	T Cells
NCOA21	7.01E-145	1.54393086	0.136	0.347	1.078E-140	T Cells
B9D2	7.132E-145	0.24087241	0.032	0.169	1.096E-140	T Cells
U2AF1L42	7.141E-145	1.10331044	0.089	0.266	1.098E-140	T Cells
GTF3C41	1.201E-144	0.49458777	0.035	0.175	1.847E-140	T Cells
ZFYVE28	1.317E-144	4.39910633	0.197	0.104	2.025E-140	T Cells
OCIAD21	1.587E-144	4.55039758	0.162	0.076	2.44E-140	T Cells
PBX24	2.403E-144	1.5384374	0.177	0.416	3.694E-140	T Cells
SPRTN1	2.752E-144	0.71685117	0.046	0.192	4.231E-140	T Cells
CDK11B1	2.761E-144	0.83620602	0.053	0.205	4.244E-140	T Cells
GPR1801	2.902E-144	0.72208675	0.045	0.192	4.461E-140	T Cells
OXNAD1	3.413E-144	3.94865814	0.338	0.256	5.246E-140	T Cells
RCE11	5.255E-144	0.48004087	0.035	0.174	8.078E-140	T Cells
SUPV3L11	5.65E-144	1.07680616	0.063	0.222	8.684E-140	T Cells
CNST2	5.856E-144	1.66124963	0.188	0.439	9.002E-140	T Cells
TIAL13	6.183E-144	1.47011995	0.301	0.659	9.504E-140	T Cells
SDHAF11	6.218E-144	0.87228477	0.05	0.201	9.558E-140	T Cells

PPP2R3B2	1.405E-143	0.67696175	0.047	0.194	2.159E-139	T Cells
PKNOX12	2.593E-143	1.22703766	0.073	0.239	3.985E-139	T Cells
HTRA11	2.657E-143	6.18106526	0.012	0.001	4.084E-139	T Cells
LATS12	3.218E-143	1.66043785	0.219	0.496	4.946E-139	T Cells
RSBN11	4.954E-143	1.61013864	0.141	0.353	7.615E-139	T Cells
C6orf136	5.062E-143	1.24119106	0.073	0.238	7.781E-139	T Cells
KBTBD7	5.326E-143	0.39090438	0.031	0.167	8.186E-139	T Cells
DPH52	5.598E-143	1.07819325	0.063	0.221	8.605E-139	T Cells
ELAC12	7.36E-143	0.25591826	0.036	0.175	1.131E-138	T Cells
RNF1701	7.682E-143	0.52628458	0.04	0.181	1.181E-138	T Cells
USP282	9.016E-143	0.79171438	0.044	0.189	1.386E-138	T Cells
CEP120	9.684E-143	1.65205208	0.164	0.394	1.489E-138	T Cells
ZNF123	1.803E-142	1.22634794	0.074	0.24	2.771E-138	T Cells
L3HYPDH1	1.823E-142	0.96315842	0.067	0.226	2.802E-138	T Cells
ZBTB62	1.855E-142	1.20212567	0.056	0.21	2.851E-138	T Cells
TP53BP23	1.893E-142	1.47513892	0.12	0.317	2.91E-138	T Cells
MARCH83	1.923E-142	1.39775669	0.103	0.286	2.955E-138	T Cells
POGLUT3	3.731E-142	0.54312697	0.037	0.176	5.735E-138	T Cells
TBL1XR13	4.314E-142	1.44909349	0.312	0.682	6.631E-138	T Cells
DUSP181	4.659E-142	0.942839	0.061	0.217	7.162E-138	T Cells
GABPB11	6.197E-142	1.30546044	0.08	0.25	9.525E-138	T Cells
TASOR2	6.468E-142	1.62679686	0.134	0.343	9.942E-138	T Cells
MPRIP1	7.054E-142	1.49022977	0.283	0.619	1.084E-137	T Cells
PCF112	8.547E-142	1.51847179	0.273	0.601	1.314E-137	T Cells
FBXO35	9.264E-142	1.36440914	0.11	0.297	1.424E-137	T Cells
CHORDC11	9.335E-142	1.26994743	0.09	0.265	1.435E-137	T Cells
PHF122	1.166E-141	1.66211817	0.193	0.447	1.792E-137	T Cells
B4GALT41	1.281E-141	0.11442047	0.033	0.168	1.969E-137	T Cells
RPGR2	1.791E-141	1.03242747	0.06	0.216	2.753E-137	T Cells
NAE12	2.093E-141	1.56172065	0.126	0.326	3.217E-137	T Cells
MYO192	2.129E-141	1.05419924	0.08	0.249	3.272E-137	T Cells
PPP1R14B2	3.673E-141	0.53169795	0.038	0.178	5.646E-137	T Cells
PHTF21	4.188E-141	1.57391999	0.272	0.596	6.438E-137	T Cells
ANK32	4.86E-141	5.31281192	0.059	0.015	7.471E-137	T Cells
DCAKD1	5.214E-141	0.84446401	0.056	0.208	8.014E-137	T Cells
VPS16	6.829E-141	0.30753298	0.033	0.169	1.05E-136	T Cells
PDS5B2	9.457E-141	1.38599934	0.093	0.269	1.454E-136	T Cells
ZNF7862	1.112E-140	0.50033583	0.04	0.18	1.709E-136	T Cells
HIF1A3	1.576E-140	0.92342348	0.063	0.22	2.422E-136	T Cells
MTG21	1.611E-140	0.55671769	0.04	0.179	2.476E-136	T Cells
CRLF32	1.624E-140	1.59352541	0.19	0.437	2.495E-136	T Cells
PACRGL2	1.697E-140	0.49755127	0.041	0.182	2.608E-136	T Cells
SMPD21	2.004E-140	0.89133808	0.064	0.22	3.08E-136	T Cells
CXorf40A1	2.25E-140	0.55379668	0.061	0.214	3.458E-136	T Cells
POLR1E1	2.403E-140	1.35980489	0.085	0.256	3.694E-136	T Cells
USP1	2.67E-140	1.56521009	0.151	0.369	4.103E-136	T Cells
OMA11	3.677E-140	1.60583766	0.149	0.365	5.651E-136	T Cells
NKRF2	4.62E-140	1.03298178	0.056	0.208	7.101E-136	T Cells
ERCC6L21	5.161E-140	1.62005379	0.149	0.366	7.933E-136	T Cells
AGK1	5.49E-140	0.79179818	0.053	0.202	8.438E-136	T Cells
MAP41	5.87E-140	0.61426639	0.423	0.921	9.023E-136	T Cells
KMT5C	6.564E-140	0.4429763	0.042	0.182	1.009E-135	T Cells

HIVEP31	7.414E-140	0.90466963	0.063	0.219	1.14E-135	T Cells
KLHDC22	7.843E-140	1.66709523	0.181	0.423	1.206E-135	T Cells
CCDC911	9.21E-140	1.72417217	0.227	0.51	1.416E-135	T Cells
SNAPC31	1.015E-139	1.03125542	0.066	0.222	1.561E-135	T Cells
ZMAT11	3.214E-139	0.8426261	0.06	0.214	4.941E-135	T Cells
MFHAS1	3.573E-139	1.68789553	0.252	0.556	5.492E-135	T Cells
CISD11	3.583E-139	0.34794149	0.032	0.167	5.507E-135	T Cells
ARHGAP52	5.244E-139	1.08685006	0.068	0.225	8.06E-135	T Cells
KIAA11091	6.429E-139	1.55732092	0.174	0.407	9.882E-135	T Cells
RNF1153	7.188E-139	1.66537511	0.205	0.465	1.105E-134	T Cells
CASD1	1.65E-138	1.38469948	0.1	0.278	2.536E-134	T Cells
ZNHIT6	1.952E-138	1.5246773	0.108	0.294	3.001E-134	T Cells
PCBP4	4.443E-138	5.57616226	0.042	0.008	6.829E-134	T Cells
ACAD10	4.83E-138	0.48829701	0.036	0.172	7.424E-134	T Cells
NCOR11	5.347E-138	1.08883705	0.38	0.837	8.219E-134	T Cells
BBS1	5.407E-138	0.62887918	0.047	0.19	8.311E-134	T Cells
CLDND12	6.403E-138	1.77958378	0.228	0.512	9.843E-134	T Cells
STK264	6.917E-138	4.743324	0.106	0.039	1.063E-133	T Cells
ATG9A3	1.249E-137	1.13236923	0.076	0.238	1.92E-133	T Cells
SEPSECS2	1.39E-137	1.05474712	0.067	0.224	2.136E-133	T Cells
SRBD11	1.566E-137	0.85717371	0.052	0.198	2.407E-133	T Cells
WDR602	1.784E-137	0.89711935	0.05	0.196	2.742E-133	T Cells
TJAP11	1.94E-137	1.01475704	0.067	0.222	2.982E-133	T Cells
PAAF11	1.985E-137	0.86679639	0.046	0.189	3.052E-133	T Cells
POLR3E2	2.331E-137	1.39057684	0.097	0.273	3.583E-133	T Cells
PALB22	2.545E-137	0.31905026	0.029	0.16	3.912E-133	T Cells
STXBP52	2.96E-137	1.61376457	0.127	0.325	4.55E-133	T Cells
FAAP1001	3.769E-137	0.53459445	0.037	0.174	5.793E-133	T Cells
KANSL13	4.471E-137	1.48252845	0.301	0.65	6.872E-133	T Cells
EVI2B1	4.561E-137	0.23423351	0.45	0.939	7.011E-133	T Cells
ZNF5952	4.786E-137	0.6538215	0.042	0.182	7.356E-133	T Cells
LCP22	4.94E-137	2.01022075	0.604	0.848	7.593E-133	T Cells
ILKAP1	8.421E-137	1.52047125	0.126	0.323	1.294E-132	T Cells
PTPN4	1.229E-136	4.28622052	0.232	0.138	1.889E-132	T Cells
PDXP	1.734E-136	0.31719199	0.035	0.17	2.665E-132	T Cells
LRP12	1.96E-136	0.23568521	0.033	0.165	3.012E-132	T Cells
VPS33B2	2.039E-136	0.3250089	0.035	0.169	3.135E-132	T Cells
ERG28	2.381E-136	1.18447481	0.125	0.32	3.66E-132	T Cells
ZNF585A2	2.424E-136	0.78908495	0.04	0.177	3.726E-132	T Cells
FANCL2	2.745E-136	0.37872706	0.034	0.166	4.219E-132	T Cells
ZFAND2A2	2.788E-136	1.38638178	0.092	0.266	4.285E-132	T Cells
KIFAP31	3.982E-136	1.1027934	0.069	0.225	6.121E-132	T Cells
PUS31	4.021E-136	0.82482115	0.054	0.199	6.181E-132	T Cells
GK51	4.03E-136	0.83542847	0.051	0.196	6.195E-132	T Cells
PRMT71	4.331E-136	1.2028138	0.07	0.228	6.657E-132	T Cells
CCDC182	4.83E-136	0.22046226	0.033	0.165	7.425E-132	T Cells
TATDN21	5.622E-136	1.61362421	0.227	0.503	8.641E-132	T Cells
TDP11	5.751E-136	1.47261998	0.118	0.309	8.84E-132	T Cells
PINX11	6.34E-136	0.86550101	0.047	0.189	9.745E-132	T Cells
NECTIN3	6.979E-136	5.85079025	0.042	0.008	1.073E-131	T Cells
CEP250	1.144E-135	0.50869517	0.036	0.17	1.758E-131	T Cells
BOP11	1.331E-135	0.48647733	0.041	0.178	2.046E-131	T Cells

NPRL21	1.637E-135	1.3801008	0.091	0.261	2.516E-131	T Cells
RHOH	1.782E-135	4.03037859	0.269	0.176	2.739E-131	T Cells
BTN3A2	1.871E-135	1.70376228	0.226	0.501	2.876E-131	T Cells
GPHN2	2.051E-135	0.30471515	0.034	0.167	3.153E-131	T Cells
VAV34	2.141E-135	0.8830774	0.052	0.197	3.291E-131	T Cells
LTBP41	2.558E-135	1.14357487	0.074	0.234	3.933E-131	T Cells
GTPBP101	2.803E-135	1.40908686	0.096	0.27	4.309E-131	T Cells
UCP22	2.838E-135	0.18365057	0.464	0.956	4.362E-131	T Cells
ARL13B	2.935E-135	0.25672821	0.035	0.168	4.511E-131	T Cells
EID21	2.935E-135	0.78723513	0.048	0.19	4.511E-131	T Cells
RNF441	3.31E-135	1.52094059	0.288	0.626	5.088E-131	T Cells
SARM1	4.48E-135	0.70544011	0.045	0.185	6.886E-131	T Cells
ZNF133	4.649E-135	0.31677686	0.04	0.175	7.147E-131	T Cells
PMS2	5.446E-135	0.91829078	0.049	0.191	8.371E-131	T Cells
DHFR2	1.356E-134	0.24600333	0.032	0.162	2.084E-130	T Cells
TRMT61A1	1.738E-134	0.69943362	0.039	0.174	2.671E-130	T Cells
CMPK1	1.741E-134	0.73550828	0.415	0.907	2.676E-130	T Cells
TRRAP1	2.585E-134	1.63712939	0.235	0.514	3.973E-130	T Cells
ZNF73	2.775E-134	0.97303068	0.056	0.204	4.266E-130	T Cells
SMIM291	3.069E-134	1.24046817	0.07	0.228	4.717E-130	T Cells
DRAP1	3.199E-134	0.21222925	0.451	0.957	4.918E-130	T Cells
APAF15	3.607E-134	1.33865699	0.113	0.297	5.544E-130	T Cells
ZNF5741	3.729E-134	1.09277257	0.065	0.217	5.731E-130	T Cells
C17orf751	4.197E-134	1.11511972	0.062	0.213	6.451E-130	T Cells
NUP2101	4.983E-134	1.52620794	0.227	0.5	7.659E-130	T Cells
TMA161	6.286E-134	0.65838769	0.039	0.174	9.662E-130	T Cells
PAQR31	7.425E-134	0.92488555	0.046	0.186	1.141E-129	T Cells
STX23	8.185E-134	1.64330267	0.145	0.354	1.258E-129	T Cells
ASB32	9.249E-134	1.30951744	0.095	0.266	1.422E-129	T Cells
CHKB2	9.707E-134	1.34837602	0.117	0.305	1.492E-129	T Cells
TEX102	1.065E-133	1.14301512	0.067	0.219	1.637E-129	T Cells
WDR441	1.424E-133	1.50866647	0.101	0.278	2.189E-129	T Cells
LPAR64	1.519E-133	1.32316111	0.087	0.253	2.334E-129	T Cells
TBCE.12	1.631E-133	0.60816559	0.035	0.166	2.507E-129	T Cells
TRIM391	2.281E-133	0.94228476	0.061	0.21	3.506E-129	T Cells
BLOC1S4	2.662E-133	1.24671657	0.086	0.25	4.091E-129	T Cells
TRMT61B1	2.986E-133	0.80837598	0.051	0.194	4.589E-129	T Cells
ZNF8231	3.117E-133	0.11457784	0.027	0.153	4.792E-129	T Cells
REXO12	4.09E-133	1.13404808	0.064	0.216	6.287E-129	T Cells
PXYLP12	4.824E-133	6.89940199	0.012	0.001	7.415E-129	T Cells
ETAA12	8.139E-133	0.96868864	0.058	0.205	1.251E-128	T Cells
TSC12	9.69E-133	1.03355411	0.061	0.21	1.489E-128	T Cells
ETV33	1.059E-132	1.30112812	0.078	0.238	1.628E-128	T Cells
MFSD31	1.061E-132	0.39992082	0.036	0.169	1.631E-128	T Cells
PRDM21	1.311E-132	1.64267347	0.251	0.548	2.015E-128	T Cells
PIGW2	1.571E-132	0.34503113	0.034	0.163	2.415E-128	T Cells
ATL21	1.693E-132	1.18843811	0.071	0.225	2.602E-128	T Cells
DGCR81	1.705E-132	1.41801679	0.099	0.273	2.621E-128	T Cells
NPHP42	2.149E-132	0.19990704	0.031	0.158	3.303E-128	T Cells
APBB33	2.2E-132	0.5039701	0.047	0.186	3.382E-128	T Cells
EIF2AK31	2.528E-132	1.10271553	0.062	0.211	3.886E-128	T Cells
YAE11	2.609E-132	0.66630408	0.04	0.174	4.01E-128	T Cells

ARMC7	3.233E-132	0.38907426	0.038	0.17	4.97E-128	T Cells
TOE11	3.24E-132	0.9821183	0.051	0.194	4.98E-128	T Cells
CGRRF11	3.345E-132	0.91128838	0.053	0.195	5.141E-128	T Cells
H2AFX	4.164E-132	0.2828323	0.041	0.175	6.401E-128	T Cells
PDCD7	4.858E-132	1.00738483	0.055	0.2	7.467E-128	T Cells
METTL94	4.918E-132	1.48231486	0.223	0.492	7.56E-128	T Cells
B3GLCT	5.213E-132	0.31890906	0.035	0.165	8.013E-128	T Cells
RTEL12	5.924E-132	0.61040018	0.046	0.184	9.106E-128	T Cells
DALRD31	5.941E-132	0.66164261	0.047	0.185	9.133E-128	T Cells
FBXL41	6.641E-132	1.36949441	0.078	0.238	1.021E-127	T Cells
CWC251	7.681E-132	1.29581029	0.08	0.241	1.181E-127	T Cells
SLC16A12	8.553E-132	1.36423145	0.077	0.237	1.315E-127	T Cells
ATE11	9.04E-132	0.99641053	0.051	0.193	1.39E-127	T Cells
KLF23	9.858E-132	1.54119281	0.32	0.678	1.515E-127	T Cells
LRRC8B	1.247E-131	1.21027002	0.071	0.226	1.917E-127	T Cells
TRIM241	2.752E-131	1.39602693	0.082	0.245	4.23E-127	T Cells
COA12	3.231E-131	0.63743138	0.04	0.174	4.967E-127	T Cells
RFX24	3.643E-131	0.13962444	0.035	0.165	5.6E-127	T Cells
INAFM11	4.031E-131	0.9962652	0.056	0.201	6.196E-127	T Cells
SYTL12	4.386E-131	3.48560063	0.283	0.192	6.742E-127	T Cells
PUSL12	4.727E-131	0.52346817	0.037	0.168	7.265E-127	T Cells
RSAD23	5.783E-131	0.84939973	0.062	0.211	8.889E-127	T Cells
CHD61	5.819E-131	1.39886438	0.1	0.274	8.944E-127	T Cells
ZKSCAN8	5.85E-131	1.55105852	0.112	0.295	8.992E-127	T Cells
PRUNE1	8.434E-131	0.94390606	0.053	0.194	1.296E-126	T Cells
KCNA2	1.19E-130	7.88361956	0.012	0.001	1.83E-126	T Cells
MON1A2	1.256E-130	0.16955545	0.025	0.148	1.931E-126	T Cells
ADGRE52	1.466E-130	0.84645457	0.739	0.985	2.253E-126	T Cells
AGBL5	1.65E-130	0.52373543	0.037	0.167	2.536E-126	T Cells
UTRN1	1.917E-130	1.68854527	0.174	0.4	2.946E-126	T Cells
ZNF1601	1.981E-130	1.12189223	0.07	0.223	3.045E-126	T Cells
FAM117B1	2.605E-130	0.53176945	0.041	0.174	4.004E-126	T Cells
GORAB2	2.849E-130	0.57902391	0.04	0.173	4.379E-126	T Cells
ZNF5981	3.23E-130	1.03637133	0.061	0.209	4.965E-126	T Cells
ZNF1972	4.517E-130	0.90149694	0.052	0.193	6.942E-126	T Cells
HNRNPA02	6.451E-130	0.53718897	0.428	0.931	9.916E-126	T Cells
CCDC612	6.908E-130	0.72809264	0.042	0.177	1.062E-125	T Cells
GMEB12	7.1E-130	1.35721206	0.081	0.24	1.091E-125	T Cells
SIAH22	7.239E-130	1.70566424	0.141	0.343	1.113E-125	T Cells
POLR1B1	9.526E-130	1.17880877	0.069	0.221	1.464E-125	T Cells
POLM1	1.019E-129	1.21534898	0.074	0.231	1.566E-125	T Cells
IMMP2L2	1.054E-129	1.21368517	0.082	0.242	1.62E-125	T Cells
PLCXD12	1.107E-129	0.96413157	0.059	0.203	1.702E-125	T Cells
FAM120C	1.147E-129	0.51884634	0.041	0.174	1.763E-125	T Cells
PDRG11	1.191E-129	0.72552204	0.043	0.177	1.831E-125	T Cells
FBXO43	1.244E-129	0.69075227	0.039	0.171	1.911E-125	T Cells
KRAS4	1.512E-129	1.69011019	0.181	0.412	2.324E-125	T Cells
RAMAC1	1.578E-129	0.87556342	0.045	0.181	2.425E-125	T Cells
MED91	1.893E-129	0.88997687	0.046	0.182	2.91E-125	T Cells
CTPS2	1.931E-129	0.13550672	0.027	0.15	2.968E-125	T Cells
DDX312	1.983E-129	1.23707821	0.063	0.211	3.048E-125	T Cells
ZNF5261	2.855E-129	1.07180023	0.056	0.199	4.388E-125	T Cells

TNFSF83	3.065E-129	5.75693524	0.039	0.007	4.711E-125	T Cells
TRIM132	3.087E-129	1.18275489	0.068	0.22	4.745E-125	T Cells
METTL251	3.191E-129	0.90188769	0.046	0.182	4.905E-125	T Cells
HEG1	3.257E-129	1.18434899	0.06	0.207	5.006E-125	T Cells
MORC21	4.426E-129	1.41576262	0.093	0.262	6.804E-125	T Cells
LSS1	5.318E-129	1.55442746	0.132	0.326	8.174E-125	T Cells
ANKRD542	6.731E-129	0.44611254	0.033	0.159	1.035E-124	T Cells
COX102	7.275E-129	1.17401952	0.07	0.222	1.118E-124	T Cells
ERCC42	7.364E-129	0.23147824	0.029	0.154	1.132E-124	T Cells
HDAC44	8.83E-129	1.35581995	0.096	0.267	1.357E-124	T Cells
FAN11	9.385E-129	0.82279237	0.045	0.18	1.443E-124	T Cells
INTS61	1.057E-128	1.62357598	0.171	0.394	1.625E-124	T Cells
SPRED21	1.171E-128	0.60273148	0.036	0.165	1.8E-124	T Cells
ESCO12	1.259E-128	1.51365111	0.098	0.268	1.936E-124	T Cells
PDP13	1.283E-128	1.32121022	0.077	0.234	1.973E-124	T Cells
AUH1	1.297E-128	0.96668486	0.046	0.182	1.993E-124	T Cells
SUFU1	1.358E-128	0.33870032	0.034	0.161	2.087E-124	T Cells
TTI11	1.618E-128	1.11065572	0.061	0.207	2.487E-124	T Cells
KAT7	1.668E-128	1.67886504	0.223	0.492	2.563E-124	T Cells
GOLGA8B	1.888E-128	4.47235199	0.152	0.072	2.902E-124	T Cells
ZNF2242	1.994E-128	1.38859431	0.091	0.258	3.065E-124	T Cells
ELL5	3.948E-128	1.37023809	0.111	0.289	6.068E-124	T Cells
PUM31	6.297E-128	0.94298729	0.054	0.194	9.679E-124	T Cells
SLC23A23	8.015E-128	1.15285626	0.066	0.215	1.232E-123	T Cells
HERPUD21	1.033E-127	1.34797746	0.352	0.756	1.589E-123	T Cells
LIPE	1.117E-127	7.01442895	0.013	0.001	1.718E-123	T Cells
MIGA11	1.119E-127	1.41772944	0.081	0.24	1.719E-123	T Cells
SCRN14	1.296E-127	0.44638678	0.036	0.165	1.991E-123	T Cells
ATR1	1.317E-127	1.42452494	0.091	0.256	2.024E-123	T Cells
CDK171	1.343E-127	1.5405166	0.127	0.316	2.064E-123	T Cells
GATD1	1.48E-127	1.35021904	0.086	0.249	2.275E-123	T Cells
SESN33	1.666E-127	1.44782725	0.088	0.252	2.561E-123	T Cells
THAP61	1.988E-127	1.43730067	0.087	0.25	3.055E-123	T Cells
CLUAP1	2.151E-127	0.39301984	0.03	0.153	3.307E-123	T Cells
SRPK12	3.086E-127	1.70147824	0.169	0.39	4.743E-123	T Cells
SNRNP481	3.129E-127	0.98089382	0.056	0.198	4.81E-123	T Cells
NSUN51	3.386E-127	0.7425821	0.045	0.178	5.205E-123	T Cells
UBOX51	3.661E-127	1.06980786	0.054	0.195	5.628E-123	T Cells
CCDC88B2	3.776E-127	1.42540722	0.312	0.665	5.803E-123	T Cells
TBC1D311	4.548E-127	1.31488875	0.071	0.223	6.991E-123	T Cells
TRIM461	6.327E-127	6.23998699	0.024	0.003	9.726E-123	T Cells
CENPH	9.887E-127	0.48280664	0.034	0.16	1.52E-122	T Cells
PACS12	1.023E-126	1.65174813	0.282	0.603	1.572E-122	T Cells
BRF12	1.105E-126	1.40178921	0.087	0.249	1.699E-122	T Cells
IKBIP	1.638E-126	0.81055577	0.048	0.182	2.518E-122	T Cells
TERF12	1.831E-126	1.40286316	0.081	0.238	2.815E-122	T Cells
CCDC127	1.846E-126	0.72533034	0.04	0.17	2.837E-122	T Cells
PNPLA72	2.075E-126	0.14576266	0.03	0.152	3.189E-122	T Cells
COIL1	2.177E-126	0.96391826	0.054	0.194	3.346E-122	T Cells
SHMT1	2.214E-126	1.07464402	0.063	0.208	3.403E-122	T Cells
TBCCD12	2.273E-126	1.3384061	0.075	0.23	3.494E-122	T Cells
CCDC144	2.293E-126	1.24306605	0.082	0.24	3.524E-122	T Cells

RINL	2.469E-126	1.29941445	0.086	0.247	3.795E-122	T Cells
CEP761	2.605E-126	0.44267515	0.03	0.154	4.004E-122	T Cells
URGCP	2.781E-126	0.8929694	0.047	0.182	4.275E-122	T Cells
RBBP61	3.731E-126	1.56111111	0.119	0.303	5.735E-122	T Cells
FYCO1	3.78E-126	1.44079017	0.088	0.249	5.81E-122	T Cells
G3BP2	4.052E-126	1.0406077	0.388	0.841	6.228E-122	T Cells
MYO1G	4.7E-126	0.36602434	0.439	0.919	7.224E-122	T Cells
ZNF2832	5.381E-126	0.56859249	0.041	0.171	8.271E-122	T Cells
TUBGCP52	5.722E-126	0.84008308	0.043	0.176	8.795E-122	T Cells
TOP2B2	7.059E-126	1.64347779	0.133	0.325	1.085E-121	T Cells
RBM121	1.171E-125	1.55712824	0.108	0.283	1.8E-121	T Cells
JMJD41	1.247E-125	0.99231438	0.055	0.196	1.917E-121	T Cells
RELL12	1.349E-125	1.62369017	0.137	0.331	2.074E-121	T Cells
KLHL221	1.392E-125	1.18468178	0.07	0.22	2.14E-121	T Cells
GCFC21	1.475E-125	0.9448863	0.051	0.187	2.267E-121	T Cells
OSGEPL11	1.614E-125	0.12960333	0.025	0.144	2.48E-121	T Cells
SRD5A11	1.879E-125	0.85513382	0.038	0.167	2.888E-121	T Cells
CHAF1A2	2.434E-125	0.44084033	0.03	0.153	3.741E-121	T Cells
GADD45A	2.506E-125	0.64862595	0.057	0.197	3.851E-121	T Cells
PUS7L1	2.591E-125	1.40421532	0.085	0.244	3.983E-121	T Cells
IKBKE1	2.798E-125	1.29868892	0.096	0.261	4.301E-121	T Cells
SUGP23	2.932E-125	1.47408398	0.209	0.458	4.507E-121	T Cells
ARID4B1	3.659E-125	1.63417806	0.256	0.551	5.624E-121	T Cells
DNAJC241	4.165E-125	1.00242204	0.053	0.191	6.402E-121	T Cells
ATP10D2	5.144E-125	1.28868659	0.085	0.243	7.907E-121	T Cells
ZNF4442	5.191E-125	0.93296094	0.056	0.196	7.98E-121	T Cells
SPATA21	6.911E-125	0.4995721	0.035	0.161	1.062E-120	T Cells
ZNF1213	7.843E-125	1.68061431	0.137	0.331	1.206E-120	T Cells
SDHAF31	8.995E-125	0.89707944	0.039	0.168	1.383E-120	T Cells
ZDHHC175	9.849E-125	1.54758339	0.106	0.279	1.514E-120	T Cells
RFC11	1.033E-124	1.45693112	0.098	0.266	1.587E-120	T Cells
DOCK22	1.107E-124	0.58561777	0.433	0.927	1.701E-120	T Cells
GLYCTK2	1.156E-124	1.40400989	0.112	0.289	1.778E-120	T Cells
MAML13	1.507E-124	1.64172446	0.257	0.553	2.316E-120	T Cells
SETD51	1.622E-124	1.62103323	0.253	0.542	2.494E-120	T Cells
C7orf312	1.785E-124	0.22303761	0.028	0.148	2.744E-120	T Cells
EIF4ENIF11	1.937E-124	1.42619189	0.077	0.23	2.978E-120	T Cells
ADD31	2.011E-124	1.8041322	0.211	0.466	3.091E-120	T Cells
SCFD22	2.157E-124	1.01555166	0.048	0.182	3.316E-120	T Cells
ZC3H6	3.335E-124	1.54259436	0.094	0.259	5.126E-120	T Cells
ATP13A2	3.744E-124	0.94561432	0.058	0.198	5.755E-120	T Cells
ZKSCAN51	4.089E-124	0.13481459	0.03	0.151	6.285E-120	T Cells
MEX3D2	4.322E-124	0.27283136	0.032	0.154	6.643E-120	T Cells
RNF1211	4.528E-124	1.16467471	0.057	0.197	6.96E-120	T Cells
SRSF101	4.578E-124	0.73677634	0.036	0.162	7.036E-120	T Cells
PEX1	6.059E-124	1.15447458	0.069	0.217	9.314E-120	T Cells
TCF201	6.915E-124	1.65088925	0.137	0.332	1.063E-119	T Cells
ZNF8211	6.951E-124	0.75648118	0.048	0.181	1.069E-119	T Cells
PHF81	7.967E-124	1.68411135	0.145	0.345	1.225E-119	T Cells
NDUFAF71	8.131E-124	1.36404018	0.073	0.224	1.25E-119	T Cells
GPSM3	1.028E-123	0.3726192	0.441	0.945	1.58E-119	T Cells
HIBCH	1.058E-123	0.64673266	0.037	0.163	1.626E-119	T Cells

TRMT2B1	1.059E-123	1.58193035	0.105	0.276	1.628E-119	T Cells
RUFY22	1.542E-123	1.42286762	0.098	0.264	2.371E-119	T Cells
TIMM8A2	2.097E-123	0.62251937	0.035	0.159	3.224E-119	T Cells
DDX551	2.288E-123	1.37739001	0.068	0.215	3.517E-119	T Cells
TRNT11	2.327E-123	1.51948202	0.101	0.27	3.577E-119	T Cells
FOXP14	3.087E-123	1.53870273	0.313	0.664	4.746E-119	T Cells
RTL101	3.711E-123	1.00760757	0.064	0.208	5.704E-119	T Cells
LIN7B2	4.039E-123	0.4591427	0.037	0.163	6.209E-119	T Cells
MED261	4.734E-123	1.2154373	0.062	0.204	7.277E-119	T Cells
SUN12	4.771E-123	1.6554888	0.127	0.314	7.333E-119	T Cells
ABI21	4.813E-123	1.50284093	0.091	0.254	7.398E-119	T Cells
RELCH3	6.324E-123	1.56389402	0.189	0.422	9.72E-119	T Cells
METTL81	6.964E-123	0.85104439	0.04	0.166	1.07E-118	T Cells
BIN11	7.677E-123	1.52208733	0.255	0.544	1.18E-118	T Cells
MICA	7.694E-123	1.12273627	0.056	0.195	1.183E-118	T Cells
ZNF2001	9.133E-123	1.11180761	0.056	0.194	1.404E-118	T Cells
CNOT31	9.234E-123	1.14415451	0.061	0.203	1.419E-118	T Cells
KLHL42	1.225E-122	0.80265882	0.05	0.184	1.883E-118	T Cells
DCTN51	1.705E-122	0.89015486	0.042	0.17	2.62E-118	T Cells
NCAPG2	1.801E-122	0.42790401	0.041	0.168	2.768E-118	T Cells
RRP8	1.98E-122	1.18769514	0.057	0.196	3.043E-118	T Cells
DPY19L41	2.22E-122	0.51613181	0.038	0.163	3.413E-118	T Cells
NFYB	2.533E-122	1.42828361	0.082	0.238	3.894E-118	T Cells
PDK12	3.046E-122	1.35505427	0.076	0.227	4.682E-118	T Cells
NOL101	3.816E-122	0.83401973	0.042	0.17	5.866E-118	T Cells
IFFO23	4.98E-122	4.19928603	0.209	0.122	7.654E-118	T Cells
ABHD17B1	4.999E-122	1.37964568	0.076	0.228	7.684E-118	T Cells
ARMCX52	5.316E-122	0.55622122	0.038	0.163	8.17E-118	T Cells
TNIK	5.511E-122	1.62753095	0.233	0.5	8.47E-118	T Cells
ATP11C1	5.646E-122	0.88422031	0.045	0.175	8.679E-118	T Cells
TNK22	5.886E-122	1.57012599	0.196	0.431	9.047E-118	T Cells
MCEE1	6.623E-122	1.26238249	0.061	0.202	1.018E-117	T Cells
UVSSA2	6.981E-122	1.04977183	0.072	0.22	1.073E-117	T Cells
SBNO12	7.022E-122	1.70112784	0.16	0.369	1.079E-117	T Cells
ZNF3331	7.406E-122	0.97682881	0.054	0.191	1.138E-117	T Cells
SLC26A62	1.095E-121	0.72099465	0.048	0.179	1.684E-117	T Cells
HIST1H1D2	1.347E-121	3.51435186	0.207	0.119	2.071E-117	T Cells
AP4M11	1.377E-121	0.11932322	0.031	0.151	2.116E-117	T Cells
ZNF31	1.583E-121	1.03287155	0.053	0.189	2.432E-117	T Cells
ZFP641	1.731E-121	0.88726828	0.047	0.177	2.661E-117	T Cells
MARCH63	2.487E-121	1.7536639	0.247	0.531	3.822E-117	T Cells
EVI2A2	4.471E-121	1.43119533	0.316	0.665	6.872E-117	T Cells
PRMT32	5.219E-121	1.12789556	0.049	0.183	8.021E-117	T Cells
SOCS4	5.474E-121	1.64131959	0.12	0.299	8.414E-117	T Cells
ZSCAN212	5.676E-121	0.67913651	0.039	0.163	8.725E-117	T Cells
FGFR1OP2	5.818E-121	1.750852	0.222	0.482	8.943E-117	T Cells
GIMAP83	6.302E-121	1.24506461	0.074	0.223	9.686E-117	T Cells
DCAF131	8.706E-121	0.81365939	0.041	0.167	1.338E-116	T Cells
PAK4	8.923E-121	0.26514671	0.032	0.151	1.372E-116	T Cells
TMEM641	1.002E-120	1.21023525	0.067	0.211	1.541E-116	T Cells
ZNF5491	1.016E-120	0.79452565	0.051	0.184	1.561E-116	T Cells
MYNN2	1.024E-120	1.58651556	0.108	0.28	1.574E-116	T Cells

EIF2S21	1.76E-120	0.14796181	0.024	0.138	2.706E-116	T Cells
SETD1B3	1.772E-120	1.64438601	0.292	0.619	2.724E-116	T Cells
IFT882	1.778E-120	0.75642597	0.04	0.166	2.733E-116	T Cells
MYO9A3	1.886E-120	1.57276543	0.121	0.3	2.899E-116	T Cells
PPP1R13B2	1.954E-120	0.63365084	0.039	0.163	3.003E-116	T Cells
ZNF4361	1.995E-120	0.54034391	0.034	0.155	3.066E-116	T Cells
C19orf48	2.282E-120	0.79247602	0.046	0.175	3.508E-116	T Cells
TAF1D3	2.568E-120	1.66359583	0.233	0.498	3.947E-116	T Cells
DNAJC161	2.95E-120	0.87937337	0.046	0.175	4.535E-116	T Cells
RBM261	3.366E-120	1.63517814	0.149	0.349	5.175E-116	T Cells
SP33	4.69E-120	1.33346674	0.359	0.765	7.21E-116	T Cells
GNAO13	5.167E-120	4.36124734	0.028	0.005	7.942E-116	T Cells
ZMYM11	5.573E-120	1.0111742	0.056	0.191	8.566E-116	T Cells
PLEKHM11	5.709E-120	0.71069211	0.034	0.156	8.775E-116	T Cells
MAU2	6.716E-120	0.37886131	0.031	0.15	1.032E-115	T Cells
ZNF4451	9.68E-120	1.0505049	0.056	0.192	1.488E-115	T Cells
FAM13A2	1.464E-119	0.10586375	0.021	0.133	2.25E-115	T Cells
SECISBP23	3.515E-119	1.71271803	0.169	0.38	5.404E-115	T Cells
STARD42	4.087E-119	1.23097835	0.089	0.247	6.283E-115	T Cells
SNX162	4.095E-119	1.03790138	0.058	0.194	6.294E-115	T Cells
PLCB11	4.41E-119	6.54001122	0.018	0.002	6.778E-115	T Cells
GXYLT12	4.509E-119	0.586318	0.04	0.164	6.93E-115	T Cells
GARS2	4.538E-119	1.47083302	0.093	0.253	6.976E-115	T Cells
TTLL13	5.096E-119	0.93230226	0.043	0.169	7.833E-115	T Cells
SLC20A2	5.457E-119	0.97758419	0.053	0.186	8.388E-115	T Cells
ZNF7831	8.315E-119	1.31908986	0.084	0.238	1.278E-114	T Cells
ZBED6CL	8.732E-119	0.81995985	0.048	0.177	1.342E-114	T Cells
LIN37	1.03E-118	0.88936448	0.049	0.178	1.583E-114	T Cells
WDR201	1.066E-118	1.66142009	0.118	0.296	1.639E-114	T Cells
HLCS2	1.085E-118	1.09031005	0.06	0.197	1.668E-114	T Cells
FAM241A1	1.257E-118	1.67366726	0.109	0.279	1.932E-114	T Cells
DIS3L21	1.45E-118	1.47846133	0.115	0.286	2.229E-114	T Cells
SHPK	1.495E-118	0.59929985	0.034	0.153	2.297E-114	T Cells
UBIAD1	1.96E-118	1.58749019	0.088	0.245	3.012E-114	T Cells
PIGH	2.039E-118	1.27214432	0.061	0.199	3.135E-114	T Cells
SUCO1	2.31E-118	1.65599111	0.119	0.296	3.551E-114	T Cells
SLC35D21	2.324E-118	1.61974813	0.166	0.375	3.573E-114	T Cells
USP481	2.329E-118	1.74623599	0.201	0.439	3.579E-114	T Cells
RCN21	2.975E-118	1.07132657	0.051	0.183	4.573E-114	T Cells
RAD172	3.074E-118	1.34473092	0.067	0.209	4.725E-114	T Cells
FASN	3.657E-118	1.13418133	0.097	0.257	5.621E-114	T Cells
EXOSC21	3.867E-118	1.3967546	0.072	0.216	5.945E-114	T Cells
SFXN41	4.511E-118	0.40867849	0.029	0.145	6.934E-114	T Cells
PDE7A3	5.121E-118	3.74576723	0.32	0.247	7.871E-114	T Cells
ASB62	5.285E-118	1.26662762	0.064	0.205	8.123E-114	T Cells
PMM1	7.21E-118	1.39256932	0.082	0.233	1.108E-113	T Cells
SPATA5L12	7.432E-118	0.82362783	0.043	0.17	1.142E-113	T Cells
GFOD11	7.57E-118	0.70312335	0.036	0.157	1.164E-113	T Cells
AC118549.11	1.235E-117	1.46841483	0.083	0.236	1.899E-113	T Cells
ELK11	1.25E-117	0.93234163	0.05	0.18	1.921E-113	T Cells
PPWD11	1.959E-117	1.68512079	0.14	0.33	3.011E-113	T Cells
DISC13	2.077E-117	1.34913478	0.086	0.24	3.192E-113	T Cells

APBB1IP2	2.461E-117	1.23420413	0.371	0.787	3.783E-113	T Cells
DLG11	2.801E-117	1.74025125	0.152	0.351	4.306E-113	T Cells
ARL5A1	3.421E-117	0.66341664	0.036	0.157	5.259E-113	T Cells
NEPRO1	3.503E-117	1.7253641	0.118	0.293	5.385E-113	T Cells
APOBEC3G1	3.622E-117	3.9135585	0.329	0.26	5.568E-113	T Cells
RBM381	4.29E-117	4.29173215	0.192	0.109	6.595E-113	T Cells
P4HTM	4.972E-117	1.30276178	0.073	0.217	7.643E-113	T Cells
ZFC3H13	5.292E-117	1.59692529	0.211	0.455	8.135E-113	T Cells
ZNF3502	5.308E-117	1.13176403	0.058	0.192	8.159E-113	T Cells
CBX65	8.961E-117	1.26510932	0.1	0.262	1.377E-112	T Cells
SLFN122	1.187E-116	0.14744434	0.023	0.133	1.825E-112	T Cells
RAD51C2	1.808E-116	0.55558938	0.034	0.152	2.779E-112	T Cells
TGDS2	2.138E-116	0.66358023	0.037	0.157	3.286E-112	T Cells
ZNF7381	2.34E-116	0.95604616	0.046	0.173	3.597E-112	T Cells
C3orf621	2.353E-116	0.17896108	0.026	0.138	3.617E-112	T Cells
PRMT61	2.709E-116	0.43496977	0.035	0.153	4.164E-112	T Cells
LXN2	3.442E-116	0.79929982	0.042	0.166	5.29E-112	T Cells
NINL	3.464E-116	6.20677985	0.019	0.002	5.325E-112	T Cells
IRAK42	3.628E-116	1.71922077	0.165	0.374	5.577E-112	T Cells
TSPAN323	4.998E-116	0.6686553	0.042	0.165	7.682E-112	T Cells
VEZT1	5.819E-116	0.89069952	0.048	0.175	8.944E-112	T Cells
ALS22	5.937E-116	0.50967766	0.036	0.155	9.125E-112	T Cells
FAM98C2	6.873E-116	1.18534894	0.063	0.2	1.056E-111	T Cells
CNTRL2	6.883E-116	1.29949072	0.351	0.74	1.058E-111	T Cells
DTWD11	7.177E-116	0.49972282	0.026	0.138	1.103E-111	T Cells
KHK1	7.903E-116	0.16071111	0.024	0.135	1.215E-111	T Cells
MCPH12	8.398E-116	1.62939114	0.138	0.325	1.291E-111	T Cells
CDKN1B1	1.004E-115	2.22413808	0.584	0.814	1.543E-111	T Cells
TRA2A3	1.036E-115	1.32026741	0.36	0.766	1.592E-111	T Cells
SPIN42	1.452E-115	0.55468485	0.034	0.152	2.232E-111	T Cells
HELB3	1.954E-115	4.07142937	0.199	0.114	3.003E-111	T Cells
ATP6V0E2	2.449E-115	1.22561059	0.053	0.185	3.765E-111	T Cells
ZFX	2.51E-115	1.19041673	0.067	0.206	3.858E-111	T Cells
LPCAT41	3.01E-115	1.71549791	0.161	0.364	4.627E-111	T Cells
ACTA21	3.017E-115	0.69910442	0.047	0.173	4.637E-111	T Cells
SRR1	3.479E-115	0.14086322	0.021	0.13	5.347E-111	T Cells
URB11	4.089E-115	1.02660017	0.052	0.181	6.286E-111	T Cells
DGKH1	4.206E-115	1.20777786	0.062	0.198	6.465E-111	T Cells
WDR92	5.009E-115	0.40764522	0.03	0.144	7.699E-111	T Cells
WDR75	5.158E-115	1.50493364	0.082	0.231	7.928E-111	T Cells
VARS2	6.323E-115	0.75574418	0.038	0.158	9.719E-111	T Cells
PYGO21	6.833E-115	1.81052744	0.193	0.422	1.05E-110	T Cells
TXLNG	9.205E-115	1.29199871	0.068	0.207	1.415E-110	T Cells
MYEF22	1.221E-114	4.76889911	0.051	0.013	1.877E-110	T Cells
MFSD9	2.035E-114	0.78165539	0.043	0.166	3.128E-110	T Cells
SNRK2	2.341E-114	1.70719687	0.252	0.531	3.599E-110	T Cells
FUNDC2	2.474E-114	0.1569424	0.024	0.133	3.803E-110	T Cells
TMEM1811	2.769E-114	1.73342791	0.111	0.28	4.257E-110	T Cells
IL4R2	3.347E-114	1.09057868	0.063	0.2	5.145E-110	T Cells
SHQ11	3.893E-114	0.73783143	0.035	0.152	5.984E-110	T Cells
PTDSS21	4.222E-114	0.44642974	0.035	0.151	6.49E-110	T Cells
ZNF8301	6.087E-114	1.74945182	0.113	0.282	9.356E-110	T Cells

BTAF14	6.178E-114	1.64668021	0.195	0.42	9.496E-110	T Cells
PLXNA11	6.237E-114	0.80891626	0.046	0.171	9.587E-110	T Cells
ZNF7001	6.253E-114	1.38997336	0.087	0.238	9.612E-110	T Cells
PCID21	9.756E-114	1.84625599	0.172	0.383	1.5E-109	T Cells
CNOT1	1.107E-113	1.34031552	0.07	0.211	1.701E-109	T Cells
SERAC12	1.153E-113	0.20265059	0.027	0.138	1.772E-109	T Cells
PCMTD2	1.161E-113	1.73074732	0.163	0.367	1.785E-109	T Cells
MTO11	1.323E-113	1.77605531	0.16	0.363	2.033E-109	T Cells
KLHL281	1.328E-113	1.5520211	0.094	0.251	2.042E-109	T Cells
CDK72	1.363E-113	0.38367995	0.033	0.148	2.095E-109	T Cells
ZNF4491	1.419E-113	0.31938098	0.027	0.139	2.182E-109	T Cells
DDX102	1.614E-113	1.09414976	0.05	0.178	2.48E-109	T Cells
KLC42	2.14E-113	0.49392657	0.031	0.145	3.29E-109	T Cells
SS18L11	2.175E-113	1.07867423	0.064	0.2	3.344E-109	T Cells
USF32	2.504E-113	1.40653501	0.07	0.209	3.849E-109	T Cells
FAM221A1	2.653E-113	0.4183189	0.029	0.141	4.078E-109	T Cells
CASP8AP21	3.127E-113	1.50396716	0.086	0.237	4.807E-109	T Cells
COX202	4.19E-113	1.34610342	0.064	0.2	6.44E-109	T Cells
INTS81	4.456E-113	1.77325338	0.153	0.347	6.849E-109	T Cells
TDG2	4.469E-113	0.62453292	0.03	0.143	6.869E-109	T Cells
TOP3B2	4.719E-113	1.23204068	0.065	0.201	7.254E-109	T Cells
SHPRH2	4.914E-113	1.39532825	0.094	0.25	7.554E-109	T Cells
R3HDM44	5.897E-113	1.6734934	0.299	0.629	9.064E-109	T Cells
C12orf452	5.944E-113	0.58439695	0.032	0.146	9.137E-109	T Cells
MNAT1	6.895E-113	1.0132923	0.054	0.183	1.06E-108	T Cells
ZFP31	7.167E-113	0.96079539	0.047	0.172	1.102E-108	T Cells
MED211	8.534E-113	1.63348711	0.092	0.247	1.312E-108	T Cells
PHLPP21	1.094E-112	1.08899426	0.055	0.184	1.682E-108	T Cells
MTRES12	1.134E-112	0.72097193	0.038	0.156	1.743E-108	T Cells
TCTN2	1.47E-112	0.28283983	0.024	0.132	2.26E-108	T Cells
KIAA05861	1.792E-112	1.28342353	0.061	0.195	2.755E-108	T Cells
POLR1C1	1.907E-112	0.77286267	0.034	0.15	2.931E-108	T Cells
NVL	2.123E-112	1.23086148	0.063	0.197	3.264E-108	T Cells
ZNF3982	2.877E-112	1.52198439	0.082	0.227	4.422E-108	T Cells
DUSP112	3.477E-112	1.77596681	0.158	0.356	5.344E-108	T Cells
RFFL	3.603E-112	1.8134545	0.208	0.446	5.538E-108	T Cells
TMEM50B	4.359E-112	1.74501923	0.131	0.31	6.7E-108	T Cells
FAM122A1	4.457E-112	1.12411085	0.046	0.17	6.85E-108	T Cells
MMS22L1	5.466E-112	0.78114413	0.04	0.159	8.402E-108	T Cells
POLR3D1	6.264E-112	1.41405814	0.072	0.212	9.628E-108	T Cells
TMEM2342	9.054E-112	0.99009873	0.051	0.176	1.392E-107	T Cells
ZBTB402	1.206E-111	1.52971058	0.088	0.238	1.854E-107	T Cells
OXSM2	1.251E-111	0.33718058	0.03	0.141	1.923E-107	T Cells
DVL1	1.253E-111	0.74520447	0.037	0.154	1.925E-107	T Cells
RAD54L21	1.332E-111	1.43119466	0.076	0.219	2.048E-107	T Cells
ZC3HC12	1.526E-111	0.99135688	0.046	0.169	2.345E-107	T Cells
TMEM2681	1.885E-111	1.00175472	0.04	0.159	2.897E-107	T Cells
LIX1L	2.227E-111	1.42084782	0.348	0.728	3.424E-107	T Cells
ZBTB42	3.003E-111	0.78351795	0.032	0.145	4.617E-107	T Cells
MFGE8	3.298E-111	0.79076137	0.04	0.159	5.07E-107	T Cells
TRIT11	3.761E-111	0.90455574	0.043	0.163	5.78E-107	T Cells
RILPL12	3.8E-111	0.34125828	0.028	0.138	5.841E-107	T Cells

PPIH1	5.432E-111	0.91897204	0.043	0.163	8.35E-107	T Cells
HMGB2	6.203E-111	1.29193301	0.117	0.285	9.534E-107	T Cells
PCM11	7.048E-111	1.31309901	0.368	0.779	1.083E-106	T Cells
SYNCRIP1	7.454E-111	0.35909631	0.027	0.137	1.146E-106	T Cells
BTF3	7.544E-111	0.89126822	0.038	0.155	1.16E-106	T Cells
HNRNPUL12	9.058E-111	1.07789047	0.398	0.849	1.392E-106	T Cells
ZRSR21	1.037E-110	0.87061795	0.047	0.17	1.595E-106	T Cells
VPS51	1.137E-110	1.45708219	0.081	0.226	1.748E-106	T Cells
CEP85L	1.313E-110	4.21077502	0.187	0.106	2.018E-106	T Cells
MIS12	1.647E-110	1.53503586	0.078	0.222	2.531E-106	T Cells
TMEM41A1	1.755E-110	0.52372344	0.032	0.145	2.698E-106	T Cells
DYRK1B	1.825E-110	1.18704356	0.065	0.199	2.806E-106	T Cells
TRIM32	2.017E-110	1.12500817	0.056	0.184	3.1E-106	T Cells
AGO31	2.252E-110	1.55441997	0.112	0.275	3.461E-106	T Cells
ZNF6241	2.887E-110	0.68146578	0.035	0.149	4.438E-106	T Cells
ZNF5324	3.413E-110	0.13293483	0.021	0.126	5.247E-106	T Cells
XXYLT13	4.287E-110	0.361133	0.024	0.131	6.59E-106	T Cells
STRADB1	4.359E-110	0.14971561	0.026	0.132	6.7E-106	T Cells
ZNF3291	4.73E-110	1.31551923	0.052	0.178	7.27E-106	T Cells
ICK1	6.1E-110	1.03877947	0.043	0.163	9.377E-106	T Cells
URB21	6.609E-110	0.58148667	0.03	0.141	1.016E-105	T Cells
TUBE1	9.678E-110	1.34639918	0.075	0.214	1.488E-105	T Cells
IPPK1	9.706E-110	0.98298907	0.046	0.168	1.492E-105	T Cells
STX162	1.091E-109	1.55257428	0.279	0.576	1.676E-105	T Cells
YAF21	1.132E-109	1.62188482	0.125	0.298	1.74E-105	T Cells
C1GALT12	1.14E-109	1.44297991	0.082	0.226	1.752E-105	T Cells
GLS1	1.404E-109	1.84723099	0.169	0.373	2.159E-105	T Cells
SETD42	1.482E-109	1.20876719	0.065	0.199	2.278E-105	T Cells
ZNF451	1.682E-109	1.23336183	0.054	0.181	2.586E-105	T Cells
ZFP90	2.202E-109	1.45281278	0.075	0.216	3.384E-105	T Cells
SH3YL11	2.256E-109	1.41421323	0.069	0.206	3.467E-105	T Cells
UBL4A	2.284E-109	0.53172488	0.032	0.144	3.51E-105	T Cells
TEX30	2.311E-109	1.00073438	0.04	0.157	3.553E-105	T Cells
FAM217B1	2.427E-109	1.39889933	0.069	0.205	3.731E-105	T Cells
PRDX2	2.477E-109	1.24070981	0.213	0.448	3.808E-105	T Cells
NMRK12	2.509E-109	1.81446362	0.164	0.365	3.857E-105	T Cells
BMPR1A2	3.042E-109	0.80862029	0.04	0.157	4.676E-105	T Cells
MAPK15	3.482E-109	0.69559359	0.432	0.923	5.352E-105	T Cells
CROT1	3.757E-109	0.41294358	0.03	0.141	5.775E-105	T Cells
BRAP	3.888E-109	0.91919036	0.041	0.159	5.977E-105	T Cells
EIF1AY	4.506E-109	1.12614654	0.073	0.21	6.927E-105	T Cells
TFEB5	4.912E-109	0.96298996	0.053	0.179	7.551E-105	T Cells
ZNF7771	4.991E-109	1.25962565	0.06	0.19	7.672E-105	T Cells
ARID21	5.777E-109	1.74755008	0.136	0.316	8.88E-105	T Cells
SIRT32	7.467E-109	0.66842479	0.039	0.154	1.148E-104	T Cells
NOP58	8.067E-109	1.87015111	0.17	0.375	1.24E-104	T Cells
RITA1	1.204E-108	0.88841925	0.036	0.151	1.85E-104	T Cells
CCNT23	1.295E-108	1.66969234	0.178	0.387	1.991E-104	T Cells
CPSF1	1.367E-108	1.26870724	0.069	0.205	2.102E-104	T Cells
GNPNAT11	1.43E-108	0.66575424	0.03	0.14	2.198E-104	T Cells
ZNF8411	1.565E-108	1.22582397	0.063	0.196	2.406E-104	T Cells
ARHGEF31	1.573E-108	1.74543444	0.268	0.555	2.417E-104	T Cells

RBM12B2	1.93E-108	1.46836549	0.078	0.219	2.966E-104	T Cells
TTC131	3.117E-108	1.66208816	0.116	0.282	4.791E-104	T Cells
METTL62	3.118E-108	1.22845943	0.058	0.187	4.792E-104	T Cells
ITGA2	3.141E-108	5.61160281	0.013	0.001	4.828E-104	T Cells
BATF4	3.245E-108	4.65993618	0.103	0.043	4.987E-104	T Cells
NCDN	3.345E-108	1.08462672	0.048	0.169	5.142E-104	T Cells
BCLAF32	3.384E-108	1.60069804	0.103	0.259	5.201E-104	T Cells
ZNF518B	3.667E-108	1.11781421	0.054	0.18	5.637E-104	T Cells
SCLT12	4.456E-108	1.08060121	0.064	0.195	6.849E-104	T Cells
SPSB32	4.529E-108	1.75689256	0.211	0.448	6.961E-104	T Cells
PRIM1	4.806E-108	1.04329706	0.047	0.167	7.387E-104	T Cells
PRIM22	5.789E-108	0.80751296	0.039	0.154	8.898E-104	T Cells
DPH71	6.331E-108	1.46100146	0.084	0.228	9.731E-104	T Cells
BUB32	9.483E-108	1.79900064	0.188	0.403	1.458E-103	T Cells
PUS73	1.031E-107	1.01641538	0.049	0.171	1.585E-103	T Cells
SLC25A23	1.205E-107	5.56143346	0.024	0.004	1.852E-103	T Cells
ZNF174	1.417E-107	0.61864187	0.033	0.144	2.178E-103	T Cells
AXIN12	1.512E-107	1.76575746	0.144	0.33	2.324E-103	T Cells
PLAGL22	1.606E-107	1.25559261	0.06	0.19	2.469E-103	T Cells
ADGRG52	2.391E-107	5.32343064	0.042	0.01	3.676E-103	T Cells
TTF1	2.666E-107	1.01292343	0.05	0.171	4.098E-103	T Cells
RAP1GAP21	3.095E-107	0.18542644	0.025	0.13	4.758E-103	T Cells
CPLANE1	3.174E-107	0.91152554	0.044	0.161	4.879E-103	T Cells
GADD45B4	3.528E-107	0.60961978	0.045	0.164	5.423E-103	T Cells
CDKN2C	3.972E-107	0.14210057	0.036	0.148	6.106E-103	T Cells
HAPLN33	4.357E-107	4.15976173	0.07	0.023	6.697E-103	T Cells
LBHD1	5.429E-107	0.48421113	0.032	0.141	8.345E-103	T Cells
ATP23	1.068E-106	0.36567244	0.031	0.14	1.642E-102	T Cells
ANXA2R	1.393E-106	4.58657434	0.101	0.042	2.141E-102	T Cells
BICRA	1.749E-106	1.41100307	0.077	0.214	2.688E-102	T Cells
TMEM2601	1.864E-106	1.21223938	0.057	0.182	2.865E-102	T Cells
METTL162	3.016E-106	1.45733229	0.079	0.218	4.636E-102	T Cells
LIF	3.542E-106	6.7326093	0.016	0.002	5.445E-102	T Cells
IDH22	4.77E-106	1.3866775	0.289	0.591	7.332E-102	T Cells
METRN	5.24E-106	0.59882726	0.033	0.142	8.054E-102	T Cells
PLCL21	5.523E-106	1.80038915	0.178	0.386	8.489E-102	T Cells
CRYZL11	5.971E-106	0.85594989	0.042	0.157	9.178E-102	T Cells
C1orf131	6.145E-106	0.89491191	0.04	0.155	9.445E-102	T Cells
TTC51	9.333E-106	1.34116097	0.075	0.211	1.435E-101	T Cells
RCOR32	1.082E-105	1.61067305	0.114	0.275	1.663E-101	T Cells
C16orf871	1.297E-105	1.29001118	0.056	0.181	1.994E-101	T Cells
NHEJ1	1.872E-105	1.45759977	0.064	0.195	2.877E-101	T Cells
USP212	1.875E-105	1.4153747	0.074	0.211	2.882E-101	T Cells
CDKN2AIP	2.357E-105	1.33772111	0.064	0.194	3.623E-101	T Cells
SCLY	4.339E-105	0.87447673	0.039	0.151	6.67E-101	T Cells
CISH1	4.5E-105	2.45072907	0.563	0.765	6.916E-101	T Cells
ZNF183	5.315E-105	0.80643878	0.041	0.155	8.17E-101	T Cells
RRAGB1	8.553E-105	0.42713829	0.026	0.13	1.315E-100	T Cells
RBM451	1.132E-104	0.91426413	0.042	0.157	1.741E-100	T Cells
IRF33	1.167E-104	1.5509191	0.306	0.63	1.795E-100	T Cells
SURF22	1.169E-104	1.29490206	0.065	0.194	1.796E-100	T Cells
NIBAN13	1.457E-104	1.42611567	0.347	0.716	2.239E-100	T Cells

STK383	1.48E-104	1.77082581	0.258	0.535	2.275E-100	T Cells
DCAF171	1.557E-104	1.39547708	0.074	0.211	2.393E-100	T Cells
TMEM801	1.64E-104	1.25900534	0.061	0.188	2.52E-100	T Cells
TTC21B1	2.046E-104	1.30655928	0.067	0.197	3.145E-100	T Cells
INTS52	2.757E-104	0.82063681	0.038	0.149	4.237E-100	T Cells
ZFP12	2.785E-104	0.78965634	0.034	0.144	4.281E-100	T Cells
SGSM21	3.728E-104	1.21793823	0.062	0.188	5.73E-100	T Cells
BRWD11	4.732E-104	1.36625716	0.072	0.204	7.274E-100	T Cells
TMEM161B1	5.026E-104	1.50587268	0.077	0.213	7.726E-100	T Cells
CDC37L11	5.303E-104	1.44096888	0.075	0.21	8.152E-100	T Cells
FLVCR11	5.446E-104	1.3107422	0.056	0.179	8.371E-100	T Cells
MIEF2	5.459E-104	0.25376222	0.022	0.123	8.391E-100	T Cells
BNIP1	5.521E-104	0.4549445	0.025	0.128	8.486E-100	T Cells
MTRF11	6.695E-104	1.31223529	0.063	0.191	1.029E-99	T Cells
NEURL41	7.919E-104	0.53219254	0.03	0.136	1.217E-99	T Cells
TIFA2	1.12E-103	1.09245037	0.046	0.164	1.722E-99	T Cells
PAPOLG1	1.244E-103	1.63859458	0.102	0.255	1.913E-99	T Cells
NAA351	1.309E-103	1.57924468	0.087	0.23	2.012E-99	T Cells
ZNF555	1.353E-103	0.57041051	0.028	0.132	2.079E-99	T Cells
GTPBP32	1.542E-103	1.20426488	0.05	0.17	2.37E-99	T Cells
YTHDC21	1.553E-103	1.69777052	0.147	0.33	2.387E-99	T Cells
PTPN222	1.633E-103	1.74453668	0.272	0.56	2.51E-99	T Cells
DNAJB41	1.633E-103	0.68183073	0.035	0.144	2.511E-99	T Cells
TGFBR21	1.911E-103	0.43954096	0.455	0.95	2.937E-99	T Cells
ZBTB372	3.793E-103	1.55946301	0.092	0.238	5.83E-99	T Cells
SFXN2	4.728E-103	0.76051822	0.033	0.141	7.267E-99	T Cells
ARL3	4.821E-103	1.44424979	0.063	0.191	7.41E-99	T Cells
ZNF2602	7.704E-103	1.05938101	0.041	0.154	1.1842E-98	T Cells
WWP11	7.818E-103	1.75438358	0.11	0.267	1.2016E-98	T Cells
TCF19	1.789E-102	0.31234507	0.05	0.168	2.7503E-98	T Cells
SLC25A373	2.103E-102	0.98952497	0.06	0.184	3.2329E-98	T Cells
CSAD1	2.249E-102	0.75982317	0.044	0.157	3.4564E-98	T Cells
CTPS12	2.666E-102	1.31459945	0.065	0.192	4.0974E-98	T Cells
MAZ	3.04E-102	1.21475506	0.068	0.197	4.6735E-98	T Cells
C1orf1091	3.339E-102	0.4837141	0.026	0.128	5.1321E-98	T Cells
SIMC12	3.42E-102	0.95771193	0.042	0.155	5.2574E-98	T Cells
HERC11	3.909E-102	1.77656333	0.187	0.398	6.0088E-98	T Cells
ZNF1401	4.1E-102	0.56608286	0.03	0.135	6.3017E-98	T Cells
UBR23	4.718E-102	1.40892735	0.353	0.729	7.2522E-98	T Cells
METTL151	5.502E-102	1.25401561	0.054	0.175	8.4575E-98	T Cells
TTPAL1	6.331E-102	0.68013681	0.034	0.142	9.7319E-98	T Cells
LETM11	7.02E-102	1.09151437	0.048	0.164	1.0791E-97	T Cells
ZNF2341	7.32E-102	0.63794608	0.032	0.138	1.1251E-97	T Cells
SUZ122	7.336E-102	1.84334136	0.198	0.416	1.1276E-97	T Cells
ATM3	9.032E-102	1.62981402	0.314	0.643	1.3883E-97	T Cells
BIN31	9.56E-102	0.52298373	0.031	0.136	1.4694E-97	T Cells
ZNF4801	1.002E-101	0.80916955	0.033	0.139	1.5395E-97	T Cells
SKP21	1.243E-101	0.46731839	0.03	0.134	1.9111E-97	T Cells
CCNB1IP12	1.262E-101	1.30846385	0.059	0.183	1.9404E-97	T Cells
CES4A	1.383E-101	0.53776797	0.035	0.141	2.1254E-97	T Cells
SIRT13	1.631E-101	1.59331596	0.087	0.227	2.5077E-97	T Cells
ZNF3831	1.672E-101	1.02348954	0.044	0.157	2.5704E-97	T Cells

NUDT16L12	2.061E-101	1.54377089	0.078	0.215	3.1676E-97	T Cells
TMEM186	2.087E-101	0.6269816	0.033	0.14	3.2072E-97	T Cells
AGPAT53	3.034E-101	1.49950825	0.07	0.201	4.6639E-97	T Cells
SOCS71	3.056E-101	0.89197822	0.041	0.152	4.6978E-97	T Cells
NDUFAF51	3.503E-101	0.84805444	0.034	0.141	5.3838E-97	T Cells
LYRM41	3.566E-101	0.77252342	0.037	0.146	5.482E-97	T Cells
TESK22	3.76E-101	0.12495855	0.021	0.118	5.7788E-97	T Cells
TCP11L11	4.338E-101	1.61753649	0.114	0.271	6.6677E-97	T Cells
POLR3B	5.841E-101	0.87569492	0.036	0.144	8.9785E-97	T Cells
CARD162	6.165E-101	0.41366259	0.026	0.127	9.4766E-97	T Cells
ZNF3041	6.798E-101	1.02672081	0.044	0.158	1.045E-96	T Cells
LTB4R1	7.518E-101	1.46711933	0.166	0.359	1.1556E-96	T Cells
RNASEH2B1	8.855E-101	1.53362463	0.072	0.203	1.3611E-96	T Cells
POMT11	1.141E-100	0.68529939	0.031	0.136	1.7533E-96	T Cells
DTNB1	1.323E-100	1.17952012	0.061	0.184	2.0331E-96	T Cells
SUV39H1	1.693E-100	1.00799381	0.037	0.146	2.6025E-96	T Cells
DBN12	1.706E-100	5.43359547	0.019	0.002	2.6224E-96	T Cells
ZSCAN262	1.861E-100	1.35095145	0.058	0.181	2.8605E-96	T Cells
ENTPD42	1.952E-100	1.77605011	0.157	0.344	3.001E-96	T Cells
IVNS1ABP1	2.069E-100	1.39331924	0.642	0.944	3.1797E-96	T Cells
C6orf2261	2.521E-100	0.4719388	0.03	0.133	3.875E-96	T Cells
ENTR13	3.368E-100	1.63796397	0.098	0.246	5.1763E-96	T Cells
SNAPC41	3.615E-100	1.1980294	0.058	0.18	5.5567E-96	T Cells
TMEM1431	4.372E-100	0.49172314	0.026	0.127	6.7207E-96	T Cells
MEGF81	5.013E-100	0.39863468	0.026	0.127	7.7058E-96	T Cells
STAG12	5.089E-100	1.75381627	0.24	0.491	7.8218E-96	T Cells
INPP5E1	6.688E-100	1.3846423	0.076	0.208	1.028E-95	T Cells
SPATA2L2	1.045E-99	0.60512409	0.031	0.134	1.6069E-95	T Cells
GIMAP41	1.073E-99	1.59306848	0.334	0.687	1.6497E-95	T Cells
MFSD82	1.205E-99	1.58979714	0.074	0.206	1.8514E-95	T Cells
RHOT21	1.272E-99	1.76262902	0.16	0.348	1.955E-95	T Cells
LUC7L34	1.581E-99	3.00288576	0.463	0.506	2.4305E-95	T Cells
NISCH1	1.662E-99	1.8931091	0.175	0.374	2.5544E-95	T Cells
MTA31	1.856E-99	1.07608488	0.043	0.154	2.8525E-95	T Cells
ZNF770	1.95E-99	1.15191417	0.045	0.158	2.9981E-95	T Cells
RAB33B	2.486E-99	1.77986298	0.088	0.228	3.8214E-95	T Cells
TIGD22	2.886E-99	0.32381069	0.02	0.116	4.4356E-95	T Cells
PHF101	3.517E-99	1.89794822	0.215	0.448	5.4054E-95	T Cells
EEF2KMT	3.711E-99	0.28419778	0.022	0.118	5.7037E-95	T Cells
BAG22	3.751E-99	0.72512235	0.035	0.141	5.7651E-95	T Cells
VGLL4	4.505E-99	0.57250906	0.031	0.133	6.9244E-95	T Cells
ATP5PO3	5.03E-99	0.50726987	0.029	0.13	7.7318E-95	T Cells
SETD1A2	6.369E-99	1.17626065	0.05	0.166	9.79E-95	T Cells
KIAA20261	6.511E-99	1.77116358	0.266	0.545	1.0007E-94	T Cells
STX1A1	8.376E-99	5.43760535	0.034	0.007	1.2875E-94	T Cells
AMPD35	8.426E-99	4.30906718	0.053	0.015	1.2952E-94	T Cells
ZNF2631	9.786E-99	1.31802776	0.06	0.182	1.5042E-94	T Cells
TYSND11	1.0499E-98	0.46655073	0.031	0.133	1.6139E-94	T Cells
IL6R5	1.1337E-98	1.18915573	0.061	0.183	1.7425E-94	T Cells
TUT42	1.4815E-98	1.8436809	0.209	0.435	2.2772E-94	T Cells
C12orf49	1.6466E-98	0.58159055	0.029	0.13	2.5309E-94	T Cells
HAGHL1	1.8714E-98	1.15282435	0.049	0.165	2.8765E-94	T Cells

ZDHHC13	2.0549E-98	1.64946149	0.09	0.23	3.1586E-94	T Cells
QRSL12	2.6762E-98	0.51447302	0.028	0.128	4.1135E-94	T Cells
AAK11	2.946E-98	1.49338369	0.35	0.717	4.5283E-94	T Cells
EMC9	3.9774E-98	1.06014416	0.043	0.154	6.1136E-94	T Cells
ZSWIM9	4.4388E-98	1.41495897	0.065	0.189	6.8228E-94	T Cells
NAP1L41	4.9171E-98	1.82638421	0.237	0.486	7.558E-94	T Cells
BCAS32	5.945E-98	1.43951434	0.069	0.195	9.1381E-94	T Cells
ZNF5662	6.6591E-98	1.02713631	0.04	0.148	1.0236E-93	T Cells
C14orf932	8.1512E-98	0.65269383	0.033	0.136	1.2529E-93	T Cells
SMIM132	8.1702E-98	0.66249473	0.027	0.126	1.2558E-93	T Cells
HIST1H1C2	1.0182E-97	2.60258171	0.449	0.464	1.5652E-93	T Cells
ZNF843	1.4215E-97	1.07917546	0.05	0.164	2.1851E-93	T Cells
ZNF161	1.4332E-97	0.1378374	0.022	0.117	2.203E-93	T Cells
SNRPD12	1.5078E-97	0.99567524	0.042	0.152	2.3177E-93	T Cells
CWF19L2	1.8936E-97	1.8353102	0.109	0.261	2.9107E-93	T Cells
ANKRD13C2	1.9967E-97	1.36018163	0.061	0.182	3.0692E-93	T Cells
NUFIP1	2.4182E-97	0.13622129	0.021	0.116	3.717E-93	T Cells
DUSP122	2.4626E-97	1.41647168	0.065	0.189	3.7852E-93	T Cells
INO80D1	2.6015E-97	1.77487472	0.118	0.277	3.9988E-93	T Cells
ST3GAL32	3.0929E-97	1.39379641	0.056	0.174	4.754E-93	T Cells
PPP2R5C1	3.1232E-97	1.78667507	0.309	0.634	4.8006E-93	T Cells
ABHD181	3.3076E-97	1.61259355	0.093	0.236	5.0841E-93	T Cells
EEF22	4.2059E-97	0.83971601	0.663	0.972	6.4649E-93	T Cells
TPPP	4.3931E-97	1.11449201	0.073	0.201	6.7527E-93	T Cells
CCDC1741	4.8456E-97	1.17394848	0.046	0.157	7.4482E-93	T Cells
CKAP21	4.8714E-97	1.12899452	0.056	0.174	7.4879E-93	T Cells
C1orf1122	5.0939E-97	0.92741749	0.039	0.146	7.8299E-93	T Cells
SMIM27	6.6376E-97	1.4497844	0.063	0.186	1.0203E-92	T Cells
MBNL23	7.1275E-97	1.75067094	0.136	0.304	1.0956E-92	T Cells
ZNF621	7.3865E-97	1.55373702	0.08	0.212	1.1354E-92	T Cells
CDADC11	7.8428E-97	0.90891272	0.037	0.143	1.2055E-92	T Cells
UBXN2A1	8.7807E-97	1.263058	0.056	0.174	1.3497E-92	T Cells
ALG111	9.0189E-97	0.91951178	0.039	0.145	1.3863E-92	T Cells
RUNX33	9.4934E-97	3.99452254	0.216	0.139	1.4592E-92	T Cells
GPSM13	9.7207E-97	5.73158052	0.02	0.003	1.4942E-92	T Cells
CFAP971	1.1891E-96	1.84256141	0.121	0.28	1.8278E-92	T Cells
ZNF6882	1.3195E-96	0.73693056	0.032	0.134	2.0282E-92	T Cells
PMS11	1.369E-96	1.81320479	0.125	0.287	2.1043E-92	T Cells
HS3ST3B15	1.625E-96	4.38033596	0.072	0.026	2.4978E-92	T Cells
THAP31	1.6661E-96	0.83438208	0.038	0.143	2.561E-92	T Cells
L3MBTL31	1.6817E-96	1.44133707	0.068	0.193	2.585E-92	T Cells
USE11	1.6882E-96	0.77171797	0.042	0.149	2.595E-92	T Cells
CHIC11	2.1844E-96	5.2538628	0.04	0.01	3.3576E-92	T Cells
ZMYND191	2.3465E-96	1.30904265	0.051	0.165	3.6068E-92	T Cells
STAT5B2	2.7714E-96	1.87680449	0.227	0.468	4.2599E-92	T Cells
TRAM24	3.1584E-96	0.83739929	0.039	0.145	4.8548E-92	T Cells
RFC3	3.4705E-96	0.25424829	0.02	0.114	5.3345E-92	T Cells
DHRS4	3.5054E-96	0.22187591	0.022	0.117	5.3882E-92	T Cells
C12orf661	3.6243E-96	0.99256628	0.037	0.142	5.5709E-92	T Cells
ORC52	3.9482E-96	0.92914589	0.035	0.139	6.0688E-92	T Cells
HACL11	4.3307E-96	0.57807011	0.029	0.129	6.6568E-92	T Cells
RTL6	4.8768E-96	1.62411735	0.077	0.208	7.4961E-92	T Cells

PLCD11	4.9096E-96	1.73178831	0.09	0.228	7.5466E-92	T Cells
BTG24	5.2331E-96	1.07004924	0.378	0.767	8.0438E-92	T Cells
TRPS13	5.2405E-96	1.68592217	0.167	0.356	8.0552E-92	T Cells
ASF1A1	5.5047E-96	1.78774639	0.115	0.269	8.4613E-92	T Cells
RALGDS3	5.5346E-96	1.63598504	0.268	0.544	8.5072E-92	T Cells
PHF21A3	6.4513E-96	1.73379956	0.127	0.288	9.9162E-92	T Cells
RAI1	7.3525E-96	1.43121411	0.067	0.19	1.1302E-91	T Cells
RBM431	7.483E-96	1.23395018	0.044	0.153	1.1502E-91	T Cells
ISCA2	7.5877E-96	0.72890564	0.03	0.131	1.1663E-91	T Cells
BTN2A11	7.714E-96	1.31707024	0.054	0.171	1.1857E-91	T Cells
EPB41L52	9.6273E-96	0.46911638	0.027	0.125	1.4798E-91	T Cells
CUL72	1.0919E-95	0.50709106	0.027	0.125	1.6783E-91	T Cells
WDR252	1.2589E-95	0.91033641	0.033	0.135	1.935E-91	T Cells
IKZF5	1.3573E-95	1.83788388	0.111	0.262	2.0862E-91	T Cells
ZWILCH1	1.6857E-95	1.00636875	0.042	0.149	2.5911E-91	T Cells
PIGM1	1.7006E-95	1.3497725	0.057	0.175	2.614E-91	T Cells
WWC31	1.863E-95	1.8303142	0.128	0.29	2.8636E-91	T Cells
SGTB4	2.1752E-95	1.64493575	0.08	0.211	3.3435E-91	T Cells
CLYBL1	2.4946E-95	0.96800341	0.037	0.142	3.8344E-91	T Cells
MSH51	2.5339E-95	0.81378951	0.036	0.14	3.8949E-91	T Cells
RNF2162	2.8459E-95	1.28054403	0.054	0.169	4.3744E-91	T Cells
ZKSCAN4	3.7751E-95	0.7088265	0.031	0.131	5.8027E-91	T Cells
EPS8L23	3.8221E-95	4.09481065	0.023	0.004	5.875E-91	T Cells
RBM15	3.9946E-95	1.77794396	0.204	0.421	6.1401E-91	T Cells
LRIG21	4.1184E-95	1.75219291	0.103	0.249	6.3304E-91	T Cells
ATPSCKMT1	4.3071E-95	0.37697911	0.024	0.119	6.6205E-91	T Cells
ZNF7761	4.913E-95	1.33538939	0.048	0.16	7.5517E-91	T Cells
HECTD4	6.1168E-95	1.80461845	0.156	0.337	9.4021E-91	T Cells
PLA2G61	6.9543E-95	1.57034898	0.093	0.231	1.0689E-90	T Cells
CUL91	1.1852E-94	1.08542329	0.05	0.161	1.8218E-90	T Cells
SRSF11	1.6434E-94	1.78133404	0.114	0.267	2.5261E-90	T Cells
DAAM13	1.6844E-94	0.93800621	0.044	0.152	2.589E-90	T Cells
LYSMD41	1.9494E-94	1.02489361	0.048	0.158	2.9964E-90	T Cells
UTP202	2.0186E-94	1.08971222	0.049	0.16	3.1027E-90	T Cells
PARP161	2.1347E-94	1.30461762	0.047	0.157	3.2812E-90	T Cells
ZNF6281	2.143E-94	0.82148033	0.032	0.132	3.294E-90	T Cells
ZNF2672	2.7758E-94	1.76554012	0.098	0.241	4.2666E-90	T Cells
RASSF72	4.7997E-94	1.45929213	0.085	0.217	7.3776E-90	T Cells
SAR1A	5.0824E-94	0.11188523	0.019	0.109	7.8122E-90	T Cells
ADAR	5.396E-94	0.49162602	0.455	0.946	8.2942E-90	T Cells
ITGB3BP1	5.9356E-94	0.98538563	0.036	0.138	9.1235E-90	T Cells
CAPN7	6.7234E-94	1.76309298	0.112	0.262	1.0335E-89	T Cells
ING1	6.8656E-94	0.79914052	0.033	0.133	1.0553E-89	T Cells
TADA2A1	7.2906E-94	1.34633719	0.064	0.185	1.1206E-89	T Cells
ST7L	8.7509E-94	0.77880971	0.036	0.138	1.3451E-89	T Cells
PITPNM21	1.1241E-93	5.74535078	0.031	0.007	1.7279E-89	T Cells
DDX3Y2	1.5433E-93	1.4477863	0.12	0.274	2.3722E-89	T Cells
KTI121	1.6793E-93	0.90154351	0.033	0.133	2.5813E-89	T Cells
NANP2	1.7796E-93	0.15022161	0.021	0.112	2.7355E-89	T Cells
SEL1L32	2.1499E-93	1.65004097	0.298	0.598	3.3047E-89	T Cells
HAUS81	2.1583E-93	0.54354673	0.023	0.116	3.3175E-89	T Cells
CARHSP1	2.1949E-93	1.46606635	0.09	0.225	3.3738E-89	T Cells

TRMT442	2.4823E-93	1.01285961	0.042	0.148	3.8155E-89	T Cells
LMF1	2.5268E-93	1.49662797	0.066	0.187	3.8839E-89	T Cells
DNAL11	2.6452E-93	0.3672527	0.02	0.111	4.0659E-89	T Cells
HAUS61	2.7901E-93	1.65579175	0.081	0.211	4.2886E-89	T Cells
SHMT21	3.0737E-93	0.88077549	0.033	0.134	4.7246E-89	T Cells
ELMOD3	3.384E-93	0.78234765	0.04	0.144	5.2015E-89	T Cells
ERCC62	6.4781E-93	1.42819291	0.073	0.199	9.9575E-89	T Cells
SIRT51	7.4158E-93	0.90202585	0.04	0.144	1.1399E-88	T Cells
HMBOX12	7.5224E-93	1.72457144	0.128	0.287	1.1563E-88	T Cells
MRI11	7.7307E-93	1.10249134	0.043	0.149	1.1883E-88	T Cells
GALNT41	1.2191E-92	1.7123871	0.09	0.225	1.8739E-88	T Cells
TMTC31	1.3869E-92	1.11420184	0.034	0.134	2.1318E-88	T Cells
TUBGCP62	1.493E-92	1.73814446	0.199	0.41	2.2949E-88	T Cells
DDX512	1.9564E-92	0.96171251	0.036	0.137	3.0071E-88	T Cells
RIF12	2.4268E-92	1.77663838	0.115	0.265	3.7302E-88	T Cells
ICE21	2.6697E-92	1.44509018	0.066	0.186	4.1035E-88	T Cells
NDOR11	3.0662E-92	0.80100798	0.042	0.147	4.713E-88	T Cells
TMEM201	4.3153E-92	0.65610379	0.029	0.125	6.6331E-88	T Cells
SMURF22	4.872E-92	1.8602351	0.157	0.337	7.4887E-88	T Cells
SIGIRR3	5.3093E-92	3.84168621	0.247	0.174	8.1609E-88	T Cells
ZHX31	5.5934E-92	0.41416154	0.022	0.114	8.5977E-88	T Cells
TDRKH	5.9647E-92	1.68874558	0.077	0.203	9.1683E-88	T Cells
ZNF5431	6.0446E-92	0.98098891	0.039	0.141	9.2912E-88	T Cells
MYCBP21	8.4784E-92	1.90005158	0.173	0.363	1.3032E-87	T Cells
CSTF2	9.1806E-92	0.65962879	0.033	0.132	1.4111E-87	T Cells
ZNF75A1	9.7167E-92	1.62031861	0.096	0.234	1.4936E-87	T Cells
MED161	1.1758E-91	1.68182065	0.103	0.246	1.8073E-87	T Cells
ZFP821	1.2427E-91	0.64645591	0.026	0.12	1.9101E-87	T Cells
CEP891	1.2648E-91	1.40322875	0.063	0.181	1.9442E-87	T Cells
NBEAL12	1.307E-91	0.8697995	0.037	0.139	2.009E-87	T Cells
ITGAL	1.3136E-91	0.51761932	0.453	0.937	2.0191E-87	T Cells
ZNF1362	1.5019E-91	1.13022272	0.052	0.162	2.3085E-87	T Cells
ZNF7082	1.5085E-91	1.49400653	0.08	0.207	2.3187E-87	T Cells
TNFAIP11	1.9228E-91	0.60117565	0.028	0.123	2.9556E-87	T Cells
CCHCR11	2.0404E-91	0.84525584	0.033	0.131	3.1364E-87	T Cells
PGM2L11	2.1585E-91	1.35837065	0.054	0.166	3.3179E-87	T Cells
ATF7IP1	2.4745E-91	1.87340565	0.233	0.47	3.8036E-87	T Cells
TNKS1	2.7952E-91	1.85564369	0.121	0.276	4.2965E-87	T Cells
MSTO11	4.0549E-91	1.72536277	0.08	0.209	6.2328E-87	T Cells
BORA2	5.935E-91	0.80003916	0.028	0.124	9.1227E-87	T Cells
SOS11	6.2795E-91	1.98144908	0.203	0.413	9.6522E-87	T Cells
DLG31	8.506E-91	5.41718271	0.032	0.007	1.3075E-86	T Cells
SLC25A512	9.4809E-91	1.42172544	0.055	0.166	1.4573E-86	T Cells
SLX42	9.8916E-91	1.00084114	0.041	0.144	1.5204E-86	T Cells
IFI164	1.0051E-90	1.13917132	0.408	0.843	1.545E-86	T Cells
MTRF1L2	1.2925E-90	0.71707346	0.029	0.124	1.9867E-86	T Cells
ACCS1	1.4166E-90	0.56796576	0.031	0.127	2.1774E-86	T Cells
RBIS1	1.5026E-90	0.61525839	0.027	0.12	2.3096E-86	T Cells
HELQ2	1.5155E-90	1.82783484	0.095	0.233	2.3295E-86	T Cells
GABPB2	1.6715E-90	1.74473069	0.096	0.234	2.5692E-86	T Cells
RINT12	1.8997E-90	1.60429957	0.074	0.197	2.9201E-86	T Cells
SMG61	2.5087E-90	1.49033734	0.069	0.189	3.8561E-86	T Cells

TFB1M2	2.5863E-90	1.37421262	0.058	0.171	3.9753E-86	T Cells
PAXIP1	2.7349E-90	1.0395755	0.037	0.137	4.2039E-86	T Cells
PAG11	2.8322E-90	1.24233505	0.39	0.797	4.3533E-86	T Cells
KAT14	3.0233E-90	0.54165707	0.028	0.122	4.647E-86	T Cells
DEAF12	3.0503E-90	1.62630835	0.093	0.229	4.6886E-86	T Cells
TBCC	3.2542E-90	1.94537582	0.142	0.309	5.002E-86	T Cells
AEN	3.65E-90	1.66846888	0.086	0.217	5.6104E-86	T Cells
TRIM31	3.6883E-90	0.97813883	0.032	0.128	5.6693E-86	T Cells
CSNK1G32	3.8314E-90	1.5344864	0.067	0.185	5.8892E-86	T Cells
CAMKMT2	4.0462E-90	1.45277943	0.064	0.181	6.2194E-86	T Cells
ATG102	5.3642E-90	0.69068024	0.033	0.13	8.2453E-86	T Cells
FUT101	5.6656E-90	0.7441635	0.03	0.126	8.7086E-86	T Cells
ZNF4391	6.3012E-90	0.51561542	0.027	0.119	9.6855E-86	T Cells
CNNM3	7.5862E-90	1.60571904	0.08	0.206	1.1661E-85	T Cells
ABTB12	9.2244E-90	1.5454347	0.089	0.221	1.4179E-85	T Cells
GDAP13	1.3392E-89	0.49471596	0.03	0.125	2.0585E-85	T Cells
BMT2	1.4521E-89	1.320472	0.044	0.149	2.232E-85	T Cells
NR2C11	1.8502E-89	1.09207877	0.05	0.158	2.844E-85	T Cells
MARCH73	1.9505E-89	1.20292051	0.401	0.831	2.9982E-85	T Cells
CRADD1	2.2915E-89	1.45255224	0.06	0.175	3.5222E-85	T Cells
PGBD21	2.4277E-89	1.24860237	0.044	0.148	3.7316E-85	T Cells
SKIV2L1	3.0466E-89	0.26513943	0.02	0.107	4.6829E-85	T Cells
TMEM2672	3.3518E-89	1.44579538	0.058	0.171	5.152E-85	T Cells
CEP1312	3.3834E-89	0.58317658	0.028	0.121	5.2007E-85	T Cells
SETD61	4.2167E-89	1.2614972	0.052	0.16	6.4815E-85	T Cells
F81	5.9311E-89	0.2010298	0.021	0.109	9.1167E-85	T Cells
TFB2M	6.8509E-89	0.62143939	0.024	0.114	1.053E-84	T Cells
UBXN71	6.9876E-89	1.89449102	0.131	0.29	1.0741E-84	T Cells
ZNF231	7.9241E-89	0.74311303	0.031	0.126	1.218E-84	T Cells
PLEKHA81	8.4126E-89	0.74824792	0.031	0.125	1.2931E-84	T Cells
RNF2152	1.0055E-88	0.10228429	0.019	0.105	1.5456E-84	T Cells
ZNF4961	1.0286E-88	0.5101831	0.03	0.123	1.5811E-84	T Cells
ZNF6191	1.0956E-88	0.92763901	0.03	0.124	1.6841E-84	T Cells
YBEY	1.3635E-88	0.78219301	0.031	0.125	2.0958E-84	T Cells
GSPT2	1.4316E-88	0.70280061	0.029	0.122	2.2005E-84	T Cells
KLF93	2.0914E-88	1.8742242	0.153	0.327	3.2147E-84	T Cells
MDM12	2.7035E-88	1.50670668	0.061	0.176	4.1555E-84	T Cells
PSMG4	2.7506E-88	1.69661472	0.076	0.199	4.228E-84	T Cells
BIRC61	3.3458E-88	1.70228029	0.305	0.61	5.1429E-84	T Cells
PLEKHA11	5.5347E-88	1.36218646	0.05	0.158	8.5074E-84	T Cells
DUS4L1	6.2095E-88	0.56518398	0.023	0.112	9.5446E-84	T Cells
CASTOR21	6.3272E-88	0.18320515	0.021	0.109	9.7256E-84	T Cells
VRK11	6.4895E-88	1.81125865	0.116	0.263	9.975E-84	T Cells
CDK2	7.2885E-88	1.41094692	0.058	0.17	1.1203E-83	T Cells
FAM133B2	7.688E-88	1.26981757	0.055	0.164	1.1817E-83	T Cells
LCLAT11	7.7183E-88	1.31202475	0.053	0.16	1.1864E-83	T Cells
PIGB1	9.3295E-88	1.10964995	0.045	0.149	1.434E-83	T Cells
WDPCP2	1.4067E-87	0.33324752	0.022	0.11	2.1623E-83	T Cells
EPPK1	1.4216E-87	5.00568345	0.012	0.001	2.1851E-83	T Cells
FLNB1	1.4884E-87	0.84770796	0.038	0.137	2.2878E-83	T Cells
ANKRD371	1.5344E-87	0.58549168	0.026	0.117	2.3585E-83	T Cells
FITM21	1.7254E-87	0.62268501	0.027	0.119	2.6522E-83	T Cells

CRACR2A1	1.9281E-87	4.19230054	0.138	0.073	2.9637E-83	T Cells
USP313	2.2001E-87	0.48229102	0.023	0.112	3.3818E-83	T Cells
RLF1	2.3564E-87	1.69730518	0.083	0.21	3.622E-83	T Cells
TNFSF43	2.4454E-87	5.54228506	0.027	0.005	3.7589E-83	T Cells
TIMM291	2.8105E-87	1.28368646	0.051	0.157	4.32E-83	T Cells
SP2	3.3385E-87	1.97306316	0.187	0.383	5.1315E-83	T Cells
LTBP31	4.3999E-87	1.95822409	0.188	0.383	6.7631E-83	T Cells
SWSAP1	4.9091E-87	0.2444334	0.021	0.107	7.5458E-83	T Cells
SMYD41	5.0095E-87	1.6808399	0.077	0.2	7.7002E-83	T Cells
NDUFAF61	5.7404E-87	1.04868749	0.04	0.139	8.8236E-83	T Cells
THAP52	1.0322E-86	1.47633692	0.052	0.158	1.5866E-82	T Cells
ZNF2121	1.2252E-86	0.98192153	0.037	0.134	1.8832E-82	T Cells
ZNF5502	1.3262E-86	1.30448598	0.07	0.187	2.0385E-82	T Cells
MTG12	1.3853E-86	1.82849948	0.141	0.304	2.1294E-82	T Cells
RPUSD21	1.4323E-86	0.68356294	0.029	0.12	2.2016E-82	T Cells
TRMU1	1.4338E-86	1.70058751	0.097	0.231	2.2039E-82	T Cells
ALDH18A1	1.6107E-86	1.46029265	0.055	0.165	2.4758E-82	T Cells
IKBKB4	3.0952E-86	1.50427715	0.347	0.694	4.7576E-82	T Cells
HACE1	3.472E-86	1.50743921	0.059	0.17	5.3368E-82	T Cells
ZNF627	5.0166E-86	0.7702319	0.032	0.126	7.711E-82	T Cells
TBCE2	6.0183E-86	0.93266995	0.03	0.123	9.2507E-82	T Cells
ANKRA22	6.1181E-86	1.32106691	0.048	0.151	9.4042E-82	T Cells
MYPOP1	6.9954E-86	0.49841254	0.026	0.116	1.0753E-81	T Cells
UIMC1	7.2041E-86	1.67359565	0.077	0.201	1.1073E-81	T Cells
LYRM7	9.2007E-86	0.76322877	0.029	0.121	1.4142E-81	T Cells
ZNF341	9.7676E-86	0.66937386	0.026	0.114	1.5014E-81	T Cells
INSIG12	1.0067E-85	1.34291863	0.182	0.368	1.5474E-81	T Cells
ZNF5731	1.2673E-85	1.37960339	0.056	0.165	1.948E-81	T Cells
MINDY21	1.27E-85	1.80823776	0.108	0.247	1.9521E-81	T Cells
FAM207A	1.6279E-85	1.18801303	0.045	0.147	2.5023E-81	T Cells
APOL31	2.3039E-85	1.60602926	0.113	0.256	3.5413E-81	T Cells
SP1402	2.4203E-85	1.8930323	0.23	0.458	3.7202E-81	T Cells
PCYOX1L	2.7008E-85	1.69361503	0.098	0.231	4.1513E-81	T Cells
STK43	2.8966E-85	2.03770362	0.578	0.846	4.4524E-81	T Cells
TP53I131	2.9126E-85	1.35578234	0.073	0.191	4.4769E-81	T Cells
PRPSAP2	3.008E-85	1.81386653	0.087	0.214	4.6236E-81	T Cells
CDKN2AIPNL2	3.1628E-85	0.88766638	0.031	0.123	4.8616E-81	T Cells
CD53	3.2831E-85	0.1444442	0.489	0.963	5.0464E-81	T Cells
ARHGAP153	5.3082E-85	2.9321669	0.451	0.497	8.1592E-81	T Cells
SLC35A21	6.0114E-85	0.48387181	0.022	0.108	9.2401E-81	T Cells
FANCF1	6.193E-85	1.69053603	0.079	0.2	9.5192E-81	T Cells
SRGAP32	7.7374E-85	5.21792194	0.029	0.006	1.1893E-80	T Cells
XRN11	8.0535E-85	1.76220443	0.299	0.595	1.2379E-80	T Cells
DUSP28	8.7564E-85	0.35248134	0.024	0.112	1.3459E-80	T Cells
PLK34	1.1143E-84	1.61818471	0.148	0.312	1.7128E-80	T Cells
SYNJ23	1.3491E-84	5.04082273	0.033	0.008	2.0737E-80	T Cells
PIBF11	1.3784E-84	1.57985173	0.063	0.175	2.1188E-80	T Cells
CAB39L2	1.4613E-84	0.36429026	0.019	0.102	2.2462E-80	T Cells
CSRNP21	1.499E-84	1.04784969	0.039	0.137	2.3042E-80	T Cells
CEP2952	1.5052E-84	0.72392098	0.027	0.117	2.3137E-80	T Cells
TTC381	1.5938E-84	0.51427166	0.021	0.107	2.4498E-80	T Cells
F8A1	1.7514E-84	1.09292809	0.043	0.142	2.6921E-80	T Cells

PSPC11	2.1882E-84	1.81416801	0.246	0.488	3.3635E-80	T Cells
ZNF1951	2.2185E-84	1.2728598	0.046	0.147	3.4101E-80	T Cells
ZNF8081	2.3626E-84	1.85696982	0.088	0.216	3.6315E-80	T Cells
ZSCAN251	2.4329E-84	0.97123944	0.04	0.138	3.7396E-80	T Cells
ZNF8391	2.4344E-84	1.09627176	0.047	0.148	3.7419E-80	T Cells
SOCS21	2.5896E-84	2.1201683	0.149	0.315	3.9805E-80	T Cells
CDKN2D2	2.7935E-84	1.82105023	0.164	0.339	4.2939E-80	T Cells
SLC2A4RG	2.9307E-84	1.82299924	0.219	0.437	4.5048E-80	T Cells
IARS2	3.0305E-84	1.81042027	0.076	0.196	4.6583E-80	T Cells
DPY19L31	3.1723E-84	0.88466007	0.033	0.126	4.8761E-80	T Cells
LRWD11	4.5111E-84	0.81678142	0.03	0.121	6.934E-80	T Cells
PPARA2	5.1438E-84	1.15555268	0.043	0.142	7.9065E-80	T Cells
CABP42	8.9014E-84	0.31662655	0.023	0.108	1.3682E-79	T Cells
GEMIN81	1.0316E-83	0.47935836	0.023	0.109	1.5857E-79	T Cells
NUSAP1	1.1561E-83	0.3278012	0.048	0.148	1.777E-79	T Cells
ZNF510	1.1915E-83	1.37012514	0.048	0.149	1.8314E-79	T Cells
ATG2B1	1.3795E-83	1.85294321	0.103	0.238	2.1205E-79	T Cells
SLC2A132	1.4558E-83	0.90476424	0.027	0.115	2.2377E-79	T Cells
LMNB13	1.4719E-83	1.35359275	0.114	0.254	2.2625E-79	T Cells
EDEM14	1.4827E-83	1.12893261	0.404	0.824	2.2791E-79	T Cells
ZNF262	1.6229E-83	1.48646703	0.066	0.179	2.4946E-79	T Cells
EHBP11	1.678E-83	1.51017515	0.06	0.169	2.5792E-79	T Cells
ZNF7872	1.7532E-83	0.36587565	0.026	0.114	2.6949E-79	T Cells
PHF15	2.0073E-83	1.89804374	0.251	0.5	3.0854E-79	T Cells
TATDN11	2.633E-83	0.93073984	0.031	0.122	4.0472E-79	T Cells
G2E31	2.7268E-83	1.50169636	0.05	0.153	4.1913E-79	T Cells
PSTPIP23	2.7527E-83	0.67353305	0.046	0.146	4.2312E-79	T Cells
ANGEL21	3.3381E-83	1.15933241	0.043	0.141	5.131E-79	T Cells
EP4001	3.5062E-83	1.1790647	0.039	0.135	5.3894E-79	T Cells
FADS31	4.1262E-83	0.1623722	0.02	0.103	6.3425E-79	T Cells
RBM8A2	4.2756E-83	1.16126012	0.038	0.133	6.5721E-79	T Cells
ADPRM	4.53E-83	1.70204429	0.067	0.181	6.963E-79	T Cells
NCS12	4.6601E-83	5.24998935	0.015	0.002	7.163E-79	T Cells
PUS101	5.1419E-83	0.33141172	0.023	0.108	7.9036E-79	T Cells
SPATA51	5.2349E-83	1.79648675	0.094	0.224	8.0466E-79	T Cells
MCM6	7.2567E-83	1.6050794	0.135	0.29	1.1154E-78	T Cells
COLGALT2	8.186E-83	6.37785596	0.016	0.002	1.2583E-78	T Cells
CEP833	8.6174E-83	1.49071382	0.067	0.179	1.3246E-78	T Cells
RTN4IP12	1.123E-82	0.58427642	0.02	0.103	1.7261E-78	T Cells
ZNF832	2.2309E-82	1.83069959	0.114	0.254	3.4291E-78	T Cells
PORCN	2.7036E-82	1.05578422	0.037	0.131	4.1558E-78	T Cells
PFKM	3.0445E-82	0.17760345	0.021	0.103	4.6797E-78	T Cells
BOLA3	3.9544E-82	0.82336566	0.025	0.11	6.0782E-78	T Cells
ZSCAN92	4.4052E-82	0.92794402	0.033	0.124	6.7713E-78	T Cells
HAUS5	5.0531E-82	1.11873437	0.042	0.139	7.7671E-78	T Cells
SMC51	5.7404E-82	1.57245211	0.065	0.176	8.8236E-78	T Cells
SAAL1	7.9235E-82	0.66506176	0.024	0.109	1.2179E-77	T Cells
UPF3B2	8.1446E-82	1.16273359	0.045	0.143	1.2519E-77	T Cells
SLX4IP	1.0175E-81	0.57265959	0.026	0.112	1.564E-77	T Cells
ZSWIM5	1.064E-81	6.8626452	0.01	0.001	1.6355E-77	T Cells
APPL22	1.0701E-81	1.54490132	0.073	0.188	1.6448E-77	T Cells
POFUT21	1.4256E-81	1.3362324	0.061	0.169	2.1913E-77	T Cells

PMEPA1	1.7068E-81	0.95358567	0.037	0.13	2.6236E-77	T Cells
MED311	1.8928E-81	0.730212	0.026	0.112	2.9095E-77	T Cells
TXNL4B2	2.3351E-81	1.29568945	0.049	0.149	3.5892E-77	T Cells
ID22	2.7636E-81	1.41421738	0.34	0.666	4.2479E-77	T Cells
ODF21	2.7797E-81	1.58332483	0.076	0.193	4.2727E-77	T Cells
TBC1D24	2.8432E-81	0.82085165	0.029	0.117	4.3703E-77	T Cells
MARS3	3.4737E-81	1.52862228	0.06	0.166	5.3395E-77	T Cells
BTN3A11	3.9283E-81	1.9420991	0.167	0.342	6.0382E-77	T Cells
C2orf761	3.9505E-81	0.4555078	0.022	0.106	6.0723E-77	T Cells
BCKDHB	4.413E-81	1.1211876	0.041	0.137	6.7831E-77	T Cells
TIMELESS	4.4867E-81	0.42318509	0.023	0.107	6.8964E-77	T Cells
GPX71	4.5656E-81	1.15455992	0.041	0.136	7.0178E-77	T Cells
FAM76B1	5.1901E-81	1.59772239	0.072	0.186	7.9776E-77	T Cells
ADAL	5.1938E-81	0.39069139	0.019	0.1	7.9834E-77	T Cells
WDYHV11	6.0794E-81	1.34187308	0.046	0.144	9.3447E-77	T Cells
TUBGCP4	6.2967E-81	1.29725523	0.043	0.139	9.6786E-77	T Cells
NAALADL11	6.8483E-81	0.79631907	0.031	0.12	1.0527E-76	T Cells
UBE2V21	7.2625E-81	0.5849587	0.02	0.101	1.1163E-76	T Cells
ZNF8051	1.0062E-80	1.53208011	0.065	0.175	1.5466E-76	T Cells
RTN4R	1.0336E-80	0.84043407	0.027	0.114	1.5887E-76	T Cells
ZNF3372	1.5838E-80	1.85612265	0.127	0.274	2.4345E-76	T Cells
TMEM2231	1.6713E-80	0.46557715	0.026	0.111	2.5689E-76	T Cells
DOLPP11	2.0366E-80	0.45961402	0.021	0.102	3.1305E-76	T Cells
TRIM231	2.0566E-80	1.67772841	0.06	0.167	3.1612E-76	T Cells
HIKESHI2	2.0614E-80	0.43363704	0.022	0.104	3.1686E-76	T Cells
CYFIP23	2.0667E-80	3.47737866	0.338	0.303	3.1767E-76	T Cells
ACBD41	2.2558E-80	0.59449575	0.024	0.107	3.4674E-76	T Cells
MRFAP12	2.3154E-80	0.79440172	0.026	0.111	3.559E-76	T Cells
NFXL11	2.395E-80	1.3775213	0.046	0.144	3.6814E-76	T Cells
SLC12A21	2.6818E-80	1.37620707	0.054	0.156	4.1222E-76	T Cells
BBC33	2.9201E-80	1.76707071	0.096	0.223	4.4884E-76	T Cells
FAM185A	3.2497E-80	0.19836041	0.015	0.093	4.9951E-76	T Cells
ZSCAN301	3.95E-80	0.46875641	0.029	0.115	6.0716E-76	T Cells
ZNF228	4.339E-80	1.82680471	0.103	0.236	6.6695E-76	T Cells
CENPU	4.8556E-80	0.1442267	0.02	0.101	7.4635E-76	T Cells
SLC36A12	5.2453E-80	1.08991479	0.033	0.123	8.0625E-76	T Cells
CYB561	5.3245E-80	1.24787542	0.044	0.14	8.1843E-76	T Cells
ZRANB22	5.3669E-80	1.9455112	0.231	0.456	8.2494E-76	T Cells
MICALL1	5.6983E-80	0.87931638	0.029	0.116	8.7588E-76	T Cells
SLC25A221	5.7165E-80	1.3155094	0.049	0.149	8.7868E-76	T Cells
TBC1D10C	6.1818E-80	1.2188648	0.385	0.779	9.5021E-76	T Cells
WDR832	6.6856E-80	0.73292395	0.027	0.112	1.0276E-75	T Cells
CDPF11	6.9384E-80	1.22391309	0.04	0.133	1.0665E-75	T Cells
ASPHD2	7.4157E-80	0.54477458	0.023	0.106	1.1399E-75	T Cells
ANP32E1	8.2083E-80	1.82013382	0.116	0.255	1.2617E-75	T Cells
ZSCAN24	8.5126E-80	1.34441486	0.044	0.14	1.3085E-75	T Cells
CD2443	1.0095E-79	5.12881485	0.035	0.009	1.5518E-75	T Cells
RNF2133	1.0502E-79	1.64349095	0.601	0.909	1.6143E-75	T Cells
ZBED52	1.0943E-79	1.72665804	0.086	0.207	1.6821E-75	T Cells
ACAD111	1.3752E-79	1.36042447	0.067	0.177	2.1138E-75	T Cells
ZNF7071	1.3819E-79	1.23307901	0.049	0.147	2.1241E-75	T Cells
ZNF443	1.4476E-79	1.55471224	0.066	0.176	2.2252E-75	T Cells

ZNF5141	1.4955E-79	1.46904403	0.055	0.159	2.2988E-75	T Cells
CYTH33	1.6464E-79	1.30699198	0.049	0.147	2.5308E-75	T Cells
TRMT112	2.087E-79	1.21166948	0.042	0.136	3.2079E-75	T Cells
MYLIP3	2.1802E-79	1.00220048	0.041	0.134	3.3512E-75	T Cells
B3GNTL11	2.6013E-79	0.79074632	0.031	0.118	3.9984E-75	T Cells
SPTBN12	2.7032E-79	4.20219058	0.059	0.021	4.155E-75	T Cells
THAP8	2.7418E-79	0.27238789	0.014	0.09	4.2144E-75	T Cells
GON4L2	3.065E-79	1.83107826	0.226	0.444	4.7112E-75	T Cells
GRK41	4.1631E-79	0.57683604	0.021	0.102	6.3991E-75	T Cells
PPP1R13L2	4.7595E-79	0.53683552	0.024	0.106	7.3158E-75	T Cells
LDB12	4.8892E-79	1.77459262	0.301	0.591	7.5153E-75	T Cells
GFOD22	4.924E-79	1.55833244	0.072	0.183	7.5687E-75	T Cells
POLI1	5.5294E-79	1.80349277	0.104	0.236	8.4993E-75	T Cells
BBS71	7.5847E-79	1.13941811	0.038	0.129	1.1658E-74	T Cells
EDRF11	8.4963E-79	1.707841	0.082	0.2	1.306E-74	T Cells
MXI11	9.3652E-79	1.69552802	0.082	0.2	1.4395E-74	T Cells
POC53	1.0953E-78	1.16563427	0.038	0.128	1.6836E-74	T Cells
CDYL22	1.2237E-78	1.70635185	0.081	0.198	1.881E-74	T Cells
ZNF2541	1.9355E-78	1.42080149	0.067	0.175	2.975E-74	T Cells
RIMS33	1.9716E-78	5.23031714	0.028	0.006	3.0306E-74	T Cells
HSPA14	2.3889E-78	1.07718399	0.036	0.126	3.672E-74	T Cells
CENPJ	2.7956E-78	1.19231243	0.041	0.134	4.2972E-74	T Cells
MTFP11	2.8116E-78	1.31258793	0.045	0.141	4.3218E-74	T Cells
KLF133	2.906E-78	1.87699858	0.248	0.485	4.4668E-74	T Cells
CAMK2G1	3.6843E-78	1.68100389	0.073	0.185	5.6631E-74	T Cells
ZNF4411	4.3155E-78	1.25498451	0.041	0.133	6.6334E-74	T Cells
NMT2	5.0236E-78	4.58527409	0.101	0.048	7.7218E-74	T Cells
PIAS3	5.6539E-78	1.42995379	0.06	0.165	8.6905E-74	T Cells
TSEN15	5.7071E-78	1.22342558	0.04	0.131	8.7723E-74	T Cells
PFKFB34	5.7323E-78	1.77906618	0.223	0.437	8.8111E-74	T Cells
MCM81	6.2821E-78	1.17392381	0.038	0.129	9.6562E-74	T Cells
ZNF5122	6.346E-78	1.85151762	0.121	0.261	9.7545E-74	T Cells
FARS21	6.6093E-78	0.98867228	0.039	0.13	1.0159E-73	T Cells
AIFM22	7.1263E-78	0.91556484	0.032	0.118	1.0954E-73	T Cells
ZFP281	7.414E-78	0.92784454	0.037	0.126	1.1396E-73	T Cells
TMEM67	8.6783E-78	0.30157041	0.018	0.095	1.3339E-73	T Cells
TEPSIN2	9.3128E-78	1.15892026	0.045	0.139	1.4315E-73	T Cells
SPICE11	9.7165E-78	1.34213572	0.046	0.142	1.4935E-73	T Cells
LUC7L6	1.1024E-77	1.83174782	0.204	0.402	1.6944E-73	T Cells
CBFA2T21	1.2591E-77	1.88657674	0.142	0.296	1.9353E-73	T Cells
C2CD31	1.2992E-77	1.5876956	0.064	0.17	1.997E-73	T Cells
ZNF230	1.5616E-77	0.22806471	0.019	0.096	2.4003E-73	T Cells
FUBP13	1.7927E-77	1.73773138	0.316	0.619	2.7555E-73	T Cells
SNTA1	2.724E-77	1.61573324	0.075	0.188	4.187E-73	T Cells
UTP151	2.8543E-77	0.95305809	0.03	0.114	4.3873E-73	T Cells
KNTC11	3.1796E-77	1.22549043	0.048	0.143	4.8874E-73	T Cells
COMTD12	3.2797E-77	1.71390422	0.071	0.181	5.0412E-73	T Cells
RAB40C1	3.7245E-77	0.77961072	0.026	0.108	5.725E-73	T Cells
C18orf251	4.303E-77	2.03492399	0.175	0.35	6.6141E-73	T Cells
HOOK11	4.426E-77	4.90931249	0.012	0.001	6.8032E-73	T Cells
SP140L2	5.3654E-77	1.8903143	0.125	0.267	8.2472E-73	T Cells
BPTF1	5.4616E-77	1.85582386	0.295	0.575	8.395E-73	T Cells

ZNF134	5.5015E-77	0.56935013	0.023	0.104	8.4564E-73	T Cells
PRKCI1	5.5107E-77	1.51503638	0.061	0.165	8.4704E-73	T Cells
ADA3	5.888E-77	4.39030908	0.094	0.043	9.0504E-73	T Cells
NPAT2	7.3879E-77	1.80576615	0.086	0.204	1.1356E-72	T Cells
SCAPER1	7.8038E-77	1.39072291	0.055	0.154	1.1995E-72	T Cells
WARS2	1.4446E-76	1.12411024	0.04	0.13	2.2205E-72	T Cells
GPATCH13	1.502E-76	0.9879397	0.032	0.117	2.3087E-72	T Cells
TTBK21	1.7416E-76	1.48011939	0.057	0.158	2.677E-72	T Cells
ARID3B	1.9194E-76	1.7866575	0.108	0.239	2.9503E-72	T Cells
AKAP132	2.0771E-76	0.92553033	0.442	0.908	3.1926E-72	T Cells
DENND4A2	2.4149E-76	1.77547945	0.315	0.619	3.7119E-72	T Cells
PIGBOS1	2.9059E-76	0.23199217	0.017	0.092	4.4667E-72	T Cells
CASP22	5.3147E-76	1.92314589	0.136	0.284	8.1692E-72	T Cells
FAM126B1	5.3985E-76	1.79596311	0.1	0.227	8.298E-72	T Cells
PPM1L2	6.3994E-76	0.42739691	0.02	0.097	9.8365E-72	T Cells
TTLL111	6.5821E-76	0.51328935	0.022	0.101	1.0117E-71	T Cells
WRN1	6.8782E-76	1.54058232	0.057	0.158	1.0572E-71	T Cells
ITGA3	7.1065E-76	0.98607929	0.04	0.13	1.0923E-71	T Cells
MOSMO1	7.5223E-76	1.4110309	0.05	0.147	1.1563E-71	T Cells
FANCG	7.8707E-76	0.79725643	0.027	0.109	1.2098E-71	T Cells
COG31	1.0437E-75	1.92914651	0.144	0.296	1.6043E-71	T Cells
PPP2R1A2	1.1271E-75	0.88039111	0.027	0.108	1.7325E-71	T Cells
NME71	1.2769E-75	1.41134645	0.053	0.151	1.9627E-71	T Cells
SSX2IP	1.5539E-75	0.4663465	0.021	0.099	2.3884E-71	T Cells
CBY1	1.5948E-75	0.72325366	0.024	0.103	2.4514E-71	T Cells
PIGL1	1.897E-75	1.34291302	0.077	0.188	2.9159E-71	T Cells
SENP11	2.085E-75	1.75509799	0.091	0.211	3.2049E-71	T Cells
JADE21	2.1559E-75	1.96374354	0.142	0.293	3.3139E-71	T Cells
YDJC	2.4246E-75	1.26875444	0.042	0.133	3.7268E-71	T Cells
GLCCI12	2.4282E-75	4.3322007	0.118	0.061	3.7324E-71	T Cells
EDA3	2.9737E-75	0.21223233	0.015	0.088	4.5709E-71	T Cells
SZT21	2.9865E-75	1.34719732	0.054	0.152	4.5905E-71	T Cells
FBF12	3.9196E-75	1.18308824	0.044	0.137	6.0248E-71	T Cells
SNRNP2004	3.9361E-75	1.47694736	0.385	0.772	6.0502E-71	T Cells
ZNF5681	4.8388E-75	1.81583399	0.082	0.197	7.4377E-71	T Cells
AVEN	5.5722E-75	1.36235709	0.037	0.125	8.565E-71	T Cells
TAF1A1	5.7099E-75	1.15115266	0.039	0.127	8.7766E-71	T Cells
CEP1352	5.9673E-75	1.67339492	0.077	0.189	9.1724E-71	T Cells
TBPL12	7.5334E-75	1.815843	0.089	0.207	1.158E-70	T Cells
CDKL11	7.6403E-75	0.40008176	0.023	0.101	1.1744E-70	T Cells
UBE2T	9.5201E-75	0.28563223	0.023	0.102	1.4633E-70	T Cells
PWWP2A	1.0403E-74	1.83248092	0.103	0.23	1.5991E-70	T Cells
ARMCX61	1.2537E-74	0.42169349	0.022	0.099	1.9271E-70	T Cells
VPS13B3	1.2691E-74	1.73161713	0.251	0.486	1.9508E-70	T Cells
SLC45A32	1.4228E-74	0.11488191	0.013	0.084	2.187E-70	T Cells
ZNF684	1.4651E-74	0.41500762	0.019	0.094	2.2521E-70	T Cells
PARP113	1.7846E-74	1.55609989	0.056	0.155	2.7431E-70	T Cells
ATAT11	1.8665E-74	0.72008549	0.026	0.107	2.8689E-70	T Cells
PIK3R11	2.2933E-74	1.88622013	0.305	0.596	3.5251E-70	T Cells
PAOX	2.4385E-74	1.30371961	0.049	0.143	3.7483E-70	T Cells
ZBTB23	2.5448E-74	1.97673537	0.121	0.259	3.9116E-70	T Cells
CHPF	2.6653E-74	1.00918957	0.042	0.131	4.0968E-70	T Cells

DGKZ2	3.5406E-74	1.90037756	0.129	0.272	5.4422E-70	T Cells
ATG4D1	5.8125E-74	1.57174046	0.064	0.167	8.9344E-70	T Cells
FXYD11	6.4481E-74	5.71547599	0.012	0.001	9.9113E-70	T Cells
TAF1C2	6.6536E-74	1.47587935	0.058	0.157	1.0227E-69	T Cells
ZNF1411	6.9226E-74	1.52459211	0.081	0.193	1.0641E-69	T Cells
ZNF354B	7.2296E-74	0.88225113	0.031	0.114	1.1113E-69	T Cells
GMEB2	7.5621E-74	1.63981527	0.06	0.16	1.1624E-69	T Cells
LCMT2	1.0785E-73	0.80828624	0.023	0.1	1.6578E-69	T Cells
FBXO411	1.3013E-73	1.05565398	0.037	0.123	2.0003E-69	T Cells
ZNF6752	1.506E-73	0.81435055	0.027	0.107	2.3149E-69	T Cells
COX191	1.5365E-73	1.63940608	0.065	0.168	2.3618E-69	T Cells
DNAJC181	2.0365E-73	0.58303887	0.023	0.1	3.1303E-69	T Cells
RNF1453	2.073E-73	2.03116176	0.16	0.322	3.1865E-69	T Cells
MDC1	2.2831E-73	1.83532643	0.077	0.187	3.5093E-69	T Cells
SYT112	2.3047E-73	1.90245101	0.128	0.268	3.5425E-69	T Cells
ZFP692	2.4758E-73	0.73998531	0.021	0.096	3.8055E-69	T Cells
DDX39B2	2.6922E-73	1.98820783	0.187	0.369	4.1383E-69	T Cells
BCR1	2.9574E-73	1.75491222	0.099	0.222	4.5458E-69	T Cells
CEACAM11	3.1338E-73	0.74696134	0.037	0.123	4.817E-69	T Cells
TRIM68	3.2528E-73	0.33132167	0.015	0.087	4.9999E-69	T Cells
USP13	3.7041E-73	0.58361458	0.021	0.098	5.6936E-69	T Cells
ARL161	3.773E-73	1.4490922	0.046	0.137	5.7995E-69	T Cells
GNPDA22	3.8885E-73	1.31295943	0.044	0.135	5.977E-69	T Cells
PIM23	3.9381E-73	3.80992459	0.308	0.267	6.0533E-69	T Cells
C17orf801	4.6221E-73	0.50445886	0.019	0.094	7.1047E-69	T Cells
FANCC	5.052E-73	0.8139035	0.025	0.104	7.7655E-69	T Cells
ZNF921	7.4847E-73	1.79565413	0.086	0.2	1.1505E-68	T Cells
BCS1L1	7.676E-73	0.84771645	0.029	0.109	1.1799E-68	T Cells
MEAK7	1.2337E-72	1.20588882	0.039	0.125	1.8964E-68	T Cells
SNAP47	1.6595E-72	2.17662386	0.17	0.338	2.5508E-68	T Cells
DENND34	1.8166E-72	1.70307725	0.122	0.258	2.7922E-68	T Cells
PPP1R351	1.8535E-72	1.92709763	0.164	0.326	2.8489E-68	T Cells
ZNF5712	2.7872E-72	0.43818475	0.021	0.095	4.2842E-68	T Cells
ACTR51	3.0684E-72	1.58214458	0.058	0.157	4.7165E-68	T Cells
DCAF4	3.259E-72	1.36336339	0.041	0.129	5.0095E-68	T Cells
ZNF613	3.5569E-72	0.51568919	0.021	0.096	5.4674E-68	T Cells
SNRPF1	3.6975E-72	0.54363647	0.021	0.097	5.6835E-68	T Cells
CEP781	4.6305E-72	1.68308994	0.067	0.17	7.1176E-68	T Cells
MTERF11	4.6385E-72	1.33221867	0.047	0.137	7.1298E-68	T Cells
MITD13	4.9333E-72	1.91990708	0.109	0.237	7.583E-68	T Cells
TSNARE11	5.4477E-72	1.05433649	0.029	0.109	8.3736E-68	T Cells
SUPT3H1	6.423E-72	1.73172232	0.074	0.18	9.8727E-68	T Cells
PROCA11	7.2956E-72	0.87690017	0.028	0.108	1.1214E-67	T Cells
SNRPA11	8.5958E-72	1.63687962	0.056	0.152	1.3213E-67	T Cells
TNRC6B1	1.0078E-71	1.96502638	0.254	0.489	1.549E-67	T Cells
DIDO11	1.0829E-71	2.00317456	0.225	0.433	1.6645E-67	T Cells
GIMAP1	1.8571E-71	1.99301297	0.155	0.311	2.8546E-67	T Cells
FRYL1	1.8892E-71	1.97242602	0.215	0.415	2.9038E-67	T Cells
TAF14	2.3462E-71	1.76659159	0.076	0.184	3.6063E-67	T Cells
TMEM120B1	2.6068E-71	0.71168398	0.026	0.103	4.007E-67	T Cells
MBOAT23	2.6878E-71	5.0022228	0.017	0.003	4.1314E-67	T Cells
ZSWIM31	3.0916E-71	0.62398645	0.024	0.1	4.7521E-67	T Cells

ZNF4461	3.1419E-71	1.18162812	0.031	0.112	4.8295E-67	T Cells
AHCTF11	4.7432E-71	1.43862341	0.043	0.131	7.2908E-67	T Cells
KIAA15861	4.8257E-71	1.6200136	0.05	0.143	7.4176E-67	T Cells
EXT12	5.3197E-71	1.27732917	0.046	0.135	8.1769E-67	T Cells
QSOX22	5.3268E-71	1.28496926	0.04	0.126	8.1878E-67	T Cells
ZNF354C	8.7844E-71	1.15363897	0.034	0.116	1.3502E-66	T Cells
USP371	9.7409E-71	1.6838669	0.072	0.176	1.4973E-66	T Cells
MYL53	1.0922E-70	0.12834482	0.016	0.087	1.6789E-66	T Cells
AK11	1.1009E-70	1.56112935	0.111	0.237	1.6923E-66	T Cells
USP46	1.1846E-70	0.71732822	0.021	0.095	1.8208E-66	T Cells
ENGASE2	1.2843E-70	1.22323589	0.058	0.154	1.9742E-66	T Cells
IER24	2.1712E-70	1.47726214	0.354	0.692	3.3373E-66	T Cells
TRMT122	2.4272E-70	0.50182176	0.019	0.091	3.7309E-66	T Cells
CHKA2	2.4947E-70	0.39158647	0.019	0.091	3.8347E-66	T Cells
LIN52	2.4997E-70	1.43319201	0.039	0.125	3.8422E-66	T Cells
TAMM411	3.268E-70	1.49868279	0.065	0.164	5.0232E-66	T Cells
NEK1	5.2948E-70	1.23708657	0.035	0.117	8.1386E-66	T Cells
GADD45G3	5.4207E-70	0.59150138	0.023	0.098	8.3321E-66	T Cells
AK91	5.9454E-70	1.21426989	0.039	0.123	9.1386E-66	T Cells
MFSD4B3	6.2186E-70	1.39497786	0.057	0.153	9.5587E-66	T Cells
FANCE	6.7844E-70	0.91846105	0.027	0.104	1.0428E-65	T Cells
PHC3	7.8434E-70	1.95058469	0.257	0.493	1.2056E-65	T Cells
ZNF6521	7.9315E-70	2.08150492	0.146	0.295	1.2191E-65	T Cells
MYSM12	8.8665E-70	1.93138728	0.168	0.331	1.3629E-65	T Cells
ARL5B2	9.1062E-70	1.32412651	0.039	0.124	1.3997E-65	T Cells
NAA161	1.018E-69	1.80049068	0.082	0.192	1.5648E-65	T Cells
BTBD92	1.0793E-69	1.9838347	0.152	0.305	1.659E-65	T Cells
XRCC3	1.0886E-69	0.13454814	0.014	0.081	1.6732E-65	T Cells
PRDM101	1.1064E-69	1.05227033	0.032	0.113	1.7007E-65	T Cells
BCOR1	1.1803E-69	1.96039674	0.214	0.412	1.8142E-65	T Cells
NRDE21	1.335E-69	1.06098183	0.025	0.101	2.052E-65	T Cells
BBIP12	1.381E-69	1.43888875	0.045	0.132	2.1227E-65	T Cells
ZNF1821	1.3851E-69	1.52544617	0.046	0.135	2.1291E-65	T Cells
ZNF6261	1.4453E-69	1.61058801	0.059	0.154	2.2216E-65	T Cells
CYB5RL1	1.5622E-69	0.45284616	0.018	0.088	2.4012E-65	T Cells
SERGEF2	1.7572E-69	2.01913827	0.12	0.25	2.701E-65	T Cells
FHIT1	1.8969E-69	0.17341008	0.016	0.084	2.9158E-65	T Cells
FRS31	2.1108E-69	0.55334581	0.022	0.096	3.2445E-65	T Cells
CEP1283	2.2818E-69	1.71746466	0.082	0.191	3.5074E-65	T Cells
ASXL1	2.4732E-69	1.85696704	0.294	0.561	3.8016E-65	T Cells
FIZ1	2.5178E-69	0.22265958	0.016	0.085	3.8701E-65	T Cells
MTX32	2.6087E-69	1.96531697	0.079	0.188	4.0098E-65	T Cells
POLE1	2.8745E-69	1.78175359	0.08	0.188	4.4183E-65	T Cells
FGFR1OP1	2.9927E-69	1.8274005	0.081	0.19	4.6E-65	T Cells
SEPTIN62	3.135E-69	1.92944516	0.26	0.493	4.8188E-65	T Cells
UTP14A1	3.83E-69	0.43700301	0.016	0.084	5.8871E-65	T Cells
ZNF5991	3.9027E-69	0.65124276	0.022	0.096	5.9989E-65	T Cells
ARSG1	9.5553E-69	1.70394174	0.058	0.153	1.4687E-64	T Cells
TRMT10B	9.583E-69	1.18855384	0.037	0.12	1.473E-64	T Cells
PDCD2L	1.7238E-68	0.87981641	0.026	0.101	2.6496E-64	T Cells
ZNF7991	1.7372E-68	0.34184859	0.016	0.085	2.6703E-64	T Cells
BBS10	1.7466E-68	0.97319631	0.028	0.105	2.6846E-64	T Cells

SIK34	1.8575E-68	2.03327338	0.156	0.31	2.8551E-64	T Cells
KLHL55	2.0025E-68	1.80243973	0.104	0.226	3.0781E-64	T Cells
ALKBH2	2.0758E-68	0.32458183	0.015	0.082	3.1908E-64	T Cells
SCYL31	2.5029E-68	1.78233608	0.071	0.174	3.8471E-64	T Cells
XPOT2	2.6834E-68	2.19891209	0.13	0.268	4.1246E-64	T Cells
ZNF7651	2.9076E-68	0.50895716	0.02	0.091	4.4692E-64	T Cells
DDHD22	3.0212E-68	2.05185298	0.124	0.257	4.6438E-64	T Cells
WDR532	3.3112E-68	1.28882362	0.038	0.121	5.0896E-64	T Cells
RNF1221	3.5528E-68	0.6330018	0.023	0.096	5.461E-64	T Cells
TULP31	3.8316E-68	0.54501263	0.017	0.087	5.8895E-64	T Cells
ZNF5831	4.0597E-68	0.38476559	0.019	0.089	6.2401E-64	T Cells
ATP1A31	4.1226E-68	6.78853439	0.01	0.001	6.3369E-64	T Cells
CRTC11	4.6778E-68	0.71637032	0.018	0.088	7.1903E-64	T Cells
ATAD5	5.6391E-68	0.73473021	0.023	0.096	8.6678E-64	T Cells
ZNF155	6.1793E-68	0.6098646	0.018	0.088	9.4983E-64	T Cells
HCLS13	6.6208E-68	0.20181996	0.498	0.971	1.0177E-63	T Cells
MYO18A4	7.7248E-68	1.94626751	0.142	0.285	1.1874E-63	T Cells
ZNF5292	8.2632E-68	1.34603709	0.045	0.131	1.2701E-63	T Cells
GCLM1	9.5592E-68	1.89995666	0.082	0.189	1.4694E-63	T Cells
THADA2	1.1549E-67	2.00155775	0.157	0.31	1.7751E-63	T Cells
ZNF131	1.1635E-67	1.99527949	0.106	0.227	1.7884E-63	T Cells
CSPP1	1.5264E-67	1.45167824	0.051	0.14	2.3463E-63	T Cells
KBTBD81	1.539E-67	1.24251059	0.038	0.119	2.3656E-63	T Cells
KLHL261	1.5391E-67	0.72054556	0.021	0.093	2.3658E-63	T Cells
ZNF2272	1.8254E-67	1.42279802	0.041	0.125	2.8058E-63	T Cells
MCC2	2.1284E-67	6.12441735	0.013	0.002	3.2715E-63	T Cells
PHLDB2	2.2087E-67	4.835498	0.021	0.004	3.395E-63	T Cells
ANKRD261	2.2634E-67	0.46669706	0.022	0.094	3.4791E-63	T Cells
H2AFY21	2.561E-67	0.81809586	0.025	0.098	3.9365E-63	T Cells
CABLES22	3.2295E-67	0.72478195	0.022	0.093	4.964E-63	T Cells
TTC121	3.309E-67	1.17503294	0.034	0.112	5.0863E-63	T Cells
TP53INP12	3.8019E-67	1.77363233	0.078	0.183	5.8439E-63	T Cells
CEP1521	3.9137E-67	0.57988214	0.025	0.097	6.0158E-63	T Cells
ATP6V0A24	4.5352E-67	2.05909854	0.149	0.296	6.9711E-63	T Cells
ARL61	6.4215E-67	0.22217291	0.015	0.082	9.8705E-63	T Cells
SMIM81	6.5803E-67	1.13971963	0.038	0.118	1.0115E-62	T Cells
MCM7	6.7476E-67	1.42643623	0.187	0.354	1.0372E-62	T Cells
ICOS	7.3289E-67	1.73117614	0.224	0.422	1.1265E-62	T Cells
ACACA1	9.3795E-67	1.53475815	0.047	0.133	1.4417E-62	T Cells
NAF1	1.4046E-66	1.34062716	0.035	0.115	2.159E-62	T Cells
FYB13	1.4128E-66	2.9133129	0.452	0.528	2.1716E-62	T Cells
MTFMT1	2.358E-66	0.24071898	0.015	0.081	3.6245E-62	T Cells
MINDY12	2.4806E-66	0.50794277	0.022	0.093	3.813E-62	T Cells
ZNF2362	2.6184E-66	1.88114356	0.099	0.216	4.0247E-62	T Cells
PTCD21	3.0832E-66	0.86181222	0.023	0.095	4.7392E-62	T Cells
CD692	3.3713E-66	2.57799622	0.406	0.423	5.182E-62	T Cells
GGT71	3.5741E-66	0.43391006	0.022	0.092	5.4937E-62	T Cells
SELL4	3.6892E-66	4.92170199	0.102	0.052	5.6706E-62	T Cells
SIN3A1	4.0942E-66	1.92784791	0.272	0.516	6.2932E-62	T Cells
ZNF33A4	5.1858E-66	1.94497744	0.141	0.28	7.9711E-62	T Cells
NEK112	7.3803E-66	0.4140798	0.023	0.093	1.1344E-61	T Cells
TSPOAP13	9.8936E-66	4.00472684	0.067	0.028	1.5207E-61	T Cells

SARDH	1.0176E-65	5.17427044	0.033	0.009	1.5641E-61	T Cells
ULK41	1.197E-65	1.78956513	0.099	0.215	1.8399E-61	T Cells
TCEANC2	1.3843E-65	0.75242444	0.025	0.098	2.1278E-61	T Cells
AEBP22	1.6149E-65	1.99951979	0.25	0.473	2.4822E-61	T Cells
NIPA1	1.7212E-65	1.96667135	0.089	0.199	2.6456E-61	T Cells
DMTF13	2.1973E-65	1.87313054	0.185	0.356	3.3774E-61	T Cells
C2orf422	2.3011E-65	1.32925449	0.041	0.122	3.5371E-61	T Cells
FRAT12	2.3178E-65	0.19615992	0.018	0.084	3.5627E-61	T Cells
CBX5	2.352E-65	1.89546378	0.082	0.188	3.6153E-61	T Cells
ZFAT3	3.1213E-65	1.0166971	0.024	0.096	4.7978E-61	T Cells
RFX71	3.306E-65	1.91178914	0.09	0.2	5.0816E-61	T Cells
EFNA42	3.3594E-65	0.22418137	0.014	0.079	5.1638E-61	T Cells
PROSER31	3.408E-65	1.70894234	0.064	0.16	5.2384E-61	T Cells
KIAA07531	4.0099E-65	1.341427	0.038	0.118	6.1636E-61	T Cells
MIGA21	4.3777E-65	1.57666755	0.058	0.149	6.7289E-61	T Cells
PLEKHA53	5.9116E-65	4.44886923	0.069	0.029	9.0867E-61	T Cells
STK36	6.0874E-65	0.59220243	0.02	0.088	9.357E-61	T Cells
WDR63	6.2671E-65	2.00327391	0.174	0.335	9.6331E-61	T Cells
PUM22	6.7092E-65	1.6593761	0.362	0.701	1.0313E-60	T Cells
CACNA2D42	7.4911E-65	0.48398529	0.019	0.086	1.1514E-60	T Cells
ZNF649	9.0171E-65	1.02400094	0.029	0.103	1.386E-60	T Cells
ZNF6141	9.3591E-65	0.87136009	0.026	0.099	1.4386E-60	T Cells
RAD181	9.6785E-65	1.28946425	0.032	0.109	1.4877E-60	T Cells
ALG62	9.8819E-65	1.72855593	0.062	0.155	1.5189E-60	T Cells
ZNF5481	1.0847E-64	1.99945955	0.095	0.207	1.6673E-60	T Cells
ZNF8	1.2451E-64	0.98969956	0.028	0.101	1.9139E-60	T Cells
DTD21	2.282E-64	1.2467022	0.036	0.114	3.5076E-60	T Cells
LRFN3	2.5168E-64	5.07084328	0.038	0.012	3.8686E-60	T Cells
ZNF452	2.7255E-64	0.74870261	0.02	0.088	4.1894E-60	T Cells
BIVM1	3.7055E-64	1.45979818	0.043	0.124	5.6958E-60	T Cells
FAM184A	3.8605E-64	5.35615698	0.019	0.004	5.934E-60	T Cells
DXO1	4.7958E-64	0.73114577	0.024	0.094	7.3716E-60	T Cells
USP491	5.5632E-64	0.37389335	0.018	0.084	8.5513E-60	T Cells
PLP23	5.8795E-64	2.60952916	0.472	0.574	9.0373E-60	T Cells
ZNF5972	5.8835E-64	0.24241264	0.013	0.076	9.0435E-60	T Cells
C9orf40	6.1034E-64	0.90461149	0.027	0.099	9.3815E-60	T Cells
ZNF2481	6.8307E-64	0.74780463	0.024	0.095	1.0499E-59	T Cells
C10orf1431	7.4518E-64	0.66211068	0.023	0.093	1.1454E-59	T Cells
IGFBP41	1.1849E-63	4.85611257	0.016	0.003	1.8213E-59	T Cells
BBS92	1.3945E-63	1.69969818	0.067	0.163	2.1436E-59	T Cells
PAXX2	1.5785E-63	2.06829113	0.174	0.334	2.4263E-59	T Cells
BCDIN3D2	1.6057E-63	1.14193099	0.028	0.1	2.4682E-59	T Cells
LYRM91	1.725E-63	0.68668515	0.021	0.088	2.6515E-59	T Cells
CAPRIN21	2.2043E-63	2.05588094	0.111	0.231	3.3883E-59	T Cells
LRIG11	2.2315E-63	4.26939818	0.083	0.039	3.4301E-59	T Cells
ZNF2531	2.4542E-63	1.17451106	0.033	0.109	3.7723E-59	T Cells
AP4B12	2.4802E-63	1.97513455	0.105	0.222	3.8123E-59	T Cells
ZBTB241	2.7758E-63	1.72671436	0.061	0.152	4.2667E-59	T Cells
POP11	2.841E-63	0.68854108	0.021	0.088	4.367E-59	T Cells
ORC21	2.9584E-63	1.8754788	0.079	0.18	4.5473E-59	T Cells
THAP21	3.1523E-63	0.8887939	0.028	0.101	4.8454E-59	T Cells
LEMD31	3.4694E-63	1.98959289	0.106	0.222	5.3329E-59	T Cells

SCMH11	4.1403E-63	1.96527439	0.08	0.183	6.364E-59	T Cells
ATRIP	4.1968E-63	1.23988829	0.038	0.117	6.451E-59	T Cells
FASTKD3	5.1133E-63	1.09201301	0.028	0.101	7.8597E-59	T Cells
SGPP21	5.4548E-63	5.69164544	0.011	0.001	8.3846E-59	T Cells
ZNF2251	7.9456E-63	1.09034269	0.03	0.103	1.2213E-58	T Cells
COQ10A	8.1978E-63	0.84818994	0.023	0.092	1.2601E-58	T Cells
ZNF5671	8.7159E-63	1.61631206	0.05	0.135	1.3397E-58	T Cells
ZKSCAN7	1.0951E-62	0.28185932	0.016	0.079	1.6833E-58	T Cells
ACVR2B1	1.1837E-62	1.32663588	0.041	0.119	1.8194E-58	T Cells
POMT21	1.5011E-62	0.42372447	0.016	0.08	2.3073E-58	T Cells
NLE1	1.5362E-62	0.94554115	0.024	0.092	2.3613E-58	T Cells
KATNBL12	1.7271E-62	1.31404948	0.038	0.115	2.6547E-58	T Cells
ZNF6061	1.8191E-62	1.30230467	0.043	0.123	2.7961E-58	T Cells
ARHGEF122	1.9951E-62	1.73431352	0.071	0.167	3.0666E-58	T Cells
PTP4A32	2.4859E-62	5.21403849	0.026	0.006	3.8211E-58	T Cells
TMEM177	3.4395E-62	0.91100862	0.021	0.087	5.2868E-58	T Cells
ZNF587B1	3.724E-62	1.66046581	0.064	0.157	5.7242E-58	T Cells
KYAT12	4.0018E-62	0.42550492	0.014	0.077	6.1511E-58	T Cells
ABT1	5.1136E-62	1.27189705	0.035	0.11	7.8601E-58	T Cells
POLH1	5.1862E-62	1.70213481	0.075	0.173	7.9717E-58	T Cells
ZC3H12D3	5.6827E-62	4.46744968	0.091	0.045	8.7349E-58	T Cells
ZNF1802	6.4491E-62	1.05770697	0.029	0.1	9.9129E-58	T Cells
ATAD2B4	8.8718E-62	1.94465871	0.129	0.258	1.3637E-57	T Cells
PCOLCE1	1.1356E-61	0.34191097	0.015	0.077	1.7455E-57	T Cells
ZNF880	1.1617E-61	0.82852944	0.021	0.088	1.7857E-57	T Cells
SNTB21	1.2564E-61	2.12989597	0.154	0.298	1.9312E-57	T Cells
ZFPL1	1.2649E-61	0.78407479	0.025	0.093	1.9442E-57	T Cells
CYTH13	1.3947E-61	1.22797512	0.424	0.847	2.1437E-57	T Cells
DBF4B	1.5886E-61	0.22392088	0.014	0.075	2.4418E-57	T Cells
ASB1	2.0193E-61	1.65252809	0.061	0.151	3.1038E-57	T Cells
PCGF62	2.6005E-61	0.87810962	0.022	0.089	3.9972E-57	T Cells
TWNK1	3.1931E-61	1.15163531	0.027	0.097	4.9081E-57	T Cells
HPF12	3.3516E-61	0.71107705	0.02	0.086	5.1517E-57	T Cells
SBK1	3.3563E-61	5.44426968	0.03	0.008	5.159E-57	T Cells
GMCL12	3.4523E-61	1.60408855	0.047	0.129	5.3066E-57	T Cells
IGF1R3	3.5482E-61	0.7400882	0.022	0.088	5.4539E-57	T Cells
BRWD33	4.6111E-61	2.05105832	0.122	0.246	7.0878E-57	T Cells
CTSW	4.8078E-61	2.33076274	0.236	0.442	7.3901E-57	T Cells
TAF51	4.8491E-61	1.38725306	0.035	0.109	7.4536E-57	T Cells
WDR822	4.9795E-61	1.80428365	0.349	0.67	7.654E-57	T Cells
GKAP1	5.1453E-61	1.16774908	0.027	0.096	7.9089E-57	T Cells
ZBTB491	6.1171E-61	1.23378729	0.031	0.103	9.4026E-57	T Cells
VANGL1	8.1116E-61	1.23142506	0.031	0.104	1.2468E-56	T Cells
MAP3K33	8.6612E-61	1.98354137	0.15	0.29	1.3313E-56	T Cells
CCDC77	8.911E-61	0.25835691	0.012	0.072	1.3697E-56	T Cells
CDAN12	9.2149E-61	1.12075837	0.024	0.092	1.4164E-56	T Cells
SPATA331	1.0398E-60	1.2881842	0.044	0.122	1.5983E-56	T Cells
PRPF392	1.1342E-60	1.92742995	0.129	0.255	1.7434E-56	T Cells
BRD23	1.4105E-60	1.24183232	0.426	0.847	2.1682E-56	T Cells
FKRP1	1.4799E-60	1.27473482	0.033	0.106	2.2748E-56	T Cells
SUCLA22	1.5054E-60	0.57222556	0.022	0.087	2.314E-56	T Cells
ZNF512B	1.5199E-60	1.64037359	0.061	0.151	2.3363E-56	T Cells

TNFAIP83	1.5478E-60	2.05157258	0.253	0.473	2.3791E-56	T Cells
ZNF879	1.8552E-60	0.73415203	0.017	0.08	2.8516E-56	T Cells
CHRNB11	1.9391E-60	1.23014122	0.033	0.106	2.9806E-56	T Cells
ZNF702	1.986E-60	0.83744137	0.021	0.086	3.0527E-56	T Cells
PRELID22	2.037E-60	0.63563135	0.018	0.082	3.1311E-56	T Cells
TSTD1	2.0928E-60	1.91876483	0.095	0.203	3.2168E-56	T Cells
MRM11	2.1968E-60	0.95523321	0.022	0.088	3.3767E-56	T Cells
TARS1	2.7333E-60	1.11401273	0.024	0.091	4.2013E-56	T Cells
TNFRSF12A1	2.7499E-60	0.75062488	0.021	0.086	4.2269E-56	T Cells
RCSD12	3.2012E-60	3.15212027	0.306	0.273	4.9205E-56	T Cells
ZNF4861	3.3734E-60	0.79444042	0.018	0.081	5.1853E-56	T Cells
DONSON	4.0085E-60	1.22865724	0.037	0.112	6.1615E-56	T Cells
ZNF3431	4.2199E-60	0.70398467	0.023	0.089	6.4864E-56	T Cells
CAPN12	6.0197E-60	6.30088079	0.011	0.001	9.2529E-56	T Cells
ARHGDIB1	6.7448E-60	0.42330435	0.758	0.981	1.0367E-55	T Cells
COG61	6.9663E-60	0.22586582	0.013	0.073	1.0708E-55	T Cells
EIF14	7.1957E-60	1.03140575	0.627	0.951	1.106E-55	T Cells
PEX12	9.8915E-60	0.50384189	0.014	0.074	1.5204E-55	T Cells
ZNF527	1.1117E-59	1.07582463	0.023	0.088	1.7089E-55	T Cells
KCTD61	1.3799E-59	1.14958238	0.03	0.1	2.121E-55	T Cells
KCTD11	1.4003E-59	0.74232909	0.022	0.087	2.1525E-55	T Cells
BCL9L2	1.5951E-59	3.66921931	0.251	0.202	2.4518E-55	T Cells
THBS32	1.7772E-59	1.23361466	0.045	0.124	2.7317E-55	T Cells
SLC27A5	2.053E-59	1.12614356	0.026	0.094	3.1556E-55	T Cells
IL4I13	2.2589E-59	0.758695	0.028	0.097	3.4722E-55	T Cells
XRRA12	2.2769E-59	1.55188561	0.054	0.137	3.4998E-55	T Cells
MALT14	2.8661E-59	2.0737544	0.159	0.303	4.4055E-55	T Cells
PRIMPOL	2.9033E-59	0.94827411	0.024	0.091	4.4627E-55	T Cells
TCFL51	3.0116E-59	1.23153351	0.024	0.091	4.6291E-55	T Cells
EED1	3.0723E-59	2.01048007	0.098	0.207	4.7224E-55	T Cells
C15orf611	3.2059E-59	1.88720116	0.059	0.146	4.9278E-55	T Cells
LIPT1	3.734E-59	0.88255422	0.021	0.085	5.7396E-55	T Cells
ZNF5581	3.7392E-59	1.56315072	0.048	0.127	5.7475E-55	T Cells
AURKA	3.8064E-59	0.1898868	0.014	0.073	5.8509E-55	T Cells
ZNF5861	3.8907E-59	1.32619036	0.034	0.106	5.9804E-55	T Cells
TYW12	4.1049E-59	0.67034252	0.02	0.083	6.3097E-55	T Cells
NFE2L33	4.1731E-59	1.32289152	0.043	0.12	6.4145E-55	T Cells
CIDEB1	4.5265E-59	1.8245943	0.083	0.182	6.9577E-55	T Cells
ZNF585B1	4.8541E-59	1.03048402	0.025	0.092	7.4613E-55	T Cells
C12orf29	5.4075E-59	1.77892963	0.056	0.14	8.3119E-55	T Cells
BRMS1L1	5.5448E-59	0.69729438	0.018	0.081	8.523E-55	T Cells
ARNTL1	6.2734E-59	1.76995873	0.075	0.17	9.6428E-55	T Cells
MPP61	6.6838E-59	5.77549678	0.022	0.005	1.0274E-54	T Cells
VAMP2	6.9072E-59	1.91124766	0.301	0.564	1.0617E-54	T Cells
LRMP4	7.1764E-59	1.71388031	0.05	0.131	1.1031E-54	T Cells
RBM4B1	8.3121E-59	1.21555144	0.024	0.091	1.2777E-54	T Cells
SLC2A112	9.591E-59	0.49000337	0.017	0.079	1.4742E-54	T Cells
KCTD91	1.002E-58	2.09129065	0.149	0.286	1.5401E-54	T Cells
MBD22	1.1077E-58	0.30690312	0.498	0.97	1.7027E-54	T Cells
CARS1	1.1118E-58	1.54720838	0.044	0.121	1.7089E-54	T Cells
WDR59	1.1291E-58	1.73467511	0.072	0.164	1.7355E-54	T Cells
PIK3C2B	1.1547E-58	1.74428045	0.066	0.156	1.7749E-54	T Cells

PLXNC15	1.2E-58	3.8044639	0.076	0.036	1.8445E-54	T Cells
MDN11	1.2152E-58	1.93943926	0.084	0.184	1.8679E-54	T Cells
ZNF41	3.2062E-58	1.16033282	0.028	0.096	4.9283E-54	T Cells
MT-ND33	3.24E-58	2.32277532	0.412	0.441	4.9802E-54	T Cells
SMG7	3.3772E-58	0.33168006	0.014	0.073	5.1911E-54	T Cells
GIMAP51	3.3882E-58	4.070418	0.176	0.12	5.2081E-54	T Cells
USP421	4.4853E-58	1.61874498	0.044	0.121	6.8944E-54	T Cells
WDR19	4.935E-58	1.04587336	0.022	0.086	7.5856E-54	T Cells
FIGNL12	5.0947E-58	1.01953685	0.025	0.091	7.8311E-54	T Cells
RGS17	7.8647E-58	1.42417509	0.275	0.496	1.2089E-53	T Cells
FBXL181	8.7425E-58	1.43462274	0.037	0.111	1.3438E-53	T Cells
RFC51	9.6959E-58	0.66805314	0.016	0.076	1.4904E-53	T Cells
ENDOD1	1.5323E-57	2.02323394	0.085	0.184	2.3553E-53	T Cells
ZNF2111	1.7328E-57	1.38644855	0.038	0.111	2.6634E-53	T Cells
CENPT2	2.1837E-57	1.92495302	0.121	0.241	3.3565E-53	T Cells
TRGC21	2.5404E-57	2.23702588	0.103	0.212	3.9048E-53	T Cells
ELK41	4.1908E-57	1.92607642	0.314	0.587	6.4418E-53	T Cells
TTC39C	6.5024E-57	3.24449234	0.391	0.418	9.9948E-53	T Cells
TRIM521	6.8462E-57	2.09008134	0.112	0.227	1.0523E-52	T Cells
TNFRSF13C1	8.4862E-57	6.1501564	0.01	0.001	1.3044E-52	T Cells
CUBN1	9.3107E-57	4.99844576	0.026	0.007	1.4311E-52	T Cells
RAB37	9.7503E-57	4.82234746	0.044	0.016	1.4987E-52	T Cells
NR2C21	1.0107E-56	2.09279451	0.192	0.356	1.5535E-52	T Cells
CRY11	1.0292E-56	2.07259655	0.093	0.196	1.582E-52	T Cells
BOLA1	1.2178E-56	0.46673822	0.018	0.078	1.8718E-52	T Cells
EIF1AX1	1.635E-56	0.90327658	0.026	0.09	2.5132E-52	T Cells
KAT2A1	1.8517E-56	0.75467072	0.023	0.086	2.8462E-52	T Cells
ATP8B31	1.9579E-56	0.60779779	0.018	0.077	3.0096E-52	T Cells
ZNF5541	2.3578E-56	0.75443479	0.023	0.086	3.6241E-52	T Cells
MSL21	2.4545E-56	1.88034645	0.346	0.656	3.7729E-52	T Cells
TASP11	2.7166E-56	1.13375981	0.03	0.097	4.1757E-52	T Cells
ZNF3191	3.47E-56	1.36127158	0.04	0.112	5.3337E-52	T Cells
CRAMP11	3.4922E-56	1.94613852	0.077	0.17	5.3678E-52	T Cells
XPNPEP31	3.9831E-56	1.40148266	0.031	0.098	6.1224E-52	T Cells
SIPA1L13	4.1033E-56	1.74193726	0.06	0.145	6.3073E-52	T Cells
MARK41	4.1389E-56	1.21000247	0.027	0.093	6.3619E-52	T Cells
GPR354	4.4618E-56	4.32992384	0.051	0.02	6.8582E-52	T Cells
PARPBP1	4.4968E-56	0.19551604	0.012	0.068	6.9121E-52	T Cells
ZNF5691	5.0731E-56	1.28021166	0.027	0.093	7.7979E-52	T Cells
CCDC151	5.2886E-56	0.3460229	0.013	0.07	8.1291E-52	T Cells
METTL4	6.2648E-56	1.89217813	0.076	0.169	9.6297E-52	T Cells
FBXO441	7.1609E-56	1.79823017	0.058	0.141	1.1007E-51	T Cells
ZNF851	7.5427E-56	0.66458619	0.016	0.074	1.1594E-51	T Cells
PPT21	8.6228E-56	0.30417588	0.015	0.072	1.3254E-51	T Cells
ZNF5461	9.0514E-56	0.92981535	0.022	0.084	1.3913E-51	T Cells
ZNF888	9.2001E-56	0.76073599	0.015	0.073	1.4141E-51	T Cells
ALKBH82	9.2204E-56	1.15275974	0.03	0.096	1.4173E-51	T Cells
B3GNT101	1.1767E-55	0.90281752	0.017	0.076	1.8087E-51	T Cells
ZNF342	1.1886E-55	1.11425559	0.027	0.092	1.827E-51	T Cells
SPTAN1	1.2752E-55	1.16455139	0.42	0.82	1.9601E-51	T Cells
SWT11	1.3919E-55	1.5824369	0.035	0.106	2.1395E-51	T Cells
CHST72	1.6488E-55	1.34186974	0.035	0.105	2.5343E-51	T Cells

WDR89	1.6894E-55	1.59312721	0.038	0.109	2.5968E-51	T Cells
KIAA13281	1.8048E-55	1.81677625	0.071	0.16	2.7742E-51	T Cells
TMCO6	2.285E-55	0.75718579	0.021	0.082	3.5123E-51	T Cells
KIAA0895L2	2.4899E-55	0.73128075	0.021	0.082	3.8272E-51	T Cells
IKZF4	2.6152E-55	1.61550091	0.046	0.122	4.0198E-51	T Cells
ZNF565	2.6244E-55	1.19885309	0.025	0.088	4.034E-51	T Cells
C10orf881	2.6849E-55	1.27610374	0.033	0.102	4.127E-51	T Cells
FANCI	3.3292E-55	1.04330596	0.039	0.11	5.1173E-51	T Cells
ENDOV2	3.5214E-55	1.50742681	0.041	0.113	5.4128E-51	T Cells
SFMBT1	3.6127E-55	1.55352939	0.049	0.126	5.5531E-51	T Cells
C3orf331	3.8931E-55	0.6596229	0.012	0.067	5.9841E-51	T Cells
UST	4.0277E-55	1.00728696	0.022	0.083	6.191E-51	T Cells
PREX15	5.2412E-55	1.97897665	0.292	0.54	8.0563E-51	T Cells
CD442	5.4279E-55	0.37377424	0.826	0.993	8.3432E-51	T Cells
NSUN62	5.4704E-55	1.86117155	0.065	0.151	8.4085E-51	T Cells
DDX174	5.8273E-55	1.7828654	0.315	0.588	8.9572E-51	T Cells
PCNX42	7.4373E-55	1.72795025	0.064	0.148	1.1432E-50	T Cells
DNAJC92	1.0816E-54	1.64584358	0.034	0.103	1.6626E-50	T Cells
ZNF75D1	1.1442E-54	1.82141769	0.069	0.156	1.7587E-50	T Cells
EMSY1	1.2386E-54	1.9602979	0.08	0.173	1.9039E-50	T Cells
RANBP6	1.2559E-54	1.85957294	0.061	0.144	1.9305E-50	T Cells
MAGEF13	1.3774E-54	0.92041727	0.023	0.084	2.1172E-50	T Cells
ZNF5751	1.4315E-54	0.69430993	0.022	0.083	2.2003E-50	T Cells
TPD52	1.6674E-54	2.05173614	0.076	0.167	2.5629E-50	T Cells
ZNF570	1.7477E-54	1.49302035	0.038	0.108	2.6863E-50	T Cells
NCR3LG12	1.922E-54	0.72471197	0.017	0.074	2.9543E-50	T Cells
HINFP1	2.6744E-54	1.55852289	0.036	0.106	4.1109E-50	T Cells
LPAR21	3.2993E-54	1.74952279	0.121	0.235	5.0713E-50	T Cells
N6AMT12	3.4826E-54	1.36908555	0.027	0.091	5.3531E-50	T Cells
BDP12	3.8822E-54	2.08966366	0.222	0.405	5.9673E-50	T Cells
ZGRF11	4.3657E-54	0.96401248	0.025	0.088	6.7105E-50	T Cells
RPAP2	5.6521E-54	2.00846763	0.08	0.173	8.6878E-50	T Cells
HEATR5A1	6.7452E-54	0.75187476	0.024	0.086	1.0368E-49	T Cells
SPATA71	7.8356E-54	0.28120959	0.013	0.068	1.2044E-49	T Cells
CEP290	8.6038E-54	1.57145489	0.044	0.118	1.3225E-49	T Cells
DHRS111	1.3093E-53	1.04322157	0.022	0.083	2.0125E-49	T Cells
TXNDC161	1.489E-53	1.42554556	0.03	0.095	2.2887E-49	T Cells
UPF3A2	1.9264E-53	1.97711143	0.098	0.2	2.9611E-49	T Cells
KIAA1324L1	2.0735E-53	5.33521209	0.021	0.005	3.1872E-49	T Cells
TUBD12	3.4331E-53	1.90241525	0.067	0.151	5.277E-49	T Cells
ZNF416	3.7089E-53	0.80323341	0.016	0.072	5.701E-49	T Cells
DNPH14	3.7743E-53	1.65084385	0.051	0.127	5.8015E-49	T Cells
MANEAL	4.2573E-53	0.83373258	0.02	0.078	6.5439E-49	T Cells
ZNF4401	4.3788E-53	0.84000991	0.019	0.076	6.7307E-49	T Cells
ARHGEF405	6.2653E-53	0.64387378	0.028	0.091	9.6304E-49	T Cells
CDKN2A	6.2746E-53	1.18970413	0.026	0.089	9.6447E-49	T Cells
ANAPC102	8.225E-53	0.46587795	0.013	0.067	1.2643E-48	T Cells
TRMT10A	8.6009E-53	0.93716249	0.018	0.075	1.322E-48	T Cells
NRF11	1.2027E-52	1.91281969	0.088	0.184	1.8486E-48	T Cells
CCAR11	1.3124E-52	1.00464081	0.024	0.085	2.0173E-48	T Cells
RFC4	1.9691E-52	1.26314522	0.026	0.087	3.0267E-48	T Cells
CSNK2A11	2.6252E-52	1.11830472	0.025	0.086	4.0352E-48	T Cells

DZANK11	2.699E-52	0.50390418	0.016	0.072	4.1487E-48	T Cells
NLRP13	2.7919E-52	1.59311526	0.352	0.657	4.2915E-48	T Cells
ERO1B1	3.1677E-52	1.85420105	0.063	0.144	4.869E-48	T Cells
SSBP31	3.1695E-52	1.57022538	0.046	0.118	4.8718E-48	T Cells
RALGAPA12	3.7918E-52	1.86934941	0.067	0.151	5.8284E-48	T Cells
ZNF7912	5.0501E-52	1.90438779	0.084	0.176	7.7626E-48	T Cells
IBA572	7.1796E-52	1.44663732	0.03	0.093	1.1036E-47	T Cells
MSANTD2	8.719E-52	1.81036989	0.068	0.151	1.3402E-47	T Cells
FANCD2	9.1717E-52	0.94926773	0.019	0.076	1.4098E-47	T Cells
RTL8A2	1.1261E-51	0.97049226	0.02	0.077	1.731E-47	T Cells
ZNF4201	1.3242E-51	1.29351636	0.025	0.085	2.0355E-47	T Cells
CBLN32	1.5827E-51	0.65076179	0.013	0.066	2.4328E-47	T Cells
KCTD21	1.6259E-51	1.5158756	0.034	0.1	2.4991E-47	T Cells
P2RY144	1.7152E-51	0.11631242	0.012	0.063	2.6365E-47	T Cells
CNOT71	1.742E-51	1.07343944	0.021	0.079	2.6777E-47	T Cells
TCP11L23	2.4765E-51	1.97091323	0.102	0.202	3.8066E-47	T Cells
C14orf282	3.9027E-51	1.46424472	0.023	0.082	5.9988E-47	T Cells
ZNF5001	5.0596E-51	1.12986481	0.026	0.087	7.7771E-47	T Cells
KHDC42	5.5897E-51	2.16401978	0.121	0.233	8.5919E-47	T Cells
NAA253	5.6573E-51	1.80079097	0.063	0.143	8.6958E-47	T Cells
CDCA7	8.5359E-51	5.70600291	0.016	0.003	1.3121E-46	T Cells
CKS2	8.5742E-51	1.20864319	0.038	0.105	1.3179E-46	T Cells
PSTK1	8.983E-51	0.63351122	0.019	0.075	1.3808E-46	T Cells
TMEM1871	9.6207E-51	1.07419802	0.02	0.077	1.4788E-46	T Cells
PPM1K1	1.1456E-50	2.10135132	0.094	0.192	1.7609E-46	T Cells
PIGA	1.4364E-50	1.24210042	0.023	0.081	2.2079E-46	T Cells
NCOA34	1.4806E-50	2.1613089	0.228	0.411	2.2759E-46	T Cells
ZNF5822	1.5319E-50	0.83702658	0.017	0.072	2.3547E-46	T Cells
SEMA4B4	2.2346E-50	1.4249183	0.04	0.108	3.4349E-46	T Cells
MAGOHB1	2.671E-50	0.53710889	0.013	0.065	4.1055E-46	T Cells
ZNF8161	3.272E-50	1.27181972	0.028	0.088	5.0294E-46	T Cells
FBXO311	3.3463E-50	1.80600924	0.05	0.123	5.1436E-46	T Cells
TAS2R142	4.5958E-50	1.39921595	0.042	0.111	7.0642E-46	T Cells
OTUB21	5.2991E-50	0.23090443	0.013	0.063	8.1452E-46	T Cells
GIN1	6.1142E-50	1.03897196	0.021	0.078	9.3981E-46	T Cells
ZNF6441	6.5463E-50	1.92682597	0.328	0.609	1.0062E-45	T Cells
MORC4	7.0696E-50	1.10700559	0.023	0.081	1.0867E-45	T Cells
TBC1D25	7.8618E-50	1.232519	0.021	0.078	1.2084E-45	T Cells
DBF4	9.2399E-50	1.74657917	0.055	0.131	1.4203E-45	T Cells
PARS2	9.5641E-50	0.43901839	0.011	0.061	1.4701E-45	T Cells
ZNF8501	1.325E-49	0.58863667	0.013	0.064	2.0367E-45	T Cells
KIF271	2.2693E-49	0.65691252	0.017	0.071	3.4881E-45	T Cells
TMEM1822	2.4066E-49	0.78443588	0.016	0.069	3.6992E-45	T Cells
RASA21	2.8357E-49	2.18090326	0.193	0.348	4.3587E-45	T Cells
ZFYVE92	3.4171E-49	0.93044173	0.015	0.068	5.2524E-45	T Cells
ZNF7241	5.0476E-49	0.43452664	0.013	0.063	7.7586E-45	T Cells
ZNF7471	5.1495E-49	0.62505844	0.016	0.069	7.9154E-45	T Cells
PLSCR33	5.4911E-49	2.13381275	0.127	0.24	8.4403E-45	T Cells
ZNF2751	5.9307E-49	1.45729294	0.03	0.09	9.1161E-45	T Cells
OGA4	8.5666E-49	2.03449989	0.533	0.761	1.3168E-44	T Cells
PELI24	9.1781E-49	0.9093051	0.02	0.074	1.4108E-44	T Cells
DNAAF21	9.2804E-49	1.38730976	0.028	0.087	1.4265E-44	T Cells

NDE13	9.6051E-49	2.06153386	0.215	0.386	1.4764E-44	T Cells
RECK1	1.0178E-48	1.04663951	0.021	0.077	1.5644E-44	T Cells
HSF2	1.0421E-48	1.65499905	0.039	0.105	1.6018E-44	T Cells
NATD1	1.0548E-48	0.4959183	0.017	0.07	1.6214E-44	T Cells
ZSCAN221	1.2488E-48	0.92209985	0.021	0.075	1.9195E-44	T Cells
ACYP11	1.3055E-48	1.7599668	0.047	0.118	2.0067E-44	T Cells
CHST115	1.3309E-48	1.88227515	0.29	0.526	2.0457E-44	T Cells
TMEM1545	1.4148E-48	4.1568342	0.07	0.034	2.1747E-44	T Cells
ZNF563	1.7247E-48	0.33704074	0.01	0.058	2.651E-44	T Cells
ZGLP11	1.787E-48	0.61675917	0.016	0.067	2.7468E-44	T Cells
DGKE	1.799E-48	4.24530528	0.09	0.048	2.7652E-44	T Cells
CLK22	2.0318E-48	1.5344307	0.037	0.101	3.123E-44	T Cells
CEMIP23	2.9145E-48	2.1917036	0.118	0.225	4.4798E-44	T Cells
USP343	3.0071E-48	1.89401878	0.328	0.6	4.6222E-44	T Cells
CREB3L41	3.0644E-48	0.20642482	0.009	0.056	4.7103E-44	T Cells
ZNF171	3.072E-48	1.73226328	0.036	0.1	4.722E-44	T Cells
RNASEH2A	3.2335E-48	0.14141218	0.01	0.057	4.9702E-44	T Cells
IDS1	3.6786E-48	0.83218478	0.464	0.918	5.6544E-44	T Cells
BARD11	3.8952E-48	1.16186552	0.026	0.083	5.9873E-44	T Cells
POLG22	4.0133E-48	1.81305016	0.076	0.159	6.1688E-44	T Cells
TBC1D18	4.162E-48	3.94310962	0.106	0.062	6.3974E-44	T Cells
ZNF235	4.2827E-48	1.13520173	0.02	0.074	6.5829E-44	T Cells
ZNF191	4.3534E-48	0.50765083	0.016	0.067	6.6915E-44	T Cells
SFXN11	5.1164E-48	3.78950954	0.179	0.13	7.8644E-44	T Cells
PGGHG3	5.1827E-48	4.11394191	0.108	0.064	7.9663E-44	T Cells
ASMTL3	5.5129E-48	1.7635402	0.061	0.138	8.4739E-44	T Cells
MID22	7.071E-48	0.69216488	0.013	0.063	1.0869E-43	T Cells
WDR51	7.5685E-48	1.60468415	0.04	0.105	1.1634E-43	T Cells
ZNF280B	8.005E-48	0.51204912	0.01	0.057	1.2305E-43	T Cells
CEP953	9.0826E-48	1.97092719	0.088	0.178	1.3961E-43	T Cells
ARMC51	1.0372E-47	0.27554199	0.015	0.065	1.5942E-43	T Cells
RPP401	1.0763E-47	0.49037561	0.014	0.063	1.6544E-43	T Cells
MUC201	1.0844E-47	1.25704674	0.027	0.085	1.6668E-43	T Cells
NETO22	1.1614E-47	1.37973928	0.037	0.101	1.7852E-43	T Cells
FGFR11	1.1707E-47	0.76299936	0.017	0.07	1.7995E-43	T Cells
MLH32	1.2456E-47	1.78859035	0.054	0.127	1.9147E-43	T Cells
ZNF2021	1.446E-47	1.2963265	0.028	0.086	2.2227E-43	T Cells
PARD6A	1.9115E-47	1.08853022	0.025	0.082	2.9381E-43	T Cells
IL161	2.0761E-47	1.81076628	0.335	0.615	3.1911E-43	T Cells
TOMM7	2.1208E-47	0.83233821	0.46	0.908	3.26E-43	T Cells
ANKMY11	2.309E-47	1.96395391	0.086	0.174	3.5492E-43	T Cells
RP91	2.9084E-47	1.49468501	0.028	0.087	4.4704E-43	T Cells
ZBTB20	3.0022E-47	2.26270337	0.121	0.228	4.6147E-43	T Cells
ZNF718	3.0705E-47	1.40787651	0.027	0.085	4.7196E-43	T Cells
HECA1	3.321E-47	1.25079697	0.433	0.853	5.1047E-43	T Cells
TADA1	3.6501E-47	1.26444794	0.028	0.085	5.6106E-43	T Cells
SLC9A93	4.1064E-47	4.70705888	0.027	0.008	6.3119E-43	T Cells
ZNF780B1	4.1216E-47	1.40205338	0.032	0.092	6.3353E-43	T Cells
ZNF2841	4.266E-47	1.03073266	0.019	0.072	6.5573E-43	T Cells
KLHDC11	5.0092E-47	1.32764226	0.023	0.078	7.6997E-43	T Cells
NSD21	5.3313E-47	1.930263	0.074	0.157	8.1948E-43	T Cells
GTPBP8	5.3947E-47	1.88072459	0.048	0.117	8.2923E-43	T Cells

CNN24	5.815E-47	1.16651021	0.422	0.817	8.9382E-43	T Cells
USP452	5.8326E-47	1.82149386	0.038	0.102	8.9653E-43	T Cells
ZNF766	5.9554E-47	2.12701595	0.08	0.164	9.1541E-43	T Cells
KIZ2	6.7153E-47	2.03886354	0.077	0.161	1.0322E-42	T Cells
EEF1E1	8.3936E-47	1.10024807	0.019	0.071	1.2902E-42	T Cells
DFFB2	8.6844E-47	1.21123601	0.026	0.083	1.3349E-42	T Cells
EMP13	1.0592E-46	0.82064321	0.06	0.134	1.6281E-42	T Cells
PKD1	1.1711E-46	1.76588169	0.045	0.113	1.8001E-42	T Cells
PLEKHN13	1.4758E-46	4.63180774	0.03	0.01	2.2685E-42	T Cells
INPP5D2	1.6993E-46	2.1042718	0.223	0.397	2.6119E-42	T Cells
CEP193	2.1326E-46	0.58546755	0.012	0.059	3.278E-42	T Cells
JRKL2	2.3592E-46	1.99848873	0.067	0.145	3.6263E-42	T Cells
DYNC2H11	2.5377E-46	1.19232895	0.026	0.082	3.9007E-42	T Cells
ADM23	2.7442E-46	5.36945957	0.01	0.001	4.2181E-42	T Cells
PHLPP11	3.0226E-46	0.66738088	0.014	0.062	4.6461E-42	T Cells
ZSCAN201	3.1367E-46	0.42753029	0.011	0.057	4.8214E-42	T Cells
C16orf91	3.3081E-46	0.98639076	0.021	0.074	5.0849E-42	T Cells
C15orf411	3.5761E-46	1.11950112	0.023	0.077	5.4968E-42	T Cells
RNF144A1	3.7078E-46	5.28059424	0.019	0.005	5.6992E-42	T Cells
MPV17L	3.8787E-46	1.27947342	0.019	0.072	5.9619E-42	T Cells
SLC26A113	5.2159E-46	2.09217283	0.11	0.21	8.0174E-42	T Cells
PAICS1	5.3532E-46	0.92888473	0.015	0.064	8.2284E-42	T Cells
SLC25A14	7.5906E-46	0.86694618	0.017	0.067	1.1668E-41	T Cells
TAF9B1	7.7469E-46	0.91935452	0.016	0.065	1.1908E-41	T Cells
TMEM229B	1.1539E-45	1.92414687	0.064	0.14	1.7737E-41	T Cells
WDR42	1.465E-45	1.44212511	0.029	0.086	2.2518E-41	T Cells
TNFRSF10D2	1.5447E-45	0.17168599	0.012	0.059	2.3744E-41	T Cells
USP541	1.6454E-45	0.35324989	0.011	0.056	2.5292E-41	T Cells
ZNF1322	2.1046E-45	0.66451964	0.012	0.059	3.235E-41	T Cells
ZFAND41	2.5207E-45	1.56214183	0.032	0.091	3.8746E-41	T Cells
SUV39H2	2.8862E-45	0.88065929	0.012	0.059	4.4363E-41	T Cells
WDHD1	2.9907E-45	0.54522242	0.013	0.06	4.597E-41	T Cells
ANKRD491	4.3758E-45	2.19227997	0.098	0.19	6.726E-41	T Cells
VPS13A2	4.6996E-45	1.93338855	0.058	0.13	7.2238E-41	T Cells
CDC14B1	6.3769E-45	0.75241495	0.013	0.06	9.8019E-41	T Cells
ATXN73	7.1867E-45	2.14612431	0.219	0.386	1.1047E-40	T Cells
TRIM59	9.5608E-45	4.06972047	0.094	0.053	1.4696E-40	T Cells
CAPN23	1.0412E-44	1.00631481	0.446	0.863	1.6004E-40	T Cells
DDX6	1.0632E-44	1.65124475	0.383	0.726	1.6342E-40	T Cells
MXRA72	1.1116E-44	1.86282925	0.102	0.195	1.7087E-40	T Cells
HERC42	1.1752E-44	1.88050272	0.293	0.524	1.8065E-40	T Cells
MBD51	1.227E-44	1.56897842	0.035	0.095	1.886E-40	T Cells
MUSTN11	1.2694E-44	1.08137924	0.028	0.083	1.9511E-40	T Cells
TMTC4	1.3185E-44	1.62270995	0.029	0.086	2.0266E-40	T Cells
ZNF142	1.3257E-44	2.09066603	0.089	0.176	2.0378E-40	T Cells
WDR5B1	1.7567E-44	1.69360798	0.039	0.101	2.7002E-40	T Cells
PSMD51	2.2466E-44	0.86408065	0.013	0.059	3.4533E-40	T Cells
ZNF778	2.3628E-44	1.78549382	0.046	0.112	3.6318E-40	T Cells
ZNF6921	2.4539E-44	1.00497917	0.023	0.076	3.7719E-40	T Cells
KLC2	3.1567E-44	2.03403544	0.086	0.17	4.8522E-40	T Cells
EZH11	3.8614E-44	2.17943149	0.127	0.234	5.9354E-40	T Cells
ISYNA13	4.1692E-44	4.8934263	0.019	0.005	6.4085E-40	T Cells

CLK43	4.2351E-44	1.93981774	0.105	0.2	6.5098E-40	T Cells
SPIN31	4.3321E-44	0.63007731	0.014	0.061	6.6589E-40	T Cells
HDGFL3	4.7058E-44	1.89096967	0.057	0.127	7.2332E-40	T Cells
ARHGAP47	4.9531E-44	1.73382822	0.353	0.654	7.6135E-40	T Cells
RPP14	5.021E-44	0.56058087	0.011	0.056	7.7177E-40	T Cells
JMY1	5.0426E-44	2.17204404	0.1	0.193	7.7509E-40	T Cells
USP534	5.1061E-44	1.50703215	0.03	0.087	7.8485E-40	T Cells
SASS6	5.8404E-44	1.43327041	0.024	0.077	8.9773E-40	T Cells
ZNF3471	6.0921E-44	0.34394099	0.009	0.053	9.3642E-40	T Cells
MPHOSPH91	6.2379E-44	2.15026477	0.142	0.256	9.5883E-40	T Cells
DIPK1A	6.6063E-44	1.52867976	0.03	0.086	1.0155E-39	T Cells
PYCR3	7.241E-44	0.74175731	0.013	0.059	1.113E-39	T Cells
GNB1L1	9.1562E-44	1.41395293	0.032	0.09	1.4074E-39	T Cells
FKBP114	9.7511E-44	3.49191442	0.159	0.112	1.4988E-39	T Cells
LNX21	1.1343E-43	1.64505097	0.038	0.098	1.7435E-39	T Cells
CNPY4	1.1678E-43	1.53782953	0.031	0.088	1.795E-39	T Cells
ING51	1.4455E-43	2.14195651	0.088	0.174	2.2218E-39	T Cells
ZNF77	1.514E-43	0.75262511	0.016	0.063	2.3271E-39	T Cells
TRDMT11	1.6604E-43	1.98138721	0.057	0.128	2.5521E-39	T Cells
ZNF408	2.1725E-43	1.16546626	0.02	0.07	3.3393E-39	T Cells
BEX31	2.1873E-43	0.10316589	0.014	0.059	3.3622E-39	T Cells
OPTN	2.262E-43	1.53895951	0.377	0.7	3.4769E-39	T Cells
BICRAL1	2.4586E-43	2.19698399	0.207	0.363	3.7791E-39	T Cells
PGAP12	2.502E-43	4.62646246	0.035	0.013	3.8459E-39	T Cells
HIST1H4C	2.7972E-43	1.76819841	0.077	0.156	4.2995E-39	T Cells
TRAF43	3.0438E-43	3.92917788	0.053	0.024	4.6786E-39	T Cells
SFT2D3	3.0907E-43	1.20618356	0.024	0.076	4.7507E-39	T Cells
ZNF4851	3.2502E-43	0.56829426	0.012	0.057	4.9958E-39	T Cells
GPSM2	3.5942E-43	0.15412468	0.011	0.054	5.5247E-39	T Cells
FAAH2	4.4158E-43	5.55272036	0.012	0.002	6.7876E-39	T Cells
GGACT	4.8308E-43	0.74734122	0.018	0.067	7.4254E-39	T Cells
PPAT	4.9633E-43	1.62010766	0.023	0.074	7.629E-39	T Cells
ZNF699	5.0926E-43	1.617294	0.032	0.088	7.8278E-39	T Cells
ZNF2	5.2461E-43	0.3194138	0.01	0.053	8.0638E-39	T Cells
THUMPD21	5.9459E-43	0.97085432	0.021	0.071	9.1395E-39	T Cells
PHF192	6.0966E-43	3.01561191	0.201	0.156	9.3712E-39	T Cells
NRM1	6.3614E-43	1.51630543	0.034	0.091	9.7781E-39	T Cells
FAM160B11	6.9298E-43	1.95636946	0.06	0.131	1.0652E-38	T Cells
C11orf212	8.2598E-43	1.37059652	0.029	0.084	1.2696E-38	T Cells
ADCK5	1.0303E-42	1.47396161	0.033	0.089	1.5836E-38	T Cells
PXMP2	1.0531E-42	1.6582462	0.035	0.093	1.6188E-38	T Cells
IL15RA1	1.1704E-42	1.7672832	0.045	0.108	1.799E-38	T Cells
TIPIN	1.1713E-42	0.89381858	0.013	0.058	1.8004E-38	T Cells
L2HGDH	1.3273E-42	0.64830193	0.012	0.057	2.0402E-38	T Cells
RNF242	1.8096E-42	1.50011961	0.032	0.088	2.7815E-38	T Cells
PRXL2B1	1.8157E-42	1.21286822	0.027	0.081	2.7909E-38	T Cells
ANKRD46	1.9499E-42	1.60475574	0.03	0.085	2.9971E-38	T Cells
ZNF3021	2.1496E-42	0.87120534	0.016	0.064	3.3041E-38	T Cells
NAPEPLD	2.2082E-42	0.91353182	0.016	0.063	3.3942E-38	T Cells
OGT4	2.244E-42	2.72503289	0.445	0.552	3.4493E-38	T Cells
LINS12	2.3122E-42	2.21431985	0.18	0.318	3.554E-38	T Cells
DPH1	2.5148E-42	0.78768083	0.013	0.058	3.8655E-38	T Cells

RDH131	2.5412E-42	1.65214049	0.042	0.103	3.9061E-38	T Cells
CLASP1	2.5549E-42	2.19827704	0.116	0.215	3.9271E-38	T Cells
CARD193	2.684E-42	2.21522359	0.124	0.227	4.1255E-38	T Cells
FBXO48	2.9055E-42	1.30530503	0.02	0.069	4.466E-38	T Cells
SEMA4F	2.9128E-42	1.64270722	0.032	0.088	4.4772E-38	T Cells
CATSPERG1	3.4539E-42	1.4671604	0.035	0.093	5.3089E-38	T Cells
TMEM991	3.4897E-42	1.0501604	0.013	0.058	5.3641E-38	T Cells
TRAF21	3.8224E-42	1.91282106	0.06	0.13	5.8753E-38	T Cells
ZNF4701	4.0953E-42	1.02196171	0.016	0.063	6.2948E-38	T Cells
GPAT2	4.9319E-42	0.1489632	0.007	0.047	7.5809E-38	T Cells
NSD32	5.474E-42	2.26977749	0.193	0.338	8.4141E-38	T Cells
RNPC33	6.0951E-42	1.96626822	0.084	0.165	9.3688E-38	T Cells
HOOK21	6.573E-42	0.34566435	0.014	0.059	1.0103E-37	T Cells
TTYH23	7.2808E-42	1.19999915	0.021	0.07	1.1191E-37	T Cells
TUT72	7.6499E-42	1.02799128	0.457	0.895	1.1759E-37	T Cells
MICB	8.2432E-42	0.85531076	0.013	0.058	1.2671E-37	T Cells
FCHSD1	8.6288E-42	1.66729108	0.042	0.102	1.3263E-37	T Cells
GPR89B	9.1459E-42	1.55334605	0.028	0.082	1.4058E-37	T Cells
EXD31	1.0853E-41	1.71108979	0.056	0.124	1.6682E-37	T Cells
NEO11	1.1988E-41	1.08329362	0.019	0.066	1.8427E-37	T Cells
TNFAIP32	1.5274E-41	2.8331775	0.436	0.535	2.3477E-37	T Cells
YOD11	1.5304E-41	1.67671374	0.035	0.092	2.3523E-37	T Cells
NDST23	1.674E-41	1.89757268	0.048	0.111	2.5731E-37	T Cells
PTAR13	1.9127E-41	2.17871463	0.179	0.313	2.94E-37	T Cells
ZNF775	3.1725E-41	0.5202187	0.009	0.05	4.8765E-37	T Cells
ZNF3821	4.1032E-41	1.53447394	0.026	0.078	6.307E-37	T Cells
ZNF741	4.3507E-41	0.65910921	0.012	0.054	6.6875E-37	T Cells
GLI42	4.7102E-41	1.6845176	0.049	0.112	7.24E-37	T Cells
KRBA2	4.9133E-41	1.46254252	0.026	0.077	7.5522E-37	T Cells
ZNF5841	4.9156E-41	0.48071037	0.013	0.056	7.5557E-37	T Cells
TM7SF2	4.973E-41	1.48559346	0.029	0.082	7.644E-37	T Cells
RAB11FIP5	5.3171E-41	1.63882574	0.038	0.095	8.1729E-37	T Cells
KIF3A2	5.6711E-41	2.12360988	0.065	0.136	8.7171E-37	T Cells
SLC35D1	7.7117E-41	2.22495769	0.075	0.152	1.1854E-36	T Cells
IL7R2	8.0029E-41	2.69699824	0.494	0.661	1.2301E-36	T Cells
PSMC3IP	8.2955E-41	0.67706344	0.014	0.059	1.2751E-36	T Cells
ZNF430	8.5053E-41	1.67764785	0.032	0.086	1.3074E-36	T Cells
GEMIN21	1.0229E-40	0.89676478	0.017	0.062	1.5723E-36	T Cells
CEP683	1.0862E-40	2.03752486	0.062	0.131	1.6696E-36	T Cells
ZFP41	1.9405E-40	1.79345842	0.051	0.114	2.9827E-36	T Cells
PRKCH1	2.169E-40	2.11114055	0.259	0.452	3.3339E-36	T Cells
RFXAP1	2.5404E-40	1.73380211	0.033	0.088	3.9048E-36	T Cells
MED271	2.6105E-40	0.40024038	0.012	0.053	4.0126E-36	T Cells
ZNF794	2.6461E-40	1.49987078	0.022	0.071	4.0673E-36	T Cells
PCP21	3.151E-40	0.40148068	0.013	0.055	4.8434E-36	T Cells
RTL52	3.6091E-40	0.38885816	0.014	0.057	5.5475E-36	T Cells
SOX12	3.898E-40	0.67943897	0.014	0.058	5.9916E-36	T Cells
ARHGAP19	3.959E-40	1.52835371	0.029	0.081	6.0854E-36	T Cells
IL11RA1	4.4581E-40	2.27625567	0.115	0.211	6.8525E-36	T Cells
SLC25A38	4.7092E-40	2.31456113	0.139	0.246	7.2385E-36	T Cells
MEX3B4	6.6093E-40	0.20594402	0.01	0.051	1.0159E-35	T Cells
GDPD32	6.8774E-40	4.81201691	0.025	0.008	1.0571E-35	T Cells

NAGS1	7.5352E-40	0.93514143	0.01	0.051	1.1582E-35	T Cells
TUFT11	7.8136E-40	0.81207799	0.014	0.057	1.201E-35	T Cells
HDAC95	8.2511E-40	1.58036031	0.029	0.08	1.2683E-35	T Cells
LSM111	1.0385E-39	1.58280165	0.026	0.077	1.5963E-35	T Cells
PFKP3	1.332E-39	1.98510252	0.235	0.405	2.0474E-35	T Cells
HAUS31	1.5589E-39	2.22364348	0.102	0.189	2.3962E-35	T Cells
UBALD21	2.5971E-39	2.30982318	0.175	0.305	3.992E-35	T Cells
NUDT7	2.8974E-39	1.1665093	0.027	0.077	4.4535E-35	T Cells
ZHX21	3.1574E-39	2.05290233	0.071	0.143	4.8533E-35	T Cells
NFAT53	3.6573E-39	2.24434038	0.226	0.392	5.6216E-35	T Cells
SSBP4	3.9277E-39	2.06238357	0.068	0.138	6.0373E-35	T Cells
ATG16L1	4.9596E-39	2.25341503	0.114	0.208	7.6234E-35	T Cells
TRANK1	5.2303E-39	2.24853595	0.154	0.269	8.0395E-35	T Cells
PIWIL44	6.3086E-39	0.46243033	0.014	0.056	9.697E-35	T Cells
GPAM1	7.5311E-39	0.55714109	0.012	0.053	1.1576E-34	T Cells
TPRN1	7.7572E-39	0.52731454	0.015	0.057	1.1924E-34	T Cells
CHRNA5	9.639E-39	0.57267448	0.009	0.048	1.4816E-34	T Cells
CCDC571	9.7396E-39	1.4819871	0.041	0.098	1.4971E-34	T Cells
ZNF5171	1.0569E-38	0.89746612	0.015	0.057	1.6246E-34	T Cells
SERPINE12	1.0681E-38	0.79176622	0.011	0.051	1.6419E-34	T Cells
ULK3	1.0803E-38	1.42151691	0.032	0.085	1.6605E-34	T Cells
DNA21	1.1523E-38	1.33411705	0.021	0.068	1.7711E-34	T Cells
ASTE11	1.1927E-38	1.67311055	0.03	0.081	1.8333E-34	T Cells
TRMT131	1.2421E-38	2.1222562	0.085	0.163	1.9092E-34	T Cells
MEGF64	1.243E-38	3.97862958	0.074	0.04	1.9106E-34	T Cells
NAPB	1.4792E-38	1.85833169	0.04	0.097	2.2737E-34	T Cells
IPO4	1.6154E-38	0.69071171	0.013	0.054	2.4831E-34	T Cells
CXorf652	1.6867E-38	4.1682859	0.064	0.032	2.5927E-34	T Cells
MBNL14	1.7384E-38	1.44116484	0.574	0.916	2.6721E-34	T Cells
ZNF5021	2.042E-38	0.43631301	0.009	0.047	3.1387E-34	T Cells
CYB5D11	2.2395E-38	0.47967472	0.014	0.055	3.4424E-34	T Cells
NLRP34	2.3235E-38	0.52034087	0.016	0.058	3.5715E-34	T Cells
BX255925.32	2.86E-38	1.2564537	0.027	0.076	4.3961E-34	T Cells
ZFP621	2.9416E-38	2.14168358	0.068	0.138	4.5216E-34	T Cells
ITGA6	4.3639E-38	2.02778708	0.059	0.124	6.7077E-34	T Cells
RCAN2	4.3991E-38	4.50493533	0.047	0.021	6.7618E-34	T Cells
MUTYH	5.1086E-38	1.19540109	0.021	0.067	7.8524E-34	T Cells
LIN9	5.4713E-38	1.18666119	0.019	0.064	8.41E-34	T Cells
MAP3K213	6.0192E-38	1.07515315	0.016	0.058	9.2522E-34	T Cells
MBLAC2	6.8359E-38	1.05944583	0.018	0.063	1.0508E-33	T Cells
NEIL11	7.276E-38	0.35017919	0.014	0.056	1.1184E-33	T Cells
MYL6B1	8.0854E-38	0.59715674	0.013	0.054	1.2428E-33	T Cells
VCPKMT2	8.7893E-38	1.89809764	0.063	0.13	1.351E-33	T Cells
BHLHE402	9.5239E-38	1.09482888	0.608	0.937	1.4639E-33	T Cells
ZNF8362	9.563E-38	1.83931408	0.037	0.092	1.4699E-33	T Cells
CCDC841	1.5511E-37	1.79195792	0.049	0.109	2.3842E-33	T Cells
ZNF3241	1.5957E-37	1.90291792	0.041	0.097	2.4528E-33	T Cells
ZBTB5	1.675E-37	2.13840767	0.062	0.128	2.5746E-33	T Cells
ZFP141	2.1567E-37	1.70621595	0.034	0.087	3.3151E-33	T Cells
ZNF138	2.1995E-37	1.56530063	0.03	0.081	3.3808E-33	T Cells
AZIN23	2.2727E-37	4.99645209	0.023	0.007	3.4934E-33	T Cells
TTC21A	2.6583E-37	0.55022305	0.013	0.053	4.086E-33	T Cells

KRT101	3.3913E-37	2.17497236	0.076	0.148	5.2127E-33	T Cells
ZNF7901	4.7391E-37	0.91352488	0.014	0.054	7.2844E-33	T Cells
BRAF1	5.3905E-37	1.92657653	0.049	0.109	8.2858E-33	T Cells
PRDM151	7.7338E-37	1.62924822	0.034	0.085	1.1888E-32	T Cells
NUDT171	8.7769E-37	1.05139749	0.013	0.053	1.3491E-32	T Cells
PFDN41	9.5705E-37	1.02640825	0.01	0.048	1.4711E-32	T Cells
ZDHHC231	9.7576E-37	0.78801949	0.015	0.055	1.4998E-32	T Cells
PRKCA2	1.1224E-36	1.92518247	0.04	0.094	1.7252E-32	T Cells
PPP1R12B3	1.1441E-36	1.93739291	0.075	0.146	1.7585E-32	T Cells
ZCCHC71	1.1779E-36	2.31700721	0.126	0.224	1.8105E-32	T Cells
CHTF181	1.2063E-36	0.79132547	0.012	0.052	1.8543E-32	T Cells
CDC42SE22	1.223E-36	2.76559321	0.448	0.573	1.8799E-32	T Cells
APOBEC3C	1.2342E-36	2.24236662	0.262	0.451	1.8971E-32	T Cells
EEPD12	1.2901E-36	1.23307057	0.022	0.067	1.983E-32	T Cells
LARP1B	1.4964E-36	1.84243184	0.037	0.089	2.3001E-32	T Cells
AMT1	1.5075E-36	1.68106384	0.04	0.094	2.3171E-32	T Cells
ZNF287	1.5529E-36	0.86506528	0.015	0.056	2.387E-32	T Cells
PPOX	1.572E-36	1.88749768	0.052	0.113	2.4164E-32	T Cells
ZNF2761	1.6072E-36	2.17898844	0.173	0.297	2.4704E-32	T Cells
ZNF2501	1.744E-36	1.65971536	0.028	0.077	2.6806E-32	T Cells
SPAG161	1.904E-36	1.80748517	0.035	0.087	2.9266E-32	T Cells
RAB11FIP31	1.9133E-36	2.15556948	0.074	0.145	2.941E-32	T Cells
TFAP41	2.0074E-36	0.81722599	0.013	0.053	3.0856E-32	T Cells
S1PR23	2.2968E-36	1.89354076	0.047	0.105	3.5304E-32	T Cells
SLC15A24	2.6813E-36	0.20424704	0.009	0.046	4.1214E-32	T Cells
CPLANE21	2.7567E-36	0.48517478	0.009	0.045	4.2373E-32	T Cells
NAA801	3.462E-36	2.07241418	0.075	0.146	5.3214E-32	T Cells
ALMS1	3.5976E-36	2.30267043	0.099	0.181	5.5299E-32	T Cells
COQ3	4.1568E-36	0.7375525	0.009	0.046	6.3894E-32	T Cells
ZNF3451	4.1747E-36	1.95257506	0.049	0.107	6.4169E-32	T Cells
ZNF547	4.4859E-36	0.29509982	0.006	0.04	6.8952E-32	T Cells
ZNF6781	5.023E-36	1.84007968	0.037	0.089	7.7208E-32	T Cells
ZNF1812	6.068E-36	1.43355255	0.018	0.06	9.3272E-32	T Cells
TCEANC	6.1504E-36	1.4563693	0.021	0.066	9.4538E-32	T Cells
NEDD4L5	6.18E-36	0.60476999	0.011	0.048	9.4993E-32	T Cells
IGIP1	7.1834E-36	2.14507488	0.064	0.129	1.1042E-31	T Cells
STIL	8.0056E-36	0.18675366	0.008	0.044	1.2305E-31	T Cells
C4orf46	9.4674E-36	0.84484267	0.012	0.051	1.4552E-31	T Cells
CYP2R11	9.5548E-36	2.23536142	0.084	0.159	1.4687E-31	T Cells
BANK14	1.1573E-35	0.90470117	0.011	0.049	1.7788E-31	T Cells
NUDT81	1.2123E-35	0.9836509	0.013	0.052	1.8634E-31	T Cells
RASGEF1A	1.2913E-35	1.46259323	0.025	0.071	1.9849E-31	T Cells
BBOF11	1.3457E-35	0.44140744	0.012	0.051	2.0685E-31	T Cells
ZNF901	1.5E-35	0.29911307	0.01	0.047	2.3056E-31	T Cells
DCLRE1A2	1.6234E-35	1.52107575	0.017	0.059	2.4953E-31	T Cells
RPGRIP1L	1.6646E-35	1.0796499	0.014	0.054	2.5586E-31	T Cells
TMEM1161	1.6659E-35	2.1780498	0.062	0.126	2.5606E-31	T Cells
SH2D4A1	1.7518E-35	0.60622053	0.011	0.048	2.6926E-31	T Cells
TSC22D33	2.187E-35	1.85333718	0.544	0.815	3.3616E-31	T Cells
ANKRD122	2.3389E-35	2.3163717	0.231	0.393	3.5951E-31	T Cells
NSMF3	2.3728E-35	2.1453173	0.092	0.169	3.6472E-31	T Cells
ZNF572	3.1436E-35	1.01309801	0.014	0.053	4.8321E-31	T Cells

ZNF7853	3.3545E-35	1.52929979	0.031	0.079	5.1562E-31	T Cells
YWHAZ1	3.6956E-35	0.65849698	0.479	0.935	5.6806E-31	T Cells
CCDC34	4.4276E-35	0.53146696	0.011	0.048	6.8056E-31	T Cells
DUSP25	5.8431E-35	3.69994161	0.104	0.066	8.9814E-31	T Cells
AJM1	6.0786E-35	5.61778135	0.011	0.002	9.3435E-31	T Cells
ZNF696	6.6425E-35	1.2040078	0.022	0.065	1.021E-30	T Cells
ZNF4931	7.6813E-35	2.06087929	0.092	0.169	1.1807E-30	T Cells
HIST2H2BE4	8.3606E-35	1.62586702	0.073	0.141	1.2851E-30	T Cells
ZSCAN16	1.171E-34	1.7253231	0.029	0.076	1.8E-30	T Cells
ZNF1771	1.3577E-34	0.36586736	0.01	0.046	2.0869E-30	T Cells
ZNF2511	1.6572E-34	1.7443179	0.041	0.094	2.5472E-30	T Cells
OSBPL32	1.9678E-34	2.24284642	0.125	0.219	3.0248E-30	T Cells
ZNF8131	2.1336E-34	1.05444098	0.011	0.048	3.2796E-30	T Cells
NUMBL1	2.6537E-34	1.01743553	0.015	0.054	4.079E-30	T Cells
TRIM451	2.7305E-34	0.18583581	0.008	0.043	4.197E-30	T Cells
KRBOX41	2.8791E-34	1.11810079	0.017	0.057	4.4254E-30	T Cells
TTC143	3.7266E-34	2.08449058	0.219	0.37	5.7282E-30	T Cells
ZKSCAN3	3.878E-34	1.2754346	0.016	0.056	5.9608E-30	T Cells
AP1S32	4.0761E-34	1.06074674	0.014	0.053	6.2654E-30	T Cells
CDKN2B	4.0788E-34	1.48495476	0.019	0.059	6.2695E-30	T Cells
TYW51	4.1361E-34	1.44215168	0.02	0.062	6.3576E-30	T Cells
KAT6B1	4.2143E-34	2.24851421	0.205	0.346	6.4778E-30	T Cells
IMMP1L1	4.2336E-34	1.58310818	0.023	0.067	6.5075E-30	T Cells
RELL22	4.2876E-34	1.19935809	0.02	0.061	6.5905E-30	T Cells
MZF11	5.042E-34	0.10453861	0.008	0.043	7.7501E-30	T Cells
ZNF201	5.3708E-34	0.21307995	0.01	0.045	8.2554E-30	T Cells
ZNF144	5.3765E-34	2.19012354	0.063	0.125	8.2642E-30	T Cells
KIAA1324	5.3851E-34	4.11081497	0.02	0.006	8.2775E-30	T Cells
KIFC23	5.8768E-34	1.89587628	0.054	0.112	9.0332E-30	T Cells
SPECC12	7.4221E-34	0.96077571	0.012	0.048	1.1409E-29	T Cells
TSHZ32	7.5876E-34	0.3068149	0.008	0.042	1.1663E-29	T Cells
EEF1AKMT12	7.7215E-34	1.27805981	0.016	0.054	1.1869E-29	T Cells
CNNM21	9.0946E-34	1.9972927	0.039	0.091	1.3979E-29	T Cells
AMN11	9.9455E-34	1.79823267	0.035	0.084	1.5287E-29	T Cells
EBPL	9.981E-34	1.81751476	0.032	0.08	1.5342E-29	T Cells
AOC21	1.0691E-33	0.98486964	0.012	0.049	1.6434E-29	T Cells
KBTBD31	1.2331E-33	1.61742127	0.027	0.072	1.8954E-29	T Cells
DLG51	1.2472E-33	1.15896819	0.015	0.053	1.9171E-29	T Cells
TRIM371	1.2791E-33	2.0301145	0.054	0.112	1.9661E-29	T Cells
MTERF21	1.2805E-33	1.95677629	0.058	0.118	1.9683E-29	T Cells
TTC261	1.3838E-33	0.61100435	0.007	0.039	2.127E-29	T Cells
FAM122C	1.8166E-33	1.80099744	0.033	0.081	2.7922E-29	T Cells
MICAL24	1.9061E-33	2.13193248	0.114	0.2	2.9299E-29	T Cells
ZNF607	2.1456E-33	1.19426289	0.014	0.052	3.298E-29	T Cells
BTBD194	2.3387E-33	1.28972941	0.02	0.062	3.5948E-29	T Cells
ZNF6301	2.5651E-33	1.42209448	0.021	0.063	3.9427E-29	T Cells
ABHD17C	3.1509E-33	1.34581685	0.018	0.058	4.8433E-29	T Cells
CEP971	3.197E-33	1.76992092	0.034	0.082	4.9141E-29	T Cells
FAM104A	3.8685E-33	1.56181302	0.021	0.062	5.9463E-29	T Cells
PSAT12	4.9138E-33	4.11311897	0.042	0.019	7.553E-29	T Cells
DCUN1D21	5.4268E-33	1.69429681	0.033	0.08	8.3416E-29	T Cells
PNISR4	5.8184E-33	2.09669533	0.291	0.499	8.9435E-29	T Cells

PHC12	6.2398E-33	2.31349121	0.131	0.226	9.5913E-29	T Cells
SOGA31	6.2745E-33	3.8174159	0.071	0.04	9.6446E-29	T Cells
HSPA1L	6.6148E-33	1.46391809	0.021	0.062	1.0168E-28	T Cells
AIM22	7.9509E-33	1.63222145	0.027	0.071	1.2221E-28	T Cells
MAP7D3	8.4558E-33	1.99333428	0.048	0.102	1.2997E-28	T Cells
NRL	9.5273E-33	0.5467264	0.012	0.048	1.4644E-28	T Cells
CENPC2	9.9877E-33	2.29221933	0.101	0.18	1.5352E-28	T Cells
TSPAN131	1.3595E-32	0.26291755	0.006	0.037	2.0896E-28	T Cells
ZRANB3	1.8719E-32	0.65341564	0.008	0.041	2.8773E-28	T Cells
ZNF709	2.1649E-32	1.62423224	0.026	0.068	3.3277E-28	T Cells
PIP4P24	2.242E-32	1.05038431	0.015	0.052	3.4463E-28	T Cells
PLPP6	2.7772E-32	1.4119994	0.022	0.064	4.2688E-28	T Cells
SYNRG1	3.584E-32	1.99099483	0.348	0.619	5.5089E-28	T Cells
UTY	4.9369E-32	2.06019919	0.097	0.172	7.5885E-28	T Cells
TNRC6C1	5.0685E-32	2.34724856	0.121	0.208	7.7909E-28	T Cells
STXBP41	6.0392E-32	2.22675386	0.05	0.105	9.2829E-28	T Cells
BCORL1	6.6527E-32	1.39585672	0.023	0.064	1.0226E-27	T Cells
ZNF5772	6.6719E-32	1.4752886	0.027	0.07	1.0255E-27	T Cells
PRR31	6.7465E-32	1.58621331	0.022	0.062	1.037E-27	T Cells
FAAH3	7.3687E-32	0.94908005	0.014	0.05	1.1326E-27	T Cells
ADAT21	7.7584E-32	2.00892861	0.052	0.107	1.1925E-27	T Cells
AGAP21	8.9177E-32	2.31475312	0.164	0.274	1.3707E-27	T Cells
FANCM2	8.966E-32	1.76320007	0.022	0.063	1.3782E-27	T Cells
ATP11B1	9.1921E-32	1.88538889	0.369	0.664	1.4129E-27	T Cells
RNF34	9.2132E-32	1.84734171	0.034	0.081	1.4162E-27	T Cells
SLAMF74	9.7529E-32	3.25247775	0.054	0.028	1.4991E-27	T Cells
ZNF3351	1.0449E-31	2.09526342	0.07	0.132	1.6061E-27	T Cells
PNMA3	1.1223E-31	4.81118659	0.022	0.007	1.7251E-27	T Cells
TRIB32	1.3574E-31	2.10825898	0.057	0.114	2.0865E-27	T Cells
DZIP31	1.425E-31	1.90563044	0.034	0.081	2.1903E-27	T Cells
SQLE	1.5033E-31	1.86641914	0.044	0.095	2.3107E-27	T Cells
EEF1G1	1.9755E-31	0.77467631	0.482	0.926	3.0366E-27	T Cells
SAPCD11	2.0146E-31	0.93296204	0.013	0.048	3.0966E-27	T Cells
NT5DC32	2.0545E-31	1.72191464	0.025	0.067	3.158E-27	T Cells
GSTK1	2.0558E-31	0.78716272	0.485	0.942	3.16E-27	T Cells
HNRNPLL3	2.228E-31	2.33249365	0.282	0.481	3.4247E-27	T Cells
MST11	2.7043E-31	0.22741687	0.009	0.042	4.1568E-27	T Cells
RNF19A1	2.8684E-31	2.20635119	0.286	0.492	4.409E-27	T Cells
ZDHHC142	3.3832E-31	1.70967122	0.028	0.071	5.2004E-27	T Cells
THAP91	3.7965E-31	0.47892074	0.011	0.045	5.8356E-27	T Cells
UBFD11	3.9477E-31	1.15616332	0.015	0.051	6.0681E-27	T Cells
CCDC1911	4.3124E-31	1.01038089	0.014	0.049	6.6285E-27	T Cells
NBPF111	5.1881E-31	0.39461894	0.01	0.043	7.9747E-27	T Cells
OGG12	5.191E-31	2.36924508	0.123	0.211	7.9791E-27	T Cells
AMER11	5.7848E-31	1.28436214	0.016	0.053	8.8918E-27	T Cells
ZNF7141	6.4186E-31	1.04041428	0.013	0.048	9.866E-27	T Cells
FNBP43	7.0027E-31	2.26780735	0.218	0.361	1.0764E-26	T Cells
MAD2L1	7.2777E-31	1.36842989	0.022	0.062	1.1187E-26	T Cells
ZNF232	7.421E-31	1.40181548	0.016	0.053	1.1407E-26	T Cells
CEP701	7.8698E-31	1.60065141	0.022	0.062	1.2097E-26	T Cells
CCDC66	7.9472E-31	2.30915563	0.087	0.156	1.2216E-26	T Cells
FAHD2B	8.0394E-31	1.49813144	0.017	0.053	1.2357E-26	T Cells

C19orf441	8.3578E-31	0.64279355	0.009	0.041	1.2847E-26	T Cells
CENPO	8.4455E-31	1.35637894	0.02	0.059	1.2982E-26	T Cells
NPHP31	9.7672E-31	2.24466459	0.113	0.195	1.5013E-26	T Cells
RAD52	1.035E-30	1.40460999	0.022	0.062	1.5909E-26	T Cells
ARHGAP94	1.0909E-30	2.03563415	0.302	0.513	1.6768E-26	T Cells
FRMD4B2	1.3722E-30	2.50323836	0.165	0.275	2.1093E-26	T Cells
KAT2B3	1.495E-30	3.15579258	0.36	0.406	2.298E-26	T Cells
ELOVL54	1.5483E-30	1.97878677	0.342	0.599	2.3799E-26	T Cells
ADHFE12	1.6291E-30	4.35251335	0.036	0.016	2.5041E-26	T Cells
C1orf159	1.7133E-30	0.87828193	0.011	0.044	2.6335E-26	T Cells
MTMR81	2.1679E-30	0.69176798	0.009	0.041	3.3323E-26	T Cells
ACVR2A	2.8404E-30	2.03961431	0.046	0.097	4.3659E-26	T Cells
ATAD2	3.4301E-30	1.63561093	0.034	0.08	5.2724E-26	T Cells
NMUR1	3.5427E-30	5.39166808	0.012	0.003	5.4454E-26	T Cells
KCNC41	3.6983E-30	2.09334032	0.05	0.103	5.6847E-26	T Cells
TNFAIP8L1	3.8539E-30	1.4024206	0.025	0.066	5.9238E-26	T Cells
ZNF5011	3.9858E-30	0.95614382	0.01	0.042	6.1265E-26	T Cells
ZNF6002	4.5597E-30	2.10272277	0.052	0.105	7.0087E-26	T Cells
RALGPS12	5.1119E-30	1.9917584	0.045	0.095	7.8575E-26	T Cells
MTHFD2L1	5.212E-30	0.94861127	0.016	0.051	8.0113E-26	T Cells
CENPL	5.3299E-30	1.80374743	0.035	0.08	8.1925E-26	T Cells
CDK11A	5.5442E-30	1.38319401	0.026	0.066	8.522E-26	T Cells
ST3GAL44	7.1089E-30	1.93567316	0.036	0.082	1.0927E-25	T Cells
ATP6V1E2	7.1107E-30	0.84939603	0.01	0.043	1.093E-25	T Cells
MTURN2	7.275E-30	1.46390941	0.027	0.068	1.1182E-25	T Cells
RBL12	8.704E-30	2.26251373	0.098	0.172	1.3379E-25	T Cells
MTBP2	8.9943E-30	1.25627614	0.011	0.044	1.3825E-25	T Cells
GSTM3	9.2138E-30	0.26910449	0.008	0.038	1.4163E-25	T Cells
PLPPR23	9.5069E-30	1.34565925	0.027	0.069	1.4613E-25	T Cells
FKBP1B2	1.2151E-29	0.6594779	0.009	0.04	1.8677E-25	T Cells
SPATS24	1.3614E-29	1.52556628	0.025	0.064	2.0926E-25	T Cells
EGR14	1.3808E-29	1.19509042	0.046	0.096	2.1224E-25	T Cells
ZNF2661	1.3872E-29	2.32913943	0.111	0.19	2.1323E-25	T Cells
CREBZF2	1.8913E-29	2.11096056	0.219	0.359	2.9071E-25	T Cells
EPM2AIP13	1.9053E-29	2.28809027	0.121	0.204	2.9286E-25	T Cells
CARF1	2.4483E-29	1.7886979	0.034	0.078	3.7632E-25	T Cells
TRGC1	2.864E-29	4.30541338	0.077	0.047	4.4023E-25	T Cells
POGLUT21	3.1187E-29	0.59713433	0.01	0.041	4.7938E-25	T Cells
MAGEH1	3.2303E-29	2.22572076	0.054	0.107	4.9653E-25	T Cells
ZNF4832	3.7215E-29	1.19720654	0.013	0.047	5.7203E-25	T Cells
GBP52	4.1416E-29	2.78657542	0.348	0.38	6.3661E-25	T Cells
ABCC44	5.1032E-29	2.19017213	0.067	0.125	7.8441E-25	T Cells
DEXI1	5.1143E-29	2.09818953	0.052	0.104	7.8612E-25	T Cells
TMEM238	5.5136E-29	2.14823885	0.059	0.114	8.475E-25	T Cells
MOK1	6.4758E-29	0.32678719	0.007	0.036	9.9539E-25	T Cells
ZNF30	7.0434E-29	1.19796669	0.011	0.042	1.0826E-24	T Cells
CDK31	9.2977E-29	0.71620079	0.01	0.041	1.4291E-24	T Cells
RICTOR1	1.041E-28	2.388441	0.147	0.243	1.6002E-24	T Cells
TES3	1.1733E-28	2.2290723	0.24	0.396	1.8034E-24	T Cells
ZNF7721	1.2567E-28	0.67553963	0.011	0.043	1.9317E-24	T Cells
ZNF875	1.2885E-28	1.07417399	0.012	0.045	1.9806E-24	T Cells
HIST1H1E2	1.3967E-28	2.29566	0.18	0.294	2.1468E-24	T Cells

BRD11	1.676E-28	1.9945016	0.349	0.616	2.5762E-24	T Cells
AC092835.1	2.3687E-28	0.96029973	0.009	0.038	3.641E-24	T Cells
IGFBP73	2.5967E-28	0.44528119	0.008	0.038	3.9914E-24	T Cells
BCL2L115	2.6704E-28	2.46790671	0.177	0.29	4.1047E-24	T Cells
ANKZF1	2.7573E-28	2.35137362	0.117	0.197	4.2382E-24	T Cells
KLHL351	2.8918E-28	0.19708375	0.005	0.032	4.4449E-24	T Cells
CIRBP2	2.9807E-28	0.95252562	0.477	0.917	4.5816E-24	T Cells
SBF1	3.0311E-28	2.35125073	0.149	0.246	4.6591E-24	T Cells
ZBTB391	3.6152E-28	1.91877916	0.037	0.082	5.557E-24	T Cells
SLCO4C12	4.7872E-28	0.2693443	0.007	0.036	7.3584E-24	T Cells
TBC1D2B1	4.7996E-28	1.94248037	0.355	0.618	7.3775E-24	T Cells
HIST1H2BH	4.9799E-28	0.55543775	0.033	0.074	7.6546E-24	T Cells
PIK3IP1	5.1187E-28	3.71350483	0.147	0.112	7.868E-24	T Cells
ZNF4841	5.1305E-28	2.13937124	0.047	0.096	7.8861E-24	T Cells
C8orf581	5.7848E-28	1.37172537	0.02	0.056	8.8918E-24	T Cells
SMG12	6.1494E-28	2.17416249	0.084	0.148	9.4522E-24	T Cells
WDCP	6.2829E-28	1.77523188	0.026	0.065	9.6575E-24	T Cells
TMEM79	6.987E-28	1.74454766	0.029	0.07	1.074E-23	T Cells
EZH22	7.0797E-28	1.87215811	0.062	0.116	1.0882E-23	T Cells
ZNF4072	7.9939E-28	2.27854551	0.167	0.274	1.2287E-23	T Cells
HDAC111	8.0901E-28	0.34194301	0.008	0.037	1.2435E-23	T Cells
GRAMD2B	8.2526E-28	1.52711584	0.02	0.055	1.2685E-23	T Cells
KLHDC4	8.2999E-28	2.32872621	0.179	0.294	1.2758E-23	T Cells
SYTL32	9.7595E-28	2.37621391	0.099	0.17	1.5001E-23	T Cells
KIAA0408	1.0384E-27	3.97197583	0.052	0.028	1.5961E-23	T Cells
LIPT2	1.0406E-27	0.42646441	0.005	0.032	1.5995E-23	T Cells
SLC5A33	1.1215E-27	3.47722107	0.202	0.174	1.7238E-23	T Cells
ZNF5191	1.1285E-27	1.20859483	0.02	0.055	1.7347E-23	T Cells
BANP2	1.1995E-27	1.00018688	0.012	0.044	1.8437E-23	T Cells
CMTM33	1.3215E-27	1.3191514	0.405	0.724	2.0313E-23	T Cells
FRMD82	1.5022E-27	1.17688482	0.018	0.052	2.309E-23	T Cells
MIB11	1.7537E-27	2.45403907	0.15	0.247	2.6955E-23	T Cells
TXNIP1	1.8644E-27	0.32094213	0.937	0.998	2.8658E-23	T Cells
C18orf54	1.8783E-27	1.64025423	0.021	0.056	2.8871E-23	T Cells
POC1B1	1.8827E-27	2.21196939	0.054	0.105	2.894E-23	T Cells
CTNNAL11	2.0343E-27	1.56862372	0.019	0.055	3.1269E-23	T Cells
CTSK2	2.0992E-27	2.09159445	0.028	0.068	3.2267E-23	T Cells
JUND1	2.2852E-27	0.59270799	0.658	0.983	3.5127E-23	T Cells
ANKEF1	2.3821E-27	1.34667227	0.012	0.044	3.6616E-23	T Cells
SEMA4D1	2.5842E-27	2.24470921	0.255	0.423	3.9721E-23	T Cells
TOB12	2.7132E-27	2.02747589	0.317	0.538	4.1705E-23	T Cells
ZNF10	2.8945E-27	1.38694843	0.015	0.048	4.4492E-23	T Cells
AP4S1	3.0254E-27	2.12355354	0.039	0.083	4.6503E-23	T Cells
ACTR3B	3.2296E-27	1.53639773	0.018	0.052	4.9643E-23	T Cells
METTL181	3.2659E-27	2.12118627	0.039	0.083	5.0199E-23	T Cells
CLECL13	3.4025E-27	0.90588129	0.008	0.037	5.2299E-23	T Cells
HYLS11	3.755E-27	1.89211088	0.026	0.064	5.7719E-23	T Cells
ZNF891	3.9858E-27	1.7293161	0.025	0.063	6.1265E-23	T Cells
ABCA52	4.0201E-27	3.81298369	0.094	0.062	6.1794E-23	T Cells
LRRC11	4.7083E-27	0.79285225	0.01	0.04	7.2371E-23	T Cells
NHSL23	4.8832E-27	3.63538034	0.058	0.033	7.506E-23	T Cells
CCSER23	7.1202E-27	2.91429995	0.396	0.481	1.0944E-22	T Cells

MARCKSL14	7.2246E-27	3.68688021	0.052	0.028	1.1105E-22	T Cells
ZNF5641	7.2393E-27	1.80714564	0.024	0.061	1.1128E-22	T Cells
SP1002	9.6481E-27	1.50060923	0.441	0.822	1.483E-22	T Cells
RAB302	1.0513E-26	0.6481748	0.012	0.041	1.616E-22	T Cells
CEP1261	1.1232E-26	1.22487864	0.012	0.042	1.7265E-22	T Cells
RALGPS21	1.2199E-26	0.99751642	0.015	0.047	1.8751E-22	T Cells
CHAC21	1.3804E-26	1.21739246	0.016	0.049	2.1219E-22	T Cells
LDOC1	1.412E-26	5.03303015	0.017	0.005	2.1704E-22	T Cells
MAP103	1.4711E-26	1.07165863	0.01	0.04	2.2613E-22	T Cells
CFL21	2.0653E-26	1.8937366	0.029	0.067	3.1746E-22	T Cells
FAM71F2	2.1531E-26	0.56488347	0.009	0.038	3.3095E-22	T Cells
HIST3H2BB1	2.1923E-26	4.86473969	0.013	0.004	3.3698E-22	T Cells
STXBP11	2.3411E-26	1.38842207	0.013	0.044	3.5985E-22	T Cells
HECTD22	2.3443E-26	1.7885648	0.023	0.059	3.6034E-22	T Cells
FAS3	2.8452E-26	2.27326169	0.127	0.208	4.3733E-22	T Cells
HOXA11	2.8541E-26	0.17649295	0.006	0.032	4.387E-22	T Cells
WDR271	3.2854E-26	1.63960666	0.031	0.071	5.0501E-22	T Cells
ATP8A13	3.6593E-26	3.86818398	0.103	0.07	5.6247E-22	T Cells
MELK	3.9109E-26	0.1971293	0.005	0.031	6.0114E-22	T Cells
ZMYM51	4.2566E-26	2.33509373	0.099	0.167	6.5428E-22	T Cells
VMAC	4.4507E-26	2.28602442	0.069	0.125	6.8412E-22	T Cells
TSPYL4	4.5021E-26	2.04702805	0.042	0.087	6.9202E-22	T Cells
EME21	4.5226E-26	1.12718343	0.016	0.048	6.9517E-22	T Cells
TSPYL21	4.6257E-26	2.35377625	0.076	0.135	7.1102E-22	T Cells
TNF2	5.1293E-26	2.25373222	0.092	0.158	7.8842E-22	T Cells
ZNF7821	5.2024E-26	1.64448187	0.025	0.061	7.9966E-22	T Cells
CDC25B1	5.487E-26	2.41070496	0.175	0.284	8.4341E-22	T Cells
ZNF4191	5.6361E-26	1.23939066	0.011	0.04	8.6632E-22	T Cells
TRIM662	5.8895E-26	1.58827875	0.021	0.056	9.0527E-22	T Cells
TMEM1691	7.1901E-26	0.52910179	0.008	0.034	1.1052E-21	T Cells
DUSP161	7.2156E-26	3.61155332	0.197	0.169	1.1091E-21	T Cells
TNFRSF93	9.6214E-26	5.51934828	0.01	0.003	1.4789E-21	T Cells
STARD92	1.0086E-25	4.34301963	0.047	0.025	1.5504E-21	T Cells
ARHGAP352	1.0979E-25	2.47370648	0.224	0.363	1.6875E-21	T Cells
DPF3	1.1683E-25	4.51969461	0.037	0.018	1.7958E-21	T Cells
SAMD12	1.2451E-25	1.36026268	0.016	0.048	1.9138E-21	T Cells
HELZ1	1.2728E-25	2.35201089	0.244	0.399	1.9565E-21	T Cells
TRIM471	1.3806E-25	1.94537605	0.04	0.084	2.1221E-21	T Cells
PIK3R3	1.6635E-25	1.22221091	0.014	0.045	2.5569E-21	T Cells
ZNRF31	1.7249E-25	0.29024286	0.003	0.027	2.6514E-21	T Cells
ZNF6531	1.9621E-25	1.94081042	0.035	0.075	3.016E-21	T Cells
TCEA21	1.9655E-25	2.08040486	0.045	0.09	3.0212E-21	T Cells
SMARCA22	2.0076E-25	1.61281832	0.429	0.789	3.0859E-21	T Cells
ZNF4611	2.0269E-25	0.40860002	0.007	0.034	3.1156E-21	T Cells
GRPEL2	2.4829E-25	2.28218739	0.05	0.098	3.8165E-21	T Cells
ZBTB252	2.5285E-25	2.27080938	0.23	0.372	3.8866E-21	T Cells
ANAPC11	2.9815E-25	1.25435108	0.012	0.04	4.5829E-21	T Cells
CCNF	3.7254E-25	0.73798517	0.012	0.04	5.7263E-21	T Cells
ZFP36L14	3.862E-25	2.34479729	0.437	0.561	5.9363E-21	T Cells
PLCL12	3.8678E-25	1.73678022	0.021	0.055	5.9453E-21	T Cells
PITPNC1	4.036E-25	2.32300466	0.27	0.444	6.2037E-21	T Cells
ITPKB1	4.613E-25	2.21119652	0.305	0.512	7.0906E-21	T Cells

POM121C1	4.9933E-25	1.62743937	0.02	0.053	7.6752E-21	T Cells
DUSP54	5.6887E-25	3.6061424	0.097	0.066	8.7441E-21	T Cells
SHLD31	5.7816E-25	2.00352309	0.026	0.062	8.8869E-21	T Cells
ENO31	6.6211E-25	1.44860788	0.017	0.049	1.0177E-20	T Cells
DYRK3	6.9281E-25	0.42480585	0.005	0.029	1.0649E-20	T Cells
ARMC2	7.9301E-25	1.39827992	0.016	0.047	1.2189E-20	T Cells
DISP2	7.9772E-25	0.18569343	0.006	0.03	1.2262E-20	T Cells
PTBP21	8.1224E-25	1.82399855	0.033	0.071	1.2485E-20	T Cells
NR1D1	8.7184E-25	2.19201067	0.06	0.111	1.3401E-20	T Cells
NEDD4	1.0638E-24	1.56135683	0.019	0.051	1.6352E-20	T Cells
PIDD11	1.4622E-24	1.44078322	0.016	0.047	2.2475E-20	T Cells
NEDD92	1.5238E-24	2.37217946	0.137	0.222	2.3422E-20	T Cells
CIP2A	1.9283E-24	1.20404948	0.011	0.038	2.964E-20	T Cells
DOCK104	1.9895E-24	1.76202719	0.402	0.717	3.0581E-20	T Cells
KRBA11	2.0162E-24	1.12762	0.014	0.044	3.0991E-20	T Cells
SCN8A1	2.2741E-24	0.13149458	0.005	0.028	3.4956E-20	T Cells
RTTN1	2.4341E-24	1.24836264	0.013	0.042	3.7415E-20	T Cells
RAD54B1	3.593E-24	1.29746939	0.009	0.035	5.5228E-20	T Cells
PLK4	3.8485E-24	0.99057684	0.01	0.036	5.9155E-20	T Cells
SLC35G11	3.8505E-24	0.83599463	0.007	0.032	5.9186E-20	T Cells
SLC9A3R1	3.8595E-24	1.95594908	0.358	0.625	5.9324E-20	T Cells
ZNF300	3.9545E-24	0.5842462	0.007	0.031	6.0785E-20	T Cells
ZBTB43	4.1978E-24	1.76212187	0.028	0.064	6.4524E-20	T Cells
AURKC	4.388E-24	0.67586506	0.006	0.029	6.7448E-20	T Cells
ARMCX41	4.4662E-24	0.35777815	0.007	0.032	6.865E-20	T Cells
AGAP1	4.698E-24	1.35637233	0.012	0.039	7.2212E-20	T Cells
JAK12	5.1451E-24	1.49418551	0.441	0.828	7.9085E-20	T Cells
ZNF432	5.3763E-24	1.88616663	0.019	0.05	8.2638E-20	T Cells
ZFY	6.0431E-24	1.79365169	0.02	0.052	9.2889E-20	T Cells
FAM169A2	6.3071E-24	5.37722636	0.014	0.004	9.6947E-20	T Cells
RAD9A1	6.6724E-24	1.77135356	0.038	0.078	1.0256E-19	T Cells
ERVK3-11	8.5185E-24	1.08934961	0.012	0.039	1.3094E-19	T Cells
POPDC22	8.6365E-24	1.61397014	0.018	0.049	1.3275E-19	T Cells
ZNF222	8.9295E-24	1.42055391	0.011	0.038	1.3725E-19	T Cells
SLC41A11	1.3896E-23	1.97315668	0.045	0.088	2.1359E-19	T Cells
TNFRSF1B4	1.4686E-23	2.1961116	0.272	0.447	2.2575E-19	T Cells
GNB51	1.9673E-23	3.76271835	0.131	0.1	3.0239E-19	T Cells
WDR90	2.0512E-23	1.02186279	0.011	0.037	3.1529E-19	T Cells
RBM481	2.2028E-23	2.05860891	0.03	0.067	3.3858E-19	T Cells
ASH1L3	2.271E-23	1.98401921	0.354	0.611	3.4907E-19	T Cells
KIF2A1	2.3026E-23	2.45813043	0.202	0.322	3.5393E-19	T Cells
MEX3C3	2.6242E-23	3.81345233	0.136	0.105	4.0336E-19	T Cells
SPTBN51	3.0854E-23	0.18458031	0.007	0.031	4.7426E-19	T Cells
CYB561D11	3.2469E-23	2.24115236	0.062	0.11	4.9908E-19	T Cells
MAP2K64	3.3222E-23	1.97632619	0.034	0.072	5.1065E-19	T Cells
NEMP21	3.4499E-23	2.16344968	0.049	0.093	5.3028E-19	T Cells
EIF5A21	4.2116E-23	2.33016533	0.052	0.097	6.4737E-19	T Cells
AGO23	4.745E-23	2.32881002	0.255	0.413	7.2935E-19	T Cells
ZFP69B1	4.903E-23	1.17390166	0.013	0.04	7.5365E-19	T Cells
KBTBD61	5.0978E-23	2.04452702	0.03	0.065	7.8358E-19	T Cells
NBPF3	5.1026E-23	0.9985686	0.012	0.039	7.8432E-19	T Cells
BBS121	5.6525E-23	1.39740277	0.014	0.042	8.6885E-19	T Cells

PRC1	6.5347E-23	0.31677026	0.008	0.033	1.0044E-18	T Cells
MAL	6.637E-23	3.33194068	0.052	0.029	1.0202E-18	T Cells
TIGD71	6.8859E-23	1.69588927	0.022	0.054	1.0584E-18	T Cells
NBPF201	6.889E-23	0.50152707	0.007	0.032	1.0589E-18	T Cells
ADGRG31	7.1901E-23	2.75347848	0.01	0.002	1.1052E-18	T Cells
TLE21	7.3798E-23	0.11204436	0.004	0.026	1.1343E-18	T Cells
TMC8	7.7605E-23	1.82589411	0.388	0.687	1.1929E-18	T Cells
FBXW71	8.9433E-23	2.13595993	0.325	0.548	1.3747E-18	T Cells
GRB103	9.0582E-23	1.19128749	0.019	0.049	1.3923E-18	T Cells
FBXO51	9.9775E-23	1.8062476	0.025	0.058	1.5336E-18	T Cells
ZNF43	1.0703E-22	1.66007845	0.016	0.044	1.6452E-18	T Cells
ATF7IP21	1.1012E-22	2.34811246	0.066	0.116	1.6926E-18	T Cells
PPP1R3F2	1.1603E-22	2.00617559	0.029	0.064	1.7835E-18	T Cells
ZNF6821	1.3227E-22	0.73759737	0.008	0.033	2.0332E-18	T Cells
DLEC1	1.3257E-22	3.66063627	0.024	0.01	2.0377E-18	T Cells
GALM2	1.3828E-22	2.4512489	0.083	0.14	2.1255E-18	T Cells
ZKSCAN21	1.4047E-22	0.28340515	0.005	0.026	2.1592E-18	T Cells
NR3C2	1.4133E-22	2.35152032	0.06	0.107	2.1724E-18	T Cells
WDR351	1.6447E-22	1.74706414	0.023	0.055	2.5281E-18	T Cells
RBKS1	2.2001E-22	0.62570655	0.007	0.031	3.3817E-18	T Cells
ZNF256	2.3809E-22	2.29171287	0.034	0.071	3.6596E-18	T Cells
FBXO321	2.6663E-22	2.47140577	0.099	0.161	4.0984E-18	T Cells
RELB4	2.6777E-22	1.88946623	0.095	0.156	4.1159E-18	T Cells
GATA3	2.7579E-22	3.45490022	0.191	0.166	4.2392E-18	T Cells
MYOM12	2.9805E-22	0.94856146	0.013	0.04	4.5814E-18	T Cells
KRT81	3.0815E-22	1.51285048	0.015	0.043	4.7365E-18	T Cells
KLRF11	3.3179E-22	5.78119097	0.01	0.003	5.1E-18	T Cells
ZNF2731	3.3915E-22	1.09194856	0.008	0.033	5.2131E-18	T Cells
ZNF674	3.4045E-22	1.25363185	0.013	0.04	5.2331E-18	T Cells
SFR11	3.4352E-22	0.39948297	0.007	0.03	5.2803E-18	T Cells
SGCB	3.4456E-22	4.31130079	0.042	0.023	5.2963E-18	T Cells
MAGI3	3.6185E-22	1.26729081	0.009	0.034	5.562E-18	T Cells
PLAG11	3.6223E-22	1.44882848	0.013	0.039	5.5678E-18	T Cells
PMAIP13	4.8657E-22	3.73549964	0.066	0.041	7.4791E-18	T Cells
CHAF1B	5.2379E-22	1.03197194	0.009	0.033	8.0511E-18	T Cells
ZNF789	6.4617E-22	0.38438883	0.006	0.029	9.9323E-18	T Cells
PTTG1	6.8709E-22	1.38135585	0.038	0.076	1.0561E-17	T Cells
EBLN22	7.2446E-22	1.98895401	0.047	0.088	1.1136E-17	T Cells
NIPSNAP3B4	8.8121E-22	0.60139798	0.007	0.029	1.3545E-17	T Cells
C15orf621	9.8001E-22	0.62889643	0.006	0.028	1.5064E-17	T Cells
LEPROTL1	1.4263E-21	1.32733833	0.461	0.868	2.1924E-17	T Cells
RASGRP34	1.5303E-21	1.60266471	0.023	0.054	2.3522E-17	T Cells
NUF2	1.6435E-21	0.1463171	0.005	0.026	2.5262E-17	T Cells
KAT6A3	2.3053E-21	2.29131505	0.284	0.467	3.5436E-17	T Cells
ATAD3B2	2.8995E-21	1.97828359	0.036	0.072	4.4568E-17	T Cells
MCOLN23	3.4851E-21	3.59892375	0.095	0.067	5.3569E-17	T Cells
RAVER21	3.8843E-21	1.13388112	0.016	0.044	5.9706E-17	T Cells
APOM	4.7061E-21	0.29231347	0.004	0.025	7.2337E-17	T Cells
POU2F25	6.7243E-21	3.69093154	0.047	0.026	1.0336E-16	T Cells
ARNTL24	7.0425E-21	1.44943381	0.013	0.038	1.0825E-16	T Cells
AC119396.11	7.2198E-21	1.45074297	0.028	0.06	1.1098E-16	T Cells
ZNF6692	9.6829E-21	1.91502897	0.018	0.046	1.4884E-16	T Cells

PRR72	1.1023E-20	4.30496534	0.016	0.006	1.6943E-16	T Cells
RAB39B1	1.3171E-20	4.4774299	0.034	0.017	2.0245E-16	T Cells
TRGJP11	1.3205E-20	1.98778973	0.026	0.058	2.0298E-16	T Cells
MARCH33	1.4976E-20	1.08895403	0.01	0.034	2.302E-16	T Cells
CBX72	1.7209E-20	2.46619723	0.108	0.172	2.6453E-16	T Cells
NUP35	1.7267E-20	2.17669916	0.03	0.063	2.6542E-16	T Cells
LRRIQ3	1.9406E-20	0.98153334	0.004	0.024	2.9829E-16	T Cells
DENND2C1	1.9547E-20	0.37987264	0.004	0.024	3.0045E-16	T Cells
TIMD42	1.9823E-20	0.2647334	0.007	0.029	3.0471E-16	T Cells
YPEL1	2.0267E-20	4.24567457	0.035	0.018	3.1152E-16	T Cells
DIP2C1	2.2431E-20	1.60652869	0.013	0.038	3.4479E-16	T Cells
PRKAB21	2.3366E-20	2.22206026	0.043	0.08	3.5916E-16	T Cells
CTU11	2.5176E-20	0.52999809	0.006	0.027	3.8698E-16	T Cells
RPA41	2.9588E-20	0.77147359	0.009	0.031	4.548E-16	T Cells
AL135905.2	3.6704E-20	1.03513692	0.009	0.031	5.6418E-16	T Cells
ABCB91	4.3881E-20	0.26897075	0.004	0.024	6.7449E-16	T Cells
DENND6B1	4.7929E-20	1.13156407	0.016	0.041	7.3671E-16	T Cells
CCDC301	4.9742E-20	0.80439869	0.009	0.032	7.6458E-16	T Cells
CASK	5.21E-20	3.4441874	0.221	0.207	8.0084E-16	T Cells
CDR21	5.4492E-20	2.55837098	0.152	0.236	8.3759E-16	T Cells
TNS2	5.6864E-20	1.03777151	0.008	0.03	8.7405E-16	T Cells
CCL281	6.1021E-20	4.60833526	0.021	0.009	9.3795E-16	T Cells
S100A51	6.2815E-20	0.88513415	0.007	0.028	9.6553E-16	T Cells
ZNF1691	6.4208E-20	1.57388299	0.02	0.047	9.8694E-16	T Cells
PUDP1	6.8286E-20	1.22442251	0.011	0.034	1.0496E-15	T Cells
GALNT35	6.8758E-20	4.0084861	0.021	0.009	1.0569E-15	T Cells
C12orf651	7.2597E-20	2.68852432	0.077	0.128	1.1159E-15	T Cells
NEURL11	7.3169E-20	0.56472827	0.005	0.025	1.1247E-15	T Cells
CA113	7.4522E-20	1.31366665	0.011	0.035	1.1455E-15	T Cells
LTO1	7.4912E-20	2.00905874	0.032	0.064	1.1515E-15	T Cells
HSF42	7.7396E-20	4.16678332	0.027	0.013	1.1897E-15	T Cells
BLM	8.6523E-20	1.81266827	0.013	0.038	1.3299E-15	T Cells
TNFSF91	1.179E-19	0.92709888	0.006	0.026	1.8123E-15	T Cells
ZNF5301	1.1817E-19	1.66080606	0.013	0.038	1.8164E-15	T Cells
NAP1L5	1.4203E-19	1.74419299	0.017	0.044	2.1831E-15	T Cells
MAPK8IP34	1.4492E-19	2.17409769	0.072	0.119	2.2276E-15	T Cells
AC015871.11	1.565E-19	1.69437069	0.015	0.041	2.4056E-15	T Cells
WNK13	1.9307E-19	1.43565455	0.455	0.847	2.9676E-15	T Cells
LRRC631	2.4909E-19	0.99527539	0.013	0.037	3.8288E-15	T Cells
ZBTB261	2.7962E-19	2.00245489	0.029	0.06	4.298E-15	T Cells
DCLK24	3.0207E-19	0.4540068	0.004	0.023	4.6431E-15	T Cells
C1QTNF61	3.0453E-19	4.28808621	0.022	0.01	4.681E-15	T Cells
KCTD73	3.1642E-19	3.51171342	0.135	0.108	4.8637E-15	T Cells
TLCD1	3.2816E-19	0.73214081	0.005	0.024	5.0442E-15	T Cells
ANKS61	3.3468E-19	0.80818559	0.006	0.025	5.1444E-15	T Cells
DEPDC1B	3.4993E-19	0.20128186	0.007	0.027	5.3788E-15	T Cells
ZNF620	3.6036E-19	1.54744262	0.013	0.037	5.5391E-15	T Cells
KIAA00402	4.2054E-19	3.52776919	0.197	0.18	6.4641E-15	T Cells
ARL10	4.4545E-19	2.14691016	0.027	0.057	6.847E-15	T Cells
KLHL111	4.664E-19	2.15415088	0.023	0.051	7.169E-15	T Cells
ZNF605	4.6998E-19	1.8323892	0.024	0.053	7.2241E-15	T Cells
KLHDC7B2	5.0066E-19	1.92751564	0.041	0.076	7.6956E-15	T Cells

CAPS	6.2998E-19	1.83484582	0.054	0.095	9.6835E-15	T Cells
AMIGO11	6.5955E-19	1.2487772	0.007	0.027	1.0138E-14	T Cells
GPR1552	6.6103E-19	3.84138217	0.081	0.055	1.0161E-14	T Cells
KIF11	6.8515E-19	0.94621492	0.017	0.043	1.0531E-14	T Cells
ANKH3	7.2191E-19	2.39565812	0.072	0.119	1.1097E-14	T Cells
C12orf73	7.7492E-19	0.29710029	0.004	0.023	1.1911E-14	T Cells
SLC39A82	8.3267E-19	2.74482144	0.135	0.108	1.2799E-14	T Cells
SREBF21	1.1001E-18	1.5815614	0.395	0.686	1.6909E-14	T Cells
ZNF587	1.1291E-18	2.12749221	0.026	0.055	1.7356E-14	T Cells
SLC16A131	1.2319E-18	1.70199951	0.019	0.045	1.8936E-14	T Cells
ASTL2	1.2895E-18	3.65065453	0.011	0.003	1.9821E-14	T Cells
VLDLR1	1.3499E-18	1.49377894	0.017	0.043	2.0749E-14	T Cells
ARHGAP11A	1.7283E-18	1.97663325	0.032	0.063	2.6565E-14	T Cells
AMMECR1	1.8411E-18	1.74509782	0.021	0.048	2.8299E-14	T Cells
CFLAR4	1.9729E-18	1.52819067	0.553	0.887	3.0326E-14	T Cells
EFCAB71	2.2344E-18	1.70575436	0.015	0.039	3.4345E-14	T Cells
ABRAXAS12	2.8369E-18	2.54082853	0.063	0.106	4.3606E-14	T Cells
HABP41	3.1221E-18	2.63901979	0.142	0.218	4.799E-14	T Cells
CHN1	3.2428E-18	4.70699355	0.021	0.009	4.9845E-14	T Cells
RAB3IP1	3.3379E-18	3.78192476	0.098	0.072	5.1306E-14	T Cells
C8orf371	3.5221E-18	0.89476353	0.007	0.027	5.4138E-14	T Cells
ROM11	3.5693E-18	0.86323913	0.009	0.029	5.4863E-14	T Cells
C21orf911	3.7827E-18	2.5520581	0.118	0.183	5.8143E-14	T Cells
CATSPER22	3.9609E-18	1.57496914	0.012	0.035	6.0882E-14	T Cells
AKR1C8P2	3.9715E-18	0.6778084	0.005	0.023	6.1047E-14	T Cells
LRRC693	4.2086E-18	0.99977598	0.011	0.033	6.4691E-14	T Cells
ANKS3	4.4986E-18	2.06597904	0.027	0.056	6.9148E-14	T Cells
FANCB	4.5855E-18	1.66665096	0.012	0.034	7.0484E-14	T Cells
NBEAL23	4.7707E-18	3.06556764	0.284	0.299	7.3331E-14	T Cells
LINC00672	5.613E-18	4.07930805	0.026	0.013	8.6277E-14	T Cells
CEP72	5.9836E-18	2.31791016	0.039	0.072	9.1974E-14	T Cells
DET11	7.0267E-18	1.84096809	0.013	0.036	1.0801E-13	T Cells
TTC321	8.2216E-18	1.72495827	0.019	0.044	1.2637E-13	T Cells
N4BP2L22	8.3465E-18	1.47776566	0.451	0.822	1.2829E-13	T Cells
RAB6B1	8.5046E-18	1.70577456	0.011	0.033	1.3072E-13	T Cells
CFAP441	9.6739E-18	1.73686977	0.022	0.048	1.487E-13	T Cells
CCNI1	1.0487E-17	0.43741306	0.528	0.959	1.6119E-13	T Cells
CLDND2	1.2196E-17	4.26590079	0.032	0.017	1.8746E-13	T Cells
ARSK	1.2961E-17	0.82750583	0.006	0.024	1.9922E-13	T Cells
TBKBP14	1.3505E-17	3.83296105	0.057	0.036	2.0758E-13	T Cells
FAM193B2	1.421E-17	2.32598618	0.214	0.33	2.1843E-13	T Cells
FAM53A	1.4325E-17	0.50505239	0.005	0.022	2.2019E-13	T Cells
CCDC167	1.4627E-17	2.62209767	0.109	0.168	2.2482E-13	T Cells
ZNF280C	1.5309E-17	1.90888899	0.018	0.043	2.3532E-13	T Cells
SPDL1	1.5968E-17	0.92220512	0.005	0.023	2.4544E-13	T Cells
SMC4	1.6117E-17	2.3234756	0.149	0.228	2.4773E-13	T Cells
SFMBT22	1.6219E-17	2.34965901	0.266	0.417	2.493E-13	T Cells
ZNF764	1.8069E-17	2.24253564	0.036	0.068	2.7774E-13	T Cells
IRAK1BP1	1.9865E-17	0.94820163	0.006	0.024	3.0535E-13	T Cells
CDC42BPG1	2.0186E-17	1.08435166	0.008	0.028	3.1029E-13	T Cells
YPEL35	2.1481E-17	3.13722744	0.236	0.23	3.3019E-13	T Cells
HCN31	2.1977E-17	1.24132441	0.01	0.031	3.3781E-13	T Cells

E2F2	2.5701E-17	0.65263851	0.006	0.024	3.9505E-13	T Cells
FCGBP3	2.5871E-17	2.38666016	0.024	0.012	3.9767E-13	T Cells
PSME1	2.6883E-17	0.58055037	0.512	0.952	4.1323E-13	T Cells
ZNF6651	2.8366E-17	0.59339312	0.006	0.024	4.3602E-13	T Cells
PRELID3A2	2.9283E-17	0.38842649	0.004	0.021	4.5011E-13	T Cells
TBX193	3.2016E-17	1.81185611	0.018	0.042	4.9211E-13	T Cells
KLKB11	3.261E-17	0.64582146	0.009	0.029	5.0124E-13	T Cells
ARHGEF91	3.2983E-17	2.16329562	0.039	0.071	5.0698E-13	T Cells
ZNF460	3.4332E-17	2.26544562	0.046	0.081	5.2771E-13	T Cells
KIF3C1	3.7304E-17	2.30650233	0.038	0.071	5.734E-13	T Cells
SINHCAF3	3.8111E-17	1.85329612	0.014	0.036	5.858E-13	T Cells
D2HGDH1	3.9185E-17	2.0571847	0.038	0.07	6.0232E-13	T Cells
NPTXR	4.0757E-17	4.95175528	0.01	0.003	6.2647E-13	T Cells
HSF51	4.1981E-17	0.86374049	0.007	0.025	6.4529E-13	T Cells
THNSL11	4.2889E-17	1.95876583	0.013	0.035	6.5925E-13	T Cells
LMTK21	4.6475E-17	2.44725523	0.055	0.093	7.1437E-13	T Cells
ZNF8291	5.6779E-17	1.77322031	0.009	0.028	8.7274E-13	T Cells
ABCA3	6.9488E-17	2.40045918	0.045	0.08	1.0681E-12	T Cells
SHF2	7.7499E-17	0.91205656	0.005	0.022	1.1912E-12	T Cells
ZNF852	7.8429E-17	1.66516619	0.009	0.029	1.2055E-12	T Cells
HMGA12	8.0554E-17	1.12511415	0.006	0.024	1.2382E-12	T Cells
RBM341	8.5368E-17	1.09825065	0.007	0.026	1.3122E-12	T Cells
TPM22	8.7794E-17	1.14203307	0.012	0.033	1.3495E-12	T Cells
ZNF2921	9.1287E-17	2.54019558	0.143	0.217	1.4032E-12	T Cells
CTC1	9.3014E-17	2.56229282	0.106	0.164	1.4297E-12	T Cells
SMCHD13	9.7017E-17	2.77475902	0.393	0.494	1.4912E-12	T Cells
TRPM25	9.8487E-17	1.51503275	0.015	0.037	1.5138E-12	T Cells
SLC12A81	9.9023E-17	0.45089038	0.005	0.022	1.5221E-12	T Cells
MINDY41	9.9093E-17	0.43034272	0.004	0.021	1.5232E-12	T Cells
ELK34	1.0953E-16	3.58505792	0.091	0.067	1.6836E-12	T Cells
B3GNT9	1.1295E-16	4.26775465	0.033	0.018	1.7362E-12	T Cells
SCX1	1.1569E-16	1.30911671	0.007	0.025	1.7783E-12	T Cells
PIK3R65	1.1994E-16	1.49266024	0.013	0.034	1.8436E-12	T Cells
C1R1	1.2278E-16	0.49064548	0.006	0.023	1.8872E-12	T Cells
C2orf921	1.2847E-16	1.04231025	0.009	0.028	1.9746E-12	T Cells
C21orf58	1.3906E-16	1.01869733	0.006	0.023	2.1374E-12	T Cells
BEND32	1.4876E-16	0.41267818	0.006	0.023	2.2865E-12	T Cells
MARS2	1.5464E-16	1.7206146	0.012	0.033	2.377E-12	T Cells
SMARCD31	1.5683E-16	1.0213069	0.01	0.03	2.4107E-12	T Cells
IRAK23	1.6151E-16	1.82121646	0.024	0.049	2.4826E-12	T Cells
PCSK1N	1.6877E-16	4.04464579	0.011	0.004	2.5941E-12	T Cells
KDM8	1.7322E-16	1.41404696	0.01	0.03	2.6626E-12	T Cells
CSRP22	1.8632E-16	0.20954853	0.006	0.023	2.864E-12	T Cells
FAM216A1	2.2656E-16	1.87118759	0.015	0.037	3.4825E-12	T Cells
PRX	3.1468E-16	1.3022389	0.012	0.032	4.8369E-12	T Cells
DNAJC27	3.574E-16	1.86751429	0.014	0.035	5.4936E-12	T Cells
IFITM24	3.9138E-16	2.47421201	0.238	0.367	6.0158E-12	T Cells
FP565260.11	5.165E-16	1.20621978	0.009	0.027	7.9392E-12	T Cells
PKP41	5.3393E-16	1.18292528	0.007	0.024	8.2071E-12	T Cells
CCL4L22	5.5751E-16	1.58555374	0.01	0.029	8.5695E-12	T Cells
ZNF7932	6.8472E-16	4.66729992	0.011	0.004	1.0525E-11	T Cells
MYORG1	7E-16	1.66080687	0.008	0.026	1.076E-11	T Cells

ADSSL1	7.1155E-16	0.78662058	0.006	0.022	1.0937E-11	T Cells
TMEM86B3	1.0178E-15	2.23484718	0.043	0.075	1.5644E-11	T Cells
GPC1	1.0229E-15	2.24349457	0.031	0.058	1.5723E-11	T Cells
RAPGEFL11	1.0344E-15	1.16466099	0.007	0.024	1.5899E-11	T Cells
CD992	1.1601E-15	1.86167455	0.502	0.754	1.7832E-11	T Cells
MARCH94	1.2052E-15	3.61456986	0.091	0.067	1.8526E-11	T Cells
BUB1B	1.2263E-15	0.49784714	0.005	0.02	1.8849E-11	T Cells
PRKCZ	1.2484E-15	2.47378394	0.053	0.088	1.9189E-11	T Cells
ZIK1	1.5376E-15	1.83485636	0.013	0.034	2.3634E-11	T Cells
FKBP7	1.6075E-15	0.20353541	0.002	0.016	2.4709E-11	T Cells
MEF2B3	1.6556E-15	2.02126935	0.023	0.048	2.5448E-11	T Cells
PODXL21	1.7369E-15	0.89620455	0.008	0.025	2.6698E-11	T Cells
ZNF596	1.9587E-15	1.03727622	0.006	0.023	3.0107E-11	T Cells
ZNF6811	2.0147E-15	1.42055967	0.008	0.025	3.0967E-11	T Cells
INTS6L3	2.0506E-15	3.68166716	0.08	0.058	3.152E-11	T Cells
AASS1	2.7919E-15	0.67034144	0.005	0.02	4.2915E-11	T Cells
TBC1D321	2.9698E-15	1.44900913	0.012	0.031	4.5649E-11	T Cells
ZNF540	3.0846E-15	1.85048361	0.015	0.036	4.7413E-11	T Cells
SLC7A15	3.4567E-15	2.55235254	0.099	0.15	5.3133E-11	T Cells
CBR31	3.8702E-15	1.36764277	0.01	0.028	5.9489E-11	T Cells
CAVIN21	3.9629E-15	0.14088199	0.005	0.021	6.0913E-11	T Cells
CCDC1864	4.5458E-15	2.60214927	0.125	0.187	6.9874E-11	T Cells
ACTL10	4.7365E-15	1.734887	0.012	0.031	7.2804E-11	T Cells
ZNF2963	5.3617E-15	1.71746589	0.021	0.043	8.2414E-11	T Cells
S100Z4	6.2285E-15	0.46860595	0.004	0.019	9.5739E-11	T Cells
GLIPR1L21	6.2533E-15	0.75310028	0.005	0.02	9.6119E-11	T Cells
SLC20A14	6.4064E-15	2.21499762	0.281	0.439	9.8472E-11	T Cells
CBLB1	6.5447E-15	3.27638483	0.252	0.259	1.006E-10	T Cells
OTUD31	6.6198E-15	2.28426703	0.052	0.086	1.0175E-10	T Cells
BCL91	8.2279E-15	1.70867121	0.017	0.038	1.2647E-10	T Cells
ZNF1651	8.3552E-15	0.67092843	0.004	0.018	1.2843E-10	T Cells
GAMT1	8.4352E-15	4.52310338	0.01	0.004	1.2966E-10	T Cells
TFAP2E	9.7058E-15	1.51743525	0.01	0.029	1.4919E-10	T Cells
TPCN14	9.9608E-15	3.90154571	0.02	0.009	1.5311E-10	T Cells
MT-ND44	1.0885E-14	2.28384083	0.357	0.422	1.6732E-10	T Cells
TLR33	1.0975E-14	0.64599901	0.006	0.021	1.687E-10	T Cells
ZSCAN121	1.1513E-14	1.76722923	0.01	0.028	1.7697E-10	T Cells
FGD32	1.3301E-14	2.26931169	0.305	0.484	2.0446E-10	T Cells
PRKD2	1.6245E-14	2.48502762	0.219	0.329	2.4971E-10	T Cells
TMEM1171	1.6598E-14	1.14050142	0.006	0.022	2.5512E-10	T Cells
TMEM44	1.9927E-14	1.37512678	0.008	0.025	3.063E-10	T Cells
AK42	2.0072E-14	1.70503947	0.01	0.028	3.0853E-10	T Cells
USP27X	2.0402E-14	1.53149358	0.008	0.025	3.136E-10	T Cells
ANGPTL62	2.0779E-14	0.24054312	0.004	0.018	3.194E-10	T Cells
SMYD3	2.2093E-14	2.15327483	0.02	0.042	3.396E-10	T Cells
PYROXD23	2.4375E-14	4.25290423	0.014	0.006	3.7467E-10	T Cells
TMSB4Y	2.5521E-14	0.69779373	0.004	0.019	3.9228E-10	T Cells
RAPSN1	2.5703E-14	1.10053296	0.007	0.023	3.9509E-10	T Cells
PRKAR1B1	2.6887E-14	2.1348278	0.024	0.048	4.1327E-10	T Cells
ERBB2	3.1046E-14	0.12750907	0.004	0.018	4.7721E-10	T Cells
BEGAIN2	4.3606E-14	0.30148788	0.002	0.015	6.7027E-10	T Cells
IPP	4.5541E-14	2.550579	0.054	0.087	7.0001E-10	T Cells

RASA32	4.569E-14	2.71018741	0.173	0.257	7.023E-10	T Cells
LRATD2	4.7233E-14	4.00669638	0.053	0.035	7.2602E-10	T Cells
MTERF4	4.7541E-14	2.60804174	0.164	0.244	7.3075E-10	T Cells
ARF62	5.0222E-14	1.11971117	0.486	0.904	7.7196E-10	T Cells
LPIN11	5.6064E-14	2.51982482	0.131	0.193	8.6176E-10	T Cells
RCAN31	5.7133E-14	3.17396474	0.309	0.35	8.7819E-10	T Cells
ZNF8621	5.771E-14	2.39759908	0.066	0.104	8.8706E-10	T Cells
CACNB13	6.125E-14	3.70845813	0.051	0.033	9.4148E-10	T Cells
ZNF6672	6.325E-14	1.09500108	0.005	0.02	9.7222E-10	T Cells
NEK32	6.5427E-14	0.86768829	0.007	0.022	1.0057E-09	T Cells
L3MBTL1	6.6417E-14	1.93786373	0.023	0.045	1.0209E-09	T Cells
DDTL1	7.0364E-14	1.53671319	0.009	0.025	1.0816E-09	T Cells
LPAR52	7.1154E-14	2.55055632	0.054	0.087	1.0937E-09	T Cells
TAS2R30	8.3944E-14	2.1628491	0.024	0.047	1.2903E-09	T Cells
KANTR1	8.8845E-14	1.91055363	0.017	0.037	1.3656E-09	T Cells
WDR311	1.0108E-13	0.4192974	0.003	0.016	1.5538E-09	T Cells
NAT142	1.0331E-13	1.90408777	0.021	0.042	1.5879E-09	T Cells
CCZ1	1.1274E-13	0.15592737	0.003	0.015	1.7329E-09	T Cells
CSKMT	1.2789E-13	1.75396426	0.009	0.026	1.9658E-09	T Cells
ESYT23	1.3626E-13	2.27940442	0.483	0.725	2.0945E-09	T Cells
TGIF25	1.5013E-13	2.40687061	0.047	0.077	2.3076E-09	T Cells
GPR1351	1.5951E-13	1.48536192	0.012	0.029	2.4518E-09	T Cells
TNFRSF6B2	1.6118E-13	0.83296728	0.006	0.021	2.4775E-09	T Cells
FAM24B1	1.686E-13	2.04626348	0.023	0.046	2.5916E-09	T Cells
HOXB33	2.1333E-13	3.91289548	0.04	0.025	3.2791E-09	T Cells
SLF11	2.1385E-13	3.63932811	0.144	0.125	3.2871E-09	T Cells
TCP10L1	2.3837E-13	0.34662645	0.003	0.015	3.664E-09	T Cells
EID32	2.592E-13	2.07206596	0.035	0.06	3.9842E-09	T Cells
ZNF4312	2.6733E-13	1.83366474	0.015	0.033	4.1091E-09	T Cells
MYC3	2.8019E-13	2.64138666	0.093	0.14	4.3068E-09	T Cells
EID2B1	2.9521E-13	1.74252804	0.009	0.026	4.5377E-09	T Cells
PRR222	3.0523E-13	0.25187884	0.006	0.02	4.6917E-09	T Cells
HOXC4	3.1037E-13	1.39021155	0.006	0.021	4.7707E-09	T Cells
PASK1	3.3009E-13	2.34467542	0.041	0.069	5.0737E-09	T Cells
TNKS1BP12	3.3599E-13	1.1549545	0.007	0.021	5.1646E-09	T Cells
RHEBL11	3.4916E-13	1.7987715	0.012	0.03	5.3669E-09	T Cells
CCDC1201	3.5908E-13	1.23794476	0.012	0.03	5.5195E-09	T Cells
TAB23	3.5918E-13	2.63939629	0.437	0.612	5.521E-09	T Cells
H1FX3	3.5983E-13	1.77283604	0.382	0.633	5.5309E-09	T Cells
SLC25A42	3.669E-13	4.29083774	0.021	0.011	5.6397E-09	T Cells
SYNM	3.9945E-13	4.89912249	0.013	0.006	6.14E-09	T Cells
KLHL171	4.1071E-13	2.00290032	0.02	0.041	6.3131E-09	T Cells
CCNE2	4.1915E-13	0.73454919	0.004	0.018	6.4428E-09	T Cells
MXD3	4.242E-13	1.02095578	0.013	0.031	6.5204E-09	T Cells
C16orf541	4.2619E-13	2.3649721	0.244	0.368	6.5509E-09	T Cells
AP002495.11	4.8321E-13	2.1272491	0.027	0.05	7.4273E-09	T Cells
XRCC2	5.3454E-13	0.54866196	0.002	0.014	8.2165E-09	T Cells
CDCA41	5.8769E-13	2.28233856	0.033	0.058	9.0334E-09	T Cells
PARP152	6.47E-13	2.43577534	0.186	0.272	9.9451E-09	T Cells
AC073111.42	7.5201E-13	3.80325817	0.051	0.034	1.1559E-08	T Cells
WDR54	7.798E-13	2.33189978	0.033	0.058	1.1986E-08	T Cells
CCDC163	8.1513E-13	1.20888647	0.006	0.02	1.2529E-08	T Cells

C6orf1633	8.7121E-13	1.43564333	0.01	0.027	1.3391E-08	T Cells
SLC9B21	8.7385E-13	2.345919	0.029	0.053	1.3432E-08	T Cells
GAS64	8.7526E-13	0.49432183	0.003	0.015	1.3454E-08	T Cells
C3orf181	8.9337E-13	2.49562249	0.03	0.053	1.3732E-08	T Cells
SPHK14	9.2883E-13	0.44615387	0.003	0.016	1.4277E-08	T Cells
POLE2	9.6821E-13	1.78701567	0.009	0.024	1.4882E-08	T Cells
GPRIN1	9.9504E-13	2.145444	0.024	0.046	1.5295E-08	T Cells
ZNF367	1.0992E-12	1.75062019	0.02	0.04	1.6896E-08	T Cells
NLGN23	1.2653E-12	4.67605089	0.012	0.005	1.9449E-08	T Cells
NPIPB151	1.2709E-12	1.46668935	0.017	0.035	1.9535E-08	T Cells
EXOG1	1.3547E-12	1.84370149	0.018	0.038	2.0824E-08	T Cells
JRK	1.4346E-12	2.32150664	0.035	0.06	2.2052E-08	T Cells
PPP3CC	1.4946E-12	2.61653487	0.18	0.263	2.2974E-08	T Cells
SPACA62	1.5003E-12	0.38652556	0.003	0.015	2.3061E-08	T Cells
TRIM62	1.5091E-12	2.43820175	0.031	0.055	2.3197E-08	T Cells
EFEMP21	1.5572E-12	0.83909555	0.005	0.019	2.3936E-08	T Cells
CDK5R11	1.5898E-12	4.46449879	0.016	0.007	2.4437E-08	T Cells
MREG2	1.6231E-12	2.26938219	0.062	0.096	2.4948E-08	T Cells
FMN14	1.6516E-12	2.01939581	0.025	0.047	2.5387E-08	T Cells
UNC119B1	1.865E-12	2.49584903	0.044	0.071	2.8667E-08	T Cells
ADGRB11	1.9453E-12	1.20602285	0.008	0.022	2.9901E-08	T Cells
HOXB4	2.0501E-12	2.64750213	0.056	0.089	3.1512E-08	T Cells
DNAH12	2.1545E-12	2.1714417	0.055	0.087	3.3117E-08	T Cells
AC091057.62	3.0204E-12	2.12601397	0.033	0.057	4.6427E-08	T Cells
PPIL6	3.0694E-12	0.35494855	0.002	0.013	4.718E-08	T Cells
PHKA1	4.5636E-12	0.45665278	0.002	0.013	7.0147E-08	T Cells
PTPN13	4.9012E-12	4.27162074	0.018	0.009	7.5336E-08	T Cells
GCH13	5.0302E-12	2.33842699	0.039	0.065	7.7319E-08	T Cells
SCN9A4	5.0407E-12	0.9526249	0.004	0.016	7.7481E-08	T Cells
NKTR4	6.1246E-12	2.35591641	0.247	0.372	9.4142E-08	T Cells
C19orf571	6.5748E-12	0.89819971	0.002	0.013	1.0106E-07	T Cells
SMOX4	6.7297E-12	0.90554705	0.006	0.019	1.0344E-07	T Cells
NFKB24	6.7675E-12	2.32415527	0.147	0.212	1.0402E-07	T Cells
GAS11	7.0532E-12	0.8520335	0.003	0.015	1.0841E-07	T Cells
ORMDL31	7.3566E-12	2.66922883	0.187	0.273	1.1308E-07	T Cells
CCDC1221	7.5761E-12	0.94005355	0.005	0.018	1.1645E-07	T Cells
ZNF1071	7.7635E-12	2.39732343	0.04	0.066	1.1933E-07	T Cells
HIST4H41	7.982E-12	1.76421646	0.016	0.033	1.2269E-07	T Cells
PAIP2B	8.0577E-12	2.36452371	0.016	0.034	1.2385E-07	T Cells
CCDC1382	8.1788E-12	1.85345115	0.013	0.03	1.2572E-07	T Cells
KPNA51	8.2262E-12	2.62894415	0.098	0.142	1.2644E-07	T Cells
ZNF286A	8.2769E-12	2.13009592	0.016	0.034	1.2722E-07	T Cells
C5orf341	9.1222E-12	0.97404519	0.003	0.014	1.4022E-07	T Cells
MORN3	9.7112E-12	4.22807611	0.015	0.007	1.4927E-07	T Cells
TMEM2651	1.0285E-11	1.3010682	0.007	0.021	1.581E-07	T Cells
ZC3H12C3	1.0295E-11	0.46229286	0.002	0.013	1.5825E-07	T Cells
ARIH2OS	1.0768E-11	2.07329311	0.014	0.031	1.6552E-07	T Cells
TRIM61	1.1169E-11	0.63561166	0.003	0.014	1.7167E-07	T Cells
RBM334	1.1873E-11	2.12418144	0.483	0.728	1.825E-07	T Cells
FZD23	1.2761E-11	0.30676399	0.002	0.013	1.9615E-07	T Cells
ZDBF21	1.3908E-11	1.97792096	0.015	0.032	2.1379E-07	T Cells
KIF20B	1.3951E-11	2.47879309	0.058	0.089	2.1444E-07	T Cells

CENPK	1.4577E-11	2.01931776	0.013	0.03	2.2407E-07	T Cells
CASTOR12	1.5383E-11	2.26497232	0.025	0.046	2.3645E-07	T Cells
CLDN151	1.6005E-11	2.45357501	0.064	0.097	2.4602E-07	T Cells
ATP13A4	1.8463E-11	0.89934961	0.003	0.015	2.838E-07	T Cells
HIST1H4J	1.8491E-11	1.5671237	0.006	0.019	2.8422E-07	T Cells
SYNGAP11	1.9261E-11	2.52085335	0.089	0.13	2.9606E-07	T Cells
LETM21	1.9471E-11	2.07947928	0.013	0.029	2.9928E-07	T Cells
ALDOC	1.9728E-11	2.50821764	0.045	0.071	3.0324E-07	T Cells
PTGES3	2.0332E-11	0.71355958	0.002	0.013	3.1253E-07	T Cells
MRNIP1	2.1582E-11	2.64483485	0.106	0.154	3.3173E-07	T Cells
TGM11	2.1609E-11	1.17977167	0.008	0.022	3.3216E-07	T Cells
SHISAL2A1	2.3555E-11	2.62471095	0.046	0.073	3.6207E-07	T Cells
C12orf60	2.4553E-11	0.69908171	0.005	0.017	3.774E-07	T Cells
CCL32	2.6036E-11	1.07500116	0.016	0.033	4.0019E-07	T Cells
IL20RB1	2.6332E-11	1.75177891	0.008	0.022	4.0475E-07	T Cells
IFT80	2.672E-11	2.52190936	0.068	0.101	4.1071E-07	T Cells
RIBC11	2.756E-11	0.14643721	0.005	0.017	4.2363E-07	T Cells
MAP3K121	3.3196E-11	2.43386842	0.04	0.065	5.1025E-07	T Cells
ACTRT31	3.4349E-11	0.47475806	0.003	0.014	5.2798E-07	T Cells
NPM21	3.4909E-11	1.03354181	0.003	0.014	5.3659E-07	T Cells
PPP1R183	3.6894E-11	1.18567235	0.485	0.897	5.671E-07	T Cells
MT-ND65	4.0631E-11	3.1089291	0.165	0.152	6.2453E-07	T Cells
AP3M21	4.2591E-11	2.72325218	0.093	0.135	6.5466E-07	T Cells
SOCS11	4.3063E-11	2.89730033	0.303	0.346	6.6192E-07	T Cells
LBR4	4.5986E-11	2.49617734	0.24	0.354	7.0685E-07	T Cells
TESMIN1	6.1196E-11	1.4167465	0.008	0.021	9.4064E-07	T Cells
POU5F22	6.3661E-11	1.97275414	0.026	0.046	9.7853E-07	T Cells
BMP8B	6.4442E-11	1.39940761	0.006	0.018	9.9054E-07	T Cells
CELSR3	7.0327E-11	1.46096823	0.007	0.02	1.081E-06	T Cells
CELSR12	8.1455E-11	1.07770118	0.006	0.018	1.252E-06	T Cells
ZXDB	8.2454E-11	2.0023608	0.011	0.025	1.2674E-06	T Cells
REC82	8.6365E-11	2.3797622	0.071	0.105	1.3275E-06	T Cells
KANK2	8.7301E-11	0.26714546	0.002	0.012	1.3419E-06	T Cells
PPFIA31	9.5991E-11	0.47813557	0.002	0.013	1.4755E-06	T Cells
OR1B11	1.0061E-10	0.44567174	0.002	0.012	1.5465E-06	T Cells
EPC12	1.0235E-10	2.45860745	0.258	0.388	1.5731E-06	T Cells
NCAPH	1.0908E-10	1.51016677	0.009	0.022	1.6766E-06	T Cells
SDCBP21	1.2888E-10	1.0797881	0.004	0.016	1.981E-06	T Cells
ZNF720	1.4018E-10	1.10251357	0.005	0.017	2.1547E-06	T Cells
SLC4A73	1.4531E-10	3.33939863	0.177	0.169	2.2336E-06	T Cells
SH3D211	1.5249E-10	0.97074108	0.005	0.016	2.344E-06	T Cells
MT1X	1.5284E-10	0.70253222	0.021	0.039	2.3493E-06	T Cells
FBXL82	1.5759E-10	3.79146174	0.071	0.054	2.4222E-06	T Cells
C11orf95	1.763E-10	1.1960257	0.005	0.017	2.7098E-06	T Cells
ZNF433	1.8999E-10	0.44505972	0.002	0.011	2.9203E-06	T Cells
LRRC241	2.102E-10	1.3353263	0.004	0.015	3.231E-06	T Cells
ANKRD551	2.1066E-10	1.20926498	0.005	0.017	3.2381E-06	T Cells
DCHS14	2.2316E-10	1.29510216	0.004	0.014	3.4301E-06	T Cells
S100PBP1	2.2731E-10	2.42219405	0.239	0.352	3.494E-06	T Cells
MTSS21	2.2767E-10	0.49738912	0.004	0.015	3.4995E-06	T Cells
EGR34	2.465E-10	3.39714041	0.011	0.005	3.7889E-06	T Cells
ARAP21	2.4896E-10	2.69476246	0.157	0.223	3.8268E-06	T Cells

DDR13	3.0159E-10	1.07194382	0.008	0.02	4.6358E-06	T Cells
CRYGS1	3.4222E-10	1.85842366	0.016	0.031	5.2602E-06	T Cells
PDCD4	3.5168E-10	1.44390987	0.469	0.851	5.4057E-06	T Cells
C16orf46	3.7417E-10	1.54934784	0.008	0.021	5.7513E-06	T Cells
ERCC6L	3.8529E-10	1.46015185	0.003	0.012	5.9222E-06	T Cells
GAN1	3.8971E-10	2.27887311	0.019	0.036	5.9902E-06	T Cells
TNFRSF10A2	3.9752E-10	2.78434637	0.089	0.128	6.1102E-06	T Cells
RAB40B	4.4561E-10	0.76847652	0.002	0.012	6.8495E-06	T Cells
RBSN1	4.6576E-10	1.38466969	0.005	0.015	7.1592E-06	T Cells
UFSP11	4.7634E-10	1.0536865	0.004	0.014	7.3219E-06	T Cells
ULBP21	4.835E-10	0.26632026	0.002	0.01	7.4319E-06	T Cells
DHRS132	5.0316E-10	2.68398162	0.045	0.07	7.734E-06	T Cells
GRK52	5.0454E-10	2.59651836	0.061	0.09	7.7553E-06	T Cells
TBC1D19	5.0944E-10	2.50125106	0.06	0.089	7.8306E-06	T Cells
DEPDC41	5.1067E-10	0.23209224	0.002	0.012	7.8496E-06	T Cells
NDFIP2	5.1456E-10	2.66859431	0.103	0.147	7.9093E-06	T Cells
HOXA51	5.3789E-10	0.21713259	0.001	0.01	8.2679E-06	T Cells
SKA2	5.4001E-10	2.16614128	0.009	0.022	8.3005E-06	T Cells
CLTCL11	5.7804E-10	1.05184711	0.003	0.013	8.885E-06	T Cells
MMP172	6.0345E-10	0.16287007	0.002	0.011	9.2756E-06	T Cells
CLCN2	6.1759E-10	1.40102296	0.006	0.017	9.493E-06	T Cells
HYAL3	6.2566E-10	1.11177172	0.005	0.015	9.617E-06	T Cells
KIF18A	6.4693E-10	1.30338281	0.006	0.017	9.9439E-06	T Cells
MYO102	7.1815E-10	0.14180935	0.002	0.01	1.1039E-05	T Cells
OTUD7B1	7.359E-10	4.09175567	0.029	0.018	1.1312E-05	T Cells
PJVK	7.6152E-10	1.59371624	0.006	0.017	1.1705E-05	T Cells
PRR5	7.76E-10	4.01655508	0.033	0.021	1.1928E-05	T Cells
PCBP31	9.2291E-10	0.99642981	0.004	0.014	1.4186E-05	T Cells
ARHGEF19	9.412E-10	1.77739731	0.012	0.026	1.4467E-05	T Cells
GRASP4	1.0033E-09	1.03747995	0.01	0.023	1.5422E-05	T Cells
GDPD11	1.048E-09	1.55519303	0.007	0.018	1.6109E-05	T Cells
FAM222A1	1.1143E-09	1.43865652	0.005	0.015	1.7128E-05	T Cells
RIMKLA1	1.1347E-09	0.34542768	0.003	0.012	1.7442E-05	T Cells
MYLK4	1.1661E-09	0.90176139	0.003	0.013	1.7923E-05	T Cells
DNHD12	1.1793E-09	2.48061952	0.056	0.083	1.8127E-05	T Cells
DOCK31	1.1847E-09	1.66188929	0.005	0.015	1.821E-05	T Cells
ZNF7921	1.1855E-09	2.42871523	0.028	0.047	1.8222E-05	T Cells
CKLF2	1.2236E-09	1.71931895	0.438	0.761	1.8808E-05	T Cells
ECM11	1.2372E-09	0.74885982	0.004	0.014	1.9017E-05	T Cells
PGBD4	1.4677E-09	2.58047886	0.018	0.033	2.2561E-05	T Cells
TAP15	1.4825E-09	1.59410085	0.433	0.741	2.2787E-05	T Cells
SACS	1.5101E-09	2.78667387	0.136	0.192	2.3212E-05	T Cells
SLC6A16	1.5223E-09	1.24978661	0.004	0.013	2.34E-05	T Cells
STN1	1.7672E-09	2.76980173	0.155	0.219	2.7164E-05	T Cells
MYO5B	1.9989E-09	0.24148189	0.002	0.011	3.0725E-05	T Cells
KCTD14	2.0923E-09	1.03116797	0.003	0.013	3.216E-05	T Cells
CASS44	2.4226E-09	3.58948154	0.047	0.033	3.7238E-05	T Cells
GDPD52	2.4565E-09	3.76755733	0.042	0.029	3.7759E-05	T Cells
IZUMO42	2.47E-09	3.95922573	0.016	0.008	3.7966E-05	T Cells
CROCC1	2.6444E-09	2.3992055	0.047	0.072	4.0647E-05	T Cells
SFI11	2.7878E-09	2.66181785	0.153	0.215	4.2852E-05	T Cells
PRDM111	2.8397E-09	2.18150369	0.018	0.034	4.3649E-05	T Cells

GRK23	3.0865E-09	0.76524584	0.518	0.956	4.7443E-05	T Cells
ZFP371	3.2569E-09	1.49970238	0.005	0.015	5.0062E-05	T Cells
RAB27A	3.3993E-09	2.64927141	0.385	0.509	5.2251E-05	T Cells
MC1R1	3.6101E-09	1.49919289	0.005	0.014	5.5491E-05	T Cells
TRPV13	3.6165E-09	2.08648931	0.023	0.04	5.5589E-05	T Cells
SLC35E42	3.6548E-09	2.20129143	0.019	0.035	5.6178E-05	T Cells
WSCD21	4.0765E-09	0.19055661	0.002	0.01	6.266E-05	T Cells
HIVEP22	4.1112E-09	3.5314509	0.113	0.097	6.3193E-05	T Cells
TAGAP4	4.1906E-09	2.28834303	0.419	0.579	6.4413E-05	T Cells
SMIM31	4.2416E-09	2.04691041	0.009	0.021	6.5197E-05	T Cells
E2F71	4.7315E-09	0.92580284	0.002	0.01	7.2727E-05	T Cells
CCDC1462	5.32E-09	2.12288461	0.022	0.038	8.1773E-05	T Cells
TMPRSS131	6.6636E-09	1.32542683	0.005	0.014	0.00010243	T Cells
HS3ST12	6.9885E-09	1.18624685	0.004	0.014	0.00010742	T Cells
MAPK8IP11	7.1879E-09	0.85384317	0.004	0.013	0.00011048	T Cells
TSSK6	7.4825E-09	1.55368897	0.005	0.014	0.00011501	T Cells
HIST3H2A3	8.029E-09	3.92237619	0.031	0.02	0.00012341	T Cells
ZNF233	8.3971E-09	1.0154568	0.003	0.012	0.00012907	T Cells
E2F52	8.7852E-09	1.25539214	0.005	0.015	0.00013504	T Cells
YY2	9.0464E-09	0.4332993	0.002	0.01	0.00013905	T Cells
ZBP11	1.0529E-08	2.38643904	0.131	0.182	0.00016184	T Cells
TAF3	1.0861E-08	0.98691392	0.003	0.012	0.00016694	T Cells
SLC18B13	1.1708E-08	2.43381072	0.026	0.043	0.00017997	T Cells
DCAF4L1	1.2427E-08	1.0741968	0.004	0.013	0.00019101	T Cells
HOXB2	1.2443E-08	2.69021836	0.156	0.216	0.00019125	T Cells
SYCE1	1.4941E-08	0.41512954	0.003	0.011	0.00022965	T Cells
ZNF4675	1.61E-08	0.63610547	0.003	0.012	0.00024748	T Cells
ATP5MGL	1.6595E-08	1.61503753	0.007	0.018	0.00025508	T Cells
ANKRD35	1.6599E-08	1.47597643	0.004	0.013	0.00025514	T Cells
CBFB1	1.6888E-08	1.66767109	0.458	0.812	0.00025958	T Cells
RABGAP1L2	1.7304E-08	2.20276116	0.461	0.684	0.00026599	T Cells
ULBP3	1.7332E-08	1.22695202	0.004	0.012	0.00026641	T Cells
CCDC28B	2.0558E-08	1.61060943	0.005	0.015	0.00031599	T Cells
BEX5	2.1534E-08	2.02954597	0.013	0.025	0.00033099	T Cells
PAQR63	2.1556E-08	1.64079341	0.011	0.023	0.00033133	T Cells
PFKFB11	2.1892E-08	0.48278534	0.002	0.01	0.0003365	T Cells
F51	2.2041E-08	2.06521245	0.021	0.036	0.00033879	T Cells
TAF73	2.2233E-08	2.25100899	0.32	0.495	0.00034174	T Cells
C5orf301	2.244E-08	2.10815114	0.013	0.026	0.00034492	T Cells
LDLR	2.2534E-08	2.24134997	0.24	0.252	0.00034637	T Cells
CCDC157	2.4398E-08	1.52318218	0.006	0.015	0.00037503	T Cells
NASP	2.4431E-08	0.90731939	0.003	0.011	0.00037552	T Cells
ZNF415	2.7583E-08	1.1717023	0.004	0.012	0.00042398	T Cells
CCNL24	2.7637E-08	2.2327582	0.324	0.501	0.0004248	T Cells
SVIP1	2.8578E-08	3.99914816	0.03	0.02	0.00043927	T Cells
SLC25A342	2.8957E-08	0.72495124	0.003	0.011	0.0004451	T Cells
GNMT2	3.3077E-08	1.72098382	0.009	0.02	0.00050843	T Cells
TNIP31	4.3441E-08	1.87908593	0.024	0.04	0.00066773	T Cells
C19orf73	4.4116E-08	1.40556276	0.004	0.013	0.00067811	T Cells
CCDC1543	4.5783E-08	1.39081406	0.007	0.017	0.00070373	T Cells
COL6A21	4.5904E-08	0.8466245	0.015	0.027	0.00070559	T Cells
MTCP11	4.643E-08	2.06727425	0.011	0.022	0.00071367	T Cells

PCSK41	4.7292E-08	0.99419091	0.002	0.01	0.00072693	T Cells
HOXA41	5.0358E-08	0.67154453	0.002	0.01	0.00077406	T Cells
EMILIN13	5.2553E-08	0.35000736	0.002	0.01	0.00080779	T Cells
PLGLB21	6.203E-08	2.44559625	0.035	0.053	0.00095346	T Cells
C12orf762	6.8459E-08	1.75572073	0.006	0.015	0.00105229	T Cells
CD24	7.1749E-08	0.50262916	0.004	0.012	0.00110285	T Cells
CERK3	7.5033E-08	3.16825101	0.214	0.223	0.00115333	T Cells
ZNF48	7.706E-08	2.35527545	0.021	0.035	0.00118449	T Cells
DCAF161	8.1213E-08	2.7428977	0.153	0.211	0.00124833	T Cells
TMEM811	8.3425E-08	2.29263372	0.015	0.027	0.00128233	T Cells
LSMEM11	8.566E-08	0.50242539	0.003	0.01	0.00131668	T Cells
TTLL101	9.3758E-08	1.93629892	0.009	0.02	0.00144116	T Cells
BRICD51	1.1635E-07	1.34898114	0.004	0.012	0.00178848	T Cells
CBFA2T35	1.1785E-07	1.20230683	0.006	0.015	0.0018115	T Cells
USP511	1.1844E-07	1.4246769	0.004	0.012	0.00182049	T Cells
GABBR15	1.1922E-07	3.04772495	0.028	0.018	0.00183254	T Cells
LRRC37A32	1.2394E-07	1.41922288	0.003	0.01	0.00190513	T Cells
LY6G5C	1.3604E-07	1.63845165	0.005	0.014	0.00209114	T Cells
JADE1	1.4237E-07	3.34585731	0.131	0.12	0.0021884	T Cells
N4BP23	1.4259E-07	2.70880986	0.152	0.208	0.00219168	T Cells
FBXO36	1.5237E-07	1.53234464	0.005	0.014	0.00234202	T Cells
CYLD2	1.6273E-07	2.5807565	0.424	0.602	0.00250127	T Cells
WNT2B2	1.6338E-07	1.59999984	0.004	0.013	0.00251131	T Cells
SPIN2B	1.6541E-07	1.96392316	0.008	0.018	0.00254247	T Cells
UCN1	2.1479E-07	1.50607734	0.009	0.019	0.0033015	T Cells
FAM161A1	2.228E-07	1.78978428	0.006	0.015	0.00342469	T Cells
KDM5B	2.2343E-07	2.67002305	0.167	0.23	0.0034344	T Cells
ATP6V1FNB1	2.6067E-07	1.5607946	0.006	0.015	0.00400675	T Cells
ABTB22	2.7409E-07	0.6345322	0.003	0.01	0.00421311	T Cells
CCDC694	2.9117E-07	2.59345613	0.214	0.302	0.00447557	T Cells
NEIL31	2.9124E-07	1.79924907	0.007	0.016	0.00447664	T Cells
SAPCD2	2.9149E-07	1.23477488	0.003	0.01	0.00448048	T Cells
DDX43	2.9985E-07	1.0305266	0.003	0.011	0.00460906	T Cells
ZNF346	3.4296E-07	2.55122407	0.035	0.052	0.00527169	T Cells
ZNF101	3.6531E-07	3.45094427	0.166	0.162	0.00561522	T Cells
PDE4B3	3.6813E-07	2.29211662	0.362	0.462	0.00565852	T Cells
ANK11	4.5184E-07	1.8976086	0.009	0.018	0.00694524	T Cells
MSS51	5.6443E-07	2.32675333	0.018	0.031	0.00867585	T Cells
CCDC172	5.9221E-07	1.37960687	0.012	0.022	0.0091028	T Cells
EFNA31	5.992E-07	2.03304436	0.008	0.017	0.00921029	T Cells
ST6GALNAC33	6.0944E-07	1.38181345	0.005	0.013	0.00936767	T Cells
OAF2	6.1952E-07	0.58790966	0.003	0.01	0.00952269	T Cells
PPP1R3E	6.2858E-07	2.13302361	0.017	0.029	0.00966192	T Cells
CACNA1F	6.9053E-07	1.05093171	0.004	0.011	0.01061411	T Cells
MT-ND4L5	7.6244E-07	2.41058544	0.261	0.291	0.01171943	T Cells
TEX9	8.0973E-07	0.57346226	0.003	0.01	0.01244633	T Cells
PSIP1	8.1697E-07	2.83670185	0.11	0.148	0.01255764	T Cells
NAV2	8.391E-07	0.56625081	0.003	0.01	0.01289783	T Cells
NPAS2	9.3993E-07	1.4257794	0.003	0.01	0.01444773	T Cells
USP20	9.5237E-07	2.72890221	0.156	0.21	0.01463893	T Cells
DUSP141	9.9899E-07	3.87848939	0.02	0.012	0.01535552	T Cells
TVP23C1	1.0156E-06	1.87035758	0.007	0.016	0.01561023	T Cells

HIST1H2AC	1.0332E-06	1.91107697	0.005	0.012	0.01588137	T Cells
CCDC1801	1.1324E-06	2.0894852	0.008	0.017	0.01740631	T Cells
PLAAT4	1.1481E-06	2.26068393	0.35	0.546	0.0176478	T Cells
IPCEF11	1.2414E-06	2.86164647	0.119	0.16	0.01908105	T Cells
RIC3	1.2421E-06	2.36473054	0.022	0.036	0.01909198	T Cells
SORBS1	1.2808E-06	1.62345661	0.003	0.01	0.01968708	T Cells
FAM214A2	1.3526E-06	2.94365032	0.262	0.294	0.02079078	T Cells
OCLN2	1.4631E-06	2.0176776	0.01	0.019	0.02248912	T Cells
TREML22	1.5874E-06	2.35999878	0.011	0.021	0.02439969	T Cells
GLMN	1.6427E-06	2.72268693	0.078	0.106	0.02524969	T Cells
ANKRD36	1.7923E-06	2.5653683	0.031	0.046	0.02754976	T Cells
MELTF2	1.7962E-06	2.01169831	0.004	0.012	0.0276098	T Cells
C11orf651	1.8511E-06	1.23679183	0.004	0.012	0.02845355	T Cells
LLGL2	2.0218E-06	2.73016107	0.08	0.108	0.03107692	T Cells
UBE3D1	2.0801E-06	1.60360409	0.005	0.013	0.0319725	T Cells
AVIL4	2.3014E-06	1.55731844	0.007	0.015	0.03537474	T Cells
SPSB1	2.3274E-06	2.58837974	0.05	0.069	0.03577402	T Cells
FCHO13	2.4849E-06	2.72766375	0.097	0.13	0.03819525	T Cells
FAM107B	2.7157E-06	1.39351743	0.476	0.846	0.04174366	T Cells
ADM52	3.0924E-06	3.9795005	0.012	0.006	0.04753344	T Cells
SPEF21	3.1439E-06	3.3518857	0.044	0.034	0.0483255	T Cells
ICAM34	3.3087E-06	2.8935184	0.198	0.205	0.05085817	T Cells
C1orf56	3.4192E-06	2.11481583	0.004	0.01	0.05255663	T Cells
FAM131B	3.4659E-06	4.33273854	0.012	0.006	0.05327505	T Cells
PAQR73	3.5098E-06	2.13672763	0.005	0.012	0.05394909	T Cells
RFTN13	3.595E-06	2.770753	0.147	0.197	0.05525947	T Cells
ASIC3	3.7014E-06	1.32751529	0.005	0.013	0.0568944	T Cells
ZNF594	4.0879E-06	1.93060804	0.006	0.014	0.06283482	T Cells
DAB2IP	4.203E-06	2.30929102	0.005	0.011	0.06460414	T Cells
PIF1	4.6036E-06	1.28196113	0.005	0.011	0.07076246	T Cells
TSGA10	4.7867E-06	1.60503978	0.005	0.013	0.07357619	T Cells
STRBP2	5.1378E-06	2.9270392	0.092	0.122	0.07897332	T Cells
ADGRL13	5.2253E-06	2.11228856	0.011	0.02	0.08031854	T Cells
LRRC37B1	5.3711E-06	2.63069701	0.056	0.076	0.0825595	T Cells
FAM209B	5.5033E-06	1.33714013	0.004	0.011	0.08459095	T Cells
PKN3	6.0125E-06	2.10054448	0.009	0.017	0.09241748	T Cells
FAM83G1	6.5012E-06	2.54371634	0.018	0.029	0.0999295	T Cells
SEC61A21	6.5661E-06	2.49575616	0.013	0.022	0.10092737	T Cells
GOLGA7B	6.782E-06	3.46029575	0.067	0.055	0.10424642	T Cells
VILL1	7.7298E-06	2.57949056	0.043	0.06	0.11881441	T Cells
C12orf57	7.7433E-06	2.8800966	0.325	0.405	0.11902298	T Cells
CDH235	7.905E-06	0.93956216	0.004	0.01	0.12150835	T Cells
KLHL3	8.9858E-06	1.77281037	0.006	0.014	0.13812084	T Cells
ZNF257	9.1861E-06	1.99819685	0.005	0.012	0.14119967	T Cells
MYRF1	9.5943E-06	1.44399937	0.004	0.01	0.14747457	T Cells
CLEC18A1	9.7999E-06	1.4827577	0.005	0.012	0.15063452	T Cells
MEIS31	1.053E-05	1.64560843	0.004	0.011	0.16185642	T Cells
MAGEE1	1.0629E-05	4.31562459	0.015	0.009	0.16337951	T Cells
SLC4A51	1.0765E-05	4.10064588	0.01	0.005	0.16546399	T Cells
ARGLU14	1.0901E-05	2.33772518	0.419	0.595	0.1675613	T Cells
ZNF33B3	1.1789E-05	3.5530858	0.051	0.041	0.18120985	T Cells
OFD13	1.2121E-05	2.79909691	0.122	0.161	0.18630568	T Cells

LY6G5B3	1.2521E-05	2.52547439	0.076	0.1	0.19245748	T Cells
LANCL3	1.443E-05	1.9854031	0.005	0.012	0.22180118	T Cells
PRSS23	1.4974E-05	1.71168654	0.004	0.01	0.23016562	T Cells
SCART1	1.6134E-05	3.91062891	0.03	0.021	0.24799836	T Cells
ERP272	1.8806E-05	3.74800322	0.037	0.028	0.28906305	T Cells
ZSWIM43	2.5524E-05	2.46675919	0.024	0.035	0.39232365	T Cells
CDRT4	3.2015E-05	2.51669728	0.032	0.045	0.49210351	T Cells
SYCP2	3.3321E-05	3.70777935	0.019	0.012	0.51218435	T Cells
C11orf801	3.5441E-05	2.84983907	0.075	0.098	0.5447586	T Cells
CEP112	3.6096E-05	2.33496485	0.009	0.017	0.55483049	T Cells
RFX32	3.6462E-05	2.76012261	0.107	0.14	0.56045341	T Cells
ANKRD39	3.6488E-05	2.34810198	0.01	0.018	0.56086064	T Cells
RNF2271	3.7717E-05	2.06984667	0.008	0.015	0.57975249	T Cells
SLC29A21	3.8872E-05	2.13401302	0.013	0.022	0.59750594	T Cells
RAPGEF6	4.132E-05	3.08972588	0.239	0.268	0.63512532	T Cells
AKTIP	5.0939E-05	2.95205287	0.171	0.228	0.78298958	T Cells
CHD23	5.7682E-05	2.23776706	0.404	0.561	0.88663634	T Cells
KMT2A	6.0069E-05	2.88350565	0.314	0.39	0.92332036	T Cells
CCDC1341	6.3222E-05	3.09227185	0.057	0.076	0.97179297	T Cells
ELMO32	6.9377E-05	2.16646227	0.014	0.022	1	T Cells
RBM44	7.4556E-05	2.13620187	0.007	0.013	1	T Cells
ATXN7L21	7.5197E-05	2.13859188	0.006	0.012	1	T Cells
ZC3HAV1L	7.5644E-05	4.30413092	0.01	0.006	1	T Cells
CPT1B2	8.238E-05	2.45548227	0.057	0.075	1	T Cells
TMEM8B2	8.8112E-05	3.88709139	0.014	0.009	1	T Cells
EMP33	9.1447E-05	0.47862681	0.624	0.97	1	T Cells
TMEM652	9.8628E-05	2.89373325	0.112	0.144	1	T Cells
MAP3K143	0.00010357	3.24786495	0.05	0.041	1	T Cells
PM20D22	0.00010872	2.77732117	0.053	0.07	1	T Cells
ADAM201	0.00011109	2.20562246	0.006	0.012	1	T Cells
PLEKHG41	0.00012215	2.66413148	0.021	0.031	1	T Cells
SCGB1A1	0.00013915	1.66167791	0.015	0.023	1	T Cells
LRRC56	0.00014211	3.45081293	0.016	0.01	1	T Cells
DND13	0.00017008	1.94155485	0.008	0.014	1	T Cells
FOXD2	0.0001762	2.66263714	0.008	0.014	1	T Cells
PARP81	0.00017631	2.80086508	0.348	0.456	1	T Cells
KATNAL1	0.0001886	2.81041224	0.089	0.114	1	T Cells
EDN1	0.0001978	2.00397358	0.005	0.011	1	T Cells
TGFB3	0.0002188	2.44484047	0.006	0.012	1	T Cells
LCAT3	0.00021994	2.52206913	0.022	0.032	1	T Cells
SPTY2D1OS1	0.0002528	1.89017218	0.006	0.012	1	T Cells
SLC7A63	0.00025624	2.64705987	0.205	0.275	1	T Cells
SENP73	0.00026667	3.34492106	0.118	0.113	1	T Cells
SUN2	0.00027076	0.61573919	0.584	0.968	1	T Cells
SP41	0.00028769	2.90088762	0.151	0.197	1	T Cells
MT-ND25	0.0003049	2.84729471	0.2	0.215	1	T Cells
GLB1L2	0.000308	2.69869645	0.007	0.013	1	T Cells
CTRL1	0.00031568	2.36125452	0.028	0.039	1	T Cells
DYRK22	0.00033725	2.79680161	0.241	0.331	1	T Cells
SUMO42	0.00035103	3.59408697	0.018	0.013	1	T Cells
PANK11	0.00036843	2.31011867	0.006	0.012	1	T Cells
KCNJ141	0.00041887	2.17316441	0.007	0.013	1	T Cells

MT-CYB4	0.0004433	2.63503653	0.213	0.233	1	T Cells
ANKAR1	0.00045557	2.45054335	0.018	0.027	1	T Cells
NRN1L1	0.00045872	2.36387117	0.009	0.016	1	T Cells
LZTS3	0.00050554	2.52619822	0.009	0.016	1	T Cells
SAP253	0.00055655	3.13614995	0.105	0.099	1	T Cells
PARD6B	0.00055914	2.602423	0.016	0.024	1	T Cells
OTOF1	0.00056491	1.11262593	0.01	0.016	1	T Cells
VN1R12	0.0005684	2.36188843	0.005	0.01	1	T Cells
MUC12	0.00057539	2.10102574	0.012	0.008	1	T Cells
ODF2L	0.00062296	2.69979426	0.022	0.031	1	T Cells
UPK3B	0.00068371	1.39605361	0.005	0.01	1	T Cells
KCTD154	0.00072636	3.24037818	0.013	0.009	1	T Cells
KIF22	0.00074403	2.54573169	0.124	0.157	1	T Cells
OR5B21	0.00076523	2.09309007	0.007	0.013	1	T Cells
HIST1H2BG4	0.00076667	3.05696705	0.024	0.018	1	T Cells
ZC3HAV11	0.00077511	2.12369973	0.451	0.685	1	T Cells
IL10RA3	0.00083011	1.57285354	0.476	0.829	1	T Cells
HELLS	0.00083138	2.14745863	0.082	0.104	1	T Cells
UCP31	0.0008423	2.34267304	0.014	0.02	1	T Cells
PGBD11	0.00096891	2.4384564	0.007	0.013	1	T Cells
PILRB4	0.00107384	2.47971108	0.311	0.389	1	T Cells
LRFN12	0.00130746	2.83286877	0.027	0.036	1	T Cells
PHOSPHO21	0.00139355	2.89131591	0.024	0.032	1	T Cells
CKB2	0.00143343	2.03450501	0.006	0.01	1	T Cells
ITGB71	0.00143703	2.69574741	0.252	0.292	1	T Cells
BLK2	0.00159297	3.78711854	0.016	0.012	1	T Cells
SPATA24	0.00172796	2.69770912	0.015	0.022	1	T Cells
IQCN3	0.00193446	3.05814627	0.032	0.025	1	T Cells
EGFL81	0.00243487	1.95069992	0.006	0.01	1	T Cells
CDC42SE13	0.00250204	1.4081215	0.525	0.892	1	T Cells
NFATC32	0.00253725	2.47414363	0.344	0.516	1	T Cells
EHD32	0.00270592	2.38706543	0.007	0.012	1	T Cells
FMNL14	0.00273516	0.71368521	0.538	0.959	1	T Cells
BICD12	0.00273881	3.40829609	0.048	0.041	1	T Cells
NDC80	0.0027766	1.57655874	0.021	0.029	1	T Cells
MPZL31	0.00301325	3.38291706	0.065	0.059	1	T Cells
ZC3H83	0.00305364	2.91491535	0.055	0.069	1	T Cells
MYH3	0.00307938	2.27571305	0.007	0.011	1	T Cells
FUT75	0.00312671	3.33939235	0.027	0.021	1	T Cells
ECE14	0.00317507	2.82088801	0.119	0.117	1	T Cells
SLC39A141	0.00328623	2.75240086	0.013	0.019	1	T Cells
RDH5	0.00356684	2.37694834	0.01	0.015	1	T Cells
APOBEC3F	0.0036782	3.36404666	0.064	0.058	1	T Cells
ERMN3	0.0038191	3.45536803	0.016	0.012	1	T Cells
SLC25A151	0.00391305	2.91566471	0.029	0.038	1	T Cells
SUSD31	0.00421907	2.82743869	0.157	0.199	1	T Cells
ADCY43	0.00452516	2.39802132	0.019	0.025	1	T Cells
DIP2A2	0.004552	2.74494766	0.194	0.252	1	T Cells
KCNN44	0.00460148	2.86168148	0.071	0.065	1	T Cells
RSRP14	0.00492219	1.31730679	0.45	0.721	1	T Cells
CCDC1941	0.00498573	2.46982276	0.008	0.013	1	T Cells
TCTEX1D21	0.00543791	2.62645395	0.028	0.037	1	T Cells

BPIFB1	0.00553386	2.22141203	0.01	0.007	1	T Cells
FUT81	0.00565428	2.96979389	0.118	0.146	1	T Cells
SH3KBP14	0.00577382	2.58165127	0.289	0.409	1	T Cells
TMED62	0.00587174	2.50692854	0.007	0.011	1	T Cells
MDFIC	0.00589151	1.77930636	0.475	0.822	1	T Cells
PDP2	0.005993	2.91376963	0.055	0.067	1	T Cells
ADAM285	0.0060453	1.86786407	0.006	0.01	1	T Cells
TCTE31	0.0061286	2.36105948	0.008	0.012	1	T Cells
USP352	0.00616565	3.23848717	0.047	0.041	1	T Cells
MT-ND15	0.00626642	2.60403616	0.195	0.213	1	T Cells
TARSL21	0.00639995	3.46136666	0.063	0.058	1	T Cells
IL23A	0.0064212	2.43484313	0.011	0.016	1	T Cells
ANKDD1A3	0.00801845	2.59682252	0.021	0.028	1	T Cells
SIGLEC61	0.00817523	4.19954477	0.01	0.015	1	T Cells
CLK13	0.00827582	2.37504034	0.338	0.495	1	T Cells
KIAA08251	0.00838439	2.98003841	0.068	0.083	1	T Cells
MDGA1	0.00916469	3.76904678	0.017	0.013	1	T Cells
RAB3D2	0.00921192	2.17856763	0.01	0.015	1	T Cells
SAMD10	0.00947068	2.82498738	0.028	0.036	1	T Cells
ELF3	0	10.6445635	0.586	0.006	0	Epi
WFDC2	0	10.5602034	0.579	0.004	0	Epi
PIGR	0	10.9343696	0.574	0.003	0	Epi
SLPI	0	10.6891189	0.563	0.005	0	Epi
TSPAN1	0	12.6398798	0.551	0.001	0	Epi
MYH141	0	7.55510886	0.543	0.015	0	Epi
MUC14	0	9.50292154	0.518	0.007	0	Epi
CYP4B1	0	12.0276058	0.5	0.001	0	Epi
MUC4	0	13.4623091	0.487	0	0	Epi
DDR14	0	7.36458087	0.485	0.019	0	Epi
LCN2	0	11.9205359	0.452	0.002	0	Epi
TACSTD23	0	9.17652927	0.452	0.003	0	Epi
FXYD3	0	11.2801574	0.449	0.001	0	Epi
SLC44A4	0	13.0106453	0.444	0	0	Epi
SYNE21	0	2.98612748	0.467	0.034	0	Epi
CD241	0	8.90970237	0.442	0.011	0	Epi
EPS8L24	0	8.30477533	0.434	0.003	0	Epi
ATP2C2	0	13.1400233	0.429	0	0	Epi
MUC16	0	12.1933911	0.426	0.001	0	Epi
EHF2	0	7.42449447	0.437	0.017	0	Epi
BAIAP2L11	0	11.6418435	0.416	0	0	Epi
TRAF44	0	6.09096443	0.439	0.023	0	Epi
LMO72	0	7.2679522	0.429	0.014	0	Epi
CLU2	0	7.27091655	0.411	0.006	0	Epi
CELSR13	0	7.16378708	0.421	0.016	0	Epi
PLPP2	0	9.24731554	0.404	0.001	0	Epi
KRT19	0	11.2876763	0.401	0.001	0	Epi
AGR2	0	10.7063382	0.396	0.001	0	Epi
SELENBP1	0	11.9467352	0.393	0	0	Epi
CLDN4	0	12.732843	0.393	0	0	Epi
PCDH1	0	13.8310626	0.391	0	0	Epi
PERP1	0	6.10540943	0.398	0.009	0	Epi
MS4A8	0	12.3005523	0.388	0	0	Epi

TMC5	0	8.96927402	0.388	0.007	0	Epi
TPPP34	0	9.95534244	0.386	0.006	0	Epi
SMAD34	0	4.85598904	0.386	0.008	0	Epi
TC2N1	0	3.11224479	0.391	0.015	0	Epi
BPIFB11	0	10.513386	0.376	0.006	0	Epi
PLEKHA54	0	5.06922806	0.396	0.03	0	Epi
MTSS22	0	7.22784169	0.378	0.014	0	Epi
CGN	0	11.1701931	0.36	0.001	0	Epi
CKB3	0	7.88538834	0.363	0.009	0	Epi
ABCA13	0	12.7175917	0.353	0	0	Epi
PATJ2	0	4.52274853	0.36	0.008	0	Epi
TJP3	0	8.90289275	0.35	0.001	0	Epi
PFN2	0	9.4428927	0.35	0.002	0	Epi
PLEKHS1	0	11.9888764	0.348	0	0	Epi
CFAP157	0	13.3174388	0.348	0.001	0	Epi
DNAAF1	0	13.4731549	0.348	0.001	0	Epi
PROM1	0	11.5563561	0.345	0.001	0	Epi
RSPH1	0	11.4037437	0.345	0.002	0	Epi
PTPRF3	0	10.6977707	0.343	0.001	0	Epi
ADGRF1	0	13.2075383	0.34	0	0	Epi
C12orf741	0	11.1022626	0.34	0	0	Epi
FOXA1	0	12.2223257	0.338	0	0	Epi
MAPK151	0	8.52896777	0.355	0.018	0	Epi
CCDC78	0	8.88063943	0.345	0.008	0	Epi
LRRC46	0	8.99485678	0.348	0.012	0	Epi
GSTA1	0	12.5327346	0.335	0	0	Epi
EPCAM2	0	10.1778947	0.335	0.001	0	Epi
SCGB1A11	0	10.8106422	0.355	0.022	0	Epi
TACC2	0	12.9926914	0.332	0	0	Epi
CFAP731	0	13.3539971	0.332	0	0	Epi
TMC41	0	6.84862585	0.348	0.016	0	Epi
CDHR3	0	13.2304177	0.332	0.001	0	Epi
AC007906.2	0	8.63400475	0.345	0.014	0	Epi
DNAI1	0	13.015937	0.33	0	0	Epi
WDR66	0	9.3799567	0.335	0.005	0	Epi
CTXN1	0	9.97386523	0.332	0.004	0	Epi
CDHR4	0	12.2941134	0.327	0	0	Epi
KIAA1217	0	11.5767495	0.327	0	0	Epi
SCNN1A1	0	5.96382135	0.353	0.026	0	Epi
MYO5C2	0	8.96305758	0.327	0.001	0	Epi
CCDC1141	0	11.6639961	0.327	0.001	0	Epi
DSP	0	6.66237384	0.345	0.019	0	Epi
SYTL21	0	4.60334227	0.345	0.019	0	Epi
NEK102	0	10.2717026	0.327	0.002	0	Epi
CFAP100	0	13.4950553	0.325	0	0	Epi
ZBBX	0	12.4711668	0.325	0	0	Epi
CTTN2	0	6.53886357	0.338	0.013	0	Epi
TEKT2	0	11.2216129	0.325	0.001	0	Epi
DNAH121	0	12.8022594	0.325	0.001	0	Epi
CFAP43	0	10.4439565	0.327	0.003	0	Epi
FANK1	0	8.54209581	0.332	0.009	0	Epi
DNAH7	0	8.50884545	0.332	0.01	0	Epi

C6orf118	0	13.8536044	0.322	0	0	Epi
FAM92B	0	13.4082193	0.322	0	0	Epi
FOXJ1	0	13.4534855	0.322	0	0	Epi
ALOX152	0	11.9019468	0.322	0.001	0	Epi
ERBB21	0	6.45077202	0.338	0.017	0	Epi
CCDC187	0	14.5231207	0.32	0	0	Epi
DLEC11	0	7.83817657	0.327	0.01	0	Epi
ZMYND10	0	7.39102799	0.338	0.021	0	Epi
GRHL2	0	13.638938	0.317	0	0	Epi
VWA3A	0	12.2267991	0.317	0	0	Epi
DNAH9	0	14.1038055	0.317	0	0	Epi
CHST9	0	13.4027191	0.317	0	0	Epi
CFAP45	0	8.49473414	0.322	0.006	0	Epi
DTHD11	0	7.88217845	0.317	0.001	0	Epi
SAXO2	0	9.12899065	0.322	0.006	0	Epi
ERICH3	0	13.2023407	0.315	0	0	Epi
MAPK10	0	13.204369	0.315	0	0	Epi
PRR29	0	9.37626321	0.32	0.005	0	Epi
ANK33	0	5.36384058	0.327	0.015	0	Epi
CFAP70	0	8.02611962	0.32	0.008	0	Epi
DNAH10	0	11.6684123	0.312	0	0	Epi
CFAP54	0	10.6569039	0.31	0.001	0	Epi
TOGARAM22	0	8.2820507	0.315	0.007	0	Epi
DRC3	0	9.95202194	0.31	0.002	0	Epi
EFCAB1	0	9.33568466	0.312	0.005	0	Epi
BICDL2	0	11.7461331	0.307	0	0	Epi
CCDC176	0	7.11235052	0.327	0.021	0	Epi
C20orf85	0	12.1652759	0.307	0.001	0	Epi
ROPN1L	0	11.4539896	0.305	0	0	Epi
FRMPD2	0	13.7472097	0.305	0	0	Epi
CFAP46	0	9.82766293	0.307	0.003	0	Epi
KIF21A1	0	6.04534686	0.307	0.003	0	Epi
PIFO	0	9.35623732	0.31	0.007	0	Epi
SYNE11	0	6.781659	0.312	0.009	0	Epi
LDLRAD1	0	13.5100658	0.302	0	0	Epi
ARMC3	0	14.0828124	0.302	0	0	Epi
HYDIN	0	13.197124	0.302	0	0	Epi
MISP	0	13.4655067	0.302	0	0	Epi
SPAG8	0	7.00926468	0.322	0.023	0	Epi
KRT7	0	13.079925	0.299	0.001	0	Epi
CERS44	0	6.28075685	0.302	0.004	0	Epi
C6orf132	0	14.4965473	0.297	0	0	Epi
NWD1	0	13.0508398	0.297	0	0	Epi
TSPAN61	0	7.18407502	0.315	0.019	0	Epi
PSCA	0	9.52962797	0.299	0.005	0	Epi
PRSS22	0	11.3416503	0.294	0	0	Epi
DNAI2	0	10.9788629	0.294	0	0	Epi
FCGBP4	0	9.15865313	0.305	0.011	0	Epi
PLXNB1	0	8.41003439	0.294	0.001	0	Epi
CCDC190	0	14.4672182	0.292	0	0	Epi
TRIM29	0	11.7509765	0.292	0	0	Epi
MYO1D4	0	4.50881861	0.302	0.011	0	Epi

CFAP47	0	11.5595982	0.292	0.001	0	Epi
MDH1B	0	9.32143566	0.292	0.003	0	Epi
C11orf16	0	14.3660013	0.289	0	0	Epi
MYO5B1	0	6.93365186	0.299	0.01	0	Epi
PTPRT	0	14.0413158	0.289	0	0	Epi
CNN3	0	9.18511373	0.289	0.002	0	Epi
LRRC10B	0	13.1012911	0.287	0	0	Epi
CEACAM6	0	11.7354344	0.287	0	0	Epi
IQUB	0	9.46117985	0.287	0.002	0	Epi
RRAD2	0	9.68217718	0.287	0.002	0	Epi
SPAG17	0	13.249732	0.284	0	0	Epi
SLC34A2	0	12.4130101	0.284	0	0	Epi
SCGB3A1	0	11.285796	0.292	0.008	0	Epi
CFAP52	0	11.9789049	0.284	0	0	Epi
RHPN2	0	11.5096702	0.284	0	0	Epi
CXCL17	0	10.8229348	0.284	0	0	Epi
C9orf24	0	9.09693244	0.294	0.011	0	Epi
DEGS21	0	9.83106152	0.284	0.001	0	Epi
CRIP25	0	5.10378187	0.294	0.011	0	Epi
CP	0	10.9853211	0.282	0	0	Epi
CCDC60	0	13.3082823	0.282	0	0	Epi
PPL	0	11.7240963	0.282	0	0	Epi
FYB2	0	9.90723664	0.282	0.001	0	Epi
ADGRG12	0	6.34018054	0.282	0.001	0	Epi
DNAAF31	0	7.06785559	0.294	0.014	0	Epi
DRC1	0	11.7426225	0.279	0	0	Epi
TTC29	0	11.9856488	0.279	0	0	Epi
TUSC3	0	12.2828546	0.279	0	0	Epi
ERN2	0	13.5414616	0.279	0	0	Epi
HOOK12	0	8.13350878	0.279	0.001	0	Epi
TTLL102	0	6.68195143	0.297	0.019	0	Epi
PTPRU	0	7.0972528	0.287	0.009	0	Epi
ENAH	0	8.81277038	0.279	0.002	0	Epi
AGR3	0	12.4109385	0.277	0	0	Epi
MAL2	0	10.19684	0.277	0	0	Epi
EXPH51	0	8.55613875	0.279	0.002	0	Epi
LRRIQ1	0	14.1682465	0.277	0	0	Epi
TEKT1	0	13.0240197	0.277	0	0	Epi
LSR4	0	5.46029254	0.282	0.005	0	Epi
TFF3	0	9.54584746	0.277	0.001	0	Epi
TMEM232	0	7.82396637	0.284	0.008	0	Epi
DNAH5	0	9.97901196	0.277	0.002	0	Epi
SPATA18	0	7.13315913	0.294	0.019	0	Epi
TTLL91	0	8.124388	0.282	0.007	0	Epi
FAM183A	0	13.1508052	0.274	0	0	Epi
SNTN	0	12.4816178	0.274	0	0	Epi
EPB41L4B	0	12.0494746	0.274	0	0	Epi
DMKN	0	10.3246843	0.274	0	0	Epi
SH3BGRL2	0	10.9188549	0.274	0.001	0	Epi
PARVA1	0	8.10598766	0.277	0.004	0	Epi
AGBL2	0	8.23291871	0.277	0.004	0	Epi
FAM227A	0	8.45141519	0.277	0.004	0	Epi

PRSS231	0	7.69957484	0.282	0.009	0	Epi
AUTS23	0	4.75736265	0.277	0.005	0	Epi
ESRP2	0	12.0306489	0.272	0	0	Epi
CLIC6	0	11.2213546	0.272	0	0	Epi
RSPH4A	0	8.75789114	0.274	0.003	0	Epi
ERBB3	0	7.81449135	0.274	0.003	0	Epi
RSPH9	0	9.52324341	0.272	0.002	0	Epi
RAB36	0	9.82131605	0.272	0.002	0	Epi
TCTEX1D41	0	6.61967837	0.287	0.017	0	Epi
IGFBP5	0	13.9621069	0.269	0	0	Epi
C11orf88	0	13.322788	0.269	0	0	Epi
TSPAN19	0	13.2642019	0.269	0	0	Epi
GAS2L2	0	12.958443	0.269	0	0	Epi
TP73	0	8.17106957	0.272	0.004	0	Epi
EPPK11	0	8.10781932	0.269	0.001	0	Epi
LRRC561	0	6.77928709	0.277	0.01	0	Epi
RAB25	0	10.1238638	0.266	0	0	Epi
C3orf52	0	9.87696553	0.266	0	0	Epi
CAPSL	0	13.9436094	0.266	0	0	Epi
SDK12	0	10.8950638	0.266	0	0	Epi
MSMB	0	10.5503822	0.269	0.003	0	Epi
CCDC33	0	14.6596945	0.266	0	0	Epi
FAM83E	0	11.343319	0.266	0	0	Epi
MEIS11	0	8.67521348	0.266	0.001	0	Epi
SLC22A232	0	7.96935751	0.266	0.001	0	Epi
ANKRD45	0	11.9524777	0.264	0	0	Epi
DNAH111	0	12.7184144	0.264	0	0	Epi
CLDN3	0	12.2466541	0.264	0	0	Epi
ARMC4	0	12.0769264	0.264	0	0	Epi
STK33	0	11.6863846	0.264	0	0	Epi
OVOL2	0	14.1022141	0.264	0	0	Epi
TTC91	0	6.52608304	0.266	0.004	0	Epi
LRRC71	0	13.1952351	0.261	0	0	Epi
NDRG24	0	6.35259661	0.272	0.011	0	Epi
PDLIM42	0	7.33983833	0.266	0.006	0	Epi
DCDC2B	0	12.8620069	0.259	0	0	Epi
C1orf87	0	13.6067687	0.259	0	0	Epi
C5orf49	0	12.8289468	0.259	0	0	Epi
CFAP61	0	11.4056172	0.259	0.001	0	Epi
EFCAB61	0	6.27608239	0.274	0.017	0	Epi
INAVA	0	8.89362391	0.256	0	0	Epi
DAW1	0	10.69072	0.256	0	0	Epi
CFAP99	0	11.4455033	0.256	0	0	Epi
CCDC65	0	8.71206229	0.256	0	0	Epi
FAM216B	0	11.8691492	0.256	0	0	Epi
KIAA16711	0	5.36171696	0.264	0.008	0	Epi
PBX1	0	8.67448064	0.256	0.001	0	Epi
DNAH61	0	11.2661571	0.256	0.001	0	Epi
MORN31	0	6.5204728	0.261	0.006	0	Epi
CCN21	0	12.2243837	0.254	0	0	Epi
SCIN2	0	8.23237288	0.256	0.002	0	Epi
ESRP1	0	12.4237702	0.254	0	0	Epi

RIBC12	0	6.74700648	0.269	0.016	0	Epi
TRIM210	0	8.25333448	0.254	0.002	0	Epi
SMIM52	0	6.35745114	0.261	0.01	0	Epi
CFAP53	0	7.91490197	0.254	0.004	0	Epi
AKAP141	0	9.71194542	0.251	0.001	0	Epi
LYPD6B	0	12.4732766	0.249	0	0	Epi
ANKUB1	0	12.1582369	0.249	0	0	Epi
TMEM184A	0	14.1774564	0.249	0	0	Epi
WHRN3	0	5.60288271	0.254	0.005	0	Epi
TMPRSS2	0	13.1549469	0.249	0	0	Epi
EFCAB12	0	8.70452946	0.249	0.003	0	Epi
SOX2	0	12.6690639	0.246	0	0	Epi
WDR38	0	13.5140606	0.246	0	0	Epi
CFAP69	0	7.7284446	0.251	0.006	0	Epi
IQCD2	0	8.56046471	0.246	0.001	0	Epi
TSGA101	0	6.45263651	0.256	0.012	0	Epi
PTPRK2	0	10.472269	0.244	0	0	Epi
MORN5	0	12.7935884	0.244	0	0	Epi
TMPRSS4	0	10.8123049	0.244	0	0	Epi
EFCAB10	0	9.53241563	0.244	0.001	0	Epi
PLPPR3	0	9.61509966	0.244	0.002	0	Epi
ADGB1	0	11.923202	0.241	0	0	Epi
FAM166B	0	10.8498285	0.241	0	0	Epi
KIF19	0	12.3762638	0.241	0	0	Epi
ST6GALNAC1	0	9.73756548	0.241	0	0	Epi
ABLIM11	0	4.48983595	0.244	0.004	0	Epi
CFAP57	0	12.0880558	0.239	0	0	Epi
C1orf194	0	12.555328	0.239	0	0	Epi
EFEMP1	0	12.8488784	0.239	0	0	Epi
KIAA2012	0	13.7543407	0.239	0	0	Epi
CCDC39	0	11.8429284	0.239	0	0	Epi
PRSS12	0	10.4055828	0.239	0	0	Epi
C9orf135	0	13.5064847	0.239	0	0	Epi
DNAJB131	0	12.7415675	0.239	0	0	Epi
SIX1	0	12.7744541	0.239	0	0	Epi
DNAH3	0	14.0927749	0.239	0	0	Epi
ITGB4	0	13.3073065	0.239	0	0	Epi
WWC1	0	9.94977919	0.239	0.001	0	Epi
RSPH10B2	0	10.726417	0.239	0.001	0	Epi
EMP22	0	8.11369859	0.241	0.003	0	Epi
CMTM43	0	7.35689708	0.241	0.003	0	Epi
C10orf95	0	7.26114547	0.244	0.007	0	Epi
DRC71	0	7.37615233	0.246	0.009	0	Epi
WDR78	0	7.44038377	0.241	0.005	0	Epi
ARHGEF38	0	14.70051	0.236	0	0	Epi
C16orf71	0	7.73835653	0.241	0.005	0	Epi
ALDH3A1	0	7.98440223	0.244	0.008	0	Epi
CROCC2	0	10.7985922	0.236	0.001	0	Epi
CASC1	0	10.5461145	0.236	0.001	0	Epi
MYEF23	0	5.39791027	0.249	0.014	0	Epi
C22orf15	0	9.54525324	0.236	0.001	0	Epi
EFHC2	0	9.27613406	0.236	0.001	0	Epi

MAP3K19	0	14.3152169	0.234	0	0	Epi
IRX3	0	11.7491425	0.234	0	0	Epi
PACRG	0	11.0239658	0.234	0.001	0	Epi
MAATS1	0	8.3820909	0.236	0.003	0	Epi
HS3ST13	0	6.01089814	0.246	0.013	0	Epi
AK7	0	8.95986619	0.234	0.002	0	Epi
CHST6	0	11.6446557	0.231	0	0	Epi
BEST4	0	8.42870551	0.234	0.004	0	Epi
CFAP206	0	9.66448666	0.231	0.001	0	Epi
ZNF7033	0	7.19847764	0.234	0.004	0	Epi
LCA5	0	6.57395126	0.239	0.01	0	Epi
CDH26	0	6.75589044	0.236	0.007	0	Epi
CFAP74	0	13.3806063	0.228	0	0	Epi
KIAA13241	0	7.05789025	0.234	0.006	0	Epi
FAM81B	0	9.06570011	0.231	0.003	0	Epi
ECT2L	0	11.2110926	0.228	0	0	Epi
MUC5AC	0	9.6622873	0.234	0.006	0	Epi
CCDC81	0	8.6528503	0.231	0.003	0	Epi
NECTIN41	0	7.58113818	0.231	0.004	0	Epi
KNDC1	0	9.8396991	0.228	0.001	0	Epi
LAMA52	0	8.98147776	0.228	0.001	0	Epi
VWA3B	0	13.9972606	0.226	0	0	Epi
IQCH	0	7.0380759	0.231	0.005	0	Epi
MARCH10	0	12.2538409	0.226	0	0	Epi
LRRC74B	0	13.5324145	0.226	0	0	Epi
TEX91	0	6.75252268	0.236	0.01	0	Epi
MYO1B1	0	8.67025592	0.226	0.001	0	Epi
C4orf471	0	8.50476828	0.228	0.003	0	Epi
DOCK91	0	3.96911636	0.231	0.007	0	Epi
SEPTIN51	0	9.0884552	0.226	0.002	0	Epi
DYDC1	0	13.3534919	0.223	0	0	Epi
NCKAP1	0	7.62109515	0.223	0.001	0	Epi
LIMCH12	0	8.73804438	0.223	0.001	0	Epi
DYNLRB2	0	9.71665682	0.223	0.001	0	Epi
DUSP142	0	5.64004278	0.234	0.012	0	Epi
CYP4X1	0	11.8603042	0.221	0	0	Epi
FAM174B1	0	6.53967375	0.226	0.005	0	Epi
SPATA6L	0	8.03605056	0.221	0.002	0	Epi
NME5	0	13.5572204	0.218	0	0	Epi
NFIB	0	11.3590473	0.218	0	0	Epi
DCDC1	0	10.7703681	0.218	0	0	Epi
EVPL	0	9.95946633	0.218	0	0	Epi
CFAP58	0	7.14593226	0.223	0.006	0	Epi
DTX31	0	6.32725235	0.218	0.001	0	Epi
TMEM125	0	11.536566	0.216	0	0	Epi
C1orf116	0	10.3189925	0.216	0	0	Epi
GABRP	0	12.9840884	0.216	0	0	Epi
AK8	0	8.67594461	0.218	0.002	0	Epi
YAP1	0	12.0188501	0.216	0	0	Epi
ITGA21	0	7.29605599	0.216	0.001	0	Epi
NTN4	0	8.36882898	0.216	0.001	0	Epi
GIPR2	0	8.05117447	0.216	0.001	0	Epi

KLK11	0	10.4305233	0.216	0.001	0	Epi
TMEM190	0	10.0231252	0.216	0.001	0	Epi
TMEM45B1	0	8.00581338	0.216	0.002	0	Epi
MUC5B	0	12.0360681	0.216	0.002	0	Epi
LRATD1	0	12.5091322	0.213	0	0	Epi
RBPMS1	0	9.6553945	0.213	0	0	Epi
EYA1	0	13.799762	0.213	0	0	Epi
CFAP77	0	10.2117382	0.213	0.001	0	Epi
TTC25	0	6.4031346	0.223	0.011	0	Epi
NME9	0	11.1321445	0.211	0	0	Epi
SORBS2	0	11.4008212	0.211	0	0	Epi
NTN11	0	10.5152103	0.211	0	0	Epi
CNKSR11	0	8.03787322	0.211	0.002	0	Epi
LAMC2	0	12.5241557	0.208	0	0	Epi
SLC7A2	0	10.6076788	0.208	0	0	Epi
MTSS14	0	5.12161777	0.213	0.005	0	Epi
ENKUR	0	13.7118143	0.208	0	0	Epi
OSBPL61	0	9.54737305	0.208	0.001	0	Epi
PARD31	0	9.54650737	0.208	0.001	0	Epi
ECRG4	0	12.8659428	0.206	0	0	Epi
CCDC173	0	10.4064229	0.206	0	0	Epi
MAP62	0	10.4192905	0.206	0	0	Epi
CFAP300	0	13.3318864	0.206	0	0	Epi
ANKRD65	0	9.66374476	0.206	0.001	0	Epi
CERKL	0	8.38757051	0.206	0.002	0	Epi
ZNF474	0	7.51529177	0.208	0.004	0	Epi
VNN31	0	6.97034866	0.208	0.004	0	Epi
CFAP221	0	13.4889068	0.203	0	0	Epi
PCDH7	0	13.8344098	0.203	0	0	Epi
LMNTD1	0	13.145924	0.203	0	0	Epi
SIX4	0	12.4009977	0.203	0	0	Epi
PLCB41	0	11.4193951	0.203	0	0	Epi
NR2F2	0	10.3626735	0.201	0	0	Epi
TEAD1	0	6.74069484	0.208	0.007	0	Epi
C1orf189	0	7.66352809	0.203	0.003	0	Epi
FSTL1	0	8.71868261	0.201	0.001	0	Epi
IQANK1	0	8.51067823	0.203	0.003	0	Epi
ERICH51	0	7.78082333	0.203	0.004	0	Epi
LIPH	0	11.0741863	0.198	0	0	Epi
TCTE1	0	10.6103729	0.198	0	0	Epi
WNK2	0	11.5439147	0.198	0	0	Epi
SLC2A10	0	11.3352172	0.198	0	0	Epi
DYDC2	0	10.6478028	0.198	0.001	0	Epi
SRGAP13	0	6.74614437	0.201	0.004	0	Epi
MCF2L	0	7.50147736	0.198	0.001	0	Epi
SLC27A21	0	6.46674067	0.198	0.001	0	Epi
DMWD3	0	3.90193362	0.203	0.006	0	Epi
STOX1	0	7.17329859	0.201	0.005	0	Epi
IL5RA	0	8.77940912	0.195	0	0	Epi
ASB14	0	12.883222	0.195	0	0	Epi
TDRP	0	10.0219631	0.195	0.001	0	Epi
SH3D191	0	11.2619417	0.193	0	0	Epi

DMTN1	0	4.92696151	0.198	0.005	0	Epi
SHANK2	0	12.6286043	0.193	0	0	Epi
KRT80	0	11.854819	0.193	0	0	Epi
WFDC6	0	14.5782411	0.193	0	0	Epi
EYA21	0	6.82283585	0.198	0.005	0	Epi
RIBC2	0	7.53427562	0.198	0.005	0	Epi
CFAP126	0	10.608051	0.193	0.001	0	Epi
GALNT36	0	5.24605105	0.201	0.009	0	Epi
PLS1	0	6.39884891	0.198	0.007	0	Epi
TCTEX1D1	0	9.60177323	0.19	0.001	0	Epi
WDR631	0	8.951096	0.19	0.001	0	Epi
CCNO	0	10.4537586	0.19	0.001	0	Epi
GRIN3B1	0	10.5382783	0.19	0.001	0	Epi
SPATA17	0	7.25189642	0.193	0.005	0	Epi
RAB17	0	6.13735696	0.195	0.009	0	Epi
STEAP44	0	7.652546	0.188	0.002	0	Epi
PTPRN2	0	8.44405795	0.188	0.002	0	Epi
VSIG21	0	8.30749265	0.188	0.002	0	Epi
CD164L2	0	12.1284295	0.185	0	0	Epi
RIIAD1	0	12.801985	0.185	0	0	Epi
GRM7	0	13.9217215	0.185	0	0	Epi
INPP4B	0	7.23773564	0.185	0	0	Epi
TMEM30B	0	7.33142115	0.185	0	0	Epi
CYP2F1	0	12.1403865	0.185	0	0	Epi
VSTM2L	0	10.6221681	0.185	0	0	Epi
PPP1R324	0	6.65794098	0.19	0.006	0	Epi
BCO23	0	6.59254748	0.188	0.004	0	Epi
C1orf158	0	15.0970194	0.183	0	0	Epi
AGBL4	0	11.5545541	0.183	0	0	Epi
ARFGEF3	0	10.645772	0.183	0	0	Epi
LRRC4	0	7.89551114	0.185	0.002	0	Epi
GGT6	0	12.1801361	0.183	0	0	Epi
C19orf331	0	6.67042792	0.19	0.007	0	Epi
TMPRSS31	0	5.43047887	0.185	0.002	0	Epi
FZD6	0	7.77630492	0.183	0.001	0	Epi
SPEF1	0	10.0035367	0.183	0.001	0	Epi
NEBL	0	7.41601774	0.183	0.003	0	Epi
SH2D3A1	0	4.34772632	0.183	0.004	0	Epi
SRGAP33	0	5.3916941	0.185	0.007	0	Epi
FAM3D	0	11.1277877	0.178	0	0	Epi
TPST11	0	8.21953707	0.178	0	0	Epi
SLC37A34	0	4.02249009	0.183	0.005	0	Epi
LYPD2	0	12.6752679	0.178	0	0	Epi
CYP2J2	0	10.1587983	0.178	0.001	0	Epi
AKAP63	0	8.40993627	0.178	0.001	0	Epi
KCNK11	0	10.199035	0.175	0	0	Epi
FABP6	0	12.1418768	0.175	0	0	Epi
DDAH1	0	8.34348194	0.175	0.002	0	Epi
PLCH11	0	10.3010469	0.173	0	0	Epi
COL21A1	0	14.0668997	0.173	0	0	Epi
C8orf34	0	12.1775128	0.173	0	0	Epi
FGF14	0	11.4117483	0.173	0	0	Epi

C11orf52	0	8.61159383	0.173	0.001	0	Epi
MEIS2	0	7.61220552	0.175	0.003	0	Epi
MAP1A1	0	6.19371984	0.178	0.006	0	Epi
MAB21L4	0	11.6448209	0.17	0	0	Epi
EFHB	0	10.4977847	0.17	0	0	Epi
FOXP2	0	13.3214677	0.17	0	0	Epi
FGFR2	0	10.7383142	0.17	0	0	Epi
CFAP97D2	0	12.1869267	0.17	0	0	Epi
MB	0	10.332997	0.17	0	0	Epi
STMND1	0	10.9161563	0.17	0	0	Epi
FAM184A1	0	6.07444838	0.173	0.004	0	Epi
MECOM	0	11.7118764	0.168	0	0	Epi
TMEM212	0	12.409833	0.168	0	0	Epi
DZIP1	0	10.8688575	0.168	0	0	Epi
FUT6	0	12.0774571	0.168	0	0	Epi
SLC6A14	0	12.1737772	0.168	0	0	Epi
METTL271	0	8.89569342	0.168	0.001	0	Epi
HIP1R1	0	5.47636268	0.168	0.002	0	Epi
KCNRG	0	6.48671136	0.17	0.004	0	Epi
NBL12	0	3.72327668	0.17	0.005	0	Epi
CAPN9	0	10.858329	0.165	0	0	Epi
COBL	0	10.1117804	0.165	0	0	Epi
ANKRD18A	0	8.04294008	0.165	0	0	Epi
TSPAN8	0	10.6175129	0.165	0	0	Epi
TSNAXIP1	0	7.60147183	0.168	0.003	0	Epi
EPPIN	0	12.5577507	0.165	0	0	Epi
C7orf57	0	7.30659408	0.168	0.004	0	Epi
ASAP24	0	6.96683271	0.165	0.002	0	Epi
GLI3	0	6.77855274	0.168	0.005	0	Epi
FBXL13	0	5.93791232	0.17	0.007	0	Epi
MPDZ	0	7.9711476	0.165	0.002	0	Epi
FBXO15	0	6.42556152	0.17	0.007	0	Epi
ALS2CL	0	9.26239681	0.162	0	0	Epi
TTC23L	0	11.2359026	0.162	0	0	Epi
ASS14	0	6.16471193	0.165	0.003	0	Epi
MUC15	0	11.9995815	0.162	0	0	Epi
C12orf753	0	3.89068487	0.168	0.006	0	Epi
ALDH1A3	0	11.8188393	0.162	0	0	Epi
SCNN1B	0	12.4388967	0.162	0	0	Epi
ALPL1	0	7.6985369	0.162	0.001	0	Epi
CCND13	0	6.03461633	0.165	0.004	0	Epi
SH3RF14	0	5.55889713	0.165	0.006	0	Epi
ANKDD1B1	0	7.16679554	0.162	0.003	0	Epi
SMAGP2	0	7.4758451	0.16	0.001	0	Epi
CFAP161	0	7.49966403	0.162	0.003	0	Epi
SEMA3F	0	11.0521472	0.157	0	0	Epi
GSTA2	0	11.096313	0.157	0	0	Epi
DUOXA1	0	10.897295	0.157	0	0	Epi
USP431	0	7.01182448	0.16	0.004	0	Epi
BNIPL	0	6.85399758	0.157	0.002	0	Epi
AQP5	0	12.0532543	0.155	0	0	Epi
MARVELD31	0	10.4575256	0.155	0	0	Epi

DEFB124	0	10.2905952	0.155	0	0	Epi
TTC222	0	5.21375707	0.157	0.003	0	Epi
TSHZ21	0	6.08897731	0.155	0.001	0	Epi
SEMA4C4	0	4.86504763	0.155	0.002	0	Epi
NCALD3	0	5.03487789	0.155	0.002	0	Epi
PITPNM3	0	6.62798525	0.157	0.004	0	Epi
SLC25A231	0	6.18462708	0.157	0.004	0	Epi
C2orf50	0	11.0131456	0.152	0	0	Epi
RSPH14	0	9.90561709	0.152	0	0	Epi
TTC39A1	0	8.59585493	0.152	0.001	0	Epi
HHLA2	0	9.87123325	0.15	0	0	Epi
PTK71	0	9.27944467	0.15	0	0	Epi
EGFR	0	10.3084597	0.15	0	0	Epi
SDR16C5	0	13.228785	0.15	0	0	Epi
PAWR2	0	7.84744268	0.15	0	0	Epi
TANC1	0	6.36367064	0.152	0.004	0	Epi
GRAMD1C1	0	8.38303018	0.147	0	0	Epi
PPP1R9A	0	7.62460533	0.147	0	0	Epi
C10orf67	0	10.1106082	0.147	0	0	Epi
GPRC5A	0	12.0880822	0.147	0	0	Epi
ATP12A	0	11.9433377	0.147	0	0	Epi
S100A14	0	9.17353253	0.147	0.001	0	Epi
CCDC891	0	8.0309266	0.147	0.001	0	Epi
SDC1	0	7.84566437	0.147	0.002	0	Epi
BMPR1B	0	10.5704972	0.145	0	0	Epi
SLC23A11	0	9.55754506	0.145	0	0	Epi
CNGA4	0	7.92824983	0.147	0.002	0	Epi
FUT3	0	11.8276146	0.145	0	0	Epi
MBOAT24	0	5.18118133	0.147	0.003	0	Epi
SLC4A4	0	9.06915986	0.145	0.001	0	Epi
ENO4	0	7.23447009	0.147	0.003	0	Epi
SOX52	0	8.44809214	0.145	0.001	0	Epi
CRYM1	0	9.37573658	0.145	0.001	0	Epi
PRSS8	0	9.22694353	0.145	0.001	0	Epi
HIST3H2BB2	0	5.51430855	0.147	0.004	0	Epi
CCDC80	0	10.8318174	0.142	0	0	Epi
ADGRV1	0	9.85172444	0.142	0	0	Epi
PAX9	0	10.5714775	0.142	0	0	Epi
WDR93	0	7.66457134	0.145	0.003	0	Epi
KLHDC7A	0	9.58621659	0.142	0	0	Epi
SGPP22	0	6.18573368	0.142	0.001	0	Epi
MARVELD21	0	6.91335824	0.145	0.004	0	Epi
C4orf19	0	11.04631	0.14	0	0	Epi
PITX1	0	12.0116923	0.14	0	0	Epi
TUB	0	8.65067201	0.14	0	0	Epi
SPTBN2	0	11.894982	0.14	0	0	Epi
BTG4	0	10.2462024	0.14	0	0	Epi
SERPINB3	0	12.4146515	0.14	0	0	Epi
B3GNT3	0	11.3787123	0.14	0	0	Epi
FAM83F	0	9.88490632	0.14	0	0	Epi
SETBP13	0	3.96468332	0.145	0.005	0	Epi
FAM149A3	0	7.11964557	0.14	0.001	0	Epi

LRRC73	0	9.3171847	0.14	0.001	0	Epi
KRT23	0	8.53429107	0.14	0.001	0	Epi
MYCBPAP	0	7.94999811	0.14	0.001	0	Epi
PHYHD11	0	7.18582013	0.142	0.003	0	Epi
ARHGEF26	0	6.46894603	0.142	0.004	0	Epi
INHBB	0	9.90813572	0.137	0	0	Epi
CCDC148	0	10.8415164	0.137	0	0	Epi
ADH1C	0	11.9274136	0.137	0	0	Epi
IL20RA	0	13.6723132	0.137	0	0	Epi
C11orf97	0	12.6421956	0.137	0	0	Epi
PLA2G10	0	13.2406336	0.137	0	0	Epi
ANKFN1	0	10.9466355	0.137	0	0	Epi
CX3CL1	0	13.5546737	0.137	0	0	Epi
ANKRD66	0	12.5650696	0.137	0	0	Epi
C2orf73	0	12.1582119	0.135	0	0	Epi
TNFRSF19	0	10.2177123	0.135	0	0	Epi
TBX1	0	10.1837525	0.135	0	0	Epi
CAP2	0	9.38891167	0.135	0.001	0	Epi
FER1L6	0	7.59833822	0.137	0.004	0	Epi
CAPN8	0	11.1587389	0.132	0	0	Epi
CLDN17	0	5.35821261	0.135	0.003	0	Epi
COL28A1	0	10.3347505	0.132	0	0	Epi
PZP	0	14.3639981	0.132	0	0	Epi
BAIAP3	0	9.14570492	0.132	0	0	Epi
WNT7B	0	13.4508035	0.132	0	0	Epi
LRRC43	0	9.49997696	0.132	0.001	0	Epi
DMPK2	0	4.79953954	0.132	0.002	0	Epi
FAAH21	0	5.80870884	0.132	0.002	0	Epi
KIAA15491	0	8.29861484	0.129	0	0	Epi
RASEF	0	12.1832448	0.129	0	0	Epi
TTC16	0	7.33502495	0.129	0	0	Epi
VWA21	0	9.56500302	0.129	0	0	Epi
TTLL6	0	12.6545181	0.129	0	0	Epi
MYH10	0	7.96173311	0.129	0.001	0	Epi
TEKT3	0	10.2267584	0.127	0	0	Epi
WNT9A2	0	6.05135342	0.127	0.001	0	Epi
OXTR	0	8.97038826	0.127	0.001	0	Epi
BCL7A2	0	7.30341983	0.127	0.001	0	Epi
PLIN51	0	6.56495151	0.127	0.001	0	Epi
KPNA7	0	11.4126146	0.124	0	0	Epi
MAT1A	0	10.7037747	0.124	0	0	Epi
RASSF9	0	11.9052089	0.124	0	0	Epi
PPP1R42	0	13.6010968	0.124	0	0	Epi
SLC6A92	0	7.62938135	0.124	0.001	0	Epi
KIAA0319	0	8.41983291	0.124	0.001	0	Epi
PKP3	0	10.9495247	0.122	0	0	Epi
PAMR1	0	11.0377914	0.122	0	0	Epi
STOML3	0	12.0281189	0.122	0	0	Epi
APCDD12	0	9.58965555	0.122	0	0	Epi
FZD32	0	7.22175547	0.122	0.001	0	Epi
IRX5	0	7.66752378	0.122	0.001	0	Epi
TMEM54	0	6.74393123	0.122	0.003	0	Epi

C1orf141	0	11.6057582	0.119	0	0	Epi
EFNA1	0	9.91462528	0.119	0	0	Epi
LNX1	0	12.0964554	0.119	0	0	Epi
SCARA3	0	12.7954373	0.119	0	0	Epi
BDKRB21	0	12.7494983	0.119	0	0	Epi
LRRC361	0	11.2803911	0.119	0	0	Epi
CAPN13	0	10.9953217	0.119	0	0	Epi
SEC14L3	0	13.1821275	0.119	0	0	Epi
ACKR4	0	9.05803638	0.119	0.001	0	Epi
LRIG3	0	8.18497699	0.119	0.001	0	Epi
RHBDL2	0	13.6545841	0.117	0	0	Epi
IL33	0	11.3999761	0.117	0	0	Epi
PTPRS1	0	5.60098938	0.119	0.002	0	Epi
RAI141	0	7.69933238	0.117	0.001	0	Epi
KRT4	0	11.502032	0.117	0.001	0	Epi
HSPG22	0	6.54452602	0.117	0.002	0	Epi
RORC2	0	4.5097898	0.117	0.002	0	Epi
TRNP1	0	9.08605422	0.114	0	0	Epi
ATP6V1C2	0	11.4026679	0.114	0	0	Epi
MAGI1	0	6.64499339	0.117	0.003	0	Epi
AMOTL2	0	11.8989541	0.114	0	0	Epi
CXCL6	0	10.9274346	0.114	0	0	Epi
FAM160A12	0	8.53135203	0.114	0	0	Epi
MAP1B	0	14.3080054	0.114	0	0	Epi
FILIP1	0	9.45908776	0.114	0	0	Epi
SPAG6	0	9.80173328	0.114	0	0	Epi
PLEKHH1	0	6.66021697	0.117	0.003	0	Epi
SPNS2	0	12.2730424	0.114	0	0	Epi
PIH1D3	0	12.6200708	0.114	0	0	Epi
MROH9	0	13.1416188	0.114	0	0	Epi
EYA41	0	11.8933852	0.114	0	0	Epi
PRR15L	0	12.4424944	0.114	0	0	Epi
NCS13	0	5.46815194	0.114	0.002	0	Epi
TTC6	0	13.4839728	0.112	0	0	Epi
EML11	0	8.84597639	0.112	0	0	Epi
RHOV	0	10.8203483	0.112	0	0	Epi
C16orf89	0	10.7935465	0.112	0	0	Epi
SERPINB111	0	13.0451988	0.112	0	0	Epi
CEACAM5	0	12.6081733	0.112	0	0	Epi
BCAS42	0	4.28457137	0.114	0.002	0	Epi
GP1BB	0	10.3362501	0.112	0	0	Epi
B4GALNT3	0	7.86893925	0.112	0.001	0	Epi
ANKK1	0	6.36035235	0.112	0.002	0	Epi
BPIFA1	0	12.7208912	0.112	0.002	0	Epi
SIDT11	0	3.50245576	0.112	0.003	0	Epi
CREB3L1	0	10.4570961	0.109	0	0	Epi
DSG22	0	8.15360481	0.109	0	0	Epi
POF1B	0	11.0273726	0.109	0	0	Epi
TUBA4B	0	13.2582268	0.109	0	0	Epi
AC013470.2	0	12.5029814	0.109	0	0	Epi
CABCOCO1	0	12.4133739	0.107	0	0	Epi
KRT15	0	13.4893816	0.107	0	0	Epi

MET	0	11.6027954	0.107	0	0	Epi
GBP6	0	8.20113603	0.107	0.001	0	Epi
RAMP13	0	6.24213729	0.107	0.001	0	Epi
FAM81A1	0	7.95737931	0.107	0.001	0	Epi
TTLL7	0	6.57846757	0.107	0.002	0	Epi
NXN	0	7.58448039	0.107	0.002	0	Epi
ZNF4621	0	7.51684452	0.104	0	0	Epi
FER1L5	0	7.44715305	0.104	0.002	0	Epi
ZNF6082	0	8.16621122	0.102	0	0	Epi
ZNF750	0	12.8910964	0.102	0	0	Epi
NKX2-1	0	11.4339684	0.102	0	0	Epi
CCDC1811	0	10.9131324	0.102	0	0	Epi
SPATS1	0	12.2124647	0.102	0	0	Epi
EFNB21	0	7.56765539	0.102	0.001	0	Epi
PPP4R4	0	8.0640424	0.102	0.001	0	Epi
LRP2BP	0	7.24249656	0.102	0.002	0	Epi
MANSC1	0	6.71545276	0.102	0.002	0	Epi
CHL1	0	10.9906666	0.099	0	0	Epi
UGT2A1	0	10.9251312	0.099	0	0	Epi
SEMA3A	0	8.7157662	0.099	0	0	Epi
ATP6V0A4	0	11.8344342	0.099	0	0	Epi
RGS221	0	10.7191621	0.099	0	0	Epi
SCEL	0	13.3157082	0.099	0	0	Epi
TMEM98	0	13.0331664	0.099	0	0	Epi
MUC13	0	10.6089857	0.099	0	0	Epi
C14orf132	0	9.29736064	0.099	0	0	Epi
CMTM83	0	5.36992704	0.099	0.001	0	Epi
VSIG8	0	6.43921926	0.099	0.002	0	Epi
ADH7	0	11.9482326	0.096	0	0	Epi
SYBU	0	9.61109457	0.096	0	0	Epi
DEUP11	0	9.83451705	0.096	0	0	Epi
HID1	0	8.76310932	0.096	0	0	Epi
ADIRF	0	8.30138515	0.096	0.001	0	Epi
PDZK1IP1	0	11.5100698	0.094	0	0	Epi
APOBEC4	0	12.000899	0.094	0	0	Epi
OBSL1	0	7.99213497	0.096	0.002	0	Epi
SLC2A122	0	10.0736371	0.094	0	0	Epi
STYK11	0	6.05143518	0.094	0	0	Epi
PIEZO2	0	11.8920894	0.094	0	0	Epi
CBLC	0	13.0387122	0.094	0	0	Epi
SRPX2	0	7.18283926	0.096	0.002	0	Epi
TSPAN11	0	11.4543254	0.094	0	0	Epi
THSD4	0	11.4931536	0.094	0	0	Epi
PLAAT21	0	7.20510234	0.094	0.002	0	Epi
FXYD12	0	6.13952613	0.094	0.002	0	Epi
SIX2	0	10.8520908	0.091	0	0	Epi
WDR86	0	6.85604556	0.091	0	0	Epi
CHAD	0	10.8728557	0.091	0	0	Epi
COL4A5	0	13.4322171	0.091	0	0	Epi
HS3ST6	0	15.0501754	0.091	0	0	Epi
SPRR3	0	11.294331	0.091	0.001	0	Epi
C22orf23	0	8.36792268	0.091	0.001	0	Epi

MARK1	0	8.38297625	0.091	0.001	0	Epi
VWA1	0	9.54966837	0.089	0	0	Epi
CLCA2	0	10.4352986	0.089	0	0	Epi
FMO2	0	12.6008945	0.089	0	0	Epi
SH3RF32	0	8.44323279	0.089	0	0	Epi
GULP1	0	11.3031083	0.089	0	0	Epi
ADCY2	0	13.281356	0.089	0	0	Epi
KIF6	0	9.48279335	0.089	0	0	Epi
SLC5A8	0	12.0921083	0.089	0	0	Epi
BCAM	0	9.07350017	0.089	0	0	Epi
CCDC8	0	11.7161669	0.089	0	0	Epi
CDC42EP5	0	10.6151087	0.089	0	0	Epi
SLC13A31	0	9.00688	0.089	0	0	Epi
PTGFR	0	12.8438286	0.089	0	0	Epi
TEX26	0	10.8305625	0.089	0	0	Epi
EFNB3	0	12.1738395	0.089	0	0	Epi
SCD5	0	7.91453598	0.089	0.001	0	Epi
NOTCH3	0	8.30341533	0.089	0.001	0	Epi
CDON	0	6.57734911	0.089	0.002	0	Epi
ARHGEF4	0	8.33847189	0.086	0	0	Epi
RARB	0	11.1596865	0.086	0	0	Epi
RASSF10	0	13.4942024	0.086	0	0	Epi
KCNA1	0	13.5170808	0.086	0	0	Epi
LRP6	0	8.04354039	0.086	0	0	Epi
FA2H	0	10.3656033	0.086	0	0	Epi
CHST5	0	8.96172285	0.086	0	0	Epi
LTK2	0	5.61228531	0.086	0.001	0	Epi
PLIN41	0	5.78929593	0.086	0.001	0	Epi
BTC	0	10.8187184	0.084	0	0	Epi
ARHGEF37	0	10.7251431	0.084	0	0	Epi
ADRB1	0	7.20178118	0.084	0	0	Epi
CACNG6	0	13.2957062	0.084	0	0	Epi
PRICKLE2	0	10.4703723	0.084	0	0	Epi
PTPRZ1	0	10.6067825	0.084	0	0	Epi
TM4SF1	0	9.85928816	0.084	0.001	0	Epi
AMN2	0	6.87288915	0.084	0.001	0	Epi
KRT13	0	11.5064274	0.084	0.001	0	Epi
SLC9C2	0	9.43713643	0.081	0	0	Epi
ERICH2	0	10.7977666	0.081	0	0	Epi
TMEM40	0	11.2909441	0.081	0	0	Epi
LAYN	0	5.32576705	0.081	0	0	Epi
HSPB8	0	12.2966899	0.081	0	0	Epi
ACSBG1	0	10.9097127	0.081	0	0	Epi
SCNN1G	0	13.7182864	0.081	0	0	Epi
EPN3	0	14.4297326	0.081	0	0	Epi
SERPINB21	0	9.05888577	0.081	0	0	Epi
CLDN8	0	11.9389283	0.081	0	0	Epi
SAA4	0	8.72867288	0.081	0.001	0	Epi
CCDC102A3	0	5.11246166	0.081	0.001	0	Epi
PI31	0	5.87447192	0.081	0.001	0	Epi
SUSD4	0	8.66584748	0.079	0	0	Epi
ALDH1L1	0	12.5358565	0.079	0	0	Epi

AADAT	0	9.06985883	0.079	0	0	Epi
OSMR	0	10.0630279	0.079	0	0	Epi
SOX21	0	11.5142552	0.079	0	0	Epi
FNDC11	0	8.33037047	0.079	0	0	Epi
LRRC18	0	13.5337737	0.079	0	0	Epi
TNNT3	0	6.4208319	0.079	0.001	0	Epi
ARHGAP441	0	7.30785939	0.079	0.001	0	Epi
UPK1B	0	9.66310783	0.076	0	0	Epi
TEAD3	0	8.73211338	0.076	0	0	Epi
KLHL32	0	7.99193052	0.076	0	0	Epi
POU2AF11	0	7.42166644	0.076	0	0	Epi
SLC16A111	0	8.71553467	0.076	0	0	Epi
SSUH2	0	10.6791614	0.076	0	0	Epi
TFAP2A	0	7.71690068	0.076	0.001	0	Epi
PCLO	0	7.26132436	0.076	0.001	0	Epi
ASIC1	0	8.77225875	0.076	0.001	0	Epi
CCN1	0	13.5223336	0.074	0	0	Epi
OTX1	0	11.1139565	0.074	0	0	Epi
TMPRSS11B	0	12.2900674	0.074	0	0	Epi
MUC21	0	10.9171115	0.074	0	0	Epi
LMO3	0	13.1290143	0.074	0	0	Epi
GRTP1	0	11.3561801	0.074	0	0	Epi
ARHGAP8	0	9.44818023	0.074	0	0	Epi
SLIT2	0	12.5215769	0.074	0	0	Epi
BEND7	0	12.5307878	0.074	0	0	Epi
BMP3	0	12.0750461	0.074	0	0	Epi
CXXC4	0	11.8633268	0.074	0	0	Epi
SLC44A3	0	7.98817889	0.074	0.001	0	Epi
GALNT5	0	10.8083431	0.071	0	0	Epi
RASSF6	0	8.95102719	0.071	0	0	Epi
EFNA5	0	8.81248962	0.071	0	0	Epi
ID4	0	12.963655	0.071	0	0	Epi
PRR18	0	9.67896999	0.071	0	0	Epi
NRG4	0	8.21277923	0.071	0	0	Epi
KCNJ16	0	10.5965671	0.071	0	0	Epi
TGM3	0	9.33536549	0.071	0	0	Epi
PANX22	0	7.7008899	0.071	0	0	Epi
KRT78	0	11.9155675	0.071	0.001	0	Epi
ABCA10	0	7.21444871	0.071	0.001	0	Epi
KALRN	0	7.88519329	0.071	0.001	0	Epi
PLEKHG5	0	7.10979887	0.069	0	0	Epi
PADI1	0	13.518551	0.069	0	0	Epi
ANKRD18B	0	14.917301	0.069	0	0	Epi
TP53AIP1	0	9.49821745	0.069	0	0	Epi
LRRN11	0	8.16686098	0.069	0	0	Epi
CAV2	0	7.52216009	0.069	0.001	0	Epi
NRXN3	0	10.1767028	0.066	0	0	Epi
RHCG	0	10.8831244	0.066	0	0	Epi
SERTAD4	0	12.6509978	0.066	0	0	Epi
TOX31	0	7.99344471	0.066	0	0	Epi
ESRRG	0	13.246661	0.066	0	0	Epi
MST1R	0	7.21948866	0.066	0.001	0	Epi

PRRT2	0	5.70932442	0.066	0.001	0	Epi
DNER	0	9.94876376	0.063	0	0	Epi
HHATL	0	9.75135363	0.063	0	0	Epi
RNF39	0	13.9949683	0.063	0	0	Epi
HNF4G	0	9.10452867	0.063	0	0	Epi
PLCE1	0	10.6375473	0.063	0	0	Epi
NCCRP12	0	10.5898309	0.063	0	0	Epi
SHROOM2	0	11.1061806	0.063	0	0	Epi
LIFR	0	9.86583787	0.063	0	0	Epi
HEY2	0	10.0414103	0.063	0	0	Epi
MYCT1	0	13.4992306	0.063	0	0	Epi
CACHD1	0	11.8501176	0.061	0	0	Epi
CRCT1	0	12.3537128	0.061	0	0	Epi
MRAP2	0	11.9043759	0.061	0	0	Epi
CNKSR3	0	9.72228913	0.061	0	0	Epi
EPHB41	0	6.08003736	0.061	0	0	Epi
DMRT2	0	9.00719184	0.061	0	0	Epi
SLC16A9	0	11.8037642	0.061	0	0	Epi
CLDN10	0	12.3009736	0.061	0	0	Epi
KRT17	0	12.2322947	0.061	0	0	Epi
CHP2	0	10.2047161	0.061	0	0	Epi
CLDN16	0	13.0092638	0.061	0	0	Epi
CSMD1	0	8.10189561	0.061	0.001	0	Epi
BEND52	0	7.39774679	0.058	0	0	Epi
DMRTA2	0	8.7392193	0.058	0	0	Epi
TRIM17	0	10.0227155	0.058	0	0	Epi
SLC10A5	0	7.9738632	0.058	0	0	Epi
RNASE7	0	13.4858854	0.058	0	0	Epi
FBXW10	0	12.724262	0.058	0	0	Epi
CYP4F11	0	8.82929445	0.058	0	0	Epi
OSBP22	0	6.36159961	0.058	0	0	Epi
SLITRK6	0	12.9884135	0.058	0	0	Epi
SFTA3	0	9.86297832	0.058	0	0	Epi
CRB3	0	9.1503703	0.058	0	0	Epi
SFN	0	7.44924928	0.058	0.001	0	Epi
ARHGAP421	0	7.85566372	0.058	0.001	0	Epi
WNT4	0	11.2187376	0.056	0	0	Epi
FHL21	0	8.70627764	0.056	0	0	Epi
TMPRSS11D	0	12.024606	0.056	0	0	Epi
LARGE2	0	7.15778687	0.056	0	0	Epi
ALDH3B2	0	10.8512901	0.056	0	0	Epi
DUOX2	0	12.0505738	0.056	0	0	Epi
PRKAA2	0	11.8748409	0.056	0	0	Epi
IRX2	0	10.0975451	0.056	0	0	Epi
GRAMD2A	0	11.5483424	0.056	0	0	Epi
CFAP299	0	13.370478	0.056	0	0	Epi
SYT8	0	13.2236657	0.056	0	0	Epi
GNAL	0	7.21450744	0.056	0.001	0	Epi
SEC14L21	0	4.94085673	0.056	0.001	0	Epi
SPRR1B	0	13.6233497	0.053	0	0	Epi
AIF1L	0	9.2737719	0.053	0	0	Epi
A2ML1	0	10.4560829	0.053	0	0	Epi

NT5E	0	7.51242778	0.053	0	0	Epi
CDH3	0	11.2147604	0.053	0	0	Epi
CNTD1	0	8.83360068	0.053	0	0	Epi
KDF1	0	8.81407527	0.051	0	0	Epi
MKRN2OS	0	11.1464691	0.051	0	0	Epi
PDZD21	0	8.16022352	0.051	0	0	Epi
ATP10B	0	10.261538	0.051	0	0	Epi
SPDEF	0	9.64049752	0.051	0	0	Epi
CRISP3	0	13.0869345	0.051	0	0	Epi
ATG9B	0	12.0254052	0.051	0	0	Epi
RAB26	0	9.43086711	0.051	0	0	Epi
EBF42	0	6.10734815	0.051	0	0	Epi
LONRF21	0	7.04548258	0.051	0	0	Epi
SVOPL	0	9.29543654	0.051	0	0	Epi
MCIDAS	0	12.8319326	0.051	0	0	Epi
BICC1	0	9.70031518	0.051	0	0	Epi
VTCN1	0	10.707162	0.048	0	0	Epi
IGSF9	0	9.24686285	0.048	0	0	Epi
GXYLT21	0	9.71219042	0.048	0	0	Epi
FGFR3	0	9.12060463	0.048	0	0	Epi
SPINK5	0	13.1424914	0.048	0	0	Epi
FAM167A	0	6.72585871	0.048	0	0	Epi
PTGES1	0	7.61800267	0.048	0	0	Epi
FOLH1	0	10.9928182	0.048	0	0	Epi
MYEOV	0	10.4972939	0.048	0	0	Epi
TMEM238L	0	11.0261598	0.048	0	0	Epi
PCYT1B	0	12.5548715	0.048	0	0	Epi
LTF	0	12.4616513	0.048	0	0	Epi
LOX	0	9.67266743	0.048	0	0	Epi
JPH1	0	10.5755005	0.048	0	0	Epi
LURAP1L	0	7.43322508	0.048	0	0	Epi
HMGA2	0	13.0804165	0.048	0	0	Epi
FHOD3	0	12.2118601	0.048	0	0	Epi
FAM13C	0	13.0728578	0.048	0	0	Epi
CCDC160	0	12.9879376	0.048	0	0	Epi
RAP1GAP	0	8.97190105	0.046	0	0	Epi
IGSF32	0	7.79849975	0.046	0	0	Epi
CRNN	0	11.1965978	0.046	0	0	Epi
NFASC	0	11.2298824	0.046	0	0	Epi
REEP1	0	12.8429207	0.046	0	0	Epi
SPATA4	0	10.0973829	0.046	0	0	Epi
AC010255.3	0	7.57716559	0.046	0	0	Epi
IGFBP31	0	5.34142411	0.046	0	0	Epi
SLURP2	0	10.6462992	0.046	0	0	Epi
CATSPERB	0	5.26223572	0.046	0	0	Epi
TMEM1212	0	5.17780743	0.046	0	0	Epi
STRA6	0	13.3521736	0.046	0	0	Epi
KRT16	0	13.6975373	0.046	0	0	Epi
AP1M2	0	9.71147051	0.046	0	0	Epi
RAB3B	0	11.0258798	0.046	0	0	Epi
APOD	0	11.9969844	0.046	0	0	Epi
C6	0	13.1546065	0.046	0	0	Epi

CDRT1	0	8.78388727	0.046	0	0	Epi
GRB7	0	10.1698195	0.046	0	0	Epi
C1orf210	0	10.0024594	0.043	0	0	Epi
SOWAHB	0	11.1279598	0.043	0	0	Epi
FOXQ1	0	13.6748109	0.043	0	0	Epi
BARX2	0	13.8942748	0.043	0	0	Epi
ENOX12	0	7.31524402	0.043	0	0	Epi
SGSM1	0	7.19816871	0.043	0	0	Epi
HS6ST2	0	6.84220641	0.043	0	0	Epi
CNTN3	0	13.0555492	0.043	0	0	Epi
LRRC261	0	8.56513779	0.043	0	0	Epi
FCN3	0	10.773488	0.043	0	0	Epi
GALNT18	0	9.42814679	0.043	0	0	Epi
CLDN5	0	13.695874	0.043	0	0	Epi
TMEM61	0	10.8534501	0.041	0	0	Epi
TMPRSS11A	0	12.9923236	0.041	0	0	Epi
ADGRF5	0	13.7779308	0.041	0	0	Epi
STEAP11	0	10.0472322	0.041	0	0	Epi
SLC5A1	0	10.4951905	0.041	0	0	Epi
SRD5A2	0	10.8936305	0.041	0	0	Epi
CAND2	0	8.3048537	0.041	0	0	Epi
USH1C	0	12.0789441	0.041	0	0	Epi
IL36A	0	12.524933	0.038	0	0	Epi
MDFI	0	9.2959646	0.038	0	0	Epi
FAM83A	0	10.7702898	0.038	0	0	Epi
MRLN	0	8.34850345	0.038	0	0	Epi
ADRA2A	0	8.48704238	0.038	0	0	Epi
B3GALT5	0	10.9909249	0.038	0	0	Epi
KLHL131	0	5.44286552	0.038	0	0	Epi
VGLL1	0	11.6368347	0.038	0	0	Epi
NPNT	0	9.14075388	0.038	0	0	Epi
DMRT3	0	9.40475671	0.038	0	0	Epi
PLLP	0	11.2981977	0.038	0	0	Epi
KLHDC8A	0	12.6175294	0.038	0	0	Epi
KIAA1211	0	8.6579826	0.036	0	0	Epi
C9orf152	0	8.44084642	0.036	0	0	Epi
SH3GL3	0	11.0392588	0.036	0	0	Epi
GAREM1	0	8.55786365	0.036	0	0	Epi
SLC7A4	0	8.58369543	0.036	0	0	Epi
ITGB6	0	12.7053156	0.036	0	0	Epi
GYG2	0	13.1284865	0.036	0	0	Epi
ARHGAP29	0	10.9425153	0.033	0	0	Epi
FBLN5	0	10.0633104	0.033	0	0	Epi
FUT12	0	6.85342148	0.033	0	0	Epi
RBMS31	0	7.98088079	0.033	0	0	Epi
RNF183	0	9.90383526	0.033	0	0	Epi
IL22RA1	0	8.61280001	0.033	0	0	Epi
GPR392	0	9.11430641	0.033	0	0	Epi
RAET1E	0	9.09633596	0.033	0	0	Epi
SHH	0	11.0024871	0.033	0	0	Epi
GPR87	0	13.6139878	0.03	0	0	Epi
ADGRF2	0	10.0223307	0.03	0	0	Epi

GPX2	0	11.864669	0.03	0	0	Epi
KCNMB2	0	11.9795438	0.03	0	0	Epi
NR2F1	0	12.7782108	0.03	0	0	Epi
TMEM246	0	12.3558913	0.03	0	0	Epi
DSCAML1	0	8.28650845	0.03	0	0	Epi
AQP4	0	11.0625449	0.03	0	0	Epi
RNF152	0	9.7322182	0.03	0	0	Epi
LRRC8E	0	10.4762805	0.03	0	0	Epi
NMBR	0	10.684911	0.03	0	0	Epi
FBLIM1	0	11.1685573	0.028	0	0	Epi
ADGRB2	0	8.66881582	0.028	0	0	Epi
SMIM311	0	11.7550233	0.028	0	0	Epi
PLEKHG4B2	0	7.65919735	0.028	0	0	Epi
ZSCAN231	0	8.37305159	0.028	0	0	Epi
TCIM	0	11.2865145	0.028	0	0	Epi
NTS	0	11.5732489	0.028	0	0	Epi
SMIM22	0	11.6549136	0.028	0	0	Epi
LY6H	0	12.7751711	0.028	0	0	Epi
SCGB2A1	0	9.9390558	0.028	0	0	Epi
CRLF1	0	10.82483	0.028	0	0	Epi
COLCA2	0	11.2257955	0.028	0	0	Epi
FMO3	0	12.8077114	0.028	0	0	Epi
RNF223	0	10.5907967	0.025	0	0	Epi
SPRR2A	0	11.4881346	0.025	0	0	Epi
CDC20B	0	13.5877893	0.025	0	0	Epi
RGS7BP	0	11.2916323	0.025	0	0	Epi
RGS171	0	7.98606974	0.025	0	0	Epi
NDNF	0	11.6171546	0.025	0	0	Epi
CCDC184	0	10.8812558	0.025	0	0	Epi
SIAH3	0	8.16657954	0.025	0	0	Epi
KCNB1	0	10.0993359	0.025	0	0	Epi
SYTL5	0	11.5111272	0.025	0	0	Epi
PRRX2	0	11.1053287	0.025	0	0	Epi
GRIK2	0	11.4756106	0.025	0	0	Epi
ARX	0	12.3366964	0.025	0	0	Epi
SLC26A5	0	10.1355322	0.025	0	0	Epi
C14orf39	0	11.9504256	0.025	0	0	Epi
NOS2	0	10.3569194	0.023	0	0	Epi
SHISA2	0	10.3673973	0.023	0	0	Epi
RIMS1	0	12.467026	0.023	0	0	Epi
SHISA9	0	12.1311789	0.023	0	0	Epi
HUNK	0	9.7083674	0.023	0	0	Epi
CYP2C18	0	11.6819432	0.023	0	0	Epi
TAGLN3	0	11.1689141	0.023	0	0	Epi
FBXO161	0	10.4454362	0.023	0	0	Epi
CEACAM7	0	14.2867526	0.02	0	0	Epi
PCDH20	0	11.4403534	0.02	0	0	Epi
CACNA1H	0	9.54221667	0.02	0	0	Epi
PTPN141	0	10.587059	0.02	0	0	Epi
TMEM213	0	11.577892	0.02	0	0	Epi
PIP	0	12.4130228	0.02	0	0	Epi
DOC2B	0	9.13581074	0.02	0	0	Epi

NGEF	0	10.877127	0.02	0	0	Epi
LY6D	0	12.8315142	0.02	0	0	Epi
STEAP2	0	10.6224078	0.02	0	0	Epi
CDH12	0	11.0169102	0.02	0	0	Epi
SGCE	0	9.86252669	0.018	0	0	Epi
CFTR	0	13.0717552	0.018	0	0	Epi
CA8	0	11.8758476	0.018	0	0	Epi
FOXA2	0	10.9970138	0.018	0	0	Epi
NCMAP	0	9.08995225	0.018	0	0	Epi
STRIT1	0	11.4620488	0.018	0	0	Epi
BOC	0	9.11337123	0.018	0	0	Epi
TMEM150C	0	10.4174431	0.018	0	0	Epi
FAM83B	0	11.886854	0.018	0	0	Epi
ADAMTSL3	0	12.2851347	0.018	0	0	Epi
IP6K3	0	12.5865843	0.018	0	0	Epi
PTN	0	9.79220824	0.018	0	0	Epi
DNAJC221	0	11.1765169	0.018	0	0	Epi
KCNE4	0	12.4546324	0.018	0	0	Epi
RAI2	0	11.1610912	0.018	0	0	Epi
IVL	0	11.9862261	0.015	0	0	Epi
SPRR2D	0	10.5389423	0.015	0	0	Epi
GLI2	0	12.3048808	0.015	0	0	Epi
FAXC	0	11.0613746	0.015	0	0	Epi
SEMA5A	0	12.720798	0.015	0	0	Epi
CWH43	0	10.6022792	0.015	0	0	Epi
KISS1	0	13.8362245	0.015	0	0	Epi
TRIM311	0	11.5719957	0.015	0	0	Epi
MKX	0	11.9761454	0.015	0	0	Epi
CHST4	0	12.2074798	0.015	0	0	Epi
ADH6	0	11.7253022	0.015	0	0	Epi
LOXL41	0	10.1074093	0.015	0	0	Epi
PRKD1	0	10.3293346	0.015	0	0	Epi
ACP7	0	7.5336994	0.015	0	0	Epi
C2CD4B	0	11.9067637	0.015	0	0	Epi
PPARGC1A	0	9.36689008	0.015	0	0	Epi
DMGDH	0	9.97697621	0.015	0	0	Epi
PNMA8C	0	12.0001869	0.015	0	0	Epi
CARD14	0	12.3097267	0.013	0	0	Epi
KY	0	12.3598982	0.013	0	0	Epi
CNGA3	0	10.448046	0.013	0	0	Epi
EFS	0	11.2738704	0.013	0	0	Epi
ACOX2	0	9.68162371	0.013	0	0	Epi
CRISP2	0	11.8717803	0.013	0	0	Epi
CLCA4	0	13.431965	0.01	0	0	Epi
PPP2R2C	0	12.0074392	0.01	0	0	Epi
SLURP1	0	11.2203964	0.01	0	0	Epi
KLC3	0	12.3642189	0.01	0	0	Epi
SIM2	0	12.0454347	0.01	0	0	Epi
TLL1	0	10.980455	0.01	0	0	Epi
FLRT3	0	11.2547002	0.01	0	0	Epi
ACADL	0	12.9306175	0.01	0	0	Epi
UCN3	0	11.7423994	0.01	0	0	Epi

HNF1A	0	12.2248167	0.01	0	0	Epi
SEMA3E	0	10.979056	0.01	0	0	Epi
GJB7	0	11.113617	0.01	0	0	Epi
BMP4	0	11.0020744	0.01	0	0	Epi
CACNA1D	5.385E-307	6.97976165	0.081	0.002	8.277E-303	Epi
EGLN32	9.597E-306	6.10087295	0.056	0.001	1.475E-301	Epi
C21orf581	9.977E-306	6.19005331	0.294	0.022	1.534E-301	Epi
FOXA3	2.138E-304	9.14712099	0.015	0	3.287E-300	Epi
SLC6A20	2.171E-304	8.86276577	0.015	0	3.337E-300	Epi
ENTPD2	2.178E-304	7.85393394	0.015	0	3.348E-300	Epi
KIAA0895	9.961E-304	6.04827266	0.135	0.005	1.531E-299	Epi
TTPA	2.07E-302	10.8446717	0.013	0	3.182E-298	Epi
CYP2W1	2.108E-302	9.89616158	0.013	0	3.241E-298	Epi
SLC25A47	5.567E-302	5.2562026	0.206	0.011	8.558E-298	Epi
SMARCA1	2.531E-301	7.19107411	0.081	0.002	3.89E-297	Epi
MARC21	3.635E-301	6.37556934	0.117	0.003	5.587E-297	Epi
NEDD4L6	5.281E-300	5.40074628	0.426	0.046	8.118E-296	Epi
LEKR1	5.819E-298	6.32167561	0.099	0.002	8.945E-294	Epi
AJUBA	3.496E-297	7.73199415	0.043	0	5.374E-293	Epi
NAV21	7.921E-297	5.52719481	0.195	0.01	1.218E-292	Epi
PIH1D21	3.132E-296	6.29240663	0.231	0.014	4.815E-292	Epi
SYT7	1.605E-295	9.67320053	0.018	0	2.467E-291	Epi
NELL21	1.872E-295	3.19113553	0.147	0.005	2.877E-291	Epi
AHNAK21	6.167E-294	6.31558195	0.127	0.004	9.479E-290	Epi
ATP13A41	3.415E-292	6.11648647	0.231	0.014	5.25E-288	Epi
RAB27B1	7.59E-292	4.09727836	0.071	0.001	1.167E-287	Epi
LAMB31	2.015E-291	5.99996418	0.132	0.005	3.098E-287	Epi
PLCH23	5.804E-289	5.12675349	0.058	0.001	8.921E-285	Epi
SPTBN13	9.255E-287	4.32194821	0.289	0.022	1.423E-282	Epi
ENTPD3	4.32E-284	9.78140714	0.023	0	6.64E-280	Epi
C9orf1161	1.503E-283	6.87987666	0.256	0.018	2.31E-279	Epi
CAPS21	9.023E-282	6.0433834	0.188	0.01	1.387E-277	Epi
HSPA4L	7.202E-280	6.56575305	0.094	0.002	1.107E-275	Epi
PCSK61	3.851E-279	7.0734627	0.084	0.002	5.92E-275	Epi
SLC1A1	3.23E-277	6.24657436	0.033	0	4.964E-273	Epi
CSF3	1.058E-276	10.2830447	0.015	0	1.626E-272	Epi
ARHGAP40	9.371E-276	8.98001454	0.018	0	1.44E-271	Epi
FOXN4	2.189E-271	11.2488863	0.01	0	3.364E-267	Epi
FMN2	2.198E-271	10.7825008	0.01	0	3.378E-267	Epi
KRT6A	2.198E-271	11.8313277	0.01	0	3.378E-267	Epi
CLDN22	2.198E-271	11.2993732	0.01	0	3.378E-267	Epi
ISL1	2.198E-271	10.8658111	0.01	0	3.378E-267	Epi
MFAP2	2.225E-271	8.00809765	0.01	0	3.42E-267	Epi
SLC51A	5.787E-271	6.27121719	0.107	0.003	8.895E-267	Epi
KRT82	2.24E-270	6.13106389	0.386	0.042	3.443E-266	Epi
CATIP	7.514E-270	5.8494502	0.188	0.01	1.155E-265	Epi
AQP6	1.052E-269	9.59105434	0.02	0	1.617E-265	Epi
THSD7A	1.069E-269	9.25989786	0.02	0	1.643E-265	Epi
ZNF8273	7.48E-269	3.78878212	0.074	0.001	1.15E-264	Epi
TMEM8B3	2.26E-267	5.55848143	0.178	0.009	3.474E-263	Epi
ZNRF14	5.956E-267	4.07549915	0.264	0.019	9.156E-263	Epi
PALMD	1.071E-266	8.48167454	0.033	0	1.646E-262	Epi

MSLN1	5.881E-266	7.27063445	0.145	0.006	9.04E-262	Epi
KLK13	6.779E-265	8.31730562	0.051	0.001	1.042E-260	Epi
FAM227B	3.034E-264	6.00450201	0.074	0.002	4.664E-260	Epi
VANGL2	3.76E-264	6.57880224	0.058	0.001	5.78E-260	Epi
HOGA1	5.9E-264	7.85438241	0.036	0	9.07E-260	Epi
VEPH1	4.014E-262	7.64305626	0.03	0	6.169E-258	Epi
MAST42	1.218E-261	3.46274845	0.203	0.012	1.872E-257	Epi
BSPRY1	1.706E-261	6.15706826	0.102	0.003	2.622E-257	Epi
DUSP44	6.392E-261	2.83613342	0.188	0.01	9.825E-257	Epi
NEURL32	2.097E-260	6.17326466	0.038	0	3.224E-256	Epi
PPIL61	3.376E-260	6.29424081	0.206	0.012	5.189E-256	Epi
HRK	3.401E-260	8.23133179	0.028	0	5.228E-256	Epi
CCDC1802	8.503E-259	5.75001842	0.239	0.017	1.307E-254	Epi
CDH14	1.102E-258	3.69524291	0.358	0.036	1.693E-254	Epi
PROM2	1.118E-258	6.50349811	0.104	0.003	1.719E-254	Epi
JCAD	1.866E-258	6.39434853	0.018	0	2.869E-254	Epi
ATP7B	1.744E-257	7.02356292	0.096	0.003	2.681E-253	Epi
TRMT9B	3.361E-257	6.04538791	0.147	0.006	5.166E-253	Epi
RHBDF1	7.998E-257	7.95848	0.033	0	1.229E-252	Epi
FLG	8.32E-257	11.1172132	0.02	0	1.279E-252	Epi
ABHD12B	1.806E-256	5.95415615	0.17	0.009	2.777E-252	Epi
PRR36	1.905E-256	6.88112576	0.03	0	2.928E-252	Epi
ZNF5071	1.428E-253	2.78092585	0.152	0.007	2.195E-249	Epi
LAMA3	2.55E-253	6.75399439	0.048	0.001	3.92E-249	Epi
MID11	2.917E-252	5.99282082	0.203	0.012	4.483E-248	Epi
LCA5L	2.988E-251	5.99887873	0.155	0.007	4.593E-247	Epi
FNBP1L3	7.817E-250	5.63767629	0.145	0.006	1.202E-245	Epi
STXBP6	1.171E-249	7.77170666	0.069	0.001	1.8E-245	Epi
MAK	6.803E-249	6.31053006	0.104	0.003	1.046E-244	Epi
MAPK8IP12	3.458E-248	5.79581341	0.201	0.012	5.315E-244	Epi
DBNDD1	9.904E-248	8.04869258	0.033	0	1.522E-243	Epi
CCL151	1.14E-247	4.65151486	0.142	0.006	1.752E-243	Epi
F3	1.737E-247	7.34736371	0.317	0.03	2.67E-243	Epi
CORO2B	4.055E-245	6.82699196	0.076	0.002	6.232E-241	Epi
EPHB33	1.491E-244	5.77978373	0.086	0.002	2.292E-240	Epi
SPEF22	2.293E-240	6.0311913	0.327	0.033	3.525E-236	Epi
ARHGAP39	2.269E-238	5.82217512	0.236	0.018	3.487E-234	Epi
SELENOP2	4.399E-236	3.94773272	0.16	0.008	6.762E-232	Epi
FBLN2	3.414E-235	7.92637551	0.013	0	5.248E-231	Epi
OCLN3	4.799E-234	5.21367319	0.241	0.019	7.376E-230	Epi
FAM110C	4.903E-234	5.66468522	0.015	0	7.536E-230	Epi
LRGUK1	5.172E-234	5.89045336	0.254	0.021	7.949E-230	Epi
RNF180	4.242E-231	6.70426562	0.074	0.002	6.521E-227	Epi
MUC202	8.45E-231	5.61924339	0.497	0.082	1.299E-226	Epi
CCDC1892	8.432E-230	6.44715647	0.259	0.022	1.296E-225	Epi
MUC2	1.152E-229	11.2612416	0.018	0	1.771E-225	Epi
LRP52	1.601E-229	5.25872721	0.18	0.011	2.461E-225	Epi
FRK1	5.822E-229	5.16987993	0.226	0.017	8.948E-225	Epi
AXIN2	5.515E-228	5.50282979	0.043	0.001	8.477E-224	Epi
VIPR1	1.205E-226	5.36291794	0.036	0	1.851E-222	Epi
FAM221B	3.167E-226	10.2606994	0.01	0	4.868E-222	Epi
FAM71E2	3.178E-226	11.7750699	0.01	0	4.885E-222	Epi

LRRC31	3.189E-226	10.4440969	0.01	0	4.901E-222	Epi
SHC2	3.221E-226	7.96168859	0.01	0	4.951E-222	Epi
ADGRL2	3.221E-226	8.98334801	0.01	0	4.951E-222	Epi
EVA1A	3.232E-226	8.56963637	0.01	0	4.968E-222	Epi
SHISA8	3.254E-226	8.20270316	0.01	0	5.002E-222	Epi
ANO92	1.268E-224	3.34841407	0.104	0.004	1.949E-220	Epi
GPRC5C1	4.371E-224	5.38528984	0.155	0.008	6.719E-220	Epi
SLC10A6	1.123E-223	6.79370552	0.041	0.001	1.726E-219	Epi
AMPD36	1.871E-222	4.3036678	0.221	0.016	2.875E-218	Epi
PYROXD24	1.598E-221	5.10253768	0.127	0.005	2.456E-217	Epi
MAOA1	2.03E-221	6.32917332	0.094	0.003	3.12E-217	Epi
RNF431	3.338E-221	6.07797871	0.109	0.004	5.131E-217	Epi
PKIB3	4.006E-221	4.49273085	0.135	0.006	6.157E-217	Epi
FBXO172	1.031E-220	7.14952041	0.025	0	1.584E-216	Epi
MOK2	1.746E-220	5.90727861	0.317	0.034	2.684E-216	Epi
CCL14	1.148E-219	8.00500576	0.043	0.001	1.765E-215	Epi
PRR73	6.391E-218	4.86882953	0.127	0.006	9.823E-214	Epi
FAM181A	2.63E-217	8.54254103	0.015	0	4.042E-213	Epi
WNK4	1.611E-215	9.21559661	0.02	0	2.477E-211	Epi
CCDC302	5.616E-215	5.68443173	0.297	0.03	8.633E-211	Epi
ID1	1.037E-214	7.12226437	0.084	0.002	1.594E-210	Epi
HGD	3.161E-213	5.45235501	0.069	0.002	4.859E-209	Epi
CCDC1511	6.084E-213	5.87333484	0.208	0.015	9.352E-209	Epi
MUC22	1.088E-211	13.382027	0.013	0	1.672E-207	Epi
TSPOAP14	1.959E-211	4.24840689	0.287	0.028	3.011E-207	Epi
ZNF704	1.528E-209	5.4428207	0.089	0.003	2.348E-205	Epi
ARHGEF33	5.801E-208	6.26137667	0.081	0.002	8.917E-204	Epi
PARD6B1	5.908E-208	5.50888223	0.256	0.023	9.081E-204	Epi
PXN4	1.84E-206	3.52844495	0.264	0.024	2.829E-202	Epi
LRIG12	7.009E-205	4.86811286	0.335	0.04	1.077E-200	Epi
TIGD4	1.304E-204	6.45676642	0.063	0.001	2.005E-200	Epi
ZBTB161	3.049E-203	3.84115068	0.104	0.004	4.686E-199	Epi
SMIM61	4.99E-203	7.41412171	0.051	0.001	7.67E-199	Epi
COX6B2	3.545E-201	7.48795914	0.058	0.001	5.449E-197	Epi
FLACC12	4.68E-201	5.10970018	0.193	0.014	7.193E-197	Epi
CAMK2N11	1.219E-200	5.62801503	0.048	0.001	1.873E-196	Epi
FOXC1	3.767E-199	5.62163314	0.168	0.011	5.79E-195	Epi
MNS1	6.301E-199	5.81990337	0.259	0.025	9.686E-195	Epi
DOC2A2	7.422E-199	6.16732478	0.157	0.009	1.141E-194	Epi
SULT2B1	1.755E-198	6.95673925	0.079	0.002	2.698E-194	Epi
RNF207	5.16E-198	6.43376584	0.041	0.001	7.931E-194	Epi
CDH13	1.005E-196	8.63433176	0.018	0	1.545E-192	Epi
CCDC1463	7.392E-196	5.43305559	0.315	0.037	1.136E-191	Epi
TMEM200C	5.815E-194	9.74948001	0.01	0	8.937E-190	Epi
SPINK7	5.831E-194	10.332997	0.01	0	8.963E-190	Epi
SLC2A16	8.091E-194	3.59119957	0.213	0.017	1.244E-189	Epi
GJA1	1.947E-192	10.4900161	0.013	0	2.993E-188	Epi
IL36RN	1.997E-192	8.2559705	0.013	0	3.07E-188	Epi
SH3RF21	2.049E-191	6.27367618	0.061	0.001	3.15E-187	Epi
CYP4F12	4.434E-189	7.47490274	0.053	0.001	6.816E-185	Epi
ARMH1	1.978E-187	5.57907796	0.183	0.013	3.041E-183	Epi
CCDC1571	4.727E-187	5.06680931	0.19	0.014	7.266E-183	Epi

CCDC113	6.55E-187	5.90335091	0.221	0.019	1.007E-182	Epi
KIAA1211L1	6.119E-186	5.64770072	0.307	0.037	9.406E-182	Epi
CDC42BPG2	8.333E-185	4.99698243	0.261	0.027	1.281E-180	Epi
RHPN11	1.053E-184	5.14687505	0.32	0.04	1.619E-180	Epi
KLF51	2.745E-183	5.56571394	0.414	0.068	4.22E-179	Epi
MYO103	5.833E-183	5.03679672	0.155	0.01	8.966E-179	Epi
SLC25A27	2.708E-182	6.56963107	0.071	0.002	4.162E-178	Epi
APBB11	3.82E-181	3.57592181	0.081	0.003	5.872E-177	Epi
KCNN3	6.72E-181	7.91959839	0.038	0.001	1.033E-176	Epi
KCNH32	2.02E-180	5.64705765	0.142	0.008	3.104E-176	Epi
FBXO361	5.967E-180	5.34726137	0.178	0.013	9.173E-176	Epi
LEXM	1.727E-179	4.31378508	0.018	0	2.655E-175	Epi
CGNL1	9.383E-179	6.66291706	0.046	0.001	1.442E-174	Epi
NINL1	1.516E-178	5.08733817	0.076	0.002	2.331E-174	Epi
C1orf214	1.026E-177	4.78327597	0.162	0.011	1.577E-173	Epi
CRYBG21	9.162E-177	5.11658798	0.063	0.002	1.408E-172	Epi
TSPAN132	6.253E-175	4.92847926	0.294	0.036	9.611E-171	Epi
SFTPD	3.403E-174	9.09685729	0.023	0	5.23E-170	Epi
TMEM178A	2.28E-172	5.99588138	0.086	0.003	3.504E-168	Epi
GP6	2.271E-171	6.01072199	0.056	0.001	3.492E-167	Epi
MCTP22	7.081E-171	2.47560663	0.094	0.004	1.088E-166	Epi
PCSK1N1	7.605E-170	4.687627	0.091	0.004	1.169E-165	Epi
SFTA2	9.204E-170	11.4321229	0.01	0	1.415E-165	Epi
EEF1A2	9.686E-170	5.7041491	0.023	0	1.489E-165	Epi
NRG2	4.53E-169	6.66982211	0.033	0	6.964E-165	Epi
ENO24	2.257E-167	2.49444432	0.14	0.009	3.469E-163	Epi
PDGFA2	5.953E-165	4.82716231	0.046	0.001	9.15E-161	Epi
CRISPLD1	2.206E-163	7.28300225	0.051	0.001	3.391E-159	Epi
SMPD32	5.088E-163	4.87405695	0.168	0.013	7.82E-159	Epi
LRP4	8.34E-163	6.39365841	0.013	0	1.282E-158	Epi
DCDC2	3.901E-161	6.12799818	0.089	0.004	5.996E-157	Epi
EPHA21	5.64E-161	5.86939367	0.335	0.05	8.669E-157	Epi
PRRT1	9.947E-161	7.09997213	0.025	0	1.529E-156	Epi
LZTS31	3.938E-160	5.25424134	0.18	0.015	6.053E-156	Epi
PEG10	1.072E-158	6.11085761	0.018	0	1.647E-154	Epi
MYLK3	2.958E-158	5.37649994	0.063	0.002	4.546E-154	Epi
PLEKHG13	1.099E-157	3.74999224	0.058	0.002	1.689E-153	Epi
ZC2HC1C	1.23E-156	5.62014965	0.228	0.024	1.891E-152	Epi
THRB2	1.6E-156	6.40757838	0.071	0.002	2.46E-152	Epi
RIPK42	4.194E-156	4.92373336	0.173	0.014	6.447E-152	Epi
NFATC41	4.933E-156	6.20578421	0.061	0.002	7.582E-152	Epi
ZSWIM51	5.624E-156	5.02020327	0.046	0.001	8.645E-152	Epi
GSTO2	8.599E-155	6.0550472	0.074	0.003	1.322E-150	Epi
SDR42E11	1.518E-154	5.2641463	0.038	0.001	2.334E-150	Epi
DMRTA1	9.583E-154	7.08300283	0.041	0.001	1.473E-149	Epi
NPDC13	1.16E-153	3.30624616	0.104	0.005	1.784E-149	Epi
IGFBP74	1.674E-152	5.58255068	0.279	0.036	2.574E-148	Epi
ADGRA31	5.29E-152	6.73961732	0.038	0.001	8.131E-148	Epi
SLC16A21	3.714E-151	5.18616304	0.013	0	5.709E-147	Epi
IQCA11	6.025E-151	5.39459584	0.157	0.012	9.26E-147	Epi
CLDN18	6.218E-151	12.1704155	0.01	0	9.558E-147	Epi
CDKL2	6.625E-151	5.19845157	0.084	0.004	1.018E-146	Epi

BBOF12	6.78E-151	5.45072793	0.322	0.049	1.042E-146	Epi
PTPN31	2.437E-150	5.03116373	0.241	0.028	3.746E-146	Epi
CST63	2.468E-150	5.74439129	0.091	0.004	3.794E-146	Epi
B9D11	2.368E-149	5.74037073	0.317	0.048	3.639E-145	Epi
ILDR1	5.132E-149	5.77271253	0.114	0.007	7.888E-145	Epi
GPR1624	6.002E-149	5.42703509	0.289	0.04	9.226E-145	Epi
FAT1	6.041E-149	6.20286286	0.079	0.003	9.285E-145	Epi
NPAS3	3.753E-148	7.24364092	0.028	0	5.769E-144	Epi
CFAP442	5.313E-148	5.02388351	0.312	0.046	8.167E-144	Epi
NEGR12	2.193E-146	5.71762748	0.025	0	3.371E-142	Epi
HES4	2.517E-145	5.35700637	0.173	0.015	3.869E-141	Epi
SNCG	3.457E-145	8.45142804	0.02	0	5.313E-141	Epi
FAM47E1	1.858E-144	4.89735945	0.234	0.027	2.856E-140	Epi
GLB1L21	3.14E-143	5.20383061	0.155	0.012	4.826E-139	Epi
KRTCAP32	5.262E-143	4.30258911	0.193	0.019	8.088E-139	Epi
ACBD7	6.485E-143	6.7161472	0.041	0.001	9.968E-139	Epi
CAPS1	8.216E-143	7.01160605	0.431	0.093	1.263E-138	Epi
EML6	2.299E-142	5.37271651	0.114	0.007	3.534E-138	Epi
PPP1R1A1	7.328E-142	7.67266373	0.036	0.001	1.126E-137	Epi
ZNF2141	1.046E-141	5.314267	0.135	0.009	1.608E-137	Epi
POU6F14	1.125E-141	3.57958562	0.069	0.003	1.73E-137	Epi
MAP91	3.199E-141	4.56306394	0.193	0.019	4.917E-137	Epi
CD200R1L	4.227E-141	9.23282839	0.013	0	6.498E-137	Epi
B3GNT6	4.343E-141	7.80359646	0.013	0	6.675E-137	Epi
SH2D1B1	2.042E-140	4.57734377	0.036	0.001	3.139E-136	Epi
HSPA12A1	8.752E-140	7.02547972	0.028	0	1.345E-135	Epi
DLL11	2.143E-139	5.58198656	0.127	0.009	3.294E-135	Epi
GNA141	6.561E-139	5.15447945	0.198	0.02	1.008E-134	Epi
SNCAIP	7.103E-139	5.66924304	0.112	0.007	1.092E-134	Epi
BDH1	8.151E-139	4.48926573	0.266	0.036	1.253E-134	Epi
KRT51	4.375E-138	6.8297095	0.015	0	6.725E-134	Epi
GCNT4	8.709E-138	4.88971464	0.028	0	1.339E-133	Epi
DLG32	1.233E-137	3.87670685	0.119	0.008	1.896E-133	Epi
AXDND1	2.493E-137	5.84508511	0.058	0.002	3.832E-133	Epi
SLC52A1	5.981E-136	5.99271555	0.061	0.002	9.194E-132	Epi
NPAS1	7.138E-136	10.2397651	0.01	0	1.097E-131	Epi
ANTXR11	1.023E-135	6.70235987	0.051	0.001	1.573E-131	Epi
RNF128	3.527E-134	7.4697297	0.02	0	5.422E-130	Epi
SOX15	3.606E-134	7.1271522	0.02	0	5.543E-130	Epi
LAD12	5.69E-134	4.75572625	0.058	0.002	8.745E-130	Epi
C16orf461	8.713E-134	4.92244581	0.193	0.02	1.339E-129	Epi
BIK2	4.167E-133	4.8890965	0.112	0.007	6.404E-129	Epi
FGFBP1	2.851E-132	9.49760122	0.013	0	4.382E-128	Epi
TTC21A1	7.25E-132	4.98361529	0.312	0.051	1.114E-127	Epi
CLIC33	2.586E-131	4.59042732	0.066	0.003	3.975E-127	Epi
CMYA5	4.598E-131	6.39975122	0.051	0.001	7.068E-127	Epi
CROCC3	1.454E-130	5.76611009	0.363	0.07	2.235E-126	Epi
MALL	6.65E-130	7.14391508	0.046	0.001	1.022E-125	Epi
KLK10	6.643E-128	5.97836455	0.112	0.007	1.021E-123	Epi
ZNF853	1.05E-127	5.48821911	0.02	0	1.614E-123	Epi
SAMD15	4.908E-127	5.59129633	0.129	0.01	7.543E-123	Epi
DOK41	9.811E-126	4.9460506	0.071	0.003	1.508E-121	Epi

LKAAEAR1	1.036E-124	6.73876236	0.02	0	1.592E-120	Epi
EVA1C	1.437E-124	4.24679379	0.206	0.024	2.209E-120	Epi
GABRB3	1.69E-124	7.51703889	0.033	0.001	2.598E-120	Epi
DUOXA2	1.736E-124	11.5760015	0.013	0	2.668E-120	Epi
GLRB	1.532E-123	8.21131026	0.01	0	2.355E-119	Epi
CCDC131	4.298E-123	5.25694486	0.299	0.05	6.607E-119	Epi
ABCA53	1.329E-122	4.10764842	0.335	0.062	2.042E-118	Epi
TLCD51	2.131E-122	5.94610443	0.028	0	3.275E-118	Epi
DENND6B2	2.349E-122	5.25832372	0.266	0.04	3.611E-118	Epi
CHAC13	1.915E-121	6.05122834	0.061	0.002	2.944E-117	Epi
EPHX2	2.119E-121	5.31453536	0.084	0.004	3.257E-117	Epi
FHDC11	2.869E-120	5.4596713	0.074	0.003	4.41E-116	Epi
TCTEX1D22	3.898E-120	4.8269163	0.249	0.036	5.991E-116	Epi
DERL31	5.999E-120	5.56832002	0.089	0.005	9.221E-116	Epi
MIPOL1	5.827E-119	6.4320143	0.071	0.003	8.956E-115	Epi
PROC3	5.98E-118	5.80895688	0.028	0	9.192E-114	Epi
KCNS3	9.488E-118	6.64868637	0.03	0.001	1.458E-113	Epi
EVC3	1.198E-117	4.12615037	0.071	0.003	1.842E-113	Epi
FAM171A1	1.552E-117	5.0946848	0.013	0	2.385E-113	Epi
CARD102	9.609E-117	5.82593655	0.015	0	1.477E-112	Epi
KCNK51	1.795E-116	4.1061371	0.03	0.001	2.759E-112	Epi
CLIC51	2.65E-116	3.34714936	0.076	0.004	4.073E-112	Epi
TMEM2311	3.37E-116	5.91946984	0.345	0.07	5.18E-112	Epi
ITGA91	1.49E-115	4.87239254	0.036	0.001	2.291E-111	Epi
ITM2C3	3.817E-115	1.85757872	0.137	0.012	5.867E-111	Epi
SLC15A25	4.628E-115	4.61068226	0.274	0.044	7.114E-111	Epi
HPX	5.501E-115	5.46616217	0.046	0.001	8.456E-111	Epi
NYAP1	1.634E-114	6.05675101	0.023	0	2.511E-110	Epi
C1orf226	6.686E-114	7.49108417	0.028	0.001	1.028E-109	Epi
AGAP11	7.016E-114	4.05171365	0.251	0.038	1.078E-109	Epi
TFAP2C	7.753E-114	5.3362486	0.043	0.001	1.192E-109	Epi
CAPN51	2.172E-113	4.92335724	0.244	0.036	3.338E-109	Epi
MMP24	2.871E-113	7.27257291	0.01	0	4.414E-109	Epi
GIPC2	3.507E-113	6.162911	0.051	0.002	5.39E-109	Epi
CHST154	4.177E-113	4.40334911	0.056	0.002	6.421E-109	Epi
PROSER2	2.44E-112	5.7391641	0.051	0.002	3.75E-108	Epi
ARSJ	2.709E-112	7.07489745	0.025	0	4.164E-108	Epi
RAB153	3.023E-112	4.64634057	0.046	0.001	4.646E-108	Epi
TGFBR32	3.746E-112	2.7535591	0.056	0.002	5.758E-108	Epi
TENT5B	8.032E-112	6.37142883	0.048	0.002	1.235E-107	Epi
ABO	5.807E-111	5.51716113	0.076	0.004	8.926E-107	Epi
ADAM286	6.395E-111	4.66307973	0.119	0.009	9.83E-107	Epi
MAOB	4.782E-110	5.10359895	0.084	0.005	7.351E-106	Epi
TTC262	7E-110	4.78312095	0.246	0.038	1.076E-105	Epi
CEP1262	7.221E-110	4.51623309	0.256	0.041	1.11E-105	Epi
NPHP11	1.712E-109	5.14265249	0.305	0.057	2.631E-105	Epi
GCNT3	4.485E-109	7.54401296	0.076	0.004	6.894E-105	Epi
HTRA12	5.493E-109	4.23603824	0.036	0.001	8.443E-105	Epi
PTPN131	1.508E-108	4.45753508	0.117	0.009	2.318E-104	Epi
PRRG21	2.212E-108	5.11157831	0.102	0.007	3.4E-104	Epi
SPAG162	5.844E-108	5.04786086	0.368	0.084	8.982E-104	Epi
PDE4D2	1.41E-107	2.15179241	0.206	0.026	2.167E-103	Epi

ADAMTS142	7.055E-107	6.01001906	0.03	0.001	1.084E-102	Epi
NOS1AP	1.868E-106	5.90792078	0.051	0.002	2.871E-102	Epi
EFHC11	7.119E-106	5.38356062	0.398	0.1	1.094E-101	Epi
NEIL12	3.579E-105	4.4788105	0.292	0.054	5.501E-101	Epi
C2orf66	6.262E-105	6.10145887	0.02	0	9.625E-101	Epi
LLGL21	1.004E-104	3.80795274	0.414	0.106	1.543E-100	Epi
SMIM35	1.263E-104	7.0225144	0.015	0	1.942E-100	Epi
DNALI1	3.569E-104	5.17135	0.368	0.087	5.485E-100	Epi
MEX3A	4.297E-104	7.36127091	0.036	0.001	6.605E-100	Epi
GATA6	5.855E-104	5.41076794	0.036	0.001	9E-100	Epi
MEIG1	8.209E-104	5.23847627	0.099	0.007	1.262E-99	Epi
BMERB13	8.294E-103	6.3057525	0.053	0.002	1.2749E-98	Epi
KIAA1257	4.142E-102	5.13535451	0.119	0.01	6.3666E-98	Epi
FBXO23	4.165E-102	3.29731766	0.038	0.001	6.4016E-98	Epi
TNNI3	1.651E-101	5.70215728	0.102	0.007	2.5375E-97	Epi
LRRC23	3.796E-101	4.66912064	0.284	0.054	5.8347E-97	Epi
TMEM132A	6.398E-101	6.4466435	0.02	0	9.8346E-97	Epi
NHLRC4	9.738E-101	5.06240607	0.112	0.009	1.4968E-96	Epi
CCDC88C2	2.831E-100	2.80459336	0.282	0.05	4.351E-96	Epi
SYNE4	2.849E-99	5.18636876	0.069	0.004	4.3793E-95	Epi
EPHA41	4.236E-99	3.00775581	0.036	0.001	6.5114E-95	Epi
PCSK42	5.056E-99	4.66106036	0.114	0.01	7.7713E-95	Epi
N4BP33	4.48E-98	3.21634815	0.038	0.001	6.8863E-94	Epi
SLCO4A1	7.3624E-98	6.31268848	0.018	0	1.1317E-93	Epi
MUCL1	7.7796E-98	5.239331	0.028	0.001	1.1958E-93	Epi
ARMH4	1.3363E-97	6.39652742	0.056	0.002	2.0541E-93	Epi
UPK3B1	1.8925E-97	4.86491356	0.114	0.01	2.9089E-93	Epi
TLCD3A2	5.1538E-97	5.29177572	0.071	0.004	7.9219E-93	Epi
PPP1R14C1	4.0241E-96	4.329179	0.193	0.026	6.1855E-92	Epi
KHDRBS31	7.8451E-96	5.58018903	0.053	0.002	1.2059E-91	Epi
NAALADL21	1.3508E-95	4.62417156	0.132	0.013	2.0764E-91	Epi
NEURL1B	1.9209E-95	4.27470457	0.086	0.006	2.9527E-91	Epi
PON3	1.9279E-95	7.1093451	0.02	0	2.9634E-91	Epi
CASTOR31	3.75E-95	4.41504365	0.16	0.019	5.7641E-91	Epi
EHD2	4.6776E-95	6.99148618	0.028	0.001	7.19E-91	Epi
CLDN91	1.422E-94	5.82452044	0.03	0.001	2.1858E-90	Epi
ABLIM31	1.7829E-94	7.33900099	0.041	0.001	2.7405E-90	Epi
LRRC491	3.3099E-94	4.78210612	0.132	0.013	5.0877E-90	Epi
NAT143	3.3398E-94	4.69422869	0.241	0.041	5.1336E-90	Epi
NPAS21	6.8663E-94	4.76732523	0.112	0.01	1.0554E-89	Epi
CFH1	2.201E-93	4.67271405	0.104	0.008	3.3832E-89	Epi
SERPINF13	2.6896E-93	4.23693288	0.343	0.08	4.1341E-89	Epi
DAB2IP1	6.933E-93	4.75689988	0.119	0.011	1.0657E-88	Epi
TPCN15	5.6378E-92	4.43309061	0.109	0.009	8.6659E-88	Epi
PAQR74	8.9215E-92	4.49447328	0.122	0.012	1.3713E-87	Epi
TMEM521	1.2222E-90	5.14993151	0.114	0.01	1.8787E-86	Epi
C3orf67	3.0148E-90	5.09776953	0.096	0.008	4.6341E-86	Epi
MYCL3	1.096E-89	4.5799989	0.051	0.002	1.6847E-85	Epi
GSDMB4	1.9954E-89	3.18214616	0.165	0.021	3.0671E-85	Epi
MGP	5.8495E-89	5.66144905	0.02	0	8.9913E-85	Epi
WDR901	6.3873E-89	4.54336479	0.218	0.036	9.8178E-85	Epi
GPM6B1	9.8968E-89	6.89104091	0.03	0.001	1.5212E-84	Epi

KANK14	8.8017E-88	4.33396717	0.058	0.003	1.3529E-83	Epi
AMOT2	8.9488E-88	4.92452273	0.081	0.006	1.3755E-83	Epi
CCL282	1.6655E-87	4.14919725	0.104	0.009	2.56E-83	Epi
NBEA1	1.9571E-87	4.42141874	0.228	0.039	3.0083E-83	Epi
GLS2	2.3824E-87	6.18988984	0.018	0	3.662E-83	Epi
GLIS2	1.47E-86	4.98233912	0.015	0	2.2596E-82	Epi
OSBPL74	2.2483E-86	3.70908763	0.076	0.005	3.4558E-82	Epi
FAM167B	5.1581E-86	4.61870546	0.033	0.001	7.9286E-82	Epi
PACSIN3	1.2592E-85	5.33478773	0.084	0.006	1.9355E-81	Epi
IL34	5.3167E-85	7.38962147	0.01	0	8.1723E-81	Epi
FAM177B1	5.3911E-85	6.22730629	0.01	0	8.2866E-81	Epi
CCNJL	5.4252E-85	4.80937951	0.01	0	8.3391E-81	Epi
RRAS21	7.5319E-85	3.1178475	0.15	0.018	1.1577E-80	Epi
ETNK2	1.9972E-84	5.70091102	0.033	0.001	3.0699E-80	Epi
TNK1	2.7472E-84	4.73087722	0.046	0.002	4.2227E-80	Epi
ZNF2732	1.5573E-83	4.14694338	0.198	0.031	2.3938E-79	Epi
IFT811	3.8781E-83	4.4776483	0.317	0.077	5.961E-79	Epi
KATNAL2	7.6672E-83	4.33827807	0.19	0.029	1.1785E-78	Epi
HSD17B13	1.0173E-82	5.90548302	0.076	0.005	1.5637E-78	Epi
OTUD7B2	1.1422E-82	3.74060242	0.147	0.018	1.7557E-78	Epi
FMN15	1.4406E-82	3.75682302	0.244	0.046	2.2144E-78	Epi
ZDHHC1	1.6125E-82	4.62500525	0.208	0.035	2.4786E-78	Epi
CXCL13	2.9408E-82	5.5635246	0.14	0.016	4.5202E-78	Epi
PDGFRL	5.0153E-82	6.85973503	0.02	0	7.709E-78	Epi
MSRB31	5.0805E-82	5.28387558	0.018	0	7.8093E-78	Epi
ARMC21	2.046E-81	4.40499771	0.239	0.045	3.1449E-77	Epi
KCNQ3	2.5858E-81	7.38565138	0.013	0	3.9746E-77	Epi
CTTNBP22	9.573E-81	6.00153312	0.02	0	1.4715E-76	Epi
SLC9A3R21	1.7093E-80	4.22016675	0.378	0.108	2.6274E-76	Epi
GPX81	2.8689E-80	5.57396204	0.061	0.003	4.4099E-76	Epi
RASAL13	1.7625E-79	4.54237523	0.02	0	2.7091E-75	Epi
CDS12	3.0304E-79	4.22204733	0.272	0.058	4.658E-75	Epi
TRPC11	3.047E-79	5.71945474	0.048	0.002	4.6836E-75	Epi
GRAMD2B1	4.0497E-79	3.91726251	0.259	0.053	6.2248E-75	Epi
FAM229B1	8.5088E-79	4.88976242	0.228	0.043	1.3079E-74	Epi
ATP9A1	9.8267E-79	3.54305197	0.327	0.083	1.5105E-74	Epi
HLF1	1.4898E-78	2.63186357	0.053	0.003	2.29E-74	Epi
GAS81	2.3074E-78	4.25729484	0.338	0.09	3.5467E-74	Epi
FBXO272	3.642E-78	4.93023234	0.041	0.002	5.5981E-74	Epi
MAP71	4.6591E-78	3.87986357	0.287	0.065	7.1614E-74	Epi
PRDM85	5.5479E-78	2.65242206	0.056	0.003	8.5277E-74	Epi
SPON21	6.0583E-78	4.07859443	0.036	0.001	9.3122E-74	Epi
CYSRT1	1.2978E-76	6.56503547	0.061	0.004	1.9949E-72	Epi
FAM161A2	1.3551E-76	4.63870687	0.124	0.014	2.0829E-72	Epi
CNFN1	2.0293E-76	8.27908393	0.066	0.004	3.1193E-72	Epi
STX19	3.4282E-76	5.91129777	0.038	0.001	5.2695E-72	Epi
INTU2	4.5603E-76	4.41397326	0.274	0.062	7.0097E-72	Epi
C11orf911	1.2321E-75	5.07158038	0.03	0.001	1.8939E-71	Epi
DCT	1.3828E-75	8.57091746	0.01	0	2.1255E-71	Epi
PARM13	1.3832E-75	3.35757295	0.046	0.002	2.1261E-71	Epi
UNC79	1.389E-75	7.72104857	0.01	0	2.135E-71	Epi
SDCBP22	2.0063E-75	5.59560776	0.127	0.015	3.0838E-71	Epi

RPGRIP1L1	2.4731E-75	4.32591751	0.249	0.052	3.8014E-71	Epi
RGL14	4.2058E-75	3.87173024	0.135	0.017	6.4647E-71	Epi
STOX2	7.3984E-75	4.67479211	0.081	0.006	1.1372E-70	Epi
FAM118A5	1.2555E-74	3.46629503	0.15	0.02	1.9298E-70	Epi
ESYT3	7.9955E-74	7.26144434	0.015	0	1.229E-69	Epi
KIF26B2	8.3679E-74	5.37864765	0.015	0	1.2862E-69	Epi
NUP62CL	2.8957E-73	4.85599878	0.089	0.008	4.4509E-69	Epi
LRRC61	3.53E-73	4.43885409	0.277	0.065	5.426E-69	Epi
LGALS9C	6.9585E-73	7.18908652	0.013	0	1.0696E-68	Epi
UGT81	7.1234E-73	5.85051781	0.013	0	1.0949E-68	Epi
MINDY42	1.0513E-71	4.5858894	0.145	0.02	1.616E-67	Epi
PLSCR41	1.7143E-71	5.18173023	0.079	0.006	2.6351E-67	Epi
TNKS1BP13	1.8296E-71	4.23021871	0.147	0.02	2.8122E-67	Epi
LRTOMT1	7.0163E-71	4.45738251	0.269	0.063	1.0785E-66	Epi
RND2	7.8467E-71	5.35281101	0.046	0.002	1.2061E-66	Epi
ARMC91	9.534E-71	4.19425974	0.208	0.039	1.4655E-66	Epi
CTNNAL12	1.0407E-70	3.64158408	0.246	0.053	1.5996E-66	Epi
PLEKHG61	1.0882E-70	4.27688432	0.132	0.017	1.6726E-66	Epi
RAPGEF51	2.1745E-70	4.63203337	0.023	0.001	3.3424E-66	Epi
IRF61	2.8557E-70	4.07385731	0.234	0.049	4.3895E-66	Epi
ST6GAL14	4.6084E-70	2.87024518	0.254	0.055	7.0835E-66	Epi
ATOH8	4.7285E-70	6.07157706	0.03	0.001	7.2682E-66	Epi
PCDHA4	6.1089E-70	6.1825944	0.02	0	9.39E-66	Epi
ABCA93	2.3222E-69	5.95393752	0.018	0	3.5695E-65	Epi
PHLDB32	4.443E-69	4.24484396	0.122	0.015	6.8293E-65	Epi
ASTN2	4.6691E-69	5.06819957	0.091	0.009	7.1769E-65	Epi
FAM107A	4.8747E-69	7.85484267	0.02	0	7.493E-65	Epi
IGF2BP23	5.4261E-69	4.43133728	0.168	0.027	8.3405E-65	Epi
CCDC401	1.4073E-68	4.65954939	0.325	0.093	2.1632E-64	Epi
VILL2	1.6171E-68	3.57018628	0.259	0.059	2.4856E-64	Epi
MPPED22	5.0231E-68	5.62100902	0.01	0	7.7211E-64	Epi
MARCKS3	1.0218E-67	2.99851931	0.317	0.085	1.5707E-63	Epi
FAM102A1	1.2584E-67	2.43568999	0.338	0.096	1.9343E-63	Epi
CIART2	4.4491E-67	5.36614949	0.033	0.001	6.8387E-63	Epi
TINCR	1.9292E-66	5.52875917	0.043	0.002	2.9653E-62	Epi
FOXD1	6.2972E-66	4.83783764	0.018	0	9.6795E-62	Epi
EVC21	8.2893E-66	2.95563094	0.036	0.001	1.2742E-61	Epi
ACHE2	1.0882E-65	4.91661594	0.015	0	1.6727E-61	Epi
TOM1L11	3.1758E-65	4.35406149	0.16	0.026	4.8815E-61	Epi
HES1	8.1891E-65	4.86989513	0.206	0.041	1.2587E-60	Epi
SOX6	8.3963E-65	7.49128487	0.01	0	1.2906E-60	Epi
STAP2	8.7068E-65	3.99537167	0.18	0.032	1.3383E-60	Epi
PYCR11	1.0442E-64	4.77463435	0.033	0.001	1.6051E-60	Epi
CCDC175	2.6728E-64	5.50554673	0.048	0.003	4.1084E-60	Epi
CDRT41	3.9568E-64	3.48944994	0.216	0.044	6.0819E-60	Epi
IZUMO43	9.6532E-64	4.12502558	0.086	0.008	1.4838E-59	Epi
CEMP1	3.9873E-63	6.08913345	0.02	0	6.1289E-59	Epi
SLC52A3	5.8904E-63	5.90026725	0.038	0.002	9.0541E-59	Epi
CCSER13	1.326E-62	3.55343697	0.046	0.003	2.0382E-58	Epi
STRBP3	1.3369E-62	3.41111388	0.365	0.12	2.0549E-58	Epi
DHRS91	2.4189E-62	3.76961782	0.398	0.146	3.7182E-58	Epi
CCDC1383	2.5258E-62	4.07080756	0.168	0.029	3.8823E-58	Epi

S100A121	3.0535E-62	6.25036272	0.03	0.001	4.6936E-58	Epi
GSDMC	3.7564E-62	6.39661634	0.036	0.002	5.774E-58	Epi
FAM83D	5.5581E-62	4.63741903	0.099	0.011	8.5433E-58	Epi
DYNC2H12	7.2211E-62	4.15006159	0.289	0.08	1.11E-57	Epi
CPAMD8	1.1923E-61	5.80584764	0.015	0	1.8326E-57	Epi
IGF1R4	1.4995E-61	3.36844696	0.302	0.085	2.3048E-57	Epi
TNFRSF252	3.2967E-61	1.88332079	0.081	0.008	5.0673E-57	Epi
EGFL82	5.4448E-61	4.66890932	0.091	0.01	8.3692E-57	Epi
TNFAIP8L33	9.4018E-61	4.90407688	0.033	0.001	1.4452E-56	Epi
BAIAP21	1.9837E-60	3.40348325	0.444	0.182	3.0491E-56	Epi
ZNF497	1.0741E-59	5.53325429	0.058	0.004	1.651E-55	Epi
KLHDC92	2.748E-59	4.13469838	0.165	0.029	4.224E-55	Epi
IGSF11	3.5062E-59	7.15389246	0.01	0	5.3893E-55	Epi
TMEM441	3.8751E-59	3.62418438	0.15	0.024	5.9564E-55	Epi
TMEM92	6.6746E-59	6.40652989	0.023	0.001	1.026E-54	Epi
ADHFE13	2.2471E-58	3.19045802	0.119	0.016	3.4539E-54	Epi
OSCP11	2.5901E-58	4.04784955	0.272	0.074	3.9813E-54	Epi
MCC3	3.3545E-58	3.81032663	0.041	0.002	5.1562E-54	Epi
HEY1	4.2114E-58	5.13262278	0.069	0.006	6.4733E-54	Epi
SIX5	4.4985E-58	4.9417055	0.066	0.005	6.9147E-54	Epi
SPAG11	7.7138E-58	3.78239194	0.289	0.083	1.1857E-53	Epi
TSPAN12	9.8567E-58	5.59705976	0.053	0.004	1.5151E-53	Epi
SNED11	1.6781E-57	4.47395912	0.14	0.022	2.5794E-53	Epi
ANKRD351	2.3514E-57	4.68314075	0.102	0.012	3.6143E-53	Epi
ISYNA14	2.8126E-57	4.18008967	0.063	0.005	4.3232E-53	Epi
C2CD4D	4.8874E-57	3.99254177	0.013	0	7.5124E-53	Epi
FLNB2	2.8E-56	3.51412767	0.371	0.133	4.3039E-52	Epi
EDARADD2	5.4994E-56	3.70004697	0.038	0.002	8.4532E-52	Epi
PLS32	1.519E-55	4.96795057	0.099	0.012	2.3348E-51	Epi
PBX41	2.082E-55	2.50201901	0.084	0.009	3.2002E-51	Epi
TPM23	2.1001E-55	3.85805991	0.168	0.031	3.228E-51	Epi
SPACA9	3.5258E-55	4.09555704	0.183	0.037	5.4196E-51	Epi
IL1RL23	1.3278E-54	5.08318827	0.028	0.001	2.041E-50	Epi
SAMD121	1.3322E-54	3.84640972	0.208	0.047	2.0477E-50	Epi
GLIS32	1.4408E-54	3.7764556	0.211	0.048	2.2146E-50	Epi
USP542	6.5973E-54	3.54871979	0.223	0.054	1.0141E-49	Epi
LAPTM4B1	9.0085E-54	4.08855035	0.099	0.012	1.3847E-49	Epi
PPP1R3C	1.8893E-53	6.84886375	0.038	0.002	2.904E-49	Epi
CLUAP11	2.5444E-53	3.98003642	0.381	0.148	3.911E-49	Epi
PODXL1	6.393E-53	4.20758264	0.14	0.024	9.8266E-49	Epi
XDH	6.7188E-53	7.00253382	0.015	0	1.0327E-48	Epi
ESAM1	6.7708E-53	5.17958962	0.036	0.002	1.0407E-48	Epi
SOX9	9.2912E-53	6.90070383	0.013	0	1.4281E-48	Epi
PPP1R361	1.2986E-52	4.1920948	0.096	0.012	1.9961E-48	Epi
RAB3IP2	2.7573E-52	3.08401259	0.261	0.073	4.2382E-48	Epi
SATB15	3.3764E-52	2.52258631	0.305	0.094	5.1899E-48	Epi
ZNF4402	3.6835E-52	3.8517819	0.261	0.074	5.6619E-48	Epi
ATP8B11	3.7529E-52	3.51233571	0.195	0.043	5.7686E-48	Epi
EMB4	4.5279E-52	1.09657568	0.14	0.023	6.9599E-48	Epi
CAV11	5.4971E-52	7.37419799	0.015	0	8.4496E-48	Epi
SERPINI21	9.7212E-52	3.78337092	0.299	0.096	1.4942E-47	Epi
ST8SIA5	1.0127E-51	6.5059098	0.02	0.001	1.5566E-47	Epi

IGFBP42	1.1456E-51	3.15017722	0.046	0.003	1.761E-47	Epi
TMEM671	2.7544E-51	3.8733406	0.289	0.092	4.2338E-47	Epi
RTN4RL1	9.9669E-51	4.53669786	0.028	0.001	1.532E-46	Epi
PIK3R31	1.2106E-50	3.56104162	0.193	0.043	1.8609E-46	Epi
ALG1L	1.4644E-50	5.03157903	0.071	0.007	2.2509E-46	Epi
AHDC13	2.9219E-50	3.18921814	0.223	0.057	4.4912E-46	Epi
DNAH13	3.9495E-50	3.58228816	0.279	0.086	6.0707E-46	Epi
FAM13A3	5.809E-50	3.98820353	0.345	0.129	8.929E-46	Epi
LRATD21	6.8533E-50	3.26818714	0.17	0.035	1.0534E-45	Epi
CLBA12	1.4328E-49	3.87910455	0.188	0.042	2.2023E-45	Epi
MT-CYB5	1.4336E-49	3.55415433	0.475	0.232	2.2035E-45	Epi
AFAP11	1.8682E-49	2.92951913	0.079	0.009	2.8716E-45	Epi
USP23	1.9079E-49	3.96489895	0.175	0.038	2.9326E-45	Epi
REC83	1.9513E-49	3.3771167	0.31	0.103	2.9993E-45	Epi
PDZD4	2.4015E-49	3.376252	0.015	0	3.6913E-45	Epi
ADCY6	2.6979E-49	4.0890244	0.155	0.03	4.1469E-45	Epi
TRERF14	3.3859E-49	2.49988842	0.102	0.014	5.2045E-45	Epi
DLG52	4.5818E-49	3.63995906	0.208	0.051	7.0427E-45	Epi
RTKN3	5.4094E-49	4.44267728	0.056	0.005	8.3148E-45	Epi
HOPX1	5.4781E-49	4.14576075	0.104	0.015	8.4204E-45	Epi
PRSS27	9.1219E-49	8.09997627	0.071	0.007	1.4021E-44	Epi
MTURN3	1.0101E-48	3.37538486	0.241	0.066	1.5527E-44	Epi
FBLN72	1.043E-48	2.8561158	0.025	0.001	1.6032E-44	Epi
WEE1	1.636E-48	2.94416998	0.173	0.036	2.5147E-44	Epi
UBXN101	1.9003E-48	4.22570401	0.355	0.139	2.921E-44	Epi
HIST3H2A4	2.214E-48	4.52394267	0.124	0.021	3.4031E-44	Epi
PPOX1	2.6507E-48	3.65357205	0.315	0.11	4.0745E-44	Epi
SLFN13	2.8421E-48	3.11423068	0.122	0.02	4.3686E-44	Epi
TMEM45A1	6.8693E-48	6.12211462	0.053	0.004	1.0559E-43	Epi
MAGIX1	8.1569E-48	3.49991324	0.183	0.041	1.2538E-43	Epi
ZBED21	9.419E-48	3.0365718	0.015	0	1.4478E-43	Epi
TENT5C2	1.0367E-47	2.61967563	0.142	0.026	1.5935E-43	Epi
EFCAB72	1.0582E-47	3.56572086	0.175	0.038	1.6266E-43	Epi
FERMT21	1.2838E-47	5.89441591	0.03	0.001	1.9734E-43	Epi
TINAGL1	1.2963E-47	6.18870172	0.058	0.005	1.9925E-43	Epi
SLC6A81	1.4499E-47	4.30089047	0.063	0.006	2.2286E-43	Epi
CCDC1363	1.8628E-47	2.65763271	0.03	0.001	2.8633E-43	Epi
SFTPB	1.9438E-47	11.045197	0.02	0.001	2.9878E-43	Epi
GEM3	2.1969E-47	4.76338929	0.02	0.001	3.3768E-43	Epi
C19orf18	4.2865E-47	4.73972282	0.071	0.008	6.5887E-43	Epi
ITPR31	6.4062E-47	2.68170662	0.239	0.066	9.8469E-43	Epi
ANGPTL4	7.5329E-47	7.44475152	0.013	0	1.1579E-42	Epi
PTPN211	8.7036E-47	4.61566159	0.063	0.006	1.3378E-42	Epi
TXLNB1	9.5446E-47	3.97073801	0.129	0.023	1.4671E-42	Epi
CCDC1912	1.0457E-46	3.77068125	0.195	0.047	1.6074E-42	Epi
RFX33	1.7791E-46	4.04646627	0.35	0.138	2.7346E-42	Epi
ODF2L1	2.9405E-46	3.40099746	0.152	0.03	4.5198E-42	Epi
RIC31	2.9478E-46	3.5697395	0.165	0.035	4.531E-42	Epi
TRIM73	3.6201E-46	4.0271388	0.048	0.004	5.5644E-42	Epi
IFT1401	4.3457E-46	3.73475204	0.279	0.091	6.6797E-42	Epi
BASP15	5.1177E-46	3.27784594	0.254	0.075	7.8664E-42	Epi
STXBP12	8.6431E-46	3.2908531	0.183	0.042	1.3285E-41	Epi

ATP2A35	9.4819E-46	1.95864239	0.216	0.055	1.4575E-41	Epi
KCTD16	1.0318E-45	4.27215558	0.091	0.012	1.586E-41	Epi
PDE4C	1.3086E-45	6.56995781	0.018	0.001	2.0114E-41	Epi
ADAMTS17	1.3792E-45	4.78149818	0.018	0.001	2.12E-41	Epi
UNC119B2	1.5604E-45	3.50653614	0.241	0.07	2.3985E-41	Epi
ZNF454	2.584E-45	6.18294592	0.02	0.001	3.9719E-41	Epi
TTC122	2.6252E-45	3.3914427	0.307	0.109	4.0352E-41	Epi
HDC	7.3201E-45	1.934965	0.015	0	1.1252E-40	Epi
TNFRSF11A3	9.4948E-45	4.22179426	0.028	0.001	1.4594E-40	Epi
TNC	1.3688E-44	6.32164818	0.018	0.001	2.104E-40	Epi
MROH73	2.9381E-44	4.51780581	0.063	0.006	4.5161E-40	Epi
CAVIN11	3.9942E-44	6.22456195	0.02	0.001	6.1395E-40	Epi
SPATA241	4.691E-44	3.833427	0.122	0.021	7.2105E-40	Epi
AFAP1L1	5.1715E-44	4.6828747	0.063	0.007	7.9491E-40	Epi
OSR2	6.3306E-44	6.03685682	0.01	0	9.7308E-40	Epi
DZANK12	6.927E-44	3.67705855	0.236	0.07	1.0647E-39	Epi
CCDC1531	7.745E-44	3.69508623	0.241	0.072	1.1905E-39	Epi
CD200R14	9.7227E-44	1.58398072	0.061	0.006	1.4945E-39	Epi
CES4A1	1.0331E-43	3.9175703	0.343	0.137	1.588E-39	Epi
FSIP1	1.4207E-43	4.77548337	0.053	0.005	2.1838E-39	Epi
SEC14L5	1.4234E-43	6.12458886	0.018	0.001	2.1879E-39	Epi
WASF31	1.4303E-43	5.77929991	0.015	0	2.1986E-39	Epi
GPC11	2.0174E-43	3.75717661	0.211	0.057	3.1009E-39	Epi
SYNGAP12	2.8046E-43	3.74115926	0.33	0.128	4.311E-39	Epi
PXYLP13	3.1392E-43	2.88787869	0.023	0.001	4.8253E-39	Epi
SRCIN1	3.8363E-43	4.19154308	0.132	0.025	5.8968E-39	Epi
CITED4	5.0055E-43	3.26756422	0.17	0.039	7.6939E-39	Epi
FAM221A2	7.8999E-43	3.30854683	0.345	0.137	1.2143E-38	Epi
MT-ATP66	8.934E-43	3.13626498	0.642	0.459	1.3732E-38	Epi
LRRC37A33	9.8797E-43	4.43493076	0.079	0.01	1.5186E-38	Epi
C20orf961	1.1818E-42	3.75246132	0.297	0.106	1.8165E-38	Epi
MATN2	1.2694E-42	5.47233497	0.02	0.001	1.9513E-38	Epi
OIT3	1.4893E-42	6.1102592	0.01	0	2.2893E-38	Epi
CABYR	1.9037E-42	4.32971648	0.03	0.002	2.9261E-38	Epi
C2orf81	1.9449E-42	4.04285416	0.15	0.032	2.9895E-38	Epi
SLC22A43	5.0379E-42	3.53713565	0.292	0.104	7.7437E-38	Epi
AL160269.11	5.7153E-42	4.77238534	0.056	0.005	8.7849E-38	Epi
ADSSL11	8.1571E-42	4.09180487	0.119	0.021	1.2538E-37	Epi
KIAA15222	9.851E-42	3.04272487	0.553	0.368	1.5142E-37	Epi
NEK113	1.1571E-41	3.8513933	0.266	0.091	1.7786E-37	Epi
TNFAIP8L11	1.3758E-41	3.71820403	0.221	0.064	2.1147E-37	Epi
IQCN4	1.4258E-41	2.74689888	0.132	0.025	2.1916E-37	Epi
JAG21	1.6309E-41	3.01762018	0.096	0.015	2.5068E-37	Epi
F2RL12	1.9659E-41	4.02272673	0.051	0.005	3.0217E-37	Epi
UMODL1	2.1609E-41	5.87840469	0.043	0.003	3.3215E-37	Epi
LBH1	2.2906E-41	1.81918353	0.14	0.028	3.5209E-37	Epi
AGBL3	4.9719E-41	5.12329525	0.023	0.001	7.6423E-37	Epi
SSBP23	5.3623E-41	3.13328471	0.165	0.038	8.2424E-37	Epi
LMTK3	5.3631E-41	4.35911561	0.069	0.008	8.2436E-37	Epi
PLOD2	2.0099E-40	5.61860092	0.046	0.004	3.0894E-36	Epi
SLC4A111	2.3589E-40	3.6111477	0.216	0.062	3.6258E-36	Epi
P2RX6	2.3764E-40	4.57214864	0.03	0.002	3.6527E-36	Epi

SYNJ24	3.1106E-40	3.342988	0.071	0.009	4.7814E-36	Epi
WDR541	5.0429E-40	3.75797402	0.203	0.057	7.7514E-36	Epi
MT-ND55	5.0873E-40	2.9693869	0.503	0.278	7.8197E-36	Epi
CCDC241	5.61E-40	3.67897807	0.256	0.084	8.6231E-36	Epi
GDF151	9.1838E-40	4.1457999	0.162	0.038	1.4116E-35	Epi
GMNC	1.0153E-39	5.33926349	0.028	0.002	1.5607E-35	Epi
C17orf972	1.69E-39	3.56446422	0.239	0.075	2.5978E-35	Epi
MT-CO35	1.8203E-39	2.85627005	0.678	0.523	2.798E-35	Epi
WFDC3	4.0527E-39	4.46975596	0.036	0.002	6.2295E-35	Epi
TSPAN23	5.1922E-39	1.8381581	0.046	0.004	7.981E-35	Epi
TRPV61	5.2141E-39	5.89691752	0.02	0.001	8.0146E-35	Epi
MIA4	6.1573E-39	3.45878733	0.053	0.005	9.4644E-35	Epi
KIF272	6.2791E-39	3.17937405	0.226	0.069	9.6515E-35	Epi
S100A92	6.4144E-39	0.88841344	0.64	0.95	9.8596E-35	Epi
BICDL12	6.4163E-39	2.70331061	0.112	0.02	9.8624E-35	Epi
CYP2E11	9.739E-39	4.37134396	0.056	0.006	1.497E-34	Epi
TMCC22	1.1265E-38	4.17924989	0.048	0.004	1.7316E-34	Epi
EFCAB21	1.3178E-38	3.58457784	0.228	0.071	2.0256E-34	Epi
ARHGEF16	2.1202E-38	4.54306209	0.091	0.014	3.259E-34	Epi
KIAA08252	2.1491E-38	3.04089351	0.249	0.082	3.3034E-34	Epi
IFT801	2.6104E-38	3.02873387	0.277	0.1	4.0124E-34	Epi
MT-CO25	2.8707E-38	2.92378602	0.624	0.45	4.4126E-34	Epi
MICALL2	3.9434E-38	3.54071869	0.256	0.087	6.0613E-34	Epi
ISG205	6.9787E-38	1.78459439	0.185	0.048	1.0727E-33	Epi
ADGRG63	7.1375E-38	4.98794477	0.03	0.002	1.0971E-33	Epi
AC008397.1	7.8175E-38	6.60807359	0.01	0	1.2016E-33	Epi
C16orf74	7.9313E-38	5.59364565	0.01	0	1.2191E-33	Epi
ADPRHL1	1.2022E-37	4.46529309	0.053	0.005	1.8479E-33	Epi
KIF91	2.1644E-37	3.63417629	0.297	0.115	3.3269E-33	Epi
EPS8L11	2.6567E-37	4.6767857	0.315	0.125	4.0836E-33	Epi
CYB5611	4.6523E-37	2.88424622	0.327	0.136	7.151E-33	Epi
SULF24	5.5072E-37	2.85054104	0.051	0.005	8.4651E-33	Epi
AQP11	5.5503E-37	3.14786282	0.048	0.005	8.5313E-33	Epi
HAGHL2	6.3856E-37	3.80614503	0.353	0.16	9.8153E-33	Epi
TMEM95	8.2302E-37	6.42658483	0.01	0	1.2651E-32	Epi
BTNL2	8.2346E-37	6.68170432	0.01	0	1.2657E-32	Epi
MT-ND34	1.2485E-36	3.21801537	0.599	0.44	1.9191E-32	Epi
ECM12	2.1412E-36	9.21542786	0.086	0.013	3.2912E-32	Epi
C6orf52	3.1478E-36	4.48589042	0.033	0.002	4.8384E-32	Epi
WTIP	4.5156E-36	4.20449699	0.058	0.007	6.9409E-32	Epi
NSUN72	4.6838E-36	3.66253785	0.358	0.166	7.1995E-32	Epi
SLC6A161	5.5361E-36	3.73375346	0.084	0.013	8.5095E-32	Epi
OMP	7.714E-36	6.41953832	0.01	0	1.1857E-31	Epi
P2RY22	1.0218E-35	4.94419193	0.061	0.007	1.5706E-31	Epi
CTSE	1.0806E-35	7.8371271	0.02	0.001	1.661E-31	Epi
SPSB11	1.1412E-35	3.23800394	0.218	0.068	1.7542E-31	Epi
PLD61	1.2484E-35	3.39556268	0.102	0.018	1.9189E-31	Epi
ST8SIA46	1.3104E-35	1.85621762	0.249	0.083	2.0142E-31	Epi
COLQ	1.3196E-35	3.92422183	0.043	0.004	2.0284E-31	Epi
RALGPS13	1.3668E-35	2.57754561	0.264	0.093	2.1009E-31	Epi
MRNIP2	1.911E-35	2.86461381	0.343	0.152	2.9375E-31	Epi
TSPAN51	2.4364E-35	2.30943245	0.028	0.002	3.7451E-31	Epi

EPHA11	6.4812E-35	1.63159667	0.043	0.004	9.9623E-31	Epi
PPP1R16B3	7.6413E-35	1.36192293	0.063	0.008	1.1746E-30	Epi
GDPD33	1.3257E-34	3.71743031	0.066	0.009	2.0377E-30	Epi
MAP3K91	2.0323E-34	3.5372283	0.099	0.018	3.1238E-30	Epi
ZMYND122	2.5273E-34	3.83405167	0.114	0.023	3.8847E-30	Epi
MT-ND45	2.9126E-34	2.9835484	0.576	0.419	4.4769E-30	Epi
LRRC341	2.9414E-34	3.7363519	0.18	0.051	4.5212E-30	Epi
CEL	3.037E-34	5.71462838	0.03	0.002	4.6682E-30	Epi
C1orf1151	3.3608E-34	5.89892888	0.02	0.001	5.166E-30	Epi
IFT172	4.7829E-34	3.42473253	0.376	0.19	7.3518E-30	Epi
MSI2	4.945E-34	2.70006436	0.386	0.189	7.601E-30	Epi
LAMP33	6.1533E-34	3.08943739	0.056	0.006	9.4582E-30	Epi
TJP11	6.3804E-34	3.17738591	0.294	0.118	9.8074E-30	Epi
KLHL231	8.0928E-34	4.08099964	0.036	0.003	1.2439E-29	Epi
ABCB62	9.6148E-34	2.7626495	0.129	0.029	1.4779E-29	Epi
CEP834	9.8709E-34	3.29522657	0.363	0.175	1.5172E-29	Epi
TMEM1546	1.8217E-33	2.64857913	0.145	0.035	2.8001E-29	Epi
ELMO33	1.9403E-33	3.21185699	0.109	0.022	2.9824E-29	Epi
WDR191	3.028E-33	3.29848757	0.239	0.084	4.6543E-29	Epi
IRS1	3.2967E-33	4.41979038	0.01	0	5.0674E-29	Epi
DCST2	3.4178E-33	4.54123106	0.033	0.002	5.2534E-29	Epi
PLEKHA62	3.8111E-33	3.20568414	0.18	0.051	5.858E-29	Epi
L3MBTL11	4.4723E-33	3.14516236	0.165	0.044	6.8744E-29	Epi
SYTL13	7.647E-33	2.38410245	0.388	0.195	1.1754E-28	Epi
ZPBP	7.9286E-33	5.49638625	0.013	0	1.2187E-28	Epi
HOOK22	1.4882E-32	3.16977312	0.193	0.057	2.2875E-28	Epi
JUP2	1.6403E-32	3.08303819	0.36	0.172	2.5213E-28	Epi
ACPP4	1.6947E-32	3.46953978	0.046	0.005	2.605E-28	Epi
CYP2C8	1.9104E-32	4.54136832	0.025	0.002	2.9365E-28	Epi
DPYSL33	2.0854E-32	3.95172744	0.048	0.005	3.2055E-28	Epi
NOL32	2.3893E-32	3.34095049	0.061	0.008	3.6726E-28	Epi
SSC5D	2.5838E-32	5.85132485	0.013	0	3.9715E-28	Epi
ANKRD31	2.8604E-32	4.84131028	0.023	0.001	4.3967E-28	Epi
AK92	3.5106E-32	3.37587844	0.289	0.12	5.3961E-28	Epi
PABPC1L3	4.0158E-32	2.73694022	0.279	0.11	6.1727E-28	Epi
C11orf652	4.1084E-32	4.38687619	0.074	0.011	6.315E-28	Epi
DZIP32	4.5411E-32	3.07040971	0.228	0.079	6.9801E-28	Epi
MTUS12	9.2237E-32	3.17289563	0.254	0.095	1.4178E-27	Epi
MT-ND4L6	1.0806E-31	3.06472821	0.48	0.289	1.6609E-27	Epi
ACY32	1.1496E-31	4.30011479	0.01	0	1.7671E-27	Epi
MT-ND26	2.5603E-31	2.9430867	0.409	0.214	3.9354E-27	Epi
GPR1352	4.3427E-31	3.26424139	0.124	0.028	6.6751E-27	Epi
ETV1	4.634E-31	2.42592506	0.015	0.001	7.1229E-27	Epi
COL5A2	5.8939E-31	5.31643697	0.028	0.002	9.0595E-27	Epi
C9orf43	7.1756E-31	4.71831522	0.043	0.004	1.103E-26	Epi
MTCL11	7.7856E-31	4.17269628	0.018	0.001	1.1967E-26	Epi
CLHC11	9.8791E-31	3.34866116	0.165	0.047	1.5185E-26	Epi
TDRD12	1.6418E-30	5.25390882	0.023	0.001	2.5236E-26	Epi
SFTPA1	1.8342E-30	8.39067144	0.015	0.001	2.8193E-26	Epi
IQCK2	1.8459E-30	3.26771386	0.269	0.11	2.8373E-26	Epi
IL12A	1.9167E-30	5.45624266	0.025	0.002	2.9462E-26	Epi
WDR272	2.5021E-30	3.06088294	0.208	0.069	3.846E-26	Epi

TMEM25	3.4135E-30	4.65082518	0.028	0.002	5.2469E-26	Epi
P4HTM1	9.8814E-30	2.99541891	0.388	0.212	1.5189E-25	Epi
KIF171	1.1235E-29	4.18981409	0.079	0.013	1.727E-25	Epi
LGI4	1.4951E-29	5.45411629	0.013	0	2.2981E-25	Epi
WDR603	2.3121E-29	3.05230373	0.363	0.19	3.5539E-25	Epi
WDR34	3.7968E-29	2.85714927	0.299	0.131	5.8361E-25	Epi
SLC25A291	4.189E-29	3.25811686	0.261	0.105	6.4389E-25	Epi
ZNF6062	5.306E-29	3.11979116	0.282	0.12	8.1559E-25	Epi
NYNRIN2	5.578E-29	2.90733357	0.188	0.06	8.5739E-25	Epi
CYB5D12	6.1909E-29	2.95215593	0.175	0.053	9.516E-25	Epi
CEP1121	9.9244E-29	3.16943429	0.086	0.016	1.5255E-24	Epi
MAP2	1.1127E-28	4.82405063	0.033	0.003	1.7103E-24	Epi
LIF1	1.8658E-28	3.69283889	0.028	0.002	2.8679E-24	Epi
SYT173	2.0738E-28	3.53684306	0.104	0.022	3.1876E-24	Epi
HNF1B	2.2208E-28	7.57107277	0.01	0	3.4135E-24	Epi
ITGA31	2.4583E-28	2.70283587	0.292	0.127	3.7787E-24	Epi
NUCB21	2.5878E-28	2.85884108	0.523	0.377	3.9778E-24	Epi
SCARF21	3.2949E-28	3.77821497	0.056	0.008	5.0646E-24	Epi
CRISPLD21	3.9457E-28	3.78436538	0.02	0.001	6.065E-24	Epi
CADM42	5.3901E-28	3.3649934	0.076	0.013	8.2851E-24	Epi
KRTAP5-9	5.8921E-28	4.16963516	0.025	0.002	9.0567E-24	Epi
WDR97	6.0328E-28	3.99484848	0.051	0.006	9.273E-24	Epi
DAPK23	7.0982E-28	2.05306573	0.046	0.005	1.0911E-23	Epi
CERS3	8.1202E-28	5.49639361	0.015	0.001	1.2482E-23	Epi
ECE15	8.1955E-28	3.09080281	0.277	0.117	1.2597E-23	Epi
FGF17	8.661E-28	6.1392316	0.01	0	1.3313E-23	Epi
ADM1	8.6981E-28	5.39480406	0.053	0.007	1.337E-23	Epi
FBXL21	1.7387E-27	3.27585599	0.145	0.04	2.6726E-23	Epi
TLCD11	1.882E-27	3.30279271	0.104	0.023	2.8929E-23	Epi
CCDC572	2.9412E-27	3.19433799	0.241	0.095	4.5209E-23	Epi
PLEKHH2	2.9568E-27	4.07627187	0.043	0.005	4.5449E-23	Epi
PPM1H1	5.5183E-27	2.85996995	0.261	0.109	8.4822E-23	Epi
CSPP11	6.1691E-27	3.33904918	0.292	0.137	9.4825E-23	Epi
EPS83	8.2917E-27	3.44566304	0.162	0.05	1.2745E-22	Epi
TRIB22	1.1277E-26	1.48586175	0.124	0.031	1.7333E-22	Epi
CYP7B1	1.3085E-26	5.04095598	0.033	0.003	2.0113E-22	Epi
CHKA3	1.3975E-26	2.6665369	0.231	0.088	2.1481E-22	Epi
RNF32	1.7014E-26	4.36097228	0.038	0.004	2.6152E-22	Epi
GPR156	1.977E-26	4.56972825	0.025	0.002	3.0388E-22	Epi
WDR312	2.1969E-26	4.14583308	0.081	0.015	3.3768E-22	Epi
TRAF3IP1	2.2804E-26	3.23984393	0.378	0.22	3.5052E-22	Epi
LGR42	2.7348E-26	3.02501229	0.147	0.042	4.2037E-22	Epi
CUBN2	5.0555E-26	3.47419276	0.053	0.007	7.7708E-22	Epi
GAS65	5.686E-26	3.05807905	0.079	0.015	8.74E-22	Epi
NDFIP21	5.6983E-26	2.65979377	0.307	0.145	8.7588E-22	Epi
IL1RN3	5.9261E-26	1.07648905	0.348	0.698	9.109E-22	Epi
MARCKSL15	6.9555E-26	2.91501739	0.117	0.029	1.0691E-21	Epi
LIPE1	7.5616E-26	2.61195252	0.02	0.001	1.1623E-21	Epi
S100P1	1.2843E-25	4.34419614	0.401	0.233	1.9741E-21	Epi
ARG2	1.3181E-25	4.57250593	0.038	0.004	2.0261E-21	Epi
GPLD1	1.3239E-25	4.51291896	0.02	0.001	2.0349E-21	Epi
POLN	1.4009E-25	4.42485032	0.036	0.004	2.1534E-21	Epi

ANO1	1.426E-25	4.23111737	0.025	0.002	2.1918E-21	Epi
FAM83H2	1.556E-25	2.49447483	0.33	0.165	2.3917E-21	Epi
OVGP13	2.4645E-25	4.17501559	0.03	0.003	3.7883E-21	Epi
CHD36	2.7885E-25	1.82305307	0.261	0.107	4.2862E-21	Epi
DNAJA41	3.1757E-25	3.02611833	0.596	0.52	4.8814E-21	Epi
ANKMY12	3.7776E-25	2.71630551	0.332	0.171	5.8066E-21	Epi
HSD17B6	4.5904E-25	4.97268109	0.02	0.001	7.0559E-21	Epi
ACKR2	5.2241E-25	3.52563133	0.043	0.005	8.0299E-21	Epi
IQCG3	6.1352E-25	3.37818559	0.406	0.255	9.4304E-21	Epi
PLEKHF11	6.2079E-25	1.83420169	0.069	0.012	9.5421E-21	Epi
INMT	6.275E-25	5.191055	0.015	0.001	9.6453E-21	Epi
ARHGAP322	6.8928E-25	2.82289567	0.218	0.084	1.0595E-20	Epi
AFDN4	7.5393E-25	2.30365184	0.596	0.525	1.1589E-20	Epi
MPP7	8.4469E-25	2.33542971	0.226	0.089	1.2984E-20	Epi
STPG2	8.9016E-25	4.00750654	0.053	0.008	1.3683E-20	Epi
TMEM63C	1.1656E-24	4.50805875	0.028	0.002	1.7916E-20	Epi
WDR352	1.3116E-24	2.78363416	0.165	0.053	2.016E-20	Epi
CRABP21	1.4407E-24	3.91133793	0.048	0.007	2.2144E-20	Epi
CSTA2	1.5036E-24	0.21884708	0.492	0.878	2.3112E-20	Epi
RADX	1.7122E-24	3.22268096	0.061	0.01	2.6319E-20	Epi
DNMBP1	2.0451E-24	2.72415946	0.234	0.095	3.1435E-20	Epi
ANKRD531	2.7793E-24	4.70879273	0.015	0.001	4.2721E-20	Epi
ULK42	3.3268E-24	3.30792477	0.36	0.21	5.1137E-20	Epi
NRARP3	3.5597E-24	4.66059532	0.079	0.016	5.4716E-20	Epi
NRSN22	4.1743E-24	4.40000775	0.051	0.007	6.4163E-20	Epi
FRMD6	4.8278E-24	5.13740644	0.033	0.003	7.4207E-20	Epi
ABHD17C1	6.0864E-24	3.16606221	0.168	0.056	9.3554E-20	Epi
CACNB41	6.5473E-24	4.12427087	0.025	0.002	1.0064E-19	Epi
HYKK1	6.7775E-24	3.56714104	0.061	0.01	1.0418E-19	Epi
MERTK2	8.2388E-24	3.50196382	0.036	0.004	1.2664E-19	Epi
DOCK61	1.1185E-23	3.11457041	0.096	0.022	1.7192E-19	Epi
PLXNB24	1.1631E-23	1.26961381	0.787	0.898	1.7879E-19	Epi
ELF51	1.4662E-23	3.12232509	0.094	0.021	2.2537E-19	Epi
STK361	1.7579E-23	2.97688949	0.216	0.086	2.7021E-19	Epi
PGAP13	2.0042E-23	2.30876112	0.071	0.013	3.0807E-19	Epi
RBFOX23	2.0224E-23	2.84235982	0.127	0.035	3.1086E-19	Epi
STX1A2	2.4756E-23	2.82381147	0.053	0.008	3.8053E-19	Epi
CENPM	2.5186E-23	1.67971072	0.178	0.06	3.8713E-19	Epi
ZC3H12C4	2.5275E-23	3.14119324	0.069	0.013	3.885E-19	Epi
LRP111	2.6291E-23	2.94272707	0.378	0.229	4.0411E-19	Epi
PPP1R16A1	4.9197E-23	3.24707753	0.322	0.177	7.562E-19	Epi
PRKCZ1	5.271E-23	2.51052739	0.216	0.087	8.1021E-19	Epi
LRG11	5.9757E-23	3.26698278	0.175	0.061	9.1852E-19	Epi
MAP1LC3A1	6.1969E-23	2.78936368	0.277	0.131	9.5252E-19	Epi
HCAR1	6.9592E-23	6.23879581	0.018	0.001	1.0697E-18	Epi
SLC7A113	7.5732E-23	3.87829103	0.023	0.002	1.1641E-18	Epi
KIF24	8.0225E-23	3.42538706	0.091	0.021	1.2331E-18	Epi
KLF15	8.7397E-23	4.08713233	0.043	0.006	1.3434E-18	Epi
PLEKHA73	9.8225E-23	2.57441205	0.261	0.119	1.5098E-18	Epi
POLQ	1.1089E-22	2.84869814	0.112	0.029	1.7045E-18	Epi
DENND2C2	1.3691E-22	3.29080901	0.096	0.023	2.1044E-18	Epi
ARVCF1	1.3974E-22	3.25201452	0.122	0.034	2.148E-18	Epi

C32	1.4371E-22	4.47948692	0.272	0.129	2.209E-18	Epi
MT-ND16	1.4639E-22	2.58428716	0.368	0.212	2.2501E-18	Epi
TM7SF21	1.5899E-22	2.63112717	0.206	0.079	2.4438E-18	Epi
ZNF7933	1.7875E-22	2.74975488	0.036	0.004	2.7475E-18	Epi
FAM169A3	1.9632E-22	3.31044637	0.038	0.005	3.0176E-18	Epi
SPATA332	2.2186E-22	2.74507886	0.259	0.119	3.4102E-18	Epi
P3H3	2.5428E-22	5.31849974	0.01	0	3.9086E-18	Epi
MED12L1	2.6113E-22	3.72103907	0.01	0	4.0139E-18	Epi
CASZ12	2.7266E-22	2.41703152	0.353	0.202	4.191E-18	Epi
RND11	3.9342E-22	4.94813812	0.025	0.002	6.0473E-18	Epi
DNAL12	4.5045E-22	2.96260461	0.241	0.107	6.9238E-18	Epi
RALGPS22	4.6879E-22	3.06897562	0.145	0.046	7.2058E-18	Epi
PID12	6.3951E-22	3.65476452	0.02	0.001	9.8299E-18	Epi
GOLM1	7.7454E-22	2.64691012	0.416	0.268	1.1905E-17	Epi
CHIC12	8.4391E-22	2.58926708	0.061	0.011	1.2972E-17	Epi
KRTAP5-10	1.3348E-21	6.60945156	0.01	0	2.0517E-17	Epi
GLCCI13	1.3584E-21	2.14292952	0.175	0.063	2.0881E-17	Epi
ICA1L1	1.7318E-21	2.89738724	0.185	0.07	2.662E-17	Epi
PTCH2	1.805E-21	4.19228321	0.028	0.003	2.7744E-17	Epi
NEK33	1.8929E-21	2.88644499	0.091	0.022	2.9096E-17	Epi
ARHGAP23	2.1336E-21	3.14825191	0.112	0.03	3.2795E-17	Epi
PNLDC1	2.1798E-21	4.77211332	0.025	0.002	3.3505E-17	Epi
SLC18B14	2.2284E-21	2.86502503	0.135	0.042	3.4253E-17	Epi
MYORG2	2.3283E-21	3.01142225	0.099	0.025	3.5789E-17	Epi
NEXMIF	2.8389E-21	5.93684256	0.01	0	4.3637E-17	Epi
CRACR2A2	3.7388E-21	2.73319838	0.193	0.075	5.7469E-17	Epi
PLCB12	3.8711E-21	2.64617319	0.025	0.002	5.9502E-17	Epi
ENKD11	3.9506E-21	2.74105622	0.388	0.25	6.0724E-17	Epi
SLC6A13	4.1586E-21	4.45217401	0.023	0.002	6.3921E-17	Epi
ANKS62	5.0489E-21	3.26518954	0.096	0.024	7.7607E-17	Epi
SLC4A52	6.4727E-21	3.19456729	0.041	0.005	9.9493E-17	Epi
RAPGEFL12	1.0761E-20	3.0060907	0.094	0.023	1.6541E-16	Epi
PRR15	1.2183E-20	3.38083278	0.142	0.046	1.8727E-16	Epi
CDNF1	1.2485E-20	3.63578496	0.053	0.009	1.919E-16	Epi
LMCD13	1.452E-20	3.90191772	0.053	0.009	2.2318E-16	Epi
CCDC1703	1.848E-20	3.17131015	0.325	0.186	2.8406E-16	Epi
ADAM32	1.971E-20	4.80538769	0.013	0.001	3.0296E-16	Epi
ABCA63	1.9973E-20	3.89531476	0.038	0.005	3.07E-16	Epi
CCDC962	2.3698E-20	3.33742208	0.142	0.047	3.6427E-16	Epi
CHRNA101	2.4845E-20	3.6272771	0.041	0.006	3.8189E-16	Epi
TGFB1I11	2.6013E-20	5.74224336	0.01	0	3.9984E-16	Epi
CLCNKA	3.2909E-20	5.31983992	0.033	0.004	5.0584E-16	Epi
METRN1	3.2971E-20	3.15153185	0.274	0.138	5.068E-16	Epi
SEMA3B	3.772E-20	4.00878256	0.02	0.002	5.7979E-16	Epi
ZC3HAV1L1	4.1187E-20	3.53973914	0.041	0.006	6.3309E-16	Epi
RBM241	4.4505E-20	2.88706818	0.114	0.033	6.8409E-16	Epi
C6orf2232	5.3305E-20	3.73725366	0.01	0	8.1934E-16	Epi
ANKRD372	5.3614E-20	3.0874507	0.241	0.113	8.2411E-16	Epi
SLC44A5	5.3691E-20	3.90461261	0.038	0.005	8.2528E-16	Epi
RNF144A2	5.918E-20	2.3777882	0.038	0.005	9.0966E-16	Epi
SOBP1	6.4422E-20	3.05175359	0.096	0.025	9.9023E-16	Epi
SH2D4A2	6.9392E-20	3.1523578	0.14	0.046	1.0666E-15	Epi

ARHGAP33	7.3066E-20	3.32516074	0.081	0.019	1.1231E-15	Epi
EZR3	7.9757E-20	1.65730486	0.723	0.831	1.2259E-15	Epi
ZSWIM44	8.31E-20	2.91811113	0.117	0.034	1.2773E-15	Epi
B3GNT91	8.3755E-20	2.41207029	0.079	0.018	1.2874E-15	Epi
ADAMTS131	9.5478E-20	3.81379515	0.066	0.013	1.4676E-15	Epi
SUN13	1.5611E-19	2.25775389	0.442	0.308	2.3995E-15	Epi
TF	1.7995E-19	4.92186017	0.015	0.001	2.766E-15	Epi
SUSD2	1.8252E-19	3.99233558	0.023	0.002	2.8055E-15	Epi
ARHGAP122	1.9421E-19	2.19740247	0.185	0.073	2.9852E-15	Epi
CFI	2.1229E-19	4.78550943	0.03	0.003	3.2631E-15	Epi
CDC14A2	2.252E-19	1.6659457	0.201	0.082	3.4615E-15	Epi
EPHX3	2.4496E-19	4.18141737	0.046	0.007	3.7652E-15	Epi
KCP1	2.6357E-19	3.5567839	0.015	0.001	4.0513E-15	Epi
SLC20A21	2.7213E-19	2.7573625	0.322	0.182	4.1828E-15	Epi
ALMS11	2.8684E-19	2.39415584	0.315	0.178	4.4091E-15	Epi
SMIM1	3.512E-19	4.09412028	0.015	0.001	5.3984E-15	Epi
ANXA32	3.5315E-19	3.49964485	0.152	0.054	5.4283E-15	Epi
CEP702	3.7874E-19	2.57148728	0.162	0.06	5.8216E-15	Epi
S100A83	4.3311E-19	0.86433612	0.515	0.844	6.6573E-15	Epi
ARHGEF171	5.0242E-19	3.10897261	0.096	0.026	7.7228E-15	Epi
TTLL34	5.1863E-19	2.44012363	0.454	0.326	7.9719E-15	Epi
NT5DC22	6.1523E-19	4.63696642	0.033	0.004	9.4567E-15	Epi
LOXL1	6.5145E-19	2.88171408	0.03	0.004	1.0013E-14	Epi
PHLPP12	7.05E-19	2.81399712	0.162	0.06	1.0837E-14	Epi
MORN41	7.2503E-19	3.21356226	0.107	0.031	1.1144E-14	Epi
CPLANE11	7.7409E-19	2.47587579	0.292	0.157	1.1899E-14	Epi
JPT23	9.5131E-19	2.22225532	0.447	0.33	1.4623E-14	Epi
DNAAF22	9.624E-19	2.88135159	0.201	0.085	1.4793E-14	Epi
KIT3	1.0305E-18	1.28084307	0.013	0.001	1.584E-14	Epi
ABCC53	1.3341E-18	2.72199445	0.353	0.215	2.0506E-14	Epi
RSPO4	1.4481E-18	4.8139597	0.013	0.001	2.2258E-14	Epi
SIGIRR4	2.1075E-18	1.91971397	0.315	0.177	3.2394E-14	Epi
PALLD3	3.039E-18	2.64726406	0.175	0.07	4.6712E-14	Epi
CDKN1C3	3.1133E-18	3.54221187	0.051	0.009	4.7855E-14	Epi
TYRO3	3.1566E-18	4.60501122	0.025	0.003	4.852E-14	Epi
E2F53	3.1625E-18	3.15779025	0.066	0.014	4.8611E-14	Epi
DPCD1	3.3209E-18	2.98526215	0.31	0.179	5.1046E-14	Epi
TMEM120B2	3.9825E-18	2.82708918	0.218	0.1	6.1215E-14	Epi
UBQLN3	4.376E-18	4.94819518	0.015	0.001	6.7264E-14	Epi
RFX25	4.767E-18	2.67352207	0.289	0.16	7.3273E-14	Epi
NOXRED11	5.0203E-18	4.01148891	0.023	0.002	7.7166E-14	Epi
GVQW3	5.2473E-18	3.36015056	0.048	0.008	8.0656E-14	Epi
ICA13	6.1234E-18	2.61289251	0.198	0.085	9.4123E-14	Epi
AASS2	6.8288E-18	3.29037441	0.079	0.019	1.0496E-13	Epi
EXT13	6.8441E-18	2.1323972	0.261	0.132	1.052E-13	Epi
PLSCR2	6.9063E-18	4.35063458	0.028	0.003	1.0616E-13	Epi
ABHD112	7.6264E-18	2.22376729	0.414	0.287	1.1723E-13	Epi
CCDC68	7.699E-18	3.82505951	0.028	0.003	1.1834E-13	Epi
TBC1D81	1.2905E-17	2.48948246	0.33	0.199	1.9836E-13	Epi
MFSD6L3	2.3255E-17	3.91377291	0.023	0.002	3.5745E-13	Epi
TFPI1	2.5454E-17	4.57124172	0.036	0.005	3.9126E-13	Epi
N6AMT13	2.5456E-17	2.66911035	0.201	0.089	3.9128E-13	Epi

TUFT12	2.6434E-17	4.07816228	0.147	0.055	4.0632E-13	Epi
LAMA2	2.7593E-17	4.32234746	0.023	0.002	4.2414E-13	Epi
ABCD22	2.8791E-17	1.89964518	0.025	0.003	4.4255E-13	Epi
SHROOM31	3.2571E-17	1.99261782	0.307	0.172	5.0065E-13	Epi
IL1A2	3.2891E-17	0.13516132	0.107	0.317	5.0556E-13	Epi
RDH132	3.7974E-17	2.87166837	0.216	0.1	5.837E-13	Epi
PIAS31	4.1249E-17	2.26179234	0.292	0.161	6.3404E-13	Epi
EML51	4.4226E-17	3.91491018	0.015	0.001	6.7981E-13	Epi
KCTD155	4.6034E-17	2.51684329	0.048	0.009	7.0759E-13	Epi
RNF2121	5.0303E-17	3.18696911	0.025	0.003	7.732E-13	Epi
ELL31	5.1467E-17	2.88988013	0.124	0.042	7.9111E-13	Epi
ASAP32	5.3907E-17	3.94464815	0.061	0.013	8.286E-13	Epi
CCNA1	6.5687E-17	5.64016471	0.018	0.001	1.0097E-12	Epi
NFIX1	7.3272E-17	2.74023706	0.213	0.098	1.1263E-12	Epi
CELSR2	7.463E-17	4.08263309	0.028	0.003	1.1471E-12	Epi
MFSD4A1	7.7819E-17	3.51744859	0.076	0.019	1.1962E-12	Epi
OVOL11	7.9629E-17	4.49157633	0.048	0.009	1.224E-12	Epi
SEZ6L2	9.6137E-17	4.36090151	0.018	0.001	1.4777E-12	Epi
USP512	9.6354E-17	4.06202284	0.056	0.011	1.4811E-12	Epi
OFD14	1.0651E-16	1.83916515	0.292	0.159	1.6372E-12	Epi
TRABD4	1.2312E-16	0.13217917	0.444	0.728	1.8925E-12	Epi
ST8SIA13	1.2389E-16	0.69313339	0.025	0.003	1.9044E-12	Epi
SHOC1	1.9109E-16	4.17231403	0.023	0.002	2.9373E-12	Epi
ODF3B4	1.9751E-16	3.56020329	0.548	0.526	3.036E-12	Epi
DEPTOR4	2.0091E-16	3.62892644	0.043	0.007	3.0881E-12	Epi
IFT883	2.186E-16	2.65545746	0.282	0.162	3.3601E-12	Epi
SAPCD12	2.2557E-16	2.67776256	0.132	0.047	3.4673E-12	Epi
SDC43	2.3711E-16	2.45004485	0.475	0.388	3.6446E-12	Epi
FAM218A	2.5566E-16	3.79858787	0.023	0.002	3.9297E-12	Epi
TEAD2	2.5711E-16	6.24763077	0.013	0.001	3.9521E-12	Epi
PKD11	2.5831E-16	2.65983928	0.226	0.11	3.9705E-12	Epi
TP53INP13	2.6703E-16	2.21111486	0.305	0.179	4.1046E-12	Epi
CATSPERE	3.1094E-16	3.43169501	0.048	0.009	4.7794E-12	Epi
SCN4B	3.4652E-16	4.27509993	0.015	0.001	5.3263E-12	Epi
ANKRD262	3.757E-16	2.58525016	0.201	0.092	5.7749E-12	Epi
RAD9A2	4.114E-16	2.96375098	0.178	0.076	6.3236E-12	Epi
AZIN24	4.6235E-16	1.96815548	0.043	0.008	7.1068E-12	Epi
ZSCAN183	5.7513E-16	2.43021646	0.325	0.203	8.8403E-12	Epi
ZNF33B4	5.943E-16	2.361265	0.119	0.041	9.1349E-12	Epi
LRRIQ31	6.377E-16	3.09835483	0.084	0.023	9.8021E-12	Epi
MAGI31	7.2745E-16	2.85104811	0.104	0.033	1.1182E-11	Epi
MXD14	8.0197E-16	1.14754771	0.244	0.496	1.2327E-11	Epi
WFDC10B	8.3998E-16	5.35479008	0.01	0.001	1.2911E-11	Epi
C6orf1634	1.0779E-15	3.63612832	0.089	0.026	1.6569E-11	Epi
PAIP2B1	1.1032E-15	2.58296489	0.104	0.033	1.6958E-11	Epi
BNC21	1.1245E-15	3.25469707	0.036	0.005	1.7284E-11	Epi
AC099489.11	1.1997E-15	2.95288	0.046	0.009	1.844E-11	Epi
NECTIN31	1.2704E-15	2.56176862	0.048	0.009	1.9527E-11	Epi
KLHL41	1.2758E-15	4.04674782	0.038	0.006	1.961E-11	Epi
ACOT113	1.3629E-15	2.44168706	0.132	0.048	2.0948E-11	Epi
BCL9L3	1.5167E-15	1.95162312	0.327	0.204	2.3314E-11	Epi
ZNF4443	1.6507E-15	2.39500191	0.317	0.191	2.5374E-11	Epi

KIAA05561	2.0567E-15	2.27043685	0.378	0.256	3.1613E-11	Epi
LCN121	2.6092E-15	4.11402186	0.013	0.001	4.0106E-11	Epi
DIO2	2.8573E-15	8.04753362	0.01	0.001	4.3919E-11	Epi
ZNF7271	3.4609E-15	4.91273428	0.018	0.002	5.3198E-11	Epi
NOXO1	3.4687E-15	4.43370913	0.015	0.001	5.3317E-11	Epi
GTF2IRD13	3.772E-15	2.57761106	0.206	0.097	5.7979E-11	Epi
NDUFB112	4.0996E-15	0.25886409	0.322	0.598	6.3015E-11	Epi
SPTY2D1OS2	5.2147E-15	3.31710916	0.053	0.011	8.0154E-11	Epi
NUMBL2	5.7792E-15	2.57280253	0.137	0.053	8.8832E-11	Epi
RGL25	5.9607E-15	2.1330033	0.437	0.345	9.1622E-11	Epi
MPZL32	6.1044E-15	2.19874782	0.147	0.059	9.3831E-11	Epi
C19orf442	6.2578E-15	2.60667079	0.114	0.04	9.6188E-11	Epi
TNNC1	6.5486E-15	3.18610556	0.01	0.001	1.0066E-10	Epi
IFT122	6.7045E-15	2.53041604	0.32	0.204	1.0305E-10	Epi
PTPRH	7.5131E-15	4.0538225	0.036	0.006	1.1548E-10	Epi
GNAZ2	7.944E-15	2.77994047	0.028	0.004	1.2211E-10	Epi
C17orf113	7.9549E-15	3.65075685	0.041	0.007	1.2228E-10	Epi
GNA154	8.1446E-15	0.12551436	0.396	0.679	1.2519E-10	Epi
PMAIP14	8.7694E-15	4.73250536	0.117	0.041	1.3479E-10	Epi
LMTK22	9.125E-15	2.45566692	0.195	0.092	1.4026E-10	Epi
RAB3D3	1.0318E-14	2.44240781	0.061	0.014	1.5859E-10	Epi
KDM7A5	1.1452E-14	2.00776628	0.216	0.106	1.7603E-10	Epi
TMPRSS11E	1.2449E-14	6.19367143	0.074	0.02	1.9136E-10	Epi
TIMP4	1.2499E-14	3.16061504	0.066	0.017	1.9212E-10	Epi
IDO13	1.3981E-14	2.60995786	0.033	0.005	2.149E-10	Epi
FBXW9	1.4383E-14	3.2498377	0.104	0.035	2.2108E-10	Epi
CBX42	1.4643E-14	0.20582061	0.368	0.628	2.2508E-10	Epi
VPS37C3	1.4888E-14	0.1373004	0.216	0.442	2.2884E-10	Epi
POFUT22	1.8474E-14	2.6579798	0.284	0.165	2.8396E-10	Epi
FMO51	1.904E-14	2.30692872	0.297	0.177	2.9266E-10	Epi
MLST8	1.9281E-14	0.18501009	0.251	0.478	2.9637E-10	Epi
SPTSSA2	1.9287E-14	0.33585294	0.338	0.596	2.9647E-10	Epi
INPP5A2	2.3137E-14	2.45019351	0.137	0.054	3.5563E-10	Epi
CNIH21	2.3658E-14	2.76206535	0.053	0.012	3.6364E-10	Epi
TMEM372	2.8637E-14	3.07526892	0.056	0.013	4.4018E-10	Epi
CCNK2	3.2848E-14	0.19954589	0.358	0.603	5.0491E-10	Epi
MT1E	3.3495E-14	3.17840894	0.043	0.008	5.1484E-10	Epi
HDAC73	3.3982E-14	1.80856441	0.348	0.226	5.2233E-10	Epi
PHKA11	3.6016E-14	3.04151819	0.056	0.013	5.536E-10	Epi
HECW2	3.7747E-14	3.37794825	0.043	0.008	5.8022E-10	Epi
DUSP101	4.0038E-14	0.31176565	0.325	0.583	6.1543E-10	Epi
ZNF1351	4.3957E-14	3.45574665	0.02	0.002	6.7566E-10	Epi
AGPAT44	4.9782E-14	0.17565022	0.221	0.441	7.652E-10	Epi
TPBG	5.3321E-14	2.53419586	0.061	0.015	8.1959E-10	Epi
STK311	7.0757E-14	3.62756707	0.023	0.003	1.0876E-09	Epi
CBY11	7.6113E-14	2.43055328	0.203	0.1	1.1699E-09	Epi
RHOBTB31	8.0606E-14	2.88152356	0.112	0.04	1.239E-09	Epi
DGKH2	8.4069E-14	2.2424986	0.31	0.193	1.2922E-09	Epi
PARD3B2	8.4822E-14	3.4616499	0.074	0.021	1.3038E-09	Epi
ZNF192	8.6747E-14	2.6407073	0.152	0.065	1.3334E-09	Epi
CAPN24	1.0598E-13	1.0400264	0.728	0.849	1.629E-09	Epi
SMPD22	1.1202E-13	2.29358068	0.325	0.215	1.7218E-09	Epi

CLGN	1.1374E-13	3.62781295	0.02	0.002	1.7483E-09	Epi
APPL23	1.2351E-13	2.15003178	0.299	0.184	1.8984E-09	Epi
TCP11L24	1.2652E-13	2.34147208	0.315	0.199	1.9447E-09	Epi
RASD11	1.3875E-13	3.51066829	0.02	0.002	2.1327E-09	Epi
CFB2	1.4055E-13	2.77081136	0.292	0.182	2.1605E-09	Epi
TSPAN7	1.4775E-13	5.32765782	0.01	0.001	2.271E-09	Epi
DDIT43	1.501E-13	1.65242633	0.381	0.259	2.3071E-09	Epi
OXCT13	1.5095E-13	1.90439505	0.063	0.016	2.3203E-09	Epi
LYSMD1	1.6137E-13	3.71370114	0.03	0.005	2.4804E-09	Epi
PCSK51	1.8724E-13	2.13113723	0.31	0.192	2.8781E-09	Epi
TRAPPC41	2.0028E-13	0.20987794	0.416	0.68	3.0785E-09	Epi
ZDHHC232	2.0691E-13	2.21772363	0.135	0.054	3.1804E-09	Epi
S100A161	2.076E-13	4.0295309	0.221	0.117	3.1911E-09	Epi
TMEM178B	2.1585E-13	3.5365454	0.02	0.002	3.3178E-09	Epi
PLCXD21	2.3368E-13	1.07767403	0.028	0.004	3.592E-09	Epi
DIO1	2.8016E-13	4.17608494	0.036	0.006	4.3063E-09	Epi
PTK22	2.8094E-13	2.29630315	0.246	0.139	4.3183E-09	Epi
IFT22	2.9171E-13	2.64837497	0.274	0.168	4.4838E-09	Epi
RAVER22	3.5175E-13	2.35769998	0.114	0.042	5.4068E-09	Epi
KIF3A3	3.5868E-13	2.14070767	0.241	0.133	5.5133E-09	Epi
B3GNT82	3.6569E-13	3.74833586	0.03	0.005	5.621E-09	Epi
PKD2L2	3.6807E-13	4.08715372	0.01	0.001	5.6577E-09	Epi
TNFRSF212	4.0911E-13	1.96912902	0.325	0.211	6.2884E-09	Epi
SLC38A13	4.263E-13	0.14349511	0.069	0.018	6.5527E-09	Epi
COL1A11	4.4267E-13	0.86251856	0.084	0.026	6.8044E-09	Epi
DBN13	5.6016E-13	1.93106563	0.023	0.003	8.6103E-09	Epi
IL184	6.1142E-13	0.34910686	0.34	0.595	9.3981E-09	Epi
SPA171	6.1792E-13	3.15269169	0.249	0.147	9.4981E-09	Epi
RAB6B2	6.3367E-13	3.12684745	0.094	0.032	9.7401E-09	Epi
CPNE71	6.3925E-13	1.91623686	0.018	0.002	9.8259E-09	Epi
ROBO12	7.7317E-13	2.29591268	0.091	0.03	1.1884E-08	Epi
DUSP191	7.768E-13	3.3909433	0.061	0.016	1.194E-08	Epi
CCDC1222	7.9107E-13	3.09427601	0.063	0.017	1.2159E-08	Epi
LDOC11	8.2325E-13	3.51925128	0.033	0.006	1.2654E-08	Epi
KRTAP5-8	8.5969E-13	4.96370945	0.01	0.001	1.3214E-08	Epi
NCK2	9.5855E-13	1.90747382	0.261	0.153	1.4734E-08	Epi
KREMEN12	9.6888E-13	2.13805506	0.147	0.064	1.4893E-08	Epi
GSTP11	1.1083E-12	1.06801677	0.777	0.914	1.7035E-08	Epi
FOXN34	1.1108E-12	0.16549548	0.236	0.454	1.7073E-08	Epi
NAB22	1.1966E-12	0.2153385	0.185	0.38	1.8393E-08	Epi
LEF12	1.49E-12	1.20717853	0.025	0.004	2.2903E-08	Epi
C2orf922	1.6867E-12	2.68450119	0.084	0.027	2.5927E-08	Epi
BBS93	1.7657E-12	2.08704821	0.264	0.159	2.7141E-08	Epi
PPP1CC1	1.7736E-12	0.12659921	0.269	0.488	2.7262E-08	Epi
CACFD13	1.9892E-12	2.40171404	0.188	0.093	3.0577E-08	Epi
FBF13	1.9958E-12	2.51697461	0.236	0.133	3.0678E-08	Epi
TSPYL41	2.1847E-12	2.28001328	0.175	0.085	3.3581E-08	Epi
CEP170B2	2.4809E-12	2.14901589	0.277	0.167	3.8134E-08	Epi
PLCD31	2.662E-12	2.93474697	0.036	0.007	4.0917E-08	Epi
ANKZF11	2.7608E-12	2.04243263	0.299	0.194	4.2436E-08	Epi
RBMS12	2.8829E-12	0.14387273	0.457	0.711	4.4314E-08	Epi
FOXO6	3.3846E-12	4.06245127	0.028	0.004	5.2024E-08	Epi

SNAPC42	3.4524E-12	2.1371706	0.282	0.176	5.3067E-08	Epi
INAFM24	3.826E-12	0.16996717	0.162	0.341	5.8809E-08	Epi
FAAH4	3.8714E-12	2.45384514	0.122	0.049	5.9507E-08	Epi
ARGLU15	4.0404E-12	1.94187982	0.574	0.59	6.2105E-08	Epi
LRRC12	4.2331E-12	2.45611809	0.104	0.039	6.5066E-08	Epi
TRIM55	4.2354E-12	3.37625443	0.041	0.009	6.5102E-08	Epi
MYRF2	4.5089E-12	3.61339712	0.043	0.009	6.9306E-08	Epi
FAM214A3	4.5761E-12	1.94891697	0.386	0.293	7.0339E-08	Epi
ZNF3962	4.6213E-12	2.34378675	0.109	0.041	7.1034E-08	Epi
SLC16A14	4.7249E-12	4.16306109	0.013	0.001	7.2626E-08	Epi
UBQLNL	5.0629E-12	4.04471168	0.023	0.003	7.7821E-08	Epi
NXT12	5.2902E-12	0.63762739	0.216	0.422	8.1315E-08	Epi
SLC5A9	5.3898E-12	5.38414432	0.013	0.001	8.2847E-08	Epi
LLPH1	5.499E-12	0.10763996	0.193	0.385	8.4525E-08	Epi
METTL7A3	5.534E-12	2.45266899	0.35	0.253	8.5063E-08	Epi
IER3IP12	5.7018E-12	0.12926952	0.282	0.509	8.7643E-08	Epi
IFT572	6.2638E-12	2.53449469	0.416	0.349	9.6281E-08	Epi
TBRG43	7.0073E-12	0.1255386	0.264	0.47	1.0771E-07	Epi
SLC39A31	7.0705E-12	0.2509472	0.244	0.458	1.0868E-07	Epi
ANKRD543	7.8562E-12	2.22344016	0.256	0.155	1.2076E-07	Epi
IFT461	8.1192E-12	2.31176193	0.292	0.187	1.248E-07	Epi
CADM13	8.2803E-12	2.31319266	0.041	0.009	1.2728E-07	Epi
USP535	8.435E-12	2.4469956	0.173	0.085	1.2965E-07	Epi
WDR493	9.8303E-12	2.43582122	0.358	0.271	1.511E-07	Epi
ARL4D2	9.9246E-12	4.07839155	0.041	0.009	1.5255E-07	Epi
AGAP34	9.9843E-12	0.22426875	0.279	0.5	1.5347E-07	Epi
PASK2	1.0279E-11	2.32218963	0.147	0.067	1.58E-07	Epi
BMP21	1.0656E-11	4.78671699	0.013	0.001	1.6379E-07	Epi
EOGT	1.1095E-11	0.17166457	0.388	0.619	1.7054E-07	Epi
LRBA3	1.146E-11	1.59220214	0.355	0.253	1.7615E-07	Epi
HOOK33	1.3104E-11	0.11730857	0.406	0.657	2.0143E-07	Epi
SSBP41	1.5123E-11	2.25546161	0.234	0.135	2.3246E-07	Epi
DYNC2LI11	1.6313E-11	2.16232931	0.305	0.201	2.5075E-07	Epi
SLC40A13	1.6626E-11	2.06529676	0.043	0.01	2.5555E-07	Epi
TTLL52	1.7905E-11	1.95453046	0.459	0.398	2.7522E-07	Epi
FAM71E1	1.9481E-11	3.67946917	0.028	0.005	2.9945E-07	Epi
SGCB1	1.9671E-11	1.99497094	0.074	0.023	3.0237E-07	Epi
ZNF243	1.9941E-11	0.11167325	0.312	0.544	3.0651E-07	Epi
NME72	2.0093E-11	2.13347992	0.246	0.147	3.0885E-07	Epi
DIP2C2	2.0176E-11	2.55993438	0.099	0.037	3.1013E-07	Epi
TAX1BP31	2.0283E-11	0.11434052	0.523	0.788	3.1177E-07	Epi
GCLC1	2.2475E-11	2.25279234	0.424	0.356	3.4547E-07	Epi
SPTLC31	2.3463E-11	3.07855161	0.036	0.007	3.6064E-07	Epi
RNF1352	2.5639E-11	0.1345022	0.234	0.43	3.941E-07	Epi
NIPAL1	2.6768E-11	2.59065976	0.025	0.004	4.1145E-07	Epi
ARNTL25	2.7387E-11	2.5258719	0.099	0.037	4.2096E-07	Epi
TBC1D322	2.7971E-11	2.30290865	0.086	0.03	4.2993E-07	Epi
PLEKHH31	2.8404E-11	2.90783376	0.127	0.055	4.366E-07	Epi
NOTCH24	2.981E-11	0.32980762	0.264	0.476	4.582E-07	Epi
SHARPIN	3.1187E-11	0.11186202	0.421	0.702	4.7937E-07	Epi
UBL51	3.1983E-11	0.11646586	0.289	0.499	4.9161E-07	Epi
KLLN	3.2486E-11	4.64839528	0.015	0.002	4.9934E-07	Epi

TNPO33	3.263E-11	0.12457826	0.277	0.481	5.0156E-07	Epi
TCF44	3.4075E-11	1.84653365	0.162	0.077	5.2377E-07	Epi
OXSR13	3.6361E-11	0.14277044	0.353	0.593	5.5891E-07	Epi
RPIA2	3.6692E-11	0.19958791	0.284	0.506	5.6399E-07	Epi
LTBP22	3.7014E-11	4.34715416	0.013	0.001	5.6895E-07	Epi
RNF1852	3.801E-11	0.10109836	0.282	0.51	5.8425E-07	Epi
GRHL11	4.2009E-11	3.02162693	0.213	0.12	6.4573E-07	Epi
KDM6B2	4.2644E-11	0.10550159	0.388	0.641	6.5548E-07	Epi
HSF43	4.5124E-11	2.4731889	0.051	0.013	6.936E-07	Epi
BLCAP1	4.9822E-11	0.11503892	0.419	0.683	7.6581E-07	Epi
IFT271	5.0587E-11	2.14998082	0.439	0.374	7.7758E-07	Epi
IL36G	5.1196E-11	6.13023429	0.02	0.003	7.8693E-07	Epi
ZDHHC131	5.1382E-11	1.938669	0.32	0.225	7.8979E-07	Epi
VAMP13	5.3066E-11	2.42355099	0.069	0.021	8.1568E-07	Epi
NQO11	6.3166E-11	2.60779983	0.292	0.199	9.7093E-07	Epi
PRPF40B1	6.7538E-11	2.56684832	0.127	0.055	1.0381E-06	Epi
SH3BP43	6.7884E-11	0.2307979	0.338	0.556	1.0434E-06	Epi
KRTAP5-7	7.2036E-11	3.28877552	0.015	0.002	1.1073E-06	Epi
ZKSCAN12	7.2203E-11	1.80612298	0.467	0.405	1.1098E-06	Epi
PER11	7.3482E-11	0.26604655	0.343	0.585	1.1295E-06	Epi
ZNF1652	7.617E-11	2.51975906	0.061	0.018	1.1708E-06	Epi
CHMP1B2	7.7783E-11	0.25748132	0.53	0.794	1.1956E-06	Epi
SCART11	7.9624E-11	2.07049403	0.069	0.021	1.2239E-06	Epi
PAK6	8.1955E-11	3.36644691	0.038	0.008	1.2597E-06	Epi
TPPP1	8.2906E-11	2.14104893	0.302	0.197	1.2743E-06	Epi
METTL152	8.4381E-11	1.90641664	0.272	0.171	1.297E-06	Epi
EPN1	8.608E-11	0.12544338	0.396	0.646	1.3231E-06	Epi
TMEM171	8.9227E-11	5.6691178	0.013	0.001	1.3715E-06	Epi
D2HGDH2	9.1247E-11	2.24651128	0.147	0.069	1.4026E-06	Epi
CC2D2A1	1.0357E-10	2.46735443	0.343	0.257	1.592E-06	Epi
MYZAP2	1.0509E-10	3.87832577	0.076	0.026	1.6154E-06	Epi
TRAPPC121	1.093E-10	0.11185861	0.426	0.686	1.6801E-06	Epi
SPPL2B3	1.1506E-10	2.29100449	0.426	0.36	1.7686E-06	Epi
PHGDH3	1.1586E-10	1.44416934	0.124	0.052	1.7809E-06	Epi
KITLG	1.1858E-10	3.94489216	0.018	0.002	1.8227E-06	Epi
CD383	1.2082E-10	2.3094271	0.221	0.127	1.8571E-06	Epi
MAL1	1.2091E-10	7.01880947	0.084	0.03	1.8586E-06	Epi
CCDC1865	1.2518E-10	1.70148086	0.287	0.185	1.9242E-06	Epi
NOB11	1.3064E-10	0.21574719	0.152	0.317	2.0081E-06	Epi
ZNF3342	1.3121E-10	2.95769313	0.094	0.036	2.0169E-06	Epi
HMGCL1	1.5246E-10	0.25481187	0.35	0.572	2.3435E-06	Epi
EPAS11	1.5292E-10	0.20652319	0.622	0.909	2.3505E-06	Epi
SERINC54	1.55E-10	0.10485022	0.332	0.549	2.3825E-06	Epi
RARRES12	1.6071E-10	3.58200422	0.122	0.053	2.4703E-06	Epi
ST3GAL45	1.6603E-10	3.14902013	0.16	0.08	2.552E-06	Epi
FAM234A	1.7113E-10	0.11081054	0.373	0.6	2.6304E-06	Epi
AP1S33	1.7989E-10	2.01098994	0.119	0.051	2.765E-06	Epi
AGMAT1	1.8172E-10	2.23815565	0.01	0.001	2.7933E-06	Epi
ATF6B	1.9505E-10	0.12591752	0.388	0.631	2.9981E-06	Epi
MLF12	2.0454E-10	2.27350134	0.294	0.202	3.1439E-06	Epi
CDC14B2	2.0593E-10	2.2670793	0.129	0.058	3.1653E-06	Epi
SLC24A3	2.1264E-10	4.78762977	0.01	0.001	3.2685E-06	Epi

LENG85	2.2516E-10	1.43409831	0.614	0.651	3.4609E-06	Epi
CD553	2.661E-10	0.40534425	0.566	0.865	4.0902E-06	Epi
FHAD12	2.9074E-10	1.9561448	0.256	0.158	4.469E-06	Epi
UQCRH3	3.0496E-10	0.34919139	0.175	0.35	4.6876E-06	Epi
FAM166C	3.1117E-10	4.41357587	0.01	0.001	4.783E-06	Epi
PILRB5	3.2559E-10	1.67542289	0.447	0.386	5.0047E-06	Epi
UBE2N2	3.3536E-10	0.35579753	0.188	0.368	5.1548E-06	Epi
IVD1	3.3753E-10	0.14607605	0.299	0.516	5.1881E-06	Epi
NR2F6	3.4401E-10	2.16599633	0.312	0.218	5.2878E-06	Epi
MPST	3.4874E-10	0.43591702	0.279	0.473	5.3605E-06	Epi
SSNA12	3.562E-10	0.22189492	0.261	0.462	5.4751E-06	Epi
PLIN31	3.6221E-10	0.52766858	0.51	0.784	5.5675E-06	Epi
CA3	3.8435E-10	2.99461944	0.028	0.005	5.9079E-06	Epi
ARL62	4.1125E-10	2.23178353	0.157	0.079	6.3213E-06	Epi
KCTD141	4.2091E-10	4.38203288	0.018	0.002	6.4699E-06	Epi
IFT431	4.3489E-10	2.21313757	0.363	0.288	6.6847E-06	Epi
KIAA18412	4.3874E-10	2.28021352	0.371	0.286	6.7439E-06	Epi
TTC21B2	4.4245E-10	2.53173999	0.282	0.192	6.8009E-06	Epi
EGR15	4.7288E-10	1.87484583	0.18	0.094	7.2686E-06	Epi
GAMT2	4.7486E-10	3.11349002	0.023	0.004	7.2991E-06	Epi
PSMD62	4.8815E-10	0.13600956	0.348	0.571	7.5033E-06	Epi
MLLT13	4.8846E-10	0.24422215	0.338	0.55	7.5081E-06	Epi
DAAM14	4.9442E-10	1.90691808	0.241	0.149	7.5997E-06	Epi
SYDE2	5.0964E-10	3.41260398	0.028	0.005	7.8337E-06	Epi
PISD2	5.1544E-10	0.11409233	0.297	0.491	7.9229E-06	Epi
HSF11	5.178E-10	0.10204754	0.541	0.838	7.9592E-06	Epi
TBC1D191	5.4503E-10	2.16986275	0.168	0.088	8.3777E-06	Epi
WIPF3	5.6688E-10	3.45227258	0.018	0.002	8.7135E-06	Epi
TOR2A2	5.7492E-10	0.34056218	0.137	0.288	8.8371E-06	Epi
TMEM183A3	5.7621E-10	0.14202282	0.396	0.618	8.8569E-06	Epi
ASPRV1	6.0205E-10	3.14059035	0.025	0.004	9.2541E-06	Epi
SEC24C1	6.4193E-10	0.26002446	0.365	0.599	9.8671E-06	Epi
NIBAN23	6.4498E-10	2.63800932	0.485	0.438	9.9139E-06	Epi
TMPRSS132	6.4837E-10	2.07539003	0.051	0.014	9.9661E-06	Epi
DUSP55	6.7817E-10	4.69044854	0.14	0.067	1.0424E-05	Epi
MTCH1	6.9052E-10	0.1519271	0.574	0.846	1.0614E-05	Epi
MAPRE31	7.0368E-10	2.37890266	0.431	0.389	1.0816E-05	Epi
SEMA4B5	7.4526E-10	1.98078686	0.193	0.106	1.1455E-05	Epi
YIPF6	7.695E-10	0.30812058	0.274	0.461	1.1828E-05	Epi
MNAT11	8.2969E-10	1.89519178	0.272	0.179	1.2753E-05	Epi
TMEM2343	8.3443E-10	2.26900815	0.266	0.172	1.2826E-05	Epi
PPFIBP23	8.502E-10	0.12118614	0.261	0.454	1.3068E-05	Epi
B4GALT11	8.6406E-10	0.27786638	0.561	0.849	1.3281E-05	Epi
COQ42	8.9464E-10	1.80907059	0.464	0.412	1.3752E-05	Epi
SNX192	9.2434E-10	0.1359552	0.388	0.622	1.4208E-05	Epi
ARL153	1.001E-09	0.22069755	0.157	0.315	1.5387E-05	Epi
CRACR2B2	1.0337E-09	2.8023631	0.348	0.262	1.589E-05	Epi
CLTC1	1.0487E-09	0.11599546	0.256	0.435	1.612E-05	Epi
STARD103	1.065E-09	2.24285141	0.368	0.289	1.637E-05	Epi
ZDHHC204	1.0974E-09	0.11684403	0.388	0.639	1.6869E-05	Epi
CHAMP12	1.0983E-09	0.31723541	0.155	0.315	1.6882E-05	Epi
KAT52	1.1136E-09	0.17986618	0.251	0.438	1.7118E-05	Epi

RERE4	1.2343E-09	1.45620616	0.523	0.501	1.8972E-05	Epi
HDLBP	1.2604E-09	0.10066832	0.48	0.746	1.9374E-05	Epi
GCAT	1.3506E-09	2.91744935	0.038	0.009	2.076E-05	Epi
HDAC22	1.388E-09	0.21863063	0.198	0.37	2.1335E-05	Epi
MPC22	1.4071E-09	0.15607922	0.345	0.551	2.1629E-05	Epi
PET1003	1.42E-09	0.19071063	0.327	0.542	2.1827E-05	Epi
SMOC11	1.4386E-09	5.85357452	0.01	0.001	2.2113E-05	Epi
ARAP33	1.4655E-09	2.89063976	0.033	0.007	2.2526E-05	Epi
CCDC71L3	1.5188E-09	0.21887498	0.122	0.264	2.3345E-05	Epi
TTC30B	1.5231E-09	2.01867655	0.099	0.04	2.3412E-05	Epi
ZFHX21	1.5828E-09	2.5536126	0.16	0.083	2.4329E-05	Epi
POLK2	1.6642E-09	0.12015571	0.284	0.476	2.5581E-05	Epi
SLC26A4	1.6883E-09	5.97135859	0.018	0.003	2.5951E-05	Epi
ERO1A1	1.7653E-09	0.83176057	0.525	0.829	2.7134E-05	Epi
PPIL11	1.7815E-09	0.25991911	0.155	0.316	2.7384E-05	Epi
EIPR12	1.7867E-09	0.42375868	0.19	0.363	2.7463E-05	Epi
ACAD9	1.8241E-09	0.33878391	0.264	0.467	2.8039E-05	Epi
TMEM1162	1.8766E-09	2.16184619	0.208	0.124	2.8845E-05	Epi
ZNF837	1.9429E-09	3.92503498	0.03	0.006	2.9864E-05	Epi
IQCE2	1.9475E-09	2.14506215	0.411	0.359	2.9935E-05	Epi
STK252	1.9574E-09	0.23431235	0.325	0.537	3.0087E-05	Epi
VMO11	2.0254E-09	0.29195821	0.31	0.5	3.1133E-05	Epi
IKZF22	2.1724E-09	1.97048161	0.34	0.257	3.3392E-05	Epi
HDHD21	2.2761E-09	0.28122932	0.317	0.518	3.4986E-05	Epi
SGK2	2.3039E-09	3.90369982	0.01	0.001	3.5414E-05	Epi
CLDN121	2.3119E-09	2.06399785	0.221	0.135	3.5536E-05	Epi
RNF201	2.4635E-09	0.18955538	0.353	0.577	3.7867E-05	Epi
CHD92	2.5231E-09	0.25960643	0.358	0.578	3.8783E-05	Epi
TOP11	2.5772E-09	0.20726274	0.424	0.676	3.9614E-05	Epi
DDX3Y3	2.6245E-09	0.33230152	0.117	0.269	4.0341E-05	Epi
PHOSPHO22	2.6616E-09	2.17727924	0.084	0.032	4.0911E-05	Epi
C15orf652	2.7472E-09	2.93384567	0.061	0.02	4.2227E-05	Epi
KHNYN2	2.7484E-09	0.13945907	0.353	0.572	4.2246E-05	Epi
ADAM93	2.7885E-09	0.31270145	0.322	0.552	4.2862E-05	Epi
PGBD12	2.9266E-09	2.96962396	0.046	0.012	4.4985E-05	Epi
SOD12	2.9315E-09	0.12748364	0.388	0.644	4.5059E-05	Epi
ZNF1851	2.9815E-09	0.41937455	0.31	0.522	4.5828E-05	Epi
TMEM2081	3.0203E-09	0.11084741	0.284	0.49	4.6425E-05	Epi
PSMA12	3.0664E-09	0.18416674	0.228	0.415	4.7134E-05	Epi
MAGEF14	3.1458E-09	1.90629057	0.157	0.082	4.8354E-05	Epi
HIST4H42	3.1588E-09	3.20837229	0.084	0.032	4.8554E-05	Epi
DAP1	3.2042E-09	0.22202504	0.396	0.641	4.9252E-05	Epi
FUZ	3.2829E-09	2.41989148	0.315	0.234	5.0461E-05	Epi
BLOC1S32	3.59E-09	0.37599551	0.157	0.306	5.5181E-05	Epi
NDUFS62	3.7441E-09	0.26744883	0.259	0.437	5.755E-05	Epi
INPP5E2	3.7945E-09	2.0228187	0.287	0.203	5.8326E-05	Epi
YJU22	3.8639E-09	0.16064437	0.256	0.428	5.9392E-05	Epi
SUMO22	4.0157E-09	0.14617967	0.393	0.617	6.1726E-05	Epi
GDF112	4.1052E-09	0.76472139	0.015	0.002	6.3101E-05	Epi
GFOD23	4.3338E-09	1.84990444	0.269	0.179	6.6615E-05	Epi
SFTPC	4.5281E-09	9.78222324	0.015	0.002	6.9602E-05	Epi
GRAMD1A4	4.5542E-09	0.12701853	0.236	0.42	7.0002E-05	Epi

ZBTB442	4.6917E-09	0.16838786	0.31	0.514	7.2116E-05	Epi
TMEM38A	4.9199E-09	3.29592758	0.028	0.006	7.5624E-05	Epi
GNB52	4.9211E-09	1.99265865	0.18	0.101	7.5642E-05	Epi
KIAA05132	4.9809E-09	0.27670126	0.355	0.586	7.6561E-05	Epi
SRA12	4.9931E-09	0.2519269	0.373	0.566	7.6749E-05	Epi
STMP13	5.0009E-09	0.10735727	0.401	0.646	7.6869E-05	Epi
MAD2L1BP1	5.047E-09	0.31590004	0.272	0.449	7.7577E-05	Epi
ACADM	5.1166E-09	0.29785123	0.388	0.61	7.8647E-05	Epi
WIPI23	5.3756E-09	0.11166587	0.386	0.608	8.2628E-05	Epi
P3H4	5.4662E-09	3.81098929	0.02	0.003	8.4021E-05	Epi
TOM13	5.4715E-09	0.12004421	0.325	0.513	8.4102E-05	Epi
ST51	5.5501E-09	1.96724337	0.421	0.376	8.531E-05	Epi
STX81	5.9585E-09	0.13557279	0.264	0.446	9.1587E-05	Epi
PITPNB2	6.1853E-09	0.44262322	0.338	0.541	9.5075E-05	Epi
POLR2L	6.3767E-09	0.12469509	0.546	0.805	9.8016E-05	Epi
RSRP15	6.7308E-09	1.2635009	0.655	0.711	0.00010346	Epi
ALCAM3	7.0624E-09	1.22070505	0.617	0.682	0.00010856	Epi
C11orf491	7.0935E-09	2.06707535	0.284	0.202	0.00010903	Epi
HHAT1	7.1563E-09	2.24723253	0.208	0.125	0.00011	Epi
MED291	7.2264E-09	0.28048425	0.292	0.47	0.00011108	Epi
PFN41	7.354E-09	2.22773633	0.041	0.011	0.00011304	Epi
HTATSF11	7.4175E-09	0.17332539	0.246	0.417	0.00011401	Epi
C2orf15	7.4385E-09	2.89139602	0.025	0.005	0.00011434	Epi
PPP1R12B4	7.4807E-09	2.06075107	0.228	0.143	0.00011499	Epi
TRAK12	8.0372E-09	1.55620318	0.561	0.609	0.00012354	Epi
ODF22	8.1117E-09	2.09624892	0.272	0.188	0.00012468	Epi
DCXR2	8.1518E-09	0.6966582	0.241	0.417	0.0001253	Epi
SLC49A43	8.1534E-09	0.40327793	0.36	0.576	0.00012533	Epi
PA2G41	9.2387E-09	0.20723358	0.378	0.591	0.00014201	Epi
UQCC21	9.2462E-09	0.11598637	0.284	0.483	0.00014212	Epi
CAMTA23	9.3636E-09	0.10594129	0.213	0.38	0.00014393	Epi
RAB371	9.6103E-09	2.08933619	0.053	0.017	0.00014772	Epi
POLR2B1	9.7494E-09	0.27436385	0.434	0.668	0.00014986	Epi
TAS2R43	9.7865E-09	1.89350688	0.063	0.022	0.00015043	Epi
FDX21	9.8213E-09	0.20555598	0.17	0.331	0.00015096	Epi
AP1S1	9.845E-09	0.16582964	0.236	0.415	0.00015133	Epi
NRBP11	1.0171E-08	0.1345626	0.515	0.798	0.00015634	Epi
TGFB22	1.0368E-08	3.15537738	0.018	0.003	0.00015937	Epi
P3H23	1.0439E-08	1.9221097	0.015	0.002	0.00016046	Epi
BSCL21	1.05E-08	0.19312367	0.386	0.625	0.0001614	Epi
CLDN72	1.0836E-08	1.57584363	0.584	0.654	0.00016655	Epi
GJB21	1.0934E-08	4.41441179	0.01	0.001	0.00016806	Epi
LARGE11	1.1388E-08	2.7790501	0.061	0.021	0.00017504	Epi
SCAF42	1.1389E-08	0.16503683	0.279	0.465	0.00017506	Epi
USP103	1.1448E-08	0.28266082	0.317	0.519	0.00017597	Epi
PBLD	1.1898E-08	1.93551681	0.19	0.108	0.00018289	Epi
BBS11	1.2047E-08	2.10396139	0.272	0.185	0.00018518	Epi
HIST1H2AC1	1.2246E-08	3.23057709	0.043	0.012	0.00018823	Epi
RAF13	1.2716E-08	0.1058456	0.345	0.558	0.00019546	Epi
CD2BP21	1.2791E-08	0.10219595	0.325	0.512	0.00019662	Epi
TBC1D110	1.5279E-08	2.04321141	0.129	0.063	0.00023486	Epi
OCIAD22	1.5964E-08	1.54349331	0.15	0.079	0.00024538	Epi

ADSS2	1.6374E-08	0.12294858	0.419	0.681	0.00025169	Epi
VNN13	1.6897E-08	2.82067216	0.081	0.032	0.00025972	Epi
GNMT3	1.7618E-08	2.24307752	0.058	0.019	0.00027081	Epi
PXDC12	1.7757E-08	0.20000732	0.168	0.324	0.00027294	Epi
NDEL13	1.837E-08	0.24726573	0.355	0.561	0.00028236	Epi
UBR33	1.8632E-08	0.1279154	0.256	0.431	0.00028639	Epi
TSEN22	1.8632E-08	0.3375397	0.178	0.324	0.00028639	Epi
N4BP2L13	1.8683E-08	0.10168481	0.302	0.487	0.00028718	Epi
ANP32A1	1.9023E-08	0.38350728	0.206	0.377	0.00029241	Epi
TRIM16	1.9227E-08	2.54612448	0.162	0.089	0.00029553	Epi
OAF3	1.9233E-08	3.14176914	0.038	0.01	0.00029563	Epi
EXOSC72	1.9295E-08	0.13419224	0.236	0.403	0.00029659	Epi
NOP562	2.0136E-08	0.26492122	0.327	0.533	0.00030951	Epi
IL1R14	2.0325E-08	2.12997896	0.084	0.034	0.00031241	Epi
ERH2	2.0973E-08	0.11526041	0.299	0.492	0.00032238	Epi
ARID4A2	2.1265E-08	0.15753709	0.175	0.325	0.00032687	Epi
CCDC1202	2.1594E-08	2.3343504	0.076	0.029	0.00033193	Epi
NUDT24	2.1637E-08	0.25193825	0.249	0.424	0.00033258	Epi
RHOD2	2.2064E-08	2.26286658	0.185	0.108	0.00033915	Epi
CENPX	2.2775E-08	0.21614961	0.18	0.325	0.00035008	Epi
PNPLA83	2.2834E-08	0.18427089	0.213	0.367	0.00035099	Epi
CREBBP2	2.3099E-08	0.16326275	0.401	0.615	0.00035506	Epi
TLE22	2.444E-08	2.96959885	0.069	0.025	0.00037566	Epi
CCDC912	2.4511E-08	0.26600075	0.307	0.5	0.00037676	Epi
PIH1D11	2.452E-08	0.14780361	0.284	0.47	0.00037689	Epi
E2F8	2.4953E-08	1.6718851	0.048	0.014	0.00038356	Epi
CDKN2B1	2.5445E-08	4.48971299	0.119	0.058	0.00039111	Epi
YIPF32	2.5482E-08	0.10248902	0.508	0.776	0.00039169	Epi
PHC25	2.5562E-08	0.19008057	0.381	0.609	0.00039292	Epi
C1QTNF3	2.6026E-08	2.53425586	0.038	0.01	0.00040004	Epi
SERTAD32	2.9237E-08	0.23344965	0.299	0.491	0.0004494	Epi
CPLANE22	2.9271E-08	2.62717145	0.099	0.044	0.00044992	Epi
ORMDL12	2.9445E-08	0.10596786	0.368	0.582	0.00045259	Epi
LMAN1	2.974E-08	0.21376865	0.495	0.748	0.00045713	Epi
CHMP4B	3.0372E-08	0.25626054	0.52	0.789	0.00046685	Epi
GAN2	3.0429E-08	2.28115921	0.086	0.036	0.00046773	Epi
TRPT12	3.0962E-08	1.82359315	0.365	0.293	0.00047592	Epi
U2SURP1	3.1585E-08	0.16477288	0.378	0.609	0.0004855	Epi
ANKRD13D2	3.2265E-08	0.2512457	0.454	0.71	0.00049595	Epi
CLMN2	3.299E-08	1.94043113	0.315	0.235	0.0005071	Epi
CEP2501	3.358E-08	2.01684663	0.246	0.165	0.00051617	Epi
MACROD2	3.3596E-08	4.32663135	0.01	0.001	0.00051641	Epi
HSPB12	3.4162E-08	0.22102225	0.746	0.95	0.0005251	Epi
HMG20B	3.4641E-08	0.38527671	0.424	0.654	0.00053247	Epi
VPS4B1	3.7395E-08	0.17292695	0.429	0.67	0.0005748	Epi
RABGEF14	3.7811E-08	0.11027123	0.231	0.396	0.00058119	Epi
GCN11	3.8836E-08	0.10336386	0.302	0.484	0.00059694	Epi
DNAJC122	3.9013E-08	3.86217399	0.018	0.003	0.00059967	Epi
OR52H1	4.0622E-08	3.58210497	0.013	0.002	0.00062441	Epi
DENND1A3	4.0836E-08	0.31338402	0.315	0.514	0.00062769	Epi
ZNF7131	4.0986E-08	2.44967985	0.099	0.044	0.00063	Epi
DNM31	4.1716E-08	3.48111067	0.03	0.007	0.00064121	Epi

TSC22D21	4.3222E-08	0.32203782	0.353	0.564	0.00066437	Epi
SEPTIN102	4.3879E-08	0.13176132	0.249	0.419	0.00067447	Epi
PCDHGA7	4.4019E-08	4.10138028	0.013	0.002	0.00067661	Epi
VPS542	4.4817E-08	0.11106477	0.317	0.501	0.00068888	Epi
AVIL5	4.4821E-08	2.33753288	0.048	0.015	0.00068894	Epi
KIAA1324L2	4.5536E-08	3.10833418	0.025	0.005	0.00069993	Epi
SDAD12	4.689E-08	0.28534507	0.142	0.273	0.00072075	Epi
ADAM22	4.8392E-08	2.57023755	0.015	0.002	0.00074384	Epi
UBE2E31	4.995E-08	0.27625214	0.272	0.44	0.00076778	Epi
WRAP532	5.0773E-08	2.12808242	0.256	0.177	0.00078043	Epi
LRWD12	5.0831E-08	2.0596479	0.193	0.118	0.00078132	Epi
LRFN31	5.1337E-08	1.86371164	0.043	0.013	0.00078911	Epi
PRPF38B2	5.1452E-08	0.2501346	0.398	0.618	0.00079087	Epi
CEP1043	5.2736E-08	0.33365023	0.19	0.346	0.00081061	Epi
DLAT	5.3883E-08	0.14240611	0.249	0.419	0.00082824	Epi
CORO72	5.4526E-08	0.18921227	0.475	0.738	0.00083811	Epi
CLDND13	5.591E-08	0.13353554	0.312	0.503	0.00085939	Epi
GALNT17	5.5935E-08	0.6151987	0.381	0.608	0.00085978	Epi
GAR12	6.0554E-08	0.16622234	0.269	0.443	0.00093078	Epi
SYNJ13	6.0807E-08	0.39483972	0.107	0.229	0.00093467	Epi
PECR1	6.1901E-08	0.14375664	0.325	0.518	0.00095147	Epi
NCOA52	6.2279E-08	0.17027759	0.173	0.313	0.0009573	Epi
AK12	6.4429E-08	1.77405878	0.312	0.232	0.00099034	Epi
DHX383	6.5199E-08	0.14373189	0.297	0.468	0.00100218	Epi
SLC25A252	6.6114E-08	0.11915197	0.345	0.535	0.00101624	Epi
ITGAV3	6.7006E-08	0.16454449	0.335	0.541	0.00102994	Epi
TPRKB2	6.8469E-08	0.26049684	0.19	0.347	0.00105243	Epi
ZNF323	6.8523E-08	0.37999618	0.279	0.464	0.00105327	Epi
FAM174A1	6.9562E-08	2.23267221	0.373	0.32	0.00106924	Epi
NECTIN1	6.9794E-08	0.24545471	0.183	0.328	0.0010728	Epi
CMAS1	7.0113E-08	0.22685553	0.34	0.531	0.00107771	Epi
KCNJ22	7.0689E-08	2.91448509	0.051	0.016	0.00108656	Epi
FHOD14	7.7544E-08	0.28822019	0.168	0.314	0.00119193	Epi
PNRC23	7.7825E-08	0.17715212	0.287	0.466	0.00119624	Epi
ABTB23	7.9072E-08	2.84916208	0.036	0.009	0.00121542	Epi
DYRK1B1	7.9384E-08	1.771615	0.274	0.194	0.00122021	Epi
BCL92	8.0307E-08	2.015592	0.086	0.037	0.0012344	Epi
ZC3H7A2	8.1162E-08	0.13484562	0.254	0.417	0.00124754	Epi
COQ10B2	8.2691E-08	0.326106	0.287	0.468	0.00127104	Epi
LRRC582	8.3075E-08	0.21245717	0.223	0.383	0.00127695	Epi
SH3YL12	8.4756E-08	1.88552311	0.279	0.202	0.00130278	Epi
NOL4L2	8.5736E-08	0.12428688	0.249	0.413	0.00131785	Epi
DCTN3	8.7905E-08	0.15121651	0.411	0.633	0.00135119	Epi
ALG141	8.8248E-08	0.16192852	0.155	0.292	0.00135646	Epi
NUBP22	9.1021E-08	0.13458176	0.17	0.314	0.00139909	Epi
GATB1	9.1274E-08	0.12710854	0.165	0.303	0.00140297	Epi
RNMT5	9.143E-08	0.12039336	0.193	0.339	0.00140538	Epi
SAMD92	9.3408E-08	0.38394007	0.231	0.398	0.00143577	Epi
GOSR13	1.0066E-07	0.33314133	0.299	0.487	0.00154726	Epi
ZNF4151	1.0103E-07	3.1629978	0.041	0.012	0.00155287	Epi
ABI22	1.0354E-07	1.83901586	0.322	0.248	0.00159157	Epi
MIOS5	1.037E-07	0.56534719	0.195	0.338	0.00159396	Epi

PDHB2	1.0535E-07	0.39390027	0.373	0.596	0.00161931	Epi
SEMA6C	1.0735E-07	3.4820234	0.015	0.002	0.00165004	Epi
PIGP3	1.0804E-07	0.12747644	0.198	0.347	0.00166063	Epi
PPP1R12C3	1.0831E-07	0.17929131	0.302	0.494	0.00166477	Epi
MIEN11	1.0999E-07	0.16923485	0.358	0.566	0.00169071	Epi
ZNF6722	1.1059E-07	0.26283781	0.18	0.328	0.0016999	Epi
NUDCD31	1.1165E-07	0.19174648	0.368	0.573	0.00171621	Epi
DTX42	1.1392E-07	0.34710275	0.363	0.566	0.00175106	Epi
CXorf58	1.1403E-07	4.57321348	0.01	0.001	0.00175278	Epi
PRADC1	1.146E-07	0.19765525	0.124	0.248	0.00176158	Epi
SPATA72	1.1511E-07	2.49095509	0.127	0.066	0.0017693	Epi
GPATCH32	1.1794E-07	0.23732788	0.145	0.274	0.00181278	Epi
CXXC13	1.1874E-07	0.18969866	0.307	0.498	0.00182516	Epi
ARL1	1.191E-07	0.23039944	0.325	0.528	0.00183073	Epi
SORBS31	1.2272E-07	0.35511773	0.396	0.611	0.00188639	Epi
PTER1	1.2275E-07	0.21446811	0.135	0.261	0.00188679	Epi
NIN1	1.2454E-07	0.24207223	0.437	0.674	0.0019143	Epi
GATAD12	1.2469E-07	0.20977984	0.312	0.49	0.00191661	Epi
NUP852	1.2623E-07	0.1192081	0.292	0.477	0.00194029	Epi
AP1G13	1.2778E-07	0.13755692	0.332	0.521	0.00196415	Epi
MXD31	1.2877E-07	1.68730624	0.076	0.031	0.00197925	Epi
ADGRL14	1.2896E-07	2.72209887	0.056	0.019	0.00198229	Epi
TRIP44	1.3049E-07	0.10215005	0.249	0.413	0.00200577	Epi
SRSF42	1.3052E-07	0.1451089	0.497	0.744	0.00200617	Epi
C12orf41	1.3123E-07	0.19272181	0.185	0.323	0.00201707	Epi
WDPCP3	1.357E-07	2.0060308	0.18	0.107	0.00208592	Epi
JMJD8	1.3709E-07	0.13409672	0.335	0.532	0.00210723	Epi
PLRG11	1.42E-07	0.13055926	0.404	0.612	0.00218263	Epi
PLAAT41	1.4293E-07	1.59288918	0.52	0.539	0.00219698	Epi
CYTH24	1.4302E-07	1.35930676	0.525	0.546	0.00219841	Epi
STX101	1.4308E-07	0.33313179	0.434	0.666	0.00219933	Epi
CATSPER23	1.4463E-07	2.11731583	0.081	0.034	0.0022231	Epi
COG22	1.4787E-07	0.20407573	0.173	0.31	0.00227284	Epi
TCTE32	1.512E-07	2.73385667	0.041	0.012	0.00232415	Epi
PRPS12	1.5259E-07	0.1508024	0.221	0.377	0.0023454	Epi
ZNF5992	1.5383E-07	2.21786795	0.162	0.093	0.00236444	Epi
RBKS2	1.5402E-07	2.42926099	0.074	0.03	0.00236751	Epi
TCTN12	1.557E-07	2.35094613	0.322	0.263	0.00239327	Epi
PHF123	1.5942E-07	0.1133783	0.269	0.439	0.00245044	Epi
XPC2	1.6667E-07	0.17672825	0.246	0.412	0.00256193	Epi
TTC30A	1.7177E-07	2.79849903	0.081	0.035	0.00264022	Epi
SRD5A3	1.7558E-07	0.57195833	0.33	0.526	0.00269889	Epi
ZNF3912	1.7745E-07	3.4348952	0.058	0.021	0.00272753	Epi
DNAJC271	1.7829E-07	2.2991733	0.081	0.035	0.00274051	Epi
KLHL31	1.787E-07	2.46786018	0.043	0.013	0.00274673	Epi
PGPEP12	1.7873E-07	0.22497287	0.307	0.496	0.00274723	Epi
DHRSX2	1.7996E-07	0.15621982	0.33	0.508	0.0027662	Epi
POLR2F1	1.8135E-07	0.24537625	0.266	0.441	0.0027876	Epi
COX7A1	1.8136E-07	4.17033972	0.018	0.003	0.00278768	Epi
USP363	1.8609E-07	0.12205609	0.216	0.364	0.00286041	Epi
SREBF13	1.8677E-07	0.31273019	0.393	0.58	0.00287082	Epi
MPP2	1.8874E-07	3.89381579	0.01	0.001	0.00290115	Epi

MMADHC3	1.8889E-07	0.19093257	0.299	0.485	0.00290343	Epi
CWC272	1.912E-07	0.3703279	0.195	0.352	0.00293897	Epi
USP44	1.9853E-07	0.11832773	0.335	0.515	0.00305163	Epi
MDK	1.99E-07	1.98389568	0.292	0.215	0.0030588	Epi
BRD42	2.0221E-07	0.11275615	0.482	0.735	0.00310817	Epi
PSMB5	2.0306E-07	0.25794814	0.419	0.655	0.00312119	Epi
ZFC3H14	2.1271E-07	0.13299068	0.277	0.447	0.00326964	Epi
THNSL22	2.129E-07	2.10181233	0.178	0.108	0.00327242	Epi
ZNF5542	2.1588E-07	2.28571303	0.15	0.083	0.00331825	Epi
SLC25A132	2.1989E-07	0.2687064	0.261	0.423	0.00337991	Epi
PDE8B3	2.2573E-07	3.16897363	0.089	0.04	0.00346968	Epi
LRRC403	2.2876E-07	0.15004111	0.173	0.307	0.00351628	Epi
UBAP12	2.3226E-07	0.14696552	0.373	0.576	0.00357007	Epi
CHST103	2.3253E-07	3.11051593	0.015	0.002	0.00357428	Epi
RGMA	2.4757E-07	2.63821024	0.018	0.003	0.00380538	Epi
ARF54	2.4758E-07	0.11085103	0.206	0.356	0.0038055	Epi
KIAA07532	2.5153E-07	2.10257162	0.188	0.115	0.00386629	Epi
CLASP22	2.5204E-07	0.11442441	0.18	0.323	0.00387412	Epi
C16orf724	2.5709E-07	0.46146979	0.409	0.625	0.00395172	Epi
FGF13	2.6132E-07	2.89259914	0.03	0.008	0.00401673	Epi
TRMT16	2.6268E-07	0.10481345	0.234	0.378	0.00403762	Epi
SEC23IP1	2.6736E-07	0.32117743	0.355	0.558	0.00410963	Epi
LRRC273	2.7674E-07	2.25546498	0.157	0.091	0.00425373	Epi
GPRC5B1	2.7696E-07	3.36099526	0.056	0.02	0.00425712	Epi
WDR551	2.7943E-07	0.14499403	0.228	0.388	0.00429504	Epi
ITPA2	2.9058E-07	0.24382993	0.411	0.626	0.00446657	Epi
ARHGEF282	3.0007E-07	0.15061758	0.434	0.645	0.00461232	Epi
SYNPO1	3.0106E-07	1.45719386	0.114	0.056	0.00462762	Epi
RABL61	3.0732E-07	0.23284433	0.424	0.652	0.00472385	Epi
SLC25A141	3.0771E-07	2.4201575	0.124	0.065	0.00472979	Epi
COPRS1	3.165E-07	0.28387843	0.383	0.616	0.00486487	Epi
CABIN11	3.2677E-07	0.49927257	0.259	0.423	0.00502282	Epi
COPS81	3.367E-07	0.2822426	0.241	0.394	0.00517541	Epi
TNK23	3.3886E-07	0.13098951	0.261	0.423	0.00520867	Epi
TSPAN10	3.4074E-07	3.59233134	0.015	0.002	0.00523745	Epi
RNF1502	3.4212E-07	2.81919138	0.01	0.001	0.00525865	Epi
TMEM1752	3.4233E-07	0.29907985	0.315	0.499	0.00526196	Epi
ABCF22	3.6301E-07	0.11203976	0.132	0.25	0.00557982	Epi
AQP71	3.6441E-07	3.01463835	0.056	0.02	0.00560127	Epi
EDEM33	3.6607E-07	0.14036452	0.388	0.584	0.0056269	Epi
ARID5B2	3.6703E-07	1.42935293	0.157	0.09	0.00564166	Epi
BBS52	3.757E-07	2.23504021	0.178	0.11	0.00577493	Epi
MTMR32	3.8243E-07	0.11152371	0.454	0.674	0.00587839	Epi
SREK1IP12	3.853E-07	0.21406219	0.14	0.263	0.0059225	Epi
ECI11	3.8601E-07	0.10717607	0.345	0.549	0.00593342	Epi
GUK11	4.0253E-07	0.18798304	0.386	0.591	0.00618725	Epi
PLEKHG32	4.0503E-07	0.14888244	0.203	0.344	0.00622579	Epi
DNPH15	4.1138E-07	2.38984582	0.195	0.125	0.00632332	Epi
ROCK23	4.233E-07	0.13315152	0.279	0.454	0.00650657	Epi
GGA12	4.2563E-07	0.23486849	0.332	0.524	0.00654235	Epi
CUL31	4.2912E-07	0.12234682	0.426	0.649	0.00659603	Epi
SIRT71	4.4217E-07	0.21352473	0.327	0.533	0.0067966	Epi

SERTAD25	4.4232E-07	0.2721635	0.409	0.629	0.00679884	Epi
TOB13	4.4534E-07	1.52178678	0.533	0.531	0.00684536	Epi
PBXIP1	4.5062E-07	0.10615948	0.206	0.346	0.00692649	Epi
KLHL242	4.5563E-07	0.33411492	0.244	0.408	0.00700348	Epi
NUP1332	4.7867E-07	0.18576398	0.284	0.454	0.00735764	Epi
WRNIP12	4.8764E-07	0.33188679	0.376	0.579	0.0074955	Epi
GSR1	4.8816E-07	0.13417324	0.482	0.706	0.00750358	Epi
ATF32	4.8821E-07	2.87857503	0.254	0.182	0.00750431	Epi
ATP6AP1L	4.8956E-07	2.74848527	0.033	0.009	0.00752496	Epi
RPGR3	4.9267E-07	1.97729614	0.279	0.211	0.00757279	Epi
TOR1AIP22	4.9886E-07	0.31109977	0.315	0.504	0.00766801	Epi
IPP1	5.0238E-07	1.92907004	0.15	0.086	0.00772204	Epi
DDX461	5.0999E-07	0.10098377	0.464	0.703	0.00783908	Epi
C1orf561	5.133E-07	2.21503284	0.036	0.01	0.00788995	Epi
YPEL36	5.2499E-07	1.54217261	0.302	0.23	0.00806961	Epi
HM133	5.2786E-07	0.2527115	0.444	0.662	0.00811368	Epi
OXR11	5.3919E-07	0.1124835	0.365	0.581	0.00828794	Epi
ARMCX13	5.455E-07	0.25646882	0.084	0.189	0.00838493	Epi
SFXN33	5.4714E-07	0.31898406	0.464	0.698	0.00841002	Epi
TSTD11	5.5313E-07	1.87820321	0.269	0.199	0.00850209	Epi
SPAG93	5.6495E-07	0.12308625	0.409	0.624	0.00868378	Epi
SLC35A32	5.7584E-07	1.43750601	0.419	0.388	0.00885117	Epi
ZNF6092	5.792E-07	0.43719425	0.368	0.562	0.00890285	Epi
MESP1	5.811E-07	2.90105802	0.028	0.007	0.00893213	Epi
SNORC	5.9806E-07	3.0549672	0.025	0.006	0.00919274	Epi
SYF22	6.0285E-07	0.29601055	0.404	0.604	0.00926645	Epi
ZMYM42	6.0938E-07	0.24169607	0.266	0.432	0.00936672	Epi
DNAJC61	6.1808E-07	0.30480987	0.076	0.175	0.00950056	Epi
IRAK1BP11	6.2536E-07	2.64127247	0.061	0.023	0.00961234	Epi
LONP22	6.2678E-07	0.25714049	0.383	0.59	0.00963428	Epi
CASP92	6.2772E-07	0.23131929	0.34	0.519	0.00964873	Epi
EBNA1BP2	6.487E-07	0.16237653	0.371	0.589	0.00997113	Epi
PLA2R12	6.6563E-07	2.3920529	0.084	0.038	0.01023141	Epi
SRP91	6.7304E-07	0.2025398	0.294	0.463	0.01034524	Epi
SNX25	6.8342E-07	2.33819686	0.081	0.036	0.01050491	Epi
ZNF4694	6.8379E-07	3.15904785	0.033	0.009	0.01051057	Epi
NUTF24	6.8551E-07	0.17749849	0.327	0.512	0.01053701	Epi
OSBP3	6.9688E-07	0.32412857	0.376	0.591	0.0107117	Epi
EDC43	7.051E-07	0.1810086	0.279	0.444	0.01083816	Epi
HAS3	7.0832E-07	3.92835237	0.023	0.005	0.01088757	Epi
FGFR1OP3	7.1309E-07	1.88565683	0.256	0.186	0.01096089	Epi
DUS3L2	7.1738E-07	0.2091672	0.19	0.323	0.01102684	Epi
CEP633	7.1811E-07	0.28318071	0.261	0.438	0.01103814	Epi
DDX19B2	7.2479E-07	0.13052946	0.175	0.306	0.01114074	Epi
DNAJC212	7.2646E-07	0.31010372	0.345	0.544	0.0111664	Epi
TBL32	7.2788E-07	0.1375654	0.216	0.362	0.01118827	Epi
SH3D212	7.3036E-07	2.12869614	0.046	0.015	0.01122638	Epi
PPP1R3D1	7.3976E-07	0.26819641	0.155	0.284	0.01137084	Epi
TGFA1	7.4245E-07	2.13330874	0.112	0.056	0.01141214	Epi
COMMD22	7.4366E-07	0.36962898	0.279	0.441	0.01143075	Epi
EMD2	7.5101E-07	0.1863589	0.393	0.597	0.01154378	Epi
NAT1	7.5197E-07	1.95806033	0.195	0.124	0.01155846	Epi

AAAS1	7.5252E-07	0.32695973	0.284	0.452	0.01156704	Epi
SRP541	7.5268E-07	0.2382724	0.482	0.712	0.01156951	Epi
TP53I112	7.5451E-07	0.27684611	0.221	0.371	0.01159763	Epi
POLG3	7.5685E-07	0.17412558	0.183	0.318	0.01163351	Epi
SLC35B23	7.9884E-07	0.22394007	0.228	0.378	0.01227891	Epi
DDX61	8.2343E-07	0.11722672	0.472	0.715	0.01265697	Epi
TPRG1L3	8.2491E-07	0.23063104	0.335	0.521	0.01267973	Epi
RPF23	8.2637E-07	0.13699905	0.137	0.259	0.01270215	Epi
MARF13	8.2803E-07	0.18534071	0.203	0.347	0.01272766	Epi
LRRFIP22	8.5965E-07	0.28063972	0.302	0.46	0.01321375	Epi
PMPCB3	8.7858E-07	0.2825848	0.424	0.638	0.0135046	Epi
BLVRB2	8.8021E-07	0.33644229	0.365	0.547	0.01352971	Epi
PTP4A33	8.807E-07	2.45701294	0.028	0.007	0.01353719	Epi
TMOD32	8.888E-07	0.35048581	0.51	0.789	0.0136618	Epi
FTSJ32	9.2963E-07	0.23627639	0.249	0.405	0.01428938	Epi
ALPK12	9.3851E-07	0.23967638	0.221	0.366	0.01442584	Epi
PDCD52	9.5466E-07	0.16695255	0.188	0.327	0.01467414	Epi
SWAP703	9.6182E-07	0.14502816	0.388	0.604	0.0147842	Epi
TRIOBP1	1.0248E-06	0.22209535	0.464	0.686	0.01575275	Epi
FBH13	1.0254E-06	0.13614677	0.254	0.409	0.01576115	Epi
ZNF4932	1.0321E-06	1.48809809	0.241	0.167	0.01586425	Epi
NDUFA3	1.0388E-06	0.13844787	0.581	0.831	0.01596698	Epi
CUL4A2	1.0597E-06	0.34541541	0.335	0.515	0.01628872	Epi
NME11	1.0682E-06	0.20942945	0.228	0.37	0.01642002	Epi
DCAF102	1.1012E-06	0.30041704	0.231	0.381	0.01692717	Epi
NPRL22	1.1153E-06	0.13963651	0.14	0.256	0.01714335	Epi
NAA602	1.1201E-06	0.35705957	0.411	0.611	0.01721642	Epi
CTDP12	1.1334E-06	0.28331913	0.332	0.519	0.01742088	Epi
TMEM2381	1.1471E-06	1.63555606	0.18	0.112	0.01763132	Epi
PTK62	1.1638E-06	2.54690497	0.119	0.063	0.017889	Epi
PGRMC1	1.1779E-06	0.20351039	0.404	0.626	0.01810572	Epi
SEC24B2	1.1978E-06	0.1648115	0.299	0.476	0.01841108	Epi
LAMC12	1.2025E-06	2.20223303	0.109	0.056	0.01848338	Epi
PGAM52	1.2236E-06	0.41176635	0.203	0.35	0.01880766	Epi
SLC22A181	1.2479E-06	0.33610013	0.256	0.413	0.01918126	Epi
TCTN21	1.2555E-06	2.11792179	0.193	0.128	0.01929797	Epi
TSC22D12	1.26E-06	1.62813672	0.325	0.257	0.01936718	Epi
PHC13	1.2685E-06	1.55916297	0.294	0.223	0.01949855	Epi
CYTH14	1.2709E-06	0.10382153	0.586	0.833	0.01953518	Epi
ZNF3162	1.2739E-06	0.14166978	0.206	0.347	0.01958045	Epi
ZNF203	1.2771E-06	1.8283559	0.091	0.043	0.01963021	Epi
LRRC472	1.2805E-06	0.31474875	0.393	0.588	0.01968235	Epi
PEX101	1.2819E-06	0.14676838	0.198	0.334	0.01970419	Epi
RNF144B3	1.2904E-06	0.19630526	0.32	0.498	0.0198342	Epi
HIRA1	1.2929E-06	0.489155	0.208	0.35	0.01987339	Epi
MTFR21	1.3334E-06	0.20888184	0.069	0.159	0.02049503	Epi
TOGARAM13	1.3375E-06	1.63183731	0.272	0.205	0.02055912	Epi
THRAP32	1.3392E-06	0.12400185	0.495	0.728	0.02058498	Epi
BAIAP2L2	1.3417E-06	2.65088335	0.028	0.007	0.02062261	Epi
CASC42	1.3602E-06	0.21404294	0.325	0.505	0.0209075	Epi
RASSF73	1.3656E-06	1.95550337	0.279	0.213	0.02099007	Epi
FAM114A21	1.3874E-06	0.23224598	0.145	0.265	0.02132554	Epi

CCDC1711	1.393E-06	2.36696778	0.142	0.083	0.02141185	Epi
CTNNBIP12	1.3983E-06	0.14809863	0.266	0.421	0.02149282	Epi
UBE3B1	1.411E-06	0.12380881	0.188	0.324	0.02168796	Epi
RNF113	1.4527E-06	0.15201513	0.464	0.705	0.02232951	Epi
NOC2L3	1.4758E-06	0.12189371	0.249	0.395	0.02268384	Epi
WDR911	1.4839E-06	0.41123994	0.069	0.163	0.02280879	Epi
HIST1H2BG5	1.5009E-06	3.38860832	0.051	0.018	0.02307099	Epi
ITPRID24	1.502E-06	0.23271417	0.426	0.634	0.02308759	Epi
SGTA1	1.5251E-06	0.6454803	0.33	0.513	0.02344166	Epi
SLC7A6OS3	1.5421E-06	0.22952051	0.119	0.225	0.02370345	Epi
CHD15	1.5449E-06	0.23805802	0.246	0.405	0.02374736	Epi
TBC1D123	1.5457E-06	0.20509619	0.129	0.245	0.02375883	Epi
GNAI1	1.5594E-06	1.82188287	0.137	0.076	0.0239697	Epi
ENTPD62	1.5851E-06	0.19990162	0.322	0.505	0.02436474	Epi
AQR2	1.5928E-06	0.11629368	0.277	0.439	0.0244823	Epi
PDCD63	1.5966E-06	0.21427767	0.52	0.749	0.02454098	Epi
RIMKLB3	1.6096E-06	1.91836432	0.269	0.199	0.02474046	Epi
CHN11	1.6101E-06	1.48771082	0.033	0.009	0.02474856	Epi
HINT2	1.611E-06	0.44915223	0.292	0.455	0.02476305	Epi
GSKIP2	1.6644E-06	0.11830692	0.218	0.362	0.02558339	Epi
UBD3	1.679E-06	2.83997416	0.033	0.01	0.02580798	Epi
AMD13	1.6855E-06	0.32767057	0.241	0.378	0.02590848	Epi
DUSP222	1.724E-06	0.1995201	0.307	0.473	0.02649992	Epi
BZW13	1.7488E-06	0.23382124	0.272	0.432	0.02688027	Epi
PROSER32	1.7512E-06	1.9119983	0.226	0.156	0.02691749	Epi
AGO44	1.7598E-06	0.11925823	0.299	0.456	0.02705059	Epi
TM9SF1	1.7747E-06	0.1937304	0.447	0.676	0.02727968	Epi
MAST21	1.7911E-06	0.24899508	0.226	0.364	0.02753057	Epi
PKIG1	1.8124E-06	2.28676559	0.234	0.167	0.02785913	Epi
WBP112	1.8322E-06	0.26221614	0.183	0.308	0.02816258	Epi
ERMP11	1.8333E-06	0.153558	0.259	0.41	0.02818018	Epi
MANEA3	1.8652E-06	0.27757859	0.178	0.305	0.02866997	Epi
SASH11	1.8699E-06	0.87669447	0.211	0.354	0.02874214	Epi
YBX33	1.8706E-06	1.00545686	0.624	0.705	0.02875357	Epi
FBXL83	1.8826E-06	1.59052025	0.107	0.054	0.02893708	Epi
ITPKC3	1.892E-06	3.3024452	0.35	0.301	0.02908187	Epi
COBLL12	1.8921E-06	2.54767846	0.099	0.049	0.02908417	Epi
RCAN12	1.8938E-06	0.21991913	0.127	0.238	0.02910965	Epi
ELOVL55	1.9414E-06	0.15307244	0.406	0.59	0.02984176	Epi
USP393	1.956E-06	0.39851866	0.195	0.326	0.03006623	Epi
JOSD21	1.9747E-06	0.19566019	0.307	0.477	0.03035242	Epi
CLTA1	1.9778E-06	0.18867494	0.467	0.7	0.03040149	Epi
SIVA12	1.9881E-06	0.20387458	0.416	0.623	0.0305588	Epi
PRORP3	1.9904E-06	0.11955343	0.16	0.287	0.03059466	Epi
ARFGEF23	2.0156E-06	0.12592728	0.312	0.477	0.03098147	Epi
LRIF12	2.0452E-06	0.24208984	0.145	0.259	0.03143644	Epi
MAGED12	2.0482E-06	1.81040911	0.307	0.241	0.03148336	Epi
EVI52	2.0772E-06	0.25871576	0.393	0.606	0.03192835	Epi
IQCC	2.0837E-06	2.84385981	0.066	0.028	0.0320287	Epi
UCP32	2.1043E-06	2.36378258	0.053	0.02	0.0323453	Epi
GDF9	2.1059E-06	3.60018409	0.013	0.002	0.03237028	Epi
UCK12	2.1306E-06	0.4707763	0.279	0.445	0.03274897	Epi

COMMD32	2.1394E-06	0.23149796	0.234	0.387	0.03288441	Epi
POLR3C2	2.1399E-06	0.11058695	0.282	0.437	0.032892	Epi
ZNF1311	2.2407E-06	0.27413806	0.117	0.223	0.0344422	Epi
ARIH22	2.291E-06	0.10915732	0.317	0.483	0.03521556	Epi
TMEM713	2.309E-06	1.01231674	0.061	0.024	0.03549195	Epi
NR3C21	2.3575E-06	2.15302131	0.168	0.105	0.03623649	Epi
EWSR13	2.3782E-06	0.10484362	0.401	0.6	0.0365549	Epi
MON23	2.3898E-06	0.15759652	0.228	0.377	0.03673438	Epi
SPRYD43	2.4033E-06	0.12114308	0.122	0.233	0.03694162	Epi
SLC38A94	2.4621E-06	0.23210572	0.127	0.232	0.03784561	Epi
APBA21	2.4634E-06	1.21062077	0.01	0.001	0.03786447	Epi
PCED1A2	2.4847E-06	0.22699588	0.343	0.523	0.03819291	Epi
OBSCN3	2.4948E-06	1.1964923	0.053	0.02	0.03834775	Epi
TMEM1441	2.5105E-06	0.14022013	0.185	0.309	0.03858938	Epi
PSMC62	2.5628E-06	0.11493216	0.259	0.405	0.03939233	Epi
ZNF7091	2.5719E-06	2.04196757	0.122	0.067	0.03953224	Epi
MAPK133	2.6174E-06	0.26302964	0.282	0.445	0.04023206	Epi
EID11	2.7193E-06	0.23811059	0.551	0.795	0.04179893	Epi
UBE2K2	2.7391E-06	0.26486392	0.195	0.328	0.04210296	Epi
SMARCC21	2.7884E-06	0.26845361	0.249	0.395	0.04286064	Epi
U2AF1L43	2.8112E-06	0.30123626	0.145	0.26	0.04321109	Epi
NPRL31	2.8503E-06	0.3596729	0.256	0.416	0.04381172	Epi
ZNRD22	2.8742E-06	0.36880734	0.18	0.305	0.04417954	Epi
ABAT3	2.875E-06	0.2649196	0.234	0.376	0.04419099	Epi
XPO14	2.8817E-06	0.11293366	0.244	0.389	0.04429397	Epi
SPIDR2	2.9108E-06	0.32824804	0.335	0.511	0.04474119	Epi
TRIM16L	2.9373E-06	4.52633493	0.018	0.004	0.04514958	Epi
ERGIC13	2.9559E-06	0.2953551	0.332	0.508	0.04543563	Epi
IFFO24	2.9896E-06	1.21749336	0.193	0.125	0.04595294	Epi
MORN2	3.0419E-06	2.78960276	0.315	0.266	0.04675695	Epi
PHKB4	3.0625E-06	0.15883071	0.302	0.46	0.04707403	Epi
ZNF518A3	3.0655E-06	0.1610113	0.246	0.399	0.04711946	Epi
RARG3	3.0944E-06	1.61472207	0.084	0.039	0.04756428	Epi
JADE22	3.0948E-06	0.5590628	0.168	0.289	0.04757068	Epi
AKAP17A5	3.1206E-06	0.39251453	0.312	0.486	0.04796726	Epi
DIMT12	3.1571E-06	0.25504606	0.274	0.443	0.04852794	Epi
SLC30A91	3.1952E-06	0.36158356	0.327	0.494	0.04911366	Epi
CCDC341	3.2231E-06	2.16632143	0.094	0.047	0.04954294	Epi
RANBP17	3.3632E-06	3.86277753	0.028	0.008	0.05169555	Epi
ZFP366	3.3736E-06	0.74589292	0.607	0.871	0.05185617	Epi
NUMA11	3.4014E-06	0.21892575	0.586	0.828	0.05228355	Epi
NUDT71	3.4223E-06	2.09622872	0.132	0.075	0.05260429	Epi
SNAP234	3.4938E-06	0.1217618	0.228	0.372	0.05370324	Epi
TARBP21	3.4991E-06	0.36238886	0.145	0.257	0.0537854	Epi
PIP4K2C1	3.5147E-06	0.10249322	0.373	0.578	0.05402405	Epi
B4GALT52	3.523E-06	0.26658249	0.419	0.614	0.05415248	Epi
ACP12	3.6656E-06	0.30655454	0.216	0.356	0.05634356	Epi
CHERP2	3.6681E-06	0.13440702	0.294	0.454	0.05638194	Epi
CYB561A33	3.7164E-06	0.22148642	0.292	0.463	0.05712435	Epi
ACY1	3.7968E-06	0.22267911	0.107	0.209	0.05836094	Epi
RNASE12	3.8082E-06	0.76106403	0.096	0.047	0.05853602	Epi
HIF1AN2	3.8639E-06	0.10584096	0.241	0.387	0.05939244	Epi

FAM114A11	3.8693E-06	1.69169295	0.198	0.133	0.05947469	Epi
USP111	3.9324E-06	0.17217049	0.322	0.5	0.06044527	Epi
SP21	3.9476E-06	0.15150031	0.244	0.377	0.06067842	Epi
TMEM192	3.955E-06	0.12146996	0.16	0.284	0.06079295	Epi
CLNS1A2	4.0782E-06	0.17480422	0.282	0.448	0.06268637	Epi
CFDP1	4.104E-06	0.21512122	0.416	0.645	0.06308203	Epi
ACACA2	4.1175E-06	1.85309997	0.198	0.13	0.06328993	Epi
PICK13	4.1214E-06	0.29541228	0.241	0.384	0.06335006	Epi
PRMT11	4.2136E-06	0.17910595	0.289	0.443	0.06476798	Epi
RBM15B1	4.356E-06	0.39223354	0.406	0.613	0.06695639	Epi
RBBP92	4.373E-06	0.158861	0.168	0.285	0.06721745	Epi
JADE11	4.4051E-06	1.23197918	0.188	0.12	0.06771087	Epi
PIK3IP11	4.4363E-06	1.74957786	0.178	0.113	0.06819001	Epi
SF13	4.4415E-06	0.27322109	0.264	0.41	0.0682702	Epi
PATL21	4.4576E-06	2.35972026	0.079	0.036	0.0685178	Epi
NFIA1	4.4927E-06	1.78691165	0.335	0.274	0.06905681	Epi
JAGN12	4.5161E-06	0.20390931	0.165	0.287	0.06941645	Epi
EPB41L11	4.5288E-06	0.16356719	0.495	0.71	0.06961245	Epi
WDR742	4.5373E-06	0.29684494	0.17	0.289	0.06974334	Epi
SAP30BP3	4.5548E-06	0.10966823	0.274	0.424	0.07001244	Epi
GALE	4.6854E-06	0.48447098	0.239	0.384	0.07201882	Epi
CALM2	4.7101E-06	0.13370667	0.655	0.916	0.07239901	Epi
EPHX11	4.7197E-06	1.39078869	0.657	0.797	0.07254634	Epi
ZBTB142	4.7275E-06	0.26610772	0.188	0.317	0.07266619	Epi
SYMPK4	4.787E-06	0.23964037	0.437	0.635	0.07358086	Epi
DCBLD2	4.8091E-06	2.19446941	0.137	0.081	0.0739213	Epi
ST6GALNAC63	4.8264E-06	0.15435175	0.533	0.769	0.07418695	Epi
MAP2K53	4.8706E-06	0.15679153	0.114	0.217	0.07486554	Epi
CCNC3	4.9748E-06	0.12588403	0.261	0.403	0.07646716	Epi
ATG2A2	5.0656E-06	0.13401239	0.162	0.272	0.07786358	Epi
GPT21	5.1508E-06	2.37227211	0.117	0.064	0.07917256	Epi
ANXA91	5.1998E-06	4.01537	0.041	0.014	0.07992664	Epi
FBXL122	5.2681E-06	0.23568009	0.142	0.253	0.08097671	Epi
PRRC2C4	5.3053E-06	0.10951302	0.462	0.664	0.08154776	Epi
FBXO223	5.3804E-06	0.28335689	0.226	0.363	0.08270139	Epi
PDCD111	5.403E-06	0.12499888	0.206	0.333	0.08304972	Epi
LGALS84	5.4092E-06	0.13066302	0.485	0.707	0.08314539	Epi
WDR202	5.4107E-06	0.1851211	0.17	0.29	0.08316737	Epi
NDUFV3	5.4791E-06	0.10538194	0.475	0.699	0.08421919	Epi
IL17RC1	5.5723E-06	0.1787427	0.244	0.385	0.08565247	Epi
TVP23C2	5.7076E-06	2.00654007	0.043	0.015	0.08773082	Epi
CFLAR5	5.7964E-06	0.71821242	0.721	0.876	0.08909655	Epi
DEPP12	5.8485E-06	5.47140791	0.02	0.005	0.08989737	Epi
VTI1A3	5.9577E-06	0.11112576	0.211	0.351	0.09157629	Epi
SPTY2D12	6.0353E-06	0.15712377	0.277	0.431	0.09276803	Epi
FAM53B2	6.1492E-06	0.27291794	0.266	0.426	0.09451957	Epi
NFKBIL11	6.225E-06	0.27212661	0.165	0.281	0.09568509	Epi
ENSA1	6.2423E-06	0.39238947	0.495	0.728	0.09595088	Epi
SUCLG21	6.3164E-06	0.19160688	0.327	0.488	0.09708896	Epi
ZC3H12A5	6.3956E-06	0.22757739	0.17	0.292	0.09830731	Epi
C11orf11	6.416E-06	0.46286977	0.147	0.262	0.0986208	Epi
LZTFL11	6.4281E-06	1.70179437	0.32	0.266	0.09880588	Epi

MBP2	6.4982E-06	0.1291172	0.365	0.534	0.09988313	Epi
GANC2	6.5081E-06	0.12761821	0.254	0.398	0.10003654	Epi
IDS2	6.6227E-06	0.10071772	0.67	0.903	0.10179715	Epi
DAPK32	6.624E-06	0.51818477	0.269	0.433	0.10181726	Epi
STK32	6.7187E-06	0.27725866	0.221	0.348	0.10327256	Epi
MGAT4B3	6.894E-06	0.18307207	0.457	0.673	0.1059678	Epi
ZNF762	6.9283E-06	0.40754057	0.201	0.327	0.10649422	Epi
CXADR2	7.2086E-06	1.95961856	0.114	0.062	0.11080388	Epi
SHLD13	7.3545E-06	0.4140792	0.203	0.332	0.11304586	Epi
FAM3A1	7.4375E-06	0.27981189	0.185	0.313	0.11432188	Epi
TUBA4A1	7.5206E-06	0.35122562	0.046	0.017	0.11559843	Epi
UBL32	7.6047E-06	0.18974723	0.513	0.755	0.11689234	Epi
DPY19L32	7.64E-06	1.81249254	0.185	0.122	0.11743493	Epi
PCF113	7.6482E-06	0.18388561	0.398	0.59	0.11756106	Epi
STK242	7.67E-06	0.25188314	0.627	0.893	0.11789541	Epi
SNRNP351	7.8197E-06	0.17920975	0.223	0.357	0.12019674	Epi
GGNBP22	7.8994E-06	0.18656264	0.414	0.613	0.12142232	Epi
HSD17B83	8.0158E-06	0.23167175	0.198	0.329	0.12321097	Epi
AGBL51	8.1046E-06	1.85192738	0.226	0.163	0.12457548	Epi
INO80B2	8.1462E-06	1.77780329	0.426	0.415	0.12521594	Epi
ATP7A2	8.1629E-06	0.13274312	0.157	0.27	0.12547156	Epi
SPTLC22	8.2594E-06	0.10918525	0.386	0.565	0.12695509	Epi
SLC39A92	8.3142E-06	0.23327753	0.353	0.524	0.12779719	Epi
PIK3C33	8.3606E-06	0.16040015	0.249	0.383	0.12851099	Epi
ZCCHC31	8.4232E-06	0.27811059	0.239	0.373	0.12947243	Epi
CENPS	8.5388E-06	1.92142827	0.168	0.107	0.13124943	Epi
ZHX12	8.5803E-06	0.18572494	0.231	0.367	0.13188766	Epi
C4orf31	8.5845E-06	0.14323712	0.558	0.814	0.13195203	Epi
IL23A1	8.8363E-06	4.2430664	0.043	0.016	0.13582256	Epi
FPGT1	8.8994E-06	0.24919142	0.211	0.339	0.13679218	Epi
ANAPC21	8.9465E-06	0.37611633	0.198	0.323	0.13751663	Epi
AS3MT2	9.0794E-06	1.83811413	0.02	0.005	0.13955899	Epi
TLE11	9.1304E-06	0.3423635	0.084	0.173	0.14034268	Epi
ASH1L4	9.1552E-06	0.12990177	0.411	0.602	0.14072469	Epi
CCDC323	9.2159E-06	0.14138289	0.256	0.395	0.14165811	Epi
XBP12	9.2202E-06	0.68196809	0.52	0.761	0.14172398	Epi
SNN2	9.3647E-06	0.10710813	0.043	0.116	0.14394498	Epi
ATP5ME1	9.4472E-06	0.38479823	0.34	0.527	0.1452129	Epi
IRF2BPL1	9.5247E-06	0.15187617	0.305	0.456	0.1464041	Epi
NT5C22	9.525E-06	0.21590402	0.485	0.722	0.14640858	Epi
MTAP2	9.6312E-06	0.22690044	0.132	0.233	0.14804129	Epi
PACS13	9.6343E-06	0.3476936	0.388	0.593	0.14808905	Epi
MED102	9.6779E-06	0.12729039	0.348	0.507	0.14875905	Epi
GPKOW2	9.7173E-06	0.27663743	0.216	0.344	0.1493649	Epi
CCDC121	9.8855E-06	1.97884406	0.081	0.039	0.15195013	Epi
C15orf622	9.9754E-06	2.86902169	0.063	0.027	0.15333205	Epi
UBN13	1.0033E-05	0.28635534	0.259	0.411	0.15421997	Epi
SPOUT11	1.009E-05	0.34996565	0.152	0.264	0.15509327	Epi
PDZD81	1.015E-05	0.20509339	0.213	0.345	0.15601427	Epi
DDR2	1.0239E-05	3.72388321	0.013	0.002	0.1573776	Epi
DQX1	1.0314E-05	3.95676396	0.018	0.004	0.15854257	Epi
YRDC2	1.0347E-05	0.53284585	0.137	0.243	0.15904404	Epi

SAT14	1.0541E-05	0.89362067	0.843	0.962	0.16202588	Epi
COQ63	1.0654E-05	0.23142084	0.16	0.27	0.16376796	Epi
CAPN14	1.0776E-05	0.17864143	0.589	0.847	0.16563081	Epi
LXN3	1.0791E-05	1.76617011	0.223	0.161	0.16586116	Epi
BTBD3	1.0801E-05	1.78798896	0.236	0.175	0.16602389	Epi
MARCH34	1.0882E-05	2.11677146	0.071	0.033	0.16726733	Epi
NPAT3	1.0967E-05	0.19812291	0.107	0.2	0.16857626	Epi
CREB3L42	1.0969E-05	2.39357828	0.102	0.054	0.16860736	Epi
NUMB3	1.0998E-05	0.23170504	0.553	0.779	0.16905485	Epi
REPS11	1.1159E-05	0.37011728	0.381	0.571	0.17153022	Epi
CARM12	1.1178E-05	0.14918274	0.31	0.466	0.17182282	Epi
ARL14EP1	1.1283E-05	0.17239128	0.17	0.288	0.17342894	Epi
SVIP2	1.1359E-05	1.41184536	0.051	0.02	0.17460462	Epi
RASA22	1.1377E-05	0.22775458	0.206	0.343	0.17487326	Epi
HSPD12	1.1622E-05	0.11675924	0.404	0.593	0.17864191	Epi
PIGB2	1.1641E-05	0.29180246	0.066	0.145	0.17892997	Epi
DGCR23	1.1651E-05	0.18904499	0.208	0.342	0.17909352	Epi
ITCH2	1.1659E-05	0.15631224	0.518	0.749	0.17920918	Epi
DHX153	1.1675E-05	0.12011551	0.503	0.732	0.1794598	Epi
ZNF6442	1.1806E-05	0.12444344	0.414	0.6	0.18146299	Epi
RBFA2	1.1839E-05	0.18757167	0.173	0.288	0.18196982	Epi
PCDHGA11	1.1965E-05	3.70283807	0.01	0.002	0.18391454	Epi
SYTL33	1.2218E-05	1.89460618	0.226	0.167	0.18780472	Epi
CLTB2	1.2254E-05	0.34367442	0.365	0.564	0.18836061	Epi
TLK23	1.2553E-05	0.28741531	0.168	0.28	0.19295298	Epi
RALGAPB2	1.2591E-05	0.23459035	0.34	0.509	0.19354324	Epi
CEP572	1.2636E-05	0.37273137	0.223	0.352	0.19423407	Epi
MTF23	1.2699E-05	0.20431458	0.206	0.327	0.19520162	Epi
XPNPEP1	1.2963E-05	0.3859721	0.294	0.439	0.19925248	Epi
MZF12	1.3161E-05	2.18796243	0.084	0.041	0.20230326	Epi
KHDC43	1.3333E-05	1.20021683	0.297	0.229	0.20493966	Epi
ZNF2762	1.3583E-05	0.45070503	0.17	0.293	0.2087918	Epi
FICD	1.3742E-05	0.40913771	0.168	0.284	0.21123504	Epi
ZNF4141	1.4023E-05	0.18732658	0.157	0.269	0.21554073	Epi
SPOP2	1.4048E-05	0.21992023	0.35	0.528	0.21593675	Epi
TRAPPC83	1.4355E-05	0.26006834	0.294	0.442	0.22064421	Epi
CCP1101	1.4393E-05	1.93513011	0.269	0.217	0.22123657	Epi
RTKN21	1.4399E-05	0.13510848	0.015	0.003	0.22133068	Epi
CSGALNACT13	1.4413E-05	0.16838886	0.018	0.004	0.22154293	Epi
ERCC31	1.444E-05	0.28322251	0.274	0.435	0.22196432	Epi
UVSSA3	1.4485E-05	1.64760447	0.277	0.215	0.2226534	Epi
MARCH74	1.4834E-05	0.16777599	0.553	0.817	0.22802061	Epi
POLR2D1	1.4864E-05	0.25179916	0.193	0.315	0.22847242	Epi
PCMTD13	1.5102E-05	0.18482291	0.525	0.762	0.23213494	Epi
NPLOC43	1.5129E-05	0.23899318	0.548	0.791	0.2325473	Epi
MVD1	1.5305E-05	0.25932766	0.221	0.35	0.23524858	Epi
PIGH1	1.5379E-05	0.18112826	0.104	0.195	0.23638752	Epi
TRIM362	1.5421E-05	0.11520822	0.114	0.212	0.23703856	Epi
SUZ123	1.584E-05	0.23053567	0.264	0.409	0.24347953	Epi
CLOCK3	1.6086E-05	0.11076484	0.185	0.297	0.24725106	Epi
HNRNPH34	1.6201E-05	0.12348352	0.538	0.774	0.24902877	Epi
MAMDC24	1.6425E-05	1.7180892	0.028	0.008	0.25246694	Epi

CRLS11	1.6504E-05	0.15888812	0.401	0.58	0.25367992	Epi
TRIM282	1.6597E-05	0.128619	0.183	0.3	0.25510698	Epi
DNAJB42	1.6886E-05	1.78904513	0.201	0.14	0.25955936	Epi
DNAAF42	1.7445E-05	1.86365657	0.371	0.346	0.26814996	Epi
SEC61G1	1.7486E-05	0.20054148	0.201	0.315	0.26878265	Epi
NXF14	1.7514E-05	0.26940129	0.317	0.477	0.26920469	Epi
PLEKHM33	1.7555E-05	0.1805502	0.122	0.223	0.26983378	Epi
CHPF21	1.7712E-05	0.26997585	0.315	0.462	0.27224819	Epi
CHD41	1.7911E-05	0.11118439	0.358	0.526	0.2753155	Epi
NIF3L11	1.8156E-05	0.41473431	0.129	0.229	0.27907843	Epi
UBR42	1.8187E-05	0.16721464	0.49	0.717	0.27955302	Epi
TSPYL12	1.845E-05	0.25595584	0.33	0.509	0.28359038	Epi
TNKS22	1.8488E-05	0.12550804	0.373	0.55	0.28418195	Epi
TAB24	1.8495E-05	0.1052829	0.396	0.606	0.28428027	Epi
TICAM1	1.8496E-05	1.1301917	0.201	0.327	0.28430287	Epi
RXRB2	1.8511E-05	0.54508527	0.343	0.512	0.28452908	Epi
UBA62	1.8652E-05	0.3260401	0.398	0.599	0.28670591	Epi
EID33	1.8717E-05	1.72629263	0.109	0.059	0.28769983	Epi
SH3GLB21	1.8733E-05	1.5226584	0.414	0.394	0.28795196	Epi
CCDC1262	1.8741E-05	0.51083106	0.107	0.203	0.28806212	Epi
PKP42	1.9002E-05	2.47200154	0.056	0.024	0.29208042	Epi
ERCC6L22	1.9166E-05	0.12140347	0.226	0.359	0.29459647	Epi
RFX12	1.9216E-05	0.22294171	0.193	0.318	0.29536642	Epi
TMEM2032	1.9338E-05	0.53143986	0.157	0.266	0.29724913	Epi
FASTK1	1.939E-05	0.2812099	0.256	0.394	0.29804896	Epi
LTBP42	1.9831E-05	0.12762332	0.132	0.228	0.30482805	Epi
NPTXR1	1.995E-05	3.30223975	0.015	0.003	0.30665694	Epi
VASN1	1.9985E-05	3.55322736	0.033	0.011	0.30718545	Epi
PFAS	2.0024E-05	0.24759137	0.152	0.263	0.30778624	Epi
ANTXR26	2.0062E-05	0.16988504	0.061	0.026	0.30837615	Epi
MT-ND66	2.0233E-05	2.48854103	0.213	0.152	0.31100132	Epi
ASCC33	2.0323E-05	0.16066113	0.221	0.351	0.31238736	Epi
LRRC37B2	2.0392E-05	1.33859222	0.129	0.075	0.3134472	Epi
PALB23	2.0442E-05	0.37734693	0.074	0.155	0.31421886	Epi
PPM1A3	2.0518E-05	0.22093905	0.388	0.574	0.31537999	Epi
C16orf861	2.0641E-05	0.1594261	0.051	0.123	0.31726556	Epi
CYP20A12	2.0895E-05	0.18894186	0.223	0.354	0.3211764	Epi
MST12	2.0927E-05	1.97377493	0.081	0.04	0.32167348	Epi
KAT71	2.1166E-05	0.23233539	0.317	0.483	0.32534381	Epi
MEST1	2.1204E-05	2.69182113	0.02	0.005	0.32592305	Epi
HIBADH2	2.1253E-05	0.25464801	0.338	0.51	0.32668727	Epi
B3GNT53	2.1269E-05	0.31232009	0.459	0.658	0.32692305	Epi
MCMDC2	2.1575E-05	3.19232835	0.025	0.007	0.33163026	Epi
UFL13	2.1602E-05	0.30634803	0.256	0.39	0.33204681	Epi
TBC1D72	2.161E-05	0.68694592	0.175	0.287	0.33216646	Epi
CCNG22	2.1624E-05	0.31506725	0.294	0.448	0.33237553	Epi
TULP32	2.1738E-05	1.67334978	0.14	0.084	0.3341316	Epi
ALDH1A11	2.1967E-05	0.88978091	0.766	0.906	0.33764957	Epi
BCCIP4	2.1989E-05	0.23423889	0.388	0.571	0.3379967	Epi
GARS3	2.2011E-05	0.22363237	0.15	0.248	0.33833262	Epi
MPLKIP1	2.2085E-05	0.33065318	0.234	0.369	0.33946965	Epi
PTCH1	2.2452E-05	3.24370634	0.03	0.01	0.34510217	Epi

PHF20L14	2.2621E-05	0.20858565	0.317	0.471	0.34770917	Epi
TMEM912	2.2667E-05	0.35741105	0.147	0.249	0.34841294	Epi
PRKAR2A3	2.3128E-05	0.17294642	0.287	0.436	0.3554953	Epi
RBM254	2.3345E-05	0.21402607	0.475	0.686	0.35883779	Epi
RMND5A3	2.3405E-05	0.42847212	0.434	0.638	0.35975193	Epi
FBXO112	2.3566E-05	0.2350134	0.312	0.466	0.36222714	Epi
SF3B13	2.41E-05	0.32367177	0.289	0.434	0.37043673	Epi
CIZ13	2.4173E-05	0.33929962	0.16	0.269	0.37156406	Epi
CASP23	2.4201E-05	0.1141839	0.17	0.279	0.37199324	Epi
SNRNP2005	2.4419E-05	0.32190044	0.541	0.76	0.37535082	Epi
LSG12	2.4488E-05	0.22513894	0.297	0.444	0.37640614	Epi
RAB9B	2.4532E-05	3.98028531	0.013	0.002	0.37708195	Epi
VWA83	2.4774E-05	0.23570074	0.244	0.379	0.38080809	Epi
GPRASP11	2.4847E-05	1.84392022	0.041	0.015	0.38192749	Epi
ELK35	2.5022E-05	1.79742147	0.117	0.068	0.38461995	Epi
SCAF12	2.5034E-05	0.34867068	0.365	0.548	0.38480492	Epi
RTCA2	2.5111E-05	0.16555671	0.305	0.444	0.3859844	Epi
TOP2B3	2.525E-05	0.10953917	0.193	0.319	0.38812289	Epi
STXBP42	2.5284E-05	1.55526361	0.16	0.103	0.38864782	Epi
OTUD52	2.54E-05	0.31132163	0.371	0.55	0.3904208	Epi
BUD312	2.5546E-05	0.19386108	0.34	0.514	0.39266641	Epi
CFAP298	2.5644E-05	1.70967075	0.49	0.507	0.39417339	Epi
RAPGEF32	2.5745E-05	0.13723933	0.114	0.208	0.39571955	Epi
AKT21	2.6065E-05	0.27817106	0.348	0.524	0.40063855	Epi
MDM13	2.6431E-05	1.62678066	0.231	0.172	0.40627154	Epi
ELP61	2.6525E-05	0.3790464	0.201	0.314	0.40772011	Epi
KLC11	2.7293E-05	0.11094469	0.378	0.564	0.41951643	Epi
LRP121	2.7449E-05	0.35781471	0.079	0.161	0.42191692	Epi
PPP1R82	2.7597E-05	0.4747671	0.241	0.37	0.42419596	Epi
ZCCHC41	2.7699E-05	0.15803374	0.168	0.275	0.42576691	Epi
TERF13	2.77E-05	0.17046475	0.132	0.233	0.42578189	Epi
QPCTL2	2.792E-05	0.1392543	0.145	0.243	0.42916304	Epi
EXOSC82	2.7941E-05	0.32325356	0.193	0.316	0.42947909	Epi
PARP62	2.82E-05	0.14180739	0.155	0.256	0.4334568	Epi
MED13L6	2.8372E-05	0.32691521	0.261	0.397	0.43609879	Epi
ABL13	2.8395E-05	0.22274867	0.223	0.351	0.43646175	Epi
INPP5B4	2.8747E-05	0.13314849	0.31	0.456	0.44186255	Epi
NGLY11	2.8816E-05	0.14103194	0.201	0.313	0.44293284	Epi
SGSH4	2.9236E-05	0.36568142	0.16	0.265	0.44939267	Epi
TCF253	2.9454E-05	0.13703666	0.594	0.844	0.45273412	Epi
BACE21	2.9871E-05	1.40280028	0.178	0.117	0.45914304	Epi
GNL33	3E-05	0.33962043	0.368	0.543	0.46112976	Epi
RMDN11	3.0002E-05	0.14443695	0.307	0.457	0.46116322	Epi
MTERF33	3.0207E-05	0.21220603	0.14	0.237	0.46430455	Epi
DPH53	3.0273E-05	0.20607634	0.119	0.215	0.46532591	Epi
CEP892	3.0498E-05	1.8762014	0.234	0.177	0.46877736	Epi
GRWD12	3.0596E-05	0.43276802	0.228	0.355	0.47028768	Epi
BCL35	3.0824E-05	0.45135231	0.287	0.438	0.473794	Epi
BRCC31	3.118E-05	0.61066512	0.119	0.217	0.47927057	Epi
SMARCD13	3.1319E-05	0.48723803	0.208	0.326	0.48141026	Epi
ANXA43	3.1736E-05	0.23314235	0.607	0.861	0.48781924	Epi
FDPS2	3.1902E-05	0.28611997	0.142	0.238	0.49036794	Epi

S100PBP2	3.2428E-05	0.14809521	0.226	0.349	0.49845522	Epi
ARFIP24	3.3041E-05	1.3041393	0.34	0.291	0.5078678	Epi
PCDHGB6	3.4226E-05	4.212153	0.02	0.005	0.52608846	Epi
ICAM35	3.439E-05	0.22917148	0.114	0.205	0.52861275	Epi
VTA11	3.445E-05	0.1644784	0.33	0.479	0.52952457	Epi
SLC1A2	3.4509E-05	2.84943377	0.023	0.006	0.53043291	Epi
KANSL32	3.4734E-05	0.31263766	0.254	0.391	0.53389245	Epi
RPUSD12	3.4836E-05	0.22909347	0.114	0.205	0.53545804	Epi
PLEKHB12	3.5121E-05	2.31815169	0.249	0.195	0.53984704	Epi
SLC16A74	3.5426E-05	0.67446288	0.043	0.016	0.54452936	Epi
GPSM14	3.5484E-05	2.47658484	0.015	0.003	0.54542516	Epi
TAF1D4	3.5666E-05	0.23296548	0.34	0.489	0.54822571	Epi
IL4R3	3.6081E-05	0.2186417	0.107	0.195	0.55460209	Epi
IFT202	3.6117E-05	0.23765045	0.33	0.486	0.55515993	Epi
EPS15L13	3.6635E-05	0.12860201	0.254	0.382	0.56312223	Epi
KMT2E4	3.7472E-05	0.16738195	0.452	0.654	0.57598441	Epi
AP5S11	3.763E-05	0.2106373	0.145	0.245	0.57841541	Epi
APAF16	3.7771E-05	0.58327288	0.18	0.291	0.58057501	Epi
ALDH6A11	3.7897E-05	0.3301165	0.109	0.197	0.58250777	Epi
SAT22	3.8223E-05	0.23429751	0.414	0.621	0.5875234	Epi
C2orf741	3.8311E-05	0.33861003	0.261	0.403	0.58888319	Epi
NEMF2	3.8622E-05	0.10588457	0.378	0.564	0.59365713	Epi
STX24	3.9092E-05	1.61434046	0.381	0.347	0.60088149	Epi
TBCD2	3.9172E-05	0.16841749	0.287	0.431	0.60211506	Epi
STRN33	3.9326E-05	0.21094736	0.251	0.38	0.60448644	Epi
STAG24	3.9374E-05	0.14932269	0.421	0.61	0.60521789	Epi
ZNF7863	3.9698E-05	0.644448	0.091	0.175	0.61019087	Epi
NIT21	3.9896E-05	0.38280142	0.251	0.393	0.6132362	Epi
REXO22	4.0466E-05	0.2666025	0.543	0.767	0.62200026	Epi
TRIM691	4.0873E-05	4.3174172	0.01	0.002	0.62825752	Epi
TNXB	4.1426E-05	2.16288904	0.015	0.003	0.63675409	Epi
UPF3A3	4.1433E-05	1.5232922	0.256	0.196	0.63686479	Epi
PHF5A2	4.1571E-05	0.46225755	0.343	0.496	0.63899212	Epi
LNX22	4.2303E-05	1.40252522	0.152	0.096	0.65024688	Epi
C16orf872	4.2774E-05	0.6884968	0.094	0.177	0.65748342	Epi
OLFML2A1	4.2779E-05	2.67505018	0.051	0.021	0.6575606	Epi
SFPQ2	4.3237E-05	0.23207704	0.211	0.329	0.66459435	Epi
SEMA3C1	4.3347E-05	0.31574137	0.452	0.662	0.66628072	Epi
PRPF38A4	4.3415E-05	0.78169857	0.272	0.406	0.66733379	Epi
TET34	4.3502E-05	0.23227968	0.201	0.312	0.66867434	Epi
ZNF3172	4.3572E-05	0.1497525	0.147	0.242	0.66973885	Epi
CCT33	4.3843E-05	0.53448986	0.231	0.358	0.67390863	Epi
HMGCS2	4.386E-05	3.9926655	0.01	0.002	0.6741675	Epi
MACROD1	4.412E-05	1.67765562	0.234	0.173	0.67816759	Epi
MAFF3	4.4144E-05	1.19124365	0.206	0.33	0.67853281	Epi
SEC16A1	4.4314E-05	0.28143803	0.419	0.621	0.68115066	Epi
TRMT10C2	4.5055E-05	0.18037292	0.162	0.265	0.69253729	Epi
DNAJC54	4.5767E-05	0.31875728	0.513	0.732	0.70348108	Epi
CANT13	4.6654E-05	0.45412345	0.345	0.498	0.71711631	Epi
WBP1L1	4.6998E-05	0.53998512	0.307	0.457	0.7224014	Epi
DLG42	4.7727E-05	0.55937481	0.19	0.3	0.73360426	Epi
WDR5B2	4.8097E-05	1.52126151	0.152	0.098	0.73930095	Epi

SCRN15	4.811E-05	1.52875238	0.221	0.161	0.73949785	Epi
VPS533	4.8741E-05	0.29387776	0.363	0.521	0.74919409	Epi
AKIRIN12	4.9032E-05	0.15954088	0.386	0.561	0.75367113	Epi
MKS11	4.9628E-05	2.20121876	0.168	0.112	0.76283114	Epi
TRAPPC6B3	4.9691E-05	0.4098245	0.203	0.315	0.7638006	Epi
FAM210B2	5.0197E-05	0.21101175	0.241	0.373	0.77158458	Epi
CCDC152	5.1275E-05	2.40610279	0.025	0.008	0.78815521	Epi
COX203	5.1582E-05	0.24066111	0.107	0.195	0.79287123	Epi
GHRL3	5.1826E-05	2.72364864	0.013	0.002	0.79662437	Epi
MAEA3	5.2461E-05	0.31007203	0.398	0.586	0.80638121	Epi
ZFX1	5.2632E-05	0.2375174	0.114	0.202	0.8090078	Epi
SPEN2	5.3032E-05	0.37113011	0.391	0.562	0.81515197	Epi
FKRP2	5.3094E-05	1.59741991	0.157	0.104	0.81611423	Epi
GDA1	5.3633E-05	2.85440929	0.081	0.042	0.82438974	Epi
ACSF32	5.4101E-05	0.21172468	0.236	0.365	0.83158768	Epi
ZNF6382	5.4244E-05	0.26847149	0.371	0.551	0.83378016	Epi
SLC25A33	5.4374E-05	0.24172111	0.117	0.209	0.83577748	Epi
UBXN42	5.5013E-05	0.16869952	0.576	0.815	0.84560408	Epi
ATG123	5.6101E-05	0.21165013	0.206	0.316	0.86232383	Epi
NPEPL12	5.6305E-05	0.38707434	0.419	0.61	0.86546694	Epi
NPIPB152	5.659E-05	1.97699553	0.071	0.035	0.86984176	Epi
ME32	5.7228E-05	0.36754112	0.135	0.231	0.87964905	Epi
FAAP24	5.723E-05	0.1736901	0.041	0.103	0.87968506	Epi
NR1D23	5.7291E-05	0.17811497	0.404	0.571	0.88061853	Epi
PPP1R13B3	5.8677E-05	1.67671721	0.218	0.159	0.90192802	Epi
PEAK15	5.8696E-05	1.53640072	0.269	0.216	0.90222013	Epi
MTFR1L3	5.9154E-05	0.28653457	0.272	0.409	0.90925596	Epi
ERRFI11	5.988E-05	0.67097487	0.246	0.373	0.92041163	Epi
CDK64	6.0159E-05	0.56827375	0.043	0.017	0.92470067	Epi
TMEM9	6.0174E-05	0.58529783	0.16	0.262	0.92493527	Epi
ANKRD123	6.0212E-05	0.27727026	0.266	0.388	0.92552467	Epi
RIOX22	6.0239E-05	0.38799867	0.289	0.425	0.92593808	Epi
NOP581	6.1548E-05	0.11373488	0.241	0.368	0.94605283	Epi
TRANK11	6.2121E-05	0.26903811	0.157	0.266	0.95486214	Epi
GORASP12	6.2778E-05	0.25516636	0.343	0.499	0.96495387	Epi
CDC42BPA1	6.2814E-05	1.91115578	0.173	0.118	0.9655125	Epi
ZNF5761	6.3205E-05	0.60514634	0.109	0.196	0.97152625	Epi
ZDHHC11B	6.3226E-05	3.49562677	0.023	0.007	0.97184511	Epi
SLC16A132	6.3381E-05	1.91965931	0.084	0.044	0.97422178	Epi
GTF2H13	6.3415E-05	0.39713349	0.343	0.513	0.97474519	Epi
TTC173	6.3581E-05	0.26777788	0.312	0.453	0.97729721	Epi
RRH	6.3695E-05	2.11705234	0.013	0.003	0.97905518	Epi
RAB40B1	6.3832E-05	3.0948039	0.033	0.012	0.9811555	Epi
ING43	6.3943E-05	0.1576968	0.17	0.274	0.98286645	Epi
C4orf33	6.4913E-05	0.38044871	0.157	0.259	0.99777379	Epi
NKIRAS12	6.5376E-05	0.42028448	0.211	0.332	1	Epi
CBX82	6.5952E-05	0.32691265	0.079	0.156	1	Epi
TAF42	6.6331E-05	0.75125656	0.259	0.395	1	Epi
EIF2B52	6.6384E-05	0.19769506	0.261	0.387	1	Epi
EID2B2	6.6534E-05	1.99370553	0.056	0.025	1	Epi
SNX181	6.6848E-05	0.48740686	0.147	0.246	1	Epi
NKAPD13	6.749E-05	0.16549051	0.218	0.335	1	Epi

CLK32	6.8435E-05	0.40889261	0.249	0.386	1	Epi
SMYD42	6.8564E-05	0.16109663	0.109	0.196	1	Epi
ACTR1B1	6.8688E-05	0.33840872	0.495	0.707	1	Epi
EML22	6.8777E-05	0.19113144	0.335	0.507	1	Epi
DZIP1L2	6.9225E-05	2.0556895	0.424	0.443	1	Epi
ACBD63	6.9442E-05	0.254595	0.254	0.382	1	Epi
UBR52	7.0514E-05	0.2658122	0.396	0.578	1	Epi
TBC1D145	7.1003E-05	0.28739078	0.307	0.451	1	Epi
PIN11	7.2013E-05	0.30574619	0.376	0.547	1	Epi
CCDC1832	7.2134E-05	2.44221426	0.02	0.006	1	Epi
PRDM51	7.2135E-05	3.04016819	0.033	0.012	1	Epi
C22orf393	7.2247E-05	0.11539892	0.122	0.209	1	Epi
HNRNPR3	7.2264E-05	0.18252076	0.223	0.346	1	Epi
MICAL31	7.231E-05	1.35121363	0.142	0.091	1	Epi
YTHDF13	7.2324E-05	0.26318533	0.109	0.191	1	Epi
PITRM11	7.2877E-05	0.26114944	0.32	0.472	1	Epi
ENDOV3	7.4178E-05	1.85675964	0.168	0.111	1	Epi
MLYCD1	7.4185E-05	0.21864547	0.195	0.306	1	Epi
GPANK1	7.4735E-05	0.17056643	0.18	0.288	1	Epi
ANKLE23	7.4892E-05	0.12182577	0.365	0.529	1	Epi
DNHD13	7.4954E-05	1.33085562	0.132	0.082	1	Epi
CR1L1	7.5208E-05	3.36180707	0.036	0.013	1	Epi
CCDC9B2	7.5466E-05	0.21663579	0.094	0.174	1	Epi
SBDS3	7.5592E-05	0.49578302	0.124	0.215	1	Epi
C2CD2L1	7.5681E-05	0.21308081	0.345	0.499	1	Epi
VSIR5	7.6062E-05	0.46762602	0.208	0.325	1	Epi
OGT5	7.663E-05	0.22281898	0.371	0.549	1	Epi
TMCC12	7.6735E-05	0.33737616	0.15	0.25	1	Epi
ATMIN3	7.6852E-05	0.249996	0.226	0.359	1	Epi
XPA3	7.7435E-05	0.10498299	0.223	0.338	1	Epi
PROS11	7.8087E-05	0.56720714	0.256	0.398	1	Epi
HMG20A2	7.8451E-05	0.16934283	0.218	0.331	1	Epi
SPECC13	7.8489E-05	2.21856987	0.086	0.047	1	Epi
PRKD33	7.908E-05	0.11984595	0.236	0.354	1	Epi
AJM11	7.9837E-05	3.18860526	0.013	0.003	1	Epi
ZNF5773	8.1203E-05	0.18479536	0.018	0.069	1	Epi
CRYBG32	8.1618E-05	0.31589211	0.127	0.218	1	Epi
GUCA1B2	8.2276E-05	2.4991893	0.03	0.01	1	Epi
NFAT54	8.2312E-05	1.24095888	0.416	0.386	1	Epi
XPNPEP32	8.3237E-05	1.93781129	0.15	0.096	1	Epi
ZRANB12	8.4965E-05	0.40863669	0.284	0.422	1	Epi
STRN2	8.5308E-05	0.34602874	0.371	0.544	1	Epi
DOHH	8.574E-05	0.25643621	0.152	0.245	1	Epi
AVL94	8.618E-05	0.35163519	0.302	0.44	1	Epi
COPB2	8.6423E-05	0.15571783	0.546	0.777	1	Epi
DMD2	8.6717E-05	2.3225236	0.109	0.064	1	Epi
ZBTB103	8.7194E-05	0.52520183	0.168	0.269	1	Epi
C7orf61	8.7278E-05	2.93345064	0.02	0.006	1	Epi
ZFP36L15	8.7569E-05	1.26064872	0.53	0.557	1	Epi
MYBBP1A3	8.8747E-05	0.38042284	0.297	0.434	1	Epi
C9orf724	8.9594E-05	0.11827134	0.429	0.618	1	Epi
TBC1D201	8.9624E-05	0.10544574	0.383	0.549	1	Epi

SPRY24	8.9681E-05	1.7000516	0.038	0.014	1	Epi
LTN12	9.0282E-05	0.19568232	0.264	0.402	1	Epi
MBTD12	9.0792E-05	0.55127302	0.183	0.289	1	Epi
ARMC71	9.1029E-05	0.15717584	0.089	0.165	1	Epi
FER3	9.2067E-05	0.40080908	0.302	0.447	1	Epi
SMARCA42	9.2276E-05	0.35098829	0.434	0.644	1	Epi
SEC24A4	9.2911E-05	0.18232867	0.294	0.439	1	Epi
MIS18A1	9.3933E-05	0.11229881	0.099	0.18	1	Epi
DCAF172	9.4012E-05	0.13773168	0.119	0.206	1	Epi
OAT1	9.4437E-05	0.48917474	0.17	0.277	1	Epi
SMIM10L12	9.515E-05	0.51316718	0.124	0.215	1	Epi
PLEKHN14	9.5736E-05	1.30143344	0.03	0.01	1	Epi
SELENOO2	9.8324E-05	0.31043334	0.231	0.355	1	Epi
DNAJC242	9.8781E-05	0.23849755	0.102	0.186	1	Epi
TMEM2592	9.9536E-05	0.26890924	0.543	0.771	1	Epi
RMDN22	9.9674E-05	0.20126481	0.096	0.174	1	Epi
SLC11A22	0.00010007	0.30545047	0.434	0.613	1	Epi
KANK21	0.00010066	2.11210609	0.033	0.012	1	Epi
TSPAN171	0.00010066	0.40964831	0.165	0.262	1	Epi
ABCD43	0.00010147	0.26156012	0.162	0.264	1	Epi
TRIM310	0.00010169	1.74937418	0.18	0.125	1	Epi
NR1D11	0.00010169	3.21303417	0.162	0.109	1	Epi
MBOAT72	0.00010178	0.41932198	0.36	0.519	1	Epi
SMARCD24	0.00010182	0.26228696	0.401	0.57	1	Epi
ATG52	0.00010345	0.20765401	0.33	0.478	1	Epi
ANAPC52	0.00010345	0.1592362	0.437	0.625	1	Epi
TRIM334	0.00010347	0.40139365	0.345	0.492	1	Epi
NFATC24	0.00010397	0.3688691	0.094	0.051	1	Epi
PHF133	0.0001043	0.30423408	0.112	0.193	1	Epi
TNRC183	0.0001056	0.31334105	0.322	0.463	1	Epi
PPDPF1	0.00010612	0.80639426	0.591	0.84	1	Epi
GSTZ1	0.00010646	0.45749196	0.109	0.196	1	Epi
CRELD22	0.00010675	0.24400474	0.332	0.494	1	Epi
ADAM202	0.00010719	1.56364671	0.033	0.012	1	Epi
LYPD3	0.00010791	5.064517	0.015	0.004	1	Epi
ZNF1214	0.00010804	0.18092455	0.213	0.325	1	Epi
NAT92	0.0001104	0.50085337	0.201	0.309	1	Epi
CLEC18A2	0.00011117	2.03790277	0.033	0.012	1	Epi
SLX43	0.00011181	0.14030323	0.071	0.141	1	Epi
LPIN31	0.00011213	2.14747867	0.066	0.032	1	Epi
GOLGA32	0.0001124	0.17520238	0.33	0.472	1	Epi
CLSTN13	0.00011294	1.18815253	0.462	0.469	1	Epi
ZNF5842	0.00011503	1.84578099	0.096	0.055	1	Epi
SORL14	0.00011548	0.74745604	0.292	0.42	1	Epi
SLC35A11	0.00011655	0.47590586	0.332	0.491	1	Epi
TSTA31	0.00011662	0.5002286	0.386	0.565	1	Epi
TP53I132	0.00011754	0.32376597	0.107	0.187	1	Epi
RDH51	0.0001177	2.19564266	0.038	0.015	1	Epi
PPIE3	0.00011797	0.26094176	0.299	0.441	1	Epi
ZFAND2A3	0.0001183	0.67608536	0.162	0.26	1	Epi
FAM122B3	0.00011897	0.57472258	0.244	0.364	1	Epi
KIFC24	0.00011929	1.68703453	0.162	0.11	1	Epi

CASP71	0.00011933	0.20075242	0.414	0.594	1	Epi
ZGPAT2	0.00011943	0.15273734	0.431	0.601	1	Epi
UROS3	0.00012177	0.34025888	0.228	0.349	1	Epi
PRKAA12	0.00012295	0.24863939	0.447	0.633	1	Epi
CREB31	0.00012482	0.22926493	0.226	0.35	1	Epi
TBCE.13	0.00012542	0.16581791	0.089	0.162	1	Epi
WDR921	0.00012546	1.98990019	0.19	0.14	1	Epi
MYO15B6	0.00012608	1.54231703	0.284	0.234	1	Epi
RSAD24	0.00012621	0.41089857	0.122	0.206	1	Epi
GIGYF22	0.00012709	0.16465926	0.218	0.323	1	Epi
ZFP2	0.00012754	1.90203049	0.013	0.003	1	Epi
CXCL81	0.00012792	1.31301074	0.157	0.103	1	Epi
RABIF2	0.00012883	0.39111005	0.213	0.325	1	Epi
ESS23	0.00012991	0.2069762	0.122	0.207	1	Epi
SARM11	0.00012996	0.1605973	0.102	0.18	1	Epi
IMPG2	0.00012997	4.10225081	0.01	0.002	1	Epi
RNF252	0.00013144	0.19875575	0.18	0.28	1	Epi
CDK124	0.00013149	0.2929771	0.249	0.375	1	Epi
LYSMD33	0.00013192	0.27050329	0.249	0.375	1	Epi
CRYZ3	0.00013203	0.21918008	0.165	0.262	1	Epi
CYP4V22	0.00013351	0.3339563	0.312	0.46	1	Epi
AC073111.43	0.00013414	2.00169904	0.069	0.035	1	Epi
HSH2D5	0.00013492	1.16675029	0.107	0.062	1	Epi
LY6K	0.00013619	2.62036058	0.025	0.008	1	Epi
PTGES22	0.00013699	0.45792149	0.322	0.463	1	Epi
RAB3GAP12	0.00013799	0.22868222	0.414	0.585	1	Epi
STAP11	0.00013815	1.67822336	0.036	0.092	1	Epi
WDFY35	0.00013999	0.14162202	0.279	0.405	1	Epi
HERC43	0.00014055	0.11505561	0.371	0.516	1	Epi
GLIPR1L22	0.00014077	2.63417452	0.046	0.019	1	Epi
CDC261	0.00014283	0.23811817	0.216	0.329	1	Epi
MIEF21	0.00014365	1.77453986	0.173	0.12	1	Epi
PCBP41	0.00014472	2.09341423	0.028	0.009	1	Epi
SNAPC5	0.00014496	0.11899469	0.173	0.27	1	Epi
FAM3C2	0.00014568	0.28344951	0.378	0.549	1	Epi
PAGR13	0.00014685	0.17335787	0.305	0.447	1	Epi
OSGIN12	0.00014785	2.2039729	0.041	0.016	1	Epi
EIF4EBP33	0.00014879	0.36226213	0.183	0.281	1	Epi
ANKRD522	0.00015084	0.32588383	0.216	0.331	1	Epi
VDAC31	0.00015195	0.45991826	0.363	0.534	1	Epi
PURB2	0.00015197	0.39637955	0.381	0.549	1	Epi
ZNF5941	0.00015222	2.29084523	0.036	0.014	1	Epi
SHF3	0.00015235	2.43327823	0.048	0.021	1	Epi
CCNL13	0.00015389	0.21157219	0.378	0.54	1	Epi
HNRNPDL2	0.00015664	0.19421964	0.477	0.677	1	Epi
C2CD52	0.00015721	0.26926655	0.335	0.486	1	Epi
LENG13	0.00015746	0.16245068	0.188	0.291	1	Epi
MAPK62	0.00015782	0.43611655	0.302	0.459	1	Epi
ABHD62	0.00015791	0.73110205	0.188	0.297	1	Epi
CREBZF3	0.00015889	1.30080988	0.383	0.354	1	Epi
SETD1B4	0.00015984	0.10392586	0.439	0.608	1	Epi
TBL23	0.00015999	0.18881446	0.277	0.396	1	Epi

CNIH31	0.00016107	3.74504572	0.013	0.003	1	Epi
BCL66	0.00016109	1.15705568	0.444	0.449	1	Epi
THAP81	0.00016291	0.2048297	0.033	0.087	1	Epi
MCAT1	0.00016518	1.67311792	0.244	0.19	1	Epi
GAS2	0.00016581	3.25126642	0.015	0.004	1	Epi
COASY	0.00016609	0.36581703	0.31	0.442	1	Epi
DOT1L4	0.00016655	0.31641021	0.234	0.345	1	Epi
BAMBI1	0.00016687	2.02223818	0.071	0.037	1	Epi
ZNF2964	0.00016719	2.55930744	0.079	0.042	1	Epi
ANP32E2	0.00016865	0.15184153	0.16	0.251	1	Epi
RBM151	0.00016914	0.21210319	0.284	0.414	1	Epi
TBCK3	0.00016921	0.11459115	0.203	0.314	1	Epi
FZD42	0.0001697	2.24623828	0.046	0.02	1	Epi
SPOCK1	0.00017091	2.83569809	0.018	0.005	1	Epi
POP52	0.00017105	0.26572805	0.198	0.308	1	Epi
ZBTB8A1	0.00017111	0.22367326	0.048	0.109	1	Epi
ANO82	0.00017181	0.41660736	0.16	0.251	1	Epi
AKR7A21	0.00017957	0.32749553	0.284	0.426	1	Epi
SHANK3	0.00017962	2.35184348	0.013	0.003	1	Epi
NLRP22	0.00018088	0.34238377	0.137	0.222	1	Epi
ARNT4	0.00018168	0.35892953	0.302	0.429	1	Epi
REPS23	0.00018215	1.72777191	0.058	0.028	1	Epi
XIAP3	0.00018657	0.15376212	0.269	0.389	1	Epi
ZBED41	0.00019182	0.13074408	0.185	0.284	1	Epi
KANSL14	0.0001923	0.42197683	0.452	0.638	1	Epi
ZBTB172	0.00019237	0.17563795	0.175	0.274	1	Epi
PDSS21	0.00019293	0.57337117	0.256	0.382	1	Epi
TTLL13P	0.00019455	2.37225957	0.015	0.004	1	Epi
BPTF2	0.00019686	0.14843456	0.388	0.566	1	Epi
MAPK8IP35	0.00019717	1.7196842	0.168	0.117	1	Epi
NOX5	0.0001974	3.89868027	0.013	0.003	1	Epi
ASPSCR13	0.00020174	0.41685788	0.249	0.378	1	Epi
FJX11	0.00020279	2.54812194	0.025	0.008	1	Epi
ZXDC2	0.00020351	0.14396734	0.335	0.481	1	Epi
UBE2E13	0.00020408	0.22170894	0.246	0.364	1	Epi
UFSP23	0.00020443	0.11003373	0.198	0.303	1	Epi
KIAA20262	0.0002064	0.33339773	0.371	0.536	1	Epi
ZNF6552	0.00020972	0.40651299	0.434	0.603	1	Epi
ZNF6753	0.00021086	0.43656084	0.046	0.104	1	Epi
RAD502	0.00021189	0.31172883	0.325	0.475	1	Epi
RBM335	0.00021323	0.25541008	0.528	0.721	1	Epi
SYPL13	0.00021368	0.50431686	0.234	0.343	1	Epi
LDLRAD43	0.00021513	0.2184998	0.297	0.438	1	Epi
MYL93	0.00021634	2.41879544	0.066	0.033	1	Epi
CARF2	0.00021767	1.88787483	0.122	0.077	1	Epi
PNN3	0.00022091	0.25027715	0.254	0.372	1	Epi
TCF7L11	0.00022115	2.35956937	0.048	0.022	1	Epi
ZC3HC13	0.00022129	0.46894649	0.091	0.165	1	Epi
NIPSNAP22	0.000223	0.39352554	0.431	0.629	1	Epi
EIF4A21	0.00022323	0.16661565	0.53	0.737	1	Epi
LIG11	0.00022365	0.24809794	0.183	0.278	1	Epi
CEP2953	0.00022788	0.34591582	0.053	0.114	1	Epi

TTC273	0.00023288	0.38403497	0.17	0.269	1	Epi
MOB23	0.00023291	0.30009156	0.279	0.408	1	Epi
RELL13	0.00023303	0.33331734	0.216	0.325	1	Epi
CAVIN22	0.00023454	2.29406498	0.046	0.02	1	Epi
MAP3K203	0.00023522	0.51561624	0.14	0.227	1	Epi
APP4	0.00023611	0.89649923	0.523	0.527	1	Epi
ARHGEF123	0.00023688	1.51067523	0.216	0.164	1	Epi
POLA22	0.00023725	0.1788187	0.132	0.215	1	Epi
RRBP11	0.00023801	0.2930088	0.541	0.751	1	Epi
C8orf331	0.00023908	0.2521706	0.198	0.295	1	Epi
ASMTL4	0.00023914	0.44859192	0.069	0.135	1	Epi
C19orf252	0.00024161	0.14886672	0.208	0.31	1	Epi
CSNK1A13	0.00024196	0.39520667	0.297	0.446	1	Epi
C12orf101	0.00024263	0.7535352	0.198	0.306	1	Epi
TFG2	0.00024329	0.20936832	0.335	0.481	1	Epi
FGFRL11	0.00024512	0.10425013	0.099	0.176	1	Epi
PLEKHA33	0.00024771	0.26304876	0.277	0.408	1	Epi
SESN14	0.00025111	0.35323291	0.274	0.406	1	Epi
MED243	0.00025289	0.15636549	0.289	0.425	1	Epi
CAMK1D5	0.00025533	0.19074675	0.32	0.463	1	Epi
IP6K12	0.00025749	0.37498175	0.376	0.546	1	Epi
BOD13	0.00025943	0.44260664	0.178	0.272	1	Epi
MED252	0.00026013	1.25179577	0.617	0.759	1	Epi
CDK132	0.0002651	0.51712953	0.338	0.493	1	Epi
TUBB4B	0.0002651	2.05022766	0.508	0.577	1	Epi
NAT103	0.00026606	0.574535	0.213	0.32	1	Epi
EIF2AK11	0.00027066	0.27512823	0.523	0.719	1	Epi
IER5L4	0.00027204	0.7925483	0.122	0.203	1	Epi
BCL2L15	0.0002728	0.26979774	0.51	0.765	1	Epi
ZNF1543	0.0002737	2.04135287	0.038	0.016	1	Epi
MAGED22	0.00027398	1.30096906	0.546	0.645	1	Epi
NFKBIB3	0.00027484	0.37987967	0.162	0.253	1	Epi
SLC30A63	0.00027529	0.30608974	0.19	0.288	1	Epi
AKAP3	0.00027618	2.73377697	0.023	0.007	1	Epi
CCDC1592	0.00027769	0.31792043	0.289	0.42	1	Epi
WDR114	0.00028115	0.16439077	0.317	0.443	1	Epi
DISP12	0.00028162	0.37112779	0.109	0.187	1	Epi
PMM11	0.0002818	1.70824047	0.274	0.227	1	Epi
SURF61	0.00028276	0.20288947	0.145	0.233	1	Epi
SMU13	0.00028343	0.2110959	0.119	0.2	1	Epi
RNPC34	0.00028442	1.47594078	0.218	0.162	1	Epi
ZFP303	0.00028678	0.15765515	0.117	0.198	1	Epi
LPAR13	0.00028747	0.60030416	0.155	0.243	1	Epi
RBM8A3	0.00028836	0.12673953	0.066	0.13	1	Epi
LDLRAP11	0.00028973	0.48667423	0.236	0.355	1	Epi
ILF33	0.00028983	0.27065195	0.52	0.761	1	Epi
MAFK3	0.00029141	0.54285728	0.289	0.418	1	Epi
PALM2-AKAP2	0.00029222	1.29617713	0.122	0.077	1	Epi
ATRX1	0.00029477	0.39652599	0.355	0.509	1	Epi
EVI5L1	0.00029704	0.19555427	0.147	0.232	1	Epi
RBM382	0.00030083	1.43350132	0.16	0.111	1	Epi
SEL1L33	0.00030141	0.37563255	0.401	0.588	1	Epi

CHTOP2	0.00030289	0.24969392	0.457	0.636	1	Epi
ZNF5982	0.00030761	0.48623314	0.127	0.204	1	Epi
BBS41	0.00030829	1.48061439	0.221	0.172	1	Epi
TMEM172	0.00030907	2.07635657	0.058	0.029	1	Epi
SUPT5H1	0.00031014	0.16335392	0.508	0.743	1	Epi
ATRN3	0.00031417	0.51963941	0.307	0.447	1	Epi
FMR13	0.0003142	0.27755775	0.325	0.469	1	Epi
ACOT83	0.0003146	0.1433883	0.135	0.218	1	Epi
WDR72	0.00031583	0.41583598	0.145	0.226	1	Epi
IKZF51	0.00031832	0.35516244	0.165	0.257	1	Epi
SIN3A2	0.00031942	0.35605558	0.35	0.508	1	Epi
CCDC695	0.00031962	1.33049884	0.335	0.299	1	Epi
TASP12	0.00032046	1.59955348	0.142	0.094	1	Epi
SYAP12	0.00032242	0.24141984	0.536	0.786	1	Epi
ZG16B2	0.00032468	2.91650082	0.036	0.014	1	Epi
TBX6	0.00032683	2.29636006	0.069	0.036	1	Epi
RNF2081	0.00032772	2.49280198	0.069	0.036	1	Epi
ZNF5164	0.00032939	1.39188526	0.322	0.278	1	Epi
MYO193	0.00033058	0.28733437	0.155	0.243	1	Epi
ATPAF22	0.00033149	0.68177396	0.041	0.094	1	Epi
ZBTB343	0.00033209	0.46287012	0.14	0.222	1	Epi
C8orf763	0.0003331	0.23649221	0.213	0.32	1	Epi
PIK3R2	0.00033414	0.24409365	0.406	0.588	1	Epi
TMEM198	0.00033564	2.96791405	0.018	0.005	1	Epi
CCDC28A1	0.00034091	0.25247481	0.33	0.478	1	Epi
SORBS11	0.00034303	2.1916468	0.028	0.01	1	Epi
ECHDC23	0.00034386	1.32221835	0.434	0.42	1	Epi
PTOV12	0.00035094	1.41748142	0.439	0.456	1	Epi
CCAR21	0.00035705	0.21867192	0.345	0.497	1	Epi
FNTB2	0.00035966	0.18455653	0.094	0.165	1	Epi
AFF15	0.00036969	0.50741393	0.492	0.695	1	Epi
RYK4	0.00036992	0.15316455	0.102	0.178	1	Epi
C5orf151	0.00037642	0.51038979	0.297	0.448	1	Epi
MAMSTR	0.00037715	1.67958358	0.018	0.005	1	Epi
DVL22	0.00037795	0.46963484	0.206	0.305	1	Epi
ZNF6071	0.00037911	1.38076711	0.089	0.051	1	Epi
TMEM87B2	0.00037929	0.45006385	0.236	0.345	1	Epi
VLDLR2	0.00038267	1.49864472	0.076	0.042	1	Epi
C4orf36	0.00038388	4.29821429	0.013	0.003	1	Epi
B4GALT42	0.00038496	1.48264274	0.213	0.164	1	Epi
CHCHD6	0.00038684	1.97440992	0.16	0.113	1	Epi
EIF4ENIF12	0.00038912	0.25843333	0.14	0.225	1	Epi
INTS103	0.00039016	0.27372126	0.315	0.465	1	Epi
PTCD1	0.00039056	0.39609465	0.145	0.23	1	Epi
DCAF41	0.00039084	0.98815308	0.063	0.127	1	Epi
DOP1B4	0.00039205	0.27261844	0.14	0.225	1	Epi
EI241	0.00039213	0.3214074	0.315	0.449	1	Epi
BOLA31	0.00039753	0.24002674	0.051	0.108	1	Epi
PFKM1	0.00040115	1.44518069	0.147	0.1	1	Epi
ERAL1	0.00040214	0.3621936	0.282	0.398	1	Epi
NAA383	0.00040447	0.40194722	0.325	0.475	1	Epi
MMP24OS1	0.0004055	0.19019193	0.586	0.818	1	Epi

MIPEP1	0.00040756	1.81120171	0.241	0.196	1	Epi
COA13	0.00041207	0.14178262	0.099	0.169	1	Epi
WDR171	0.00041327	2.01143198	0.058	0.029	1	Epi
TBP2	0.00041401	0.16870703	0.155	0.242	1	Epi
TDP12	0.00041805	0.21015109	0.203	0.302	1	Epi
ZNF4073	0.00041873	0.12201567	0.173	0.271	1	Epi
RBMXL11	0.00042403	0.30923164	0.206	0.31	1	Epi
INO802	0.00042478	0.271757	0.259	0.379	1	Epi
ALG133	0.00042556	0.16799447	0.391	0.555	1	Epi
PMS21	0.00042753	0.25712896	0.112	0.186	1	Epi
TLR34	0.00043076	1.61384698	0.046	0.02	1	Epi
AC104389.5	0.00043318	4.25742918	0.015	0.004	1	Epi
RFK2	0.00043751	0.72336352	0.201	0.311	1	Epi
NEK12	0.00043866	1.67536629	0.162	0.114	1	Epi
FAM120AOS3	0.00045288	0.55753392	0.299	0.433	1	Epi
TMEM14B2	0.00045322	0.24791291	0.17	0.257	1	Epi
PTGR1	0.00045334	0.3500594	0.292	0.434	1	Epi
SCAND1	0.00045448	0.38363979	0.289	0.421	1	Epi
GOLPH31	0.00045588	0.12028198	0.571	0.808	1	Epi
GPR355	0.00046241	1.54097673	0.046	0.021	1	Epi
DCAF61	0.00047379	0.20230636	0.305	0.434	1	Epi
ZFP32	0.0004762	1.49652439	0.216	0.167	1	Epi
KCTD23	0.00047914	0.18379701	0.168	0.259	1	Epi
DENND103	0.0004808	0.42031883	0.249	0.361	1	Epi
ZNF4331	0.00048304	2.51823863	0.028	0.01	1	Epi
GPBAR12	0.00048573	0.42640822	0.038	0.089	1	Epi
METTL172	0.0004908	0.1259382	0.272	0.395	1	Epi
HDAC52	0.00049124	0.21784031	0.327	0.464	1	Epi
URI13	0.00049133	0.29218187	0.424	0.604	1	Epi
AAGAB2	0.00049388	0.42656125	0.31	0.457	1	Epi
SLC19A22	0.00049432	0.11498698	0.157	0.245	1	Epi
SDR39U11	0.00049543	0.51367527	0.297	0.431	1	Epi
MYL6B2	0.00049938	1.99339196	0.089	0.052	1	Epi
FAM8A13	0.00050126	0.11789102	0.216	0.315	1	Epi
RAPGEF61	0.00050132	0.29495801	0.175	0.267	1	Epi
CCNDBP11	0.00050255	0.17740024	0.528	0.751	1	Epi
TSSK4	0.00050278	2.37730283	0.013	0.003	1	Epi
CLPX3	0.0005035	0.19681316	0.261	0.375	1	Epi
IMPDH22	0.00050477	0.49166217	0.208	0.303	1	Epi
PREPL1	0.00050587	0.41662847	0.249	0.378	1	Epi
KLF123	0.00050783	0.5714214	0.084	0.048	1	Epi
GPT3	0.00050859	2.73857024	0.036	0.015	1	Epi
SCML13	0.00051146	0.2460285	0.117	0.192	1	Epi
SLC25A122	0.00051162	0.16567265	0.185	0.279	1	Epi
SLC22A17	0.00051714	2.62272872	0.013	0.003	1	Epi
RDX1	0.00051727	0.17613615	0.231	0.331	1	Epi
POLL2	0.00051825	0.33040969	0.152	0.239	1	Epi
SCX2	0.00052561	1.91987905	0.051	0.024	1	Epi
GSTA41	0.00052683	2.53334552	0.051	0.024	1	Epi
TBL1XR14	0.00053078	0.25893205	0.462	0.67	1	Epi
POLR2C3	0.00053535	0.25897051	0.279	0.407	1	Epi
GK52	0.00054285	1.18072816	0.239	0.191	1	Epi

UGGT22	0.00054361	0.15319054	0.168	0.256	1	Epi
PHTF12	0.00054593	1.40764129	0.305	0.276	1	Epi
YPEL22	0.00055191	0.20980046	0.244	0.355	1	Epi
PCDHGC31	0.00055422	2.44338046	0.033	0.013	1	Epi
THAP72	0.00056324	0.1642912	0.147	0.229	1	Epi
TPRA13	0.00056403	0.51584544	0.279	0.408	1	Epi
MAPK114	0.0005641	0.36403396	0.018	0.005	1	Epi
PPP6R32	0.00056944	0.3258745	0.365	0.52	1	Epi
NCAPG21	0.00057081	0.28972138	0.096	0.164	1	Epi
CC2D1A	0.00057108	0.35490915	0.264	0.38	1	Epi
ZCWPW11	0.00057877	2.0524857	0.086	0.05	1	Epi
FZD81	0.00058104	2.46704125	0.069	0.037	1	Epi
C12orf491	0.00058146	0.20753845	0.066	0.127	1	Epi
TCEA32	0.00058586	0.64322086	0.315	0.455	1	Epi
DPY302	0.00058621	0.61366978	0.317	0.473	1	Epi
TST1	0.00058636	0.69391908	0.175	0.264	1	Epi
C5orf242	0.00058816	0.2154228	0.228	0.335	1	Epi
CEP2901	0.00059119	1.88362129	0.16	0.115	1	Epi
USP383	0.000597	0.33030527	0.256	0.375	1	Epi
KMT2B3	0.00060244	0.55786358	0.338	0.471	1	Epi
PRKAG23	0.00060655	0.6091434	0.147	0.235	1	Epi
WWTR11	0.00060693	1.93423319	0.142	0.099	1	Epi
PPP1R71	0.00061102	0.35055444	0.536	0.756	1	Epi
HIVEP23	0.00061281	1.16461102	0.145	0.098	1	Epi
SEC22A2	0.00062132	0.17485364	0.17	0.258	1	Epi
UBR24	0.0006214	0.1343685	0.5	0.717	1	Epi
COL9A21	0.00062269	0.2759771	0.19	0.284	1	Epi
ZNF4452	0.00062409	0.26783823	0.117	0.187	1	Epi
TMEM1812	0.00062996	0.26598266	0.18	0.274	1	Epi
COG81	0.0006335	0.20334556	0.183	0.278	1	Epi
PRKRA2	0.00063537	0.32223659	0.269	0.388	1	Epi
RALGAPA23	0.0006401	1.32372623	0.266	0.224	1	Epi
UBE3A4	0.00064073	0.20248195	0.462	0.647	1	Epi
TIAF12	0.00064155	1.43767288	0.033	0.013	1	Epi
MFSD91	0.0006436	0.89585531	0.091	0.162	1	Epi
BRWD12	0.00065436	0.10086383	0.122	0.2	1	Epi
BTBD72	0.00065803	0.3349725	0.338	0.475	1	Epi
STAG13	0.00066093	0.36242594	0.35	0.483	1	Epi
DHFR21	0.00066136	0.15096433	0.091	0.158	1	Epi
RNF2272	0.00066195	2.30565249	0.036	0.015	1	Epi
SLC25A172	0.00066271	0.10291504	0.142	0.225	1	Epi
DIPK2A4	0.00066285	0.22659368	0.305	0.423	1	Epi
BBS122	0.00066704	1.98248954	0.074	0.041	1	Epi
C11orf951	0.00066981	2.33018023	0.038	0.016	1	Epi
CNTNAP11	0.00067246	2.53972886	0.023	0.008	1	Epi
SEMA4A4	0.00067596	0.29800001	0.122	0.198	1	Epi
SAMD101	0.00067786	2.04807483	0.066	0.035	1	Epi
NHS1	0.00068301	2.27715228	0.058	0.03	1	Epi
NOL123	0.00068332	0.51040432	0.203	0.297	1	Epi
C16orf95	0.00068588	0.77011317	0.036	0.085	1	Epi
MTMR13	0.00069103	0.23123545	0.234	0.332	1	Epi
OR6A2	0.00069626	2.64884229	0.015	0.004	1	Epi

SIRT4	0.0007067	2.21838811	0.018	0.005	1	Epi
MYSM13	0.00070711	0.19911859	0.223	0.326	1	Epi
ANKRD13A1	0.00071394	0.32211255	0.35	0.497	1	Epi
TNIP15	0.00072174	0.46245418	0.34	0.492	1	Epi
MARCH95	0.0007265	0.99961547	0.109	0.068	1	Epi
RUVBL22	0.00072662	1.85928163	0.335	0.315	1	Epi
RAB27A1	0.0007423	0.26755606	0.353	0.505	1	Epi
PLPPR24	0.000744	2.06166736	0.107	0.067	1	Epi
RBM473	0.00074533	0.16295645	0.637	0.867	1	Epi
GLYCTK3	0.0007533	0.27571517	0.193	0.283	1	Epi
PRDM42	0.00075426	0.28626335	0.274	0.407	1	Epi
INSR2	0.00075702	1.58631083	0.32	0.287	1	Epi
VAPB2	0.0007578	0.47395968	0.223	0.327	1	Epi
HSP90AB11	0.00075881	0.13250256	0.66	0.896	1	Epi
GTF2E22	0.00075982	0.28929477	0.305	0.442	1	Epi
SNAI22	0.00076195	0.36837259	0.033	0.079	1	Epi
NANP3	0.00076636	0.25158535	0.053	0.109	1	Epi
NET12	0.00077405	1.31898869	0.348	0.321	1	Epi
CAB39L3	0.00077413	1.60186338	0.145	0.1	1	Epi
SFI12	0.00078285	1.24682722	0.259	0.213	1	Epi
TMEM622	0.00078865	0.28582685	0.162	0.249	1	Epi
PRDX61	0.00079374	0.54388173	0.439	0.621	1	Epi
MSH62	0.00079999	0.62816623	0.183	0.274	1	Epi
TRUB2	0.00080329	0.24237767	0.195	0.286	1	Epi
TUSC21	0.00080416	0.5214566	0.254	0.365	1	Epi
NAB12	0.00080942	0.26623065	0.419	0.595	1	Epi
EFEMP22	0.00081083	2.77866962	0.041	0.018	1	Epi
RAB11FIP32	0.00081182	1.57646712	0.19	0.142	1	Epi
DCAF132	0.00081406	0.57265279	0.096	0.163	1	Epi
MTFR12	0.00081665	0.43559732	0.168	0.264	1	Epi
TSEN541	0.0008239	0.1071337	0.066	0.123	1	Epi
SDHAF4	0.00082497	0.10959527	0.17	0.26	1	Epi
PLPP53	0.0008251	1.54686433	0.398	0.404	1	Epi
ZSCAN27	0.00083188	0.11547957	0.076	0.137	1	Epi
EGLN22	0.00083246	0.37093597	0.426	0.61	1	Epi
EMC21	0.000833	0.31889548	0.426	0.608	1	Epi
PSMG33	0.00084337	0.33138178	0.299	0.442	1	Epi
SMAD42	0.00084425	0.47435878	0.381	0.535	1	Epi
ZHX22	0.00085386	0.19554238	0.081	0.141	1	Epi
CEP954	0.00085432	1.48107242	0.221	0.175	1	Epi
KATNAL11	0.0008565	1.41776933	0.16	0.113	1	Epi
ZNF2243	0.0008572	0.41026216	0.162	0.253	1	Epi
INO80E3	0.00085997	0.56601949	0.383	0.548	1	Epi
YDJC1	0.000872	0.2872267	0.071	0.13	1	Epi
BORCS72	0.00087423	0.2905759	0.254	0.362	1	Epi
SPATA45	0.00088189	3.43047552	0.01	0.002	1	Epi
CEP972	0.00088528	1.6545362	0.119	0.08	1	Epi
ELP42	0.00088655	0.19023194	0.142	0.219	1	Epi
DNASE1	0.00089021	1.75652189	0.066	0.036	1	Epi
POLDIP33	0.00089092	0.38669643	0.464	0.638	1	Epi
ARMC62	0.00089862	0.31849661	0.223	0.323	1	Epi
SON1	0.00091592	0.45918426	0.371	0.523	1	Epi

XPR13	0.00091642	0.16381241	0.18	0.268	1	Epi
MARCH84	0.00092492	0.25941271	0.185	0.28	1	Epi
LYSMD42	0.00092934	0.21352688	0.091	0.155	1	Epi
POLR3A2	0.00093076	0.1442312	0.168	0.252	1	Epi
NUDT151	0.0009325	0.38240614	0.135	0.216	1	Epi
ARL31	0.00093561	1.68513265	0.231	0.187	1	Epi
TIGD61	0.00094044	0.28103408	0.02	0.06	1	Epi
MOV103	0.00094174	0.3917568	0.411	0.585	1	Epi
SPICE12	0.00095459	1.62025974	0.185	0.139	1	Epi
B4GAT1	0.00096464	0.20986884	0.099	0.164	1	Epi
LEAP21	0.0009706	3.33447055	0.015	0.004	1	Epi
YEATS43	0.00097461	0.74746855	0.147	0.225	1	Epi
ZNF5624	0.00097526	0.47803694	0.218	0.322	1	Epi
PCDHGB71	0.00097683	2.05372782	0.025	0.009	1	Epi
DDX3X4	0.00097737	0.17810296	0.325	0.454	1	Epi
SLC12A42	0.00097947	0.16616012	0.188	0.285	1	Epi
RTN4RL21	0.00098248	2.34101967	0.01	0.002	1	Epi
MCCC2	0.00099803	0.28937507	0.234	0.333	1	Epi
ACSS31	0.00100147	1.93066484	0.063	0.034	1	Epi
RPP211	0.0010026	0.30687605	0.185	0.276	1	Epi
PEX11G2	0.00101241	1.48129783	0.104	0.065	1	Epi
PCNT2	0.00102322	0.46484863	0.256	0.374	1	Epi
GTF3C42	0.00102819	0.44555775	0.104	0.171	1	Epi
ARMCX62	0.00103184	0.50585712	0.046	0.096	1	Epi
PGC	0.00103393	6.60574857	0.01	0.002	1	Epi
PGS14	0.00103421	0.19596728	0.249	0.352	1	Epi
L3MBTL21	0.00104245	0.32558506	0.244	0.36	1	Epi
CCDC145	0.00104809	0.17781329	0.155	0.235	1	Epi
NOSIP1	0.00105172	0.39219689	0.236	0.338	1	Epi
WNT5A2	0.00105259	2.45263529	0.023	0.008	1	Epi
MED191	0.00105579	0.36572801	0.129	0.205	1	Epi
SOCS41	0.00105716	0.23077999	0.201	0.293	1	Epi
NME31	0.00105793	0.66710796	0.34	0.484	1	Epi
PDLIM52	0.00106335	0.28702492	0.325	0.454	1	Epi
RBM262	0.00107209	0.2462632	0.244	0.342	1	Epi
SLC25A362	0.00107435	1.30390032	0.462	0.487	1	Epi
GCLM2	0.00107539	1.47057161	0.226	0.185	1	Epi
ZSCAN263	0.00107891	0.26413448	0.104	0.177	1	Epi
FRA10AC13	0.00108216	0.186735	0.193	0.285	1	Epi
ADAM152	0.00108688	0.44290914	0.383	0.551	1	Epi
ZSWIM64	0.00108822	0.29519329	0.358	0.499	1	Epi
TMEM1412	0.00108857	0.42892793	0.302	0.429	1	Epi
NDN1	0.00109073	2.53722846	0.056	0.029	1	Epi
SLC39A133	0.00109929	0.61035401	0.127	0.202	1	Epi
TARS2	0.00111177	0.38240301	0.041	0.089	1	Epi
DCTN52	0.00111274	1.05423403	0.099	0.166	1	Epi
NR4A24	0.001113	0.12476913	0.368	0.488	1	Epi
FXR12	0.00112819	0.14519877	0.234	0.34	1	Epi
CDK172	0.00113039	0.40005531	0.213	0.31	1	Epi
FRRS12	0.00113253	0.17411166	0.183	0.269	1	Epi
UBOX52	0.00114079	0.74004811	0.119	0.19	1	Epi
LUC7L22	0.0011467	0.22838304	0.51	0.737	1	Epi

RFX72	0.00114827	0.56575824	0.122	0.196	1	Epi
PAM1	0.00115464	0.32358448	0.208	0.305	1	Epi
CLDN232	0.00115611	0.32776482	0.254	0.368	1	Epi
DMTF14	0.00117943	0.27758861	0.246	0.35	1	Epi
SULT1A12	0.00118007	1.00495136	0.096	0.16	1	Epi
SETD43	0.00118099	1.68922602	0.239	0.194	1	Epi
STIL1	0.00119361	1.66934995	0.074	0.042	1	Epi
SLC12A22	0.00119809	2.12483009	0.195	0.152	1	Epi
ZFYVE191	0.00120716	0.88401897	0.19	0.28	1	Epi
DCTN1	0.00121144	0.27210899	0.551	0.769	1	Epi
PPCDC3	0.00121253	0.41730488	0.16	0.245	1	Epi
HEMK13	0.00121634	0.20565003	0.145	0.225	1	Epi
KDM5B1	0.00121915	1.62860435	0.264	0.228	1	Epi
UCKL13	0.00122344	0.28503944	0.261	0.375	1	Epi
ARFIP1	0.00122798	0.24267693	0.297	0.421	1	Epi
MARS4	0.00123049	0.18819204	0.096	0.163	1	Epi
MAML33	0.00123807	0.36355515	0.155	0.234	1	Epi
XRCC11	0.00125036	0.44646148	0.289	0.418	1	Epi
SSBP32	0.00125191	1.71164547	0.16	0.116	1	Epi
PGM2L12	0.00127746	1.3830953	0.203	0.162	1	Epi
ZNF5961	0.00127772	1.68919106	0.046	0.022	1	Epi
ENDOG1	0.00127781	1.39236637	0.297	0.266	1	Epi
PIGO	0.00128582	0.46914496	0.251	0.362	1	Epi
YPEL11	0.00130592	1.18192386	0.041	0.019	1	Epi
PLCXD13	0.00130628	0.53032414	0.129	0.198	1	Epi
JAG13	0.00131079	0.72696504	0.14	0.213	1	Epi
MARC12	0.00131238	2.20433512	0.041	0.019	1	Epi
TBCE3	0.00131261	0.16426646	0.066	0.12	1	Epi
SRSF72	0.00131749	0.31652598	0.31	0.44	1	Epi
CDK1	0.00132762	0.74640609	0.099	0.061	1	Epi
HIKESHI3	0.00134752	0.14570501	0.051	0.101	1	Epi
RSF12	0.00135177	0.2915547	0.236	0.329	1	Epi
SMIM191	0.0013545	0.70502928	0.348	0.499	1	Epi
CETN21	0.00135678	2.2164866	0.482	0.554	1	Epi
ZNF6491	0.00136118	0.35063075	0.051	0.101	1	Epi
PGAP3	0.00136374	0.58641183	0.216	0.306	1	Epi
CEP1313	0.00137229	1.97896081	0.16	0.118	1	Epi
BAG23	0.00137448	0.10344467	0.079	0.137	1	Epi
SMKR1	0.0013831	2.2999716	0.02	0.007	1	Epi
PAK41	0.00138545	1.54122968	0.193	0.147	1	Epi
NUDT52	0.00138667	0.65454812	0.358	0.506	1	Epi
VPS251	0.0013956	0.28881098	0.272	0.391	1	Epi
OSBPL33	0.00140367	1.10164635	0.259	0.215	1	Epi
FAM217B2	0.00142287	0.1761882	0.129	0.2	1	Epi
MEN12	0.0014262	0.33607887	0.183	0.275	1	Epi
SMIM271	0.00143515	0.2859451	0.114	0.182	1	Epi
TTC192	0.0014356	0.26455438	0.244	0.351	1	Epi
SPATA52	0.00143586	0.268311	0.145	0.22	1	Epi
C15orf612	0.00144451	1.05210788	0.084	0.144	1	Epi
CCDC933	0.0014519	0.46969993	0.135	0.209	1	Epi
FBXO332	0.00145838	0.39282649	0.208	0.3	1	Epi
MTMR122	0.00147786	0.27531905	0.338	0.483	1	Epi

NEU41	0.00149409	3.06255522	0.023	0.008	1	Epi
EDC32	0.00151767	0.31495937	0.201	0.293	1	Epi
CATSPER3	0.00153824	2.50182309	0.018	0.006	1	Epi
APPBP23	0.00154002	0.6091673	0.208	0.303	1	Epi
AP002495.12	0.00154295	1.74742537	0.081	0.049	1	Epi
SYCP21	0.0015495	1.68589616	0.03	0.013	1	Epi
PSKH12	0.00155076	0.40705008	0.236	0.347	1	Epi
ANKRD391	0.00155293	2.63664089	0.038	0.017	1	Epi
C10orf1432	0.00155294	0.35292855	0.043	0.09	1	Epi
RXYLT13	0.00157614	0.47384573	0.208	0.301	1	Epi
POLD3	0.00160604	0.19113884	0.36	0.499	1	Epi
TEN12	0.00161551	0.94240614	0.19	0.286	1	Epi
RALA5	0.001616	0.46649304	0.251	0.357	1	Epi
B3GALT4	0.00162726	0.3239939	0.17	0.254	1	Epi
SAP30L2	0.00163589	0.26202703	0.421	0.586	1	Epi
CTTNBP2NL2	0.00164067	0.44577965	0.31	0.446	1	Epi
RPAIN3	0.00164598	0.54899866	0.335	0.473	1	Epi
APOOL	0.00165977	0.41673714	0.117	0.186	1	Epi
GTPBP102	0.00166394	0.11581979	0.178	0.265	1	Epi
CDC42EP22	0.00166602	1.40840369	0.094	0.058	1	Epi
DUSP123	0.00166947	0.16466274	0.117	0.185	1	Epi
ARRDC11	0.00167119	0.49064497	0.429	0.611	1	Epi
FBXO212	0.00168713	0.59194403	0.18	0.264	1	Epi
LYRM71	0.00170743	0.42307433	0.066	0.118	1	Epi
DDX563	0.00171078	0.454923	0.261	0.378	1	Epi
OR2AG2	0.00171486	2.52145259	0.015	0.005	1	Epi
FANCG1	0.00171754	0.31947016	0.056	0.106	1	Epi
PPM1D3	0.00173712	0.30673812	0.127	0.194	1	Epi
GOT13	0.0017375	0.32116739	0.129	0.201	1	Epi
PTGFRN1	0.00173925	0.24553583	0.157	0.239	1	Epi
NBR11	0.00174714	0.26987316	0.586	0.822	1	Epi
ZBTB9	0.00174934	0.7234882	0.165	0.251	1	Epi
UQCRHL1	0.0017506	3.37155756	0.013	0.003	1	Epi
SNRPE2	0.00175481	0.51921157	0.18	0.274	1	Epi
RABEP22	0.00175754	1.64169837	0.299	0.262	1	Epi
ZNF258	0.00176354	0.36351252	0.18	0.269	1	Epi
FAM193A2	0.0017668	0.25167152	0.241	0.344	1	Epi
SYT5	0.00177814	3.11561496	0.015	0.005	1	Epi
CEP1923	0.0018081	0.10067657	0.211	0.3	1	Epi
LRCH32	0.00181112	0.15400563	0.277	0.386	1	Epi
ERC13	0.00182661	0.39134084	0.223	0.323	1	Epi
GABRE1	0.00184993	2.20048064	0.046	0.023	1	Epi
DDHD23	0.00189042	0.29985173	0.173	0.253	1	Epi
CNOT63	0.00190042	0.37097071	0.284	0.408	1	Epi
SBNO13	0.00190205	0.1602463	0.259	0.363	1	Epi
MRC24	0.0019073	2.14693959	0.018	0.006	1	Epi
TP53INP22	0.00191889	2.42656605	0.292	0.267	1	Epi
EAPP1	0.00193923	0.40281734	0.332	0.464	1	Epi
SSRP12	0.00195121	0.33252706	0.388	0.546	1	Epi
MORC22	0.00195305	0.37549461	0.178	0.257	1	Epi
FAIM1	0.001958	1.63145598	0.254	0.215	1	Epi
CIAO31	0.00197172	0.44989145	0.188	0.274	1	Epi

NCOA22	0.00197392	0.21601349	0.241	0.34	1	Epi
DYRK31	0.00197493	2.93407493	0.053	0.028	1	Epi
RIOK32	0.00198683	0.77648189	0.505	0.713	1	Epi
ZNF7631	0.00200194	1.9651021	0.03	0.013	1	Epi
AGL1	0.00201587	0.18559659	0.168	0.249	1	Epi
UNK2	0.00201813	0.48155523	0.198	0.286	1	Epi
CYB5R22	0.00201827	0.57131854	0.178	0.256	1	Epi
SQLE1	0.0020358	1.29138188	0.135	0.093	1	Epi
DDIT33	0.00205144	0.46684733	0.244	0.349	1	Epi
GPR1553	0.00206003	0.6947511	0.02	0.056	1	Epi
ARHGAP213	0.00206467	0.20556806	0.135	0.205	1	Epi
TRPV14	0.0020773	2.23006907	0.069	0.039	1	Epi
NR6A12	0.00208243	2.57500623	0.036	0.016	1	Epi
MYLIP4	0.0020853	1.39526062	0.175	0.131	1	Epi
SYNM1	0.00208702	1.94612238	0.018	0.006	1	Epi
GEMIN74	0.00211605	0.16128449	0.168	0.247	1	Epi
PRPF34	0.00213131	0.53665161	0.332	0.471	1	Epi
CAAP13	0.00214105	0.55637664	0.168	0.25	1	Epi
ALDH5A12	0.00214996	0.23217325	0.127	0.195	1	Epi
METTL32	0.00215297	0.34313169	0.305	0.429	1	Epi
APOBEC3D1	0.00216021	0.36313597	0.023	0.009	1	Epi
CPSF62	0.00217312	0.37502079	0.269	0.38	1	Epi
RNF1454	0.00218927	1.27467327	0.34	0.316	1	Epi
COLGALT21	0.00219032	2.96650563	0.01	0.002	1	Epi
KDM3A3	0.00219385	0.49564455	0.277	0.388	1	Epi
CDC163	0.00219707	0.33396264	0.404	0.575	1	Epi
PAQR32	0.00220385	0.27579611	0.114	0.181	1	Epi
KDM2B4	0.00222646	0.54699473	0.282	0.401	1	Epi
ZNF33	0.00223747	1.4564435	0.223	0.184	1	Epi
RPAP21	0.00224391	0.41240975	0.107	0.17	1	Epi
TRIM6	0.00224686	0.31527236	0.038	0.081	1	Epi
KLHL282	0.00224724	0.51266618	0.165	0.246	1	Epi
DOCK55	0.00225501	1.21710105	0.32	0.293	1	Epi
TSEN34	0.00225617	0.23545456	0.165	0.243	1	Epi
SDHA3	0.00225771	0.36888268	0.274	0.38	1	Epi
OPRL13	0.00226148	0.17699942	0.033	0.074	1	Epi
CMSS12	0.00226383	0.40816972	0.119	0.184	1	Epi
HSD11B21	0.00227191	3.25659007	0.025	0.01	1	Epi
USP213	0.00228565	1.14378466	0.249	0.206	1	Epi
RTEL13	0.00229153	0.61469927	0.114	0.18	1	Epi
TRIM83	0.00229272	0.30971136	0.571	0.812	1	Epi
INTS44	0.00230196	0.20781644	0.373	0.505	1	Epi
IL72	0.00234153	0.49825156	0.091	0.153	1	Epi
ABTB13	0.00234647	0.16645142	0.147	0.217	1	Epi
SLC12A71	0.00234886	0.29293956	0.503	0.726	1	Epi
TRMT1L2	0.00236247	0.59987576	0.348	0.495	1	Epi
TEDC14	0.00238165	0.48556725	0.195	0.286	1	Epi
ZNF425	0.00238855	2.94778496	0.015	0.005	1	Epi
COX112	0.00238972	0.14985098	0.211	0.305	1	Epi
SAFB22	0.00239927	0.23504107	0.47	0.651	1	Epi
C2orf684	0.00240489	0.23273602	0.33	0.458	1	Epi
RMND5B2	0.00240642	0.23761896	0.244	0.346	1	Epi

ZNF354A3	0.00241527	0.44115848	0.124	0.193	1	Epi
TPD52L11	0.00242294	0.89331993	0.089	0.147	1	Epi
IER34	0.00242717	2.50349931	0.221	0.325	1	Epi
ZNF5672	0.00243504	0.26419605	0.079	0.133	1	Epi
LRRC75B2	0.00244135	2.179963	0.013	0.004	1	Epi
BBS22	0.00247449	0.41668699	0.335	0.47	1	Epi
MAP2K42	0.00248621	0.1582461	0.193	0.281	1	Epi
POMGNT21	0.00251074	0.7952736	0.114	0.179	1	Epi
ANKRD272	0.00251639	0.50845579	0.261	0.363	1	Epi
LIN7B3	0.00251934	0.13390782	0.102	0.159	1	Epi
TYW13	0.00251938	0.5314902	0.038	0.081	1	Epi
KIF1B5	0.00252376	0.28749204	0.32	0.436	1	Epi
C1QTNF62	0.0025293	1.02271665	0.025	0.01	1	Epi
SRPK13	0.00253612	0.25631655	0.277	0.383	1	Epi
ELOVL72	0.00256175	0.49328763	0.033	0.073	1	Epi
CYBRD14	0.00256311	1.21775141	0.048	0.025	1	Epi
LZTR1	0.00256996	0.77353522	0.178	0.26	1	Epi
FBXW82	0.00258672	0.20348283	0.14	0.213	1	Epi
WWP22	0.00258902	0.43704852	0.406	0.584	1	Epi
PHLDA1	0.00258902	1.83326252	0.162	0.243	1	Epi
DHRS122	0.00258999	0.29627375	0.18	0.261	1	Epi
NUP503	0.00261665	0.34079214	0.305	0.431	1	Epi
PPM1L3	0.00261688	1.5968068	0.135	0.094	1	Epi
PIBF12	0.00265816	1.45399969	0.213	0.171	1	Epi
CMBL1	0.00267893	2.14345548	0.051	0.027	1	Epi
NFATC13	0.00271965	0.30439594	0.223	0.321	1	Epi
CUL52	0.00273759	0.26776651	0.254	0.357	1	Epi
MICA1	0.00275777	0.38612348	0.122	0.191	1	Epi
OTUD42	0.00277659	0.30496741	0.335	0.473	1	Epi
YIPF21	0.00277753	0.38208644	0.244	0.342	1	Epi
FBRS2	0.00279891	0.33980553	0.464	0.659	1	Epi
FAM131A2	0.0028021	0.25386641	0.19	0.279	1	Epi
TPP23	0.00281294	0.2563074	0.325	0.45	1	Epi
PLEKHJ12	0.00282163	0.26642334	0.221	0.312	1	Epi
ARPC5L1	0.00283565	0.59150001	0.327	0.472	1	Epi
FOSL13	0.0028443	3.47905927	0.043	0.022	1	Epi
HECTD23	0.00284678	1.38241122	0.091	0.058	1	Epi
RANBP61	0.0028482	0.39610458	0.086	0.142	1	Epi
BFSP1	0.00287858	1.99727538	0.023	0.009	1	Epi
PNPLA43	0.00288179	0.60609686	0.119	0.187	1	Epi
STK162	0.00288803	0.57252703	0.203	0.292	1	Epi
FAS4	0.00289245	0.41655207	0.137	0.205	1	Epi
ZNF585A3	0.00289471	0.4033567	0.109	0.172	1	Epi
TCEA22	0.00290936	1.88718262	0.124	0.088	1	Epi
AIG13	0.00292222	0.42545949	0.246	0.354	1	Epi
TMEM1592	0.00292895	0.88657786	0.216	0.312	1	Epi
COX103	0.00294864	0.25065609	0.147	0.217	1	Epi
RRP81	0.00295949	0.46184681	0.124	0.191	1	Epi
ANG1	0.00297209	1.2392232	0.155	0.115	1	Epi
DNAH142	0.00297725	1.63251377	0.071	0.042	1	Epi
DDX421	0.0030047	0.34039729	0.416	0.571	1	Epi
DUSP113	0.00302204	0.3199165	0.251	0.35	1	Epi

PPP2R2A1	0.0030249	0.45125838	0.279	0.401	1	Epi
FOXO32	0.00302554	0.39896234	0.373	0.503	1	Epi
STPG12	0.00303449	1.37892127	0.307	0.278	1	Epi
ZNF237	0.0030589	1.6312068	0.162	0.123	1	Epi
ITGA61	0.00305971	0.50545237	0.071	0.122	1	Epi
RGP12	0.00307481	0.25961682	0.223	0.309	1	Epi
NBEAL13	0.00310727	1.77602002	0.175	0.135	1	Epi
TMEM1352	0.00313124	0.23825358	0.129	0.194	1	Epi
AASDH3	0.0031562	0.38932829	0.142	0.217	1	Epi
TECR1	0.00316023	0.52196548	0.256	0.356	1	Epi
FBXL42	0.00316714	0.22739851	0.16	0.233	1	Epi
CYB561D12	0.003169	0.12854007	0.061	0.109	1	Epi
NPM22	0.00317492	2.34129054	0.03	0.013	1	Epi
GYS15	0.00317739	0.44621733	0.188	0.264	1	Epi
PRPF183	0.00318464	0.27521611	0.198	0.28	1	Epi
UBA53	0.00318565	0.53911989	0.226	0.316	1	Epi
UGCG4	0.0031989	0.92473217	0.482	0.517	1	Epi
ZCCHC82	0.00320395	0.21182907	0.185	0.267	1	Epi
LIG42	0.00322593	0.21755017	0.17	0.245	1	Epi
DTX21	0.00328966	2.61806679	0.132	0.094	1	Epi
SH3GL12	0.00329492	0.54938753	0.244	0.343	1	Epi
NOMO1	0.003297	0.49694104	0.183	0.267	1	Epi
TPD52L23	0.00332453	0.39091947	0.454	0.638	1	Epi
ZNF7402	0.00335004	0.48395072	0.185	0.262	1	Epi
GNA111	0.00335326	1.29745877	0.386	0.386	1	Epi
GFER3	0.00335695	0.36938012	0.173	0.253	1	Epi
NFIC2	0.00338092	0.41558681	0.439	0.636	1	Epi
MCRIP21	0.00339431	0.43198515	0.066	0.115	1	Epi
GAK1	0.00340826	0.31281086	0.525	0.72	1	Epi
RBM233	0.00342772	0.25968473	0.523	0.723	1	Epi
EPN22	0.00343503	1.25940095	0.264	0.225	1	Epi
CYB5D21	0.0034487	0.60479134	0.183	0.264	1	Epi
CNOT102	0.00345832	0.52501605	0.249	0.358	1	Epi
DESI22	0.00352264	0.16924693	0.201	0.282	1	Epi
PRDM13	0.00353493	1.85533649	0.084	0.053	1	Epi
DBF41	0.00358824	0.53256691	0.076	0.129	1	Epi
MAZ1	0.00359916	0.12629347	0.127	0.192	1	Epi
SLCO4C13	0.00361689	2.27385515	0.061	0.035	1	Epi
USP372	0.00361717	0.31247175	0.109	0.173	1	Epi
KAZN1	0.00363105	1.9063493	0.322	0.316	1	Epi
ATG143	0.00364993	0.55883513	0.173	0.259	1	Epi
DUSP281	0.00365018	0.34841798	0.061	0.109	1	Epi
ZNF6522	0.00367026	0.26277784	0.201	0.29	1	Epi
GUF13	0.00367812	0.14993051	0.135	0.2	1	Epi
LIME12	0.00369801	0.40552266	0.056	0.031	1	Epi
BOLA11	0.00371623	0.10895322	0.036	0.076	1	Epi
TCP111	0.00373626	2.37556296	0.01	0.003	1	Epi
GOLPH3L1	0.00375229	1.14959237	0.416	0.428	1	Epi
PHYKPL5	0.003766	0.64299633	0.279	0.396	1	Epi
TET25	0.00376975	0.361042	0.216	0.304	1	Epi
SBK11	0.00378175	1.11044616	0.023	0.009	1	Epi
KANSL1L2	0.00378682	1.14978534	0.299	0.271	1	Epi

FAM189A22	0.0038124	1.9602516	0.051	0.027	1	Epi
CLYBL2	0.0038129	0.15512514	0.084	0.138	1	Epi
TTC231	0.00384714	0.84753859	0.129	0.196	1	Epi
UQCC12	0.00386274	0.30253445	0.239	0.336	1	Epi
TMEM161B2	0.00386548	0.80270105	0.14	0.209	1	Epi
CLK44	0.00387052	1.3155808	0.236	0.197	1	Epi
PAK15	0.00388338	0.53936887	0.305	0.429	1	Epi
ZNF5101	0.00388547	0.37226263	0.089	0.146	1	Epi
NCBP2AS23	0.00390208	0.3437413	0.17	0.241	1	Epi
ZSWIM83	0.00391101	0.43592336	0.358	0.513	1	Epi
LY6G5C1	0.00394693	2.91218265	0.03	0.014	1	Epi
PRXL2A1	0.00395723	0.28218307	0.254	0.347	1	Epi
CTBP23	0.00397464	0.28391003	0.541	0.771	1	Epi
KDM81	0.00398682	1.7998533	0.053	0.029	1	Epi
LRRC20	0.00399304	0.17255312	0.069	0.117	1	Epi
VPS13C4	0.00400316	0.42929235	0.546	0.779	1	Epi
HIST1H1C3	0.0040414	0.63605035	0.343	0.463	1	Epi
IP6K22	0.00407388	1.40019876	0.34	0.328	1	Epi
VAV21	0.00407653	0.34693469	0.132	0.195	1	Epi
PCYOX1L1	0.00409656	0.16721524	0.155	0.227	1	Epi
ZNF6532	0.0041363	1.08102588	0.109	0.073	1	Epi
BCLAF33	0.00416921	0.25567079	0.175	0.254	1	Epi
ARPC1A2	0.00418269	0.53722945	0.244	0.34	1	Epi
TCFL52	0.00419267	0.4798204	0.046	0.089	1	Epi
CRAT1	0.00419923	0.20098627	0.109	0.163	1	Epi
ZNF1062	0.00419999	0.35766663	0.444	0.626	1	Epi
ANKRD423	0.00420113	1.46641858	0.277	0.251	1	Epi
BCL2L122	0.00421451	0.78512555	0.107	0.168	1	Epi
SHB1	0.00423617	0.53320926	0.239	0.332	1	Epi
ETV66	0.00425481	0.48900528	0.508	0.707	1	Epi
RUSC11	0.00426628	0.32237786	0.16	0.23	1	Epi
CCDC974	0.00427789	0.23339055	0.36	0.477	1	Epi
BPHL1	0.0042983	0.1054753	0.206	0.288	1	Epi
ZNF4462	0.00429885	1.78145693	0.147	0.109	1	Epi
C8orf372	0.00433986	2.03228165	0.048	0.026	1	Epi
ZC3H141	0.00434205	0.54393512	0.236	0.329	1	Epi
EPDR11	0.00440857	2.10726847	0.084	0.054	1	Epi
UCK24	0.00443895	1.44794551	0.053	0.029	1	Epi
FSD1L3	0.00445805	1.35289745	0.297	0.275	1	Epi
VPS13A3	0.00446701	1.0004705	0.168	0.127	1	Epi
MTERF12	0.0044697	0.19458172	0.081	0.134	1	Epi
POLR3G	0.00447786	2.73157668	0.02	0.008	1	Epi
CCNT12	0.00447966	0.28474454	0.249	0.338	1	Epi
RANBP92	0.00449324	0.58418857	0.363	0.51	1	Epi
CRIPT1	0.00451483	0.38983851	0.297	0.409	1	Epi
KLHDC102	0.00453736	0.56497966	0.259	0.363	1	Epi
FAAP201	0.00454436	0.35462723	0.277	0.38	1	Epi
ARID22	0.00454726	0.37371532	0.221	0.31	1	Epi
PAN34	0.00455233	0.22922906	0.317	0.438	1	Epi
METTL163	0.00456616	0.29775982	0.147	0.214	1	Epi
ETV34	0.00456656	0.33159471	0.16	0.233	1	Epi
SUGT13	0.00457575	0.28764266	0.358	0.494	1	Epi

SLC17A94	0.00457591	0.10645884	0.086	0.137	1	Epi
DIPK1A1	0.0045886	0.17001663	0.043	0.084	1	Epi
NSMCE4A3	0.0045904	0.39701222	0.261	0.371	1	Epi
PNMA12	0.00459323	0.58032402	0.401	0.556	1	Epi
HELQ3	0.00459579	0.1346141	0.155	0.229	1	Epi
PLA2G12A2	0.00460024	0.20916347	0.15	0.222	1	Epi
SLC35F24	0.00461395	0.3706937	0.01	0.003	1	Epi
CDK2AP11	0.00463928	0.49390853	0.292	0.402	1	Epi
NFRKB3	0.00465076	0.27471869	0.18	0.256	1	Epi
EHMT12	0.00465638	0.35177262	0.495	0.697	1	Epi
SLTM2	0.00467018	0.4016143	0.492	0.68	1	Epi
KDM3B2	0.00472651	0.3364411	0.431	0.604	1	Epi
RNF19A2	0.00478025	0.19904673	0.35	0.485	1	Epi
ZBED53	0.00480353	1.15786751	0.241	0.203	1	Epi
RRAGB2	0.00484607	0.37155602	0.076	0.126	1	Epi
MGMT1	0.00488183	0.41982971	0.368	0.515	1	Epi
CALML4	0.00496199	1.39119623	0.211	0.174	1	Epi
NAE13	0.00498151	0.46129666	0.231	0.319	1	Epi
PLEKHA82	0.00500248	0.55687852	0.074	0.122	1	Epi
DGCR82	0.00502346	0.4024241	0.19	0.267	1	Epi
CCER2	0.00502413	2.57086949	0.013	0.004	1	Epi
NR2C12	0.0050705	0.37224372	0.099	0.155	1	Epi
BAHCC11	0.00510042	1.70044041	0.094	0.063	1	Epi
DOLPP12	0.00511625	0.32355744	0.056	0.1	1	Epi
HSPA1B2	0.00514641	1.69129806	0.556	0.652	1	Epi
TRIM111	0.00516346	0.8185056	0.188	0.272	1	Epi
KLHL56	0.0051753	1.04963613	0.254	0.221	1	Epi
RGL32	0.00518006	0.23142305	0.074	0.123	1	Epi
KCNJ142	0.00520055	2.04676473	0.028	0.012	1	Epi
ZBTB412	0.00521373	0.28789762	0.198	0.275	1	Epi
MC1R2	0.00522252	2.40569173	0.03	0.014	1	Epi
ZMYND192	0.00524612	0.15053208	0.107	0.161	1	Epi
TAOK22	0.00537568	0.44434618	0.34	0.469	1	Epi
STN11	0.00545023	1.38062307	0.246	0.217	1	Epi
PLA2G4B1	0.00552489	1.52957218	0.104	0.07	1	Epi
DTD22	0.00552596	0.33589692	0.063	0.111	1	Epi
EEF2KMT1	0.0055331	0.21552894	0.069	0.115	1	Epi
ZNF3332	0.00554357	0.20569139	0.124	0.186	1	Epi
CNNM42	0.00556383	0.34911688	0.198	0.271	1	Epi
COQ8B2	0.00556911	1.83192343	0.292	0.28	1	Epi
WDR771	0.00560173	0.78171878	0.216	0.304	1	Epi
LRRC422	0.00563326	0.25136767	0.162	0.236	1	Epi
EPHB22	0.00563529	1.8513838	0.02	0.008	1	Epi
SMUG1	0.00563844	0.40485143	0.165	0.236	1	Epi
SRBD12	0.00564682	0.31232348	0.129	0.193	1	Epi
ZNF239	0.00565079	2.08592434	0.01	0.003	1	Epi
USP472	0.00565101	0.4657686	0.239	0.33	1	Epi
C11orf742	0.00566627	1.69048394	0.211	0.179	1	Epi
STK11IP2	0.00579257	0.30357853	0.289	0.399	1	Epi
SLAMF61	0.00579642	0.16444664	0.025	0.058	1	Epi
DALRD32	0.00583551	1.58558593	0.213	0.18	1	Epi
H3F3B5	0.00583991	0.41863882	0.193	0.269	1	Epi

CDK5RAP21	0.0058509	0.40634756	0.322	0.44	1	Epi
UBE2G12	0.00588915	0.64027715	0.401	0.562	1	Epi
ANKHD14	0.00589631	0.31035475	0.515	0.711	1	Epi
INTS16	0.00591178	0.53345361	0.391	0.532	1	Epi
RPRD22	0.00591662	0.4562401	0.287	0.39	1	Epi
KLKB12	0.00592528	2.14925039	0.051	0.028	1	Epi
PIGL2	0.00593408	1.7464074	0.218	0.185	1	Epi
KAT141	0.00594932	0.39460776	0.071	0.119	1	Epi
PKD1L1	0.00594937	2.56659734	0.013	0.004	1	Epi
SLC25A10	0.00596323	1.92116102	0.084	0.054	1	Epi
BAZ1A3	0.00598971	0.39814204	0.383	0.526	1	Epi
KTN11	0.00601103	0.26973497	0.48	0.687	1	Epi
HSBP1L11	0.00611974	1.40068221	0.208	0.171	1	Epi
PPFIBP13	0.00615688	0.21213615	0.096	0.15	1	Epi
TKFC2	0.00616916	1.33905599	0.343	0.331	1	Epi
KLHL73	0.00617486	0.30510931	0.193	0.271	1	Epi
CKAP41	0.00618327	0.69908053	0.213	0.295	1	Epi
GPATCH23	0.00618615	0.37719689	0.201	0.287	1	Epi
KIF13A2	0.00619078	0.56654037	0.437	0.59	1	Epi
SLC27A51	0.00621137	0.60053753	0.051	0.092	1	Epi
CSNK1E3	0.00623028	1.49257353	0.462	0.494	1	Epi
ZNF1772	0.00624995	1.72924145	0.071	0.044	1	Epi
ZNF3042	0.00626462	0.30867777	0.102	0.154	1	Epi
ARHGAP183	0.00628025	0.26631797	0.65	0.875	1	Epi
INTS62	0.0063623	0.77920469	0.277	0.387	1	Epi
MARCH52	0.00636787	0.61718843	0.325	0.462	1	Epi
GAS74	0.00637311	0.33382648	0.332	0.441	1	Epi
FGF111	0.00638673	3.22858026	0.02	0.008	1	Epi
S1PR24	0.00639502	0.14123357	0.061	0.103	1	Epi
ZNF5032	0.00640478	0.15126472	0.119	0.176	1	Epi
FOXRED11	0.00641329	0.6614453	0.185	0.26	1	Epi
SLC7A64	0.00645332	1.27602504	0.292	0.272	1	Epi
CDKL53	0.00646563	0.57190703	0.162	0.231	1	Epi
PPP6R22	0.00649386	0.44285767	0.452	0.607	1	Epi
DEAF13	0.00650655	0.54293191	0.157	0.224	1	Epi
GON4L3	0.00654209	0.32186286	0.312	0.437	1	Epi
CTDSPL	0.00655115	0.38672165	0.102	0.157	1	Epi
MPHOSPH92	0.00659515	0.40048268	0.175	0.253	1	Epi
NIPSNAP3B5	0.00660729	1.95316977	0.051	0.029	1	Epi
DHX302	0.00666987	0.49817537	0.35	0.494	1	Epi
SEC14L13	0.0066723	0.35134756	0.464	0.641	1	Epi
PCMTD21	0.00671626	0.38839582	0.261	0.36	1	Epi
CTNS3	0.0067602	0.33227332	0.208	0.287	1	Epi
IMPA13	0.00681107	0.21839172	0.239	0.329	1	Epi
MANEAL1	0.00681807	0.25956974	0.038	0.076	1	Epi
LRRC572	0.00685933	0.42946988	0.325	0.424	1	Epi
PHF24	0.00687639	0.26641522	0.345	0.467	1	Epi
CASD11	0.00688543	0.42346921	0.193	0.272	1	Epi
PPP5D1	0.00688745	3.29566697	0.013	0.004	1	Epi
LANCL21	0.0069076	0.48345233	0.185	0.263	1	Epi
ZMYND83	0.00697049	0.57443791	0.388	0.529	1	Epi
JMJD42	0.00698431	0.48788904	0.129	0.191	1	Epi

PRMT72	0.0070433	0.51538891	0.155	0.222	1	Epi
ZFAND12	0.00704709	0.36592145	0.246	0.337	1	Epi
NAPSA	0.00704904	6.04305431	0.018	0.007	1	Epi
METTL2A3	0.00706186	0.14817449	0.17	0.237	1	Epi
RPRD1A2	0.00706593	0.33432556	0.32	0.43	1	Epi
ST144	0.00709995	0.82084599	0.561	0.659	1	Epi
MT1X1	0.00710386	1.40774328	0.063	0.038	1	Epi
DFFB3	0.0071208	0.49051683	0.043	0.081	1	Epi
CENPC3	0.00713528	0.19310298	0.117	0.178	1	Epi
PHRF11	0.0072414	0.27178455	0.277	0.362	1	Epi
ALG63	0.00724971	0.14002747	0.099	0.152	1	Epi
CEP194	0.00727766	1.93124067	0.086	0.057	1	Epi
C1orf1092	0.00733024	1.03436614	0.079	0.125	1	Epi
PCM12	0.0073491	0.97996807	0.609	0.765	1	Epi
SIAE	0.00736202	0.5064455	0.185	0.264	1	Epi
CCHCR12	0.00736647	1.22162039	0.165	0.128	1	Epi
YOD12	0.00739278	1.86971615	0.122	0.09	1	Epi
ZKSCAN81	0.00743534	0.23066841	0.206	0.289	1	Epi
AHRR1	0.00747019	2.28273062	0.015	0.005	1	Epi
ZNF8412	0.00753158	0.4653703	0.132	0.191	1	Epi
HACE11	0.00755257	0.49984375	0.109	0.166	1	Epi
GMPPB2	0.00755439	0.46317831	0.348	0.471	1	Epi
PSD	0.00759829	2.46536189	0.013	0.004	1	Epi
ZNF1602	0.00760043	0.51942816	0.147	0.218	1	Epi
MFSD32	0.00761609	0.6995992	0.109	0.164	1	Epi
BRD12	0.00764067	0.38096621	0.447	0.607	1	Epi
ZNF1242	0.00765365	0.48437506	0.03	0.063	1	Epi
FOLR11	0.00768528	0.73831203	0.124	0.185	1	Epi
FAM172A2	0.00769118	0.27441682	0.272	0.374	1	Epi
NEDD12	0.00769341	0.55955576	0.16	0.233	1	Epi
KLF33	0.00770737	0.48349405	0.444	0.631	1	Epi
ABCA21	0.00772023	1.20218775	0.234	0.203	1	Epi
MAP3K43	0.00776055	0.45025938	0.266	0.368	1	Epi
PRUNE21	0.00779617	0.20643734	0.203	0.283	1	Epi
EXTL21	0.00780316	0.57633109	0.132	0.198	1	Epi
PRR51	0.00781073	1.95479909	0.041	0.021	1	Epi
SLC25A374	0.00783981	1.16277798	0.216	0.18	1	Epi
SMURF23	0.00786086	0.20662857	0.241	0.331	1	Epi
TMTC22	0.0078803	0.54994024	0.188	0.264	1	Epi
ZNF5612	0.00789096	0.44930343	0.244	0.34	1	Epi
SLC37A41	0.00789348	0.26298184	0.206	0.282	1	Epi
ACAD101	0.00792168	0.47896083	0.109	0.167	1	Epi
TEX29	0.00792456	2.65503179	0.015	0.005	1	Epi
TRRAP2	0.00792975	0.45882863	0.378	0.505	1	Epi
RNF1692	0.00795265	1.00952285	0.335	0.471	1	Epi
TUT12	0.00800432	0.22508908	0.15	0.216	1	Epi
POGZ2	0.00800821	0.58748999	0.345	0.483	1	Epi
CA12	0.0080111	2.51208456	0.018	0.007	1	Epi
LGALSL2	0.0080399	0.45001305	0.107	0.162	1	Epi
HDAC96	0.00805146	1.70516591	0.112	0.079	1	Epi
CEP1353	0.00810208	0.60286	0.124	0.185	1	Epi
ANXA111	0.00811765	0.25591751	0.779	0.952	1	Epi

EP4002	0.0081406	0.5910795	0.081	0.132	1	Epi
FBXO24	0.00815462	4.02480944	0.015	0.005	1	Epi
BMI12	0.00816292	0.3151113	0.254	0.349	1	Epi
PRDM22	0.00824577	0.33699266	0.401	0.538	1	Epi
SLC26A21	0.00826864	0.55630324	0.185	0.261	1	Epi
NBPF202	0.00828825	1.95612155	0.053	0.031	1	Epi
ACTR3B1	0.00834991	1.57850747	0.079	0.051	1	Epi
TADA11	0.00836756	0.24113516	0.046	0.083	1	Epi
GOLGA13	0.00845875	0.36177607	0.274	0.371	1	Epi
ITGA72	0.00845949	3.50275634	0.01	0.003	1	Epi
ABRAXAS13	0.00850665	1.22665768	0.14	0.104	1	Epi
YJEFN31	0.00853383	1.77721027	0.028	0.013	1	Epi
ORC33	0.00855356	0.53198764	0.147	0.208	1	Epi
UIMC11	0.00863945	0.19408075	0.137	0.196	1	Epi
BRD3OS2	0.00864816	1.53332192	0.282	0.264	1	Epi
TMED41	0.00865882	0.39154843	0.49	0.675	1	Epi
ERCC12	0.00869216	0.64019376	0.388	0.528	1	Epi
ALDH3B11	0.00871294	1.58615243	0.576	0.736	1	Epi
NUDCD11	0.00874867	0.45216043	0.17	0.236	1	Epi
SH3PXD2B2	0.00883579	0.48463991	0.145	0.208	1	Epi
CFAP972	0.00887019	0.47997818	0.193	0.275	1	Epi
SEPHS13	0.0088736	0.4367009	0.14	0.204	1	Epi
HSPA141	0.00888159	0.3336587	0.076	0.123	1	Epi
GNA12	0.00905801	0.57592733	0.056	0.095	1	Epi
CHIC23	0.00906328	0.44959739	0.32	0.437	1	Epi
KCTD111	0.00908969	1.62888533	0.117	0.085	1	Epi
EHD33	0.00909001	2.40564661	0.025	0.011	1	Epi
AREG1	0.0091646	0.40155163	0.119	0.173	1	Epi
VPS523	0.00916923	0.58869225	0.332	0.451	1	Epi
CCDC85C1	0.00917735	0.72022027	0.104	0.16	1	Epi
LGALS12	0.00921403	0.97095408	0.013	0.038	1	Epi
FBXL192	0.00934074	0.36539803	0.185	0.259	1	Epi
ZNF774	0.00934101	2.7239905	0.013	0.004	1	Epi
TJAP12	0.00938081	0.37351434	0.152	0.217	1	Epi
TRADD1	0.00938309	0.40982784	0.365	0.503	1	Epi
BBIP13	0.00940138	1.12173524	0.165	0.129	1	Epi
ZNF1323	0.0094192	1.92840219	0.086	0.058	1	Epi
CUX12	0.00945504	0.73892153	0.365	0.486	1	Epi
HNRNPF2	0.0094764	0.30665807	0.614	0.865	1	Epi
CCNT24	0.00948112	0.51409468	0.284	0.381	1	Epi
PPP1R13L3	0.00950492	1.69572895	0.137	0.104	1	Epi
FARP12	0.00952343	1.33599498	0.33	0.316	1	Epi
CDC344	0.00953833	0.59294864	0.315	0.451	1	Epi
ZNF3942	0.00954742	0.6422645	0.287	0.385	1	Epi
UBAP22	0.00963266	0.57623888	0.198	0.27	1	Epi
MEF2B4	0.00965782	1.39482501	0.074	0.047	1	Epi
PKN31	0.00966295	1.94559569	0.033	0.017	1	Epi
BAZ2A2	0.00969104	0.46505121	0.335	0.449	1	Epi
FYCO11	0.00969338	0.38035577	0.175	0.244	1	Epi
TCHH1	0.00969697	0.57661516	0.018	0.045	1	Epi
ATP13A21	0.00973655	0.32361416	0.135	0.194	1	Epi
EIF2AK32	0.00985645	0.19686583	0.145	0.206	1	Epi

TMEM422	0.00986457	0.63436614	0.142	0.199	1	Epi
ARFRP12	0.00994986	0.569284	0.272	0.363	1	Epi
CDT1	0	6.47668202	0.624	0.019	0	Cycling
TCF191	0	4.43694769	0.694	0.116	0	Cycling
E2F1	0	5.19676543	0.609	0.049	0	Cycling
MCM2	0	3.59771409	0.674	0.146	0	Cycling
TK1	0	5.2683755	0.575	0.059	0	Cycling
PHF193	0	3.24772045	0.609	0.117	0	Cycling
MCM71	0	2.96078102	0.777	0.309	0	Cycling
ZWINT	0	3.44335101	0.588	0.121	0	Cycling
CENPM1	0	5.50662481	0.479	0.022	0	Cycling
HELLS1	0	3.94926953	0.505	0.066	0	Cycling
MCM61	0	2.58047766	0.681	0.249	0	Cycling
ASF1B	0	5.04275675	0.47	0.038	0	Cycling
SHCBP1	0	4.86884475	0.451	0.028	0	Cycling
TYMS	0	7.42879261	0.427	0.016	0	Cycling
LMNB14	0	2.61862579	0.624	0.215	0	Cycling
NUSAP11	0	4.12818037	0.519	0.111	0	Cycling
MCM4	0	2.6285376	0.754	0.35	0	Cycling
GGH	0	3.15969007	0.508	0.11	0	Cycling
CENPU1	0	3.67692329	0.455	0.066	0	Cycling
SMC21	0	2.00058641	0.657	0.271	0	Cycling
FEN1	0	2.86719017	0.532	0.146	0	Cycling
FANCI1	0	3.55335569	0.457	0.076	0	Cycling
RAD51AP1	0	3.52700154	0.443	0.063	0	Cycling
NCAPG22	0	2.81372905	0.511	0.132	0	Cycling
MCM31	0	2.21612478	0.694	0.32	0	Cycling
BRCA1	0	2.96919657	0.449	0.076	0	Cycling
CDK11	0	6.03944724	0.397	0.031	0	Cycling
KIFC1	0	5.59926076	0.387	0.024	0	Cycling
WDR76	0	3.25281151	0.413	0.05	0	Cycling
LIG12	0	2.00109423	0.608	0.248	0	Cycling
UHRF1	0	7.16076183	0.366	0.007	0	Cycling
SMC41	0	2.27265258	0.553	0.195	0	Cycling
KIF221	0	2.68140721	0.484	0.126	0	Cycling
CEP55	0	6.24971966	0.368	0.012	0	Cycling
CCNB2	0	4.35216718	0.4	0.045	0	Cycling
TPX2	0	6.21570608	0.373	0.02	0	Cycling
PCLAF	0	6.82090301	0.355	0.006	0	Cycling
DTL	0	7.06681033	0.351	0.004	0	Cycling
BUB1	0	4.62021675	0.378	0.033	0	Cycling
MAD2L11	0	4.02670719	0.374	0.032	0	Cycling
CHEK1	0	3.47526076	0.38	0.039	0	Cycling
MKI67	0	7.58367447	0.345	0.008	0	Cycling
KNTC12	0	2.20326654	0.447	0.112	0	Cycling
POLD1	0	1.66930652	0.716	0.381	0	Cycling
CENPW	0	1.82656722	0.64	0.306	0	Cycling
ATAD21	0	3.4379311	0.384	0.05	0	Cycling
BRCA2	0	1.95049276	0.498	0.164	0	Cycling
CENPN	0	1.78626027	0.597	0.263	0	Cycling
ZNF3671	0	4.95290081	0.345	0.012	0	Cycling
TACC32	0	2.32360306	0.665	0.333	0	Cycling

TMEM106C1	0	2.33108285	0.663	0.333	0	Cycling
ORC6	0	3.4277694	0.371	0.041	0	Cycling
RRM11	0	2.06459201	0.676	0.348	0	Cycling
PRIM11	0	2.09805151	0.464	0.136	0	Cycling
GINS21	0	4.47013973	0.347	0.019	0	Cycling
TIMELESS1	0	2.66106892	0.404	0.077	0	Cycling
POC1A	0	3.0896398	0.395	0.072	0	Cycling
CENPH1	0	2.04775163	0.452	0.129	0	Cycling
CIT	0	5.08010581	0.342	0.02	0	Cycling
CHAF1A3	0	2.29693866	0.444	0.122	0	Cycling
PCNA1	0	2.37263905	0.529	0.209	0	Cycling
FANCA	0	2.67034495	0.396	0.076	0	Cycling
FOXM11	0	6.43721235	0.327	0.008	0	Cycling
SHMT11	0	1.88714361	0.496	0.177	0	Cycling
DSN12	0	1.67139587	0.514	0.196	0	Cycling
CDKN2C1	0	3.44665375	0.434	0.118	0	Cycling
CDK21	0	2.06985631	0.456	0.14	0	Cycling
CARHSP11	0	1.96779128	0.51	0.194	0	Cycling
MCM82	0	2.23263416	0.414	0.1	0	Cycling
CLSPN	0	5.31498002	0.32	0.011	0	Cycling
CENPP2	0	1.59108698	0.512	0.203	0	Cycling
GINS11	0	4.22885729	0.329	0.021	0	Cycling
GMNN1	0	2.017505	0.462	0.155	0	Cycling
FAM111B	0	6.645808	0.313	0.006	0	Cycling
SPAG5	0	4.12725387	0.349	0.042	0	Cycling
DTYMK	0	1.76265567	0.636	0.33	0	Cycling
RFC31	0	2.53880366	0.391	0.085	0	Cycling
NEMP12	0	1.61928762	0.518	0.213	0	Cycling
UBE2T1	0	3.10653442	0.377	0.074	0	Cycling
CENPF	0	4.82664112	0.343	0.041	0	Cycling
STMN1	0	1.87446095	0.823	0.522	0	Cycling
WDR341	0	2.49473829	0.406	0.107	0	Cycling
OIP5	0	4.34337384	0.322	0.023	0	Cycling
H2AFX1	0	2.91269124	0.443	0.146	0	Cycling
PKMYT1	0	7.73818934	0.299	0.002	0	Cycling
CKS1B	0	1.60425926	0.76	0.464	0	Cycling
KIF111	0	5.23284644	0.314	0.018	0	Cycling
NCAPD32	0	1.54919536	0.58	0.285	0	Cycling
POGLUT31	0	2.17400011	0.44	0.147	0	Cycling
ORC1	0	3.76140256	0.318	0.025	0	Cycling
RAD51	0	4.74061797	0.308	0.016	0	Cycling
HMGB21	0	2.48199273	0.545	0.255	0	Cycling
NRM2	0	2.70253038	0.354	0.065	0	Cycling
DHFR3	0	1.72891686	0.569	0.281	0	Cycling
NUDT11	0	1.20468923	0.694	0.407	0	Cycling
WEE11	0	4.0858922	0.3	0.013	0	Cycling
EZH23	0	2.67316077	0.375	0.091	0	Cycling
C19orf481	0	1.71782771	0.431	0.147	0	Cycling
POLA23	0	1.46472792	0.475	0.191	0	Cycling
TOP2A	0	7.13223452	0.291	0.007	0	Cycling
CEP1284	0	1.45890394	0.446	0.164	0	Cycling
POLE5	0	1.52601388	0.442	0.161	0	Cycling

DNMT13	0	1.40433162	0.792	0.511	0	Cycling
TMPO2	0	1.62226302	0.692	0.411	0	Cycling
NCAPG	0	6.74255015	0.285	0.005	0	Cycling
TFDP12	0	1.22148249	0.626	0.347	0	Cycling
CDC45	0	6.94512214	0.281	0.003	0	Cycling
VRK12	0	1.16851588	0.511	0.235	0	Cycling
MELK1	0	5.62445335	0.281	0.007	0	Cycling
BIRC5	0	7.78284777	0.277	0.003	0	Cycling
MND1	0	6.61442121	0.277	0.004	0	Cycling
ANLN	0	7.52969936	0.277	0.005	0	Cycling
CKS21	0	3.11227409	0.352	0.08	0	Cycling
ZWILCH2	0	1.77056091	0.393	0.123	0	Cycling
SUV39H11	0	1.76326585	0.389	0.12	0	Cycling
MYO194	0	1.34570692	0.489	0.221	0	Cycling
NCAPD22	0	2.00263377	0.567	0.299	0	Cycling
USP17	0	1.38361311	0.606	0.34	0	Cycling
NRGN2	0	1.79637359	0.419	0.154	0	Cycling
MYBL21	0	7.20596019	0.268	0.004	0	Cycling
TOPBP11	0	1.1634743	0.5	0.237	0	Cycling
PRC11	0	5.56959168	0.274	0.011	0	Cycling
HIRIP31	0	1.39371068	0.465	0.202	0	Cycling
RBL13	0	1.37830699	0.409	0.147	0	Cycling
HMMR	0	6.15356976	0.273	0.012	0	Cycling
CBX51	0	1.3676332	0.424	0.163	0	Cycling
PMM12	0	1.24444492	0.465	0.206	0	Cycling
CENPS1	0	2.14632125	0.344	0.086	0	Cycling
NUCB22	0	1.27768854	0.614	0.356	0	Cycling
CDCA51	0	6.23286652	0.263	0.006	0	Cycling
PSMC3IP1	0	3.17963633	0.292	0.036	0	Cycling
PRR11	0	3.0289165	0.326	0.07	0	Cycling
KIAA15223	0	1.09025807	0.602	0.347	0	Cycling
NDC801	0	5.73160068	0.263	0.008	0	Cycling
IDH23	0	1.67093098	0.814	0.559	0	Cycling
RRM2	0	7.75206137	0.26	0.006	0	Cycling
CKAP2L	0	6.53326426	0.259	0.005	0	Cycling
CENPE	0	2.9522304	0.327	0.073	0	Cycling
DEK2	0	1.09726535	0.75	0.496	0	Cycling
SAE12	0	1.301754	0.641	0.388	0	Cycling
IMPA21	0	1.25697973	0.535	0.284	0	Cycling
NUP2102	0	0.95207675	0.72	0.47	0	Cycling
LRP112	0	1.58549976	0.459	0.209	0	Cycling
MCM10	0	7.42712754	0.251	0.001	0	Cycling
RMI23	0	2.55396607	0.297	0.047	0	Cycling
MSH63	0	1.27325256	0.502	0.253	0	Cycling
PHGDH4	0	4.1562488	0.28	0.032	0	Cycling
CD109	0	0.89963158	0.685	0.438	0	Cycling
ARHGAP11A1	0	3.25088468	0.288	0.042	0	Cycling
SLC2A4RG1	0	0.94752328	0.654	0.409	0	Cycling
KIF20B1	0	2.46431952	0.312	0.067	0	Cycling
ATAD51	0	2.25333314	0.319	0.074	0	Cycling
ECT22	0	1.72538789	0.409	0.165	0	Cycling
HIST1H1B2	0	6.31968384	0.278	0.036	0	Cycling

NCAPH21	0	1.10011498	0.69	0.448	0	Cycling
FAM111A1	0	1.43697182	0.768	0.527	0	Cycling
E2F21	0	6.21813954	0.242	0.003	0	Cycling
KIF2C	0	6.22454579	0.246	0.007	0	Cycling
IQGAP3	0	4.88125603	0.262	0.024	0	Cycling
KNL1	0	5.06979211	0.249	0.012	0	Cycling
EXO1	0	5.60958158	0.243	0.007	0	Cycling
DCK4	0	1.35981639	0.744	0.508	0	Cycling
PTTG11	0	3.87312286	0.291	0.055	0	Cycling
SNRNP25	0	1.15526011	0.711	0.475	0	Cycling
BRIP1	0	5.15721573	0.243	0.007	0	Cycling
RFWD31	0	1.24112155	0.486	0.251	0	Cycling
MGST11	0	0.79502885	0.688	0.453	0	Cycling
EPB41L22	0	0.99060265	0.522	0.288	0	Cycling
FANCG2	0	1.96893483	0.32	0.087	0	Cycling
BARD12	0	2.38664798	0.295	0.062	0	Cycling
MMS22L2	0	1.43693529	0.367	0.135	0	Cycling
POLA11	0	1.4281191	0.409	0.177	0	Cycling
POLQ1	0	4.88481431	0.241	0.01	0	Cycling
H2AFZ	0	1.74273937	0.857	0.626	0	Cycling
MTFR22	0	1.50227379	0.371	0.14	0	Cycling
HIST1H1D3	0	2.7828148	0.333	0.103	0	Cycling
TUBG1	0	1.2034295	0.656	0.426	0	Cycling
SAP304	0	1.15329149	0.474	0.244	0	Cycling
CKAP42	0	1.00848045	0.506	0.276	0	Cycling
HIST1H1C4	0	2.52760902	0.673	0.444	0	Cycling
LMNB21	0	1.30611655	0.686	0.457	0	Cycling
DONSON1	0	1.92018394	0.32	0.091	0	Cycling
PAFAH1B31	0	1.03520371	0.491	0.262	0	Cycling
MAP3K204	0	1.06487434	0.435	0.208	0	Cycling
CDC6	0	5.42857749	0.233	0.006	0	Cycling
SPC24	0	6.96383598	0.229	0.002	0	Cycling
CENPI1	0	3.37332511	0.257	0.03	0	Cycling
TTK	0	4.8848168	0.238	0.011	0	Cycling
CASP24	0	0.9451648	0.487	0.26	0	Cycling
HJURP	0	7.57060672	0.228	0.002	0	Cycling
IFI27L11	0	1.37031257	0.381	0.155	0	Cycling
RMI1	0	1.23171919	0.421	0.195	0	Cycling
NCAPH1	0	5.81755253	0.228	0.003	0	Cycling
HASPIN1	0	5.3156708	0.232	0.007	0	Cycling
MIS18A2	0	1.27276461	0.386	0.161	0	Cycling
CENPX1	0	1.00589787	0.53	0.306	0	Cycling
SAC3D1	0	1.75346994	0.432	0.208	0	Cycling
LIN542	0	1.00440819	0.517	0.294	0	Cycling
HIST1H4C1	0	2.68923908	0.358	0.135	0	Cycling
MASTL1	0	1.38075809	0.414	0.191	0	Cycling
SGO21	0	2.83717072	0.274	0.052	0	Cycling
CENPJ1	0	1.57470729	0.334	0.112	0	Cycling
RBBP73	0	0.98745996	0.529	0.307	0	Cycling
STIL2	0	3.58045776	0.246	0.024	0	Cycling
MSH22	0	1.02933702	0.476	0.254	0	Cycling
UBR71	0	1.11770363	0.503	0.281	0	Cycling

INCENP	0	1.63871951	0.558	0.337	0	Cycling
TRIP13	0	4.17344076	0.239	0.018	0	Cycling
NR2C2AP3	0	1.12533552	0.464	0.244	0	Cycling
NUF21	0	5.61081878	0.226	0.007	0	Cycling
ESPL1	0	5.46676818	0.227	0.008	0	Cycling
WDHD11	0	2.65480909	0.259	0.04	0	Cycling
SLC1A44	0	1.53616321	0.412	0.193	0	Cycling
JPT24	0	0.88599419	0.531	0.313	0	Cycling
IL17RB	0	1.34944428	0.426	0.208	0	Cycling
SAMD16	0	1.04952278	0.576	0.358	0	Cycling
DEPDC1B1	0	5.37927862	0.225	0.008	0	Cycling
CKAP22	0	1.76260778	0.369	0.152	0	Cycling
TEDC15	0	0.64828776	0.485	0.268	0	Cycling
BCL2L123	0	1.37192588	0.366	0.15	0	Cycling
GTSE1	0	7.41417806	0.218	0.002	0	Cycling
ZGRF12	0	2.12513508	0.284	0.068	0	Cycling
CDK42	0	0.89755988	0.757	0.541	0	Cycling
BRI3BP1	0	0.97995055	0.493	0.277	0	Cycling
THOP12	0	0.8094245	0.528	0.313	0	Cycling
RAD54L	0	6.1817094	0.217	0.003	0	Cycling
RCC1	0	1.33512643	0.385	0.172	0	Cycling
CEP782	0	1.35050115	0.362	0.149	0	Cycling
GINS41	0	3.01429594	0.241	0.029	0	Cycling
PARPBP2	0	2.65873708	0.26	0.049	0	Cycling
GEN11	0	1.14892915	0.405	0.195	0	Cycling
HIST1H2BH1	0	4.57176791	0.265	0.055	0	Cycling
LRRC451	0	1.17196675	0.392	0.182	0	Cycling
PRPS2	0	0.91218385	0.675	0.465	0	Cycling
CENPA	0	5.5379923	0.22	0.01	0	Cycling
CDC25B2	0	1.21607779	0.473	0.263	0	Cycling
PLP24	0	0.84411992	0.763	0.553	0	Cycling
CENPL1	0	2.21094487	0.27	0.061	0	Cycling
RFC41	0	1.98718612	0.277	0.068	0	Cycling
DNAJC17	0	0.67591658	0.56	0.352	0	Cycling
DDX39A3	0	0.78715773	0.632	0.424	0	Cycling
AURKB	0	6.49929517	0.213	0.005	0	Cycling
DNAJC93	0	1.65022072	0.292	0.084	0	Cycling
CDCA8	0	3.93864721	0.232	0.025	0	Cycling
MPHOSPH93	0	1.05724668	0.442	0.235	0	Cycling
FN3KRP1	0	0.72776552	0.626	0.419	0	Cycling
SKA1	0	6.4950271	0.21	0.003	0	Cycling
C9orf401	0	2.06418421	0.286	0.079	0	Cycling
GINS3	0	2.81308539	0.24	0.034	0	Cycling
MAD2L22	0	0.96855013	0.517	0.312	0	Cycling
PSIP11	0	1.15872817	0.335	0.13	0	Cycling
TMEM971	0	1.44898757	0.33	0.125	0	Cycling
CDCA2	0	6.00610801	0.21	0.005	0	Cycling
BUB1B1	0	6.33325118	0.208	0.003	0	Cycling
UBE2C	0	5.92417233	0.212	0.007	0	Cycling
KIF23	0	4.33133629	0.231	0.027	0	Cycling
CHAF1B1	0	3.82306835	0.219	0.015	0	Cycling
DLGAP5	0	7.94228937	0.206	0.002	0	Cycling

NETO23	0	2.13825493	0.286	0.082	0	Cycling
SLC37A42	0	0.95204361	0.468	0.265	0	Cycling
SPC25	0	8.04809732	0.204	0.001	0	Cycling
SGO1	0	5.36419498	0.207	0.006	0	Cycling
CCNA2	0	5.56099239	0.207	0.006	0	Cycling
MCM52	0	2.13199141	0.831	0.63	0	Cycling
KIF15	0	6.30382	0.204	0.004	0	Cycling
SKA3	0	4.80367943	0.209	0.009	0	Cycling
CCNB1	0	3.73354799	0.25	0.051	0	Cycling
CDC20	0	6.5711532	0.204	0.005	0	Cycling
ACD2	0	0.75411856	0.472	0.273	0	Cycling
NDC13	0	0.84811644	0.51	0.312	0	Cycling
GPSM21	0	3.5521053	0.234	0.036	0	Cycling
IPO94	0	0.60981209	0.605	0.407	0	Cycling
GMPS2	0	0.90656677	0.451	0.253	0	Cycling
ESCO2	0	5.41070045	0.205	0.007	0	Cycling
POLD31	0	0.80003902	0.68	0.483	0	Cycling
EXOSC83	0	0.7455273	0.497	0.3	0	Cycling
NEMP22	0	1.71508187	0.271	0.075	0	Cycling
CENPK1	0	3.67139833	0.209	0.013	0	Cycling
TPGS21	0	0.66654929	0.578	0.382	0	Cycling
POLE32	0	0.76441411	0.786	0.591	0	Cycling
PMP222	0	0.60642371	0.653	0.458	0	Cycling
HLTF2	0	0.82854486	0.471	0.276	0	Cycling
TTF22	0	0.82144512	0.467	0.273	0	Cycling
SEPTIN8	0	0.65335572	0.602	0.408	0	Cycling
KNSTRN	0	1.48987607	0.44	0.246	0	Cycling
RUSC12	0	0.98480277	0.408	0.214	0	Cycling
RASSF12	0	0.63285901	0.588	0.394	0	Cycling
C1orf1123	0	1.34419279	0.319	0.126	0	Cycling
NAP1L42	0	0.5387664	0.654	0.461	0	Cycling
XRCC31	0	1.93210163	0.256	0.063	0	Cycling
ADAM153	0	0.58850778	0.727	0.534	0	Cycling
PCNT3	0	0.65488253	0.55	0.358	0	Cycling
KPNA2	0	1.74036659	0.447	0.255	0	Cycling
KIF4A	0	6.99149537	0.193	0.001	0	Cycling
RPA11	0	0.69598821	0.734	0.542	0	Cycling
ANP32E3	0	1.19076527	0.426	0.235	0	Cycling
PLK1	0	5.84192331	0.206	0.015	0	Cycling
FADS11	0	0.84661365	0.568	0.377	0	Cycling
ASRGL11	0	1.05593001	0.402	0.212	0	Cycling
KIF14	0	6.21385013	0.194	0.004	0	Cycling
KIF20A	0	8.10712198	0.191	0.001	0	Cycling
NABP21	0	0.66038561	0.614	0.424	0	Cycling
PXMP21	0	1.77002121	0.265	0.075	0	Cycling
MZT12	0	0.95396278	0.642	0.452	0	Cycling
NFATC2IP3	0	0.55760918	0.637	0.447	0	Cycling
BTG32	0	1.07809007	0.439	0.249	0	Cycling
SMC31	0	0.71020367	0.708	0.518	0	Cycling
VCAN2	0	0.6547828	0.429	0.24	0	Cycling
TCOF11	0	0.57835777	0.651	0.462	0	Cycling
PRRG4	0	1.50286728	0.306	0.117	0	Cycling

RPP25	0	0.82300494	0.498	0.309	0	Cycling
ACTL6A1	0	0.70301016	0.538	0.35	0	Cycling
RECQL4	0	3.874139	0.203	0.015	0	Cycling
TROAP	0	6.7256213	0.191	0.003	0	Cycling
PPP2R3B3	0	1.00410003	0.361	0.173	0	Cycling
MGME13	0	0.66630381	0.532	0.345	0	Cycling
FANCC1	0	1.61063464	0.273	0.086	0	Cycling
ITGB3BP2	0	1.31546782	0.306	0.119	0	Cycling
CDCA7L	0	0.88117696	0.47	0.283	0	Cycling
MCUB2	0	0.53313059	0.588	0.401	0	Cycling
HAUS4	0	0.58102815	0.718	0.531	0	Cycling
CCL231	0	0.46452442	0.565	0.378	0	Cycling
CGAS1	0	0.53079511	0.677	0.491	0	Cycling
HPRT12	0	0.70575648	0.56	0.374	0	Cycling
DNA22	0	2.16351615	0.236	0.051	0	Cycling
TEX301	0	1.18631238	0.323	0.138	0	Cycling
ERI2	0	1.09897527	0.383	0.198	0	Cycling
RCCD12	0	1.23279893	0.356	0.171	0	Cycling
ICMT	0	0.86112073	0.758	0.574	0	Cycling
CPNE23	0	0.72791843	0.466	0.282	0	Cycling
MXD32	0	4.41452207	0.199	0.015	0	Cycling
VSIG102	0	1.82955749	0.262	0.078	0	Cycling
CCNF1	0	3.84895483	0.208	0.024	0	Cycling
NUP504	0	0.59637221	0.6	0.416	0	Cycling
FANCD21	0	1.96580397	0.241	0.059	0	Cycling
ZDHHC122	0	0.82715429	0.459	0.277	0	Cycling
CCDC342	0	2.86806903	0.214	0.032	0	Cycling
SPATA53	0	0.7631183	0.386	0.205	0	Cycling
NEDD13	0	0.83779411	0.399	0.218	0	Cycling
NSD22	0	1.17879665	0.32	0.139	0	Cycling
SFXN12	0	0.95626615	0.297	0.116	0	Cycling
FIGNL13	0	1.65682466	0.254	0.073	0	Cycling
RPA32	0	1.05360401	0.344	0.163	0	Cycling
LRR13	0	1.04568554	0.347	0.166	0	Cycling
HAT12	0	0.67242349	0.728	0.547	0	Cycling
RAD212	0	0.80466787	0.564	0.383	0	Cycling
SRPK14	0	0.45280566	0.547	0.367	0	Cycling
CDK5RAP22	0	0.72426247	0.605	0.425	0	Cycling
NUP1552	0	0.85356283	0.41	0.23	0	Cycling
NUP2052	0	0.4490929	0.613	0.434	0	Cycling
TMEM107	0	0.80093501	0.454	0.275	0	Cycling
KIF18B	0	8.32353355	0.18	0.001	0	Cycling
PFKP4	0	0.46420132	0.564	0.385	0	Cycling
NUP624	0	0.54697848	0.736	0.557	0	Cycling
HADH	0	0.69190063	0.777	0.599	0	Cycling
RFC21	0	0.60561233	0.655	0.477	0	Cycling
NDE14	0	0.81090719	0.543	0.365	0	Cycling
ALYREF2	0	0.63959487	0.586	0.408	0	Cycling
SUZ124	0	0.51977072	0.572	0.394	0	Cycling
TDP13	0	0.78125577	0.464	0.287	0	Cycling
NFIC3	0	0.74873112	0.798	0.621	0	Cycling
SLC16A15	0	0.9083556	0.394	0.217	0	Cycling

KATNAL12	0	1.26495164	0.276	0.099	0	Cycling
EXO52	0	0.82300178	0.379	0.202	0	Cycling
CCNE21	0	5.9492327	0.18	0.003	0	Cycling
HACD3	0	0.73317339	0.624	0.447	0	Cycling
TUBB64	0	0.65109066	0.692	0.515	0	Cycling
MLH12	0	0.49383648	0.651	0.475	0	Cycling
PARP17	0	0.68484832	0.431	0.255	0	Cycling
PIGX2	0	0.63862866	0.533	0.358	0	Cycling
DUT	0	0.96245095	0.745	0.57	0	Cycling
FAM122B4	0	0.66168158	0.525	0.35	0	Cycling
RAD51D2	0	0.91914246	0.36	0.185	0	Cycling
COMMD4	0	0.66621823	0.708	0.533	0	Cycling
PLK41	0	3.14867256	0.195	0.021	0	Cycling
YEATS44	0	0.84878574	0.384	0.21	0	Cycling
NME4	0	0.9155065	0.41	0.236	0	Cycling
CDK161	0	0.59725522	0.627	0.453	0	Cycling
TUBB4B1	0	1.06544218	0.736	0.562	0	Cycling
SNRPA2	0	0.45457673	0.688	0.514	0	Cycling
ANP32B1	0	0.67662673	0.77	0.597	0	Cycling
SUPT16H3	0	0.57170907	0.703	0.53	0	Cycling
HERC51	0	0.92453805	0.657	0.485	0	Cycling
BUB33	0	0.64782536	0.554	0.382	0	Cycling
CCL181	0	0.5451697	0.788	0.616	0	Cycling
CTPS13	0	0.91927727	0.345	0.173	0	Cycling
SPIN43	0	1.13812487	0.306	0.134	0	Cycling
BAZ1B1	0	0.48897786	0.727	0.555	0	Cycling
SSRP13	0	0.53693852	0.703	0.531	0	Cycling
SF3A21	0	0.52061814	0.707	0.535	0	Cycling
HDGF	0	0.52432034	0.643	0.472	0	Cycling
RNASE64	0	0.76001426	0.465	0.294	0	Cycling
SNRPB3	0	0.50838428	0.755	0.584	0	Cycling
HYI2	0	1.06909451	0.337	0.166	0	Cycling
EAF23	0	0.98342826	0.339	0.168	0	Cycling
RAC31	0	2.15779791	0.231	0.06	0	Cycling
RBBP81	0	1.18649291	0.318	0.147	0	Cycling
GLA2	0	0.55212268	0.786	0.616	0	Cycling
WDR621	0	3.48765322	0.188	0.018	0	Cycling
INTS72	0	0.57397867	0.438	0.268	0	Cycling
FBXO52	0	2.56816507	0.212	0.042	0	Cycling
DDB22	0	0.66623782	0.413	0.243	0	Cycling
RHNO13	0	0.67835672	0.477	0.307	0	Cycling
ATL3	0	0.52149418	0.689	0.519	0	Cycling
SLBP2	0	1.04490296	0.789	0.62	0	Cycling
CKAP51	0	0.79904304	0.654	0.485	0	Cycling
CEP852	0	0.79120755	0.417	0.248	0	Cycling
UNG1	0	1.14345913	0.367	0.198	0	Cycling
TP533	0	0.51444022	0.623	0.454	0	Cycling
HDHD52	0	0.73225163	0.627	0.458	0	Cycling
CHEK21	0	0.91217832	0.357	0.188	0	Cycling
RANGAP12	0	0.68794011	0.539	0.371	0	Cycling
CENPQ	0	1.43282128	0.257	0.089	0	Cycling
WRAP533	0	0.85740496	0.331	0.163	0	Cycling

AURKA1	0	2.59861721	0.225	0.057	0	Cycling
LIN91	0	2.01983666	0.217	0.049	0	Cycling
POLE21	0	3.84846114	0.178	0.01	0	Cycling
HMGXB43	0	0.62014778	0.445	0.277	0	Cycling
JADE12	0	1.21789574	0.274	0.107	0	Cycling
MYO1C1	0	0.48262662	0.756	0.589	0	Cycling
CCDC185	0	1.09058512	0.313	0.146	0	Cycling
FOXRED22	0	1.50865293	0.253	0.086	0	Cycling
CAD2	0	0.65817057	0.424	0.257	0	Cycling
TICRR1	0	5.23288808	0.17	0.005	0	Cycling
CENPO1	0	2.30403057	0.209	0.044	0	Cycling
CDKN3	0	4.94581146	0.173	0.008	0	Cycling
ANKRD9	0	1.20341523	0.285	0.12	0	Cycling
HAUS51	0	1.08987073	0.287	0.122	0	Cycling
POLD22	0	0.61726597	0.446	0.282	0	Cycling
DIAPH3	0	6.81422966	0.165	0.001	0	Cycling
NEK2	0	7.3036527	0.164	0.001	0	Cycling
E2F81	0	7.30862622	0.164	0.001	0	Cycling
EFCAB11	0	1.01086243	0.303	0.141	0	Cycling
CEP2502	0	0.9274153	0.314	0.152	0	Cycling
SIVA13	0	0.73673887	0.77	0.609	0	Cycling
CTDSPL1	0	1.12537253	0.304	0.144	0	Cycling
CDCA3	0	5.43853678	0.166	0.006	0	Cycling
HMCES2	0	0.60473746	0.766	0.607	0	Cycling
AGPAT54	0	0.74782055	0.342	0.183	0	Cycling
SCMH12	0	0.84114562	0.325	0.166	0	Cycling
RFC52	0	1.68605922	0.22	0.061	0	Cycling
PAQR41	0	1.55869282	0.235	0.076	0	Cycling
MEX3D3	0	1.09393305	0.296	0.137	0	Cycling
METTL7A4	0	1.21807611	0.398	0.24	0	Cycling
CHTF182	0	2.14595094	0.195	0.037	0	Cycling
SLFN131	0	4.02953725	0.165	0.007	0	Cycling
PRKDC1	0	0.50595374	0.774	0.617	0	Cycling
ASPM	0	7.12946484	0.159	0.002	0	Cycling
EMC91	0	1.50035135	0.294	0.137	0	Cycling
MTFP12	0	1.08957868	0.282	0.125	0	Cycling
SLF12	0	0.64924819	0.269	0.113	0	Cycling
CCHCR13	0	1.11684039	0.271	0.115	0	Cycling
HAUS12	0	0.80830182	0.335	0.179	0	Cycling
SLC7A16	0	0.80660441	0.292	0.136	0	Cycling
NUDT152	0	0.71599564	0.359	0.203	0	Cycling
CD993	0	0.41041413	0.887	0.732	0	Cycling
CCDC147	0	0.7061619	0.377	0.222	0	Cycling
CSE1L2	0	0.68455733	0.36	0.205	0	Cycling
CCP1102	0	0.98582443	0.358	0.204	0	Cycling
NUCKS12	0	0.86149587	0.824	0.67	0	Cycling
RHEB1	0	0.56123538	0.744	0.591	0	Cycling
DEPDC1	0	3.52713082	0.174	0.021	0	Cycling
CDC25A1	0	5.46247014	0.156	0.003	0	Cycling
TONSL	0	2.71185822	0.178	0.025	0	Cycling
CTNNAL13	0	2.17339856	0.194	0.041	0	Cycling
H1FX4	0	0.76556154	0.764	0.612	0	Cycling

TRIM591	0	1.78016131	0.194	0.042	0	Cycling
PBK	0	8.15964165	0.153	0.001	0	Cycling
PRIM23	0	0.90133188	0.29	0.138	0	Cycling
TUBB	0	1.64002051	0.874	0.723	0	Cycling
DSCC1	0	3.47235714	0.166	0.015	0	Cycling
GSTCD1	0	0.8393354	0.305	0.154	0	Cycling
PARP21	0	1.33490691	0.243	0.092	0	Cycling
TRIOBP2	0	0.56004038	0.824	0.673	0	Cycling
RAD182	0	1.16993416	0.244	0.094	0	Cycling
C4orf461	0	2.20794038	0.187	0.037	0	Cycling
TUBA1C1	0	0.76163359	0.696	0.546	0	Cycling
CRAT2	0	1.21547361	0.299	0.151	0	Cycling
VANGL11	0	1.5165619	0.236	0.089	0	Cycling
POLH2	0	0.97005634	0.304	0.157	0	Cycling
CNTROB3	0	0.90171428	0.364	0.217	0	Cycling
CIP2A1	0	2.70990886	0.171	0.025	0	Cycling
HIST1H1E3	0	1.2932554	0.424	0.278	0	Cycling
RAD54B2	0	2.73146427	0.167	0.022	0	Cycling
PDXP1	0	0.89070756	0.297	0.153	0	Cycling
MIS18BP12	0	0.53749953	0.807	0.665	0	Cycling
CEP1522	0	1.37898185	0.225	0.083	0	Cycling
SASS61	0	1.46839276	0.206	0.064	0	Cycling
TIFA3	0	0.8557338	0.29	0.148	0	Cycling
HMGN12	0	0.44670047	0.831	0.69	0	Cycling
NEIL32	0	4.97560049	0.145	0.004	0	Cycling
PSMC3	0	0.54277958	0.808	0.668	0	Cycling
FANCL3	0	0.78064073	0.29	0.15	0	Cycling
LRRCC12	0	0.95222877	0.282	0.142	0	Cycling
PHF134	0	0.83195404	0.32	0.181	0	Cycling
SUV39H21	0	1.87025285	0.185	0.046	0	Cycling
CLN6	0	0.63013588	0.841	0.702	0	Cycling
BLM1	0	2.54929367	0.164	0.025	0	Cycling
SLC43A32	0	0.42878037	0.802	0.666	0	Cycling
ZMYND193	0	0.77660909	0.286	0.15	0	Cycling
SLC1A53	0	0.46270122	0.808	0.673	0	Cycling
ALDH7A11	0	1.31525989	0.227	0.093	0	Cycling
LRRC201	0	1.212585	0.24	0.106	0	Cycling
CALM31	0	0.78856066	0.829	0.695	0	Cycling
PTPA1	0	0.59507221	0.818	0.684	0	Cycling
NUDT212	0	0.4510023	0.821	0.687	0	Cycling
CD1802	0	1.66266376	0.208	0.075	0	Cycling
ATP8B32	0	2.113959	0.197	0.064	0	Cycling
LYPD51	0	1.08394067	0.255	0.122	0	Cycling
TMEM2011	0	1.09954101	0.243	0.111	0	Cycling
CDKN2A1	0	1.52804245	0.208	0.076	0	Cycling
DHX154	0	0.35931028	0.852	0.721	0	Cycling
C17orf53	0	3.57479344	0.142	0.011	0	Cycling
KHK2	0	0.82921115	0.25	0.121	0	Cycling
SLC39A83	0	0.28936456	0.227	0.098	0	Cycling
EME11	0	3.94545166	0.137	0.008	0	Cycling
ANAPC111	0	0.47728415	0.83	0.701	0	Cycling
C18orf541	0	1.77102534	0.173	0.044	0	Cycling

HNRNPD4	0	0.35782917	0.836	0.708	0	Cycling
ERCC6L1	0	5.51055871	0.128	0.001	0	Cycling
HMGB31	0	2.8100461	0.152	0.025	0	Cycling
SMC1A2	0	0.85319508	0.879	0.753	0	Cycling
AC091057.63	0	2.05724671	0.171	0.046	0	Cycling
RNASEH2A1	0	1.71136355	0.17	0.045	0	Cycling
ACYP12	0	0.92083608	0.228	0.105	0	Cycling
CBR32	0	3.13273557	0.14	0.017	0	Cycling
CDCA42	0	1.56669468	0.169	0.047	0	Cycling
CCNE1	0	2.55765868	0.153	0.031	0	Cycling
SPATA333	0	0.89050084	0.23	0.109	0	Cycling
TRAIP	0	2.7049953	0.138	0.019	0	Cycling
FAAP241	0	1.0331632	0.212	0.093	0	Cycling
B4GALT21	0	1.07891576	0.21	0.092	0	Cycling
C51	0	1.3751223	0.182	0.064	0	Cycling
FANCB1	0	2.31955074	0.141	0.023	0	Cycling
TMEM237	0	1.32494835	0.187	0.07	0	Cycling
MS4A6A4	0	0.50379171	0.929	0.812	0	Cycling
EIF4A32	0	0.50642842	0.875	0.758	0	Cycling
CDC71	0	2.20543404	0.134	0.018	0	Cycling
TIPIN1	0	1.56452978	0.163	0.047	0	Cycling
SLC25A101	0	1.71155688	0.161	0.045	0	Cycling
PPM1G1	0	0.46459427	0.868	0.752	0	Cycling
ARHGEF39	0	5.22285117	0.12	0.004	0	Cycling
ITPR32	0	0.56811077	0.171	0.056	0	Cycling
FAM83D1	0	5.02225955	0.117	0.002	0	Cycling
DMC1	0	2.98244672	0.13	0.015	0	Cycling
PASK3	0	1.16929635	0.173	0.058	0	Cycling
FANCE1	0	0.81201444	0.207	0.092	0	Cycling
TMEM1093	0	0.48646851	0.859	0.745	0	Cycling
CCDC155	0	1.39716439	0.171	0.058	0	Cycling
E2F72	0	5.92628946	0.113	0.001	0	Cycling
EZR4	0	0.40839195	0.932	0.821	0	Cycling
PSRC11	0	3.05880054	0.133	0.022	0	Cycling
MYBL14	0	1.26552267	0.12	0.01	0	Cycling
TMEM714	0	2.2539108	0.125	0.015	0	Cycling
CABLES23	0	1.02360405	0.191	0.081	0	Cycling
CEP721	0	1.09376144	0.171	0.062	0	Cycling
KIF18A1	0	3.84602586	0.117	0.008	0	Cycling
CDC25C1	0	7.18486017	0.109	0	0	Cycling
DBF4B1	0	1.32158286	0.173	0.064	0	Cycling
TUBA1B	0	0.77663298	0.865	0.757	0	Cycling
CEP973	0	1.09259838	0.178	0.071	0	Cycling
MNS11	0	2.6441267	0.124	0.017	0	Cycling
KLK12	0	1.62952059	0.155	0.048	0	Cycling
FANCM3	0	1.15392605	0.159	0.053	0	Cycling
HYLS12	0	1.50131774	0.159	0.054	0	Cycling
KDELR3	0	1.33085552	0.167	0.062	0	Cycling
FN11	0	0.56439649	0.898	0.793	0	Cycling
TEDC2	0	3.38893763	0.114	0.01	0	Cycling
GPC12	0	1.51503743	0.153	0.049	0	Cycling
ZNF854	0	1.11562724	0.167	0.064	0	Cycling

PIMREG	0	7.40012265	0.104	0.001	0	Cycling
KANK22	0	4.77039612	0.104	0.004	0	Cycling
LSM4	0	0.63277694	0.887	0.787	0	Cycling
AP2S12	0	0.43591876	0.909	0.809	0	Cycling
SPTBN14	0	2.36275706	0.113	0.014	0	Cycling
PKN32	0	3.59254074	0.107	0.008	0	Cycling
C21orf582	0	2.64415545	0.113	0.014	0	Cycling
YWHAH3	0	0.39752213	0.931	0.833	0	Cycling
ST3GAL46	0	0.91078628	0.17	0.072	0	Cycling
ZMYND101	0	2.2828456	0.112	0.014	0	Cycling
HIST1H2BB2	0	3.81307449	0.106	0.009	0	Cycling
ZNF7242	0	1.25461583	0.15	0.053	0	Cycling
MTBP3	0	1.58403587	0.131	0.035	0	Cycling
SMTN1	0	2.25401843	0.123	0.027	0	Cycling
KIF241	0	2.90949834	0.108	0.013	0	Cycling
CBFB2	0	0.37172035	0.887	0.792	0	Cycling
CYBRD15	0	2.62987278	0.111	0.017	0	Cycling
REXO5	0	3.22433627	0.105	0.011	0	Cycling
MCMBP1	0	0.39791743	0.88	0.786	0	Cycling
SAPCD21	0	5.1731464	0.094	0.002	0	Cycling
PHLPP13	0	1.11694156	0.145	0.053	0	Cycling
HNRNPUL13	0	0.34242228	0.917	0.826	0	Cycling
XRCC21	0	3.66470471	0.097	0.006	0	Cycling
CHRNA51	0	1.44791577	0.129	0.039	0	Cycling
NUDT82	0	1.40625002	0.133	0.044	0	Cycling
AZU1	0	1.91658426	0.134	0.045	0	Cycling
CEP703	0	1.6420374	0.142	0.053	0	Cycling
NAV22	0	4.90995536	0.09	0.003	0	Cycling
PMAIP15	0	0.92838583	0.121	0.034	0	Cycling
GUSB	0	0.46382569	0.963	0.88	0	Cycling
YPEL12	0	1.98825182	0.094	0.012	0	Cycling
EPS84	0	1.38298079	0.124	0.043	0	Cycling
ZNF5192	0	1.24976241	0.128	0.047	0	Cycling
HNRNPF3	0	0.51581479	0.938	0.857	0	Cycling
APOBEC3B	0	7.45944651	0.08	0	0	Cycling
OXCT14	0	1.45262569	0.088	0.01	0	Cycling
WDR902	0	1.49552127	0.107	0.03	0	Cycling
ENO12	0	0.35765986	0.955	0.88	0	Cycling
PIDD12	0	1.11747066	0.114	0.04	0	Cycling
RNASE22	0	2.57637092	0.089	0.019	0	Cycling
AUNIP	0	6.21631809	0.07	0.001	0	Cycling
SPDL11	0	2.10555982	0.086	0.017	0	Cycling
HIST1H2AE2	0	2.12181211	0.08	0.014	0	Cycling
ARHGAP331	0	2.17500732	0.079	0.014	0	Cycling
SLC12A82	0	2.27730803	0.08	0.016	0	Cycling
LPL1	0	0.3691935	0.975	0.911	0	Cycling
C1orf215	0	2.4872007	0.069	0.006	0	Cycling
FBXO431	0	6.73592518	0.064	0.001	0	Cycling
SKA21	0	1.96698803	0.079	0.016	0	Cycling
TESMIN2	0	2.14867744	0.078	0.016	0	Cycling
C19orf572	0	2.66687712	0.068	0.008	0	Cycling
NEURL1B1	0	5.84858158	0.061	0.001	0	Cycling

HIST1H2BN	0	3.29752221	0.059	0.005	0	Cycling
HNRNPA2B14	0	0.29050612	0.97	0.916	0	Cycling
SUMO3	0	0.30272717	0.963	0.909	0	Cycling
HIST1H1A	0	3.54570145	0.058	0.005	0	Cycling
PIF11	0	3.35241262	0.059	0.007	0	Cycling
LMO73	0	1.36778385	0.059	0.011	0	Cycling
RIBC21	0	3.80582404	0.049	0.001	0	Cycling
CTSV	0	3.17757784	0.052	0.005	0	Cycling
FAM161A3	0	1.86537053	0.057	0.011	0	Cycling
HES41	0	1.20657314	0.056	0.012	0	Cycling
CCDC150	0	3.3119409	0.047	0.003	0	Cycling
C5orf342	0	2.15104274	0.054	0.01	0	Cycling
ACADVL	0	0.24449567	0.987	0.944	0	Cycling
CR1L2	0	2.09145803	0.051	0.01	0	Cycling
TP731	0	3.27618477	0.041	0.001	0	Cycling
STRIP22	0	2.66365279	0.041	0.003	0	Cycling
NNAT	0	3.17674357	0.042	0.006	0	Cycling
RDM1	0	5.06732092	0.035	0	0	Cycling
SYCE2	0	5.32901395	0.035	0	0	Cycling
CDCA71	0	2.24585308	0.036	0.001	0	Cycling
DERL32	0	2.54113919	0.035	0.002	0	Cycling
ARHGAP11B	0	2.47097242	0.037	0.005	0	Cycling
ITPKA	0	2.82408477	0.035	0.004	0	Cycling
C3orf671	0	2.22501945	0.036	0.005	0	Cycling
MARCO2	0	0.23007578	0.996	0.966	0	Cycling
C1QL1	0	5.9766066	0.03	0	0	Cycling
RNF144A3	0	1.50496528	0.032	0.003	0	Cycling
ZNF695	0	4.32695943	0.03	0.001	0	Cycling
GPR137C	0	2.73575825	0.032	0.004	0	Cycling
C8orf88	0	3.66869821	0.03	0.002	0	Cycling
FAM184A2	0	1.52899583	0.029	0.002	0	Cycling
GPR19	0	3.10190316	0.025	0.001	0	Cycling
RTKN22	0	0.64556587	0.023	0.001	0	Cycling
CDH24	0	2.95555986	0.019	0.001	0	Cycling
C1QTNF2	0	6.88103304	0.017	0	0	Cycling
GJC1	0	5.27064349	0.017	0	0	Cycling
CSPG5	0	3.80316524	0.018	0.001	0	Cycling
CRIP11	0	0.25563178	0.999	0.987	0	Cycling
TLCD3B	0	5.48182197	0.012	0	0	Cycling
PDS5B3	1.092E-306	0.6537389	0.406	0.25	1.678E-302	Cycling
ZFP912	4.992E-306	0.41837658	0.833	0.71	7.673E-302	Cycling
TUBGCP32	5.376E-306	0.53186556	0.577	0.405	8.263E-302	Cycling
MEGF65	1.026E-304	0.70383883	0.105	0.035	1.577E-300	Cycling
MAP3K63	1.455E-304	0.95411274	0.202	0.095	2.236E-300	Cycling
HSPA21	1.707E-304	1.489467	0.103	0.035	2.623E-300	Cycling
PLCXD14	2.181E-304	0.6142406	0.331	0.186	3.352E-300	Cycling
CENPB	2.814E-303	0.4721067	0.747	0.603	4.325E-299	Cycling
DBF42	7.302E-303	0.82938603	0.237	0.119	1.122E-298	Cycling
PI4K2B1	1.657E-302	0.43750107	0.721	0.567	2.548E-298	Cycling
KLHL222	1.829E-302	0.68775481	0.348	0.203	2.812E-298	Cycling
B3GNT92	2.066E-301	1.18223528	0.06	0.014	3.176E-297	Cycling
HAUS62	1.469E-300	0.61251286	0.341	0.195	2.258E-296	Cycling

SLC35B24	1.093E-298	0.51771519	0.535	0.363	1.68E-294	Cycling
RAD13	1.336E-298	0.5867002	0.39	0.234	2.054E-294	Cycling
RPA23	1.9E-298	0.57584191	0.468	0.303	2.921E-294	Cycling
RNFT2	2.264E-298	1.16269167	0.162	0.07	3.481E-294	Cycling
NREP5	8.63E-298	0.80087581	0.233	0.116	1.327E-293	Cycling
ODF23	1.613E-297	0.74493729	0.314	0.177	2.479E-293	Cycling
ACAP33	2.688E-295	0.55842663	0.431	0.271	4.131E-291	Cycling
SKP22	5.047E-295	0.83567405	0.238	0.12	7.758E-291	Cycling
S100PBP3	9.82E-295	0.46106958	0.502	0.335	1.509E-290	Cycling
NT5M	1.12E-294	2.01239187	0.028	0.003	1.721E-290	Cycling
POLR2D2	1.086E-292	0.59254977	0.463	0.302	1.669E-288	Cycling
HAUS2	6.313E-292	0.45561381	0.761	0.619	9.703E-288	Cycling
LMF22	7.248E-292	0.50275935	0.634	0.473	1.114E-287	Cycling
NUP853	3.866E-289	0.43608141	0.63	0.462	5.943E-285	Cycling
TMEM1601	7.418E-289	0.45185966	0.657	0.492	1.14E-284	Cycling
SLC44A14	1.705E-288	0.60002025	0.425	0.268	2.621E-284	Cycling
FBXO47	2.504E-288	0.68278977	0.288	0.156	3.849E-284	Cycling
FPR33	6.62E-288	0.30823579	0.663	0.478	1.018E-283	Cycling
HAUS82	1.033E-287	0.78754934	0.213	0.103	1.587E-283	Cycling
MAST22	4.404E-287	0.61153569	0.512	0.35	6.769E-283	Cycling
ASPH3	7.394E-287	0.49428478	0.635	0.464	1.136E-282	Cycling
GAS66	1.754E-286	1.14337895	0.052	0.011	2.695E-282	Cycling
CEP573	8.111E-286	0.50384842	0.502	0.338	1.247E-281	Cycling
LGMN2	6.184E-285	0.37859918	0.669	0.499	9.506E-281	Cycling
RALY2	1.345E-284	0.442344	0.72	0.567	2.068E-280	Cycling
HDGFL31	2.826E-284	0.93178835	0.226	0.116	4.343E-280	Cycling
IQCC1	3.242E-284	1.51936846	0.078	0.023	4.984E-280	Cycling
HNRNPA03	4.146E-284	0.25542499	0.96	0.91	6.372E-280	Cycling
KLRG11	5.72E-284	0.33592456	0.048	0.01	8.792E-280	Cycling
PCGF22	6.714E-284	0.92468956	0.185	0.086	1.032E-279	Cycling
PIEZO13	1.444E-283	0.40235084	0.651	0.483	2.22E-279	Cycling
PPP5C1	1.836E-283	0.58235956	0.374	0.224	2.822E-279	Cycling
RHEBL12	7.74E-283	1.35569611	0.082	0.025	1.19E-278	Cycling
CNP	2.537E-282	0.39233326	0.788	0.639	3.899E-278	Cycling
ZNF902	2.948E-282	1.26071044	0.11	0.04	4.531E-278	Cycling
NNT2	4.433E-282	0.49113305	0.614	0.446	6.814E-278	Cycling
USP131	1.446E-281	1.02726273	0.185	0.087	2.222E-277	Cycling
ARL6IP62	7.017E-281	0.52208737	0.45	0.288	1.079E-276	Cycling
IFT1221	3.026E-280	0.83441343	0.326	0.193	4.651E-276	Cycling
HEG11	6.138E-280	0.61733385	0.329	0.19	9.435E-276	Cycling
CDKN2D3	7.143E-280	1.24227336	0.458	0.321	1.098E-275	Cycling
TXNDC5	3.232E-279	0.46120542	0.743	0.612	4.967E-275	Cycling
PEA153	4.335E-279	0.30389475	0.858	0.743	6.663E-275	Cycling
NUP1072	6.035E-279	0.43889665	0.628	0.454	9.276E-275	Cycling
TPRKB3	5.521E-278	0.52875408	0.499	0.333	8.486E-274	Cycling
ABHD17C2	1.69E-277	1.04470688	0.127	0.05	2.597E-273	Cycling
TMSB15A	2.393E-277	5.99169719	0.011	0	3.678E-273	Cycling
SNCA4	3.321E-277	1.30847877	0.154	0.067	5.105E-273	Cycling
NUP432	9.069E-277	0.42084093	0.602	0.433	1.394E-272	Cycling
SMPD43	1.231E-276	0.47342125	0.548	0.378	1.892E-272	Cycling
RBM152	6.707E-276	0.48942264	0.563	0.4	1.031E-271	Cycling
HNRNPA34	4.071E-275	0.36495877	0.794	0.651	6.257E-271	Cycling

ATRIP1	4.241E-275	0.77300755	0.213	0.105	6.519E-271	Cycling
BORA3	4.592E-275	1.04141006	0.219	0.112	7.058E-271	Cycling
ZNF8502	4.894E-275	1.10711102	0.136	0.056	7.523E-271	Cycling
SPAG12	1.584E-273	0.93386583	0.169	0.076	2.434E-269	Cycling
CTDSPL21	7.717E-273	0.55278359	0.411	0.259	1.186E-268	Cycling
RELL14	3.186E-271	0.44978362	0.474	0.311	4.898E-267	Cycling
PALLD4	1.184E-270	1.19115076	0.146	0.063	1.82E-266	Cycling
STT3A1	4.39E-270	0.34861312	0.828	0.707	6.748E-266	Cycling
COPS31	1.402E-269	0.45098236	0.618	0.451	2.155E-265	Cycling
STK265	2.165E-269	0.38377952	0.102	0.036	3.328E-265	Cycling
PRSS363	3.806E-269	0.66733258	0.289	0.161	5.85E-265	Cycling
CTSG1	4.373E-269	1.377123	0.09	0.03	6.722E-265	Cycling
MDK1	1.138E-268	0.69645291	0.341	0.204	1.749E-264	Cycling
CREB3L43	5.129E-268	1.15179839	0.122	0.048	7.884E-264	Cycling
CFAP202	9.43E-268	0.56565314	0.404	0.252	1.449E-263	Cycling
UBE2A1	4.522E-267	0.32833004	0.875	0.774	6.951E-263	Cycling
HIST1H4J1	6.805E-267	1.85996899	0.059	0.015	1.046E-262	Cycling
UCK25	5.912E-266	0.95326236	0.08	0.025	9.087E-262	Cycling
IGFBP75	1.299E-265	1.15345623	0.093	0.032	1.996E-261	Cycling
LRP33	5.127E-265	1.14883321	0.117	0.045	7.88E-261	Cycling
EXOSC91	2.328E-264	0.43135291	0.589	0.428	3.578E-260	Cycling
MTHFD23	7.784E-264	0.44034845	0.691	0.538	1.196E-259	Cycling
ZNF2193	3.835E-263	0.69828081	0.273	0.15	5.895E-259	Cycling
CCDC823	1.025E-262	0.45357223	0.587	0.421	1.576E-258	Cycling
ACOT71	2.112E-262	0.40278272	0.734	0.585	3.246E-258	Cycling
LLGL11	3.721E-262	0.49375257	0.525	0.363	5.72E-258	Cycling
GPN3	4.374E-262	0.48077226	0.6	0.436	6.724E-258	Cycling
BTBD61	1.758E-261	0.43977795	0.564	0.399	2.702E-257	Cycling
PHF102	7.7E-261	0.40766793	0.585	0.427	1.183E-256	Cycling
FAM222A2	5.676E-260	1.72725815	0.05	0.011	8.724E-256	Cycling
UQCC22	9.974E-260	0.45591481	0.629	0.469	1.533E-255	Cycling
KHSRP	1.98E-259	0.39659775	0.707	0.549	3.044E-255	Cycling
VKORC1	3.623E-259	0.30185181	0.896	0.805	5.568E-255	Cycling
ACTN41	7.793E-259	0.26251882	0.955	0.886	1.198E-254	Cycling
IFI165	1.028E-258	0.30077707	0.916	0.82	1.581E-254	Cycling
RDX2	1.202E-258	0.56366283	0.474	0.318	1.847E-254	Cycling
OAS31	2.541E-258	0.27553057	0.92	0.824	3.906E-254	Cycling
ALDH16A11	7.748E-258	0.42147088	0.659	0.497	1.191E-253	Cycling
RAD51C3	1.836E-257	0.75708529	0.256	0.139	2.823E-253	Cycling
DCAF152	7.414E-256	0.40690191	0.578	0.41	1.14E-251	Cycling
CCDC1671	1.246E-255	0.45776753	0.28	0.156	1.915E-251	Cycling
LNPK1	1.579E-255	0.42058426	0.671	0.519	2.427E-251	Cycling
CEP2954	1.38E-253	0.80703323	0.208	0.105	2.12E-249	Cycling
ITGA62	6.529E-253	0.7744225	0.218	0.114	1.004E-248	Cycling
YWHAQ1	1.568E-252	0.35931963	0.729	0.579	2.41E-248	Cycling
CLGN1	2.051E-252	3.31373587	0.016	0.001	3.152E-248	Cycling
CYB5B1	4.561E-252	0.28949253	0.877	0.772	7.011E-248	Cycling
CMPK21	2.238E-251	0.35510653	0.704	0.538	3.439E-247	Cycling
SOGA1	3.253E-251	0.54820462	0.652	0.504	5E-247	Cycling
TTLL53	1.308E-250	0.47174947	0.542	0.385	2.011E-246	Cycling
MYB1	5.588E-250	0.45044666	0.493	0.331	8.59E-246	Cycling
TCN21	6.051E-250	0.35670733	0.793	0.647	9.301E-246	Cycling

PGP	6.487E-250	0.35210867	0.818	0.706	9.972E-246	Cycling
SPINDOC1	3.157E-249	0.49602869	0.461	0.305	4.853E-245	Cycling
TTC39C1	4.752E-249	0.1798828	0.567	0.404	7.305E-245	Cycling
PHTF13	1.124E-248	0.48617134	0.413	0.264	1.728E-244	Cycling
FUS3	2.465E-248	0.42496819	0.693	0.547	3.788E-244	Cycling
SESTD11	2.729E-248	0.5060118	0.515	0.357	4.195E-244	Cycling
GBA3	4.908E-247	0.32472629	0.855	0.739	7.543E-243	Cycling
SEC22C2	1.465E-246	0.39050521	0.744	0.606	2.251E-242	Cycling
VDAC32	1.496E-246	0.44437038	0.67	0.521	2.3E-242	Cycling
TOR3A1	2.016E-246	0.46614988	0.592	0.432	3.099E-242	Cycling
TBC1D312	2.266E-245	0.62242517	0.339	0.207	3.484E-241	Cycling
GRN2	6.778E-245	0.15316579	0.998	0.979	1.042E-240	Cycling
SLC20A15	1.01E-244	0.59121295	0.562	0.422	1.552E-240	Cycling
LBR5	1.045E-244	0.51647937	0.48	0.339	1.606E-240	Cycling
B3GNTL12	4.696E-244	0.76162907	0.208	0.107	7.219E-240	Cycling
SPTSSA3	5.703E-244	0.3964579	0.729	0.584	8.766E-240	Cycling
PPP2R5D1	7.034E-244	0.47938685	0.603	0.451	1.081E-239	Cycling
THAP10	2.257E-243	0.86339686	0.189	0.093	3.47E-239	Cycling
APOLD1	3.597E-243	3.44578347	0.014	0.001	5.529E-239	Cycling
PTGR11	1.261E-242	0.48270693	0.582	0.421	1.939E-238	Cycling
CEP1924	1.705E-242	0.54499213	0.434	0.287	2.621E-238	Cycling
GOLM11	6.058E-242	0.65466696	0.396	0.257	9.311E-238	Cycling
MAP4K12	7.844E-242	0.29922596	0.422	0.274	1.206E-237	Cycling
KHDRBS11	3.375E-241	0.40147773	0.729	0.584	5.188E-237	Cycling
CTSZ3	4.582E-241	0.16685254	0.996	0.972	7.044E-237	Cycling
PELP12	7.653E-241	0.42060382	0.562	0.398	1.176E-236	Cycling
CALU1	8.882E-241	0.33127164	0.84	0.726	1.365E-236	Cycling
FARP13	1.666E-240	0.69071431	0.441	0.305	2.562E-236	Cycling
MTHFD1	3.204E-240	0.34225089	0.844	0.74	4.925E-236	Cycling
RNF261	9.924E-240	0.6222523	0.541	0.39	1.525E-235	Cycling
LRRC8A2	9.934E-240	0.42726487	0.448	0.296	1.527E-235	Cycling
LIN521	2.821E-239	0.64332233	0.218	0.114	4.336E-235	Cycling
AGPAT31	7.956E-238	0.2839153	0.889	0.782	1.223E-233	Cycling
ERO1A2	6.162E-236	0.25465328	0.912	0.821	9.472E-232	Cycling
TIMM10	1.647E-235	0.36168615	0.797	0.679	2.531E-231	Cycling
MCUR11	2.917E-235	0.46959621	0.504	0.351	4.483E-231	Cycling
ARHGEF161	6.535E-234	1.80979314	0.048	0.011	1.004E-229	Cycling
MAP3K34	6.945E-234	0.3653984	0.42	0.273	1.067E-229	Cycling
C11orf802	1.578E-233	0.590989	0.182	0.09	2.426E-229	Cycling
PLEKHG33	2.578E-233	0.45318158	0.484	0.331	3.962E-229	Cycling
AKR1C22	2.858E-233	0.74312286	0.278	0.162	4.393E-229	Cycling
ETFB	3.696E-233	0.3345811	0.793	0.661	5.682E-229	Cycling
MYO1G1	4.655E-233	0.25123677	0.968	0.896	7.155E-229	Cycling
CDR22	8.004E-233	0.42842841	0.356	0.222	1.23E-228	Cycling
ILF34	1.761E-232	0.26053685	0.858	0.751	2.707E-228	Cycling
MRE111	2.477E-232	0.51467458	0.348	0.213	3.807E-228	Cycling
GSDME3	5.476E-232	1.08579201	0.106	0.042	8.417E-228	Cycling
CHAMP13	9.324E-232	0.49432397	0.449	0.302	1.433E-227	Cycling
PKP43	1.027E-231	1.4286431	0.066	0.02	1.578E-227	Cycling
TPR1	1.306E-231	0.37394243	0.69	0.543	2.007E-227	Cycling
RFC12	2.798E-231	0.48463265	0.388	0.249	4.301E-227	Cycling
RAB27A2	1.993E-230	0.25214523	0.639	0.493	3.064E-226	Cycling

XPO15	2.342E-230	0.42429409	0.535	0.376	3.599E-226	Cycling
GMPPB3	3.064E-230	0.40902566	0.61	0.458	4.709E-226	Cycling
TMEM183	4.763E-230	0.51538574	0.414	0.27	7.321E-226	Cycling
PLEK23	7.39E-230	1.37880894	0.066	0.02	1.136E-225	Cycling
SRD5A31	7.262E-229	0.44924882	0.657	0.514	1.116E-224	Cycling
GUCD1	8.461E-229	0.33911562	0.808	0.69	1.301E-224	Cycling
NECTIN11	1.056E-228	0.52237894	0.465	0.315	1.624E-224	Cycling
STAG14	8.4E-228	0.35224018	0.622	0.47	1.291E-223	Cycling
RB15	2.749E-227	0.42502768	0.575	0.42	4.225E-223	Cycling
GPRIN11	3.024E-227	0.98471594	0.102	0.04	4.648E-223	Cycling
OTULINL3	3.414E-227	0.3491518	0.727	0.571	5.248E-223	Cycling
RECQL2	6.005E-227	0.28620844	0.822	0.688	9.23E-223	Cycling
MSH52	9.168E-227	0.64940811	0.234	0.128	1.409E-222	Cycling
METTL41	5.403E-226	0.63044038	0.27	0.157	8.305E-222	Cycling
PEAK16	6.985E-226	0.53569937	0.335	0.205	1.074E-221	Cycling
NEDD41	8.744E-226	0.9759686	0.11	0.045	1.344E-221	Cycling
PM20D23	1.258E-225	0.65110241	0.14	0.063	1.934E-221	Cycling
XRCC12	4.112E-225	0.42961296	0.561	0.405	6.321E-221	Cycling
AP1M11	1.064E-224	0.28611889	0.868	0.762	1.636E-220	Cycling
PRDX4	4.287E-224	0.36375696	0.762	0.629	6.589E-220	Cycling
SNTA11	9.271E-224	0.58130429	0.293	0.174	1.425E-219	Cycling
TBC1D10B1	1.043E-223	0.3714322	0.705	0.557	1.603E-219	Cycling
MARCH35	1.232E-223	1.15502976	0.081	0.028	1.894E-219	Cycling
IMPDH23	3.83E-223	0.44809143	0.439	0.29	5.887E-219	Cycling
KIF2A2	4.354E-223	0.32672378	0.452	0.305	6.692E-219	Cycling
ABCG21	9.851E-223	0.48779867	0.605	0.455	1.514E-218	Cycling
LIN7A1	5.624E-222	0.63185013	0.412	0.278	8.645E-218	Cycling
FOXRED12	2.494E-221	0.50178545	0.388	0.249	3.834E-217	Cycling
MANEA4	9.797E-221	0.47824099	0.435	0.293	1.506E-216	Cycling
ACAT1	1.216E-220	0.29880224	0.865	0.76	1.87E-216	Cycling
PPIH2	1.243E-220	0.56482103	0.263	0.149	1.91E-216	Cycling
ELP62	5.447E-220	0.49022599	0.447	0.302	8.372E-216	Cycling
PAK16	1.681E-219	0.43151281	0.565	0.416	2.583E-215	Cycling
SWAP704	4.537E-219	0.34161129	0.742	0.591	6.974E-215	Cycling
FZD13	1.314E-218	0.95479859	0.127	0.056	2.02E-214	Cycling
RAB23	1.833E-218	1.06799144	0.118	0.05	2.817E-214	Cycling
TSPAN334	2.1E-218	0.62626077	0.133	0.059	3.227E-214	Cycling
ADCK21	3.752E-218	0.39085603	0.677	0.54	5.767E-214	Cycling
ADGRA21	1.332E-217	0.86867018	0.189	0.097	2.048E-213	Cycling
IFT802	1.287E-216	0.61633943	0.182	0.093	1.978E-212	Cycling
PAXIP11	1.38E-216	0.64417697	0.228	0.125	2.122E-212	Cycling
PRPSAP11	2.495E-216	0.50395057	0.485	0.341	3.835E-212	Cycling
NOP563	4.929E-216	0.38524332	0.66	0.521	7.576E-212	Cycling
YIF1B2	6.045E-216	0.36556323	0.662	0.507	9.292E-212	Cycling
CAMK13	6.905E-216	0.69230953	0.208	0.111	1.061E-211	Cycling
KITLG1	1.114E-215	3.42973848	0.015	0.001	1.712E-211	Cycling
HINT21	1.483E-215	0.4266092	0.594	0.442	2.28E-211	Cycling
FBF14	1.679E-215	0.60493056	0.228	0.125	2.582E-211	Cycling
KLF105	4.188E-215	0.40899382	0.628	0.474	6.438E-211	Cycling
ILVBL	1.964E-214	0.38806247	0.712	0.574	3.018E-210	Cycling
PMVK1	7.596E-214	0.33666399	0.695	0.546	1.168E-209	Cycling
SELENON1	8.724E-214	0.28627643	0.921	0.844	1.341E-209	Cycling

TACC13	1.271E-213	0.23648938	0.908	0.827	1.954E-209	Cycling
USP373	2.075E-213	0.51681868	0.278	0.163	3.19E-209	Cycling
PRIMPOL1	4.512E-213	0.78010589	0.166	0.081	6.935E-209	Cycling
NASP1	8.921E-213	1.76034476	0.039	0.009	1.371E-208	Cycling
DARS21	9.435E-213	0.48580771	0.466	0.32	1.45E-208	Cycling
DERA	1.531E-212	0.40169662	0.664	0.522	2.353E-208	Cycling
NONO1	2.653E-212	0.40753925	0.642	0.493	4.077E-208	Cycling
GPAA1	3.253E-212	0.30911886	0.812	0.691	4.999E-208	Cycling
CLEC11A2	4.201E-212	1.36498958	0.096	0.038	6.457E-208	Cycling
ARNTL26	1.427E-211	1.06497877	0.088	0.033	2.193E-207	Cycling
BCAT14	3.661E-211	0.61776461	0.261	0.151	5.627E-207	Cycling
SFI13	3.9E-211	0.2766664	0.33	0.203	5.995E-207	Cycling
NUDT53	9.687E-211	0.33339182	0.645	0.493	1.489E-206	Cycling
FAM171A2	1.71E-210	2.59806394	0.02	0.002	2.628E-206	Cycling
SEPHS14	7.732E-210	0.58031561	0.314	0.194	1.188E-205	Cycling
CCDC771	3.456E-209	0.85795095	0.139	0.064	5.312E-205	Cycling
MS4A4E5	3.9E-209	1.23939111	0.076	0.027	5.995E-205	Cycling
AKIP1	5.731E-209	0.38818361	0.649	0.497	8.809E-205	Cycling
CLPP2	6.566E-209	0.33050727	0.678	0.531	1.009E-204	Cycling
HIST1H2AK	1.773E-208	2.49853223	0.017	0.002	2.725E-204	Cycling
BMP8B1	2.764E-208	1.43711164	0.053	0.015	4.249E-204	Cycling
TELO21	4.397E-208	0.37741762	0.514	0.362	6.759E-204	Cycling
SRSF21	4.748E-208	0.28460466	0.824	0.71	7.299E-204	Cycling
COLEC121	6.14E-208	0.30885923	0.905	0.824	9.438E-204	Cycling
PPP1CC2	8.983E-208	0.43478829	0.62	0.476	1.381E-203	Cycling
USP314	1.139E-207	0.67246725	0.194	0.101	1.751E-203	Cycling
RAB324	3.692E-207	0.39227704	0.652	0.503	5.675E-203	Cycling
POLR3K1	6.981E-207	0.37645865	0.62	0.473	1.073E-202	Cycling
KPNB12	4.577E-206	0.37083968	0.667	0.522	7.035E-202	Cycling
BUD133	6.482E-206	0.41654704	0.467	0.321	9.964E-202	Cycling
ILF22	1.456E-205	0.42452121	0.614	0.471	2.238E-201	Cycling
DCTPP11	1.617E-205	0.42264857	0.627	0.486	2.485E-201	Cycling
KIAA18413	3.738E-205	0.46528088	0.412	0.274	5.745E-201	Cycling
VBP12	4.584E-205	0.38831542	0.57	0.419	7.047E-201	Cycling
CD384	2.327E-204	0.9142717	0.214	0.119	3.577E-200	Cycling
EIF4EBP12	3.071E-204	0.32750998	0.782	0.664	4.72E-200	Cycling
TMUB12	3.453E-204	0.35910072	0.589	0.437	5.307E-200	Cycling
GLRX51	4.435E-204	0.44914387	0.505	0.359	6.817E-200	Cycling
SPATS25	6.192E-204	0.83875979	0.127	0.057	9.517E-200	Cycling
NAA384	9.579E-204	0.37032238	0.613	0.462	1.472E-199	Cycling
MIS121	4.38E-203	0.43738549	0.331	0.207	6.733E-199	Cycling
SLC29A11	7.484E-203	0.40331604	0.718	0.584	1.15E-198	Cycling
TMX42	7.881E-203	0.2517564	0.807	0.685	1.211E-198	Cycling
ANG2	1.25E-202	0.78042801	0.199	0.107	1.921E-198	Cycling
SLC25A192	2.251E-202	0.3546978	0.736	0.596	3.46E-198	Cycling
LSM51	2.329E-202	0.61387842	0.279	0.167	3.58E-198	Cycling
ZFYVE192	8.265E-202	0.45673425	0.407	0.269	1.27E-197	Cycling
FAF12	8.807E-202	0.38464878	0.66	0.525	1.354E-197	Cycling
DIS3L3	1.348E-201	0.37549989	0.519	0.369	2.073E-197	Cycling
IKBIP1	1.833E-201	0.56654845	0.281	0.168	2.818E-197	Cycling
EBP1	2.252E-201	0.31410502	0.632	0.482	3.462E-197	Cycling
CEP1354	3.27E-201	0.42393465	0.292	0.176	5.027E-197	Cycling

MDM14	4.42E-201	0.53736324	0.273	0.163	6.794E-197	Cycling
BLOC1S1	3.886E-200	0.25556891	0.892	0.798	5.973E-196	Cycling
DAZAP12	4.907E-200	0.38968907	0.569	0.423	7.542E-196	Cycling
DBI1	5.633E-200	0.29342124	0.818	0.695	8.658E-196	Cycling
NFYB1	7.643E-200	0.4716401	0.348	0.222	1.175E-195	Cycling
SORL15	1.033E-199	0.3604252	0.554	0.407	1.587E-195	Cycling
TAF62	1.759E-199	0.31584269	0.625	0.47	2.704E-195	Cycling
ATOH81	1.15E-198	3.79564047	0.01	0	1.768E-194	Cycling
DDX11	1.763E-198	1.84846675	0.033	0.007	2.709E-194	Cycling
CASP8AP22	4.261E-198	0.43387715	0.347	0.221	6.55E-194	Cycling
RTTN2	4.65E-198	1.03832038	0.092	0.036	7.147E-194	Cycling
CENPC4	9.684E-198	0.42637808	0.28	0.168	1.489E-193	Cycling
AP1S26	1.419E-197	0.38193851	0.449	0.306	2.181E-193	Cycling
NENF	2.11E-197	0.31133514	0.826	0.709	3.243E-193	Cycling
MAZ2	5.577E-197	0.52473707	0.298	0.183	8.572E-193	Cycling
PPIF2	7.598E-197	0.32766184	0.614	0.466	1.168E-192	Cycling
ABHD121	1.299E-196	0.26424478	0.872	0.771	1.996E-192	Cycling
ANO63	1.855E-196	0.39851869	0.506	0.358	2.851E-192	Cycling
ZNF833	9.686E-196	0.47738513	0.364	0.239	1.489E-191	Cycling
TRIM363	1.339E-195	0.58474591	0.321	0.202	2.058E-191	Cycling
PLEKHH32	1.374E-195	0.9702815	0.113	0.049	2.113E-191	Cycling
JOSD13	1.45E-195	0.29207505	0.833	0.72	2.228E-191	Cycling
NUP372	2.853E-195	0.45339984	0.416	0.279	4.385E-191	Cycling
AKT1	8.692E-195	0.23913806	0.916	0.828	1.336E-190	Cycling
COBLL13	2.089E-194	0.94655319	0.105	0.044	3.211E-190	Cycling
ATP8B44	4.285E-194	0.70587111	0.09	0.036	6.586E-190	Cycling
COQ22	5.494E-194	0.34946306	0.729	0.586	8.444E-190	Cycling
ZNF7301	7.895E-194	3.01552881	0.015	0.001	1.213E-189	Cycling
TAOK23	8.36E-194	0.33088882	0.609	0.456	1.285E-189	Cycling
ANKLE24	1.138E-193	0.26575024	0.666	0.516	1.75E-189	Cycling
DCTN31	1.231E-193	0.44266202	0.744	0.622	1.893E-189	Cycling
PFAS1	5.708E-193	0.44049172	0.385	0.251	8.773E-189	Cycling
ENOSF1	6.135E-193	0.36486926	0.629	0.48	9.43E-189	Cycling
KLHL421	7.631E-193	0.52449146	0.281	0.17	1.173E-188	Cycling
SENP12	1.374E-192	0.41754231	0.317	0.197	2.111E-188	Cycling
GGCT2	2.559E-192	0.46804704	0.418	0.284	3.933E-188	Cycling
GLB1L22	2.769E-192	1.35445255	0.041	0.01	4.257E-188	Cycling
RNASE42	3.875E-192	0.57184371	0.385	0.261	5.956E-188	Cycling
PA2G42	4.657E-192	0.3081327	0.712	0.58	7.159E-188	Cycling
SLF23	6.934E-192	0.36942515	0.555	0.409	1.066E-187	Cycling
ZNF7041	2.318E-191	1.92733325	0.017	0.002	3.563E-187	Cycling
TRAF22	3.975E-191	0.52508233	0.215	0.12	6.11E-187	Cycling
NIBAN14	5.74E-191	0.2003583	0.823	0.693	8.823E-187	Cycling
ORC34	6.631E-190	0.45156591	0.317	0.198	1.019E-185	Cycling
KMT5A1	8.947E-190	0.47315991	0.523	0.384	1.375E-185	Cycling
HSPB113	2.913E-189	0.37030297	0.507	0.362	4.478E-185	Cycling
SSX2IP1	1.481E-188	0.75198225	0.171	0.089	2.277E-184	Cycling
CEBPG3	1.909E-188	0.3163249	0.716	0.582	2.934E-184	Cycling
RBMX3	4.204E-188	0.44244731	0.52	0.383	6.461E-184	Cycling
LPCAT3	1.099E-187	0.34131457	0.725	0.591	1.689E-183	Cycling
RUVBL23	1.377E-187	0.4874047	0.437	0.304	2.116E-183	Cycling
HACE12	1.68E-187	0.46467806	0.264	0.157	2.582E-183	Cycling

UCK13	4.769E-187	0.35394448	0.579	0.432	7.33E-183	Cycling
CNPY41	7.885E-187	0.66708808	0.158	0.08	1.212E-182	Cycling
MPST1	9.031E-187	0.34373144	0.61	0.461	1.388E-182	Cycling
MPC23	2.507E-186	0.36419728	0.674	0.539	3.854E-182	Cycling
TRIM242	2.775E-186	0.43667855	0.352	0.229	4.265E-182	Cycling
PARD6A1	1.061E-185	0.70177871	0.149	0.073	1.63E-181	Cycling
CYREN3	1.27E-185	0.38034773	0.518	0.373	1.951E-181	Cycling
FGFR12	4.383E-184	0.90985442	0.13	0.062	6.737E-180	Cycling
PRADC11	5.497E-184	0.51377581	0.361	0.237	8.449E-180	Cycling
P4HTM2	1.109E-183	0.4305493	0.32	0.202	1.704E-179	Cycling
ABI23	1.124E-183	0.37698437	0.363	0.238	1.728E-179	Cycling
SEPTIN3	1.502E-183	0.69793996	0.242	0.143	2.309E-179	Cycling
NTAN12	2.184E-183	0.41451742	0.557	0.416	3.357E-179	Cycling
MPP12	3.238E-183	0.23565405	0.922	0.835	4.977E-179	Cycling
ZNF7382	3.555E-183	0.43391497	0.267	0.16	5.464E-179	Cycling
OGG13	4.289E-183	0.28882452	0.316	0.198	6.592E-179	Cycling
SRR2	5.657E-183	0.63160167	0.211	0.118	8.696E-179	Cycling
PNP2	1.291E-182	0.23821601	0.849	0.736	1.984E-178	Cycling
TAGLN21	1.712E-182	0.23736771	0.92	0.827	2.631E-178	Cycling
TRNAU1AP2	3.33E-182	0.35749834	0.567	0.426	5.119E-178	Cycling
B3GLCT1	5.382E-182	0.53600096	0.256	0.152	8.272E-178	Cycling
RIF13	7.245E-182	0.39121626	0.376	0.249	1.114E-177	Cycling
AP2A11	7.801E-182	0.26154225	0.856	0.748	1.199E-177	Cycling
PTBP13	8.387E-182	0.34595523	0.536	0.392	1.289E-177	Cycling
APAF17	1.052E-181	0.38559526	0.41	0.28	1.617E-177	Cycling
SMC62	1.667E-181	0.33965485	0.627	0.485	2.562E-177	Cycling
SHMT22	3.541E-181	0.60227924	0.216	0.122	5.442E-177	Cycling
TFR21	3.766E-181	1.61797847	0.026	0.005	5.789E-177	Cycling
DPY19L11	7.436E-181	0.52064148	0.308	0.195	1.143E-176	Cycling
TNFRSF13C2	2.831E-180	1.15115823	0.011	0.001	4.351E-176	Cycling
FXYD54	8.469E-180	0.13986946	0.985	0.951	1.302E-175	Cycling
CEP762	2.754E-179	0.57180452	0.24	0.141	4.234E-175	Cycling
LMCD14	2.755E-179	1.69413309	0.032	0.007	4.234E-175	Cycling
ITGB1BP11	2.769E-179	0.29708512	0.556	0.412	4.256E-175	Cycling
TMEM1432	4.272E-179	0.64334709	0.206	0.116	6.567E-175	Cycling
ING21	8.672E-179	0.46551997	0.371	0.245	1.333E-174	Cycling
DIPK2A5	1.672E-178	0.36067357	0.555	0.411	2.569E-174	Cycling
NUDT25	3.448E-178	0.35230272	0.555	0.411	5.3E-174	Cycling
CD300E2	6.749E-178	0.9224942	0.188	0.105	1.037E-173	Cycling
EDNRB	7.97E-178	3.877095	0.01	0	1.225E-173	Cycling
MFGE81	9.702E-178	0.80363147	0.242	0.147	1.491E-173	Cycling
REEP42	8.959E-177	0.56650914	0.329	0.213	1.377E-172	Cycling
DCBLD21	8.997E-177	0.80893507	0.148	0.075	1.383E-172	Cycling
LRP85	1.309E-176	0.46968618	0.147	0.073	2.012E-172	Cycling
DCAF122	2.275E-176	0.35962764	0.748	0.624	3.497E-172	Cycling
PBX32	2.472E-176	0.33318433	0.729	0.595	3.8E-172	Cycling
DCLRE1A3	6.069E-176	0.83624587	0.114	0.052	9.329E-172	Cycling
NUP1881	8.729E-176	0.32065112	0.632	0.492	1.342E-171	Cycling
CNOT92	1.288E-175	0.30669589	0.603	0.459	1.98E-171	Cycling
CMSS13	2.066E-175	0.47845398	0.284	0.175	3.176E-171	Cycling
MDC11	2.411E-175	0.38758046	0.283	0.174	3.706E-171	Cycling
LCORL3	2.585E-175	0.5224453	0.429	0.303	3.974E-171	Cycling

STRBP4	2.701E-175	0.26659799	0.203	0.113	4.151E-171	Cycling
DHRS133	3.225E-175	0.58880717	0.132	0.064	4.958E-171	Cycling
SOX4	5.701E-175	0.33191838	0.541	0.394	8.763E-171	Cycling
AAAS2	6.108E-175	0.29505746	0.588	0.44	9.388E-171	Cycling
EXOC62	1.475E-174	0.33851569	0.523	0.384	2.267E-170	Cycling
SCLT13	4.086E-174	0.57016008	0.288	0.182	6.281E-170	Cycling
HSP90B1	4.333E-174	0.26320368	0.879	0.775	6.661E-170	Cycling
VMA212	4.342E-173	0.29709492	0.706	0.573	6.675E-169	Cycling
LUC7L23	5.985E-173	0.22634853	0.832	0.727	9.199E-169	Cycling
RAVER12	7.497E-173	0.2775592	0.66	0.514	1.152E-168	Cycling
ZDHHC132	1.311E-172	0.38348959	0.332	0.215	2.016E-168	Cycling
PRKD34	2.652E-172	0.37420083	0.476	0.342	4.077E-168	Cycling
CALHM24	2.839E-172	0.28099841	0.715	0.57	4.364E-168	Cycling
CBX2	3.708E-172	1.90169417	0.032	0.007	5.699E-168	Cycling
ARHGAP191	6.958E-172	0.79740025	0.145	0.074	1.069E-167	Cycling
ACAD91	1.178E-171	0.34330978	0.599	0.454	1.811E-167	Cycling
GSDMD1	2.983E-171	0.24280669	0.851	0.747	4.586E-167	Cycling
GIT11	5.206E-171	0.3429501	0.617	0.48	8.003E-167	Cycling
CDAN13	5.847E-171	0.6222643	0.159	0.083	8.988E-167	Cycling
HNRNPL1	6.385E-171	0.25878714	0.792	0.676	9.814E-167	Cycling
CCSAP1	3.039E-170	0.39312046	0.559	0.419	4.671E-166	Cycling
SIN3A3	3.323E-170	0.30944634	0.627	0.497	5.108E-166	Cycling
F52	4.435E-170	1.30260222	0.08	0.032	6.818E-166	Cycling
NEDD4L7	8.297E-170	0.65920176	0.098	0.042	1.275E-165	Cycling
PRUNE22	1.01E-169	0.44075887	0.401	0.272	1.552E-165	Cycling
RAPH13	1.228E-169	0.50144549	0.263	0.16	1.888E-165	Cycling
HP1BP33	1.342E-169	0.42038199	0.676	0.554	2.063E-165	Cycling
ZNF5302	1.694E-169	0.90168531	0.082	0.033	2.604E-165	Cycling
SURF43	2.879E-169	0.26885745	0.732	0.605	4.425E-165	Cycling
CD361	5.562E-169	0.26673836	0.401	0.274	8.549E-165	Cycling
RAD9A3	2.256E-168	0.5871868	0.14	0.07	3.468E-164	Cycling
CAPN25	5.567E-168	0.16962523	0.927	0.841	8.557E-164	Cycling
DCLRE1B1	1.025E-167	0.54839421	0.282	0.176	1.575E-163	Cycling
GUCY1B11	1.702E-167	1.79205721	0.037	0.009	2.617E-163	Cycling
LRRC583	3.499E-167	0.3643413	0.509	0.371	5.378E-163	Cycling
RTN4R1	6.457E-167	0.63774481	0.186	0.104	9.924E-163	Cycling
ERLIN1	6.73E-167	0.29640111	0.749	0.623	1.034E-162	Cycling
ZNF7142	9.976E-167	0.89292875	0.097	0.042	1.533E-162	Cycling
FNTB3	1.28E-166	0.51713491	0.257	0.156	1.968E-162	Cycling
PRPS13	9.62E-166	0.36186936	0.498	0.366	1.479E-161	Cycling
AMOTL12	2.337E-165	0.35098391	0.68	0.544	3.593E-161	Cycling
LIPN3	3.901E-165	1.05161819	0.107	0.049	5.995E-161	Cycling
MEX3C4	3.956E-165	0.1698049	0.18	0.099	6.081E-161	Cycling
GNB53	4.204E-165	0.14754121	0.174	0.094	6.462E-161	Cycling
PSAT13	4.619E-165	0.75498923	0.052	0.017	7.1E-161	Cycling
LAMB23	6.254E-165	0.5690709	0.281	0.178	9.613E-161	Cycling
UBE2Q23	6.38E-165	0.2704886	0.661	0.513	9.806E-161	Cycling
DGCR83	7.592E-165	0.35459013	0.38	0.257	1.167E-160	Cycling
HMGN31	1.093E-164	0.26903151	0.851	0.743	1.681E-160	Cycling
TBCD3	2.438E-164	0.26245864	0.558	0.419	3.748E-160	Cycling
HMG20B1	1.536E-163	0.53132718	0.752	0.644	2.36E-159	Cycling
MOV104	2.437E-163	0.2645557	0.71	0.573	3.745E-159	Cycling

ANKRD523	3.466E-163	0.31444995	0.454	0.32	5.328E-159	Cycling
CDC42EP4	1.079E-162	0.54629731	0.274	0.171	1.658E-158	Cycling
CHAC22	1.65E-162	0.85469882	0.098	0.043	2.537E-158	Cycling
ZW10	1.913E-162	0.35727168	0.501	0.364	2.94E-158	Cycling
TOE12	1.975E-162	0.43439204	0.285	0.18	3.036E-158	Cycling
RRAS1	5.137E-162	0.37171296	0.503	0.363	7.897E-158	Cycling
DOT1L5	7.248E-162	0.3981319	0.463	0.334	1.114E-157	Cycling
REC84	7.57E-162	0.3236851	0.176	0.097	1.164E-157	Cycling
TM7SF3	1.513E-161	0.26613946	0.754	0.623	2.325E-157	Cycling
BAG24	1.661E-161	0.58452741	0.219	0.13	2.553E-157	Cycling
ATAD3A	6.106E-161	0.52840718	0.231	0.138	9.385E-157	Cycling
PIK3CD6	7.445E-161	0.11590869	0.614	0.482	1.144E-156	Cycling
C7orf571	3.849E-160	2.15118739	0.019	0.003	5.916E-156	Cycling
PLOD11	4.796E-160	0.2552904	0.85	0.746	7.372E-156	Cycling
GOLT1B4	6.969E-160	0.23912025	0.735	0.603	1.071E-155	Cycling
CPSF3	9.927E-160	0.31772782	0.564	0.423	1.526E-155	Cycling
LSM21	1.068E-159	0.33744855	0.516	0.38	1.642E-155	Cycling
SNRNP402	1.072E-159	0.2510585	0.75	0.632	1.648E-155	Cycling
MFSD14B	1.108E-159	0.23050376	0.903	0.817	1.703E-155	Cycling
SERINC55	1.204E-159	0.27975141	0.676	0.537	1.851E-155	Cycling
ASXL11	1.252E-159	0.2268018	0.674	0.541	1.924E-155	Cycling
GTF3C5	1.277E-159	0.4022341	0.504	0.369	1.963E-155	Cycling
CEP893	1.779E-159	0.4168963	0.271	0.169	2.734E-155	Cycling
FKBPL	2.454E-159	0.67641188	0.167	0.091	3.772E-155	Cycling
UBXN2A2	2.593E-159	0.45517655	0.26	0.161	3.986E-155	Cycling
POT11	3.247E-159	0.43463079	0.367	0.248	4.991E-155	Cycling
TMED9	4.779E-159	0.21838727	0.89	0.804	7.345E-155	Cycling
GNB43	7.6E-159	0.24403814	0.862	0.759	1.168E-154	Cycling
CENPT3	7.629E-159	0.31724475	0.342	0.227	1.173E-154	Cycling
CCDC1342	1.433E-158	0.40074362	0.137	0.069	2.202E-154	Cycling
SMCHD14	3.128E-158	0.13516217	0.612	0.479	4.808E-154	Cycling
CEP41	4.554E-158	0.54007128	0.214	0.125	6.999E-154	Cycling
UBAP23	4.631E-158	0.40659996	0.381	0.26	7.119E-154	Cycling
TTLL71	1.027E-157	2.03301286	0.015	0.002	1.579E-153	Cycling
SCCPDH1	1.343E-157	0.22491544	0.915	0.843	2.064E-153	Cycling
SLC19A11	1.974E-157	0.24990033	0.771	0.638	3.034E-153	Cycling
CANT14	2.445E-157	0.35692559	0.617	0.487	3.758E-153	Cycling
PRPF41	2.465E-157	0.33490771	0.552	0.415	3.789E-153	Cycling
RNF45	3.373E-157	0.22024972	0.798	0.681	5.184E-153	Cycling
ENKD12	5.208E-157	0.42438039	0.357	0.241	8.006E-153	Cycling
BCAP29	8.465E-157	0.30201574	0.701	0.57	1.301E-152	Cycling
AP002495.13	1.042E-156	0.68125203	0.099	0.044	1.601E-152	Cycling
MCU1	1.071E-156	0.38935388	0.35	0.233	1.646E-152	Cycling
PAXX3	1.169E-156	0.20955691	0.449	0.318	1.797E-152	Cycling
GTPBP33	1.331E-156	0.36488685	0.257	0.158	2.046E-152	Cycling
H2AFJ	2.234E-156	0.31059495	0.756	0.624	3.435E-152	Cycling
IFT221	2.856E-156	0.52083581	0.259	0.16	4.389E-152	Cycling
IER3IP13	1.096E-155	0.30165399	0.633	0.497	1.685E-151	Cycling
FNDC10	3.729E-155	0.46777344	0.402	0.283	5.732E-151	Cycling
MFSD13A1	4.751E-155	0.33171311	0.537	0.396	7.302E-151	Cycling
IFRD2	5.064E-155	0.30191524	0.638	0.506	7.783E-151	Cycling
P4HB1	6.347E-155	0.28977657	0.732	0.609	9.756E-151	Cycling

EEPD13	8.797E-155	0.59611393	0.122	0.06	1.352E-150	Cycling
SIGLEC121	1.13E-154	0.54680179	0.25	0.155	1.736E-150	Cycling
MT2A1	1.798E-154	0.33538788	0.559	0.43	2.764E-150	Cycling
RAD23A	2.37E-154	0.22822192	0.859	0.768	3.642E-150	Cycling
PRPF191	2.929E-154	0.26627248	0.711	0.579	4.502E-150	Cycling
PIGK	2.953E-154	0.33376284	0.623	0.487	4.539E-150	Cycling
HMGN52	3.882E-154	0.83631349	0.142	0.075	5.966E-150	Cycling
SLX44	5.586E-154	0.51921289	0.222	0.133	8.585E-150	Cycling
HDGFL23	8.524E-154	0.28344237	0.662	0.526	1.31E-149	Cycling
L2HGDH1	2.318E-153	0.77598858	0.108	0.051	3.563E-149	Cycling
ZDHHC143	2.554E-153	0.63163532	0.128	0.065	3.925E-149	Cycling
HSPA131	4.496E-153	0.34381148	0.504	0.371	6.911E-149	Cycling
NPM31	5.296E-153	0.36517372	0.345	0.23	8.14E-149	Cycling
CNTLN1	8.441E-153	0.46803251	0.302	0.196	1.297E-148	Cycling
BRAT11	1.102E-152	0.29268558	0.582	0.443	1.694E-148	Cycling
ECI12	2.453E-152	0.32831764	0.662	0.539	3.77E-148	Cycling
GEMIN41	2.559E-152	0.35698977	0.385	0.262	3.933E-148	Cycling
SNTB22	3.157E-152	0.28135521	0.404	0.283	4.852E-148	Cycling
PNN4	4.966E-152	0.25876776	0.496	0.361	7.633E-148	Cycling
TRIM283	7.441E-152	0.36710688	0.414	0.29	1.144E-147	Cycling
RAB8A1	1.564E-151	0.18487362	0.953	0.898	2.404E-147	Cycling
STX25	3.546E-151	0.21978741	0.467	0.336	5.45E-147	Cycling
RAB42	7.122E-151	0.41360896	0.392	0.271	1.095E-146	Cycling
TRA2B1	1.068E-150	0.24080401	0.703	0.573	1.642E-146	Cycling
CTNNBIP13	2.017E-150	0.31101582	0.545	0.41	3.1E-146	Cycling
NET13	2.644E-150	0.33463497	0.44	0.31	4.064E-146	Cycling
SMS1	3.082E-150	0.22332514	0.865	0.769	4.737E-146	Cycling
NHSL24	6.032E-150	0.51315966	0.074	0.03	9.272E-146	Cycling
PITHD13	1.177E-149	0.24882173	0.659	0.527	1.81E-145	Cycling
SGMS22	1.265E-149	0.23090072	0.919	0.831	1.945E-145	Cycling
PRRT42	1.346E-149	0.69814871	0.133	0.069	2.069E-145	Cycling
PRELID23	1.463E-149	0.66035907	0.142	0.074	2.249E-145	Cycling
PCBP32	1.842E-149	1.40524136	0.039	0.011	2.831E-145	Cycling
SLC25A52	2.329E-149	0.3437053	0.597	0.465	3.579E-145	Cycling
DEPDC74	3.006E-149	1.71564033	0.03	0.007	4.62E-145	Cycling
ZBTB471	4.235E-149	0.3069126	0.636	0.499	6.509E-145	Cycling
HADHA	5.091E-149	0.22541343	0.955	0.893	7.826E-145	Cycling
TAF152	5.51E-149	0.3026286	0.508	0.374	8.469E-145	Cycling
CTSA1	5.568E-149	0.18134306	0.98	0.941	8.558E-145	Cycling
RPN11	6.748E-149	0.254067	0.789	0.681	1.037E-144	Cycling
PDK2	7.094E-149	0.42397484	0.347	0.234	1.09E-144	Cycling
PTGFRN2	1.25E-148	0.45327658	0.34	0.23	1.921E-144	Cycling
SFMBT11	2.593E-148	0.52964142	0.199	0.117	3.986E-144	Cycling
SRSF73	2.845E-148	0.2453828	0.565	0.429	4.373E-144	Cycling
QSER11	3.445E-148	0.27737103	0.694	0.557	5.295E-144	Cycling
RNF1683	4.929E-148	0.22640915	0.843	0.731	7.576E-144	Cycling
FAM126A3	4.943E-148	0.25797412	0.638	0.5	7.598E-144	Cycling
SLFN111	7.105E-148	0.18424621	0.961	0.907	1.092E-143	Cycling
CEP1122	7.76E-148	1.3223603	0.044	0.014	1.193E-143	Cycling
SRRT2	1.279E-147	0.31572061	0.53	0.398	1.966E-143	Cycling
PIN12	1.384E-147	0.28204566	0.665	0.536	2.128E-143	Cycling
INF21	3.392E-147	0.1772603	0.965	0.908	5.214E-143	Cycling

TSHZ13	3.65E-147	0.32300731	0.429	0.304	5.611E-143	Cycling
MAGEF15	5.339E-147	0.62136976	0.144	0.077	8.207E-143	Cycling
DCAKD2	7.731E-147	0.40801113	0.298	0.194	1.188E-142	Cycling
ALMS12	7.955E-147	0.28360695	0.268	0.17	1.223E-142	Cycling
GLO1	9.788E-147	0.35109194	0.561	0.432	1.504E-142	Cycling
SCML2	1.523E-146	2.78051759	0.014	0.002	2.34E-142	Cycling
PANK12	2.166E-146	1.48022401	0.035	0.009	3.33E-142	Cycling
TRAM25	2.24E-146	0.46354213	0.222	0.134	3.444E-142	Cycling
ATL22	2.714E-146	0.4001755	0.317	0.211	4.172E-142	Cycling
RFX26	4.969E-146	0.44392565	0.245	0.152	7.637E-142	Cycling
POP7	6.517E-146	0.34452962	0.492	0.36	1.002E-141	Cycling
TMEM1163	1.352E-145	0.41506566	0.199	0.117	2.078E-141	Cycling
NDOR12	2.585E-145	0.38966506	0.225	0.136	3.974E-141	Cycling
BCL7C2	2.99E-145	0.28038252	0.628	0.495	4.596E-141	Cycling
MICALL21	4.873E-145	0.62419503	0.151	0.082	7.49E-141	Cycling
SFXN51	5.86E-145	0.47398562	0.275	0.176	9.007E-141	Cycling
GSS	6.271E-145	0.28068063	0.679	0.545	9.639E-141	Cycling
OTULIN4	1.438E-144	0.22995681	0.54	0.404	2.21E-140	Cycling
ZNF6802	1.674E-144	0.26726249	0.423	0.298	2.573E-140	Cycling
COG42	3.837E-144	0.27709531	0.582	0.444	5.898E-140	Cycling
MAPK92	5.301E-144	0.22673044	0.697	0.563	8.148E-140	Cycling
C12orf571	5.656E-144	0.18183905	0.513	0.392	8.693E-140	Cycling
SIMC13	6.542E-144	0.45051046	0.234	0.144	1.006E-139	Cycling
LSM112	1.111E-143	0.56292049	0.134	0.07	1.708E-139	Cycling
ASH2L	2.117E-143	0.37614403	0.444	0.32	3.255E-139	Cycling
ME33	2.818E-143	0.36826728	0.331	0.222	4.331E-139	Cycling
ARPIN1	4.61E-143	0.25817058	0.741	0.616	7.086E-139	Cycling
FAM20C3	4.989E-143	0.34260487	0.433	0.31	7.668E-139	Cycling
FGFR1OP4	6.113E-143	0.34146903	0.277	0.178	9.397E-139	Cycling
DAG11	8.425E-143	0.36205425	0.329	0.22	1.295E-138	Cycling
RNPS11	9.971E-143	0.23171298	0.677	0.536	1.533E-138	Cycling
SCML14	1.37E-142	0.48329097	0.282	0.184	2.105E-138	Cycling
PDIA62	1.634E-142	0.23879805	0.718	0.589	2.511E-138	Cycling
LRRK13	1.762E-142	0.30969789	0.516	0.383	2.708E-138	Cycling
INTS141	1.764E-142	0.30362572	0.517	0.384	2.712E-138	Cycling
ARL6IP1	4.824E-142	0.64024429	0.839	0.754	7.415E-138	Cycling
HACD25	6.996E-142	0.33596559	0.422	0.301	1.075E-137	Cycling
CIDEB2	8.246E-142	0.35463917	0.267	0.17	1.267E-137	Cycling
THEM6	1.336E-141	0.38527475	0.353	0.239	2.053E-137	Cycling
ELANE1	2.285E-141	2.54879797	0.023	0.005	3.513E-137	Cycling
ST145	4.019E-141	0.26486614	0.764	0.649	6.178E-137	Cycling
GPA332	7.111E-141	0.34499122	0.529	0.397	1.093E-136	Cycling
SF3A32	9.761E-141	0.25932177	0.639	0.506	1.5E-136	Cycling
PMF11	1.386E-140	0.24591534	0.588	0.453	2.13E-136	Cycling
FIBP	1.663E-140	0.23116587	0.799	0.684	2.556E-136	Cycling
UBA63	2.381E-140	0.23120212	0.717	0.587	3.66E-136	Cycling
TMEM2382	4.035E-140	0.49320537	0.182	0.106	6.203E-136	Cycling
ATPAF11	5.233E-140	0.28398153	0.538	0.408	8.043E-136	Cycling
PSMG34	8.897E-140	0.27658142	0.562	0.431	1.368E-135	Cycling
UBA23	1.062E-139	0.26769929	0.594	0.465	1.633E-135	Cycling
B4GAT11	1.337E-139	0.48786578	0.248	0.156	2.056E-135	Cycling
CASP32	1.805E-139	0.27253475	0.491	0.364	2.775E-135	Cycling

NRP12	1.884E-139	0.19766319	0.912	0.816	2.896E-135	Cycling
RNF1383	5.393E-139	0.2522599	0.491	0.361	8.289E-135	Cycling
NUP62CL1	5.412E-139	1.56010257	0.027	0.006	8.319E-135	Cycling
CPLANE12	9.938E-139	0.41226667	0.24	0.15	1.528E-134	Cycling
FOXP41	2.257E-138	0.25869318	0.632	0.497	3.47E-134	Cycling
AHCY	3.693E-138	0.32466364	0.571	0.44	5.676E-134	Cycling
GKAP11	4.072E-138	0.54592043	0.158	0.088	6.26E-134	Cycling
TCHP1	6.604E-138	0.32533694	0.414	0.295	1.015E-133	Cycling
TRIM452	9.194E-138	0.93835322	0.084	0.038	1.413E-133	Cycling
RPP301	1.181E-137	0.27614521	0.606	0.475	1.816E-133	Cycling
C12orf652	1.206E-137	0.30609221	0.2	0.119	1.854E-133	Cycling
TLCD3A3	1.857E-137	1.54213662	0.018	0.003	2.854E-133	Cycling
EXOSC22	3.155E-137	0.32242184	0.307	0.203	4.849E-133	Cycling
SUSD11	3.282E-137	0.2039679	0.817	0.687	5.044E-133	Cycling
FH	3.649E-137	0.25257418	0.735	0.61	5.609E-133	Cycling
EED2	4.354E-137	0.29740492	0.296	0.195	6.692E-133	Cycling
TMEM112	8.798E-137	0.2717948	0.583	0.448	1.352E-132	Cycling
MXRA73	1.06E-136	0.3187245	0.28	0.184	1.63E-132	Cycling
CIAO2A3	1.064E-136	0.24788733	0.781	0.668	1.635E-132	Cycling
KCTD32	2.099E-136	0.43994295	0.309	0.206	3.226E-132	Cycling
PHACTR44	2.115E-136	0.30488097	0.574	0.448	3.251E-132	Cycling
NXT13	2.177E-136	0.2769491	0.538	0.41	3.346E-132	Cycling
CDC232	2.322E-136	0.26779663	0.458	0.328	3.569E-132	Cycling
LRRC423	2.364E-136	0.43110647	0.332	0.227	3.634E-132	Cycling
TES4	2.515E-136	0.1342362	0.513	0.379	3.865E-132	Cycling
HSPD13	3.621E-136	0.25764712	0.706	0.582	5.566E-132	Cycling
FMR14	3.786E-136	0.22393072	0.589	0.458	5.819E-132	Cycling
H2AFY3	1.131E-135	0.19693218	0.914	0.832	1.738E-131	Cycling
LIMD15	1.284E-135	0.22524359	0.572	0.437	1.974E-131	Cycling
LRPAP11	1.939E-135	0.13922716	0.993	0.959	2.98E-131	Cycling
TICAM2	3.203E-135	0.32038473	0.515	0.386	4.923E-131	Cycling
PSMG12	3.718E-135	0.3436322	0.424	0.306	5.714E-131	Cycling
HCFC11	4.53E-135	0.25464494	0.666	0.533	6.963E-131	Cycling
MAPK32	4.863E-135	0.2946911	0.508	0.381	7.475E-131	Cycling
ULK21	5.29E-135	0.41707436	0.29	0.191	8.132E-131	Cycling
LNPEP1	6.274E-135	0.21349524	0.858	0.759	9.643E-131	Cycling
INIP1	6.975E-135	0.27962447	0.585	0.451	1.072E-130	Cycling
FES3	1.19E-134	0.26046224	0.657	0.517	1.829E-130	Cycling
LRRC8C3	1.804E-134	0.22089274	0.693	0.557	2.772E-130	Cycling
IGFBP21	2.012E-134	0.43458781	0.583	0.477	3.093E-130	Cycling
DENND6A2	2.053E-134	0.25069959	0.532	0.403	3.155E-130	Cycling
PCSK52	2.463E-134	0.41740125	0.281	0.184	3.785E-130	Cycling
ARL2	4.502E-134	0.23781247	0.806	0.709	6.919E-130	Cycling
PKIA2	5.153E-134	0.29532147	0.532	0.405	7.92E-130	Cycling
TXNDC162	7.83E-134	0.53768837	0.155	0.087	1.204E-129	Cycling
MED253	1.183E-133	0.18602483	0.855	0.75	1.818E-129	Cycling
[truncated: 228,432 more chars]
