# Supplementary material for: Alveolar macrophage subtypes express cholesterol and inflammation genes in cystic fibrosis
Source: Life Sci Alliance. 2026 Apr 30;9(7):e202503482. doi: 10.26508/lsa.202503482 (PMC13139742; doi:10.26508/lsa.202503482)
Supplement: Reviewer comments [file LSA-2025-03482_review_history.pdf]

Alveolar Macrophage Subtypes Express Cholesterol and Inflammation Genes in Cystic Fibrosis

Xin Li, Fred Kolling, Daniel Aridgides, Lorraine Gwilt, Diane Mellinger, Claudia Jakubzick, and Alix Ashare
DOI: https://doi.org/10.26508/lsa.202503482

Corresponding author(s): Alix Ashare, Dartmouth-Hitchcock Medical Center

Review Timeline:

Submission Date:	2025-08-09
Editorial Decision:	2025-10-17
Revision Received:	2026-02-09
Editorial Decision:	2026-03-16
Revision Received:	2026-04-03
Accepted:	2026-04-15

Scientific Editor: Sarita Hebbar

Transaction Report:

(Note: With the exception of the correction of typographical or spelling errors that could be a source of ambiguity, letters and reports are not edited. The original formatting of letters and referee reports may not be reflected in this compilation.)

No full Peer Review Process File is available with this article, as the authors have chosen not to make the review process public in this case.

October 17, 2025

Re: Life Science Alliance manuscript #LSA-2025-03482

Dr. Alix Ashare
Dartmouth-Hitchcock Medical Center
Medicine
One Medical Center Drive
Lebanon, NH 03766

Dear Dr. Ashare,

Thank you for submitting your manuscript entitled "Alveolar Macrophage Subtypes Differentially Regulate Cholesterol and Inflammation in Cystic Fibrosis" to Life Science Alliance. Your manuscript has been reviewed by two expert reviewers whose comments are appended below. As you will read, both reviewers find that your study, on BAL-fluid characterisation in CF, novel to the field and that the described RNA seq analyses will be of value. However they also raised concerns that preclude publication in LSA at this stage.

We invite you to submit a revised manuscript addressing the reviewers' comments. When submitting the revision, please include a letter addressing the reviewers' comments point by point. While a rebuttal must respond to all points in some form, additional experiments to resolve these points, other than indicated above, are not required.

The typical timeframe for revisions is three months. Please note that papers are generally considered through only one revision cycle, so strong support from the referees on the revised version is needed for acceptance.

To upload the revised version of your manuscript, please log in to your account: https://lsa.msubmit.net/cgi-bin/main.plex

You will be guided to complete the submission of your revised manuscript and to fill in all necessary information. Please get in touch in case you do not know or remember your login name.

While you are revising your manuscript, please also attend to the below editorial points to help expedite the publication of your manuscript. Please direct any editorial questions to the journal office.

We hope that the comments below will prove constructive as your work progresses.

Thank you for this interesting contribution to Life Science Alliance. We are looking forward to receiving your revised manuscript.

Sincerely,

Sarita Hebbar, PhD
Scientific Editor
Life Science Alliance
http://www.lsajournal.org

A. THESE ITEMS ARE REQUIRED FOR REVISIONS

-- A letter addressing the reviewers' comments point by point.

-- An editable version of the final text (.DOC or .DOCX is needed for copyediting (no PDFs.

-- High-resolution figure, supplementary figure and video files uploaded as individual files: See our detailed guidelines for preparing your production-ready images, https://www.life-science-alliance.org/authors

-- Summary blurb (enter in submission system: A short text summarizing in a single sentence the study (max. 200 characters including spaces. This text is used in conjunction with the titles of papers, hence should be informative and complementary to the title and running title. It should describe the context and significance of the findings for a general readership; it should be written in the present tense and refer to the work in the third person. Author names should not be mentioned.

-- By submitting a revision, you attest that you are aware of our payment policies found here: https://www.life-science-alliance.org/copyright-license-fee

B. MANUSCRIPT ORGANIZATION AND FORMATTING:

Full guidelines are available on our Instructions for Authors page, https://www.life-science-alliance.org/authors

We encourage our authors to provide original source data, particularly uncropped/-processed electrophoretic blots and spreadsheets for the main figures of the manuscript. If you would like to add source data, we would welcome one PDF/Excel-file per figure for this information. These files will be linked as supplementary "Source Data" files.

IMPORTANT: It is Life Science Alliance policy that if requested, original data images must be made available. Failure to provide original images upon request will result in unavoidable delays in publication. Please ensure that you have access to all original microscopy and blot data images before submitting your revision.

March 16, 2026

RE: Life Science Alliance Manuscript #LSA-2025-03482R

Dr. Alix Ashare
Dartmouth-Hitchcock Medical Center
Medicine
One Medical Center Drive
Lebanon, NH 03766

Dear Dr. Ashare,

Thank you for submitting your revised manuscript entitled "Alveolar Macrophage Subtypes Express Cholesterol and Inflammation Genes in Cystic Fibrosis".

Your manuscript was revised by one of the original reviewers.

Overall, In line with this reviewer's evaluation, we would be happy to publish your paper in Life Science Alliance pending resolution of all of the above points and final revisions necessary to meet our formatting guidelines. We request you to submit a revised manuscript document with all these changes highlighted along with a point-by-point response to this reviewer's comments.

MANUSCRIPT ORGANIZATION AND FORMATTING:

To avoid unnecessary delays in the acceptance and publication of your paper, please read the following information carefully. Full guidelines are available on our Instructions for Authors page, https://www.life-science-alliance.org/authors

Submission of a paper that does not conform to Life Science Alliance guidelines will delay the acceptance of your manuscript.

-For experiments with human subjects, please include a statement confirming that the experiments conformed to the principles set out in the WMA Declaration of Helsinki and the Department of Health and Human Services Belmont Report (https://www.life-science-alliance.org/editorial-policies#humans).
-Thank you for indicating that you have deposited the RNA sequencing data in the Gene Expression Omnibus repository. Please provide an accession ID or identifier associated with this data. Please indicate details for the repository and ID for the Metabolomics data.
-For figure 3G, please indicate sample size and specify if replicates are biological or technical replicates. Please also define that Y axes in the figure and the legend.
-Please expand the methods for metabolomics measurements reported in Figure 3G.
-Please upload all figure files as individual ones, including the supplementary figure files; all figure legends should only appear in the main manuscript file.
-Please consult our manuscript preparation guidelines https://www.life-science-alliance.org/manuscript-prep and make sure your manuscript sections are in the correct order and labeled correctly.
-Please move information such as Funding, COI, Author contributions, and Conflict of interest after the Materials and Methods section and before references. They shouldn't appear on the title page.
-Please add the X and Bluesky handles of your host institute/organization, as well as your own and/or one of the authors, in our system.
-Please use the [10 author names et al.] format in your references (i.e., limit the author names to the first 10).
-please add your main, supplementary figure, and table legends to the main manuscript text after the references section.
-LSA allows supplementary figures, but no EV (E) Figures; please update your callouts for the Supplementary Figures in the manuscript Fig E1=Fig S1; while supplementary figures use the system supplementary Fig S1
-please add callouts for Figures S1B, S2A and Tables S1, S2, S3 to your main manuscript text.
-Please be sure that the authorship listing and order is correct.

We encourage our authors to provide original source data, particularly uncropped/-processed electrophoretic blots and spreadsheets for the main figures of the manuscript. If you would like to add source data, we would welcome one PDF/Excel-file per figure for this information. These files will be linked as supplementary "Source Data" files.

It is Life Science Alliance policy that if requested, original data images must be made available to the editors. Failure to provide

original images upon request will result in unavoidable delays in publication. Please ensure that you have access to all original data images prior to final submission.

If you are planning a press release on your work, please inform us immediately to allow informing our production team and scheduling a release date.

We welcome submissions of potential cover images for the issue of LSA in which your work would appear. If you have high quality images associated with this work, please feel free to email these, with a caption, to the journal office.

LSA encourages authors to provide a 30-60 second video where the study is briefly explained. We will use these videos on social media to promote the published paper and the presenting author (for examples, see https://docs.google.com/document/d/1-UWCfbE4pGcDdcgzcmiuJl2XMBJnxKYeqRvLLrLSo8s/edit?usp=sharing). Corresponding or first-authors are welcome to submit the video. Please submit only one video per manuscript. The video can be emailed to contact@life-science-alliance.org

FINAL FILES:

To upload the final version of your manuscript, please log in to your account: https://lsa.msubmit.net/cgi-bin/main.plex
You will be guided to complete the submission of your revised manuscript and to fill in all necessary information. Please get in touch in case you do not know or remember your login name.

The following items are required for acceptance.

-- An editable version of the final text (.DOC or .DOCX) is needed for copyediting (no PDFs).

-- High-resolution figure, supplementary figure and video files uploaded as individual files: See our detailed guidelines for preparing your production-ready images, https://www.life-science-alliance.org/authors

-- Summary blurb (enter in submission system): A short text summarizing in a single sentence the study (max. 200 characters including spaces). This text is used in conjunction with the titles of papers, hence should be informative and complementary to the title. It should describe the context and significance of the findings for a general readership; it should be written in the present tense and refer to the work in the third person. Author names should not be mentioned.

The license to publish form must be signed before your manuscript can be sent to production. A link to the license to publish form will be available to the corresponding author only. Please take a moment to check your funder requirements.

Reviews, decision letters, and point-by-point responses associated with peer-review at Life Science Alliance will be published, alongside the manuscript. If you do want to opt out of having the reviewer reports and your point-by-point responses displayed, please let us know immediately.

Thank you for your attention to these final processing requirements. Please revise and format the manuscript and upload materials as soon as you are able.

Thank you for this interesting contribution to the literature. We look forward to publishing your paper in Life Science Alliance.

Sincerely,

Sarita Hebbar, PhD
Scientific Editor
Life Science Alliance
http://www.lsajournal.org

April 15, 2026

RE: Life Science Alliance Manuscript #LSA-2025-03482RR

Dr. Alix Ashare
Dartmouth-Hitchcock Medical Center
Medicine
One Medical Center Drive
Lebanon, NH 03766

Dear Dr. Ashare,

Thank you for submitting your Research Article entitled "Alveolar Macrophage Subtypes Express Cholesterol and Inflammation Genes in Cystic Fibrosis" and for responding to our requests. It is a pleasure to let you know that your manuscript is now accepted for publication in Life Science Alliance. Congratulations on this interesting work.

The final published version of your manuscript will be deposited by us to PubMed Central upon publication.

Once the full metabolomics data is published, do contact us with the relevant details to update this paper with the accession ID related to the cholesterol measurements.

Your manuscript will now progress through copyediting and proofing. It is journal policy that authors provide original data upon request.

Your article will publish open access upon publication under a CC-BY license.

Reviews, decision letters, and point-by-point responses associated with peer-review at Life Science Alliance will be published, alongside the manuscript. If you do want to opt out of having the reviewer reports and your point-by-point responses displayed, please let us know immediately.

IMPORTANT: If you will be unreachable at any time, please provide us with the email address of an alternate author. Failure to respond to routine queries may lead to unavoidable delays in publication.

Scheduling details will be available from our production department. You will receive proofs shortly before the publication date. Only essential corrections can be made at the proof stage so if there are any minor final changes you wish to make to the manuscript, please let the journal office know now.

DISTRIBUTION OF MATERIALS:
Authors are required to distribute freely any materials used in experiments published in Life Science Alliance. Authors are encouraged to deposit materials used in their studies to the appropriate repositories for distribution to researchers.

You can contact the journal office with any questions, contact@life-science-alliance.org

Again, congratulations on a very nice paper. I hope you found the review process to be constructive and are pleased with how the manuscript was handled editorially. We look forward to future exciting submissions from your lab.

Sincerely,

Sarita Hebbar, PhD
Scientific Editor
Life Science Alliance
http://www.lsajournal.org
